# Efficient Green Extraction of Nutraceutical Compounds from *Nannochloropsis gaditana*: A Comparative Electrospray Ionization LC-MS and GC-MS Analysis for Lipid Profiling

**DOI:** 10.3390/foods13244117

**Published:** 2024-12-19

**Authors:** Cristina Blanco-Llamero, Paz García-García, Francisco Javier Señoráns

**Affiliations:** Healthy Lipids Group, Faculty of Sciences, Universidad Autónoma de Madrid, Francisco Tomás y Valiente, 7, 28049 Madrid, Spainmariap.garcia@uam.es (P.G.-G.)

**Keywords:** *Nannochloropsis gaditana*, enzymatic pretreatment, advanced extraction, LC-MS, GC-MS, omega-3 PUFA, polar lipids, functional food

## Abstract

Microalgae have been described as a potential alternative source of a wide range of bioactive compounds, including polar lipids and carotenoids. Specifically, *Nannochloropsis gaditana* is described as producing large amounts of polar lipids, such as glycolipids and phospholipids. These natural active compounds serve as key ingredients for food, cosmetic, or nutraceutical applications. However, microalgae usually possess a rigid cell wall that complicates the extraction of these compounds. Thus, an ultrasound-assisted enzymatic pretreatment is necessary to efficiently extract bioactives from microalgae, and it was studied in this article. Pretreated biomass was extracted using different advanced and green methodologies and compared to traditional extraction. Furthermore, the analysis, characterization, and identification of valuable compounds using GC-MS and LC-MS analytical methods were also investigated. Interestingly, major results demonstrated the efficiency of the pretreatment, enriching polar lipids’ distribution in all extracts produced no matter the extraction technique, although they presented differences in their concentration. Pressurized liquid extraction and microwave-assisted extraction were found to be the techniques with the highest yields, whereas ultrasound-assisted extraction achieved the highest percentage of glycolipids. In summary, green extraction techniques showed their effectiveness compared to traditional extraction.

## 1. Introduction

In recent decades, microalgae have been described as a potential alternative source of a wide range of bioactives. Among the different species, *Nannochloropsis gaditana* has been studied as a lipid producer due mainly to its ability to accumulate large amounts of polar lipids, including omega-3 fatty acids, which have been widely described as health promoting compounds [1,2,3,4,5,6,7,8,9,10].

However, microalgae often possess a rigid cell wall that complicates bioactives’ extraction. Indeed, a pretreatment step is even necessary to break the microalgal cell wall, which enables the extraction of bioactive compounds. Previous work has focused on the development of an efficient pretreatment method by combining enzymes and physical methods, triplicating the oil yield achieved when a cocktail of proteases and carbohydrases was combined with ultrasounds for long times, which may be due to the *Nannochloropsis gaditana* (*N. gaditana*) cell wall composition, which includes fibers and proteins [9,10,11,12,13,14,15,16,17,18,19].

Moreover, an efficient extraction method is also a key point of the process to produce the target compounds. Recently, the chemical industry has been focused on the development of alternative extraction methods instead of traditional ones due to several factors, such as the environmental and economic impact [20,21,22,23,24,25]. The most commonly used techniques include pressurized liquids, ultrasounds, or microwave-assisted extraction. Microwave-assisted extraction (MAE) provides faster performance than conventional extraction techniques. It consists of the extraction of an analyte by a solvent by applying microwave energy. Microwaves are electromagnetic waves (300–3,000,000 MHz) that interact with matter, causing movement of the ions without producing changes in their molecular structure. This movement causes heating that depends on both the chemical nature of the matrix and the solvent. The higher the dielectric constant, the greater the energy absorption. The solvent–sample mixture is heated under pressure above the boiling point of the solvent, which increases the diffusivity, facilitating matter transfer. Its main disadvantage is the great difficulty of performing the process continuously and the degradation of thermolabile compounds due to the high temperatures that are reached [26,27,28,29].

Ultrasonic extraction (UAE) is emerging as an alternative to conventional extraction methods, with the potential to reduce energy consumption and enhance lipid extraction yields. Cavitation is considered the underlying mechanism of UAE, in which microbubbles form near cells and collapse, causing cell destruction and the release of compounds. In addition, UAE has the advantages of high efficiency, low solvent volume and extraction time, moderate cost, and easy operation. Additionally, ultrasonic extraction can generally be performed at low temperatures, minimizing potential thermal damage to bioactive components or loss of volatile components during extraction [2,8,24,28,30,31,32,33].

On the other hand, pressurized liquid extraction (PLE) uses high temperatures and pressures to improve the process and increase the extraction yield. The increasing temperature affects extraction kinetics by, among other things, increasing mass transfer rates, compound diffusion, and solubility and decreasing solvent viscosity. Additionally, because PLE stores the raw material in the extraction cell, there is no need to filter the extract after the process is completed, which allows for performing the process continuously [20,21,24,34,35,36,37,38,39,40].

While efficient extraction of valuable compounds is achieved, the precise characterization and quantification of the compounds present in the extracts is crucial for subsequent applications. The choice of the method of characterization depends on both variables related to the sample (quantity, physicochemical characteristics, stability), and on the method itself (price, accuracy, precision, sensitivity, selectivity, limit of detection). High-performance liquid chromatography (HPLC) is a technique for separating low-volatility and thermolabile compounds depending on their solubility in the mobile phase (liquid state) and their adsorption capacity in the stationary phase contained in the column chromatography, with application to polar lipids. Then, the equipment can be coupled to different detectors, such as evaporative light scattering (ELSD), diode array (DAD), or mass spectrometry (MS) [41]. Mass spectrometry is an analytical technique that is based on the separation of ions in the gas phase as a function of its mass/charge ratio (*m*/*z*). In this way, this technique can provide information on the molecular mass and chemical structure of molecules [42]. The main advantages of MS over other detection modes are the ability to analyze complex mixtures and the sensitivity [43]. Prior to the analysis, the compounds present in the sample must be ionized. Nowadays, different ionization systems are used, including matrix-assisted laser desorption/ionization (MALDI), Atmospheric Pressure Chemical Ionization (APCI), or Electrospray Ionization (ESI), among others. LC-MS ESI and GC-MS have been described as the best methods for definitely identifying compounds as lipids in the samples [39].

In the present study, different *N. gaditana* extracts produced with and without a combined enzymatic and ultrasound pretreatment will be thoroughly analyzed and compared through LC-MS and GC-MS profiling in terms of polar lipids and omega 3 PUFA content, taking into account the extraction technique used (MAE; PLE; UAE; Folch).

## 2. Materials and Methods

### 2.1. Materials

The dried biomass of Nannochloropsis gaditana was acquired from Algaenergy S.A. in Alcobendas, Spain. Hexane and high-performance liquid chromatography (HPLC)-grade solvents, such as 2,2,4-trimethylpentane and methyl tert-butyl ether (MTBE), were purchased from Macron Fine Chemicals in Gliwice, Poland. Chloroform and isopropyl alcohol were acquired from Scharlab S.L. in Sentmenat, Spain, while methanol was bought from Labscan Analytical Sciences in Gliwice, Poland. Panreac Química S.A in Barcelona, Spain supplied anhydrous ethanol (PRS-grade), sodium bicarbonate, and potassium hydroxide. Milli-Q quality water from Millipore Sigma in Burlington, MA, USA was utilized. Enzymes, such as Viscozyme^®^ from Aspergillus aculeatus with carbohydrases, like arabinase, cellulase, beta-glucanase, hemicellulase, and xylanase, along with Celluclast^®^ with cellulases from Trichoderma reesei and Alcalase^®^ were supplied by Novozymes in Bagsværd, Denmark.

Various HPLC standards, including glycerol trilinoleate, a mixture of 1,3- and 1,2-isomers of dioleoylglycerol, 1-oleoyl-rac-glycerol, oleic acid, and ethyl linoleate, were bought from Sigma-Aldrich in St. Louis, MO, USA. Analytical or HPLC-grade reagents and solvents were utilized. 2-methyl-THF was acquired from Pennakem, located in Memphis, TN, USA. Fatty acid methyl ester standards (Supelco 37 FAME Mix) were purchased from Supelco, located in Bellefonte, PA, USA.

### 2.2. Combined Pretreatment Method

Solutions of enzyme mixtures, each comprising 46 mg of commercial enzymes, were prepared with either Viscozyme and Celluclast or Viscozyme, Celluclast, and Alcalase [44]. The enzyme blends were then placed in ideal conditions, precisely pH 5.0 and 55 °C, for a period of 6 h. The process of incubation occurred in a water bath in order to guarantee steady and regulated temperature conditions (United States Thermal). Afterward, the bottle’s contents were spun at 3000 rpm for 10 min, the liquid on top was removed, and the solid biomass was kept at 4 °C. They were then placed in a water bath set at optimal conditions (pH 5.0 and 55 °C) for 6 h. The bottle’s contents were spun at 3000 rpm for 10 min, the liquid floating above was thrown away, and the solid biomass at the bottom was kept at 4 °C for extraction and characterization. Experiments were conducted a minimum of three times in every instance [1,2].

### 2.3. Extraction of Microalgal Biomass

Extraction of *Nannochloropsis gaditana* was performed using different green advanced techniques: ultrasound-assisted extractions (UAE), pressurized liquid extraction (PLE), and microwave-assisted extraction (MAE) [45]. The results were compared with traditional extraction using the Folch method. The experiments were conducted in all cases with a minimum of two replicates.

#### 2.3.1. Ultrasound-Assisted Extraction

The UAE process utilized an Elma Elmasonic S40H ultrasonic bath from Singen, Germany equipped with automatic time and temperature control and an ultrasonic frequency of 37 kHz. Weighing samples (0.5 g of dry biomass) and adding ethanol at a 1:10 ratio were part of the procedure. Drawing from past research on microalgae extraction, each solvent was used for extraction at a temperature of 50 °C for a duration of 30 min. The samples were evaporated under reduced pressure at 35 °C using a rotary evaporator (Heidolph Hei-Vap Value HB/G3, Schwabach, Germany). Afterward, they were dried under a nitrogen flow until a constant weight was reached. The amount of lipids was measured through gravimetric analysis and reported as a percentage of the weight of dry biomass. The lipid extracts produced were kept in a dark container with a nitrogen atmosphere at 4 °C until analysis [24].

#### 2.3.2. Pressurized Liquid Extraction

The PLE technique was carried out with an ASE 350 DIONEX extractor from Sunnyvale, CA, USA, which came with a 10 mL stainless-steel extraction cell. First, 1 g of dry microalgal biomass was measured, combined with sea sand at a 1:10 ratio, and subsequently placed in the extraction cell. Ethanol was poured into the extraction cell and then heated to the chosen temperature of 120 °C. During every trial, the fixed time for extracting was 15 min, and the amount of solvent ranged from 20 to 25 mL, based on the cell’s temperature and pressure. In the end, the samples were gathered in 50 mL vials while a stream of nitrogen was used. Samples were evaporated and treated following the same procedures outlined for the UAE technique [24].

#### 2.3.3. Microwave-Assisted Extraction

First, 1.0 g of microalgae was introduced in a single extraction cell into the microwave, and then 20 mL of extraction solvent (ethanol) was added and introduced into the microwave for 5 min of extraction. The maximum working power depended on the solvent used (800 W for ethanol). The maximum temperature should not exceed 130 °C, as the desirable compounds are heat-labile.

After that, the samples from the microwave were removed and placed to cool in an ice bath until depressurized. Once the extraction was performed, the contents of the cells were filtered. Then, samples were evaporated and treated as previously described.

#### 2.3.4. Traditional Lipid Extraction of Microalgal Biomass

The lipid extraction from Nannochloropsis gaditana microalgae biomass was carried out using the widely accepted Folch method, as outlined by Folch et al. [45]. First, 1 g of microalgae biomass was extracted with 20 mL of chloroform:methanol (2:1) for a duration of 2 min. After being centrifuged at 3000 rpm for 10 min, the resulting mixture was collected, making sure to separate the organic layer. The organic layer was purified by being washed with water and then centrifuged at 3000 rpm for 10 min. This purification process was repeated three times on the same organic layer. The layer with chloroform, which had the lipids extracted, was then gathered. Afterward, the samples were evaporated and handled in the same way as outlined in the earlier method.

### 2.4. LC-MS Analysis Method and Equipment

The analysis of compounds in the extracts was performed using a high-resolution Bruker LC-MS system equipped with a quadrupole time-of-flight (QTOF) mass analyzer (Bruker, Billerica, MA, USA). The instrument features a mass range of 20 to 40,000 Da and operates at a resolution of 80,000 for ions at *m*/*z* 1222. Ionization was achieved with an Electrospray Ionization (ESI) source in positive mode, although the system is also capable of Atmospheric Pressure Chemical Ionization (APCI). Chromatographic separation was carried out using a Luna column (150 mm × 2.1 mm, 5 μm particle size). The mobile phases were Channel A, 10 mM of Ammonium Acetate: methanol (1:1) + 0.1% formic acid, and Channel B, Isopropanol + 0.1% formic acid. A gradient elution was applied over 43 min, from 30% B to 100% B, at a constant flow rate of 0.3 mL/min. Samples were dissolved in ethanol at a concentration of 0.1 mg/mL.

The instrument operated within a scan range of 50 to 3000 *m*/*z*. The acquisition parameters were as follows: nebulizer pressure of 3.0 bar, capillary voltage of 3500 V, dry heater temperature of 250 °C, dry gas flow rate of 6.0 L/min, end plate offset at −500 V, and charging voltage of 2000 V. The corona current and APCI heater temperature were set to 0 nA and 0 °C, respectively. The system supports both MS and MS/MS analyses with or without prior separation via U/HPLC, with exact mass determination when required. Peaks were identified and assigned using the mass library of the equipment for compound identification, the *m*/*z* ratio and fragments detected through MS/MS, and with the support of SciFinder (Supplementary Materials).

### 2.5. HPLC–ELSD-DAD Analysis

The HPLC analysis was performed with an Agilent 1260 Infinity HPLC system, featuring ELSD and Agilent 385 UV-Vis-DAD instruments, situated in Palo Alto, CA, USA. The separation through chromatography of various lipid types (neutral and polar lipids) was carried out with an ACE silica column in normal phase (ID 250 mm × 4.6 mm 0.5 μm) and a C18 reverse phase column kept at 30°. The column C ternary gradient is described as follows. Starting at 0 to 3 min, it transitions from 95% B and 5% C to 50% B and 50% C, with 2% A, 48% B, and 50% C at t = 3 min. At 9 min, A was 60% and B was 5%. At 17 min, C was 35%. At 21 min, A was 75%, B was 5%, and C was 20%. At 31 min, B was 50% and C was 50%. At 33 min, B was 95% and C was 5%. Eluent A contained methanol, eluent B contained 2,2,4-trimethylpentane, and eluent C contained MTBE. The flow rate fluctuated, set to either 1.0 or 2.0 mL/min. The best signal and clarity were achieved with the following ELSD settings: vaporizer temperature = 30 °C; nebulizer temperature = 30 °C; and N2 vaporizing gas = 1.6 SLM. Lipid species were identified by utilizing standard commercial products for neutral lipids, such as triglycerides (TAG), diglycerides (DAG), monoglycerides (MAG), glycolipids (GL), and free fatty acids (FFA). The results are shown as the relative percentages of each lipid species in the sample (within the normal range) and also as mg/g extract with the use of external standards. HPLC-ELSD analysis was conducted a minimum of two times in each instance.

### 2.6. Fatty Acid Composition Through GC-MS

Gas chromatography–mass spectrometry (GC-MS) was utilized to analyze the fatty acid composition of the extracted sample. Fatty Acid Methyl Esters (FAMEs) were recently produced by methanolysis of glycerides with KOH in methanol. The examination was performed on an Agilent GC-MS Series 5975 MSD situated in Palo Alto, CA, USA. The separation of the FAMEs was performed with an HP 88 capillary column provided by Agilent in Santa Clara, CA, USA. It had dimensions of 100 m × 0.25 mm (inner diameter) and a film thickness of 0.2 μm. A 1:100 split ratio was used to inject 1 μL of the sample. Following injection, the column was maintained at a temperature of 175 °C for 10 min, and then the temperature was increased at a rate of 3 °C per minute for an additional 20 min until reaching 220 °C. Helium was used as the carrier gas, with a constant flow rate of 1.5 mL/min. The injector was heated to 250 °C, while the detector was set to 230 °C. The mass spectrometer used a voltage of 70 eV and analyzed molecules in the 30–400 amu range. Fatty Acid Methyl Esters (FAMEs) were detected by comparing their retention times and mass spectra (using the NIST Mass Spectral Library version 2.0) with established standards (Supplementary Materials). The results were expressed as the individual relative percentages of each fatty acid in relation to the total number of FAMEs in each sample.

### 2.7. Statistical Analysis

The findings are shown as the average and variation of the study. Statistical analysis was performed with the web-based tool Simple Interactive Statistical Analysis (SISA), which can be found at http://www.quantitativeskills.com/sisa/index.htm (accessed on 20 September 2023). Statistical analysis was performed using ANOVA to evaluate differences between groups, followed by an F test to assess the overall significance. When comparing two specific groups, a *t*-test was used (*p* < 0.05). To address multiple comparisons, a post hoc Bonferroni correction was applied to control the Type I error rate. This combination of ANOVA, t-tests, and Bonferroni correction ensures that conclusions regarding significant differences are robust and appropriately adjusted for multiple testing scenarios.

## 3. Results and Discussion

### 3.1. Comparison of Extraction Yield Obtained Through Different Extraction Methods After Pretreatment

Combined enzymatic and ultrasound pretreatment has demonstrated its usefulness for the microalgae biomass in terms of extraction yield compared to the biomass without pretreatment or compared to the biomass with other pretreatment methods, such as only ultrasounds. These results have been demonstrated employing traditional lipid extraction methods, such as the Folch method. Nowadays, a new trend is observed in the food industry of supporting alternative and environmentally friendly extraction methods. Thus, in the first stage of this study, different extraction techniques, such as PLE, MAE, and UAE, were applied to the pretreated *N. gaditana* biomass and compared to the traditional Folch method.

**Figure 1:**
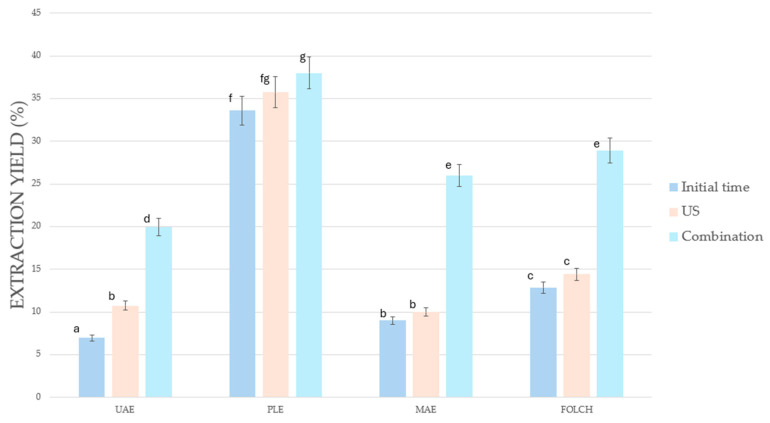
*N. gaditana* extraction yield obtained using different techniques (UAE, PLE, MAE, and Folch) comparing the initial time with the combined pretreatment and the ultrasounds on their own. Lowercase letters (a–g) indicate significant differences between values within the same column. Different letters denote that the corresponding values are statistically different (*p* < 0.05, according to the applied multiple comparison test).

All of the extraction techniques were applied to the pretreated biomass. The results represented in Figure 1 compare the yields achieved with the ultrasound-assisted enzymatic pretreatment to the initial time and to the pretreated biomass without enzymes, only taking into account the action of ultrasounds. As a result, the combined pretreated biomass achieved a clearly remarkable increase in the oil yield compared to the other two samples using either one extraction technique or another.

Comparing further the extraction techniques, PLE was the one that achieved the highest results, followed by MAE, Folch, and UAE. However, PLE was also the technique with lower influence of the pretreatment, which may be due to the fact that the PLE extraction yield obtained was high enough without the need for a pretreatment, as this technique is able to break the microalgae cell wall at a greater rate than the others due to the action of the pressure [37]. A major pretreatment influence could be seen on the composition of the extracts, though, when the extracts were analyzed in these cases.

### 3.2. Chemical Characterization of N. gaditana Extracts Through LC-MS

The chemical composition of the different extracts was analyzed through HPLC-MS. In this study, we chose to focus exclusively on lipid bioactives due to their unique biological relevance in the context of inflammation, metabolic health, etc. While other nutraceutical compounds were detectable via LC-MS and GC-MS, our aim was to thoroughly investigate the lipidic fraction given its specific role. Future work will likely explore these additional compounds.

It was possible to confirm the presence of polar lipids, such as glycolipids and phospholipids. As an example of microalgae composition, data from the extract using PLE are shown. Table 1 shows the information obtained by analyzing them under an MS detector (retention time, compound identification, *m*/*z* ratio, and fragments detected through MS/MS). See Supplementary Materials for further details.

The analysis revealed a variety of lipid species, including phospholipids, such as PC, PE, and glycolipids, like MGDG and DGDG. These compounds are essential components of cellular membranes, and their identification is crucial for understanding the lipid profile of the sample. PC derivatives were identified at retention times of 4.7 and 17.2 min (Table 1, compounds **4** and **8**). The observed *m*/*z* values, such as 494.3270 and 520.3640, matched closely with the theoretical *m*/*z* values, indicating the presence of phosphatidylcholine species, potentially with ammonium or sodium adducts ([M+NH4]+ and [M+Na]+). These compounds are often involved in membrane stability and signaling processes. PE species were also detected at retention times of 19.7 min (compound **10**), with a highly accurate *m*/*z* value of 806.5927. This species is known for its role in membrane fusion, and it is essential for mitochondrial function. The error in the *m*/*z* values was minimal, suggesting high confidence in this identification. MGDG and DGDG, which are major components of the thylakoid membranes in plants, were observed at retention times of 4.9 and 28.4 min (Table 1, compounds **5** and **12**). These glycolipids are involved in light-harvesting and photosynthesis. The *m*/*z* values for these compounds (520.3645 and 600.5194) were in excellent agreement with the theoretical values, confirming their identification as MGDG and DGDG derivatives. Their abundance aligns with the observations of Martin et al. (2014), who noted that glycolipids constitute a significant fraction of total lipids in *Nannochloropsis*, particularly in cells grown under high light intensity [46].

In addition to the core phospholipids and glycolipids, several lipid derivatives were also detected, including triglycerides and other esterified lipids. These species were identified based on their fragmentation patterns, although the confidence in their exact identification varied. For instance, a triglyceride derivative at 20.2 min (compound **11**) had an observed *m*/*z* of 758.5687, but due to its fragment spectrum, the identification was classified as low confidence. Such derivatives are common in biological samples, particularly in cellular storage systems.

Peaks at 17.2 min and 20.2 min show adducts with possible compositions suggesting glycolipids, although additional fragmentation data would be needed to confirm these identities.

Sphingolipids are often characterized by their specific fragmentation patterns, and the presence of long-chain fatty acids could indicate their involvement. Peaks like *m*/*z* 600.5194 and *m*/*z* 553.4586 could be indicative of such molecules if they correlate with sphingolipid-like fragmentation. The *m*/*z* 820.7375 at 37.5 min could correspond to a ceramide derivative or a sphingomyelin, especially if it is accompanied by [M+NH4]+ or [M+Na]+ adducts. However, further confirmation via fragmentation analysis is needed.

Triglycerides (TAGs) were abundant at higher retention times, such as the compound with *m*/*z* 902.8166 ([M+NH4]+), consistent with the well-documented ability of *Nannochloropsis* to accumulate TAGs under stress. This observation is in agreement with previous studies, such as those by Couto et al. (2022), which demonstrated that nutrient deprivation, particularly nitrogen, induces TAG accumulation in *Nannochloropsis* [47].

The great abundance of TAGs, coupled with their structural diversity, underscores the potential of *N. gaditana* as a feedstock for biodiesel production. TAGs from *Nannochloropsis* are particularly attractive due to their favorable fatty acid composition, including a high proportion of saturated and monounsaturated fatty acids, which enhance oxidative stability in biofuels. *m*/*z* 758.5687 at 20.2 min and *m*/*z* 806.5927 at 19.7 min suggest the presence of triglyceride derivatives. *m*/*z* 548.5028 at 30.5 min also points to possible triglycerides or their related fatty acid esters, especially in cases where esterified fatty acids could be linked to glycerol or sphingosine backbones. At 4.9 min, a peak at *m*/*z* 520.3640 could be associated with a phosphoethanolamine or amino lipid, important in cell signaling and membrane composition. The compound at 3.4 min (*m*/*z* 197.1171) could represent a short-chain fatty acid or an amino lipid that might be involved in signaling pathways or membrane dynamics.

Finally, the identification of these compounds was also confirmed using commercial standards. The results achieved were in agreement with previous work on *N. gaditana* characterization. The presence of these diverse lipid classes highlights the complexity of the sample and suggests a variety of functional lipid species in the biological system under study. These results are consistent with previous studies on *Nannochloropsis gaditana*, where lipid profiling using LC-MS revealed a similar composition, dominated by glycolipids and phospholipids. Furthermore, Couto et al. (2022) also reported significant levels of phosphatidylcholine (PC) and phosphatidylethanolamine (PE), with *m*/*z* values that closely match those observed in this analysis. By comparing our results with these previous studies, we confirm that the lipid profiles of *N. gaditana* are highly consistent, regardless of variations in cultivation or extraction methods, demonstrating the robustness of this species’ lipidome [5,17,22,24,37,47,48,49].

Moreover, Martin et al. (2014) conducted a comprehensive study on lipid profile remodeling in response to nitrogen deprivation in microalgae, including *Nannochloropsis gaditana*. They utilized LC-MS to analyze the fatty acid composition of glycolipids, confirming the utility of this technique in elucidating the complex lipid profiles of microalgae. The integration of LC-MS with advanced data acquisition techniques, such as those proposed by Hendriks, 2024 and Vasilopoulou et al., 2020, allows for in-depth lipidomic analysis from minimal sample amounts. These methodologies enhance the sensitivity and resolution of lipid detection, making them suitable for analyzing the diverse lipid classes present in *Nannochloropsis gaditana.* Several lipids with ambiguous identification were detected in the present study, though. For instance, the compound at *m*/*z* 758.5374 ([M+NH4]+) was tentatively assigned as a phosphatidylcholine species (C34H85N3O10PS), but the lack of sufficient fragmentation data reduced the confidence of this identification. Similar challenges were reported by previous studies, which emphasized the limitations of MS-based lipid identification in complex matrices without complementary structural elucidation methods, such as NMR [46,47,50,51]. The lipid profiles observed in this study broadly align with the trends reported across *Nannochloropsis* species, with PC, PE, MGDG, DGDG, and TAGs as the primary lipid classes. However, subtle differences in lipid abundances and compositions were noted, likely reflecting variations in growth conditions and extraction protocols. For instance, the higher abundance of MGDG relative to DGDG in this study contrasts with the findings of Martin et al. (2014), who reported a more balanced ratio under nutrient-replete conditions. Similarly, the prominence of TAGs in this study suggests that the growth conditions favored lipid accumulation, potentially through nitrogen limitation or other stressors. This is consistent with the previous observations, which demonstrated that Nannochloropsis’ lipid composition is highly sensitive to environmental factors, with TAGs serving as an adaptive energy reserve [46].

Upon studying the chemical composition and the differences amongst the produced extracts, polar lipids’ peaks were compared and quantified to compare each technique and the pretreatment influence.

Indeed, polar lipids in microalgae bind to the cell wall; thus, the application of a pretreatment increased the accessibility of them for posterior extraction, as observed when comparing the initial time and the different pretreatments [52]. *N. gaditana* has a rigid cell wall composed mainly of carbohydrates, such as cellulose, hemicellulose, pectin, and proteins, which provide a strong structure; thus, the application of enzymes contributes to the breakdown of this structure [46]. In any case, enzymes used as pretreatment had a greater influence on chemical extraction than ultrasounds alone, showing them to be an effective method to enhance enzyme action in microalgae [2].

Once the influence of the different pretreatments was studied, the optimized method was applied to the extracts coupled with different extraction techniques to study its influence on the composition of the extracts as a comparison between extraction methods.

#### Comparison Between PLE, UAE, and MAE Extracts: Influence of the Pretreatment on Polar Lipids

The extraction of lipids from *Nannochloropsis gaditana* has garnered significant attention due to its potential applications in nutraceuticals and biofuels, particularly given its high content of eicosapentaenoic acid (EPA). Various extraction methods have been investigated to optimize lipid yield and composition, with notable advancements in techniques like subcritical water extraction (SWE) and enzymatic hydrolysis. Ho et al. (2018) demonstrated that SWE can effectively extract lipids and EPA from *Nannochloropsis gaditana*, highlighting the method’s efficiency compared to traditional extraction techniques. This study emphasizes the importance of optimizing extraction conditions to maximize the lipid yield [49].

Table 2 shows the percentage and amount of GL in the extracts, comparing extraction techniques coupled with the optimized pretreatment and their influence on lipid class composition. Lowercase letters (a–e) indicate significant differences between values within the same column based on the Bonferroni test. Values sharing the same letter are not significantly different, while those with different letters are. It can be observed taking into account either the percentage or mg how the results follow the same trend as the one observed for the traditional extraction (the Folch method). It is interesting to point out the remarkable increase of GL % in all of the extracts produced, especially the ones from UAE, enriching the extracts in polar lipids compared to their initial time, which highlights the importance of an adequate pretreatment to enhance the glycolipids’ presence and to modify the chemical composition of the extracts according to our objectives. It is also important to highlight the effect of the ultrasounds as pretreatment in the enrichment of %GL compared to the initial time, which had a greater influence than the one observed for the mg in them, being similar to the initial time. Thus, the combined pretreatment seemed to be highly selective for the posterior extraction of polar lipids compared to the other lipid classes, as the increase in the % of GL implies a decrease in other classes.. This fact may be due to the action of the selective enzymes on the disruption of the microalga cell wall. In fact, polar lipids are usually bound to proteins in the cell membrane and the wall; thus, the disruption of cells facilitates the release of these compounds to the medium, increasing their ratio in the resulting extracts.

It is interesting to compare the different extraction methods, as they have different mechanisms of action. On the one hand, PLE and MAE achieve higher recovery results (mg) than UAE, which may be due to the high pressure achieved inside of the extraction cell, whereas UAE’s pressure in the vials of extraction is lower, and it does not achieve the ratios of the other techniques. Even so, all of the methods benefit from the combined pretreatment, increasing considerably the results achieved.

Moreover, the use of enzyme-assisted extraction methods has been shown to improve lipid recovery. Previous studies investigated green extraction technologies, combining enzymatic methods with ultrasound, which resulted in a more sustainable approach to producing omega-3 fatty acids from microalgae [29,32]. This aligns with the findings of Zuorro et al. (2016) [5], who reported that enzymatic hydrolysis can effectively release high-value polyunsaturated fatty acids from *Nannochloropsis gaditana*, thus enhancing the overall lipid extraction process. Additionally, Blanco-Llamero and Señoráns (2021) explored the use of biobased solvents in pressurized liquid extraction, further supporting the notion that solvent choice significantly impacts lipid extraction efficiency. Their findings indicate that specific solvents can enhance the yield of omega-3 lipids, including EPA, from *Nannochloropsis gaditana* [2,23,24].

### 3.3. Omega 3 PUFA Content in the Different Extracts

To further compare the pretreatment and extraction techniques, all of the extracts produced were injected into GC-MS equipment. This led to comparable fatty acid profiles with notable variations in fatty acid ratios (Table 3). The identified fatty acids included myristic acid (14:0), palmitic acid (16:0), palmitoleic acid (16:1), stearic acid (18:0), oleic acid (18:1), linoleic acid (18:2), γ-linolenic acid (20:3), and eicosapentaenoic acid (20:5). Two key aspects can be emphasized. PUFAs were the primary group of fatty acids (54.31–46.15%), with monounsaturated FAs (MUFAs) following behind at 30.12–24.72% and saturated FAs at 24.13–20.80%. However, in the PUFA group, there was a greater presence of n-3 PUFA compared to n-6 PUFA, leading to a favorable n-6/n-3 ratio [24,48,49,53,54,55,56,57].

Indeed, EPA (20:5) was the major peak found in the extracts (35%), producing extracts rich in omega-3 lipids, which is in agreement with other work that described *N. gaditana* as an alternative source for omega-3 PUFA. The traditional source of this FA has been fish or krill oil, which both have a negative environmental impact due to the overexploitation of the oceans. Thus, EPA from microalgae source emerges as a sustainable and greener alternative to produce this PUFA that could be used in the food industry. Microalgae have a fast growth rate and do not require arable land for cultivation, being able to grow in wastewater [58,59,60,61,62,63,64].

EPA content was studied among the different extracts obtained (Table 3). As expected, corresponding differences were observed depending on the relative proportions of glycolipids or acylglycerols in the extracts; thus, the extracts richer in GL and PL coincided with the ones with higher EPA content, which is related to the location of this FA in microalgae cells, which used to be bound to polar lipids in the cell walls. Then, the extracts after the pretreatment were richer in omega-3 compared to its initial time for all of the extraction techniques used, as as it has been shown that the pretreatment seems to be selective in enriching polar lipids in *N. gaditana* extracts, including EPA, which is related to the disruption rate reached during the process.

On the other hand, important differences among the extraction techniques were observed, as mentioned above (Table 2). Considering the initial time, meaning lipid extraction avoiding the pretreatment step, the Folch method was the one that achieved the lowest EPA content in its extracts, whereas PLE was the one that achieved the highest one. This fact could be due to the mechanism of its method. On the one hand, PLE uses high pressures, which help to break microalgae cell wall, releasing into the medium higher amounts of polar lipids, as shown before. On the other hand, the Folch method is performed at room temperature, and it uses mixes of solvents with affinity for the cell wall (chloroform: methanol) but does not achieve maximum cell wall disruption. Therefore, the application of the pretreatment avoids these differences between extraction methods, showing similar EPA content after it in all of the extracts, although with low differences, as the content achieved using the Folch method was still slightly lower.

## 4. Conclusions

Efficient identification of the different compounds in the sample is crucial for subsequent processes using microalgal lipids. Furthermore, LC-MS and GC-MS have been described as the best methods for identifying lipid classes and the fatty acid profile in the samples.

A new pretreatment method based on the combination of synergistic enzymes and ultrasounds was studied not only in terms of oil yield but also in terms of chemical composition regarding its influence on the amount and enrichment of polar lipids and omega-3 PUFAs in the extracts by comparing different alternative extraction techniques, including PLE, MAE, and UAE. LC-MS made it possible to identify the main polar lipids (GL and PL), whereas GC-MS showed that the major fatty acid in the samples was EPA, indicating that the extracts were rich in omega-3 fatty acids, as the n3/n6 ratio showed. As major results, the combined pretreatment increased the extraction of polar lipids, both glycolipids and PE, supporting the need for optimized pretreatment before the extraction of bioactive compounds.

The scope of this study was intentionally limited to lipid bioactives to allow for a deeper and more detailed analysis of their classes and composition. While LC-MS and GC-MS are capable of detecting other nutraceuticals, such as polyphenols or flavonoids, we opted to narrow our focus for a more targeted investigation. Further studies could expand this work to include additional compound classes.

## Figures and Tables

**Table 1:** LC-MS analysis of *N. gaditana* extract: data for retention time, ions, *m*/*z* values, error, adducts, and identified compounds.

Retention Time (min)	Observed *m*/*z*	Theoretical *m*/*z*	Error (ppm)	Adduct Ion Type *m*/*z*	Type of Second Adduct	Second Adduct *m*/*z*	Compound or Lipid Type	Identified Compound	Confidence	Comments
3.0	205.0682	205.0680	1.2	[M+Na]+		-	Manitol Derivative	C6H14NaO6 (sodium Manitol derivative)	High	Structure suggested by SciFinder.
3.2	205.0680	205.0680	0.0	[M+Na]+		-	Manitol Derivative	C6H14NaO6 (isomer of previous compound)	High	Identified as isomer with 91.14% score.
3.4	197.1171	197.1171	0.0	[M+H]+	[M+Na]+	219.0988	Fatty Acid Derivative	C11H17O3	High	Identified with 100% score in SciFinder. Possible fatty acid derivative.
4.7	494.3269	494.3270	−0.2	[M+NH4]+		-	Lipid With Phosphates	C31H44NO4, C23H50N3O4P2	High	High confidence with 100% score. C23H50N3O4P2 also identified.
4.9	520.3640	520.3645	−0.1	[M+H]+		-	Lipid With Ammonium	C27H49NO6	Medium	Score of 73.32%. Identified as possible phospholipid derivative.
8.1	282.2791	282.2789	0.7	[M+H]+		-	Amide (Erucamide)	Octadecenamide	High	Found in blank, as well; possible contamination or common background signal.
13.8	338.3415	338.3420	−0.2	[M+H]+		-	Erucamide	Erucamide	High	Typical of plastic material additive; high confidence.
17.2	730.5373	730.5370	0.4	[M+H]+	[M+Na]+	752.5226	Lipid Phosphates	C34H78N5O7P2	High	Identified with 100% score.
18.1	804.5773	804.5773	0.0	[M+NH4]+		-	Lipid	C50H78NO7	Medium	Possibility of ammonium adduct. Score of 76.08%.
19.7	806.5927	806.5925	0.2	[M+NH4]+		-	Lipid	C50H80NO7	Medium	Similar to previous peak; ammonium adduct; possibly an isomer.
20.2	758.5687	758.5690	−0.4	[M+NH4]+		-	Lipid Phosphates	C34H85N3O10PS	Low	Possible diacylglycerol phosphate, with low confidence due to fragmentation.
24.0	840.5619	840.5624	−0.6	[M+NH4]+	[M+Na]+	845.5173	Galactosyl Diacyl Glycerol (MGDG)	C49H74NaO10	Low	Possibility of ammonium adduct.
28.4	600.5194	600.5190	0.6	[M+NH4]+	[M+Na]+	605.4752	Triglyceride Ester	C35H70NO6	Medium	Possible triglyceride esterified with fatty acids; moderate confidence.
30.5	548.5028	548.5027	0.2	[M+NH4]+	[M+Na]+	553.4586	Lipid Derivative	C35H66NO3	High	Identified with high confidence as a triglyceride ester.
33.2	871.5726	871.5725	0.1	[M+H]+		-	Phytopigment	Pheophytin A	High	Identified with a score of 79.34%; good match with the literature.
34.4	871.5719	871.5720	0.0	[M+H]+		-	Phytopigment	Pheophytin A isomer	Medium	Possible isomer of Pheophytin A; could indicate slight matrix effect.
35.1	918.8105	918.8100	0.5	[M+NH4]+		-	Lipid Ester	C57H108NO7	Low	Esterified lipid with fatty acids, identified with low confidence.
36.2	813.5666	813.5660	0.7	[M+H]+	2 [M+H]+	1626.12	Lipid	C52H77O7	Medium	Identified as lipid ester with 54.27% score; low confidence due to fragmentation.
36.8	818.7220	818.7223	−0.4	[M+NH4]+		-	Lipid Phosphate	C45H97N5O5P	High	High confidence; identified as lipid with nitrogen; [M+NH4]+ clearly dominant.
37.5	820.7375	820.7380	−0.6	[M+NH4]+	[M+Na]+	825.6935	Lipid	C51H98NO6	Medium	Triglyceride derivative; possible fatty acid ester; moderate confidence.
38.2	848.7688	848.7685	0.4	[M+NH4]+	[M+Na]+	853.7249	Lipid Ester	C53H102NO6	Medium	Esterified lipid with high score but moderate confidence.
39.0	902.8166	902.8165	0.1	[M+NH4]+	[M+Na]+	907.7715	Lipid Phosphates	C56H104N4O8P	Low	Possible triglyceride with ammonium adduct; further validation needed.

**Table 2:** Lipid classes’ composition using different extraction techniques coupled with the optimized pretreatment.

Extraction Technique	GL (%) ^1^	GL (mg) ^2^	Lutein (mg) ^2^	EPA (%) ^1^
PLE				
Initial time	22.82 ^a^	112.73 ± 0.93 ^a^	4.34 ± 0.11 ^a^	35.24 ^a^
Combined pretreatment	33.46 ^b^	186.54 ± 2.11 ^b^	8.95 ± 0.08 ^b^	37.29 ^b^
UAE				
Initial time	33.51 ^b^	101.01 ± 1.13 ^c^	7.55 ± 0.45 ^c^	29.38 ^c^
Combined pretreatment	55.52 ^c^	133.36 ± 3.01 ^d^	8.36 ± 0.26 ^c^	38.63 ^b^
MAE				
Initial time	20.47 ^a^	110.57 ± 1.12 ^a^	5.79 ± 0.17 ^d^	34.52 ^d^
Combined pretreatment	38.90 ^d^	170.38 ± 0.89 ^e^	8.56 ± 0.63 ^e^	36.46 ^e^

^1^ GL (%) and EPA (%) expressed as a relative percentage of total peak areas in the chromatogram; ^2^ GL and lutein expressed as mg per g extract; data are expressed as the mean of at least three repetitions ± SD; lowercase letters (a–e) indicate significant differences between values within the same column. Different letters denote that the corresponding values are statistically different (*p* < 0.05, according to the applied multiple comparison test).

**Table 3:** PLE initial time GC-MS analysis as an example of the fatty acid composition, expressed as a percentage of total fatty acids, was determined through gas chromatography–mass spectrometry (GC-MS) analysis of the extracts produced from *Nannochloropsis gaditana*.

Identified Fatty Acid	Rt (min)	%FAME
14:0	9.70	4.74
16:0	11.77	16.64
16:1	12.50	23.07
18:0	14.59	0.49
18:1	15.59	4.17
18:2	17.12	3.37
20:3	21.90	0.51
20:4	22.69	7.92
20:5	24.61	37.38
SFA		21.87
MUFA		27.24
PUFA		49.18
n-3		37.89
n-6		11.29
n-6/n-3		0.30

“Rt” refers to the retention time, and the abbreviations used are as follows: SFA for saturated fatty acids, MUFA for monounsaturated fatty acids, and PUFA for polyunsaturated fatty acids. The results are expressed as a percentage of the total content, indicating the relative composition of each type of fatty acid. The values provided represent the mean ± standard deviation and are based on three determinations.

## Data Availability

The original contributions presented in the study are included in the article/Supplementary Materials, further inquiries can be directed to the corresponding author.

## Supplementary Materials

The following supporting information can be downloaded at: www.mdpi.com/article/10.3390/foods13244117/s1.
