# Supplementary material for: Efficient Green Extraction of Nutraceutical Compounds from Nannochloropsis gaditana: A Comparative Electrospray Ionization LC-MS and GC-MS Analysis for Lipid Profiling
Source: Foods. 2024 Dec 19;13(24):4117. doi: 10.3390/foods13244117 (PMC11675803; doi:10.3390/foods13244117)

The GC-MS analysis chromatogram of the *Nannochloropsis gaditana* sample presented in the manuscript, illustrating the separation and identification of various compounds. This chromatogram serves as a key tool for understanding the complex lipid profile extracted from the sample, providing insights into the bioactive compounds present:

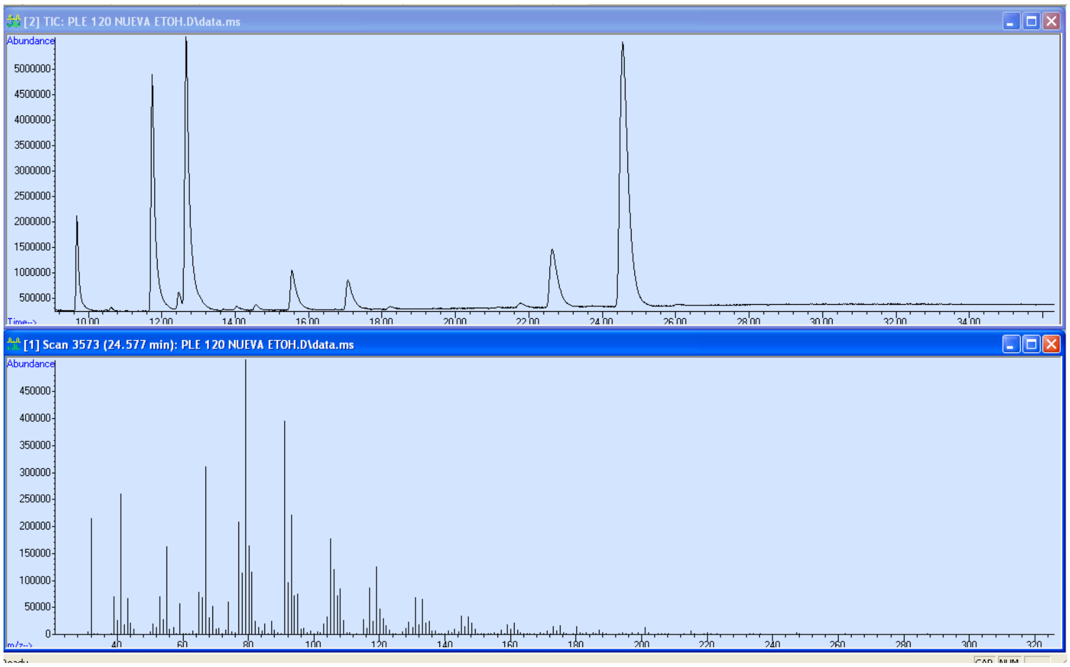

Peak identification in the GC-MS chromatogram was exemplified using the last chromatographic peak, accompanied by its corresponding data and spectral analysis. This approach highlights how we systematically identify and analyze each peak, ensuring accurate interpretation of the compounds present in the sample:

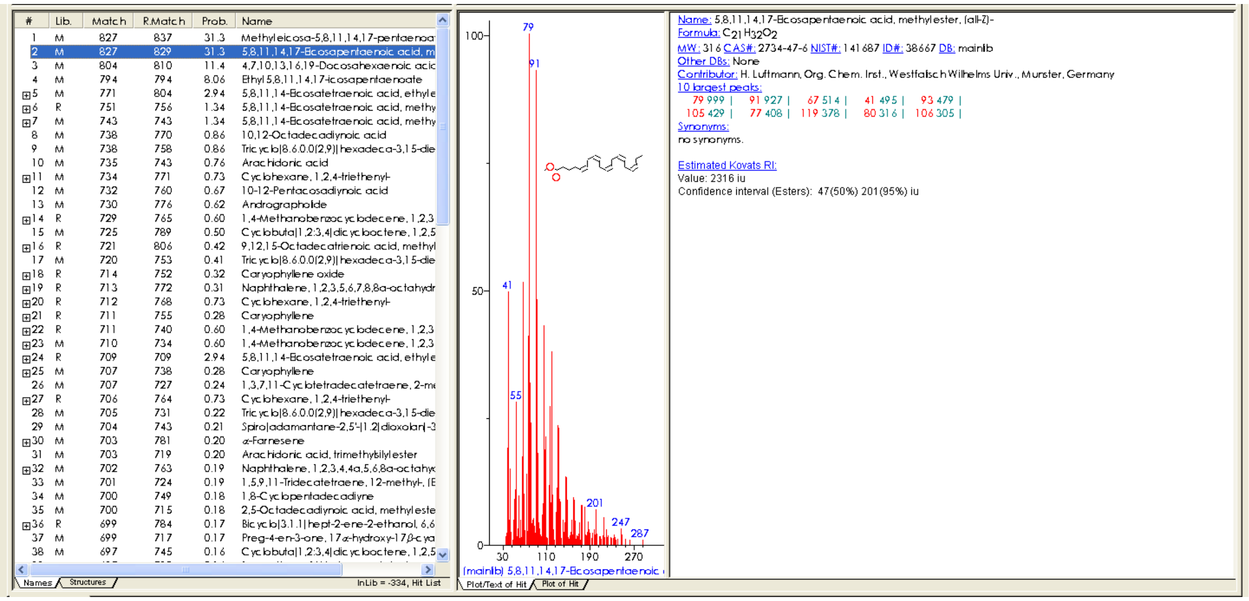

Supplement: Supplementary file 1 [file foods-13-04117-s001.zip › MS Results/GC-MS data .pdf]
