# Supplementary material for: Efficient Green Extraction of Nutraceutical Compounds from Nannochloropsis gaditana: A Comparative Electrospray Ionization LC-MS and GC-MS Analysis for Lipid Profiling
Source: Foods. 2024 Dec 19;13(24):4117. doi: 10.3390/foods13244117 (PMC11675803; doi:10.3390/foods13244117)
Supplement: Supplementary file 1 [file foods-13-04117-s001.zip › MS Results/HPLC-MS PLE -Results-MC/Hoja de resultados.pdf]

Nombre y dirección del laboratorio	Identificación del Cliente
Laboratorio de Espectrometría de Masas<br>Servicio Interdepartamental de Investigación (SIdI)<br>Facultad de Ciencias, módulo C-IX 1ª planta<br>Universidad Autónoma de Madrid<br>28049 Madrid	Francisco Javier Señorans Rodríguez<br>UAM. Facultad de Ciencias<br>Química Física Aplicada (Ciencias de la alimentación)

**Número ENTRADA: 120-12712**
**Identificación de las muestras: PLE MC**
**Tipo de Análisis: HPLC-MS**
**Referencia de los ensayos: LC2167**
**RESULTADOS**

La muestra con referencia **PLE MC** fue analizada mediante HPLC-MS empleando una fuente de Electrospray para producir la ionización de la muestra. Para la separación cromatográfica se empleó una columna Luna de 15 cmx 2.1 mm y 5 um.
Las fases móviles empleadas fueron las siguientes: En el canal A AcONH4 10 mM: Isopropanol (1:1) +0.1 % de fórmico. En el canal B: Isopropanol+0.1% de ácido fórmico. Se realizó un gradiente en 43 min partiendo de 30 % de B hasta 100 % B a un flujo de 0.2 ml/min.
La muestra se disolvió en etanol a una concentración de 0.1 mg/ml. Los resultados obtenidos se detallan a continuación:

Pico a 3.0 min: Predomina en el espectro el pico a m/z 205.0682 que podría corresponder a la composición C6H14NaO6 que podría ser el [M+Na]+ de un derivado de manitol de acuerdo con la búsqueda en la base de datos Scifinder. Se observa por otra parte un posible [M+H]+ a m/z 236.1494 acompañado de un [M+Na]+ a m/z 258.1314 que podría corresponder a una composición $C_{10}H_{21}NNaO_5$. Se adjuntan las estructuras que propone la base de datos Scifinder para esa fórmula molecular.

Pico a 3.2 min.: Se observa un isómero del producto anterior a m/z 205.0680 correspondiente a la composición $C_6H_{14}NaO_6$ con 1.2 ppm de error y un score de 91.14%. Se encuentra también la relación m/z 236.1492 ($C_{10}H_{22}NO_5$ con un score de 100%)

Pico a 3.4 min: Se observa como pico base del espectro el de relación m/z 197.1171, posible [M+H]+ por presencia de [M+Na]+ a m/z 219.0988. Podría corresponder a una composición $C_{11}H_{17}O_3$ con un score de 100 % y -0.8 ppm de error. Se adjuntan las 10 composiciones que encuentra la base de datos Scifinder ordenadas por número de referencias.

Pico a 4.7 min.: Presenta un pico base a m/z 494.3269 que podría corresponder a la composición $C_{31}H_{44}NO_4$ con un score de 50.36% y -0.8 % de error o a la

composición $C_{23}H_{50}N_3O_4P_2$ con un score de 100% y 0.5 ppm de error. Si se tratase de un [M+NH4]+ la fórmula molecular podría se $C_{31}H_{40}O_4$.

Pico a 4.9 min.: Pico base del espectro a m/z 520.3640 [M+H]+. Se observa también [M+Na]+ a m/z 542.3465. Este compuesto podría tener la composición $C_{27}H_{49}NO_6$ de acuerdo con los errores encontrados en los listados de composiciones moleculares para el [M+H]+ -1.5 ppm, mσ=12.4 y score de 73.32%. El score del 100% lo da la composición $C_{27}H_{52}N_3NaO_3P$ para el [M+NH]+ aunque parece poco probable.

Pico a 8.1 min.: Pico a 282.2791 como [M+H]+ de octadecenamida también encontrada en el blanco. [M+Na]+ a m/z 304.2612 y [M+K]+ a m/z 320.2354

Pico a 13.8 min, Picos a 338.3415 y 675.6766 típicos de la molécula Erucamida, aditivo de los materiales de tipo plástico empleados en el laboratorio, que también aparece en el blanco.

Pico a 17.2 min.: Se observan al menos 2 productos posibles: [M+H]+ con pico base a m/z 730.5373 al estar acompañado del 752.5226 [M+Na]+ Posible composición $C_{34}H_{78}N_5O_7P_2$ para el [M+H]+ con un score del 100% y -0.3ppm de error. La base de datos Scifinder no encuentra ninguna estructura para esta composición. Aparece además otro pico a m/z 758.5374 con posibles composiciones $C_{34}H_{69}N_{17}P_5$ con un score del 100% o $C_{42}H_{78}NNaO_8P$ con un score de 48.68%.

Pico a 18.1 min.: Se observa el pico a m/z 804.5773 que podría ser un $[M+NH_4]^+$ de acuerdo con lo ya observado en la muestra FOLCH. Posibles composiciones para este ion $C_{50}H_{78}NO_7$ (0.0 ppm, mσ=29.7 score= 76.08), $C_{43}H_{83}NO_{10}P$ (-2.9 ppm, mσ=17.6 y score= 24.09) o $C_{45}H_{75}N_9O_2P$ (0.4 ppm, mσ=10.9 y score= 100%).

Se adjuntan las composiciones que sugiere la base de datos Scifinder para las dos primeras formulas moleculares. Para la tercera no se encuentra ninguna composición.

Pico a 19.7 min.: Se observa el pico a m/z 806.5927. Por similitud con lo observado en el pico anterior y lo observado en la muestra FOLCH podría tratarse de un $[M+NH_4]^+$. Se identifican dos posibles compuestos que podrían corresponder a este ion pseudomolecular descartando la presencia de nitrógeno en su composición.

1.- $C_{50}H_{80}NO_7$ 0.3 ppm mσ=28.5 score= 60.38
2.- $C_{43}H_{85}NO_{10}P$ -2.6 ppm mσ=12.6 y score= 27.70%

Pico a 20.2 min: Se observa el pico a m/z 758.5687 que podría tener las siguientes composiciones moleculares si se tratase de un $[M+NH_4]^+$ y la molécula no presentase nitrógeno en su composición.:

1.-$C_{42}H_{81}NO_8P$ 1.0 ppm de error, 27.1 mσ, score= 46.81%. Diacil glycerol fosfato.
2.-$C_{31}H_{84}NO_{18}$ -0.5 ppm, 31.9 mσ score= 51.12%. No se encuentra estructura
3.- $C_{32}H_{88}NO_3S_2$ 0.7 ppm, 39.5 mσ score= 48.95%. No se encuentra estructura al eliminar el amonio.

El score 100% lo da la composición $C_{34}H_{85}N_3O_{10}PS$, pero no se encuentran estructuras probables en Scifinder al eliminar el amonio de la composición.

Pico a 28.4 min.: Se observa posible [M+NH_4]+ a m/z 600.5194 y [M+Na]+ a m/z 605.4752. Posibles fórmulas moleculares para [M+NH4]+:

1.- $C_{35}H_{70}NO_6$ 0.6 ppm de error, mσ= 13.2 y score= 90.26%. Posible triglicérido esterificado con ácidos grasos de acuerdo con Scifinder para la composición $C_{35}H_{66}O_6$ al restar el amonio.

2.- $C_{27}H_{67}N_{11}NaS$ score= 100 %. No se encuentra ninguna estructura en Scifinder. Además, dado el número tan elevado de nitrógeno no parece muy probable.

Pico a 30.5 min.: Se observa pico a m/z 548.5028 como posible [M+NH_4]+ como pico base del espectro y 553.4586 como [M+Na]+

Para el [M+NH_4]+ podría tener las composiciones $C_{35}H_{66}NO_3$ (1.6 ppm, mσ=6.8 y score= 90.99%) o $C_{28}H_{71}NO_6P$ (-2.7 ppm. mσ= 31.5 y score: 37.80%) (Para esta última composición no aparecen estructuras en Scifinder)

El [M+Na]+ podría tener la composición $C_{35}H_{62}NaO_3$ (1.0 ppm. mσ=11.2 y score= 85.67%)

En este mismo pico cromatográfico parece coeluir otro producto adicional con [M+H]+ 628.55º5 y [M+Na]+ 633.5067 con composiciones $C_{37}H_{74}NO_6$ (0.9ppm, mσ 3.0, score=100%) y $C_{37}H_{70}$ NaO_6 (-0.4ppm y score 100%) respectivamente que podría corresponder a un triglicérido esterificado con ácidos grasos.

Pico a 33.2 min.: Pico a 871.5726 ya identificado en muestras FOLCH. De acuerdo con la bibliografía podría coincidir con la molécula Pheophytin A con composición $C_{55}H_{75}N_4O_5$ [M+H]+ 0.7 ppm. mσ= 25.9 y score 79.34%.

Aparecen otras composiciones en el listado de posibles fórmulas moleculares con un Score mayor. Cabe señalar las siguientes $C_{57}H_{80}N_2OPS$ con un score de 100, $C_{54}H_{72}N_8ONa$ (con -0.5 ppm de error y un score de 91.84%) y $C_{54}H_{79}O_9$ (con un error de -0.8 ppm, mσ= 33.2 y score del 81.10 %).

Pico a 34.4 min. Se observa en el espectro de masas pico a m/z 871.5719 que podría ser un isómero del producto anterior.

Pico a m/z 35.1 min Pico base a 918.8105 [M+NH4]+ y 923.7662[M+Na]+. Se observa adición de isopropilo, probablemente procedente de la fase móvil a m/z 960.8574.

Composiciones probables para [M+NH_4]+:

1.- $C_{57}H_{108}NO_7$ (1.6 ppm, mσ=17.3 y un score= 56.54%) Típica de.ésteres con moléculas de ácido graso de mayor tamaño molecular y la molécula de glicerol.

2. $C_{50}H_{113}NO_{10}P$ (-0.9 ppm, mσ 28.1 y score= 52.50%). No se encuentran estructuras en Scifinder al quitarle el amonio.

3.- $C_{54}H_{100}N_{11}O$ score 100 pero poco probable.

Pico a 36.2 min: Se observa en el espectro de masas pico a m/z 813.5666 como [M+H]+ acompañado del pico a m/z 1626.12 [2M+H]+. Posibles composiciones para [M+H]+ son las siguientes

1.- $C_{42}H_{86}O_{10}PS$ (1.0 ppm, mσ = 30.8, score = 54.27%)

2.- $C_{52}H_{77}O_{7}$ (-0.3ppm, mσ= 25.3, score= 65.05%)

3.- $C_{44}H_{83}N_{2}O_{7}P_{2}$ (0.5 ppm, mσ= 20.3, score= 66.78%)

4.- $C_{47}H_{74}N_{8}O_{2}P$ (0.1 ppm, mσ= 6.8 score= 100%). Parece poco probable

La única composición que devuelve resultados al buscarla en Scifinder es C52H76O7.

Pico a 36.8 min.: Se observa posible [M+NH_4]+ a m/z 818.7220. En menor proporción el [M+Na]+ a m/z 823.6774. Posibles composiciones para [M+NH_4]+ son las siguientes:

1.- $C_{45}H_{97}N_{5}O_{5}P$ (0.3 ppm, mσ= 9.2, score = 100%)

2.- $C_{44}H_{101}NO_{9}P$ (-1.4 ppm, mσ = 14.5 y score = 57.21 %)

3.- $C_{51}H_{96}NO_{6}$ (1.5 ppm, mσ= 34.6 y score 33.36%). La fórmula resultante sin amonio $C_{51}H_{92}O_{6}$ da como estructuras probables en Scifinder triglicéridos.

Pico a 37.5 min. Se observa pico a m/z 820.7375 correspondiente a [M+NH_4]+ acompañado del correspondiente [M+Na]+ a m/z 825.6935. Las composiciones probables para [M+NH4]+ teniendo en cuenta que la molécula presenta ausencia de N, P y S podrían ser:

1.- $C_{51}H_{98}NO_{6}$ (1.7 ppm, mσ 28.7 y score = 43.34%). Al restarle el amonio queda la composición $C_{51}H_{94}O_{6}$ que podría corresponder a otro triglicérido según Scifinder.

2.- $C_{44}H_{103}NO_{9}P$ (-1.2 ppm, mσ= 29.2 score= 53.90%). No devuelve estructuras Scifinder al restarle el amonio.

Además de este producto parece coeluir otro con [M+H]+ 846.7532 y [M+Na]+ 868.7374 y que podría tener la composición para el [M+NH4]+ $C_{46}H_{105}NO_{9}$ (-1.3 ppm, mσ= 35.2 y score = 55.24%) que no devuelve ninguna composición en Scifinder o $C_{53}H_{100}NO_{6}$ (1.5 ppm, mσ= 42.8 y score=40.70%) y [M+Na]+ podría tener la composición ($C_{53}H_{99}NNaO_{6}$.), un derivado de ácidos grasos.

Pico a 38.2 min.: Coeluyen varios productos:

Posible [M+NH_4]+/[M+Na]+ a m/z 848.7688 y 853.7249 respectivamente con composiciones probables para [M+NH_4]+ $C_{53}H_{102}NO_{6}$ (1.6 ppm, mσ= 32.1 y 35.52% de score) que se corresponde con un posible triglicérido esterificado con ácidos grasos y $C_{46}H_{107}NO_{9}P$ (--1.2 ppm, mσ 18.3 y score = 62.77%) que no devuelve resultados en la base de datos.

Pico a 39.0 min. Coeluyen varios productos:

Posible [M+NH4]+/ [M+Na]+ a m/z 902.8166 y 907.7715 que parece corresponder a la composición $C_{57}H_{108}NO_6$ (0.5 ppm, mσ= 65.2 y score= 100%) que se relaciona con el [M+NH4]+ de un triglicérido esterificado con ácidos grasos.

Otro posible [M+NH_4]+/ [M+Na]+ a m/z 876.8010 y 881.7584 con posible composición $C_{55}H_{106}NO_6$ para el [M+NH4]+ parece relacionarse con otro triglicérido con otros ácidos grasos

Pico a 39.5 min. Posible [M+NH_4]+/ [M+Na]+ a m/z 904.8311 y 909.7885 respectivamente. Composición para [M+NH_4]+ $C_{57}H_{110}NO_6$ (1.9 ppm, mσ 16.4 y score 41.83%). Otras posibles composiciones $C_{53}H_{116}N_3OP_2S$ con un score de 96.88 % y $C_{50}H_{115}NO_9P$ con un score de 54.41%. La composición $C_{57}H_{106}O_6$ resultante de sustraer el NH_4+ se relaciona según la búsqueda en Scifinder con otro triglicérido esterificado de nuevo con ácidos grasos. El listado que ofrece la base de datos Scifinder está ordenado en todos los casos por número de referencias encontradas en la literatura.
