# Supplementary material for: Efficient Green Extraction of Nutraceutical Compounds from Nannochloropsis gaditana: A Comparative Electrospray Ionization LC-MS and GC-MS Analysis for Lipid Profiling
Source: Foods. 2024 Dec 19;13(24):4117. doi: 10.3390/foods13244117 (PMC11675803; doi:10.3390/foods13244117)
Supplement: Supplementary file 1 [file foods-13-04117-s001.zip › MS Results/HPLC-MS PLE -Results-MC/Pico a 18.1 min_C43H79NO10P.pdf]

CAS SciFinder^n

Task History

Initiating Search

November 25, 2022, 11:31AM

Substances:

Advanced Search:

Molecular Formula: C43H79O10P

Search Tasks

Task	Search Type	View
Exported: Returned Substance Results (5)	Substances	View Results

Copyright © 2022 American Chemical Society (ACS). All Rights Reserved.
Internal use only. Redistribution is subject to the terms of your SciFinder^n License Agreement and CAS information Use Policies.

CAS SciFinder^{n}

Substances (5)

View in SciFinder^{n}

1

959392-48-4

959392-47-3

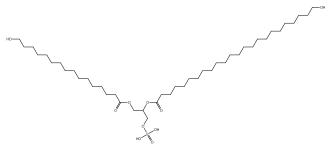

$C_{43}H_{79}O_{10}P$

Tetracosatrienoic acid, 24-hydroxy-, 2-[(16-hydroxy-1-oxohexadecyl)oxy]-1-[(phosphonooxy)methyl]ethyl ester, (*Z*,*Z*,*Z*)-

1 Reference | 0 Reactions | 0 Suppliers

There are no Key Physical Properties to display for this substance.

2

336786-70-0

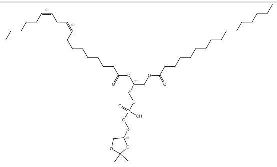

Absolute stereochemistry shown
Double bond geometry shown

$C_{43}H_{79}O_{10}P$

(1*R*)-1-[[[[[(4*R*)-2,2-Dimethyl-1,3-dioxolan-4-yl]methoxy]hydroxyphosphinyl]oxy]methyl]-2-[(1-oxohexadecyl)oxy]ethyl (9*Z*,12*Z*)-9,12-octadecadienoate

1 Reference | 0 Reactions | 0 Suppliers

Key Physical Properties	Value	Condition
Molecular Weight	787.06	-
Boiling Point (Predicted)	778.9±70.0 °C	Press: 760 Torr
Density (Predicted)	1.022±0.06 g/cm^3	Temp: 20 °C; Press: 760 Torr
pKa (Predicted)	1.38±0.50	Most Acidic Temp: 25 °C

3

126716-38-9

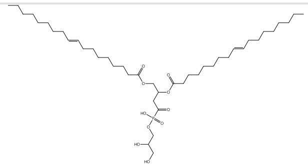

$C_{43}H_{79}O_{10}P$

9-Octadecenoic acid (9*Z*)-, (1*R*)-1-[2-[[(2*S*)-2,3-dihydroxypropoxy]hydroxyphosphinyl]-2-oxoethyl]-1,2-ethanediyl ester

1 Reference | 0 Reactions | 0 Suppliers

Key Physical Properties	Value	Condition
Molecular Weight	787.06	-
Boiling Point (Predicted)	813.3±75.0 °C	Press: 760 Torr
Density (Predicted)	1.044±0.06 g/cm^3	Temp: 20 °C; Press: 760 Torr
pKa (Predicted)	0.39±0.50	Most Acidic Temp: 25 °C

4

123736-75-4

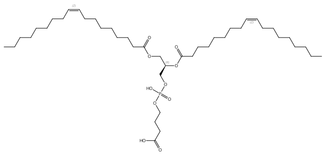

Absolute stereochemistry shown
Double bond geometry shown

$C_{43}H_{79}O_{10}P$

5,7,11-Trioxa-6-phosphanonacos-20-enoic acid, 6-hydroxy-12-oxo-9-[(1-oxo-9-octadecenyl)oxy]-, 6-oxide, [*R*-(*Z*,*Z*)]-

1 Reference | 0 Reactions | 0 Suppliers

Key Physical Properties	Value	Condition
Molecular Weight	787.06	-
Boiling Point (Predicted)	804.8±75.0 °C	Press: 760 Torr
Density (Predicted)	1.033±0.06 g/cm^3	Temp: 20 °C; Press: 760 Torr
pKa (Predicted)	1.42±0.50	Most Acidic Temp: 25 °C

5

2342575-51-1

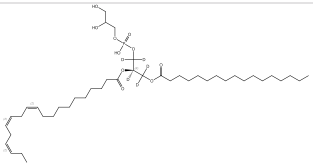

Absolute stereochemistry shown
Double bond geometry shown

$C_{43}H_{74}D_5O_{10}P$

(1*R*)-1-[[[(2,3-Dihydroxypropoxy)hydroxyphosphinyl]oxy]methyl-*d*$_2$]-2-[(1-oxoheptadecyl)oxy]ethyl-*1,2,2-d*$_3$ (11*Z*,14*Z*,17*Z*)-11,14,17-eicosatrienoate

0 References | 0 Reactions | 0 Suppliers

Key Physical Properties	Value	Condition
Molecular Weight	792.09	-

Copyright © 2022 American Chemical Society (ACS). All Rights Reserved.
Internal use only. Redistribution is subject to the terms of your SciFinderⁿ License Agreement and CAS information Use Policies.
