# Supplementary material for: Efficient Green Extraction of Nutraceutical Compounds from Nannochloropsis gaditana: A Comparative Electrospray Ionization LC-MS and GC-MS Analysis for Lipid Profiling
Source: Foods. 2024 Dec 19;13(24):4117. doi: 10.3390/foods13244117 (PMC11675803; doi:10.3390/foods13244117)
Supplement: Supplementary file 1 [file foods-13-04117-s001.zip › MS Results/HPLC-MS PLE -Results-MC/Pico a 18.1 min_C50H74O7.pdf]

CAS SciFinder^n

Task History

Initiating Search

November 25, 2022, 11:29AM

Substances:

Advanced Search:

Molecular Formula: $C_{50}H_{74}O_7$

Search Tasks

Task	Search Type	View
Exported: Returned Substance Results (5)	Substances	View Results

Copyright © 2022 American Chemical Society (ACS). All Rights Reserved.
Internal use only. Redistribution is subject to the terms of your SciFinder^n License Agreement and CAS information Use Policies.

SciFinderⁿ

Substances (5)

View in SciFinderⁿ

1

343628-09-1

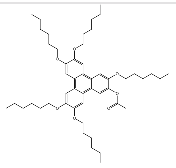

$C_{50}H_{74}O_7$

2-Triphenylenol, 3,6,7,10,11-pentakis (hexyloxy)-, 2-acetate

2 References | 3 Reactions | 0 Suppliers

Key Physical Properties	Value	Condition
Molecular Weight	787.12	-
Boiling Point (Predicted)	813.0±60.0 °C	Press: 760 Torr
Density (Predicted)	1.032±0.06 g/cm^3	Temp: 20 °C; Press: 760 Torr

2

148808-66-6

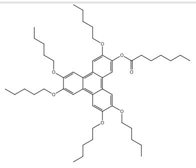

$C_{50}H_{74}O_7$

Heptanoic acid, 3,6,7,10,11-pentakis (pentyloxy)-2-triphenylenyl ester

2 References | 4 Reactions | 0 Suppliers

Key Physical Properties	Value	Condition
Molecular Weight	787.12	-
Boiling Point (Predicted)	813.0±60.0 °C	Press: 760 Torr
Density (Predicted)	1.032±0.06 g/cm^3	Temp: 20 °C; Press: 760 Torr

3

2088880-29-7

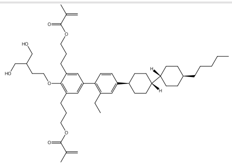

Relative stereochemistry shown

$C_{50}H_{74}O_7$

1,1'-[[2'-Ethyl-4-[4-hydroxy-3-(hydroxymethyl)butoxy]-4'-[(*trans*,*trans*)-4'-pentyl[1,1'-bicyclohexyl]-4-yl][1,1'-biphenyl]-3,5-diyl]di-3,1-propanediyl] bis(2-methyl-2-propenoate)

1 Reference | 0 Reactions | 0 Suppliers

Key Physical Properties	Value	Condition
Molecular Weight	787.12	-
Boiling Point (Predicted)	856.6±65.0 °C	Press: 760 Torr
Density (Predicted)	1.051±0.06 g/cm^3	Temp: 20 °C; Press: 760 Torr
pKa (Predicted)	14.40±0.10	Most Acidic Temp: 25 °C

4

302944-02-1

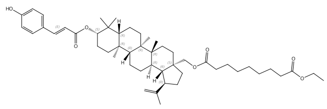

Absolute stereochemistry shown,
Rotation (+)
Double bond geometry shown

$C_{50}H_{74}O_7$

Lup-20(29)-ene-3,28-diol, 28-(ethyl nonanedioate) 3-[(2*E*)-3-(4-hydroxyphenyl)-2-propenoate], (3β)-

1 Reference | 0 Reactions | 0 Suppliers

Key Physical Properties	Value	Condition
Molecular Weight	787.12	-
Boiling Point (Predicted)	767.6±40.0 °C	Press: 760 Torr
Density (Predicted)	1.11±0.1 g/cm^3	Temp: 20 °C; Press: 760 Torr
pKa (Predicted)	9.68±0.26	Most Acidic Temp: 25 °C
Spectra		

5

173426-32-9

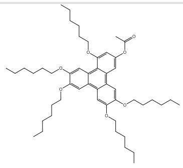

$C_{50}H_{74}O_7$

2-Triphenylenol, 4,6,7,10,11-pentakis(hexyloxy)-, 2-acetate

1 Reference | 0 Reactions | 0 Suppliers

Key Physical Properties	Value	Condition
Molecular Weight	787.12	-
Boiling Point (Predicted)	829.4±65.0 °C	Press: 760 Torr
Density (Predicted)	1.032±0.06 g/cm^3	Temp: 20 °C; Press: 760 Torr

Copyright © 2022 American Chemical Society (ACS). All Rights Reserved.
Internal use only. Redistribution is subject to the terms of your SciFinder^n License Agreement and CAS information Use Policies.
