# Supplementary material for: Efficient Green Extraction of Nutraceutical Compounds from Nannochloropsis gaditana: A Comparative Electrospray Ionization LC-MS and GC-MS Analysis for Lipid Profiling
Source: Foods. 2024 Dec 19;13(24):4117. doi: 10.3390/foods13244117 (PMC11675803; doi:10.3390/foods13244117)
Supplement: Supplementary file 1 [file foods-13-04117-s001.zip › MS Results/HPLC-MS PLE -Results-MC/Pico a 19.7 min C43H81O10P.pdf]

SciFinder^n

Task History

Initiating Search

November 25, 2022, 11:54AM

Substances:

Advanced Search:

Molecular Formula: C43H81O10P

Search Tasks

Task	Search Type	View
Exported: Returned Substance Results (15)	Substances	View Results

Copyright © 2022 American Chemical Society (ACS). All Rights Reserved.
Internal use only. Redistribution is subject to the terms of your SciFinder^n License Agreement and CAS information Use Policies.

CAS SciFinderⁿ

Substances (10)

View in SciFinderⁿ

1

721455-81-8

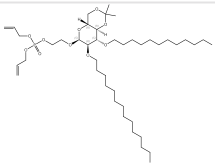

Absolute stereochemistry shown

$C_{43}H_{81}O_{10}P$

2-[[Bis(2-propen-1-yloxy)phosphinyl]oxy]ethyl 3-*O*-dodecyl-4,6-*O*-(1-methylethylidene)-2-*O*-tetradecyl-α-D-glucopyranoside

2 References | 2 Reactions | 0 Suppliers

Key Physical Properties	Value	Condition
Molecular Weight	789.07	-
Boiling Point (Predicted)	750.1±60.0 °C	Press: 760 Torr
Density (Predicted)	1.03±0.1 g/cm^3	Temp: 20 °C; Press: 760 Torr

2

102505-39-5

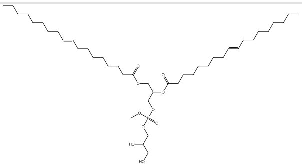

$C_{43}H_{81}O_{10}P$

9-Octadecenoic acid (9*Z*)-, 1-[[[(2,3-dihydroxypropoxy)methoxyphosphinyl]oxy]methyl]-1,2-ethanediyl ester

2 References | 2 Reactions | 0 Suppliers

Key Physical Properties	Value	Condition
Molecular Weight	789.07	-
Boiling Point (Predicted)	788.5±60.0 °C	Press: 760 Torr
Density (Predicted)	1.022±0.06 g/cm^3	Temp: 20 °C; Press: 760 Torr
pKa (Predicted)	13.07±0.20	Most Acidic Temp: 25 °C

3

2416959-90-3

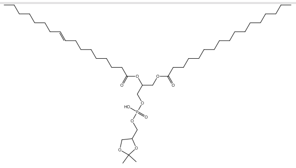

$C_{43}H_{81}O_{10}P$

1-[[[[(2,2-Dimethyl-1,3-dioxolan-4-yl)methoxy]hydroxyphosphinyl]oxy]methyl]-2-[(1-oxoheptadecyl)oxy]ethyl 9-heptadecenoate

1 Reference | 5 Reactions | 0 Suppliers

Key Physical Properties	Value	Condition
Molecular Weight	789.07	-
Boiling Point (Predicted)	779.4±70.0 °C	Press: 760 Torr
Density (Predicted)	1.016±0.06 g/cm^3	Temp: 20 °C; Press: 760 Torr
pKa (Predicted)	1.38±0.50	Most Acidic Temp: 25 °C

4

2376106-13-5

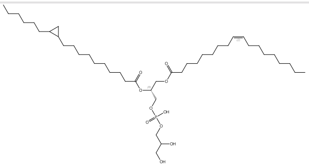

Absolute stereochemistry shown
Double bond geometry shown

$C_{43}H_{81}O_{10}P$

1 Reference | 0 Reactions | 0 Suppliers

Key Physical Properties	Value	Condition
Molecular Weight	789.07	-
Boiling Point (Predicted)	805.9±75.0 °C	Press: 760 Torr
Density (Predicted)	1.047±0.06 g/cm^3	Temp: 20 °C; Press: 760 Torr
pKa (Predicted)	1.39±0.50	Most Acidic Temp: 25 °C

5

2365355-54-8

2365355-53-7

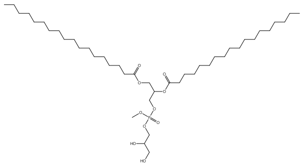

$C_{43}H_{81}O_{10}P$

1 Reference | 0 Reactions | 0 Suppliers

There are no Key Physical Properties to display for this substance.

6

2365355-52-6

There are no Key Physical Properties to display for this substance.

2365355-51-5

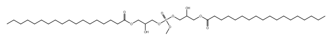

$C_{43}H_{81}O_{10}P$

1 Reference | 0 Reactions | 0 Suppliers

7

2365355-50-4

There are no Key Physical Properties to display for this substance.

2365355-49-1

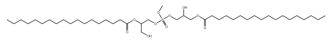

$C_{43}H_{81}O_{10}P$

1 Reference | 0 Reactions | 0 Suppliers

8

2365355-48-0

There are no Key Physical Properties to display for this substance.

2365355-47-9

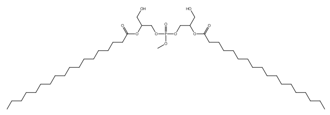

$C_{43}H_{81}O_{10}P$

1 Reference | 0 Reactions | 0 Suppliers

9

1303612-21-6

1303612-20-5

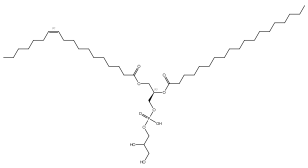

Absolute stereochemistry shown
Double bond geometry shown

$C_{43}H_{81}O_{10}P$

Nonadecenoic acid, (1*R*)-1-[[[(2,3-dihydroxypropoxy)hydroxyphosphinyl]oxy]methyl]-2-[[(11*Z*)-1-oxo-11-octadecen-1-yl]oxy]ethyl ester, (*Z*)-

1 Reference | 0 Reactions | 0 Suppliers

There are no Key Physical Properties to display for this substance.

10

1223590-04-2

1223590-03-1

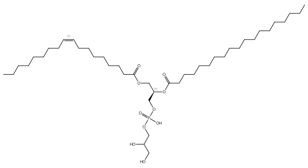

Absolute stereochemistry shown
Double bond geometry shown

$C_{43}H_{81}O_{10}P$

Nonadecenoic acid, (1*R*)-1-[[[(2,3-dihydroxypropoxy)hydroxyphosphinyl]oxy]methyl]-2-[[(9*Z*)-1-oxo-9-octadecen-1-yl]oxy]ethyl ester, (*Z*)-

1 Reference | 0 Reactions | 0 Suppliers

There are no Key Physical Properties to display for this substance.

Spectra

Copyright © 2022 American Chemical Society (ACS). All Rights Reserved.
Internal use only. Redistribution is subject to the terms of your SciFinder^{n} License Agreement and CAS information Use Policies.
