# Supplementary material for: Efficient Green Extraction of Nutraceutical Compounds from Nannochloropsis gaditana: A Comparative Electrospray Ionization LC-MS and GC-MS Analysis for Lipid Profiling
Source: Foods. 2024 Dec 19;13(24):4117. doi: 10.3390/foods13244117 (PMC11675803; doi:10.3390/foods13244117)
Supplement: Supplementary file 1 [file foods-13-04117-s001.zip › MS Results/HPLC-MS PLE -Results-MC/Pico a 19.7 min C50H76O7.pdf]

CAS SciFinder^n Task History

Initiating Search

November 25, 2022, 11:52AM

Substances:

Advanced Search:

Molecular Formula: c50h76o7

Search Tasks

Task	Search Type	View
Exported: Returned Substance Results (8)	Substances	View Results

Copyright © 2022 American Chemical Society (ACS). All Rights Reserved.
Internal use only. Redistribution is subject to the terms of your SciFinder^n License Agreement and CAS information Use Policies.

CAS SciFinderⁿ

Substances (8)

View in SciFinderⁿ

1

1808284-18-5

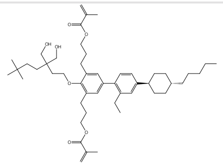

Relative stereochemistry shown

$C_{50}H_{76}O_7$

2-Propenoic acid, 2-methyl-, 3-[4-[[3,3-bis(hydroxymethyl)-6,6-dimethylheptyl]oxy]-2'-ethyl-5-[3-[(2-methyl-1-oxo-2-propen-1-yl)oxy]propyl]-4'-(*trans*-4-pentylcyclohexyl)[1,1'-biphenyl]-3-yl]propyl ester

1 Reference | 0 Reactions | 0 Suppliers

Key Physical Properties	Value	Condition
Molecular Weight	789.14	-
Boiling Point (Predicted)	843.3±65.0 °C	Press: 760 Torr
Density (Predicted)	1.028±0.06 g/cm^3	Temp: 20 °C; Press: 760 Torr
pKa (Predicted)	14.41±0.10	Most Acidic Temp: 25 °C

2

1609958-10-2

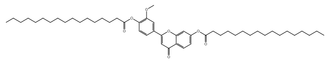

$C_{50}H_{76}O_7$

Heptadecanoic acid, 2-[3-methoxy-4-[(1-oxoheptadecyl)oxy]phenyl]-4-oxo-4*H*-1-benzopyran-7-yl ester

1 Reference | 2 Reactions | 0 Suppliers

Key Physical Properties	Value	Condition
Molecular Weight	789.14	-
Boiling Point (Predicted)	797.6±60.0 °C	Press: 760 Torr
Density (Predicted)	1.019±0.06 g/cm^3	Temp: 20 °C; Press: 760 Torr

3

1413933-89-7

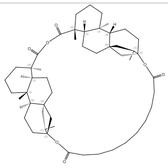

Absolute stereochemistry shown

$C_{50}H_{76}O_7$

Kauran-18-oic acid, 13,13'-[(1,10-dioxo-1,10-decanediyl)bis(oxy)]bis-, 18,18'-anhydride, (4α)-(4'α)-

1 Reference | 1 Reaction | 0 Suppliers

Key Physical Properties	Value	Condition
Molecular Weight	789.14	-
Density (Predicted)	1.15±0.1 g/cm^3	Temp: 20 °C; Press: 760 Torr

4

1028175-95-2

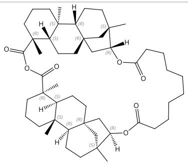

Absolute stereochemistry shown

$C_{50}H_{76}O_7$

17-Norkauran-18-oic acid, 16,16'-[(1,10-dioxo-1,10-decanediyl)bis(oxy)]bis[13-methyl-, cyclic 18,18'-anhydride, (4α,16α)-(4'α,16'α)-

1 Reference | 1 Reaction | 0 Suppliers

Key Physical Properties	Value	Condition
Molecular Weight	789.14	-
Density (Predicted)	1.15±0.1 g/cm^3	Temp: 20 °C; Press: 760 Torr

5

335457-87-9

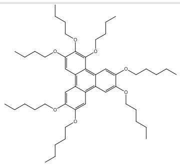

$C_{50}H_{76}O_7$

1,2,3-Tributoxy-6,7,10,11-tetrakis(pentyloxy)triphenylene

1 Reference | 1 Reaction | 0 Suppliers

Key Physical Properties	Value	Condition
Molecular Weight	789.14	-
Boiling Point (Predicted)	817.3±60.0 °C	Press: 760 Torr
Density (Predicted)	1.021±0.06 g/cm^3	Temp: 20 °C; Press: 760 Torr

6

314051-34-8

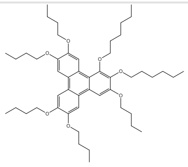

$C_{50}H_{76}O_7$

3,6,7,10,11-Pentabutoxy-1,2-bis(hexyloxy)triphenylene

1 Reference | 0 Reactions | 0 Suppliers

Key Physical Properties	Value	Condition
Molecular Weight	789.14	-
Boiling Point (Predicted)	817.3±60.0 °C	Press: 760 Torr
Density (Predicted)	1.021±0.06 g/cm^3	Temp: 20 °C; Press: 760 Torr

7

303174-01-8

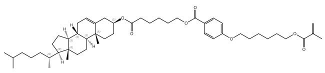

Absolute stereochemistry shown

$C_{50}H_{76}O_7$

Cholest-5-en-3-ol (3β)-, 6-[[4-[[6-[(2-methyl-1-oxo-2-propenyl)oxy]hexyl]oxy]benzoyl]oxy]hexanoate

1 Reference | 0 Reactions | 0 Suppliers

Key Physical Properties	Value	Condition
Molecular Weight	789.14	-
Boiling Point (Predicted)	788.3±60.0 °C	Press: 760 Torr
Density (Predicted)	1.07±0.1 g/cm^3	Temp: 20 °C; Press: 760 Torr

8

24904-70-9

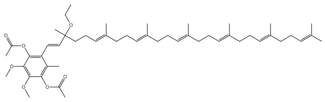

$C_{50}H_{76}O_7$

Hydroquinone, 2-(3-ethoxy-3,7,11,15,19,23,27-heptamethyl-1,6,10,14,18,22,26-octacosaheptaenyl)-5,6-dimethoxy-3-methyl-, diacetate, stereoisomer

1 Reference | 1 Reaction | 0 Suppliers

Key Physical Properties	Value	Condition
Molecular Weight	789.14	-
Boiling Point (Predicted)	796.2±60.0 °C	Press: 760 Torr
Density (Predicted)	0.988±0.06 g/cm^3	Temp: 20 °C; Press: 760 Torr

Copyright © 2022 American Chemical Society (ACS). All Rights Reserved.
Internal use only. Redistribution is subject to the terms of your SciFinder^n License Agreement and CAS information Use Policies.
