# Supplementary material for: Efficient Green Extraction of Nutraceutical Compounds from Nannochloropsis gaditana: A Comparative Electrospray Ionization LC-MS and GC-MS Analysis for Lipid Profiling
Source: Foods. 2024 Dec 19;13(24):4117. doi: 10.3390/foods13244117 (PMC11675803; doi:10.3390/foods13244117)
Supplement: Supplementary file 1 [file foods-13-04117-s001.zip › MS Results/HPLC-MS PLE -Results-MC/Pico a 20.2 min_C42H77O8P.pdf]

CAS SciFinder^n

Task History

Initiating Search

November 25, 2022, 12:42PM

Substances:

Advanced Search:

Molecular Formula: $C_{42}H_{77}O_8P$

Search Tasks

Task	Search Type	View
Exported: Returned Substance Results (3)	Substances	View Results

Copyright © 2022 American Chemical Society (ACS). All Rights Reserved.
Internal use only. Redistribution is subject to the terms of your SciFinder^n License Agreement and CAS information Use Policies.

CAS SciFinderⁿ

Substances (3)

View in SciFinderⁿ

1

2241545-70-8

27213-43-0

57-11-4

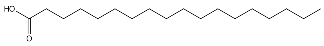

2363-71-5

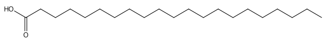

57-03-4

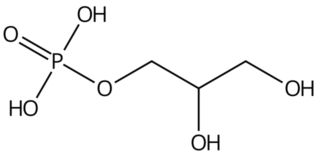

$C_{42}H_{77}O_8P$

1 Reference | 0 Reactions | 0 Suppliers

There are no Key Physical Properties to display for this substance.

2

336786-74-4

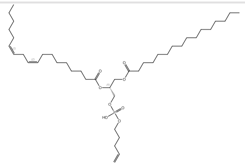

Absolute stereochemistry shown
Double bond geometry shown

$C_{42}H_{77}O_8P$

(1*R*)-1-[[[Hydroxy(4-penten-1-yloxy)phosphinyl]oxy]methyl]-2-[(1-oxohexadecyl)oxy]ethyl (9*Z*,12*Z*)-9,12-octadecadienoate

1 Reference | 0 Reactions | 0 Suppliers

Key Physical Properties	Value	Condition
Molecular Weight	741.03	-
Boiling Point (Predicted)	743.9±70.0 °C	Press: 760 Torr
Density (Predicted)	0.993±0.06 g/cm^3	Temp: 20 °C; Press: 760 Torr
pKa (Predicted)	1.45±0.50	Most Acidic Temp: 25 °C

3

2237220-49-2

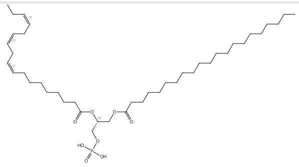

Absolute stereochemistry shown
Double bond geometry shown

$C_{42}H_{77}O_8P$

0 References | 0 Reactions | 0 Suppliers

Key Physical Properties	Value	Condition
Molecular Weight	741.03	-
Boiling Point (Predicted)	769.6±70.0 °C	Press: 760 Torr
Density (Predicted)	1.007±0.06 g/cm^3	Temp: 20 °C; Press: 760 Torr
pKa (Predicted)	1.83±0.10	Most Acidic Temp: 25 °C

Copyright © 2022 American Chemical Society (ACS). All Rights Reserved.
Internal use only. Redistribution is subject to the terms of your SciFinder^n License Agreement and CAS information Use Policies.
