# Supplementary material for: Efficient Green Extraction of Nutraceutical Compounds from Nannochloropsis gaditana: A Comparative Electrospray Ionization LC-MS and GC-MS Analysis for Lipid Profiling
Source: Foods. 2024 Dec 19;13(24):4117. doi: 10.3390/foods13244117 (PMC11675803; doi:10.3390/foods13244117)
Supplement: Supplementary file 1 [file foods-13-04117-s001.zip › MS Results/HPLC-MS PLE -Results-MC/Pico a 28.4_C35H66O6.pdf]

SciFinder^n

Task History

Initiating Search

November 25, 2022, 12:54PM

Substances:

Advanced Search:

Molecular Formula: $C_{35}H_{66}O_6$

Search Tasks

Task	Search Type	View
Exported: Returned Substance Results (73)	Substances	View Results

Copyright © 2022 American Chemical Society (ACS). All Rights Reserved.
Internal use only. Redistribution is subject to the terms of your SciFinder^n License Agreement and CAS information Use Policies.

CAS SciFinderⁿ

Substances (10)

View in SciFinderⁿ

1

28063-11-8

There are no Key Physical Properties to display for this substance.

Spectra

334-48-5

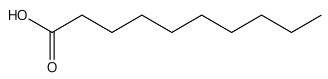

143-07-7

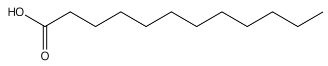

56-81-5

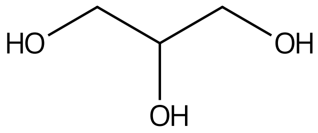

$C_{35}H_{66}O_6$

Triglyceride CCLa

34 References | 0 Reactions | 0 Suppliers

2

60138-10-5

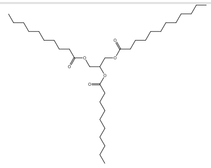

$C_{35}H_{66}O_6$

2,3-Bis[(1-oxodecyl)oxy]propyl dodecanoate

18 References | 0 Reactions | 0 Suppliers

Key Physical Properties	Value	Condition
Molecular Weight	582.90	-
Melting Point (Experimental)	0 °C	-
Boiling Point (Predicted)	600.7±22.0 °C	Press: 760 Torr
Density (Predicted)	0.944±0.06 g/cm^3	Temp: 20 °C; Press: 760 Torr

Experimental Properties

3

30283-09-1

143-07-7

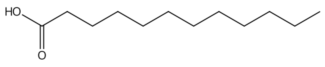

124-07-2

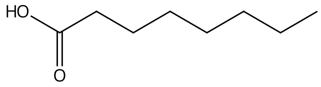

56-81-5

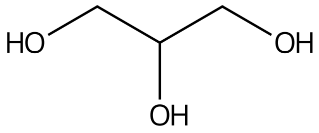

$C_{35}H_{66}O_6$

Triglyceride CyLaLa

16 References | 0 Reactions | 0 Suppliers

There are no Key Physical Properties to display for this substance.

Spectra

4

118530-12-4

143-07-7

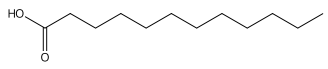

107-92-6

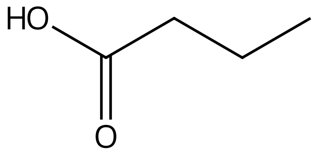

57-10-3

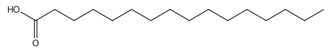

56-81-5

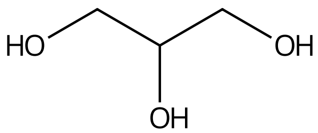

$C_{35}H_{66}O_6$

Hexadecanoic acid, ester with 1,2,3-propanetriol monobutanoate monododecanoate

15 References | 0 Reactions | 0 Suppliers

There are no Key Physical Properties to display for this substance.

5

108559-68-8

544-63-8

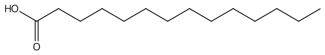

107-92-6

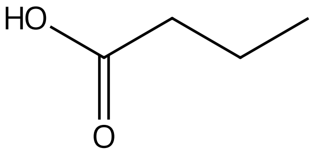

56-81-5

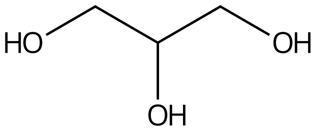

$C_{35}H_{66}O_6$

Tetradecanoic acid, diester with 1,2,3-propanetriol monobutanoate

15 References | 0 Reactions | 0 Suppliers

There are no Key Physical Properties to display for this substance.

6

150591-72-3

544-63-8

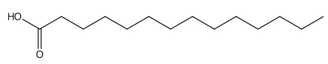

143-07-7

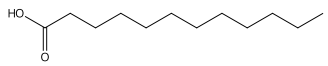

142-62-1

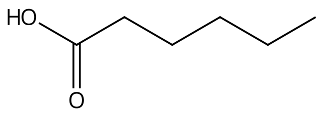

56-81-5

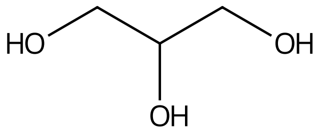

$C_{35}H_{66}O_{6}$

Triglyceride CoLaM

11 References | 0 Reactions | 0 Suppliers

There are no Key Physical Properties to display for this substance.

7

113338-11-7

544-63-8

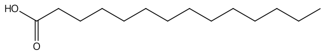

334-48-5

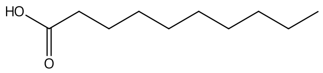

124-07-2

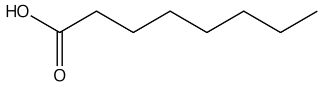

56-81-5

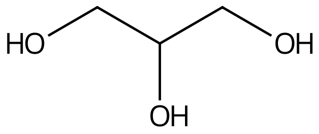

$C_{35}H_{66}O_6$

Tetradecanoic acid, ester with 1,2,3-propanetriol monodecanoate monooctanoate

11 References | 0 Reactions | 0 Suppliers

There are no Key Physical Properties to display for this substance.

8

67874-05-9

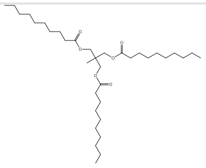

$C_{35}H_{66}O_6$

1,1'-[2-Methyl-2-[[(1-oxodecyl)oxy]methyl]-1,3-propanediyl] didecanoate

11 References | 0 Reactions | 7 Suppliers

Key Physical Properties	Value	Condition
Molecular Weight	582.90	-
Boiling Point (Predicted)	619.9±35.0 °C	Press: 760 Torr
Density (Predicted)	0.945±0.06 g/cm^3	Temp: 20 °C; Press: 760 Torr

9

60138-21-8

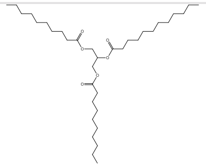

$C_{35}H_{66}O_6$

2-[(1-Oxodecyl)oxy]-1-[[(1-oxodecyl)oxy] methyl]ethyl dodecanoate

11 References | 0 Reactions | 0 Suppliers

Key Physical Properties	Value	Condition
Molecular Weight	582.90	-
Boiling Point (Predicted)	600.7±22.0 °C	Press: 760 Torr
Density (Predicted)	0.944±0.06 g/cm^3	Temp: 20 °C; Press: 760 Torr

10

106069-01-6

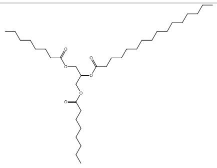

$C_{35}H_{66}O_6$

2-[(1-Oxooctyl)oxy]-1-[[(1-oxooctyl)oxy] methyl]ethyl hexadecanoate

10 References | 1 Reaction | 0 Suppliers

Key Physical Properties	Value	Condition
Molecular Weight	582.90	-
Boiling Point (Predicted)	600.7±22.0 °C	Press: 760 Torr
Density (Predicted)	0.944±0.06 g/cm^3	Temp: 20 °C; Press: 760 Torr

Copyright © 2022 American Chemical Society (ACS). All Rights Reserved.
Internal use only. Redistribution is subject to the terms of your SciFinder^n License Agreement and CAS information Use Policies.
