# Supplementary material for: Efficient Green Extraction of Nutraceutical Compounds from Nannochloropsis gaditana: A Comparative Electrospray Ionization LC-MS and GC-MS Analysis for Lipid Profiling
Source: Foods. 2024 Dec 19;13(24):4117. doi: 10.3390/foods13244117 (PMC11675803; doi:10.3390/foods13244117)
Supplement: Supplementary file 1 [file foods-13-04117-s001.zip › MS Results/HPLC-MS PLE -Results-MC/Pico a 3.0 min_C10H21NO5.pdf]

CAS SciFinder^n

Task History

Initiating Search

November 25, 2022, 10:34AM

Substances:

Advanced Search:

Molecular Formula: C10H21NO5

Search Tasks

Task	Search Type	View
Exported: Returned Substance Results (656)	Substances	View Results

Copyright © 2022 American Chemical Society (ACS). All Rights Reserved.
Internal use only. Redistribution is subject to the terms of your SciFinder^n License Agreement and CAS information Use Policies.

CAS SciFinder^n

Substances (10)

View in SciFinder^n

1

56323-09-2

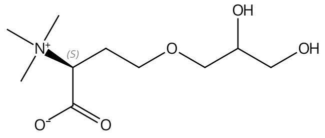

Available stereochemistry shown

$C_{10}H_{21}NO_5$

1-Propanaminium, 1-carboxy-3-(2,3-dihydroxypropoxy)-*N*,*N*,*N*-trimethyl-, inner salt, (1*S*)-

62 References | 0 Reactions | 0 Suppliers

Key Physical Properties	Value	Condition
Molecular Weight	235.28	-

2

116747-80-9

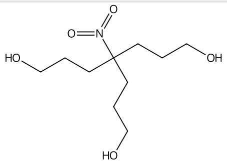

$C_{10}H_{21}NO_5$

4-(3-Hydroxypropyl)-4-nitro-1,7-heptanediol

32 References | 18 Reactions | 12 Suppliers

Key Physical Properties	Value	Condition
Molecular Weight	235.28	-
Boiling Point (Experimental)	>150 °C	Press: 0.3 Torr
Density (Predicted)	1.182±0.06 g/cm^3	Temp: 20 °C; Press: 760 Torr
pKa (Predicted)	14.43±0.10	Most Acidic Temp: 25 °C

Experimental Properties | Spectra

3

66807-99-6

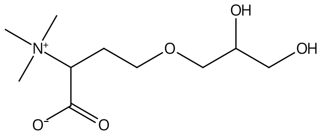

$C_{10}H_{21}NO_5$

1-Propanaminium, 1-carboxy-3-(2,3-dihydroxypropoxy)-*N*,*N*,*N*-trimethyl-, inner salt

27 References | 0 Reactions | 1 Supplier

Key Physical Properties	Value	Condition
Molecular Weight	235.28	-

4

3679-04-7

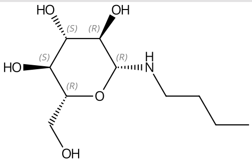

Absolute stereochemistry shown

$C_{10}H_{21}NO_5$

N-Butyl-β-D-glucopyranosylamine

19 References | 10 Reactions | 1 Supplier

Key Physical Properties	Value	Condition
Molecular Weight	235.28	-
Melting Point (Experimental)	97-98 °C	-
Boiling Point (Predicted)	427.7±45.0 °C	Press: 760 Torr
Density (Predicted)	1.28±0.1 g/cm^3	Temp: 20 °C; Press: 760 Torr
pKa (Predicted)	13.25±0.70	Most Acidic Temp: 25 °C

Experimental Properties | Spectra

5

127902-54-9

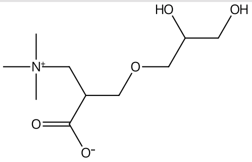

$C_{10}H_{21}NO_5$

1-Propanaminium, 2-carboxy-3-(2,3-dihydroxypropoxy)-*N*,*N*,*N*-trimethyl-, inner salt

17 References | 0 Reactions | 0 Suppliers

Key Physical Properties	Value	Condition
Molecular Weight	235.28	-

6

29352-40-7

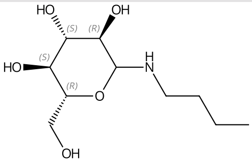

Absolute stereochemistry shown

$C_{10}H_{21}NO_5$

N-Butyl-D-glucopyranosylamine

16 References | 2 Reactions | 8 Suppliers

Key Physical Properties	Value	Condition
Molecular Weight	235.28	-
Melting Point (Experimental)	88-89 °C	-
Boiling Point (Predicted)	427.7±45.0 °C	Press: 760 Torr
Density (Predicted)	1.28±0.1 g/cm^3	Temp: 20 °C; Press: 760 Torr
pKa (Predicted)	13.25±0.70	Most Acidic Temp: 25 °C

Experimental Properties

7

118988-07-1

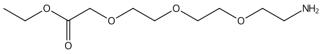

$C_{10}H_{21}NO_5$

Ethyl 2-[2-[2-(2-aminoethoxy)ethoxy]ethoxy] acetate

10 References | 23 Reactions | 4 Suppliers

Key Physical Properties	Value	Condition
Molecular Weight	235.28	-
Boiling Point (Predicted)	323.0±27.0 °C	Press: 760 Torr
Density (Predicted)	1.070±0.06 g/cm^3	Temp: 20 °C; Press: 760 Torr
pKa (Predicted)	8.74±0.10	Most Basic Temp: 25 °C

8

64559-34-8

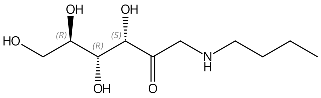

Absolute stereochemistry shown

$C_{10}H_{21}NO_5$

1-(Butylamino)-1-deoxy-D-fructose

10 References | 1 Reaction | 1 Supplier

Key Physical Properties	Value	Condition
Molecular Weight	235.28	-
Boiling Point (Predicted)	485.0±45.0 °C	Press: 760 Torr
Density (Predicted)	1.233±0.06 g/cm^3	Temp: 20 °C; Press: 760 Torr
pKa (Predicted)	12.02±0.20	Most Acidic Temp: 25 °C

9

32739-01-8

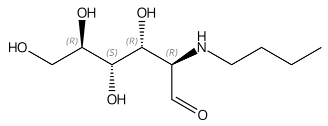

Absolute stereochemistry shown

$C_{10}H_{21}NO_5$

2-(Butylamino)-2-deoxy-D-glucose

9 References | 3 Reactions | 0 Suppliers

Key Physical Properties	Value	Condition
Molecular Weight	235.28	-
Melting Point (Experimental)	147-148 °C (decomp)	-
Boiling Point (Predicted)	479.0±45.0 °C	Press: 760 Torr
Density (Predicted)	1.230±0.06 g/cm^3	Temp: 20 °C; Press: 760 Torr
pKa (Predicted)	13.32±0.20	Most Acidic Temp: 25 °C

Experimental Properties

10

97553-90-7

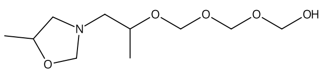

$C_{10}H_{21}NO_5$

1-[[[1-Methyl-2-(5-methyl-3-oxazolidinyl)ethoxy]methoxy]methoxy]methanol

7 References | 2 Reactions | 3 Suppliers

Key Physical Properties	Value	Condition
Molecular Weight	235.28	-
Boiling Point (Predicted)	262.4±40.0 °C	Press: 760 Torr
Density (Predicted)	1.113±0.06 g/cm^3	Temp: 20 °C; Press: 760 Torr
pKa (Predicted)	12.76±0.10	Most Acidic Temp: 25 °C

Copyright © 2022 American Chemical Society (ACS). All Rights Reserved.
Internal use only. Redistribution is subject to the terms of your SciFinder^n License Agreement and CAS information Use Policies.
