# Supplementary material for: Efficient Green Extraction of Nutraceutical Compounds from Nannochloropsis gaditana: A Comparative Electrospray Ionization LC-MS and GC-MS Analysis for Lipid Profiling
Source: Foods. 2024 Dec 19;13(24):4117. doi: 10.3390/foods13244117 (PMC11675803; doi:10.3390/foods13244117)
Supplement: Supplementary file 1 [file foods-13-04117-s001.zip › MS Results/HPLC-MS PLE -Results-MC/Pico a 3.4 min.pdf]

SciFinder^n

Task History

Initiating Search

November 25, 2022, 10:44AM

Substances:

Advanced Search:

Molecular Formula: $C_{11}H_{16}O_3$

Search Tasks

Task	Search Type	View
Exported: Returned Substance Results (10,068)	Substances	View Results

Copyright © 2022 American Chemical Society (ACS). All Rights Reserved.
Internal use only. Redistribution is subject to the terms of your SciFinder^n License Agreement and CAS information Use Policies.

CAS SciFinderⁿ

Substances (10)

View in SciFinderⁿ

1

82428-30-6

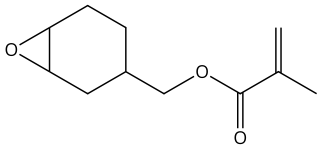

$C_{11}H_{16}O_3$

7-Oxabicyclo[4.1.0]hept-3-ylmethyl 2-methyl-2-propenoate

1,171 References | 1,095 Reactions | 40 Suppliers

Key Physical Properties	Value	Condition
Molecular Weight	196.24	-
Boiling Point (Predicted)	274.8±13.0 °C	Press: 760 Torr
Density (Predicted)	1.079±0.06 g/cm^3	Temp: 20 °C; Press: 760 Torr

Spectra

2

5989-02-6

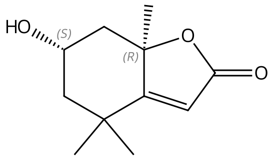

Absolute stereochemistry shown

$C_{11}H_{16}O_3$

Loliolide

748 References | 9 Reactions | 21 Suppliers

Key Physical Properties	Value	Condition
Molecular Weight	196.24	-
Melting Point (Experimental)	151-152 °C	-
Boiling Point (Predicted)	352.7±42.0 °C	Press: 760 Torr
Density (Predicted)	1.16±0.1 g/cm^3	Temp: 20 °C; Press: 760 Torr
pKa (Predicted)	14.39±0.60	Most Acidic Temp: 25 °C

Experimental Properties | Spectra

3

6766-82-1

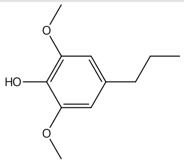

$C_{11}H_{16}O_3$

2,6-Dimethoxy-4-propylphenol

529 References | 164 Reactions | 23 Suppliers

Key Physical Properties	Value	Condition
Molecular Weight	196.24	-
Boiling Point (Experimental)	285 °C	-
Density (Predicted)	1.059±0.06 g/cm^3	Temp: 20 °C; Press: 760 Torr
pKa (Predicted)	10.06±0.23	Most Acidic Temp: 25 °C

Experimental Properties | Spectra

4

2050-25-1

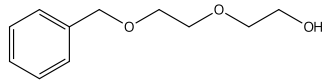

$C_{11}H_{16}O_3$

Diethylene glycol monobenzyl ether

349 References | 163 Reactions | 81 Suppliers

Key Physical Properties	Value	Condition
Molecular Weight	196.24	-
Boiling Point (Experimental)	142-145 °C	Press: 2 Torr
Density (Predicted)	1.078±0.06 g/cm^3	Temp: 20 °C; Press: 760 Torr
pKa (Predicted)	14.36±0.10	Most Acidic Temp: 25 °C

Experimental Properties | Spectra

5

42711-75-1

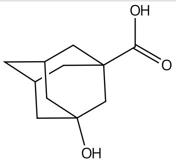

$C_{11}H_{16}O_3$

3-Hydroxy-1-adamantanecarboxylic acid

294 References | 345 Reactions | 110 Suppliers

Key Physical Properties	Value	Condition
Molecular Weight	196.24	-
Melting Point (Experimental)	202-203 °C	-
Boiling Point (Predicted)	357.2±25.0 °C	Press: 760 Torr
Density (Predicted)	1.419±0.06 g/cm^3	Temp: 20 °C; Press: 760 Torr
pKa (Predicted)	4.60±0.40	Most Acidic Temp: 25 °C

Experimental Properties | Spectra

6

3929-47-3

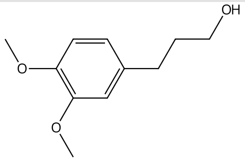

$C_{11}H_{16}O_3$

3-(3',4'-Dimethoxyphenyl)-1-propanol

212 References | 247 Reactions | 46 Suppliers

Key Physical Properties	Value	Condition
Molecular Weight	196.24	-
Boiling Point (Experimental)	171 °C	Press: 1 Torr
Density (Predicted)	1.064±0.06 g/cm^3	Temp: 20 °C; Press: 760 Torr
pKa (Predicted)	15.04±0.10	Most Acidic Temp: 25 °C

Experimental Properties | Spectra

7

5073-65-4

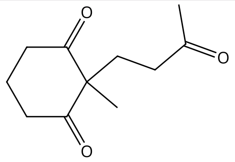

$C_{11}H_{16}O_3$

2-Methyl-2-(3-oxobutyl)-1,3-cyclohexanedione

133 References | 248 Reactions | 5 Suppliers

Key Physical Properties	Value	Condition
Molecular Weight	196.24	-
Boiling Point (Experimental)	95-100 °C	Press: 0.01 Torr
Density (Predicted)	1.057±0.06 g/cm^3	Temp: 20 °C; Press: 760 Torr

Experimental Properties | Spectra

8

135362-69-5

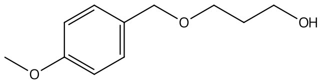

$C_{11}H_{16}O_3$

3-[(4-Methoxyphenyl)methoxy]-1-propanol

125 References | 449 Reactions | 52 Suppliers

Key Physical Properties	Value	Condition
Molecular Weight	196.24	-
Boiling Point (Predicted)	334.2±22.0 °C	Press: 760 Torr
Density (Predicted)	1.071±0.06 g/cm^3	Temp: 20 °C; Press: 760 Torr
pKa (Predicted)	14.87±0.10	Most Acidic Temp: 25 °C

Spectra

9

38274-00-9

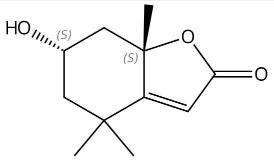

Absolute stereochemistry shown

$C_{11}H_{16}O_3$

(6*S*,7a*S*)-5,6,7,7a-Tetrahydro-6-hydroxy-4,4,7a-trimethyl-2(4*H*)-benzofuranone

112 References | 12 Reactions | 9 Suppliers

Key Physical Properties	Value	Condition
Molecular Weight	196.24	-
Boiling Point (Predicted)	352.7±42.0 °C	Press: 760 Torr
Density (Predicted)	1.16±0.1 g/cm^3	Temp: 20 °C; Press: 760 Torr
pKa (Predicted)	14.39±0.60	Most Acidic Temp: 25 °C

Experimental Properties | Spectra

10

81995-38-2

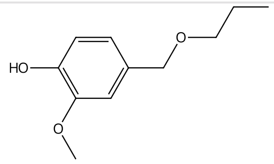

$C_{11}H_{16}O_3$

Vanillyl propyl ether

91 References | 3 Reactions | 6 Suppliers

Key Physical Properties	Value	Condition
Molecular Weight	196.24	-
Boiling Point (Predicted)	291.7±25.0 °C	Press: 760 Torr
Density (Predicted)	1.066±0.06 g/cm^3	Temp: 20 °C; Press: 760 Torr
pKa (Predicted)	9.73±0.20	Most Acidic Temp: 25 °C

Copyright © 2022 American Chemical Society (ACS). All Rights Reserved.
Internal use only. Redistribution is subject to the terms of your SciFinder^n License Agreement and CAS information Use Policies.
