# Supplementary material for: Efficient Green Extraction of Nutraceutical Compounds from Nannochloropsis gaditana: A Comparative Electrospray Ionization LC-MS and GC-MS Analysis for Lipid Profiling
Source: Foods. 2024 Dec 19;13(24):4117. doi: 10.3390/foods13244117 (PMC11675803; doi:10.3390/foods13244117)
Supplement: Supplementary file 1 [file foods-13-04117-s001.zip › MS Results/HPLC-MS PLE -Results-MC/Pico a 30.5 min_C35H62O3.pdf]

SciFinder^n

Task History

Initiating Search

November 25, 2022, 1:06PM

Substances:

Advanced Search:

Molecular Formula: $C_{35}H_{62}O_3$

Search Tasks

Task	Search Type	View
Exported: Returned Substance Results (59)	Substances	View Results

Copyright © 2022 American Chemical Society (ACS). All Rights Reserved.
Internal use only. Redistribution is subject to the terms of your SciFinder^n License Agreement and CAS information Use Policies.

CAS SciFinder^n

Substances (10)

View in SciFinder^n

1

2082-79-3

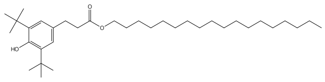

$C_{35}H_{62}O_3$

Antioxidant 1076

17K References | 1,168 Reactions | 74 Suppliers

Key Physical Properties	Value	Condition
Molecular Weight	530.87	-
Melting Point (Experimental)	49-50 °C	-
Boiling Point (Predicted)	568.1±45.0 °C	Press: 760 Torr
Density (Predicted)	0.929±0.06 g/cm^3	Temp: 20 °C; Press: 760 Torr
pKa (Predicted)	12.33±0.40	Most Acidic Temp: 25 °C

Experimental Properties | Spectra

2

2269-80-9

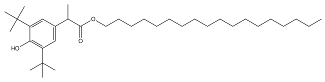

$C_{35}H_{62}O_3$

Octadecyl 3,5-bis(1,1-dimethylethyl)-4-hydroxy-α-methylbenzeneacetate

43 References | 2 Reactions | 0 Suppliers

Key Physical Properties	Value	Condition
Molecular Weight	530.87	-
Melting Point (Experimental)	46-47 °C	-
Boiling Point (Predicted)	554.4±45.0 °C	Press: 760 Torr
Density (Predicted)	0.928±0.06 g/cm^3	Temp: 20 °C; Press: 760 Torr
pKa (Predicted)	10.83±0.40	Most Acidic Temp: 25 °C

Experimental Properties

3

131525-54-7

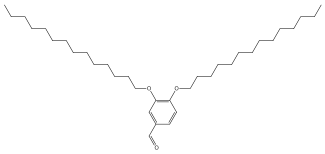

$C_{35}H_{62}O_3$

3,4-Bis(tetradecyloxy)benzaldehyde

24 References | 36 Reactions | 1 Supplier

Key Physical Properties	Value	Condition
Molecular Weight	530.87	-
Melting Point (Experimental)	72 °C	Solvent: Ethanol
Boiling Point (Predicted)	612.6±35.0 °C	Press: 760 Torr
Density (Predicted)	0.917±0.06 g/cm^3	Temp: 20 °C; Press: 760 Torr

Experimental Properties | Spectra

4

90147-09-4

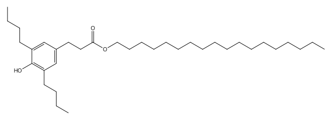

$C_{35}H_{62}O_3$

Octadecyl 3,5-dibutyl-4-hydroxybenzenepropanoate

14 References | 0 Reactions | 1 Supplier

Key Physical Properties	Value	Condition
Molecular Weight	530.87	-
Boiling Point (Predicted)	605.3±50.0 °C	Press: 760 Torr
Density (Predicted)	0.932±0.06 g/cm^3	Temp: 20 °C; Press: 760 Torr
pKa (Predicted)	11.23±0.25	Most Acidic Temp: 25 °C

5

315188-92-2

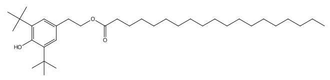

$C_{35}H_{62}O_3$

2-[3,5-Bis(1,1-dimethylethyl)-4-hydroxyphenyl]ethyl nonadecanoate

10 References | 0 Reactions | 4 Suppliers

Key Physical Properties	Value	Condition
Molecular Weight	530.87	-
Boiling Point (Predicted)	571.0±45.0 °C	Press: 760 Torr
Density (Predicted)	0.929±0.06 g/cm^3	Temp: 20 °C; Press: 760 Torr
pKa (Predicted)	12.22±0.40	Most Acidic Temp: 25 °C

6

35190-74-0

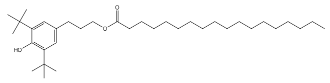

$C_{35}H_{62}O_3$

3-[3,5-Bis(1,1-dimethylethyl)-4-hydroxyphenyl]propyl octadecanoate

9 References | 1 Reaction | 0 Suppliers

Key Physical Properties	Value	Condition
Molecular Weight	530.87	-
Melting Point (Experimental)	45 °C	-
Boiling Point (Predicted)	576.2±38.0 °C	Press: 760 Torr
Density (Predicted)	0.929±0.06 g/cm^3	Temp: 20 °C; Press: 760 Torr
pKa (Predicted)	12.47±0.40	Most Acidic Temp: 25 °C

Experimental Properties

7

151484-64-9

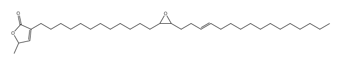

$C_{35}H_{62}O_3$

rel-3-[12-[(2*R*,3*S*)-3-[(3*E*)-3-Hexadecen-1-yl]-2-oxiranyl]dodecyl]-5-methyl-2(5*H*)-furanone

8 References | 0 Reactions | 4 Suppliers

Key Physical Properties	Value	Condition
Molecular Weight	530.87	-
Boiling Point (Predicted)	625.5±28.0 °C	Press: 760 Torr
Density (Predicted)	0.926±0.06 g/cm^3	Temp: 20 °C; Press: 760 Torr

8

1012332-22-7

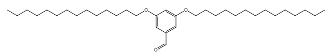

$C_{35}H_{62}O_3$

3,5-Bis(tetradecyloxy)benzaldehyde

7 References | 15 Reactions | 0 Suppliers

Key Physical Properties	Value	Condition
Molecular Weight	530.87	-
Boiling Point (Predicted)	618.1±35.0 °C	Press: 760 Torr
Density (Predicted)	0.917±0.06 g/cm^3	Temp: 20 °C; Press: 760 Torr

Experimental Properties | Spectra

9

23271-32-1

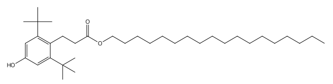

$C_{35}H_{62}O_3$

Octadecyl 2,6-bis(1,1-dimethylethyl)-4-hydroxybenzenepropanoate

6 References | 0 Reactions | 0 Suppliers

Key Physical Properties	Value	Condition
Molecular Weight	530.87	-
Boiling Point (Predicted)	582.1±45.0 °C	Press: 760 Torr
Density (Predicted)	0.929±0.06 g/cm^3	Temp: 20 °C; Press: 760 Torr
pKa (Predicted)	10.27±0.25	Most Acidic Temp: 25 °C

10

260368-47-6

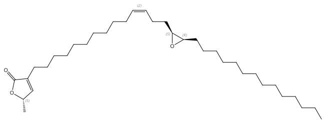

Absolute stereochemistry shown,
Rotation (+)
Double bond geometry shown

$C_{35}H_{62}O_3$

(5*S*)-5-Methyl-3-[(11*Z*)-14-[(2*S*,3*R*)-3-tetradecyl-2-oxiranyl]-11-tetradecen-1-yl]-2(5*H*)-furanone

5 References | 0 Reactions | 0 Suppliers

Key Physical Properties	Value	Condition
Molecular Weight	530.87	-
Boiling Point (Predicted)	625.5±28.0 °C	Press: 760 Torr
Density (Predicted)	0.926±0.06 g/cm^3	Temp: 20 °C; Press: 760 Torr

Copyright © 2022 American Chemical Society (ACS). All Rights Reserved.
Internal use only. Redistribution is subject to the terms of your SciFinder^n License Agreement and CAS information Use Policies.
