# Supplementary material for: Efficient Green Extraction of Nutraceutical Compounds from Nannochloropsis gaditana: A Comparative Electrospray Ionization LC-MS and GC-MS Analysis for Lipid Profiling
Source: Foods. 2024 Dec 19;13(24):4117. doi: 10.3390/foods13244117 (PMC11675803; doi:10.3390/foods13244117)
Supplement: Supplementary file 1 [file foods-13-04117-s001.zip › MS Results/HPLC-MS PLE -Results-MC/Pico a 30.5 min_C37H70O6.pdf]

CAS SciFinder^{n}

Task History

Initiating Search

November 25, 2022, 1:12PM

Substances:

Advanced Search:

Molecular Formula: $C_{37}H_{70}O_6$

Search Tasks

Task	Search Type	View
Exported: Returned Substance Results (91)	Substances	View Results

Copyright © 2022 American Chemical Society (ACS). All Rights Reserved.
Internal use only. Redistribution is subject to the terms of your SciFinder^{n} License Agreement and CAS information Use Policies.

CAS SciFinder^n

Substances (10)

View in SciFinder^n

1

28211-63-4

334-48-5

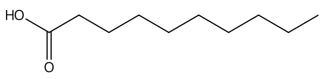

143-07-7

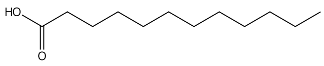

56-81-5

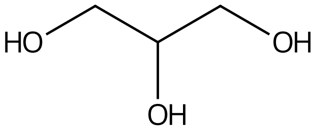

$C_{37}H_{70}O_6$

Triglyceride CLaLa

44 References | 0 Reactions | 0 Suppliers

There are no Key Physical Properties to display for this substance.

Spectra

2

108559-69-9

544-63-8

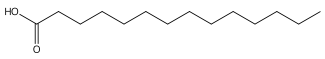

107-92-6

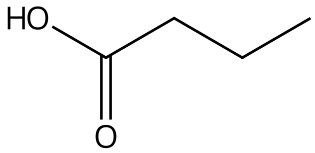

57-10-3

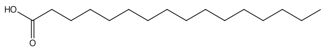

56-81-5

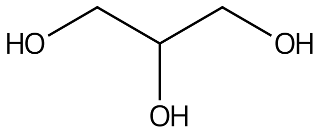

$C_{37}H_{70}O_6$

Hexadecanoic acid, ester with 1,2,3-propanetriol monobutanoate monotetradecanoate

22 References | 0 Reactions | 0 Suppliers

There are no Key Physical Properties to display for this substance.

3

30283-10-4

544-63-8

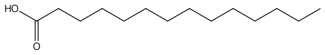

143-07-7

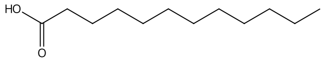

124-07-2

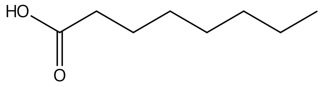

56-81-5

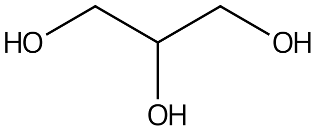

$C_{37}H_{70}O_6$

Triglyceride CyLaM

22 References | 0 Reactions | 0 Suppliers

Key Physical Properties	Value	Condition
Melting Point (Experimental)	15.0 °C	-

Experimental Properties | Spectra

4

60138-11-6

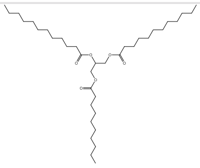

$C_{37}H_{70}O_6$

Dodecanoic acid, 1-[[(1-oxodecyl)oxy]methyl]-1,2-ethanediyl ester

19 References | 0 Reactions | 0 Suppliers

Key Physical Properties	Value	Condition
Molecular Weight	610.95	-
Melting Point (Experimental)	32.6 °C	-
Boiling Point (Predicted)	622.3±22.0 °C	Press: 760 Torr
Density (Predicted)	0.939±0.06 g/cm^3	Temp: 20 °C; Press: 760 Torr

Experimental Properties

5

87553-94-4

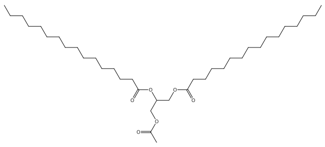

$C_{37}H_{70}O_6$

Hexadecanoic acid, 1-[(acetyloxy)methyl]-1,2-ethanediyl ester

16 References | 0 Reactions | 0 Suppliers

Key Physical Properties	Value	Condition
Molecular Weight	610.95	-
Boiling Point (Predicted)	622.3±22.0 °C	Press: 760 Torr
Density (Predicted)	0.939±0.06 g/cm^3	Temp: 20 °C; Press: 760 Torr

6

113338-12-8

544-63-8

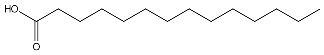

334-48-5

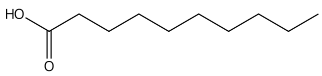

56-81-5

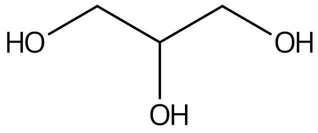

$C_{37}H_{70}O_6$

Triglyceride CCM

14 References | 2 Reactions | 0 Suppliers

There are no Key Physical Properties to display for this substance.

Spectra

7

118569-35-0

334-48-5

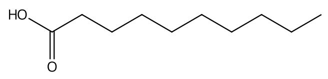

124-07-2

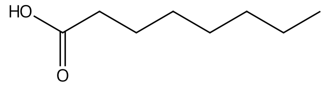

57-10-3

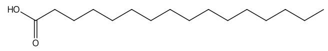

56-81-5

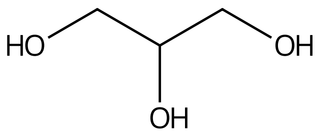

$C_{37}H_{70}O_6$

Triglyceride CCyP

13 References | 0 Reactions | 0 Suppliers

There are no Key Physical Properties to display for this substance.

Spectra

8

62833-19-6

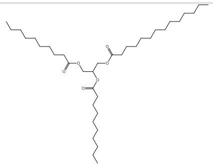

$C_{37}H_{70}O_6$

2,3-Bis[(1-oxodecyl)oxy]propyl tetradecanoate

13 References | 0 Reactions | 4 Suppliers

Key Physical Properties	Value	Condition
Molecular Weight	610.95	-
Melting Point (Experimental)	3 °C	-
Boiling Point (Predicted)	622.3±22.0 °C	Press: 760 Torr
Density (Predicted)	0.939±0.06 g/cm^3	Temp: 20 °C; Press: 760 Torr

Experimental Properties

9

118569-37-2

143-07-7

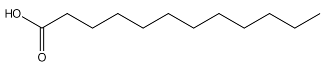

142-62-1

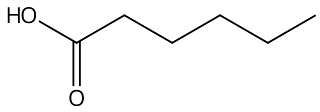

57-10-3

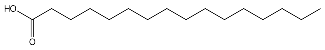

56-81-5

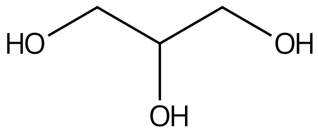

$C_{37}H_{70}O_6$

Triglyceride CoLaP

12 References | 0 Reactions | 0 Suppliers

There are no Key Physical Properties to display for this substance.

10

99431-58-0

143-07-7

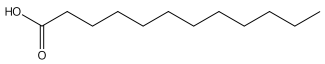

107-92-6

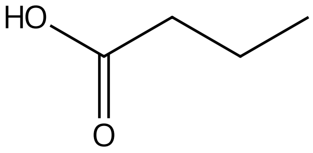

57-11-4

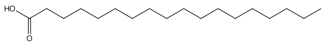

56-81-5

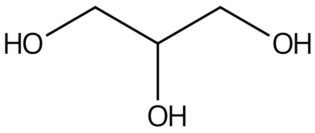

$C_{37}H_{70}O_6$

Octadecanoic acid, ester with 1,2,3-propanetriol monobutanoate monododecanoate

11 References | 0 Reactions | 0 Suppliers

There are no Key Physical Properties to display for this substance.

Copyright © 2022 American Chemical Society (ACS). All Rights Reserved.
Internal use only. Redistribution is subject to the terms of your SciFinder^{n} License Agreement and CAS information Use Policies.
