# Supplementary material for: Efficient Green Extraction of Nutraceutical Compounds from Nannochloropsis gaditana: A Comparative Electrospray Ionization LC-MS and GC-MS Analysis for Lipid Profiling
Source: Foods. 2024 Dec 19;13(24):4117. doi: 10.3390/foods13244117 (PMC11675803; doi:10.3390/foods13244117)
Supplement: Supplementary file 1 [file foods-13-04117-s001.zip › MS Results/HPLC-MS PLE -Results-MC/Pico a 35.1 min_C57H104O7.pdf]

SciFinder^{n}

Task History

Initiating Search

November 25, 2022, 1:30PM

Substances:

Advanced Search:

Molecular Formula: C57H104O7

Search Tasks

Task	Search Type	View
Exported: Returned Substance Results (20)	Substances	View Results

Copyright © 2022 American Chemical Society (ACS). All Rights Reserved.
Internal use only. Redistribution is subject to the terms of your SciFinder^{n} License Agreement and CAS information Use Policies.

SciFinderⁿ

Substances (10)

View in SciFinderⁿ

1

246518-15-0

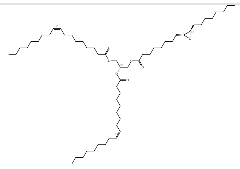

Relative stereochemistry shown
Double bond geometry shown

$C_{57}H_{104}O_7$

rel-(2*R*)-2,3-Bis[[(9*Z*)-1-oxo-9-octadecen-1-yl]oxy]propyl (2*S*,3*R*)-3-octyl-2-oxiraneoctanoate

3 References | 0 Reactions | 0 Suppliers

Key Physical Properties	Value	Condition
Molecular Weight	901.43	-
Boiling Point (Predicted)	831.2±54.0 °C	Press: 760 Torr
Density (Predicted)	0.939±0.06 g/cm^3	Temp: 20 °C; Press: 760 Torr

2

110771-88-5

There are no Key Physical Properties to display for this substance.

141-22-0

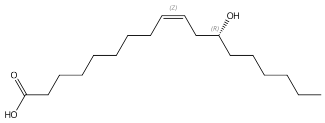

Absolute stereochemistry shown,
Rotation (-)
Double bond geometry shown

112-80-1

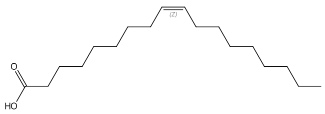

Double bond geometry shown

56-81-5

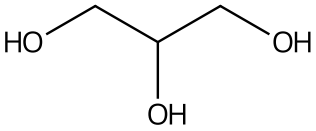

$C_{57}H_{104}O_{7}$

9-Octadecenoic acid, 12-hydroxy-, (9*Z*,12*R*)-, ester with 1,2,3-propanetriol di-(9*Z*)-9-octadecenoate

3 References | 0 Reactions | 0 Suppliers

3

2423090-07-5

There are no Key Physical Properties to display for this substance.

2423090-05-3

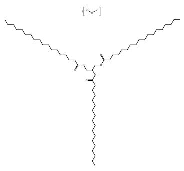

$C_{57}H_{104}O_{7}$

1 Reference | 0 Reactions | 0 Suppliers

4

2229014-88-2

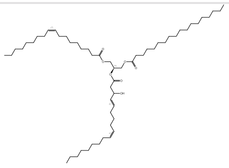

Absolute stereochemistry shown
Double bond geometry shown

$C_{57}H_{104}O_7$

(1*R*)-1-[[[(9*Z*)-1-Oxo-9-octadecen-1-yl]oxy]methyl]-2-[(1-oxooctadecyl)oxy]ethyl (4*E*,9*Z*)-3-hydroxy-4,9-octadecadienoate

1 Reference | 0 Reactions | 0 Suppliers

Key Physical Properties	Value	Condition
Molecular Weight	901.43	-
Boiling Point (Predicted)	838.7±65.0 °C	Press: 760 Torr
Density (Predicted)	0.939±0.06 g/cm^3	Temp: 20 °C; Press: 760 Torr
pKa (Predicted)	13.48±0.20	Most Acidic Temp: 25 °C

5

2229014-82-6

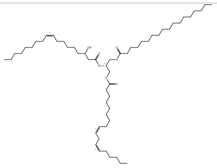

Absolute stereochemistry shown
Double bond geometry shown

$C_{57}H_{104}O_7$

(2*R*)-2-[[(9*Z*)-3-Hydroxy-1-oxo-9-octadecen-1-yl]oxy]-3-[(1-oxooctadecyl)oxy]propyl (9*Z*,12*Z*)-9,12-octadecadienoate

1 Reference | 0 Reactions | 0 Suppliers

Key Physical Properties	Value	Condition
Molecular Weight	901.43	-
Boiling Point (Predicted)	838.7±65.0 °C	Press: 760 Torr
Density (Predicted)	0.939±0.06 g/cm^3	Temp: 20 °C; Press: 760 Torr
pKa (Predicted)	14.25±0.20	Most Acidic Temp: 25 °C

6

2131173-80-1

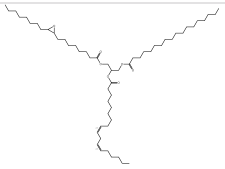

Double bond geometry shown

$C_{57}H_{104}O_7$

2-[[(9*Z*,12*Z*)-1-Oxo-9,12-octadecadien-1-yl]oxy]-3-[(1-oxooctadecyl)oxy]propyl 3-octyl-2-oxiraneoctanoate

1 Reference | 0 Reactions | 0 Suppliers

Key Physical Properties	Value	Condition
Molecular Weight	901.43	-
Boiling Point (Predicted)	830.7±44.0 °C	Press: 760 Torr
Density (Predicted)	0.939±0.06 g/cm^3	Temp: 20 °C; Press: 760 Torr

7

1784723-00-7

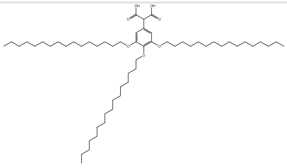

$C_{57}H_{104}O_7$

2-[3,4,5-Tris(hexadecyloxy)phenyl]propanedioic acid

1 Reference | 5 Reactions | 0 Suppliers

Key Physical Properties	Value	Condition
Molecular Weight	901.43	-
Boiling Point (Predicted)	873.6±65.0 °C	Press: 760 Torr
Density (Predicted)	0.955±0.06 g/cm^3	Temp: 20 °C; Press: 760 Torr
pKa (Predicted)	2.72±0.10	Most Acidic Temp: 25 °C

8

1529812-04-1

4103-20-2

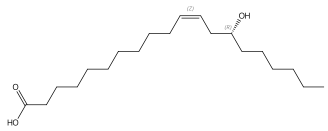

Absolute stereochemistry shown
Double bond geometry shown

373-49-9

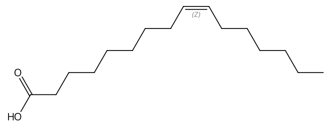

Double bond geometry shown

112-80-1

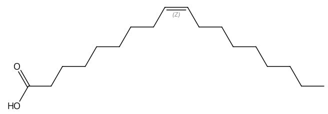

Double bond geometry shown

56-81-5

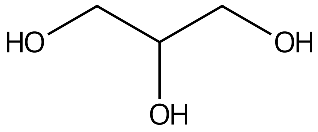

$C_{57}H_{104}O_7$

11-Eicosenoic acid, 14-hydroxy-, ester with 1,2,3-propanetriol mono-(9*Z*)-9-hexadecenoate mono-(9*Z*)-9-octadecenoate, (11*Z*,14*R*)-

1 Reference | 0 Reactions | 0 Suppliers

There are no Key Physical Properties to display for this substance.

9

1071726-86-7

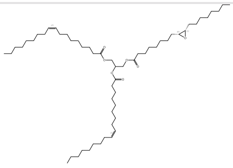

Relative stereochemistry shown
Double bond geometry shown

$C_{57}H_{104}O_7$

rel-2,3-Bis[[(9*Z*)-1-oxo-9-octadecen-1-yl]oxy]propyl (2*R*,3*S*)-3-octyl-2-oxiraneoctanoate

1 Reference | 0 Reactions | 0 Suppliers

Key Physical Properties	Value	Condition
Molecular Weight	901.43	-
Boiling Point (Predicted)	831.2±54.0 °C	Press: 760 Torr
Density (Predicted)	0.939±0.06 g/cm^3	Temp: 20 °C; Press: 760 Torr

10

676319-22-5

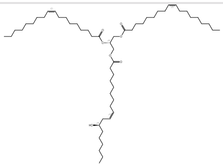

Absolute stereochemistry shown
Double bond geometry shown

$C_{57}H_{104}O_7$

(2*R*)-2,3-Bis[[(9*Z*)-1-oxo-9-octadecen-1-yl]oxy]propyl (9*Z*,12*R*)-12-hydroxy-9-octadecenoate

1 Reference | 0 Reactions | 0 Suppliers

Key Physical Properties	Value	Condition
Molecular Weight	901.43	-
Boiling Point (Predicted)	838.7±64.0 °C	Press: 760 Torr
Density (Predicted)	0.939±0.06 g/cm^3	Temp: 20 °C; Press: 760 Torr
pKa (Predicted)	15.10±0.20	Most Acidic Temp: 25 °C

Copyright © 2022 American Chemical Society (ACS). All Rights Reserved.
Internal use only. Redistribution is subject to the terms of your SciFinderⁿ License Agreement and CAS information Use Policies.
