# Supplementary material for: Efficient Green Extraction of Nutraceutical Compounds from Nannochloropsis gaditana: A Comparative Electrospray Ionization LC-MS and GC-MS Analysis for Lipid Profiling
Source: Foods. 2024 Dec 19;13(24):4117. doi: 10.3390/foods13244117 (PMC11675803; doi:10.3390/foods13244117)
Supplement: Supplementary file 1 [file foods-13-04117-s001.zip › MS Results/HPLC-MS PLE -Results-MC/Pico a 36.2 min C52H76O7.pdf]

SciFinder^n

Task History

Initiating Search

November 25, 2022, 1:42PM

Substances:

Advanced Search:

Molecular Formula: $C_{52}H_{76}O_7$

Search Tasks

Task	Search Type	View
Exported: Returned Substance Results (4)	Substances	View Results

Copyright © 2022 American Chemical Society (ACS). All Rights Reserved.
Internal use only. Redistribution is subject to the terms of your SciFinder^n License Agreement and CAS information Use Policies.

CAS SciFinderⁿ

Substances (4)

View in SciFinderⁿ

1

1666940-41-5

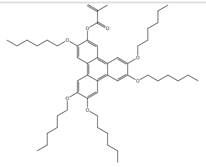

$C_{52}H_{76}O_7$

3,6,7,10,11-Pentakis(hexyloxy)-2-triphenylenyl 2-methyl-2-propenoate

4 References | 8 Reactions | 0 Suppliers

Key Physical Properties	Value	Condition
Molecular Weight	813.16	-
Boiling Point (Predicted)	853.2±65.0 °C	Press: 760 Torr
Density (Predicted)	1.029±0.06 g/cm^3	Temp: 20 °C; Press: 760 Torr

2

1629444-19-4

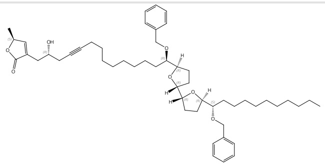

Relative stereochemistry shown

$C_{52}H_{76}O_7$

rel-(5*S*)-3-[(2*R*,14*R*)-2-Hydroxy-14-[(2*R*,2'*R*,5*R*,5'*R*)-octahydro-5'-[(1*S*)-1-(phenylmethoxy)undecyl][2,2'-bifuran]-5-yl]-14-(phenylmethoxy)-4-tetradecyn-1-yl]-5-methyl-2(5*H*)-furanone

1 Reference | 1 Reaction | 0 Suppliers

Key Physical Properties	Value	Condition
Molecular Weight	813.16	-
Boiling Point (Predicted)	872.6±65.0 °C	Press: 760 Torr
Density (Predicted)	1.065±0.06 g/cm^3	Temp: 20 °C; Press: 760 Torr
pKa (Predicted)	13.75±0.20	Most Acidic Temp: 25 °C

3

848397-94-4

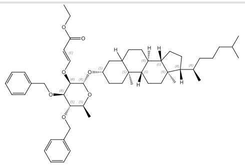

Absolute stereochemistry shown
Double bond geometry shown

$C_{52}H_{76}O_7$

(3β)-Cholestan-3-yl 6-deoxy-2-*O*-[(1*E*)-3-ethoxy-3-oxo-1-propen-1-yl]-3,4-bis-*O*-(phenylmethyl)-α-L-mannopyranoside

1 Reference | 2 Reactions | 0 Suppliers

Key Physical Properties	Value	Condition
Molecular Weight	813.16	-
Boiling Point (Predicted)	809.1±65.0 °C	Press: 760 Torr
Density (Predicted)	1.10±0.1 g/cm^3	Temp: 20 °C; Press: 760 Torr

Experimental Properties | Spectra

4

848397-93-3

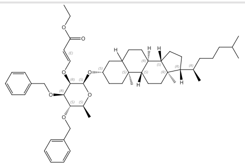

Absolute stereochemistry shown
Double bond geometry shown

$C_{52}H_{76}O_7$

(3β)-Cholestan-3-yl 6-deoxy-2-*O*-[(1*E*)-3-ethoxy-3-oxo-1-propen-1-yl]-3,4-bis-*O*-(phenylmethyl)-β-L-mannopyranoside

1 Reference | 2 Reactions | 0 Suppliers

Key Physical Properties	Value	Condition
Molecular Weight	813.16	-
Boiling Point (Predicted)	809.1±65.0 °C	Press: 760 Torr
Density (Predicted)	1.10±0.1 g/cm^3	Temp: 20 °C; Press: 760 Torr

Experimental Properties | Spectra

Copyright © 2022 American Chemical Society (ACS). All Rights Reserved.
Internal use only. Redistribution is subject to the terms of your SciFinderⁿ License Agreement and CAS information Use Policies.
