# Supplementary material for: Efficient Green Extraction of Nutraceutical Compounds from Nannochloropsis gaditana: A Comparative Electrospray Ionization LC-MS and GC-MS Analysis for Lipid Profiling
Source: Foods. 2024 Dec 19;13(24):4117. doi: 10.3390/foods13244117 (PMC11675803; doi:10.3390/foods13244117)
Supplement: Supplementary file 1 [file foods-13-04117-s001.zip › MS Results/HPLC-MS PLE -Results-MC/Pico a 36.8 min_C45H93N4O5P.pdf]

SciFinder^{n}

Task History

Initiating Search

November 25, 2022, 1:50PM

Substances:

Advanced Search:

Molecular Formula: $C_{45}H_{93}N_4O_5P$

Search Tasks

Task	Search Type	View
Exported: Returned Substance Results (1)	Substances	View Results

Copyright © 2022 American Chemical Society (ACS). All Rights Reserved.
Internal use only. Redistribution is subject to the terms of your SciFinder^{n} License Agreement and CAS information Use Policies.

CAS SciFinderⁿ

Substances (1)

View in SciFinderⁿ

1

2084102-08-7

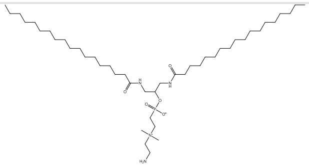

$C_{45}H_{93}N_4O_5P$

Ethanaminium, *N*-(2-aminoethyl)-2-[hydroxy[2-[(1-oxooctadecyl)amino]-1-[[(1-oxooctadecyl)amino]methyl]ethoxy]phosphinyl]-*N*,*N*-dimethyl-, inner salt

1 Reference | 0 Reactions | 0 Suppliers

Key Physical Properties	Value	Condition
Molecular Weight	801.22	-

Copyright © 2022 American Chemical Society (ACS). All Rights Reserved.
Internal use only. Redistribution is subject to the terms of your SciFinderⁿ License Agreement and CAS information Use Policies.
