# Supplementary material for: Efficient Green Extraction of Nutraceutical Compounds from Nannochloropsis gaditana: A Comparative Electrospray Ionization LC-MS and GC-MS Analysis for Lipid Profiling
Source: Foods. 2024 Dec 19;13(24):4117. doi: 10.3390/foods13244117 (PMC11675803; doi:10.3390/foods13244117)
Supplement: Supplementary file 1 [file foods-13-04117-s001.zip › MS Results/HPLC-MS PLE -Results-MC/Pico a 36.8 min_C51H92O6.pdf]

SciFinder^n

Task History

Initiating Search

November 25, 2022, 1:52PM

Substances:

Advanced Search:

Molecular Formula: $C_{51}H_{92}O_6$

Search Tasks

Task	Search Type	View
Exported: Returned Substance Results (74)	Substances	View Results

Copyright © 2022 American Chemical Society (ACS). All Rights Reserved.
Internal use only. Redistribution is subject to the terms of your SciFinder^n License Agreement and CAS information Use Policies.

CAS SciFinder^n

Substances (74)

View in SciFinder^n

1

20246-55-3

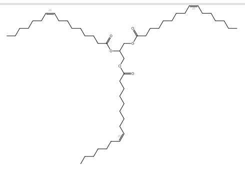

Double bond geometry shown

$C_{51}H_{92}O_6$

Tripalmitolein

177 References | 4 Reactions | 15 Suppliers

Key Physical Properties	Value	Condition
Molecular Weight	801.27	-
Boiling Point (Predicted)	765.9±50.0 °C	Press: 760 Torr
Density (Predicted)	0.929±0.06 g/cm^3	Temp: 20 °C; Press: 760 Torr

Spectra

2

125527-67-5

143-07-7

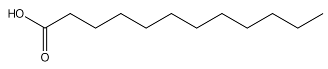

112-80-1

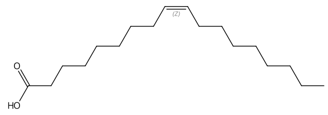

Double bond geometry shown

60-33-3

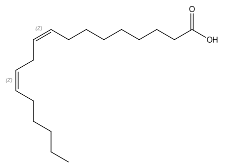

Double bond geometry shown

56-81-5

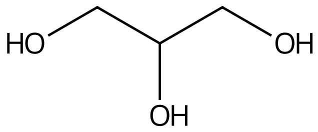

$C_{51}H_{92}O_6$

Triglyceride LLaO

24 References | 0 Reactions | 0 Suppliers

There are no Key Physical Properties to display for this substance.

Spectra

3

125527-68-6

544-63-8

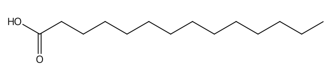

373-49-9

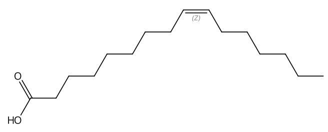

Double bond geometry shown

60-33-3

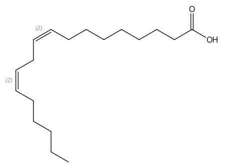

Double bond geometry shown

56-81-5

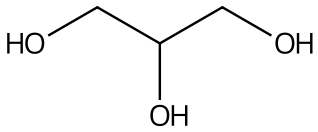

$C_{51}H_{92}O_6$

1,2,3-Propanetriol (9*Z*)-9-hexadecenoate (9*Z*,12*Z*)-9,12-octadecadienoate tetradecanoate

12 References | 0 Reactions | 0 Suppliers

There are no Key Physical Properties to display for this substance.

4

129784-33-4

555-44-2

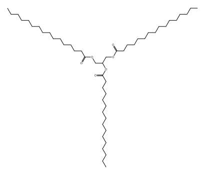

$C_{51}H_{92}O_6$

Hexadecanoic acid, 1,1',1''-(1,2,3-propanetriyl) ester, (?*Z*,?*Z*,?*Z*)-

10 References | 0 Reactions | 1 Supplier

There are no Key Physical Properties to display for this substance.

5

132970-91-3

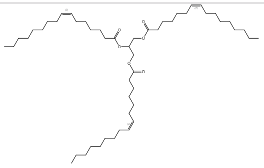

Double bond geometry shown

$C_{51}H_{92}O_6$

7-Hexadecenoic acid, 1,2,3-propanetriyl ester, (7*Z*,7'*Z*,7''*Z*)-

8 References | 0 Reactions | 0 Suppliers

Key Physical Properties	Value	Condition
Molecular Weight	801.27	-
Boiling Point (Predicted)	765.9±50.0 °C	Press: 760 Torr
Density (Predicted)	0.929±0.06 g/cm^3	Temp: 20 °C; Press: 760 Torr

6

134907-85-0

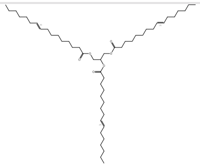

Double bond geometry shown

$C_{51}H_{92}O_6$

1,1',1''-(1,2,3-Propanetriyl) tri-(9*E*)-9-hexadec enoate

7 References | 0 Reactions | 13 Suppliers

Key Physical Properties	Value	Condition
Molecular Weight	801.27	-
Boiling Point (Predicted)	765.9±50.0 °C	Press: 760 Torr
Density (Predicted)	0.929±0.06 g/cm^3	Temp: 20 °C; Press: 760 Torr

Spectra

7

125527-75-5

544-63-8

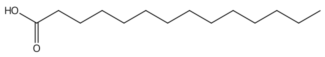

463-40-1

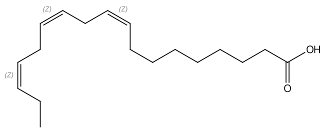

Double bond geometry shown

57-10-3

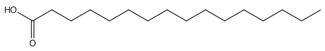

56-81-5

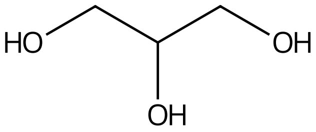

$C_{51}H_{92}O_6$

9,12,15-Octadecatrienoic acid, ester with 1,2,3-propanetriol monohexadecanoate monotetradecanoate, (*all*-*Z*)-

7 References | 0 Reactions | 0 Suppliers

There are no Key Physical Properties to display for this substance.

8

35017-29-9

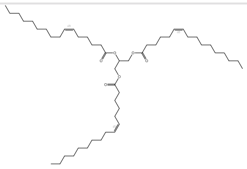

Double bond geometry shown

$C_{51}H_{92}O_6$

6-Hexadecenoic acid, 1,2,3-propanetriyl ester, (6*Z*,6'*Z*,6''*Z*)-

7 References | 0 Reactions | 0 Suppliers

Key Physical Properties	Value	Condition
Molecular Weight	801.27	-
Boiling Point (Predicted)	765.9±50.0 °C	Press: 760 Torr
Density (Predicted)	0.929±0.06 g/cm^3	Temp: 20 °C; Press: 760 Torr

9

884647-09-0

There are no Key Physical Properties to display for this substance.

544-64-9

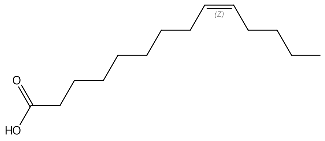

Double bond geometry shown

60-33-3

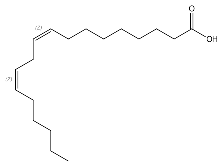

Double bond geometry shown

57-10-3

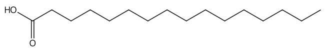

56-81-5

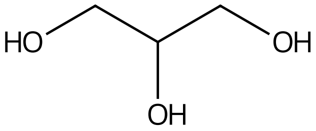

$C_{51}H_{92}O_6$

Triglyceride LMoP

4 References | 0 Reactions | 0 Suppliers

10

127028-32-4

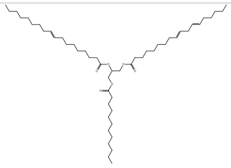

$C_{51}H_{92}O_6$

3-[(1-Oxododecyl)oxy]-2-[[(9*Z*)-1-oxo-9-octadecenyl]oxy]propyl (9*Z*,12*Z*)-9,12-octadecadienoate

4 References | 0 Reactions | 0 Suppliers

Key Physical Properties	Value	Condition
Molecular Weight	801.27	-
Boiling Point (Predicted)	765.9±50.0 °C	Press: 760 Torr
Density (Predicted)	0.929±0.06 g/cm^3	Temp: 20 °C; Press: 760 Torr

11

906541-02-4

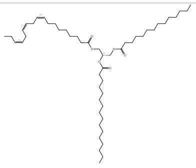

Absolute stereochemistry shown
Double bond geometry shown

$C_{51}H_{92}O_6$

(2*S*)-2-[(1-Oxohexadecyl)oxy]-3-[(1-oxotetradecyl)oxy]propyl (9*Z*,12*Z*,15*Z*)-9,12,15-octadecatrienoate

3 References | 0 Reactions | 1 Supplier

Key Physical Properties	Value	Condition
Molecular Weight	801.27	-
Boiling Point (Predicted)	765.9±50.0 °C	Press: 760 Torr
Density (Predicted)	0.929±0.06 g/cm^3	Temp: 20 °C; Press: 760 Torr

12

887566-78-1

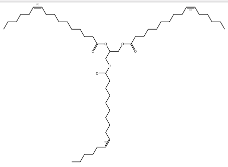

Double bond geometry shown

$C_{51}H_{92}O_6$

10-Hexadecenoic acid, 1,2,3-propanetriyl ester, (10*Z*,10'*Z*,10''*Z*)-

3 References | 9 Reactions | 0 Suppliers

Key Physical Properties	Value	Condition
Molecular Weight	801.27	-
Boiling Point (Predicted)	765.9±50.0 °C	Press: 760 Torr
Density (Predicted)	0.929±0.06 g/cm^3	Temp: 20 °C; Press: 760 Torr

13

355807-79-3

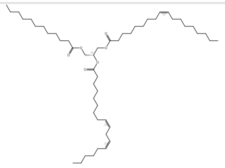

Absolute stereochemistry shown
Double bond geometry shown

$C_{51}H_{92}O_6$

(1*R*)-1-[[(1-Oxododecyl)oxy]methyl]-2-[[(9*Z*)-1-oxo-9-octadecen-1-yl]oxy]ethyl (9*Z*,12*Z*)-9,12-octadecadienoate

3 References | 0 Reactions | 0 Suppliers

Key Physical Properties	Value	Condition
Molecular Weight	801.27	-
Boiling Point (Predicted)	765.9±50.0 °C	Press: 760 Torr
Density (Predicted)	0.929±0.06 g/cm^3	Temp: 20 °C; Press: 760 Torr

14

171916-88-4

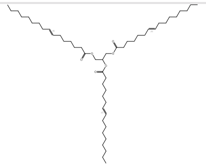

Double bond geometry shown

$C_{51}H_{92}O_6$

7-Hexadecenoic acid, 1,2,3-propanetriyl ester, (*all*-*E*)-

3 References | 0 Reactions | 0 Suppliers

Key Physical Properties	Value	Condition
Molecular Weight	801.27	-
Boiling Point (Predicted)	765.9±50.0 °C	Press: 760 Torr
Density (Predicted)	0.929±0.06 g/cm^3	Temp: 20 °C; Press: 760 Torr

15

106885-87-4

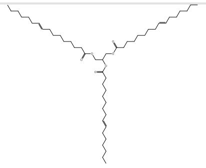

$C_{51}H_{92}O_6$

9-Hexadecenoin, tri-

3 References | 0 Reactions | 0 Suppliers

Key Physical Properties	Value	Condition
Molecular Weight	801.27	-
Boiling Point (Predicted)	765.9±50.0 °C	Press: 760 Torr
Density (Predicted)	0.929±0.06 g/cm^3	Temp: 20 °C; Press: 760 Torr

16

30773-83-2

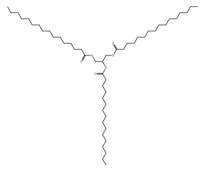

$C_{51}H_{92}O_6$

Hexadecenoic acid, 1,2,3-propanetriyl ester

3 References | 0 Reactions | 0 Suppliers

There are no Key Physical Properties to display for this substance.

17

2412512-89-9

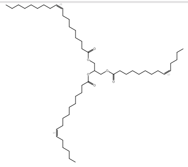
Double bond geometry shown

$C_{51}H_{92}O_6$

2-[[(10*Z*)-1-Oxo-10-hexadecen-1-yl]oxy]-3-[[(9*Z*)-1-oxo-9-tetradecen-1-yl]oxy]propyl (9*Z*)-9-octadecenoate

2 References | 0 Reactions | 0 Suppliers

Key Physical Properties	Value	Condition
Molecular Weight	801.27	-
Boiling Point (Predicted)	765.9±50.0 °C	Press: 760 Torr
Density (Predicted)	0.929±0.06 g/cm^3	Temp: 20 °C; Press: 760 Torr

18

1401437-14-6

80558-45-8

506-30-9

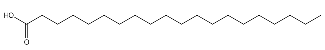

544-63-8

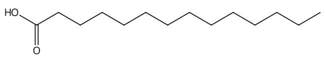

56-81-5

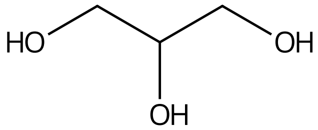

$C_{51}H_{92}O_6$

Eicosatrienoic acid, ester with 1,2,3-propanetriol ditetradecanoate, (*Z*,*Z*,*Z*)-

2 References | 0 Reactions | 0 Suppliers

There are no Key Physical Properties to display for this substance.

19

944261-46-5

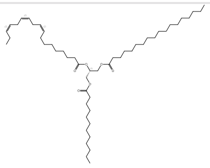

Absolute stereochemistry shown
Double bond geometry shown

$C_{51}H_{92}O_6$

(1*S*)-1-[[(1-Oxododecyl)oxy]methyl]-2-[(1-oxooctadecyl)oxy]ethyl (9*Z*,12*Z*,15*Z*)-9,12,15-octadecatrienoate

2 References | 0 Reactions | 0 Suppliers

Key Physical Properties	Value	Condition
Molecular Weight	801.27	-
Boiling Point (Predicted)	765.9±50.0 °C	Press: 760 Torr
Density (Predicted)	0.929±0.06 g/cm^3	Temp: 20 °C; Press: 760 Torr

20

944261-22-7

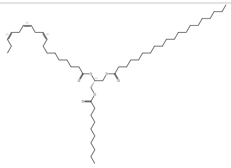

Absolute stereochemistry shown
Double bond geometry shown

$C_{51}H_{92}O_6$

(2*S*)-3-[(1-Oxodecyl)oxy]-2-[[(9*Z*,12*Z*,15*Z*)-1-oxo-9,12,15-octadecatrien-1-yl]oxy]propyl eicosanoate

2 References | 0 Reactions | 0 Suppliers

Key Physical Properties	Value	Condition
Molecular Weight	801.27	-
Boiling Point (Predicted)	765.9±50.0 °C	Press: 760 Torr
Density (Predicted)	0.929±0.06 g/cm^3	Temp: 20 °C; Press: 760 Torr

21

944261-21-6

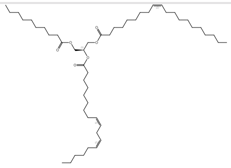

Absolute stereochemistry shown
Double bond geometry shown

$C_{51}H_{92}O_6$

(2*S*)-3-[(1-Oxodecyl)oxy]-2-[[(9*Z*,12*Z*)-1-oxo-9,12-octadecadien-1-yl]oxy]propyl (9*Z*)-9-eicosenoate

2 References | 0 Reactions | 0 Suppliers

Key Physical Properties	Value	Condition
Molecular Weight	801.27	-
Boiling Point (Predicted)	765.9±50.0 °C	Press: 760 Torr
Density (Predicted)	0.929±0.06 g/cm^3	Temp: 20 °C; Press: 760 Torr

22

875583-97-4

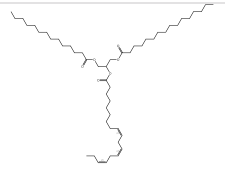

Double bond geometry shown

$C_{51}H_{92}O_6$

1-[[(1-Oxohexadecyl)oxy]methyl]-2-[(1-oxotetradecyl)oxy]ethyl (9*Z*,12*Z*,15*Z*)-9,12,15-octadecatrienoate

2 References | 0 Reactions | 0 Suppliers

Key Physical Properties	Value	Condition
Molecular Weight	801.27	-
Boiling Point (Predicted)	765.9±50.0 °C	Press: 760 Torr
Density (Predicted)	0.929±0.06 g/cm^3	Temp: 20 °C; Press: 760 Torr

23

869989-80-0

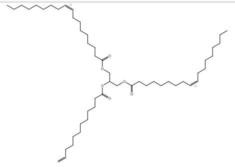

Double bond geometry shown

$C_{51}H_{92}O_6$

1,1'-[2-[(1-Oxo-11-dodecen-1-yl)oxy]-1,3-propanediyl] di-(9*Z*)-9-octadecenoate

2 References | 1 Reaction | 0 Suppliers

Key Physical Properties	Value	Condition
Molecular Weight	801.27	-
Boiling Point (Predicted)	764.6±40.0 °C	Press: 760 Torr
Density (Predicted)	0.927±0.06 g/cm^3	Temp: 20 °C; Press: 760 Torr

24

361437-84-5

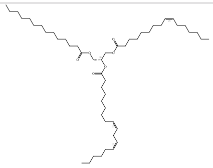

Absolute stereochemistry shown
Double bond geometry shown

$C_{51}H_{92}O_6$

9,12-Octadecadienoic acid (9*Z*,12*Z*)-, (1*R*)-1-[[[(9*Z*)-1-oxo-9-hexadecenyl]oxy]methyl]-2-[(1-oxotetradecyl)oxy]ethyl ester

2 References | 0 Reactions | 0 Suppliers

Key Physical Properties	Value	Condition
Molecular Weight	801.27	-
Boiling Point (Predicted)	765.9±50.0 °C	Press: 760 Torr
Density (Predicted)	0.929±0.06 g/cm^3	Temp: 20 °C; Press: 760 Torr

25

158415-38-4

28039-98-7

544-63-8

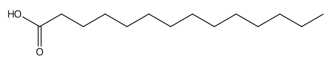

60-33-3

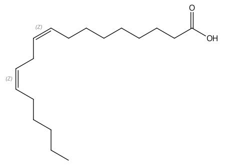

Double bond geometry shown

57-10-3

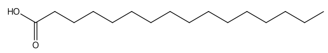

56-81-5

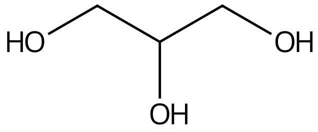

$C_{51}H_{92}O_6$

9,12-Octadecadienoic acid (9*Z*,12*Z*)-, ester with 1,2,3-propanetriol monohexadecanoate mono-(*Z*)-tetradecenoate

2 References | 0 Reactions | 0 Suppliers

There are no Key Physical Properties to display for this substance.

26

150736-57-5

28039-99-8

57-10-3

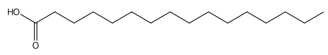

28039-98-7

544-63-8

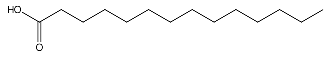

112-80-1

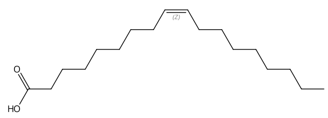

Double bond geometry shown

56-81-5

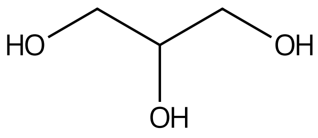

$C_{51}H_{92}O_6$

9-Octadecenoic acid (9*Z*)-, ester with 1,2,3-propanetriol mono-(*Z*)-hexadecenoate mono-(*Z*)-tetradecenoate

2 References

0 Reactions

0 Suppliers

There are no Key Physical Properties to display for this substance.

27

150446-34-7

28984-77-2

57-11-4

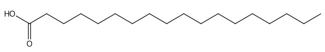

28039-99-8

57-10-3

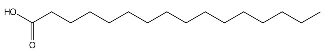

544-63-8

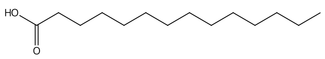

56-81-5

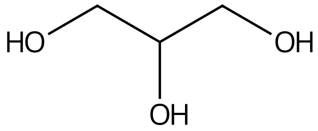

$C_{51}H_{92}O_6$

Octadecadienoic acid, ester with 1,2,3-propanetriol monohexadecenoate monotetradecanoate, (*Z*,*Z*,*Z*)-

2 References | 0 Reactions | 0 Suppliers

There are no Key Physical Properties to display for this substance.

28

147648-34-8

26764-26-1

57-11-4

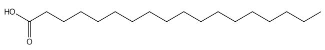

26764-25-0

57-11-4

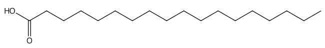

143-07-7

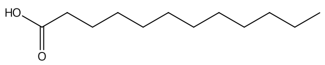

56-81-5

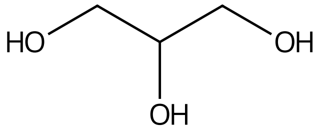

$C_{51}H_{92}O_6$

Octadecadienoic acid, ester with 1,2,3-propanetriol monododecanoate monooctadecenoate

2 References | 0 Reactions | 0 Suppliers

There are no Key Physical Properties to display for this substance.

29

147599-58-4

There are no Key Physical Properties to display for this substance.

27213-43-0

57-11-4

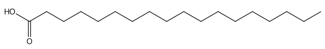

544-63-8

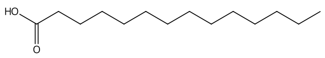

57-10-3

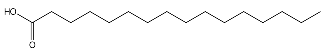

56-81-5

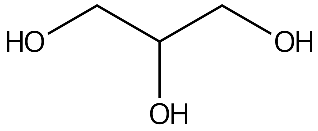

$C_{51}H_{92}O_6$

Octadecatrienoic acid, ester with 1,2,3-propanetriol monohexadecanoate monotetradecanoate, (*Z*,*Z*,*Z*)-

2 References | 0 Reactions | 0 Suppliers

30

127028-33-5

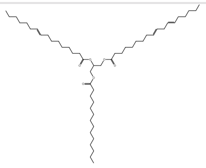

$C_{51}H_{92}O_6$

9,12-Octadecadienoic acid (9*Z*,12*Z*)-, 2-[[(9*Z*)-1-oxo-9-hexadecenyl]oxy]-3-[(1-oxotetradecyl)oxy]propyl ester

2 References | 0 Reactions | 0 Suppliers

Key Physical Properties	Value	Condition
Molecular Weight	801.27	-
Boiling Point (Predicted)	765.9±50.0 °C	Press: 760 Torr
Density (Predicted)	0.929±0.06 g/cm^3	Temp: 20 °C; Press: 760 Torr

31

64513-72-0

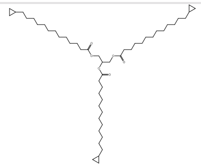

$C_{51}H_{92}O_6$

Cyclopropanetridecanoic acid, 1,2,3-propanetriyl ester

2 References | 0 Reactions | 0 Suppliers

Key Physical Properties	Value	Condition
Molecular Weight	801.27	-
Boiling Point (Predicted)	768.1±27.0 °C	Press: 760 Torr
Density (Predicted)	0.976±0.06 g/cm^3	Temp: 20 °C; Press: 760 Torr

32

2711865-01-7

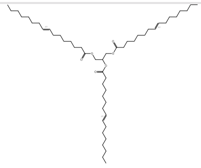

Double bond geometry shown

$C_{51}H_{92}O_6$

1 Reference | 0 Reactions | 0 Suppliers

Key Physical Properties	Value	Condition
Molecular Weight	801.27	-
Boiling Point (Predicted)	765.9±50.0 °C	Press: 760 Torr
Density (Predicted)	0.929±0.06 g/cm^3	Temp: 20 °C; Press: 760 Torr

33

2416372-30-8

26764-25-0

57-11-4

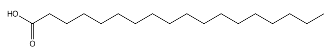

25447-95-4

57-10-3

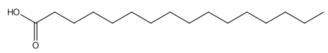

544-63-8

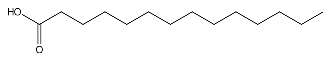

56-81-5

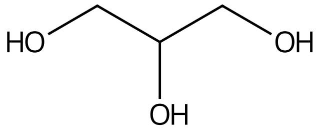

$C_{51}H_{92}O_{6}$

1 Reference	0 Reactions	0 Suppliers

There are no Key Physical Properties to display for this substance.

34

2271035-61-9

There are no Key Physical Properties to display for this substance.

26444-03-1

544-63-8

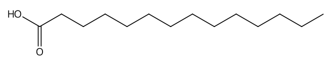

60-33-3

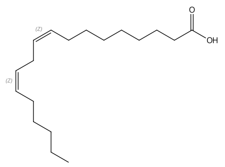

Double bond geometry shown

57-10-3

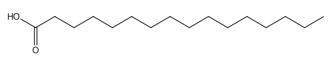

56-81-5

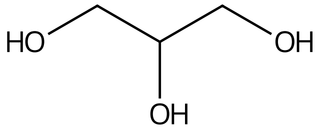

$C_{51}H_{92}O_6$

1 Reference	0 Reactions	0 Suppliers

35

2135451-76-0

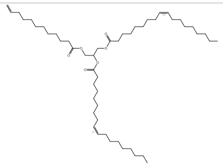

Double bond geometry shown

$C_{51}H_{92}O_6$

1 Reference	1 Reaction	0 Suppliers

Key Physical Properties	Value	Condition
Molecular Weight	801.27	-
Boiling Point (Predicted)	764.6±40.0 °C	Press: 760 Torr
Density (Predicted)	0.927±0.06 g/cm^3	Temp: 20 °C; Press: 760 Torr

36

2134133-25-6

56554-26-8

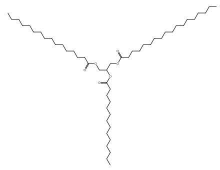

$C_{51}H_{92}O_6$

1 Reference | 0 Reactions | 0 Suppliers

There are no Key Physical Properties to display for this substance.

37

2134115-07-2

35405-55-1

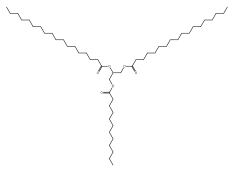

$C_{51}H_{92}O_6$

1 Reference | 0 Reactions | 0 Suppliers

There are no Key Physical Properties to display for this substance.

38

2134115-06-1

56554-26-8

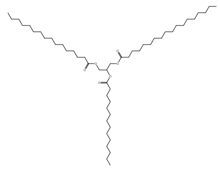

$C_{51}H_{92}O_6$

1 Reference | 0 Reactions | 0 Suppliers

There are no Key Physical Properties to display for this substance.

39

1788057-45-3

32839-24-0

57-10-3

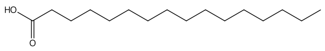

57-10-3

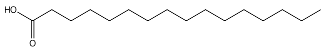

56-81-5

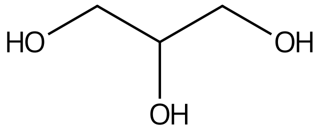

$C_{51}H_{92}O_6$

1 Reference | 0 Reactions | 0 Suppliers

There are no Key Physical Properties to display for this substance.

40

1675217-47-6

28039-98-7

544-63-8

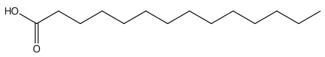

373-49-9

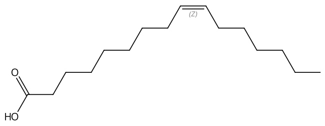

Double bond geometry shown

112-80-1

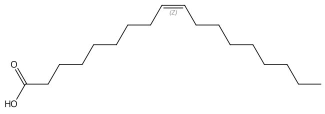

Double bond geometry shown

56-81-5

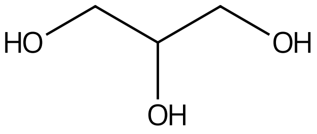

$C_{51}H_{92}O_6$

1 Reference

0 Reactions

0 Suppliers

There are no Key Physical Properties to display for this substance.

41

1623092-54-5

28039-99-8

57-10-3

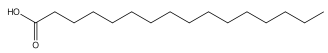

28039-98-7

544-63-8

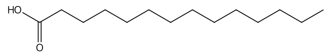

27104-13-8

57-11-4

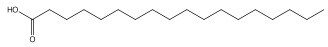

56-81-5

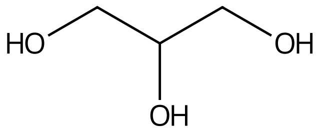

$C_{51}H_{92}O_6$

Octadecenoic acid, ester with 1,2,3-propanetriol mono-(*Z*)-hexadecenoate mono-(*Z*)-tetradecenoate, (*Z*)-

1 Reference | 0 Reactions | 0 Suppliers

There are no Key Physical Properties to display for this substance.

42

1623092-53-4

32839-19-3

143-07-7

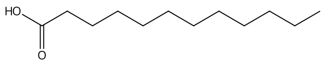

27104-13-8

57-11-4

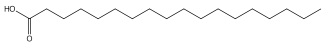

56-81-5

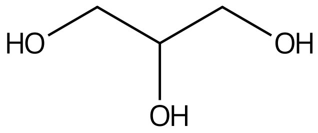

$C_{51}H_{92}O_6$

Octadecenoic acid, diester with 1,2,3-propanetriol mono-(*Z*)-dodecenoate, (*Z*,*Z*)-

1 Reference | 0 Reactions | 0 Suppliers

There are no Key Physical Properties to display for this substance.

43

1623092-52-3

28984-77-2

57-11-4

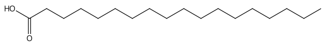

27104-13-8

57-11-4

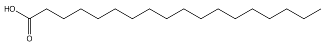

143-07-7

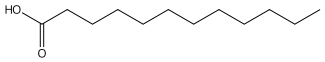

56-81-5

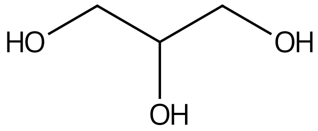

$C_{51}H_{92}O_6$

Octadecadienoic acid, ester with 1,2,3-propanetriol monododecanoate mono-(*Z*)-octadecenoate, (*Z*,*Z*)-

1 Reference | 0 Reactions | 0 Suppliers

There are no Key Physical Properties to display for this substance.

44

1615229-62-3

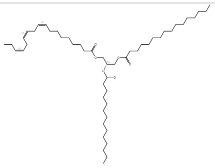

Absolute stereochemistry shown
Double bond geometry shown

$C_{51}H_{92}O_6$

(2*S*)-3-[(1-Oxohexadecyl)oxy]-2-[(1-oxotetradecyl)oxy]propyl (9*Z*,12*Z*,15*Z*)-9,12,15-octadecatrienoate

1 Reference | 0 Reactions | 1 Supplier

Key Physical Properties	Value	Condition
Molecular Weight	801.27	-
Boiling Point (Predicted)	765.9±50.0 °C	Press: 760 Torr
Density (Predicted)	0.929±0.06 g/cm^3	Temp: 20 °C; Press: 760 Torr

45

1615229-61-2

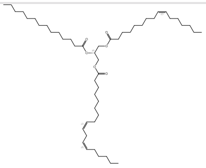

Absolute stereochemistry shown
Double bond geometry shown

$C_{51}H_{92}O_6$

(2*R*)-3-[[(9*Z*)-1-Oxo-9-hexadecen-1-yl]oxy]-2-[(1-oxotetradecyl)oxy]propyl (9*Z*,12*Z*)-9,12-octadecadienoate

1 Reference | 0 Reactions | 0 Suppliers

Key Physical Properties	Value	Condition
Molecular Weight	801.27	-
Boiling Point (Predicted)	765.9±50.0 °C	Press: 760 Torr
Density (Predicted)	0.929±0.06 g/cm^3	Temp: 20 °C; Press: 760 Torr

46

1498208-08-4

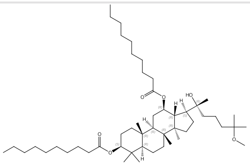

Absolute stereochemistry shown,
Rotation (+)

$C_{51}H_{92}O_6$

Dammarane-3,12,20-triol, 25-methoxy-, 3,12-didecanoate, (3β,12β,20*R*)-

1 Reference | 1 Reaction | 0 Suppliers

Key Physical Properties	Value	Condition
Molecular Weight	801.27	-
Boiling Point (Predicted)	752.8±55.0 °C	Press: 760 Torr
Density (Predicted)	1.00±0.1 g/cm^3	Temp: 20 °C; Press: 760 Torr
pKa (Predicted)	15.07±0.29	Most Acidic Temp: 25 °C

47

1329115-61-8

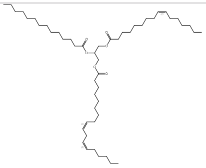

Double bond geometry shown

$C_{51}H_{92}O_6$

3-[[(9*Z*)-1-Oxo-9-hexadecen-1-yl]oxy]-2-[(1-oxotetradecyl)oxy]propyl (9*Z*,12*Z*)-9,12-octadecadienoate

1 Reference | 0 Reactions | 0 Suppliers

Key Physical Properties	Value	Condition
Molecular Weight	801.27	-
Boiling Point (Predicted)	765.9±50.0 °C	Press: 760 Torr
Density (Predicted)	0.929±0.06 g/cm^3	Temp: 20 °C; Press: 760 Torr

48

1189192-99-1

99131-43-8

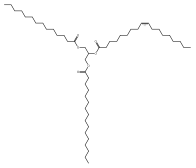

Double bond geometry shown

$C_{51}H_{92}O_6$

9-Octadecenoic acid (9*Z*)-, 1-[[(1-oxohexadecen-1-yl)oxy]methyl]-2-[(1-oxotetradecen-1-yl)oxy]ethyl ester

1 Reference | 0 Reactions | 0 Suppliers

There are no Key Physical Properties to display for this substance.

49

1189192-97-9

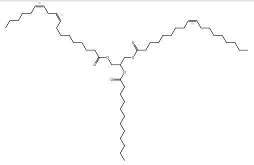

Double bond geometry shown

$C_{51}H_{92}O_6$

2-[(1-Oxododecyl)oxy]-3-[[(9*Z*)-1-oxo-9-octadecen-1-yl]oxy]propyl (9*Z*,12*Z*)-9,12-octadecadienoate

1 Reference | 0 Reactions | 0 Suppliers

Key Physical Properties	Value	Condition
Molecular Weight	801.27	-
Boiling Point (Predicted)	765.9±50.0 °C	Press: 760 Torr
Density (Predicted)	0.929±0.06 g/cm^3	Temp: 20 °C; Press: 760 Torr

50

1025825-90-4

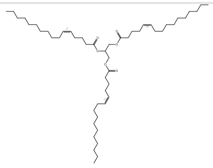

Double bond geometry shown

$C_{51}H_{92}O_6$

5-Hexadecenoic acid, 2,3-bis[[(5*Z*)-1-oxo-5-hexadecen-1-yl]oxy]propyl ester, (5*Z*)-

1 Reference | 0 Reactions | 0 Suppliers

Key Physical Properties	Value	Condition
Molecular Weight	801.27	-
Boiling Point (Predicted)	760.4±50.0 °C	Press: 760 Torr
Density (Predicted)	0.929±0.06 g/cm^3	Temp: 20 °C; Press: 760 Torr

51

950208-85-2

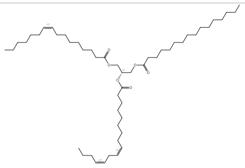

Absolute stereochemistry shown
Double bond geometry shown

$C_{51}H_{92}O_6$

(1*S*)-1-[[[(9*Z*)-1-Oxo-9-hexadecen-1-yl]oxy]methyl]-2-[(1-oxohexadecyl)oxy]ethyl (9*Z*,12*Z*)-9,12-hexadecadienoate

1 Reference | 0 Reactions | 0 Suppliers

Key Physical Properties	Value	Condition
Molecular Weight	801.27	-
Boiling Point (Predicted)	765.9±50.0 °C	Press: 760 Torr
Density (Predicted)	0.929±0.06 g/cm^3	Temp: 20 °C; Press: 760 Torr

52

906067-04-7

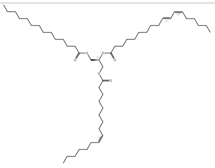

Absolute stereochemistry shown
Double bond geometry shown

$C_{51}H_{92}O_6$

(1*R*)-1-[[[(9*Z*)-1-Oxo-9-hexadecen-1-yl]oxy]methyl]-2-[(1-oxotetradecyl)oxy]ethyl (10*E*,12*Z*)-10,12-octadecadienoate

1 Reference | 0 Reactions | 0 Suppliers

Key Physical Properties	Value	Condition
Molecular Weight	801.27	-
Boiling Point (Predicted)	771.3±40.0 °C	Press: 760 Torr
Density (Predicted)	0.929±0.06 g/cm^3	Temp: 20 °C; Press: 760 Torr

53

887566-81-6

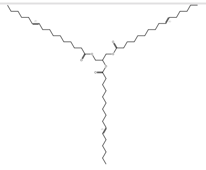

Double bond geometry shown

$C_{51}H_{92}O_6$

10-Hexadecenoic acid, 1,2,3-propanetriyl ester, (10*E*,10'*E*,10''*E*)-

1 Reference | 1 Reaction | 0 Suppliers

Key Physical Properties	Value	Condition
Molecular Weight	801.27	-
Boiling Point (Predicted)	765.9±50.0 °C	Press: 760 Torr
Density (Predicted)	0.929±0.06 g/cm^3	Temp: 20 °C; Press: 760 Torr

54

887566-79-2

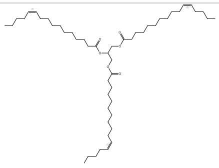

Double bond geometry shown

$C_{51}H_{92}O_6$

11-Hexadecenoic acid, 1,2,3-propanetriyl ester, (11*Z*,11'*Z*,11''*Z*)-

1 Reference | 1 Reaction | 0 Suppliers

Key Physical Properties	Value	Condition
Molecular Weight	801.27	-
Boiling Point (Predicted)	757.3±27.0 °C	Press: 760 Torr
Density (Predicted)	0.929±0.06 g/cm^3	Temp: 20 °C; Press: 760 Torr

55

867062-57-5

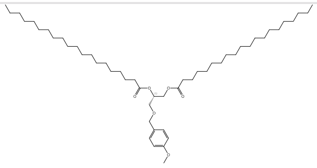

Absolute stereochemistry shown

$C_{51}H_{92}O_6$

Eicosanoic acid, (1*S*)-1-[[(4-methoxyphenyl)methoxy]methyl]-1,2-ethanediyl ester

1 Reference | 3 Reactions | 0 Suppliers

Key Physical Properties	Value	Condition
Molecular Weight	801.27	-
Boiling Point (Predicted)	786.3±55.0 °C	Press: 760 Torr
Density (Predicted)	0.939±0.06 g/cm^3	Temp: 20 °C; Press: 760 Torr

56

713123-68-3

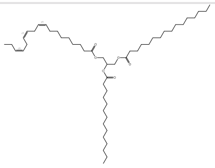

Double bond geometry shown

$C_{51}H_{92}O_6$

3-[(1-Oxohexadecyl)oxy]-2-[(1-oxotetradecyl)oxy]propyl (9*Z*,12*Z*,15*Z*)-9,12,15-octadecatrienoate

1 Reference | 0 Reactions | 0 Suppliers

Key Physical Properties	Value	Condition
Molecular Weight	801.27	-
Boiling Point (Predicted)	765.9±50.0 °C	Press: 760 Torr
Density (Predicted)	0.929±0.06 g/cm^3	Temp: 20 °C; Press: 760 Torr

57

355807-70-4

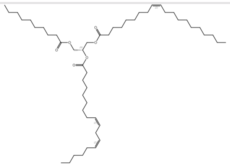

Absolute stereochemistry shown
Double bond geometry shown

$C_{51}H_{92}O_6$

(2*R*)-3-[(1-Oxodecyl)oxy]-2-[[(9*Z*,12*Z*)-1-oxo-9,12-octadecadien-1-yl]oxy]propyl (9*Z*)-9-eicosenoate

1 Reference | 0 Reactions | 0 Suppliers

Key Physical Properties	Value	Condition
Molecular Weight	801.27	-
Boiling Point (Predicted)	765.9±50.0 °C	Press: 760 Torr
Density (Predicted)	0.929±0.06 g/cm^3	Temp: 20 °C; Press: 760 Torr

58

296788-62-0

121250-47-3

57-11-4

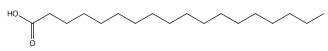

143-07-7

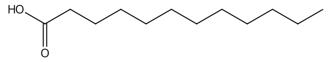

112-79-8

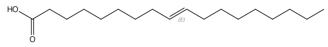

Double bond geometry shown

56-81-5

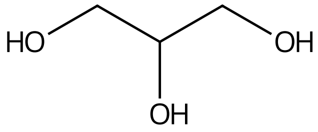

$C_{51}H_{92}O_6$

Octadecadienoic acid, ester with 1,2,3-propanetriol monododecanoate mono-(9*E*)-9-octadecenoate

1 Reference | 0 Reactions | 0 Suppliers

There are no Key Physical Properties to display for this substance.

59

296788-61-9

There are no Key Physical Properties to display for this substance.

121250-47-3

57-11-4

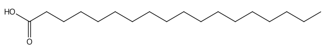

143-07-7

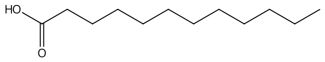

112-80-1

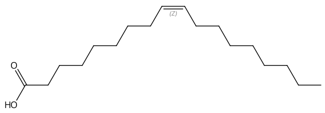

Double bond geometry shown

56-81-5

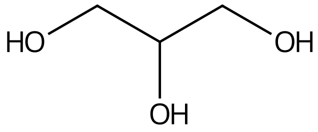

$C_{51}H_{92}O_{6}$

Octadecadienoic acid, ester with 1,2,3-propanetriol monododecanoate mono-(9*Z*)-9-octadecenoate

1 Reference | 0 Reactions | 0 Suppliers

60

224619-80-1

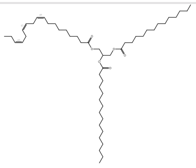

Double bond geometry shown

$C_{51}H_{92}O_6$

2-[(1-Oxohexadecyl)oxy]-3-[(1-oxotetradecyl)oxy]propyl (9*Z*,12*Z*,15*Z*)-9,12,15-octadecatrienoate

1 Reference | 0 Reactions | 0 Suppliers

Key Physical Properties	Value	Condition
Molecular Weight	801.27	-
Boiling Point (Predicted)	765.9±50.0 °C	Press: 760 Torr
Density (Predicted)	0.929±0.06 g/cm^3	Temp: 20 °C; Press: 760 Torr

61

213036-25-0

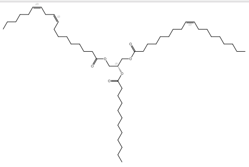

Absolute stereochemistry shown
Double bond geometry shown

$C_{51}H_{92}O_6$

(2*S*)-2-[(1-Oxododecyl)oxy]-3-[[(9*Z*)-1-oxo-9-octadecen-1-yl]oxy]propyl (9*Z*,12*Z*)-9,12-octadecadienoate

1 Reference | 0 Reactions | 0 Suppliers

Key Physical Properties	Value	Condition
Molecular Weight	801.27	-
Boiling Point (Predicted)	765.9±50.0 °C	Press: 760 Torr
Density (Predicted)	0.929±0.06 g/cm^3	Temp: 20 °C; Press: 760 Torr

62

213036-24-9

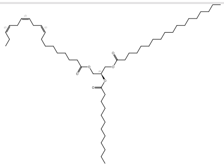

Absolute stereochemistry shown
Double bond geometry shown

$C_{51}H_{92}O_6$

(2*R*)-2-[(1-Oxododecyl)oxy]-3-[(1-oxooct adecyl)oxy]propyl (9*Z*,12*Z*,15*Z*)-9,12,15-octadecatrienoate

1 Reference | 0 Reactions | 0 Suppliers

Key Physical Properties	Value	Condition
Molecular Weight	801.27	-
Boiling Point (Predicted)	765.9±50.0 °C	Press: 760 Torr
Density (Predicted)	0.929±0.06 g/cm^3	Temp: 20 °C; Press: 760 Torr

63

150821-88-8

27213-43-0

57-11-4

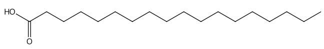

143-07-7

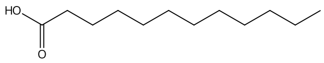

57-11-4

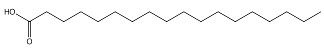

56-81-5

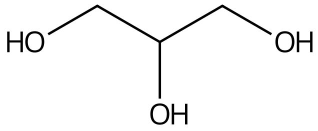

$C_{51}H_{92}O_6$

Octadecatrienoic acid, ester with 1,2,3-propan etriol monododecanoate monooctad ecanoate, (*Z*,*Z*,*Z*)-

1 Reference | 0 Reactions | 0 Suppliers

There are no Key Physical Properties to display for this substance.

64

150736-54-2

28984-77-2

57-11-4

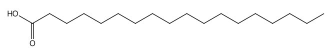

28039-98-7

544-63-8

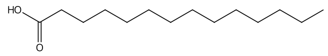

57-10-3

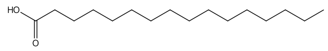

56-81-5

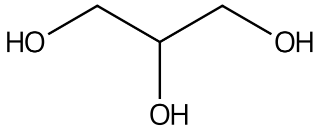

$C_{51}H_{92}O_6$

Octadecadienoic acid, ester with 1,2,3-propanetriol monohexadecanoate monotetradecenoate, (*Z*,*Z*,*Z*)-

1 Reference | 0 Reactions | 0 Suppliers

There are no Key Physical Properties to display for this substance.

65

150653-67-1

28984-77-2

57-11-4

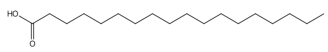

143-07-7

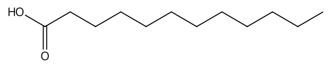

112-80-1

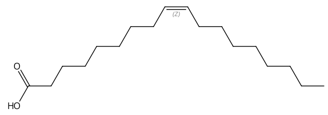

Double bond geometry shown

56-81-5

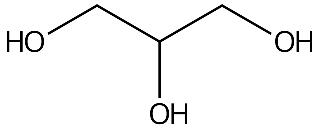

$C_{51}H_{92}O_6$

Octadecadienoic acid, ester with 1,2,3-propanetriol monododecanoate mono-9-octadecenoate, (*Z*,*Z*,*Z*)-

1 Reference | 0 Reactions | 0 Suppliers

There are no Key Physical Properties to display for this substance.

66

150446-51-8

1207371-40-1

57-11-4

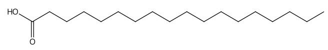

32839-28-4

506-30-9

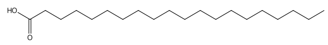

334-48-5

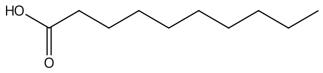

56-81-5

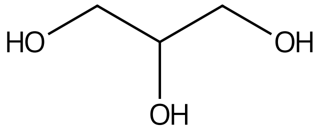

$C_{51}H_{92}O_6$

Eicosadienoic acid, ester with 1,2,3-propanetriol monodecanoate mono[9(*or* 11)-octadecenoate], (*Z*,*Z*,*Z*)-

1 Reference | 0 Reactions | 0 Suppliers

There are no Key Physical Properties to display for this substance.

67

150446-21-2

1207371-40-1

57-11-4

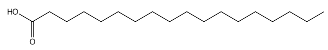

28984-77-2

57-11-4

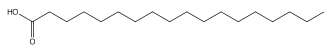

143-07-7

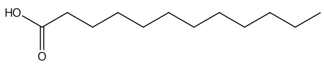

56-81-5

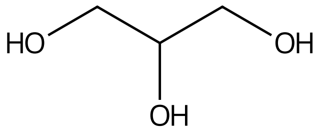

$C_{51}H_{92}O_6$

Octadecadienoic acid, ester with 1,2,3-propanetriol monododecanoate mono[9(*or* 11)-octadecenoate], (*Z*,*Z*,*Z*)-

1 Reference

0 Reactions

0 Suppliers

There are no Key Physical Properties to display for this substance.

68

122607-92-5

544-63-8

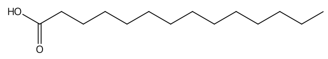

506-26-3

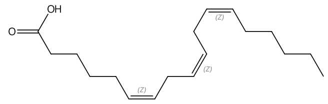

Double bond geometry shown

57-10-3

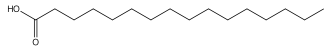

56-81-5

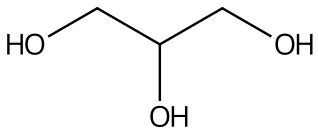

$C_{51}H_{92}O_{6}$

6,9,12-Octadecatrienoic acid, monoester with 1,2,3-propanetriol monohexadecanoate monotetradecanoate, (*Z*,*Z*,*Z*)-

1 Reference | 0 Reactions | 0 Suppliers

There are no Key Physical Properties to display for this substance.

69

118450-52-5

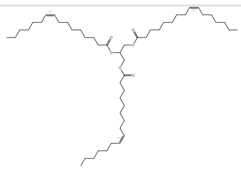

Double bond geometry shown

$C_{51}H_{92}O_{6}$

9-Hexadecenoic acid, 1,2,3-propanetriyl ester, labeled with carbon-14, (*Z*,*Z*,*Z*)-

1 Reference | 1 Reaction | 0 Suppliers

Key Physical Properties	Value	Condition
Molecular Weight	801.27	-

70

116111-46-7

116111-45-6

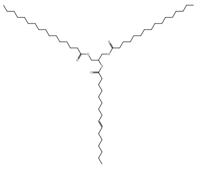

$C_{51}H_{92}O_6$

Hexadecadienoic acid, 2-[(1-oxo-9-hexadecenyl)oxy]-3-[(1-oxohexadecyl)oxy]propyl ester

1 Reference | 0 Reactions | 0 Suppliers

There are no Key Physical Properties to display for this substance.

71

116111-44-5

116111-43-4

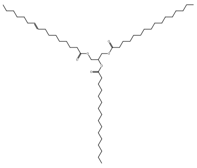

$C_{51}H_{92}O_6$

Hexadecadienoic acid, 1-[[(1-oxo-9-hexadecenyl)oxy]methyl]-2-[(1-oxohexadecyl)oxy]ethyl ester

1 Reference | 0 Reactions | 0 Suppliers

There are no Key Physical Properties to display for this substance.

72

110089-71-9

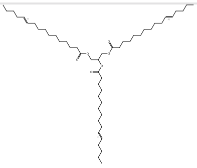

Double bond geometry shown

$C_{51}H_{92}O_6$

11-Hexadecenoic acid, 1,2,3-propanetriyl ester, (*E*,*E*,*E*)-

1 Reference | 0 Reactions | 0 Suppliers

Key Physical Properties	Value	Condition
Molecular Weight	801.27	-
Boiling Point (Predicted)	757.3±27.0 °C	Press: 760 Torr
Density (Predicted)	0.929±0.06 g/cm^3	Temp: 20 °C; Press: 760 Torr

73

1189193-00-7

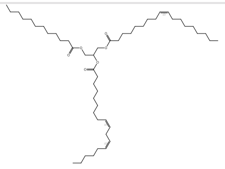

Double bond geometry shown

$C_{51}H_{92}O_6$

1-[[(1-Oxododecyl)oxy]methyl]-2-[[(9*Z*)-1-oxo-9-octadecen-1-yl]oxy]ethyl (9*Z*,12*Z*)-9,12-octadecadienoate

0 References | 0 Reactions | 0 Suppliers

Key Physical Properties	Value	Condition
Molecular Weight	801.27	-
Boiling Point (Predicted)	765.9±50.0 °C	Press: 760 Torr
Density (Predicted)	0.929±0.06 g/cm^3	Temp: 20 °C; Press: 760 Torr

74

35017-30-2

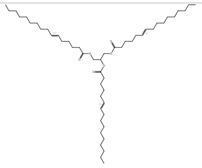

Double bond geometry shown

$C_{51}H_{92}O_6$

6-Hexadecenoic acid, 1,2,3-propanetriyl ester, (*E*,*E*,*E*)-

0 References | 0 Reactions | 0 Suppliers

Key Physical Properties	Value	Condition
Molecular Weight	801.27	-
Boiling Point (Predicted)	765.9±50.0 °C	Press: 760 Torr
Density (Predicted)	0.929±0.06 g/cm^3	Temp: 20 °C; Press: 760 Torr

Copyright © 2022 American Chemical Society (ACS). All Rights Reserved.
Internal use only. Redistribution is subject to the terms of your SciFinder^n License Agreement and CAS information Use Policies.
