# Supplementary material for: Efficient Green Extraction of Nutraceutical Compounds from Nannochloropsis gaditana: A Comparative Electrospray Ionization LC-MS and GC-MS Analysis for Lipid Profiling
Source: Foods. 2024 Dec 19;13(24):4117. doi: 10.3390/foods13244117 (PMC11675803; doi:10.3390/foods13244117)
Supplement: Supplementary file 1 [file foods-13-04117-s001.zip › MS Results/HPLC-MS PLE -Results-MC/Pico a 37.5 min_C51H94O6.pdf]

CAS SciFinder^{n}

Task History

Initiating Search

November 25, 2022, 2:15PM

Substances:

Advanced Search:

Molecular Formula: C51H94O6

Search Tasks

Task	Search Type	View
Exported: Returned Substance Results (81)	Substances	View Results

Copyright © 2022 American Chemical Society (ACS). All Rights Reserved.
Internal use only. Redistribution is subject to the terms of your SciFinder^{n} License Agreement and CAS information Use Policies.

CAS SciFinderⁿ

Substances (10)

View in SciFinderⁿ

1

87973-00-0

544-63-8

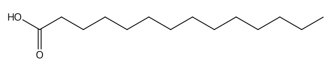

60-33-3

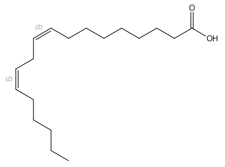

Double bond geometry shown

57-10-3

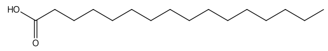

56-81-5

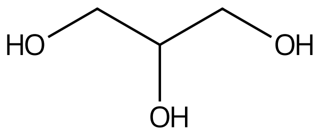

$C_{51}H_{94}O_6$

Triglyceride LMP

84 References | 0 Reactions | 0 Suppliers

There are no Key Physical Properties to display for this substance.

Spectra

2

28518-59-4

143-07-7

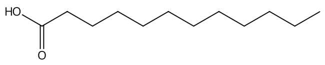

112-80-1

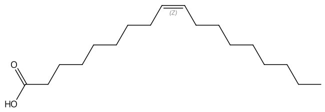

Double bond geometry shown

56-81-5

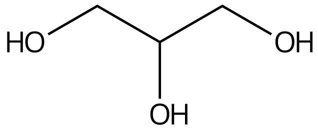

$C_{51}H_{94}O_6$

Triglyceride LaOO

47 References | 0 Reactions | 0 Suppliers

There are no Key Physical Properties to display for this substance.

3

28630-67-3

373-49-9

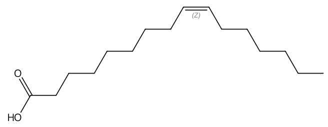

Double bond geometry shown

57-10-3

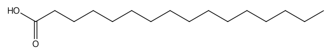

56-81-5

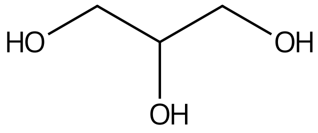

$C_{51}H_{94}O_6$

Triglyceride PPoPo

37 References | 0 Reactions | 0 Suppliers

There are no Key Physical Properties to display for this substance.

4

125527-78-8

There are no Key Physical Properties to display for this substance.

544-63-8

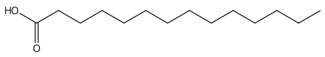

373-49-9

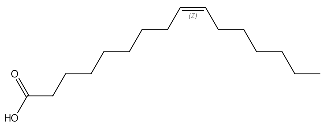

Double bond geometry shown

112-80-1

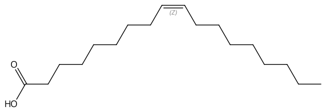

Double bond geometry shown

56-81-5

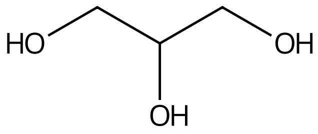

$C_{51}H_{94}O_6$

Triglyceride MOPo

27 References	0 Reactions	0 Suppliers

5

4016-52-8

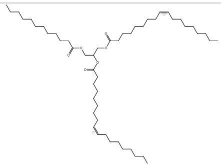

Double bond geometry shown

$C_{51}H_{94}O_6$

Glyceryl 1,2-dioleate 3-laurate

21 References	6 Reactions	9 Suppliers

Key Physical Properties	Value	Condition
Molecular Weight	803.29	-
Melting Point (Experimental)	5.5-6.5 °C	-
Boiling Point (Predicted)	766.6±40.0 °C	Press: 760 Torr
Density (Predicted)	0.924±0.06 g/cm^3	Temp: 20 °C; Press: 760 Torr

Experimental Properties

6

105595-41-3

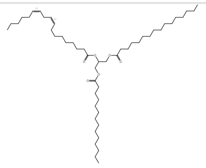

Double bond geometry shown

$C_{51}H_{94}O_6$

1-[[(1-Oxohexadecyl)oxy]methyl]-2-[(1-oxotetradecyl)oxy]ethyl (9*Z*,12*Z*)-9,12-octadecadienoate

16 References | 0 Reactions | 7 Suppliers

Key Physical Properties	Value	Condition
Molecular Weight	803.29	-
Boiling Point (Predicted)	766.1±40.0 °C	Press: 760 Torr
Density (Predicted)	0.924±0.06 g/cm^3	Temp: 20 °C; Press: 760 Torr
Spectra		

7

74257-22-0

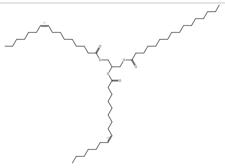

Double bond geometry shown

$C_{51}H_{94}O_6$

1,1'-[1-[[(1-Oxohexadecyl)oxy]methyl]-1,2-ethanediyl] di-(9*Z*)-9-hexadecenoate

16 References | 0 Reactions | 9 Suppliers

Key Physical Properties	Value	Condition
Molecular Weight	803.29	-
Boiling Point (Predicted)	766.6±40.0 °C	Press: 760 Torr
Density (Predicted)	0.924±0.06 g/cm^3	Temp: 20 °C; Press: 760 Torr
Spectra		

8

383189-71-7

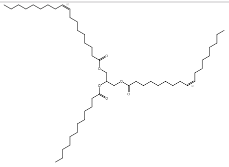

Double bond geometry shown

$C_{51}H_{94}O_6$

1,1'-[2-[(1-Oxododecyl)oxy]-1,3-propanediyl] di-(9*Z*)-9-octadecenoate

9 References | 9 Reactions | 9 Suppliers

Key Physical Properties	Value	Condition
Molecular Weight	803.29	-
Boiling Point (Predicted)	766.6±40.0 °C	Press: 760 Torr
Density (Predicted)	0.924±0.06 g/cm^3	Temp: 20 °C; Press: 760 Torr

9

127028-31-3

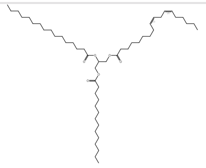

Double bond geometry shown

$C_{51}H_{94}O_6$

2-[(1-Oxohexadecyl)oxy]-3-[(1-oxotetradecyl)oxy]propyl (9*Z*,12*Z*)-9,12-octadecadienoate

9 References | 0 Reactions | 0 Suppliers

Key Physical Properties	Value	Condition
Molecular Weight	803.29	-
Boiling Point (Predicted)	766.1±40.0 °C	Press: 760 Torr
Density (Predicted)	0.924±0.06 g/cm^3	Temp: 20 °C; Press: 760 Torr

Spectra

10

127028-30-2

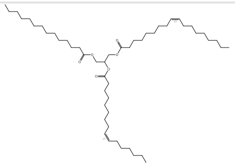

Double bond geometry shown

$C_{51}H_{94}O_6$

2-[[(9*Z*)-1-Oxo-9-hexadecen-1-yl]oxy]-3-[(1-oxotetradecyl)oxy]propyl (9*Z*)-9-octadecenoate

9 References | 0 Reactions | 0 Suppliers

Key Physical Properties	Value	Condition
Molecular Weight	803.29	-
Boiling Point (Predicted)	766.6±40.0 °C	Press: 760 Torr
Density (Predicted)	0.924±0.06 g/cm^3	Temp: 20 °C; Press: 760 Torr

Copyright © 2022 American Chemical Society (ACS). All Rights Reserved.
Internal use only. Redistribution is subject to the terms of your SciFinderⁿ License Agreement and CAS information Use Policies.
