# Supplementary material for: Efficient Green Extraction of Nutraceutical Compounds from Nannochloropsis gaditana: A Comparative Electrospray Ionization LC-MS and GC-MS Analysis for Lipid Profiling
Source: Foods. 2024 Dec 19;13(24):4117. doi: 10.3390/foods13244117 (PMC11675803; doi:10.3390/foods13244117)
Supplement: Supplementary file 1 [file foods-13-04117-s001.zip › MS Results/HPLC-MS PLE -Results-MC/Pico a 37.5 min_C53H99NO6.pdf]

CAS SciFinder^n

Task History

Initiating Search

November 25, 2022, 2:24PM

Substances:

Advanced Search:

Molecular Formula: C53H99NO6

Search Tasks

Task	Search Type	View
Exported: Returned Substance Results (5)	Substances	View Results

Copyright © 2022 American Chemical Society (ACS). All Rights Reserved.
Internal use only. Redistribution is subject to the terms of your SciFinder^n License Agreement and CAS information Use Policies.

CAS SciFinderⁿ

Substances (5)

View in SciFinderⁿ

1

1443522-77-7

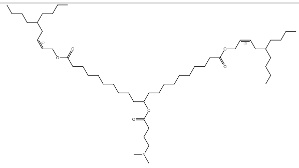

Double bond geometry shown

$C_{53}H_{99}NO_6$

1,21-Bis[(2*Z*)-5-butyl-2-nonen-1-yl] 11-[4-(dimethylamino)-1-oxobutoxy]heneicosanedioate

1 Reference | 1 Reaction | 0 Suppliers

Key Physical Properties	Value	Condition
Molecular Weight	846.36	-
Boiling Point (Predicted)	793.3±45.0 °C	Press: 760 Torr
Density (Predicted)	0.930±0.06 g/cm^3	Temp: 20 °C; Press: 760 Torr
pKa (Predicted)	9.37±0.28	Most Basic Temp: 25 °C

2

1443522-72-2

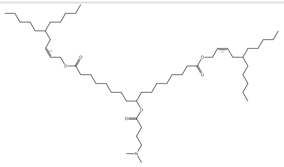

Double bond geometry shown

$C_{53}H_{99}NO_6$

Heptadecanedioic acid, 9-[4-(dimethylamino)-1-oxobutoxy]-, 1,17-bis[(2*Z*)-5-pentyl-2-decen-1-yl] ester

1 Reference | 0 Reactions | 0 Suppliers

Key Physical Properties	Value	Condition
Molecular Weight	846.36	-
Boiling Point (Predicted)	793.3±60.0 °C	Press: 760 Torr
Density (Predicted)	0.930±0.06 g/cm^3	Temp: 20 °C; Press: 760 Torr
pKa (Predicted)	9.37±0.28	Most Basic Temp: 25 °C

3

1443522-31-3

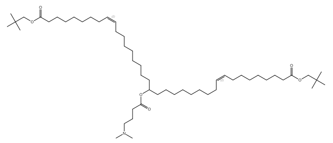

Double bond geometry shown

$C_{53}H_{99}NO_6$

1,37-Bis(2,2-dimethylpropyl) (9*Z*,28*Z*)-19-[4-(dimethylamino)-1-oxobutoxy]-9,28-heptatriacontadienedioate

1 Reference | 15 Reactions | 0 Suppliers

Key Physical Properties	Value	Condition
Molecular Weight	846.36	-
Boiling Point (Predicted)	788.5±55.0 °C	Press: 760 Torr
Density (Predicted)	0.931±0.06 g/cm^3	Temp: 20 °C; Press: 760 Torr
pKa (Predicted)	9.37±0.28	Most Basic Temp: 25 °C

4

1414586-43-8

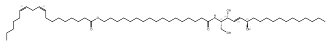

Absolute stereochemistry shown
Double bond geometry shown

$C_{53}H_{99}NO_6$

17-[[(1*S*,2*R*,3*E*,5*R*)-2,5-Dihydroxy-1-(hydroxymethyl)-3-heptadecen-1-yl]amino]-17-oxoheptadecyl (9*Z*,12*Z*)-9,12-octadecadienoate

1 Reference | 0 Reactions | 0 Suppliers

Key Physical Properties	Value	Condition
Molecular Weight	846.36	-
Boiling Point (Predicted)	884.1±65.0 °C	Press: 760 Torr
Density (Predicted)	0.953±0.06 g/cm^3	Temp: 20 °C; Press: 760 Torr
pKa (Predicted)	13.33±0.20	Most Acidic Temp: 25 °C

5

1219133-42-2

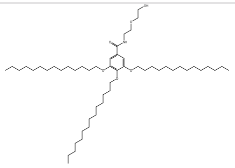

$C_{53}H_{99}NO_6$

N-[2-(2-Hydroxyethoxy)ethyl]-3,4,5-tris(tetradecyloxy)benzamide

1 Reference | 3 Reactions | 0 Suppliers

Key Physical Properties	Value	Condition
Molecular Weight	846.36	-
Melting Point (Experimental)	77.2-79.5 °C	-
Boiling Point (Predicted)	828.4±65.0 °C	Press: 760 Torr
Density (Predicted)	0.941±0.06 g/cm^3	Temp: 20 °C; Press: 760 Torr
pKa (Predicted)	13.56±0.46	Most Acidic Temp: 25 °C

Experimental Properties

Copyright © 2022 American Chemical Society (ACS). All Rights Reserved.
Internal use only. Redistribution is subject to the terms of your SciFinder^n License Agreement and CAS information Use Policies.
