# Supplementary material for: Efficient Green Extraction of Nutraceutical Compounds from Nannochloropsis gaditana: A Comparative Electrospray Ionization LC-MS and GC-MS Analysis for Lipid Profiling
Source: Foods. 2024 Dec 19;13(24):4117. doi: 10.3390/foods13244117 (PMC11675803; doi:10.3390/foods13244117)
Supplement: Supplementary file 1 [file foods-13-04117-s001.zip › MS Results/HPLC-MS PLE -Results-MC/Pico a 38.2 min_C53H98O6.pdf]

CAS SciFinder^n

Task History

Initiating Search

November 25, 2022, 2:34PM

Substances:

Advanced Search:

Molecular Formula: $C_{53}H_{98}O_6$

Search Tasks

Task	Search Type	View
Exported: Returned Substance Results (96)	Substances	View Results

Copyright © 2022 American Chemical Society (ACS). All Rights Reserved.
Internal use only. Redistribution is subject to the terms of your SciFinder^n License Agreement and CAS information Use Policies.

CAS SciFinderⁿ

Substances (10)

View in SciFinderⁿ

1

26836-30-6

60-33-3

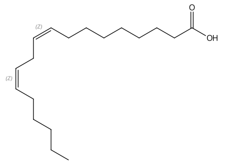

Double bond geometry shown

57-10-3

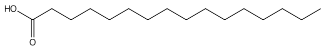

56-81-5

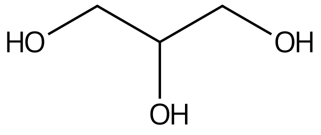

$C_{53}H_{98}O_6$

Triglyceride LPP

570 References | 0 Reactions | 0 Suppliers

There are no Key Physical Properties to display for this substance.

Spectra

2

2442-56-0

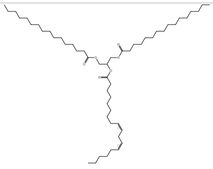

Double bond geometry shown

$C_{53}H_{98}O_6$

Triglyceride PLP,sn

248 References | 7 Reactions | 15 Suppliers

Key Physical Properties	Value	Condition
Molecular Weight	831.34	-
Melting Point (Experimental)	25.5-26 °C	-
Boiling Point (Predicted)	784.1±40.0 °C	Press: 760 Torr
Density (Predicted)	0.922±0.06 g/cm^3	Temp: 20 °C; Press: 760 Torr

Experimental Properties | Spectra

3

38703-18-3

373-49-9

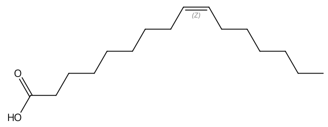

Double bond geometry shown

112-80-1

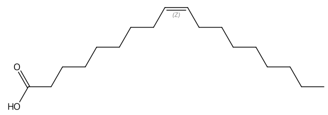

Double bond geometry shown

57-10-3

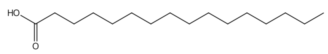

56-81-5

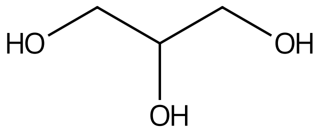

$C_{53}H_{98}O_6$

Triglyceride OPPo

134 References | 0 Reactions | 0 Suppliers

There are no Key Physical Properties to display for this substance.

Spectra

4

2535-35-5

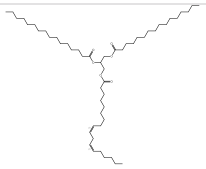

Double bond geometry shown

$C_{53}H_{98}O_6$

1,2-Dipalmito-3-linolein

112 References | 6 Reactions | 13 Suppliers

Key Physical Properties	Value	Condition
Molecular Weight	831.34	-
Melting Point (Experimental)	36.5-38 °C	-
Boiling Point (Predicted)	784.1±40.0 °C	Press: 760 Torr
Density (Predicted)	0.922±0.06 g/cm^3	Temp: 20 °C; Press: 760 Torr

Experimental Properties | Spectra

5

99431-68-2

544-63-8

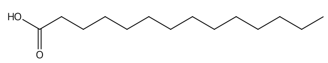

112-80-1

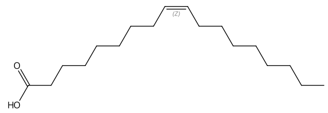

Double bond geometry shown

56-81-5

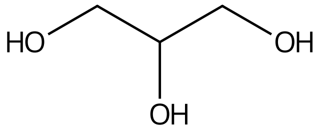

$C_{53}H_{98}O_6$

Triglyceride MOO

71 References | 0 Reactions | 0 Suppliers

There are no Key Physical Properties to display for this substance.

6

84234-82-2

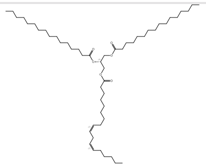

Absolute stereochemistry shown
Double bond geometry shown

$C_{53}H_{98}O_6$

(2*R*)-2,3-Bis[(1-oxohexadecyl)oxy]propyl (9*Z*,12*Z*)-9,12-octadecadienoate

43 References | 1 Reaction | 2 Suppliers

Key Physical Properties	Value	Condition
Molecular Weight	831.34	-
Boiling Point (Predicted)	784.1±40.0 °C	Press: 760 Torr
Density (Predicted)	0.922±0.06 g/cm^3	Temp: 20 °C; Press: 760 Torr

7

35804-98-9

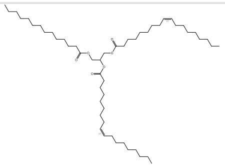

Double bond geometry shown

$C_{53}H_{98}O_6$

1,1'-[1-[[(1-Oxotetradecyl)oxy]methyl]-1,2-ethanediyl] di-(9*Z*)-9-octadecenoate

29 References | 1 Reaction | 11 Suppliers

Key Physical Properties	Value	Condition
Molecular Weight	831.34	-
Melting Point (Experimental)	12.5-13.5 °C	-
Boiling Point (Predicted)	784.6±40.0 °C	Press: 760 Torr
Density (Predicted)	0.922±0.06 g/cm^3	Temp: 20 °C; Press: 760 Torr

Experimental Properties | Spectra

8

153548-86-8

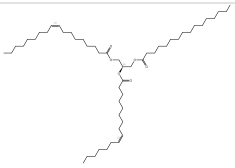

Absolute stereochemistry shown
Double bond geometry shown

$C_{53}H_{98}O_6$

(2*R*)-2-[[(9*Z*)-1-Oxo-9-hexadecen-1-yl]oxy]-3-[(1-oxohexadecyl)oxy]propyl (9*Z*)-9-octadecenoate

19 References | 0 Reactions | 1 Supplier

Key Physical Properties	Value	Condition
Molecular Weight	831.34	-
Boiling Point (Predicted)	784.6±40.0 °C	Press: 760 Torr
Density (Predicted)	0.922±0.06 g/cm^3	Temp: 20 °C; Press: 760 Torr

9

102491-55-4

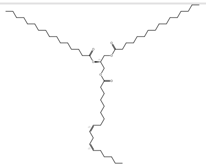

Absolute stereochemistry shown, Rotation (-)
Double bond geometry shown

$C_{53}H_{98}O_6$

(2*S*)-2,3-Bis[(1-oxohexadecyl)oxy]propyl (9*Z*,12*Z*)-9,12-octadecadienoate

17 References | 5 Reactions | 1 Supplier

Key Physical Properties	Value	Condition
Molecular Weight	831.34	-
Boiling Point (Predicted)	784.1±40.0 °C	Press: 760 Torr
Density (Predicted)	0.922±0.06 g/cm^3	Temp: 20 °C; Press: 760 Torr

Experimental Properties

10

158298-95-4

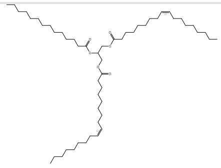

Double bond geometry shown

$C_{53}H_{98}O_6$

Triglyceride OMO,sn

16 References | 2 Reactions | 11 Suppliers

Key Physical Properties	Value	Condition
Molecular Weight	831.34	-
Boiling Point (Predicted)	784.6±40.0 °C	Press: 760 Torr
Density (Predicted)	0.922±0.06 g/cm^3	Temp: 20 °C; Press: 760 Torr
Spectra		

Copyright © 2022 American Chemical Society (ACS). All Rights Reserved.
Internal use only. Redistribution is subject to the terms of your SciFinderⁿ License Agreement and CAS information Use Policies.
