# Supplementary material for: Efficient Green Extraction of Nutraceutical Compounds from Nannochloropsis gaditana: A Comparative Electrospray Ionization LC-MS and GC-MS Analysis for Lipid Profiling
Source: Foods. 2024 Dec 19;13(24):4117. doi: 10.3390/foods13244117 (PMC11675803; doi:10.3390/foods13244117)
Supplement: Supplementary file 1 [file foods-13-04117-s001.zip › MS Results/HPLC-MS PLE -Results-MC/Pico a 39.0 min_C55H102O6.pdf]

SciFinder^n

Task History

Initiating Search

November 25, 2022, 2:45PM

Substances:

Advanced Search:

Molecular Formula: $C_{55}H_{102}O_6$

Search Tasks

Task	Search Type	View
Exported: Returned Substance Results (136)	Substances	View Results

Copyright © 2022 American Chemical Society (ACS). All Rights Reserved.
Internal use only. Redistribution is subject to the terms of your SciFinder^n License Agreement and CAS information Use Policies.

CAS SciFinderⁿ

Substances (10)

View in SciFinderⁿ

1

27071-84-7

112-80-1

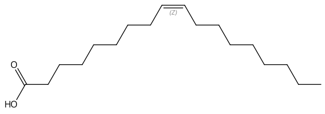

Double bond geometry shown

57-10-3

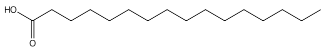

56-81-5

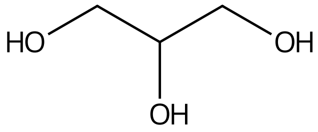

$C_{55}H_{102}O_6$

Triglyceride OOP

863 References | 3 Reactions | 0 Suppliers

There are no Key Physical Properties to display for this substance.

Spectra

2

1716-07-0

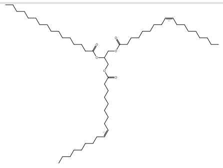

Double bond geometry shown

$C_{55}H_{102}O_6$

Triglyceride OPO

414 References | 69 Reactions | 25 Suppliers

Key Physical Properties	Value	Condition
Molecular Weight	859.40	-
Melting Point (Experimental)	19 °C	-
Boiling Point (Predicted)	802.2±45.0 °C	Press: 760 Torr
Density (Experimental)	0.9046 g/cm^3	Temp: 20 °C

Experimental Properties | Spectra

3

2190-30-9

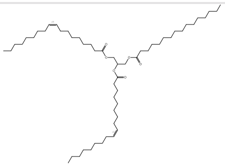

Double bond geometry shown

$C_{55}H_{102}O_6$

1,2-Dioleoyl-3-palmitoylglycerol

341 References | 13 Reactions | 37 Suppliers

Key Physical Properties	Value	Condition
Molecular Weight	859.40	-
Melting Point (Experimental)	18-19 °C	-
Boiling Point (Predicted)	802.2±45.0 °C	Press: 760 Torr
Density (Experimental)	0.9027 g/cm^3	Temp: 24 °C

Experimental Properties | Spectra

4

26836-32-8

60-33-3

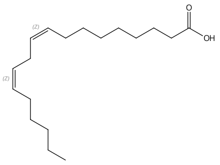

Double bond geometry shown

57-11-4

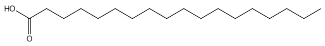

57-10-3

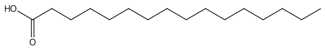

56-81-5

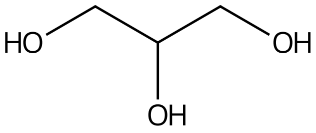

$C_{55}H_{102}O_6$

Triglyceride LPSt

247 References | 0 Reactions | 0 Suppliers

There are no Key Physical Properties to display for this substance.

Spectra

5

14960-35-1

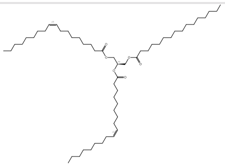

Absolute stereochemistry shown
Double bond geometry shown

$C_{55}H_{102}O_6$

Triglyceride POO,sn

119 References | 2 Reactions | 2 Suppliers

Key Physical Properties	Value	Condition
Molecular Weight	859.40	-
Melting Point (Experimental)	19 °C	-
Boiling Point (Predicted)	802.2±45.0 °C	Press: 760 Torr
Density (Predicted)	0.919±0.06 g/cm^3	Temp: 20 °C; Press: 760 Torr

Experimental Properties | Spectra

6

14863-26-4

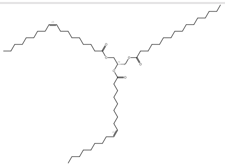

Absolute stereochemistry shown,
Rotation (-)
Double bond geometry shown

$C_{55}H_{102}O_6$

Triglyceride OOP,sn

71 References | 7 Reactions | 1 Supplier

Key Physical Properties	Value	Condition
Molecular Weight	859.40	-
Boiling Point (Predicted)	802.2±45.0 °C	Press: 760 Torr
Density (Predicted)	0.919±0.06 g/cm^3	Temp: 20 °C; Press: 760 Torr

Experimental Properties

7

2190-12-7

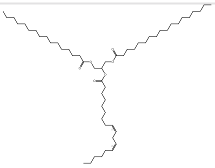

Double bond geometry shown

$C_{55}H_{102}O_6$

1-Palmito-2-linoleo-3-stearin

66 References | 0 Reactions | 12 Suppliers

Key Physical Properties	Value	Condition
Molecular Weight	859.40	-
Melting Point (Experimental)	10-10.5 °C	-
Boiling Point (Predicted)	801.7±45.0 °C	Press: 760 Torr
Density (Predicted)	0.919±0.06 g/cm^3	Temp: 20 °C; Press: 760 Torr

Experimental Properties | Spectra

8

102517-53-3

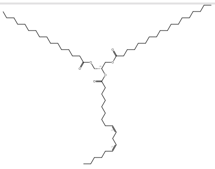

Absolute stereochemistry shown
Double bond geometry shown

$C_{55}H_{102}O_6$

Triglyceride PLSt,sn

40 References | 0 Reactions | 0 Suppliers

Key Physical Properties	Value	Condition
Molecular Weight	859.40	-
Boiling Point (Predicted)	801.7±45.0 °C	Press: 760 Torr
Density (Predicted)	0.919±0.06 g/cm^3	Temp: 20 °C; Press: 760 Torr

9

2680-57-1

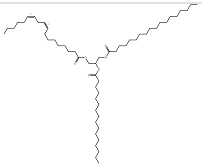

Double bond geometry shown

$C_{55}H_{102}O_6$

9,12-Octadecadienoic acid (9*Z*,12*Z*)-, 2-[(1-oxohexadecyl)oxy]-3-[(1-oxooctadecyl)oxy]propyl ester

29 References | 0 Reactions | 0 Suppliers

Key Physical Properties	Value	Condition
Molecular Weight	859.40	-
Melting Point (Experimental)	34-34.5 °C	-
Boiling Point (Predicted)	801.7±45.0 °C	Press: 760 Torr
Density (Predicted)	0.919±0.06 g/cm^3	Temp: 20 °C; Press: 760 Torr

Experimental Properties | Spectra

10

2534-96-5

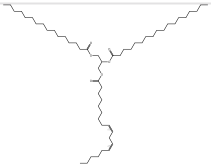

Double bond geometry shown

$C_{55}H_{102}O_6$

3-[(1-Oxohexadecyl)oxy]-2-[(1-oxooctadecyl)oxy]propyl (9*Z*,12*Z*)-9,12-octadecadienoate

28 References | 0 Reactions | 0 Suppliers

Key Physical Properties	Value	Condition
Molecular Weight	859.40	-
Melting Point (Experimental)	36-37 °C	-
Boiling Point (Predicted)	801.7±45.0 °C	Press: 760 Torr
Density (Predicted)	0.919±0.06 g/cm^3	Temp: 20 °C; Press: 760 Torr

Experimental Properties | Spectra

Copyright © 2022 American Chemical Society (ACS). All Rights Reserved.

Internal use only. Redistribution is subject to the terms of your SciFinder^n License Agreement and CAS information Use Policies.
