# Supplementary material for: Efficient Green Extraction of Nutraceutical Compounds from Nannochloropsis gaditana: A Comparative Electrospray Ionization LC-MS and GC-MS Analysis for Lipid Profiling
Source: Foods. 2024 Dec 19;13(24):4117. doi: 10.3390/foods13244117 (PMC11675803; doi:10.3390/foods13244117)
Supplement: Supplementary file 1 [file foods-13-04117-s001.zip › MS Results/HPLC-MS PLE -Results-MC/Pico a 39.0 min_C57H104O6.pdf]

SciFinder^n

Task History

Initiating Search

November 25, 2022, 2:41PM

Substances:

Advanced Search:

Molecular Formula: C57H104O6

Search Tasks

Task	Search Type	View
Exported: Returned Substance Results (216)	Substances	View Results

Copyright © 2022 American Chemical Society (ACS). All Rights Reserved.
Internal use only. Redistribution is subject to the terms of your SciFinder^n License Agreement and CAS information Use Policies.

SciFinder^n

Substances (10)

View in SciFinder^n

1

122-32-7

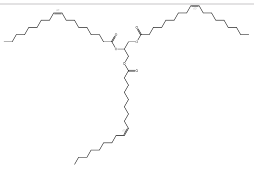

Double bond geometry shown

$C_{57}H_{104}O_6$

Triolein

10K References | 324 Reactions | 98 Suppliers

Key Physical Properties	Value	Condition
Molecular Weight	885.43	-
Melting Point (Experimental)	5.5 °C (approx)	-
Boiling Point (Experimental)	235-240 °C	Press: 15 Torr
Density (Experimental)	0.915 g/cm^3	Temp: 15 °C

Experimental Properties | Spectra

2

26836-38-4

There are no Key Physical Properties to display for this substance.

112-80-1

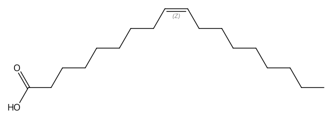

Double bond geometry shown

60-33-3

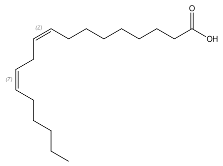

Double bond geometry shown

57-11-4

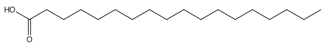

56-81-5

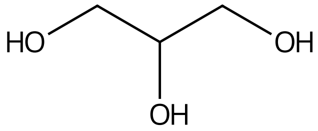

$C_{57}H_{104}O_6$

Triglyceride LOSt

370 References | 0 Reactions | 0 Suppliers

3

537-39-3

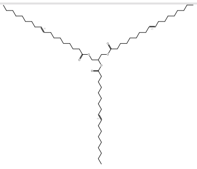

Double bond geometry shown

$C_{57}H_{104}O_6$

Trielaidin

236 References | 7 Reactions | 46 Suppliers

Key Physical Properties	Value	Condition
Molecular Weight	885.43	-
Melting Point (Experimental)	40.7 °C	-
Boiling Point (Predicted)	818.7±55.0 °C	Press: 760 Torr
Density (Predicted)	0.921±0.06 g/cm^3	Temp: 20 °C; Press: 760 Torr

Experimental Properties | Spectra

4

2190-14-9

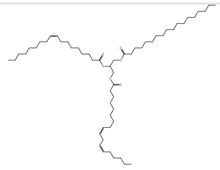

Double bond geometry shown

$C_{57}H_{104}O_6$

1-Linoleoyl-2-oleoyl-3-stearoyl-rac-glycerol

75 References | 1 Reaction | 24 Suppliers

Key Physical Properties	Value	Condition
Molecular Weight	885.43	-
Melting Point (Experimental)	-17--15 °C	-
Boiling Point (Predicted)	818.7±55.0 °C	Press: 760 Torr
Density (Experimental)	0.9044 g/cm^3	Temp: 20 °C

Experimental Properties | Spectra

5

7162-26-7

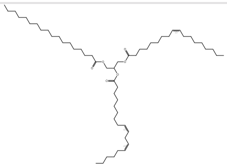

Double bond geometry shown

$C_{57}H_{104}O_6$

1-[[[(9*Z*)-1-Oxo-9-octadecen-1-yl]oxy]methyl]-2-[(1-oxooctadecyl)oxy]ethyl (9*Z*,12*Z*)-9,12-octadecadienoate

71 References | 0 Reactions | 8 Suppliers

Key Physical Properties	Value	Condition
Molecular Weight	885.43	-
Melting Point (Experimental)	-19--18 °C	-
Boiling Point (Predicted)	818.7±55.0 °C	Press: 760 Torr
Density (Experimental)	0.9046 g/cm^3	Temp: 20 °C

Experimental Properties | Spectra

6

6915-08-8

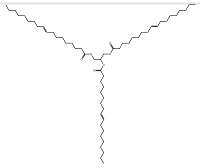

$C_{57}H_{104}O_6$

1,1',1''-(1,2,3-Propanetriyl) tri-9-octadec enoate

56 References | 47 Reactions | 1 Supplier

Key Physical Properties	Value	Condition
Molecular Weight	885.43	-
Boiling Point (Predicted)	818.7±55.0 °C	Press: 760 Torr
Density (Predicted)	0.921±0.06 g/cm^3	Temp: 20 °C; Press: 760 Torr

7

3296-43-3

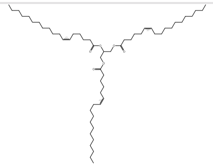

Double bond geometry shown

$C_{57}H_{104}O_6$

Tripetroselinin

42 References | 1 Reaction | 11 Suppliers

Key Physical Properties	Value	Condition
Molecular Weight	885.43	-
Melting Point (Experimental)	26.20001953125 °C	-
Boiling Point (Predicted)	818.7±55.0 °C	Press: 760 Torr
Density (Predicted)	0.921±0.06 g/cm^3	Temp: 20 °C; Press: 760 Torr

Experimental Properties | Spectra

8

83148-24-7

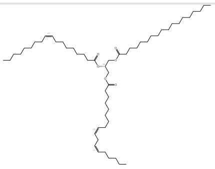

Absolute stereochemistry shown
Double bond geometry shown

$C_{57}H_{104}O_6$

Triglyceride StOL,sn

39 References | 1 Reaction | 2 Suppliers

Key Physical Properties	Value	Condition
Molecular Weight	885.43	-
Boiling Point (Predicted)	818.7±55.0 °C	Press: 760 Torr
Density (Predicted)	0.921±0.06 g/cm^3	Temp: 20 °C; Press: 760 Torr

9

122022-33-7

There are no Key Physical Properties to display for this substance.

463-40-1

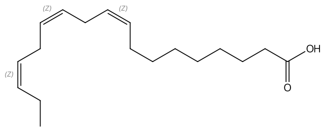

Double bond geometry shown

57-11-4

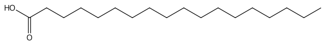

56-81-5

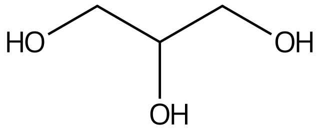

$C_{57}H_{104}O_6$

Triglyceride LnStSt

34 References | 0 Reactions | 0 Suppliers

10

79517-10-5

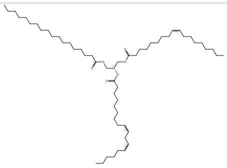

Absolute stereochemistry shown
Double bond geometry shown

Key Physical Properties	Value	Condition
Molecular Weight	885.43	-
Boiling Point (Predicted)	818.7±55.0 °C	Press: 760 Torr
Density (Predicted)	0.921±0.06 g/cm^3	Temp: 20 °C; Press: 760 Torr

$C_{57}H_{104}O_6$

(1*R*)-1-[[[(9*Z*)-1-Oxo-9-octadecen-1-yl]oxy]methyl]-2-[(1-oxooctadecyl)oxy]ethyl (9*Z*,12*Z*)-9,12-octadecadienoate

31 References | 0 Reactions | 0 Suppliers

Copyright © 2022 American Chemical Society (ACS). All Rights Reserved.
Internal use only. Redistribution is subject to the terms of your SciFinderⁿ License Agreement and CAS information Use Policies.
