# Supplementary material for: Efficient Green Extraction of Nutraceutical Compounds from Nannochloropsis gaditana: A Comparative Electrospray Ionization LC-MS and GC-MS Analysis for Lipid Profiling
Source: Foods. 2024 Dec 19;13(24):4117. doi: 10.3390/foods13244117 (PMC11675803; doi:10.3390/foods13244117)
Supplement: Supplementary file 1 [file foods-13-04117-s001.zip › MS Results/HPLC-MS PLE -Results-MC/Pico a 39.5 min_C57H106O6.pdf]

SciFinder^n

Task History

Initiating Search

November 25, 2022, 2:50PM

Substances:

Advanced Search:

Molecular Formula: $C_{57}H_{106}O_6$

Search Tasks

Task	Search Type	View
Exported: Returned Substance Results (136)	Substances	View Results

Copyright © 2022 American Chemical Society (ACS). All Rights Reserved.
Internal use only. Redistribution is subject to the terms of your SciFinder^n License Agreement and CAS information Use Policies.

CAS SciFinderⁿ

Substances (10)

View in SciFinderⁿ

1

29590-02-1

There are no Key Physical Properties to display for this substance.

Spectra

112-80-1

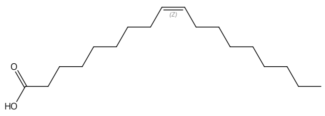

Double bond geometry shown

57-11-4

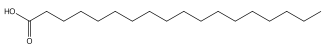

56-81-5

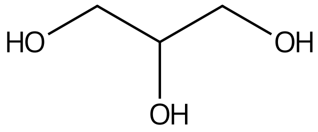

$C_{57}H_{106}O_6$

Triglyceride OOSt

654 References | 1 Reaction | 0 Suppliers

2

2410-28-8

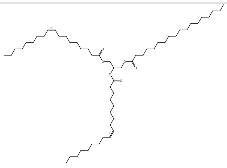

Double bond geometry shown

$C_{57}H_{106}O_6$

1,2-Dioleoyl-3-stearoylglycerol

244 References | 6 Reactions | 25 Suppliers

Key Physical Properties	Value	Condition
Molecular Weight	887.45	-
Melting Point (Experimental)	22.5-23.5 °C	-
Boiling Point (Predicted)	819.5±45.0 °C	Press: 760 Torr
Density (Predicted)	0.917±0.06 g/cm^3	Temp: 20 °C; Press: 760 Torr

Experimental Properties | Spectra

3

34521-51-2

60-33-3

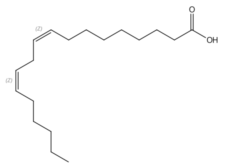

Double bond geometry shown

57-11-4

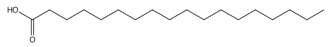

56-81-5

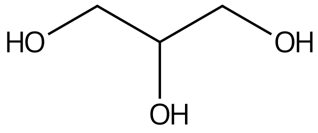

$C_{57}H_{106}O_6$

Triglyceride LStSt

164 References	0 Reactions	0 Suppliers

There are no Key Physical Properties to display for this substance.

Spectra

4

2410-29-9

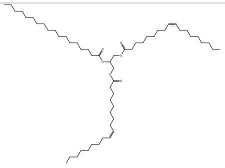

Double bond geometry shown

$C_{57}H_{106}O_6$

Triglyceride OStO,sn

142 References	9 Reactions	16 Suppliers

Key Physical Properties	Value	Condition
Molecular Weight	887.45	-
Melting Point (Experimental)	39.5-40.5 °C	-
Boiling Point (Predicted)	819.5±45.0 °C	Press: 760 Torr
Density (Predicted)	0.917±0.06 g/cm^3	Temp: 20 °C; Press: 760 Torr

Experimental Properties | Spectra

5

2190-13-8

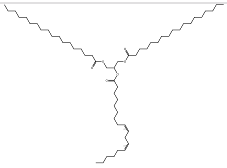

Double bond geometry shown

$C_{57}H_{106}O_6$

1,3-Distearoyl-2-linolein

122 References | 4 Reactions | 14 Suppliers

Key Physical Properties	Value	Condition
Molecular Weight	887.45	-
Melting Point (Experimental)	36 °C	-
Boiling Point (Predicted)	819.0±45.0 °C	Press: 760 Torr
Density (Predicted)	0.917±0.06 g/cm^3	Temp: 20 °C; Press: 760 Torr

Experimental Properties | Spectra

6

79517-07-0

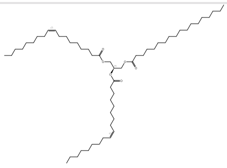

Absolute stereochemistry shown
Double bond geometry shown

$C_{57}H_{106}O_6$

Triglyceride StOO,sn

98 References | 1 Reaction | 1 Supplier

Key Physical Properties	Value	Condition
Molecular Weight	887.45	-
Melting Point (Experimental)	22.5 °C	-
Boiling Point (Predicted)	819.5±45.0 °C	Press: 760 Torr
Density (Predicted)	0.917±0.06 g/cm^3	Temp: 20 °C; Press: 760 Torr

Experimental Properties

7

2442-53-7

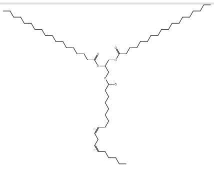

Double bond geometry shown

$C_{57}H_{106}O_6$

1-Linoleyl-2,3-distearin

53 References | 0 Reactions | 9 Suppliers

Key Physical Properties	Value	Condition
Molecular Weight	887.45	-
Melting Point (Experimental)	33.5-34 °C	-
Boiling Point (Predicted)	819.0±45.0 °C	Press: 760 Torr
Density (Predicted)	0.917±0.06 g/cm^3	Temp: 20 °C; Press: 760 Torr

Experimental Properties | Spectra

8

79517-06-9

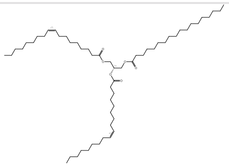

Absolute stereochemistry shown
Double bond geometry shown

$C_{57}H_{106}O_6$

Triglyceride OOSt,sn

48 References | 1 Reaction | 1 Supplier

Key Physical Properties	Value	Condition
Molecular Weight	887.45	-
Boiling Point (Predicted)	819.5±45.0 °C	Press: 760 Torr
Density (Predicted)	0.917±0.06 g/cm^3	Temp: 20 °C; Press: 760 Torr

9

82181-44-0

506-30-9

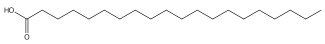

60-33-3

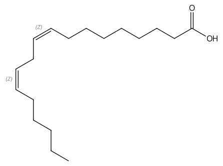

Double bond geometry shown

57-10-3

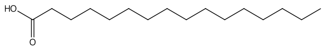

56-81-5

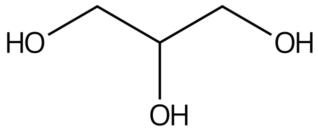

$C_{57}H_{106}O_6$

Triglyceride ALP

29 References | 0 Reactions | 0 Suppliers

There are no Key Physical Properties to display for this substance.

10

133997-79-2

29204-02-2

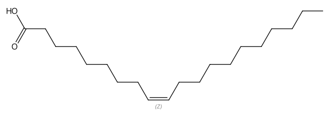

Double bond geometry shown

112-80-1

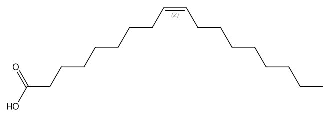

Double bond geometry shown

57-10-3

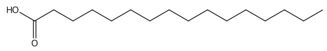

56-81-5

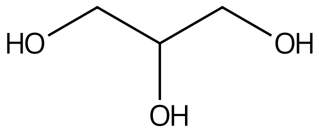

$C_{57}H_{106}O_6$

Triglyceride GaOP

17 References | 0 Reactions | 0 Suppliers

There are no Key Physical Properties to display for this substance.

Copyright © 2022 American Chemical Society (ACS). All Rights Reserved.
Internal use only. Redistribution is subject to the terms of your SciFinder^n License Agreement and CAS information Use Policies.
