# Supplementary material for: Efficient Green Extraction of Nutraceutical Compounds from Nannochloropsis gaditana: A Comparative Electrospray Ionization LC-MS and GC-MS Analysis for Lipid Profiling
Source: Foods. 2024 Dec 19;13(24):4117. doi: 10.3390/foods13244117 (PMC11675803; doi:10.3390/foods13244117)
Supplement: Supplementary file 1 [file foods-13-04117-s001.zip › MS Results/HPLC-MS PLE -Results-MC/Pico a 4.9 min_C30H49NO6.pdf]

CAS SciFinder^n

Task History

Initiating Search

November 25, 2022, 11:00AM

Substances:

Advanced Search:

Molecular Formula: C30H49NO6

Search Tasks

Task	Search Type	View
Exported: Returned Substance Results (110)	Substances	View Results

Copyright © 2022 American Chemical Society (ACS). All Rights Reserved.
Internal use only. Redistribution is subject to the terms of your SciFinder^n License Agreement and CAS information Use Policies.

CAS SciFinderⁿ

Substances (10)

View in SciFinderⁿ

1

111340-91-1

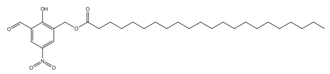

$C_{30}H_{49}NO_6$

(3-Formyl-2-hydroxy-5-nitrophenyl)methyl docosanoate

9 References | 4 Reactions | 2 Suppliers

Key Physical Properties	Value	Condition
Molecular Weight	519.71	-
Boiling Point (Predicted)	606.5±55.0 °C	Press: 760 Torr
Density (Predicted)	1.051±0.06 g/cm^3	Temp: 20 °C; Press: 760 Torr
pKa (Predicted)	4.31±0.38	Most Acidic Temp: 25 °C

2

185817-11-2

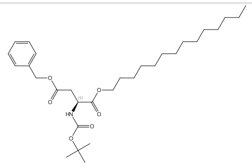

Absolute stereochemistry shown

$C_{30}H_{49}NO_6$

4-(Phenylmethyl) 1-tetradecyl *N*-[(1,1-dimethylethoxy)carbonyl]-L-aspartate

8 References | 15 Reactions | 0 Suppliers

Key Physical Properties	Value	Condition
Molecular Weight	519.71	-
Boiling Point (Predicted)	606.0±50.0 °C	Press: 760 Torr
Density (Predicted)	1.028±0.06 g/cm^3	Temp: 20 °C; Press: 760 Torr
pKa (Predicted)	10.61±0.46	Most Acidic Temp: 25 °C

3

89756-47-8

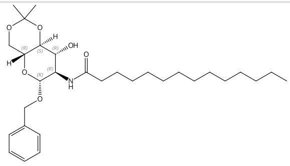

Absolute stereochemistry shown

$C_{30}H_{49}NO_6$

Phenylmethyl 2-deoxy-4,6-*O*-(1-methylet hylidene)-2-[(1-oxotetradecyl)amino]-β-D-glucopyranoside

7 References | 9 Reactions | 1 Supplier

Key Physical Properties	Value	Condition
Molecular Weight	519.71	-
Melting Point (Experimental)	117-119 °C	-
Boiling Point (Predicted)	669.3±55.0 °C	Press: 760 Torr
Density (Predicted)	1.09±0.1 g/cm^3	Temp: 20 °C; Press: 760 Torr
pKa (Predicted)	12.84±0.70	Most Acidic Temp: 25 °C

Experimental Properties | Spectra

4

65536-31-4

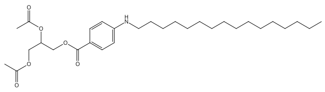

$C_{30}H_{49}NO_6$

2,3-Bis(acetyloxy)propyl 4-(hexadecylamino) benzoate

5 References | 5 Reactions | 2 Suppliers

Key Physical Properties	Value	Condition
Molecular Weight	519.71	-
Boiling Point (Predicted)	595.9±29.0 °C	Press: 760 Torr
Density (Predicted)	1.037±0.06 g/cm^3	Temp: 20 °C; Press: 760 Torr
pKa (Predicted)	2.13±0.50	Most Basic Temp: 25 °C

5

65536-24-5

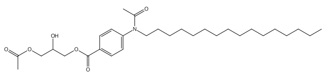

$C_{30}H_{49}NO_6$

3-(Acetyloxy)-2-hydroxypropyl 4-(acetylhexa decylamino)benzoate

5 References | 5 Reactions | 1 Supplier

Key Physical Properties	Value	Condition
Molecular Weight	519.71	-
Boiling Point (Predicted)	643.3±34.0 °C	Press: 760 Torr
Density (Predicted)	1.052±0.06 g/cm^3	Temp: 20 °C; Press: 760 Torr
pKa (Predicted)	12.83±0.20	Most Acidic Temp: 25 °C

6

91739-77-4

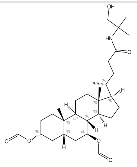

Absolute stereochemistry shown

$C_{30}H_{49}NO_6$

(3α,5β,7β)-3,7-Bis(formyloxy)-*N*-(2-hydroxy-1,1-dimethylethyl)cholan-24-amide

4 References | 9 Reactions | 1 Supplier

Key Physical Properties	Value	Condition
Molecular Weight	519.71	-
Boiling Point (Predicted)	642.6±48.0 °C	Press: 760 Torr
Density (Predicted)	1.13±0.1 g/cm^3	Temp: 20 °C; Press: 760 Torr
pKa (Predicted)	15.02±0.10	Most Acidic Temp: 25 °C

7

909406-77-5

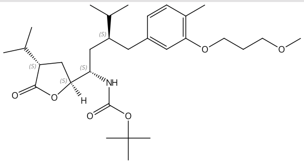

Absolute stereochemistry shown

$C_{30}H_{49}NO_6$

1,1-Dimethylethyl *N*-[(1*S*,3*S*)-3-[[3-(3-methoxypropoxy)-4-methylphenyl]methyl]-4-methyl-1-[(2*S*,4*S*)-tetrahydro-4-(1-methylethyl)-5-oxo-2-furanyl]pentyl]carbamate

3 References | 18 Reactions | 0 Suppliers

Key Physical Properties	Value	Condition
Molecular Weight	519.71	-
Boiling Point (Predicted)	640.2±50.0 °C	Press: 760 Torr
Density (Predicted)	1.041±0.06 g/cm^3	Temp: 20 °C; Press: 760 Torr
pKa (Predicted)	11.81±0.46	Most Acidic Temp: 25 °C

8

177260-25-2

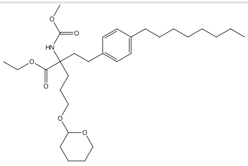

$C_{30}H_{49}NO_6$

Ethyl α-[(methoxycarbonyl)amino]-4-octyl-α-[3-[(tetrahydro-2*H*-pyran-2-yl)oxy]propyl]benzenebutanoate

3 References | 8 Reactions | 1 Supplier

Key Physical Properties	Value	Condition
Molecular Weight	519.71	-
Boiling Point (Predicted)	629.2±55.0 °C	Press: 760 Torr
Density (Predicted)	1.06±0.1 g/cm^3	Temp: 20 °C; Press: 760 Torr
pKa (Predicted)	10.35±0.46	Most Acidic Temp: 25 °C

9

150456-73-8

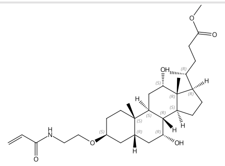

Absolute stereochemistry shown

$C_{30}H_{49}NO_6$

Cholan-24-oic acid, 7,12-dihydroxy-3-[2-[(1-oxo-2-propenyl)amino]ethoxy]-, methyl ester, (3β,5β,7α,12α)-

3 References | 3 Reactions | 0 Suppliers

Key Physical Properties	Value	Condition
Molecular Weight	519.71	-
Boiling Point (Predicted)	660.3±55.0 °C	Press: 760 Torr
Density (Predicted)	1.14±0.1 g/cm^3	Temp: 20 °C; Press: 760 Torr
pKa (Predicted)	14.33±0.46	Most Acidic Temp: 25 °C

10

52717-90-5

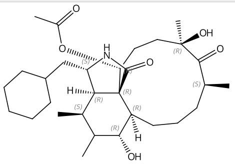

Absolute stereochemistry shown

$C_{30}H_{49}NO_6$

[11]Cytochalasan-1,17-dione, 21-(acetyloxy)-10-cyclohexyl-7,18-dihydroxy-16,18-dimethyl-, (6ξ,7*R*,16*S*,18*R*,21*R*)-

3 References | 0 Reactions | 0 Suppliers

Key Physical Properties	Value	Condition
Molecular Weight	519.71	-
Boiling Point (Predicted)	685.7±55.0 °C	Press: 760 Torr
Density (Predicted)	1.15±0.1 g/cm^3	Temp: 20 °C; Press: 760 Torr
pKa (Predicted)	12.86±0.70	Most Acidic Temp: 25 °C

Copyright © 2022 American Chemical Society (ACS). All Rights Reserved.
Internal use only. Redistribution is subject to the terms of your SciFinderⁿ License Agreement and CAS information Use Policies.
