# Supplementary material for: Efficient Green Extraction of Nutraceutical Compounds from Nannochloropsis gaditana: A Comparative Electrospray Ionization LC-MS and GC-MS Analysis for Lipid Profiling
Source: Foods. 2024 Dec 19;13(24):4117. doi: 10.3390/foods13244117 (PMC11675803; doi:10.3390/foods13244117)
Supplement: Supplementary file 1 [file foods-13-04117-s001.zip › MS Results/HPLC-MS PLE MC.pdf]

Equipo MAXIS II

Nombre muestra	LC2167
Nombre registro	C:\Data\Ficheros Datos MAXIS\HPLC\noviembre 2022\LC2167_21_01_4548.d
Metodo	ESI Positive hplc1100_ 50 min_50-3000 t=200 6l-min.m
Comentarios	PLE MC 0.1 mg/ml en EtOH

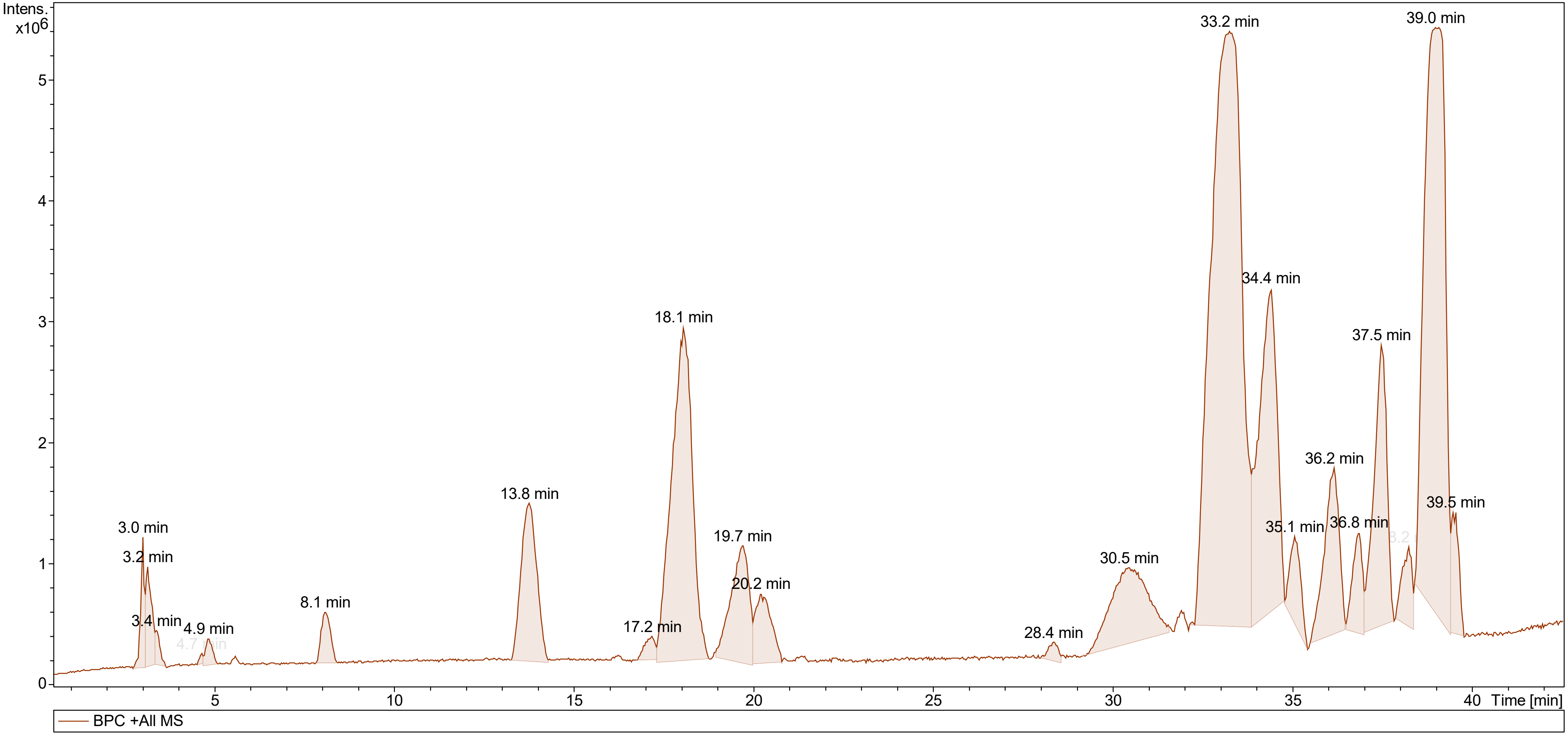

Analysis Info

Analysis Name	C:\Data\Ficheros Datos MAXIS\HPLC\noviembre 2022\LC2167_21_01_4548.d
Method	hplc1100_ 50 min_50-3000 t=200 6l-min.m
Sample Name	LC2167
Instrument	maXis II
Comment	PLE MC 0.1 mg/ml en EtOH

Acquisition Parameter

Source Type	ESI	Ion Polarity	Positive	Set Nebulizer	3.0 Bar
Focus	Active	Set Capillary	3500 V	Set Dry Heater	250 °C
Scan Begin	50 m/z	Set End Plate Offset	-500 V	Set Dry Gas	6.0 l/min
Scan End	3000 m/z	Set Charging Voltage	2000 V	Set Divert Valve	Source
		Set Corona	0 nA	Set APCI Heater	0 °C

#	RT [min]	Area	Int. Type	I	S/N	Chromatogram	Max. m/z	FWHM [min]
1	3.0	8128229	Manual	1223995	43.6	BPC +All MS	205.0682	0.1
2	3.2	10331193	Manual	980110	33.4	BPC +All MS	205.0680	0.2
3	3.4	2925820	Manual	450811	11.5	BPC +All MS	197.1171	0.1
4	4.7	603108	Manual	261049	3.5	BPC +All MS	494.3269	0.1
5	4.9	3100737	Manual	383660	8.9	BPC +All MS	520.3640	0.2
6	8.1	7773097	Manual	604796	17.0	BPC +All MS	282.2791	0.3
7	13.8	40099004	Manual	1500669	52.6	BPC +All MS	338.3415	0.5
8	17.2	4155074	Manual	399710	7.8	BPC +All MS	730.5373	0.4
9	18.1	111153448	Manual	2959632	111.1	BPC +All MS	804.5773	0.6
10	19.7	34402828	Manual	1153648	39.2	BPC +All MS	806.5927	0.6
11	20.2	18296114	Manual	757580	23.4	BPC +All MS	758.5687	0.6
12	28.4	3103986	Manual	355255	6.4	BPC +All MS	600.5194	0.3
13	30.5	46073780	Manual	969180	25.2	BPC +All MS	548.5028	1.2
14	33.2	296420640	Manual	5406768	198.3	BPC +All MS	871.5726	1.0
15	34.4	96701896	Manual	3282710	107.7	BPC +All MS	871.5719	0.6
16	35.1	15116911	Manual	1227665	28.9	BPC +All MS	918.8105	0.4
17	36.2	39393868	Manual	1793838	55.3	BPC +All MS	813.5666	0.4
18	36.8	16078802	Manual	1264700	33.8	BPC +All MS	818.7220	0.3
19	37.5	58457652	Manual	2815415	93.7	BPC +All MS	820.7375	0.4
20	38.2	12993419	Manual	1141230	26.6	BPC +All MS	898.7845	0.4
21	39.0	199031376	Manual	5436637	194.9	BPC +All MS	902.8166	0.7
22	39.5	15176457	Manual	1430704	40.6	BPC +All MS	904.8311	0.2

Compound Spectrum List Report

Analysis Info

Analysis Name	C:\Data\Ficheros Datos MAXIS\HPLC\noviembre 2022\LC2167_21_01_4548.d	Acquisition Date	21/11/2022 16:56:00
Method	hplc1100_ 50 min_50-3000 t=200 6l-min.m	Operator	AMP
Sample Name	LC2167	Instrument	maXis II 1828979.22347
Comment	PLE MC 0.1 mg/ml en EtOH		

Acquisition Parameter

Source Type	ESI	Ion Polarity	Positive	Set Nebulizer	3.0 Bar
Focus	Active	Set Capillary	3500 V	Set Dry Heater	250 °C
Scan Begin	50 m/z	Set End Plate Offset	-500 V	Set Dry Gas	6.0 l/min
Scan End	3000 m/z	Set Charging Voltage	2000 V	Set Divert Valve	Source
		Set Corona	0 nA	Set APCI Heater	0 °C

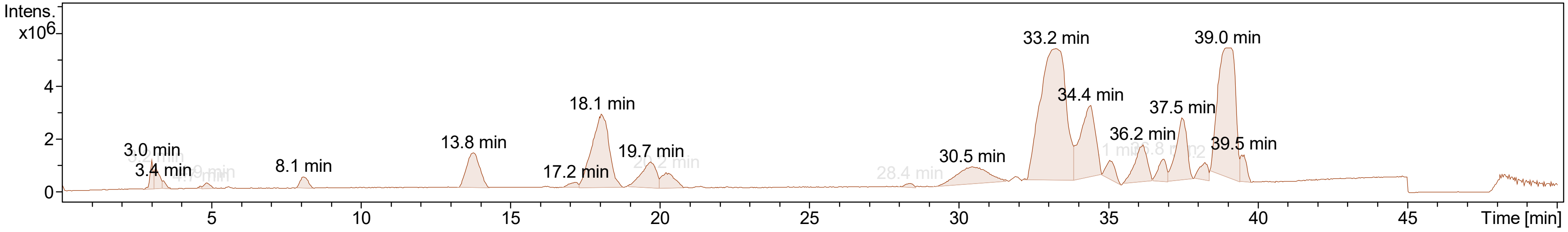

#	RT [min]	Area	Int. Type	I	S/N	Chromatogram	Max. m/z	FWHM [min]
1	3.0	8128229	Manual	1223995	43.6	BPC +All MS	205.0682	0.1
2	3.2	10331193	Manual	980110	33.4	BPC +All MS	205.0680	0.2
3	3.4	2925820	Manual	450811	11.5	BPC +All MS	197.1171	0.1
4	4.7	603108	Manual	261049	3.5	BPC +All MS	494.3269	0.1
5	4.9	3100737	Manual	383660	8.9	BPC +All MS	520.3640	0.2
6	8.1	7773097	Manual	604796	17.0	BPC +All MS	282.2791	0.3
7	13.8	40099004	Manual	1500669	52.6	BPC +All MS	338.3415	0.5
8	17.2	4155074	Manual	399710	7.8	BPC +All MS	730.5373	0.4
9	18.1	111153448	Manual	2959632	111.1	BPC +All MS	804.5773	0.6
10	19.7	34402828	Manual	1153648	39.2	BPC +All MS	806.5927	0.6
11	20.2	18296114	Manual	757580	23.4	BPC +All MS	758.5687	0.6
12	28.4	3103986	Manual	355255	6.4	BPC +All MS	600.5194	0.3
13	30.5	46073780	Manual	969180	25.2	BPC +All MS	548.5028	1.2
14	33.2	296420640	Manual	5406768	198.3	BPC +All MS	871.5726	1.0
15	34.4	96701896	Manual	3282710	107.7	BPC +All MS	871.5719	0.6
16	35.1	15116911	Manual	1227665	28.9	BPC +All MS	918.8105	0.4

#	RT [min]	Area	Int. Type	I	S/N	Chromatogram	Max. m/z	FWHM [min]
17	36.2	39393868	Manual	1793838	55.3	BPC +All MS	813.5666	0.4
18	36.8	16078802	Manual	1264700	33.8	BPC +All MS	818.7220	0.3
19	37.5	58457652	Manual	2815415	93.7	BPC +All MS	820.7375	0.4
20	38.2	12993419	Manual	1141230	26.6	BPC +All MS	898.7845	0.4
21	39.0	199031376	Manual	5436637	194.9	BPC +All MS	902.8166	0.7
22	39.5	15176457	Manual	1430704	40.6	BPC +All MS	904.8311	0.2

Cmpd 1, 3.0 min

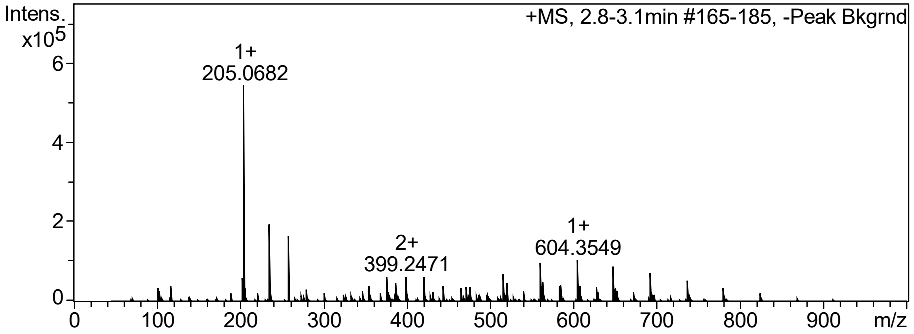

#	m/z	Res.	S/N	I	I %	FWHM
1	205.0682	41724	11943.2	543182	100.0	0.0049
2	236.1494	40523	3747.5	193998	35.7	0.0058
3	258.1314	40491	2948.6	166826	30.7	0.0064
4	377.2336	44079	700.8	62599	11.5	0.0086
5	399.2471	43021	660.1	63191	11.6	0.0093
6	516.3026	42209	544.2	67662	12.5	0.0122
7	560.3286	47771	731.2	96999	17.9	0.0117
8	604.3549	48569	757.8	105362	19.4	0.0124
9	648.3811	45226	620.6	89125	16.4	0.0143
10	692.4076	47064	498.2	72792	13.4	0.0147

Cmpd 2, 3.2 min

Compound Spectrum List Report

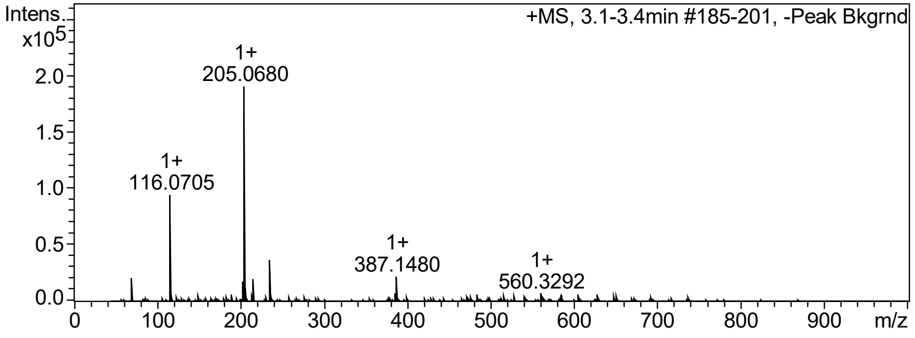

#	m/z	Res.	S/N	I	I %	FWHM
1	70.0646	21464	1153.6	20800	10.9	0.0033
2	116.0705	28721	4838.8	94312	49.5	0.0040
3	203.0524	40010	694.2	17359	9.1	0.0051
4	205.0680	43033	7544.7	190465	100.0	0.0048
5	206.0721	33955	459.7	11662	6.1	0.0061
6	215.0701	33355	759.6	20118	10.6	0.0064
7	236.1492	36793	1243.6	36292	19.1	0.0064
8	385.1321	42396	125.0	7246	3.8	0.0091
9	387.1480	41667	385.6	22553	11.8	0.0093
10	560.3292	37663	72.1	7037	3.7	0.0149

Cmpd 3, 3.4 min

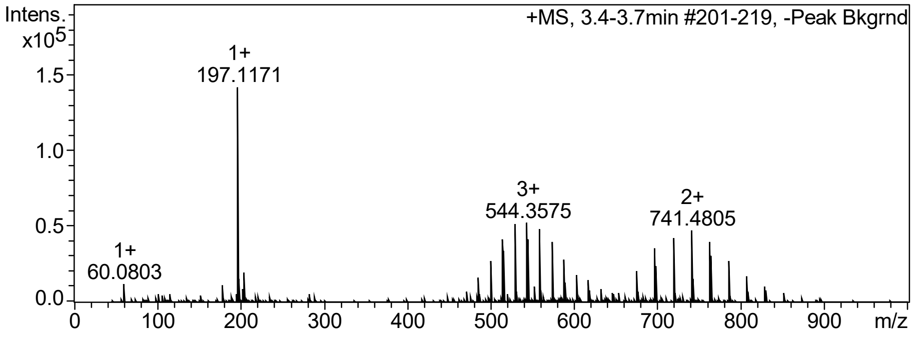

#	m/z	Res.	S/N	I	I %	FWHM
1	197.1171	38256	4388.1	141790	100.0	0.0052

#	m/z	Res.	S/N	I	I %	FWHM
2	515.0071	47061	402.3	41873	29.5	0.0109
3	529.6821	46207	479.1	51377	36.2	0.0115
4	544.3575	47400	475.2	52202	36.8	0.0115
5	544.6919	45427	375.4	41261	29.1	0.0120
6	559.0332	45821	430.0	48407	34.1	0.0122
7	559.3674	50933	397.7	44789	31.6	0.0110
8	719.4672	55188	328.6	42394	29.9	0.0130
9	741.4805	53320	366.2	47431	33.5	0.0139
10	763.4936	57252	311.7	40342	28.5	0.0133

Cmpd 4, 4.7 min

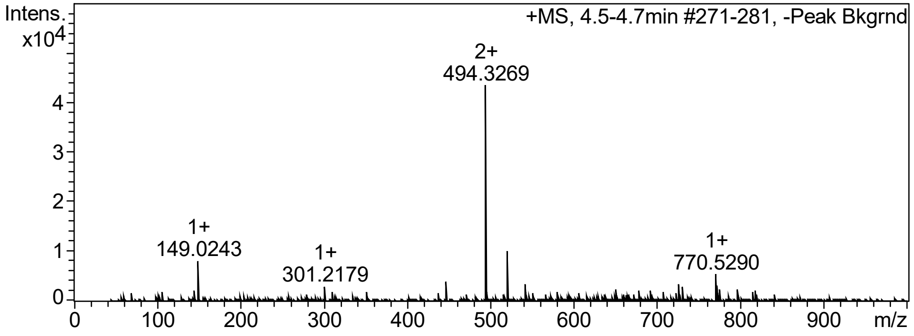

#	m/z	Res.	S/N	I	I %	FWHM
1	149.0243	56289	234.3	8095	18.7	0.0026
2	301.2179	46703	58.1	2973	6.9	0.0064
3	446.3505	44623	53.2	4012	9.2	0.0100
4	494.3269	52958	512.0	43398	100.0	0.0093
5	495.3291	40055	132.2	11234	25.9	0.0124
6	520.3673	45285	112.0	10062	23.2	0.0115
7	542.3285	63320	36.9	3481	8.0	0.0086
8	726.5022	34347	28.6	3449	7.9	0.0212
9	770.5290	47850	44.5	5488	12.6	0.0161
10	771.5310	40716	25.9	3203	7.4	0.0189

Cmpd 5, 4.9 min

Compound Spectrum List Report

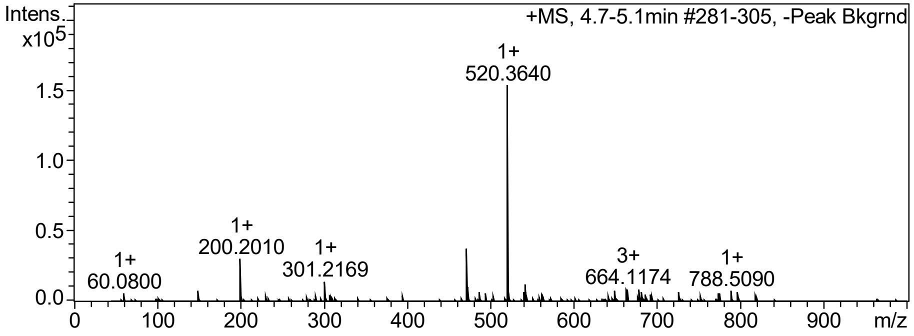

#	m/z	Res.	S/N	I	I %	FWHM
1	200.2010	33824	1121.5	30027	19.6	0.0059
2	301.2169	37261	358.1	14175	9.2	0.0081
3	472.3644	40084	494.7	37183	24.3	0.0118
4	473.3675	38478	137.1	10333	6.7	0.0123
5	520.3640	49528	1783.5	153327	100.0	0.0105
6	521.3677	43999	586.1	50500	32.9	0.0118
7	542.3465	38327	137.8	12499	8.2	0.0142
8	663.7827	44639	72.3	8170	5.3	0.0149
9	664.1174	44369	78.5	8871	5.8	0.0150
10	678.4577	48459	73.5	8455	5.5	0.0140

Cmpd 6, 8.1 min

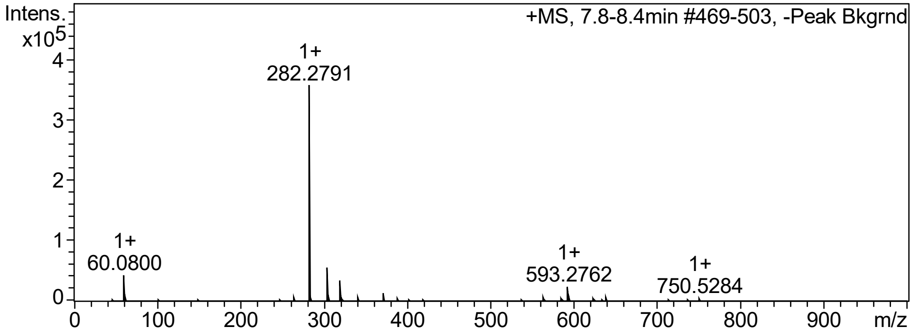

#	m/z	Res.	S/N	I	I %	FWHM
1	60.0800	21319	2383.5	44030	12.3	0.0028

#	m/z	Res.	S/N	I	I %	FWHM
2	62.0594	20306	461.6	8584	2.4	0.0031
3	282.2791	43536	10026.9	357456	100.0	0.0065
4	283.2825	38731	2052.8	73445	20.5	0.0073
5	304.2612	39118	1442.5	55246	15.5	0.0078
6	305.2646	38240	271.0	10414	2.9	0.0080
7	320.2354	38365	841.2	33972	9.5	0.0083
8	371.3162	39573	295.6	14047	3.9	0.0094
9	593.2762	37543	289.3	24382	6.8	0.0158
10	594.2796	38190	117.9	9956	2.8	0.0156

Cmpd 7, 13.8 min

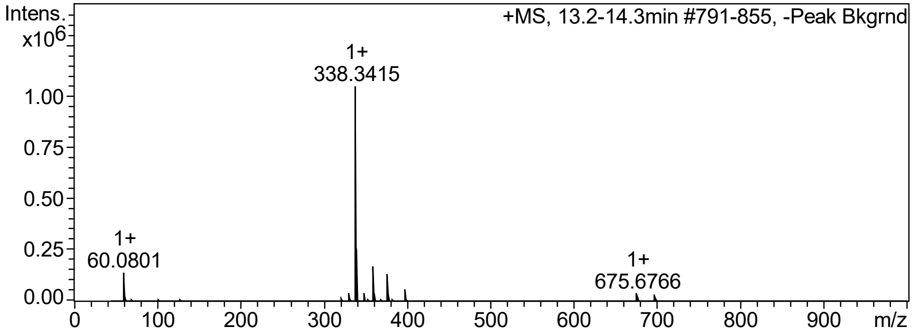

#	m/z	Res.	S/N	I	I %	FWHM
1	60.0801	22275	7852.2	143028	13.6	0.0027
2	331.2633	39782	1044.9	39710	3.8	0.0083
3	338.3415	49630	27017.6	1050751	100.0	0.0068
4	339.3452	41091	6579.1	256628	24.4	0.0083
5	348.2900	40043	981.9	39435	3.8	0.0087
6	360.3239	42403	4079.7	169818	16.2	0.0085
7	361.3274	37200	925.3	38646	3.7	0.0097
8	376.2979	42029	3072.3	134561	12.8	0.0090
9	397.4157	40131	1274.1	59662	5.7	0.0099
10	675.6766	39585	458.6	40240	3.8	0.0171

Cmpd 8, 17.2 min

Compound Spectrum List Report

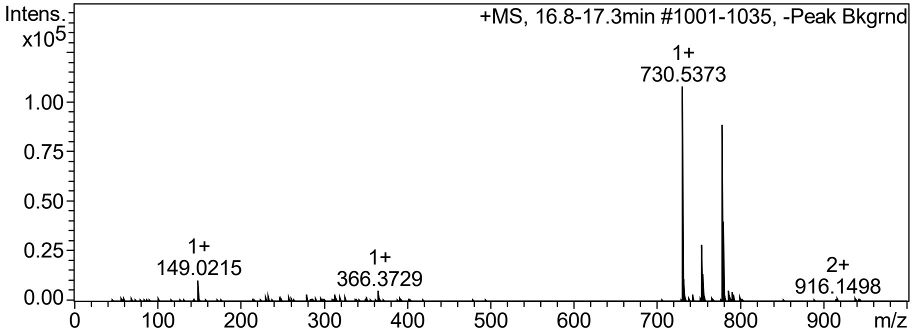

#	m/z	Res.	S/N	I	I %	FWHM
1	149.0215	43717	429.6	10803	10.0	0.0034
2	366.3729	33373	120.8	5398	5.0	0.0110
3	730.5373	59108	1109.1	107655	100.0	0.0124
4	731.5408	48099	450.0	43743	40.6	0.0152
5	732.5438	38728	111.8	10883	10.1	0.0189
6	754.5370	46494	287.2	28703	26.7	0.0162
7	755.5409	40597	140.4	14045	13.0	0.0186
8	778.5374	57266	866.3	88846	82.5	0.0136
9	779.5408	48187	392.1	40241	37.4	0.0162
10	780.5432	42297	114.4	11749	10.9	0.0185

Cmpd 9, 18.1 min

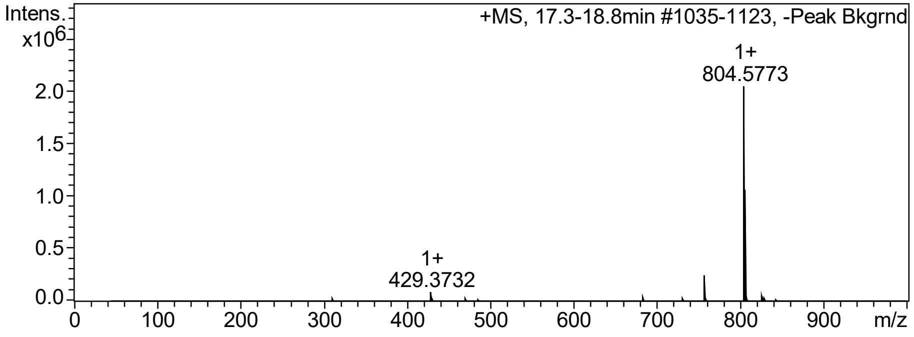

#	m/z	Res.	S/N	I	I %	FWHM
1	429.3732	41497	1714.7	90689	4.4	0.0103

#	m/z	Res.	S/N	I	I %	FWHM
2	682.5619	40890	440.3	43548	2.1	0.0167
3	730.5635	57424	309.5	32953	1.6	0.0127
4	756.5552	53238	2249.4	247052	12.0	0.0142
5	757.5588	44699	1032.2	113491	5.5	0.0169
6	804.5773	72601	17860.9	2052430	100.0	0.0111
7	805.5807	66931	9206.7	1058267	51.6	0.0120
8	806.5831	54434	2218.1	255025	12.4	0.0148
9	807.5844	51308	413.5	47571	2.3	0.0157
10	826.5595	39386	435.5	50693	2.5	0.0210

Cmpd 10, 19.7 min

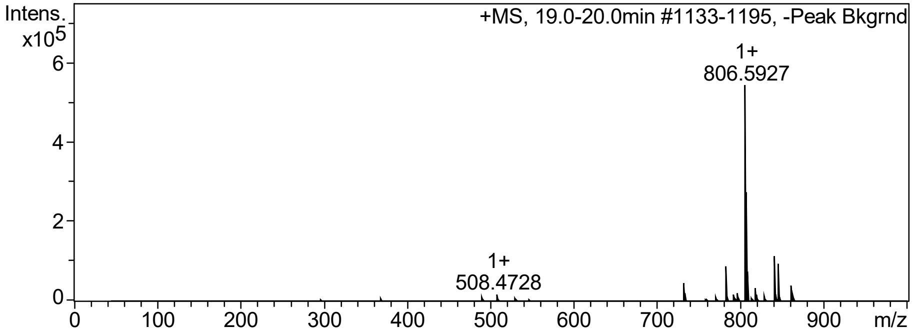

#	m/z	Res.	S/N	I	I %	FWHM
1	732.5552	40284	484.1	47132	8.7	0.0182
2	782.5712	45986	859.7	88249	16.3	0.0170
3	783.5747	37829	420.2	43173	8.0	0.0207
4	806.5927	67089	5176.9	542617	100.0	0.0120
5	807.5960	58046	2623.8	275226	50.7	0.0139
6	808.5994	47372	714.0	74951	13.8	0.0171
7	840.5625	57380	1063.7	114181	21.0	0.0146
8	841.5655	50447	531.9	57126	10.5	0.0167
9	845.5175	56845	882.4	95000	17.5	0.0149
10	846.5214	48236	463.1	49889	9.2	0.0175

Cmpd 11, 20.2 min

Compound Spectrum List Report

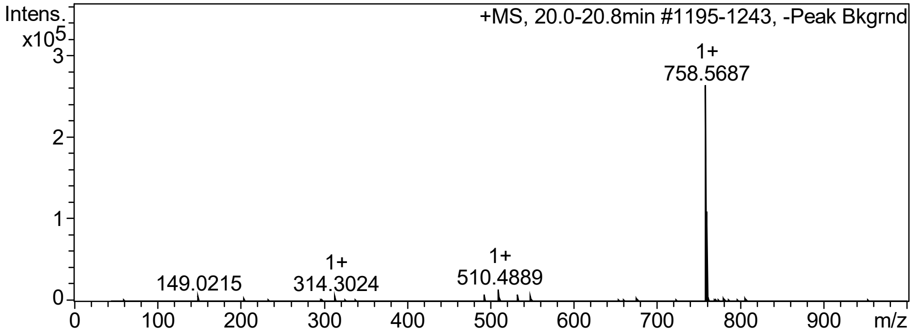

#	m/z	Res.	S/N	I	I %	FWHM
1	149.0215	53325	296.3	7119	2.7	0.0028
2	314.3024	79772	184.1	6748	2.6	0.0039
3	492.4779	39083	134.7	8230	3.1	0.0126
4	510.4889	42023	226.5	14468	5.5	0.0121
5	532.4702	34696	125.0	8402	3.2	0.0153
6	548.4455	39675	95.7	6676	2.5	0.0138
7	758.5687	63862	2623.8	262913	100.0	0.0119
8	759.5722	53972	1093.8	109722	41.7	0.0141
9	760.5754	42911	298.5	29980	11.4	0.0177
10	761.5791	38789	53.0	5330	2.0	0.0196

Cmpd 12, 28.4 min

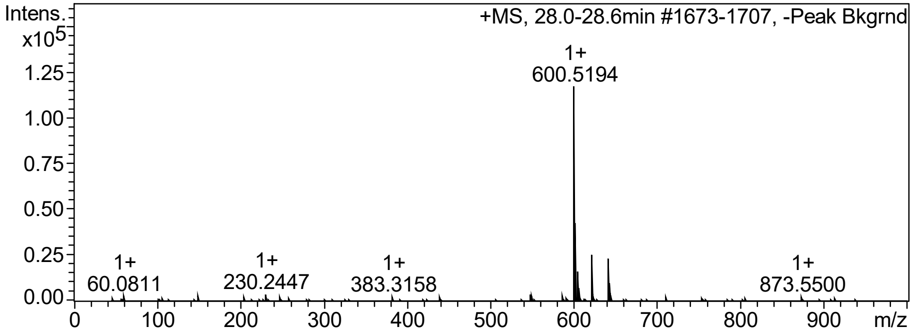

#	m/z	Res.	S/N	I	I %	FWHM
1	548.4982	86117	55.0	4016	3.4	0.0064

#	m/z	Res.	S/N	I	I %	FWHM
2	600.5194	55914	1471.5	117127	100.0	0.0107
3	601.5225	44019	539.0	42953	36.7	0.0137
4	602.5263	40761	121.6	9715	8.3	0.0148
5	605.4752	36304	205.2	16460	14.1	0.0167
6	606.4781	39023	94.4	7585	6.5	0.0155
7	621.4486	44569	305.5	25080	21.4	0.0139
8	622.4528	35240	101.2	8322	7.1	0.0177
9	642.5659	40390	275.6	23265	19.9	0.0159
10	643.5696	37296	117.2	9902	8.5	0.0173

Cmpd 13, 30.5 min

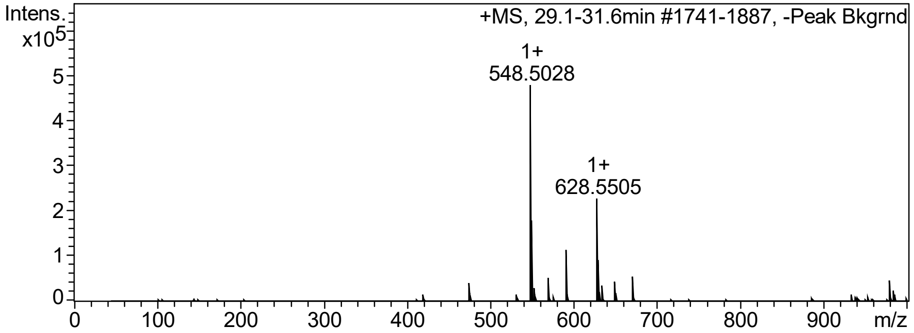

#	m/z	Res.	S/N	I	I %	FWHM
1	548.5028	58182	6938.8	477777	100.0	0.0094
2	549.5064	48663	2617.7	180526	37.8	0.0113
3	569.4326	41957	718.5	51303	10.7	0.0136
4	590.5498	49908	1529.3	113049	23.7	0.0118
5	591.5532	43550	647.8	47968	10.0	0.0136
6	628.5505	47926	2883.0	226008	47.3	0.0131
7	629.5539	42444	1177.1	92400	19.3	0.0148
8	649.4800	39093	545.5	44049	9.2	0.0166
9	670.5975	39853	649.4	53754	11.3	0.0168
10	978.8325	39452	475.0	46145	9.7	0.0248

Cmpd 14, 33.2 min

Compound Spectrum List Report

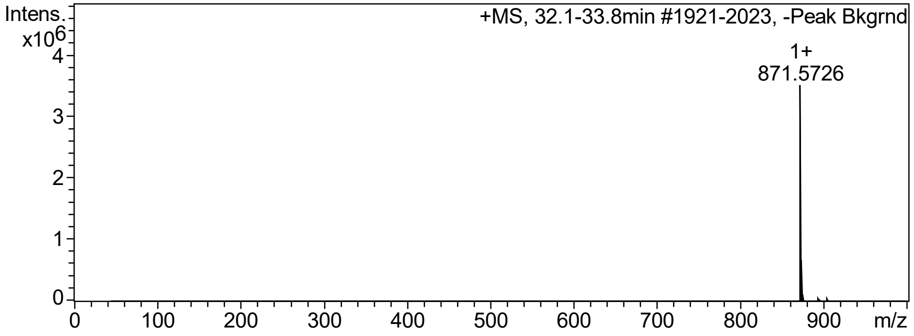

#	m/z	Res.	S/N	I	I %	FWHM
1	871.5726	51233	37973.5	3496555	100.0	0.0170
2	871.8627	24327	434.3	39994	1.1	0.0358
3	872.5755	77973	25434.9	2342678	67.0	0.0112
4	873.5788	66920	7189.3	662427	18.9	0.0131
5	874.5820	44557	1335.8	123082	3.5	0.0196
6	893.5545	41463	516.7	48025	1.4	0.0216
7	903.5619	58109	612.5	57252	1.6	0.0155
8	1742.1365	37501	1392.6	84764	2.4	0.0465
9	1743.1398	37390	1655.4	100741	2.9	0.0466
10	1744.1430	35797	1055.5	64228	1.8	0.0487

Cmpd 15, 34.4 min

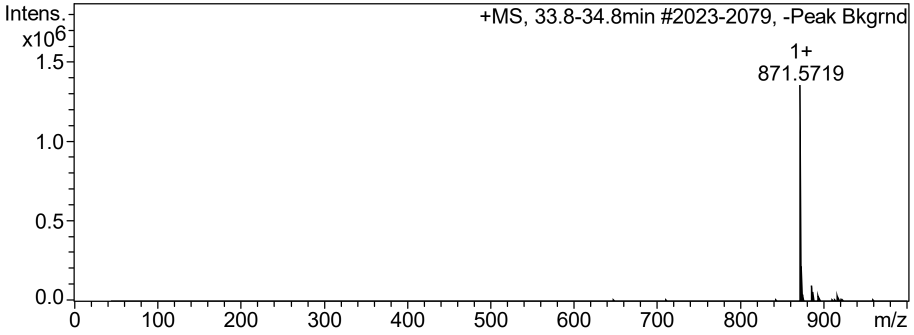

#	m/z	Res.	S/N	I	I %	FWHM
1	871.5719	80783	15407.3	1350644	100.0	0.0108

#	m/z	Res.	S/N	I	I %	FWHM
2	871.6012	51061	1073.1	94074	7.0	0.0171
3	872.5752	76432	8976.3	787022	58.3	0.0114
4	873.5785	61791	2480.2	217474	16.1	0.0141
5	874.5818	40349	474.5	41615	3.1	0.0217
6	885.5869	69615	1164.3	102695	7.6	0.0127
7	886.5907	60520	684.0	60346	4.5	0.0146
8	893.5542	41174	379.6	33551	2.5	0.0217
9	916.7959	46037	385.9	34252	2.5	0.0199
10	1743.1401	31661	458.8	24062	1.8	0.0551

Cmpd 16, 35.1 min

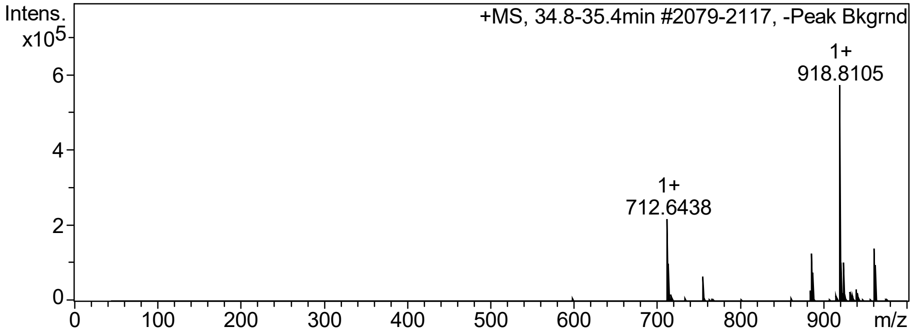

#	m/z	Res.	S/N	I	I %	FWHM
1	712.6438	55107	2569.3	216556	37.9	0.0129
2	713.6472	46140	1164.8	98300	17.2	0.0155
3	885.5877	54638	1349.9	126554	22.1	0.0162
4	886.5910	47670	809.0	75860	13.3	0.0186
5	918.8105	69926	6085.4	571736	100.0	0.0131
6	919.8141	63610	3756.4	352848	61.7	0.0145
7	920.8165	53433	1097.6	103080	18.0	0.0172
8	923.7662	55160	1111.6	104383	18.3	0.0167
9	960.8574	53361	1511.5	141310	24.7	0.0180
10	961.8606	48073	1012.0	94586	16.5	0.0200

Cmpd 17, 36.2 min

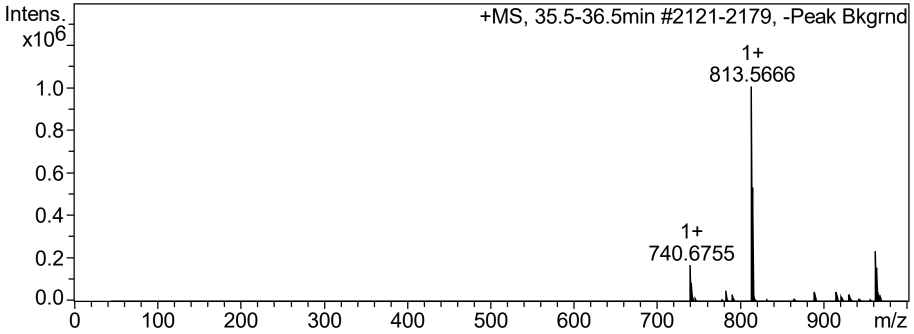

#	m/z	Res.	S/N	I	I %	FWHM
1	740.6755	52375	2001.9	169145	16.9	0.0141
2	741.6786	44495	1004.4	84960	8.5	0.0167
3	782.7229	40872	600.2	52889	5.3	0.0192
4	813.5666	73270	11155.1	1003636	100.0	0.0111
5	814.5699	65089	5959.4	536200	53.4	0.0125
6	815.5731	49900	1628.2	146519	14.6	0.0163
7	962.7222	39872	684.7	62232	6.2	0.0241
8	962.8368	61268	2614.1	237597	23.7	0.0157
9	963.7256	40961	498.4	45289	4.5	0.0235
10	963.8402	58066	1717.5	156065	15.5	0.0166

Cmpd 18, 36.8 min

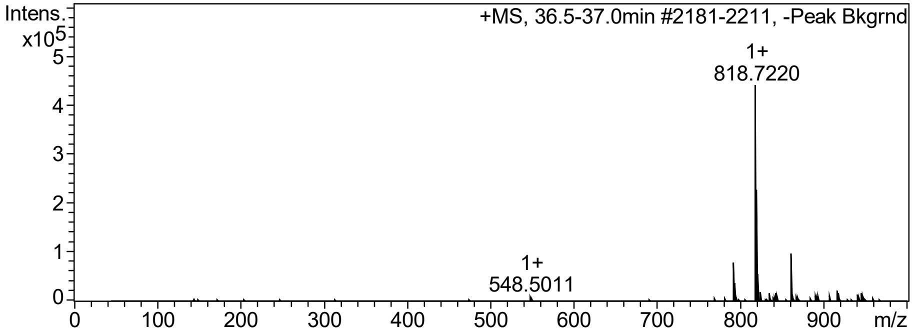

#	m/z	Res.	S/N	I	I %	FWHM
1	792.7055	61818	926.2	78248	17.8	0.0128

#	m/z	Res.	S/N	I	I %	FWHM
2	793.7089	43711	433.5	36649	8.3	0.0182
3	818.7220	69993	5136.9	439788	100.0	0.0117
4	819.7255	63794	2660.5	227797	51.8	0.0128
5	820.7237	77156	666.4	57075	13.0	0.0106
6	823.6774	41754	232.1	19898	4.5	0.0197
7	834.7526	45751	206.2	17752	4.0	0.0182
8	860.7690	55668	1138.4	98431	22.4	0.0155
9	861.7726	45291	624.2	53981	12.3	0.0190
10	916.7397	56493	260.5	22530	5.1	0.0162

Cmpd 19, 37.5 min

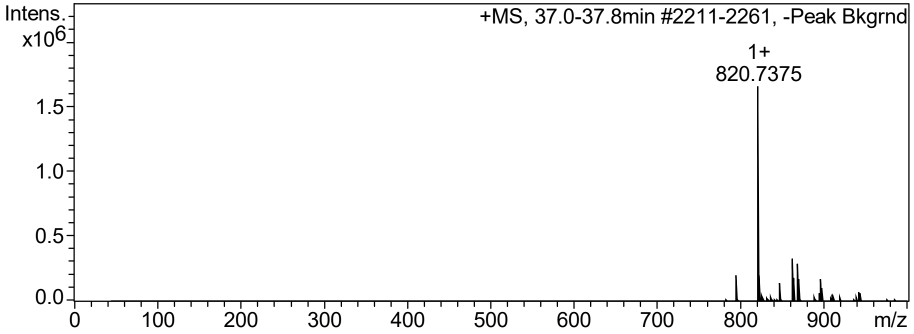

#	m/z	Res.	S/N	I	I %	FWHM
1	794.7220	55370	2356.5	199146	12.0	0.0144
2	820.7375	75924	19365.4	1660444	100.0	0.0108
3	821.7410	71085	10639.4	912159	54.9	0.0116
4	822.7410	73372	2336.8	200377	12.1	0.0112
5	846.7532	54973	1696.9	146533	8.8	0.0154
6	862.7849	64016	3765.6	325241	19.6	0.0135
7	863.7880	55948	2142.5	185027	11.1	0.0154
8	868.7374	59139	3315.4	286362	17.2	0.0147
9	869.7408	54091	1981.4	171128	10.3	0.0161
10	896.7685	57071	1960.7	168406	10.1	0.0157

Cmpd 20, 38.2 min

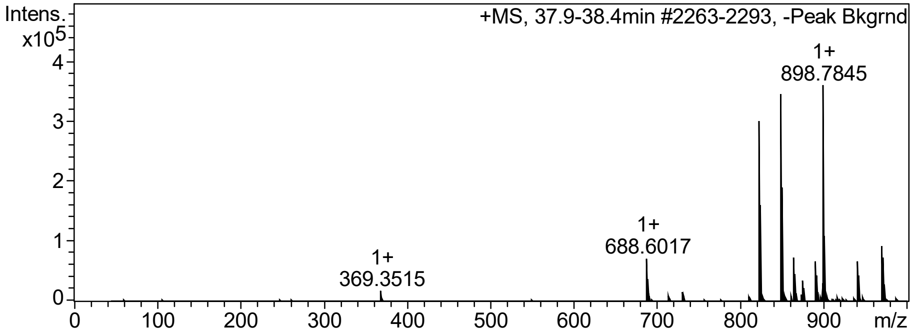

#	m/z	Res.	S/N	I	I %	FWHM
1	822.7535	68047	3901.9	299671	83.5	0.0121
2	823.7567	58418	2074.6	159359	44.4	0.0141
3	848.7688	73805	4484.7	345338	96.2	0.0115
4	849.7724	65355	2468.3	190058	52.9	0.0130
5	864.8005	54272	945.5	72778	20.3	0.0159
6	898.7845	70172	4707.0	359064	100.0	0.0128
7	899.7880	63935	2829.6	215778	60.1	0.0141
8	900.7915	69911	1440.7	109817	30.6	0.0129
9	970.7867	62758	1254.4	92369	25.7	0.0155
10	971.7885	55326	997.3	73385	20.4	0.0176

Cmpd 21, 39.0 min

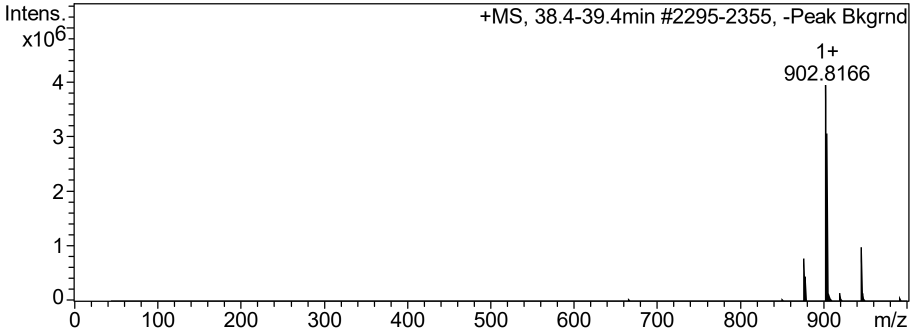

#	m/z	Res.	S/N	I	I %	FWHM
1	876.8010	73439	8414.9	771770	19.6	0.0119

#	m/z	Res.	S/N	I	I %	FWHM
2	877.8043	65713	4898.1	449438	11.4	0.0134
3	902.8166	39749	42757.4	3937857	100.0	0.0227
4	903.8192	70728	33331.1	3068050	77.9	0.0128
5	904.8208	84253	9653.3	888092	22.6	0.0107
6	905.8208	77837	1494.8	137454	3.5	0.0116
7	918.8479	52214	1603.3	146888	3.7	0.0176
8	944.8629	71030	10847.3	979108	24.9	0.0133
9	945.8663	64036	6808.6	614168	15.6	0.0148
10	946.8648	60015	1804.5	162673	4.1	0.0158

Cmpd 22, 39.5 min

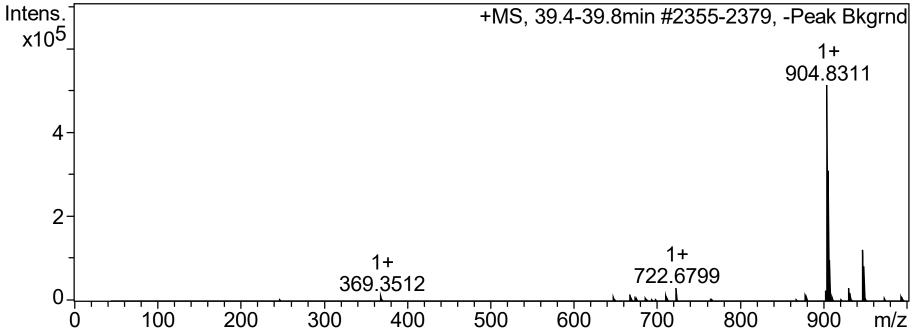

#	m/z	Res.	S/N	I	I %	FWHM
1	722.6799	38180	452.4	31083	6.1	0.0189
2	902.8025	97346	373.3	24683	4.8	0.0093
3	904.8311	68966	7751.1	511957	100.0	0.0131
4	905.8347	63021	4677.5	308754	60.3	0.0144
5	906.8376	52144	1510.8	99664	19.5	0.0174
6	907.8403	41944	323.8	21353	4.2	0.0216
7	930.8469	45615	495.3	32082	6.3	0.0204
8	946.8780	54434	1929.2	123388	24.1	0.0174
9	947.8816	47109	1297.2	82873	16.2	0.0201
10	948.8835	40237	455.1	29051	5.7	0.0236

+MS, 48.3-49.2min #2887-2939

Compound Spectrum List Report

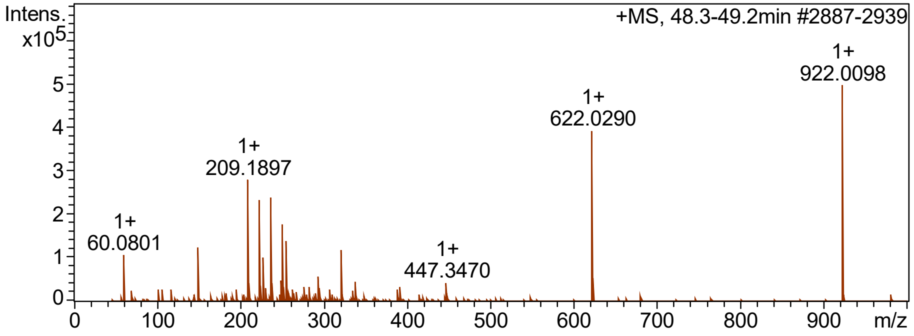

#	m/z	Res.	S/N	I	I %	FWHM
1	150.1122	32324	4761.4	125426	25.3	0.0046
2	209.1897	37099	11156.1	279631	56.3	0.0056
3	223.2053	36700	9491.9	233237	47.0	0.0061
4	237.2211	38145	9935.6	239874	48.3	0.0062
5	251.2367	37049	7382.0	178667	36.0	0.0068
6	255.2680	38637	5781.9	139432	28.1	0.0066
7	622.0290	50891	11668.6	392429	79.1	0.0122
8	922.0098	58765	16958.4	496338	100.0	0.0157
9	1221.9908	52721	15131.7	326338	65.7	0.0232
10	1521.9713	43756	10850.6	179520	36.2	0.0348

Compound Spectrum SmartFormula Report

Analysis Info

Analysis Name	C:\Data\Ficheros Datos MAXIS\HPLC\noviembre 2022\LC2167_21_01_4548.d	Acquisition Date	21/11/2022 16:56:00
Method	hplc1100_ 50 min_50-3000 t=200 6l-min.m	Operator	AMP
Sample Name	LC2167	Instrument	maXis II 1828979.22347
Comment	PLE MC 0.1 mg/ml en EtOH		

Acquisition Parameter

Source Type	ESI	Ion Polarity	Positive	Set Nebulizer	3.0 Bar
Focus	Active	Set Capillary	3500 V	Set Dry Heater	250 °C
Scan Begin	50 m/z	Set End Plate Offset	-500 V	Set Dry Gas	6.0 l/min
Scan End	3000 m/z	Set Charging Voltage	2000 V	Set Divert Valve	Source
		Set Corona	0 nA	Set APCI Heater	0 °C

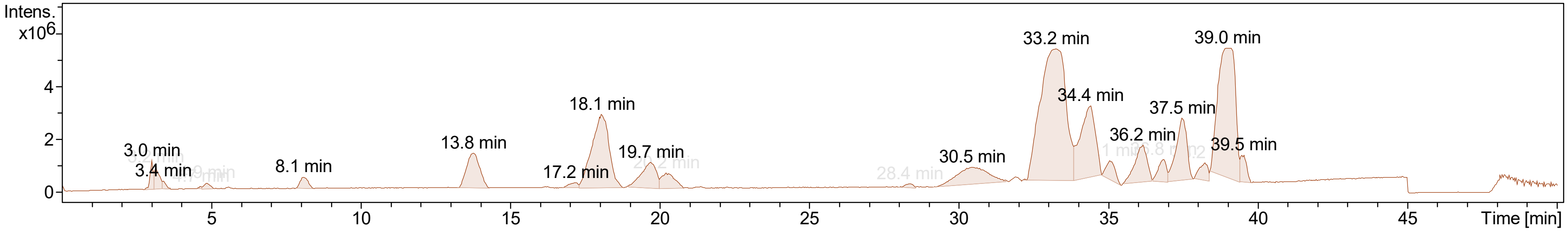

#	RT [min]	Area	Int. Type	I	S/N	Chromatogram	Max. m/z	FWHM [min]
1	3.0	8128229	Manual	1223995	43.6	BPC +All MS	205.0682	0.1
2	3.2	10331193	Manual	980110	33.4	BPC +All MS	205.0680	0.2
3	3.4	2925820	Manual	450811	11.5	BPC +All MS	197.1171	0.1
4	4.7	603108	Manual	261049	3.5	BPC +All MS	494.3269	0.1
5	4.9	3100737	Manual	383660	8.9	BPC +All MS	520.3640	0.2
6	8.1	7773097	Manual	604796	17.0	BPC +All MS	282.2791	0.3
7	13.8	40099004	Manual	1500669	52.6	BPC +All MS	338.3415	0.5
8	17.2	4155074	Manual	399710	7.8	BPC +All MS	730.5373	0.4
9	18.1	111153448	Manual	2959632	111.1	BPC +All MS	804.5773	0.6
10	19.7	34402828	Manual	1153648	39.2	BPC +All MS	806.5927	0.6
11	20.2	18296114	Manual	757580	23.4	BPC +All MS	758.5687	0.6
12	28.4	3103986	Manual	355255	6.4	BPC +All MS	600.5194	0.3
13	30.5	46073780	Manual	969180	25.2	BPC +All MS	548.5028	1.2
14	33.2	296420640	Manual	5406768	198.3	BPC +All MS	871.5726	1.0
15	34.4	96701896	Manual	3282710	107.7	BPC +All MS	871.5719	0.6
16	35.1	15116911	Manual	1227665	28.9	BPC +All MS	918.8105	0.4
17	36.2	39393868	Manual	1793838	55.3	BPC +All MS	813.5666	0.4
18	36.8	16078802	Manual	1264700	33.8	BPC +All MS	818.7220	0.3
19	37.5	58457652	Manual	2815415	93.7	BPC +All MS	820.7375	0.4
20	38.2	12993419	Manual	1141230	26.6	BPC +All MS	898.7845	0.4

Compound Spectrum SmartFormula Report

#	RT [min]	Area	Int. Type	I	S/N	Chromatogram	Max. m/z	FWHM [min]
21	39.0	199031376	Manual	5436637	194.9	BPC +All MS	902.8166	0.7
22	39.5	15176457	Manual	1430704	40.6	BPC +All MS	904.8311	0.2

Cmpd 1, 3.0 min

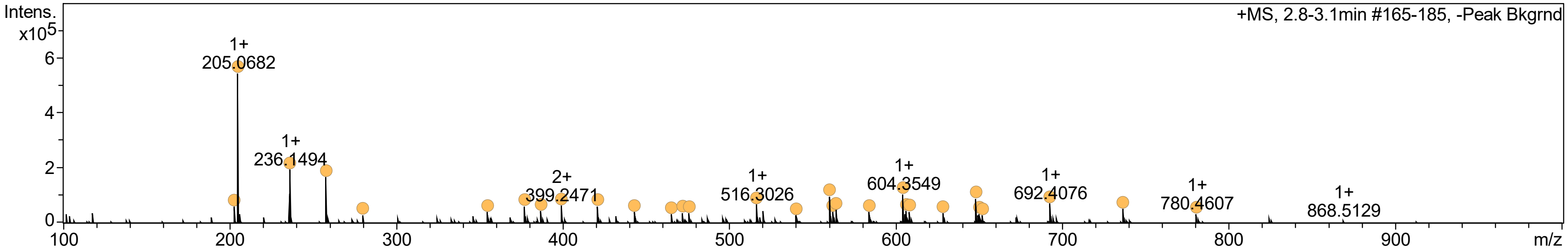

Meas. m/z	#	Ion Formula	m/z	err [ppm]	mSigma	# mSigma	Score	rdb	e¯ Conf	N-Rule
102.1273	1	C6H16N	102.1277	3.7	7.3	1	100.00	0.0	even	ok
118.0867	1	C5H12NO2	118.0863	-3.7	5.2	1	100.00	1.0	even	ok
203.0527	1	C4H7N6O4	203.0523	-1.9	6.5	1	91.69	5.0	even	ok
	2	C6H12NaO6	203.0526	-0.5	8.6	2	100.00	1.0	even	ok
	3	C5H3N10	203.0537	4.7	10.9	3	63.69	10.0	even	ok
	4	C12H11OS	203.0525	-1.0	44.3	4	55.63	8.0	even	ok
	5	C7H16NaOS2	203.0535	3.7	49.5	5	36.70	0.0	even	ok
205.0682	1	C6H14NaO6	205.0683	0.4	2.3	1	100.00	0.0	even	ok
	2	C4H9N6O4	205.0680	-1.0	2.7	2	93.96	4.0	even	ok
	3	C12H13OS	205.0682	-0.1	43.0	3	55.09	7.0	even	ok
	4	C7H18NaOS2	205.0691	4.6	43.5	4	34.92	-1.0	even	ok
236.1494	1	C10H22NO5	236.1492	-0.7	0.4	1	100.00	1.0	even	ok
	2	C11H18N5O	236.1506	5.0	13.8	2	46.94	6.0	even	ok
	3	C11H26NS2	236.1501	3.0	43.5	3	39.97	0.0	even	ok
258.1314	1	C10H21NNaO5	258.1312	-0.8	3.3	1	100.00	1.0	even	ok
	2	C8H16N7O3	258.1309	-1.9	3.5	2	87.76	5.0	even	ok
	3	C11H17N5NaO	258.1325	4.4	17.1	3	48.39	6.0	even	ok
	4	C8H24N3O2S2	258.1304	-3.7	42.1	4	38.80	-1.0	even	ok
	5	C11H25NNaS2	258.1321	2.6	44.3	5	42.84	0.0	even	ok
	6	C16H20NS	258.1311	-1.2	44.5	6	50.43	8.0	even	ok
280.0925	1	C8H20NNaO6P	280.0920	-1.7	8.6	1	100.00	0.0	even	ok
	2	C6H15N7O4P	280.0918	-2.7	8.9	2	86.53	4.0	even	ok
	3	C7H19N3NaO5S	280.0938	4.4	22.2	3	66.65	0.0	even	ok
	4	C9H16N5NaO2P	280.0934	3.1	22.5	4	61.86	5.0	even	ok
	5	C7H11N11P	280.0931	2.1	22.6	5	71.42	9.0	even	ok
	6	C5H14N9O3S	280.0935	3.4	23.0	6	57.87	4.0	even	ok
	7	C12H14N3O5	280.0928	1.0	37.0	7	58.19	8.0	even	ok

Compound Spectrum SmartFormula Report

Meas. m/z	#	Ion Formula	m/z	err [ppm]	mSigma	# mSigma	Score	rdb	e⁻ Conf	N-Rule
	8	C11H11N7NaO	280.0917	-2.8	38.4	8	43.75	10.0	even	ok
	9	C9H24NNaOPS2	280.0929	1.4	44.4	9	59.98	-1.0	even	ok
	10	C14H19NOPS	280.0919	-2.0	47.4	10	50.63	7.0	even	ok
	11	C11H19N3NaS2	280.0913	-4.5	52.9	11	29.15	4.0	even	ok
	12	C13H18N3S2	280.0937	4.1	61.6	12	23.43	7.0	even	ok
355.2206	1	C35H66O14	355.2221	4.3	3.8	1	56.03	4.0	even	ok
	2	C34H63N4NaO10	355.2215	2.8	6.4	2	74.34	6.0	even	ok
	3	C31H65N10NaOP2S	355.2213	2.0	8.5	3	83.01	6.0	even	ok
	4	C32H58N10O8	355.2214	2.4	8.8	4	76.94	10.0	even	ok
	5	C37H69NaO7P2	355.2203	-0.9	9.0	5	100.00	5.0	even	ok
	6	C30H59N14O2PS	355.2196	-2.6	9.3	6	72.63	10.0	even	ok
	7	C34H68N2O9P2	355.2195	-3.1	10.3	7	64.20	4.0	even	ok
	8	C35H64N6O5P2	355.2201	-1.3	10.4	8	91.37	9.0	even	ok
	9	C35H59N8NaO6	355.2222	4.6	11.3	9	44.66	11.0	even	ok
	10	C32H64N8NaO4PS	355.2198	-2.2	11.4	10	75.33	6.0	even	ok
	11	C33H54N14O4	355.2221	4.2	12.3	11	48.20	15.0	even	ok
	12	C32H68N6O5P2S	355.2218	3.5	12.4	12	57.05	4.0	even	ok
	13	C31H55N14NaO4	355.2209	0.9	12.6	13	93.44	12.0	even	ok
	14	C33H65N6NaO5P2	355.2189	-4.6	13.4	14	42.90	6.0	even	ok
	15	C34H61N10NaOP2	355.2196	-2.8	14.2	15	64.28	11.0	even	ok
	16	C34H73NaO7P2S	355.2219	3.9	14.3	16	50.32	0.0	even	ok
	17	C33H60N12NaPS	355.2204	-0.3	14.8	17	97.00	11.0	even	ok
	18	C29H50N20O2	355.2207	0.5	15.0	18	94.73	16.0	even	ok
	19	C33H67NaO14	355.2209	0.9	15.1	19	88.70	1.0	even	ok
	20	C33H67N4O8PS	355.2203	-0.7	15.5	20	90.12	4.0	even	ok
	21	C31H60N12O3P2	355.2188	-5.0	15.8	21	37.06	10.0	even	ok
	22	C32H51N18Na	355.2215	2.7	15.8	22	62.43	17.0	even	ok
	23	C31H62N6O12	355.2207	0.5	16.6	23	91.39	5.0	even	ok
	24	C30H56N16NaOP	355.2224	5.1	17.1	24	35.77	12.0	even	ok
	25	C29H63N10O6PS	355.2190	-4.5	17.1	25	41.21	5.0	even	ok
	26	C32H54N16OS	355.2188	-4.9	17.3	26	36.77	15.0	even	ok
	27	C39H68O7P2	355.2215	2.5	17.6	27	62.87	8.0	even	ok
	28	C34H63N8O4PS	355.2210	1.2	17.6	28	80.33	9.0	even	ok
	29	C34H66N2O11S	355.2188	-4.9	17.6	29	45.83	4.0	even	ok
	30	C28H64N12O3P2S	355.2205	-0.3	17.7	30	92.03	5.0	even	ok
	31	C30H69N6NaO5P2S	355.2206	0.1	17.9	31	93.99	1.0	even	ok
	32	C31H68N4NaO8PS	355.2191	-4.1	18.0	32	44.46	1.0	even	ok
	33	C30H59N10NaO8	355.2202	-1.0	18.3	33	81.07	7.0	even	ok
	34	C32H68N2NaO11P	355.2224	5.1	19.1	34	34.19	1.0	even	ok
	35	C38H65N4NaO3P2	355.2209	1.0	19.2	35	79.50	10.0	even	ok
	36	C32H71O12PS	355.2196	-2.6	19.3	36	74.76	-1.0	even	ok
	37	C36H60N10OP2	355.2208	0.6	19.6	37	84.04	14.0	even	ok
	38	C34H59N10NaO3S	355.2190	-4.5	19.6	38	38.70	11.0	even	ok
	39	C36H68N2NaO6PS	355.2211	1.6	20.0	39	71.44	5.0	even	ok
	40	C28H54N16O6	355.2201	-1.4	20.3	40	72.71	11.0	even	ok
	41	C35H62N6O7S	355.2195	-3.0	20.3	41	66.81	9.0	even	ok

Compound Spectrum SmartFormula Report

Meas. m/z	#	Ion Formula	m/z	err [ppm]	mSigma	# mSigma	Score	rdb	e⁻ Conf	N-Rule
	42	C37H59N8O4P	355.2193	-3.6	20.5	42	47.49	14.0	even	ok
	43	C31H72N2O9P2S	355.2211	1.6	20.6	43	69.92	-1.0	even	ok
	44	C39H64N2NaO6P	355.2194	-3.2	20.8	44	51.41	10.0	even	ok
	45	C30H63N8O9P	355.2222	4.7	20.8	45	36.36	5.0	even	ok
	46	C36H56N12NaP	355.2188	-5.1	22.5	46	31.78	16.0	even	ok
	47	C37H67NaO9S	355.2196	-2.6	22.5	47	69.05	5.0	even	ok
	48	C29H60N12NaO5P	355.2217	3.2	22.8	48	49.14	7.0	even	ok
	49	C27H51N20NaO2	355.2195	-2.9	22.9	49	51.85	13.0	even	ok
	50	C24H55N20NaO2S	355.2212	1.8	24.9	50	61.20	8.0	even	ok
	51	C27H55N18O3P	355.2216	2.8	24.9	51	50.85	11.0	even	ok
	52	C25H46N26	355.2194	-3.3	25.1	52	45.38	17.0	even	ok
	53	C22H50N26S	355.2211	1.4	25.5	53	64.71	12.0	even	ok
	54	C35H59N12PS	355.2217	3.0	25.7	54	47.34	14.0	even	ok
	55	C27H63N10NaO8S	355.2219	3.7	26.2	55	40.49	2.0	even	ok
	56	C25H58N16O6S	355.2217	3.3	26.2	56	44.12	6.0	even	ok
	57	C38H67N2O6PS	355.2223	4.9	27.0	57	37.10	8.0	even	ok
	58	C30H66N2O16	355.2201	-1.4	27.3	58	62.28	0.0	even	ok
	59	C27H59N16NaOS2	355.2193	-3.6	27.5	59	50.65	7.0	even	ok
	60	C37H64N6NaO2PS	355.2218	3.4	27.7	60	41.56	10.0	even	ok
	61	C30H64N12NaPS2	355.2221	4.4	27.8	61	41.59	6.0	even	ok
	62	C26H65N12NaO3P2S	355.2193	-3.7	27.9	62	39.33	2.0	even	ok
	63	C29H63N6NaO12	355.2195	-2.9	28.0	63	46.28	2.0	even	ok
	64	C24H60N18OP2S	355.2191	-4.1	28.5	64	35.46	6.0	even	ok
	65	C27H63N14O2PS2	355.2213	2.1	28.6	65	66.29	5.0	even	ok
	66	C29H73N2NaO9P2S	355.2199	-1.8	29.1	66	55.91	-4.0	even	ok
	67	C38H63N4NaO5S	355.2203	-0.8	29.2	67	82.77	10.0	even	ok
	68	C27H68N8O7P2S	355.2198	-2.2	29.2	68	51.92	0.0	even	ok
	69	C40H64N4O3P2	355.2221	4.4	29.2	69	32.11	13.0	even	ok
	70	C29H58N16OS2	355.2205	-0.2	29.4	70	89.83	10.0	even	ok
	71	C27H58N12O10	355.2194	-3.3	29.5	71	41.06	6.0	even	ok
	72	C29H68N8NaO4PS2	355.2215	2.5	30.3	72	59.03	1.0	even	ok
	73	C36H58N10O3S	355.2202	-1.2	30.3	73	60.41	14.0	even	ok
	74	C23H59N20PS2	355.2200	-1.6	30.6	74	69.01	6.0	even	ok
	75	C29H67N4O13P	355.2216	2.8	30.9	75	44.00	0.0	even	ok
	76	C26H55N16NaO6	355.2189	-4.8	30.9	76	27.99	8.0	even	ok
	77	C28H62N12O5S2	355.2198	-2.1	31.2	77	62.94	5.0	even	ok
	78	C39H66O9S	355.2208	0.7	31.3	78	78.86	8.0	even	ok
	79	C31H63N10NaO3S2	355.2206	0.2	31.5	79	85.17	6.0	even	ok
	80	C25H64N14NaO2PS2	355.2201	-1.3	31.6	80	72.01	2.0	even	ok
	81	C41H63N2O6P	355.2206	0.2	31.7	81	67.71	13.0	even	ok
	82	C28H64N8NaO9P	355.2210	1.3	31.9	82	56.74	2.0	even	ok
	83	C38H55N12P	355.2200	-1.7	32.1	83	52.78	19.0	even	ok
	84	C40H60N6NaO2P	355.2201	-1.3	32.5	84	55.92	15.0	even	ok
	85	C34H69N6NaP2S2	355.2194	-3.4	32.7	85	46.36	5.0	even	ok
	86	C30H67N6NaO7S2	355.2200	-1.7	33.0	86	64.58	1.0	even	ok
	87	C26H59N14O7P	355.2209	0.9	33.6	87	58.03	6.0	even	ok

Compound Spectrum SmartFormula Report

Meas. m/z	#	Ion Formula	m/z	err [ppm]	mSigma	# mSigma	Score	rdb	e¯ Conf	N-Rule
	88	C25H68N12O3P2S2	355.2222	4.5	33.7	88	35.50	0.0	even	ok
	89	C30H71N4O8PS2	355.2220	4.0	33.9	89	39.12	-1.0	even	ok
	90	C26H67N10O6PS2	355.2207	0.3	34.7	90	77.92	0.0	even	ok
	91	C27H73N6NaO5P2S2	355.2223	4.9	34.7	91	31.39	-4.0	even	ok
	92	C42H62O9	355.2191	-4.0	34.8	92	30.77	13.0	even	ok
	93	C32H66N6O7S2	355.2212	1.7	35.1	93	60.94	4.0	even	ok
	94	C25H56N18NaO3P	355.2204	-0.6	35.2	94	58.45	8.0	even	ok
	95	C28H72N4NaO8PS2	355.2208	0.6	36.0	95	71.11	-4.0	even	ok
	96	C35H72N2O4P2S2	355.2199	-1.9	36.0	96	57.36	3.0	even	ok
	97	C27H66N8O9S2	355.2192	-4.0	36.2	97	37.45	0.0	even	ok
	98	C20H51N26NaS	355.2199	-2.0	36.6	98	44.84	9.0	even	ok
	99	C23H59N16NaO6S	355.2205	-0.1	36.6	99	61.04	3.0	even	ok
	100	C31H70N2O11S2	355.2205	-0.2	36.7	100	74.83	-1.0	even	ok
	101	C33H62N10O3S2	355.2218	3.6	36.9	101	40.08	9.0	even	ok
	102	C21H60N20NaPS2	355.2188	-5.0	36.9	102	28.35	3.0	even	ok
	103	C23H51N24OP	355.2202	-1.0	37.1	103	52.23	12.0	even	ok
	104	C21H54N22O4S	355.2204	-0.5	37.2	104	56.77	7.0	even	ok
	105	C34H71NaO9S2	355.2213	2.1	37.3	105	53.62	0.0	even	ok
	106	C39H64N6P2S	355.2189	-4.8	37.5	106	23.84	13.0	even	ok
	107	C29H71N2NaO11S2	355.2193	-3.6	37.7	107	39.40	-4.0	even	ok
	108	C26H67N6NaO12S	355.2212	1.8	37.7	108	44.52	-3.0	even	ok
	109	C24H62N12O10S	355.2211	1.4	37.8	109	47.50	1.0	even	ok
	110	C28H67N2NaO16	355.2189	-4.8	38.7	110	23.00	-3.0	even	ok
	111	C22H60N18NaO3PS	355.2220	4.2	39.0	111	26.61	3.0	even	ok
	112	C35H67N4NaO5S2	355.2220	4.0	39.1	112	34.54	5.0	even	ok
	113	C29H75O12PS2	355.2213	2.1	39.3	113	50.35	-6.0	even	ok
	114	C40H62N4O5S	355.2215	2.6	39.3	114	45.95	13.0	even	ok
	115	C22H63N16O4PS2	355.2193	-3.5	39.3	115	37.98	1.0	even	ok
	116	C41H69NaO2P2S	355.2190	-4.4	39.4	116	24.96	9.0	even	ok
	117	C27H70N2O16S	355.2218	3.3	39.5	117	31.47	-5.0	even	ok
	118	C20H55N24OPS	355.2219	3.8	39.8	118	28.52	7.0	even	ok
	119	C36H68N6P2S2	355.2206	-0.0	40.0	119	70.07	8.0	even	ok
	120	C23H69N12NaO3P2S2	355.2209	1.1	40.0	120	59.67	-3.0	even	ok
	121	C24H68N10NaO6PS2	355.2195	-3.1	40.0	121	40.58	-3.0	even	ok
	122	C26H72N4O11P2S	355.2191	-4.1	40.9	122	25.91	-5.0	even	ok
	123	C28H71O17P	355.2209	0.9	41.8	123	46.55	-5.0	even	ok
	124	C39H59N8NaOS	355.2210	1.1	42.1	124	44.75	15.0	even	ok
	125	C38H73NaO2P2S2	355.2207	0.4	42.2	125	62.73	4.0	even	ok
	126	C27H68N4NaO13P	355.2204	-0.6	42.2	126	48.55	-3.0	even	ok
	127	C24H72N8O7P2S2	355.2215	2.6	42.4	127	42.49	-5.0	even	ok
	128	C25H71N6O10PS2	355.2200	-1.6	42.7	128	50.28	-5.0	even	ok
	129	C26H77N2NaO9P2S2	355.2216	3.0	43.1	129	38.49	-9.0	even	ok
	130	C42H59N6O2P	355.2213	2.1	43.5	130	36.21	18.0	even	ok
	131	C25H63N10O11P	355.2202	-1.0	43.6	131	43.86	1.0	even	ok
	132	C27H76NaO12PS2	355.2201	-1.2	43.6	132	52.45	-9.0	even	ok
	133	C44H64NaO4P	355.2214	2.5	44.4	133	32.78	14.0	even	ok

Compound Spectrum SmartFormula Report

Meas. m/z	#	Ion Formula	m/z	err [ppm]	mSigma	# mSigma	Score	rdb	e⁻ Conf	N-Rule
	134	C37H67N4O3PS2	355.2191	-4.2	44.4	134	28.02	8.0	even	ok
	135	C24H60N14NaO7P	355.2197	-2.5	44.6	135	32.61	3.0	even	ok
	136	C24H57N20NaP2	355.2219	3.6	45.0	136	19.05	8.0	even	ok
	137	C26H69N6NaO10P2	355.2219	3.6	45.9	137	24.70	-3.0	even	ok
	138	C43H68O2P2S	355.2202	-1.0	45.9	138	51.12	12.0	even	ok
	139	C22H55N20O5P	355.2195	-2.9	46.2	139	28.75	7.0	even	ok
	140	C43H58N4O5	355.2198	-2.1	46.5	140	33.00	18.0	even	ok
	141	C42H55N8NaO	355.2193	-3.6	46.8	141	24.04	20.0	even	ok
	142	C24H64N12O8P2	355.2217	3.2	47.4	142	25.74	1.0	even	ok
	143	C19H55N22NaO4S	355.2192	-3.8	48.1	143	22.05	4.0	even	ok
	144	C22H63N12NaO10S	355.2199	-1.9	48.3	144	32.33	-2.0	even	ok
	145	C41H58N8OS	355.2222	4.5	48.4	145	23.42	18.0	even	ok
	146	C23H61N16NaO4P2	355.2212	1.7	48.6	146	33.31	3.0	even	ok
	147	C20H58N18O8S	355.2197	-2.3	48.8	147	29.56	2.0	even	ok
	148	C17H50N28O2S	355.2191	-4.2	49.0	148	19.64	8.0	even	ok
	149	C25H71N2NaO16S	355.2206	-0.0	49.3	149	42.73	-8.0	even	ok
	150	C23H66N8O14S	355.2204	-0.4	49.5	150	40.05	-4.0	even	ok
	151	C43H63N2NaO3S	355.2223	4.9	50.0	151	20.21	14.0	even	ok
	152	C40H72O2P2S2	355.2219	3.8	50.8	152	25.92	7.0	even	ok
	153	C24H72N4NaO13PS	355.2220	4.2	51.1	153	18.68	-8.0	even	ok
	154	C40H68N2NaOPS2	355.2199	-2.0	52.7	154	35.16	9.0	even	ok
	155	C26H72NaO17P	355.2197	-2.5	53.0	155	25.36	-8.0	even	ok
	156	C24H67N6O15P	355.2196	-2.9	54.2	156	22.58	-4.0	even	ok
	157	C21H52N24NaOP	355.2190	-4.4	54.5	157	12.00	9.0	even	ok
	158	C23H64N10NaO11P	355.2190	-4.4	54.8	158	15.91	-2.0	even	ok
	159	C21H59N16O9P	355.2189	-4.8	56.2	159	13.81	2.0	even	ok
	160	C45H60N4NaP	355.2221	4.4	56.2	160	15.17	19.0	even	ok
	161	C25H73N2NaO14P2	355.2212	1.7	56.4	161	26.00	-8.0	even	ok
	162	C18H50N26O5	355.2223	5.0	57.3	162	12.65	8.0	even	ok
	163	C21H56N22O2P2	355.2210	1.3	57.5	163	20.18	7.0	even	ok
	164	C23H68N8O12P2	355.2210	1.4	57.7	164	26.69	-4.0	even	ok
	165	C44H54N8O	355.2205	-0.2	58.3	165	31.20	23.0	even	ok
	166	C22H65N12NaO8P2	355.2205	-0.1	58.5	166	31.47	-2.0	even	ok
	167	C46H59N2NaO3	355.2206	0.2	59.3	167	30.56	19.0	even	ok
	168	C45H63N2OPS	355.2194	-3.3	59.4	168	21.65	17.0	even	ok
	169	C20H60N18O6P2	355.2204	-0.5	59.9	169	28.24	2.0	even	ok
	170	C42H67N2OPS2	355.2211	1.4	62.5	170	28.06	12.0	even	ok
	171	C42H63N4NaS2	355.2190	-4.3	64.7	171	14.47	14.0	even	ok
	172	C43H66O4S2	355.2196	-2.8	67.0	172	18.56	12.0	even	ok
	173	C17H47N30NaO	355.2218	3.4	67.1	173	9.65	10.0	even	ok
	174	C19H57N22NaO2P2	355.2198	-2.0	70.0	174	11.50	4.0	even	ok
	175	C48H58N2O3	355.2218	3.5	71.2	175	10.86	22.0	even	ok
	176	C44H62N4S2	355.2202	-0.9	75.1	176	19.29	17.0	even	ok
377.2336	1	C35H71NaO15	377.2340	1.0	5.9	1	94.29	1.0	even	ok
	2	C33H66N6O13	377.2338	0.7	6.1	2	100.00	5.0	even	ok
	3	C36H72N2O10P2	377.2326	-2.7	7.4	3	66.04	4.0	even	ok

Compound Spectrum SmartFormula Report

Meas. m/z	#	Ion Formula	m/z	err [ppm]	mSigma	# mSigma	Score	rdb	e¯ Conf	N-Rule
	4	C32H63N10NaO9	377.2333	-0.7	7.6	4	96.41	7.0	even	ok
	5	C34H72N2NaO12P	377.2355	5.0	7.7	5	37.28	1.0	even	ok
	6	C32H67N8O10P	377.2353	4.6	9.2	6	40.17	5.0	even	ok
	7	C30H58N16O7	377.2332	-1.1	9.5	7	87.34	11.0	even	ok
	8	C35H69N6NaO6P2	377.2320	-4.1	9.5	8	45.48	6.0	even	ok
	9	C34H62N10O9	377.2345	2.4	9.8	9	67.08	10.0	even	ok
	10	C36H67N4NaO11	377.2347	2.8	10.3	10	61.34	6.0	even	ok
	11	C33H64N12O4P2	377.2319	-4.5	10.7	11	40.41	10.0	even	ok
	12	C29H65N16NaP2S	377.2330	-1.5	10.7	12	80.11	7.0	even	ok
	13	C37H70O15	377.2352	4.2	10.9	13	43.30	4.0	even	ok
	14	C31H64N12NaO6P	377.2348	3.2	11.6	14	54.73	7.0	even	ok
	15	C33H59N14NaO5	377.2340	1.0	11.7	15	84.98	12.0	even	ok
	16	C31H54N20O3	377.2338	0.7	12.5	16	89.11	16.0	even	ok
	17	C32H60N16NaO2P	377.2355	5.0	12.6	17	34.17	12.0	even	ok
	18	C29H55N20NaO3	377.2326	-2.5	12.7	18	62.41	13.0	even	ok
	19	C29H59N18O4P	377.2347	2.9	13.7	19	57.06	11.0	even	ok
	20	C30H55N22P	377.2353	4.6	14.2	20	36.67	16.0	even	ok
	21	C31H64N16P2S	377.2342	1.7	14.2	21	71.23	10.0	even	ok
	22	C30H68N12O4P2S	377.2336	-0.0	14.3	22	94.86	5.0	even	ok
	23	C27H50N26O	377.2325	-2.9	15.0	23	55.09	17.0	even	ok
	24	C31H67N6NaO13	377.2326	-2.5	15.9	24	58.80	2.0	even	ok
	25	C32H70N2O17	377.2332	-1.1	16.0	25	77.22	0.0	even	ok
	26	C32H73N6NaO6P2S	377.2337	0.3	16.1	26	87.60	1.0	even	ok
	27	C24H54N26OS	377.2342	1.6	16.4	27	70.29	12.0	even	ok
	28	C33H69N10NaO2P2S	377.2344	2.1	16.5	28	63.28	6.0	even	ok
	29	C26H59N20NaO3S	377.2343	1.9	16.9	29	64.81	8.0	even	ok
	30	C28H56N22NaP	377.2341	1.4	17.0	30	71.14	13.0	even	ok
	31	C29H62N12O11	377.2325	-2.9	17.3	31	52.78	6.0	even	ok
	32	C31H67N10O7PS	377.2321	-4.0	17.3	32	40.46	5.0	even	ok
	33	C37H68N6O6P2	377.2332	-1.0	17.9	33	76.18	9.0	even	ok
	34	C39H73NaO8P2	377.2334	-0.6	18.5	34	80.11	5.0	even	ok
	35	C28H59N16NaO7	377.2320	-4.3	18.6	35	36.51	8.0	even	ok
	36	C32H63N14O3PS	377.2327	-2.2	18.8	36	58.65	10.0	even	ok
	37	C31H71N4O14P	377.2347	2.9	18.9	37	51.24	0.0	even	ok
	38	C26H64N18O2P2S	377.2322	-3.6	19.1	38	42.99	6.0	even	ok
	39	C36H65N10NaO2P2	377.2327	-2.4	19.4	39	56.39	11.0	even	ok
	40	C33H72N4NaO9PS	377.2322	-3.6	19.4	40	42.41	1.0	even	ok
	41	C28H69N12NaO4P2S	377.2324	-3.2	19.4	41	46.59	2.0	even	ok
	42	C30H68N8NaO10P	377.2341	1.5	19.5	42	67.32	2.0	even	ok
	43	C34H60N16P2	377.2326	-2.7	19.6	43	51.84	15.0	even	ok
	44	C27H62N16O7S	377.2349	3.4	19.8	44	44.97	6.0	even	ok
	45	C34H72N6O6P2S	377.2349	3.5	19.9	45	43.25	4.0	even	ok
	46	C33H76N2O10P2S	377.2342	1.7	20.0	46	63.05	-1.0	even	ok
	47	C36H77NaO8P2S	377.2351	3.9	20.1	47	49.13	0.0	even	ok
	48	C38H67N4O9P	377.2317	-4.9	20.4	48	29.79	9.0	even	ok
	49	C26H54N22O5	377.2318	-4.7	20.5	49	31.79	12.0	even	ok

Compound Spectrum SmartFormula Report

Meas. m/z	#	Ion Formula	m/z	err [ppm]	mSigma	# mSigma	Score	rdb	e⁻ Conf	N-Rule
	50	C35H58N14O5	377.2352	4.2	20.8	50	35.58	15.0	even	ok
	51	C29H67N10NaO9S	377.2350	3.7	20.9	51	40.19	2.0	even	ok
	52	C34H68N8NaO5PS	377.2329	-1.9	21.1	52	60.10	6.0	even	ok
	53	C28H63N14O8P	377.2340	1.1	21.2	53	69.49	6.0	even	ok
	54	C34H75O13PS	377.2328	-2.2	21.2	54	69.91	-1.0	even	ok
	55	C37H63N8NaO7	377.2353	4.6	21.4	55	31.86	11.0	even	ok
	56	C35H71N4O9PS	377.2334	-0.5	22.1	56	94.58	4.0	even	ok
	57	C29H72N8O8P2S	377.2329	-1.8	22.1	57	59.37	0.0	even	ok
	58	C34H55N18NaO	377.2346	2.8	22.2	58	48.34	17.0	even	ok
	59	C27H60N18NaO4P	377.2335	-0.3	22.9	59	75.70	8.0	even	ok
	60	C31H77N2NaO10P2S	377.2330	-1.4	23.0	60	62.41	-4.0	even	ok
	61	C25H55N24O2P	377.2333	-0.7	24.9	61	68.14	12.0	even	ok
	62	C27H64N16O5P2	377.2355	5.0	25.3	62	25.78	6.0	even	ok
	63	C22H55N26NaOS	377.2330	-1.6	25.9	63	56.60	9.0	even	ok
	64	C36H70N2O12S	377.2319	-4.4	26.4	64	37.34	4.0	even	ok
	65	C30H71N2NaO17	377.2320	-4.3	26.9	65	30.51	-3.0	even	ok
	66	C26H61N20NaOP2	377.2350	3.6	27.3	66	35.59	8.0	even	ok
	67	C23H58N22O5S	377.2335	-0.2	27.4	67	69.76	7.0	even	ok
	68	C25H63N16NaO7S	377.2337	0.2	27.5	68	70.03	3.0	even	ok
	69	C28H66N8O15	377.2318	-4.7	27.9	69	27.00	1.0	even	ok
	70	C35H64N12NaOPS	377.2336	-0.1	28.4	70	69.35	11.0	even	ok
	71	C24H64N18NaO4PS	377.2351	4.1	28.8	71	30.33	3.0	even	ok
	72	C22H59N24O2PS	377.2350	3.8	29.1	72	33.03	7.0	even	ok
	73	C26H69N16NaP2S2	377.2347	3.0	29.4	73	49.00	2.0	even	ok
	74	C38H64N10O2P2	377.2339	0.8	29.4	74	60.23	14.0	even	ok
	75	C38H72N2NaO7PS	377.2342	1.7	29.6	75	64.01	5.0	even	ok
	76	C41H72O8P2	377.2346	2.6	29.7	76	42.51	8.0	even	ok
	77	C26H66N12O11S	377.2342	1.6	29.7	77	52.08	1.0	even	ok
	78	C29H74N2O17S	377.2349	3.4	29.8	78	44.59	-5.0	even	ok
	79	C25H63N20OPS2	377.2331	-1.3	29.9	79	67.92	6.0	even	ok
	80	C40H69N4NaO4P2	377.2340	1.2	30.0	80	55.64	10.0	even	ok
	81	C30H75O18P	377.2340	1.1	30.0	81	56.54	-5.0	even	ok
	82	C29H72N4NaO14P	377.2335	-0.3	30.0	82	64.29	-3.0	even	ok
	83	C28H71N6NaO13S	377.2343	2.0	30.3	83	47.85	-3.0	even	ok
	84	C27H73N8NaO8P2S	377.2317	-5.0	30.3	84	23.14	-3.0	even	ok
	85	C27H58N22S2	377.2323	-3.5	30.5	85	42.36	11.0	even	ok
	86	C36H67N8O5PS	377.2341	1.3	30.7	86	53.46	9.0	even	ok
	87	C34H58N16O2S	377.2319	-4.4	30.9	87	26.68	15.0	even	ok
	88	C27H67N10O12P	377.2333	-0.7	31.3	88	58.70	1.0	even	ok
	89	C27H68N14NaO3PS2	377.2332	-1.0	31.6	89	69.64	2.0	even	ok
	90	C37H66N6O8S	377.2326	-2.6	31.9	90	49.90	9.0	even	ok
	91	C39H63N8O5P	377.2324	-3.1	31.9	91	35.67	14.0	even	ok
	92	C26H64N14NaO8P	377.2328	-2.1	32.1	92	44.35	3.0	even	ok
	93	C28H76N4O12P2S	377.2322	-3.6	32.5	93	31.68	-5.0	even	ok
	94	C29H63N16NaO2S2	377.2324	-3.1	32.6	94	43.83	7.0	even	ok
	95	C29H67N14O3PS2	377.2344	2.2	32.6	95	53.44	5.0	even	ok

Compound Spectrum SmartFormula Report

Meas. m/z	#	Ion Formula	m/z	err [ppm]	mSigma	# mSigma	Score	rdb	e⁻ Conf	N-Rule
	96	C38H60N12NaOP	377.2319	-4.6	32.6	96	24.71	16.0	even	ok
	97	C27H72N12O4P2S2	377.2353	4.4	32.8	97	31.85	0.0	even	ok
	98	C41H68N2NaO7P	377.2325	-2.8	32.9	98	37.85	10.0	even	ok
	99	C23H64N20NaOPS2	377.2319	-4.5	33.0	99	30.99	3.0	even	ok
	100	C36H63N10NaO4S	377.2321	-4.1	33.0	100	27.88	11.0	even	ok
	101	C29H76N2O15P2	377.2355	5.1	33.1	101	21.30	-5.0	even	ok
	102	C28H73N6NaO11P2	377.2350	3.6	33.5	102	30.49	-3.0	even	ok
	103	C24H59N20O6P	377.2327	-2.5	33.7	103	39.46	7.0	even	ok
	104	C39H71NaO10S	377.2327	-2.3	33.9	104	51.25	5.0	even	ok
	105	C28H67N12NaO6S2	377.2317	-4.9	34.3	105	26.86	2.0	even	ok
	106	C29H77N6NaO6P2S2	377.2354	4.8	34.4	106	27.69	-4.0	even	ok
	107	C31H72N8NaO5PS2	377.2346	2.6	34.7	107	46.92	1.0	even	ok
	108	C26H68N12O9P2	377.2348	3.3	35.0	108	32.09	1.0	even	ok
	109	C23H68N18O2P2S2	377.2339	0.9	35.0	109	64.86	1.0	even	ok
	110	C28H71N10O7PS2	377.2338	0.5	35.2	110	69.09	0.0	even	ok
	111	C23H56N24NaO2P	377.2321	-3.9	35.2	111	27.44	9.0	even	ok
	112	C32H68N12NaOPS2	377.2352	4.4	35.7	112	29.99	6.0	even	ok
	113	C24H67N16O5PS2	377.2324	-3.1	36.1	113	40.52	1.0	even	ok
	114	C25H73N12NaO4P2S2	377.2341	1.2	36.1	114	59.18	-3.0	even	ok
	115	C25H65N16NaO5P2	377.2343	1.9	36.1	115	42.15	3.0	even	ok
	116	C30H66N12O6S2	377.2329	-1.7	36.2	116	53.77	5.0	even	ok
	117	C27H75N2NaO17S	377.2337	0.2	36.8	117	69.25	-8.0	even	ok
	118	C30H76N4NaO9PS2	377.2339	0.8	37.0	118	62.07	-4.0	even	ok
	119	C39H68N6NaO3PS	377.2349	3.5	37.3	119	36.03	10.0	even	ok
	120	C26H72N10NaO7PS2	377.2326	-2.7	37.3	120	42.59	-3.0	even	ok
	121	C21H59N22NaO5S	377.2323	-3.4	37.5	121	29.16	4.0	even	ok
	122	C31H62N16O2S2	377.2336	0.0	37.7	122	69.11	10.0	even	ok
	123	C23H60N22O3P2	377.2341	1.5	37.8	123	43.23	7.0	even	ok
	124	C29H70N8O10S2	377.2323	-3.5	37.9	124	35.13	0.0	even	ok
	125	C19H54N28O3S	377.2322	-3.8	38.0	125	26.35	8.0	even	ok
	126	C32H75N4O9PS2	377.2351	4.0	38.3	126	30.65	-1.0	even	ok
	127	C32H71N6NaO8S2	377.2331	-1.4	38.3	127	54.48	1.0	even	ok
	128	C24H67N12NaO11S	377.2330	-1.6	38.8	128	41.14	-2.0	even	ok
	129	C22H62N18O9S	377.2328	-2.0	38.9	129	38.19	2.0	even	ok
	130	C35H77N2NaO5P2S2	377.2318	-4.8	39.1	130	24.66	0.0	even	ok
	131	C40H71N2O7PS	377.2354	4.9	39.2	131	23.86	8.0	even	ok
	132	C26H76N8O8P2S2	377.2346	2.7	39.2	132	41.20	-5.0	even	ok
	133	C37H63N12OPS	377.2348	3.1	39.6	133	29.53	14.0	even	ok
	134	C38H62N10O4S	377.2333	-0.9	39.7	134	57.35	14.0	even	ok
	135	C31H75N2NaO12S2	377.2324	-3.1	39.8	135	36.41	-4.0	even	ok
	136	C20H60N24NaO2PS	377.2338	0.6	39.8	136	48.01	4.0	even	ok
	137	C33H67N10NaO4S2	377.2337	0.4	39.9	137	61.43	6.0	even	ok
	138	C23H68N14NaO8PS	377.2345	2.4	40.4	138	33.91	-2.0	even	ok
	139	C28H81N2NaO10P2S2	377.2347	3.0	40.4	139	36.73	-9.0	even	ok
	140	C31H79O13PS2	377.2344	2.2	40.6	140	43.17	-6.0	even	ok
	141	C27H75N6O11PS2	377.2331	-1.3	40.6	141	51.64	-5.0	even	ok

Compound Spectrum SmartFormula Report

Meas. m/z	#	Ion Formula	m/z	err [ppm]	mSigma	# mSigma	Score	rdb	e¯ Conf	N-Rule
	142	C21H51N30P	377.2320	-4.3	40.7	142	16.20	13.0	even	ok
	143	C25H70N8O15S	377.2335	-0.2	40.7	143	49.74	-4.0	even	ok
	144	C21H63N20O6PS	377.2343	2.0	40.8	144	36.21	2.0	even	ok
	145	C28H76NaO18P	377.2328	-2.1	41.2	145	35.03	-8.0	even	ok
	146	C42H68N4O4P2	377.2352	4.4	41.3	146	20.63	13.0	even	ok
	147	C40H67N4NaO6S	377.2334	-0.5	41.5	147	57.99	10.0	even	ok
	148	C41H65N8NaP2	377.2347	3.0	41.7	148	28.71	15.0	even	ok
	149	C24H71N10O12PS	377.2350	3.8	42.0	149	23.59	-4.0	even	ok
	150	C26H76N4NaO14PS	377.2352	4.1	42.0	150	21.50	-8.0	even	ok
	151	C33H74N2O12S2	377.2336	0.1	42.0	151	61.36	-1.0	even	ok
	152	C29H80NaO13PS2	377.2332	-0.9	42.0	152	53.05	-9.0	even	ok
	153	C26H71N6O16P	377.2327	-2.5	42.2	153	31.53	-4.0	even	ok
	154	C37H59N14NaS	377.2327	-2.3	42.4	154	32.55	16.0	even	ok
	155	C25H68N10NaO12P	377.2321	-3.9	42.5	155	22.61	-2.0	even	ok
	156	C36H73N6NaOP2S2	377.2325	-3.0	42.8	156	34.60	5.0	even	ok
	157	C34H70N6O8S2	377.2343	1.8	43.3	157	43.57	4.0	even	ok
	158	C41H70O10S	377.2339	0.9	43.5	158	51.15	8.0	even	ok
	159	C40H59N12OP	377.2331	-1.4	43.7	159	37.53	19.0	even	ok
	160	C23H63N16O10P	377.2320	-4.3	43.8	160	19.82	2.0	even	ok
	161	C34H63N14NaS2	377.2344	2.2	44.2	161	39.51	11.0	even	ok
	162	C27H77N2NaO15P2	377.2343	1.9	44.3	162	33.52	-8.0	even	ok
	163	C43H67N2O7P	377.2337	0.4	44.5	163	43.21	13.0	even	ok
	164	C42H64N6NaO3P	377.2332	-1.0	44.5	164	39.10	15.0	even	ok
	165	C20H54N26O6	377.2354	4.9	44.9	165	16.24	8.0	even	ok
	166	C36H75NaO10S2	377.2344	2.2	45.5	166	37.96	0.0	even	ok
	167	C25H72N8O13P2	377.2342	1.5	45.5	167	34.75	-4.0	even	ok
	168	C37H76N2O5P2S2	377.2330	-1.6	45.8	168	42.45	3.0	even	ok
	169	C24H69N12NaO9P2	377.2336	0.1	46.1	169	43.42	-2.0	even	ok
	170	C19H51N30NaO2	377.2349	3.5	46.1	170	22.54	10.0	even	ok
	171	C41H68N6OP2S	377.2320	-4.3	46.2	171	23.06	13.0	even	ok
	172	C35H66N10O4S2	377.2349	3.6	47.1	172	26.51	9.0	even	ok
	173	C22H64N18O7P2	377.2335	-0.3	47.5	173	40.31	2.0	even	ok
	174	C43H63N4NaO6	377.2317	-5.0	47.7	174	14.62	15.0	even	ok
	175	C43H73NaO3P2S	377.2321	-3.9	47.9	175	24.09	9.0	even	ok
	176	C44H66O10	377.2323	-3.6	48.0	176	20.90	13.0	even	ok
	177	C21H61N22NaO3P2	377.2329	-1.7	48.6	177	30.52	4.0	even	ok
	178	C37H71N4NaO6S2	377.2351	4.0	49.3	178	22.65	5.0	even	ok
	179	C41H63N8NaO2S	377.2341	1.3	50.3	179	39.29	15.0	even	ok
	180	C38H72N6OP2S2	377.2337	0.2	51.3	180	45.64	8.0	even	ok
	181	C42H66N4O6S	377.2346	2.7	51.8	181	28.30	13.0	even	ok
	182	C17H46N36	377.2348	3.1	53.4	182	14.77	14.0	even	ok
	183	C40H77NaO3P2S2	377.2338	0.6	53.4	183	40.22	4.0	even	ok
	184	C25H75N2O20P	377.2320	-4.2	53.5	184	14.90	-9.0	even	ok
	185	C22H66N12O16	377.2354	4.9	53.6	185	12.42	-3.0	even	ok
	186	C21H63N16NaO12	377.2349	3.5	53.9	186	17.71	-1.0	even	ok
	187	C39H58N14S	377.2339	0.9	54.2	187	29.66	19.0	even	ok

Compound Spectrum SmartFormula Report

Meas. m/z	#	Ion Formula	m/z	err [ppm]	mSigma	# mSigma	Score	rdb	e⁻ Conf	N-Rule
	188	C39H71N4O4PS2	377.2322	-3.8	55.8	188	19.47	8.0	even	ok
	189	C44H63N6O3P	377.2344	2.2	56.2	189	21.91	18.0	even	ok
	190	C19H56N28OP2	377.2328	-2.1	56.3	190	16.71	8.0	even	ok
	191	C46H68NaO5P	377.2346	2.6	57.3	191	19.54	14.0	even	ok
	192	C42H54N14	377.2322	-3.6	58.4	192	15.00	24.0	even	ok
	193	C45H72O3P2S	377.2333	-0.7	58.5	193	33.30	12.0	even	ok
	194	C44H59N8NaO2	377.2324	-3.2	59.4	194	15.81	20.0	even	ok
	195	C45H62N4O6	377.2329	-1.8	59.6	195	21.23	18.0	even	ok
	196	C43H62N8O2S	377.2353	4.5	61.0	196	13.72	18.0	even	ok
	197	C45H67N2NaO4S	377.2354	4.8	62.7	197	11.71	14.0	even	ok
	198	C42H76O3P2S2	377.2350	3.8	62.7	198	15.47	7.0	even	ok
	199	C40H67N8PS2	377.2328	-2.0	62.8	199	22.81	13.0	even	ok
	200	C42H72N2NaO2PS2	377.2330	-1.6	64.8	200	22.80	9.0	even	ok
	201	C47H64N4NaOP	377.2352	4.3	69.1	201	8.53	19.0	even	ok
	202	C46H58N8O2	377.2336	-0.0	71.3	202	18.95	23.0	even	ok
	203	C47H67N2O2PS	377.2325	-2.9	72.1	203	13.46	17.0	even	ok
	204	C48H63N2NaO4	377.2337	0.4	72.5	204	17.12	19.0	even	ok
	205	C44H71N2O2PS2	377.2342	1.6	74.9	205	15.87	12.0	even	ok
	206	C44H67N4NaOS2	377.2322	-3.8	77.2	206	8.90	14.0	even	ok
	207	C45H70O5S2	377.2327	-2.4	79.4	207	11.25	12.0	even	ok
	208	C50H68NaPS	377.2333	-0.8	83.5	208	12.95	18.0	even	ok
	209	C49H59N6Na	377.2344	2.1	84.4	209	7.76	24.0	even	ok
	210	C50H62N2O4	377.2349	3.6	84.6	210	5.61	22.0	even	ok
	211	C47H72NaPS2	377.2350	3.7	85.3	211	6.56	13.0	even	ok
	212	C49H62N4OS	377.2317	-5.1	85.9	212	4.45	22.0	even	ok
	213	C46H66N4OS2	377.2334	-0.6	87.8	213	11.04	17.0	even	ok
	214	C52H67PS	377.2345	2.4	94.7	214	5.80	21.0	even	ok
387.1481	1	C10H16N14NaO2	387.1473	-2.1	4.9	1	76.43	10.0	even	ok
	2	C11H19N10O6	387.1484	0.7	5.3	2	100.00	8.0	even	ok
	3	C8H11N20	387.1470	-2.8	5.4	3	64.80	14.0	even	ok
	4	C13H24N4NaO8	387.1486	1.4	7.5	4	84.44	4.0	even	ok
	5	C14H27O12	387.1497	4.1	11.1	5	42.33	2.0	even	ok
	6	C8H21N12NaO3P	387.1489	2.2	11.5	6	67.07	5.0	even	ok
	7	C10H23N6O10	387.1470	-2.8	11.7	7	58.11	3.0	even	ok
	8	C9H24N8O7P	387.1500	4.9	12.7	8	33.05	3.0	even	ok
	9	C6H16N18OP	387.1487	1.4	12.7	9	75.70	9.0	even	ok
	10	C12H28NaO12	387.1473	-2.1	12.8	10	66.63	-1.0	even	ok
	11	C12H15N14O2	387.1497	4.1	17.3	11	37.72	13.0	even	ok
	12	C14H20N8NaO4	387.1500	4.8	18.3	12	30.34	9.0	even	ok
	13	C11H29N6O3P2S	387.1492	2.7	22.3	13	63.32	2.0	even	ok
	14	C13H34NaO5P2S	387.1494	3.5	24.4	14	50.95	-2.0	even	ok
	15	C12H28N4O6PS	387.1462	-5.0	25.9	15	32.68	2.0	even	ok
	16	C13H24N8O2PS	387.1475	-1.5	27.4	16	72.29	7.0	even	ok
	17	C18H29O5P2	387.1485	1.0	27.6	17	60.12	6.0	even	ok
	18	C17H26N4NaOP2	387.1474	-1.8	28.0	18	50.83	8.0	even	ok
	19	C15H29N2NaO4PS	387.1478	-0.8	29.6	19	78.46	3.0	even	ok

Compound Spectrum SmartFormula Report

Meas. m/z	#	Ion Formula	m/z	err [ppm]	mSigma	# mSigma	Score	rdb	e¯ Conf	N-Rule
	20	C16H25N6NaPS	387.1491	2.6	35.5	20	47.17	8.0	even	ok
	21	C17H24N4NaO3S	387.1461	-5.1	39.6	21	22.52	8.0	even	ok
	22	C19H25N4OP2	387.1498	4.4	40.6	22	19.86	11.0	even	ok
	23	C18H27O7S	387.1472	-2.3	41.8	23	42.54	6.0	even	ok
	24	C20H24N2O4P	387.1468	-3.3	43.8	24	24.11	11.0	even	ok
	25	C10H24N10NaOS2	387.1468	-3.3	44.0	25	31.94	4.0	even	ok
	26	C11H27N6O5S2	387.1479	-0.6	47.9	26	50.07	2.0	even	ok
	27	C12H23N10OS2	387.1492	2.9	48.4	27	31.00	7.0	even	ok
	28	C19H23N4O3S	387.1485	1.1	49.3	28	43.33	11.0	even	ok
	29	C13H32NaO7S2	387.1482	0.2	50.0	29	49.96	-2.0	even	ok
	30	C14H28N4NaO3S2	387.1495	3.6	50.6	30	24.38	3.0	even	ok
	31	C17H34NaP2S2	387.1469	-3.0	52.8	31	26.37	2.0	even	ok
	32	C21H20N6P	387.1482	0.1	57.0	32	30.22	16.0	even	ok
	33	C23H25NaO2P	387.1484	0.9	57.9	33	25.95	12.0	even	ok
	34	C19H33P2S2	387.1493	3.2	60.6	34	19.49	5.0	even	ok
	35	C25H20N2NaO	387.1468	-3.4	74.3	35	8.48	17.0	even	ok
	36	C27H19N2O	387.1492	2.8	87.2	36	5.77	20.0	even	ok
399.2471	1	C31H69N16NaOP2S	399.2461	-2.3	6.6	1	65.37	7.0	even	ok
	2	C29H59N24NaS	399.2481	2.6	7.1	2	60.21	13.0	even	ok
	3	C32H72N12O5P2S	399.2467	-0.9	8.3	3	83.12	5.0	even	ok
	4	C30H62N20O4S	399.2486	3.9	8.5	4	41.98	11.0	even	ok
	5	C37H75NaO16	399.2471	0.1	8.9	5	95.39	1.0	even	ok
	6	C34H77N6NaO7P2S	399.2468	-0.6	9.9	6	86.00	1.0	even	ok
	7	C32H67N14NaO6S	399.2488	4.3	10.3	7	36.89	7.0	even	ok
	8	C35H70N6O14	399.2470	-0.3	10.5	8	89.97	5.0	even	ok
	9	C33H71N10O8PS	399.2452	-4.7	10.6	9	40.87	5.0	even	ok
	10	C28H63N20NaO4S	399.2474	0.9	11.2	10	78.95	8.0	even	ok
	11	C26H58N26O2S	399.2473	0.6	11.8	11	83.19	12.0	even	ok
	12	C36H76N2NaO13P	399.2486	3.8	11.9	12	40.64	1.0	even	ok
	13	C35H80N2O11P2S	399.2474	0.7	12.3	13	100.00	-1.0	even	ok
	14	C31H71N10NaO10S	399.2481	2.6	12.5	14	67.95	2.0	even	ok
	15	C35H76N4NaO10PS	399.2453	-4.3	12.5	15	43.64	1.0	even	ok
	16	C29H66N16O8S	399.2480	2.3	12.9	16	58.40	6.0	even	ok
	17	C33H68N16OP2S	399.2473	0.7	13.4	17	78.46	10.0	even	ok
	18	C27H64N22NaOPS	399.2489	4.7	13.6	18	31.00	8.0	even	ok
	19	C30H73N12NaO5P2S	399.2455	-4.0	13.8	19	37.87	2.0	even	ok
	20	C34H67N10NaO10	399.2464	-1.6	13.9	20	65.88	7.0	even	ok
	21	C34H71N8O11P	399.2484	3.5	14.0	21	42.79	5.0	even	ok
	22	C38H76N2O11P2	399.2457	-3.5	14.3	22	42.56	4.0	even	ok
	23	C33H81N2NaO11P2S	399.2462	-2.3	14.4	23	70.86	-4.0	even	ok
	24	C28H68N18O3P2S	399.2453	-4.3	14.4	24	33.97	6.0	even	ok
	25	C39H74O16	399.2483	3.1	14.4	25	46.72	4.0	even	ok
	26	C34H74N2O18	399.2463	-1.9	14.8	26	60.32	0.0	even	ok
	27	C35H73N10NaO3P2S	399.2475	1.1	15.0	27	71.33	6.0	even	ok
	28	C32H74N6O14S	399.2486	4.0	15.2	28	46.01	0.0	even	ok
	29	C31H76N8O9P2S	399.2460	-2.6	15.3	29	51.44	0.0	even	ok

Compound Spectrum SmartFormula Report

Meas. m/z	#	Ion Formula	m/z	err [ppm]	mSigma	# mSigma	Score	rdb	e⁻ Conf	N-Rule
	30	C36H76N6O7P2S	399.2480	2.4	15.5	30	67.00	4.0	even	ok
	31	C36H79O14PS	399.2459	-3.0	15.7	31	58.24	-1.0	even	ok
	32	C38H71N4NaO12	399.2478	1.8	15.9	32	61.23	6.0	even	ok
	33	C32H62N16O8	399.2463	-2.0	16.0	33	58.82	11.0	even	ok
	34	C33H64N18NaPS	399.2453	-4.4	16.2	34	32.35	12.0	even	ok
	35	C36H66N10O10	399.2476	1.4	16.4	35	65.09	10.0	even	ok
	36	C34H79NaO16S	399.2488	4.3	16.5	36	40.71	-4.0	even	ok
	37	C33H71N6NaO14	399.2457	-3.3	16.9	37	42.49	2.0	even	ok
	38	C37H73N6NaO7P2	399.2451	-4.8	17.3	38	27.60	6.0	even	ok
	39	C34H67N14O4PS	399.2459	-3.0	17.4	39	44.88	10.0	even	ok
	40	C38H81NaO9P2S	399.2482	2.8	17.4	40	59.45	0.0	even	ok
	41	C36H72N8NaO6PS	399.2460	-2.7	17.5	41	60.78	6.0	even	ok
	42	C33H68N12NaO7P	399.2479	2.1	17.6	42	54.63	7.0	even	ok
	43	C31H66N12O12	399.2456	-3.6	19.0	43	37.20	6.0	even	ok
	44	C33H75N4O15P	399.2478	1.8	19.0	44	56.96	0.0	even	ok
	45	C35H63N14NaO6	399.2471	0.1	19.2	45	77.96	12.0	even	ok
	46	C37H75N4O10PS	399.2465	-1.3	19.6	46	77.37	4.0	even	ok
	47	C31H59N20NaO4	399.2457	-3.3	19.8	47	39.88	13.0	even	ok
	48	C31H63N18O5P	399.2478	1.8	19.8	48	56.14	11.0	even	ok
	49	C33H58N20O4	399.2469	-0.3	20.4	49	73.53	16.0	even	ok
	50	C34H64N16NaO3P	399.2486	3.8	20.7	50	34.09	12.0	even	ok
	51	C28H70N12O12S	399.2473	0.6	20.9	51	86.25	1.0	even	ok
	52	C30H75N6NaO14S	399.2474	0.9	20.9	52	80.83	-3.0	even	ok
	53	C32H72N8NaO11P	399.2472	0.5	21.3	53	69.80	2.0	even	ok
	54	C27H67N16NaO8S	399.2468	-0.7	21.8	54	65.86	3.0	even	ok
	55	C30H63N16NaO8	399.2451	-5.0	21.8	55	23.98	8.0	even	ok
	56	C29H54N26O2	399.2456	-3.6	22.0	56	34.77	17.0	even	ok
	57	C28H73N16NaOP2S2	399.2478	1.9	22.1	57	64.75	2.0	even	ok
	58	C24H59N26NaO2S	399.2461	-2.4	22.2	58	46.39	9.0	even	ok
	59	C32H59N22OP	399.2484	3.5	22.3	59	36.06	16.0	even	ok
	60	C25H62N22O6S	399.2466	-1.1	22.4	60	60.96	7.0	even	ok
	61	C27H67N20O2PS2	399.2462	-2.2	22.8	61	60.84	6.0	even	ok
	62	C31H78N2O18S	399.2480	2.3	23.0	62	58.92	-5.0	even	ok
	63	C30H72N16OP2S2	399.2490	5.0	23.2	63	29.22	5.0	even	ok
	64	C30H80N4O13P2S	399.2453	-4.3	23.4	64	35.26	-5.0	even	ok
	65	C22H54N32S	399.2459	-2.8	23.5	65	41.64	13.0	even	ok
	66	C30H68N18NaPS2	399.2470	-0.1	23.5	66	88.05	7.0	even	ok
	67	C30H67N14O9P	399.2471	0.1	23.5	67	70.54	6.0	even	ok
	68	C30H60N22NaOP	399.2472	0.5	23.6	68	66.48	13.0	even	ok
	69	C41H77NaO9P2	399.2465	-1.5	23.7	69	55.25	5.0	even	ok
	70	C39H72N6O7P2	399.2463	-1.8	23.8	70	51.37	9.0	even	ok
	71	C38H74N2O13S	399.2450	-5.1	23.9	71	27.74	4.0	even	ok
	72	C32H55N24Na	399.2464	-1.6	23.9	72	53.25	18.0	even	ok
	73	C26H68N18NaO5PS	399.2483	3.0	24.3	73	38.78	3.0	even	ok
	74	C29H76N8NaO11PS	399.2489	4.7	24.5	74	24.55	-3.0	even	ok
	75	C29H72N14NaO4PS2	399.2463	-1.8	24.5	75	63.05	2.0	even	ok

Compound Spectrum SmartFormula Report

Meas. m/z	#	Ion Formula	m/z	err [ppm]	mSigma	# mSigma	Score	rdb	e^- Conf	N-Rule
	76	C27H71N14O9PS	399.2488	4.3	24.9	76	26.92	1.0	even	ok
	77	C39H77N4NaO5P2S	399.2488	4.4	25.1	77	32.50	5.0	even	ok
	78	C24H63N24O3PS	399.2481	2.7	25.3	78	41.21	7.0	even	ok
	79	C29H62N22OS2	399.2454	-4.2	25.3	79	34.42	11.0	even	ok
	80	C40H70N4O12	399.2490	4.8	25.3	80	23.51	9.0	even	ok
	81	C32H75N2NaO18	399.2451	-5.0	25.4	81	22.24	-3.0	even	ok
	82	C29H76N12O5P2S2	399.2484	3.3	25.5	82	44.01	0.0	even	ok
	83	C23H62N26O2S2	399.2490	4.8	25.7	83	28.89	7.0	even	ok
	84	C31H73N10NaO8P2	399.2487	4.2	25.9	84	27.26	2.0	even	ok
	85	C38H69N10NaO3P2	399.2458	-3.1	26.0	85	36.01	11.0	even	ok
	86	C37H72N10O3P2S	399.2487	4.1	26.1	86	28.06	9.0	even	ok
	87	C29H64N18NaO5P	399.2466	-1.2	26.4	87	54.31	8.0	even	ok
	88	C37H62N14O6	399.2483	3.1	26.5	88	36.13	15.0	even	ok
	89	C39H67N8NaO8	399.2484	3.4	26.5	89	33.08	11.0	even	ok
	90	C36H64N16OP2	399.2457	-3.5	26.7	90	32.46	15.0	even	ok
	91	C31H71N14O4PS2	399.2475	1.2	26.9	91	67.23	5.0	even	ok
	92	C31H81N6NaO7P2S2	399.2485	3.6	27.2	92	38.71	-4.0	even	ok
	93	C38H71N8O6PS	399.2472	0.3	27.4	93	77.67	9.0	even	ok
	94	C35H63N18PS	399.2465	-1.3	27.4	94	51.81	15.0	even	ok
	95	C31H67N16NaO3S2	399.2455	-3.9	27.5	95	36.01	7.0	even	ok
	96	C25H72N18O3P2S2	399.2470	-0.1	27.7	96	80.54	1.0	even	ok
	97	C29H68N16O6P2	399.2486	3.9	28.1	97	28.49	6.0	even	ok
	98	C30H75N10O8PS2	399.2469	-0.5	28.1	98	74.77	0.0	even	ok
	99	C32H79O19P	399.2471	0.1	28.2	99	63.19	-5.0	even	ok
	100	C36H62N16O3S	399.2450	-5.1	28.3	100	24.93	15.0	even	ok
	101	C26H71N16O6PS2	399.2455	-3.8	28.5	101	35.57	1.0	even	ok
	102	C27H77N12NaO5P2S2	399.2472	0.3	28.6	102	76.56	-3.0	even	ok
	103	C36H59N18NaO2	399.2478	1.7	28.6	103	46.47	17.0	even	ok
	104	C27H59N24O3P	399.2464	-1.6	28.7	104	48.02	12.0	even	ok
	105	C37H68N12NaO2PS	399.2467	-1.0	29.0	105	53.38	11.0	even	ok
	106	C40H76N2NaO8PS	399.2473	0.7	29.1	106	70.17	5.0	even	ok
	107	C33H76N8NaO6PS2	399.2477	1.6	29.1	107	59.64	1.0	even	ok
	108	C34H54N24	399.2476	1.4	29.1	108	49.23	21.0	even	ok
	109	C31H76N4NaO15P	399.2466	-1.2	29.4	109	50.69	-3.0	even	ok
	110	C28H76N10NaO8PS2	399.2457	-3.5	29.7	110	37.89	-3.0	even	ok
	111	C32H67N18PS2	399.2482	2.9	29.9	111	43.79	10.0	even	ok
	112	C38H67N10NaO5S	399.2452	-4.7	29.9	112	26.67	11.0	even	ok
	113	C32H80N4NaO10PS2	399.2470	-0.1	30.0	113	75.84	-4.0	even	ok
	114	C29H79N2NaO18S	399.2468	-0.7	30.7	114	67.11	-8.0	even	ok
	115	C32H84N2O11P2S2	399.2490	5.0	30.8	115	24.38	-6.0	even	ok
	116	C27H74N8O16S	399.2466	-1.1	30.8	116	62.77	-4.0	even	ok
	117	C32H70N12O7S2	399.2460	-2.5	30.9	117	46.23	5.0	even	ok
	118	C28H65N20NaO2P2	399.2481	2.5	31.1	118	36.97	8.0	even	ok
	119	C31H74N8O11S2	399.2454	-4.2	31.1	119	30.07	0.0	even	ok
	120	C29H71N10O13P	399.2464	-1.6	31.2	120	45.29	1.0	even	ok
	121	C39H70N6O9S	399.2457	-3.4	31.4	121	37.11	9.0	even	ok

Compound Spectrum SmartFormula Report

Meas. m/z	#	Ion Formula	m/z	err [ppm]	mSigma	# mSigma	Score	rdb	e⁻ Conf	N-Rule
	122	C28H80N8O9P2S2	399.2477	1.6	31.5	122	55.71	-5.0	even	ok
	123	C21H63N26NaO2S2	399.2478	1.8	31.9	123	53.15	4.0	even	ok
	124	C34H72N12NaO2PS2	399.2483	3.2	32.1	124	38.09	6.0	even	ok
	125	C31H80N2O16P2	399.2486	3.9	32.1	125	25.74	-5.0	even	ok
	126	C28H80N4NaO15PS	399.2483	3.0	32.2	126	40.05	-8.0	even	ok
	127	C34H79N4O10PS2	399.2482	2.9	32.6	127	40.75	-1.0	even	ok
	128	C30H85N2NaO11P2S2	399.2478	2.0	32.7	128	50.32	-9.0	even	ok
	129	C29H79N6O12PS2	399.2462	-2.1	32.9	129	48.04	-5.0	even	ok
	130	C33H79N2NaO13S2	399.2455	-3.9	33.1	130	31.51	-4.0	even	ok
	131	C26H71N12NaO12S	399.2461	-2.4	33.1	131	36.00	-2.0	even	ok
	132	C34H75N6NaO9S2	399.2462	-2.2	33.1	132	47.33	1.0	even	ok
	133	C28H68N14NaO9P	399.2459	-2.9	33.2	133	32.16	3.0	even	ok
	134	C41H75NaO11S	399.2458	-3.0	33.2	134	38.69	5.0	even	ok
	135	C23H63N22NaO6S	399.2454	-4.1	33.3	135	23.49	4.0	even	ok
	136	C26H60N26P2	399.2479	2.2	33.4	136	37.74	12.0	even	ok
	137	C29H83O19PS	399.2488	4.4	33.5	137	27.27	-10.0	even	ok
	138	C33H83O14PS2	399.2475	1.2	33.6	138	57.04	-6.0	even	ok
	139	C30H77N6NaO12P2	399.2481	2.5	33.7	139	34.53	-3.0	even	ok
	140	C24H66N18O10S	399.2460	-2.8	33.7	140	32.71	2.0	even	ok
	141	C43H76O9P2	399.2477	1.6	33.8	141	42.51	8.0	even	ok
	142	C22H66N22O6S2	399.2483	3.1	34.1	142	37.09	2.0	even	ok
	143	C33H66N16O3S2	399.2467	-0.9	34.3	143	59.92	10.0	even	ok
	144	C21H58N28O4S	399.2453	-4.5	34.3	144	20.74	8.0	even	ok
	145	C31H84NaO14PS2	399.2463	-1.8	34.3	145	49.95	-9.0	even	ok
	146	C40H68N10O3P2	399.2470	-0.1	34.6	146	53.90	14.0	even	ok
	147	C24H71N16NaO8S2	399.2484	3.5	34.7	147	33.43	-2.0	even	ok
	148	C42H73N4NaO5P2	399.2471	0.2	34.8	148	52.81	10.0	even	ok
	149	C35H75N8O6PS2	399.2489	4.6	35.1	149	24.54	4.0	even	ok
	150	C26H63N20O7P	399.2458	-3.2	35.2	150	28.03	7.0	even	ok
	151	C25H72N14NaO9PS	399.2476	1.3	35.6	151	42.50	-2.0	even	ok
	152	C28H72N12O10P2	399.2479	2.2	35.6	152	35.57	1.0	even	ok
	153	C26H75N10O13PS	399.2481	2.7	36.3	153	31.36	-4.0	even	ok
	154	C22H64N24NaO3PS	399.2469	-0.4	36.4	154	49.54	4.0	even	ok
	155	C23H67N20O7PS	399.2474	1.0	36.4	155	44.42	2.0	even	ok
	156	C41H67N8O6P	399.2455	-3.9	36.4	156	23.08	14.0	even	ok
	157	C35H71N10NaO5S2	399.2469	-0.5	36.5	157	60.20	6.0	even	ok
	158	C39H67N12O2PS	399.2479	2.0	36.6	158	44.83	14.0	even	ok
	159	C35H78N2O13S2	399.2467	-0.9	36.6	159	56.51	-1.0	even	ok
	160	C43H72N2NaO8P	399.2457	-3.5	36.9	160	24.96	10.0	even	ok
	161	C25H74N12O12S2	399.2490	4.8	37.4	161	21.53	-4.0	even	ok
	162	C37H80N2NaO8PS2	399.2490	4.9	37.4	162	20.87	0.0	even	ok
	163	C38H58N18O2	399.2490	4.8	37.5	163	17.45	20.0	even	ok
	164	C20H59N30OPS	399.2468	-0.7	37.6	164	45.16	8.0	even	ok
	165	C25H60N24NaO3P	399.2452	-4.6	37.6	165	18.33	9.0	even	ok
	166	C27H69N16NaO6P2	399.2474	0.9	37.6	166	43.98	3.0	even	ok
	167	C41H72N6NaO4PS	399.2480	2.4	38.1	167	39.89	10.0	even	ok

Compound Spectrum SmartFormula Report

Meas. m/z	#	Ion Formula	m/z	err [ppm]	mSigma	# mSigma	Score	rdb	e⁻ Conf	N-Rule
	168	C36H72N12P2S2	399.2454	-4.1	38.5	168	25.83	9.0	even	ok
	169	C30H80NaO19P	399.2459	-2.9	39.3	169	27.49	-8.0	even	ok
	170	C39H63N14NaOS	399.2458	-3.1	39.3	170	32.87	16.0	even	ok
	171	C42H75N2O8PS	399.2485	3.7	39.4	171	27.78	8.0	even	ok
	172	C36H74N6O9S2	399.2474	0.8	39.6	172	52.48	4.0	even	ok
	173	C25H64N22O4P2	399.2473	0.5	39.7	173	44.26	7.0	even	ok
	174	C23H55N30OP	399.2451	-4.9	39.8	174	15.58	13.0	even	ok
	175	C25H75N8NaO16S	399.2454	-4.1	40.0	175	24.69	-7.0	even	ok
	176	C28H83N2O16PS2	399.2455	-3.8	40.1	176	26.55	-10.0	even	ok
	177	C40H66N10O5S	399.2464	-1.7	40.4	177	43.11	14.0	even	ok
	178	C38H77N6NaO2P2S2	399.2456	-3.7	40.6	178	26.79	5.0	even	ok
	179	C28H75N6O17P	399.2458	-3.2	40.8	179	24.22	-4.0	even	ok
	180	C26H78N4O20S	399.2460	-2.8	41.0	180	33.76	-9.0	even	ok
	181	C38H79NaO11S2	399.2475	1.2	41.8	181	46.23	0.0	even	ok
	182	C27H72N10NaO13P	399.2452	-4.6	42.0	182	16.31	-2.0	even	ok
	183	C42H71N4NaO7S	399.2465	-1.4	42.0	183	44.20	10.0	even	ok
	184	C24H61N26NaP2	399.2467	-0.8	42.2	184	38.92	9.0	even	ok
	185	C36H67N14NaOS2	399.2475	1.2	42.4	185	45.57	11.0	even	ok
	186	C29H81N2NaO16P2	399.2474	0.9	43.1	186	37.74	-8.0	even	ok
	187	C39H80N2O6P2S2	399.2461	-2.4	43.3	187	34.43	3.0	even	ok
	188	C43H74O11S	399.2470	-0.0	43.5	188	53.72	8.0	even	ok
	189	C25H67N16O11P	399.2451	-4.9	43.8	189	14.00	2.0	even	ok
	190	C24H63N20NaO9	399.2487	4.1	43.8	190	17.90	4.0	even	ok
	191	C27H76N8O14P2	399.2473	0.5	44.7	191	38.35	-4.0	even	ok
	192	C37H70N10O5S2	399.2481	2.5	45.0	192	32.03	9.0	even	ok
	193	C44H72N4O5P2	399.2483	3.2	45.3	193	21.29	13.0	even	ok
	194	C22H58N26O7	399.2485	3.7	45.7	194	18.64	8.0	even	ok
	195	C26H73N12NaO10P2	399.2467	-0.8	46.2	195	34.84	-2.0	even	ok
	196	C43H69N8NaOP2	399.2478	1.9	46.3	196	28.16	15.0	even	ok
	197	C39H75N4NaO7S2	399.2482	2.8	47.2	197	27.70	5.0	even	ok
	198	C43H72N6O2P2S	399.2451	-4.9	47.5	198	15.62	13.0	even	ok
	199	C21H55N30NaO3	399.2480	2.4	47.7	199	24.36	10.0	even	ok
	200	C42H68N10NaPS	399.2487	4.0	47.9	200	19.96	15.0	even	ok
	201	C42H63N12O2P	399.2462	-2.2	48.0	201	25.10	19.0	even	ok
	202	C24H68N18O8P2	399.2466	-1.2	48.0	202	30.95	2.0	even	ok
	203	C45H71N2O8P	399.2469	-0.5	48.0	203	34.86	13.0	even	ok
	204	C44H68N6NaO4P	399.2463	-1.8	48.6	204	26.58	15.0	even	ok
	205	C45H77NaO4P2S	399.2452	-4.6	49.0	205	16.56	9.0	even	ok
	206	C19H50N36O	399.2479	2.0	49.7	206	24.75	14.0	even	ok
	207	C23H65N22NaO4P2	399.2461	-2.5	49.8	207	22.13	4.0	even	ok
	208	C40H78O11S2	399.2487	4.2	49.9	208	18.04	3.0	even	ok
	209	C41H62N14OS	399.2470	-0.0	50.1	209	44.20	19.0	even	ok
	210	C40H76N6O2P2S2	399.2468	-0.7	50.1	210	39.47	8.0	even	ok
	211	C26H75N6NaO19	399.2487	4.1	50.6	211	14.56	-7.0	even	ok
	212	C46H70O11	399.2454	-4.3	51.1	212	13.67	13.0	even	ok
	213	C27H79N2O21P	399.2451	-4.9	51.2	213	11.29	-9.0	even	ok

Compound Spectrum SmartFormula Report

Meas. m/z	#	Ion Formula	m/z	err [ppm]	mSigma	# mSigma	Score	rdb	e¯ Conf	N-Rule
	214	C43H67N8NaO3S	399.2472	0.3	51.6	214	40.49	15.0	even	ok
	215	C21H60N28O2P2	399.2459	-2.9	51.8	215	19.21	8.0	even	ok
	216	C24H70N12O17	399.2485	3.7	52.1	216	15.30	-3.0	even	ok
	217	C38H66N14OS2	399.2487	4.2	52.2	217	16.92	14.0	even	ok
	218	C42H81NaO4P2S2	399.2469	-0.4	52.2	218	39.32	4.0	even	ok
	219	C44H70N4O7S	399.2477	1.6	52.8	219	30.48	13.0	even	ok
	220	C23H67N16NaO13	399.2480	2.4	53.1	220	20.53	-1.0	even	ok
	221	C40H71N8NaO3S2	399.2489	4.5	54.2	221	14.36	10.0	even	ok
	222	C41H75N4O5PS2	399.2453	-4.5	54.6	222	14.43	8.0	even	ok
	223	C21H62N22O11	399.2479	2.0	54.8	223	21.04	3.0	even	ok
	224	C20H59N26NaO7	399.2473	0.7	56.2	224	26.09	5.0	even	ok
	225	C45H68N8OP2	399.2490	4.9	57.0	225	9.36	18.0	even	ok
	226	C18H54N32O5	399.2472	0.4	58.0	226	26.13	9.0	even	ok
	227	C46H73N4NaP2S	399.2459	-2.9	58.9	227	18.85	14.0	even	ok
	228	C46H67N6O4P	399.2475	1.2	59.8	228	21.33	18.0	even	ok
	229	C47H76O4P2S	399.2464	-1.6	59.9	229	24.48	12.0	even	ok
	230	C43H77N4NaP2S2	399.2476	1.3	60.1	230	25.61	9.0	even	ok
	231	C45H64N10NaP	399.2470	-0.2	60.3	231	24.96	20.0	even	ok
	232	C48H72NaO6P	399.2477	1.5	60.7	232	19.31	14.0	even	ok
	233	C44H58N14O	399.2454	-4.3	62.2	233	9.47	24.0	even	ok
	234	C44H80O4P2S2	399.2481	2.6	62.2	234	17.92	7.0	even	ok
	235	C45H66N8O3S	399.2484	3.3	62.5	235	15.11	18.0	even	ok
	236	C42H71N8OPS2	399.2459	-2.8	62.6	236	17.14	13.0	even	ok
	237	C47H66N4O7	399.2460	-2.6	62.8	237	14.28	18.0	even	ok
	238	C46H63N8NaO3	399.2455	-3.9	63.0	238	10.14	20.0	even	ok
	239	C47H71N2NaO5S	399.2485	3.7	64.1	239	13.08	14.0	even	ok
	240	C44H76N2NaO3PS2	399.2461	-2.4	64.6	240	17.33	9.0	even	ok
	241	C17H51N36NaO	399.2467	-1.0	68.8	241	12.04	11.0	even	ok
	242	C48H72N4P2S	399.2471	0.1	69.9	242	22.44	17.0	even	ok
	243	C45H76N4P2S2	399.2488	4.3	70.7	243	8.59	12.0	even	ok
	244	C47H63N10P	399.2482	2.8	71.6	244	9.79	23.0	even	ok
	245	C50H71O6P	399.2489	4.5	72.4	245	6.09	17.0	even	ok
	246	C49H68N4NaO2P	399.2483	3.2	72.5	246	8.68	19.0	even	ok
	247	C49H71N2O3PS	399.2456	-3.6	73.7	247	9.22	17.0	even	ok
	248	C48H62N8O3	399.2467	-0.9	74.6	248	13.07	23.0	even	ok
	249	C46H75N2O3PS2	399.2473	0.6	75.2	249	16.96	12.0	even	ok
	250	C50H67N2NaO5	399.2468	-0.6	75.7	250	13.35	19.0	even	ok
	251	C44H66N10S2	399.2451	-4.9	75.8	251	6.07	18.0	even	ok
	252	C46H71N4NaO2S2	399.2453	-4.5	77.7	252	6.23	14.0	even	ok
	253	C47H74O6S2	399.2458	-3.2	79.7	253	8.23	12.0	even	ok
	254	C51H68N4P2	399.2454	-4.1	83.4	254	4.40	22.0	even	ok
	255	C52H72NaOPS	399.2464	-1.6	85.4	255	9.19	18.0	even	ok
	256	C49H76NaOPS2	399.2481	2.6	86.1	256	7.21	13.0	even	ok
	257	C51H63N6NaO	399.2475	1.1	87.6	257	7.40	24.0	even	ok
	258	C52H66N2O5	399.2480	2.5	87.7	258	5.59	22.0	even	ok
	259	C48H70N4O2S2	399.2465	-1.5	88.7	259	8.21	17.0	even	ok

Compound Spectrum SmartFormula Report

Meas. m/z	#	Ion Formula	m/z	err [ppm]	mSigma	# mSigma	Score	rdb	e¯ Conf	N-Rule
	260	C54H71OPS	399.2476	1.4	96.7	260	5.83	21.0	even	ok
	261	C53H62N6O	399.2487	4.1	99.6	261	2.14	27.0	even	ok
	262	C54H67N2NaS	399.2456	-3.7	99.7	262	3.00	23.0	even	ok
	263	C51H71N2NaS2	399.2473	0.5	100.0	263	5.87	18.0	even	ok
421.2600	1	C25H67N22NaO7S	421.2585	-3.4	2.5	1	47.09	4.0	even	ok
	2	C23H62N28O5S	421.2584	-3.8	2.6	2	42.84	8.0	even	ok
	3	C25H71N20O8PS	421.2606	1.4	2.7	3	76.55	2.0	even	ok
	4	C27H76N14NaO10PS	421.2607	1.7	3.8	4	69.98	-2.0	even	ok
	5	C24H68N24NaO4PS	421.2600	0.1	3.9	5	95.43	4.0	even	ok
	6	C26H70N18O11S	421.2591	-2.2	5.2	6	62.11	2.0	even	ok
	7	C24H72N22O5P2S	421.2621	4.9	5.6	7	28.15	2.0	even	ok
	8	C22H63N30O2PS	421.2599	-0.2	6.1	8	90.75	8.0	even	ok
	9	C22H59N32NaOS	421.2579	-5.0	6.1	9	27.27	10.0	even	ok
	10	C28H75N12NaO13S	421.2592	-1.8	7.4	10	80.59	-2.0	even	ok
	11	C28H79N10O14PS	421.2612	3.0	7.4	11	60.50	-4.0	even	ok
	12	C23H69N26NaOP2S	421.2615	3.7	8.3	12	39.60	4.0	even	ok
	13	C30H84N4NaO16PS	421.2614	3.3	9.3	13	53.56	-8.0	even	ok
	14	C26H67N24O4PS	421.2612	3.0	10.2	14	46.21	7.0	even	ok
	15	C32H84NaO20P	421.2590	-2.3	10.2	15	55.30	-8.0	even	ok
	16	C24H71N18NaO11S	421.2579	-5.0	10.4	16	25.33	-1.0	even	ok
	17	C29H78N8O17S	421.2597	-0.6	10.7	17	98.03	-4.0	even	ok
	18	C28H72N18NaO6PS	421.2614	3.3	11.1	18	41.54	3.0	even	ok
	19	C34H83O20P	421.2602	0.6	11.2	19	77.02	-5.0	even	ok
	20	C25H74N14O15S	421.2584	-3.7	11.4	20	45.95	-3.0	even	ok
	21	C25H64N28NaPS	421.2607	1.7	11.5	21	61.07	9.0	even	ok
	22	C27H79N8NaO17S	421.2585	-3.4	12.0	22	49.85	-7.0	even	ok
	23	C29H75N14O10PS	421.2619	4.6	12.1	23	28.11	1.0	even	ok
	24	C31H81N8NaO10P2S	421.2579	-4.9	12.3	24	25.62	-3.0	even	ok
	25	C33H84N2O17P2	421.2617	4.1	12.4	25	31.98	-5.0	even	ok
	26	C30H79N6O18P	421.2589	-2.6	12.6	26	48.80	-4.0	even	ok
	27	C31H83N2NaO19S	421.2599	-0.2	12.8	27	100.00	-8.0	even	ok
	28	C31H87O20PS	421.2619	4.6	12.8	28	34.50	-10.0	even	ok
	29	C24H58N32OS	421.2591	-2.2	12.8	29	53.80	13.0	even	ok
	30	C31H80N8NaO12PS	421.2620	4.9	13.0	30	31.07	-3.0	even	ok
	31	C32H84N4O14P2S	421.2585	-3.6	13.3	31	46.09	-5.0	even	ok
	32	C26H63N26NaO3S	421.2592	-1.8	13.4	32	57.47	9.0	even	ok
	33	C27H66N22O7S	421.2597	-0.6	13.4	33	74.36	7.0	even	ok
	34	C34H79N2NaO19	421.2582	-4.2	13.9	34	30.37	-3.0	even	ok
	35	C33H80N4NaO16P	421.2597	-0.7	14.4	35	71.45	-3.0	even	ok
	36	C32H74N8O17	421.2580	-4.6	14.6	36	27.05	1.0	even	ok
	37	C31H85N2NaO17P2	421.2605	1.3	14.7	37	62.89	-8.0	even	ok
	38	C29H71N16NaO9S	421.2599	-0.2	14.9	38	76.59	3.0	even	ok
	39	C28H82N4O21S	421.2591	-2.1	14.9	39	64.97	-9.0	even	ok
	40	C30H74N12O13S	421.2604	1.0	15.2	40	81.91	1.0	even	ok
	41	C31H75N10O14P	421.2595	-1.0	15.8	41	65.22	1.0	even	ok
	42	C32H81N6NaO13P2	421.2612	2.9	16.3	42	42.18	-3.0	even	ok

Compound Spectrum SmartFormula Report

Meas. m/z	#	Ion Formula	m/z	err [ppm]	mSigma	# mSigma	Score	rdb	e⁻ Conf	N-Rule
	43	C29H76N10NaO14P	421.2583	-3.9	16.4	43	32.18	-2.0	even	ok
	44	C21H62N32OS2	421.2607	1.8	16.5	44	67.43	8.0	even	ok
	45	C29H80N8O15P2	421.2604	1.0	17.0	45	64.08	-4.0	even	ok
	46	C32H79N6NaO15S	421.2605	1.4	17.1	46	73.70	-3.0	even	ok
	47	C30H76N12O11P2	421.2610	2.5	18.1	47	44.14	1.0	even	ok
	48	C29H83N2O22P	421.2582	-4.2	18.4	48	28.12	-9.0	even	ok
	49	C23H67N26NaO3S2	421.2609	2.2	18.6	49	59.81	4.0	even	ok
	50	C28H79N6NaO20	421.2618	4.3	18.7	50	26.62	-7.0	even	ok
	51	C27H71N16O12P	421.2582	-4.2	18.8	51	27.78	2.0	even	ok
	52	C33H82N2O19S	421.2611	2.6	19.4	52	52.49	-5.0	even	ok
	53	C30H72N14NaO10P	421.2590	-2.3	19.5	53	45.81	3.0	even	ok
	54	C26H67N20NaO10	421.2618	4.3	20.4	54	25.75	4.0	even	ok
	55	C26H73N22NaP2S2	421.2596	-0.9	20.7	55	75.40	3.0	even	ok
	56	C28H77N12NaO11P2	421.2598	-0.3	20.7	56	66.95	-2.0	even	ok
	57	C26H74N12O18	421.2617	4.0	20.8	57	28.12	-3.0	even	ok
	58	C28H67N20O8P	421.2589	-2.6	21.3	58	40.71	7.0	even	ok
	59	C33H80N8O10P2S	421.2591	-2.0	21.4	59	58.41	0.0	even	ok
	60	C30H72N18O4P2S	421.2584	-3.6	21.7	60	30.93	6.0	even	ok
	61	C27H63N28PS	421.2619	4.6	21.8	61	23.14	12.0	even	ok
	62	C35H79N4O16P	421.2609	2.2	21.9	62	44.44	0.0	even	ok
	63	C29H69N22NaP2S	421.2579	-4.9	22.0	63	20.90	8.0	even	ok
	64	C24H70N22O7S2	421.2614	3.4	22.1	64	40.13	2.0	even	ok
	65	C29H73N16NaO7P2	421.2605	1.3	22.1	65	53.91	3.0	even	ok
	66	C29H68N22NaO2PS	421.2620	4.9	22.6	66	20.44	8.0	even	ok
	67	C24H62N26O8	421.2616	4.0	22.7	67	27.03	8.0	even	ok
	68	C28H84N4O19P2	421.2597	-0.6	22.8	68	60.40	-9.0	even	ok
	69	C25H67N26OPS2	421.2580	-4.8	22.9	69	26.64	7.0	even	ok
	70	C32H77N12NaO6P2S	421.2586	-3.3	22.9	70	33.01	2.0	even	ok
	71	C35H85N2NaO12P2S	421.2593	-1.7	23.0	71	60.78	-4.0	even	ok
	72	C26H72N18O9P2	421.2597	-0.6	23.1	72	59.83	2.0	even	ok
	73	C36H78N2O19	421.2594	-1.4	23.5	73	51.42	0.0	even	ok
	74	C25H71N16NaO14	421.2611	2.7	23.5	74	37.46	-1.0	even	ok
	75	C27H76N18O4P2S2	421.2601	0.4	23.7	75	77.27	1.0	even	ok
	76	C31H70N16O9S	421.2611	2.6	23.9	76	47.78	6.0	even	ok
	77	C34H76N8NaO12P	421.2604	0.9	24.1	77	55.53	2.0	even	ok
	78	C33H77N10NaO9P2	421.2618	4.5	24.1	78	22.70	2.0	even	ok
	79	C27H68N22O5P2	421.2604	0.9	24.2	79	55.10	7.0	even	ok
	80	C26H75N16NaO9S2	421.2616	3.8	24.3	80	34.85	-2.0	even	ok
	81	C28H62N26O3S	421.2604	1.0	24.6	81	53.67	12.0	even	ok
	82	C32H71N14O10P	421.2602	0.6	24.6	82	58.37	6.0	even	ok
	83	C25H66N26O3S2	421.2621	5.0	24.9	83	23.33	7.0	even	ok
	84	C35H75N6NaO15	421.2589	-2.6	24.9	84	37.23	2.0	even	ok
	85	C27H72N20NaO3PS2	421.2581	-4.4	25.0	85	28.19	3.0	even	ok
	86	C33H70N12O13	421.2587	-3.0	25.1	86	34.14	6.0	even	ok
	87	C31H72N16O7P2	421.2617	4.1	25.2	87	24.55	6.0	even	ok
	88	C27H81N8NaO15P2	421.2592	-1.9	25.2	88	44.10	-7.0	even	ok

Compound Spectrum SmartFormula Report

Meas. m/z	#	Ion Formula	m/z	err [ppm]	mSigma	# mSigma	Score	rdb	e¯ Conf	N-Rule
	89	C27H64N24NaO4P	421.2583	-3.9	25.3	89	26.49	9.0	even	ok
	90	C33H75N10NaO11S	421.2612	3.0	25.4	90	42.46	2.0	even	ok
	91	C27H79N12O11PS2	421.2580	-4.7	25.8	91	25.04	-4.0	even	ok
	92	C23H66N22O12	421.2610	2.4	25.8	92	38.62	3.0	even	ok
	93	C30H67N20NaO5S	421.2605	1.4	25.8	93	48.86	8.0	even	ok
	94	C29H81N12NaO6P2S2	421.2603	0.7	25.9	94	69.17	-3.0	even	ok
	95	C23H59N30NaO4	421.2611	2.7	26.7	95	34.91	10.0	even	ok
	96	C34H78N6O15S	421.2617	4.2	26.8	96	28.79	0.0	even	ok
	97	C25H69N22NaO5P2	421.2592	-1.9	26.9	97	42.24	4.0	even	ok
	98	C25H59N30O2P	421.2582	-4.2	27.2	98	22.96	13.0	even	ok
	99	C32H67N16NaO9	421.2582	-4.2	27.4	99	22.63	8.0	even	ok
	100	C31H68N18NaO6P	421.2597	-0.7	27.6	100	53.55	8.0	even	ok
	101	C27H78N12O13S2	421.2621	5.0	27.9	101	21.68	-4.0	even	ok
	102	C29H84N6NaO13PS2	421.2581	-4.4	27.9	102	26.46	-8.0	even	ok
	103	C28H75N16O7PS2	421.2586	-3.2	28.0	103	37.90	1.0	even	ok
	104	C30H62N22O7	421.2580	-4.6	28.1	104	20.11	12.0	even	ok
	105	C26H65N26NaOP2	421.2598	-0.3	28.2	105	56.33	9.0	even	ok
	106	C36H83NaO17S	421.2619	4.6	28.5	106	24.92	-4.0	even	ok
	107	C30H69N20NaO3P2	421.2612	2.9	28.6	107	32.24	8.0	even	ok
	108	C29H63N24O4P	421.2595	-1.0	28.6	108	49.01	12.0	even	ok
	109	C22H63N26NaO8	421.2604	1.1	28.9	109	47.47	5.0	even	ok
	110	C30H84N8O10P2S2	421.2608	2.0	29.0	110	49.27	-5.0	even	ok
	111	C21H54N36O2	421.2610	2.4	29.1	111	35.85	14.0	even	ok
	112	C24H55N34Na	421.2618	4.3	29.2	112	21.14	15.0	even	ok
	113	C23H64N28O3P2	421.2590	-2.2	29.3	113	36.98	8.0	even	ok
	114	C28H72N22P2S2	421.2608	2.0	29.3	114	49.10	6.0	even	ok
	115	C28H64N26OP2	421.2610	2.5	30.0	115	33.89	12.0	even	ok
	116	C30H80N10NaO9PS2	421.2588	-2.8	30.1	116	39.22	-3.0	even	ok
	117	C34H76N12O6P2S	421.2598	-0.4	31.1	117	64.57	5.0	even	ok
	118	C32H89N2NaO12P2S2	421.2609	2.3	31.2	118	43.21	-9.0	even	ok
	119	C20H58N32O6	421.2603	0.8	31.3	119	47.88	9.0	even	ok
	120	C30H77N16NaO2P2S2	421.2609	2.3	31.3	120	43.28	2.0	even	ok
	121	C30H87N2O17PS2	421.2586	-3.1	31.5	121	34.91	-10.0	even	ok
	122	C28H60N28NaP	421.2590	-2.3	32.0	122	34.24	14.0	even	ok
	123	C36H81N6NaO8P2S	421.2599	-0.1	32.4	123	66.26	1.0	even	ok
	124	C31H68N22P2S	421.2591	-2.0	33.2	124	35.31	11.0	even	ok
	125	C36H75N8O12P	421.2616	3.8	33.2	125	22.49	5.0	even	ok
	126	C31H83N6O13PS2	421.2593	-1.6	33.3	126	48.83	-5.0	even	ok
	127	C37H84N2O12P2S	421.2605	1.2	33.3	127	52.81	-1.0	even	ok
	128	C38H80N2NaO14P	421.2617	4.1	33.5	128	20.27	1.0	even	ok
	129	C29H71N20O3PS2	421.2593	-1.6	33.6	129	48.30	6.0	even	ok
	130	C31H80N12O6P2S2	421.2615	3.6	33.8	130	29.27	0.0	even	ok
	131	C33H73N16NaO2P2S	421.2593	-1.7	34.3	131	37.05	7.0	even	ok
	132	C35H75N10O9PS	421.2583	-4.0	34.5	132	25.66	5.0	even	ok
	133	C19H55N36NaO2	421.2598	-0.5	34.6	133	46.88	11.0	even	ok
	134	C34H71N14NaO7S	421.2619	4.5	34.9	134	21.34	7.0	even	ok

Compound Spectrum SmartFormula Report

Meas. m/z	#	Ion Formula	m/z	err [ppm]	mSigma	# mSigma	Score	rdb	e¯ Conf	N-Rule
	135	C35H72N12NaO8P	421.2610	2.5	35.0	135	30.12	7.0	even	ok
	136	C37H74N6O15	421.2601	0.2	35.1	136	48.29	5.0	even	ok
	137	C33H67N18O6P	421.2609	2.2	35.2	137	32.45	11.0	even	ok
	138	C33H88NaO15PS2	421.2594	-1.2	35.5	138	49.46	-9.0	even	ok
	139	C31H76N14NaO5PS2	421.2594	-1.2	35.7	139	49.15	2.0	even	ok
	140	C39H79NaO17	421.2602	0.6	35.7	140	44.83	1.0	even	ok
	141	C33H85N6NaO8P2S2	421.2616	3.9	35.9	141	25.22	-4.0	even	ok
	142	C37H80N4NaO11PS	421.2584	-3.6	36.0	142	27.20	1.0	even	ok
	143	C34H66N16O9	421.2594	-1.4	36.2	143	37.62	11.0	even	ok
	144	C36H71N10NaO11	421.2595	-1.0	36.4	144	40.11	7.0	even	ok
	145	C38H83O15PS	421.2590	-2.4	37.2	145	36.69	-1.0	even	ok
	146	C31H63N24NaOS	421.2612	2.9	37.3	146	25.40	13.0	even	ok
	147	C32H66N20O5S	421.2617	4.2	37.5	147	17.68	11.0	even	ok
	148	C32H64N22NaO2P	421.2603	0.9	37.5	148	39.99	13.0	even	ok
	149	C30H59N28P	421.2602	0.6	38.1	149	41.92	17.0	even	ok
	150	C33H63N20NaO5	421.2589	-2.6	38.2	150	26.76	13.0	even	ok
	151	C32H79N10O9PS2	421.2600	0.0	38.2	151	57.60	0.0	even	ok
	152	C31H58N26O3	421.2587	-3.0	38.4	152	24.41	17.0	even	ok
	153	C32H75N12NaO8S2	421.2579	-4.8	40.0	153	17.24	2.0	even	ok
	154	C34H84N4NaO11PS2	421.2601	0.4	40.3	154	51.34	-4.0	even	ok
	155	C17H50N42	421.2596	-0.8	41.3	155	27.66	15.0	even	ok
	156	C37H77N10NaO4P2S	421.2606	1.5	42.4	156	38.85	6.0	even	ok
	157	C33H78N8O12S2	421.2585	-3.5	42.6	157	23.50	0.0	even	ok
	158	C38H80N6O8P2S	421.2611	2.8	43.0	158	28.44	4.0	even	ok
	159	C35H87O15PS2	421.2607	1.6	43.1	159	37.02	-6.0	even	ok
	160	C32H72N18NaOPS2	421.2601	0.3	43.2	160	47.47	7.0	even	ok
	161	C40H80N2O12P2	421.2588	-2.8	43.8	161	21.87	4.0	even	ok
	162	C40H85NaO10P2S	421.2613	3.1	44.4	162	25.08	0.0	even	ok
	163	C36H71N14O5PS	421.2590	-2.4	44.4	163	29.94	10.0	even	ok
	164	C37H72N12O6P2	421.2581	-4.4	44.7	164	13.57	10.0	even	ok
	165	C35H83N2NaO14S2	421.2586	-3.2	44.7	165	24.21	-4.0	even	ok
	166	C39H77N6NaO8P2	421.2582	-4.1	45.0	166	14.91	6.0	even	ok
	167	C33H75N14O5PS2	421.2606	1.6	45.2	167	34.94	5.0	even	ok
	168	C31H66N22O2S2	421.2585	-3.5	45.5	168	21.52	11.0	even	ok
	169	C38H76N8NaO7PS	421.2591	-2.1	45.7	169	31.19	6.0	even	ok
	170	C35H72N16O2P2S	421.2605	1.2	45.9	170	30.04	10.0	even	ok
	171	C34H63N22O2P	421.2615	3.8	46.2	171	15.82	16.0	even	ok
	172	C36H68N16NaO4P	421.2617	4.1	46.3	172	14.35	12.0	even	ok
	173	C39H79N4O11PS	421.2596	-0.8	46.6	173	39.72	4.0	even	ok
	174	C38H70N10O11	421.2607	1.8	46.9	174	25.51	10.0	even	ok
	175	C41H78O17	421.2614	3.4	46.9	175	17.11	4.0	even	ok
	176	C35H80N8NaO7PS2	421.2608	1.9	47.1	176	30.64	1.0	even	ok
	177	C33H71N16NaO4S2	421.2586	-3.2	47.4	177	22.29	7.0	even	ok
	178	C40H75N4NaO13	421.2609	2.1	47.5	178	23.23	6.0	even	ok
	179	C35H62N20O5	421.2601	0.2	47.7	179	34.15	16.0	even	ok
	180	C37H67N14NaO7	421.2602	0.5	48.0	180	31.92	12.0	even	ok

Compound Spectrum SmartFormula Report

Meas. m/z	#	Ion Formula	m/z	err [ppm]	mSigma	# mSigma	Score	rdb	e⁻ Conf	N-Rule
	181	C35H68N18NaOPS	421.2584	-3.7	49.2	181	14.96	12.0	even	ok
	182	C34H59N24NaO	421.2595	-1.1	49.3	182	27.76	18.0	even	ok
	183	C36H83N4O11PS2	421.2613	3.2	49.4	183	20.92	-1.0	even	ok
	184	C34H74N12O8S2	421.2592	-1.9	49.5	184	28.64	5.0	even	ok
	185	C40H78N2O14S	421.2581	-4.3	50.4	185	14.75	4.0	even	ok
	186	C36H79N6NaO10S2	421.2593	-1.6	51.5	186	28.98	1.0	even	ok
	187	C39H76N10O4P2S	421.2618	4.4	53.0	187	13.51	9.0	even	ok
	188	C34H71N18OPS2	421.2613	3.2	53.5	188	18.52	10.0	even	ok
	189	C37H82N2O14S2	421.2598	-0.3	53.8	189	34.56	-1.0	even	ok
	190	C41H81N4NaO6P2S	421.2619	4.7	54.3	190	11.69	5.0	even	ok
	191	C37H67N18OPS	421.2596	-0.8	54.6	191	30.93	15.0	even	ok
	192	C36H76N12NaO3PS2	421.2615	3.5	55.3	192	15.96	6.0	even	ok
	193	C37H80N8O5P2S2	421.2579	-5.0	55.4	193	10.25	4.0	even	ok
	194	C41H76N6O8P2	421.2594	-1.2	55.6	194	22.03	9.0	even	ok
	195	C39H72N12NaO3PS	421.2598	-0.5	55.8	195	31.66	11.0	even	ok
	196	C34H67N20NaS2	421.2593	-1.6	56.0	196	25.10	12.0	even	ok
	197	C43H81NaO10P2	421.2596	-0.9	56.2	197	23.07	5.0	even	ok
	198	C38H68N16O2P2	421.2588	-2.8	56.3	198	14.92	15.0	even	ok
	199	C40H75N8O7PS	421.2603	0.8	56.5	199	29.15	9.0	even	ok
	200	C40H73N10NaO4P2	421.2589	-2.5	56.6	200	16.03	11.0	even	ok
	201	C37H79N8O7PS2	421.2620	4.8	57.2	201	10.25	4.0	even	ok
	202	C39H85N2NaO7P2S2	421.2580	-4.6	57.3	202	10.72	0.0	even	ok
	203	C35H70N16O4S2	421.2598	-0.3	57.7	203	30.47	10.0	even	ok
	204	C42H80N2NaO9PS	421.2604	1.1	57.8	204	26.09	5.0	even	ok
	205	C38H66N16O4S	421.2581	-4.3	58.0	205	11.53	15.0	even	ok
	206	C42H75N4O11P	421.2579	-4.8	58.1	206	8.00	9.0	even	ok
	207	C39H66N14O7	421.2614	3.4	58.6	207	11.90	15.0	even	ok
	208	C42H74N4O13	421.2621	5.0	58.7	208	7.32	9.0	even	ok
	209	C38H73N14NaP2S	421.2613	3.1	58.7	209	12.90	11.0	even	ok
	210	C41H71N8NaO9	421.2615	3.7	59.2	210	10.62	11.0	even	ok
	211	C36H58N24O	421.2607	1.8	59.2	211	17.31	21.0	even	ok
	212	C40H71N10NaO6S	421.2583	-4.0	59.3	212	12.20	11.0	even	ok
	213	C37H75N10NaO6S2	421.2600	-0.0	59.5	213	30.33	6.0	even	ok
	214	C38H63N18NaO3	421.2609	2.1	59.6	214	15.86	17.0	even	ok
	215	C41H74N6O10S	421.2588	-2.7	60.1	215	16.81	9.0	even	ok
	216	C38H78N6O10S2	421.2605	1.3	61.5	216	22.52	4.0	even	ok
	217	C43H79NaO12S	421.2589	-2.4	61.6	217	17.35	5.0	even	ok
	218	C40H83NaO12S2	421.2606	1.6	63.5	218	19.62	0.0	even	ok
	219	C38H76N12OP2S2	421.2585	-3.4	64.2	219	12.30	9.0	even	ok
	220	C40H81N6NaO3P2S2	421.2587	-3.1	66.0	220	12.63	5.0	even	ok
	221	C41H71N12O3PS	421.2610	2.4	66.6	221	14.61	14.0	even	ok
	222	C36H66N20S2	421.2605	1.2	66.6	222	18.87	15.0	even	ok
	223	C42H72N10O4P2	421.2601	0.4	67.3	223	17.44	14.0	even	ok
	224	C45H80O10P2	421.2608	2.0	67.4	224	12.58	8.0	even	ok
	225	C41H84N2O7P2S2	421.2592	-1.8	67.7	225	16.13	3.0	even	ok
	226	C43H76N6NaO5PS	421.2611	2.7	67.9	226	12.84	10.0	even	ok

Compound Spectrum SmartFormula Report

Meas. m/z	#	Ion Formula	m/z	err [ppm]	mSigma	# mSigma	Score	rdb	e⁻ Conf	N-Rule
	227	C44H77N4NaO6P2	421.2603	0.7	68.0	227	16.03	10.0	even	ok
	228	C39H62N20S	421.2588	-2.8	68.2	228	12.57	20.0	even	ok
	229	C41H69N14NaP2	421.2596	-0.9	68.3	229	15.16	16.0	even	ok
	230	C38H71N14NaO2S2	421.2606	1.6	68.4	230	16.51	11.0	even	ok
	231	C44H79N2O9PS	421.2616	4.0	68.7	231	8.84	8.0	even	ok
	232	C41H67N14NaO2S	421.2589	-2.4	69.5	232	13.04	16.0	even	ok
	233	C43H71N8O7P	421.2586	-3.2	69.9	233	8.44	14.0	even	ok
	234	C40H63N18OP	421.2579	-4.8	70.0	234	5.24	20.0	even	ok
	235	C39H74N10O6S2	421.2612	2.8	70.0	235	11.53	9.0	even	ok
	236	C42H70N10O6S	421.2595	-1.2	70.1	236	16.88	14.0	even	ok
	237	C40H62N18O3	421.2621	5.0	70.4	237	4.87	20.0	even	ok
	238	C42H68N12NaO3P	421.2581	-4.5	70.6	238	5.70	16.0	even	ok
	239	C45H76N2NaO9P	421.2588	-2.9	70.7	239	8.94	10.0	even	ok
	240	C44H75N4NaO8S	421.2596	-0.8	71.5	240	17.11	10.0	even	ok
	241	C41H79N4NaO8S2	421.2613	3.2	71.9	241	9.86	5.0	even	ok
	242	C45H78O12S	421.2602	0.4	72.4	242	17.76	8.0	even	ok
	243	C42H82O12S2	421.2618	4.4	73.8	243	6.37	3.0	even	ok
	244	C42H80N6O3P2S2	421.2599	-0.2	76.7	244	15.70	8.0	even	ok
	245	C45H76N6O3P2S	421.2582	-4.2	77.7	245	5.88	13.0	even	ok
	246	C44H72N10NaOPS	421.2618	4.3	78.2	246	5.58	15.0	even	ok
	247	C44H85NaO5P2S2	421.2600	0.1	78.5	247	14.81	4.0	even	ok
	248	C47H81NaO5P2S	421.2583	-3.9	79.0	248	6.15	9.0	even	ok
	249	C43H68N14P2	421.2608	1.9	79.1	249	8.06	19.0	even	ok
	250	C40H70N14O2S2	421.2618	4.4	79.2	250	5.17	14.0	even	ok
	251	C46H76N4O6P2	421.2615	3.5	79.3	251	5.35	13.0	even	ok
	252	C42H76N8NaO2PS2	421.2578	-5.0	79.4	252	4.24	10.0	even	ok
	253	C45H73N8NaO2P2	421.2609	2.3	79.8	253	7.27	15.0	even	ok
	254	C43H66N14O2S	421.2601	0.4	80.4	254	13.03	19.0	even	ok
	255	C43H79N4O6PS2	421.2584	-3.8	80.9	255	5.89	8.0	even	ok
	256	C42H75N8NaO4S2	421.2620	4.8	80.9	256	4.33	10.0	even	ok
	257	C45H71N8NaO4S	421.2603	0.8	81.7	257	11.60	15.0	even	ok
	258	C44H67N12O3P	421.2593	-1.6	81.7	258	7.80	19.0	even	ok
	259	C47H75N2O9P	421.2600	-0.0	82.3	259	10.38	13.0	even	ok
	260	C46H74N4O8S	421.2608	2.0	82.5	260	8.62	13.0	even	ok
	261	C46H72N6NaO5P	421.2594	-1.3	82.6	261	8.09	15.0	even	ok
	262	C48H74O12	421.2585	-3.6	85.4	262	4.12	13.0	even	ok
	263	C47H71N4NaO8	421.2579	-4.8	85.5	263	2.80	15.0	even	ok
	264	C45H81N4NaOP2S2	421.2607	1.7	87.9	264	7.35	9.0	even	ok
	265	C48H77N4NaOP2S	421.2590	-2.3	89.4	265	6.06	14.0	even	ok
	266	C46H84O5P2S2	421.2612	3.0	89.4	266	5.08	7.0	even	ok
	267	C49H80O5P2S	421.2595	-1.0	90.0	267	7.74	12.0	even	ok
	268	C44H75N8O2PS2	421.2591	-2.2	90.3	268	5.97	13.0	even	ok
	269	C46H67N12NaS	421.2610	2.3	92.1	269	5.29	20.0	even	ok
	270	C46H80N2NaO4PS2	421.2592	-1.8	92.1	270	5.95	9.0	even	ok
	271	C47H70N8O4S	421.2615	3.6	92.7	271	3.69	18.0	even	ok
	272	C48H71N6O5P	421.2606	1.6	94.1	272	4.60	18.0	even	ok

Compound Spectrum SmartFormula Report

Meas. m/z	#	Ion Formula	m/z	err [ppm]	mSigma	# mSigma	Score	rdb	e⁻ Conf	N-Rule
	273	C49H75N2NaO6S	421.2616	3.9	94.2	273	3.15	14.0	even	ok
	274	C47H68N10NaOP	421.2601	0.3	94.5	274	5.80	20.0	even	ok
	275	C50H76NaO7P	421.2608	1.9	95.2	275	4.08	14.0	even	ok
	276	C46H62N14O2	421.2585	-3.6	96.5	276	2.52	24.0	even	ok
	277	C49H70N4O8	421.2591	-2.0	97.3	277	3.64	18.0	even	ok
	278	C48H67N8NaO4	421.2586	-3.2	97.5	278	2.64	20.0	even	ok
	279	C47H80N4OP2S2	421.2619	4.6	99.0	279	2.08	12.0	even	ok
443.2732	1	C38H75N14O6PS	443.2721	-2.6	8.7	1	66.70	10.0	even	ok
	2	C40H80N8NaO8PS	443.2722	-2.3	9.0	2	72.08	6.0	even	ok
	3	C39H81N10NaO5P2S	443.2737	1.1	9.1	3	95.18	6.0	even	ok
	4	C40H84N6O9P2S	443.2742	2.3	9.3	4	71.73	4.0	even	ok
	5	C37H72N18NaO2PS	443.2715	-3.8	9.5	5	46.43	12.0	even	ok
	6	C42H89NaO11P2S	443.2744	2.6	9.8	6	65.44	0.0	even	ok
	7	C37H76N16O3P2S	443.2736	0.8	10.0	7	100.00	10.0	even	ok
	8	C41H83N4O12PS	443.2727	-1.1	10.5	8	92.80	4.0	even	ok
	9	C35H67N24PS	443.2714	-4.1	11.4	9	32.47	16.0	even	ok
	10	C41H80N10O5P2S	443.2749	3.8	12.0	10	44.57	9.0	even	ok
	11	C40H77N14NaOP2S	443.2744	2.6	12.2	11	62.74	11.0	even	ok
	12	C43H85N4NaO7P2S	443.2750	4.1	13.0	12	39.52	5.0	even	ok
	13	C42H82N2O15S	443.2712	-4.5	13.3	13	35.08	4.0	even	ok
	14	C36H75N14NaO8S	443.2750	4.0	13.3	14	41.06	7.0	even	ok
	15	C39H71N18O2PS	443.2727	-1.1	13.4	15	87.56	15.0	even	ok
	16	C39H84N4NaO12PS	443.2715	-3.8	13.5	16	43.21	1.0	even	ok
	17	C37H79N10O10PS	443.2714	-4.1	13.9	17	38.80	5.0	even	ok
	18	C41H76N12NaO4PS	443.2729	-0.8	14.1	18	92.12	11.0	even	ok
	19	C34H70N20O6S	443.2748	3.7	14.3	19	44.42	11.0	even	ok
	20	C43H82O18	443.2745	2.9	14.8	20	43.87	4.0	even	ok
	21	C40H87O16PS	443.2721	-2.6	14.9	21	59.52	-1.0	even	ok
	22	C42H79N8O8PS	443.2734	0.4	14.9	22	97.38	9.0	even	ok
	23	C38H85N6NaO9P2S	443.2730	-0.4	15.4	23	96.27	1.0	even	ok
	24	C39H88N2O13P2S	443.2736	0.8	15.8	24	89.17	-1.0	even	ok
	25	C40H70N16O5S	443.2712	-4.5	15.9	25	33.19	15.0	even	ok
	26	C36H80N12O7P2S	443.2729	-0.7	16.4	26	88.73	5.0	even	ok
	27	C44H84N2NaO10PS	443.2736	0.7	16.4	27	88.78	5.0	even	ok
	28	C33H67N24NaO2S	443.2743	2.5	16.9	28	47.37	13.0	even	ok
	29	C42H75N10NaO7S	443.2714	-4.2	17.1	29	35.89	11.0	even	ok
	30	C32H71N24PS2	443.2731	-0.3	17.3	30	94.53	11.0	even	ok
	31	C44H78N4O14	443.2752	4.4	17.9	31	26.00	9.0	even	ok
	32	C42H79N4NaO14	443.2740	1.7	18.3	32	55.57	6.0	even	ok
	33	C34H76N18NaO2PS2	443.2732	-0.0	18.5	33	97.60	7.0	even	ok
	34	C43H78N6O11S	443.2719	-3.0	18.5	34	50.10	9.0	even	ok
	35	C31H62N30S	443.2742	2.1	18.8	35	49.33	17.0	even	ok
	36	C33H70N22O3S2	443.2716	-3.7	19.1	36	39.81	11.0	even	ok
	37	C35H77N16NaO3P2S	443.2724	-1.9	19.1	37	51.44	7.0	even	ok
	38	C44H79N4O12P	443.2711	-4.9	19.3	38	21.43	9.0	even	ok
	39	C45H85NaO11P2	443.2727	-1.2	19.8	39	60.07	5.0	even	ok

Compound Spectrum SmartFormula Report

Meas. m/z	#	Ion Formula	m/z	err [ppm]	mSigma	# mSigma	Score	rdb	e⁻ Conf	N-Rule
	40	C36H75N18O2PS2	443.2744	2.7	19.9	40	52.13	10.0	even	ok
	41	C34H80N16O3P2S2	443.2752	4.6	19.9	41	29.51	5.0	even	ok
	42	C45H83NaO13S	443.2721	-2.6	20.3	42	52.56	5.0	even	ok
	43	C36H71N20NaOS2	443.2724	-1.9	20.5	43	63.57	12.0	even	ok
	44	C40H74N10O12	443.2738	1.4	20.5	44	56.96	10.0	even	ok
	45	C35H75N16NaO5S2	443.2717	-3.4	20.7	45	42.28	7.0	even	ok
	46	C33H72N22OP2S	443.2722	-2.3	20.9	46	45.75	11.0	even	ok
	47	C42H84N2O13P2	443.2719	-3.0	21.0	47	37.25	4.0	even	ok
	48	C43H75N8NaO10	443.2746	3.2	21.1	48	35.32	11.0	even	ok
	49	C36H85N10NaO5P2S2	443.2754	4.9	21.1	49	25.78	1.0	even	ok
	50	C43H80N6O9P2	443.2726	-1.5	21.6	50	53.94	9.0	even	ok
	51	C35H79N14O6PS2	443.2738	1.2	21.6	51	72.29	5.0	even	ok
	52	C38H80N12NaO4PS2	443.2746	3.0	22.0	52	45.68	6.0	even	ok
	53	C41H83NaO18	443.2733	0.2	22.2	53	69.91	1.0	even	ok
	54	C35H79N10NaO12S	443.2743	2.5	22.2	54	52.76	2.0	even	ok
	55	C38H87NaO18S	443.2750	4.0	22.3	55	33.93	-4.0	even	ok
	56	C41H80N6NaO11P	443.2755	5.1	22.4	56	18.80	6.0	even	ok
	57	C36H82N6O16S	443.2749	3.7	22.6	57	37.16	0.0	even	ok
	58	C30H72N24NaPS2	443.2719	-3.0	22.7	58	44.75	8.0	even	ok
	59	C41H70N14O8	443.2745	2.9	22.7	59	37.33	15.0	even	ok
	60	C33H74N16O10S	443.2742	2.2	23.1	60	56.03	6.0	even	ok
	61	C37H84N8NaO8PS2	443.2739	1.5	23.2	61	65.12	1.0	even	ok
	62	C42H72N16NaPS	443.2735	0.7	23.3	62	76.85	16.0	even	ok
	63	C39H84N8O6P2S2	443.2710	-5.1	23.3	63	22.97	4.0	even	ok
	64	C32H71N20NaO6S	443.2736	0.9	23.6	64	72.94	8.0	even	ok
	65	C43H75N12O4PS	443.2741	1.9	23.6	65	58.47	14.0	even	ok
	66	C37H74N16O5S2	443.2729	-0.7	23.8	66	76.90	10.0	even	ok
	67	C47H84O11P2	443.2739	1.5	24.0	67	51.18	8.0	even	ok
	68	C36H78N12O9S2	443.2723	-2.2	24.1	68	54.42	5.0	even	ok
	69	C39H71N14NaO8	443.2733	0.2	24.2	69	66.87	12.0	even	ok
	70	C39H78N6O16	443.2732	-0.1	24.4	70	67.23	5.0	even	ok
	71	C31H75N20O4PS2	443.2724	-1.8	24.6	71	58.57	6.0	even	ok
	72	C41H81N6NaO9P2	443.2713	-4.2	24.7	72	23.83	6.0	even	ok
	73	C37H89N2NaO13P2S	443.2724	-1.9	24.7	73	57.03	-4.0	even	ok
	74	C39H75N12O9P	443.2753	4.8	24.7	74	19.94	10.0	even	ok
	75	C45H80N6NaO6PS	443.2742	2.2	25.0	75	52.46	10.0	even	ok
	76	C30H76N22OP2S2	443.2739	1.5	25.2	76	61.90	6.0	even	ok
	77	C33H80N14NaO6PS2	443.2726	-1.5	25.2	77	62.21	2.0	even	ok
	78	C41H66N20OS	443.2719	-3.0	25.2	78	43.18	20.0	even	ok
	79	C32H81N16NaO3P2S2	443.2740	1.9	25.3	79	57.30	2.0	even	ok
	80	C42H77N10NaO5P2	443.2720	-2.7	25.4	80	36.75	11.0	even	ok
	81	C39H83N8O8PS2	443.2751	4.2	25.4	81	29.32	4.0	even	ok
	82	C41H89N2NaO8P2S2	443.2711	-4.8	25.5	82	24.47	0.0	even	ok
	83	C39H76N16NaPS2	443.2752	4.5	25.6	83	26.40	11.0	even	ok
	84	C35H84N8O11P2S	443.2722	-2.3	25.6	84	51.68	0.0	even	ok
	85	C34H79N12NaO9S2	443.2711	-4.9	25.8	85	23.16	2.0	even	ok

Compound Spectrum SmartFormula Report

Meas. m/z	#	Ion Formula	m/z	err [ppm]	mSigma	# mSigma	Score	rdb	e¯ Conf	N-Rule
	86	C38H83N6NaO11S2	443.2724	-1.9	26.0	86	56.38	1.0	even	ok
	87	C39H79N10NaO7S2	443.2731	-0.4	26.0	87	77.74	6.0	even	ok
	88	C34H81N12NaO7P2S	443.2717	-3.5	26.0	88	36.77	2.0	even	ok
	89	C46H83N2O10PS	443.2748	3.5	26.2	89	36.64	8.0	even	ok
	90	C40H67N18NaO4	443.2740	1.7	26.3	90	46.64	17.0	even	ok
	91	C43H71N14NaO3S	443.2721	-2.6	26.3	91	45.81	16.0	even	ok
	92	C40H80N12O2P2S2	443.2716	-3.6	26.4	92	35.27	9.0	even	ok
	93	C47H80N2NaO10P	443.2719	-3.1	26.5	93	32.53	10.0	even	ok
	94	C37H66N20O6	443.2732	-0.1	26.6	94	63.79	16.0	even	ok
	95	C46H81N4NaO7P2	443.2734	0.3	26.6	95	61.80	10.0	even	ok
	96	C38H87N4O12PS2	443.2744	2.7	26.6	96	44.68	-1.0	even	ok
	97	C40H84N2NaO15P	443.2748	3.6	26.8	97	27.98	1.0	even	ok
	98	C39H76N12O7P2	443.2712	-4.5	27.0	98	20.31	10.0	even	ok
	99	C45H75N8O8P	443.2717	-3.4	27.1	99	29.35	14.0	even	ok
	100	C44H74N10O7S	443.2726	-1.4	27.2	100	60.39	14.0	even	ok
	101	C38H75N10NaO12	443.2726	-1.3	27.4	101	49.27	7.0	even	ok
	102	C40H72N16O3P2	443.2719	-3.0	27.4	102	32.16	15.0	even	ok
	103	C33H84N12O7P2S2	443.2746	3.1	27.4	103	39.90	0.0	even	ok
	104	C41H88N2NaO10PS2	443.2752	4.5	27.6	104	25.10	0.0	even	ok
	105	C44H76N10O5P2	443.2732	-0.0	27.6	105	63.80	14.0	even	ok
	106	C34H83N10O10PS2	443.2731	-0.3	27.7	106	75.19	0.0	even	ok
	107	C30H66N26O4S	443.2735	0.6	27.7	107	56.56	12.0	even	ok
	108	C38H70N20OS2	443.2736	0.8	27.9	108	67.58	15.0	even	ok
	109	C35H89N6NaO9P2S2	443.2747	3.4	28.0	109	35.91	-4.0	even	ok
	110	C38H62N24O2	443.2738	1.4	28.2	110	47.99	21.0	even	ok
	111	C38H72N16NaO5P	443.2748	3.5	28.5	111	26.98	12.0	even	ok
	112	C42H85N6NaO4P2S2	443.2718	-3.2	28.6	112	36.73	5.0	even	ok
	113	C36H88N4NaO12PS2	443.2732	0.0	28.7	113	77.75	-4.0	even	ok
	114	C46H79N4NaO9S	443.2727	-1.1	28.8	114	62.34	10.0	even	ok
	115	C35H82N8O13S2	443.2716	-3.7	28.8	115	32.13	0.0	even	ok
	116	C44H71N12NaO6	443.2753	4.7	28.8	116	18.38	16.0	even	ok
	117	C38H79N8O13P	443.2747	3.2	29.1	117	29.10	5.0	even	ok
	118	C29H63N30NaS	443.2730	-0.6	29.3	118	55.19	14.0	even	ok
	119	C39H68N20NaOP	443.2755	5.1	29.3	119	16.11	17.0	even	ok
	120	C40H82N6O11S2	443.2736	0.9	29.4	120	64.98	4.0	even	ok
	121	C39H86N2O15S2	443.2729	-0.7	29.5	121	67.41	-1.0	even	ok
	122	C42H66N18O4	443.2752	4.4	29.6	122	20.06	20.0	even	ok
	123	C36H70N16O10	443.2725	-1.6	29.7	123	43.37	11.0	even	ok
	124	C44H72N12NaO4P	443.2712	-4.6	29.8	124	18.76	16.0	even	ok
	125	C37H87N2NaO15S2	443.2717	-3.4	30.1	125	34.25	-4.0	even	ok
	126	C40H75N14NaO3S2	443.2737	1.2	30.1	126	60.00	11.0	even	ok
	127	C31H72N22NaO3PS	443.2751	4.3	30.1	127	20.32	8.0	even	ok
	128	C47H82O13S	443.2733	0.1	30.3	128	73.80	8.0	even	ok
	129	C32H76N18O5P2S	443.2716	-3.8	30.4	129	24.10	6.0	even	ok
	130	C36H63N24NaO2	443.2726	-1.3	30.5	130	45.62	18.0	even	ok
	131	C36H92N2O13P2S2	443.2753	4.6	30.6	131	23.01	-6.0	even	ok

Compound Spectrum SmartFormula Report

Meas. m/z	#	Ion Formula	m/z	err [ppm]	mSigma	# mSigma	Score	rdb	e¯ Conf	N-Rule
	132	C42H67N18O2P	443.2711	-4.9	30.8	132	16.41	20.0	even	ok
	133	C43H73N14NaOP2	443.2727	-1.2	30.8	133	46.59	16.0	even	ok
	134	C36H67N22O3P	443.2747	3.2	30.9	134	27.94	16.0	even	ok
	135	C26H67N30NaS2	443.2747	3.2	31.1	135	34.77	9.0	even	ok
	136	C37H91O16PS2	443.2738	1.2	31.7	136	57.10	-6.0	even	ok
	137	C42H87NaO13S2	443.2737	1.2	31.7	137	57.58	0.0	even	ok
	138	C31H73N22NaOP2S	443.2710	-5.0	31.7	138	15.61	8.0	even	ok
	139	C43H88N2O8P2S2	443.2723	-2.0	31.7	139	46.99	3.0	even	ok
	140	C29H67N28OPS	443.2750	4.0	31.9	140	21.55	12.0	even	ok
	141	C34H83N6NaO16S	443.2736	1.0	32.0	141	59.63	-3.0	even	ok
	142	C29H76N20NaO4PS2	443.2712	-4.5	32.1	142	22.43	3.0	even	ok
	143	C37H76N12NaO9P	443.2741	2.0	32.1	143	37.25	7.0	even	ok
	144	C38H82N2O20	443.2725	-1.6	32.2	144	40.97	0.0	even	ok
	145	C29H75N20NaO6S2	443.2753	4.8	32.3	145	20.84	3.0	even	ok
	146	C27H70N26O4S2	443.2752	4.4	32.3	146	23.19	7.0	even	ok
	147	C27H71N26O2PS2	443.2711	-4.9	32.3	147	20.02	7.0	even	ok
	148	C35H86N2O20S	443.2742	2.2	32.5	148	44.73	-5.0	even	ok
	149	C41H63N22Na	443.2746	3.2	32.7	149	27.04	22.0	even	ok
	150	C34H58N30	443.2725	-1.7	32.9	150	40.04	22.0	even	ok
	151	C32H78N12O14S	443.2735	0.6	32.9	151	62.25	1.0	even	ok
	152	C31H75N16NaO10S	443.2730	-0.6	33.0	152	63.00	3.0	even	ok
	153	C41H78N10O7S2	443.2743	2.4	33.1	153	41.99	9.0	even	ok
	154	C35H67N20NaO6	443.2720	-2.9	33.1	154	29.45	13.0	even	ok
	155	C44H71N16PS	443.2747	3.4	33.5	155	30.79	19.0	even	ok
	156	C30H79N16O8PS2	443.2717	-3.3	33.7	156	31.56	1.0	even	ok
	157	C28H77N22NaOP2S2	443.2727	-1.2	33.7	157	54.64	3.0	even	ok
	158	C32H84N10NaO10PS2	443.2719	-3.0	33.9	158	34.37	-3.0	even	ok
	159	C48H80N4O7P2	443.2746	3.0	34.0	159	27.46	13.0	even	ok
	160	C29H70N22O8S	443.2728	-0.9	34.2	160	57.41	7.0	even	ok
	161	C37H79N6NaO16	443.2720	-2.8	34.3	161	28.65	2.0	even	ok
	162	C47H80N6O4P2S	443.2713	-4.3	34.3	162	22.76	13.0	even	ok
	163	C35H71N18O7P	443.2740	1.7	34.5	163	37.82	11.0	even	ok
	164	C33H84N8NaO13PS	443.2751	4.3	34.7	164	22.56	-3.0	even	ok
	165	C31H85N12NaO7P2S2	443.2734	0.3	34.7	165	62.83	-3.0	even	ok
	166	C46H76N10NaO2PS	443.2749	3.8	34.8	166	27.12	15.0	even	ok
	167	C29H80N18O5P2S2	443.2732	0.0	34.8	167	66.42	1.0	even	ok
	168	C43H81N10NaP2S2	443.2725	-1.7	35.0	168	46.56	10.0	even	ok
	169	C43H83N4NaO9S2	443.2744	2.7	35.3	169	36.46	5.0	even	ok
	170	C49H79N2O10P	443.2731	-0.4	35.4	170	49.32	13.0	even	ok
	171	C34H88N4O15P2S	443.2716	-3.8	35.5	171	26.60	-5.0	even	ok
	172	C33H62N26O4	443.2718	-3.2	35.5	172	25.33	17.0	even	ok
	173	C33H85N8NaO11P2S	443.2710	-5.0	35.6	173	17.77	-3.0	even	ok
	174	C47H79N6O6PS	443.2754	5.0	35.6	174	17.79	13.0	even	ok
	175	C31H79N14O11PS	443.2750	4.0	35.8	175	24.33	1.0	even	ok
	176	C49H85NaO6P2S	443.2714	-4.0	35.8	176	24.29	9.0	even	ok
	177	C33H87N6O14PS2	443.2724	-1.8	36.0	177	44.51	-5.0	even	ok

Compound Spectrum SmartFormula Report

Meas. m/z	#	Ion Formula	m/z	err [ppm]	mSigma	# mSigma	Score	rdb	e¯ Conf	N-Rule
	178	C47H77N8NaO3P2	443.2740	1.8	36.0	178	35.61	15.0	even	ok
	179	C35H74N12O14	443.2718	-3.2	36.4	179	24.82	6.0	even	ok
	180	C32H88N8O11P2S2	443.2739	1.6	36.4	180	46.82	-5.0	even	ok
	181	C45H72N14OP2	443.2739	1.5	36.5	181	37.86	19.0	even	ok
	182	C35H92NaO16PS2	443.2726	-1.5	36.5	182	47.21	-9.0	even	ok
	183	C37H83N4O17P	443.2740	1.7	36.7	183	35.67	0.0	even	ok
	184	C34H93N2NaO13P2S2	443.2741	1.9	36.7	184	43.18	-9.0	even	ok
	185	C48H76N6NaO6P	443.2725	-1.6	36.9	185	36.95	15.0	even	ok
	186	C45H70N14O3S	443.2733	0.1	36.9	186	62.50	19.0	even	ok
	187	C46H71N12O4P	443.2724	-1.9	37.0	187	34.25	19.0	even	ok
	188	C44H84N6O4P2S2	443.2730	-0.5	37.5	188	56.38	8.0	even	ok
	189	C34H68N22NaO3P	443.2735	0.5	37.8	189	44.84	13.0	even	ok
	190	C47H75N8NaO5S	443.2734	0.4	38.4	190	56.77	15.0	even	ok
	191	C44H86O13S2	443.2749	3.9	38.5	191	23.64	3.0	even	ok
	192	C49H75N4NaO9	443.2710	-4.9	38.5	192	13.31	15.0	even	ok
	193	C36H80N8NaO13P	443.2735	0.5	38.9	193	43.45	2.0	even	ok
	194	C34H71N16NaO10	443.2713	-4.4	39.0	194	15.97	8.0	even	ok
	195	C28H67N26NaO4S	443.2723	-2.1	39.0	195	30.84	9.0	even	ok
	196	C32H59N30Na	443.2713	-4.4	39.0	196	15.89	19.0	even	ok
	197	C45H68N16NaP	443.2719	-3.1	39.1	197	23.62	21.0	even	ok
	198	C34H72N20O4P2	443.2755	5.1	39.3	198	12.26	11.0	even	ok
	199	C42H74N14O3S2	443.2749	3.9	39.4	199	23.14	14.0	even	ok
	200	C48H78N4O9S	443.2739	1.6	39.5	200	42.82	13.0	even	ok
	201	C46H89NaO6P2S2	443.2731	-0.2	39.7	201	56.38	4.0	even	ok
	202	C32H63N28OP	443.2733	0.2	40.3	202	44.55	17.0	even	ok
	203	C30H76N18NaO7PS	443.2745	2.8	40.4	203	24.55	3.0	even	ok
	204	C26H62N32O2S	443.2722	-2.4	40.7	204	27.22	13.0	even	ok
	205	C34H75N14O11P	443.2733	0.2	41.1	205	43.45	6.0	even	ok
	206	C25H71N26NaO4S2	443.2740	1.7	41.2	206	39.58	4.0	even	ok
	207	C32H66N22O8	443.2712	-4.7	41.2	207	13.49	12.0	even	ok
	208	C44H79N8NaO5S2	443.2751	4.2	41.6	208	19.70	10.0	even	ok
	209	C23H66N32O2S2	443.2738	1.4	41.7	209	41.97	8.0	even	ok
	210	C28H71N24O5PS	443.2743	2.5	41.9	210	25.61	7.0	even	ok
	211	C28H79N16NaO10S2	443.2747	3.2	42.1	211	25.97	-2.0	even	ok
	212	C33H87N2NaO20S	443.2730	-0.5	42.1	212	49.59	-8.0	even	ok
	213	C45H83N4O7PS2	443.2715	-3.9	42.2	213	21.17	8.0	even	ok
	214	C26H74N22O8S2	443.2745	2.9	42.2	214	28.26	2.0	even	ok
	215	C33H69N24NaP2	443.2749	3.9	42.7	215	16.79	13.0	even	ok
	216	C30H79N12NaO14S	443.2723	-2.1	42.9	216	34.75	-2.0	even	ok
	217	C31H82N8O18S	443.2728	-0.9	42.9	217	45.55	-4.0	even	ok
	218	C27H68N28NaOPS	443.2738	1.3	43.1	218	33.08	9.0	even	ok
	219	C36H83N2NaO20	443.2713	-4.4	43.2	219	14.28	-3.0	even	ok
	220	C24H72N28NaOPS2	443.2755	5.1	43.3	220	13.74	4.0	even	ok
	221	C29H83N12O12PS2	443.2711	-4.9	43.4	221	14.94	-4.0	even	ok
	222	C31H88N6NaO14PS2	443.2712	-4.5	43.4	222	16.66	-8.0	even	ok
	223	C29H82N12O14S2	443.2752	4.4	43.6	223	17.08	-4.0	even	ok

Compound Spectrum SmartFormula Report

Meas. m/z	#	Ion Formula	m/z	err [ppm]	mSigma	# mSigma	Score	rdb	e¯ Conf	N-Rule
	224	C35H81N10NaO10P2	443.2750	3.9	43.6	224	16.27	2.0	even	ok
	225	C31H87N6NaO16S2	443.2753	4.8	43.7	225	15.29	-8.0	even	ok
	226	C33H72N18NaO7P	443.2728	-1.0	43.8	226	34.66	8.0	even	ok
	227	C27H81N18NaO5P2S2	443.2720	-2.7	43.8	227	28.88	-2.0	even	ok
	228	C28H74N18O12S	443.2722	-2.4	43.9	228	31.15	2.0	even	ok
	229	C48H76N10P2S	443.2720	-2.8	44.4	229	27.28	18.0	even	ok
	230	C30H89N8NaO11P2S2	443.2727	-1.2	44.6	230	40.72	-8.0	even	ok
	231	C32H88N4NaO17PS	443.2745	2.8	44.7	231	27.09	-8.0	even	ok
	232	C28H84N14O9P2S2	443.2726	-1.5	44.9	232	37.76	-4.0	even	ok
	233	C49H76N8O3P2	443.2752	4.5	44.9	233	12.80	18.0	even	ok
	234	C33H91O21PS	443.2750	4.0	44.9	234	18.84	-10.0	even	ok
	235	C34H78N8O18	443.2712	-4.7	45.0	235	12.17	1.0	even	ok
	236	C51H81N2NaO5P2	443.2754	4.8	45.2	236	11.39	14.0	even	ok
	237	C32H91N2O18PS2	443.2717	-3.3	45.2	237	23.16	-10.0	even	ok
	238	C45H80N10P2S2	443.2737	1.0	45.2	238	41.68	13.0	even	ok
	239	C30H83N10O15PS	443.2743	2.5	45.7	239	28.69	-4.0	even	ok
	240	C50H81N4NaO2P2S	443.2721	-2.5	45.8	240	28.55	14.0	even	ok
	241	C33H76N16O8P2	443.2748	3.6	45.8	241	16.83	6.0	even	ok
	242	C36H87O21P	443.2733	0.2	45.9	242	37.85	-5.0	even	ok
	243	C29H80N14NaO11PS	443.2738	1.3	45.9	243	38.06	-2.0	even	ok
	244	C31H67N24O5P	443.2726	-1.3	46.0	244	30.33	12.0	even	ok
	245	C31H92N4O15P2S2	443.2732	0.0	46.1	245	48.60	-10.0	even	ok
	246	C51H84O6P2S	443.2726	-1.3	46.7	246	37.14	12.0	even	ok
	247	C50H75N6O6P	443.2737	1.2	46.8	247	30.64	18.0	even	ok
	248	C47H85N4NaO2P2S2	443.2738	1.3	47.3	248	36.66	9.0	even	ok
	249	C52H80NaO8P	443.2739	1.5	47.3	249	28.12	14.0	even	ok
	250	C35H84N4NaO17P	443.2728	-1.0	47.5	250	31.12	-3.0	even	ok
	251	C47H67N16P	443.2731	-0.4	47.8	251	34.89	24.0	even	ok
	252	C49H72N10NaO2P	443.2732	-0.0	48.0	252	36.77	20.0	even	ok
	253	C48H71N12NaOS	443.2741	1.9	48.5	253	30.84	20.0	even	ok
	254	C32H73N20NaO4P2	443.2743	2.4	48.5	254	21.77	8.0	even	ok
	255	C30H64N28NaOP	443.2721	-2.5	49.0	255	20.81	14.0	even	ok
	256	C51H74N4O9	443.2722	-2.2	49.2	256	22.28	18.0	even	ok
	257	C49H74N8O5S	443.2746	3.1	49.3	257	21.92	18.0	even	ok
	258	C33H79N10O15P	443.2727	-1.3	49.5	258	27.45	1.0	even	ok
	259	C48H66N14O3	443.2716	-3.7	49.5	259	14.43	24.0	even	ok
	260	C27H71N22NaO8S	443.2716	-3.6	49.5	260	15.09	4.0	even	ok
	261	C48H88O6P2S2	443.2743	2.5	49.6	261	25.58	7.0	even	ok
	262	C46H79N8O3PS2	443.2722	-2.4	49.8	262	26.07	13.0	even	ok
	263	C50H71N8NaO5	443.2717	-3.4	50.0	263	15.65	20.0	even	ok
	264	C35H88N2O18P2	443.2748	3.6	50.2	264	14.74	-5.0	even	ok
	265	C30H68N26O2P2	443.2741	2.1	50.8	265	21.98	12.0	even	ok
	266	C51H79N2NaO7S	443.2747	3.4	50.9	266	19.06	14.0	even	ok
	267	C25H66N28O6S	443.2715	-3.9	51.0	267	13.09	8.0	even	ok
	268	C32H76N14NaO11P	443.2721	-2.5	51.5	268	19.30	3.0	even	ok
	269	C48H84N2NaO5PS2	443.2723	-2.1	51.9	269	26.45	9.0	even	ok

Compound Spectrum SmartFormula Report

Meas. m/z	#	Ion Formula	m/z	err [ppm]	mSigma	# mSigma	Score	rdb	e⁻ Conf	N-Rule
	270	C34H85N6NaO14P2	443.2743	2.4	52.0	270	19.53	-3.0	even	ok
	271	C27H75N20O9PS	443.2737	1.0	52.5	271	26.65	2.0	even	ok
	272	C29H83N8NaO18S	443.2716	-3.6	52.9	272	17.05	-7.0	even	ok
	273	C30H86N4O22S	443.2722	-2.4	53.1	273	23.75	-9.0	even	ok
	274	C26H72N24NaO5PS	443.2731	-0.2	53.3	274	30.26	4.0	even	ok
	275	C30H71N20O9P	443.2720	-2.8	53.6	275	16.62	7.0	even	ok
	276	C27H78N14O16S	443.2715	-3.9	53.9	276	15.01	-3.0	even	ok
	277	C32H80N12O12P2	443.2741	2.1	54.0	277	19.87	1.0	even	ok
	278	C26H75N18NaO12S	443.2710	-5.1	54.1	278	9.92	-1.0	even	ok
	279	C28H81N16NaO8P2S	443.2753	4.7	54.8	279	8.99	-2.0	even	ok
	280	C31H92NaO21PS	443.2738	1.3	54.8	280	28.87	-13.0	even	ok
	281	C24H67N30O3PS	443.2730	-0.5	54.9	281	27.13	8.0	even	ok
	282	C29H87N6O19PS	443.2737	1.0	55.7	282	30.04	-9.0	even	ok
	283	C28H84N10NaO15PS	443.2731	-0.2	55.8	283	35.06	-7.0	even	ok
	284	C29H68N24NaO5P	443.2714	-4.0	56.0	284	10.79	9.0	even	ok
	285	C31H77N16NaO8P2	443.2736	0.9	56.1	285	24.34	3.0	even	ok
	286	C27H62N30O5	443.2754	5.0	56.1	286	7.80	13.0	even	ok
	287	C26H76N22O6P2S	443.2752	4.4	56.3	287	9.55	2.0	even	ok
	288	C52H80N4O2P2S	443.2733	0.2	56.9	288	33.85	17.0	even	ok
	289	C34H88NaO21P	443.2721	-2.5	57.1	289	16.19	-8.0	even	ok
	290	C25H73N26NaO2P2S	443.2746	3.2	57.3	290	13.41	4.0	even	ok
	291	C49H84N4O2P2S2	443.2750	4.0	58.0	291	12.68	12.0	even	ok
	292	C27H63N30O3P	443.2713	-4.3	58.1	292	9.07	13.0	even	ok
	293	C29H72N22O6P2	443.2735	0.6	58.2	293	24.19	7.0	even	ok
	294	C51H71N10O2P	443.2744	2.7	58.4	294	14.82	23.0	even	ok
	295	C54H79O8P	443.2751	4.2	58.5	295	9.40	17.0	even	ok
	296	C26H59N34NaO	443.2749	3.8	58.7	296	10.70	15.0	even	ok
	297	C32H83N6O19P	443.2720	-2.8	58.9	297	14.06	-4.0	even	ok
	298	C23H68N32P2S	443.2745	2.8	58.9	298	13.87	8.0	even	ok
	299	C53H76N4NaO4P	443.2745	3.0	59.0	299	13.30	19.0	even	ok
	300	C50H70N12OS	443.2753	4.6	59.6	300	9.87	23.0	even	ok
	301	C52H76N6NaOPS	443.2713	-4.4	59.8	301	10.57	19.0	even	ok
	302	C31H80N10NaO15P	443.2714	-4.0	60.4	302	9.34	-2.0	even	ok
	303	C49H80N6NaOPS2	443.2730	-0.6	60.5	303	27.89	14.0	even	ok
	304	C29H74N16O15	443.2754	5.0	60.6	304	6.69	2.0	even	ok
	305	C28H69N26NaO2P2	443.2729	-0.6	60.7	305	21.93	9.0	even	ok
	306	C53H79N2O5PS	443.2718	-3.2	60.7	306	14.86	17.0	even	ok
	307	C52H70N8O5	443.2729	-0.7	61.0	307	21.44	23.0	even	ok
	308	C52H75N6NaO3S	443.2754	4.9	61.1	308	8.41	19.0	even	ok
	309	C33H89N2NaO18P2	443.2736	0.9	61.3	309	20.43	-8.0	even	ok
	310	C51H67N12NaO	443.2724	-1.9	61.6	310	16.09	25.0	even	ok
	311	C54H75N2NaO7	443.2730	-0.4	61.8	311	22.14	19.0	even	ok
	312	C29H75N16O13P	443.2713	-4.3	62.3	312	7.91	2.0	even	ok
	313	C28H71N20NaO11	443.2749	3.8	62.4	313	9.40	4.0	even	ok
	314	C50H83N2O5PS2	443.2735	0.6	62.7	314	25.68	12.0	even	ok
	315	C26H64N32P2	443.2728	-1.0	62.8	315	19.08	13.0	even	ok

Compound Spectrum SmartFormula Report

Meas. m/z	#	Ion Formula	m/z	err [ppm]	mSigma	# mSigma	Score	rdb	e¯ Conf	N-Rule
	316	C31H84N8O16P2	443.2735	0.6	63.1	316	20.46	-4.0	even	ok
	317	C48H74N10O2S2	443.2713	-4.3	63.1	317	9.79	18.0	even	ok
	318	C26H66N26O9	443.2748	3.5	64.4	318	9.66	8.0	even	ok
	319	C30H81N12NaO12P2	443.2729	-0.6	64.8	319	19.05	-2.0	even	ok
	320	C50H79N4NaO4S2	443.2715	-3.9	65.2	320	10.09	14.0	even	ok
	321	C25H63N30NaO5	443.2742	2.2	66.5	321	12.48	10.0	even	ok
	322	C28H76N18O10P2	443.2728	-0.9	66.7	322	16.69	2.0	even	ok
	323	C51H82O8S2	443.2720	-2.7	67.4	323	13.27	12.0	even	ok
	324	C31H86N2O25	443.2754	5.0	67.5	324	5.24	-9.0	even	ok
	325	C23H58N36O3	443.2741	1.9	68.5	325	12.52	14.0	even	ok
	326	C30H83N6NaO21	443.2749	3.8	68.5	326	7.53	-7.0	even	ok
	327	C27H73N22NaO6P2	443.2723	-2.2	68.7	327	11.80	4.0	even	ok
	328	C55H81N2NaP2S	443.2741	2.0	68.7	328	15.20	18.0	even	ok
	329	C31H87N2O23P	443.2713	-4.3	68.8	329	6.29	-9.0	even	ok
	330	C55H76N4O2P2	443.2716	-3.6	69.7	330	7.61	22.0	even	ok
	331	C28H78N12O19	443.2748	3.5	70.2	331	7.79	-3.0	even	ok
	332	C50H78O13	443.2716	-3.7	70.5	332	8.92	13.0	even	ok
	333	C25H68N28O4P2	443.2721	-2.5	70.7	333	10.10	8.0	even	ok
	334	C54H72N8NaP	443.2752	4.5	70.8	334	5.50	24.0	even	ok
	335	C54H75N6OPS	443.2725	-1.7	71.1	335	15.19	22.0	even	ok
	336	C27H75N16NaO15	443.2742	2.3	71.6	336	10.29	-1.0	even	ok
	337	C51H79N6OPS2	443.2742	2.1	71.7	337	13.24	17.0	even	ok
	338	C56H80NaO3PS	443.2726	-1.3	72.6	338	15.39	18.0	even	ok
	339	C30H88N4O20P2	443.2728	-0.9	72.8	339	13.35	-9.0	even	ok
	340	C53H66N12O	443.2736	0.8	72.8	340	13.71	28.0	even	ok
	341	C24H65N32NaP2	443.2716	-3.7	72.9	341	6.60	10.0	even	ok
	342	C25H70N22O13	443.2741	1.9	73.4	342	10.40	3.0	even	ok
	343	C53H84NaO3PS2	443.2743	2.5	73.7	343	11.31	13.0	even	ok
	344	C55H71N6NaO3	443.2737	1.1	73.7	344	12.41	24.0	even	ok
	345	C29H85N8NaO16P2	443.2723	-2.1	74.1	345	9.64	-7.0	even	ok
	346	C51H75N8NaS2	443.2721	-2.4	74.4	346	11.07	19.0	even	ok
	347	C55H74N4O4S	443.2710	-5.0	75.0	347	4.85	22.0	even	ok
	348	C24H67N26NaO9	443.2736	0.7	75.1	348	12.71	5.0	even	ok
	349	C27H80N14O14P2	443.2721	-2.5	75.9	349	8.31	-3.0	even	ok
	350	C52H78N4O4S2	443.2727	-1.2	76.3	350	13.67	17.0	even	ok
	351	C22H62N32O7	443.2734	0.4	77.0	351	12.55	9.0	even	ok
	352	C26H77N18NaO10P2	443.2716	-3.7	77.5	352	5.55	-1.0	even	ok
	353	C29H87N2NaO25	443.2742	2.3	78.7	353	7.79	-12.0	even	ok
	354	C21H59N36NaO3	443.2729	-0.8	79.0	354	10.84	11.0	even	ok
	355	C24H72N24O8P2	443.2715	-4.0	79.4	355	4.67	3.0	even	ok
	356	C23H68N28NaO6P	443.2750	4.1	79.5	356	4.45	5.0	even	ok
	357	C57H80N2P2S	443.2753	4.7	80.1	357	4.40	21.0	even	ok
	358	C19H54N42O	443.2727	-1.1	81.0	358	9.37	15.0	even	ok
	359	C21H63N34O4P	443.2749	3.8	81.4	359	4.55	9.0	even	ok
	360	C58H77N2NaP2	443.2724	-1.8	82.6	360	7.50	23.0	even	ok
	361	C55H84OP2S2	443.2714	-4.1	83.3	361	4.74	16.0	even	ok

Compound Spectrum SmartFormula Report

Meas. m/z	#	Ion Formula	m/z	err [ppm]	mSigma	# mSigma	Score	rdb	e⁻ Conf	N-Rule
	362	C20H60N38NaP	443.2744	2.6	83.4	362	5.92	11.0	even	ok
	363	C58H79O3PS	443.2738	1.4	84.1	363	9.69	21.0	even	ok
	364	C56H70N8S	443.2717	-3.5	85.5	364	5.21	27.0	even	ok
	365	C57H70N6O3	443.2749	3.8	85.6	365	3.78	27.0	even	ok
	366	C53H74N8S2	443.2733	0.3	85.8	366	11.25	22.0	even	ok
	367	C58H75N2NaO2S	443.2718	-3.2	87.1	367	5.34	23.0	even	ok
	368	C55H79N2NaO2S2	443.2735	0.6	87.8	368	9.72	18.0	even	ok
	369	C56H74N2O7	443.2743	2.3	90.1	369	5.95	22.0	even	ok
	370	C60H76N2P2	443.2736	0.9	94.6	370	5.38	26.0	even	ok
	371	C60H74N2O2S	443.2730	-0.5	98.8	371	6.04	26.0	even	ok
	372	C59H75NaO5	443.2751	4.1	99.0	372	2.35	23.0	even	ok
	373	C57H78N2O2S2	443.2747	3.3	99.5	373	2.97	21.0	even	ok
465.2862	1	C28H66N32O3S	465.2853	-2.0	3.8	1	58.83	13.0	even	ok
	2	C30H71N26NaO5S	465.2854	-1.7	4.3	2	62.99	9.0	even	ok
	3	C29H72N28NaO2PS	465.2869	1.5	4.4	3	66.58	9.0	even	ok
	4	C30H75N24O6PS	465.2874	2.6	4.7	4	61.25	7.0	even	ok
	5	C32H80N18NaO8PS	465.2876	2.9	5.8	5	55.12	3.0	even	ok
	6	C27H67N34PS	465.2868	1.2	6.3	6	69.18	13.0	even	ok
	7	C38H87N2NaO21	465.2844	-3.9	6.3	7	32.36	-3.0	even	ok
	8	C38H91O22P	465.2864	0.5	6.7	8	80.10	-5.0	even	ok
	9	C31H74N22O9S	465.2859	-0.6	7.0	9	97.43	7.0	even	ok
	10	C33H84N14O10P2S	465.2840	-4.8	7.1	10	29.10	1.0	even	ok
	11	C36H82N8O19	465.2843	-4.2	8.3	11	28.14	1.0	even	ok
	12	C33H83N14O12PS	465.2881	4.1	8.8	12	36.14	1.0	even	ok
	13	C35H89N8NaO12P2S	465.2841	-4.5	8.8	13	31.54	-3.0	even	ok
	14	C33H79N16NaO11S	465.2861	-0.3	8.9	14	100.00	3.0	even	ok
	15	C29H75N22NaO9S	465.2847	-3.2	10.0	15	47.79	4.0	even	ok
	16	C37H88N4NaO18P	465.2859	-0.7	10.1	16	72.27	-3.0	even	ok
	17	C35H88N8NaO14PS	465.2883	4.4	10.6	17	31.41	-3.0	even	ok
	18	C31H72N28P2S	465.2840	-4.8	10.7	18	21.71	12.0	even	ok
	19	C27H70N28O7S	465.2846	-3.5	11.1	19	34.09	8.0	even	ok
	20	C37H92N2O19P2	465.2879	3.7	11.1	20	31.62	-5.0	even	ok
	21	C31H71N28O2PS	465.2881	4.1	11.2	21	27.74	12.0	even	ok
	22	C34H80N18O6P2S	465.2847	-3.3	11.3	22	44.16	6.0	even	ok
	23	C33H77N22NaO2P2S	465.2841	-4.5	11.4	23	23.93	8.0	even	ok
	24	C39H87N4O18P	465.2871	1.9	11.4	24	52.87	0.0	even	ok
	25	C26H67N32NaO3S	465.2841	-4.6	11.4	25	22.82	10.0	even	ok
	26	C33H76N22NaO4PS	465.2882	4.4	11.5	26	30.99	8.0	even	ok
	27	C40H86N2O21	465.2856	-1.3	11.7	27	76.33	0.0	even	ok
	28	C30H78N18O13S	465.2853	-2.0	11.7	28	63.95	2.0	even	ok
	29	C36H92N4O16P2S	465.2847	-3.3	12.2	29	43.60	-5.0	even	ok
	30	C34H82N12O15S	465.2866	0.9	12.2	30	83.22	1.0	even	ok
	31	C35H83N10O16P	465.2858	-1.0	12.4	31	64.79	1.0	even	ok
	32	C32H83N12NaO15S	465.2854	-1.7	12.5	32	68.09	-2.0	even	ok
	33	C31H84N14NaO12PS	465.2869	1.5	12.5	33	71.61	-2.0	even	ok
	34	C29H79N20O10PS	465.2868	1.2	12.6	34	76.75	2.0	even	ok

Compound Spectrum SmartFormula Report

Meas. m/z	#	Ion Formula	m/z	err [ppm]	mSigma	# mSigma	Score	rdb	e¯ Conf	N-Rule
	35	C36H85N12NaO8P2S	465.2848	-3.0	13.0	35	46.93	2.0	even	ok
	36	C28H76N24NaO6PS	465.2862	0.1	13.1	36	77.24	4.0	even	ok
	37	C24H62N38OS	465.2839	-4.9	13.1	37	19.72	14.0	even	ok
	38	C32H70N26O5S	465.2866	0.9	13.6	38	81.13	12.0	even	ok
	39	C30H85N16NaO9P2S	465.2884	4.7	14.0	39	26.08	-2.0	even	ok
	40	C39H83N6NaO17	465.2851	-2.4	14.2	40	43.41	2.0	even	ok
	41	C38H84N8NaO14P	465.2866	0.8	14.3	41	65.41	2.0	even	ok
	42	C36H87N6NaO17S	465.2868	1.2	14.3	42	74.71	-3.0	even	ok
	43	C31H67N30NaOS	465.2861	-0.3	14.3	43	72.04	14.0	even	ok
	44	C26H71N30O4PS	465.2861	-0.2	14.3	44	72.59	8.0	even	ok
	45	C36H89N6NaO15P2	465.2874	2.5	14.6	45	42.00	-3.0	even	ok
	46	C32H87N10O16PS	465.2874	2.6	14.6	46	50.83	-4.0	even	ok
	47	C37H78N12O15	465.2849	-2.8	14.7	47	39.48	6.0	even	ok
	48	C37H88N8O12P2S	465.2853	-1.9	15.1	48	61.94	0.0	even	ok
	49	C34H75N20NaO7S	465.2868	1.2	15.2	49	73.63	8.0	even	ok
	50	C33H86N8O19S	465.2859	-0.6	15.4	50	83.37	-4.0	even	ok
	51	C34H92N4NaO18PS	465.2876	2.9	15.4	51	45.85	-8.0	even	ok
	52	C36H79N14O12P	465.2864	0.5	15.6	52	67.88	6.0	even	ok
	53	C25H68N34NaPS	465.2856	-1.4	15.6	53	55.28	10.0	even	ok
	54	C37H85N10NaO11P2	465.2881	4.0	16.0	54	26.10	2.0	even	ok
	55	C28H80N22O7P2S	465.2883	4.4	16.2	55	22.32	2.0	even	ok
	56	C34H80N14NaO12P	465.2852	-2.1	16.3	56	45.47	3.0	even	ok
	57	C36H92NaO22P	465.2852	-2.1	16.5	57	45.39	-8.0	even	ok
	58	C27H77N26NaO3P2S	465.2877	3.3	16.7	58	32.48	4.0	even	ok
	59	C35H91N2NaO21S	465.2861	-0.3	16.7	59	86.19	-8.0	even	ok
	60	C35H78N16O11S	465.2873	2.3	16.9	60	53.36	6.0	even	ok
	61	C34H84N12O13P2	465.2873	2.2	17.0	61	43.44	1.0	even	ok
	62	C39H93N2NaO14P2S	465.2855	-1.6	17.1	62	64.03	-4.0	even	ok
	63	C35H96O20P2S	465.2840	-4.8	17.2	63	24.07	-10.0	even	ok
	64	C36H75N16NaO11	465.2844	-3.9	17.6	64	25.93	8.0	even	ok
	65	C37H90N2O21S	465.2873	2.3	17.8	65	52.25	-5.0	even	ok
	66	C26H77N28NaP2S2	465.2845	-3.7	17.8	66	34.00	4.0	even	ok
	67	C28H76N28P2S2	465.2857	-1.2	17.8	67	69.76	7.0	even	ok
	68	C35H80N16O9P2	465.2879	3.7	17.8	68	27.81	6.0	even	ok
	69	C24H71N34PS2	465.2884	4.8	17.9	69	23.27	8.0	even	ok
	70	C25H72N32OP2S	465.2876	3.0	18.3	70	34.41	8.0	even	ok
	71	C35H95O22PS	465.2881	4.1	18.4	71	29.82	-10.0	even	ok
	72	C34H87N6O20P	465.2851	-2.4	18.5	72	40.22	-4.0	even	ok
	73	C32H75N20O10P	465.2851	-2.4	18.7	73	39.89	7.0	even	ok
	74	C34H70N22O9	465.2843	-4.2	18.9	74	22.78	12.0	even	ok
	75	C37H83N10NaO13S	465.2874	2.6	18.9	75	47.12	2.0	even	ok
	76	C35H76N18NaO8P	465.2859	-0.7	19.2	76	60.12	8.0	even	ok
	77	C25H70N32O3S2	465.2870	1.6	19.8	77	60.34	8.0	even	ok
	78	C28H79N18NaO13S	465.2841	-4.6	19.9	78	24.15	-1.0	even	ok
	79	C30H81N22NaO2P2S2	465.2858	-0.9	19.9	79	71.33	3.0	even	ok
	80	C28H71N30NaOS2	465.2878	3.3	20.2	80	36.89	9.0	even	ok

Compound Spectrum SmartFormula Report

Meas. m/z	#	Ion Formula	m/z	err [ppm]	mSigma	# mSigma	Score	rdb	e⁻ Conf	N-Rule
	81	C26H74N24O11S	465.2839	-4.9	20.4	81	21.36	3.0	even	ok
	82	C33H81N16NaO9P2	465.2867	1.1	20.7	82	53.30	3.0	even	ok
	83	C35H76N22O2P2S	465.2853	-1.9	20.8	83	54.87	11.0	even	ok
	84	C33H84N10NaO16P	465.2846	-3.6	20.8	84	27.14	-2.0	even	ok
	85	C31H78N16O16	465.2885	5.0	20.9	85	16.25	2.0	even	ok
	86	C33H71N24O6P	465.2858	-1.0	21.0	86	54.31	12.0	even	ok
	87	C35H93N2NaO19P2	465.2867	1.1	21.0	87	52.88	-8.0	even	ok
	88	C27H80N24O4P2S2	465.2850	-2.6	21.1	88	45.11	2.0	even	ok
	89	C29H82N14O17S	465.2846	-3.5	21.2	89	34.81	-3.0	even	ok
	90	C31H87N8NaO19S	465.2847	-3.2	21.3	90	38.13	-7.0	even	ok
	91	C38H86N6O17S	465.2880	3.8	21.4	91	31.38	0.0	even	ok
	92	C27H75N26NaO5S2	465.2871	1.9	21.5	92	53.97	4.0	even	ok
	93	C40H92NaO17PS	465.2840	-4.8	21.5	93	21.64	-4.0	even	ok
	94	C29H75N26O3PS2	465.2842	-4.4	21.6	94	25.25	7.0	even	ok
	95	C34H77N20NaO5P2	465.2874	2.5	21.8	95	36.20	8.0	even	ok
	96	C37H81N16NaO4P2S	465.2855	-1.6	22.0	96	57.53	7.0	even	ok
	97	C27H80N20NaO10PS	465.2856	-1.4	22.1	97	60.50	-1.0	even	ok
	98	C40H83N8O14P	465.2878	3.3	22.2	98	28.15	5.0	even	ok
	99	C42H88N2NaO16P	465.2879	3.6	22.4	99	25.43	1.0	even	ok
	100	C30H88N10NaO16PS	465.2862	0.1	22.4	100	79.32	-7.0	even	ok
	101	C43H87NaO19	465.2864	0.4	22.5	101	73.73	1.0	even	ok
	102	C29H85N18NaO6P2S2	465.2851	-2.3	22.6	102	47.44	-2.0	even	ok
	103	C31H72N24NaO6P	465.2845	-3.6	22.7	103	25.96	9.0	even	ok
	104	C29H66N30O6	465.2885	5.0	22.7	104	15.71	13.0	even	ok
	105	C28H83N16O14PS	465.2861	-0.2	22.7	105	76.26	-3.0	even	ok
	106	C33H88N8O17P2	465.2866	0.8	23.0	106	54.01	-4.0	even	ok
	107	C31H79N16O14P	465.2844	-3.9	23.0	107	23.41	2.0	even	ok
	108	C38H84N12O8P2S	465.2860	-0.4	23.1	108	72.52	5.0	even	ok
	109	C31H76N22O7P2	465.2866	0.8	23.2	109	53.97	7.0	even	ok
	110	C33H66N30OS	465.2873	2.3	23.4	110	46.59	17.0	even	ok
	111	C32H90N4O23S	465.2853	-2.0	23.5	111	50.30	-9.0	even	ok
	112	C40H91NaO19S	465.2881	4.1	23.5	112	27.03	-4.0	even	ok
	113	C31H84N18O6P2S2	465.2863	0.3	23.6	113	74.08	1.0	even	ok
	114	C30H75N20NaO12	465.2880	3.9	23.8	114	23.10	4.0	even	ok
	115	C29H74N26O5S2	465.2883	4.5	23.8	115	23.15	7.0	even	ok
	116	C31H80N20NaO5PS2	465.2843	-4.1	23.8	116	26.73	3.0	even	ok
	117	C32H72N26O3P2	465.2872	2.2	23.9	117	37.58	12.0	even	ok
	118	C31H91N6O20PS	465.2868	1.2	24.0	118	60.32	-9.0	even	ok
	119	C39H80N12NaO10P	465.2872	2.2	24.1	119	37.64	7.0	even	ok
	120	C41H82N6O17	465.2863	0.1	24.1	120	60.27	5.0	even	ok
	121	C33H96NaO22PS	465.2869	1.5	24.2	121	55.91	-13.0	even	ok
	122	C37H75N18O8P	465.2871	1.9	24.6	122	40.32	11.0	even	ok
	123	C35H71N24NaO3S	465.2874	2.6	24.6	123	41.70	13.0	even	ok
	124	C40H89N6NaO10P2S	465.2861	-0.1	24.8	124	74.04	1.0	even	ok
	125	C32H68N28NaO2P	465.2852	-2.1	24.9	125	37.64	14.0	even	ok
	126	C29H67N30O4P	465.2844	-3.9	25.1	126	22.27	13.0	even	ok

Compound Spectrum SmartFormula Report

Meas. m/z	#	Ion Formula	m/z	err [ppm]	mSigma	# mSigma	Score	rdb	e¯ Conf	N-Rule
	127	C28H78N22O9S2	465.2876	3.0	25.1	127	36.17	2.0	even	ok
	128	C38H74N16O11	465.2856	-1.3	25.2	128	45.83	11.0	even	ok
	129	C40H79N10NaO13	465.2857	-1.0	25.3	129	49.00	7.0	even	ok
	130	C25H75N26O8PS	465.2854	-1.7	25.4	130	41.74	3.0	even	ok
	131	C32H85N12NaO13P2	465.2861	-0.3	25.5	131	56.10	-2.0	even	ok
	132	C36H74N20O7S	465.2880	3.7	25.5	132	28.70	11.0	even	ok
	133	C32H80N22O2P2S2	465.2870	1.7	25.7	133	51.31	6.0	even	ok
	134	C33H89N12NaO8P2S2	465.2865	0.6	25.7	134	66.43	-3.0	even	ok
	135	C38H80N14NaO7PS	465.2840	-4.8	25.7	135	19.61	7.0	even	ok
	136	C24H72N30NaO4PS	465.2849	-2.8	25.8	136	30.37	5.0	even	ok
	137	C30H76N20NaO10P	465.2839	-5.0	26.0	137	14.47	4.0	even	ok
	138	C31H79N20NaO7S2	465.2884	4.8	26.0	138	19.70	3.0	even	ok
	139	C28H70N26O10	465.2879	3.5	26.1	139	24.18	8.0	even	ok
	140	C30H88N14O10P2S2	465.2857	-1.1	26.1	140	58.30	-4.0	even	ok
	141	C41H92N2O14P2S	465.2867	1.0	26.6	141	59.60	-1.0	even	ok
	142	C28H63N34NaO2	465.2880	3.8	26.7	142	21.67	15.0	even	ok
	143	C30H63N34P	465.2851	-2.4	26.9	143	33.18	18.0	even	ok
	144	C30H83N16NaO11S2	465.2878	3.3	26.9	144	31.61	-2.0	even	ok
	145	C21H63N40NaS	465.2883	4.6	27.0	145	16.64	11.0	even	ok
	146	C33H90N2O26	465.2885	5.0	27.0	146	14.12	-9.0	even	ok
	147	C30H73N26NaO3P2	465.2860	-0.4	27.0	147	54.04	9.0	even	ok
	148	C32H76N24NaOPS2	465.2850	-2.6	27.1	148	38.93	8.0	even	ok
	149	C39H83N10O11PS	465.2845	-3.7	27.1	149	28.43	5.0	even	ok
	150	C38H79N14NaO9S	465.2881	4.0	27.2	150	24.97	7.0	even	ok
	151	C36H72N22NaO4P	465.2866	0.8	27.3	151	49.45	13.0	even	ok
	152	C37H71N20NaO7	465.2851	-2.5	27.4	152	32.54	13.0	even	ok
	153	C32H83N16O9PS2	465.2848	-2.9	27.4	153	35.56	1.0	even	ok
	154	C30H80N18O11P2	465.2859	-0.6	27.8	154	50.08	2.0	even	ok
	155	C32H93N8NaO12P2S2	465.2858	-0.8	27.8	155	59.90	-8.0	even	ok
	156	C35H66N26O5	465.2849	-2.8	27.9	156	29.51	17.0	even	ok
	157	C32H87N6NaO22	465.2880	3.9	27.9	157	20.90	-7.0	even	ok
	158	C34H85N16NaO4P2S2	465.2872	2.0	28.0	158	45.09	2.0	even	ok
	159	C35H73N24NaOP2	465.2881	4.0	28.2	159	20.04	13.0	even	ok
	160	C33H91N2O24P	465.2844	-3.8	28.2	160	20.84	-9.0	even	ok
	161	C34H67N28O2P	465.2864	0.5	28.3	161	51.44	17.0	even	ok
	162	C31H87N12O13PS2	465.2842	-4.4	28.7	162	21.54	-4.0	even	ok
	163	C41H88N4NaO13PS	465.2846	-3.4	29.0	163	29.99	1.0	even	ok
	164	C28H64N34NaP	465.2839	-5.0	29.1	164	13.39	15.0	even	ok
	165	C26H58N40	465.2879	3.5	29.1	165	22.60	19.0	even	ok
	166	C27H67N30NaO6	465.2873	2.4	29.3	166	31.58	10.0	even	ok
	167	C34H92N8O12P2S2	465.2870	1.7	29.4	167	46.92	-5.0	even	ok
	168	C28H68N32OP2	465.2859	-0.7	29.5	168	47.97	13.0	even	ok
	169	C32H88N6NaO20P	465.2839	-5.0	29.5	169	13.38	-7.0	even	ok
	170	C34H88N10NaO11PS2	465.2850	-2.6	29.6	170	36.82	-3.0	even	ok
	171	C30H82N12O20	465.2879	3.6	29.7	171	22.14	-3.0	even	ok
	172	C33H79N20O5PS2	465.2855	-1.5	30.2	172	48.92	6.0	even	ok

Compound Spectrum SmartFormula Report

Meas. m/z	#	Ion Formula	m/z	err [ppm]	mSigma	# mSigma	Score	rdb	e¯ Conf	N-Rule
	173	C33H92N6NaO15PS2	465.2843	-4.1	30.6	173	22.88	-8.0	even	ok
	174	C31H86N12O15S2	465.2883	4.5	30.6	174	19.62	-4.0	even	ok
	175	C34H63N30NaO	465.2844	-3.9	30.7	175	19.22	19.0	even	ok
	176	C29H77N22NaO7P2	465.2854	-1.8	30.7	176	35.81	4.0	even	ok
	177	C42H91O17PS	465.2852	-2.2	31.0	177	39.92	-1.0	even	ok
	178	C35H88N12O8P2S2	465.2877	3.2	31.2	178	30.11	0.0	even	ok
	179	C29H79N16NaO16	465.2873	2.4	31.4	179	29.94	-1.0	even	ok
	180	C33H96N4O16P2S2	465.2864	0.3	31.4	180	61.44	-10.0	even	ok
	181	C36H97N2NaO14P2S2	465.2872	2.0	31.5	181	41.22	-9.0	even	ok
	182	C25H62N36O4	465.2872	2.1	31.7	182	32.29	14.0	even	ok
	183	C29H87N12NaO15S2	465.2871	1.9	31.8	183	42.36	-7.0	even	ok
	184	C38H77N20NaP2S	465.2861	-0.2	32.1	184	62.06	12.0	even	ok
	185	C32H92N4O21P2	465.2859	-0.6	32.3	185	45.01	-9.0	even	ok
	186	C35H84N14NaO7PS2	465.2857	-1.2	32.4	186	49.64	2.0	even	ok
	187	C33H91N6NaO17S2	465.2884	4.8	32.5	187	16.75	-8.0	even	ok
	188	C39H80N16O4P2S	465.2867	1.0	32.7	188	51.54	10.0	even	ok
	189	C45H86O19	465.2876	3.0	32.8	189	30.32	4.0	even	ok
	190	C44H88N2O14P2	465.2850	-2.6	32.8	190	27.21	4.0	even	ok
	191	C27H72N28O5P2	465.2852	-2.1	33.1	191	31.26	8.0	even	ok
	192	C35H91N6O15PS2	465.2855	-1.5	33.2	192	45.46	-5.0	even	ok
	193	C27H74N22O14	465.2872	2.1	33.4	193	30.89	3.0	even	ok
	194	C37H93N6NaO10P2S2	465.2878	3.5	33.5	194	25.89	-4.0	even	ok
	195	C41H80N12O8P2	465.2843	-4.1	33.6	195	16.92	10.0	even	ok
	196	C41H79N12O10P	465.2884	4.8	33.7	196	13.06	10.0	even	ok
	197	C35H81N20NaP2S2	465.2878	3.5	33.7	197	25.83	7.0	even	ok
	198	C31H89N8NaO17P2	465.2854	-1.8	33.8	198	33.30	-7.0	even	ok
	199	C43H85N6NaO10P2	465.2845	-3.8	33.9	199	18.62	6.0	even	ok
	200	C43H84N6NaO12P	465.2886	5.1	34.1	200	11.51	6.0	even	ok
	201	C41H85N10NaO6P2S	465.2868	1.3	34.2	201	46.41	6.0	even	ok
	202	C37H71N24OPS	465.2845	-3.7	34.2	202	23.82	16.0	even	ok
	203	C34H95N2O19PS2	465.2849	-2.9	34.3	203	30.13	-10.0	even	ok
	204	C34H78N18O8S2	465.2840	-4.7	34.7	204	16.42	6.0	even	ok
	205	C30H90N8O19S2	465.2876	3.1	35.1	205	28.24	-9.0	even	ok
	206	C24H59N40Na	465.2867	1.0	35.2	206	39.03	16.0	even	ok
	207	C40H76N16NaO6P	465.2879	3.6	35.2	207	18.81	12.0	even	ok
	208	C38H71N22O4P	465.2878	3.3	35.2	208	20.68	16.0	even	ok
	209	C42H88N6O10P2S	465.2873	2.4	35.4	209	33.60	4.0	even	ok
	210	C37H96NaO17PS2	465.2857	-1.2	35.4	210	46.08	-9.0	even	ok
	211	C39H76N18NaO3PS	465.2846	-3.4	35.5	211	25.37	12.0	even	ok
	212	C36H87N10O11PS2	465.2862	-0.0	35.6	212	58.04	0.0	even	ok
	213	C26H71N26NaO10	465.2867	1.0	35.6	213	38.47	5.0	even	ok
	214	C29H84N14O15P2	465.2852	-2.1	35.7	214	29.35	-3.0	even	ok
	215	C42H78N10O13	465.2869	1.6	35.9	215	33.18	10.0	even	ok
	216	C34H75N24OPS2	465.2862	-0.1	36.1	216	57.20	11.0	even	ok
	217	C36H84N16O4P2S2	465.2884	4.6	36.3	217	16.28	5.0	even	ok
	218	C26H69N32NaOP2	465.2847	-3.2	36.3	218	20.69	10.0	even	ok

Compound Spectrum SmartFormula Report

Meas. m/z	#	Ion Formula	m/z	err [ppm]	mSigma	# mSigma	Score	rdb	e⁻ Conf	N-Rule
	219	C40H79N14O7PS	465.2852	-2.2	36.4	219	34.62	10.0	even	ok
	220	C39H70N20O7	465.2863	0.1	36.6	220	44.58	16.0	even	ok
	221	C32H95N2NaO21S2	465.2878	3.4	36.6	221	24.76	-13.0	even	ok
	222	C44H83N4NaO15	465.2871	1.9	36.7	222	30.19	6.0	even	ok
	223	C38H96N2O14P2S2	465.2884	4.6	36.8	223	15.99	-6.0	even	ok
	224	C41H75N14NaO9	465.2864	0.4	36.9	224	41.72	12.0	even	ok
	225	C36H83N12NaO10S2	465.2842	-4.4	36.9	225	17.28	2.0	even	ok
	226	C44H93NaO12P2S	465.2875	2.7	37.1	226	29.50	0.0	even	ok
	227	C36H90N4O18S2	465.2840	-4.7	37.3	227	15.41	-5.0	even	ok
	228	C37H68N26NaP	465.2872	2.2	37.4	228	27.25	18.0	even	ok
	229	C28H81N18NaO11P2	465.2847	-3.2	37.7	229	20.02	-1.0	even	ok
	230	C24H66N32O8	465.2865	0.7	37.8	230	38.76	9.0	even	ok
	231	C38H92N4NaO13PS2	465.2863	0.3	37.9	231	52.39	-4.0	even	ok
	232	C42H84N8NaO9PS	465.2853	-1.9	38.0	232	35.91	6.0	even	ok
	233	C34H71N26NaS2	465.2842	-4.4	38.2	233	16.63	13.0	even	ok
	234	C36H80N18NaO3PS2	465.2863	0.2	38.2	234	52.06	7.0	even	ok
	235	C38H67N24NaO3	465.2857	-1.0	38.3	235	35.43	18.0	even	ok
	236	C36H62N30O	465.2856	-1.3	38.4	236	32.95	22.0	even	ok
	237	C31H91N2NaO26	465.2873	2.4	38.4	237	24.87	-12.0	even	ok
	238	C38H89N10NaO6P2S2	465.2885	4.9	38.5	238	13.72	1.0	even	ok
	239	C43H87N4O13PS	465.2858	-0.8	39.5	239	45.23	4.0	even	ok
	240	C26H76N24O9P2	465.2846	-3.5	39.8	240	17.21	3.0	even	ok
	241	C29H86N8O24	465.2872	2.1	39.9	241	25.96	-8.0	even	ok
	242	C37H86N8O14S2	465.2847	-3.3	40.1	242	23.29	0.0	even	ok
	243	C25H72N28NaO7P	465.2882	4.2	40.3	243	13.68	5.0	even	ok
	244	C23H63N36NaO4	465.2860	-0.5	40.5	244	37.42	11.0	even	ok
	245	C35H74N22O4S2	465.2847	-3.3	40.7	245	22.87	11.0	even	ok
	246	C28H83N12NaO20	465.2867	1.0	40.9	246	33.34	-6.0	even	ok
	247	C37H83N14O7PS2	465.2869	1.4	40.9	247	37.84	5.0	even	ok
	248	C39H95O17PS2	465.2869	1.4	41.2	248	37.38	-6.0	even	ok
	249	C25H73N28NaO5P2	465.2840	-4.7	42.3	249	10.79	5.0	even	ok
	250	C39H91N2NaO16S2	465.2848	-3.0	42.4	250	23.95	-4.0	even	ok
	251	C23H67N34O5P	465.2880	3.9	42.5	251	14.27	9.0	even	ok
	252	C26H78N18O18	465.2865	0.7	42.6	252	33.93	-2.0	even	ok
	253	C21H58N42O2	465.2858	-0.8	42.7	253	33.00	15.0	even	ok
	254	C37H79N16NaO6S2	465.2848	-3.0	42.8	254	23.60	7.0	even	ok
	255	C40H76N20P2S	465.2873	2.4	42.9	255	27.51	15.0	even	ok
	256	C39H88N8NaO9PS2	465.2870	1.7	43.1	256	33.08	1.0	even	ok
	257	C46H82N4O15	465.2883	4.5	43.3	257	14.23	9.0	even	ok
	258	C44H86N2O16S	465.2844	-4.0	43.7	258	16.58	4.0	even	ok
	259	C25H75N22NaO14	465.2860	-0.5	44.1	259	33.86	0.0	even	ok
	260	C42H81N14NaO2P2S	465.2875	2.7	44.2	260	24.35	11.0	even	ok
	261	C23H68N34O3P2	465.2839	-5.0	44.5	261	9.05	9.0	even	ok
	262	C45H84N6O10P2	465.2857	-1.2	44.7	262	28.58	9.0	even	ok
	263	C43H84N10O6P2S	465.2880	3.9	45.0	263	16.60	9.0	even	ok
	264	C42H76N16O4P2	465.2850	-2.6	45.2	264	19.44	15.0	even	ok

Compound Spectrum SmartFormula Report

Meas. m/z	#	Ion Formula	m/z	err [ppm]	mSigma	# mSigma	Score	rdb	e¯ Conf	N-Rule
	265	C22H64N38NaOP	465.2875	2.7	45.2	265	18.91	11.0	even	ok
	266	C47H89NaO12P2	465.2858	-0.9	45.4	266	29.89	5.0	even	ok
	267	C38H82N12O10S2	465.2854	-1.8	45.5	267	29.97	5.0	even	ok
	268	C44H81N10NaO6P2	465.2851	-2.3	45.6	268	20.90	11.0	even	ok
	269	C40H91N4O13PS2	465.2875	2.8	46.0	269	22.33	-1.0	even	ok
	270	C41H75N18O3PS	465.2858	-0.8	46.4	270	37.10	15.0	even	ok
	271	C39H67N26P	465.2884	4.8	46.5	271	9.24	21.0	even	ok
	272	C45H89N4NaO8P2S	465.2882	4.2	46.6	272	14.31	5.0	even	ok
	273	C41H72N20NaO2P	465.2886	5.1	46.6	273	8.20	17.0	even	ok
	274	C41H73N20NaP2	465.2845	-3.8	46.6	274	13.06	17.0	even	ok
	275	C46H83N4O13P	465.2842	-4.4	47.5	275	10.30	9.0	even	ok
	276	C40H87N6NaO12S2	465.2855	-1.5	47.7	276	30.24	1.0	even	ok
	277	C43H74N14O9	465.2876	3.0	47.7	277	16.14	15.0	even	ok
	278	C43H80N12NaO5PS	465.2860	-0.5	47.8	278	37.84	11.0	even	ok
	279	C38H79N18O3PS2	465.2875	2.8	48.1	279	21.04	10.0	even	ok
	280	C40H66N24O3	465.2869	1.6	48.2	280	23.60	21.0	even	ok
	281	C36H70N26S2	465.2854	-1.8	48.4	281	27.44	16.0	even	ok
	282	C45H79N8NaO11	465.2878	3.3	48.5	282	14.39	11.0	even	ok
	283	C42H71N18NaO5	465.2871	1.9	48.6	283	21.63	17.0	even	ok
	284	C44H83N8O9PS	465.2865	0.7	48.9	284	35.36	9.0	even	ok
	285	C42H74N16O6S	465.2843	-4.0	50.1	285	13.66	15.0	even	ok
	286	C40H84N12NaO5PS2	465.2877	3.1	50.2	286	18.06	6.0	even	ok
	287	C38H75N20NaO2S2	465.2855	-1.5	50.4	287	27.81	12.0	even	ok
	288	C46H88N2NaO11PS	465.2867	1.0	50.6	288	31.53	5.0	even	ok
	289	C41H90N2O16S2	465.2860	-0.4	50.6	289	35.66	-1.0	even	ok
	290	C41H88N8O7P2S2	465.2841	-4.6	50.6	290	10.99	4.0	even	ok
	291	C44H79N10NaO8S	465.2845	-3.7	51.7	291	14.40	11.0	even	ok
	292	C41H87N8O9PS2	465.2882	4.3	52.6	292	11.48	4.0	even	ok
	293	C39H78N16O6S2	465.2860	-0.4	52.6	293	33.39	10.0	even	ok
	294	C43H93N2NaO9P2S2	465.2842	-4.3	52.7	294	11.46	0.0	even	ok
	295	C45H82N6O12S	465.2850	-2.6	52.9	295	19.65	9.0	even	ok
	296	C47H87NaO14S	465.2852	-2.3	54.7	296	20.20	5.0	even	ok
	297	C43H92N2NaO11PS2	465.2883	4.6	54.7	297	9.62	0.0	even	ok
	298	C41H83N10NaO8S2	465.2862	-0.1	54.7	298	33.10	6.0	even	ok
	299	C46H80N10O6P2	465.2863	0.3	56.6	299	24.17	14.0	even	ok
	300	C43H72N20P2	465.2857	-1.2	56.9	300	19.60	20.0	even	ok
	301	C49H88O12P2	465.2870	1.7	57.0	301	17.27	8.0	even	ok
	302	C42H86N6O12S2	465.2867	1.1	57.2	302	24.91	4.0	even	ok
	303	C48H85N4NaO8P2	465.2865	0.6	57.3	303	22.18	10.0	even	ok
	304	C45H77N14NaO2P2	465.2858	-0.9	57.3	304	20.62	16.0	even	ok
	305	C44H76N16NaOPS	465.2867	0.9	58.0	305	24.91	16.0	even	ok
	306	C41H80N16NaOPS2	465.2883	4.6	58.5	306	8.55	11.0	even	ok
	307	C42H84N12O3P2S2	465.2848	-3.1	58.7	307	13.76	9.0	even	ok
	308	C45H79N12O5PS	465.2872	2.1	58.9	308	18.36	14.0	even	ok
	309	C44H71N18O3P	465.2842	-4.4	59.2	309	7.09	20.0	even	ok
	310	C47H79N8O9P	465.2848	-3.0	59.4	310	11.33	14.0	even	ok

Compound Spectrum SmartFormula Report

Meas. m/z	#	Ion Formula	m/z	err [ppm]	mSigma	# mSigma	Score	rdb	e¯ Conf	N-Rule
	311	C44H91NaO14S2	465.2868	1.4	59.4	311	21.63	0.0	even	ok
	312	C44H70N18O5	465.2883	4.4	59.6	312	6.89	20.0	even	ok
	313	C46H76N12NaO5P	465.2843	-4.1	59.9	313	7.70	16.0	even	ok
	314	C43H70N20O2S	465.2850	-2.6	60.3	314	15.42	20.0	even	ok
	315	C49H84N2NaO11P	465.2850	-2.7	60.4	315	11.97	10.0	even	ok
	316	C47H84N6NaO7PS	465.2873	2.4	60.4	316	16.08	10.0	even	ok
	317	C43H67N22NaO	465.2877	3.3	60.4	317	9.87	22.0	even	ok
	318	C46H75N12NaO7	465.2884	4.7	60.4	318	6.00	16.0	even	ok
	319	C44H89N6NaO5P2S2	465.2849	-2.8	60.7	319	14.07	5.0	even	ok
	320	C40H74N20O2S2	465.2867	1.1	61.0	320	22.05	15.0	even	ok
	321	C48H87N2O11PS	465.2879	3.5	61.6	321	10.96	8.0	even	ok
	322	C45H75N14NaO4S	465.2852	-2.3	61.7	322	15.95	16.0	even	ok
	323	C46H78N10O8S	465.2857	-1.1	62.8	323	20.45	14.0	even	ok
	324	C45H92N2O9P2S2	465.2854	-1.7	62.9	324	17.78	3.0	even	ok
	325	C42H79N14NaO4S2	465.2868	1.4	62.9	325	19.22	11.0	even	ok
	326	C48H83N4NaO10S	465.2858	-0.8	64.4	326	20.64	10.0	even	ok
	327	C51H83N2O11P	465.2862	-0.1	64.8	327	23.65	13.0	even	ok
	328	C43H82N10O8S2	465.2874	2.5	65.1	328	13.28	9.0	even	ok
	329	C49H86O14S	465.2864	0.3	65.7	329	21.74	8.0	even	ok
	330	C45H87N4NaO10S2	465.2875	2.8	67.2	330	11.31	5.0	even	ok
	331	C46H91O12PS2	465.2839	-4.9	67.5	331	5.52	3.0	even	ok
	332	C51H79N4NaO10	465.2841	-4.4	67.7	332	6.51	15.0	even	ok
	333	C52H82O14	465.2847	-3.3	67.9	333	9.50	13.0	even	ok
	334	C47H76N14O2P2	465.2870	1.7	68.5	334	11.65	19.0	even	ok
	335	C50H84N4O8P2	465.2877	3.1	69.0	335	7.66	13.0	even	ok
	336	C46H75N16OPS	465.2879	3.5	69.1	336	8.41	19.0	even	ok
	337	C49H81N8NaO4P2	465.2871	2.0	69.3	337	10.48	15.0	even	ok
	338	C48H81N10NaOP2S	465.2839	-5.0	69.3	338	4.93	15.0	even	ok
	339	C45H85N10NaOP2S2	465.2856	-1.4	69.5	339	15.06	10.0	even	ok
	340	C46H90O14S2	465.2881	4.0	69.5	340	7.23	3.0	even	ok
	341	C49H84N6O5P2S	465.2844	-3.9	70.2	341	7.23	13.0	even	ok
	342	C48H80N10NaO3PS	465.2880	3.8	70.6	342	7.22	15.0	even	ok
	343	C48H75N12O5P	465.2855	-1.5	71.4	343	10.90	19.0	even	ok
	344	C46H88N6O5P2S2	465.2861	-0.3	71.4	344	17.95	8.0	even	ok
	345	C49H83N6O7PS	465.2885	5.0	71.6	345	4.60	13.0	even	ok
	346	C51H89NaO7P2S	465.2845	-3.6	71.8	346	7.53	9.0	even	ok
	347	C47H72N16NaOP	465.2850	-2.7	71.8	347	7.92	21.0	even	ok
	348	C43H75N18NaS2	465.2875	2.8	71.9	348	9.51	16.0	even	ok
	349	C44H79N14O2PS2	465.2839	-4.9	72.0	349	4.66	14.0	even	ok
	350	C46H71N18NaS	465.2858	-0.8	72.1	350	15.56	21.0	even	ok
	351	C50H80N6NaO7P	465.2856	-1.2	72.3	351	11.28	15.0	even	ok
	352	C47H74N14O4S	465.2864	0.3	72.9	352	16.72	19.0	even	ok
	353	C48H93NaO7P2S2	465.2862	0.0	73.4	353	17.28	4.0	even	ok
	354	C44H78N14O4S2	465.2880	3.9	73.8	354	6.17	14.0	even	ok
	355	C46H84N8NaO4PS2	465.2841	-4.6	74.0	355	4.84	10.0	even	ok
	356	C49H79N8NaO6S	465.2865	0.6	74.5	356	14.83	15.0	even	ok

Compound Spectrum SmartFormula Report

Meas. m/z	#	Ion Formula	m/z	err [ppm]	mSigma	# mSigma	Score	rdb	e⁻ Conf	N-Rule
	357	C49H74N10O8	465.2840	-4.7	74.5	357	3.62	19.0	even	ok
	358	C50H82N4O10S	465.2870	1.8	75.6	358	10.91	13.0	even	ok
	359	C46H83N8NaO6S2	465.2882	4.2	75.8	359	5.15	10.0	even	ok
	360	C47H87N4O8PS2	465.2846	-3.5	76.0	360	6.64	8.0	even	ok
	361	C54H84NaO9P	465.2870	1.7	76.5	361	10.80	14.0	even	ok
	362	C53H78N4O10	465.2853	-1.9	78.6	362	9.48	18.0	even	ok
	363	C50H80N10OP2S	465.2851	-2.4	80.6	363	7.51	18.0	even	ok
	364	C47H84N10OP2S2	465.2868	1.2	80.6	364	10.29	13.0	even	ok
	365	C51H80N8O4P2	465.2883	4.6	81.0	365	2.97	18.0	even	ok
	366	C50H77N12NaP2	465.2878	3.4	81.3	366	4.34	20.0	even	ok
	367	C52H85N4NaO3P2S	465.2852	-2.1	82.1	367	7.66	14.0	even	ok
	368	C53H85N2NaO6P2	465.2885	4.9	82.1	368	2.54	14.0	even	ok
	369	C49H89N4NaO3P2S2	465.2869	1.5	82.5	369	8.87	9.0	even	ok
	370	C53H88O7P2S	465.2858	-1.0	83.1	370	9.70	12.0	even	ok
	371	C47H80N12NaPS2	465.2847	-3.2	83.3	371	5.41	15.0	even	ok
	372	C48H70N18S	465.2870	1.8	83.3	372	8.02	24.0	even	ok
	373	C49H71N16OP	465.2862	-0.1	83.4	373	9.21	24.0	even	ok
	374	C52H79N6O7P	465.2868	1.4	84.3	374	6.78	18.0	even	ok
	375	C51H76N10NaO3P	465.2863	0.2	84.4	375	8.64	20.0	even	ok
	376	C50H92O7P2S2	465.2874	2.6	84.4	376	6.06	7.0	even	ok
	377	C50H75N12NaO2S	465.2872	2.1	84.8	377	6.98	20.0	even	ok
	378	C48H83N8O4PS2	465.2853	-2.0	85.0	378	6.96	13.0	even	ok
	379	C51H78N8O6S	465.2877	3.2	85.8	379	4.82	18.0	even	ok
	380	C50H70N14O4	465.2847	-3.3	86.5	380	3.63	24.0	even	ok
	381	C49H67N18Na	465.2841	-4.4	86.7	381	2.44	26.0	even	ok
	382	C50H88N2NaO6PS2	465.2854	-1.7	87.0	382	6.91	9.0	even	ok
	383	C53H83N2NaO8S	465.2878	3.5	87.4	383	4.09	14.0	even	ok
	384	C56H83O9P	465.2882	4.2	87.5	384	3.17	17.0	even	ok
	385	C52H75N8NaO6	465.2848	-3.0	87.7	385	3.79	20.0	even	ok
	386	C54H74N8O6	465.2860	-0.4	89.4	386	8.36	23.0	even	ok
	387	C56H79N2NaO8	465.2862	-0.1	90.6	387	8.43	19.0	even	ok
	388	C51H87NaO9S2	465.2839	-4.9	91.6	388	2.06	9.0	even	ok
	389	C54H84N4O3P2S	465.2864	0.4	93.5	389	6.93	17.0	even	ok
	390	C51H88N4O3P2S2	465.2881	4.1	93.8	390	2.56	12.0	even	ok
	391	C49H79N12PS2	465.2859	-0.6	94.6	391	6.39	18.0	even	ok
	392	C52H75N12PS	465.2843	-4.2	94.9	392	2.32	23.0	even	ok
	393	C52H74N12O2S	465.2884	4.6	96.2	393	1.87	23.0	even	ok
	394	C53H75N10O3P	465.2875	2.8	96.4	394	2.73	23.0	even	ok
	395	C54H80N6NaO2PS	465.2844	-3.9	96.4	395	2.40	19.0	even	ok
	396	C51H84N6NaO2PS2	465.2861	-0.3	96.5	396	6.23	14.0	even	ok
	397	C55H83N2O6PS	465.2849	-2.8	97.5	397	3.28	17.0	even	ok
	398	C55H80N4NaO5P	465.2877	3.1	97.6	398	2.36	19.0	even	ok
	399	C54H79N6NaO4S	465.2885	4.9	97.8	399	1.55	19.0	even	ok
	400	C52H87N2O6PS2	465.2866	0.9	98.3	400	5.10	12.0	even	ok
	401	C51H66N18	465.2853	-1.9	98.7	401	3.16	29.0	even	ok
	402	C50H78N10O3S2	465.2844	-3.8	99.1	402	2.19	18.0	even	ok

Compound Spectrum SmartFormula Report

Meas. m/z	#	Ion Formula	m/z	err [ppm]	mSigma	# mSigma	Score	rdb	e⁻ Conf	N-Rule
	403	C53H71N12NaO2	465.2855	-1.6	99.8	403	3.24	25.0	even	ok
472.2760	1	C16H35N13O2P	472.2769	1.8	16.5	1	83.94	7.0	even	ok
	2	C12H31N19P	472.2742	-3.9	18.5	2	42.07	8.0	even	ok
	3	C14H36N13NaO2P	472.2745	-3.3	19.7	3	50.00	4.0	even	ok
	4	C17H34N11O5	472.2739	-4.6	20.6	4	31.87	7.0	even	ok
	5	C15H39N9O6P	472.2755	-1.1	23.8	5	84.88	2.0	even	ok
	6	C18H30N15O	472.2752	-1.7	24.0	6	71.87	12.0	even	ok
	7	C13H35N15NaOS	472.2762	0.3	35.6	7	100.00	4.0	even	ok
	8	C17H44N7O2P2S	472.2747	-2.9	36.9	8	51.09	1.0	even	ok
	9	C20H45N5NaP2S	472.2763	0.6	38.9	9	87.12	2.0	even	ok
	10	C19H49NNaO4P2S	472.2750	-2.3	39.1	10	57.21	-3.0	even	ok
	11	C14H38N11O5S	472.2773	2.6	39.7	11	51.82	2.0	even	ok
	12	C16H43N5NaO7S	472.2775	3.2	41.7	12	41.10	-2.0	even	ok
	13	C21H48NO4P2S	472.2774	2.8	42.5	13	44.52	0.0	even	ok
	14	C23H43N3O3PS	472.2757	-0.7	49.8	14	62.00	5.0	even	ok
	15	C26H44NNaOPS	472.2773	2.7	57.6	15	28.74	6.0	even	ok
	16	C19H47NNaO6S2	472.2737	-5.0	58.5	16	16.22	-3.0	even	ok
	17	C16H43N9OPS2	472.2764	0.8	59.1	17	45.18	1.0	even	ok
	18	C25H38N5O2S	472.2741	-4.2	60.0	18	16.59	10.0	even	ok
	19	C18H48N3NaO3PS2	472.2767	1.4	61.3	19	36.66	-3.0	even	ok
	20	C21H46NO6S2	472.2761	0.1	62.0	20	58.52	0.0	even	ok
	21	C18H38N11S2	472.2748	-2.7	63.1	21	23.92	6.0	even	ok
	22	C20H43N5NaO2S2	472.2750	-2.1	65.4	22	26.10	2.0	even	ok
	23	C28H39N3NaS	472.2757	-0.8	69.4	23	31.54	11.0	even	ok
	24	C22H42N5O2S2	472.2774	3.0	71.3	24	16.62	5.0	even	ok
	25	C30H38N3S	472.2781	4.3	79.8	25	7.54	14.0	even	ok
476.3076	1	C21H42N5O7	476.3079	0.6	7.5	1	98.45	4.0	even	ok
	2	C20H46NO11	476.3065	-2.2	7.6	2	67.58	-1.0	even	ok
	3	C19H43N5NaO7	476.3055	-4.4	9.8	3	23.61	1.0	even	ok
	4	C20H39N9NaO3	476.3068	-1.6	11.9	4	54.38	6.0	even	ok
	5	C17H38N11O5	476.3052	-5.0	12.2	5	18.00	5.0	even	ok
	6	C19H47N3O8P	476.3095	4.1	12.2	6	25.21	-1.0	even	ok
	7	C20H49N5NaP2S	476.3076	0.1	12.7	7	100.00	0.0	even	ok
	8	C15H38N15OS	476.3099	4.9	12.8	8	24.84	5.0	even	ok
	9	C18H34N15O	476.3065	-2.2	13.6	9	45.08	10.0	even	ok
	10	C19H40N11NaP	476.3098	4.7	14.3	10	19.65	6.0	even	ok
	11	C17H48N7O2P2S	476.3060	-3.3	14.7	11	42.08	-1.0	even	ok
	12	C18H44N7NaO4P	476.3085	1.9	14.7	12	48.17	1.0	even	ok
	13	C19H53NNaO4P2S	476.3063	-2.7	16.1	13	49.11	-5.0	even	ok
	14	C21H52NO4P2S	476.3087	2.3	16.3	14	54.87	-2.0	even	ok
	15	C16H39N13O2P	476.3082	1.3	17.0	15	53.18	5.0	even	ok
	16	C24H48NO4P2	476.3053	-4.8	17.4	16	17.89	3.0	even	ok
	17	C13H39N15NaOS	476.3075	-0.2	19.1	17	86.55	2.0	even	ok
	18	C22H38N9O3	476.3092	3.4	20.8	18	26.62	9.0	even	ok
	19	C24H43N3NaO5	476.3095	4.0	20.8	19	21.76	5.0	even	ok
	20	C14H42N11O5S	476.3086	2.1	21.8	20	52.30	0.0	even	ok

Compound Spectrum SmartFormula Report

Meas. m/z	#	Ion Formula	m/z	err [ppm]	mSigma	# mSigma	Score	rdb	e¯ Conf	N-Rule
	21	C16H47N5NaO7S	476.3088	2.7	22.7	21	43.45	-4.0	even	ok
	22	C17H48N3NaO8P	476.3071	-0.9	24.3	22	49.11	-4.0	even	ok
	23	C23H47N3O3PS	476.3070	-1.1	24.8	23	61.87	3.0	even	ok
	24	C15H43N9O6P	476.3068	-1.5	26.0	24	41.17	0.0	even	ok
	25	C17H50NO11S	476.3099	4.9	26.0	25	18.59	-6.0	even	ok
	26	C14H40N13NaO2P	476.3058	-3.8	27.5	26	20.48	2.0	even	ok
	27	C12H35N19P	476.3055	-4.4	29.5	27	15.82	6.0	even	ok
	28	C25H44N5P2	476.3066	-1.9	30.7	28	33.09	8.0	even	ok
	29	C16H47N9OPS2	476.3077	0.3	34.1	29	59.42	-1.0	even	ok
	30	C26H48NNaOPS	476.3086	2.2	34.9	30	36.53	4.0	even	ok
	31	C18H52N3NaO3PS2	476.3080	0.9	36.2	31	49.77	-5.0	even	ok
	32	C18H42N11S2	476.3061	-3.2	36.6	32	26.51	4.0	even	ok
	33	C25H42N5O2S	476.3054	-4.6	37.2	33	15.76	8.0	even	ok
	34	C21H50NO6S2	476.3074	-0.3	38.2	34	66.01	-2.0	even	ok
	35	C20H47N5NaO2S2	476.3063	-2.6	38.9	35	29.84	0.0	even	ok
	36	C22H46N5O2S2	476.3087	2.5	45.1	36	26.03	3.0	even	ok
	37	C29H44NNaOP	476.3053	-4.8	46.7	37	8.36	9.0	even	ok
	38	C28H43N3NaS	476.3070	-1.2	48.5	38	32.52	9.0	even	ok
	39	C31H43NOP	476.3077	0.2	51.4	39	36.99	12.0	even	ok
	40	C30H42N3S	476.3094	3.8	59.8	40	10.67	12.0	even	ok
	41	C33H38N3	476.3060	-3.2	65.7	41	10.55	17.0	even	ok
516.3026	1	C15H41N15NaP2	516.3037	2.2	18.6	1	74.03	4.0	even	ok
	2	C14H35N19OP	516.3004	-4.2	18.6	2	36.00	8.0	even	ok
	3	C16H40N13NaO3P	516.3007	-3.6	20.4	3	42.96	4.0	even	ok
	4	C18H39N13O3P	516.3031	1.0	20.8	4	96.77	7.0	even	ok
	5	C16H44N11O4P2	516.3047	4.2	22.7	5	32.25	2.0	even	ok
	6	C20H44N7NaO5P	516.3034	1.6	23.3	6	79.34	3.0	even	ok
	7	C17H43N9O7P	516.3018	-1.6	24.8	7	76.68	2.0	even	ok
	8	C19H38N11O6	516.3001	-4.8	25.3	8	24.13	7.0	even	ok
	9	C21H40N11NaOP	516.3047	4.2	27.4	9	29.81	8.0	even	ok
	10	C16H47N5O11P	516.3004	-4.2	30.0	10	37.39	-3.0	even	ok
	11	C20H34N15O2	516.3014	-2.2	30.0	11	56.74	12.0	even	ok
	12	C22H39N9NaO4	516.3017	-1.6	32.4	12	62.76	8.0	even	ok
	13	C13H34N21S	516.3021	-0.8	36.2	13	93.17	8.0	even	ok
	14	C24H38N9O4	516.3041	3.0	37.7	14	47.24	11.0	even	ok
	15	C15H39N15NaO2S	516.3024	-0.3	38.3	15	100.00	4.0	even	ok
	16	C17H38N15O2S	516.3048	4.4	40.5	16	26.23	7.0	even	ok
	17	C19H48N7O3P2S	516.3009	-3.2	40.6	17	40.86	1.0	even	ok
	18	C23H35N13Na	516.3031	1.0	41.3	18	59.36	13.0	even	ok
	19	C16H42N11O6S	516.3035	1.8	42.4	19	61.98	2.0	even	ok
	20	C19H43N9NaO4S	516.3051	4.9	42.8	20	19.47	3.0	even	ok
	21	C21H53NNaO5P2S	516.3012	-2.7	42.8	21	46.29	-3.0	even	ok
	22	C22H49N5NaOP2S	516.3025	-0.1	43.7	22	90.39	2.0	even	ok
	23	C18H47N5NaO8S	516.3038	2.3	44.4	23	49.70	-2.0	even	ok
	24	C23H52NO5P2S	516.3036	2.0	47.3	24	50.33	0.0	even	ok
	25	C19H50NO12S	516.3048	4.4	48.5	25	20.69	-4.0	even	ok

Compound Spectrum SmartFormula Report

Meas. m/z	#	Ion Formula	m/z	err [ppm]	mSigma	# mSigma	Score	rdb	e¯ Conf	N-Rule
	26	C17H51NNaO12S	516.3024	-0.3	49.2	26	73.65	-7.0	even	ok
	27	C24H48N5OP2S	516.3049	4.6	50.8	27	17.64	5.0	even	ok
	28	C24H44N7NaPS	516.3009	-3.3	52.3	28	28.22	7.0	even	ok
	29	C25H47N3O4PS	516.3019	-1.2	55.3	29	48.83	5.0	even	ok
	30	C16H48N9NaO2PS2	516.3002	-4.5	60.8	30	12.98	-2.0	even	ok
	31	C26H43N7PS	516.3033	1.4	61.3	31	38.26	10.0	even	ok
	32	C18H47N9O2PS2	516.3026	0.1	63.1	32	48.59	1.0	even	ok
	33	C22H47N5NaO3S2	516.3013	-2.5	63.2	33	31.68	2.0	even	ok
	34	C28H48NNaO2PS	516.3036	1.9	63.4	34	30.54	6.0	even	ok
	35	C17H51N5O6PS2	516.3013	-2.5	64.9	35	24.49	-4.0	even	ok
	36	C20H52N3NaO4PS2	516.3029	0.7	65.3	36	39.81	-3.0	even	ok
	37	C27H42N5O3S	516.3003	-4.4	65.8	37	11.53	10.0	even	ok
	38	C21H48N7NaPS2	516.3042	3.2	66.0	38	18.02	2.0	even	ok
	39	C23H50NO7S2	516.3023	-0.5	66.4	39	49.97	0.0	even	ok
	40	C20H42N11OS2	516.3010	-3.1	68.0	40	17.80	6.0	even	ok
	41	C24H46N5O3S2	516.3037	2.1	69.0	41	29.50	5.0	even	ok
	42	C27H52NP2S2	516.3011	-2.9	71.9	42	20.88	4.0	even	ok
	43	C30H43N3NaOS	516.3019	-1.3	75.4	43	23.46	11.0	even	ok
	44	C32H42N3OS	516.3043	3.4	85.9	44	7.92	14.0	even	ok
540.3325	1	C49H85N20O6P	540.3344	3.5	30.8	1	37.04	19.0	even	ok
	2	C51H91N14NaO6P2	540.3304	-3.9	30.8	2	32.12	15.0	even	ok
	3	C49H81N22NaO5	540.3324	-0.2	31.8	3	100.00	21.0	even	ok
	4	C46H80N24O7	540.3315	-1.7	31.8	4	67.25	20.0	even	ok
	5	C49H86N20O4P2	540.3303	-4.1	32.8	5	27.37	19.0	even	ok
	6	C47H76N28O3	540.3322	-0.5	33.6	6	89.94	25.0	even	ok
	7	C47H86N20NaO6P	540.3332	1.3	33.7	7	73.18	16.0	even	ok
	8	C47H90N18O7P2	540.3352	5.0	34.3	8	17.15	14.0	even	ok
	9	C48H82N24NaO2P	540.3338	2.5	34.7	9	48.59	21.0	even	ok
	10	C52H86N18NaO4P	540.3352	5.0	34.7	10	17.25	20.0	even	ok
	11	C45H77N28NaO3	540.3310	-2.7	35.8	11	43.86	22.0	even	ok
	12	C50H81N24O2P	540.3350	4.7	35.9	12	18.94	24.0	even	ok
	13	C52H87N18NaO2P2	540.3311	-2.6	36.0	13	45.39	20.0	even	ok
	14	C45H81N26O4P	540.3330	1.0	36.2	14	73.70	20.0	even	ok
	15	C46H90N16NaO10P	540.3325	0.0	36.7	15	92.11	11.0	even	ok
	16	C46H77N30P	540.3337	2.3	36.7	16	50.24	25.0	even	ok
	17	C50H82N24P2	540.3309	-2.9	37.4	17	39.92	24.0	even	ok
	18	C50H77N26NaO	540.3330	1.0	37.8	18	71.24	26.0	even	ok
	19	C44H81N24NaO7	540.3303	-4.0	38.0	19	25.55	17.0	even	ok
	20	C46H94N14O11P2	540.3345	3.8	38.2	20	27.18	9.0	even	ok
	21	C46H87N22NaO3P2	540.3347	4.1	38.3	21	24.42	16.0	even	ok
	22	C43H72N34O	540.3309	-3.0	38.3	22	37.45	26.0	even	ok
	23	C44H85N22O8P	540.3324	-0.2	39.2	23	82.98	15.0	even	ok
	24	C44H78N30NaP	540.3325	0.0	40.2	24	83.95	22.0	even	ok
	25	C42H76N30O5	540.3302	-4.2	40.5	25	21.29	21.0	even	ok
	26	C44H82N28OP2	540.3345	3.8	40.8	26	25.45	20.0	even	ok
	27	C54H86N18O2P2	540.3323	-0.4	41.4	27	75.03	23.0	even	ok

Compound Spectrum SmartFormula Report

Meas. m/z	#	Ion Formula	m/z	err [ppm]	mSigma	# mSigma	Score	rdb	e¯ Conf	N-Rule
	28	C45H91N18NaO7P2	540.3340	2.8	41.7	28	36.27	11.0	even	ok
	29	C43H82N26NaO4P	540.3318	-1.2	42.9	29	58.55	17.0	even	ok
	30	C52H76N26O	540.3342	3.2	43.8	30	29.42	29.0	even	ok
	31	C54H82N20NaOP	540.3302	-4.1	44.0	31	19.98	25.0	even	ok
	32	C43H86N24O5P2	540.3339	2.6	44.3	32	36.96	15.0	even	ok
	33	C43H89N18O12P	540.3317	-1.4	45.1	33	51.24	10.0	even	ok
	34	C41H77N32O2P	540.3317	-1.5	45.5	34	50.55	21.0	even	ok
	35	C44H95N14NaO11P2	540.3333	1.6	47.8	35	45.63	6.0	even	ok
	36	C42H83N28NaOP2	540.3333	1.6	48.0	36	45.54	17.0	even	ok
	37	C42H86N22NaO8P	540.3312	-2.4	48.3	37	34.24	12.0	even	ok
	38	C40H80N28O8	540.3351	4.9	48.5	38	12.21	16.0	even	ok
	39	C42H90N20O9P2	540.3332	1.3	50.3	39	45.63	10.0	even	ok
	40	C40H81N28O6P	540.3310	-2.7	50.8	40	28.99	16.0	even	ok
	41	C57H87N16NaP2	540.3331	1.1	51.3	41	46.94	24.0	even	ok
	42	C39H77N32NaO4	540.3346	3.9	51.8	42	17.31	18.0	even	ok
	43	C41H89N18NaO14	540.3346	4.0	53.4	43	16.39	7.0	even	ok
	44	C41H87N24NaO5P2	540.3327	0.3	53.5	44	53.18	12.0	even	ok
	45	C39H78N32NaO2P	540.3305	-3.7	54.2	45	17.88	18.0	even	ok
	46	C37H72N38O2	540.3345	3.7	54.3	46	17.80	22.0	even	ok
	47	C41H90N18NaO12P	540.3305	-3.7	55.6	47	17.18	7.0	even	ok
	48	C43H99N10NaO15P2	540.3327	0.3	55.6	48	49.55	1.0	even	ok
	49	C39H84N24O12	540.3345	3.7	55.7	49	16.95	11.0	even	ok
	50	C39H82N30O3P2	540.3325	0.1	56.0	50	52.00	16.0	even	ok
	51	C37H73N38P	540.3304	-3.9	56.8	51	14.77	22.0	even	ok
	52	C41H94N16O13P2	540.3325	0.1	57.9	52	48.72	5.0	even	ok
	53	C39H85N24O10P	540.3304	-3.9	57.9	53	14.27	11.0	even	ok
	54	C38H81N28NaO8	540.3339	2.7	58.6	54	22.55	13.0	even	ok
	55	C40H91N20NaO9P2	540.3320	-0.9	60.6	55	36.52	7.0	even	ok
	56	C38H82N28NaO6P	540.3298	-4.9	60.8	56	8.26	13.0	even	ok
	57	C36H76N34O6	540.3338	2.4	61.0	57	22.74	17.0	even	ok
	58	C38H86N26O7P2	540.3319	-1.2	63.0	58	31.41	11.0	even	ok
	59	C35H73N38NaO2	540.3333	1.5	64.0	59	27.92	19.0	even	ok
	60	C40H94N14NaO16P	540.3298	-4.9	64.0	60	7.43	2.0	even	ok
	61	C38H88N20O16	540.3338	2.5	64.2	61	20.27	6.0	even	ok
	62	C37H83N30NaO3P2	540.3313	-2.2	65.9	62	21.09	13.0	even	ok
	63	C33H68N44	540.3331	1.2	66.5	63	27.50	23.0	even	ok
	64	C40H98N12O17P2	540.3319	-1.2	66.6	64	27.73	0.0	even	ok
	65	C37H85N24NaO12	540.3333	1.5	66.6	65	25.40	8.0	even	ok
	66	C35H78N36OP2	540.3312	-2.4	68.4	66	17.73	17.0	even	ok
	67	C35H80N30O10	540.3331	1.2	68.9	67	25.16	12.0	even	ok
	68	C39H95N16NaO13P2	540.3313	-2.1	68.9	68	19.00	2.0	even	ok
	69	C28H73N44NaS	540.3336	2.1	70.4	69	18.27	15.0	even	ok
	70	C37H90N22O11P2	540.3312	-2.4	71.1	70	16.06	6.0	even	ok
	71	C34H77N34NaO6	540.3326	0.2	71.5	71	29.22	14.0	even	ok
	72	C36H86N26NaO9P	540.3348	4.2	71.6	72	7.73	8.0	even	ok
	73	C30H83N36NaOP2S	540.3317	-1.5	73.0	73	19.76	9.0	even	ok

Compound Spectrum SmartFormula Report

Meas. m/z	#	Ion Formula	m/z	err [ppm]	mSigma	# mSigma	Score	rdb	e⁻ Conf	N-Rule
	74	C52H104O18S2	540.3327	0.3	73.2	74	40.13	2.0	even	ok
	75	C36H87N26NaO7P2	540.3306	-3.4	73.7	75	10.16	8.0	even	ok
	76	C34H81N32O7P	540.3346	4.0	73.8	76	7.94	12.0	even	ok
	77	C32H72N40O4	540.3325	-0.0	73.8	77	27.84	18.0	even	ok
	78	C36H89N20NaO16	540.3326	0.2	75.5	78	25.04	3.0	even	ok
	79	C34H82N32O5P2	540.3305	-3.7	76.0	79	8.38	12.0	even	ok
	80	C38H98N12NaO19P	540.3348	4.2	76.1	80	6.47	-3.0	even	ok
	81	C33H78N36NaO3P	540.3341	3.0	76.5	81	10.62	14.0	even	ok
	82	C31H69N44Na	540.3319	-1.0	76.7	82	19.66	20.0	even	ok
	83	C34H84N26O14	540.3325	-0.0	77.6	83	24.10	7.0	even	ok
	84	C38H99N12NaO17P2	540.3307	-3.4	77.9	84	8.63	-3.0	even	ok
	85	C36H93N18O17P	540.3346	4.0	78.1	85	6.68	1.0	even	ok
	86	C45H92N16O10S2	540.3306	-3.4	78.4	86	10.47	9.0	even	ok
	87	C47H97N10NaO12S2	540.3308	-3.2	78.4	87	11.57	5.0	even	ok
	88	C47H101N8O13PS2	540.3328	0.6	78.4	88	25.52	3.0	even	ok
	89	C46H98N12NaO9PS2	540.3323	-0.4	78.5	89	26.86	5.0	even	ok
	90	C49H106N2NaO15PS2	540.3329	0.9	78.5	90	23.87	-1.0	even	ok
	91	C44H89N20NaO6S2	540.3301	-4.4	78.5	91	6.86	11.0	even	ok
	92	C48H107N4NaO12P2S2	540.3344	3.6	78.5	92	9.55	-1.0	even	ok
	93	C48H100N6O16S2	540.3313	-2.2	78.6	93	16.30	3.0	even	ok
	94	C44H93N18O7PS2	540.3321	-0.6	78.6	94	25.12	9.0	even	ok
	95	C49H110O16P2S2	540.3350	4.6	78.6	95	6.18	-3.0	even	ok
	96	C46H102N10O10P2S2	540.3343	3.4	78.6	96	10.55	3.0	even	ok
	97	C47H98N14O6P2S2	540.3350	4.6	78.6	97	6.20	8.0	even	ok
	98	C49H103N8NaO8P2S2	540.3351	4.9	78.7	98	5.48	4.0	even	ok
	99	C42H84N26O4S2	540.3300	-4.7	78.7	99	6.05	15.0	even	ok
	100	C33H79N36NaOP2	540.3300	-4.6	78.7	100	4.88	14.0	even	ok
	101	C50H105NaO18S2	540.3315	-1.9	78.7	101	17.57	-1.0	even	ok
	102	C45H99N14NaO6P2S2	540.3338	2.4	78.8	102	15.02	5.0	even	ok
	103	C46H95N18NaO2P2S2	540.3344	3.6	78.8	103	9.48	10.0	even	ok
	104	C48H97N12O9PS2	540.3335	1.8	78.9	104	17.84	8.0	even	ok
	105	C31H73N42OP	540.3340	2.7	78.9	105	10.63	18.0	even	ok
	106	C43H90N22NaO3PS2	540.3316	-1.6	78.9	106	19.00	11.0	even	ok
	107	C45H89N22O3PS2	540.3328	0.6	78.9	107	25.15	14.0	even	ok
	108	C47H94N16NaO5PS2	540.3329	0.9	78.9	108	23.56	10.0	even	ok
	109	C44H90N24P2S2	540.3343	3.4	78.9	109	10.46	14.0	even	ok
	110	C51H106N2O13P2S2	540.3300	-4.5	79.0	110	6.30	2.0	even	ok
	111	C50H102N6NaO11PS2	540.3336	2.1	79.0	111	16.33	4.0	even	ok
	112	C43H94N20O4P2S2	540.3336	2.1	79.1	112	16.19	9.0	even	ok
	113	C46H101N6NaO16S2	540.3301	-4.4	79.1	113	6.73	0.0	even	ok
	114	C46H88N20O6S2	540.3313	-2.2	79.1	114	15.91	14.0	even	ok
	115	C53H101NaO18S	540.3298	-5.0	79.1	115	4.99	4.0	even	ok
	116	C41H85N28OPS2	540.3315	-1.9	79.2	116	17.36	15.0	even	ok
	117	C44H96N12O14S2	540.3300	-4.6	79.2	117	5.95	4.0	even	ok
	118	C45H85N24NaO2S2	540.3308	-3.2	79.2	118	11.17	16.0	even	ok
	119	C43H80N30S2	540.3306	-3.4	79.2	119	10.09	20.0	even	ok

Compound Spectrum SmartFormula Report

Meas. m/z	#	Ion Formula	m/z	err [ppm]	mSigma	# mSigma	Score	rdb	e¯ Conf	N-Rule
	120	C47H104N2O20S2	540.3306	-3.4	79.2	120	10.17	-2.0	even	ok
	121	C48H93N14NaO8S2	540.3314	-1.9	79.2	121	17.17	10.0	even	ok
	122	C52H106O16P2S	540.3333	1.5	79.3	122	19.45	2.0	even	ok
	123	C51H105N2O15PS2	540.3342	3.1	79.3	123	11.45	2.0	even	ok
	124	C49H100N4O19S	540.3346	3.9	79.4	124	8.34	3.0	even	ok
	125	C49H96N10O12S2	540.3320	-0.9	79.4	125	22.63	8.0	even	ok
	126	C52H102N2NaO15PS	540.3313	-2.3	79.4	126	15.29	4.0	even	ok
	127	C42H91N24NaP2S2	540.3331	1.1	79.5	127	21.32	11.0	even	ok
	128	C46H105N4O17PS2	540.3321	-0.6	79.5	128	24.25	-2.0	even	ok
	129	C45H102N8NaO13PS2	540.3316	-1.6	79.5	129	18.55	0.0	even	ok
	130	C56H101N2NaO12S2	540.3341	3.1	79.7	130	13.71	8.0	even	ok
	131	C51H101N4NaO14S2	540.3321	-0.7	79.7	131	23.86	4.0	even	ok
	132	C42H93N18NaO9S2	540.3351	4.8	79.7	132	5.53	6.0	even	ok
	133	C50H97N8O13PS	540.3311	-2.5	79.7	133	13.82	8.0	even	ok
	134	C43H97N14O11PS2	540.3315	-1.9	79.8	134	16.98	4.0	even	ok
	135	C49H101N4O17PS	540.3305	-3.8	79.8	135	8.61	3.0	even	ok
	136	C47H111NaO16P2S2	540.3338	2.4	79.9	136	14.34	-6.0	even	ok
	137	C33H81N30NaO10	540.3319	-1.0	79.9	137	17.35	9.0	even	ok
	138	C51H103N4NaO12P2S	540.3328	0.5	80.0	138	24.53	4.0	even	ok
	139	C36H94N18O15P2	540.3305	-3.6	80.0	139	7.16	1.0	even	ok
	140	C42H94N18NaO7PS2	540.3309	-2.9	80.0	140	12.04	6.0	even	ok
	141	C40H88N24O7S2	540.3349	4.5	80.1	141	6.15	10.0	even	ok
	142	C45H106N6O14P2S2	540.3336	2.1	80.1	142	15.49	-2.0	even	ok
	143	C48H97N8NaO15S	540.3340	2.9	80.1	143	11.95	5.0	even	ok
	144	C49H93N12NaO11S	540.3347	4.1	80.1	144	7.27	10.0	even	ok
	145	C35H90N22NaO13P	540.3341	3.0	80.2	145	9.12	3.0	even	ok
	146	C44H103N10NaO10P2S2	540.3331	1.1	80.3	146	20.63	0.0	even	ok
	147	C50H107NaO16P2S	540.3321	-0.7	80.3	147	22.93	-1.0	even	ok
	148	C54H101N2O15PS	540.3325	-0.0	80.3	148	26.98	7.0	even	ok
	149	C53H102N4O12P2S	540.3340	2.7	80.4	149	12.48	7.0	even	ok
	150	C40H89N24O5PS2	540.3308	-3.1	80.4	150	10.76	10.0	even	ok
	151	C49H98N10O10P2S	540.3326	0.3	80.5	151	25.57	8.0	even	ok
	152	C39H85N28NaO3S2	540.3344	3.5	80.5	152	9.25	12.0	even	ok
	153	C47H88N18O9S	540.3346	3.9	80.6	153	7.98	14.0	even	ok
	154	C49H94N12NaO9PS	540.3306	-3.5	80.6	154	9.25	10.0	even	ok
	155	C49H94N16O3P2S2	540.3300	-4.5	80.6	155	5.90	13.0	even	ok
	156	C55H100O18S	540.3310	-2.8	80.6	156	12.07	7.0	even	ok
	157	C48H98N8NaO13PS	540.3299	-4.7	80.6	157	5.37	5.0	even	ok
	158	C42H98N16O8P2S2	540.3330	0.9	80.6	158	21.76	4.0	even	ok
	159	C46H92N14O13S	540.3339	2.6	80.6	159	12.82	9.0	even	ok
	160	C49H93N16O5PS2	540.3341	3.1	80.8	160	10.82	13.0	even	ok
	161	C48H90N20NaOPS2	540.3336	2.1	80.8	161	15.26	15.0	even	ok
	162	C48H104O23S	540.3339	2.6	80.8	162	12.69	-2.0	even	ok
	163	C51H99N10NaO5P2S2	540.3302	-4.3	80.8	163	6.56	9.0	even	ok
	164	C53H98N6NaO11PS	540.3319	-1.0	80.9	164	20.79	9.0	even	ok
	165	C48H102N6O14P2S	540.3319	-1.0	80.9	165	20.95	3.0	even	ok

Compound Spectrum SmartFormula Report

Meas. m/z	#	Ion Formula	m/z	err [ppm]	mSigma	# mSigma	Score	rdb	e¯ Conf	N-Rule
	166	C39H86N28NaOPS2	540.3303	-4.1	81.0	166	7.04	12.0	even	ok
	167	C52H102N6O9P2S2	540.3307	-3.3	81.0	167	9.86	7.0	even	ok
	168	C54H97N4NaO14S	540.3304	-3.8	81.0	168	8.10	9.0	even	ok
	169	C37H80N34OS2	540.3342	3.3	81.0	169	10.04	16.0	even	ok
	170	C41H95N20NaO4P2S2	540.3324	-0.1	81.0	170	25.80	6.0	even	ok
	171	C47H89N18O7PS	540.3304	-3.8	81.1	171	8.16	14.0	even	ok
	172	C52H92N10O12S	540.3303	-4.0	81.1	172	7.23	13.0	even	ok
	173	C52H99N8NaO8P2S	540.3334	1.7	81.1	173	16.78	9.0	even	ok
	174	C51H98N10NaO7PS2	540.3343	3.3	81.1	174	9.67	9.0	even	ok
	175	C51H93N12O9PS	540.3318	-1.3	81.1	175	19.21	13.0	even	ok
	176	C46H93N14O11PS	540.3298	-5.0	81.2	176	4.62	9.0	even	ok
	177	C47H84N24O2S2	540.3320	-0.9	81.3	177	20.93	19.0	even	ok
	178	C48H105O21PS	540.3298	-5.0	81.3	178	4.63	-2.0	even	ok
	179	C52H101N6O11PS2	540.3348	4.3	81.3	179	6.32	7.0	even	ok
	180	C54H107NaO11P2S2	540.3308	-3.0	81.4	180	10.69	3.0	even	ok
	181	C50H94N14O6P2S	540.3333	1.5	81.4	181	17.87	13.0	even	ok
	182	C48H95N14NaO6P2S	540.3321	-0.7	81.4	182	21.88	10.0	even	ok
	183	C47H101N4NaO19S	540.3334	1.6	81.5	183	17.01	0.0	even	ok
	184	C46H85N22NaO5S	540.3340	2.9	81.5	184	11.32	16.0	even	ok
	185	C49H89N18NaO4S2	540.3321	-0.7	81.6	185	22.09	15.0	even	ok
	186	C39H90N26O2P2S2	540.3323	-0.4	81.6	186	23.82	10.0	even	ok
	187	C45H89N18NaO9S	540.3334	1.6	81.6	187	16.98	11.0	even	ok
	188	C51H89N14NaO8S	540.3298	-5.0	81.7	188	4.46	15.0	even	ok
	189	C50H92N14O8S2	540.3327	0.3	81.7	189	23.95	13.0	even	ok
	190	C44H105N4NaO19S2	540.3351	4.8	81.8	190	5.07	-5.0	even	ok
	191	C54H106NaO13PS2	540.3350	4.6	81.8	191	5.51	3.0	even	ok
	192	C47H99N10NaO10P2S	540.3314	-2.0	81.9	192	15.15	5.0	even	ok
	193	C50H90N16NaO5PS	540.3313	-2.3	81.9	193	13.76	15.0	even	ok
	194	C44H106N4NaO17PS2	540.3309	-2.9	82.0	194	11.15	-5.0	even	ok
	195	C45H109O21PS2	540.3315	-1.9	82.0	195	15.55	-7.0	even	ok
	196	C46H90N20O4P2S	540.3319	-1.0	82.0	196	19.95	14.0	even	ok
	197	C42H100N10O17S2	540.3349	4.5	82.1	197	5.65	-1.0	even	ok
	198	C31H76N36O8	540.3318	-1.3	82.1	198	14.79	13.0	even	ok
	199	C46H86N22NaO3PS	540.3299	-4.8	82.1	199	5.03	16.0	even	ok
	200	C44H80N28O3S	540.3339	2.6	82.1	200	12.12	20.0	even	ok
	201	C45H96N10O17S	540.3332	1.4	82.1	201	17.85	4.0	even	ok
	202	C25H72N46O2S	540.3328	0.6	82.2	202	17.58	14.0	even	ok
	203	C52H97N8NaO10S2	540.3328	0.6	82.2	203	22.11	9.0	even	ok
	204	C35H91N22NaO11P2	540.3300	-4.6	82.2	204	4.25	3.0	even	ok
	205	C41H97N14NaO13S2	540.3344	3.5	82.2	205	8.60	1.0	even	ok
	206	C48H85N22O3PS	540.3311	-2.5	82.3	206	12.42	19.0	even	ok
	207	C43H84N24O7S	540.3332	1.4	82.3	207	17.79	15.0	even	ok
	208	C42H101N10O15PS2	540.3308	-3.1	82.4	208	9.98	-1.0	even	ok
	209	C33H85N28O11P	540.3340	2.7	82.4	209	9.18	7.0	even	ok
	210	C49H91N18NaO2P2S	540.3328	0.5	82.4	210	22.28	15.0	even	ok
	211	C45H94N16O8P2S	540.3313	-2.2	82.6	211	13.53	9.0	even	ok

Compound Spectrum SmartFormula Report

Meas. m/z	#	Ion Formula	m/z	err [ppm]	mSigma	# mSigma	Score	rdb	e¯ Conf	N-Rule
	212	C41H98N14NaO11PS2	540.3303	-4.1	82.6	212	6.61	1.0	even	ok
	213	C53H100N4O14S2	540.3333	1.6	82.6	213	16.68	7.0	even	ok
	214	C39H92N20O11S2	540.3342	3.3	82.7	214	9.34	5.0	even	ok
	215	C44H81N28OPS	540.3298	-5.0	82.8	215	4.32	20.0	even	ok
	216	C47H106N2O18P2S	540.3313	-2.2	82.8	216	13.47	-2.0	even	ok
	217	C56H103N2NaO10P2S	540.3348	4.2	82.8	217	6.20	8.0	even	ok
	218	C47H81N26NaOS	540.3347	4.1	82.9	218	6.53	21.0	even	ok
	219	C54H98N8O8P2S	540.3346	4.0	82.9	219	6.93	12.0	even	ok
	220	C47H86N24P2S	540.3326	0.2	82.9	220	23.18	19.0	even	ok
	221	C44H110N2O18P2S2	540.3330	0.9	82.9	221	19.75	-7.0	even	ok
	222	C46H102N6NaO16PS	540.3349	4.4	82.9	222	5.69	0.0	even	ok
	223	C44H90N20NaO6PS	540.3349	4.4	83.0	223	5.70	11.0	even	ok
	224	C38H89N24NaO7S2	540.3337	2.3	83.0	224	13.11	7.0	even	ok
	225	C44H93N14NaO13S	540.3327	0.4	83.0	225	22.21	6.0	even	ok
	226	C43H107N6NaO14P2S2	540.3324	-0.1	83.1	226	23.80	-5.0	even	ok
	227	C39H93N20O9PS2	540.3301	-4.4	83.1	227	5.77	5.0	even	ok
	228	C45H87N24NaP2S	540.3314	-2.0	83.3	228	14.26	16.0	even	ok
	229	C55H97N6O11PS	540.3331	1.2	83.3	229	17.94	12.0	even	ok
	230	C57H102NaO13PS	540.3333	1.5	83.4	230	16.61	8.0	even	ok
	231	C42H81N28NaO3S	540.3327	0.4	83.5	231	21.87	17.0	even	ok
	232	C41H102N12O12P2S2	540.3323	-0.4	83.5	232	22.00	-1.0	even	ok
	233	C53H95N12NaO4P2S	540.3341	3.0	83.6	233	9.98	14.0	even	ok
	234	C36H84N30O5S2	540.3336	2.0	83.6	234	13.91	11.0	even	ok
	235	C46H103N6NaO14P2S	540.3307	-3.2	83.7	235	9.13	0.0	even	ok
	236	C52H96N4O19	540.3329	0.8	83.7	236	19.86	8.0	even	ok
	237	C44H97N12O14PS	540.3347	4.2	83.7	237	6.18	4.0	even	ok
	238	C44H91N20NaO4P2S	540.3307	-3.2	83.8	238	9.06	11.0	even	ok
	239	C51H90N18O2P2S	540.3340	2.7	83.8	239	10.87	18.0	even	ok
	240	C42H85N26O4PS	540.3347	4.1	83.8	240	6.19	15.0	even	ok
	241	C54H94N10NaO7PS	540.3326	0.2	83.8	241	22.45	14.0	even	ok
	242	C42H88N20O11S	540.3326	0.1	83.8	242	22.80	10.0	even	ok
	243	C52H89N16O5PS	540.3325	-0.0	83.9	243	23.28	18.0	even	ok
	244	C40H99N16NaO8P2S2	540.3318	-1.3	83.9	244	16.81	1.0	even	ok
	245	C56H96N4O14S	540.3316	-1.6	83.9	245	15.77	12.0	even	ok
	246	C55H97N2NaO17	540.3337	2.3	84.0	246	12.68	9.0	even	ok
	247	C51H100O23	540.3322	-0.5	84.1	247	20.88	3.0	even	ok
	248	C50H89N20OPS2	540.3348	4.3	84.1	248	5.67	18.0	even	ok
	249	C52H95N14NaOP2S2	540.3308	-3.1	84.2	249	9.50	14.0	even	ok
	250	C46H105NaO23S	540.3327	0.4	84.2	250	21.13	-5.0	even	ok
	251	C53H88N14O8S	540.3310	-2.8	84.2	251	10.36	18.0	even	ok
	252	C58H101N2O10PS2	540.3312	-2.3	84.2	252	14.60	11.0	even	ok
	253	C35H81N34NaOS2	540.3330	1.0	84.2	253	18.07	13.0	even	ok
	254	C53H102NaO18P	540.3345	3.8	84.3	254	7.10	4.0	even	ok
	255	C55H102O16P2	540.3316	-1.6	84.3	255	15.26	7.0	even	ok
	256	C55H93N8NaO10S	540.3311	-2.5	84.3	256	11.32	14.0	even	ok
	257	C40H76N34OS	540.3326	0.1	84.3	257	22.40	21.0	even	ok

Compound Spectrum SmartFormula Report

Meas. m/z	#	Ion Formula	m/z	err [ppm]	mSigma	# mSigma	Score	rdb	e⁻ Conf	N-Rule
	258	C53H98N10O5P2S2	540.3314	-2.1	84.3	258	13.29	12.0	even	ok
	259	C33H86N28O9P2	540.3298	-4.9	84.4	259	3.43	7.0	even	ok
	260	C44H98N12O12P2S	540.3306	-3.5	84.5	260	7.98	4.0	even	ok
	261	C37H90N26NaO4PS2	540.3352	5.0	84.5	261	3.96	7.0	even	ok
	262	C30H73N40NaO4	540.3313	-2.3	84.5	262	9.88	15.0	even	ok
	263	C38H94N22O6P2S2	540.3316	-1.6	84.5	263	15.20	5.0	even	ok
	264	C52H94N14NaO3PS2	540.3350	4.6	84.5	264	4.94	14.0	even	ok
	265	C42H86N26O2P2S	540.3306	-3.5	84.6	265	7.90	15.0	even	ok
	266	C53H92N8O15	540.3336	2.0	84.6	266	13.43	13.0	even	ok
	267	C51H86N20NaOPS	540.3319	-1.0	84.7	267	17.74	20.0	even	ok
	268	C32H82N32NaO7P	540.3334	1.8	84.7	268	11.54	9.0	even	ok
	269	C26H78N42NaOPS	540.3344	3.6	84.7	269	5.94	10.0	even	ok
	270	C43H94N16NaO10PS	540.3342	3.2	84.7	270	8.88	6.0	even	ok
	271	C52H85N18NaO4S	540.3304	-3.8	84.8	271	6.87	20.0	even	ok
	272	C55H103N4NaO7P2S2	540.3315	-1.8	84.9	272	14.08	8.0	even	ok
	273	C35H93N16NaO20	540.3319	-1.0	84.9	273	14.12	-2.0	even	ok
	274	C44H100N6O21S	540.3326	0.2	84.9	274	21.73	-1.0	even	ok
	275	C50H80N24O2S	540.3303	-4.1	84.9	275	6.13	24.0	even	ok
	276	C41H85N24NaO7S	540.3320	-0.8	85.0	276	18.37	12.0	even	ok
	277	C54H98N4NaO14P	540.3352	5.0	85.0	277	3.93	9.0	even	ok
	278	C41H82N30NaPS	540.3342	3.2	85.1	278	8.78	17.0	even	ok
	279	C53H103NaO16P2	540.3304	-3.8	85.1	279	6.66	4.0	even	ok
	280	C51H93N8NaO15	540.3324	-0.2	85.1	280	21.17	10.0	even	ok
	281	C51H97N6O16P	540.3344	3.5	85.1	281	7.61	8.0	even	ok
	282	C37H91N26NaO2P2S2	540.3311	-2.6	85.1	282	10.79	7.0	even	ok
	283	C35H85N32O2PS2	540.3351	4.8	85.2	283	4.36	11.0	even	ok
	284	C57H96N2O17	540.3349	4.5	85.2	284	5.02	12.0	even	ok
	285	C50H85N22NaS2	540.3328	0.6	85.3	285	19.48	20.0	even	ok
	286	C56H106O11P2S2	540.3320	-0.8	85.3	286	18.25	6.0	even	ok
	287	C43H109NaO23S2	540.3344	3.5	85.4	287	7.49	-10.0	even	ok
	288	C51H88N18O4S2	540.3333	1.5	85.4	288	14.86	18.0	even	ok
	289	C50H97N4NaO19	540.3317	-1.5	85.5	289	15.18	5.0	even	ok
	290	C43H95N16NaO8P2S	540.3301	-4.5	85.6	290	4.99	6.0	even	ok
	291	C41H89N22O8PS	540.3341	2.9	85.6	291	9.42	10.0	even	ok
	292	C57H97N2O15P	540.3308	-3.2	85.7	292	8.58	12.0	even	ok
	293	C52H93N10O12P	540.3351	4.8	85.7	293	4.31	13.0	even	ok
	294	C54H99N4NaO12P2	540.3311	-2.6	85.7	294	10.44	9.0	even	ok
	295	C43H110NaO21PS2	540.3303	-4.1	85.7	295	5.82	-10.0	even	ok
	296	C43H97N10NaO17S	540.3320	-0.8	85.8	296	17.79	1.0	even	ok
	297	C41H104N6O21S2	540.3342	3.3	85.8	297	8.15	-6.0	even	ok
	298	C50H101N2O20P	540.3337	2.3	85.8	298	11.62	3.0	even	ok
	299	C54H97N8O8PS2	540.3299	-4.8	85.8	299	4.14	12.0	even	ok
	300	C39H80N30O5S	540.3319	-1.1	85.9	300	16.50	16.0	even	ok
	301	C35H86N32P2S2	540.3309	-2.8	85.9	301	9.53	11.0	even	ok
	302	C40H101N10NaO17S2	540.3337	2.3	85.9	302	11.57	-4.0	even	ok
	303	C45H106N2NaO20PS	540.3342	3.2	85.9	303	8.41	-5.0	even	ok

Compound Spectrum SmartFormula Report

Meas. m/z	#	Ion Formula	m/z	err [ppm]	mSigma	# mSigma	Score	rdb	e⁻ Conf	N-Rule
	304	C49H88N14O13	540.3322	-0.5	86.0	304	19.20	14.0	even	ok
	305	C51H98N6O14P2	540.3303	-4.1	86.0	305	5.74	8.0	even	ok
	306	C53H93N12NaO6S2	540.3335	1.8	86.0	306	13.42	14.0	even	ok
	307	C52H89N12NaO11	540.3330	1.0	86.1	307	16.82	15.0	even	ok
	308	C41H105N6O19PS2	540.3301	-4.4	86.2	308	5.09	-6.0	even	ok
	309	C56H98N4O12P2	540.3323	-0.4	86.2	309	19.49	12.0	even	ok
	310	C48H92N10O17	540.3315	-1.7	86.4	310	13.49	9.0	even	ok
	311	C54H96N8O10S2	540.3340	2.8	86.4	311	9.48	12.0	even	ok
	312	C56H93N6NaO13	540.3344	3.5	86.4	312	7.29	14.0	even	ok
	313	C38H96N16O15S2	540.3336	2.0	86.5	313	12.30	0.0	even	ok
	314	C56H102N2NaO10PS2	540.3300	-4.6	86.5	314	4.55	8.0	even	ok
	315	C41H90N22O6P2S	540.3299	-4.7	86.5	315	4.25	10.0	even	ok
	316	C52H94N10O10P2	540.3309	-2.9	86.5	316	9.20	13.0	even	ok
	317	C41H92N16O15S	540.3319	-1.1	86.6	317	16.02	5.0	even	ok
	318	C50H94N10NaO12P	540.3339	2.5	86.7	318	10.30	10.0	even	ok
	319	C45H107N2NaO18P2S	540.3301	-4.5	86.7	319	4.78	-5.0	even	ok
	320	C43H101N8O18PS	540.3341	2.9	86.8	320	8.94	-1.0	even	ok
	321	C28H68N46O2	540.3311	-2.5	86.8	321	8.22	19.0	even	ok
	322	C37H93N20NaO11S2	540.3330	1.0	86.8	322	16.17	2.0	even	ok
	323	C50H84N18O9	540.3329	0.7	86.8	323	17.40	19.0	even	ok
	324	C40H86N26NaO4PS	540.3335	1.9	86.9	324	12.49	12.0	even	ok
	325	C33H88N22O18	540.3318	-1.3	86.9	325	12.10	2.0	even	ok
	326	C30H77N38O5P	540.3333	1.5	86.9	326	11.34	13.0	even	ok
	327	C54H88N12O11	540.3342	3.2	86.9	327	7.90	18.0	even	ok
	328	C56H94N6NaO11P	540.3302	-4.1	87.0	328	5.42	14.0	even	ok
	329	C49H101NaO23	540.3310	-2.7	87.1	329	9.51	0.0	even	ok
	330	C42H111N2NaO18P2S2	540.3318	-1.3	87.1	330	14.74	-10.0	even	ok
	331	C38H77N34NaOS	540.3314	-2.1	87.2	331	11.65	18.0	even	ok
	332	C59H102O11P2S	540.3304	-3.9	87.2	332	5.86	11.0	even	ok
	333	C51H90N14NaO8P	540.3345	3.8	87.3	333	6.26	15.0	even	ok
	334	C49H98N6NaO16P	540.3332	1.3	87.3	334	14.72	5.0	even	ok
	335	C54H91N16NaP2S	540.3348	4.2	87.4	335	5.13	19.0	even	ok
	336	C39H102N12NaO14PS2	540.3352	5.0	87.4	336	3.48	-4.0	even	ok
	337	C59H101O13PS	540.3345	3.7	87.5	337	6.43	11.0	even	ok
	338	C35H88N26O9S2	540.3329	0.8	87.5	338	16.81	6.0	even	ok
	339	C56H93N10O7PS	540.3338	2.4	87.5	339	10.26	17.0	even	ok
	340	C37H103N8NaO21P2	540.3300	-4.6	87.5	340	3.42	-8.0	even	ok
	341	C54H89N12O9P	540.3301	-4.4	87.5	341	4.72	18.0	even	ok
	342	C58H99N4NaO7P2S	540.3298	-4.9	87.6	342	3.68	13.0	even	ok
	343	C43H102N8O16P2S	540.3299	-4.7	87.6	343	4.08	-1.0	even	ok
	344	C48H85N18NaO9	540.3317	-1.5	87.6	344	13.80	16.0	even	ok
	345	C50H95N10NaO10P2	540.3297	-5.1	87.6	345	3.38	10.0	even	ok
	346	C40H106N8O16P2S2	540.3316	-1.6	87.6	346	13.35	-6.0	even	ok
	347	C48H89N16O10P	540.3337	2.3	87.6	347	10.78	14.0	even	ok
	348	C35H97N14O21P	540.3340	2.7	87.7	348	7.32	-4.0	even	ok
	349	C55H95N8NaO8P2	540.3317	-1.4	87.7	349	14.20	14.0	even	ok

Compound Spectrum SmartFormula Report

Meas. m/z	#	Ion Formula	m/z	err [ppm]	mSigma	# mSigma	Score	rdb	e⁻ Conf	N-Rule
	350	C40H89N20NaO11S	540.3314	-2.1	87.7	350	11.44	7.0	even	ok
	351	C58H98N4NaO9PS	540.3339	2.7	87.7	351	9.28	13.0	even	ok
	352	C42H98N12NaO14PS	540.3335	1.9	87.7	352	12.00	1.0	even	ok
	353	C49H102N4O17P2	540.3352	5.1	87.8	353	3.43	3.0	even	ok
	354	C38H81N32O2PS	540.3334	1.7	87.9	354	12.94	16.0	even	ok
	355	C53H85N20OPS	540.3331	1.2	87.9	355	14.75	23.0	even	ok
	356	C39H103N12NaO12P2S2	540.3311	-2.6	88.0	356	9.59	-4.0	even	ok
	357	C55H90N14NaO3PS	540.3333	1.5	88.0	357	13.68	19.0	even	ok
	358	C47H89N14NaO13	540.3310	-2.7	88.0	358	9.10	11.0	even	ok
	359	C34H85N30NaO5S2	540.3324	-0.2	88.0	359	18.77	8.0	even	ok
	360	C37H97N18O12PS2	540.3351	4.8	88.1	360	3.84	0.0	even	ok
	361	C47H96N6O21	540.3309	-3.0	88.1	361	8.27	4.0	even	ok
	362	C53H90N14O6P2	540.3316	-1.6	88.3	362	12.80	18.0	even	ok
	363	C53H85N16NaO7	540.3337	2.2	88.4	363	10.57	20.0	even	ok
	364	C47H93N12O14P	540.3330	1.0	88.4	364	15.09	9.0	even	ok
	365	C57H92N8O10S	540.3323	-0.3	88.5	365	17.92	17.0	even	ok
	366	C36H94N22NaO8PS2	540.3345	3.8	88.5	366	5.86	2.0	even	ok
	367	C54H84N18O4S	540.3316	-1.6	88.7	367	12.85	23.0	even	ok
	368	C37H98N18O10P2S2	540.3309	-2.8	88.7	368	8.49	0.0	even	ok
	369	C38H84N26O9S	540.3312	-2.3	88.7	369	10.07	11.0	even	ok
	370	C40H93N18O12PS	540.3334	1.7	88.7	370	12.46	5.0	even	ok
	371	C32H80N36O3S2	540.3322	-0.5	88.8	371	17.08	12.0	even	ok
	372	C32H85N26NaO14	540.3313	-2.3	88.8	372	8.24	4.0	even	ok
	373	C56H89N12NaO6S	540.3318	-1.3	88.9	373	13.72	19.0	even	ok
	374	C59H97N2NaO12S	540.3324	-0.1	88.9	374	18.69	13.0	even	ok
	375	C43H104N2O25S	540.3319	-1.1	88.9	375	14.58	-6.0	even	ok
	376	C54H94N14OP2S2	540.3320	-0.8	89.0	376	15.55	17.0	even	ok
	377	C39H87N28NaOP2S	540.3350	4.7	89.0	377	3.88	12.0	even	ok
	378	C51H80N22O5	540.3336	2.0	89.0	378	11.17	24.0	even	ok
	379	C60H98NaO13P	540.3316	-1.7	89.0	379	12.33	13.0	even	ok
	380	C59H99N2NaO10P2	540.3331	1.1	89.1	380	14.35	13.0	even	ok
	381	C45H84N20O11	540.3309	-3.0	89.1	381	7.91	15.0	even	ok
	382	C36H95N22NaO6P2S2	540.3304	-3.8	89.2	382	5.64	2.0	even	ok
	383	C48H102N2NaO20P	540.3325	0.1	89.2	383	18.43	0.0	even	ok
	384	C53H81N22NaS	540.3311	-2.6	89.2	384	9.13	25.0	even	ok
	385	C34H89N28O6PS2	540.3344	3.5	89.3	385	6.30	6.0	even	ok
	386	C58H93N6O11P	540.3314	-1.9	89.3	386	11.26	17.0	even	ok
	387	C59H92N4O14	540.3300	-4.7	89.4	387	3.82	17.0	even	ok
	388	C48H99N8NaO13P2	540.3347	4.1	89.4	388	5.05	5.0	even	ok
	389	C35H98N14O19P2	540.3298	-4.9	89.4	389	2.78	-4.0	even	ok
	390	C29H74N42NaOP	540.3328	0.5	89.4	390	13.20	15.0	even	ok
	391	C34H94N18NaO17P	540.3334	1.8	89.5	391	9.37	-2.0	even	ok
	392	C57H94N8O8P2	540.3329	0.9	89.5	392	15.10	17.0	even	ok
	393	C31H83N34NaO4P2	540.3349	4.5	89.6	393	3.27	9.0	even	ok
	394	C56H99N8NaO3P2S2	540.3322	-0.6	89.6	394	16.10	13.0	even	ok
	395	C42H101N6NaO21S	540.3314	-2.1	89.7	395	10.54	-4.0	even	ok

Compound Spectrum SmartFormula Report

Meas. m/z	#	Ion Formula	m/z	err [ppm]	mSigma	# mSigma	Score	rdb	e⁻ Conf	N-Rule
	396	C46H93N10NaO17	540.3303	-4.0	89.7	396	5.22	6.0	even	ok
	397	C39H90N22NaO8PS	540.3329	0.7	89.8	397	15.51	7.0	even	ok
	398	C41H99N14NaO11P2S	540.3350	4.7	89.9	398	3.71	1.0	even	ok
	399	C37H81N30NaO5S	540.3307	-3.3	89.9	399	6.67	13.0	even	ok
	400	C33H86N32NaO2PS2	540.3339	2.6	90.0	400	8.86	8.0	even	ok
	401	C34H90N28O4P2S2	540.3303	-4.1	90.0	401	4.87	6.0	even	ok
	402	C48H106O21P2	540.3345	3.8	90.0	402	5.46	-2.0	even	ok
	403	C57H102N4O7P2S2	540.3327	0.4	90.1	403	16.37	11.0	even	ok
	404	C57H89N10NaO9	540.3350	4.7	90.1	404	3.62	19.0	even	ok
	405	C46H97N8O18P	540.3324	-0.2	90.4	405	16.99	4.0	even	ok
	406	C52H84N22S2	540.3340	2.8	90.4	406	8.02	23.0	even	ok
	407	C54H90N16NaPS2	540.3300	-4.6	90.5	407	3.81	19.0	even	ok
	408	C55H84N16O7	540.3349	4.5	90.5	408	4.02	23.0	even	ok
	409	C57H90N10NaO7P	540.3309	-2.9	90.6	409	7.63	19.0	even	ok
	410	C40H96N12O19S	540.3312	-2.3	90.6	410	9.30	0.0	even	ok
	411	C40H108N2O25S2	540.3336	2.0	90.7	411	10.22	-11.0	even	ok
	412	C39H105N6NaO21S2	540.3330	1.0	90.7	412	13.59	-9.0	even	ok
	413	C33H87N32NaP2S2	540.3297	-5.1	90.8	413	2.98	8.0	even	ok
	414	C61H102NaO8PS2	540.3320	-0.9	90.8	414	17.12	12.0	even	ok
	415	C44H88N16O15	540.3302	-4.2	90.8	415	4.44	10.0	even	ok
	416	C55H93N12O4PS2	540.3305	-3.6	90.8	416	5.77	17.0	even	ok
	417	C31H81N38PS2	540.3337	2.3	90.8	417	9.31	12.0	even	ok
	418	C56H91N12NaO4P2	540.3324	-0.1	90.9	418	16.86	19.0	even	ok
	419	C30H80N32O12	540.3311	-2.5	90.9	419	6.90	8.0	even	ok
	420	C39H94N20O9P2S	540.3349	4.4	90.9	420	4.00	5.0	even	ok
	421	C37H85N28O6PS	540.3327	0.4	90.9	421	15.76	11.0	even	ok
	422	C42H105N4O22PS	540.3334	1.7	90.9	422	11.25	-6.0	even	ok
	423	C55H85N16O5P	540.3308	-3.2	91.0	423	6.80	23.0	even	ok
	424	C35H76N36O3S	540.3305	-3.6	91.0	424	5.73	17.0	even	ok
	425	C46H100N2O25	540.3302	-4.2	91.0	425	4.41	-1.0	even	ok
	426	C54H89N16NaO2S2	540.3341	3.0	91.1	426	7.07	19.0	even	ok
	427	C37H100N12O19S2	540.3329	0.8	91.3	427	14.16	-5.0	even	ok
	428	C32H89N24O15P	540.3333	1.5	91.5	428	9.27	2.0	even	ok
	429	C55H92N12O6S2	540.3347	4.0	91.5	429	4.66	17.0	even	ok
	430	C39H93N16NaO15S	540.3307	-3.3	91.5	430	6.23	2.0	even	ok
	431	C47H103N4NaO17P2	540.3340	2.8	91.6	431	7.50	0.0	even	ok
	432	C57H98N6NaO6PS2	540.3307	-3.3	91.6	432	6.18	13.0	even	ok
	433	C36H97N16NaO15S2	540.3324	-0.2	91.6	433	16.07	-3.0	even	ok
	434	C29H78N40O2P2	540.3348	4.3	91.8	434	3.33	13.0	even	ok
	435	C41H102N8NaO18PS	540.3329	0.7	91.8	435	14.18	-4.0	even	ok
	436	C54H81N20NaO3	540.3344	3.5	91.9	436	5.77	25.0	even	ok
	437	C45H94N12NaO14P	540.3318	-1.2	92.0	437	12.37	6.0	even	ok
	438	C38H91N24NaO5P2S	540.3343	3.5	92.2	438	5.76	7.0	even	ok
	439	C43H111NaO21P2S	540.3350	4.7	92.2	439	3.33	-10.0	even	ok
	440	C60H96N4O9S2	540.3304	-3.9	92.2	440	5.78	16.0	even	ok
	441	C39H109N4O22PS2	540.3351	4.8	92.3	441	3.18	-11.0	even	ok

Compound Spectrum SmartFormula Report

Meas. m/z	#	Ion Formula	m/z	err [ppm]	mSigma	# mSigma	Score	rdb	e¯ Conf	N-Rule
	442	C36H82N32NaO2PS	540.3322	-0.6	92.3	442	14.35	13.0	even	ok
	443	C34H92N22O13S2	540.3322	-0.5	92.3	443	14.65	1.0	even	ok
	444	C57H97N6NaO8S2	540.3348	4.3	92.4	444	4.00	13.0	even	ok
	445	C38H106N8NaO18PS2	540.3345	3.8	92.5	445	4.91	-9.0	even	ok
	446	C61H98N2O10P2	540.3343	3.3	92.5	446	5.93	16.0	even	ok
	447	C37H88N22O13S	540.3305	-3.6	92.6	447	5.37	6.0	even	ok
	448	C60H98N4O7P2S	540.3310	-2.7	92.6	448	7.48	16.0	even	ok
	449	C57H90N14OP2S	540.3304	-3.9	92.7	449	4.59	22.0	even	ok
	450	C45H98N10O15P2	540.3339	2.6	92.8	450	7.79	4.0	even	ok
	451	C39H110N4O20P2S2	540.3310	-2.8	92.8	451	7.10	-11.0	even	ok
	452	C39H97N14O16PS	540.3327	0.4	92.8	452	14.44	0.0	even	ok
	453	C33H89N26NaO9S2	540.3317	-1.5	92.8	453	11.06	3.0	even	ok
	454	C57H89N14O3PS	540.3345	3.7	92.9	454	5.09	22.0	even	ok
	455	C59H95N8NaO3P2S	540.3305	-3.7	93.0	455	5.05	18.0	even	ok
	456	C62H97O13P	540.3328	0.6	93.0	456	13.87	16.0	even	ok
	457	C60H97N4O9PS	540.3351	4.9	93.0	457	2.89	16.0	even	ok
	458	C38H107N8NaO16P2S2	540.3304	-3.8	93.0	458	4.77	-9.0	even	ok
	459	C29H77N36NaO8	540.3306	-3.5	93.1	459	4.34	10.0	even	ok
	460	C36H101N14O16PS2	540.3344	3.6	93.1	460	5.29	-5.0	even	ok
	461	C41H106N6O19P2S	540.3349	4.4	93.2	461	3.60	-6.0	even	ok
	462	C59H94N8NaO5PS	540.3346	3.9	93.2	462	4.49	18.0	even	ok
	463	C36H86N30O3P2S	540.3342	3.2	93.3	463	6.05	11.0	even	ok
	464	C34H77N38PS	540.3320	-0.8	93.4	464	12.77	17.0	even	ok
	465	C45H101N4O22P	540.3317	-1.4	93.5	465	10.79	-1.0	even	ok
	466	C31H86N28NaO11P	540.3328	0.5	93.5	466	10.99	4.0	even	ok
	467	C35H98N18NaO12PS2	540.3339	2.6	93.5	467	7.53	-3.0	even	ok
	468	C60H95N6NaO6P2	540.3338	2.4	93.6	468	8.07	18.0	even	ok
	469	C31H84N32O7S2	540.3316	-1.7	93.7	469	9.87	7.0	even	ok
	470	C36H85N26NaO9S	540.3300	-4.6	93.7	470	3.32	8.0	even	ok
	471	C36H102N14O14P2S2	540.3303	-4.1	93.8	471	4.13	-5.0	even	ok
	472	C38H94N18NaO12PS	540.3322	-0.6	93.9	472	13.37	2.0	even	ok
	473	C58H90N12O4P2	540.3336	2.1	93.9	473	8.68	22.0	even	ok
	474	C61H94N4NaO9P	540.3323	-0.4	94.0	474	13.78	18.0	even	ok
	475	C33H95N20NaO14P2	540.3349	4.5	94.1	475	2.66	-2.0	even	ok
	476	C59H89N10O7P	540.3321	-0.7	94.1	476	12.86	22.0	even	ok
	477	C40H103N10NaO15P2S	540.3344	3.5	94.1	477	5.25	-4.0	even	ok
	478	C55H80N22S	540.3323	-0.3	94.2	478	13.88	28.0	even	ok
	479	C58H88N12O6S	540.3330	0.9	94.2	479	12.03	22.0	even	ok
	480	C35H99N18NaO10P2S2	540.3297	-5.1	94.2	480	2.56	-3.0	even	ok
	481	C33H93N24O10PS2	540.3337	2.3	94.4	481	7.93	1.0	even	ok
	482	C30H81N36NaO3S2	540.3310	-2.7	94.4	482	6.92	9.0	even	ok
	483	C62H93N2NaO12	540.3308	-3.2	94.5	483	5.74	18.0	even	ok
	484	C41H105N2NaO25S	540.3307	-3.3	94.5	484	5.47	-9.0	even	ok
	485	C57H85N16NaO2S	540.3324	-0.1	94.5	485	14.50	24.0	even	ok
	486	C60H88N8O10	540.3306	-3.4	94.5	486	5.19	22.0	even	ok
	487	C61H96N2O12S	540.3337	2.2	94.6	487	8.20	16.0	even	ok

Compound Spectrum SmartFormula Report

Meas. m/z	#	Ion Formula	m/z	err [ppm]	mSigma	# mSigma	Score	rdb	e¯ Conf	N-Rule
	488	C60H93N6NaO8S	540.3331	1.2	94.7	488	10.99	18.0	even	ok
	489	C46H107NaO21P2	540.3333	1.6	94.8	489	9.73	-5.0	even	ok
	490	C34H80N32O7S	540.3299	-4.8	94.8	490	2.79	12.0	even	ok
	491	C32H90N28NaO6PS2	540.3332	1.3	95.0	491	10.44	3.0	even	ok
	492	C44H98N8NaO18P	540.3312	-2.4	95.0	492	7.39	1.0	even	ok
	493	C36H89N24O10PS	540.3320	-0.8	95.0	493	11.93	6.0	even	ok
	494	C42H92N14O18	540.3352	4.9	95.1	494	2.62	5.0	even	ok
	495	C27H72N42O6	540.3305	-3.8	95.2	495	3.54	14.0	even	ok
	496	C38H98N16O13P2S	540.3342	3.2	95.2	496	5.53	0.0	even	ok
	497	C58H86N14NaO3P	540.3316	-1.7	95.3	497	9.30	24.0	even	ok
	498	C28H76N42OS2	540.3309	-3.0	95.3	498	6.04	13.0	even	ok
	499	C39H100N8O23S	540.3306	-3.6	95.5	499	4.72	-5.0	even	ok
	500	C56H81N20OP	540.3314	-1.9	95.6	500	8.48	28.0	even	ok
560.3286	1	C19H40N17NaP	560.3282	-0.7	20.7	1	100.00	9.0	even	ok
	2	C17H45N15NaOP2	560.3299	2.3	23.3	2	58.22	4.0	even	ok
	3	C16H39N19O2P	560.3266	-3.6	23.4	3	35.30	8.0	even	ok
	4	C20H43N13O4P	560.3293	1.2	25.1	4	77.88	7.0	even	ok
	5	C18H44N13NaO4P	560.3269	-3.1	25.2	5	41.61	4.0	even	ok
	6	C18H48N11O5P2	560.3310	4.2	27.5	6	24.36	2.0	even	ok
	7	C21H39N17P	560.3306	3.6	27.7	7	31.23	12.0	even	ok
	8	C22H38N15O3	560.3277	-1.7	32.7	8	56.03	12.0	even	ok
	9	C15H38N21OS	560.3283	-0.5	40.4	9	86.16	8.0	even	ok
	10	C20H49N11NaP2S	560.3261	-4.6	40.6	10	19.23	3.0	even	ok
	11	C13H39N21NaOS	560.3259	-4.8	41.8	11	16.74	5.0	even	ok
	12	C17H43N15NaO3S	560.3286	0.0	42.4	12	91.41	4.0	even	ok
	13	C19H42N15O3S	560.3310	4.3	44.5	13	19.75	7.0	even	ok
	14	C21H52N7O4P2S	560.3271	-2.7	44.6	14	39.11	1.0	even	ok
	15	C22H48N11P2S	560.3285	-0.3	45.0	15	79.75	6.0	even	ok
	16	C14H42N17O5S	560.3270	-2.9	45.8	16	34.89	3.0	even	ok
	17	C18H46N11O7S	560.3297	1.9	46.5	17	48.21	2.0	even	ok
	18	C21H47N9NaO5S	560.3313	4.8	46.7	18	14.48	3.0	even	ok
	19	C23H57NNaO6P2S	560.3274	-2.2	46.8	19	43.81	-3.0	even	ok
	20	C24H53N5NaO2P2S	560.3287	0.2	47.3	20	75.71	2.0	even	ok
	21	C20H56N3O8P2S	560.3258	-5.1	47.5	21	12.39	-4.0	even	ok
	22	C16H47N11NaO7S	560.3273	-2.4	47.5	22	39.88	-1.0	even	ok
	23	C20H51N5NaO9S	560.3300	2.4	48.6	23	38.24	-2.0	even	ok
	24	C25H56NO6P2S	560.3298	2.1	50.9	24	39.33	0.0	even	ok
	25	C17H50N7O11S	560.3284	-0.5	51.5	25	62.59	-3.0	even	ok
	26	C21H54NO13S	560.3310	4.3	52.7	26	15.30	-4.0	even	ok
	27	C19H55NNaO13S	560.3286	0.0	53.3	27	65.87	-7.0	even	ok
	28	C26H52N5O2P2S	560.3311	4.5	53.9	28	13.39	5.0	even	ok
	29	C26H48N7NaOPS	560.3271	-2.7	55.3	29	27.53	7.0	even	ok
	30	C27H51N3O5PS	560.3282	-0.8	58.5	30	45.71	5.0	even	ok
	31	C20H51N9O3PS2	560.3288	0.4	60.2	31	60.29	1.0	even	ok
	32	C19H55N5O7PS2	560.3275	-2.0	61.8	32	36.07	-4.0	even	ok
	33	C22H56N3NaO5PS2	560.3291	0.9	62.2	33	49.50	-3.0	even	ok

Compound Spectrum SmartFormula Report

Meas. m/z	#	Ion Formula	m/z	err [ppm]	mSigma	# mSigma	Score	rdb	e¯ Conf	N-Rule
	34	C23H52N7NaOPS2	560.3305	3.3	62.6	34	21.74	2.0	even	ok
	35	C16H47N15OPS2	560.3262	-4.4	62.9	35	10.52	2.0	even	ok
	36	C21H50N7O6S2	560.3259	-4.9	63.7	36	9.74	1.0	even	ok
	37	C28H47N7OPS	560.3295	1.6	64.0	37	30.74	10.0	even	ok
	38	C22H46N11O2S2	560.3272	-2.6	64.4	38	27.09	6.0	even	ok
	39	C18H52N9NaO3PS2	560.3264	-3.9	65.0	39	12.31	-2.0	even	ok
	40	C23H55NNaO8S2	560.3261	-4.4	65.7	40	11.63	-3.0	even	ok
	41	C30H52NNaO3PS	560.3298	2.1	66.2	41	24.22	6.0	even	ok
	42	C24H51N5NaO4S2	560.3275	-2.1	66.5	42	29.95	2.0	even	ok
	43	C29H46N5O4S	560.3265	-3.8	68.6	43	11.40	10.0	even	ok
	44	C25H47N9NaS2	560.3288	0.3	69.1	44	44.70	7.0	even	ok
	45	C25H54NO8S2	560.3285	-0.1	69.8	45	45.43	0.0	even	ok
	46	C26H50N5O4S2	560.3299	2.2	72.1	46	22.86	5.0	even	ok
	47	C29H56NOP2S2	560.3273	-2.3	74.8	47	19.87	4.0	even	ok
	48	C30H42N9S	560.3278	-1.4	75.7	48	20.91	15.0	even	ok
	49	C27H46N9S2	560.3312	4.6	76.1	49	7.23	10.0	even	ok
	50	C32H47N3NaO2S	560.3281	-0.9	77.8	50	22.17	11.0	even	ok
	51	C34H46N3O2S	560.3305	3.4	88.2	51	6.12	14.0	even	ok
562.3433	1	C17H47N15NaOP2	562.3455	4.0	17.8	1	25.26	3.0	even	ok
	2	C16H41N19O2P	562.3423	-1.8	19.5	2	56.19	7.0	even	ok
	3	C18H46N13NaO4P	562.3426	-1.3	22.0	3	62.10	3.0	even	ok
	4	C19H49N9O8P	562.3436	0.6	23.1	4	100.00	1.0	even	ok
	5	C15H45N15O6P	562.3409	-4.2	24.6	5	19.57	2.0	even	ok
	6	C21H54N3NaO10P	562.3439	1.1	25.3	6	83.31	-3.0	even	ok
	7	C19H42N17NaP	562.3439	1.0	25.6	7	62.48	8.0	even	ok
	8	C17H50N9NaO8P	562.3412	-3.7	26.5	8	23.51	-2.0	even	ok
	9	C20H48N7O11	562.3406	-4.8	26.9	9	18.98	1.0	even	ok
	10	C18H53N5O12P	562.3423	-1.8	27.1	10	63.66	-4.0	even	ok
	11	C22H50N7NaO6P	562.3452	3.4	27.2	11	34.77	2.0	even	ok
	12	C18H36N21O	562.3406	-4.8	28.2	12	13.70	12.0	even	ok
	13	C20H45N13O4P	562.3450	2.9	28.7	13	30.79	6.0	even	ok
	14	C22H53NNaO13	562.3409	-4.3	29.2	14	22.96	-3.0	even	ok
	15	C21H44N11O7	562.3420	-2.4	29.4	15	49.81	6.0	even	ok
	16	C20H41N15NaO3	562.3409	-4.3	30.5	16	16.52	8.0	even	ok
	17	C23H49N5NaO9	562.3422	-1.9	31.6	17	55.93	2.0	even	ok
	18	C24H52NO13	562.3433	0.0	34.7	18	86.17	0.0	even	ok
	19	C24H45N9NaO5	562.3436	0.5	37.9	19	70.82	7.0	even	ok
	20	C15H40N21OS	562.3440	1.2	39.4	20	55.92	7.0	even	ok
	21	C25H48N5O9	562.3447	2.4	40.2	21	37.48	5.0	even	ok
	22	C22H40N15O3	562.3433	0.0	41.4	22	54.36	11.0	even	ok
	23	C17H45N15NaO3S	562.3443	1.7	41.7	23	45.21	3.0	even	ok
	24	C20H51N11NaP2S	562.3417	-2.8	42.4	24	29.85	2.0	even	ok
	25	C18H48N11O7S	562.3453	3.6	45.6	25	19.88	1.0	even	ok
	26	C20H58N3O8P2S	562.3414	-3.3	45.8	26	22.37	-5.0	even	ok
	27	C28H54NO6P2	562.3421	-2.2	45.8	27	34.64	4.0	even	ok
	28	C21H54N7O4P2S	562.3428	-0.9	45.9	28	50.27	0.0	even	ok

Compound Spectrum SmartFormula Report

Meas. m/z	#	Ion Formula	m/z	err [ppm]	mSigma	# mSigma	Score	rdb	e⁻ Conf	N-Rule
	29	C20H53N5NaO9S	562.3456	4.1	47.8	29	14.89	-3.0	even	ok
	30	C26H44N9O5	562.3460	4.8	48.1	30	10.71	10.0	even	ok
	31	C23H59NNaO6P2S	562.3431	-0.4	48.2	31	53.48	-4.0	even	ok
	32	C25H46N11P2	562.3407	-4.6	48.8	32	8.74	10.0	even	ok
	33	C22H50N11P2S	562.3441	1.4	49.2	33	39.64	5.0	even	ok
	34	C19H57NNaO13S	562.3443	1.7	49.8	34	35.43	-8.0	even	ok
	35	C27H51N5NaO2P2	562.3410	-4.1	50.8	35	10.44	6.0	even	ok
	36	C24H55N5NaO2P2S	562.3444	1.9	51.5	36	31.64	1.0	even	ok
	37	C25H41N13NaO	562.3449	2.9	53.6	37	15.83	12.0	even	ok
	38	C23H49N9O3PS	562.3411	-3.9	53.7	38	13.77	5.0	even	ok
	39	C25H58NO6P2S	562.3455	3.8	54.6	39	13.73	-1.0	even	ok
	40	C29H50N5O2P2	562.3434	0.2	54.9	40	46.05	9.0	even	ok
	41	C25H54N3NaO5PS	562.3414	-3.4	56.0	41	15.90	1.0	even	ok
	42	C19H57N5O7PS2	562.3432	-0.3	61.1	42	46.27	-5.0	even	ok
	43	C20H53N9O3PS2	562.3445	2.1	61.3	43	27.08	0.0	even	ok
	44	C26H50N7NaOPS	562.3427	-1.0	61.7	44	29.90	6.0	even	ok
	45	C22H58N3NaO5PS2	562.3448	2.6	63.3	45	21.14	-4.0	even	ok
	46	C18H54N9NaO3PS2	562.3421	-2.2	64.3	46	19.11	-3.0	even	ok
	47	C27H53N3O5PS	562.3438	0.9	64.4	47	28.20	4.0	even	ok
	48	C21H52N7O6S2	562.3415	-3.2	65.1	48	15.73	0.0	even	ok
	49	C23H54N7NaOPS2	562.3461	5.0	65.5	49	6.80	1.0	even	ok
	50	C32H51N3NaP2	562.3450	3.1	66.5	50	12.56	10.0	even	ok
	51	C23H57NNaO8S2	562.3418	-2.7	67.1	51	17.74	-4.0	even	ok
	52	C22H48N11O2S2	562.3428	-0.8	67.5	52	32.08	5.0	even	ok
	53	C31H45N7OP	562.3418	-2.7	68.9	53	13.25	14.0	even	ok
	54	C28H52NO8S	562.3408	-4.4	68.9	54	6.35	4.0	even	ok
	55	C24H53N5NaO4S2	562.3431	-0.3	69.5	55	33.75	1.0	even	ok
	56	C33H50NNaO3P	562.3421	-2.2	70.5	56	14.95	10.0	even	ok
	57	C28H49N7OPS	562.3451	3.3	71.4	57	9.75	9.0	even	ok
	58	C27H59NNaOP2S2	562.3406	-4.9	72.3	58	5.54	0.0	even	ok
	59	C25H56NO8S2	562.3442	1.6	72.6	59	21.48	-1.0	even	ok
	60	C30H54NNaO3PS	562.3454	3.8	73.5	60	7.30	5.0	even	ok
	61	C25H49N9NaS2	562.3445	2.0	73.6	61	17.74	6.0	even	ok
	62	C28H45N9NaS	562.3411	-4.0	73.7	62	6.66	11.0	even	ok
	63	C29H48N5O4S	562.3422	-2.1	75.9	63	12.91	9.0	even	ok
	64	C26H52N5O4S2	562.3455	3.9	76.3	64	7.54	4.0	even	ok
	65	C29H58NOP2S2	562.3430	-0.6	79.9	65	21.02	3.0	even	ok
	66	C35H49NO3P	562.3445	2.0	81.8	66	10.21	13.0	even	ok
	67	C30H44N9S	562.3435	0.3	84.1	67	15.21	14.0	even	ok
	68	C32H49N3NaO2S	562.3438	0.8	86.1	68	12.33	10.0	even	ok
	69	C31H53N3PS2	562.3413	-3.6	90.1	69	5.03	8.0	even	ok
	70	C37H44N3O2	562.3428	-0.9	96.3	70	7.70	18.0	even	ok
564.3603	1	C24H54NO13	564.3590	-2.4	3.6	1	54.83	-1.0	even	ok
	2	C23H51N5NaO9	564.3579	-4.3	5.9	2	23.86	1.0	even	ok
	3	C23H55N3O10P	564.3620	2.9	7.6	3	42.80	-1.0	even	ok
	4	C21H46N11O7	564.3576	-4.8	8.1	4	17.93	5.0	even	ok

Compound Spectrum SmartFormula Report

Meas. m/z	#	Ion Formula	m/z	err [ppm]	mSigma	# mSigma	Score	rdb	e⁻ Conf	N-Rule
	5	C25H50N5O9	564.3603	-0.0	8.8	5	100.00	4.0	even	ok
	6	C22H52N7NaO6P	564.3609	1.0	10.3	6	76.50	1.0	even	ok
	7	C24H47N9NaO5	564.3592	-1.9	11.3	7	56.32	6.0	even	ok
	8	C21H56N7O4P2S	564.3584	-3.4	13.0	8	31.79	-1.0	even	ok
	9	C23H48N11NaO2P	564.3622	3.4	13.7	9	23.61	6.0	even	ok
	10	C22H42N15O3	564.3590	-2.4	13.8	10	33.96	10.0	even	ok
	11	C21H51N9NaO5S	564.3626	4.0	13.8	11	23.38	1.0	even	ok
	12	C20H47N13O4P	564.3606	0.5	13.8	12	61.08	5.0	even	ok
	13	C24H57N5NaO2P2S	564.3600	-0.5	14.2	13	80.74	0.0	even	ok
	14	C19H46N15O3S	564.3623	3.5	14.2	14	28.90	5.0	even	ok
	15	C22H52N11P2S	564.3598	-1.0	14.3	15	70.57	4.0	even	ok
	16	C23H61NNaO6P2S	564.3587	-2.9	14.8	16	37.49	-5.0	even	ok
	17	C21H43N17P	564.3619	2.9	15.5	17	27.84	10.0	even	ok
	18	C15H42N21OS	564.3596	-1.2	17.2	18	62.77	6.0	even	ok
	19	C28H56NO6P2	564.3577	-4.6	17.4	19	16.67	3.0	even	ok
	20	C25H60NO6P2S	564.3611	1.4	17.5	20	59.30	-2.0	even	ok
	21	C19H44N17NaP	564.3595	-1.4	17.6	21	44.25	7.0	even	ok
	22	C17H47N15NaO3S	564.3599	-0.7	17.8	22	70.98	2.0	even	ok
	23	C16H48N17NaPS	564.3629	4.6	18.1	23	16.52	2.0	even	ok
	24	C21H56N3NaO10P	564.3596	-1.4	18.2	24	58.65	-4.0	even	ok
	25	C18H50N11O7S	564.3610	1.2	19.3	25	60.73	0.0	even	ok
	26	C19H51N9O8P	564.3593	-1.9	19.7	26	48.64	0.0	even	ok
	27	C26H46N9O5	564.3616	2.3	20.4	27	40.98	9.0	even	ok
	28	C20H55N5NaO9S	564.3613	1.7	20.4	28	51.07	-4.0	even	ok
	29	C28H51N3NaO7	564.3619	2.8	20.7	29	33.88	5.0	even	ok
	30	C26H56N5O2P2S	564.3624	3.8	23.3	30	21.78	3.0	even	ok
	31	C21H58NO13S	564.3623	3.6	24.0	31	23.30	-6.0	even	ok
	32	C18H48N13NaO4P	564.3582	-3.8	24.1	32	15.96	2.0	even	ok
	33	C20H57N5NaO7P2	564.3625	3.9	25.1	33	14.54	-4.0	even	ok
	34	C25H43N13NaO	564.3606	0.4	25.4	34	48.50	11.0	even	ok
	35	C26H52N7NaOPS	564.3584	-3.4	25.7	35	23.62	5.0	even	ok
	36	C16H43N19O2P	564.3579	-4.3	26.2	36	12.10	6.0	even	ok
	37	C18H52N11O5P2	564.3623	3.4	26.9	37	17.31	0.0	even	ok
	38	C27H55N3O5PS	564.3595	-1.5	27.7	38	45.04	3.0	even	ok
	39	C17H49N15NaOP2	564.3612	1.5	28.9	39	32.96	2.0	even	ok
	40	C29H52N5O2P2	564.3591	-2.2	29.1	40	34.93	8.0	even	ok
	41	C27H42N13O	564.3630	4.7	32.1	41	11.30	14.0	even	ok
	42	C20H55N9O3PS2	564.3601	-0.3	32.5	42	69.91	-1.0	even	ok
	43	C22H60N3NaO5PS2	564.3604	0.2	32.8	43	72.32	-5.0	even	ok
	44	C19H56N11P2S2	564.3631	5.0	32.8	44	9.65	-1.0	even	ok
	45	C23H56N7NaOPS2	564.3618	2.5	34.5	45	34.07	0.0	even	ok
	46	C24H59N3O5PS2	564.3628	4.4	36.1	46	14.59	-2.0	even	ok
	47	C22H50N11O2S2	564.3585	-3.3	36.3	47	24.50	4.0	even	ok
	48	C28H51N7OPS	564.3608	0.8	36.6	48	44.43	8.0	even	ok
	49	C24H55N5NaO4S2	564.3588	-2.8	36.7	49	29.56	0.0	even	ok
	50	C30H56NNaO3PS	564.3611	1.3	38.3	50	36.90	4.0	even	ok

Compound Spectrum SmartFormula Report

Meas. m/z	#	Ion Formula	m/z	err [ppm]	mSigma	# mSigma	Score	rdb	e⁻ Conf	N-Rule
	51	C25H58NO8S2	564.3598	-0.9	40.0	51	50.11	-2.0	even	ok
	52	C29H50N5O4S	564.3578	-4.5	40.8	52	10.06	8.0	even	ok
	53	C32H53N3NaP2	564.3607	0.6	41.2	53	41.17	9.0	even	ok
	54	C25H51N9NaS2	564.3601	-0.4	41.9	54	53.66	5.0	even	ok
	55	C26H54N5O4S2	564.3612	1.5	43.1	55	38.38	3.0	even	ok
	56	C33H52NNaO3P	564.3577	-4.7	43.5	56	8.52	9.0	even	ok
	57	C29H60NOP2S2	564.3586	-3.0	46.8	57	19.93	2.0	even	ok
	58	C27H50N9S2	564.3625	3.9	50.0	58	12.81	8.0	even	ok
	59	C29H55N3NaO2S2	564.3628	4.4	50.4	59	10.02	4.0	even	ok
	60	C30H46N9S	564.3591	-2.1	50.7	60	20.10	13.0	even	ok
	61	C34H52N3P2	564.3631	4.9	52.0	61	5.81	12.0	even	ok
	62	C32H51N3NaO2S	564.3594	-1.6	52.3	62	22.50	9.0	even	ok
	63	C35H51NO3P	564.3601	-0.4	54.6	63	29.40	12.0	even	ok
	64	C34H50N3O2S	564.3618	2.7	63.8	64	10.76	12.0	even	ok
	65	C37H46N3O2	564.3585	-3.3	69.1	65	6.82	17.0	even	ok
	66	C40H47NNa	564.3601	-0.5	81.7	66	10.75	18.0	even	ok
	67	C42H46N	564.3625	3.8	93.2	67	2.06	21.0	even	ok
584.3581	1	C40H116N2O32S	584.3610	5.0	23.5	1	13.88	-15.0	even	ok
	2	C40H117N2O30PS	584.3569	-2.1	23.9	2	51.38	-15.0	even	ok
	3	C41H116O33S	584.3554	-4.6	24.0	3	16.56	-15.0	even	ok
	4	C39H113N6NaO28S	584.3605	4.1	24.8	4	21.72	-13.0	even	ok
	5	C35H105N18NaO19S2	584.3566	-2.7	25.3	5	39.72	-7.0	even	ok
	6	C37H108N12O26S	584.3604	3.8	25.4	6	24.02	-9.0	even	ok
	7	C39H114N6NaO26PS	584.3564	-3.0	25.5	7	34.82	-13.0	even	ok
	8	C37H2NNaO35P2S2	584.3605	4.1	25.6	8	20.90	39.0	even	ok
	9	C36H108N14O23S2	584.3571	-1.8	26.0	9	54.64	-9.0	even	ok
	10	C37H109N12O24PS	584.3563	-3.2	26.3	10	30.88	-9.0	even	ok
	11	C38H113N8NaO25S2	584.3573	-1.5	27.1	11	57.62	-13.0	even	ok
	12	C36H105N16NaO22S	584.3598	2.9	27.4	12	34.33	-7.0	even	ok
	13	C34H100N22O20S	584.3597	2.7	28.5	14	36.90	-3.0	even	ok
	14	C39H116N4O29S2	584.3578	-0.6	28.5	15	72.94	-15.0	even	ok
	15	C36H106N16NaO20PS	584.3557	-4.1	28.5	16	19.18	-7.0	even	ok
	16	C37H111N16NaO15P2S2	584.3609	4.7	28.6	17	14.00	-8.0	even	ok
	17	C42H119N2NaO25P2S	584.3592	1.9	28.7	18	49.31	-14.0	even	ok
	18	C40H114N8O23P2S	584.3591	1.6	29.1	19	52.98	-10.0	even	ok
	19	C43H118NaO28PS	584.3577	-0.7	29.4	20	69.77	-14.0	even	ok
	20	C41H113N6O26PS	584.3576	-0.9	29.4	21	65.26	-10.0	even	ok
	21	C33H97N30O10PS2	584.3586	0.8	29.6	22	67.52	2.0	even	ok
	22	C34H101N22O18PS	584.3556	-4.4	29.7	23	16.54	-3.0	even	ok
	23	C36H105N20O16PS2	584.3593	1.9	30.0	25	46.55	-4.0	even	ok
	24	C35H102N24NaO12PS2	584.3587	1.0	30.0	26	62.35	-2.0	even	ok
	25	C38H111N14NaO16P2S2	584.3553	-4.9	30.2	27	12.62	-8.0	even	ok
	26	C30H89N40O4PS2	584.3579	-0.4	30.3	28	74.45	8.0	even	ok
	27	C32H94N34NaO6PS2	584.3581	-0.1	30.3	29	78.91	4.0	even	ok
	28	C42H112N4O29S	584.3561	-3.5	30.3	30	24.99	-10.0	even	ok
	29	C39H114N10O20P2S2	584.3558	-4.0	30.6	31	19.98	-10.0	even	ok

Compound Spectrum SmartFormula Report

Meas. m/z	#	Ion Formula	m/z	err [ppm]	mSigma	# mSigma	Score	rdb	e¯ Conf	N-Rule
	30	C38H104N16O22S	584.3610	5.0	30.7	32	11.77	-4.0	even	ok
	31	C40H110N10NaO22PS	584.3571	-1.8	30.8	33	47.30	-8.0	even	ok
	32	C38H110N14NaO18PS2	584.3594	2.2	30.9	34	41.82	-8.0	even	ok
	33	C33H97N26NaO16S	584.3592	1.8	31.0	35	48.50	-1.0	even	ok
	34	C39H111N12NaO19P2S	584.3586	0.7	31.0	36	66.59	-8.0	even	ok
	35	C38H105N16O20PS	584.3569	-2.1	31.2	37	43.05	-4.0	even	ok
	36	C31H88N38O7S2	584.3564	-2.9	31.3	38	31.18	8.0	even	ok
	37	C41H109N8NaO25S	584.3556	-4.4	31.3	39	15.70	-8.0	even	ok
	38	C34H96N28O13S2	584.3571	-1.8	31.4	40	47.89	2.0	even	ok
	39	C39H104N14O23S	584.3554	-4.6	31.4	41	13.82	-4.0	even	ok
	40	C39H113N10O22PS2	584.3599	3.1	31.6	42	28.69	-10.0	even	ok
	41	C33H93N32NaO9S2	584.3566	-2.7	31.7	43	34.04	4.0	even	ok
	42	C29H86N44NaPS2	584.3574	-1.3	31.7	44	55.71	10.0	even	ok
	43	C37H106N18O17P2S	584.3584	0.5	31.7	45	69.68	-4.0	even	ok
	44	C41H119N4NaO22P2S2	584.3560	-3.7	31.8	46	21.76	-14.0	even	ok
	45	C36H101N22NaO15S2	584.3572	-1.5	32.2	47	50.82	-2.0	even	ok
	46	C30H85N42NaO3S2	584.3559	-3.8	32.2	48	20.32	10.0	even	ok
	47	C31H92N32O14S	584.3590	1.5	32.3	49	50.80	3.0	even	ok
	48	C28H80N48OS2	584.3558	-4.1	32.3	50	18.08	14.0	even	ok
	49	C37H101N20NaO18S	584.3605	4.1	32.5	51	18.13	-2.0	even	ok
	50	C37H104N18O19S2	584.3578	-0.6	32.6	52	65.83	-4.0	even	ok
	51	C42H122O26P2S2	584.3565	-2.8	32.9	53	31.48	-16.0	even	ok
	52	C41H118N4NaO24PS2	584.3601	3.3	32.9	54	25.07	-14.0	even	ok
	53	C35H96N26O16S	584.3604	3.8	33.2	55	20.01	2.0	even	ok
	54	C37H102N20NaO16PS	584.3564	-3.0	33.3	56	28.74	-2.0	even	ok
	55	C39H109N12NaO21S2	584.3579	-0.4	33.7	57	68.06	-8.0	even	ok
	56	C35H97N26O14PS	584.3562	-3.2	34.1	58	25.46	2.0	even	ok
	57	C36H103N22NaO13P2S	584.3579	-0.4	34.1	59	66.28	-2.0	even	ok
	58	C42H121O28PS2	584.3606	4.3	34.2	60	15.76	-16.0	even	ok
	59	C41H117O33P	584.3602	3.5	34.3	61	22.43	-15.0	even	ok
	60	C40H112N8O25S2	584.3585	0.5	34.8	62	63.49	-10.0	even	ok
	61	C41H113N2NaO32	584.3582	0.0	34.8	63	71.98	-13.0	even	ok
	62	C44H118N2O25P2S	584.3604	3.9	35.0	64	18.11	-11.0	even	ok
	63	C30H89N36NaO10S	584.3585	0.6	35.1	65	61.83	5.0	even	ok
	64	C34H98N28O11P2S	584.3577	-0.7	35.2	66	60.57	2.0	even	ok
	65	C34H93N30NaO12S	584.3598	2.9	35.5	68	28.23	4.0	even	ok
	66	C38H112N4O34	584.3573	-1.3	35.6	69	49.24	-14.0	even	ok
	67	C43H112N2O32	584.3594	2.1	35.8	70	38.17	-10.0	even	ok
	68	C41H118O31P2	584.3561	-3.5	35.9	71	21.17	-15.0	even	ok
	69	C33H94N36O3P2S2	584.3608	4.5	36.0	72	13.28	7.0	even	ok
	70	C43H115N6NaO21P2S	584.3599	3.0	36.1	73	26.54	-9.0	even	ok
	71	C41H110N12O19P2S	584.3598	2.8	36.2	74	29.22	-5.0	even	ok
	72	C42H117N2NaO27S2	584.3586	0.8	36.2	75	57.31	-14.0	even	ok
	73	C32H2N3NaO37P2S2	584.3585	0.7	36.3	76	59.04	35.0	even	ok
	74	C39H108N8O30	584.3580	-0.2	36.3	77	66.43	-9.0	even	ok
	75	C35H99N30NaO5P2S2	584.3609	4.7	36.3	78	11.60	3.0	even	ok

Compound Spectrum SmartFormula Report

Meas. m/z	#	Ion Formula	m/z	err [ppm]	mSigma	# mSigma	Score	rdb	e¯ Conf	N-Rule
	76	C32H88N36O10S	584.3597	2.7	36.4	79	30.33	8.0	even	ok
	77	C34H94N30NaO10PS	584.3557	-4.2	36.5	80	15.62	4.0	even	ok
	78	C28H84N42O8S	584.3584	0.4	36.6	81	63.26	9.0	even	ok
	79	C45H117O28PS	584.3589	1.4	36.7	82	47.61	-11.0	even	ok
	80	C43H113N2O30P	584.3552	-5.0	37.0	83	10.10	-10.0	even	ok
	81	C42H109N10O22PS	584.3583	0.2	37.1	84	64.81	-5.0	even	ok
	82	C40H114N4NaO29P	584.3597	2.6	37.3	85	30.44	-13.0	even	ok
	83	C42H113N4O29P	584.3609	4.7	37.3	86	11.81	-10.0	even	ok
	84	C44H114N4NaO24PS	584.3584	0.5	37.4	87	60.66	-9.0	even	ok
	85	C37H102N24O10P2S2	584.3558	-4.0	37.6	88	16.67	1.0	even	ok
	86	C32H89N36O8PS	584.3556	-4.4	37.6	89	13.46	8.0	even	ok
	87	C34H93N34O6PS2	584.3593	1.9	37.7	90	38.41	7.0	even	ok
	88	C40H107N16NaO15P2S	584.3592	1.9	37.8	91	39.31	-3.0	even	ok
	89	C36H99N28NaO6P2S2	584.3553	-4.9	37.8	92	10.33	3.0	even	ok
	90	C33H95N32NaO7P2S	584.3572	-1.6	37.9	93	42.92	4.0	even	ok
	91	C31H85N44PS2	584.3586	0.8	38.1	94	54.44	13.0	even	ok
	92	C37H101N24O12PS2	584.3599	3.1	38.2	95	24.30	1.0	even	ok
	93	C38H102N22O13P2S	584.3591	1.6	38.2	96	42.13	1.0	even	ok
	94	C36H98N28NaO8PS2	584.3594	2.2	38.3	97	34.72	3.0	even	ok
	95	C33H90N38NaO2PS2	584.3587	1.0	38.4	98	50.53	9.0	even	ok
	96	C39H107N18NaO12P2S2	584.3560	-3.7	38.4	99	18.26	-3.0	even	ok
	97	C41H106N14NaO18PS	584.3577	-0.7	38.4	100	55.30	-3.0	even	ok
	98	C39H101N20O16PS	584.3576	-0.9	38.5	101	51.54	1.0	even	ok
	99	C42H109N6NaO28	584.3588	1.2	38.5	102	47.98	-8.0	even	ok
	100	C37H109N8NaO30	584.3568	-2.3	38.6	103	33.36	-12.0	even	ok
	101	C43H108N8O25S	584.3568	-2.3	38.6	104	32.34	-5.0	even	ok
	102	C40H110N14O16P2S2	584.3565	-2.8	38.7	105	26.97	-5.0	even	ok
	103	C42H114N4O27P2	584.3567	-2.4	38.7	106	31.63	-10.0	even	ok
	104	C40H115N4NaO27P2	584.3555	-4.5	39.0	107	12.51	-13.0	even	ok
	105	C38H109N10O27P	584.3595	2.4	39.0	108	31.88	-9.0	even	ok
	106	C31H85N40NaO6S	584.3592	1.7	39.1	109	39.42	10.0	even	ok
	107	C45H113N2NaO27S	584.3569	-2.1	39.1	110	34.84	-9.0	even	ok
	108	C39H106N18NaO14PS2	584.3601	3.3	39.2	111	21.37	-3.0	even	ok
	109	C31H90N38O5P2S	584.3571	-1.8	39.2	112	38.29	8.0	even	ok
	110	C40H100N18O19S	584.3561	-3.5	39.4	113	19.72	1.0	even	ok
	111	C38H105N12NaO26	584.3575	-1.1	39.4	114	47.77	-7.0	even	ok
	112	C42H105N12NaO21S	584.3562	-3.3	39.5	115	21.84	-3.0	even	ok
	113	C40H109N14O18PS2	584.3606	4.2	39.6	116	13.71	-5.0	even	ok
	114	C32H84N42O3S2	584.3571	-1.8	39.7	117	38.51	13.0	even	ok
	115	C27H81N46NaO4S	584.3578	-0.5	39.7	118	55.58	11.0	even	ok
	116	C40H104N12O26	584.3587	0.9	39.8	119	49.79	-4.0	even	ok
	117	C42H115N8NaO18P2S2	584.3566	-2.6	39.8	120	28.75	-9.0	even	ok
	118	C35H92N32O9S2	584.3578	-0.6	39.9	121	54.25	7.0	even	ok
	119	C34H89N36NaO5S2	584.3572	-1.5	40.2	122	41.11	9.0	even	ok
	120	C36H92N30O12S	584.3610	5.0	40.2	123	9.26	7.0	even	ok
	121	C41H110N8NaO25P	584.3603	3.7	40.3	124	17.22	-8.0	even	ok

Compound Spectrum SmartFormula Report

Meas. m/z	#	Ion Formula	m/z	err [ppm]	mSigma	# mSigma	Score	rdb	e⁻ Conf	N-Rule
	122	C38H98N24NaO12PS	584.3571	-1.9	40.3	125	36.87	3.0	even	ok
	123	C29H80N46O4S	584.3590	1.5	40.3	126	41.29	14.0	even	ok
	124	C37H99N26NaO9P2S	584.3586	0.7	40.3	127	52.34	3.0	even	ok
	125	C35H104N14O28	584.3567	-2.5	40.5	128	28.98	-8.0	even	ok
	126	C43H118N4O22P2S2	584.3572	-1.7	40.6	129	39.02	-11.0	even	ok
	127	C37H97N26NaO11S2	584.3579	-0.4	40.7	130	56.43	3.0	even	ok
	128	C38H110N10O25P2	584.3554	-4.7	40.7	131	10.52	-9.0	even	ok
	129	C30H86N42NaO3PS	584.3607	4.3	40.7	132	12.88	10.0	even	ok
	130	C36H93N30O10PS	584.3569	-2.1	40.7	133	33.49	7.0	even	ok
	131	C39H97N22NaO15S	584.3556	-4.4	40.8	134	12.21	3.0	even	ok
	132	C42H114N8NaO20PS2	584.3608	4.5	40.9	135	11.73	-9.0	even	ok
	133	C37H92N28O13S	584.3554	-4.7	41.0	136	10.72	7.0	even	ok
	134	C38H100N22O15S2	584.3584	0.5	41.0	137	53.91	1.0	even	ok
	135	C35H94N32O7P2S	584.3584	0.5	41.0	138	54.71	7.0	even	ok
	136	C36H100N18O24	584.3573	-1.4	41.1	139	42.42	-3.0	even	ok
	137	C46H113NaO30	584.3602	3.5	41.4	140	18.81	-9.0	even	ok
	138	C33H92N28O18	584.3567	-2.5	41.7	141	22.36	3.0	even	ok
	139	C39H105N14O23P	584.3602	3.5	41.7	142	18.48	-4.0	even	ok
	140	C44H116N2O27S2	584.3598	2.8	41.7	143	29.44	-11.0	even	ok
	141	C41H111N8NaO23P2	584.3562	-3.3	41.7	144	20.06	-8.0	even	ok
	142	C37H110N12O24P2	584.3610	4.9	42.0	145	9.03	-9.0	even	ok
	143	C44H108N6O28	584.3600	3.2	42.1	146	20.53	-5.0	even	ok
	144	C28H81N48OPS	584.3605	4.1	42.1	147	13.97	14.0	even	ok
	145	C40H105N16NaO17S2	584.3586	0.8	42.1	148	48.96	-3.0	even	ok
	146	C36H97N24O17P	584.3595	2.3	42.1	149	23.47	2.0	even	ok
	147	C30H87N42NaOP2S	584.3565	-2.7	42.2	150	25.15	10.0	even	ok
	148	C46H114NaO28P	584.3560	-3.6	42.2	151	17.63	-9.0	even	ok
	149	C38H103N18NaO17P2	584.3555	-4.5	42.2	152	9.13	-2.0	even	ok
	150	C37H106N14NaO23P	584.3590	1.4	42.2	153	40.04	-7.0	even	ok
	151	C35H89N34NaO8S	584.3605	4.0	42.2	154	14.09	9.0	even	ok
	152	C39H101N16NaO22	584.3582	0.0	42.7	155	58.16	-2.0	even	ok
	153	C33H84N40O6S	584.3604	3.8	42.8	156	15.53	13.0	even	ok
	154	C45H115N2NaO25P2	584.3575	-1.0	42.8	157	44.76	-9.0	even	ok
	155	C41H108N12O21S2	584.3591	1.7	42.9	158	36.28	-5.0	even	ok
	156	C35H90N34NaO6PS	584.3564	-3.0	42.9	159	22.12	9.0	even	ok
	157	C44H109N6O26P	584.3559	-3.8	42.9	160	15.44	-5.0	even	ok
	158	C34H102N22O18P2	584.3603	3.8	43.0	161	12.65	-3.0	even	ok
	159	C34H98N24NaO17P	584.3583	0.3	43.2	162	43.17	-1.0	even	ok
	160	C39H106N14O21P2	584.3561	-3.6	43.2	163	17.30	-4.0	even	ok
	161	C28H77N50NaS	584.3585	0.6	43.2	164	49.74	16.0	even	ok
	162	C36H93N26NaO16	584.3575	-1.1	43.4	165	34.10	4.0	even	ok
	163	C34H91N36NaO3P2S	584.3579	-0.4	43.5	166	51.31	9.0	even	ok
	164	C34H101N18NaO24	584.3561	-3.4	43.6	167	18.18	-6.0	even	ok
	165	C33H85N40O4PS	584.3562	-3.2	43.7	168	19.56	13.0	even	ok
	166	C43H110N8O23P2	584.3574	-1.3	43.8	169	40.52	-5.0	even	ok
	167	C45H114N6O21P2S	584.3611	5.1	43.9	170	7.84	-6.0	even	ok

Compound Spectrum SmartFormula Report

Meas. m/z	#	Ion Formula	m/z	err [ppm]	mSigma	# mSigma	Score	rdb	e⁻ Conf	N-Rule
	168	C35H101N20O21P	584.3588	1.2	44.0	172	40.99	-3.0	even	ok
	169	C37H96N22O20	584.3580	-0.2	44.1	173	53.47	2.0	even	ok
	170	C36H98N24O15P2	584.3554	-4.7	44.1	174	7.61	2.0	even	ok
	171	C44H117N2O25PS2	584.3557	-4.2	44.1	175	12.27	-11.0	even	ok
	172	C43H113N6NaO23S2	584.3593	1.9	44.3	176	32.14	-9.0	even	ok
	173	C35H97N22NaO20	584.3568	-2.3	44.3	177	28.38	-1.0	even	ok
	174	C32H86N42OP2S	584.3577	-0.7	44.5	178	46.87	13.0	even	ok
	175	C43H105N10NaO24	584.3595	2.3	44.5	179	27.67	-3.0	even	ok
	176	C42H106N16O15P2S	584.3604	3.9	44.7	180	13.97	0.0	even	ok
	177	C38H102N18NaO19P	584.3596	2.6	44.8	181	24.83	-2.0	even	ok
	178	C44H111N10NaO17P2S	584.3606	4.2	44.8	182	12.38	-4.0	even	ok
	179	C31H93N28NaO18	584.3555	-4.6	45.1	183	7.96	0.0	even	ok
	180	C35H98N26O14P2	584.3610	4.9	45.2	184	6.63	2.0	even	ok
	181	C32H81N44NaO2S	584.3598	2.9	45.2	185	21.70	15.0	even	ok
	182	C36H107N16NaO20P2	584.3605	4.0	45.2	186	13.21	-7.0	even	ok
	183	C34H88N32O14	584.3573	-1.4	45.3	187	30.06	8.0	even	ok
	184	C41H100N16O22	584.3594	2.1	45.4	188	29.41	1.0	even	ok
	185	C42H106N12NaO21P	584.3610	4.9	45.4	189	8.32	-3.0	even	ok
	186	C46H114N4O22P2S	584.3555	-4.5	45.4	190	10.00	-6.0	even	ok
	187	C32H93N30O15P	584.3582	0.0	45.5	192	42.82	3.0	even	ok
	188	C43H106N10NaO22P	584.3554	-4.7	45.5	193	9.04	-3.0	even	ok
	189	C32H96N24O22	584.3560	-3.7	45.5	194	15.43	-2.0	even	ok
	190	C32H89N32NaO14	584.3561	-3.4	45.8	195	13.59	5.0	even	ok
	191	C46H113N4O24PS	584.3596	2.5	45.8	196	24.79	-6.0	even	ok
	192	C43H105N14O18PS	584.3589	1.4	46.1	197	36.76	0.0	even	ok
	193	C30H76N50S	584.3597	2.7	46.1	198	23.29	19.0	even	ok
	194	C41H103N20NaO11P2S	584.3599	3.0	46.1	199	20.20	2.0	even	ok
	195	C35H94N28NaO13P	584.3590	1.4	46.2	200	28.65	4.0	even	ok
	196	C32H82N44NaPS	584.3557	-4.2	46.2	201	11.89	15.0	even	ok
	197	C39H98N26O9P2S	584.3598	2.8	46.3	202	22.18	6.0	even	ok
	198	C39H98N22NaO15P	584.3603	3.7	46.5	203	11.60	3.0	even	ok
	199	C45H110N8NaO20PS	584.3591	1.6	46.5	204	33.69	-4.0	even	ok
	200	C35H90N38P2S2	584.3558	-4.0	46.5	205	12.93	12.0	even	ok
	201	C41H101N16O20P	584.3552	-5.0	46.5	206	7.71	1.0	even	ok
	202	C40H101N18O19P	584.3609	4.6	46.6	207	9.17	1.0	even	ok
	203	C42H107N12NaO19P2	584.3569	-2.2	46.6	208	27.54	-3.0	even	ok
	204	C38H98N28O6P2S2	584.3565	-2.8	46.8	209	21.39	6.0	even	ok
	205	C35H89N38O2PS2	584.3599	3.1	46.9	211	19.11	12.0	even	ok
	206	C37H95N32NaO2P2S2	584.3560	-3.7	47.1	212	14.24	8.0	even	ok
	207	C40H97N24O12PS	584.3583	0.2	47.1	213	49.15	6.0	even	ok
	208	C33H99N26NaO14P2	584.3598	2.8	47.1	214	16.74	-1.0	even	ok
	209	C42H102N18NaO14PS	584.3584	0.4	47.2	215	46.18	2.0	even	ok
	210	C38H97N28O8PS2	584.3606	4.2	47.5	216	11.03	6.0	even	ok
	211	C37H94N32NaO4PS2	584.3601	3.3	47.6	217	16.90	8.0	even	ok
	212	C40H103N22NaO8P2S2	584.3566	-2.6	47.6	218	22.93	2.0	even	ok
	213	C47H114N2O25P2	584.3587	1.0	47.7	219	38.57	-6.0	even	ok

Compound Spectrum SmartFormula Report

Meas. m/z	#	Ion Formula	m/z	err [ppm]	mSigma	# mSigma	Score	rdb	e⁻ Conf	N-Rule
	214	C40H102N18O17P2	584.3567	-2.4	47.8	221	24.34	1.0	even	ok
	215	C41H106N18O12P2S2	584.3572	-1.7	47.8	222	31.67	0.0	even	ok
	216	C44H104N12O21S	584.3574	-1.2	47.9	223	36.58	0.0	even	ok
	217	C37H93N28O13P	584.3602	3.5	47.9	224	12.40	7.0	even	ok
	218	C48H113O28P	584.3573	-1.5	47.9	225	33.12	-6.0	even	ok
	219	C39H99N22NaO13P2	584.3562	-3.3	48.0	226	13.33	3.0	even	ok
	220	C38H95N30NaO5P2S	584.3592	1.9	48.1	227	29.50	8.0	even	ok
	221	C30H84N38O12	584.3560	-3.7	48.1	228	11.40	9.0	even	ok
	222	C40H97N20NaO18	584.3588	1.2	48.1	229	36.64	3.0	even	ok
	223	C33H89N34O11P	584.3588	1.2	48.2	230	29.08	8.0	even	ok
	224	C47H112N2O27S	584.3581	-0.0	48.2	231	49.40	-6.0	even	ok
	225	C41H96N22O15S	584.3568	-2.4	48.4	232	24.44	6.0	even	ok
	226	C40H102N22NaO10PS2	584.3608	4.5	48.4	233	9.49	2.0	even	ok
	227	C46H109N6NaO23S	584.3576	-1.0	48.5	234	38.57	-4.0	even	ok
	228	C36H90N36O3P2S	584.3591	1.6	48.5	235	31.55	12.0	even	ok
	229	C39H94N28NaO8PS	584.3577	-0.7	48.6	236	41.25	8.0	even	ok
	230	C43H101N16NaO17S	584.3569	-2.1	48.7	237	26.44	2.0	even	ok
	231	C37H89N34O6PS	584.3576	-0.9	48.8	238	38.34	12.0	even	ok
	232	C43H111N12NaO14P2S2	584.3573	-1.4	48.9	239	33.07	-4.0	even	ok
	233	C38H92N26O16	584.3587	0.9	49.2	240	38.02	7.0	even	ok
	234	C36H88N36O5S2	584.3584	0.5	49.3	241	42.51	12.0	even	ok
	235	C33H85N36NaO10	584.3568	-2.3	49.3	242	19.56	10.0	even	ok
	236	C34H95N30NaO10P2	584.3605	4.0	49.3	243	9.41	4.0	even	ok
	237	C47H109N4NaO26	584.3608	4.6	49.4	244	8.53	-4.0	even	ok
	238	C35H85N40NaOS2	584.3579	-0.4	49.5	245	43.60	14.0	even	ok
	239	C38H88N32O9S	584.3561	-3.5	49.6	246	14.65	12.0	even	ok
	240	C37H94N28O11P2	584.3561	-3.6	49.6	247	11.43	7.0	even	ok
	241	C40H93N26NaO11S	584.3562	-3.3	49.6	248	16.28	8.0	even	ok
	242	C44H114N8O18P2S2	584.3578	-0.5	49.6	249	42.22	-6.0	even	ok
	243	C31H90N34NaO11P	584.3576	-0.9	49.6	250	30.63	5.0	even	ok
	244	C45H104N10O24	584.3607	4.4	49.8	251	9.55	0.0	even	ok
	245	C45H112N6O23S2	584.3605	4.0	49.8	253	13.98	-6.0	even	ok
	246	C37H89N30NaO12	584.3581	0.0	49.8	254	37.94	9.0	even	ok
	247	C47H110N4NaO24P	584.3567	-2.4	49.9	255	22.68	-4.0	even	ok
	248	C46H111N6NaO21P2	584.3582	0.1	49.9	256	45.99	-4.0	even	ok
	249	C47H118NaO23PS2	584.3565	-2.8	49.9	257	23.21	-10.0	even	ok
	250	C38H93N30NaO7S2	584.3586	0.8	50.1	258	38.81	8.0	even	ok
	251	C48H109N2NaO27	584.3552	-5.0	50.3	259	6.80	-4.0	even	ok
	252	C39H96N26O11S2	584.3591	1.7	50.3	260	29.26	6.0	even	ok
	253	C45H105N10O22P	584.3566	-2.7	50.4	261	20.36	0.0	even	ok
	254	C34H80N44O2S	584.3610	5.0	50.6	262	6.87	18.0	even	ok
	255	C44H106N12O19P2	584.3581	-0.1	50.6	263	45.21	0.0	even	ok
	256	C36H86N38NaO2PS	584.3570	-1.9	50.7	264	27.13	14.0	even	ok
	257	C42H105N16O15PS2	584.3557	-4.2	50.9	265	9.98	0.0	even	ok
	258	C46H119N2NaO20P2S2	584.3580	-0.3	51.0	266	43.04	-10.0	even	ok
	259	C37H85N36NaO5S	584.3556	-4.4	51.2	267	8.97	14.0	even	ok

Compound Spectrum SmartFormula Report

Meas. m/z	#	Ion Formula	m/z	err [ppm]	mSigma	# mSigma	Score	rdb	e¯ Conf	N-Rule
	260	C34H81N44PS	584.3569	-2.1	51.2	268	24.59	18.0	even	ok
	261	C47H117NaO25S2	584.3606	4.2	51.2	269	11.89	-10.0	even	ok
	262	C31H80N42O8	584.3567	-2.5	51.2	270	16.83	14.0	even	ok
	263	C35H84N36O10	584.3580	-0.2	51.2	271	34.56	13.0	even	ok
	264	C32H90N36O8P2	584.3603	3.8	51.4	272	9.88	8.0	even	ok
	265	C35H80N42O3S	584.3554	-4.7	51.4	273	7.85	18.0	even	ok
	266	C41H101N20NaO13S2	584.3593	1.9	51.4	274	26.06	2.0	even	ok
	267	C36H90N32NaO9P	584.3596	2.6	51.6	275	16.25	9.0	even	ok
	268	C44H101N14NaO20	584.3602	3.5	51.9	276	13.86	2.0	even	ok
	269	C29H85N40O9P	584.3575	-1.1	52.0	277	26.61	9.0	even	ok
	270	C42H104N16O17S2	584.3598	2.8	52.1	278	18.14	0.0	even	ok
	271	C44H110N10NaO17PS2	584.3558	-4.0	52.2	280	10.79	-4.0	even	ok
	272	C29H81N42NaO8	584.3555	-4.6	52.3	281	6.39	11.0	even	ok
	273	C32H86N38NaO7P	584.3583	0.3	52.3	282	32.97	10.0	even	ok
	274	C41H98N22O13P2	584.3574	-1.3	52.5	283	24.96	6.0	even	ok
	275	C42H96N20O18	584.3600	3.2	52.5	284	15.10	6.0	even	ok
	276	C44H102N14NaO18P	584.3560	-3.6	52.7	285	12.86	2.0	even	ok
	277	C45H113N6O21PS2	584.3563	-3.1	53.1	286	15.88	-6.0	even	ok
	278	C43H103N16NaO15P2	584.3575	-1.0	53.2	287	32.80	2.0	even	ok
	279	C34H85N38O7P	584.3595	2.3	53.3	288	16.91	13.0	even	ok
	280	C36H91N32NaO7P2	584.3555	-4.5	53.4	289	6.51	9.0	even	ok
	281	C42H97N20O16P	584.3559	-3.8	53.4	290	11.24	6.0	even	ok
	282	C44H109N10NaO19S2	584.3599	3.1	53.4	291	15.74	-4.0	even	ok
	283	C28H81N44NaO7	584.3611	5.0	53.8	292	4.75	11.0	even	ok
	284	C43H102N20O11P2S	584.3611	5.1	54.0	294	5.82	5.0	even	ok
	285	C27H2N5NaO39P2S2	584.3565	-2.8	54.3	295	17.27	31.0	even	ok
	286	C30H81N44O5P	584.3582	0.0	54.4	296	32.72	14.0	even	ok
	287	C40H94N26NaO11P	584.3610	4.9	54.4	297	5.08	8.0	even	ok
	288	C27H76N48O6	584.3553	-4.8	54.6	298	5.23	15.0	even	ok
	289	C39H88N30O12	584.3594	2.1	54.7	299	17.76	12.0	even	ok
	290	C41H94N24NaO12P	584.3554	-4.7	54.9	300	5.41	8.0	even	ok
	291	C34H81N40NaO6	584.3575	-1.1	54.9	301	24.01	15.0	even	ok
	292	C41H93N24NaO14	584.3595	2.3	55.0	302	20.17	8.0	even	ok
	293	C28H5NO43P2S2	584.3570	-1.9	55.0	303	23.70	29.0	even	ok
	294	C34H86N38O5P2	584.3554	-4.7	55.1	304	5.44	13.0	even	ok
	295	C47H110N8O18P2S	584.3562	-3.4	55.1	305	12.94	-1.0	even	ok
	296	C40H94N30O5P2S	584.3604	3.9	55.2	306	10.20	11.0	even	ok
	297	C42H99N24NaO7P2S	584.3606	4.1	55.2	307	9.06	7.0	even	ok
	298	C30H77N46NaO4	584.3561	-3.4	55.3	308	10.12	16.0	even	ok
	299	C44H102N18O12P2S	584.3555	-4.5	55.4	309	7.33	5.0	even	ok
	300	C38H89N32O9P	584.3608	4.6	55.5	310	5.58	12.0	even	ok
	301	C31H87N40NaO4P2	584.3598	2.8	55.6	311	12.92	10.0	even	ok
	302	C47H109N8O20PS	584.3603	3.7	55.6	312	11.32	-1.0	even	ok
	303	C44H101N18O14PS	584.3596	2.5	55.7	313	18.40	5.0	even	ok
	304	C40H95N26NaO9P2	584.3569	-2.2	55.7	314	16.62	8.0	even	ok
	305	C33H86N40O4P2	584.3610	4.9	55.7	315	4.82	13.0	even	ok

Compound Spectrum SmartFormula Report

Meas. m/z	#	Ion Formula	m/z	err [ppm]	mSigma	# mSigma	Score	rdb	e¯ Conf	N-Rule
	306	C49H115N2NaO20P2S	584.3563	-3.2	55.8	316	14.07	-5.0	even	ok
	307	C46H107N12NaO14P2S	584.3556	-4.3	55.8	317	8.19	1.0	even	ok
	308	C39H89N30O10P	584.3552	-5.0	55.8	318	4.60	12.0	even	ok
	309	C50H115NaO23P2	584.3596	2.4	55.9	319	18.82	-5.0	even	ok
	310	C48H110N6O21P2	584.3594	2.2	56.1	320	20.41	-1.0	even	ok
	311	C46H106N12NaO16PS	584.3597	2.7	56.1	321	16.49	1.0	even	ok
	312	C28H82N44NaO5P	584.3570	-2.0	56.1	322	17.32	11.0	even	ok
	313	C26H76N50O5	584.3609	4.8	56.2	323	5.03	15.0	even	ok
	314	C49H114N2NaO22PS	584.3604	3.9	56.3	324	9.88	-5.0	even	ok
	315	C41H93N28O8PS	584.3589	1.4	56.4	326	26.82	11.0	even	ok
	316	C32H76N46O4	584.3573	-1.4	56.5	327	21.18	19.0	even	ok
	317	C39H94N32O2P2S2	584.3572	-1.7	56.5	328	24.04	11.0	even	ok
	318	C43H98N22NaO10PS	584.3591	1.6	56.6	329	24.67	7.0	even	ok
	319	C39H91N34NaOP2S	584.3599	3.0	56.8	330	14.57	13.0	even	ok
	320	C49H109N4O24P	584.3579	-0.4	56.8	331	34.86	-1.0	even	ok
	321	C38H90N32O7P2	584.3567	-2.4	56.8	332	14.63	12.0	even	ok
	322	C33H82N42NaO3P	584.3590	1.4	57.1	333	20.46	15.0	even	ok
	323	C28H72N52O2	584.3560	-3.7	57.3	334	8.50	20.0	even	ok
	324	C38H90N36NaPS2	584.3607	4.5	57.4	335	7.19	13.0	even	ok
	325	C41H99N26NaO4P2S2	584.3573	-1.4	57.4	336	25.23	7.0	even	ok
	326	C42H102N22O8P2S2	584.3578	-0.5	57.6	337	32.71	5.0	even	ok
	327	C40H90N32NaO4PS	584.3584	0.4	57.7	338	33.29	13.0	even	ok
	328	C45H100N16O17S	584.3581	-0.1	57.7	339	36.56	5.0	even	ok
	329	C38H85N38O2PS	584.3583	0.2	57.7	340	35.32	17.0	even	ok
	330	C29H82N46O2P2	584.3597	2.6	57.8	341	13.24	14.0	even	ok
	331	C38H85N34NaO8	584.3588	1.2	57.8	342	21.68	14.0	even	ok
	332	C50H108N2O27	584.3564	-2.9	57.9	343	14.43	-1.0	even	ok
	333	C42H92N26O11S	584.3574	-1.2	58.0	344	26.59	11.0	even	ok
	334	C47H107N10NaO17P2	584.3589	1.3	58.1	345	25.98	1.0	even	ok
	335	C48H108N6O23S	584.3588	1.1	58.1	346	27.37	-1.0	even	ok
	336	C47H105N10NaO19S	584.3582	0.2	58.4	347	34.69	1.0	even	ok
	337	C44H97N20NaO13S	584.3576	-1.0	58.4	348	28.15	7.0	even	ok
	338	C26H77N50O3P	584.3568	-2.3	58.5	349	14.71	15.0	even	ok
	339	C29H78N48NaOP	584.3576	-0.9	58.5	350	23.04	16.0	even	ok
	340	C48H106N8NaO20P	584.3574	-1.3	58.5	351	25.50	1.0	even	ok
	341	C45H102N16O15P2	584.3587	1.0	58.5	352	27.48	5.0	even	ok
	342	C44H107N16NaO10P2S2	584.3580	-0.3	58.7	353	33.51	1.0	even	ok
	343	C46H101N14O18P	584.3572	-1.5	58.8	354	23.44	5.0	even	ok
	344	C39H84N36O5S	584.3568	-2.4	58.8	355	17.48	17.0	even	ok
	345	C37H86N36NaO5P	584.3603	3.7	58.9	356	7.89	14.0	even	ok
	346	C36H80N40O6	584.3587	0.9	58.9	357	22.43	18.0	even	ok
	347	C31H77N48OP	584.3588	1.2	58.9	358	20.77	19.0	even	ok
	348	C50H113NaO25S	584.3589	1.3	59.0	359	24.71	-5.0	even	ok
	349	C41H89N30NaO7S	584.3569	-2.1	59.0	360	18.98	13.0	even	ok
	350	C37H84N40OS2	584.3591	1.7	59.2	361	22.11	17.0	even	ok
	351	C45H110N12O14P2S2	584.3585	0.6	59.2	362	30.07	-1.0	even	ok

Compound Spectrum SmartFormula Report

Meas. m/z	#	Ion Formula	m/z	err [ppm]	mSigma	# mSigma	Score	rdb	e¯ Conf	N-Rule
	352	C40H93N30O5PS2	584.3557	-4.2	59.3	363	7.59	11.0	even	ok
	353	C49H105N6NaO23	584.3559	-3.8	59.3	364	9.17	1.0	even	ok
	354	C28H2N3NaO42P2S	584.3598	2.8	59.3	365	14.48	31.0	even	ok
	355	C47H100N12O21	584.3558	-4.1	59.4	366	8.14	5.0	even	ok
	356	C49H117O23PS2	584.3577	-0.8	59.6	367	34.25	-7.0	even	ok
	357	C32H83N44NaP2	584.3605	4.0	59.7	368	6.78	15.0	even	ok
	358	C39H89N34NaO3S2	584.3593	1.9	60.1	369	19.78	13.0	even	ok
	359	C35H81N42O3P	584.3602	3.5	60.2	370	8.43	18.0	even	ok
	360	C40H92N30O7S2	584.3598	2.8	60.2	371	13.98	11.0	even	ok
	361	C38H81N40NaOS	584.3562	-3.3	60.3	372	11.51	19.0	even	ok
	362	C37H87N36NaO3P2	584.3562	-3.3	60.3	373	8.99	14.0	even	ok
	363	C45H97N18NaO16	584.3608	4.6	60.3	374	6.04	7.0	even	ok
	364	C25H73N54NaO	584.3604	3.9	60.3	375	6.97	17.0	even	ok
	365	C42H98N24NaO7PS2	584.3558	-4.0	60.4	376	8.25	7.0	even	ok
	366	C31H73N50Na	584.3568	-2.3	60.4	377	13.65	21.0	even	ok
	367	C47H115N6NaO16P2S2	584.3586	0.9	60.5	378	26.92	-5.0	even	ok
	368	C43H92N24O14	584.3607	4.4	60.7	379	6.75	11.0	even	ok
	369	C47H108N8O20S2	584.3555	-4.5	60.7	380	7.64	-1.0	even	ok
	370	C43H101N20O11PS2	584.3563	-3.1	60.7	381	12.33	5.0	even	ok
	371	C45H98N18NaO14P	584.3567	-2.4	60.8	382	15.89	7.0	even	ok
	372	C44H99N20NaO11P2	584.3582	0.1	61.1	383	25.74	7.0	even	ok
	373	C46H97N16NaO17	584.3552	-5.0	61.2	384	4.75	7.0	even	ok
	374	C42H97N24NaO9S2	584.3599	3.1	61.3	385	12.19	7.0	even	ok
	375	C43H93N24O12P	584.3566	-2.7	61.3	386	14.24	11.0	even	ok
	376	C48H118N2O20P2S2	584.3592	1.8	61.4	387	19.68	-7.0	even	ok
	377	C35H82N42OP2	584.3561	-3.6	61.6	388	7.70	18.0	even	ok
	378	C42H94N26O9P2	584.3581	-0.1	61.7	389	25.18	11.0	even	ok
	379	C43H100N20O13S2	584.3605	4.0	61.8	390	7.93	5.0	even	ok
	380	C45H106N14NaO13PS2	584.3565	-2.8	62.0	391	13.04	1.0	even	ok
	381	C49H113N2NaO22S2	584.3557	-4.2	62.1	392	8.23	-5.0	even	ok
	382	C35H77N44NaO2	584.3581	0.0	62.2	393	25.38	20.0	even	ok
	383	C46H109N10O17PS2	584.3570	-1.9	62.7	394	17.94	-1.0	even	ok
	384	C45H105N14NaO15S2	584.3606	4.2	63.1	395	6.73	1.0	even	ok
	385	C33H72N50	584.3580	-0.2	63.5	396	23.02	24.0	even	ok
	386	C42H89N28NaO10	584.3602	3.5	63.6	397	7.56	13.0	even	ok
	387	C52H114O23P2	584.3608	4.5	63.6	398	5.75	-2.0	even	ok
	388	C26H5NNaO45PS2	584.3600	3.1	63.8	399	10.86	26.0	even	ok
	389	C26H6NNaO43P2S2	584.3558	-3.9	64.1	400	7.50	26.0	even	ok
	390	C48H114N4NaO19PS2	584.3572	-1.7	64.1	401	18.51	-5.0	even	ok
	391	C40H84N34O8	584.3600	3.2	64.2	402	8.24	17.0	even	ok
	392	C42H90N28NaO8P	584.3560	-3.6	64.3	404	6.94	13.0	even	ok
	393	C41H91N30NaO5P2	584.3575	-1.0	64.5	405	17.92	13.0	even	ok
	394	C41H90N34OP2S	584.3611	5.1	64.6	406	4.09	16.0	even	ok
	395	C40H85N34O6P	584.3559	-3.8	65.0	408	6.07	17.0	even	ok
	396	C51H111N4NaO19P2	584.3602	3.6	65.1	409	8.54	0.0	even	ok
	397	C49H106N10O17P2	584.3601	3.3	65.2	410	9.48	4.0	even	ok

Compound Spectrum SmartFormula Report

Meas. m/z	#	Ion Formula	m/z	err [ppm]	mSigma	# mSigma	Score	rdb	e⁻ Conf	N-Rule
	398	C48H106N12O14P2S	584.3568	-2.3	65.2	411	14.65	4.0	even	ok
	399	C45H98N22O8P2S	584.3561	-3.4	65.3	412	9.13	10.0	even	ok
	400	C39H86N36O3P2	584.3574	-1.3	65.4	413	16.17	17.0	even	ok
	401	C51H114N2O20P2S	584.3575	-1.1	65.6	414	21.14	-2.0	even	ok
	402	C45H97N22O10PS	584.3603	3.6	65.6	415	8.09	10.0	even	ok
	403	C48H105N12O16PS	584.3609	4.8	65.7	416	4.53	4.0	even	ok
	404	C47H103N16NaO10P2S	584.3563	-3.2	65.8	417	9.96	6.0	even	ok
	405	C50H111N6NaO16P2S	584.3570	-2.0	65.9	418	15.58	0.0	even	ok
	406	C42H90N32O2P2S	584.3555	-4.6	66.0	419	5.09	16.0	even	ok
	407	C42H89N32O4PS	584.3596	2.5	66.1	420	12.93	16.0	even	ok
	408	C47H102N16NaO12PS	584.3604	3.9	66.2	421	7.09	6.0	even	ok
	409	C50H105N8O20P	584.3586	0.8	66.2	422	22.72	4.0	even	ok
	410	C44H95N26NaO4P2S	584.3556	-4.3	66.3	423	5.71	12.0	even	ok
	411	C52H110N2NaO22P	584.3587	1.0	66.3	424	21.15	0.0	even	ok
	412	C44H94N26NaO6PS	584.3597	2.7	66.4	425	11.64	12.0	even	ok
	413	C50H110N6NaO18PS	584.3611	5.0	66.5	426	3.86	0.0	even	ok
	414	C39H81N38NaO4	584.3595	2.3	66.9	428	10.79	19.0	even	ok
	415	C48H103N14NaO13P2	584.3595	2.4	66.9	429	12.94	6.0	even	ok
	416	C46H98N20O11P2	584.3594	2.2	67.2	430	14.00	10.0	even	ok
	417	C41H86N36NaPS	584.3591	1.6	67.3	431	17.14	18.0	even	ok
	418	C38H82N40NaOP	584.3610	4.9	67.3	432	3.29	19.0	even	ok
	419	C51H104N6O23	584.3571	-1.8	67.6	433	15.84	4.0	even	ok
	420	C42H95N30NaP2S2	584.3580	-0.3	67.6	434	24.53	12.0	even	ok
	421	C49H105N10O17PS	584.3553	-4.8	67.6	435	4.20	4.0	even	ok
	422	C43H98N26O4P2S2	584.3585	0.6	67.7	436	22.42	10.0	even	ok
	423	C37H76N44O2	584.3593	2.1	67.7	437	11.44	23.0	even	ok
	424	C49H102N12NaO16P	584.3581	-0.1	67.7	438	25.32	6.0	even	ok
	425	C46H96N20O13S	584.3588	1.1	67.8	439	19.57	10.0	even	ok
	426	C47H97N18O14P	584.3579	-0.4	67.8	440	23.77	10.0	even	ok
	427	C53H109NaO25	584.3572	-1.5	67.8	441	16.97	0.0	even	ok
	428	C39H82N38NaO2P	584.3554	-4.7	67.8	442	3.45	19.0	even	ok
	429	C43H88N30O7S	584.3581	-0.1	67.9	443	25.61	16.0	even	ok
	430	C49H104N10O19S	584.3594	2.2	68.3	444	13.14	4.0	even	ok
	431	C45H93N24NaO9S	584.3582	0.2	68.4	445	24.49	12.0	even	ok
	432	C52H113O23PS	584.3560	-3.7	68.4	446	7.28	-2.0	even	ok
	433	C51H110N4NaO19PS	584.3555	-4.6	68.5	447	4.63	0.0	even	ok
	434	C48H101N14NaO15S	584.3589	1.3	68.5	448	17.74	6.0	even	ok
	435	C40H80N40OS	584.3574	-1.2	68.6	449	18.33	22.0	even	ok
	436	C50H113N4O19PS2	584.3584	0.4	68.6	450	27.80	-2.0	even	ok
	437	C37H77N44P	584.3552	-5.0	68.7	451	2.93	23.0	even	ok
	438	C48H96N16O17	584.3564	-2.9	68.8	452	9.83	10.0	even	ok
	439	C45H103N20NaO6P2S2	584.3586	0.9	68.8	453	20.15	6.0	even	ok
	440	C42H85N34NaO3S	584.3576	-1.0	68.8	454	19.48	18.0	even	ok
	441	C50H101N10NaO19	584.3566	-2.7	68.8	455	10.81	6.0	even	ok
	442	C51H109N4NaO21S	584.3596	2.5	69.2	456	11.62	0.0	even	ok
	443	C46H106N16O10P2S2	584.3592	1.8	69.2	457	14.95	4.0	even	ok

Compound Spectrum SmartFormula Report

Meas. m/z	#	Ion Formula	m/z	err [ppm]	mSigma	# mSigma	Score	rdb	e¯ Conf	N-Rule
	444	C52H112O25S	584.3601	3.4	69.3	458	7.94	-2.0	even	ok
	445	C41H89N34OPS2	584.3563	-3.1	69.5	459	8.99	16.0	even	ok
	446	C46H94N22NaO10P	584.3574	-1.3	69.6	460	17.21	12.0	even	ok
	447	C47H93N20NaO13	584.3559	-3.9	70.4	462	6.18	12.0	even	ok
	448	C41H88N34O3S2	584.3605	4.0	70.4	463	5.86	16.0	even	ok
	449	C45H88N26O11	584.3557	-4.1	70.5	464	5.47	16.0	even	ok
	450	C48H111N10NaO12P2S2	584.3593	2.0	70.5	465	13.12	0.0	even	ok
	451	C43H94N28NaO3PS2	584.3565	-2.8	70.6	466	9.56	12.0	even	ok
	452	C44H97N24O7PS2	584.3570	-1.9	70.9	467	13.33	10.0	even	ok
	453	C50H109N6NaO18S2	584.3563	-3.1	71.2	468	10.09	0.0	even	ok
	454	C45H95N24NaO7P2	584.3589	1.3	71.3	469	13.07	12.0	even	ok
	455	C49H114N6O16P2S2	584.3598	2.9	71.3	470	8.99	-2.0	even	ok
	456	C43H93N28NaO5S2	584.3606	4.2	71.5	471	5.00	12.0	even	ok
	457	C43H90N30O5P2	584.3587	1.0	71.7	473	13.83	16.0	even	ok
	458	C46H102N18NaO9PS2	584.3571	-1.7	72.1	474	13.81	6.0	even	ok
	459	C51H112N2O22S2	584.3569	-2.2	72.2	475	13.92	-2.0	even	ok
	460	C44H89N28O8P	584.3572	-1.5	72.3	476	11.57	16.0	even	ok
	461	C24H3NO50S2	584.3577	-0.8	72.4	477	17.91	25.0	even	ok
	462	C47H105N14O13PS2	584.3577	-0.8	72.7	478	17.81	4.0	even	ok
	463	C51H119NaO18P2S2	584.3600	3.2	72.8	479	7.69	-6.0	even	ok
	464	C43H85N32NaO6	584.3608	4.6	74.0	481	2.96	18.0	even	ok
	465	C49H110N8NaO15PS2	584.3578	-0.5	74.1	482	18.01	0.0	even	ok
	466	C42H87N34NaOP2	584.3582	0.1	74.2	483	16.03	18.0	even	ok
	467	C45H96N22O10S2	584.3555	-4.5	74.3	484	3.88	10.0	even	ok
	468	C41H80N38O4	584.3607	4.4	74.3	485	3.31	22.0	even	ok
	469	C23H4N3O47PS2	584.3592	1.7	74.3	486	12.46	25.0	even	ok
	470	C43H86N32NaO4P	584.3567	-2.4	74.5	487	7.71	18.0	even	ok
	471	C50H102N14O13P2	584.3608	4.5	74.7	488	3.85	9.0	even	ok
	472	C52H107N8NaO15P2	584.3609	4.7	74.7	489	3.39	5.0	even	ok
	473	C41H81N38O2P	584.3566	-2.7	74.9	490	6.91	22.0	even	ok
	474	C54H110N2O20P2	584.3558	-4.0	75.0	491	4.88	3.0	even	ok
	475	C54H109N2O22P	584.3599	3.1	75.1	492	7.33	3.0	even	ok
	476	C44H85N30NaO7	584.3552	-5.0	75.2	493	2.27	18.0	even	ok
	477	C46H94N26O4P2S	584.3568	-2.3	75.5	495	9.96	15.0	even	ok
	478	C49H102N16O10P2S	584.3575	-1.1	75.5	496	14.60	9.0	even	ok
	479	C47H101N16NaO12S2	584.3557	-4.3	75.6	497	4.16	6.0	even	ok
	480	C46H93N26O6PS	584.3609	4.8	75.8	499	3.11	15.0	even	ok
	481	C51H101N12O16P	584.3593	1.9	76.0	500	11.04	9.0	even	ok
	482	C52H110N6O16P2S	584.3582	0.0	76.0	501	18.98	3.0	even	ok
	483	C48H99N20NaO6P2S	584.3570	-2.0	76.0	502	10.63	11.0	even	ok
	484	C21H2N9NaO40P2S2	584.3601	3.4	76.0	503	6.16	27.0	even	ok
	485	C53H107N6NaO16P2	584.3553	-4.9	76.1	504	2.91	5.0	even	ok
	486	C43H85N36PS	584.3603	3.6	76.1	505	5.48	21.0	even	ok
	487	C53H106N6NaO18P	584.3594	2.2	76.2	506	10.06	5.0	even	ok
	488	C51H107N10NaO12P2S	584.3576	-0.9	76.2	507	15.21	5.0	even	ok
	489	C49H99N18NaO9P2	584.3602	3.6	76.2	508	5.66	11.0	even	ok

Compound Spectrum SmartFormula Report

Meas. m/z	#	Ion Formula	m/z	err [ppm]	mSigma	# mSigma	Score	rdb	e¯ Conf	N-Rule
	490	C45H91N30NaP2S	584.3563	-3.2	76.3	509	6.69	17.0	even	ok
	491	C48H104N12O16S2	584.3562	-3.3	76.4	510	6.21	4.0	even	ok
	492	C48H98N20NaO8PS	584.3611	5.0	76.4	511	2.66	11.0	even	ok
	493	C22H5N5O44P2S2	584.3607	4.3	76.5	512	3.91	25.0	even	ok
	494	C45H90N30NaO2PS	584.3604	3.9	76.6	514	4.82	17.0	even	ok
	495	C40H77N42Na	584.3602	3.5	76.7	516	4.67	24.0	even	ok
	496	C54H115NaO18P2S	584.3583	0.3	76.9	517	17.26	-1.0	even	ok
	497	C55H108O25	584.3584	0.5	77.1	518	16.18	3.0	even	ok
	498	C48H93N22O10P	584.3586	0.8	77.3	519	15.00	15.0	even	ok
	499	C50H98N16NaO12P	584.3587	1.0	77.3	520	14.00	11.0	even	ok
	500	C52H100N10O19	584.3578	-0.6	77.6	521	15.33	9.0	even	ok
604.3549	1	C21H44N17NaOP	604.3545	-0.8	26.6	1	100.00	9.0	even	ok
	2	C23H43N17OP	604.3569	3.2	29.6	2	38.35	12.0	even	ok
	3	C24H42N15O4	604.3539	-1.8	30.0	3	90.20	12.0	even	ok
	4	C23H39N19Na	604.3528	-3.5	30.4	4	32.28	14.0	even	ok
	5	C17H44N21P2	604.3558	1.5	31.4	5	71.72	8.0	even	ok
	6	C18H43N19O3P	604.3528	-3.5	32.4	6	31.64	8.0	even	ok
	7	C19H49N15NaO2P2	604.3561	1.9	32.6	7	59.26	4.0	even	ok
	8	C20H48N13NaO5P	604.3531	-3.0	33.9	8	37.61	4.0	even	ok
	9	C25H38N19	604.3552	0.5	39.3	9	79.53	17.0	even	ok
	10	C15H45N21NaP2	604.3534	-2.5	40.4	10	39.13	5.0	even	ok
	11	C16H48N17O4P2	604.3545	-0.7	43.4	11	65.93	3.0	even	ok
	12	C20H43N19NaS	604.3562	2.1	45.1	12	54.22	9.0	even	ok
	13	C22H53N11NaOP2S	604.3523	-4.4	45.6	13	18.32	3.0	even	ok
	14	C18H48N17NaOPS	604.3578	4.8	45.8	14	14.66	4.0	even	ok
	15	C17H42N21O2S	604.3546	-0.6	46.6	15	82.66	8.0	even	ok
	16	C13H38N27S	604.3519	-5.1	47.6	16	11.88	9.0	even	ok
	17	C24H52N11OP2S	604.3547	-0.4	48.3	17	82.73	6.0	even	ok
	18	C19H47N15NaO4S	604.3548	-0.2	48.5	18	88.26	4.0	even	ok
	19	C15H43N21NaO2S	604.3522	-4.6	49.1	19	14.83	5.0	even	ok
	20	C21H46N15O4S	604.3572	3.8	49.1	20	22.33	7.0	even	ok
	21	C23H56N7O5P2S	604.3533	-2.6	49.6	21	37.71	1.0	even	ok
	22	C26H57N5NaO3P2S	604.3550	0.0	50.6	22	85.06	2.0	even	ok
	23	C23H51N9NaO6S	604.3575	4.3	51.3	23	16.44	3.0	even	ok
	24	C25H61NNaO7P2S	604.3536	-2.2	51.8	24	42.42	-3.0	even	ok
	25	C20H50N11O8S	604.3559	1.6	52.5	25	50.76	2.0	even	ok
	26	C16H46N17O6S	604.3532	-2.8	52.9	26	31.40	3.0	even	ok
	27	C22H60N3O9P2S	604.3520	-4.8	53.8	27	11.13	-4.0	even	ok
	28	C22H55N5NaO10S	604.3562	2.1	54.5	28	40.44	-2.0	even	ok
	29	C18H51N11NaO8S	604.3535	-2.4	54.5	29	36.09	-1.0	even	ok
	30	C27H60NO7P2S	604.3560	1.8	54.5	30	44.47	0.0	even	ok
	31	C26H47N13PS	604.3530	-3.2	54.6	31	25.78	11.0	even	ok
	32	C27H56N3NaO6PS	604.3520	-4.9	54.8	32	10.41	2.0	even	ok
	33	C28H56N5O3P2S	604.3574	4.0	56.0	33	16.29	5.0	even	ok
	34	C28H52N7NaO2PS	604.3533	-2.7	57.0	34	29.19	7.0	even	ok
	35	C20H60N3NaO11PS	604.3578	4.8	58.0	35	9.94	-7.0	even	ok

Compound Spectrum SmartFormula Report

Meas. m/z	#	Ion Formula	m/z	err [ppm]	mSigma	# mSigma	Score	rdb	e⁻ Conf	N-Rule
	36	C19H54N7O12S	604.3546	-0.6	58.4	36	57.37	-3.0	even	ok
	37	C23H58NO14S	604.3573	3.8	58.5	37	16.40	-4.0	even	ok
	38	C21H59NNaO14S	604.3548	-0.1	60.1	38	61.26	-7.0	even	ok
	39	C29H55N3O6PS	604.3544	-0.9	60.5	39	48.74	5.0	even	ok
	40	C20H56N9NaO4PS2	604.3527	-3.8	63.2	40	18.08	-2.0	even	ok
	41	C23H51N13PS2	604.3564	2.4	63.9	41	32.31	6.0	even	ok
	42	C20H60N7O5P2S2	604.3567	2.9	64.1	42	25.80	-4.0	even	ok
	43	C22H55N9O4PS2	604.3551	0.2	64.4	43	64.79	1.0	even	ok
	44	C30H51N7O2PS	604.3557	1.3	64.6	44	37.80	10.0	even	ok
	45	C22H65NNaO7P2S2	604.3570	3.4	65.9	45	19.65	-8.0	even	ok
	46	C25H56N7NaO2PS2	604.3567	2.9	66.0	46	24.78	2.0	even	ok
	47	C24H60N3NaO6PS2	604.3553	0.7	66.4	47	53.35	-3.0	even	ok
	48	C21H59N5O8PS2	604.3537	-2.0	66.9	48	34.02	-4.0	even	ok
	49	C32H56NNaO4PS	604.3560	1.7	66.9	49	29.79	6.0	even	ok
	50	C19H57N11NaOP2S2	604.3556	1.2	67.5	50	35.30	-2.0	even	ok
	51	C24H50N11O3S2	604.3534	-2.5	67.6	51	27.05	6.0	even	ok
	52	C23H54N7O7S2	604.3521	-4.7	67.8	52	9.18	1.0	even	ok
	53	C21H52N13NaPS2	604.3540	-1.6	67.9	53	30.66	3.0	even	ok
	54	C18H51N15O2PS2	604.3524	-4.2	68.6	54	9.43	2.0	even	ok
	55	C31H50N5O5S	604.3527	-3.7	69.3	55	12.27	10.0	even	ok
	56	C26H59N3O6PS2	604.3577	4.6	69.5	56	9.10	0.0	even	ok
	57	C26H55N5NaO5S2	604.3537	-2.1	69.7	57	29.98	2.0	even	ok
	58	C25H59NNaO9S2	604.3523	-4.3	69.8	58	11.00	-3.0	even	ok
	59	C27H51N9NaOS2	604.3550	0.1	71.4	59	51.02	7.0	even	ok
	60	C33H52N5NaPS	604.3573	4.0	73.0	60	9.23	11.0	even	ok
	61	C27H58NO9S2	604.3548	-0.3	73.2	61	45.89	0.0	even	ok
	62	C28H54N5O5S2	604.3561	1.9	74.7	62	26.30	5.0	even	ok
	63	C32H46N9OS	604.3541	-1.5	75.4	63	23.97	15.0	even	ok
	64	C31H60NO2P2S2	604.3535	-2.3	76.7	64	20.71	4.0	even	ok
	65	C34H51N3NaO3S	604.3543	-1.0	77.7	65	25.39	11.0	even	ok
	66	C29H50N9OS2	604.3574	4.1	77.8	66	8.79	10.0	even	ok
	67	C31H55N3NaO3S2	604.3577	4.6	79.9	67	6.28	6.0	even	ok
	68	C33H55N3OPS2	604.3519	-5.1	85.2	68	3.83	9.0	even	ok
	69	C36H50N3O3S	604.3567	3.0	87.6	69	8.05	14.0	even	ok
606.3695	1	C19H54N9NaO9P	606.3674	-3.4	31.5	1	44.26	-2.0	even	ok
	2	C21H63NNaO12P2	606.3718	3.7	31.9	2	37.73	-8.0	even	ok
	3	C17H49N15O7P	606.3672	-3.9	33.9	3	24.95	2.0	even	ok
	4	C20H57N5O13P	606.3685	-1.7	34.1	4	85.12	-4.0	even	ok
	5	C17H46N21P2	606.3715	3.3	39.0	5	29.62	7.0	even	ok
	6	C19H51N15NaO2P2	606.3718	3.7	40.8	6	22.62	3.0	even	ok
	7	C21H53N9O9P	606.3698	0.5	41.1	7	100.00	1.0	even	ok
	8	C23H58N3NaO11P	606.3701	1.0	42.9	8	83.04	-3.0	even	ok
	9	C18H45N19O3P	606.3685	-1.7	43.4	9	49.40	7.0	even	ok
	10	C22H52N7O12	606.3668	-4.4	45.3	10	18.75	1.0	even	ok
	11	C20H50N13NaO5P	606.3688	-1.2	45.3	11	54.50	3.0	even	ok
	12	C15H45N21NaO2S	606.3678	-2.8	46.4	12	38.99	4.0	even	ok

Compound Spectrum SmartFormula Report

Meas. m/z	#	Ion Formula	m/z	err [ppm]	mSigma	# mSigma	Score	rdb	e⁻ Conf	N-Rule
	13	C24H57NNaO14	606.3671	-3.9	47.2	13	22.59	-3.0	even	ok
	14	C16H48N17O6S	606.3689	-1.1	49.4	14	67.77	2.0	even	ok
	15	C22H49N13O5P	606.3712	2.8	50.0	15	35.90	6.0	even	ok
	16	C24H54N7NaO7P	606.3715	3.2	51.6	16	27.87	2.0	even	ok
	17	C18H53N11NaO8S	606.3691	-0.6	51.7	17	72.42	-2.0	even	ok
	18	C22H45N15NaO4	606.3671	-3.9	52.7	18	18.92	8.0	even	ok
	19	C25H57N3O11P	606.3725	5.0	53.1	19	10.60	0.0	even	ok
	20	C23H48N11O8	606.3682	-2.2	54.0	20	39.89	6.0	even	ok
	21	C17H44N21O2S	606.3702	1.2	54.7	21	55.66	7.0	even	ok
	22	C19H56N7O12S	606.3702	1.2	54.9	22	55.04	-4.0	even	ok
	23	C21H59N7NaO5P2S	606.3666	-4.8	55.2	23	10.85	-3.0	even	ok
	24	C25H53N5NaO10	606.3685	-1.7	55.7	24	44.72	2.0	even	ok
	25	C21H46N17NaOP	606.3701	1.0	56.4	25	41.55	8.0	even	ok
	26	C19H49N15NaO4S	606.3705	1.6	56.8	26	44.69	3.0	even	ok
	27	C21H61NNaO14S	606.3705	1.6	57.1	27	43.95	-8.0	even	ok
	28	C26H56NO14	606.3695	0.0	57.3	28	69.75	0.0	even	ok
	29	C22H62N3O9P2S	606.3677	-3.1	58.0	29	24.33	-5.0	even	ok
	30	C20H40N21O2	606.3668	-4.4	59.0	30	9.07	12.0	even	ok
	31	C20H52N11O8S	606.3716	3.4	59.5	31	20.03	1.0	even	ok
	32	C22H57N5NaO10S	606.3718	3.8	61.7	32	14.88	-3.0	even	ok
	33	C22H55N11NaOP2S	606.3679	-2.6	61.9	33	25.85	2.0	even	ok
	34	C24H44N15O4	606.3695	0.0	63.8	34	56.23	11.0	even	ok
	35	C20H45N19NaS	606.3718	3.8	63.9	35	13.88	8.0	even	ok
	36	C23H58N7O5P2S	606.3690	-0.9	64.2	36	44.40	0.0	even	ok
	37	C26H49N9NaO6	606.3698	0.5	65.3	37	47.32	7.0	even	ok
	38	C25H63NNaO7P2S	606.3693	-0.4	66.2	38	46.94	-4.0	even	ok
	39	C27H52N5O10	606.3709	2.2	66.5	39	25.47	5.0	even	ok
	40	C20H58N9NaO4PS2	606.3683	-2.0	67.9	40	33.36	-3.0	even	ok
	41	C23H45N17OP	606.3725	5.0	69.2	41	4.63	11.0	even	ok
	42	C21H61N5O8PS2	606.3694	-0.2	70.8	42	51.89	-5.0	even	ok
	43	C27H50N11OP2	606.3670	-4.2	71.5	43	8.65	10.0	even	ok
	44	C24H54N11OP2S	606.3703	1.4	71.9	44	28.72	5.0	even	ok
	45	C23H41N19Na	606.3685	-1.7	72.6	45	18.37	13.0	even	ok
	46	C29H55N5NaO3P2	606.3672	-3.7	72.9	46	10.38	6.0	even	ok
	47	C18H53N15O2PS2	606.3680	-2.4	73.6	47	18.06	1.0	even	ok
	48	C30H58NO7P2	606.3683	-2.0	73.8	48	21.43	4.0	even	ok
	49	C26H59N5NaO3P2S	606.3706	1.8	73.8	49	22.80	1.0	even	ok
	50	C22H57N9O4PS2	606.3707	2.0	75.0	50	25.62	0.0	even	ok
	51	C27H45N13NaO2	606.3711	2.7	75.6	51	15.12	12.0	even	ok
	52	C27H62NO7P2S	606.3717	3.6	75.9	52	10.05	-1.0	even	ok
	53	C25H53N9O4PS	606.3673	-3.6	76.2	53	9.90	5.0	even	ok
	54	C28H48N9O6	606.3722	4.5	76.5	54	6.25	10.0	even	ok
	55	C22H53N11NaO3S2	606.3666	-4.7	76.5	55	6.73	2.0	even	ok
	56	C24H62N3NaO6PS2	606.3710	2.4	77.0	56	19.84	-4.0	even	ok
	57	C30H53N3NaO8	606.3725	4.9	78.0	57	4.52	6.0	even	ok
	58	C27H58N3NaO6PS	606.3676	-3.1	78.2	58	11.36	1.0	even	ok

Compound Spectrum SmartFormula Report

Meas. m/z	#	Ion Formula	m/z	err [ppm]	mSigma	# mSigma	Score	rdb	e¯ Conf	N-Rule
	59	C23H56N7O7S2	606.3677	-3.0	79.1	59	14.73	0.0	even	ok
	60	C23H53N13PS2	606.3720	4.2	80.6	60	7.65	5.0	even	ok
	61	C21H54N13NaPS2	606.3696	0.2	81.0	61	27.91	2.0	even	ok
	62	C25H61NNaO9S2	606.3680	-2.5	81.1	62	16.49	-4.0	even	ok
	63	C25H58N7NaO2PS2	606.3723	4.6	82.6	63	5.49	1.0	even	ok
	64	C31H54N5O3P2	606.3696	0.2	84.2	64	24.40	9.0	even	ok
	65	C24H52N11O3S2	606.3691	-0.7	84.7	65	25.94	5.0	even	ok
	66	C26H49N13PS	606.3687	-1.4	85.0	66	16.91	10.0	even	ok
	67	C25H40N19	606.3709	2.2	85.7	67	9.08	16.0	even	ok
	68	C26H57N5NaO5S2	606.3693	-0.3	86.6	68	27.06	1.0	even	ok
	69	C28H54N7NaO2PS	606.3690	-0.9	86.8	69	18.07	6.0	even	ok
	70	C32H53N3O6P	606.3666	-4.7	88.0	70	3.36	9.0	even	ok
	71	C29H57N3O6PS	606.3700	0.8	88.6	71	17.05	4.0	even	ok
	72	C27H60NO9S2	606.3704	1.5	89.0	72	17.18	-1.0	even	ok
	73	C27H58N7P2S2	606.3665	-5.0	89.5	73	3.37	4.0	even	ok
	74	C29H63NNaO2P2S2	606.3668	-4.5	91.4	74	4.04	0.0	even	ok
	75	C30H56NO9S	606.3670	-4.1	93.0	75	3.80	4.0	even	ok
	76	C27H53N9NaOS2	606.3707	1.9	93.2	76	12.22	6.0	even	ok
	77	C28H56N5O5S2	606.3717	3.7	95.3	77	5.23	4.0	even	ok
	78	C34H55N3NaOP2	606.3713	2.9	96.4	78	5.74	10.0	even	ok
	79	C30H53N7O2PS	606.3714	3.1	97.7	79	5.03	9.0	even	ok
	80	C33H49N7O2P	606.3680	-2.5	98.7	80	6.05	14.0	even	ok
	81	C32H58NNaO4PS	606.3716	3.5	99.5	81	3.73	5.0	even	ok
608.3867	1	C25H59N3O11P	608.3882	2.3	2.8	1	54.23	-1.0	even	ok
	2	C26H58NO14	608.3852	-2.6	2.9	2	49.25	-1.0	even	ok
	3	C25H55N5NaO10	608.3841	-4.3	3.7	3	21.05	1.0	even	ok
	4	C23H50N11O8	608.3838	-4.8	5.1	4	15.88	5.0	even	ok
	5	C24H56N7NaO7P	608.3871	0.6	6.3	5	92.11	1.0	even	ok
	6	C22H51N13O5P	608.3868	0.1	8.5	6	100.00	5.0	even	ok
	7	C20H47N19NaS	608.3875	1.2	11.0	7	70.53	7.0	even	ok
	8	C26H55N7O7P	608.3895	4.5	11.5	8	16.33	4.0	even	ok
	9	C28H60NNaO9P	608.3898	5.0	11.6	9	12.48	0.0	even	ok
	10	C23H60N3NaO11P	608.3858	-1.6	12.7	10	59.41	-4.0	even	ok
	11	C21H48N17NaOP	608.3858	-1.6	13.0	11	44.03	7.0	even	ok
	12	C21H52N15O2P2	608.3898	5.0	13.3	12	8.85	5.0	even	ok
	13	C27H54N5O10	608.3865	-0.4	13.4	13	85.45	4.0	even	ok
	14	C17H46N21O2S	608.3859	-1.5	13.4	14	61.61	6.0	even	ok
	15	C18H52N17NaOPS	608.3891	3.9	13.8	15	21.83	2.0	even	ok
	16	C25H52N11NaO3P	608.3884	2.8	13.8	16	37.01	6.0	even	ok
	17	C21H55N9O9P	608.3855	-2.1	14.2	17	48.90	0.0	even	ok
	18	C26H51N9NaO6	608.3855	-2.1	14.6	18	47.44	6.0	even	ok
	19	C24H46N15O4	608.3852	-2.6	14.7	19	39.43	10.0	even	ok
	20	C19H51N15NaO4S	608.3861	-1.0	14.7	20	69.60	2.0	even	ok
	21	C21H50N15O4S	608.3885	2.9	14.7	21	33.86	5.0	even	ok
	22	C23H60N7O5P2S	608.3846	-3.5	14.8	22	26.65	-1.0	even	ok
	23	C23H64NO12P2	608.3898	5.1	15.7	23	11.13	-6.0	even	ok

Compound Spectrum SmartFormula Report

Meas. m/z	#	Ion Formula	m/z	err [ppm]	mSigma	# mSigma	Score	rdb	e¯ Conf	N-Rule
	24	C23H47N17OP	608.3882	2.3	16.1	24	32.07	10.0	even	ok
	25	C22H61N5NaO8P2	608.3888	3.3	16.5	25	27.84	-4.0	even	ok
	26	C25H65NNaO7P2S	608.3849	-3.0	17.0	26	31.50	-5.0	even	ok
	27	C23H55N9NaO6S	608.3888	3.4	17.0	27	26.21	1.0	even	ok
	28	C24H56N11OP2S	608.3860	-1.3	17.5	28	60.60	4.0	even	ok
	29	C20H52N13NaO5P	608.3844	-3.8	17.6	29	15.82	2.0	even	ok
	30	C20H56N11O6P2	608.3885	2.8	18.2	30	33.03	0.0	even	ok
	31	C20H54N11O8S	608.3872	0.8	18.5	31	69.51	0.0	even	ok
	32	C23H43N19Na	608.3841	-4.3	19.2	32	11.65	12.0	even	ok
	33	C26H61N5NaO3P2S	608.3863	-0.8	19.6	33	66.70	0.0	even	ok
	34	C18H47N19O3P	608.3841	-4.3	19.8	34	11.88	6.0	even	ok
	35	C22H59N5NaO10S	608.3875	1.2	20.1	35	58.40	-4.0	even	ok
	36	C30H60NO7P2	608.3840	-4.6	22.0	36	12.75	3.0	even	ok
	37	C19H53N15NaO2P2	608.3874	1.1	22.5	37	43.26	2.0	even	ok
	38	C27H64NO7P2S	608.3873	0.9	22.6	38	60.06	-2.0	even	ok
	39	C23H62NO14S	608.3886	3.0	24.1	39	27.56	-6.0	even	ok
	40	C20H59N5O13P	608.3842	-4.3	24.6	40	14.39	-5.0	even	ok
	41	C17H48N21P2	608.3871	0.6	24.7	41	47.11	6.0	even	ok
	42	C28H50N9O6	608.3879	1.8	25.0	42	42.57	9.0	even	ok
	43	C30H55N3NaO8	608.3881	2.3	25.7	43	35.27	5.0	even	ok
	44	C27H47N13NaO2	608.3868	0.1	26.2	44	70.38	11.0	even	ok
	45	C21H63NNaO14S	608.3861	-1.0	28.4	45	51.92	-9.0	even	ok
	46	C28H60N5O3P2S	608.3887	3.1	29.4	46	22.48	3.0	even	ok
	47	C25H42N19	608.3865	-0.4	29.7	47	44.66	15.0	even	ok
	48	C26H51N13PS	608.3843	-4.0	30.1	48	14.70	9.0	even	ok
	49	C14H42N25O3	608.3897	4.8	31.6	49	6.63	7.0	even	ok
	50	C28H56N7NaO2PS	608.3846	-3.5	31.9	50	17.69	5.0	even	ok
	51	C20H60N9NaO4PS2	608.3840	-4.6	32.1	51	12.56	-4.0	even	ok
	52	C23H65N5NaO3P2S2	608.3896	4.7	32.2	52	11.61	-5.0	even	ok
	53	C21H56N13NaPS2	608.3853	-2.4	33.6	53	27.85	1.0	even	ok
	54	C21H60N11OP2S2	608.3893	4.3	33.6	54	11.58	-1.0	even	ok
	55	C22H59N9O4PS2	608.3864	-0.6	33.7	55	63.00	-1.0	even	ok
	56	C31H56N5O3P2	608.3853	-2.4	33.7	56	27.77	8.0	even	ok
	57	C29H59N3O6PS	608.3857	-1.8	33.8	57	35.09	3.0	even	ok
	58	C23H55N13PS2	608.3877	1.6	35.0	58	45.89	4.0	even	ok
	59	C24H64N3NaO6PS2	608.3866	-0.2	35.7	59	67.59	-5.0	even	ok
	60	C21H63N5O8PS2	608.3850	-2.8	35.8	60	27.38	-6.0	even	ok
	61	C29H46N13O2	608.3892	4.0	36.8	61	12.19	14.0	even	ok
	62	C25H60N7NaO2PS2	608.3880	2.0	37.1	62	36.86	0.0	even	ok
	63	C31H51N7NaO4	608.3895	4.5	37.4	63	9.37	10.0	even	ok
	64	C24H54N11O3S2	608.3847	-3.4	39.1	64	19.85	4.0	even	ok
	65	C26H63N3O6PS2	608.3890	3.8	40.3	65	15.75	-2.0	even	ok
	66	C26H59N5NaO5S2	608.3850	-2.9	41.2	66	23.07	0.0	even	ok
	67	C30H55N7O2PS	608.3870	0.4	42.9	67	41.82	8.0	even	ok
	68	C27H62NO9S2	608.3861	-1.1	44.3	68	40.69	-2.0	even	ok
	69	C32H60NNaO4PS	608.3873	0.9	44.6	69	34.92	4.0	even	ok

Compound Spectrum SmartFormula Report

Meas. m/z	#	Ion Formula	m/z	err [ppm]	mSigma	# mSigma	Score	rdb	e⁻ Conf	N-Rule
	70	C27H55N9NaOS2	608.3863	-0.7	45.6	70	44.79	5.0	even	ok
	71	C34H57N3NaOP2	608.3869	0.3	46.1	71	39.83	9.0	even	ok
	72	C31H54N5O5S	608.3840	-4.5	47.1	72	7.10	8.0	even	ok
	73	C28H58N5O5S2	608.3874	1.1	48.2	73	37.34	3.0	even	ok
	74	C35H56NNaO4P	608.3839	-4.7	48.7	74	6.16	9.0	even	ok
	75	C31H64NO2P2S2	608.3848	-3.2	52.4	75	14.73	2.0	even	ok
	76	C29H54N9OS2	608.3887	3.3	54.1	76	13.42	8.0	even	ok
	77	C33H56N5NaPS	608.3886	3.1	54.7	77	11.38	9.0	even	ok
	78	C34H59NO4PS	608.3897	4.8	55.9	78	4.43	7.0	even	ok
	79	C31H59N3NaO3S2	608.3890	3.7	56.1	79	10.11	4.0	even	ok
	80	C32H50N9OS	608.3854	-2.3	57.1	80	14.70	13.0	even	ok
	81	C36H56N3OP2	608.3893	4.2	57.2	81	6.01	12.0	even	ok
	82	C34H55N3NaO3S	608.3856	-1.8	58.7	82	16.54	9.0	even	ok
	83	C37H55NO4P	608.3863	-0.7	60.1	83	22.74	12.0	even	ok
	84	C36H52N5NaP	608.3853	-2.5	60.4	84	12.30	14.0	even	ok
	85	C36H54N3O3S	608.3880	2.1	70.3	85	9.88	12.0	even	ok
	86	C38H51N5P	608.3877	1.5	71.8	86	11.69	17.0	even	ok
	87	C39H50N3O3	608.3847	-3.4	74.8	87	4.79	17.0	even	ok
	88	C39H55NNaOS	608.3897	4.8	82.5	88	1.72	13.0	even	ok
	89	C42H51NNaO	608.3863	-0.8	87.5	89	7.81	18.0	even	ok
	90	C44H50NO	608.3887	3.2	99.2	90	1.87	21.0	even	ok
628.3838	1	C54H113N8O22P	628.3848	1.5	21.5	1	67.03	4.0	even	ok
	2	C56H119N2NaO22P2	628.3808	-4.8	21.5	2	14.09	0.0	even	ok
	3	C54H114N8O20P2	628.3807	-5.0	21.5	3	12.28	4.0	even	ok
	4	C55H119N4NaO21P2	628.3864	4.1	21.5	4	20.56	0.0	even	ok
	5	C43H89N42PS	628.3851	2.1	21.5	5	54.90	22.0	even	ok
	6	C45H95N36NaP2S	628.3812	-4.3	21.6	6	19.21	18.0	even	ok
	7	C52H104N16O19	628.3826	-1.9	21.6	7	58.38	10.0	even	ok
	8	C53H110N12NaO18P	628.3843	0.7	21.6	8	88.03	6.0	even	ok
	9	C56H122O25P2	628.3870	5.0	21.7	9	12.38	-2.0	even	ok
	10	C56H118N2NaO24P	628.3849	1.8	21.7	10	61.45	0.0	even	ok
	11	C54H109N10NaO21	628.3828	-1.7	21.7	11	63.24	6.0	even	ok
	12	C53H114N10O19P2	628.3863	3.9	21.8	12	23.07	4.0	even	ok
	13	C45H94N36NaO2PS	628.3853	2.3	21.9	13	49.83	18.0	even	ok
	14	C51H105N18O16P	628.3841	0.5	22.1	14	92.98	10.0	even	ok
	15	C46H98N32O4P2S	628.3817	-3.4	22.1	15	29.82	16.0	even	ok
	16	C51H101N20NaO15	628.3821	-2.8	22.1	16	40.59	12.0	even	ok
	17	C55H112N6O25	628.3833	-0.8	22.3	17	83.04	4.0	even	ok
	18	C42H89N40NaO3S	628.3824	-2.2	22.4	18	50.73	19.0	even	ok
	19	C44H88N40O3S	628.3836	-0.3	22.6	19	96.18	22.0	even	ok
	20	C53H113N6NaO25	628.3821	-2.8	22.7	20	40.27	1.0	even	ok
	21	C49H96N26O13	628.3820	-3.0	22.7	21	36.24	16.0	even	ok
	22	C52H111N14NaO15P2	628.3858	3.1	22.7	22	34.74	6.0	even	ok
	23	C40H84N46OS	628.3823	-2.4	22.7	23	45.95	23.0	even	ok
	24	C46H97N32O6PS	628.3858	3.1	22.8	24	33.48	16.0	even	ok
	25	C48H103N26NaO6P2S	628.3818	-3.2	22.9	25	32.69	12.0	even	ok

Compound Spectrum SmartFormula Report

Meas. m/z	#	Ion Formula	m/z	err [ppm]	mSigma	# mSigma	Score	rdb	e¯ Conf	N-Rule
	26	C43H92N36O7S	628.3830	-1.4	22.9	26	68.93	17.0	even	ok
	27	C51H108N12O23	628.3820	-3.0	23.0	27	36.10	5.0	even	ok
	28	C54H110N14O15P2	628.3870	5.0	23.1	28	12.06	9.0	even	ok
	29	C54H116N2O29	628.3826	-1.9	23.1	29	56.62	-1.0	even	ok
	30	C57H117NaO27	628.3834	-0.6	23.1	30	87.12	0.0	even	ok
	31	C44H98N32NaO6PS	628.3846	1.2	23.2	31	71.81	13.0	even	ok
	32	C45H97N30NaO9S	628.3831	-1.1	23.2	32	73.68	13.0	even	ok
	33	C50H102N22NaO12P	628.3836	-0.4	23.3	33	92.77	12.0	even	ok
	34	C42H93N38O4PS	628.3845	1.0	23.4	34	76.88	17.0	even	ok
	35	C46H93N34NaO5S	628.3838	-0.1	23.4	35	100.00	18.0	even	ok
	36	C41H90N42NaPS	628.3839	0.2	23.6	36	97.83	19.0	even	ok
	37	C50H105N16NaO19	628.3814	-3.8	23.6	37	23.37	7.0	even	ok
	38	C50H106N20O13P2	628.3856	2.8	23.6	38	37.69	10.0	even	ok
	39	C45H102N28O8P2S	628.3810	-4.5	23.8	39	16.16	11.0	even	ok
	40	C53H117N4O26P	628.3841	0.5	23.8	40	89.28	-1.0	even	ok
	41	C48H102N26NaO8PS	628.3859	3.4	23.8	41	29.40	12.0	even	ok
	42	C55H110N12O16P2	628.3814	-4.0	23.8	42	21.71	9.0	even	ok
	43	C53H107N18NaO11P2	628.3864	4.1	23.9	43	19.60	11.0	even	ok
	44	C47H107N22NaO10P2S	628.3812	-4.3	24.0	44	18.29	7.0	even	ok
	45	C52H114N8NaO22P	628.3836	-0.4	24.0	45	91.53	1.0	even	ok
	46	C45H101N28O10PS	628.3851	2.1	24.0	46	51.77	11.0	even	ok
	47	C52H101N22O12P	628.3848	1.5	24.2	47	63.25	15.0	even	ok
	48	C48H93N30NaO9	628.3814	-3.8	24.2	48	22.94	18.0	even	ok
	49	C55H109N12O18P	628.3855	2.6	24.2	49	41.42	9.0	even	ok
	50	C43H99N34NaO3P2S	628.3861	3.6	24.3	50	25.76	13.0	even	ok
	51	C54H107N16NaO12P2	628.3808	-4.8	24.3	51	13.17	11.0	even	ok
	52	C51H102N24O9P2	628.3863	3.9	24.3	52	21.92	15.0	even	ok
	53	C54H106N16NaO14P	628.3849	1.8	24.3	53	58.22	11.0	even	ok
	54	C57H115N6NaO18P2	628.3815	-3.7	24.3	54	24.17	5.0	even	ok
	55	C48H97N28O10P	628.3835	-0.6	24.3	55	85.09	16.0	even	ok
	56	C46H107N24NaO9P2S	628.3868	4.7	24.4	56	14.14	7.0	even	ok
	57	C44H102N30O7P2S	628.3866	4.5	24.4	57	16.11	11.0	even	ok
	58	C49H106N22O10P2S	628.3824	-2.3	24.4	58	46.16	10.0	even	ok
	59	C52H102N22O10P2	628.3807	-5.0	24.4	59	11.43	15.0	even	ok
	60	C47H106N22NaO12PS	628.3853	2.3	24.5	60	46.85	7.0	even	ok
	61	C48H100N22O17	628.3813	-4.0	24.6	61	20.27	11.0	even	ok
	62	C46H100N26O13S	628.3836	-0.3	24.7	62	91.83	11.0	even	ok
	63	C54H123NaO25P2	628.3858	3.1	24.7	63	33.06	-5.0	even	ok
	64	C50H109N14O20P	628.3835	-0.6	24.9	64	84.31	5.0	even	ok
	65	C58H118N2O22P2	628.3820	-2.9	24.9	65	36.15	3.0	even	ok
	66	C41H94N40OP2S	628.3860	3.4	24.9	66	28.38	17.0	even	ok
	67	C57H114N6NaO20P	628.3856	2.8	25.0	67	36.91	5.0	even	ok
	68	C47H96N30O9S	628.3843	0.8	25.0	68	79.93	16.0	even	ok
	69	C53H100N20O15	628.3833	-0.8	25.2	69	77.63	15.0	even	ok
	70	C51H98N26NaO8P	628.3843	0.7	25.3	70	81.34	17.0	even	ok
	71	C46H88N36O7	628.3813	-4.1	25.4	71	19.83	22.0	even	ok

Compound Spectrum SmartFormula Report

Meas. m/z	#	Ion Formula	m/z	err [ppm]	mSigma	# mSigma	Score	rdb	e⁻ Conf	N-Rule
	72	C48H110N18O14P2S	628.3817	-3.4	25.4	72	27.83	5.0	even	ok
	73	C49H103N24NaO9P2	628.3851	2.0	25.4	73	51.74	12.0	even	ok
	74	C52H118N6O23P2	628.3856	2.9	25.4	74	36.05	-1.0	even	ok
	75	C47H94N36P2S	628.3824	-2.4	25.5	75	44.94	21.0	even	ok
	76	C52H97N24NaO11	628.3828	-1.7	25.6	76	57.84	17.0	even	ok
	77	C50H92N30O9	628.3826	-1.9	25.6	77	53.19	21.0	even	ok
	78	C48H105N20NaO15S	628.3838	-0.1	25.7	78	95.29	7.0	even	ok
	79	C49H105N22O12PS	628.3865	4.2	25.7	79	17.99	10.0	even	ok
	80	C51H111N16NaO12P2S	628.3825	-2.1	25.7	80	49.04	6.0	even	ok
	81	C55H105N14NaO17	628.3834	-0.6	25.8	81	81.82	11.0	even	ok
	82	C49H93N32O6P	628.3841	0.5	25.8	82	85.67	21.0	even	ok
	83	C58H117N2O24P	628.3861	3.7	25.9	83	23.93	3.0	even	ok
	84	C49H106N18NaO16P	628.3829	-1.4	26.0	84	62.67	7.0	even	ok
	85	C50H99N28NaO5P2	628.3858	3.1	26.0	85	32.36	17.0	even	ok
	86	C47H97N26NaO13	628.3808	-4.9	26.1	86	11.93	13.0	even	ok
	87	C47H100N24O16	628.3869	4.9	26.1	87	11.96	11.0	even	ok
	88	C50H115N12NaO16P2S	628.3818	-3.2	26.2	88	30.46	1.0	even	ok
	89	C48H109N18O16PS	628.3858	3.2	26.2	89	30.90	5.0	even	ok
	90	C51H115N10NaO19P2	628.3851	2.0	26.3	90	50.61	1.0	even	ok
	91	C56H108N10O21	628.3840	0.2	26.4	91	90.14	9.0	even	ok
	92	C49H101N24NaO11S	628.3845	1.0	26.4	92	72.26	12.0	even	ok
	93	C47H94N32NaO6P	628.3829	-1.5	26.4	93	61.90	18.0	even	ok
	94	C45H88N38O6	628.3869	4.9	26.4	94	11.92	22.0	even	ok
	95	C47H93N36O2PS	628.3865	4.2	26.5	95	17.75	21.0	even	ok
	96	C49H99N30NaO2P2S	628.3825	-2.1	26.6	96	47.92	17.0	even	ok
	97	C47H98N30O7P2	628.3850	1.8	26.9	97	54.48	16.0	even	ok
	98	C49H89N34NaO5	628.3821	-2.8	26.9	98	36.28	23.0	even	ok
	99	C50H105N20O13PS	628.3809	-4.7	27.1	99	12.96	10.0	even	ok
	100	C51H110N16NaO14PS	628.3866	4.4	27.1	100	15.31	6.0	even	ok
	101	C50H114N12NaO18PS	628.3860	3.4	27.2	101	27.10	1.0	even	ok
	102	C47H101N24O14P	628.3828	-1.7	27.3	102	56.11	11.0	even	ok
	103	C41H93N36NaO7S	628.3818	-3.3	27.4	103	28.16	14.0	even	ok
	104	C49H110N16O17P2	628.3850	1.8	27.5	104	53.53	5.0	even	ok
	105	C47H84N40O3	628.3820	-3.0	27.5	105	32.32	27.0	even	ok
	106	C58H113N4NaO23	628.3841	0.4	27.5	106	82.55	5.0	even	ok
	107	C44H101N26NaO13S	628.3824	-2.2	27.6	107	45.16	8.0	even	ok
	108	C46H97N28NaO12	628.3864	4.0	27.7	108	18.90	13.0	even	ok
	109	C42H96N32O11S	628.3823	-2.4	27.8	109	41.08	12.0	even	ok
	110	C49H98N30NaO4PS	628.3866	4.4	27.8	110	15.15	17.0	even	ok
	111	C49H108N16O19S	628.3843	0.8	27.9	111	74.57	5.0	even	ok
	112	C52H117N2NaO29	628.3814	-3.8	28.0	112	21.22	-4.0	even	ok
	113	C52H114N12O16P2S	628.3830	-1.3	28.0	113	63.47	4.0	even	ok
	114	C50H102N26O6P2S	628.3830	-1.3	28.0	114	63.25	15.0	even	ok
	115	C39H88N42O5S	628.3816	-3.5	28.1	115	24.83	18.0	even	ok
	116	C48H94N34O3P2	628.3856	2.8	28.2	116	27.27	21.0	even	ok
	117	C47H89N38NaOS	628.3844	1.0	28.2	117	69.46	23.0	even	ok

Compound Spectrum SmartFormula Report

Meas. m/z	#	Ion Formula	m/z	err [ppm]	mSigma	# mSigma	Score	rdb	e⁻ Conf	N-Rule
	118	C48H93N34O3PS	628.3809	-4.7	28.3	118	12.55	21.0	even	ok
	119	C51H118N8O20P2S	628.3824	-2.3	28.3	119	42.32	-1.0	even	ok
	120	C38H85N46NaOS	628.3811	-4.4	28.5	120	15.52	20.0	even	ok
	121	C50H104N20O15S	628.3850	1.8	28.7	121	51.06	10.0	even	ok
	122	C50H112N8O27	628.3813	-4.0	28.7	122	18.51	0.0	even	ok
	123	C52H110N14NaO15PS	628.3810	-4.5	28.7	123	14.24	6.0	even	ok
	124	C43H102N28NaO10PS	628.3839	0.2	28.8	124	86.54	8.0	even	ok
	125	C45H104N22O17S	628.3830	-1.4	28.8	125	60.28	6.0	even	ok
	126	C45H85N40NaO3	628.3808	-4.9	28.8	126	8.89	24.0	even	ok
	127	C59H116O27	628.3847	1.3	29.0	127	61.42	3.0	even	ok
	128	C48H90N36NaO2P	628.3836	-0.4	29.0	128	64.88	23.0	even	ok
	129	C48H107N20NaO13P2	628.3844	0.9	29.1	129	68.97	7.0	even	ok
	130	C45H89N38O4P	628.3828	-1.7	29.2	130	42.86	22.0	even	ok
	131	C49H109N12NaO23	628.3808	-4.9	29.2	131	11.14	2.0	even	ok
	132	C46H98N28NaO10P	628.3823	-2.5	29.2	132	38.35	13.0	even	ok
	133	C47H109N16NaO19S	628.3831	-1.1	29.2	133	64.31	2.0	even	ok
	134	C51H113N10NaO21S	628.3845	1.0	29.2	134	67.38	1.0	even	ok
	135	C52H98N28O5P2	628.3870	5.0	29.3	135	10.52	20.0	even	ok
	136	C44H92N34O10	628.3862	3.8	29.3	136	20.52	17.0	even	ok
	137	C41H97N34O8PS	628.3838	-0.1	29.3	137	87.87	12.0	even	ok
	138	C46H110N18NaO16PS	628.3846	1.2	29.4	138	62.05	2.0	even	ok
	139	C44H105N24O14PS	628.3845	1.0	29.4	139	66.68	6.0	even	ok
	140	C42H99N36NaP2S2	628.3828	-1.6	29.5	140	54.95	13.0	even	ok
	141	C40H94N38NaO4PS	628.3833	-0.9	29.5	141	68.84	14.0	even	ok
	142	C40H93N42PS2	628.3868	4.8	29.5	142	12.00	17.0	even	ok
	143	C53H123N2NaO22P2S	628.3825	-2.1	29.5	143	44.96	-5.0	even	ok
	144	C51H117N8O22PS	628.3865	4.2	29.6	144	16.34	-1.0	even	ok
	145	C48H92N34O5S	628.3850	1.8	29.6	145	50.07	21.0	even	ok
	146	C52H107N20NaO8P2S	628.3832	-1.1	29.6	146	65.52	11.0	even	ok
	147	C54H119N6NaO18P2S	628.3832	-1.0	29.7	147	65.58	0.0	even	ok
	148	C50H98N28NaO5PS	628.3810	-4.5	29.7	148	13.83	17.0	even	ok
	149	C52H121O30P	628.3835	-0.6	30.0	149	74.90	-6.0	even	ok
	150	C46H85N42P	628.3835	-0.6	30.1	150	59.38	27.0	even	ok
	151	C51H118N4NaO26P	628.3829	-1.4	30.2	151	56.90	-4.0	even	ok
	152	C53H98N26O6P2	628.3813	-4.0	30.2	152	18.62	20.0	even	ok
	153	C56H106N16O12P2	628.3820	-2.9	30.2	153	31.79	14.0	even	ok
	154	C44H85N42NaO2	628.3864	4.0	30.2	154	14.31	24.0	even	ok
	155	C37H85N48NaS	628.3867	4.6	30.3	155	13.07	20.0	even	ok
	156	C49H112N10O26	628.3869	4.9	30.3	156	10.76	0.0	even	ok
	157	C53H97N26O8P	628.3855	2.6	30.3	157	36.02	20.0	even	ok
	158	C45H111N20NaO13P2S	628.3861	3.6	30.4	158	22.20	2.0	even	ok
	159	C47H114N14O18P2S	628.3810	-4.5	30.4	159	13.91	0.0	even	ok
	160	C52H109N14NaO17S	628.3851	2.1	30.4	160	44.92	6.0	even	ok
	161	C52H117N6O23PS	628.3809	-4.7	30.4	161	12.03	-1.0	even	ok
	162	C55H103N20NaO8P2	628.3815	-3.7	30.5	162	20.83	16.0	even	ok
	163	C41H93N40OPS2	628.3812	-4.2	30.5	163	16.35	17.0	even	ok

Compound Spectrum SmartFormula Report

Meas. m/z	#	Ion Formula	m/z	err [ppm]	mSigma	# mSigma	Score	rdb	e¯ Conf	N-Rule
	164	C38H89N44O2PS	628.3831	-1.1	30.5	164	62.58	18.0	even	ok
	165	C51H95N32NaOP2	628.3864	4.1	30.6	165	16.81	22.0	even	ok
	166	C46H102N26O11P2	628.3843	0.7	30.7	166	70.99	11.0	even	ok
	167	C49H119N8NaO20P2S	628.3812	-4.2	30.7	167	15.70	-4.0	even	ok
	168	C46H95N34NaO3P2	628.3844	0.9	30.7	168	53.13	18.0	even	ok
	169	C42H103N30NaO7P2S	628.3854	2.5	30.7	169	36.52	8.0	even	ok
	170	C47H113N14O20PS	628.3851	2.1	30.8	170	44.04	0.0	even	ok
	171	C37H88N46OS2	628.3840	0.2	30.8	171	80.93	18.0	even	ok
	172	C43H106N26O11P2S	628.3860	3.4	30.8	172	24.59	6.0	even	ok
	173	C38H94N42NaPS2	628.3856	2.8	30.8	173	32.00	14.0	even	ok
	174	C55H102N20NaO10P	628.3856	2.8	30.8	174	32.26	16.0	even	ok
	175	C56H105N16O14P	628.3861	3.7	30.9	175	21.36	14.0	even	ok
	176	C44H93N34O8P	628.3821	-2.7	30.9	176	33.40	17.0	even	ok
	177	C42H98N36NaO2PS2	628.3870	5.0	30.9	177	10.11	13.0	even	ok
	178	C53H122N2NaO24PS	628.3866	4.4	30.9	178	13.91	-5.0	even	ok
	179	C58H111N10NaO14P2	628.3822	-2.7	31.0	179	34.43	10.0	even	ok
	180	C48H109N14NaO22	628.3864	4.1	31.0	180	17.38	2.0	even	ok
	181	C48H112N12O23S	628.3837	-0.3	31.1	181	79.08	0.0	even	ok
	182	C52H94N30NaO4P	628.3849	1.7	31.1	182	49.71	22.0	even	ok
	183	C50H89N36O2P	628.3848	1.5	31.2	183	53.82	26.0	even	ok
	184	C49H113N10O24P	628.3828	-1.7	31.2	184	51.26	0.0	even	ok
	185	C50H97N28NaO7S	628.3851	2.1	31.2	185	44.19	17.0	even	ok
	186	C49H118N8NaO22PS	628.3853	2.3	31.2	186	39.80	-4.0	even	ok
	187	C46H114N16O17P2S	628.3866	4.5	31.2	187	13.64	0.0	even	ok
	188	C52H95N30NaO2P2	628.3808	-4.8	31.3	188	11.11	22.0	even	ok
	189	C48H119N10NaO19P2S	628.3868	4.7	31.3	189	11.94	-4.0	even	ok
	190	C53H113N10O19PS	628.3815	-3.6	31.3	190	21.35	4.0	even	ok
	191	C59H114N6O18P2	628.3827	-1.8	31.5	191	47.99	8.0	even	ok
	192	C51H101N24O9PS	628.3815	-3.7	31.6	192	21.13	15.0	even	ok
	193	C40H98N36O5P2S	628.3853	2.3	31.6	193	39.24	12.0	even	ok
	194	C39H93N40NaO3S2	628.3841	0.5	31.7	194	74.48	14.0	even	ok
	195	C53H110N16O12P2S	628.3837	-0.2	31.7	195	79.74	9.0	even	ok
	196	C58H110N10NaO16P	628.3863	3.9	31.9	196	18.53	10.0	even	ok
	197	C50H117N6NaO25S	628.3838	-0.1	32.0	197	82.09	-4.0	even	ok
	198	C52H116N6O25S	628.3850	1.8	32.0	198	46.95	-1.0	even	ok
	199	C44H86N42NaP	628.3822	-2.5	32.0	199	28.56	24.0	even	ok
	200	C54H122NaO25PS	628.3810	-4.5	32.0	200	13.21	-5.0	even	ok
	201	C42H80N48	628.3862	3.8	32.0	201	15.42	28.0	even	ok
	202	C39H98N38O2P2S2	628.3820	-2.9	32.0	202	30.63	12.0	even	ok
	203	C41H92N40O3S2	628.3853	2.4	32.1	203	37.99	17.0	even	ok
	204	C43H98N34NaO3PS2	628.3813	-4.0	32.1	204	17.77	13.0	even	ok
	205	C51H88N34O5	628.3833	-0.9	32.1	205	65.63	26.0	even	ok
	206	C48H110N14NaO20P	628.3823	-2.5	32.2	206	35.82	2.0	even	ok
	207	C43H102N32O4P2S2	628.3834	-0.7	32.2	207	67.94	11.0	even	ok
	208	C39H95N40NaOP2S	628.3848	1.5	32.3	208	53.39	14.0	even	ok
	209	C54H96N24O11	628.3840	0.2	32.3	209	78.39	20.0	even	ok

Compound Spectrum SmartFormula Report

Meas. m/z	#	Ion Formula	m/z	err [ppm]	mSigma	# mSigma	Score	rdb	e⁻ Conf	N-Rule
	210	C46H104N20O20	628.3862	3.8	32.3	210	18.99	6.0	even	ok
	211	C51H122N2O27P2	628.3850	1.8	32.3	211	47.44	-6.0	even	ok
	212	C55H122N2O22P2S	628.3837	-0.2	32.4	212	78.49	-2.0	even	ok
	213	C50H122N4O24P2S	628.3817	-3.4	32.5	213	23.57	-6.0	even	ok
	214	C53H93N28NaO7	628.3834	-0.6	32.5	214	69.50	22.0	even	ok
	215	C44H90N40OP2	628.3843	0.7	32.6	215	54.21	22.0	even	ok
	216	C59H113N6O20P	628.3868	4.7	32.7	216	11.17	8.0	even	ok
	217	C44H98N36P2S2	628.3840	0.3	32.8	217	75.08	16.0	even	ok
	218	C45H99N30NaO7P2	628.3837	-0.1	32.8	218	79.00	13.0	even	ok
	219	C41H103N32NaO4P2S2	628.3822	-2.6	32.8	219	33.19	8.0	even	ok
	220	C61H119NaO20P2	628.3828	-1.6	32.8	220	50.44	4.0	even	ok
	221	C39H97N38O4PS2	628.3862	3.7	32.9	221	20.11	12.0	even	ok
	222	C50H119N6NaO23P2	628.3844	0.9	33.0	222	62.45	-4.0	even	ok
	223	C50H85N38NaO	628.3828	-1.7	33.1	223	48.09	28.0	even	ok
	224	C56H101N18NaO13	628.3841	0.4	33.1	224	72.21	16.0	even	ok
	225	C53H112N10O21S	628.3857	2.9	33.2	225	29.24	4.0	even	ok
	226	C55H118N4NaO21PS	628.3817	-3.4	33.2	226	22.79	0.0	even	ok
	227	C50H121N4O26PS	628.3858	3.2	33.3	227	25.94	-6.0	even	ok
	228	C51H100N24O11S	628.3857	2.9	33.3	228	29.28	15.0	even	ok
	229	C53H106N18NaO11PS	628.3817	-3.4	33.4	229	22.61	11.0	even	ok
	230	C43H89N38NaO6	628.3857	3.0	33.5	230	22.58	19.0	even	ok
	231	C50H90N36P2	628.3807	-5.0	33.5	231	7.31	26.0	even	ok
	232	C46H105N20O18P	628.3821	-2.7	33.6	232	31.37	6.0	even	ok
	233	C55H115N10NaO14P2S	628.3838	0.0	33.6	233	79.82	5.0	even	ok
	234	C45H101N24NaO16	628.3857	3.0	33.6	234	28.00	8.0	even	ok
	235	C54H121NaO27S	628.3851	2.1	33.6	235	41.32	-5.0	even	ok
	236	C57H104N14O17	628.3846	1.3	33.7	236	54.83	14.0	even	ok
	237	C43H97N34NaO5S2	628.3855	2.6	33.8	237	33.11	13.0	even	ok
	238	C45H107N26NaO6P2S2	628.3835	-0.5	33.8	238	69.63	7.0	even	ok
	239	C41H102N32NaO6PS2	628.3863	3.9	33.8	239	17.47	8.0	even	ok
	240	C59H110N8NaO17P	628.3807	-5.0	33.8	240	9.01	10.0	even	ok
	241	C61H118NaO22P	628.3870	5.0	34.1	241	9.40	4.0	even	ok
	242	C42H102N30NaO7PS2	628.3807	-5.0	34.1	242	9.05	8.0	even	ok
	243	C40H96N36O7S2	628.3847	1.3	34.2	243	53.80	12.0	even	ok
	244	C48H114N12O21P2	628.3843	0.7	34.3	244	64.69	0.0	even	ok
	245	C51H120N2O29S	628.3843	0.8	34.4	245	63.31	-6.0	even	ok
	246	C46H103N30NaO2P2S2	628.3842	0.6	34.7	246	67.14	12.0	even	ok
	247	C60H113N4O21P	628.3812	-4.2	34.8	247	14.57	8.0	even	ok
	248	C43H105N22NaO17S	628.3818	-3.3	34.9	248	23.53	3.0	even	ok
	249	C51H98N30O2P2S	628.3837	-0.2	34.9	249	73.45	20.0	even	ok
	250	C59H109N8NaO19	628.3848	1.5	35.0	250	49.11	10.0	even	ok
	251	C40H97N32NaO11S	628.3811	-4.4	35.0	251	13.28	9.0	even	ok
	252	C44H101N30O7PS2	628.3819	-3.1	35.0	252	25.36	11.0	even	ok
	253	C45H102N24NaO14P	628.3816	-3.6	35.1	253	20.11	8.0	even	ok
	254	C55H117N4NaO23S	628.3858	3.1	35.2	254	25.05	0.0	even	ok
	255	C35H89N46NaOS2	628.3828	-1.7	35.2	255	46.09	15.0	even	ok

Compound Spectrum SmartFormula Report

Meas. m/z	#	Ion Formula	m/z	err [ppm]	mSigma	# mSigma	Score	rdb	e⁻ Conf	N-Rule
	256	C53H105N18NaO13S	628.3858	3.1	35.2	256	25.13	11.0	even	ok
	257	C42H106N28O8P2S2	628.3827	-1.8	35.2	257	44.07	6.0	even	ok
	258	C43H96N30O14	628.3856	2.8	35.2	258	29.73	12.0	even	ok
	259	C41H100N28O15S	628.3816	-3.5	35.3	259	20.85	7.0	even	ok
	260	C43H90N38NaO4P	628.3816	-3.6	35.4	260	15.89	19.0	even	ok
	261	C42H101N30NaO9S2	628.3848	1.5	35.4	261	48.23	8.0	even	ok
	262	C41H84N44O4	628.3856	2.7	35.5	262	23.70	23.0	even	ok
	263	C47H111N16NaO17P2	628.3838	-0.1	35.6	263	73.81	2.0	even	ok
	264	C44H93N38NaOS2	628.3861	3.7	35.6	264	19.06	18.0	even	ok
	265	C54H109N14O15PS	628.3822	-2.6	35.8	265	31.61	9.0	even	ok
	266	C44H108N18O21S	628.3823	-2.4	35.8	266	33.78	1.0	even	ok
	267	C46H113N12NaO23S	628.3824	-2.2	35.8	267	37.01	-3.0	even	ok
	268	C38H92N38O9S	628.3810	-4.6	35.8	268	11.42	13.0	even	ok
	269	C56H118N6O18P2S	628.3844	0.9	36.2	269	58.93	3.0	even	ok
	270	C56H121O25PS	628.3822	-2.6	36.2	270	31.37	-2.0	even	ok
	271	C60H112N4O23	628.3853	2.4	36.2	271	34.32	8.0	even	ok
	272	C44H111N22NaO10P2S2	628.3828	-1.6	36.3	273	46.43	2.0	even	ok
	273	C42H105N28O10PS2	628.3868	4.8	36.4	274	10.05	6.0	even	ok
	274	C53H103N24NaO4P2S	628.3838	0.0	36.5	275	74.16	16.0	even	ok
	275	C45H97N34O3PS2	628.3825	-2.0	36.6	276	38.59	16.0	even	ok
	276	C42H106N24NaO14PS	628.3833	-0.9	36.7	277	57.61	3.0	even	ok
	277	C36H92N42O5S2	628.3833	-0.8	36.7	278	58.79	13.0	even	ok
	278	C39H97N34NaO10S	628.3867	4.6	36.7	279	11.05	9.0	even	ok
	279	C43H97N30O12P	628.3814	-3.8	36.8	280	17.13	12.0	even	ok
	280	C45H114N14NaO20PS	628.3839	0.2	36.8	281	70.55	-3.0	even	ok
	281	C44H100N30O9S2	628.3860	3.4	36.8	282	20.56	11.0	even	ok
	282	C46H110N22O10P2S2	628.3840	0.3	36.8	283	67.45	5.0	even	ok
	283	C43H105N26O11PS2	628.3812	-4.2	36.8	284	13.97	6.0	even	ok
	284	C46H106N24NaO9PS2	628.3820	-2.9	36.9	285	26.82	7.0	even	ok
	285	C49H88N38OS	628.3857	2.9	37.0	286	26.66	26.0	even	ok
	286	C43H94N36O5P2	628.3836	-0.4	37.0	287	53.41	17.0	even	ok
	287	C42H93N34NaO10	628.3850	1.9	37.1	288	40.23	14.0	even	ok
	288	C37H99N38NaO2P2S2	628.3808	-4.8	37.1	289	9.73	9.0	even	ok
	289	C43H109N20O18PS	628.3838	-0.0	37.1	290	72.27	1.0	even	ok
	290	C45H106N22O15P2	628.3836	-0.4	37.1	291	66.72	6.0	even	ok
	291	C35H94N44P2S2	628.3807	-5.0	37.2	292	8.47	13.0	even	ok
	292	C38H97N36NaO7S2	628.3835	-0.6	37.2	293	62.08	9.0	even	ok
	293	C51H94N32NaOPS	628.3817	-3.4	37.2	294	20.40	22.0	even	ok
	294	C35H93N44O2PS2	628.3848	1.6	37.2	295	45.64	13.0	even	ok
	295	C47H116N8O27S	628.3830	-1.4	37.3	296	48.80	-5.0	even	ok
	296	C37H98N38NaO4PS2	628.3849	1.8	37.4	297	41.96	9.0	even	ok
	297	C40H101N30O12PS	628.3831	-1.1	37.4	298	52.69	7.0	even	ok
	298	C41H85N44O2P	628.3814	-3.8	37.5	299	13.39	23.0	even	ok
	299	C39H98N34NaO8PS	628.3826	-2.0	37.5	300	38.79	9.0	even	ok
	300	C50H121NaO32	628.3864	4.1	37.5	301	14.64	-9.0	even	ok
	301	C44H110N22NaO12PS2	628.3870	5.0	37.6	302	8.47	2.0	even	ok

Compound Spectrum SmartFormula Report

Meas. m/z	#	Ion Formula	m/z	err [ppm]	mSigma	# mSigma	Score	rdb	e¯ Conf	N-Rule
	302	C54H108N14O17S	628.3863	4.0	37.7	303	15.28	9.0	even	ok
	303	C37H92N40O8S	628.3866	4.4	37.7	304	12.27	13.0	even	ok
	304	C49H121N2NaO29S	628.3831	-1.1	37.7	305	52.01	-9.0	even	ok
	305	C47H106N26O6P2S2	628.3847	1.4	37.8	306	47.39	10.0	even	ok
	306	C56H114N8NaO17PS	628.3823	-2.4	37.8	307	32.91	5.0	even	ok
	307	C46H117N10O24PS	628.3845	1.0	37.9	308	53.59	-5.0	even	ok
	308	C51H2NNaO32P2S	628.3821	-2.7	37.9	309	28.21	53.0	even	ok
	309	C48H122N4NaO26PS	628.3846	1.2	38.0	310	49.73	-9.0	even	ok
	310	C36H99N40NaOP2S2	628.3864	4.2	38.2	311	13.64	9.0	even	ok
	311	C56H120O27S	628.3863	4.0	38.2	312	15.00	-2.0	even	ok
	312	C36H89N44NaO4S	628.3860	3.5	38.2	313	19.15	15.0	even	ok
	313	C45H110N20NaO13PS2	628.3813	-4.0	38.3	314	15.21	2.0	even	ok
	314	C58H123NaO20P2S	628.3845	1.1	38.3	315	51.96	-1.0	even	ok
	315	C54H94N30O2P2	628.3820	-2.9	38.3	316	25.76	25.0	even	ok
	316	C43H104N26O13S2	628.3853	2.4	38.3	317	32.23	6.0	even	ok
	317	C50H122NaO30P	628.3823	-2.5	38.3	318	30.63	-9.0	even	ok
	318	C54H106N20O8P2S	628.3844	0.9	38.4	319	55.74	14.0	even	ok
	319	C40H81N48Na	628.3850	1.9	38.4	320	31.21	25.0	even	ok
	320	C49H118N12O16P2S2	628.3847	1.4	38.4	321	55.75	-1.0	even	ok
	321	C44H115N16NaO17P2S	628.3854	2.6	38.5	322	29.82	-3.0	even	ok
	322	C48H116N6O30	628.3862	3.8	38.5	323	16.10	-5.0	even	ok
	323	C38H102N34O6P2S2	628.3814	-3.9	38.6	324	15.24	7.0	even	ok
	324	C37H93N40O6PS	628.3825	-2.2	38.6	325	34.53	13.0	even	ok
	325	C45H96N34O5S2	628.3867	4.5	38.6	326	11.00	16.0	even	ok
	326	C48H115N16NaO12P2S2	628.3842	0.6	38.7	327	60.39	1.0	even	ok
	327	C54H93N30O4P	628.3861	3.7	38.7	328	17.55	25.0	even	ok
	328	C57H102N20O8P2	628.3827	-1.8	38.7	329	39.78	19.0	even	ok
	329	C51H93N32NaO3S	628.3858	3.1	38.7	330	23.00	22.0	even	ok
	330	C47H102N28NaO5PS2	628.3827	-1.8	38.7	331	39.74	12.0	even	ok
	331	C46H105N24NaO11S2	628.3861	3.7	38.7	332	17.45	7.0	even	ok
	332	C52H97N28O5PS	628.3822	-2.6	38.8	333	29.07	20.0	even	ok
	333	C56H99N24NaO4P2	628.3822	-2.7	38.8	334	28.04	21.0	even	ok
	334	C47H123N6NaO23P2S	628.3861	3.6	38.9	335	17.77	-9.0	even	ok
	335	C40H107N28NaO8P2S2	628.3815	-3.7	38.9	337	16.99	3.0	even	ok
	336	C44H103N26NaO11P2	628.3831	-1.2	38.9	338	49.23	8.0	even	ok
	337	C41H107N26NaO11P2S	628.3848	1.5	39.0	339	44.64	3.0	even	ok
	338	C47H113N10NaO26	628.3857	3.0	39.0	340	24.21	-3.0	even	ok
	339	C38H101N34O8PS2	628.3855	2.6	39.0	341	28.46	7.0	even	ok
	340	C42H110N22O15P2S	628.3853	2.3	39.1	342	32.22	1.0	even	ok
	341	C45H114N18O14P2S2	628.3834	-0.7	39.1	343	56.95	0.0	even	ok
	342	C45H118N12O21P2S	628.3860	3.4	39.1	344	19.76	-5.0	even	ok
	343	C49H126O28P2S	628.3810	-4.5	39.2	345	11.14	-11.0	even	ok
	344	C53H90N34NaP	628.3856	2.8	39.2	346	26.07	27.0	even	ok
	345	C39H100N32O11S2	628.3840	0.2	39.3	347	64.85	7.0	even	ok
	346	C48H118N10NaO19PS2	628.3820	-2.9	39.3	348	30.26	-4.0	even	ok
	347	C46H112N16O19S2	628.3860	3.5	39.3	349	22.99	0.0	even	ok

Compound Spectrum SmartFormula Report

Meas. m/z	#	Ion Formula	m/z	err [ppm]	mSigma	# mSigma	Score	rdb	e¯ Conf	N-Rule
	348	C36H90N44NaO2PS	628.3819	-3.0	39.3	350	23.38	15.0	even	ok
	349	C56H98N24NaO6P	628.3863	3.9	39.4	351	15.31	21.0	even	ok
	350	C48H117N6O28P	628.3821	-2.7	39.4	352	27.00	-5.0	even	ok
	351	C37H102N36O5P2S2	628.3870	5.0	39.5	353	7.96	7.0	even	ok
	352	C57H101N20O10P	628.3868	4.7	39.5	354	9.40	19.0	even	ok
	353	C34H84N50O2S	628.3859	3.3	39.5	355	20.67	19.0	even	ok
	354	C40H106N28NaO10PS2	628.3856	2.8	39.5	356	25.45	3.0	even	ok
	355	C49H125O30PS	628.3852	2.1	39.6	357	34.90	-11.0	even	ok
	356	C59H107N14NaO10P2	628.3828	-1.6	39.7	358	42.04	15.0	even	ok
	357	C45H109N20NaO15S2	628.3855	2.6	39.8	359	28.14	2.0	even	ok
	358	C56H113N8NaO19S	628.3865	4.2	39.8	360	12.76	5.0	even	ok
	359	C42H91N40NaOP2	628.3831	-1.2	39.8	361	38.36	19.0	even	ok
	360	C48H122N8O20P2S2	628.3841	0.3	39.9	362	74.41	-6.0	even	ok
	361	C49H111N20NaO8P2S2	628.3849	1.6	39.9	363	41.38	6.0	even	ok
	362	C39H102N32O9P2S	628.3846	1.3	40.0	364	46.94	7.0	even	ok
	363	C41H105N26NaO13S2	628.3841	0.5	40.1	365	59.72	3.0	even	ok
	364	C48H108N18O16S2	628.3811	-4.4	40.1	366	13.38	5.0	even	ok
	365	C47H109N20O13PS2	628.3826	-2.0	40.1	367	35.25	5.0	even	ok
	366	C55H108N12O18S	628.3807	-5.0	40.1	368	8.02	9.0	even	ok
	367	C48H126N2O27P2S	628.3866	4.5	40.1	369	10.77	-11.0	even	ok
	368	C47H114N10NaO24P	628.3816	-3.6	40.2	370	17.65	-3.0	even	ok
	369	C60H110N10O14P2	628.3834	-0.8	40.2	371	54.85	13.0	even	ok
	370	C51H123N6NaO18P2S2	628.3849	1.6	40.2	372	49.07	-5.0	even	ok
	371	C56H111N14NaO10P2S	628.3845	1.1	40.3	373	49.33	10.0	even	ok
	372	C45H108N16O24	628.3856	2.8	40.3	374	25.87	1.0	even	ok
	373	C49H113N14NaO17S2	628.3868	4.7	40.4	375	10.96	1.0	even	ok
	374	C52H96N28O7S	628.3863	4.0	40.5	376	14.24	20.0	even	ok
	375	C47H119N12NaO16P2S2	628.3835	-0.5	40.5	377	58.48	-4.0	even	ok
	376	C38H99N36NaO5P2S	628.3841	0.4	40.5	378	60.01	9.0	even	ok
	377	C57H117N4O21PS	628.3829	-1.5	40.5	379	42.37	3.0	even	ok
	378	C52H84N38O	628.3840	0.2	40.6	380	63.13	31.0	even	ok
	379	C59H106N14NaO12P	628.3869	5.0	40.6	381	7.95	15.0	even	ok
	380	C54H102N22NaO7PS	628.3823	-2.4	40.6	382	30.39	16.0	even	ok
	381	C34H85N50PS	628.3818	-3.3	40.7	383	20.25	19.0	even	ok
	382	C46H96N32O6S2	628.3811	-4.4	40.7	384	10.90	16.0	even	ok
	383	C47H101N28NaO7S2	628.3868	4.7	40.8	385	9.08	12.0	even	ok
	384	C48H117N10NaO21S2	628.3861	3.7	40.9	386	19.66	-4.0	even	ok
	385	C41H110N24O12P2S2	628.3820	-2.9	40.9	387	24.36	1.0	even	ok
	386	C55H92N28O7	628.3846	1.3	41.1	388	45.14	25.0	even	ok
	387	C54H89N32NaO3	628.3841	0.4	41.2	389	58.44	27.0	even	ok
	388	C57H98N22NaO7P	628.3807	-5.1	41.3	390	7.35	21.0	even	ok
	389	C46H113N16O17PS2	628.3819	-3.1	41.3	391	21.54	0.0	even	ok
	390	C49H123N2NaO27P2	628.3838	-0.1	41.3	392	63.36	-9.0	even	ok
	391	C50H127N2NaO22P2S2	628.3842	0.6	41.4	393	67.14	-10.0	even	ok
	392	C45H117N12O21PS2	628.3812	-4.2	41.4	394	14.90	-5.0	even	ok
	393	C44H105N20NaO20	628.3850	1.9	41.4	395	35.71	3.0	even	ok

Compound Spectrum SmartFormula Report

Meas. m/z	#	Ion Formula	m/z	err [ppm]	mSigma	# mSigma	Score	rdb	e¯ Conf	N-Rule
	394	C62H115N4NaO16P2	628.3835	-0.5	41.5	396	56.40	9.0	even	ok
	395	C45H109N16O22P	628.3814	-3.8	41.5	397	15.14	1.0	even	ok
	396	C58H101N18O11P	628.3812	-4.2	41.6	398	12.11	19.0	even	ok
	397	C52H119N10NaO14P2S2	628.3855	2.7	41.6	399	30.84	0.0	even	ok
	398	C43H115N18NaO14P2S2	628.3822	-2.6	41.6	400	26.40	-3.0	even	ok
	399	C50H117N10O19PS2	628.3832	-1.0	41.7	401	59.09	-1.0	even	ok
	400	C41H109N24O14PS2	628.3862	3.7	41.7	402	15.86	1.0	even	ok
	401	C48H102N30O2P2S2	628.3854	2.5	41.7	403	28.27	15.0	even	ok
	402	C36H94N42O3P2S	628.3840	0.2	41.8	404	61.52	13.0	even	ok
	403	C48H105N24O9PS2	628.3832	-1.0	41.9	405	48.83	10.0	even	ok
	404	C42H94N34NaO8P	628.3809	-4.7	41.9	406	7.39	14.0	even	ok
	405	C50H113N12NaO18S2	628.3812	-4.2	42.0	407	14.40	1.0	even	ok
	406	C47H108N20O15S2	628.3867	4.5	42.0	408	9.96	5.0	even	ok
	407	C40H88N40O8	628.3849	1.7	42.1	409	30.55	18.0	even	ok
	408	C49H114N14NaO15PS2	628.3827	-1.8	42.1	410	36.33	1.0	even	ok
	409	C57H97N22NaO9	628.3848	1.5	42.1	411	40.65	21.0	even	ok
	410	C46H122N8NaO22PS2	628.3870	5.0	42.2	412	8.92	-9.0	even	ok
	411	C57H113N6NaO20S	628.3809	-4.7	42.3	413	8.67	5.0	even	ok
	412	C49H121N6O23PS2	628.3826	-2.0	42.3	414	39.92	-6.0	even	ok
	413	C54H101N22NaO9S	628.3865	4.2	42.4	415	11.94	16.0	even	ok
	414	C44H114N16NaO17PS2	628.3807	-5.0	42.5	416	7.27	-3.0	even	ok
	415	C47H122N6NaO23PS2	628.3814	-3.9	42.5	417	16.32	-9.0	even	ok
	416	C43H114N18NaO16PS2	628.3863	3.9	42.5	418	13.78	-3.0	even	ok
	417	C45H116N12O23S2	628.3853	2.4	42.5	419	34.28	-5.0	even	ok
	418	C42H108N22O17S2	628.3847	1.3	42.6	420	42.83	1.0	even	ok
	419	C57H114N10O14P2S	628.3850	1.9	42.6	421	34.32	8.0	even	ok
	420	C47H118N8O25P2	628.3836	-0.3	42.6	422	57.63	-5.0	even	ok
	421	C57H116N4O23S	628.3870	5.0	42.6	423	7.11	3.0	even	ok
	422	C63H118O20P2	628.3840	0.3	42.6	424	57.93	7.0	even	ok
	423	C58H100N18O13	628.3853	2.4	42.6	425	28.93	19.0	even	ok
	424	C55H105N18O11PS	628.3829	-1.5	42.7	426	39.78	14.0	even	ok
	425	C44H106N20NaO18P	628.3809	-4.6	42.8	427	9.06	3.0	even	ok
	426	C60H106N12NaO13P	628.3813	-4.0	42.8	428	13.23	15.0	even	ok
	427	C42H100N26O18	628.3849	1.7	42.9	429	37.16	7.0	even	ok
	428	C48H101N26NaO8S2	628.3812	-4.2	43.0	430	11.63	12.0	even	ok
	429	C53H96N26O8S	628.3807	-5.0	43.0	431	7.36	20.0	even	ok
	430	C50H114N16O12P2S2	628.3854	2.5	43.2	432	27.07	4.0	even	ok
	431	C34H93N42NaO5S2	628.3821	-2.7	43.2	433	24.09	10.0	even	ok
	432	C52H126N2O22P2S2	628.3854	2.5	43.4	434	32.18	-7.0	even	ok
	433	C48H98N32NaOPS2	628.3834	-0.8	43.4	435	49.99	17.0	even	ok
	434	C32H88N48O3S2	628.3820	-3.0	43.4	436	21.65	14.0	even	ok
	435	C51H113N14O15PS2	628.3839	0.1	43.5	437	72.01	4.0	even	ok
	436	C42H109N18NaO21S	628.3811	-4.3	43.5	438	10.60	-2.0	even	ok
	437	C52H122N4NaO21PS2	628.3834	-0.7	43.6	439	59.93	-5.0	even	ok
	438	C46H115N12NaO21P2	628.3831	-1.2	43.6	440	43.35	-3.0	even	ok
	439	C44H113N16NaO19S2	628.3848	1.5	43.6	441	38.45	-3.0	even	ok

Compound Spectrum SmartFormula Report

Meas. m/z	#	Ion Formula	m/z	err [ppm]	mSigma	# mSigma	Score	rdb	e¯ Conf	N-Rule
	440	C47H126N4O24P2S2	628.3834	-0.7	43.7	442	60.34	-11.0	even	ok
	441	C61H109N8O17P	628.3819	-3.1	43.7	443	19.88	13.0	even	ok
	442	C47H121N6NaO25S2	628.3855	2.6	43.7	444	30.13	-9.0	even	ok
	443	C51H126NaO25PS2	628.3827	-1.8	43.9	445	41.49	-10.0	even	ok
	444	C49H120N6O25S2	628.3867	4.5	43.9	446	11.28	-6.0	even	ok
	445	C60H105N12NaO15	628.3855	2.6	44.0	447	25.33	15.0	even	ok
	446	C45H117N8NaO27S	628.3818	-3.3	44.0	448	18.44	-8.0	even	ok
	447	C44H118N14O18P2S2	628.3827	-1.8	44.0	449	34.80	-5.0	even	ok
	448	C50H107N24NaO4P2S2	628.3855	2.7	44.0	450	24.10	11.0	even	ok
	449	C40H89N40O6P	628.3808	-4.9	44.0	451	6.08	18.0	even	ok
	450	C40H104N24O19S	628.3810	-4.6	44.1	452	9.16	2.0	even	ok
	451	C50H110N18NaO11PS2	628.3834	-0.8	44.1	453	49.10	6.0	even	ok
	452	C42H98N32O9P2	628.3829	-1.4	44.2	454	31.56	12.0	even	ok
	453	C43H112N14O25S	628.3816	-3.5	44.2	455	16.40	-4.0	even	ok
	454	C42H101N26O16P	628.3808	-4.9	44.4	457	7.56	7.0	even	ok
	455	C51H109N16NaO14S2	628.3819	-3.1	44.5	458	23.32	6.0	even	ok
	456	C41H97N30NaO14	628.3844	0.8	44.5	459	47.32	9.0	even	ok
	457	C35H96N38O9S2	628.3826	-1.9	44.5	461	32.99	8.0	even	ok
	458	C39H85N44NaO4	628.3844	0.8	44.5	462	37.89	20.0	even	ok
	459	C41H94N36NaO7P	628.3865	4.3	44.5	463	8.50	14.0	even	ok
	460	C55H104N18O13S	628.3870	5.0	44.6	464	6.76	14.0	even	ok
	461	C37H101N32NaO11S2	628.3828	-1.7	44.6	465	35.71	4.0	even	ok
	462	C59H119N4NaO16P2S	628.3852	2.2	44.7	466	29.61	4.0	even	ok
	463	C53H122N6O18P2S2	628.3861	3.5	44.7	467	18.86	-2.0	even	ok
	464	C33H94N44NaO2PS2	628.3836	-0.4	44.7	468	53.95	10.0	even	ok
	465	C57H110N12NaO13PS	628.3830	-1.3	44.7	469	40.52	10.0	even	ok
	466	C46H123N8NaO20P2S2	628.3829	-1.6	45.0	471	36.70	-9.0	even	ok
	467	C55H101N20NaO10S	628.3808	-4.8	45.0	472	7.98	16.0	even	ok
	468	C44H117N14O20PS2	628.3868	4.8	45.0	473	7.87	-5.0	even	ok
	469	C61H108N8O19	628.3860	3.4	45.0	474	16.54	13.0	even	ok
	470	C50H120N4O26S2	628.3811	-4.4	45.1	475	11.68	-6.0	even	ok
	471	C43H106N22NaO17P	628.3865	4.3	45.1	476	10.41	3.0	even	ok
	472	C58H116N2O24S	628.3814	-3.9	45.1	477	12.99	3.0	even	ok
	473	C41H109N20NaO20S	628.3867	4.6	45.1	478	8.72	-2.0	even	ok
	474	C44H110N18O19P2	628.3829	-1.4	45.1	479	38.49	1.0	even	ok
	475	C54H99N28NaP2S	628.3845	1.1	45.2	481	43.09	21.0	even	ok
	476	C31H89N50PS2	628.3835	-0.6	45.2	482	50.01	14.0	even	ok
	477	C48H125N2O27PS2	628.3819	-3.1	45.2	483	23.22	-11.0	even	ok
	478	C51H116N8O22S2	628.3817	-3.3	45.2	484	20.57	-1.0	even	ok
	479	C46H120N4O31S	628.3823	-2.4	45.2	485	26.15	-10.0	even	ok
	480	C63H114N2NaO19P	628.3820	-2.9	45.2	486	21.10	9.0	even	ok
	481	C43H117N12NaO23S2	628.3841	0.5	45.4	487	61.52	-8.0	even	ok
	482	C36H102N34NaO8PS2	628.3843	0.7	45.5	488	47.76	4.0	even	ok
	483	C44H118N10NaO24PS	628.3833	-0.9	45.5	489	45.19	-8.0	even	ok
	484	C53H118N8NaO17PS2	628.3840	0.3	45.6	490	63.90	0.0	even	ok
	485	C34H97N40O6PS2	628.3841	0.5	45.6	491	50.73	8.0	even	ok

Compound Spectrum SmartFormula Report

Meas. m/z	#	Ion Formula	m/z	err [ppm]	mSigma	# mSigma	Score	rdb	e⁻ Conf	N-Rule
	486	C41H110N20NaO18PS	628.3826	-2.0	45.6	492	31.06	-2.0	even	ok
	487	C51H125NaO27S2	628.3868	4.8	45.6	493	9.40	-10.0	even	ok
	488	C49H2N5NaO28P2S2	628.3845	1.0	45.7	494	51.61	53.0	even	ok
	489	C38H101N30NaO14S	628.3860	3.5	45.7	495	15.46	4.0	even	ok
	490	C47H92N36O2S2	628.3817	-3.4	45.8	496	16.72	21.0	even	ok
	491	C39H104N26O18S	628.3866	4.4	45.8	497	9.74	2.0	even	ok
	492	C42H113N16O22PS	628.3831	-1.1	46.0	498	41.53	-4.0	even	ok
	493	C39H92N36O12	628.3842	0.6	46.2	499	48.06	13.0	even	ok
	494	C49H104N22O12S2	628.3817	-3.4	46.2	500	16.57	10.0	even	ok
	495	C47H126NaO30PS	628.3839	0.2	46.2	501	54.18	-14.0	even	ok
	496	C38H104N28O15S2	628.3833	-0.8	46.3	502	45.11	2.0	even	ok
	497	C49H101N28O5PS2	628.3839	0.1	46.3	503	55.33	15.0	even	ok
	498	C45H121N6O28PS	628.3838	-0.0	46.4	504	55.93	-10.0	even	ok
	499	C39H105N26O16PS	628.3825	-2.2	46.4	505	27.80	2.0	even	ok
	500	C41H95N36NaO5P2	628.3824	-2.3	46.5	506	21.40	14.0	even	ok
648.3811	1	C25H47N17O2P	648.3831	3.1	30.2	1	43.94	12.0	even	ok
	2	C25H43N19NaO	648.3790	-3.2	30.4	2	40.71	14.0	even	ok
	3	C21H43N23P	648.3804	-1.1	30.7	3	73.52	13.0	even	ok
	4	C23H48N17NaO2P	648.3807	-0.7	32.8	4	80.10	9.0	even	ok
	5	C20H47N19O4P	648.3791	-3.1	33.7	5	38.59	8.0	even	ok
	6	C19H44N23NaP	648.3780	-4.8	34.8	6	10.99	10.0	even	ok
	7	C27H42N19O	648.3814	0.5	37.0	7	100.00	17.0	even	ok
	8	C19H48N21OP2	648.3820	1.5	37.7	8	53.75	8.0	even	ok
	9	C21H53N15NaO3P2	648.3823	1.9	38.9	9	44.13	4.0	even	ok
	10	C15H39N29Na	648.3835	3.8	43.0	10	16.16	11.0	even	ok
	11	C17H49N21NaOP2	648.3796	-2.2	46.2	11	31.25	5.0	even	ok
	12	C22H52N17P2S	648.3782	-4.5	48.7	12	12.31	7.0	even	ok
	13	C18H52N17O5P2	648.3807	-0.6	49.3	13	51.60	3.0	even	ok
	14	C18H47N23PS	648.3838	4.1	49.5	14	14.69	8.0	even	ok
	15	C22H47N19NaOS	648.3824	2.0	50.1	15	40.91	9.0	even	ok
	16	C24H57N11NaO2P2S	648.3785	-4.0	50.8	16	14.93	3.0	even	ok
	17	C20H52N17NaO2PS	648.3840	4.5	51.2	17	10.73	4.0	even	ok
	18	C17H51N15NaO10	648.3836	3.8	51.4	18	16.62	0.0	even	ok
	19	C19H46N21O3S	648.3808	-0.5	52.0	19	65.24	8.0	even	ok
	20	C16H48N23NaPS	648.3814	0.4	52.9	20	65.10	5.0	even	ok
	21	C15H42N27OS	648.3781	-4.6	53.0	21	9.62	9.0	even	ok
	22	C26H56N11O2P2S	648.3809	-0.3	53.1	22	66.47	6.0	even	ok
	23	C21H51N15NaO5S	648.3811	-0.1	53.9	23	69.31	4.0	even	ok
	24	C23H50N15O5S	648.3835	3.6	54.2	24	16.53	7.0	even	ok
	25	C17H47N21NaO3S	648.3784	-4.2	54.5	25	11.93	5.0	even	ok
	26	C25H60N7O6P2S	648.3796	-2.4	54.8	26	30.16	1.0	even	ok
	27	C28H61N5NaO4P2S	648.3812	0.1	55.4	27	65.10	2.0	even	ok
	28	C25H55N9NaO7S	648.3837	4.1	56.4	28	12.11	3.0	even	ok
	29	C29H57N9NaP2S	648.3825	2.2	56.5	29	31.06	7.0	even	ok
	30	C17H51N19O4PS	648.3824	2.1	56.6	30	32.66	3.0	even	ok
	31	C27H65NNaO8P2S	648.3798	-1.9	56.9	31	33.74	-3.0	even	ok

Compound Spectrum SmartFormula Report

Meas. m/z	#	Ion Formula	m/z	err [ppm]	mSigma	# mSigma	Score	rdb	e¯ Conf	N-Rule
	32	C27H55N9O5PS	648.3779	-4.9	57.1	32	7.01	6.0	even	ok
	33	C22H54N11O9S	648.3821	1.6	57.9	33	37.57	2.0	even	ok
	34	C19H56N13NaO6PS	648.3827	2.5	58.0	34	25.88	-1.0	even	ok
	35	C18H50N17O7S	648.3794	-2.6	58.3	35	24.82	3.0	even	ok
	36	C28H51N13OPS	648.3792	-2.9	58.6	36	21.39	11.0	even	ok
	37	C24H64N3O10P2S	648.3782	-4.4	59.2	37	8.91	-4.0	even	ok
	38	C29H64NO8P2S	648.3822	1.8	59.3	38	33.42	0.0	even	ok
	39	C29H60N3NaO7PS	648.3782	-4.5	59.4	39	8.54	2.0	even	ok
	40	C24H59N5NaO11S	648.3824	2.0	59.8	40	29.78	-2.0	even	ok
	41	C20H55N11NaO9S	648.3797	-2.1	59.9	41	28.37	-1.0	even	ok
	42	C30H60N5O4P2S	648.3836	3.8	60.2	42	12.29	5.0	even	ok
	43	C30H56N7NaO3PS	648.3795	-2.4	61.0	43	24.05	7.0	even	ok
	44	C20H59N9O10PS	648.3838	4.1	61.8	44	9.77	-3.0	even	ok
	45	C22H64N3NaO12PS	648.3841	4.6	63.4	45	7.13	-7.0	even	ok
	46	C21H58N7O13S	648.3808	-0.5	63.8	46	44.36	-3.0	even	ok
	47	C25H62NO15S	648.3835	3.7	63.9	47	11.86	-4.0	even	ok
	48	C31H59N3O7PS	648.3806	-0.8	64.6	48	39.32	5.0	even	ok
	49	C21H61N11NaO2P2S2	648.3819	1.2	65.2	49	42.30	-2.0	even	ok
	50	C23H56N13NaOPS2	648.3802	-1.4	65.4	50	39.26	3.0	even	ok
	51	C23H63NNaO15S	648.3811	-0.0	65.5	51	47.12	-7.0	even	ok
	52	C23H60N11O2P2S2	648.3843	4.9	65.8	52	6.66	1.0	even	ok
	53	C20H55N15O3PS2	648.3786	-3.9	66.1	53	12.24	2.0	even	ok
	54	C22H60N9NaO5PS2	648.3789	-3.4	67.9	54	14.48	-2.0	even	ok
	55	C32H55N7O3PS	648.3819	1.3	68.1	55	29.39	10.0	even	ok
	56	C25H55N13OPS2	648.3826	2.3	68.2	56	24.23	6.0	even	ok
	57	C25H51N15NaS2	648.3785	-3.9	68.4	57	10.91	8.0	even	ok
	58	C22H64N7O6P2S2	648.3829	2.8	68.8	58	18.99	-4.0	even	ok
	59	C24H59N9O5PS2	648.3813	0.3	69.0	59	48.71	1.0	even	ok
	60	C32H51N9NaO2S	648.3779	-5.0	69.6	60	4.37	12.0	even	ok
	61	C27H60N7NaO3PS2	648.3829	2.8	70.3	61	18.47	2.0	even	ok
	62	C34H60NNaO5PS	648.3822	1.7	70.5	62	22.98	6.0	even	ok
	63	C24H69NNaO8P2S2	648.3832	3.3	70.6	63	14.38	-8.0	even	ok
	64	C19H56N17P2S2	648.3816	0.7	70.9	64	31.69	2.0	even	ok
	65	C26H64N3NaO7PS2	648.3816	0.7	71.0	65	39.90	-3.0	even	ok
	66	C23H63N5O9PS2	648.3799	-1.8	71.6	66	26.80	-4.0	even	ok
	67	C26H54N11O4S2	648.3796	-2.3	71.9	67	21.69	6.0	even	ok
	68	C25H58N7O8S2	648.3783	-4.3	72.3	68	7.40	1.0	even	ok
	69	C33H54N5O6S	648.3789	-3.3	72.8	69	10.18	10.0	even	ok
	70	C27H50N15S2	648.3810	-0.2	73.2	70	42.33	11.0	even	ok
	71	C28H63N3O7PS2	648.3840	4.4	73.8	71	6.66	0.0	even	ok
	72	C28H59N5NaO6S2	648.3799	-1.8	74.0	72	23.89	2.0	even	ok
	73	C27H63NNaO10S2	648.3786	-3.9	74.4	73	8.81	-3.0	even	ok
	74	C29H55N9NaO2S2	648.3812	0.2	75.4	74	38.87	7.0	even	ok
	75	C35H56N5NaOPS	648.3835	3.8	75.9	75	7.14	11.0	even	ok
	76	C29H62NO10S2	648.3810	-0.2	77.6	76	35.93	0.0	even	ok
	77	C34H50N9O2S	648.3803	-1.3	78.4	77	19.92	15.0	even	ok

Compound Spectrum SmartFormula Report

Meas. m/z	#	Ion Formula	m/z	err [ppm]	mSigma	# mSigma	Score	rdb	e¯ Conf	N-Rule
	78	C30H58N5O6S2	648.3823	1.9	78.7	78	19.67	5.0	even	ok
	79	C33H64NO3P2S2	648.3797	-2.1	80.6	79	16.69	4.0	even	ok
	80	C36H55N3NaO4S	648.3805	-0.8	80.7	80	20.93	11.0	even	ok
	81	C31H54N9O2S2	648.3836	3.9	81.5	81	6.54	10.0	even	ok
	82	C33H59N3NaO4S2	648.3839	4.4	83.7	82	4.64	6.0	even	ok
	83	C37H51N7NaS	648.3819	1.2	87.7	83	13.77	16.0	even	ok
	84	C35H59N3O2PS2	648.3781	-4.6	88.7	84	3.15	9.0	even	ok
	85	C38H54N3O4S	648.3830	2.9	90.4	85	6.22	14.0	even	ok
	86	C38H60NNaPS2	648.3797	-2.1	96.0	86	8.34	10.0	even	ok
	87	C39H50N7S	648.3843	4.9	98.2	87	1.36	19.0	even	ok
650.3957	1	C17H51N21NaOP2	650.3953	-0.6	21.4	1	95.66	4.0	even	ok
	2	C20H59N11NaO7P2	650.3966	1.4	24.7	2	89.99	-2.0	even	ok
	3	C18H54N17O5P2	650.3964	1.0	25.9	3	76.25	2.0	even	ok
	4	C19H53N15O8P	650.3934	-3.6	26.1	4	33.40	2.0	even	ok
	5	C19H50N21OP2	650.3977	3.1	26.1	5	32.56	7.0	even	ok
	6	C20H49N19O4P	650.3947	-1.5	27.1	6	82.88	7.0	even	ok
	7	C19H46N23NaP	650.3936	-3.2	27.5	7	30.06	9.0	even	ok
	8	C21H58N9NaO10P	650.3936	-3.2	28.3	8	39.69	-2.0	even	ok
	9	C21H55N15NaO3P2	650.3980	3.5	28.6	9	24.57	3.0	even	ok
	10	C21H62N7O11P2	650.3977	3.1	28.8	10	40.43	-4.0	even	ok
	11	C22H54N13NaO6P	650.3950	-1.1	29.3	11	91.42	3.0	even	ok
	12	C23H67NNaO13P2	650.3980	3.5	30.9	12	30.77	-8.0	even	ok
	13	C22H61N5O14P	650.3947	-1.5	32.3	13	73.50	-4.0	even	ok
	14	C23H57N9O10P	650.3961	0.5	32.7	14	100.00	1.0	even	ok
	15	C25H62N3NaO12P	650.3963	1.0	35.0	15	82.44	-3.0	even	ok
	16	C24H56N7O13	650.3931	-4.1	37.1	16	19.54	1.0	even	ok
	17	C22H44N21O3	650.3931	-4.1	37.2	17	19.31	12.0	even	ok
	18	C24H53N13O6P	650.3974	2.6	37.3	18	41.06	6.0	even	ok
	19	C21H45N23P	650.3960	0.5	37.7	19	66.21	12.0	even	ok
	20	C24H49N15NaO5	650.3933	-3.6	39.3	20	23.27	8.0	even	ok
	21	C26H61NNaO15	650.3933	-3.6	39.4	21	23.39	-3.0	even	ok
	22	C26H58N7NaO8P	650.3977	3.0	39.5	22	31.60	2.0	even	ok
	23	C23H50N17NaO2P	650.3963	1.0	40.0	23	54.42	8.0	even	ok
	24	C25H52N11O9	650.3944	-2.0	41.8	24	47.27	6.0	even	ok
	25	C27H61N3O12P	650.3987	4.7	42.3	25	11.57	0.0	even	ok
	26	C15H44N27OS	650.3937	-3.0	42.4	26	29.48	8.0	even	ok
	27	C27H57N5NaO11	650.3947	-1.6	44.0	27	52.57	2.0	even	ok
	28	C17H49N21NaO3S	650.3940	-2.6	44.6	28	33.89	4.0	even	ok
	29	C25H49N17O2P	650.3987	4.7	44.8	29	10.90	11.0	even	ok
	30	C27H54N11NaO4P	650.3990	5.1	46.7	30	7.78	7.0	even	ok
	31	C28H60NO15	650.3957	0.1	46.9	31	77.57	0.0	even	ok
	32	C25H45N19NaO	650.3947	-1.6	47.3	32	47.50	13.0	even	ok
	33	C18H52N17O7S	650.3951	-0.9	48.6	33	57.19	2.0	even	ok
	34	C21H58N13O4P2S	650.3925	-4.9	48.7	34	8.40	1.0	even	ok
	35	C26H48N15O5	650.3957	0.1	49.2	35	72.72	11.0	even	ok
	36	C19H48N21O3S	650.3964	1.1	49.3	36	52.83	7.0	even	ok

Compound Spectrum SmartFormula Report

Meas. m/z	#	Ion Formula	m/z	err [ppm]	mSigma	# mSigma	Score	rdb	e⁻ Conf	N-Rule
	37	C20H57N11NaO9S	650.3954	-0.5	50.8	37	61.05	-2.0	even	ok
	38	C23H63N7NaO6P2S	650.3928	-4.5	51.0	38	10.29	-3.0	even	ok
	39	C28H53N9NaO7	650.3960	0.5	51.1	39	60.73	7.0	even	ok
	40	C21H53N15NaO5S	650.3967	1.5	51.6	40	42.24	3.0	even	ok
	41	C22H54N17P2S	650.3939	-2.8	51.6	41	24.54	6.0	even	ok
	42	C29H56N5O11	650.3971	2.1	53.4	42	31.64	5.0	even	ok
	43	C24H59N11NaO2P2S	650.3941	-2.4	53.9	43	27.80	2.0	even	ok
	44	C24H66N3O10P2S	650.3939	-2.8	54.7	44	22.39	-5.0	even	ok
	45	C21H60N7O13S	650.3964	1.1	54.8	45	44.26	-4.0	even	ok
	46	C22H49N19NaOS	650.3980	3.6	55.1	46	14.82	8.0	even	ok
	47	C22H56N11O9S	650.3978	3.2	55.2	47	18.35	1.0	even	ok
	48	C29H54N11O2P2	650.3932	-3.9	55.6	48	12.47	10.0	even	ok
	49	C23H65NNaO15S	650.3967	1.6	57.0	49	35.31	-8.0	even	ok
	50	C25H62N7O6P2S	650.3952	-0.8	57.2	50	46.25	0.0	even	ok
	51	C31H59N5NaO4P2	650.3934	-3.5	57.5	51	14.86	6.0	even	ok
	52	C24H61N5NaO11S	650.3980	3.6	57.5	52	13.58	-3.0	even	ok
	53	C27H44N19O	650.3971	2.1	58.1	53	27.32	16.0	even	ok
	54	C32H62NO8P2	650.3945	-1.8	59.3	54	29.73	4.0	even	ok
	55	C27H67NNaO8P2S	650.3955	-0.3	59.5	55	48.66	-4.0	even	ok
	56	C29H49N13NaO3	650.3974	2.5	59.9	56	21.35	12.0	even	ok
	57	C30H52N9O7	650.3984	4.2	61.6	57	8.55	10.0	even	ok
	58	C26H58N11O2P2S	650.3965	1.3	62.0	58	32.84	5.0	even	ok
	59	C21H63N11NaO2P2S2	650.3975	2.8	62.3	59	22.06	-3.0	even	ok
	60	C32H57N3NaO9	650.3987	4.6	63.6	60	6.15	6.0	even	ok
	61	C26H54N13NaOPS	650.3925	-4.9	63.8	61	4.96	7.0	even	ok
	62	C20H57N15O3PS2	650.3942	-2.2	64.0	62	26.51	1.0	even	ok
	63	C28H63N5NaO4P2S	650.3968	1.7	64.2	63	25.91	1.0	even	ok
	64	C22H66N7O6P2S2	650.3986	4.4	65.7	64	8.03	-5.0	even	ok
	65	C22H62N9NaO5PS2	650.3945	-1.8	66.1	65	29.37	-3.0	even	ok
	66	C27H57N9O5PS	650.3936	-3.3	66.6	66	11.76	5.0	even	ok
	67	C32H55N9NaP2	650.3948	-1.4	67.1	67	26.54	11.0	even	ok
	68	C29H66NO8P2S	650.3979	3.4	67.2	68	11.08	-1.0	even	ok
	69	C24H71NNaO8P2S2	650.3989	4.9	67.8	69	5.68	-9.0	even	ok
	70	C23H58N13NaOPS2	650.3959	0.2	68.1	70	46.09	2.0	even	ok
	71	C33H58N5O4P2	650.3959	0.2	68.5	71	36.36	9.0	even	ok
	72	C29H62N3NaO7PS	650.3938	-2.9	68.9	72	13.42	1.0	even	ok
	73	C23H65N5O9PS2	650.3956	-0.2	69.5	73	44.68	-5.0	even	ok
	74	C22H52N17O2S2	650.3926	-4.8	70.0	74	5.48	6.0	even	ok
	75	C29H59N9NaP2S	650.3982	3.8	70.8	75	7.76	6.0	even	ok
	76	C24H61N9O5PS2	650.3969	1.9	71.3	76	23.64	0.0	even	ok
	77	C24H57N11NaO4S2	650.3929	-4.4	72.1	77	6.62	2.0	even	ok
	78	C34H57N3O7P	650.3929	-4.4	72.7	78	5.19	9.0	even	ok
	79	C24H64N3O12S2	650.3926	-4.8	73.3	79	4.90	-5.0	even	ok
	80	C28H53N13OPS	650.3949	-1.2	73.3	80	22.23	10.0	even	ok
	81	C26H66N3NaO7PS2	650.3972	2.3	73.4	81	18.25	-4.0	even	ok
	82	C25H57N13OPS2	650.3983	3.9	74.7	82	7.68	5.0	even	ok

Compound Spectrum SmartFormula Report

Meas. m/z	#	Ion Formula	m/z	err [ppm]	mSigma	# mSigma	Score	rdb	e⁻ Conf	N-Rule
	83	C25H60N7O8S2	650.3939	-2.7	75.3	83	14.17	0.0	even	ok
	84	C30H58N7NaO3PS	650.3952	-0.8	75.4	84	23.61	6.0	even	ok
	85	C25H53N15NaS2	650.3942	-2.3	76.0	85	16.58	7.0	even	ok
	86	C27H62N7NaO3PS2	650.3985	4.4	76.8	86	5.49	1.0	even	ok
	87	C27H65NNaO10S2	650.3942	-2.3	77.4	87	15.81	-4.0	even	ok
	88	C31H61N3O7PS	650.3962	0.8	78.0	88	21.21	4.0	even	ok
	89	C34H54N9P2	650.3972	2.3	78.4	89	12.04	14.0	even	ok
	90	C26H56N11O4S2	650.3953	-0.7	78.9	90	27.07	5.0	even	ok
	91	C36H59N3NaO2P2	650.3975	2.7	80.1	91	9.28	10.0	even	ok
	92	C28H61N5NaO6S2	650.3955	-0.2	81.0	92	28.17	1.0	even	ok
	93	C29H62N7OP2S2	650.3927	-4.6	82.3	93	3.80	4.0	even	ok
	94	C35H53N7O3P	650.3942	-2.3	82.6	94	10.20	14.0	even	ok
	95	C32H60NO10S	650.3932	-3.8	82.7	95	4.91	4.0	even	ok
	96	C27H52N15S2	650.3966	1.4	83.8	96	17.29	10.0	even	ok
	97	C29H64NO10S2	650.3966	1.4	83.9	97	17.12	-1.0	even	ok
	98	C37H58NNaO5P	650.3945	-1.9	84.3	98	11.34	10.0	even	ok
	99	C31H67NNaO3P2S2	650.3930	-4.2	84.4	99	4.53	0.0	even	ok
	100	C32H57N7O3PS	650.3976	2.9	85.6	100	6.88	9.0	even	ok
	101	C29H57N9NaO2S2	650.3969	1.8	85.8	101	13.48	6.0	even	ok
	102	C30H48N15S	650.3932	-3.8	85.9	102	4.26	15.0	even	ok
	103	C34H62NNaO5PS	650.3979	3.3	87.7	103	5.07	5.0	even	ok
	104	C32H53N9NaO2S	650.3935	-3.4	87.9	104	4.92	11.0	even	ok
	105	C30H60N5O6S2	650.3980	3.5	88.5	105	5.64	4.0	even	ok
	106	C33H56N5O6S	650.3946	-1.7	90.2	106	9.35	9.0	even	ok
	107	C33H66NO3P2S2	650.3954	-0.5	92.6	107	15.92	3.0	even	ok
	108	C38H54N5NaOP	650.3958	0.2	94.6	108	12.64	15.0	even	ok
	109	C39H57NO5P	650.3969	1.8	95.8	109	6.93	13.0	even	ok
	110	C37H48N9O2	650.3925	-4.8	97.1	110	1.37	19.0	even	ok
	111	C34H52N9O2S	650.3959	0.3	98.7	111	10.01	14.0	even	ok
	112	C39H53N3NaO4	650.3928	-4.4	98.7	112	1.66	15.0	even	ok
652.4129	1	C23H69NNaO13P2	652.4136	1.2	20.5	1	94.36	-9.0	even	ok
	2	C18H54N17O7S	652.4107	-3.3	20.6	2	38.79	1.0	even	ok
	3	C22H63N5O14P	652.4104	-3.8	20.9	3	28.39	-5.0	even	ok
	4	C17H51N21NaO3S	652.4097	-4.9	21.0	4	14.75	3.0	even	ok
	5	C20H59N11NaO9S	652.4110	-2.8	21.8	5	46.79	-3.0	even	ok
	6	C21H62N7O13S	652.4121	-1.2	22.6	6	90.03	-5.0	even	ok
	7	C23H67NNaO15S	652.4124	-0.8	24.2	7	100.00	-9.0	even	ok
	8	C25H68NO13P2	652.4160	4.9	28.3	8	12.64	-6.0	even	ok
	9	C18H51N23PS	652.4151	3.4	29.3	9	29.88	6.0	even	ok
	10	C20H56N17NaO2PS	652.4153	3.8	30.0	10	23.32	2.0	even	ok
	11	C24H65N5NaO9P2	652.4150	3.2	30.8	11	30.98	-4.0	even	ok
	12	C25H64N3NaO12P	652.4120	-1.3	31.4	12	69.38	-4.0	even	ok
	13	C16H46N25O4	652.4159	4.7	31.4	13	10.00	7.0	even	ok
	14	C22H60N11O7P2	652.4147	2.8	31.4	14	37.69	0.0	even	ok
	15	C23H59N9O10P	652.4117	-1.8	31.7	15	58.63	0.0	even	ok
	16	C19H50N21O3S	652.4121	-1.2	31.9	16	71.97	6.0	even	ok

Compound Spectrum SmartFormula Report

Meas. m/z	#	Ion Formula	m/z	err [ppm]	mSigma	# mSigma	Score	rdb	e¯ Conf	N-Rule
	17	C21H55N15NaO5S	652.4124	-0.8	32.8	17	81.13	2.0	even	ok
	18	C22H58N11O9S	652.4134	0.9	32.9	18	78.89	0.0	even	ok
	19	C24H63N5NaO11S	652.4137	1.3	34.1	19	66.11	-4.0	even	ok
	20	C22H56N13NaO6P	652.4106	-3.4	34.3	20	25.94	2.0	even	ok
	21	C25H66NO15S	652.4148	2.9	34.9	21	32.63	-6.0	even	ok
	22	C20H51N19O4P	652.4104	-3.8	35.0	22	20.17	6.0	even	ok
	23	C15H43N29Na	652.4148	3.0	35.5	23	22.82	9.0	even	ok
	24	C20H59N15O3PS2	652.4099	-4.6	36.7	24	15.72	0.0	even	ok
	25	C22H64N9NaO5PS2	652.4102	-4.1	38.4	25	19.50	-4.0	even	ok
	26	C21H57N15NaO3P2	652.4136	1.2	38.5	26	46.06	2.0	even	ok
	27	C19H52N21OP2	652.4133	0.7	39.4	27	51.67	6.0	even	ok
	28	C23H67N5O9PS2	652.4112	-2.5	40.4	28	43.23	-6.0	even	ok
	29	C27H63N3O12P	652.4144	2.3	41.0	29	36.31	-1.0	even	ok
	30	C25H64N7O6P2S	652.4109	-3.1	41.0	30	25.77	-1.0	even	ok
	31	C24H61N11NaO2P2S	652.4098	-4.7	41.2	31	10.09	1.0	even	ok
	32	C27H69NNaO8P2S	652.4111	-2.6	42.1	32	30.70	-5.0	even	ok
	33	C23H56N15O3P2	652.4160	4.9	42.2	33	8.91	5.0	even	ok
	34	C26H60N7NaO8P	652.4133	0.7	42.8	34	63.53	1.0	even	ok
	35	C28H62NO15	652.4114	-2.2	42.8	35	36.06	-1.0	even	ok
	36	C24H55N13O6P	652.4130	0.3	42.9	36	71.80	5.0	even	ok
	37	C23H64N11O2P2S2	652.4156	4.1	43.9	37	16.62	-1.0	even	ok
	38	C23H54N15O5S	652.4148	2.9	43.9	38	25.75	5.0	even	ok
	39	C25H54N11O9	652.4100	-4.3	44.1	39	11.96	5.0	even	ok
	40	C22H51N19NaOS	652.4137	1.3	44.2	40	50.63	7.0	even	ok
	41	C27H59N5NaO11	652.4103	-3.9	44.2	41	15.34	1.0	even	ok
	42	C25H59N9NaO7S	652.4150	3.3	45.0	42	20.13	1.0	even	ok
	43	C26H62N5O11S	652.4161	5.0	45.3	43	7.55	-1.0	even	ok
	44	C25H69N5NaO4P2S2	652.4158	4.6	45.5	44	12.21	-5.0	even	ok
	45	C23H60N13NaOPS2	652.4115	-2.1	46.2	45	43.72	1.0	even	ok
	46	C24H63N9O5PS2	652.4126	-0.4	47.7	46	74.58	-1.0	even	ok
	47	C26H68N3NaO7PS2	652.4129	-0.0	49.3	47	79.83	-5.0	even	ok
	48	C23H52N17NaO2P	652.4120	-1.4	51.3	48	29.74	7.0	even	ok
	49	C25H62N7O8S2	652.4096	-5.0	51.5	49	7.58	-1.0	even	ok
	50	C21H47N23P	652.4117	-1.8	51.7	50	25.03	11.0	even	ok
	51	C26H60N11O2P2S	652.4122	-1.0	52.4	51	43.11	4.0	even	ok
	52	C28H59N7O8P	652.4157	4.4	52.7	52	8.79	4.0	even	ok
	53	C30H64NNaO10P	652.4160	4.8	52.9	53	6.65	0.0	even	ok
	54	C27H67NNaO10S2	652.4099	-4.6	53.2	54	9.49	-5.0	even	ok
	55	C28H65N5NaO4P2S	652.4125	-0.6	53.3	55	47.90	0.0	even	ok
	56	C29H68NO8P2S	652.4135	1.0	53.4	56	41.46	-2.0	even	ok
	57	C27H56N11NaO4P	652.4147	2.8	54.3	57	20.26	6.0	even	ok
	58	C25H51N17O2P	652.4144	2.3	54.3	58	24.59	10.0	even	ok
	59	C29H58N5O11	652.4127	-0.2	54.5	59	51.84	4.0	even	ok
	60	C24H50N19OS	652.4161	5.0	55.4	60	5.62	10.0	even	ok
	61	C26H50N15O5	652.4114	-2.3	55.7	61	24.23	10.0	even	ok
	62	C28H55N9NaO7	652.4117	-1.8	55.9	62	28.77	6.0	even	ok

Compound Spectrum SmartFormula Report

Meas. m/z	#	Ion Formula	m/z	err [ppm]	mSigma	# mSigma	Score	rdb	e⁻ Conf	N-Rule
	63	C25H59N13OPS2	652.4139	1.6	56.1	63	38.85	4.0	even	ok
	64	C25H47N19NaO	652.4103	-3.9	57.5	64	10.10	12.0	even	ok
	65	C27H64N7NaO3PS2	652.4142	2.0	57.7	65	31.21	0.0	even	ok
	66	C25H55N15NaS2	652.4098	-4.6	58.7	66	7.89	6.0	even	ok
	67	C28H67N3O7PS2	652.4153	3.7	59.0	67	13.60	-2.0	even	ok
	68	C26H58N11O4S2	652.4109	-3.0	59.8	68	19.01	4.0	even	ok
	69	C28H63N5NaO6S2	652.4112	-2.5	61.4	69	22.06	0.0	even	ok
	70	C29H66NO10S2	652.4123	-0.9	62.8	70	39.63	-2.0	even	ok
	71	C32H64NO8P2	652.4102	-4.1	63.2	71	7.28	3.0	even	ok
	72	C30H64N5O4P2S	652.4149	3.1	64.7	72	12.14	3.0	even	ok
	73	C29H61N9NaP2S	652.4138	1.5	64.8	73	24.54	5.0	even	ok
	74	C28H52N15NaP	652.4160	4.8	65.9	74	4.35	11.0	even	ok
	75	C30H54N9O7	652.4141	1.9	66.2	75	19.98	9.0	even	ok
	76	C32H59N3NaO9	652.4144	2.3	66.7	76	16.46	5.0	even	ok
	77	C28H55N13OPS	652.4105	-3.6	66.8	77	8.82	9.0	even	ok
	78	C27H46N19O	652.4127	-0.2	67.3	78	33.37	15.0	even	ok
	79	C29H51N13NaO3	652.4130	0.2	67.5	79	33.00	11.0	even	ok
	80	C30H60N7NaO3PS	652.4108	-3.1	67.9	80	10.63	5.0	even	ok
	81	C31H63N3O7PS	652.4119	-1.5	68.0	81	21.58	3.0	even	ok
	82	C27H54N15S2	652.4123	-0.9	68.8	82	31.84	9.0	even	ok
	83	C29H59N9NaO2S2	652.4125	-0.5	70.3	83	34.47	5.0	even	ok
	84	C30H62N5O6S2	652.4136	1.1	71.4	84	26.92	3.0	even	ok
	85	C33H60N5O4P2	652.4115	-2.1	74.9	85	13.22	8.0	even	ok
	86	C32H57N9NaP2	652.4104	-3.7	76.2	86	5.70	10.0	even	ok
	87	C31H50N13O3	652.4154	3.9	78.0	87	4.77	14.0	even	ok
	88	C33H68NO3P2S2	652.4110	-2.8	78.1	88	10.59	2.0	even	ok
	89	C33H55N7NaO5	652.4157	4.3	78.4	89	3.64	10.0	even	ok
	90	C32H59N7O3PS	652.4132	0.6	79.3	90	19.11	8.0	even	ok
	91	C34H64NNaO5PS	652.4135	1.0	80.4	91	15.95	4.0	even	ok
	92	C31H58N9O2S2	652.4149	3.2	80.6	92	7.90	8.0	even	ok
	93	C33H63N3NaO4S2	652.4152	3.6	82.0	93	5.94	4.0	even	ok
	94	C33H58N5O6S	652.4102	-4.0	82.6	94	3.68	8.0	even	ok
	95	C34H56N9P2	652.4128	-0.0	86.7	95	16.33	13.0	even	ok
	96	C36H61N3NaO2P2	652.4131	0.4	87.1	96	14.44	9.0	even	ok
	97	C35H55N7O3P	652.4099	-4.6	88.9	97	1.98	13.0	even	ok
	98	C37H60NNaO5P	652.4101	-4.2	89.4	98	2.52	9.0	even	ok
	99	C35H60N5NaOPS	652.4148	3.0	91.9	99	4.22	9.0	even	ok
	100	C36H63NO5PS	652.4159	4.7	92.0	100	1.66	7.0	even	ok
	101	C34H54N9O2S	652.4116	-2.0	94.0	101	6.17	13.0	even	ok
	102	C36H59N3NaO4S	652.4118	-1.5	95.2	102	6.92	9.0	even	ok
	103	C38H60N3O2P2	652.4155	4.1	97.9	103	1.81	12.0	even	ok
692.4076	1	C23H47N23OP	692.4066	-1.4	29.9	1	100.00	13.0	even	ok
	2	C25H42N25	692.4050	-3.8	33.8	2	27.08	18.0	even	ok
	3	C21H48N23NaOP	692.4042	-4.9	35.6	3	9.31	10.0	even	ok
	4	C21H52N21O2P2	692.4083	0.9	37.8	4	74.12	8.0	even	ok
	5	C18H46N25O5	692.4108	4.6	39.3	5	13.74	9.0	even	ok

Compound Spectrum SmartFormula Report

Meas. m/z	#	Ion Formula	m/z	err [ppm]	mSigma	# mSigma	Score	rdb	e⁻ Conf	N-Rule
	6	C17H43N29NaO	692.4098	3.1	41.5	6	25.42	11.0	even	ok
	7	C17H48N27P2	692.4056	-2.9	43.5	7	26.24	9.0	even	ok
	8	C19H53N21NaO2P2	692.4059	-2.5	44.4	8	31.48	5.0	even	ok
	9	C17H50N21O9	692.4095	2.7	49.2	9	33.37	4.0	even	ok
	10	C20H47N25NaS	692.4059	-2.4	50.8	10	36.35	10.0	even	ok
	11	C20H51N23OPS	692.4100	3.4	51.9	11	20.91	8.0	even	ok
	12	C22H46N25S	692.4083	1.0	52.2	12	63.00	13.0	even	ok
	13	C24H56N17OP2S	692.4044	-4.6	52.6	13	9.58	7.0	even	ok
	14	C14H42N31O3	692.4081	0.8	52.7	14	51.14	10.0	even	ok
	15	C16H47N25NaO5	692.4084	1.2	53.3	15	43.41	6.0	even	ok
	16	C18H52N23NaOPS	692.4076	-0.1	53.8	16	81.93	5.0	even	ok
	17	C22H56N17NaO3PS	692.4103	3.8	53.8	17	15.44	4.0	even	ok
	18	C17H46N27O2S	692.4043	-4.8	54.3	18	8.04	9.0	even	ok
	19	C24H51N19NaO2S	692.4086	1.4	54.5	19	50.39	9.0	even	ok
	20	C26H61N11NaO3P2S	692.4047	-4.2	54.8	20	11.75	3.0	even	ok
	21	C21H50N21O4S	692.4070	-0.9	54.8	21	60.79	8.0	even	ok
	22	C19H51N21NaO4S	692.4046	-4.4	55.9	22	10.09	5.0	even	ok
	23	C23H55N15NaO6S	692.4073	-0.5	56.9	23	65.07	4.0	even	ok
	24	C19H55N19O5PS	692.4086	1.5	57.7	24	44.55	3.0	even	ok
	25	C28H60N11O3P2S	692.4071	-0.7	58.3	25	57.63	6.0	even	ok
	26	C25H54N15O6S	692.4097	3.0	58.5	26	21.60	7.0	even	ok
	27	C26H50N19O2S	692.4110	4.9	58.6	27	6.34	12.0	even	ok
	28	C27H64N7O7P2S	692.4058	-2.7	58.8	28	25.23	1.0	even	ok
	29	C21H60N13NaO7PS	692.4089	1.9	59.3	29	35.62	-1.0	even	ok
	30	C20H54N17O8S	692.4056	-2.8	59.9	30	22.28	3.0	even	ok
	31	C30H65N5NaO5P2S	692.4074	-0.3	60.6	31	60.54	2.0	even	ok
	32	C27H59N9NaO8S	692.4100	3.4	60.8	32	15.99	3.0	even	ok
	33	C24H58N11O10S	692.4083	1.0	60.9	33	47.18	2.0	even	ok
	34	C29H69NNaO9P2S	692.4060	-2.3	61.0	34	28.51	-3.0	even	ok
	35	C22H59N11NaO10S	692.4059	-2.4	61.7	35	25.71	-1.0	even	ok
	36	C26H68N3O11P2S	692.4044	-4.6	61.9	36	7.13	-4.0	even	ok
	37	C29H59N9O6PS	692.4041	-5.0	62.5	37	5.02	6.0	even	ok
	38	C31H61N9NaOP2S	692.4087	1.6	62.7	38	35.81	7.0	even	ok
	39	C26H63N5NaO12S	692.4086	1.5	63.0	39	37.72	-2.0	even	ok
	40	C22H63N9O11PS	692.4100	3.4	63.2	40	14.25	-3.0	even	ok
	41	C31H68NO9P2S	692.4085	1.2	64.5	41	39.13	0.0	even	ok
	42	C28H62N5O12S	692.4110	4.9	64.8	42	5.08	1.0	even	ok
	43	C30H55N13O2PS	692.4055	-3.1	64.9	43	16.10	11.0	even	ok
	44	C31H64N3NaO8PS	692.4044	-4.6	64.9	44	6.19	2.0	even	ok
	45	C24H68N3NaO13PS	692.4103	3.8	65.0	45	10.52	-7.0	even	ok
	46	C23H62N7O14S	692.4070	-0.9	65.6	46	42.42	-3.0	even	ok
	47	C21H60N17OP2S2	692.4078	0.3	66.3	47	63.34	2.0	even	ok
	48	C32H64N5O5P2S	692.4098	3.1	66.3	48	15.03	5.0	even	ok
	49	C27H66NO16S	692.4097	3.0	67.1	49	15.92	-4.0	even	ok
	50	C32H60N7NaO4PS	692.4057	-2.7	67.3	50	18.30	7.0	even	ok
	51	C23H55N19PS2	692.4061	-2.1	67.4	51	30.21	7.0	even	ok

Compound Spectrum SmartFormula Report

Meas. m/z	#	Ion Formula	m/z	err [ppm]	mSigma	# mSigma	Score	rdb	e¯ Conf	N-Rule
	52	C25H67NNaO16S	692.4073	-0.5	67.5	52	45.40	-7.0	even	ok
	53	C23H65N11NaO3P2S2	692.4081	0.7	68.1	53	52.19	-2.0	even	ok
	54	C21H63N7NaO14S	692.4046	-4.4	68.3	54	6.65	-6.0	even	ok
	55	C18H54N21O4S2	692.4104	4.0	69.1	55	10.43	3.0	even	ok
	56	C22H59N15O4PS2	692.4048	-4.1	69.2	56	9.82	2.0	even	ok
	57	C25H60N13NaO2PS2	692.4064	-1.7	69.5	57	33.46	3.0	even	ok
	58	C25H64N11O3P2S2	692.4105	4.1	69.7	58	9.16	1.0	even	ok
	59	C33H60N9OP2S	692.4111	5.1	70.2	59	3.71	10.0	even	ok
	60	C20H59N15NaO6S2	692.4106	4.4	70.9	60	7.51	-1.0	even	ok
	61	C33H63N3O8PS	692.4068	-1.2	70.9	61	31.63	5.0	even	ok
	62	C24H64N9NaO6PS2	692.4051	-3.7	71.1	62	11.76	-2.0	even	ok
	63	C27H69N5NaO5P2S2	692.4108	4.5	71.7	63	6.47	-3.0	even	ok
	64	C24H68N7O7P2S2	692.4091	2.2	71.8	64	24.77	-4.0	even	ok
	65	C33H56N11NaPS	692.4071	-0.8	71.8	65	35.08	12.0	even	ok
	66	C22H66N3O18S	692.4057	-2.8	72.2	66	14.49	-8.0	even	ok
	67	C26H63N9O6PS2	692.4075	-0.2	73.1	67	50.40	1.0	even	ok
	68	C27H59N13O2PS2	692.4088	1.8	73.1	68	28.84	6.0	even	ok
	69	C17H51N25NaS2	692.4093	2.4	73.2	69	16.97	5.0	even	ok
	70	C27H55N15NaOS2	692.4048	-4.1	73.5	70	8.08	8.0	even	ok
	71	C26H73NNaO9P2S2	692.4094	2.6	73.7	71	18.98	-8.0	even	ok
	72	C25H67N5O10PS2	692.4061	-2.1	74.8	72	23.10	-4.0	even	ok
	73	C34H59N7O4PS	692.4081	0.8	75.0	73	31.20	10.0	even	ok
	74	C28H68N3NaO8PS2	692.4078	0.2	75.1	74	45.97	-3.0	even	ok
	75	C29H64N7NaO4PS2	692.4091	2.2	75.2	75	22.24	2.0	even	ok
	76	C27H62N7O9S2	692.4045	-4.5	76.6	76	5.52	1.0	even	ok
	77	C28H58N11O5S2	692.4058	-2.6	77.0	77	17.07	6.0	even	ok
	78	C30H60N11NaPS2	692.4104	4.1	77.0	78	7.16	7.0	even	ok
	79	C38H63NO6PS	692.4108	4.6	77.2	79	4.89	9.0	even	ok
	80	C36H64NNaO6PS	692.4084	1.2	77.4	80	24.62	6.0	even	ok
	81	C24H71NO14PS2	692.4048	-4.0	78.1	81	7.06	-9.0	even	ok
	82	C29H67NNaO11S2	692.4048	-4.1	78.7	82	6.66	-3.0	even	ok
	83	C30H67N3O8PS2	692.4102	3.7	78.7	83	8.52	0.0	even	ok
	84	C29H54N15OS2	692.4072	-0.6	78.9	84	34.83	11.0	even	ok
	85	C30H63N5NaO7S2	692.4061	-2.2	79.1	85	19.01	2.0	even	ok
	86	C35H58N5O7S	692.4051	-3.6	79.8	86	7.10	10.0	even	ok
	87	C35H55N11PS	692.4095	2.7	80.9	87	10.95	15.0	even	ok
	88	C31H59N9NaO3S2	692.4074	-0.2	81.1	88	36.13	7.0	even	ok
	89	C31H66NO11S2	692.4072	-0.6	82.6	89	30.15	0.0	even	ok
	90	C37H60N5NaO2PS	692.4098	3.1	83.2	90	8.03	11.0	even	ok
	91	C34H65N5NaP2S2	692.4049	-3.9	83.5	91	6.05	6.0	even	ok
	92	C32H62N5O7S2	692.4085	1.3	84.4	92	21.87	5.0	even	ok
	93	C36H54N9O3S	692.4065	-1.6	85.8	93	14.60	15.0	even	ok
	94	C35H68NO4P2S2	692.4060	-2.4	86.7	94	12.46	4.0	even	ok
	95	C33H58N9O3S2	692.4099	3.2	87.7	95	7.66	10.0	even	ok
	96	C40H58N3O5S	692.4092	2.3	87.8	96	12.73	14.0	even	ok
	97	C38H59N3NaO5S	692.4068	-1.2	88.1	97	15.47	11.0	even	ok

Compound Spectrum SmartFormula Report

Meas. m/z	#	Ion Formula	m/z	err [ppm]	mSigma	# mSigma	Score	rdb	e¯ Conf	N-Rule
	98	C34H59N3NaO10	692.4093	2.4	88.4	98	9.29	7.0	even	ok
	99	C30H62NO16	692.4063	-1.9	88.8	99	11.53	1.0	even	ok
	100	C31H58N5O12	692.4076	0.1	89.0	100	21.78	6.0	even	ok
	101	C35H63N3NaO5S2	692.4101	3.6	89.9	101	5.50	6.0	even	ok
	102	C34H64NO9P2	692.4051	-3.7	90.1	102	4.35	5.0	even	ok
	103	C32H54N9O8	692.4090	2.0	90.6	103	10.16	11.0	even	ok
	104	C32H64NNaO11P	692.4109	4.8	90.7	104	2.02	2.0	even	ok
	105	C36H64N5P2S2	692.4073	-0.5	90.9	105	22.21	9.0	even	ok
	106	C35H55N7NaO6	692.4106	4.3	91.3	106	2.70	12.0	even	ok
	107	C39H60NNaO6P	692.4050	-3.7	91.3	107	4.00	11.0	even	ok
	108	C29H46N19O2	692.4076	0.0	91.7	108	19.42	17.0	even	ok
	109	C35H60N5O5P2	692.4064	-1.7	92.6	109	10.42	10.0	even	ok
	110	C41H59NO6P	692.4075	-0.2	92.7	110	17.65	14.0	even	ok
	111	C29H59N5NaO12	692.4052	-3.4	92.7	111	4.44	3.0	even	ok
	112	C29H63N3O13P	692.4093	2.4	93.0	112	7.39	1.0	even	ok
	113	C30H59N7O9P	692.4106	4.4	93.0	113	2.41	6.0	even	ok
	114	C30H55N9NaO8	692.4066	-1.5	93.1	114	11.21	8.0	even	ok
	115	C37H55N7O4P	692.4048	-4.1	93.3	115	2.84	15.0	even	ok
	116	C33H50N13O4	692.4103	3.9	93.4	116	3.19	16.0	even	ok
	117	C27H47N19NaO2	692.4052	-3.4	94.1	117	4.14	14.0	even	ok
	118	C38H61N3NaO3P2	692.4080	0.6	94.2	118	14.58	11.0	even	ok
	119	C31H51N13NaO4	692.4079	0.4	94.7	119	15.08	13.0	even	ok
	120	C41H54N7OS	692.4105	4.2	94.9	120	3.14	19.0	even	ok
	121	C27H54N11O10	692.4050	-3.8	95.1	121	3.14	7.0	even	ok
	122	C40H60N3O3P2	692.4104	4.1	95.1	122	2.64	14.0	even	ok
	123	C39H55N7NaOS	692.4081	0.7	95.3	123	13.46	16.0	even	ok
	124	C37H63N3O3PS2	692.4043	-4.8	95.3	124	2.04	9.0	even	ok
	125	C28H50N15O6	692.4063	-1.9	95.4	125	8.54	12.0	even	ok
	126	C36H56N9OP2	692.4078	0.2	96.2	126	15.07	15.0	even	ok
	127	C40H56N5NaO2P	692.4064	-1.8	96.6	127	8.52	16.0	even	ok
	128	C34H57N9NaOP2	692.4054	-3.3	96.7	128	4.05	12.0	even	ok
	129	C28H60N7NaO9P	692.4082	0.9	96.9	129	11.74	3.0	even	ok
	130	C43H59NNaO3S	692.4108	4.6	96.9	130	2.18	15.0	even	ok
	131	C29H56N11NaO5P	692.4096	2.8	97.0	131	5.04	8.0	even	ok
	132	C43H54N3O5	692.4058	-2.6	97.1	132	5.62	19.0	even	ok
	133	C32H47N17Na	692.4093	2.4	97.5	133	6.21	18.0	even	ok
	134	C25H52N17NaO3P	692.4069	-1.0	97.8	134	10.69	9.0	even	ok
	135	C27H64N3NaO13P	692.4069	-1.0	97.9	135	10.68	-2.0	even	ok
	136	C38H51N11P	692.4061	-2.2	98.3	136	6.56	20.0	even	ok
	137	C30H52N15NaOP	692.4109	4.8	98.4	137	1.44	13.0	even	ok
	138	C27H68NO14P2	692.4110	4.8	98.5	138	1.36	-4.0	even	ok
	139	C42H55N5O2P	692.4088	1.7	98.9	139	7.84	19.0	even	ok
	140	C26H55N13O7P	692.4080	0.5	99.2	140	12.03	7.0	even	ok
	141	C27H51N17O3P	692.4093	2.4	99.3	141	5.55	12.0	even	ok
736.4341	1	C30H66N5O13S	736.4372	4.2	30.2	1	17.36	1.0	even	ok
	2	C35H67N3O9PS	736.4330	-1.5	30.5	2	81.95	5.0	even	ok

Compound Spectrum SmartFormula Report

Meas. m/z	#	Ion Formula	m/z	err [ppm]	mSigma	# mSigma	Score	rdb	e⁻ Conf	N-Rule
	3	C33H72NO10P2S	736.4347	0.7	32.9	3	84.71	0.0	even	ok
	4	C33H68N3NaO9PS	736.4306	-4.8	33.1	4	8.34	2.0	even	ok
	5	C29H70NO17S	736.4359	2.4	33.8	5	49.49	-4.0	even	ok
	6	C37H72NO5P2S2	736.4322	-2.7	34.5	6	41.84	4.0	even	ok
	7	C34H68N5O6P2S	736.4360	2.5	35.2	7	35.36	5.0	even	ok
	8	C32H69N5NaO6P2S	736.4336	-0.7	35.6	8	79.10	2.0	even	ok
	9	C30H59N13NaO5S	736.4375	4.6	35.8	9	9.16	8.0	even	ok
	10	C38H68NNaO7PS	736.4346	0.7	35.8	10	100.00	6.0	even	ok
	11	C29H63N9NaO9S	736.4362	2.8	36.0	11	30.62	3.0	even	ok
	12	C34H64N7NaO5PS	736.4319	-3.0	36.3	12	26.93	7.0	even	ok
	13	C37H62N5O8S	736.4314	-3.8	36.4	13	20.29	10.0	even	ok
	14	C31H73NNaO10P2S	736.4323	-2.5	36.7	14	33.97	-3.0	even	ok
	15	C30H64N11O4P2S	736.4333	-1.1	37.2	15	65.64	6.0	even	ok
	16	C28H54N19O3S	736.4372	4.2	37.4	16	11.68	12.0	even	ok
	17	C32H59N13O3PS	736.4317	-3.3	37.4	17	20.74	11.0	even	ok
	18	C37H67N3NaO6S2	736.4363	3.0	37.5	18	31.87	6.0	even	ok
	19	C27H58N15O7S	736.4359	2.4	37.8	19	35.97	7.0	even	ok
	20	C33H65N9NaO2P2S	736.4349	1.1	37.9	20	64.80	7.0	even	ok
	21	C29H68N7O8P2S	736.4320	-2.9	38.5	21	26.16	1.0	even	ok
	22	C31H60N15P2S	736.4347	0.7	39.3	22	72.04	11.0	even	ok
	23	C28H67N5NaO13S	736.4348	0.9	39.6	23	65.45	-2.0	even	ok
	24	C37H69N3NaO4P2S	736.4376	4.7	39.9	24	7.24	6.0	even	ok
	25	C29H61N15NaP2S	736.4323	-2.6	40.3	25	30.65	8.0	even	ok
	26	C36H63N7O5PS	736.4344	0.3	40.3	26	80.64	10.0	even	ok
	27	C35H64N9O2P2S	736.4373	4.4	40.6	27	9.50	10.0	even	ok
	28	C27H71NNaO17S	736.4335	-0.9	41.1	28	80.85	-7.0	even	ok
	29	C28H65N11NaO4P2S	736.4309	-4.4	41.2	29	9.25	3.0	even	ok
	30	C26H62N11O11S	736.4345	0.6	41.4	30	71.55	2.0	even	ok
	31	C40H67NO7PS	736.4370	3.9	42.1	31	15.38	9.0	even	ok
	32	C35H60N11NaOPS	736.4333	-1.2	42.5	32	55.76	12.0	even	ok
	33	C28H72N3O12P2S	736.4306	-4.7	42.8	33	6.68	-4.0	even	ok
	34	C27H68N7NaO10PS	736.4378	5.0	43.3	34	5.30	-2.0	even	ok
	35	C38H58N9O4S	736.4327	-1.9	44.3	35	46.66	15.0	even	ok
	36	C40H63N3NaO6S	736.4330	-1.6	44.4	36	55.42	11.0	even	ok
	37	C25H63N13O8PS	736.4375	4.6	45.2	37	6.81	2.0	even	ok
	38	C39H67N3O4PS2	736.4305	-4.9	45.4	38	6.71	9.0	even	ok
	39	C25H66N7O15S	736.4332	-1.3	47.5	39	46.41	-3.0	even	ok
	40	C39H64N5NaO3PS	736.4360	2.5	48.4	40	25.12	11.0	even	ok
	41	C37H59N11OPS	736.4357	2.1	48.5	41	30.42	15.0	even	ok
	42	C24H63N11NaO11S	736.4321	-2.7	49.4	42	21.74	-1.0	even	ok
	43	C24H70N3O19S	736.4319	-3.1	49.4	43	21.94	-8.0	even	ok
	44	C26H72N3NaO14PS	736.4365	3.2	49.5	44	16.24	-7.0	even	ok
	45	C37H55N13NaS	736.4316	-3.4	51.0	45	13.62	17.0	even	ok
	46	C33H62N5O13	736.4339	-0.4	51.1	46	57.51	6.0	even	ok
	47	C36H63N3NaO11	736.4355	1.8	51.2	47	32.12	7.0	even	ok
	48	C24H67N9O12PS	736.4362	2.8	51.3	48	19.28	-3.0	even	ok

Compound Spectrum SmartFormula Report

Meas. m/z	#	Ion Formula	m/z	err [ppm]	mSigma	# mSigma	Score	rdb	e¯ Conf	N-Rule
	49	C32H66NO17	736.4325	-2.2	51.3	49	26.99	1.0	even	ok
	50	C38H62N3O11	736.4379	5.1	52.0	50	3.80	10.0	even	ok
	51	C42H62N3O6S	736.4354	1.7	52.4	51	41.13	14.0	even	ok
	52	C36H68NO10P2	736.4313	-3.9	52.6	52	9.60	5.0	even	ok
	53	C34H68NNaO12P	736.4371	4.1	52.8	53	8.17	2.0	even	ok
	54	C41H59N7NaO2S	736.4343	0.2	53.2	54	69.87	16.0	even	ok
	55	C34H58N9O9	736.4352	1.4	53.3	55	35.67	11.0	even	ok
	56	C40H63N7PS2	736.4319	-3.1	54.4	56	18.42	14.0	even	ok
	57	C32H63N7O10P	736.4369	3.7	55.1	57	9.88	6.0	even	ok
	58	C31H63N5NaO13	736.4315	-3.6	55.2	58	10.26	3.0	even	ok
	59	C32H59N9NaO9	736.4328	-1.8	55.2	59	28.51	8.0	even	ok
	60	C42H68NNaO2PS2	736.4321	-2.7	55.5	60	22.17	10.0	even	ok
	61	C31H67N3O14P	736.4355	1.9	55.7	61	27.27	1.0	even	ok
	62	C22H51N25NaOS	736.4321	-2.7	55.8	62	17.60	10.0	even	ok
	63	C37H59N7NaO7	736.4368	3.6	55.9	63	9.94	12.0	even	ok
	64	C25H56N21NaPS	736.4378	5.0	56.0	64	3.65	9.0	even	ok
	65	C37H64N5O6P2	736.4326	-2.0	56.5	65	24.64	10.0	even	ok
	66	C23H67N7NaO15S	736.4308	-4.5	56.6	66	5.20	-6.0	even	ok
	67	C18H51N29PS	736.4335	-0.9	56.7	67	40.68	9.0	even	ok
	68	C22H55N23O2PS	736.4362	2.8	56.8	68	16.30	8.0	even	ok
	69	C33H55N13NaO5	736.4341	-0.0	57.5	69	52.60	13.0	even	ok
	70	C30H54N15O7	736.4325	-2.2	57.5	70	22.00	12.0	even	ok
	71	C24H50N25OS	736.4345	0.6	57.5	71	44.12	13.0	even	ok
	72	C29H58N11O11	736.4312	-4.0	57.6	72	7.32	7.0	even	ok
	73	C35H54N13O5	736.4365	3.3	57.7	73	11.96	16.0	even	ok
	74	C26H60N17O2P2S	736.4306	-4.8	57.8	74	4.18	7.0	even	ok
	75	C20H56N23NaO2PS	736.4338	-0.5	58.3	75	44.08	5.0	even	ok
	76	C41H64NNaO7P	736.4313	-3.9	58.7	76	7.61	11.0	even	ok
	77	C27H46N25O	736.4312	-4.0	58.8	77	6.95	18.0	even	ok
	78	C24H60N17NaO4PS	736.4365	3.2	58.8	78	12.20	4.0	even	ok
	79	C19H50N27O3S	736.4305	-4.9	58.9	79	3.54	9.0	even	ok
	80	C31H60N11NaO6P	736.4358	2.2	59.1	80	20.34	8.0	even	ok
	81	C39H54N13S	736.4340	-0.1	59.2	81	47.79	20.0	even	ok
	82	C30H64N7NaO10P	736.4344	0.4	59.5	82	43.02	3.0	even	ok
	83	C31H50N19O3	736.4339	-0.4	59.6	83	43.56	17.0	even	ok
	84	C26H55N19NaO3S	736.4348	0.9	59.8	84	35.67	9.0	even	ok
	85	C23H54N21O5S	736.4332	-1.3	59.9	85	31.11	8.0	even	ok
	86	C20H60N21O3P2S	736.4378	5.0	60.1	86	3.06	3.0	even	ok
	87	C39H59N7O5P	736.4310	-4.3	60.2	87	5.52	15.0	even	ok
	88	C28H52N21NaP	736.4344	0.4	60.3	88	42.06	14.0	even	ok
	89	C36H61N9NaO2P2	736.4316	-3.5	60.5	89	9.47	12.0	even	ok
	90	C40H65N3NaO4P2	736.4343	0.2	60.6	90	45.19	11.0	even	ok
	91	C21H55N21NaO5S	736.4308	-4.5	60.6	91	4.50	5.0	even	ok
	92	C32H56N15NaO2P	736.4371	4.1	60.9	92	6.33	13.0	even	ok
	93	C29H55N17O4P	736.4355	1.9	61.5	93	22.65	12.0	even	ok
	94	C29H51N19NaO3	736.4314	-3.6	61.6	94	8.21	14.0	even	ok

Compound Spectrum SmartFormula Report

Meas. m/z	#	Ion Formula	m/z	err [ppm]	mSigma	# mSigma	Score	rdb	e⁻ Conf	N-Rule
	95	C34H51N17NaO	736.4355	1.8	61.7	95	22.98	18.0	even	ok
	96	C43H58N7O2S	736.4367	3.5	61.8	96	11.12	19.0	even	ok
	97	C28H59N13O8P	736.4342	0.0	61.9	97	44.72	7.0	even	ok
	98	C29H68N3NaO14P	736.4331	-1.4	61.9	98	27.61	-2.0	even	ok
	99	C25H59N15NaO7S	736.4335	-0.9	62.0	99	33.67	4.0	even	ok
	100	C38H60N9O2P2	736.4340	-0.2	62.2	100	41.99	15.0	even	ok
	101	C21H59N19O6PS	736.4349	1.0	62.2	101	32.20	3.0	even	ok
	102	C45H63NNaO4S	736.4370	3.9	62.4	102	8.45	15.0	even	ok
	103	C34H56N15P2	736.4313	-3.9	62.5	103	6.87	16.0	even	ok
	104	C29H72NO15P2	736.4372	4.1	63.0	104	5.62	-4.0	even	ok
	105	C30H51N21P	736.4368	3.7	63.1	105	7.64	17.0	even	ok
	106	C25H51N23O2P	736.4328	-1.8	63.1	106	22.28	13.0	even	ok
	107	C43H63NO7P	736.4337	-0.6	63.5	107	34.99	14.0	even	ok
	108	C36H50N17O	736.4379	5.1	63.8	108	2.60	21.0	even	ok
	109	C29H65N9NaO7P2	736.4374	4.5	63.8	109	4.17	3.0	even	ok
	110	C23H64N13NaO8PS	736.4351	1.4	63.9	110	26.05	-1.0	even	ok
	111	C27H63N9O12P	736.4328	-1.8	64.3	111	21.51	2.0	even	ok
	112	C42H64N3O4P2	736.4367	3.4	64.5	112	8.57	14.0	even	ok
	113	C22H58N17O9S	736.4319	-3.1	64.6	113	10.58	3.0	even	ok
	114	C26H57N19NaOP2	736.4361	2.7	64.9	114	13.36	9.0	even	ok
	115	C44H67NO2PS2	736.4345	0.5	65.5	115	41.96	13.0	even	ok
	116	C24H55N19O6P	736.4315	-3.6	65.5	116	7.39	8.0	even	ok
	117	C27H56N17NaO4P	736.4331	-1.4	65.7	117	24.01	9.0	even	ok
	118	C27H60N15O5P2	736.4372	4.1	66.2	118	5.07	7.0	even	ok
	119	C28H69N5NaO11P2	736.4361	2.7	66.4	119	12.57	-2.0	even	ok
	120	C42H60N5NaO3P	736.4326	-2.1	66.7	120	16.93	16.0	even	ok
	121	C26H60N13NaO8P	736.4318	-3.2	67.8	121	8.69	4.0	even	ok
	122	C40H55N11OP	736.4323	-2.5	67.9	122	13.34	20.0	even	ok
	123	C41H61N7NaP2	736.4356	2.0	67.9	123	17.08	16.0	even	ok
	124	C44H63N3NaOS2	736.4305	-5.0	68.2	124	3.06	15.0	even	ok
	125	C26H67N5O16P	736.4315	-3.6	68.4	125	6.73	-3.0	even	ok
	126	C26H64N11O9P2	736.4358	2.3	68.8	126	14.16	2.0	even	ok
	127	C23H52N23NaO2P	736.4304	-5.0	68.8	127	2.24	10.0	even	ok
	128	C23H56N21O3P2	736.4345	0.5	69.8	128	29.56	8.0	even	ok
	129	C19H55N25NaOS2	736.4355	1.9	69.9	129	21.02	5.0	even	ok
	130	C22H55N19NaO8	736.4373	4.3	70.6	130	3.69	5.0	even	ok
	131	C27H73NNaO15P2	736.4348	0.9	70.7	131	24.91	-7.0	even	ok
	132	C26H65N15NaP2S2	736.4356	2.0	70.8	132	18.78	3.0	even	ok
	133	C23H64N17O2P2S2	736.4340	-0.2	70.8	133	39.03	2.0	even	ok
	134	C21H62N13O13S	736.4305	-4.9	71.1	134	2.32	-2.0	even	ok
	135	C47H63NO2PS	736.4312	-4.0	71.2	135	5.58	18.0	even	ok
	136	C25H64N9NaO12P	736.4304	-5.0	71.5	136	2.04	-1.0	even	ok
	137	C25H61N15NaO5P2	736.4348	0.8	72.2	137	23.60	4.0	even	ok
	138	C25H59N19OPS2	736.4324	-2.4	72.2	138	14.55	7.0	even	ok
	139	C45H58N3O6	736.4320	-2.9	72.5	139	8.94	19.0	even	ok
	140	C44H59N5O3P	736.4350	1.2	72.6	140	20.38	19.0	even	ok

Compound Spectrum SmartFormula Report

Meas. m/z	#	Ion Formula	m/z	err [ppm]	mSigma	# mSigma	Score	rdb	e¯ Conf	N-Rule
	141	C20H50N25O6	736.4370	3.9	72.7	141	4.48	9.0	even	ok
	142	C25H69N11NaO4P2S2	736.4343	0.2	72.7	142	35.98	-2.0	even	ok
	143	C25H68N7O13P2	736.4345	0.5	72.9	143	26.17	-3.0	even	ok
	144	C20H58N21O5S2	736.4366	3.3	73.5	144	8.28	3.0	even	ok
	145	C24H63N15O5PS2	736.4310	-4.2	73.9	145	4.36	2.0	even	ok
	146	C22H60N17O7P2	736.4331	-1.4	74.0	146	18.04	3.0	even	ok
	147	C27H64N13NaO3PS2	736.4326	-2.0	74.3	147	16.32	3.0	even	ok
	148	C27H68N11O4P2S2	736.4367	3.5	74.4	148	7.20	1.0	even	ok
	149	C24H67N5NaO18	736.4373	4.3	74.7	149	3.12	-6.0	even	ok
	150	C44H55N7NaO2	736.4309	-4.3	75.2	150	3.08	21.0	even	ok
	151	C22H63N15NaO7S2	736.4369	3.7	75.3	151	6.05	-1.0	even	ok
	152	C26H68N9NaO7PS2	736.4313	-3.9	75.8	152	5.30	-2.0	even	ok
	153	C24H65N11NaO9P2	736.4334	-1.0	76.0	153	19.42	-1.0	even	ok
	154	C42H50N13	736.4307	-4.7	76.0	154	2.24	25.0	even	ok
	155	C27H54N21S2	736.4307	-4.7	76.3	155	2.87	12.0	even	ok
	156	C26H72N7O8P2S2	736.4354	1.7	76.4	156	18.01	-4.0	even	ok
	157	C29H73N5NaO6P2S2	736.4370	3.9	76.5	157	5.16	-3.0	even	ok
	158	C22H62N11O16	736.4371	4.0	76.8	158	3.78	-2.0	even	ok
	159	C21H57N21NaO3P2	736.4321	-2.8	76.9	159	7.86	5.0	even	ok
	160	C19H54N21O10	736.4357	2.1	77.9	160	10.84	4.0	even	ok
	161	C28H67N9O7PS2	736.4337	-0.6	77.9	161	25.91	1.0	even	ok
	162	C29H63N13O3PS2	736.4350	1.2	78.0	162	20.28	6.0	even	ok
	163	C28H77NNaO10P2S2	736.4356	2.0	78.3	163	13.98	-8.0	even	ok
	164	C24H72N3O17P2	736.4331	-1.3	78.4	164	15.25	-8.0	even	ok
	165	C29H59N15NaO2S2	736.4310	-4.3	78.4	165	3.52	8.0	even	ok
	166	C46H62N3OS2	736.4329	-1.7	78.6	166	16.18	18.0	even	ok
	167	C27H71N5O11PS2	736.4324	-2.4	79.5	167	11.06	-4.0	even	ok
	168	C21H59N15NaO12	736.4360	2.5	79.6	168	8.30	0.0	even	ok
	169	C30H72N3NaO9PS2	736.4340	-0.2	80.0	169	27.02	-3.0	even	ok
	170	C31H68N7NaO5PS2	736.4353	1.6	80.2	170	15.82	2.0	even	ok
	171	C18H51N25NaO6	736.4346	0.7	80.6	171	18.05	6.0	even	ok
	172	C23H69N7NaO13P2	736.4321	-2.8	81.2	172	6.69	-6.0	even	ok
	173	C29H66N7O10S2	736.4307	-4.6	81.5	173	2.35	1.0	even	ok
	174	C47H60N3NaOP	736.4366	3.4	81.8	174	4.56	20.0	even	ok
	175	C32H64N11NaOPS2	736.4367	3.4	81.9	175	5.52	7.0	even	ok
	176	C30H62N11O6S2	736.4320	-2.8	81.9	176	7.89	6.0	even	ok
	177	C48H59NNaO4	736.4336	-0.7	82.2	177	16.85	20.0	even	ok
	178	C26H75NO15PS2	736.4310	-4.2	82.5	178	3.12	-9.0	even	ok
	179	C46H54N7O2	736.4334	-1.1	82.6	179	14.37	24.0	even	ok
	180	C16H46N31O4	736.4344	0.3	82.7	180	18.82	10.0	even	ok
	181	C31H71NNaO12S2	736.4310	-4.3	83.6	181	2.88	-3.0	even	ok
	182	C32H71N3O9PS2	736.4364	3.1	83.7	182	6.43	0.0	even	ok
	183	C31H58N15O2S2	736.4334	-1.0	83.9	183	17.36	11.0	even	ok
	184	C32H67N5NaO8S2	736.4323	-2.5	84.1	184	8.90	2.0	even	ok
	185	C33H67N7O5PS2	736.4377	4.9	85.3	185	1.68	5.0	even	ok
	186	C33H63N9NaO4S2	736.4337	-0.6	86.1	186	18.18	7.0	even	ok

Compound Spectrum SmartFormula Report

Meas. m/z	#	Ion Formula	m/z	err [ppm]	mSigma	# mSigma	Score	rdb	e⁻ Conf	N-Rule
	187	C19H47N29NaO2	736.4360	2.5	87.3	187	4.55	11.0	even	ok
	188	C33H70NO12S2	736.4334	-1.0	87.6	188	14.90	0.0	even	ok
	189	C36H69N5NaOP2S2	736.4311	-4.1	88.4	189	2.61	6.0	even	ok
	190	C34H66N5O8S2	736.4347	0.8	89.5	190	14.77	5.0	even	ok
	191	C34H59N13NaS2	736.4350	1.2	89.5	191	12.80	12.0	even	ok
	192	C17H42N35	736.4357	2.1	89.9	192	4.95	15.0	even	ok
	193	C50H58NO4	736.4360	2.6	90.9	193	4.97	23.0	even	ok
	194	C19H52N27OP2	736.4318	-3.2	91.3	194	2.58	9.0	even	ok
	195	C35H62N9O4S2	736.4361	2.6	92.7	195	5.57	10.0	even	ok
	196	C49H55N5Na	736.4350	1.1	92.9	196	8.99	25.0	even	ok
	197	C38H68N5OP2S2	736.4335	-0.9	95.8	197	10.93	9.0	even	ok
	198	C36H58N13S2	736.4374	4.4	97.2	198	1.39	15.0	even	ok
	199	C15H43N35Na	736.4333	-1.1	98.7	199	5.13	12.0	even	ok
	200	C38H63N7NaO2S2	736.4377	4.8	99.4	200	0.93	11.0	even	ok
780.4607	1	C30H71N5NaO14S	780.4610	0.5	17.3	1	100.00	-2.0	even	ok
	2	C31H74NO18S	780.4621	1.8	17.3	2	55.28	-4.0	even	ok
	3	C30H76N3O13P2S	780.4569	-4.9	17.8	3	6.60	-4.0	even	ok
	4	C33H77NNaO11P2S	780.4585	-2.8	19.2	4	30.76	-3.0	even	ok
	5	C28H66N11O12S	780.4608	0.1	19.4	5	86.47	2.0	even	ok
	6	C29H75NNaO18S	780.4597	-1.2	19.7	6	70.28	-7.0	even	ok
	7	C29H72N7NaO11PS	780.4640	4.3	20.0	7	8.21	-2.0	even	ok
	8	C32H70N5O14S	780.4634	3.6	20.2	8	18.17	1.0	even	ok
	9	C27H70N7O16S	780.4594	-1.6	20.3	9	58.90	-3.0	even	ok
	10	C31H67N9NaO10S	780.4624	2.2	20.5	10	42.99	3.0	even	ok
	11	C27H63N15NaO8S	780.4597	-1.2	20.8	11	54.64	4.0	even	ok
	12	C27H67N13O9PS	780.4638	4.0	20.9	12	10.70	2.0	even	ok
	13	C31H72N7O9P2S	780.4582	-3.2	21.4	13	18.64	1.0	even	ok
	14	C28H76N3NaO15PS	780.4627	2.6	21.4	14	33.41	-7.0	even	ok
	15	C25H58N21O6S	780.4594	-1.6	21.8	15	45.26	8.0	even	ok
	16	C29H62N15O8S	780.4621	1.8	22.7	16	39.62	7.0	even	ok
	17	C26H64N17NaO5PS	780.4627	2.6	22.8	17	26.15	4.0	even	ok
	18	C30H69N11NaO5P2S	780.4571	-4.5	22.9	18	6.39	3.0	even	ok
	19	C30H81NNaO11P2S2	780.4618	1.5	23.1	19	57.59	-8.0	even	ok
	20	C26H67N11NaO12S	780.4584	-3.0	23.2	20	20.51	-1.0	even	ok
	21	C28H64N17O3P2S	780.4568	-4.9	23.6	21	4.60	7.0	even	ok
	22	C28H59N19NaO4S	780.4610	0.5	24.3	22	69.11	9.0	even	ok
	23	C24H62N17O10S	780.4581	-3.3	24.4	23	15.71	3.0	even	ok
	24	C26H71N9O13PS	780.4624	2.2	24.8	24	30.50	-3.0	even	ok
	25	C26H54N25O2S	780.4608	0.1	24.9	25	76.95	13.0	even	ok
	26	C27H60N21NaOPS	780.4640	4.3	24.9	26	7.44	9.0	even	ok
	27	C35H76NO11P2S	780.4609	0.3	25.1	27	90.71	0.0	even	ok
	28	C25H68N13NaO9PS	780.4613	0.9	26.0	28	56.92	-1.0	even	ok
	29	C34H70NO18	780.4587	-2.5	26.1	29	25.95	1.0	even	ok
	30	C23H59N21NaO6S	780.4570	-4.7	26.1	30	5.21	5.0	even	ok
	31	C26H74N3O20S	780.4581	-3.3	26.9	31	18.71	-8.0	even	ok
	32	C32H80NO11P2S2	780.4643	4.6	27.1	32	6.92	-5.0	even	ok

Compound Spectrum SmartFormula Report

Meas. m/z	#	Ion Formula	m/z	err [ppm]	mSigma	# mSigma	Score	rdb	e⁻ Conf	N-Rule
	33	C35H72N3NaO10PS	780.4568	-4.9	27.2	33	5.14	2.0	even	ok
	34	C23H63N19O7PS	780.4611	0.5	27.5	34	63.02	3.0	even	ok
	35	C32H68N11O5P2S	780.4595	-1.5	28.2	35	42.07	6.0	even	ok
	36	C32H76N3NaO10PS2	780.4602	-0.6	28.2	36	74.84	-3.0	even	ok
	37	C34H73N5NaO7P2S	780.4598	-1.1	28.4	37	49.12	2.0	even	ok
	38	C33H71N3O15P	780.4617	1.4	28.9	38	43.13	1.0	even	ok
	39	C25H75N5O17PS	780.4611	0.5	29.2	39	75.32	-8.0	even	ok
	40	C24H69N15NaO6P2S	780.4643	4.7	29.4	40	4.77	-1.0	even	ok
	41	C31H65N15NaOP2S	780.4585	-2.8	29.6	41	19.27	8.0	even	ok
	42	C33H67N5NaO14	780.4577	-3.8	30.0	42	9.46	3.0	even	ok
	43	C31H70N7O11S2	780.4569	-4.8	30.0	43	5.39	1.0	even	ok
	44	C25H71N7NaO16S	780.4570	-4.7	30.1	44	4.79	-6.0	even	ok
	45	C30H58N19O4S	780.4634	3.5	30.4	45	11.62	12.0	even	ok
	46	C32H63N13NaO6S	780.4637	3.9	30.6	46	8.85	8.0	even	ok
	47	C23H66N13O14S	780.4567	-5.0	31.3	47	3.39	-2.0	even	ok
	48	C31H72N3NaO15P	780.4593	-1.7	31.3	48	34.26	-2.0	even	ok
	49	C31H76NO16P2	780.4634	3.5	31.7	49	11.81	-4.0	even	ok
	50	C29H55N23NaS	780.4624	2.2	31.8	50	26.68	14.0	even	ok
	51	C33H75NNaO13S2	780.4572	-4.4	31.8	51	7.01	-3.0	even	ok
	52	C31H62N11O12	780.4574	-4.2	31.9	52	6.82	7.0	even	ok
	53	C35H66N5O14	780.4601	-0.8	32.8	53	50.54	6.0	even	ok
	54	C36H72NNaO13P	780.4633	3.4	32.9	54	11.85	2.0	even	ok
	55	C32H68N7NaO11P	780.4607	-0.0	33.0	55	65.39	3.0	even	ok
	56	C24H72N9NaO13PS	780.4600	-0.8	33.2	56	48.44	-6.0	even	ok
	57	C36H72N5O7P2S	780.4622	2.0	33.4	57	35.56	5.0	even	ok
	58	C29H67N9O13P	780.4590	-2.1	33.7	58	26.85	2.0	even	ok
	59	C33H72N7NaO6PS2	780.4615	1.1	33.9	59	53.32	2.0	even	ok
	60	C34H67N7O11P	780.4631	3.1	34.4	60	14.57	6.0	even	ok
	61	C30H63N13O9P	780.4604	-0.4	35.1	61	55.11	7.0	even	ok
	62	C30H73N5NaO12P2	780.4623	2.1	35.5	62	25.19	-2.0	even	ok
	63	C37H71N3O10PS	780.4592	-1.8	35.8	63	35.99	5.0	even	ok
	64	C34H75N3O10PS2	780.4626	2.5	35.8	64	25.51	0.0	even	ok
	65	C36H68N7NaO6PS	780.4582	-3.2	35.9	65	15.96	7.0	even	ok
	66	C28H71N5O17P	780.4577	-3.8	36.0	66	8.41	-3.0	even	ok
	67	C34H63N9NaO10	780.4590	-2.1	36.3	67	24.48	8.0	even	ok
	68	C31H69N9NaO8P2	780.4637	3.8	36.3	68	8.16	3.0	even	ok
	69	C33H64N15OP2S	780.4609	0.3	37.4	69	53.76	11.0	even	ok
	70	C28H64N13NaO9P	780.4580	-3.4	37.6	70	10.37	4.0	even	ok
	71	C34H71N5NaO9S2	780.4585	-2.7	37.7	71	20.84	2.0	even	ok
	72	C32H58N15O8	780.4587	-2.5	37.7	72	19.25	12.0	even	ok
	73	C35H69N9NaO3P2S	780.4612	0.6	37.8	73	46.89	7.0	even	ok
	74	C28H68N11O10P2	780.4620	1.8	37.9	74	28.52	2.0	even	ok
	75	C33H64N11NaO7P	780.4620	1.7	38.1	75	29.02	8.0	even	ok
	76	C29H77NNaO16P2	780.4610	0.4	38.3	76	50.12	-7.0	even	ok
	77	C29H64N15O6P2	780.4634	3.5	38.5	77	10.01	7.0	even	ok
	78	C29H60N17NaO5P	780.4593	-1.7	39.1	78	27.82	9.0	even	ok

Compound Spectrum SmartFormula Report

Meas. m/z	#	Ion Formula	m/z	err [ppm]	mSigma	# mSigma	Score	rdb	e⁻ Conf	N-Rule
	79	C38H72NO11P2	780.4575	-4.0	39.3	79	6.35	5.0	even	ok
	80	C34H63N13O4PS	780.4579	-3.6	39.3	80	9.07	11.0	even	ok
	81	C25H62N15O13	780.4646	5.0	39.5	81	2.72	3.0	even	ok
	82	C27H74NO23	780.4646	5.1	39.5	82	2.68	-8.0	even	ok
	83	C35H74NO13S2	780.4596	-1.4	39.7	83	40.97	0.0	even	ok
	84	C31H59N17O5P	780.4617	1.3	39.8	84	32.87	12.0	even	ok
	85	C26H59N19O7P	780.4577	-3.8	39.9	85	7.50	8.0	even	ok
	86	C33H60N17NaPS	780.4568	-4.9	40.2	86	2.92	13.0	even	ok
	87	C27H72N7O14P2	780.4607	0.0	40.6	87	53.02	-3.0	even	ok
	88	C38H67N3NaO12	780.4617	1.3	40.9	88	32.32	7.0	even	ok
	89	C27H55N23O3P	780.4590	-2.1	41.3	89	21.80	13.0	even	ok
	90	C31H55N19NaO4	780.4577	-3.9	41.4	90	6.95	14.0	even	ok
	91	C34H68N11NaO2PS2	780.4629	2.8	41.6	91	17.70	7.0	even	ok
	92	C36H62N9O10	780.4614	1.0	41.7	92	36.91	11.0	even	ok
	93	C27H65N15NaO6P2	780.4610	0.4	41.8	93	45.75	4.0	even	ok
	94	C26H71N5NaO19	780.4635	3.7	42.1	94	7.74	-6.0	even	ok
	95	C35H63N11O7P	780.4644	4.8	42.3	95	3.16	11.0	even	ok
	96	C28H61N19NaO2P2	780.4623	2.1	42.6	96	20.97	9.0	even	ok
	97	C23H50N29O3	780.4646	5.0	42.9	97	2.51	14.0	even	ok
	98	C35H71N7O6PS2	780.4639	4.2	43.0	98	6.41	5.0	even	ok
	99	C24H59N19NaO9	780.4635	3.7	43.0	99	7.62	5.0	even	ok
	100	C29H50N25O2	780.4574	-4.2	43.1	100	5.02	18.0	even	ok
	101	C39H73N3NaO5P2S	780.4638	4.1	43.4	101	7.03	6.0	even	ok
	102	C26H56N25P2	780.4620	1.7	43.6	102	24.57	13.0	even	ok
	103	C30H56N21NaOP	780.4607	-0.0	43.7	103	48.90	14.0	even	ok
	104	C26H69N11NaO10P2	780.4596	-1.3	43.9	104	29.48	-1.0	even	ok
	105	C25H60N21O4P2	780.4607	0.0	44.1	105	48.21	8.0	even	ok
	106	C24H66N11O17	780.4633	3.3	44.3	106	9.39	-2.0	even	ok
	107	C22H54N25O7	780.4633	3.3	44.6	107	9.40	9.0	even	ok
	108	C35H59N13NaO6	780.4603	-0.4	44.7	108	41.68	13.0	even	ok
	109	C37H76NNaO8PS2	780.4642	4.5	44.7	109	4.54	1.0	even	ok
	110	C38H67N7O6PS	780.4606	-0.1	45.1	110	56.66	10.0	even	ok
	111	C35H67N9NaO5S2	780.4599	-1.0	45.3	111	40.64	7.0	even	ok
	112	C34H60N15NaO3P	780.4633	3.4	45.6	112	8.48	13.0	even	ok
	113	C33H54N19O4	780.4601	-0.8	45.7	113	35.42	17.0	even	ok
	114	C26H76N3O18P2	780.4594	-1.7	46.0	114	23.63	-8.0	even	ok
	115	C40H72NNaO8PS	780.4608	0.2	46.0	115	53.48	6.0	even	ok
	116	C24H64N17O8P2	780.4594	-1.7	46.3	116	23.27	3.0	even	ok
	117	C24H57N25NaP2	780.4596	-1.3	46.6	117	27.09	10.0	even	ok
	118	C32H55N21OP	780.4631	3.1	46.9	118	10.43	17.0	even	ok
	119	C36H70N5O9S2	780.4609	0.4	46.9	119	49.84	5.0	even	ok
	120	C23H63N15NaO13	780.4622	2.0	47.2	120	19.77	0.0	even	ok
	121	C37H68N9O3P2S	780.4636	3.7	47.5	121	6.54	10.0	even	ok
	122	C21H51N29NaO3	780.4622	1.9	47.8	122	19.54	11.0	even	ok
	123	C39H66N5O9S	780.4576	-4.0	47.8	123	6.64	10.0	even	ok
	124	C25H73N7NaO14P2	780.4583	-3.0	48.7	124	9.97	-6.0	even	ok

Compound Spectrum SmartFormula Report

Meas. m/z	#	Ion Formula	m/z	err [ppm]	mSigma	# mSigma	Score	rdb	e¯ Conf	N-Rule
	125	C40H66N3O12	780.4641	4.4	49.0	125	3.64	10.0	even	ok
	126	C32H51N23Na	780.4590	-2.1	49.0	126	16.98	19.0	even	ok
	127	C39H68N5O7P2	780.4588	-2.3	49.0	127	15.25	10.0	even	ok
	128	C25H75NNaO23	780.4622	2.0	49.3	128	18.46	-11.0	even	ok
	129	C21H58N21O11	780.4619	1.6	49.4	129	22.13	4.0	even	ok
	130	C23H61N21NaO4P2	780.4583	-3.1	49.8	130	9.57	5.0	even	ok
	131	C35H59N17PS	780.4592	-1.9	49.8	131	19.22	16.0	even	ok
	132	C19H46N35O	780.4619	1.6	50.0	132	21.88	15.0	even	ok
	133	C36H68N11P2S2	780.4570	-4.7	50.1	133	3.51	10.0	even	ok
	134	C37H64N11NaO2PS	780.4595	-1.5	50.5	134	22.44	12.0	even	ok
	135	C21H56N27O2P2	780.4580	-3.4	50.8	135	7.26	9.0	even	ok
	136	C23H68N13O12P2	780.4580	-3.4	50.9	136	7.32	-2.0	even	ok
	137	C39H63N7NaO8	780.4630	3.0	51.2	137	9.35	12.0	even	ok
	138	C20H55N25NaO7	780.4608	0.2	51.6	138	36.08	6.0	even	ok
	139	C38H73N5NaO2P2S2	780.4573	-4.3	51.6	139	4.53	6.0	even	ok
	140	C37H58N13O6	780.4628	2.7	51.6	140	11.56	16.0	even	ok
	141	C38H65N9NaO3P2	780.4578	-3.7	51.7	141	5.75	12.0	even	ok
	142	C21H61N25NaP2S	780.4630	3.0	52.5	142	9.28	5.0	even	ok
	143	C36H60N15OP2	780.4575	-4.1	52.5	143	4.27	16.0	even	ok
	144	C39H76NO6P2S2	780.4584	-2.9	53.0	144	11.75	4.0	even	ok
	145	C20H55N29OPS	780.4597	-1.2	53.7	145	23.12	9.0	even	ok
	146	C18H50N31O5	780.4606	-0.1	53.7	146	34.92	10.0	even	ok
	147	C22H65N17NaO8P2	780.4569	-4.8	53.8	147	2.27	0.0	even	ok
	148	C36H63N13NaOS2	780.4612	0.7	54.0	148	35.40	12.0	even	ok
	149	C36H55N17NaO2	780.4617	1.3	54.2	149	21.89	18.0	even	ok
	150	C39H63N11O2PS	780.4619	1.6	54.8	150	23.57	15.0	even	ok
	151	C34H50N23	780.4614	0.9	55.0	151	24.94	22.0	even	ok
	152	C37H66N9O5S2	780.4623	2.1	55.3	152	18.16	10.0	even	ok
	153	C41H68N5NaO4PS	780.4622	1.9	55.7	153	19.14	11.0	even	ok
	154	C22H60N23NaO3PS	780.4600	-0.9	55.8	154	25.10	5.0	even	ok
	155	C42H71NO8PS	780.4633	3.3	55.9	155	8.34	9.0	even	ok
	156	C19H56N27NaO4P	780.4638	4.1	55.9	156	3.83	6.0	even	ok
	157	C22H50N31S	780.4581	-3.3	56.1	157	6.51	14.0	even	ok
	158	C22H64N21O4P2S	780.4641	4.3	56.6	158	2.99	3.0	even	ok
	159	C39H71N3NaO7S2	780.4626	2.4	56.9	159	14.10	6.0	even	ok
	160	C21H54N27O4S	780.4567	-5.0	57.2	160	1.59	9.0	even	ok
	161	C39H59N13NaOS	780.4578	-3.6	57.6	161	6.33	17.0	even	ok
	162	C40H62N9O5S	780.4589	-2.3	57.6	162	15.24	15.0	even	ok
	163	C24H59N23O3PS	780.4624	2.2	58.3	163	12.06	8.0	even	ok
	164	C24H55N25NaO2S	780.4583	-3.0	58.5	164	7.65	10.0	even	ok
	165	C42H67N3NaO7S	780.4592	-1.9	58.6	165	17.84	11.0	even	ok
	166	C42H69N3NaO5P2	780.4605	-0.3	59.1	166	28.04	11.0	even	ok
	167	C40H64N9O3P2	780.4602	-0.6	59.4	167	24.44	15.0	even	ok
	168	C43H68NNaO8P	780.4575	-4.1	60.1	168	3.25	11.0	even	ok
	169	C41H63N7O6P	780.4572	-4.5	60.2	169	2.42	15.0	even	ok
	170	C40H72N5O2P2S2	780.4597	-1.2	61.9	170	22.03	9.0	even	ok

Compound Spectrum SmartFormula Report

Meas. m/z	#	Ion Formula	m/z	err [ppm]	mSigma	# mSigma	Score	rdb	e¯ Conf	N-Rule
	171	C40H59N11NaO4	780.4644	4.7	61.9	171	1.79	17.0	even	ok
	172	C38H54N17O2	780.4641	4.4	62.1	172	2.40	21.0	even	ok
	173	C38H62N13OS2	780.4636	3.8	64.3	173	4.47	15.0	even	ok
	174	C17H47N35NaO	780.4595	-1.5	65.4	174	10.31	12.0	even	ok
	175	C42H64N9NaPS	780.4635	3.7	65.7	175	4.67	16.0	even	ok
	176	C41H71N3O5PS2	780.4567	-5.1	65.7	176	1.48	9.0	even	ok
	177	C43H67N5O4PS	780.4646	5.0	65.8	177	1.51	14.0	even	ok
	178	C40H67N7NaO3S2	780.4639	4.1	65.9	178	3.20	11.0	even	ok
	179	C17H51N33O2P	780.4636	3.7	66.9	179	2.59	10.0	even	ok
	180	C41H58N13OS	780.4603	-0.5	67.6	180	23.66	20.0	even	ok
	181	C19H54N31S2	780.4614	1.0	68.1	181	19.53	9.0	even	ok
	182	C44H68N3O5P2	780.4629	2.8	68.3	182	6.00	14.0	even	ok
	183	C43H63N7NaO3S	780.4605	-0.2	68.6	183	25.82	16.0	even	ok
	184	C44H66N3O7S	780.4616	1.2	68.9	184	17.37	14.0	even	ok
	185	C45H67NO8P	780.4599	-1.0	69.8	185	14.50	14.0	even	ok
	186	C43H65N7NaOP2	780.4618	1.5	70.1	186	11.84	16.0	even	ok
	187	C21H59N25NaO2S2	780.4617	1.3	70.1	187	15.54	5.0	even	ok
	188	C23H69N17NaO3P2S2	780.4578	-3.7	70.4	188	3.92	-1.0	even	ok
	189	C42H59N11O2P	780.4585	-2.7	71.3	189	5.65	20.0	even	ok
	190	C44H64N5NaO4P	780.4588	-2.4	71.4	190	6.98	16.0	even	ok
	191	C43H73N3NaP2S2	780.4613	0.9	72.8	191	17.29	10.0	even	ok
	192	C25H68N17O3P2S2	780.4602	-0.6	73.0	192	19.08	2.0	even	ok
	193	C23H58N25O2S2	780.4641	4.4	73.1	193	1.95	8.0	even	ok
	194	C22H62N21O6S2	780.4628	2.7	73.7	194	6.54	3.0	even	ok
	195	C24H72N13O7P2S2	780.4589	-2.3	74.1	195	8.27	-3.0	even	ok
	196	C42H67N7OPS2	780.4581	-3.3	75.1	196	4.13	14.0	even	ok
	197	C27H73N11NaO5P2S2	780.4605	-0.2	75.1	197	19.96	-2.0	even	ok
	198	C25H63N19NaO4S2	780.4644	4.8	75.2	198	1.32	4.0	even	ok
	199	C24H67N15NaO8S2	780.4631	3.1	75.7	199	4.82	-1.0	even	ok
	200	C28H69N15NaOP2S2	780.4618	1.5	75.8	200	11.65	3.0	even	ok
	201	C26H77N7NaO9P2S2	780.4592	-1.9	76.1	201	9.30	-7.0	even	ok
	202	C46H69N3NaP2S	780.4580	-3.5	76.1	202	3.62	15.0	even	ok
	203	C44H72NNaO3PS2	780.4583	-3.0	76.7	203	4.94	10.0	even	ok
	204	C26H67N15O6PS2	780.4572	-4.4	76.7	204	1.72	2.0	even	ok
	205	C27H63N19O2PS2	780.4586	-2.7	77.6	205	5.68	7.0	even	ok
	206	C28H76N7O9P2S2	780.4616	1.2	78.7	206	12.14	-4.0	even	ok
	207	C28H72N9NaO8PS2	780.4575	-4.0	78.8	207	2.11	-2.0	even	ok
	208	C45H62N7O3S	780.4629	2.9	79.0	208	4.72	19.0	even	ok
	209	C29H72N11O5P2S2	780.4629	2.9	79.3	209	4.79	1.0	even	ok
	210	C25H70N11O12S2	780.4641	4.4	79.4	210	1.50	-3.0	even	ok
	211	C45H64N7OP2	780.4642	4.5	79.6	211	1.10	19.0	even	ok
	212	C27H80N3O13P2S2	780.4602	-0.6	79.7	212	14.73	-9.0	even	ok
	213	C47H69NNaO3P2	780.4645	4.9	79.8	213	0.80	15.0	even	ok
	214	C29H68N13NaO4PS2	780.4588	-2.3	79.8	214	6.43	3.0	even	ok
	215	C47H67NNaO5S	780.4632	3.3	80.2	215	3.55	15.0	even	ok
	216	C46H63N5O4P	780.4612	0.7	81.3	216	10.47	19.0	even	ok

Compound Spectrum SmartFormula Report

Meas. m/z	#	Ion Formula	m/z	err [ppm]	mSigma	# mSigma	Score	rdb	e¯ Conf	N-Rule
	217	C27H75N5NaO14S2	780.4644	4.8	81.4	217	1.02	-7.0	even	ok
	218	C30H68N15OP2S2	780.4642	4.6	81.4	218	1.23	6.0	even	ok
	219	C31H77N5NaO7P2S2	780.4632	3.2	81.4	219	3.47	-3.0	even	ok
	220	C29H75N5O12PS2	780.4586	-2.7	82.4	220	4.73	-4.0	even	ok
	221	C30H64N17NaPS2	780.4602	-0.6	82.4	221	12.93	8.0	even	ok
	222	C45H60N9NaP	780.4602	-0.7	82.7	222	10.03	21.0	even	ok
	223	C28H79NO16PS2	780.4572	-4.4	83.0	223	1.35	-9.0	even	ok
	224	C30H71N9O8PS2	780.4599	-1.0	83.3	224	10.89	1.0	even	ok
	225	C47H62N3O7	780.4582	-3.1	83.4	225	2.73	19.0	even	ok
	226	C45H72N3P2S2	780.4637	3.9	83.5	226	1.90	13.0	even	ok
	227	C32H73N9NaO3P2S2	780.4645	4.9	83.6	227	0.82	2.0	even	ok
	228	C44H54N13O	780.4569	-4.9	84.2	228	0.69	25.0	even	ok
	229	C29H58N21OS2	780.4569	-4.8	84.4	229	0.89	12.0	even	ok
	230	C46H59N7NaO3	780.4572	-4.5	84.5	230	0.93	21.0	even	ok
	231	C31H67N13O4PS2	780.4613	0.7	85.6	231	10.76	6.0	even	ok
	232	C48H68N3P2S	780.4604	-0.4	86.6	232	11.81	18.0	even	ok
	233	C31H63N15NaO3S2	780.4572	-4.5	86.6	233	1.10	8.0	even	ok
	234	C46H71NO3PS2	780.4608	0.1	87.5	234	12.50	13.0	even	ok
	235	C32H63N17PS2	780.4626	2.5	89.4	235	4.00	11.0	even	ok
	236	C49H67NO3PS	780.4574	-4.2	89.8	236	1.17	18.0	even	ok
	237	C32H66N11O7S2	780.4583	-3.1	89.9	237	2.66	6.0	even	ok
	238	C46H67N3NaO2S2	780.4567	-5.1	90.2	238	0.53	15.0	even	ok
	239	C48H63N5NaOS	780.4646	5.0	90.4	239	0.59	20.0	even	ok
	240	C47H59N9P	780.4626	2.4	92.9	240	2.81	24.0	even	ok
	241	C49H64N3NaO2P	780.4628	2.8	93.4	241	2.22	20.0	even	ok
	242	C33H62N15O3S2	780.4596	-1.4	93.8	242	5.78	11.0	even	ok
	243	C48H58N7O3	780.4596	-1.4	95.1	243	4.28	24.0	even	ok
	244	C50H63NNaO5	780.4598	-1.1	95.6	244	4.88	20.0	even	ok

Cmpd 2, 3.2 min

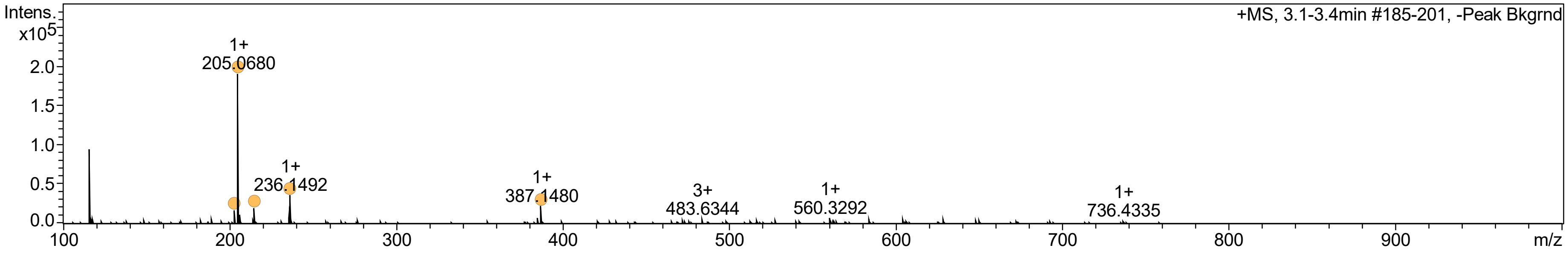

Meas. m/z	#	Ion Formula	m/z	err [ppm]	mSigma	# mSigma	Score	rdb	e¯ Conf	N-Rule
116.0705	1	C5H10NO2	116.0706	0.5	9.4	1	100.00	2.0	even	ok

Compound Spectrum SmartFormula Report

Meas. m/z	#	Ion Formula	m/z	err [ppm]	mSigma	# mSigma	Score	rdb	e¯ Conf	N-Rule
203.0524	1	C4H7N6O4	203.0523	-0.6	5.9	1	100.00	5.0	even	ok
	2	C6H12NaO6	203.0526	0.8	8.3	2	93.76	1.0	even	ok
	3	C6H12N4PS	203.0515	-4.7	30.2	3	39.78	4.0	even	ok
	4	C12H11OS	203.0525	0.3	45.2	4	54.86	8.0	even	ok
205.0680	1	C6H14NaO6	205.0683	1.2	4.4	1	91.14	0.0	even	ok
	2	C4H9N6O4	205.0680	-0.1	4.4	2	100.00	4.0	even	ok
	3	C6H14N4PS	205.0671	-4.3	25.4	3	44.01	3.0	even	ok
	4	C12H13OS	205.0682	0.8	44.5	4	50.92	7.0	even	ok
215.0701	1	C7H12N4O2P	215.0692	-3.8	11.0	1	100.00	5.0	even	ok
	2	C10H13N2NaP	215.0709	3.7	25.1	2	75.00	6.0	even	ok
	3	C6H11N6OS	215.0710	4.2	25.9	3	69.74	5.0	even	ok
	4	C5H15N2O5S	215.0696	-2.0	26.0	4	88.30	0.0	even	ok
	5	C13H11O3	215.0703	1.0	39.9	5	68.61	9.0	even	ok
236.1492	1	C9H19N5NaO	236.1482	-4.5	4.9	1	62.60	3.0	even	ok
	2	C10H22NO5	236.1492	0.0	7.8	2	100.00	1.0	even	ok
	3	C11H26NS2	236.1501	3.7	54.7	3	27.70	0.0	even	ok
387.1480	1	C8H21N12NaO3P	387.1489	2.4	10.6	1	87.35	5.0	even	ok
	2	C6H16N18OP	387.1487	1.7	11.3	2	100.00	9.0	even	ok
	3	C10H23N6O10	387.1470	-2.6	12.9	3	80.05	3.0	even	ok
	4	C12H28NaO12	387.1473	-1.9	14.7	4	90.17	-1.0	even	ok
	5	C11H19N10O6	387.1484	0.9	25.4	5	86.28	8.0	even	ok
	6	C13H24N4NaO8	387.1486	1.6	26.4	6	73.81	4.0	even	ok
	7	C10H16N14NaO2	387.1473	-1.9	26.4	7	69.60	10.0	even	ok
	8	C14H27O12	387.1497	4.4	26.6	8	38.71	2.0	even	ok
	9	C8H11N20	387.1470	-2.6	26.7	9	59.31	14.0	even	ok
	10	C16H30NaO5P2	387.1461	-5.0	36.0	10	25.33	3.0	even	ok
	11	C11H29N6O3P2S	387.1492	3.0	36.3	11	57.72	2.0	even	ok
	12	C13H34NaO5P2S	387.1494	3.7	38.4	12	45.86	-2.0	even	ok
	13	C12H15N14O2	387.1497	4.3	38.8	13	28.70	13.0	even	ok
	14	C14H20N8NaO4	387.1500	5.1	39.5	14	22.99	9.0	even	ok
	15	C12H28N4O6PS	387.1462	-4.8	40.4	15	32.35	2.0	even	ok
	16	C13H24N8O2PS	387.1475	-1.3	46.0	16	62.05	7.0	even	ok
	17	C15H29N2NaO4PS	387.1478	-0.6	47.9	17	66.71	3.0	even	ok
	18	C18H29O5P2	387.1485	1.2	48.6	18	44.17	6.0	even	ok
	19	C17H26N4NaOP2	387.1474	-1.6	49.4	19	39.99	8.0	even	ok
	20	C16H25N6NaPS	387.1491	2.9	55.5	20	33.65	8.0	even	ok
	21	C10H24N10NaOS2	387.1468	-3.1	57.7	21	29.63	4.0	even	ok
	22	C17H24N4NaO3S	387.1461	-4.9	59.5	22	17.61	8.0	even	ok
	23	C11H27N6O5S2	387.1479	-0.3	61.0	23	45.83	2.0	even	ok
	24	C18H27O7S	387.1472	-2.1	61.3	24	32.73	6.0	even	ok
	25	C19H25N4OP2	387.1498	4.6	61.9	25	12.97	11.0	even	ok
	26	C13H32NaO7S2	387.1482	0.4	63.2	26	42.17	-2.0	even	ok
	27	C12H23N10OS2	387.1492	3.1	64.6	27	23.34	7.0	even	ok
	28	C20H24N2O4P	387.1468	-3.1	64.9	28	17.39	11.0	even	ok
	29	C14H28N4NaO3S2	387.1495	3.8	66.7	29	18.13	3.0	even	ok
	30	C19H23N4O3S	387.1485	1.3	69.6	30	28.26	11.0	even	ok

Compound Spectrum SmartFormula Report

Meas. m/z	#	Ion Formula	m/z	err [ppm]	mSigma	# mSigma	Score	rdb	e¯ Conf	N-Rule
	31	C17H34NaP2S2	387.1469	-2.8	70.5	31	20.30	2.0	even	ok
	32	C21H20N6P	387.1482	0.4	78.2	32	18.09	16.0	even	ok
	33	C23H25NaO2P	387.1484	1.1	79.1	33	15.45	12.0	even	ok
	34	C19H33P2S2	387.1493	3.4	79.4	34	12.39	5.0	even	ok
	35	C25H20N2NaO	387.1468	-3.2	95.5	35	4.92	17.0	even	ok

Cmpd 3, 3.4 min

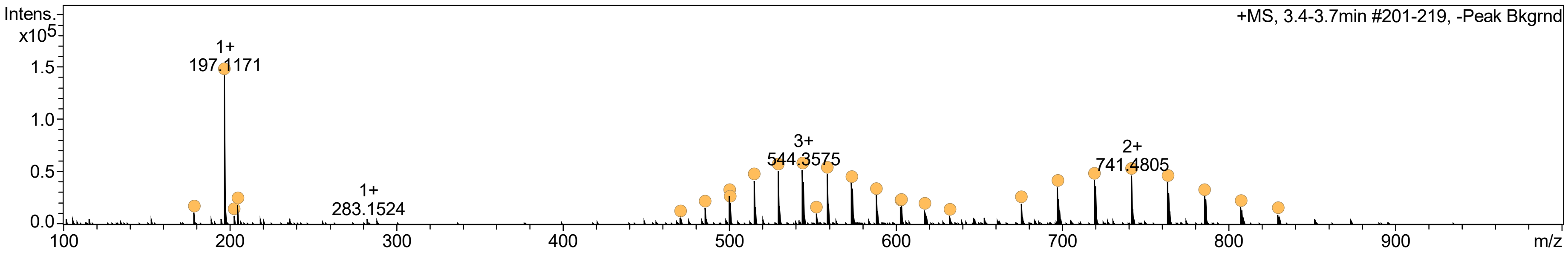

Meas. m/z	#	Ion Formula	m/z	err [ppm]	mSigma	# mSigma	Score	rdb	e¯ Conf	N-Rule
179.1067	1	C11H15O2	179.1067	-0.3	22.1	1	100.00	5.0	even	ok
197.1171	1	C11H17O3	197.1172	0.5	7.3	1	100.00	4.0	even	ok
	2	C8H19N2NaP	197.1178	3.5	9.1	2	73.77	1.0	even	ok
203.0528	1	C4H7N6O4	203.0523	-2.2	6.1	1	91.77	5.0	even	ok
	2	C6H12NaO6	203.0526	-0.9	8.3	2	100.00	1.0	even	ok
	3	C5H3N10	203.0537	4.4	12.3	3	65.63	10.0	even	ok
	4	C12H11OS	203.0525	-1.3	44.6	4	54.82	8.0	even	ok
	5	C7H16NaOS2	203.0535	3.4	49.3	5	38.89	0.0	even	ok
205.0689	1	C6H14NaO6	205.0683	-2.9	11.6	1	100.00	0.0	even	ok
	2	C4H9N6O4	205.0680	-4.3	12.0	2	85.63	4.0	even	ok
	3	C7H10N4NaO2	205.0696	3.6	25.6	3	69.40	5.0	even	ok
	4	C5H5N10	205.0693	2.2	25.8	4	79.35	9.0	even	ok
	5	C7H18NaOS2	205.0691	1.3	49.2	5	61.84	-1.0	even	ok
	6	C12H13OS	205.0682	-3.4	51.0	6	47.85	7.0	even	ok
470.9805	1	C52H152NO33P2S	470.9809	0.7	21.5	1	75.09	-20.0	even	ok
	2	C40H3N38O17P2S2	470.9813	1.6	21.5	2	61.22	61.0	even	ok
	3	C52H148N3NaO32PS	470.9795	-2.1	21.6	3	53.53	-18.0	even	ok
	4	C50H143N9O30PS	470.9794	-2.3	22.0	5	50.31	-14.0	even	ok
	5	C50H24N8NaO35P2S	470.9807	0.4	22.1	6	79.74	45.0	even	ok
	6	C51H23N6NaO38PS	470.9797	-1.7	22.1	7	70.77	45.0	even	ok
	7	C48H15N16NaO32PS	470.9793	-2.7	22.1	8	45.48	51.0	even	ok
	8	C51H20N12NaO31P2S	470.9812	1.3	22.2	9	64.62	50.0	even	ok
	9	C42H8N32NaO19P2S2	470.9814	1.8	22.3	10	68.74	57.0	even	ok
	10	C49H18N12O36PS	470.9796	-1.9	22.3	11	66.96	49.0	even	ok

Compound Spectrum SmartFormula Report

Meas. m/z	#	Ion Formula	m/z	err [ppm]	mSigma	# mSigma	Score	rdb	e⁻ Conf	N-Rule
	11	C49H15N18O29P2S	470.9811	1.1	22.3	13	67.42	54.0	even	ok
	12	C52H26N2O42PS	470.9801	-1.0	22.3	14	84.24	43.0	even	ok
	13	C51H149N5NaO29P2S	470.9805	-0.0	22.5	15	85.12	-18.0	even	ok
	14	C51H27N4O39P2S	470.9811	1.2	22.5	16	80.55	43.0	even	ok
	15	C49H139N13NaO28S	470.9818	2.7	22.5	17	44.16	-12.0	even	ok
	16	C44H132N27NaO16PS2	470.9802	-0.7	22.5	18	88.72	-6.0	even	ok
	17	C50H14N16O32PS	470.9801	-1.0	22.6	19	69.66	54.0	even	ok
	18	C52H23N8O35P2S	470.9815	2.1	22.7	20	63.36	48.0	even	ok
	19	C43H7N30NaO22PS2	470.9804	-0.3	22.7	21	96.10	57.0	even	ok
	20	C48H19N14O33P2S	470.9806	0.2	22.8	22	81.65	49.0	even	ok
	21	C43H11N28O23P2S2	470.9817	2.6	22.8	23	55.28	55.0	even	ok
	22	C46H10N22O30PS	470.9792	-2.9	22.9	24	42.12	55.0	even	ok
	23	C54H28N2NaO37P2S	470.9816	2.3	23.0	25	59.58	44.0	even	ok
	24	C49H11N20NaO28PS	470.9797	-1.7	23.1	26	57.58	56.0	even	ok
	25	C49H146N5O36S	470.9817	2.6	23.1	27	46.02	-19.0	even	ok
	26	C45H135N23O20PS2	470.9805	0.0	23.1	28	100.00	-8.0	even	ok
	27	C47H134N19O26S	470.9817	2.5	23.2	29	45.99	-8.0	even	ok
	28	C43H4N36NaO15P2S2	470.9818	2.8	23.3	31	51.74	62.0	even	ok
	29	C45H16N22NaO25P2S2	470.9818	2.8	23.3	32	51.59	51.0	even	ok
	30	C49H144N11O27P2S	470.9804	-0.2	23.4	33	80.32	-14.0	even	ok
	31	C47H6N26O26PS	470.9796	-1.9	23.4	34	54.37	60.0	even	ok
	32	C45H6N26NaO28S	470.9816	2.2	23.4	35	50.47	57.0	even	ok
	33	C52H19N10NaO34PS	470.9802	-0.8	23.4	36	85.69	50.0	even	ok
	34	C48H12N22NaO25P2S	470.9807	0.4	23.5	37	77.31	56.0	even	ok
	35	C53H148N5O29P2S	470.9813	1.7	23.7	38	57.36	-15.0	even	ok
	36	C53H22N6O38PS	470.9805	-0.0	23.8	39	99.08	48.0	even	ok
	37	C45H131N25NaO19S2	470.9792	-2.8	23.9	40	50.23	-6.0	even	ok
	38	C44H10N26O26PS2	470.9807	0.5	23.9	41	90.70	55.0	even	ok
	39	C47H140N17NaO22PS2	470.9806	0.2	23.9	42	94.55	-12.0	even	ok
	40	C47H16N18NaO29P2S	470.9803	-0.6	24.0	43	73.82	51.0	even	ok
	41	C48H143N9NaO32S	470.9814	1.8	24.0	44	55.33	-17.0	even	ok
	42	C42H124N39O7P2S2	470.9815	2.2	24.1	45	50.36	3.0	even	ok
	43	C44H6N28NaO25S2	470.9794	-2.4	24.2	46	56.18	57.0	even	ok
	44	C46H7N28O23P2S	470.9806	0.2	24.3	47	78.94	60.0	even	ok
	45	C44H129N33NaO9P2S2	470.9816	2.4	24.4	48	47.32	-1.0	even	ok
	46	C39H3N36NaO20PS2	470.9795	-2.2	24.4	49	59.28	58.0	even	ok
	47	C52H145N9NaO25P2S	470.9810	0.9	24.5	50	67.43	-13.0	even	ok
	48	C55H27NaO40PS	470.9806	0.2	24.6	52	94.41	44.0	even	ok
	49	C42H11N26NaO26PS2	470.9799	-1.2	24.6	53	75.18	52.0	even	ok
	50	C40H6N32O24PS2	470.9798	-1.4	24.6	54	71.68	56.0	even	ok
	51	C55H21N2O44	470.9817	2.5	24.7	55	45.32	48.0	even	ok
	52	C41H121N43NaO3P2S2	470.9812	1.4	24.8	56	60.10	5.0	even	ok
	53	C46H15N20NaO28PS2	470.9808	0.7	24.8	57	85.23	51.0	even	ok
	54	C50H27N2NaO42PS	470.9793	-2.7	24.8	58	51.48	40.0	even	ok
	55	C46H3N30NaO22PS	470.9793	-2.7	24.8	59	42.70	62.0	even	ok
	56	C50H140N15O23P2S	470.9809	0.7	24.8	60	69.85	-9.0	even	ok

Compound Spectrum SmartFormula Report

Meas. m/z	#	Ion Formula	m/z	err [ppm]	mSigma	# mSigma	Score	rdb	e⁻ Conf	N-Rule
	57	C53H18N8NaO37S	470.9792	-2.9	24.9	61	48.08	50.0	even	ok
	58	C51H139N13O26PS	470.9799	-1.4	24.9	62	60.09	-9.0	even	ok
	59	C44H3N34NaO18PS2	470.9808	0.6	25.0	63	85.02	62.0	even	ok
	60	C46H134N21O23S2	470.9795	-2.1	25.0	64	60.60	-8.0	even	ok
	61	C39H116N49OP2S2	470.9811	1.2	25.1	65	62.55	9.0	even	ok
	62	C46H131N23NaO22S	470.9814	1.8	25.1	66	54.15	-6.0	even	ok
	63	C53H144N7NaO28PS	470.9800	-1.2	25.1	67	62.71	-13.0	even	ok
	64	C54H147N3O32PS	470.9803	-0.4	25.2	68	88.60	-15.0	even	ok
	65	C48H141N15NaO23P2S	470.9801	-1.0	25.2	69	65.75	-12.0	even	ok
	66	C46H138N15O30S	470.9813	1.6	25.2	70	56.62	-13.0	even	ok
	67	C47H18N12NaO38S	470.9816	2.2	25.2	71	57.98	46.0	even	ok
	68	C45H11N24O27P2S	470.9802	-0.8	25.3	72	68.79	55.0	even	ok
	69	C41H12N28NaO23P2S2	470.9809	0.9	25.3	73	80.46	52.0	even	ok
	70	C48H143N13O26PS2	470.9810	1.0	25.3	74	78.14	-14.0	even	ok
	71	C40H115N47O4PS2	470.9801	-0.9	25.3	75	66.40	9.0	even	ok
	72	C48H22N8O40PS	470.9792	-2.9	25.4	76	47.93	44.0	even	ok
	73	C43H123N37O10PS2	470.9805	0.0	25.5	77	79.04	3.0	even	ok
	74	C43H14N22O30PS2	470.9803	-0.5	25.5	78	86.81	50.0	even	ok
	75	C42H120N41NaO6PS2	470.9802	-0.7	25.6	79	68.95	5.0	even	ok
	76	C39H7N34O21P2S2	470.9808	0.7	25.7	80	83.16	56.0	even	ok
	77	C42H15N24O27P2S2	470.9813	1.6	25.7	81	66.74	50.0	even	ok
	78	C45H9N24O29S2	470.9797	-1.7	25.8	82	66.19	55.0	even	ok
	79	C54H21N4O41S	470.9795	-2.1	25.8	83	58.46	48.0	even	ok
	80	C44H20N18NaO29P2S2	470.9814	1.8	25.8	84	63.38	46.0	even	ok
	81	C38H4N38NaO17P2S2	470.9805	-0.1	25.9	85	77.71	58.0	even	ok
	82	C50H136N17NaO22PS	470.9795	-2.1	26.0	86	48.43	-7.0	even	ok
	83	C45H13N18O36S	470.9815	2.0	26.0	87	50.06	50.0	even	ok
	84	C45H19N16NaO32PS2	470.9804	-0.3	26.0	88	89.37	46.0	even	ok
	85	C45H6N30O22PS2	470.9812	1.4	26.1	89	69.83	60.0	even	ok
	86	C48H139N15NaO25S2	470.9796	-1.9	26.1	90	62.15	-12.0	even	ok
	87	C50H31O43P2S	470.9806	0.2	26.2	91	90.56	38.0	even	ok
	88	C44H13N20O33S2	470.9793	-2.6	26.2	92	50.77	50.0	even	ok
	89	C39H112N51NaPS2	470.9797	-1.7	26.3	93	54.31	11.0	even	ok
	90	C44H126N29O20S	470.9813	1.6	26.4	94	55.26	-2.0	even	ok
	91	C45H4N32NaO19P2S	470.9803	-0.6	26.4	95	69.84	62.0	even	ok
	92	C45H128N31NaO12PS2	470.9806	0.2	26.4	96	74.61	-1.0	even	ok
	93	C48H131N23O20PS	470.9794	-2.3	26.4	97	45.41	-3.0	even	ok
	94	C49H137N19NaO19P2S	470.9805	-0.0	26.5	98	77.55	-7.0	even	ok
	95	C49H28N4NaO39P2S	470.9803	-0.6	26.5	99	83.77	40.0	even	ok
	96	C50H148N7NaO28PS2	470.9811	1.2	26.5	100	72.59	-18.0	even	ok
	97	C46H136N21O21P2S	470.9800	-1.2	26.6	101	60.87	-8.0	even	ok
	98	C50H11N22O25P2S	470.9815	2.1	26.6	102	48.36	59.0	even	ok
	99	C47H18N16O32PS2	470.9812	1.4	26.6	103	68.91	49.0	even	ok
	100	C54H18N6NaO40	470.9813	1.7	26.7	104	53.01	50.0	even	ok
	101	C46H131N27O16PS2	470.9810	1.0	26.9	105	75.45	-3.0	even	ok
	102	C52H16N16NaO27P2S	470.9816	2.3	26.9	106	45.49	55.0	even	ok

Compound Spectrum SmartFormula Report

Meas. m/z	#	Ion Formula	m/z	err [ppm]	mSigma	# mSigma	Score	rdb	e¯ Conf	N-Rule
	103	C45H23N14O33P2S2	470.9817	2.6	26.9	107	50.22	44.0	even	ok
	104	C55H146NO35S	470.9793	-2.5	27.0	108	50.73	-15.0	even	ok
	105	C45H135N19NaO26S	470.9809	0.8	27.0	109	64.86	-11.0	even	ok
	106	C53H22N2NaO44	470.9809	0.8	27.0	110	65.81	45.0	even	ok
	107	C47H14N18NaO31S2	470.9798	-1.5	27.0	111	67.47	51.0	even	ok
	108	C46H18N14NaO35S2	470.9794	-2.4	27.1	112	52.65	46.0	even	ok
	109	C44H10N22NaO32S	470.9811	1.3	27.1	113	58.87	52.0	even	ok
	110	C44H8N28NaO23P2S	470.9798	-1.5	27.3	114	55.17	57.0	even	ok
	111	C43H119N39NaO9S2	470.9792	-2.8	27.3	115	38.55	5.0	even	ok
	112	C47H11N24NaO24PS2	470.9813	1.6	27.4	116	64.62	56.0	even	ok
	113	C47H132N25O17P2S	470.9804	-0.2	27.4	118	73.09	-3.0	even	ok
	114	C47H127N27NaO18S	470.9818	2.7	27.5	119	39.50	-1.0	even	ok
	115	C47H28N8NaO35P2S2	470.9818	2.8	27.5	120	46.81	40.0	even	ok
	116	C49H8N26NaO21P2S	470.9811	1.3	27.5	121	57.31	61.0	even	ok
	117	C44H122N35O13S2	470.9795	-2.1	27.5	122	57.02	3.0	even	ok
	118	C47H23N10O37P2S	470.9802	-0.7	27.6	123	65.27	44.0	even	ok
	119	C46H22N12O36PS2	470.9807	0.5	27.7	124	82.99	44.0	even	ok
	120	C47H3N32O19P2S	470.9811	1.1	27.8	125	59.64	65.0	even	ok
	121	C54H23N4O39P2	470.9799	-1.2	27.8	126	58.28	48.0	even	ok
	122	C49H142N11O29S2	470.9800	-1.1	27.9	127	71.67	-14.0	even	ok
	123	C49H23N10NaO34PS2	470.9813	1.6	27.9	128	63.66	45.0	even	ok
	124	C52H13N12O38	470.9812	1.5	28.0	129	54.11	54.0	even	ok
	125	C48H136N21NaO18PS2	470.9811	1.2	28.0	130	70.31	-7.0	even	ok
	126	C51H10N20O28PS	470.9805	-0.0	28.1	131	74.58	59.0	even	ok
	127	C45H122N33O16S	470.9817	2.5	28.2	133	41.09	3.0	even	ok
	128	C50H7N24NaO24PS	470.9802	-0.8	28.4	134	63.59	61.0	even	ok
	129	C46H127N29NaO15S2	470.9796	-1.9	28.5	135	58.69	-1.0	even	ok
	130	C51H151N3O32PS2	470.9814	2.0	28.5	136	57.47	-20.0	even	ok
	131	C42H5N28O30S	470.9810	1.1	28.5	137	59.60	56.0	even	ok
	132	C48H27N6NaO38PS2	470.9808	0.7	28.6	138	77.97	40.0	even	ok
	133	C51H17N8O42	470.9808	0.6	28.6	139	66.08	49.0	even	ok
	134	C46H20N14NaO33P2S	470.9798	-1.5	28.7	140	53.47	46.0	even	ok
	135	C54H18N10O34PS	470.9810	0.9	28.8	141	73.24	53.0	even	ok
	136	C43H123N33NaO16S	470.9809	0.8	28.8	142	62.17	0.0	even	ok
	137	C58H23NaO40P	470.9795	-2.2	28.9	143	44.40	49.0	even	ok
	138	C53H15N14NaO30PS	470.9806	0.2	28.9	144	71.20	55.0	even	ok
	139	C46H5N28O25S2	470.9802	-0.7	28.9	145	76.50	60.0	even	ok
	140	C42H3N34O21P2S	470.9797	-1.7	28.9	146	50.51	61.0	even	ok
	141	C45H133N25NaO17P2S	470.9796	-1.9	29.1	147	47.68	-6.0	even	ok
	142	C47H21N10O39S2	470.9797	-1.7	29.1	148	61.27	44.0	even	ok
	143	C48H14N20O28PS2	470.9816	2.4	29.1	149	50.79	54.0	even	ok
	144	C48H17N14O35S2	470.9802	-0.7	29.2	150	76.16	49.0	even	ok
	145	C57H24N2NaO37P2	470.9805	-0.1	29.2	151	71.82	49.0	even	ok
	146	C49H139N17O22PS2	470.9814	1.9	29.3	152	56.56	-9.0	even	ok
	147	C56H18N6O38P	470.9794	-2.4	29.3	153	41.63	53.0	even	ok
	148	C53H27O43P2	470.9795	-2.2	29.4	154	44.16	43.0	even	ok

Compound Spectrum SmartFormula Report

Meas. m/z	#	Ion Formula	m/z	err [ppm]	mSigma	# mSigma	Score	rdb	e⁻ Conf	N-Rule
	149	C51H147N5NaO31S2	470.9801	-0.9	29.4	155	72.29	-18.0	even	ok
	150	C38H6N32NaO26S2	470.9818	2.7	29.4	156	45.89	53.0	even	ok
	151	C47H130N25O19S2	470.9800	-1.1	29.6	158	68.80	-3.0	even	ok
	152	C41H15N22NaO30PS2	470.9795	-2.2	29.7	159	52.52	47.0	even	ok
	153	C52H9N18O31S	470.9795	-2.1	29.7	160	53.16	59.0	even	ok
	154	C46H129N29NaO13P2S	470.9801	-1.0	29.8	161	58.97	-1.0	even	ok
	155	C56H23N4NaO36PS	470.9811	1.1	29.8	162	68.34	49.0	even	ok
	156	C54H144N9O25P2S	470.9818	2.6	29.9	163	38.43	-10.0	even	ok
	157	C39H10N28O28PS2	470.9794	-2.4	30.0	164	49.32	51.0	even	ok
	158	C55H19N8O35P2	470.9804	-0.3	30.0	166	67.73	53.0	even	ok
	159	C44H15N20O31P2S	470.9797	-1.7	30.1	167	49.18	50.0	even	ok
	160	C51H6N22NaO27S	470.9792	-2.9	30.2	168	35.29	61.0	even	ok
	161	C52H23N4NaO41P	470.9819	2.9	30.2	169	35.31	45.0	even	ok
	162	C50H26N6O38PS2	470.9816	2.4	30.2	170	49.35	43.0	even	ok
	163	C51H136N19O19P2S	470.9813	1.7	30.3	171	49.30	-4.0	even	ok
	164	C49H26N4NaO41S2	470.9798	-1.5	30.3	172	62.47	40.0	even	ok
	165	C55H143N3NaO34	470.9811	1.3	30.3	173	53.78	-13.0	even	ok
	166	C53H141N13NaO21P2S	470.9814	1.9	30.4	175	46.72	-8.0	even	ok
	167	C53H20N8NaO35P2	470.9796	-2.0	30.4	176	45.35	50.0	even	ok
	168	C42H18N18O34PS2	470.9798	-1.4	30.4	177	62.58	45.0	even	ok
	169	C48H10N22NaO27S2	470.9803	-0.5	30.4	178	76.89	56.0	even	ok
	170	C55H14N10NaO36	470.9818	2.7	30.5	179	37.50	55.0	even	ok
	171	C41H118N39O14S	470.9808	0.6	30.5	180	62.36	4.0	even	ok
	172	C44H119N37NaO12S	470.9814	1.8	30.5	181	47.74	5.0	even	ok
	173	C44H23N12NaO36PS2	470.9799	-1.2	30.5	182	65.47	41.0	even	ok
	174	C51H10N16NaO34	470.9809	0.8	30.5	183	60.51	56.0	even	ok
	175	C56H149N3NaO27P2S	470.9819	2.8	30.6	184	35.68	-14.0	even	ok
	176	C54H14N12NaO33S	470.9796	-1.9	30.6	185	54.79	55.0	even	ok
	177	C47H25N4O46S	470.9815	2.0	30.6	186	53.69	39.0	even	ok
	178	C52H142N5O36	470.9806	0.2	30.7	187	68.19	-14.0	even	ok
	179	C50H19N14NaO30PS2	470.9817	2.6	30.7	188	46.23	50.0	even	ok
	180	C51H144N11NaO24PS2	470.9815	2.1	30.7	189	51.81	-13.0	even	ok
	181	C49H30N2O42PS2	470.9812	1.4	30.7	190	62.35	38.0	even	ok
	182	C50H22N8NaO37S2	470.9803	-0.5	30.8	191	76.42	45.0	even	ok
	183	C43H128N31O15P2S	470.9795	-2.1	30.8	192	43.40	-2.0	even	ok
	184	C57H26O40PS	470.9814	1.9	30.9	193	55.38	47.0	even	ok
	185	C49H135N19NaO21S2	470.9801	-0.9	30.9	194	69.60	-7.0	even	ok
	186	C46H22N8NaO42S	470.9811	1.3	30.9	195	64.35	41.0	even	ok
	187	C55H148NO33P2	470.9797	-1.6	31.1	196	48.76	-15.0	even	ok
	188	C44H124N35O11P2S	470.9800	-1.2	31.1	197	54.59	3.0	even	ok
	189	C53H9N16O34	470.9817	2.5	31.2	198	39.02	59.0	even	ok
	190	C50H14N12NaO38	470.9804	-0.2	31.2	199	67.37	51.0	even	ok
	191	C40H16N24NaO27P2S2	470.9805	-0.1	31.2	200	82.43	47.0	even	ok
	192	C43H24N14NaO33P2S2	470.9809	0.9	31.2	201	69.74	41.0	even	ok
	193	C51H23N10O32P2S2	470.9793	-2.5	31.3	202	46.05	48.0	even	ok
	194	C55H17N8O37S	470.9800	-1.2	31.4	203	64.70	53.0	even	ok

Compound Spectrum SmartFormula Report

Meas. m/z	#	Ion Formula	m/z	err [ppm]	mSigma	# mSigma	Score	rdb	e¯ Conf	N-Rule
	195	C41H19N20O31P2S2	470.9808	0.7	31.5	204	72.35	45.0	even	ok
	196	C52H135N17O22PS	470.9803	-0.4	31.5	207	63.37	-4.0	even	ok
	197	C50H133N23NaO15P2S	470.9810	0.9	31.6	208	57.10	-2.0	even	ok
	198	C53H138N9O32	470.9810	1.1	31.6	209	54.63	-9.0	even	ok
	199	C42H114N43O10S	470.9813	1.6	31.7	210	48.73	9.0	even	ok
	200	C52H150NO35S2	470.9804	-0.2	31.7	211	80.03	-20.0	even	ok
	201	C38H11N30O25P2S2	470.9804	-0.3	31.8	212	78.08	51.0	even	ok
	202	C52H31NaO40PS2	470.9817	2.6	31.8	213	44.88	39.0	even	ok
	203	C43H12N24NaO27P2S	470.9794	-2.5	31.9	214	38.49	52.0	even	ok
	204	C45H26N8O40PS2	470.9803	-0.5	31.9	215	74.73	39.0	even	ok
	205	C48H128N29O13P2S	470.9809	0.7	31.9	216	59.04	2.0	even	ok
	206	C57H148NNaO30PS	470.9809	0.7	32.0	217	70.92	-14.0	even	ok
	207	C50H18N10O39P	470.9818	2.7	32.0	218	35.80	49.0	even	ok
	208	C54H140N11NaO24PS	470.9804	-0.2	32.1	219	65.04	-8.0	even	ok
	209	C51H15N14O33P2	470.9795	-2.2	32.1	220	41.28	54.0	even	ok
	210	C49H5N22O32	470.9808	0.6	32.1	221	60.73	60.0	even	ok
	211	C55H143N7O28PS	470.9808	0.5	32.1	222	61.40	-10.0	even	ok
	212	C44H27N10O37P2S2	470.9813	1.6	32.2	223	57.08	39.0	even	ok
	213	C41H111N51PS2	470.9805	0.0	32.2	224	67.52	14.0	even	ok
	214	C53H148NNaO35P	470.9817	2.5	32.2	225	37.92	-18.0	even	ok
	215	C49H127N27O16PS	470.9799	-1.4	32.2	226	50.44	2.0	even	ok
	216	C51H132N21NaO18PS	470.9800	-1.2	32.2	227	52.81	-2.0	even	ok
	217	C46H25N6O43S2	470.9793	-2.6	32.3	228	43.98	39.0	even	ok
	218	C46H32N4NaO39P2S2	470.9814	1.8	32.3	229	54.09	35.0	even	ok
	219	C47H31N2NaO42PS2	470.9804	-0.3	32.3	230	76.87	35.0	even	ok
	220	C54H16N12NaO31P2	470.9800	-1.0	32.4	231	54.39	55.0	even	ok
	221	C49H13N18O31S2	470.9806	0.2	32.5	232	77.20	54.0	even	ok
	222	C50H138N15O25S2	470.9804	-0.2	32.5	233	78.21	-9.0	even	ok
	223	C54H153NNaO28P2S2	470.9792	-2.7	32.7	234	41.84	-19.0	even	ok
	224	C57H22N2NaO39S	470.9801	-1.0	32.7	235	65.55	49.0	even	ok
	225	C50H21N4O46	470.9803	-0.4	32.7	236	62.38	44.0	even	ok
	226	C48H32NaO43P2S	470.9798	-1.5	32.7	237	58.20	35.0	even	ok
	227	C43H116N45NaO2PS2	470.9806	0.2	32.7	238	64.05	10.0	even	ok
	228	C50H29O45S2	470.9802	-0.7	32.8	239	69.78	38.0	even	ok
	229	C44H119N41O6PS2	470.9810	1.0	32.8	240	54.52	8.0	even	ok
	230	C48H7N28NaO20PS2	470.9817	2.6	32.9	241	43.88	61.0	even	ok
	231	C44H17N14O40S	470.9810	1.1	32.9	242	53.44	45.0	even	ok
	232	C53H28N4NaO34P2S2	470.9794	-2.3	33.0	243	46.64	44.0	even	ok
	233	C48H9N18O36	470.9803	-0.4	33.0	244	61.85	55.0	even	ok
	234	C48H30NaO45S2	470.9794	-2.4	33.0	245	45.63	35.0	even	ok
	235	C49H11N24O22P2S2	470.9793	-2.5	33.0	246	43.99	59.0	even	ok
	236	C37H8N34NaO21P2S2	470.9800	-1.0	33.1	247	53.66	53.0	even	ok
	237	C51H146NO40	470.9802	-0.8	33.3	248	56.48	-19.0	even	ok
	238	C51H25N4O41S2	470.9806	0.3	33.3	249	75.54	43.0	even	ok
	239	C47H127N31O12PS2	470.9814	1.9	33.3	250	51.29	2.0	even	ok
	240	C50H11N18NaO31P	470.9819	2.9	33.3	251	32.76	56.0	even	ok

Compound Spectrum SmartFormula Report

Meas. m/z	#	Ion Formula	m/z	err [ppm]	mSigma	# mSigma	Score	rdb	e¯ Conf	N-Rule
	241	C40H115N43NaO10S	470.9805	-0.1	33.4	252	64.52	6.0	even	ok
	242	C52H6N20NaO30	470.9813	1.7	33.4	253	45.12	61.0	even	ok
	243	C53H134N15O25S	470.9793	-2.6	33.6	254	35.91	-4.0	even	ok
	244	C56H142N5O31S	470.9798	-1.6	33.6	255	55.57	-10.0	even	ok
	245	C51H139N9NaO32	470.9802	-0.6	33.6	256	58.36	-12.0	even	ok
	246	C41H7N30O25P2S	470.9793	-2.7	33.6	257	34.82	56.0	even	ok
	247	C52H11N18O29P2	470.9799	-1.2	33.7	258	50.39	59.0	even	ok
	248	C48H124N31NaO12PS	470.9795	-2.1	33.7	259	40.11	4.0	even	ok
	249	C42H125N35NaO11P2S	470.9792	-2.9	33.7	260	32.57	0.0	even	ok
	250	C57H143N3O32P	470.9792	-2.8	33.7	261	33.17	-10.0	even	ok
	251	C40H119N41O11PS	470.9818	2.8	33.7	262	33.71	4.0	even	ok
	252	C59H23N2O37P2	470.9813	1.6	33.8	263	45.94	52.0	even	ok
	253	C47H35O43P2S2	470.9817	2.6	33.8	264	42.41	33.0	even	ok
	254	C43H14N18NaO36S	470.9807	0.3	33.9	265	61.31	47.0	even	ok
	255	C43H121N39NaO7P2S	470.9796	-1.9	33.9	266	42.23	5.0	even	ok
	256	C46H124N35NaO8PS2	470.9811	1.2	33.9	267	50.73	4.0	even	ok
	257	C54H145N5NaO29P2	470.9794	-2.4	33.9	268	37.17	-13.0	even	ok
	258	C47H125N33NaO9P2S	470.9805	-0.0	33.9	269	64.63	4.0	even	ok
	259	C51H143N7O33P	470.9816	2.3	34.0	270	38.23	-14.0	even	ok
	260	C40H3N34NaO23PS	470.9817	2.4	34.1	271	36.81	58.0	even	ok
	261	C46H119N37O10PS	470.9794	-2.3	34.1	272	37.56	8.0	even	ok
	262	C52H143N9NaO27S2	470.9805	0.0	34.2	273	77.04	-13.0	even	ok
	263	C35H3N40O19P2S2	470.9799	-1.2	34.2	274	49.89	57.0	even	ok
	264	C51H18N12NaO33S2	470.9807	0.4	34.2	275	70.98	50.0	even	ok
	265	C42H110N49O3S2	470.9795	-2.1	34.3	276	40.16	14.0	even	ok
	266	C52H135N13NaO28	470.9807	0.4	34.4	277	59.92	-7.0	even	ok
	267	C41H111N47NaO6S	470.9809	0.8	34.4	278	54.20	11.0	even	ok
	268	C56H144N5O29P2	470.9802	-0.7	34.4	279	55.77	-10.0	even	ok
	269	C55H139N9NaO27S	470.9794	-2.4	34.5	280	37.10	-8.0	even	ok
	270	C50H4N30NaO17P2S	470.9816	2.3	34.5	281	37.84	66.0	even	ok
	271	C49H132N25NaO14PS2	470.9815	2.1	34.5	283	47.19	-2.0	even	ok
	272	C51H16N18NaO24P2S2	470.9794	-2.3	34.6	284	44.69	55.0	even	ok
	273	C45H120N39O7P2S	470.9804	-0.2	34.7	285	60.91	8.0	even	ok
	274	C51H27NaO45P	470.9814	1.9	34.8	286	41.10	40.0	even	ok
	275	C49H15N14NaO35P	470.9814	1.9	34.8	288	41.14	51.0	even	ok
	276	C56H139N7NaO30	470.9816	2.3	35.0	289	37.59	-8.0	even	ok
	277	C46H27N6O41P2S	470.9797	-1.7	35.0	290	43.57	39.0	even	ok
	278	C49H18N8NaO42	470.9800	-1.1	35.0	291	50.01	46.0	even	ok
	279	C60H22O40P	470.9803	-0.5	35.1	292	57.18	52.0	even	ok
	280	C48H6N24O29P	470.9818	2.7	35.1	295	33.18	60.0	even	ok
	281	C58H20N6NaO33P2	470.9809	0.9	35.1	296	52.93	54.0	even	ok
	282	C38H110N49O8S	470.9804	-0.3	35.2	297	59.19	10.0	even	ok
	283	C44H115N43NaO5S2	470.9796	-1.9	35.2	298	41.30	10.0	even	ok
	284	C41H9N24O34S	470.9806	0.1	35.3	299	61.43	51.0	even	ok
	285	C45H115N41NaO8S	470.9818	2.7	35.4	300	32.53	10.0	even	ok
	286	C47H123N33NaO11S2	470.9801	-0.9	35.4	301	62.01	4.0	even	ok

Compound Spectrum SmartFormula Report

Meas. m/z	#	Ion Formula	m/z	err [ppm]	mSigma	# mSigma	Score	rdb	e⁻ Conf	N-Rule
	287	C54H31O38P2S2	470.9798	-1.6	35.5	302	53.39	42.0	even	ok
	288	C49H134N15O30	470.9802	-0.8	35.5	303	53.32	-8.0	even	ok
	289	C56H15N12O31P2	470.9808	0.7	35.5	304	54.74	58.0	even	ok
	290	C41H116N45O5P2S	470.9795	-2.1	35.5	305	38.45	9.0	even	ok
	291	C52H140N11O27P2	470.9793	-2.6	35.6	306	33.68	-9.0	even	ok
	292	C50H135N21O18PS2	470.9819	2.9	35.7	307	36.91	-4.0	even	ok
	293	C54H134N13O28	470.9815	2.1	35.7	308	38.89	-4.0	even	ok
	294	C45H118N39O9S2	470.9800	-1.1	35.8	309	48.93	8.0	even	ok
	295	C52H141N15NaO18P2S2	470.9792	-2.7	35.8	310	38.50	-8.0	even	ok
	296	C57H14N10O34P	470.9798	-1.5	35.9	311	45.21	58.0	even	ok
	297	C59H19N4NaO36P	470.9799	-1.3	35.9	312	47.36	54.0	even	ok
	298	C50H130N19O26	470.9806	0.2	35.9	313	59.88	-3.0	even	ok
	299	C55H14N14O30PS	470.9814	1.9	35.9	314	48.82	58.0	even	ok
	300	C45H24N10NaO37P2S	470.9794	-2.5	35.9	315	34.80	41.0	even	ok
	301	C39H106N53O4S	470.9808	0.6	35.9	316	54.41	15.0	even	ok
	302	C47H6N22NaO32	470.9800	-1.1	36.0	317	48.76	57.0	even	ok
	303	C50H143N5NaO36	470.9798	-1.5	36.0	318	44.26	-17.0	even	ok
	304	C43H110N47O6S	470.9817	2.5	36.1	319	33.82	14.0	even	ok
	305	C52H6N24O24PS	470.9810	0.9	36.2	320	50.82	64.0	even	ok
	306	C53H146N5O31S2	470.9809	0.8	36.3	321	62.66	-15.0	even	ok
	307	C51H3N28NaO20PS	470.9806	0.2	36.3	322	59.30	66.0	even	ok
	308	C48H126N29O15S2	470.9804	-0.2	36.4	323	70.76	2.0	even	ok
	309	C49H6N26NaO23S2	470.9807	0.4	36.4	324	67.22	61.0	even	ok
	310	C40H18N18NaO36S2	470.9818	2.7	36.4	325	38.41	42.0	even	ok
	311	C51H8N22NaO25P2	470.9796	-2.0	36.5	326	38.81	61.0	even	ok
	312	C52H19N14O28P2S2	470.9798	-1.6	36.5	327	51.87	53.0	even	ok
	313	C37H10N28NaO30S2	470.9813	1.7	36.6	328	49.65	48.0	even	ok
	314	C52H21N8O37S2	470.9811	1.2	36.6	329	56.57	48.0	even	ok
	315	C49H22N6O43P	470.9813	1.8	36.6	330	41.19	44.0	even	ok
	316	C40H6N28NaO30S	470.9802	-0.6	36.8	331	53.08	53.0	even	ok
	317	C39H116N45NaO7PS	470.9815	2.0	36.8	332	38.45	6.0	even	ok
	318	C47H10N20O33P	470.9813	1.7	36.8	333	41.09	55.0	even	ok
	319	C38H13N24O34S2	470.9817	2.5	36.8	334	40.25	46.0	even	ok
	320	C51H136N15NaO25P	470.9817	2.5	37.0	335	33.62	-7.0	even	ok
	321	C55H141N9NaO25P2	470.9798	-1.5	37.0	336	43.95	-8.0	even	ok
	322	C47H13N14O40	470.9799	-1.3	37.0	337	45.39	50.0	even	ok
	323	C57H19N8NaO32PS	470.9815	2.1	37.1	338	44.98	54.0	even	ok
	324	C43H27N8NaO40PS2	470.9795	-2.2	37.1	339	43.62	36.0	even	ok
	325	C50H140N11NaO29P	470.9812	1.5	37.1	340	42.99	-12.0	even	ok
	326	C54H11N18NaO26PS	470.9810	1.1	37.1	341	47.37	60.0	even	ok
	327	C50H147N3O37P	470.9812	1.3	37.2	342	44.86	-19.0	even	ok
	328	C44H117N43NaO3P2S	470.9801	-1.0	37.2	343	48.70	10.0	even	ok
	329	C35H5N34O28S2	470.9812	1.5	37.3	344	51.19	52.0	even	ok
	330	C41H22N14O38PS2	470.9794	-2.4	37.3	345	41.11	40.0	even	ok
	331	C42H15N20NaO33PS	470.9817	2.4	37.3	346	33.76	47.0	even	ok
	332	C53H144N11O22P2S2	470.9796	-2.0	37.3	347	45.70	-10.0	even	ok

Compound Spectrum SmartFormula Report

Meas. m/z	#	Ion Formula	m/z	err [ppm]	mSigma	# mSigma	Score	rdb	e⁻ Conf	N-Rule
	333	C43H19N16O35P2S	470.9793	-2.6	37.3	348	31.67	45.0	even	ok
	334	C56H11N14NaO30P	470.9795	-2.2	37.4	349	35.78	60.0	even	ok
	335	C55H12N16NaO27P2	470.9805	-0.1	37.5	350	58.18	60.0	even	ok
	336	C54H6N20O28P	470.9794	-2.4	37.8	351	33.51	64.0	even	ok
	337	C50H131N23NaO17S2	470.9805	0.0	37.8	352	70.17	-2.0	even	ok
	338	C46H29O50S	470.9810	1.1	38.0	354	56.14	34.0	even	ok
	339	C49H3N28O23P2	470.9795	-2.2	38.0	355	35.36	65.0	even	ok
	340	C58H22N4O36PS	470.9819	2.8	38.0	356	35.43	52.0	even	ok
	341	C48H138N11O34	470.9797	-1.7	38.0	357	39.92	-13.0	even	ok
	342	C52H132N23O15P2S	470.9818	2.6	38.1	358	31.27	1.0	even	ok
	343	C55H10N16NaO29S	470.9801	-1.0	38.2	359	56.85	60.0	even	ok
	344	C53H131N17NaO24	470.9811	1.3	38.2	360	44.11	-2.0	even	ok
	345	C53H136N15O23P2	470.9797	-1.7	38.2	361	40.53	-4.0	even	ok
	346	C44H30N4O44PS2	470.9798	-1.4	38.2	362	51.41	34.0	even	ok
	347	C53H7N22O25P2	470.9804	-0.3	38.2	363	54.86	64.0	even	ok
	348	C50H9N22O27S2	470.9811	1.2	38.2	364	54.33	59.0	even	ok
	349	C45H26N4NaO46S	470.9807	0.3	38.2	365	65.51	36.0	even	ok
	350	C47H3N28NaO25P	470.9814	1.9	38.3	366	37.63	62.0	even	ok
	351	C54H24N8NaO30P2S2	470.9799	-1.4	38.3	367	51.89	49.0	even	ok
	352	C37H107N53NaO4S	470.9800	-1.1	38.4	368	46.35	12.0	even	ok
	353	C42H28N10NaO37P2S2	470.9805	-0.1	38.4	369	68.54	36.0	even	ok
	354	C42H112N49OP2S	470.9800	-1.2	38.4	370	45.12	14.0	even	ok
	355	C42H107N51NaO2S	470.9814	1.8	38.5	371	38.95	16.0	even	ok
	356	C54H137N17NaO17P2S	470.9819	2.8	38.5	373	29.16	-3.0	even	ok
	357	C54H26N2NaO39S2	470.9812	1.4	38.6	374	51.30	44.0	even	ok
	358	C39H20N20NaO31P2S2	470.9800	-1.0	38.6	375	55.93	42.0	even	ok
	359	C40H113N49NaOP2S	470.9792	-2.9	38.6	376	28.54	11.0	even	ok
	360	C48H131N19NaO26	470.9798	-1.5	38.6	377	41.19	-6.0	even	ok
	361	C38H107N55OPS	470.9818	2.8	38.6	378	29.71	15.0	even	ok
	362	C49H131N21O23P	470.9816	2.3	38.7	380	33.90	-3.0	even	ok
	363	C37H111N51O5PS	470.9814	1.8	38.8	381	38.38	10.0	even	ok
	364	C40H23N16O35P2S2	470.9804	-0.3	38.8	382	65.19	40.0	even	ok
	365	C56H13N12O33S	470.9804	-0.2	38.9	383	65.30	58.0	even	ok
	366	C40H10N26O31PS	470.9816	2.2	38.9	384	34.17	51.0	even	ok
	367	C49H4N32NaO14P2S2	470.9794	-2.3	38.9	385	39.80	66.0	even	ok
	368	C49H127N23NaO22	470.9802	-0.6	39.0	386	50.57	-1.0	even	ok
	369	C55H149N5NaO24P2S2	470.9797	-1.8	39.0	387	46.00	-14.0	even	ok
	370	C38H103N57NaS	470.9805	-0.1	39.0	388	55.57	17.0	even	ok
	371	C49H124N33O9P2S	470.9813	1.7	39.0	389	39.46	7.0	even	ok
	372	C45H36NaO43P2S2	470.9809	0.9	39.0	390	56.94	30.0	even	ok
	373	C53H5N22O27S	470.9800	-1.2	39.0	391	44.24	64.0	even	ok
	374	C51H129N27NaO11P2S	470.9814	1.9	39.0	392	37.50	3.0	even	ok
	375	C48H19N10NaO39P	470.9810	1.0	39.1	393	46.21	46.0	even	ok
	376	C48H135N17O27P	470.9811	1.3	39.1	395	42.72	-8.0	even	ok
	377	C43H31N6O41P2S2	470.9808	0.7	39.1	396	59.25	34.0	even	ok
	378	C51H126N23O22	470.9810	1.1	39.3	397	44.85	2.0	even	ok

Compound Spectrum SmartFormula Report

Meas. m/z	#	Ion Formula	m/z	err [ppm]	mSigma	# mSigma	Score	rdb	e¯ Conf	N-Rule
	379	C43H21N10O44S	470.9806	0.1	39.3	398	66.25	40.0	even	ok
	380	C51H134N19O21S2	470.9809	0.8	39.3	399	57.91	-4.0	even	ok
	381	C49H25O50	470.9799	-1.3	39.3	400	42.75	39.0	even	ok
	382	C37H15N26O29P2S2	470.9799	-1.2	39.3	401	52.40	46.0	even	ok
	383	C40H102N57S	470.9813	1.6	39.5	403	39.79	20.0	even	ok
	384	C46H10N18NaO36	470.9795	-2.1	39.6	404	34.95	52.0	even	ok
	385	C46H7N24NaO29P	470.9810	1.0	39.9	405	45.35	57.0	even	ok
	386	C59H145N3NaO27P2	470.9807	0.4	40.0	406	50.75	-9.0	even	ok
	387	C53H131N21O18PS	470.9808	0.5	40.0	407	50.05	1.0	even	ok
	388	C52H14N16NaO29S2	470.9812	1.4	40.0	408	49.38	55.0	even	ok
	389	C49H144N7NaO33P	470.9808	0.6	40.1	409	49.21	-17.0	even	ok
	390	C50H123N31O12PS	470.9803	-0.4	40.1	410	50.56	7.0	even	ok
	391	C50H7N28O18P2S2	470.9798	-1.6	40.2	411	46.88	64.0	even	ok
	392	C58H18N6NaO35S	470.9805	-0.0	40.3	412	65.40	54.0	even	ok
	393	C35H102N59O2S	470.9799	-1.3	40.4	413	41.94	16.0	even	ok
	394	C59H147NO30PS	470.9817	2.4	40.4	414	37.40	-11.0	even	ok
	395	C57H140N9O25P2	470.9806	0.2	40.4	415	52.18	-5.0	even	ok
	396	C52H128N25NaO14PS	470.9804	-0.2	40.5	416	52.09	3.0	even	ok
	397	C55H27N4O34P2S2	470.9802	-0.6	40.5	417	57.87	47.0	even	ok
	398	C48H121N37NaO5P2S	470.9810	0.9	40.6	418	45.20	9.0	even	ok
	399	C60H144NNaO30P	470.9797	-1.7	40.6	419	37.82	-9.0	even	ok
	400	C46H126N25O24	470.9797	-1.7	40.7	420	37.11	-2.0	even	ok
	401	C58H139N7O28P	470.9796	-1.9	40.7	421	35.87	-5.0	even	ok
	402	C39H7N30NaO27PS	470.9812	1.5	40.7	422	39.59	53.0	even	ok
	403	C52H4N26NaO21P2	470.9800	-1.1	40.7	423	43.70	66.0	even	ok
	404	C47H122N29O20	470.9801	-0.8	40.7	424	46.23	3.0	even	ok
	405	C55H136N15NaO20PS	470.9809	0.7	40.8	425	47.01	-3.0	even	ok
	406	C56H152NO28P2S2	470.9800	-1.0	40.9	426	52.38	-16.0	even	ok
	407	C53H139N13NaO23S2	470.9810	1.0	40.9	427	52.97	-8.0	even	ok
	408	C56H139N11O24PS	470.9812	1.5	41.0	428	39.39	-5.0	even	ok
	409	C46H116N43O3P2S	470.9809	0.7	41.0	429	46.69	13.0	even	ok
	410	C49H147NNaO40	470.9794	-2.5	41.0	430	30.16	-22.0	even	ok
	411	C47H135N15NaO30	470.9794	-2.5	41.0	431	30.07	-11.0	even	ok
	412	C52H133N19NaO19P2	470.9794	-2.4	41.1	432	30.64	-2.0	even	ok
	413	C46H14N16O37P	470.9809	0.8	41.1	433	45.65	50.0	even	ok
	414	C48H123N35O8PS2	470.9819	2.9	41.2	434	31.90	7.0	even	ok
	415	C49H120N35NaO8PS	470.9800	-1.2	41.2	435	41.60	9.0	even	ok
	416	C48H22N4NaO46	470.9795	-2.1	41.2	436	33.52	41.0	even	ok
	417	C47H115N41O6PS	470.9799	-1.4	41.3	437	39.64	13.0	even	ok
	418	C45H31N2O45P2S	470.9793	-2.6	41.3	438	34.19	34.0	even	ok
	419	C50H129N29NaO8P2S2	470.9792	-2.7	41.4	439	33.10	3.0	even	ok
	420	C59H21N2O39S	470.9809	0.7	41.5	440	55.24	52.0	even	ok
	421	C57H138N9O27S	470.9802	-0.7	41.6	441	55.80	-5.0	even	ok
	422	C36H12N30NaO25P2S2	470.9796	-2.0	41.7	442	33.90	48.0	even	ok
	423	C44H5N24O34	470.9795	-2.3	41.7	443	31.28	56.0	even	ok
	424	C45H115N45O2PS2	470.9814	1.9	41.8	444	34.20	13.0	even	ok

Compound Spectrum SmartFormula Report

Meas. m/z	#	Ion Formula	m/z	err [ppm]	mSigma	# mSigma	Score	rdb	e¯ Conf	N-Rule
	425	C52H12N22NaO20P2S2	470.9799	-1.4	41.8	445	47.06	60.0	even	ok
	426	C51H122N29O15S	470.9793	-2.6	41.9	446	28.69	7.0	even	ok
	427	C48H128N25NaO19P	470.9812	1.5	42.0	448	37.71	-1.0	even	ok
	428	C60H19N6O33P2	470.9817	2.6	42.0	449	28.64	57.0	even	ok
	429	C36H108N55NaOPS	470.9810	1.0	42.1	450	42.09	12.0	even	ok
	430	C50H123N27NaO18	470.9807	0.4	42.1	451	48.80	4.0	even	ok
	431	C58H144N5NaO26PS	470.9813	1.7	42.1	452	36.33	-9.0	even	ok
	432	C42H18N14NaO40S	470.9802	-0.6	42.1	453	46.01	42.0	even	ok
	433	C47H139N13O31P	470.9807	0.4	42.2	454	48.34	-13.0	even	ok
	434	C53H17N12O33S2	470.9815	2.1	42.2	455	38.35	53.0	even	ok
	435	C51H132N25O12P2S2	470.9796	-2.0	42.3	456	39.86	1.0	even	ok
	436	C57H136N11NaO24P	470.9793	-2.6	42.4	457	27.81	-3.0	even	ok
	437	C47H132N21NaO23P	470.9808	0.6	42.4	458	46.21	-6.0	even	ok
	438	C62H24NaO35P2	470.9818	2.8	42.4	459	26.73	53.0	even	ok
	439	C56H137N13NaO21P2	470.9803	-0.5	42.5	461	46.67	-3.0	even	ok
	440	C54H130N19O21S	470.9798	-1.6	42.5	462	36.37	1.0	even	ok
	441	C50H128N25O17P2	470.9793	-2.6	42.6	463	27.80	2.0	even	ok
	442	C53H127N23NaO17S	470.9794	-2.4	42.6	464	29.78	3.0	even	ok
	443	C59H143N3NaO29S	470.9803	-0.5	42.8	465	56.26	-9.0	even	ok
	444	C54H142N9O27S2	470.9813	1.7	42.8	466	42.01	-10.0	even	ok
	445	C34H7N36O23P2S2	470.9795	-2.2	42.8	467	31.18	52.0	even	ok
	446	C55H131N17O22P	470.9792	-2.8	42.8	468	25.89	1.0	even	ok
	447	C47H120N39NaO4PS2	470.9815	2.1	42.9	469	31.40	9.0	even	ok
	448	C46H112N45NaO2PS	470.9795	-2.2	42.9	470	31.20	15.0	even	ok
	449	C57H11N16O27P2	470.9813	1.6	43.0	471	35.93	63.0	even	ok
	450	C59H16N10NaO29P2	470.9814	1.8	43.0	472	34.17	59.0	even	ok
	451	C46H17N10O44	470.9795	-2.3	43.1	473	30.13	45.0	even	ok
	452	C45H130N21O28	470.9793	-2.7	43.1	474	26.75	-7.0	even	ok
	453	C48H26N2O47P	470.9809	0.8	43.3	475	42.91	39.0	even	ok
	454	C44H27N6NaO43PS	470.9817	2.4	43.3	476	28.60	36.0	even	ok
	455	C54H132N19O19P2	470.9802	-0.7	43.3	477	43.79	1.0	even	ok
	456	C44H107N51PS	470.9794	-2.4	43.4	478	29.19	19.0	even	ok
	457	C48H118N33O16	470.9806	0.2	43.4	479	48.83	8.0	even	ok
	458	C53H15N18O24P2S2	470.9802	-0.6	43.5	480	53.20	58.0	even	ok
	459	C40H13N20O38S	470.9801	-0.8	43.5	481	42.40	46.0	even	ok
	460	C56H135N13NaO23S	470.9799	-1.4	43.6	482	37.05	-3.0	even	ok
	461	C53H137N19NaO14P2S2	470.9797	-1.8	43.7	483	40.29	-3.0	even	ok
	462	C45H11N20NaO33P	470.9805	0.0	43.9	484	49.28	52.0	even	ok
	463	C61H18N4O36P	470.9807	0.4	43.9	485	45.54	57.0	even	ok
	464	C46H123N31O17P	470.9811	1.3	43.9	486	37.48	3.0	even	ok
	465	C45H123N29NaO20	470.9793	-2.5	44.0	488	27.59	0.0	even	ok
	466	C46H119N33NaO16	470.9798	-1.5	44.1	489	35.37	5.0	even	ok
	467	C49H124N29NaO15P	470.9817	2.5	44.1	490	27.65	4.0	even	ok
	468	C58H10N14O30P	470.9803	-0.5	44.1	491	44.57	63.0	even	ok
	469	C55H22N6NaO35S2	470.9816	2.3	44.2	492	34.36	49.0	even	ok
	470	C56H10N18O26PS	470.9818	2.8	44.2	493	29.91	63.0	even	ok

Compound Spectrum SmartFormula Report

Meas. m/z	#	Ion Formula	m/z	err [ppm]	mSigma	# mSigma	Score	rdb	e⁻ Conf	N-Rule
	471	C54H127N21NaO20	470.9816	2.3	44.3	494	29.26	3.0	even	ok
	472	C39H22N14NaO40S2	470.9813	1.8	44.3	495	40.05	37.0	even	ok
	473	C58H16N12NaO26P2S	470.9792	-2.8	44.4	496	29.82	59.0	even	ok
	474	C45H111N47NaOS2	470.9801	-0.9	44.4	497	40.32	15.0	even	ok
	475	C60H15N8NaO32P	470.9804	-0.3	44.5	498	45.97	59.0	even	ok
	476	C49H122N33O11S2	470.9809	0.8	44.5	499	50.13	7.0	even	ok
	477	C45H127N27O21P	470.9807	0.4	44.5	500	45.32	-2.0	even	ok
	478	C56H8N20NaO23P2	470.9809	0.8	44.6	501	40.97	65.0	even	ok
	479	C42H22N12O41PS	470.9816	2.2	44.6	502	29.12	40.0	even	ok
	480	C36H14N24NaO34S2	470.9809	0.8	44.6	503	49.70	43.0	even	ok
	481	C56H147N3NaO29S2	470.9814	1.9	44.6	504	37.87	-14.0	even	ok
	482	C42H30N4NaO46S2	470.9818	2.7	44.7	505	30.51	31.0	even	ok
	483	C56H26N2O37PS2	470.9792	-2.7	44.7	506	30.22	47.0	even	ok
	484	C39H10N24NaO34S	470.9798	-1.6	44.7	507	34.33	48.0	even	ok
	485	C37H17N20O38S2	470.9813	1.6	44.8	508	41.46	41.0	even	ok
	486	C54H3N26O21P2	470.9808	0.7	44.9	509	42.32	69.0	even	ok
	487	C52H122N27O18	470.9815	2.1	44.9	510	30.26	7.0	even	ok
	488	C40H25N10O44S2	470.9817	2.5	44.9	511	32.08	35.0	even	ok
	489	C46H114N43O5S2	470.9804	-0.2	45.0	512	46.42	13.0	even	ok
	490	C54H140N15O18P2S2	470.9800	-1.0	45.0	513	46.51	-5.0	even	ok
	491	C59H19N8O30P2S	470.9796	-2.1	45.1	514	36.13	57.0	even	ok
	492	C46H136N17NaO27P	470.9803	-0.4	45.2	515	44.49	-11.0	even	ok
	493	C55H20N12NaO26P2S2	470.9803	-0.4	45.3	516	52.64	54.0	even	ok
	494	C57H7N18NaO26P	470.9799	-1.3	45.3	517	36.50	65.0	even	ok
	495	C34H9N30O32S2	470.9808	0.6	45.4	519	50.69	47.0	even	ok
	496	C47H23N6NaO43P	470.9805	0.0	45.4	520	47.13	41.0	even	ok
	497	C51H5N26O23S2	470.9815	2.1	45.5	522	34.94	64.0	even	ok
	498	C33H6N34NaO28S2	470.9804	-0.2	45.6	523	55.08	49.0	even	ok
	499	C41H19N16NaO37PS	470.9812	1.5	45.6	524	34.37	42.0	even	ok
	500	C47H119N35O13P	470.9816	2.3	45.7	525	27.91	8.0	even	ok
485.6555	1	C45H127N37O11PS2	485.6559	1.0	15.2	1	83.38	3.0	even	ok
	2	C47H132N31NaO13PS2	485.6560	1.2	15.5	2	79.33	-1.0	even	ok
	3	C56H151N3O33PS	485.6557	0.5	15.5	3	91.91	-15.0	even	ok
	4	C45H123N39NaO10S2	485.6546	-1.8	15.6	4	68.31	5.0	even	ok
	5	C56H147N5NaO32S	485.6544	-2.2	15.6	5	60.17	-13.0	even	ok
	6	C54H142N11O30S	485.6543	-2.4	15.6	6	56.90	-9.0	even	ok
	7	C53H17N14O36S	485.6545	-2.0	15.8	7	63.53	54.0	even	ok
	8	C55H22N8NaO38S	485.6546	-1.8	16.0	8	66.61	50.0	even	ok
	9	C46H126N35O14S2	485.6549	-1.0	16.1	9	81.19	3.0	even	ok
	10	C57H150NO36S	485.6547	-1.5	16.1	10	72.60	-15.0	even	ok
	11	C53H14N20O29PS	485.6559	0.9	16.1	12	83.16	59.0	even	ok
	12	C54H23N10NaO35PS	485.6556	0.2	16.2	13	97.19	50.0	even	ok
	13	C55H19N14NaO31PS	485.6560	1.1	16.3	14	79.17	55.0	even	ok
	14	C48H135N27O17PS2	485.6564	1.9	16.4	15	64.51	-3.0	even	ok
	15	C43H118N45O8S2	485.6545	-2.0	16.5	16	53.03	9.0	even	ok
	16	C55H26N6O39PS	485.6559	0.9	16.5	17	82.34	48.0	even	ok

Compound Spectrum SmartFormula Report

Meas. m/z	#	Ion Formula	m/z	err [ppm]	mSigma	# mSigma	Score	rdb	e⁻ Conf	N-Rule
	17	C46H7N34NaO19PS2	485.6562	1.6	16.6	18	70.24	62.0	even	ok
	18	C52H18N16O33PS	485.6555	0.0	16.6	19	100.00	54.0	even	ok
	19	C44H124N41NaO7PS2	485.6556	0.3	16.6	20	79.26	5.0	even	ok
	20	C56H22N10O35PS	485.6564	1.9	16.9	21	64.99	53.0	even	ok
	21	C57H31NaO41PS	485.6560	1.1	16.9	22	78.12	44.0	even	ok
	22	C45H130N31O18S2	485.6545	-2.0	17.0	23	63.17	-2.0	even	ok
	23	C48H131N29NaO16S2	485.6550	-0.9	17.0	24	83.31	-1.0	even	ok
	24	C55H148N7NaO29PS	485.6554	-0.2	17.0	25	80.08	-13.0	even	ok
	25	C53H10N22NaO28S	485.6546	-1.8	17.0	26	65.16	61.0	even	ok
	26	C54H13N18O32S	485.6549	-1.1	17.2	28	78.18	59.0	even	ok
	27	C47H135N25NaO20S2	485.6546	-1.8	17.2	29	66.22	-6.0	even	ok
	28	C56H25N4O42S	485.6549	-1.1	17.3	30	78.14	48.0	even	ok
	29	C42H119N47O5PS2	485.6555	0.1	17.3	31	81.20	9.0	even	ok
	30	C50H140N21NaO19PS2	485.6565	2.1	17.4	32	59.91	-7.0	even	ok
	31	C46H136N27NaO17PS2	485.6556	0.3	17.5	33	93.25	-6.0	even	ok
	32	C53H24N12NaO32P2S	485.6566	2.3	17.7	34	57.22	50.0	even	ok
	33	C53H143N13O27PS	485.6553	-0.4	17.8	35	75.78	-9.0	even	ok
	34	C58H27N4NaO37PS	485.6565	2.1	17.9	36	60.40	49.0	even	ok
	35	C44H131N33O15PS2	485.6555	0.1	18.0	37	96.05	-2.0	even	ok
	36	C52H11N24NaO25PS	485.6556	0.2	18.0	38	78.09	61.0	even	ok
	37	C56H18N12NaO34S	485.6550	-0.9	18.0	39	80.31	55.0	even	ok
	38	C57H147N7O29PS	485.6562	1.5	18.1	40	70.32	-10.0	even	ok
	39	C47H6N32NaO22S2	485.6552	-0.5	18.1	41	88.74	62.0	even	ok
	40	C51H5N28O26S	485.6545	-2.0	18.2	42	50.34	65.0	even	ok
	41	C47H10N30O23PS2	485.6566	2.3	18.2	43	55.62	60.0	even	ok
	42	C47H139N23O21PS2	485.6559	1.0	18.2	44	78.36	-8.0	even	ok
	43	C43H125N43NaO4P2S2	485.6566	2.3	18.3	45	46.29	5.0	even	ok
	44	C48H7N34O17P2S2	485.6543	-2.4	18.5	46	54.00	65.0	even	ok
	45	C49H144N17NaO23PS2	485.6560	1.2	18.5	47	74.36	-12.0	even	ok
	46	C48H138N21O24S2	485.6550	-1.0	18.6	48	77.13	-8.0	even	ok
	47	C49H134N25O20S2	485.6554	-0.1	18.7	49	94.02	-3.0	even	ok
	48	C50H6N30O23PS	485.6555	0.0	18.7	50	80.02	65.0	even	ok
	49	C51H15N20NaO29PS	485.6551	-0.7	18.8	51	69.08	56.0	even	ok
	50	C59H152NNaO31PS	485.6563	1.7	18.8	52	65.93	-14.0	even	ok
	51	C54H149N9NaO26P2S	485.6564	1.9	18.8	53	51.97	-13.0	even	ok
	52	C41H116N51NaOPS2	485.6551	-0.7	19.1	55	69.48	11.0	even	ok
	53	C48H128N35NaO9PS2	485.6565	2.1	19.2	56	57.85	4.0	even	ok
	54	C54H152N3NaO33PS	485.6549	-1.1	19.2	57	75.11	-18.0	even	ok
	55	C51H12N26NaO22P2S	485.6566	2.3	19.3	58	46.19	61.0	even	ok
	56	C57H21N8O38S	485.6554	-0.2	19.3	59	91.48	53.0	even	ok
	57	C52H140N17NaO23PS	485.6549	-1.1	19.6	60	62.03	-7.0	even	ok
	58	C50H143N15NaO26S2	485.6550	-0.8	19.6	61	79.07	-12.0	even	ok
	59	C41H120N49O2P2S2	485.6565	2.1	19.6	62	47.52	9.0	even	ok
	60	C56H144N11NaO25PS	485.6558	0.7	19.7	63	67.48	-8.0	even	ok
	61	C54H139N17O23PS	485.6557	0.5	19.7	64	70.36	-4.0	even	ok
	62	C57H143N9NaO28S	485.6548	-1.3	19.7	65	70.55	-8.0	even	ok

Compound Spectrum SmartFormula Report

Meas. m/z	#	Ion Formula	m/z	err [ppm]	mSigma	# mSigma	Score	rdb	e⁻ Conf	N-Rule
	63	C46H123N41O7PS2	485.6564	1.9	19.8	66	50.18	8.0	even	ok
	64	C43H115N51OPS2	485.6559	1.0	19.8	67	63.31	14.0	even	ok
	65	C43H128N37NaO11PS2	485.6551	-0.6	19.8	68	68.51	0.0	even	ok
	66	C50H12N28NaO19P2S2	485.6544	-2.2	19.9	69	55.36	61.0	even	ok
	67	C45H120N45NaO3PS2	485.6560	1.2	19.9	70	60.40	10.0	even	ok
	68	C44H5N34O24S2	485.6547	-1.6	19.9	71	65.99	61.0	even	ok
	69	C49H10N26O27PS	485.6550	-0.9	20.1	72	64.39	60.0	even	ok
	70	C51H19N18O30P2S	485.6565	2.1	20.1	73	47.83	54.0	even	ok
	71	C51H139N19NaO22S2	485.6555	0.1	20.2	74	92.02	-7.0	even	ok
	72	C52H144N15O24P2S	485.6563	1.7	20.2	75	53.10	-9.0	even	ok
	73	C50H147N13O27PS2	485.6564	1.9	20.2	76	59.43	-14.0	even	ok
	74	C58H146N5O32S	485.6552	-0.6	20.3	77	82.54	-10.0	even	ok
	75	C46H10N28NaO26S2	485.6548	-1.4	20.3	78	68.69	57.0	even	ok
	76	C50H19N20O27P2S2	485.6543	-2.4	20.3	79	52.00	54.0	even	ok
	77	C48H9N28O26S2	485.6556	0.3	20.4	80	87.89	60.0	even	ok
	78	C42H3N40NaO17PS2	485.6553	-0.2	20.5	81	88.25	63.0	even	ok
	79	C45H11N30NaO23PS2	485.6558	0.7	20.5	82	80.56	57.0	even	ok
	80	C55H29O46S	485.6545	-2.0	20.5	83	57.67	43.0	even	ok
	81	C49H7N32O20P2S	485.6565	2.1	20.6	84	47.43	65.0	even	ok
	82	C55H138N15O26S	485.6547	-1.5	20.6	85	54.96	-4.0	even	ok
	83	C43H6N36O21PS2	485.6557	0.5	20.6	86	83.67	61.0	even	ok
	84	C49H3N34NaO19PS	485.6551	-0.7	20.7	87	66.27	67.0	even	ok
	85	C47H122N39O10S2	485.6554	-0.1	20.7	88	89.88	8.0	even	ok
	86	C59H26N2NaO40S	485.6555	0.0	20.8	89	91.89	49.0	even	ok
	87	C54H156NO34P2S	485.6563	1.7	20.9	90	62.70	-20.0	even	ok
	88	C60H27NaO41P	485.6549	-1.2	20.9	91	59.34	49.0	even	ok
	89	C44H114N49O4S2	485.6549	-1.1	21.0	92	60.97	14.0	even	ok
	90	C50H135N23O21PS	485.6548	-1.3	21.0	93	57.38	-3.0	even	ok
	91	C41H123N43O9PS2	485.6551	-0.8	21.1	94	63.97	4.0	even	ok
	92	C53H136N21NaO19PS	485.6554	-0.2	21.1	95	73.38	-2.0	even	ok
	93	C54H135N19NaO22S	485.6544	-2.2	21.1	96	44.59	-2.0	even	ok
	94	C52H152N7NaO29PS2	485.6565	2.1	21.2	98	55.20	-18.0	even	ok
	95	C53H27N6NaO39PS	485.6551	-0.7	21.3	99	78.76	45.0	even	ok
	96	C52H130N25O20S	485.6543	-2.4	21.3	100	42.05	2.0	even	ok
	97	C54H30N2O43PS	485.6555	0.0	21.4	101	90.32	43.0	even	ok
	98	C43H111N53NaS2	485.6546	-1.8	21.4	102	50.22	16.0	even	ok
	99	C46H14N26O27PS2	485.6561	1.4	21.6	103	66.12	55.0	even	ok
	100	C46H119N43NaO6S2	485.6550	-0.9	21.7	104	62.72	10.0	even	ok
	101	C56H15N18NaO27PS	485.6565	2.1	21.7	105	55.80	60.0	even	ok
	102	C50H16N22NaO26P2S	485.6561	1.3	21.7	106	55.95	56.0	even	ok
	103	C52H24N14NaO29P2S2	485.6544	-2.2	21.8	107	53.28	50.0	even	ok
	104	C52H140N21O17P2S2	485.6545	-1.9	21.8	108	58.23	-4.0	even	ok
	105	C51H146N11O30S2	485.6554	-0.1	21.9	109	87.90	-14.0	even	ok
	106	C51H131N27O17PS	485.6553	-0.4	21.9	110	69.35	2.0	even	ok
	107	C52H147N9O31PS	485.6548	-1.3	21.9	111	56.38	-14.0	even	ok
	108	C54H152N7O27P2S2	485.6545	-1.9	21.9	112	58.25	-15.0	even	ok

Compound Spectrum SmartFormula Report

Meas. m/z	#	Ion Formula	m/z	err [ppm]	mSigma	# mSigma	Score	rdb	e⁻ Conf	N-Rule
	109	C50H14N22NaO28S2	485.6557	0.5	22.0	113	81.62	56.0	even	ok
	110	C49H127N33NaO12S2	485.6555	0.1	22.0	114	88.62	4.0	even	ok
	111	C51H15N24O23P2S2	485.6547	-1.5	22.1	115	64.29	59.0	even	ok
	112	C48H7N30NaO23PS	485.6547	-1.6	22.1	116	51.54	62.0	even	ok
	113	C51H22N12O37PS	485.6550	-0.9	22.1	117	74.06	49.0	even	ok
	114	C48H19N20NaO29PS2	485.6562	1.6	22.1	118	62.34	51.0	even	ok
	115	C47H13N24O30S2	485.6551	-0.6	22.1	119	78.29	55.0	even	ok
	116	C58H22N6O39P	485.6548	-1.4	22.3	120	54.93	53.0	even	ok
	117	C52H137N23NaO16P2S	485.6564	1.9	22.4	121	48.27	-2.0	even	ok
	118	C51H141N19NaO20P2S	485.6559	0.9	22.6	122	60.40	-7.0	even	ok
	119	C52H142N15O26S2	485.6558	0.8	22.6	123	74.80	-9.0	even	ok
	120	C40H120N47NaO5PS2	485.6547	-1.6	22.7	124	51.74	6.0	even	ok
	121	C54H10N24O25PS	485.6564	1.9	22.7	125	47.95	64.0	even	ok
	122	C62H26O41P	485.6557	0.5	22.7	126	66.78	52.0	even	ok
	123	C50H19N16NaO33PS	485.6547	-1.6	22.9	127	60.86	51.0	even	ok
	124	C53H7N28NaO21PS	485.6560	1.1	23.1	130	57.24	66.0	even	ok
	125	C49H18N18NaO32S2	485.6552	-0.4	23.1	131	79.87	51.0	even	ok
	126	C48H4N36NaO16P2S	485.6561	1.3	23.2	132	54.29	67.0	even	ok
	127	C53H31N4O40P2S	485.6565	2.1	23.2	133	53.50	43.0	even	ok
	128	C55H9N22O28S	485.6554	-0.2	23.3	134	83.90	64.0	even	ok
	129	C53H151N5NaO32S2	485.6555	0.1	23.3	135	85.86	-18.0	even	ok
	130	C49H5N32O22S2	485.6560	1.2	23.3	136	67.24	65.0	even	ok
	131	C48H11N28O24P2S	485.6560	1.1	23.4	137	56.47	60.0	even	ok
	132	C50H130N29O16S2	485.6558	0.8	23.5	138	73.45	2.0	even	ok
	133	C51H144N13NaO27PS	485.6545	-2.0	23.5	139	44.98	-12.0	even	ok
	134	C54H145N15NaO19P2S2	485.6546	-1.7	23.5	140	59.03	-8.0	even	ok
	135	C59H28N2NaO38P2	485.6559	0.9	23.5	141	59.99	49.0	even	ok
	136	C49H147N11NaO30S2	485.6546	-1.8	23.6	142	57.75	-17.0	even	ok
	137	C49H132N27NaO17PS	485.6545	-2.0	23.7	143	44.74	-1.0	even	ok
	138	C60H27N4O35P2S	485.6545	-1.9	23.7	144	54.96	52.0	even	ok
	139	C56H157NNaO29P2S2	485.6546	-1.7	23.7	145	58.93	-19.0	even	ok
	140	C52H28N8NaO36P2S	485.6561	1.3	23.7	146	64.17	45.0	even	ok
	141	C50H132N29O14P2S	485.6563	1.7	23.7	147	49.30	2.0	even	ok
	142	C53H153N5NaO30P2S	485.6559	1.0	23.8	148	58.73	-18.0	even	ok
	143	C53H20N18NaO25P2S2	485.6548	-1.3	23.9	150	64.74	55.0	even	ok
	144	C49H22N16O33PS2	485.6566	2.3	24.1	151	48.81	49.0	even	ok
	145	C49H3N38O13P2S2	485.6547	-1.5	24.2	152	61.21	70.0	even	ok
	146	C61H23N4NaO37P	485.6553	-0.3	24.2	154	67.54	54.0	even	ok
	147	C50H128N31NaO13PS	485.6549	-1.1	24.3	155	55.84	4.0	even	ok
	148	C57H14N16NaO30S	485.6555	-0.0	24.4	156	84.93	60.0	even	ok
	149	C49H136N25O18P2S	485.6558	0.8	24.4	157	60.54	-3.0	even	ok
	150	C54H147N9NaO28S2	485.6559	1.0	24.5	158	68.67	-13.0	even	ok
	151	C62H22N2NaO40	485.6543	-2.3	24.5	159	40.62	54.0	even	ok
	152	C53H27N10O33P2S2	485.6547	-1.5	24.5	160	61.01	48.0	even	ok
	153	C57H26N2O43P	485.6543	-2.3	24.6	161	40.84	48.0	even	ok
	154	C41H4N42NaO14P2S2	485.6563	1.8	24.7	162	46.48	63.0	even	ok

Compound Spectrum SmartFormula Report

Meas. m/z	#	Ion Formula	m/z	err [ppm]	mSigma	# mSigma	Score	rdb	e¯ Conf	N-Rule
	155	C51H17N18O32S2	485.6560	1.2	24.7	163	64.99	54.0	even	ok
	156	C58H143N11O25PS	485.6566	2.4	24.8	164	47.33	-5.0	even	ok
	157	C54H6N26NaO24S	485.6550	-0.9	24.9	165	57.57	66.0	even	ok
	158	C50H23N14O34P2S	485.6560	1.2	24.9	166	65.43	49.0	even	ok
	159	C59H18N10O35P	485.6552	-0.4	24.9	167	63.89	58.0	even	ok
	160	C57H19N10NaO35P	485.6544	-2.1	25.1	168	42.55	55.0	even	ok
	161	C60H24N6NaO34P2	485.6563	1.8	25.2	169	46.12	54.0	even	ok
	162	C51H10N26NaO24S2	485.6561	1.4	25.2	170	61.49	61.0	even	ok
	163	C52H135N23NaO18S2	485.6559	1.0	25.2	171	67.64	-2.0	even	ok
	164	C49H139N19O25PS	485.6544	-2.2	25.2	172	40.99	-8.0	even	ok
	165	C57H23N8O36P2	485.6558	0.7	25.3	174	60.14	53.0	even	ok
	166	C51H148N11O28P2S	485.6558	0.8	25.4	175	59.16	-14.0	even	ok
	167	C50H150N7O34S2	485.6550	-1.0	25.4	176	66.55	-19.0	even	ok
	168	C47H127N33O15PS	485.6544	-2.2	25.5	177	40.62	3.0	even	ok
	169	C61H152NO29P2S	485.6543	-2.3	25.6	178	47.07	-11.0	even	ok
	170	C58H17N12O34S	485.6558	0.7	25.6	179	71.09	58.0	even	ok
	171	C50H21N14O36S2	485.6556	0.3	25.6	180	78.15	49.0	even	ok
	172	C48H123N37O11PS	485.6548	-1.3	25.6	181	51.68	8.0	even	ok
	173	C59H147N3O33P	485.6546	-1.8	25.7	182	45.89	-10.0	even	ok
	174	C55H148N11O23P2S2	485.6550	-1.0	25.8	183	67.21	-10.0	even	ok
	175	C51H8N32NaO15P2S2	485.6548	-1.3	25.8	184	61.91	66.0	even	ok
	176	C47H8N32NaO20P2S	485.6557	0.4	25.8	185	63.00	62.0	even	ok
	177	C54H154NO36S2	485.6559	0.8	26.1	186	69.01	-20.0	even	ok
	178	C50H128N35O7P2S2	485.6545	-1.9	26.2	187	52.70	7.0	even	ok
	179	C48H14N22O31PS	485.6546	-1.8	26.2	188	44.72	55.0	even	ok
	180	C52H155NNaO36S2	485.6550	-0.8	26.2	189	68.31	-23.0	even	ok
	181	C55H32N4NaO35P2S2	485.6548	-1.3	26.2	190	61.48	44.0	even	ok
	182	C45H14N24NaO30S2	485.6543	-2.3	26.3	191	46.99	52.0	even	ok
	183	C55H135N21O19PS	485.6562	1.5	26.4	192	48.95	1.0	even	ok
	184	C58H19N12O32P2	485.6562	1.6	26.4	193	47.18	58.0	even	ok
	185	C52H31N6O37P2S2	485.6543	-2.4	26.4	194	45.51	43.0	even	ok
	186	C53H22N12NaO34S2	485.6561	1.4	26.6	195	59.41	50.0	even	ok
	187	C49H129N33NaO10P2S	485.6559	0.9	26.6	196	55.22	4.0	even	ok
	188	C54H23N14O29P2S2	485.6552	-0.6	26.6	197	72.05	53.0	even	ok
	189	C55H14N16O33P	485.6543	-2.3	26.9	198	38.66	59.0	even	ok
	190	C57H140N15NaO21PS	485.6563	1.7	26.9	199	46.04	-3.0	even	ok
	191	C52H26N8NaO38S2	485.6557	0.5	27.0	200	72.75	45.0	even	ok
	192	C58H139N13NaO24S	485.6553	-0.4	27.1	201	73.66	-3.0	even	ok
	193	C60H22N6NaO36S	485.6559	0.9	27.2	202	65.61	54.0	even	ok
	194	C57H147N3NaO35	485.6565	2.2	27.2	203	38.96	-13.0	even	ok
	195	C51H151N5O35PS	485.6544	-2.2	27.2	204	47.07	-19.0	even	ok
	196	C54H132N25NaO15PS	485.6558	0.7	27.3	205	56.99	3.0	even	ok
	197	C55H150N5O32S2	485.6563	1.7	27.3	206	53.61	-15.0	even	ok
	198	C58H15N14NaO31P	485.6549	-1.2	27.3	207	51.24	60.0	even	ok
	199	C54H17N12O39	485.6566	2.4	27.3	208	36.55	54.0	even	ok
	200	C48H133N29NaO14P2S	485.6555	0.0	27.3	209	65.93	-1.0	even	ok

Compound Spectrum SmartFormula Report

Meas. m/z	#	Ion Formula	m/z	err [ppm]	mSigma	# mSigma	Score	rdb	e⁻ Conf	N-Rule
	201	C53H138N19O22S2	485.6563	1.7	27.3	210	53.67	-4.0	even	ok
	202	C50H145N15NaO24P2S	485.6555	0.0	27.4	211	65.81	-12.0	even	ok
	203	C52H127N31O13PS	485.6557	0.5	27.4	212	59.25	7.0	even	ok
	204	C52H133N29NaO9P2S2	485.6546	-1.7	27.5	213	53.76	3.0	even	ok
	205	C44H15N26NaO27PS2	485.6553	-0.2	27.5	214	75.35	52.0	even	ok
	206	C54H36NaO39P2S2	485.6544	-2.2	27.6	215	46.75	39.0	even	ok
	207	C46H17N20O34S2	485.6547	-1.6	27.6	216	55.56	50.0	even	ok
	208	C59H142N9O28S	485.6556	0.3	27.7	217	73.70	-5.0	even	ok
	209	C52H11N28O19P2S2	485.6552	-0.6	27.7	218	70.12	64.0	even	ok
	210	C57H153N5NaO25P2S2	485.6551	-0.8	27.7	219	67.04	-14.0	even	ok
	211	C52H13N22O28S2	485.6565	2.1	27.8	220	47.67	59.0	even	ok
	212	C45H3N38O18P2S	485.6556	0.2	27.9	221	62.51	66.0	even	ok
	213	C41H7N36NaO21PS2	485.6549	-1.2	28.0	222	60.77	58.0	even	ok
	214	C48H22N14NaO36S2	485.6548	-1.4	28.0	223	57.79	46.0	even	ok
	215	C42H10N32O25PS2	485.6552	-0.4	28.0	224	71.57	56.0	even	ok
	216	C53H126N29O16S	485.6547	-1.5	28.3	225	46.08	7.0	even	ok
	217	C49H20N18NaO30P2S	485.6557	0.4	28.3	226	59.36	51.0	even	ok
	218	C47H23N16NaO33PS2	485.6558	0.7	28.3	227	67.34	46.0	even	ok
	219	C47H124N39O8P2S	485.6558	0.7	28.3	228	55.38	8.0	even	ok
	220	C45H18N22O31PS2	485.6557	0.5	28.3	229	70.16	50.0	even	ok
	221	C48H118N43O6S2	485.6558	0.8	28.4	230	65.75	13.0	even	ok
	222	C56H20N12NaO32P2	485.6554	-0.0	28.4	231	63.90	55.0	even	ok
	223	C56H134N19O22S	485.6552	-0.6	28.5	232	57.04	1.0	even	ok
	224	C56H10N20O29P	485.6548	-1.4	28.5	233	47.50	64.0	even	ok
	225	C58H148N5O30P2	485.6556	0.3	28.6	234	60.71	-10.0	even	ok
	226	C56H27N4O40P2	485.6553	-0.2	28.6	235	61.34	48.0	even	ok
	227	C47H120N41NaO7PS	485.6545	-2.0	28.6	236	39.79	10.0	even	ok
	228	C56H28N8NaO31P2S2	485.6553	-0.4	28.7	237	71.48	49.0	even	ok
	229	C55H131N23NaO18S	485.6548	-1.3	28.7	238	47.90	3.0	even	ok
	230	C53H136N25O13P2S2	485.6550	-1.0	28.9	239	62.39	1.0	even	ok
	231	C62H148NNaO31P	485.6551	-0.7	28.9	240	55.74	-9.0	even	ok
	232	C52H31N2NaO43PS	485.6547	-1.6	28.9	241	53.11	40.0	even	ok
	233	C61H25N2O40S	485.6563	1.7	29.0	243	52.49	52.0	even	ok
	234	C61H147N3NaO30S	485.6557	0.5	29.0	244	68.57	-9.0	even	ok
	235	C45H110N53S2	485.6554	-0.1	29.1	245	61.74	19.0	even	ok
	236	C55H142N9O33	485.6565	2.0	29.2	246	39.16	-9.0	even	ok
	237	C51H124N35NaO9PS	485.6554	-0.2	29.2	247	60.85	9.0	even	ok
	238	C58H15N18O25P2S	485.6545	-2.0	29.2	248	48.20	63.0	even	ok
	239	C57H16N16NaO28P2	485.6559	0.9	29.2	249	52.64	60.0	even	ok
	240	C48H140N21O22P2S	485.6554	-0.2	29.3	250	61.10	-8.0	even	ok
	241	C56H35O39P2S2	485.6552	-0.6	29.3	251	67.66	42.0	even	ok
	242	C55H143N13NaO24S2	485.6564	1.9	29.3	252	48.63	-8.0	even	ok
	243	C52H123N33NaO12S	485.6544	-2.3	29.4	253	36.79	9.0	even	ok
	244	C46H128N35O12P2S	485.6554	-0.2	29.4	254	60.82	3.0	even	ok
	245	C43H16N28NaO24P2S2	485.6563	1.8	29.5	255	49.77	52.0	even	ok
	246	C54H16N22NaO21P2S2	485.6553	-0.4	29.6	257	69.77	60.0	even	ok

Compound Spectrum SmartFormula Report

Meas. m/z	#	Ion Formula	m/z	err [ppm]	mSigma	# mSigma	Score	rdb	e⁻ Conf	N-Rule
	247	C60H143N7O29P	485.6550	-0.8	29.6	258	52.44	-5.0	even	ok
	248	C50H118N39O10S	485.6543	-2.4	29.7	259	34.62	13.0	even	ok
	249	C54H25N8O38S2	485.6565	2.1	29.7	260	45.49	48.0	even	ok
	250	C50H123N37NaO8S2	485.6559	1.0	29.7	261	60.92	9.0	even	ok
	251	C61H149N3NaO28P2	485.6561	1.4	29.8	262	45.84	-9.0	even	ok
	252	C48H26N12O37PS2	485.6561	1.4	29.8	263	54.70	44.0	even	ok
	253	C50H26N8O41PS	485.6546	-1.8	29.8	264	49.34	44.0	even	ok
	254	C53H29N4O42S2	485.6560	1.2	29.8	265	57.52	43.0	even	ok
	255	C54H18N16NaO30S2	485.6566	2.3	29.9	266	42.94	55.0	even	ok
	256	C49H119N41O7PS	485.6553	-0.4	29.9	267	57.52	13.0	even	ok
	257	C50H125N37NaO6P2S	485.6564	1.9	30.0	268	40.54	9.0	even	ok
	258	C49H25N10O40S2	485.6551	-0.6	30.0	269	65.38	44.0	even	ok
	259	C57H152NO34P2	485.6552	-0.6	30.0	270	54.51	-15.0	even	ok
	260	C47H15N24O28P2S	485.6556	0.2	30.0	271	59.26	55.0	even	ok
	261	C52H5N26O29	485.6566	2.4	30.1	273	34.25	65.0	even	ok
	262	C47H115N47NaO2S2	485.6555	0.1	30.1	274	61.21	15.0	even	ok
	263	C63H22N4O37P	485.6561	1.4	30.2	275	45.37	57.0	even	ok
	264	C50H31N6NaO39PS2	485.6562	1.6	30.2	276	51.55	40.0	even	ok
	265	C53H14N16NaO35	485.6563	1.7	30.3	277	41.76	56.0	even	ok
	266	C41H11N34O22P2S2	485.6562	1.6	30.3	278	51.30	56.0	even	ok
	267	C45H127N33NaO17S	485.6563	1.8	30.3	279	40.99	0.0	even	ok
	268	C45H115N47O5PS	485.6544	-2.2	30.3	280	36.17	14.0	even	ok
	269	C58H156NO29P2S2	485.6554	-0.0	30.4	281	73.30	-16.0	even	ok
	270	C60H20N12NaO27P2S	485.6546	-1.8	30.4	282	49.27	59.0	even	ok
	271	C54H15N18O30P2	485.6553	-0.2	30.5	283	58.55	59.0	even	ok
	272	C55H141N19NaO15P2S2	485.6551	-0.8	30.6	284	62.53	-3.0	even	ok
	273	C40H8N38NaO18P2S2	485.6559	0.9	30.7	285	60.71	58.0	even	ok
	274	C51H30N4NaO42S2	485.6552	-0.4	30.8	287	66.80	40.0	even	ok
	275	C55H11N22O26P2	485.6558	0.7	30.8	288	52.86	64.0	even	ok
	276	C59H144N9O26P2	485.6560	1.2	31.0	289	46.67	-5.0	even	ok
	277	C51H126N33O12S2	485.6563	1.7	31.0	290	49.24	7.0	even	ok
	278	C55H26N2NaO45	485.6563	1.7	31.0	291	40.94	45.0	even	ok
	279	C48H120N43O4P2S	485.6563	1.7	31.1	292	41.48	13.0	even	ok
	280	C52H35O44P2S	485.6560	1.2	31.2	293	56.22	38.0	even	ok
	281	C62H19N8NaO33P	485.6558	0.7	31.3	294	52.53	59.0	even	ok
	282	C54H3N32NaO17PS	485.6564	2.0	31.3	295	37.24	71.0	even	ok
	283	C60H14N14O31P	485.6557	0.5	31.4	296	54.60	63.0	even	ok
	284	C55H24N8NaO36P2	485.6550	-1.0	31.4	297	48.83	50.0	even	ok
	285	C61H23N8O31P2S	485.6550	-1.0	31.5	298	57.64	57.0	even	ok
	286	C56H5N26O24S	485.6558	0.7	31.5	299	61.80	69.0	even	ok
	287	C55H7N24NaO25P	485.6544	-2.1	31.5	300	36.44	66.0	even	ok
	288	C52H6N30NaO20S2	485.6566	2.3	31.5	301	41.35	66.0	even	ok
	289	C46H121N43NaO4P2S	485.6555	0.0	31.6	302	59.55	10.0	even	ok
	290	C51H32N4NaO40P2S	485.6557	0.4	31.6	303	65.62	40.0	even	ok
	291	C57H31N4O35P2S2	485.6556	0.4	31.6	304	66.51	47.0	even	ok
	292	C56H30N2NaO40S2	485.6566	2.3	31.7	305	40.98	44.0	even	ok

Compound Spectrum SmartFormula Report

Meas. m/z	#	Ion Formula	m/z	err [ppm]	mSigma	# mSigma	Score	rdb	e⁻ Conf	N-Rule
	293	C57H145N9NaO26P2	485.6552	-0.4	31.8	306	54.22	-8.0	even	ok
	294	C47H137N25NaO18P2S	485.6550	-0.9	31.9	307	49.06	-6.0	even	ok
	295	C55H19N18O25P2S2	485.6556	0.4	32.0	308	66.06	58.0	even	ok
	296	C55H135N17NaO25	485.6565	2.2	32.0	309	34.73	-2.0	even	ok
	297	C43H115N47NaO7S	485.6563	1.8	32.0	310	39.41	11.0	even	ok
	298	C46H12N28NaO24P2S	485.6552	-0.5	32.0	311	53.36	57.0	even	ok
	299	C59H140N11NaO25P	485.6547	-1.6	32.2	312	41.26	-3.0	even	ok
	300	C64H21N2O40	485.6551	-0.7	32.3	313	51.27	57.0	even	ok
	301	C51H34N2O43PS2	485.6566	2.3	32.3	314	40.10	38.0	even	ok
	302	C61H13N12O34	485.6547	-1.6	32.3	315	41.09	63.0	even	ok
	303	C48H116N45NaO3PS	485.6549	-1.1	32.4	316	45.92	15.0	even	ok
	304	C51H9N22O33	485.6562	1.5	32.5	317	41.58	60.0	even	ok
	305	C54H139N13NaO29	485.6561	1.3	32.5	318	43.76	-7.0	even	ok
	306	C56H144N15O19P2S2	485.6554	-0.0	32.5	319	69.52	-5.0	even	ok
	307	C43H122N39O15S	485.6562	1.6	32.5	320	40.85	4.0	even	ok
	308	C63H18N6NaO36	485.6548	-1.4	32.7	321	42.76	59.0	even	ok
	309	C47H139N19NaO27S	485.6563	1.8	32.7	322	38.61	-11.0	even	ok
	310	C45H125N39NaO8P2S	485.6550	-0.9	32.7	323	47.96	5.0	even	ok
	311	C53H131N27NaO14S2	485.6564	1.9	32.7	324	44.84	3.0	even	ok
	312	C58H10N20NaO26S	485.6559	0.9	32.7	325	57.38	65.0	even	ok
	313	C49H27N10O38P2S	485.6556	0.2	32.8	326	66.25	44.0	even	ok
	314	C57H27N6NaO34PS2	485.6543	-2.4	32.9	327	38.58	49.0	even	ok
	315	C53H21N8O43	485.6562	1.5	33.0	328	40.93	49.0	even	ok
	316	C63H28N2NaO33P2S	485.6550	-0.8	33.1	330	57.89	53.0	even	ok
	317	C59H11N18NaO27P	485.6553	-0.3	33.2	331	54.47	65.0	even	ok
	318	C56H149N5NaO30P2	485.6548	-1.4	33.2	332	42.44	-13.0	even	ok
	319	C54H146N5O37	485.6560	1.1	33.3	333	44.88	-14.0	even	ok
	320	C52H33O46S2	485.6556	0.3	33.3	334	64.86	38.0	even	ok
	321	C57H135N17O23P	485.6546	-1.8	33.4	335	38.08	1.0	even	ok
	322	C60H10N16NaO30	485.6543	-2.3	33.5	337	32.64	65.0	even	ok
	323	C46H111N51OPS	485.6548	-1.3	33.6	338	42.59	19.0	even	ok
	324	C44H116N49O2P2S	485.6554	-0.2	33.6	339	54.81	14.0	even	ok
	325	C53H19N14O34P2	485.6549	-1.2	33.7	340	44.16	54.0	even	ok
	326	C58H12N20NaO24P2	485.6563	1.8	33.7	341	37.61	65.0	even	ok
	327	C59H13N16O30S	485.6563	1.6	33.8	342	46.74	63.0	even	ok
	328	C57H6N24O25P	485.6552	-0.5	33.8	343	51.53	69.0	even	ok
	329	C52H4N36NaO11P2S2	485.6553	-0.4	33.8	344	62.76	71.0	even	ok
	330	C55H140N15O24P2	485.6551	-0.6	33.8	345	49.49	-4.0	even	ok
	331	C53H12N22NaO26P2	485.6550	-1.0	33.9	346	45.89	61.0	even	ok
	332	C53H130N23O23	485.6564	2.0	33.9	348	34.95	2.0	even	ok
	333	C58H141N13NaO22P2	485.6557	0.5	33.9	349	51.14	-3.0	even	ok
	334	C55H15N20NaO24PS2	485.6543	-2.4	33.9	350	37.51	60.0	even	ok
	335	C44H7N34O22P2S	485.6551	-0.7	34.0	351	48.68	61.0	even	ok
	336	C45H132N31O16P2S	485.6549	-1.1	34.0	352	44.43	-2.0	even	ok
	337	C47H117N47NaP2S	485.6559	0.9	34.0	353	46.12	15.0	even	ok
	338	C62H148N5O25P2S	485.6548	-1.4	34.1	354	49.03	-6.0	even	ok

Compound Spectrum SmartFormula Report

Meas. m/z	#	Ion Formula	m/z	err [ppm]	mSigma	# mSigma	Score	rdb	e⁻ Conf	N-Rule
	339	C57H24N12NaO27P2S2	485.6557	0.5	34.1	355	60.15	54.0	even	ok
	340	C54H8N26NaO22P2	485.6554	-0.1	34.1	356	55.46	66.0	even	ok
	341	C41H110N53O5S	485.6562	1.6	34.2	357	39.20	15.0	even	ok
	342	C58H149N9NaO21P2S2	485.6555	0.2	34.5	358	64.71	-9.0	even	ok
	343	C46H14N22NaO33S	485.6565	2.2	34.5	359	33.08	52.0	even	ok
	344	C45H134N25O25S	485.6562	1.6	34.6	360	38.69	-7.0	even	ok
	345	C52H134N19O27	485.6560	1.1	34.7	361	43.34	-3.0	even	ok
	346	C56H7N26O22P2	485.6562	1.6	34.7	362	38.53	69.0	even	ok
	347	C38H3N44O16P2S2	485.6558	0.7	34.7	363	47.77	62.0	even	ok
	348	C51H124N39O3P2S2	485.6550	-1.0	34.8	364	53.67	12.0	even	ok
	349	C55H31O44P2	485.6549	-1.1	34.9	365	42.84	43.0	even	ok
	350	C64H147NO31P	485.6559	1.0	34.9	366	44.40	-6.0	even	ok
	351	C43H120N45O6P2S	485.6549	-1.1	34.9	367	43.32	9.0	even	ok
	352	C47H26N10NaO40S2	485.6543	-2.3	35.1	368	37.93	41.0	even	ok
	353	C56H131N25O15PS	485.6566	2.4	35.3	369	30.63	6.0	even	ok
	354	C61H18N10NaO32S	485.6564	1.8	35.4	370	42.65	59.0	even	ok
	355	C59H140N15O19P2S	485.6543	-2.4	35.4	371	30.75	0.0	even	ok
	356	C53H7N32O15P2S2	485.6556	0.4	35.4	372	60.60	69.0	even	ok
	357	C56H136N19O20P2	485.6556	0.3	35.5	373	51.17	1.0	even	ok
	358	C54H144N11O28P2	485.6547	-1.6	35.5	374	38.17	-9.0	even	ok
	359	C42H119N43NaO11S	485.6559	0.9	35.5	375	45.18	6.0	even	ok
	360	C52H18N12NaO39	485.6558	0.8	35.5	376	45.74	51.0	even	ok
	361	C53H123N35O9PS	485.6562	1.5	35.6	377	39.11	12.0	even	ok
	362	C50H6N26NaO29	485.6558	0.8	35.7	378	45.63	62.0	even	ok
	363	C55H128N29NaO11PS	485.6563	1.6	35.9	380	36.95	8.0	even	ok
	364	C43H19N22NaO31PS2	485.6549	-1.2	35.9	381	49.98	47.0	even	ok
	365	C62H145N7NaO24P2	485.6566	2.3	35.9	382	30.68	-4.0	even	ok
	366	C52H3N32O20P2	485.6553	-0.2	36.0	383	50.90	70.0	even	ok
	367	C58H30N2O38PS2	485.6546	-1.7	36.0	384	43.68	47.0	even	ok
	368	C51H7N28O24P2	485.6549	-1.2	36.0	385	41.46	65.0	even	ok
	369	C44H9N28O31S	485.6564	2.0	36.1	386	33.44	56.0	even	ok
	370	C46H27N12NaO37PS2	485.6553	-0.2	36.2	387	60.88	41.0	even	ok
	371	C53H143N9NaO33	485.6557	0.4	36.2	388	48.99	-12.0	even	ok
	372	C53H129N33NaO5P2S2	485.6551	-0.8	36.2	389	54.11	8.0	even	ok
	373	C56H3N28NaO21P	485.6549	-1.2	36.2	390	41.01	71.0	even	ok
	374	C63H144N5NaO27P	485.6556	0.3	36.3	391	50.32	-4.0	even	ok
	375	C43H4N38NaO18P2S	485.6548	-1.4	36.5	392	38.45	63.0	even	ok
	376	C60H138N13O24S	485.6561	1.3	36.5	393	48.18	0.0	even	ok
	377	C44H22N18O35PS2	485.6553	-0.4	36.5	394	58.07	45.0	even	ok
	378	C56H18N16O28PS2	485.6546	-1.7	36.5	395	43.05	58.0	even	ok
	379	C61H139N11O25P	485.6555	0.1	36.5	396	51.97	0.0	even	ok
	380	C61H145N9NaO21P2S	485.6544	-2.2	36.5	397	31.57	-4.0	even	ok
	381	C41H14N28O29PS2	485.6548	-1.4	36.6	398	46.89	51.0	even	ok
	382	C40H11N32NaO25PS2	485.6544	-2.1	36.6	399	38.67	53.0	even	ok
	383	C60H140N13O22P2	485.6565	2.1	36.6	400	31.86	0.0	even	ok
	384	C48H29N6O44S2	485.6547	-1.6	36.7	401	44.46	39.0	even	ok

Compound Spectrum SmartFormula Report

Meas. m/z	#	Ion Formula	m/z	err [ppm]	mSigma	# mSigma	Score	rdb	e⁻ Conf	N-Rule
	385	C44H131N29NaO21S	485.6559	0.9	36.8	402	43.64	-5.0	even	ok
	386	C59H152N5O25P2S2	485.6559	0.9	36.8	403	52.07	-11.0	even	ok
	387	C52H16N18NaO30P2	485.6545	-1.9	36.8	404	33.79	56.0	even	ok
	388	C58H27N8O31P2S2	485.6561	1.3	36.8	405	47.39	52.0	even	ok
	389	C48H24N14NaO34P2S	485.6552	-0.5	36.8	406	47.26	46.0	even	ok
	390	C52H120N39NaO5PS	485.6558	0.7	36.8	407	44.97	14.0	even	ok
	391	C57H143N13O22PS2	485.6544	-2.1	36.9	408	38.27	-5.0	even	ok
	392	C44H129N35NaO12P2S	485.6546	-1.8	36.9	409	34.27	0.0	even	ok
	393	C50H34NaO46S2	485.6548	-1.4	37.0	410	46.21	35.0	even	ok
	394	C50H115N45O3PS	485.6557	0.5	37.0	411	46.66	18.0	even	ok
	395	C62H138N9O28	485.6545	-2.0	37.2	412	32.59	0.0	even	ok
	396	C47H30N8O41PS2	485.6557	0.5	37.3	413	55.93	39.0	even	ok
	397	C55H12N26NaO17P2S2	485.6557	0.5	37.3	414	55.41	65.0	even	ok
	398	C54H122N33O12S	485.6552	-0.6	37.3	415	45.58	12.0	even	ok
	399	C54H28N4NaO40P2	485.6545	-1.9	37.3	416	33.38	45.0	even	ok
	400	C49H35N2NaO43PS2	485.6558	0.7	37.4	417	53.55	35.0	even	ok
	401	C54H137N19NaO20P2	485.6548	-1.4	37.4	418	38.07	-2.0	even	ok
	402	C52H127N27NaO19	485.6561	1.3	37.4	419	38.64	4.0	even	ok
	403	C64H143N3NaO30	485.6546	-1.8	37.4	420	34.17	-4.0	even	ok
	404	C54H132N29O9P2S2	485.6554	-0.0	37.4	421	61.17	6.0	even	ok
	405	C38H6N38O23PS2	485.6544	-2.3	37.6	422	35.69	57.0	even	ok
	406	C55H4N30NaO18P2	485.6559	0.9	37.6	423	42.70	71.0	even	ok
	407	C55H153NNaO34P2	485.6543	-2.3	37.7	424	29.60	-18.0	even	ok
	408	C40H107N57NaOS	485.6559	0.8	37.7	425	42.72	17.0	even	ok
	409	C50H13N18O37	485.6558	0.6	37.7	426	45.06	55.0	even	ok
	410	C45H28N14NaO34P2S2	485.6563	1.8	37.8	427	40.24	41.0	even	ok
	411	C40H114N49O9S	485.6558	0.7	37.8	428	44.40	10.0	even	ok
	412	C43H6N32NaO27S	485.6561	1.3	37.8	429	38.65	58.0	even	ok
	413	C62H143N7NaO26S	485.6562	1.4	37.8	430	44.31	-4.0	even	ok
	414	C51H114N43O6S	485.6547	-1.5	37.8	431	36.14	18.0	even	ok
	415	C53H3N34NaO14PS2	485.6543	-2.4	37.9	432	33.81	71.0	even	ok
	416	C57H130N23O18S	485.6556	0.3	38.0	434	47.46	6.0	even	ok
	417	C56H127N27NaO14S	485.6553	-0.4	38.0	435	46.63	8.0	even	ok
	418	C48H26N8NaO43S	485.6565	2.2	38.0	436	36.11	41.0	even	ok
	419	C53H119N37NaO8S	485.6548	-1.3	38.1	437	37.72	14.0	even	ok
	420	C51H131N23NaO23	485.6556	0.4	38.2	438	46.60	-1.0	even	ok
	421	C42H20N24NaO28P2S2	485.6559	0.9	38.2	439	50.07	47.0	even	ok
	422	C59H11N22O21P2S	485.6550	-1.0	38.2	440	48.40	68.0	even	ok
	423	C43H23N20O32P2S2	485.6562	1.6	38.3	441	41.69	45.0	even	ok
	424	C42H117N49NaO2P2S	485.6546	-1.8	38.4	442	32.89	11.0	even	ok
	425	C46H19N20O32P2S	485.6551	-0.7	38.4	443	43.41	50.0	even	ok
	426	C60H136N15NaO21P	485.6551	-0.7	38.5	444	43.60	2.0	even	ok
	427	C51H138N15O31	485.6556	0.2	38.5	445	47.96	-8.0	even	ok
	428	C51H119N41NaO4S2	485.6564	1.9	38.6	446	38.52	14.0	even	ok
	429	C58H23N10NaO30PS2	485.6547	-1.5	38.7	447	42.66	54.0	even	ok
	430	C55H133N23NaO16P2	485.6552	-0.5	38.8	448	45.23	3.0	even	ok

Compound Spectrum SmartFormula Report

Meas. m/z	#	Ion Formula	m/z	err [ppm]	mSigma	# mSigma	Score	rdb	e¯ Conf	N-Rule
	431	C42H126N35O19S	485.6558	0.7	38.9	449	43.08	-1.0	even	ok
	432	C53H141N15NaO24P2	485.6543	-2.3	38.9	450	28.58	-7.0	even	ok
	433	C59H148N7NaO24PS2	485.6545	-1.9	38.9	451	38.16	-9.0	even	ok
	434	C63H146N3O30S	485.6565	2.2	39.0	452	35.34	-6.0	even	ok
	435	C60H32N2NaO33P2S2	485.6562	1.5	39.0	453	42.56	48.0	even	ok
	436	C59H137N17NaO18P2	485.6561	1.4	39.0	454	36.22	2.0	even	ok
	437	C40H15N30O26P2S2	485.6558	0.7	39.1	455	51.05	51.0	even	ok
	438	C56H137N23NaO11P2S2	485.6555	0.1	39.1	456	57.39	2.0	even	ok
	439	C50H11N24O28P2	485.6544	-2.1	39.1	457	30.14	60.0	even	ok
	440	C59H135N17NaO20S	485.6557	0.5	39.1	458	44.21	2.0	even	ok
	441	C58H131N21O19P	485.6550	-0.9	39.1	459	41.02	6.0	even	ok
	442	C46H21N14O41S	485.6564	2.0	39.2	460	36.90	45.0	even	ok
	443	C42H124N41O10P2S	485.6545	-2.0	39.2	461	30.60	4.0	even	ok
	444	C50H111N47NaO2S	485.6544	-2.3	39.3	462	28.54	20.0	even	ok
	445	C56H15N22O21P2S2	485.6561	1.3	39.4	463	44.31	63.0	even	ok
	446	C39H12N34NaO22P2S2	485.6554	-0.0	39.4	464	58.15	53.0	even	ok
	447	C50H122N33O17	485.6560	1.1	39.4	465	38.31	8.0	even	ok
	448	C64H18N8O33P	485.6566	2.3	39.5	467	27.95	62.0	even	ok
	449	C61H16N16NaO23P2S	485.6550	-0.8	39.5	468	48.88	64.0	even	ok
	450	C52H23N10O38P2	485.6544	-2.1	39.5	469	29.89	49.0	even	ok
	451	C52H132N25O18P2	485.6547	-1.6	39.5	470	34.25	2.0	even	ok
	452	C48H106N53S	485.6543	-2.5	39.5	471	26.81	24.0	even	ok
	453	C53H123N31NaO15	485.6565	2.2	39.6	472	28.58	9.0	even	ok
	454	C50H4N32NaO20P2	485.6545	-1.9	39.6	473	31.28	67.0	even	ok
	455	C54H6N30O18PS2	485.6546	-1.7	39.7	475	39.40	69.0	even	ok
	456	C53H150NO41	485.6556	0.2	39.8	476	46.29	-19.0	even	ok
	457	C61H10N18O27P	485.6561	1.4	39.8	477	35.46	68.0	even	ok
	458	C45H16N24NaO28P2S	485.6548	-1.4	40.1	478	34.98	52.0	even	ok
	459	C57H132N23O16P2	485.6560	1.2	40.1	479	36.92	6.0	even	ok
	460	C63H15N12NaO29P	485.6562	1.6	40.1	480	33.52	64.0	even	ok
	461	C56H3N32O15P2S	485.6545	-2.0	40.2	481	30.22	74.0	even	ok
	462	C50H36NaO44P2S	485.6552	-0.5	40.3	482	51.68	35.0	even	ok
	463	C62H19N12O27P2S	485.6554	-0.1	40.4	483	55.63	62.0	even	ok
	464	C49H126N29O21	485.6556	0.2	40.4	484	45.55	3.0	even	ok
	465	C53H128N29O14P2	485.6551	-0.6	40.6	485	41.37	7.0	even	ok
	466	C49H10N22NaO33	485.6554	-0.1	40.6	486	46.06	57.0	even	ok
	467	C37H7N40O20P2S2	485.6553	-0.2	40.6	487	54.21	57.0	even	ok
	468	C49H114N47O2S2	485.6563	1.7	40.6	488	31.98	18.0	even	ok
	469	C52H25N4O47	485.6558	0.6	40.7	490	41.53	44.0	even	ok
	470	C40H112N55P2S	485.6545	-2.0	40.7	491	29.31	15.0	even	ok
	471	C57H140N19O15P2S2	485.6559	0.9	40.7	492	46.89	0.0	even	ok
	472	C52H144N11NaO30P	485.6566	2.5	40.8	493	25.87	-12.0	even	ok
	473	C39H111N53NaO5S	485.6554	-0.1	41.0	494	46.00	12.0	even	ok
	474	C60H7N22NaO23P	485.6558	0.7	41.2	495	40.58	70.0	even	ok
	475	C51H118N37O13	485.6564	2.0	41.2	496	28.82	13.0	even	ok
	476	C58H8N26NaO17P2S	485.6546	-1.8	41.2	497	30.93	70.0	even	ok

Compound Spectrum SmartFormula Report

Meas. m/z	#	Ion Formula	m/z	err [ppm]	mSigma	# mSigma	Score	rdb	e¯ Conf	N-Rule
	477	C60H151N3O28PS2	485.6549	-1.2	41.4	499	42.98	-11.0	even	ok
	478	C55H131N27O12PS2	485.6544	-2.1	41.4	500	33.76	6.0	even	ok
	479	C58H20N16NaO23P2S2	485.6562	1.5	41.4	501	39.92	59.0	even	ok
	480	C41H123N39NaO15S	485.6554	-0.1	41.4	502	45.53	1.0	even	ok
	481	C59H26N6O34PS2	485.6551	-0.8	41.5	503	47.07	52.0	even	ok
	482	C48H31N6O42P2S	485.6551	-0.7	41.5	504	48.00	39.0	even	ok
	483	C62H9N16O30	485.6551	-0.7	41.6	505	40.04	68.0	even	ok
	484	C57H128N25NaO15P	485.6547	-1.6	41.7	506	32.06	8.0	even	ok
	485	C50H135N19NaO27	485.6552	-0.5	41.7	507	41.12	-6.0	even	ok
	486	C56H11N24NaO20PS2	485.6547	-1.5	41.7	508	39.19	65.0	even	ok
	487	C43H11N30O26P2S	485.6547	-1.6	41.9	509	31.64	56.0	even	ok
	488	C59H6N24NaO22S	485.6563	1.8	41.9	510	35.79	70.0	even	ok
	489	C64H24N6NaO29P2S	485.6555	0.1	42.0	511	53.62	58.0	even	ok
	490	C65H17N6O36	485.6556	0.3	42.0	512	43.07	62.0	even	ok
	491	C64H14N10NaO32	485.6552	-0.5	42.2	513	41.01	64.0	even	ok
	492	C52H147N5NaO37	485.6552	-0.5	42.3	515	40.50	-17.0	even	ok
	493	C61H6N20NaO26	485.6548	-1.4	42.4	516	32.96	70.0	even	ok
	494	C50H132N25NaO20P	485.6566	2.4	42.5	517	24.74	-1.0	even	ok
	495	C59H145N13NaO17P2S2	485.6560	1.1	42.6	518	42.53	-4.0	even	ok
	496	C55H123N31O13P	485.6546	-1.8	42.7	519	29.63	12.0	even	ok
	497	C51H22N8NaO43	485.6554	-0.1	42.8	520	43.37	46.0	even	ok
	498	C47H5N28O31	485.6553	-0.3	42.8	522	41.57	61.0	even	ok
	499	C56H129N27NaO12P2	485.6557	0.5	42.9	523	40.22	8.0	even	ok
	500	C67H22NaO38	485.6557	0.5	43.1	524	40.19	58.0	even	ok
500.3316	1	C18H51N3O10P	500.3307	-1.8	64.4	1	100.00	-4.0	even	ok
	2	C14H42N15O3S	500.3310	-1.0	72.2	2	91.04	2.0	even	ok
	3	C15H43N13O4P	500.3293	-4.5	73.4	3	21.58	2.0	even	ok
	4	C16H47N9NaO5S	500.3313	-0.5	73.6	4	98.01	-2.0	even	ok
	5	C17H48N7NaO6P	500.3296	-3.9	74.4	5	25.84	-2.0	even	ok
	6	C17H50N5O9S	500.3324	1.6	74.7	6	70.75	-4.0	even	ok
	7	C21H52NNaO8P	500.3323	1.4	76.4	7	69.79	-3.0	even	ok
	8	C20H46N5O9	500.3290	-5.1	78.5	8	18.24	1.0	even	ok
	9	C20H56NO6P2S	500.3298	-3.5	81.8	9	30.13	-5.0	even	ok
	10	C17H43N13NaOS	500.3326	2.2	83.7	10	42.45	3.0	even	ok
	11	C18H46N9O5S	500.3337	4.3	84.4	11	19.68	1.0	even	ok
	12	C20H51N3NaO7S	500.3340	4.9	85.9	12	14.70	-3.0	even	ok
	13	C16H39N17P	500.3306	-1.8	86.6	13	31.35	7.0	even	ok
	14	C19H47N7O6P	500.3320	0.9	87.1	14	38.78	1.0	even	ok
	15	C18H44N11NaO2P	500.3309	-1.2	87.5	15	34.97	3.0	even	ok
	16	C23H47N3NaO7	500.3306	-1.9	90.6	16	34.59	2.0	even	ok
	17	C21H52N5O2P2S	500.3311	-0.8	91.9	17	42.69	0.0	even	ok
	18	C18H52N7NaOPS2	500.3305	-2.2	93.9	18	27.19	-3.0	even	ok
	19	C19H55N3O5PS2	500.3315	-0.0	95.6	19	42.75	-5.0	even	ok
	20	C21H50N5O4S2	500.3299	-3.4	97.0	20	20.25	0.0	even	ok
500.6659	1	C24H3N53NaO24P2	500.6667	1.5	19.2	1	83.54	52.0	even	ok
	2	C23H7N49NaO28P2	500.6662	0.6	24.0	4	93.46	47.0	even	ok

Compound Spectrum SmartFormula Report

Meas. m/z	#	Ion Formula	m/z	err [ppm]	mSigma	# mSigma	Score	rdb	e¯ Conf	N-Rule
	3	C24H6N47NaO31P	500.6652	-1.4	25.8	6	73.60	47.0	even	ok
	4	C26H5N47O31P	500.6660	0.2	26.4	7	96.21	50.0	even	ok
	5	C24H10N45O32P2	500.6666	1.3	27.3	8	73.11	45.0	even	ok
	6	C28H10N41NaO33P	500.6661	0.4	28.5	10	87.96	46.0	even	ok
	7	C26H15N39NaO34P2	500.6667	1.5	28.7	11	67.32	41.0	even	ok
	8	C19H3N55NaO26P2	500.6653	-1.2	28.8	12	72.26	48.0	even	ok
	9	C25H9N43O35P	500.6656	-0.7	29.3	13	80.53	45.0	even	ok
	10	C29H6N45NaO29P	500.6666	1.3	30.0	14	68.94	51.0	even	ok
	11	C27H4N45O34	500.6650	-1.8	30.2	15	59.83	50.0	even	ok
	12	C27H14N37NaO37P	500.6657	-0.5	31.0	16	80.60	41.0	even	ok
	13	C20H6N51O30P2	500.6657	-0.5	31.1	17	80.73	46.0	even	ok
	14	C22H11N45NaO32P2	500.6658	-0.3	31.8	18	82.66	42.0	even	ok
	15	C29H13N37O37P	500.6665	1.1	32.3	20	68.15	44.0	even	ok
	16	C29H9N39NaO36	500.6651	-1.6	32.4	21	59.61	46.0	even	ok
	17	C30H9N41O33P	500.6669	2.0	33.5	22	52.26	49.0	even	ok
	18	C31H18N31NaO39P	500.6666	1.3	34.4	23	61.61	40.0	even	ok
	19	C30H5N43NaO32	500.6656	-0.7	34.5	24	70.45	51.0	even	ok
	20	C23H14N41O36P2	500.6661	0.4	34.5	25	75.47	40.0	even	ok
	21	C28H17N33O41P	500.6660	0.2	34.6	26	78.53	39.0	even	ok
	22	C24H13N39O39P	500.6651	-1.6	35.4	27	55.47	40.0	even	ok
	23	C25H19N35NaO38P2	500.6662	0.6	35.5	28	70.66	36.0	even	ok
	24	C30H12N35O40	500.6655	-0.9	36.1	29	64.86	44.0	even	ok
	25	C30H22N27NaO43P	500.6661	0.4	36.4	30	72.06	35.0	even	ok
	26	C26H18N33NaO41P	500.6652	-1.4	36.6	31	56.44	36.0	even	ok
	27	C29H16N31O44	500.6650	-1.8	37.5	32	49.76	39.0	even	ok
	28	C31H8N39O36	500.6659	-0.0	37.9	33	75.06	49.0	even	ok
	29	C32H21N27O43P	500.6669	2.0	38.2	35	46.12	38.0	even	ok
	30	C32H17N29NaO42	500.6656	-0.7	38.3	36	63.87	40.0	even	ok
	31	C19H5N49NaO35	500.6666	1.4	38.5	37	53.67	43.0	even	ok
	32	C26H22N31O42P2	500.6666	1.3	38.5	38	55.03	34.0	even	ok
	33	C18H7N51NaO30P2	500.6649	-2.1	38.6	39	44.31	43.0	even	ok
	34	C17H3N59NaO22P2S	500.6669	1.9	39.2	40	45.58	48.0	even	ok
	35	C31H21N25NaO46	500.6651	-1.6	39.4	41	49.71	35.0	even	ok
	36	C28H27N25NaO44P2	500.6667	1.5	39.7	43	50.78	30.0	even	ok
	37	C27H21N29O45P	500.6656	-0.7	39.9	44	61.49	34.0	even	ok
	38	C31H25N23O47P	500.6665	1.1	40.1	46	55.43	33.0	even	ok
	39	C33H13N33NaO38	500.6660	0.2	40.2	47	68.04	45.0	even	ok
	40	C19H10N47O34P2	500.6652	-1.4	40.5	48	51.08	41.0	even	ok
	41	C21H15N41NaO36P2	500.6653	-1.2	40.9	49	53.02	37.0	even	ok
	42	C20H8N45O39	500.6670	2.1	40.9	50	41.32	41.0	even	ok
	43	C34H14N35O33P2	500.6651	-1.7	41.0	52	46.06	48.0	even	ok
	44	C29H26N23NaO47P	500.6657	-0.5	41.3	53	61.71	30.0	even	ok
	45	C22H6N51NaO27PS	500.6668	1.7	41.9	54	44.81	47.0	even	ok
	46	C33H30N17NaO49P	500.6666	1.3	42.0	55	50.24	29.0	even	ok
	47	C33H20N25O46	500.6659	0.0	42.0	56	67.17	38.0	even	ok
	48	C32H4N43O32	500.6664	0.9	42.6	57	54.58	54.0	even	ok

Compound Spectrum SmartFormula Report

Meas. m/z	#	Ion Formula	m/z	err [ppm]	mSigma	# mSigma	Score	rdb	e¯ Conf	N-Rule
	49	C36H26N21O43P2	500.6651	-1.7	42.9	59	43.79	37.0	even	ok
	50	C32H24N21O50	500.6655	-0.9	43.1	62	53.63	33.0	even	ok
	51	C22H18N37O40P2	500.6657	-0.5	43.2	63	58.70	35.0	even	ok
	52	C36H19N29NaO35P2	500.6652	-1.5	43.4	65	45.38	44.0	even	ok
	53	C34H16N29O42	500.6664	0.9	43.7	66	52.74	43.0	even	ok
	54	C24H23N31NaO42P2	500.6658	-0.3	43.8	67	60.03	31.0	even	ok
	55	C35H25N19NaO48	500.6660	0.2	44.2	68	60.73	34.0	even	ok
	56	C26H11N45NaO27P2S	500.6649	-2.0	44.3	69	39.16	46.0	even	ok
	57	C19H5N53O29PS	500.6663	0.7	44.3	70	54.71	46.0	even	ok
	58	C30H29N19O51P	500.6660	0.2	44.7	72	59.65	28.0	even	ok
	59	C34H7N43NaO25P2	500.6652	-1.5	44.8	73	43.53	55.0	even	ok
	60	C34H9N37NaO34	500.6665	1.1	44.9	74	48.88	50.0	even	ok
	61	C23H5N49NaO30S	500.6658	-0.3	45.1	75	58.33	47.0	even	ok
	62	C34H29N15NaO52	500.6656	-0.7	45.1	76	52.92	29.0	even	ok
	63	C38H31N15NaO45P2	500.6652	-1.5	45.2	77	43.07	33.0	even	ok
	64	C27H7N49NaO23P2S	500.6654	-1.1	45.6	78	47.65	51.0	even	ok
	65	C34H33N13O53P	500.6669	2.0	45.7	79	37.31	27.0	even	ok
	66	C21H10N47NaO31PS	500.6663	0.8	45.9	80	50.11	42.0	even	ok
	67	C36H21N23NaO44	500.6665	1.1	46.0	81	47.14	39.0	even	ok
	68	C32H34N13NaO53P	500.6661	0.4	46.2	84	54.86	24.0	even	ok
	69	C25H26N27O46P2	500.6661	0.4	46.5	85	54.26	29.0	even	ok
	70	C37H22N25O39P2	500.6655	-0.8	46.5	87	49.41	42.0	even	ok
	71	C20H4N51O32S	500.6653	-1.3	46.6	88	43.48	46.0	even	ok
	72	C16H7N55NaO26P2S	500.6664	1.0	46.7	89	46.63	43.0	even	ok
	73	C31H28N17O54	500.6650	-1.8	46.8	90	38.37	28.0	even	ok
	74	C26H25N25O49P	500.6651	-1.6	47.1	91	40.23	29.0	even	ok
	75	C27H31N21NaO48P2	500.6662	0.6	47.3	92	50.80	25.0	even	ok
	76	C35H10N39O29P2	500.6655	-0.8	47.3	93	48.23	53.0	even	ok
	77	C17H6N53NaO29PS	500.6655	-0.9	47.6	95	46.46	43.0	even	ok
	78	C16H4N51O37	500.6661	0.3	47.8	96	53.05	42.0	even	ok
	79	C38H38N7O53P2	500.6651	-1.7	47.9	97	38.01	26.0	even	ok
	80	C35H12N33O38	500.6668	1.8	47.9	98	37.12	48.0	even	ok
	81	C36H28N15O52	500.6664	0.9	48.0	99	46.51	32.0	even	ok
	82	C27H14N41O31P2S	500.6653	-1.3	48.0	100	42.49	44.0	even	ok
	83	C18H9N45NaO39	500.6662	0.5	48.1	101	50.62	38.0	even	ok
	84	C28H30N19NaO51P	500.6652	-1.4	48.1	102	40.97	25.0	even	ok
	85	C25H4N49O30S	500.6666	1.3	48.2	103	41.44	50.0	even	ok
	86	C22H9N45NaO34S	500.6653	-1.2	48.3	104	43.34	42.0	even	ok
	87	C33H33N11NaO56	500.6651	-1.6	48.5	105	38.41	24.0	even	ok
	88	C39H34N11O49P2	500.6655	-0.8	48.8	106	46.23	31.0	even	ok
	89	C24H8N45O34S	500.6662	0.4	48.8	107	50.16	45.0	even	ok
	90	C35H32N11O56	500.6659	0.0	48.8	108	55.01	27.0	even	ok
	91	C39H27N19NaO41P2	500.6656	-0.6	48.9	109	48.09	38.0	even	ok
	92	C28H10N45O27P2S	500.6657	-0.4	49.2	110	50.47	49.0	even	ok
	93	C22H13N43O35PS	500.6667	1.6	49.4	112	37.77	40.0	even	ok
	94	C28H3N53NaO19P2S	500.6658	-0.2	49.5	113	51.99	56.0	even	ok

Compound Spectrum SmartFormula Report

Meas. m/z	#	Ion Formula	m/z	err [ppm]	mSigma	# mSigma	Score	rdb	e⁻ Conf	N-Rule
	95	C37H15N33NaO31P2	500.6656	-0.6	49.5	114	47.10	49.0	even	ok
	96	C37H24N19O48	500.6668	1.8	49.6	115	35.27	37.0	even	ok
	97	C17H10N51O30P2S	500.6668	1.8	49.6	116	35.56	41.0	even	ok
	98	C33H37N9O57P	500.6665	1.1	49.7	117	41.94	22.0	even	ok
	99	C40H43NNaO55P2	500.6652	-1.5	50.0	118	37.41	22.0	even	ok
	100	C19H12N41O43	500.6665	1.2	50.2	119	40.16	36.0	even	ok
	101	C28H34N17O52P2	500.6666	1.3	50.2	120	39.29	23.0	even	ok
	102	C38H33N9NaO54	500.6665	1.1	50.2	121	41.58	28.0	even	ok
	103	C29H19N35NaO33P2S	500.6654	-1.1	50.2	122	41.64	40.0	even	ok
	104	C37H17N27NaO40	500.6669	2.0	50.3	123	32.84	44.0	even	ok
	105	C27H9N43NaO32S	500.6667	1.5	50.4	124	36.89	46.0	even	ok
	106	C20H19N37NaO40P2	500.6649	-2.1	50.6	125	31.43	32.0	even	ok
	107	C21H17N35NaO45	500.6666	1.4	50.6	126	37.78	32.0	even	ok
	108	C19H15N45NaO32P2S	500.6669	1.9	50.7	127	32.68	37.0	even	ok
	109	C18H9N49O33PS	500.6658	-0.2	50.7	128	49.61	41.0	even	ok
	110	C37H37N5NaO58	500.6660	0.2	50.9	129	49.81	23.0	even	ok
	111	C26H13N39NaO36S	500.6662	0.6	50.9	130	45.23	41.0	even	ok
	112	C24H18N37NaO37PS	500.6668	1.7	51.1	131	34.16	36.0	even	ok
	113	C41H39N5NaO51P2	500.6656	-0.6	51.2	132	44.95	27.0	even	ok
	114	C29H33N15O55P	500.6656	-0.7	51.2	133	44.36	23.0	even	ok
	115	C30H39N11NaO54P2	500.6667	1.5	51.2	134	36.28	19.0	even	ok
	116	C28H23N31NaO37P2S	500.6649	-2.0	51.3	135	31.84	35.0	even	ok
	117	C35H42N3NaO59P	500.6666	1.3	51.3	136	38.09	18.0	even	ok
	118	C35H5N41NaO30	500.6669	2.0	51.5	137	31.72	55.0	even	ok
	119	C30H15N39NaO29P2S	500.6658	-0.2	51.5	138	48.88	45.0	even	ok
	120	C23H12N41O38S	500.6657	-0.4	51.9	139	45.80	40.0	even	ok
	121	C39H29N13NaO50	500.6669	2.0	51.9	140	31.16	33.0	even	ok
	122	C20H14N43NaO35PS	500.6659	-0.1	51.9	141	49.63	37.0	even	ok
	123	C34H36N7O60	500.6655	-0.9	52.1	142	41.16	22.0	even	ok
	124	C40H30N15O45P2	500.6660	0.1	52.2	143	48.95	36.0	even	ok
	125	C38H18N29O35P2	500.6660	0.1	52.3	144	48.84	47.0	even	ok
	126	C31H38N9NaO57P	500.6657	-0.5	52.4	145	44.56	19.0	even	ok
	127	C21H22N33O44P2	500.6652	-1.4	52.6	146	35.84	30.0	even	ok
	128	C12H3N61NaO24P2S	500.6656	-0.7	52.7	147	41.74	44.0	even	ok
	129	C29H6N49O23P2S	500.6662	0.5	52.7	148	43.79	54.0	even	ok
	130	C35H3N47NaO21P2	500.6656	-0.6	53.0	149	42.30	60.0	even	ok
	131	C23H27N27NaO46P2	500.6653	-1.2	53.0	150	37.11	26.0	even	ok
	132	C21H13N41NaO38S	500.6649	-2.0	53.5	151	29.06	37.0	even	ok
	133	C25H17N35NaO40S	500.6658	-0.3	53.6	152	45.09	36.0	even	ok
	134	C36H41NNaO62	500.6656	-0.7	53.8	153	40.69	18.0	even	ok
	135	C39H36N5O58	500.6668	1.8	53.9	154	30.76	26.0	even	ok
	136	C30H22N31O37P2S	500.6657	-0.4	54.0	155	43.62	38.0	even	ok
	137	C42H35N9NaO47P2	500.6661	0.3	54.5	157	43.72	32.0	even	ok
	138	C40H23N23NaO37P2	500.6661	0.3	54.6	158	43.73	43.0	even	ok
	139	C38H40NO62	500.6664	0.9	54.6	159	37.81	21.0	even	ok
	140	C27H16N35O40S	500.6666	1.3	54.6	160	33.87	39.0	even	ok

Compound Spectrum SmartFormula Report

Meas. m/z	#	Ion Formula	m/z	err [ppm]	mSigma	# mSigma	Score	rdb	e¯ Conf	N-Rule
	141	C13H6N57O28P2S	500.6659	-0.0	54.6	161	45.69	42.0	even	ok
	142	C42H42NO55P2	500.6660	0.1	54.8	162	45.03	25.0	even	ok
	143	C14H5N55O31PS	500.6649	-2.0	54.8	163	28.04	42.0	even	ok
	144	C29H26N27O41P2S	500.6653	-1.3	55.0	164	34.24	33.0	even	ok
	145	C36H6N43O25P2	500.6660	0.1	55.1	165	44.76	58.0	even	ok
	146	C31H11N43NaO25P2S	500.6663	0.7	55.1	166	38.96	50.0	even	ok
	147	C31H18N35O33P2S	500.6662	0.5	55.1	167	40.52	43.0	even	ok
	148	C21H17N39O39PS	500.6663	0.7	55.2	168	39.31	35.0	even	ok
	149	C15H11N51NaO30P2S	500.6660	0.2	55.3	169	43.73	38.0	even	ok
	150	C24H30N23O50P2	500.6657	-0.5	55.4	170	40.69	24.0	even	ok
	151	C16H10N49NaO33PS	500.6650	-1.8	55.6	171	28.82	38.0	even	ok
	152	C32H41N5O61P	500.6660	0.2	55.6	172	42.68	17.0	even	ok
	153	C21H3N59NaO17P2S2	500.6661	0.3	55.7	173	42.08	52.0	even	ok
	154	C26H35N17NaO52P2	500.6658	-0.3	55.9	174	41.55	20.0	even	ok
	155	C32H27N25NaO39P2S	500.6658	-0.2	56.2	175	42.26	34.0	even	ok
	156	C14H5N51NaO37	500.6653	-1.3	56.3	176	32.66	39.0	even	ok
	157	C20H7N55NaO21P2S2	500.6656	-0.6	56.4	177	38.10	47.0	even	ok
	158	C23H22N33NaO41PS	500.6663	0.8	56.5	179	36.05	31.0	even	ok
	159	C29H21N29NaO42S	500.6667	1.5	56.7	180	30.19	35.0	even	ok
	160	C41H29N13O48P	500.6650	-1.9	56.8	181	27.09	36.0	even	ok
	161	C22H16N37O42S	500.6653	-1.3	56.9	182	31.62	35.0	even	ok
	162	C31H31N21NaO43P2S	500.6654	-1.1	57.0	183	33.60	29.0	even	ok
	163	C39H17N27O38P	500.6650	-1.9	57.1	184	26.85	47.0	even	ok
	164	C38H11N37NaO27P2	500.6661	0.3	57.2	185	40.24	54.0	even	ok
	165	C33H40N3O64	500.6650	-1.8	57.3	186	27.77	17.0	even	ok
	166	C26H20N31O44S	500.6662	0.5	57.3	187	38.38	34.0	even	ok
	167	C30H5N47O26PS	500.6652	-1.5	57.4	188	29.98	54.0	even	ok
	168	C17H6N57O23P2S2	500.6651	-1.7	57.4	189	28.16	46.0	even	ok
	169	C33H23N29NaO35P2S	500.6663	0.7	57.5	190	36.02	39.0	even	ok
	170	C41H26N19O41P2	500.6664	1.0	57.5	191	33.90	41.0	even	ok
	171	C13H6N53NaO34P	500.6663	0.7	57.8	192	35.59	39.0	even	ok
	172	C16H14N47O34P2S	500.6664	0.9	57.9	193	34.33	36.0	even	ok
	173	C43H38N5O51P2	500.6664	1.0	57.9	194	33.40	30.0	even	ok
	174	C15H8N47O41	500.6656	-0.6	57.9	195	36.71	37.0	even	ok
	175	C17H13N41NaO43	500.6657	-0.4	58.0	196	38.13	33.0	even	ok
	176	C24H21N31NaO44S	500.6654	-1.1	58.4	200	31.56	31.0	even	ok
	177	C17H13N45O37PS	500.6654	-1.1	58.4	201	31.69	36.0	even	ok
	178	C32H14N39O29P2S	500.6666	1.4	58.4	202	29.38	48.0	even	ok
	179	C27H38N13O56P2	500.6661	0.4	58.5	203	37.13	18.0	even	ok
	180	C18H19N41NaO36P2S	500.6665	1.0	58.7	204	31.98	32.0	even	ok
	181	C28H37N11O59P	500.6651	-1.6	59.0	205	27.72	18.0	even	ok
	182	C16H14N43NaO40P	500.6667	1.6	59.1	207	27.28	33.0	even	ok
	183	C28H25N25NaO46S	500.6662	0.6	59.1	208	34.66	30.0	even	ok
	184	C19H11N51NaO25P2S2	500.6652	-1.5	59.2	209	27.97	42.0	even	ok
	185	C14H9N49O38P	500.6666	1.4	59.2	210	28.59	37.0	even	ok
	186	C43H34N7NaO50P	500.6651	-1.7	59.2	211	26.38	32.0	even	ok

Compound Spectrum SmartFormula Report

Meas. m/z	#	Ion Formula	m/z	err [ppm]	mSigma	# mSigma	Score	rdb	e¯ Conf	N-Rule
	187	C29H43N7NaO58P2	500.6662	0.6	59.3	212	34.73	14.0	even	ok
	188	C19H18N39NaO39PS	500.6655	-0.9	59.3	213	32.12	32.0	even	ok
	189	C22H6N55O21P2S2	500.6664	1.0	59.3	214	31.83	50.0	even	ok
	190	C41H22N21NaO40P	500.6651	-1.7	59.3	215	26.20	43.0	even	ok
	191	C32H17N33O36PS	500.6652	-1.5	59.5	216	28.01	43.0	even	ok
	192	C39H14N33O31P2	500.6664	1.0	59.6	217	31.69	52.0	even	ok
	193	C37H5N41O28P	500.6650	-1.9	59.7	219	24.51	58.0	even	ok
	194	C32H10N41NaO28PS	500.6653	-1.3	59.8	220	29.08	50.0	even	ok
	195	C43H31N13NaO43P2	500.6665	1.2	59.8	221	29.98	37.0	even	ok
	196	C24H25N29O45PS	500.6667	1.6	59.9	222	26.99	29.0	even	ok
	197	C30H42N5NaO61P	500.6652	-1.4	59.9	223	28.22	14.0	even	ok
	198	C18H16N37O47	500.6661	0.3	59.9	224	36.11	31.0	even	ok
	199	C33H30N21O43P2S	500.6662	0.5	59.9	225	34.56	32.0	even	ok
	200	C30H35N17NaO47P2S	500.6649	-2.0	60.0	226	24.10	24.0	even	ok
	201	C21H10N51O25P2S2	500.6660	0.1	60.0	227	37.88	45.0	even	ok
	202	C20H21N31NaO49	500.6662	0.5	60.2	228	34.36	27.0	even	ok
	203	C32H7N47NaO21P2S	500.6667	1.6	60.5	229	26.13	55.0	even	ok
	204	C34H19N33NaO31P2S	500.6667	1.6	60.8	231	25.83	44.0	even	ok
	205	C32H34N17O47P2S	500.6657	-0.4	60.8	232	34.89	27.0	even	ok
	206	C34H26N25O39P2S	500.6666	1.4	61.1	234	26.82	37.0	even	ok
	207	C26H30N23NaO47PS	500.6668	1.7	61.3	235	24.43	25.0	even	ok
	208	C19H22N37O40P2S	500.6668	1.8	61.5	236	24.19	30.0	even	ok
	209	C24H11N49NaO23P2S2	500.6665	1.2	61.5	237	28.27	46.0	even	ok
	210	C41H19N27NaO33P2	500.6665	1.1	61.7	239	28.12	48.0	even	ok
	211	C25H24N27O48S	500.6657	-0.4	61.8	240	33.12	29.0	even	ok
	212	C34H22N27NaO38PS	500.6653	-1.3	61.9	241	27.14	39.0	even	ok
	213	C39H10N35NaO30P	500.6651	-1.7	61.9	242	24.01	54.0	even	ok
	214	C18H4N55O28S2	500.6668	1.8	61.9	243	23.61	46.0	even	ok
	215	C11H7N57NaO28P2S	500.6651	-1.6	61.9	244	24.66	39.0	even	ok
	216	C23H15N45NaO27P2S2	500.6661	0.3	62.0	246	34.06	41.0	even	ok
	217	C30H46N3O62P2	500.6666	1.3	62.1	247	26.65	12.0	even	ok
	218	C12H5N55NaO33S	500.6669	1.9	62.1	248	23.04	39.0	even	ok
	219	C35H35N15NaO45P2S	500.6663	0.7	62.1	249	30.73	28.0	even	ok
	220	C42H25N17O44P	500.6654	-1.0	62.3	250	28.49	41.0	even	ok
	221	C20H21N35O43PS	500.6658	-0.2	62.3	251	34.04	30.0	even	ok
	222	C21H24N27O53	500.6665	1.2	62.4	252	26.97	25.0	even	ok
	223	C21H27N31NaO42P2S	500.6669	1.9	62.4	253	22.23	26.0	even	ok
	224	C16H5N55NaO28S2	500.6660	0.2	62.5	254	34.09	43.0	even	ok
	225	C44H37N3O54P	500.6654	-1.0	62.5	255	28.28	30.0	even	ok
	226	C22H31N23NaO50P2	500.6649	-2.1	62.7	257	21.21	21.0	even	ok
	227	C20H14N47O29P2S2	500.6655	-0.8	62.7	258	29.58	40.0	even	ok
	228	C37H47NNaO55P2S	500.6663	0.7	62.8	259	36.03	17.0	even	ok
	229	C29H28N21O50S	500.6666	1.4	62.8	260	25.75	28.0	even	ok
	230	C23H29N21NaO55	500.6666	1.4	62.8	261	25.31	21.0	even	ok
	231	C34H39N11NaO49P2S	500.6658	-0.2	62.8	262	33.84	23.0	even	ok
	232	C31H45NO65P	500.6656	-0.7	62.8	263	30.32	12.0	even	ok

Compound Spectrum SmartFormula Report

Meas. m/z	#	Ion Formula	m/z	err [ppm]	mSigma	# mSigma	Score	rdb	e⁻ Conf	N-Rule
	233	C44H34N9O47P2	500.6669	1.9	62.9	264	22.40	35.0	even	ok
	234	C25H7N53NaO19P2S2	500.6670	2.1	63.0	265	21.16	51.0	even	ok
	235	C33H13N37O32PS	500.6656	-0.6	63.1	267	30.75	48.0	even	ok
	236	C34H46N3O57P2S	500.6658	-0.3	63.2	268	38.61	16.0	even	ok
	237	C23H5N53O24PS2	500.6654	-1.0	63.2	269	27.65	50.0	even	ok
	238	C22H9N49O28PS2	500.6650	-1.9	63.4	270	21.78	45.0	even	ok
	239	C22H26N29NaO45PS	500.6659	-0.0	63.4	271	34.06	26.0	even	ok
	240	C27H29N21NaO50S	500.6658	-0.2	63.4	272	32.64	25.0	even	ok
	241	C36H31N19NaO41P2S	500.6667	1.6	63.4	273	23.56	33.0	even	ok
	242	C31H38N13O51P2S	500.6653	-1.2	63.6	274	25.72	22.0	even	ok
	243	C20H9N49NaO30S2	500.6669	2.0	63.7	275	21.03	42.0	even	ok
	244	C37H37N9O52PS	500.6656	-0.6	63.8	277	36.06	26.0	even	ok
	245	C34H29N19O46PS	500.6652	-1.5	63.9	278	24.17	32.0	even	ok
	246	C12H10N53O32P2S	500.6655	-0.9	64.0	280	27.42	37.0	even	ok
	247	C36H41N5O56PS	500.6652	-1.4	64.0	281	28.87	21.0	even	ok
	248	C40H13N31O34P	500.6654	-1.0	64.3	282	26.51	52.0	even	ok
	249	C42H22N23O37P2	500.6669	1.9	64.3	283	21.32	46.0	even	ok
	250	C14H15N47NaO34P2S	500.6656	-0.7	64.5	284	28.20	33.0	even	ok
	251	C44H30N11NaO46P	500.6655	-0.8	64.6	285	27.45	37.0	even	ok
	252	C23H25N27NaO48S	500.6649	-2.0	64.6	286	20.07	26.0	even	ok
	253	C31H33N15NaO52S	500.6667	1.5	64.7	287	22.98	24.0	even	ok
	254	C23H34N19O54P2	500.6652	-1.4	64.8	288	23.92	19.0	even	ok
	255	C46H39N3NaO49P2	500.6670	2.1	65.2	290	19.56	31.0	even	ok
	256	C25H39N13NaO56P2	500.6653	-1.2	65.2	291	24.71	15.0	even	ok
	257	C38H33N13O48PS	500.6661	0.3	65.3	292	36.03	31.0	even	ok
	258	C33H6N45NaO24PS	500.6657	-0.4	65.3	293	29.58	55.0	even	ok
	259	C22H19N41NaO31P2S2	500.6656	-0.6	65.3	294	28.16	36.0	even	ok
	260	C25H10N47NaO26PS2	500.6655	-0.8	65.4	295	26.75	46.0	even	ok
	261	C35H25N23O42PS	500.6656	-0.6	65.4	296	28.35	37.0	even	ok
	262	C33H43N7NaO53P2S	500.6654	-1.1	65.4	297	25.26	18.0	even	ok
	263	C35H18N31NaO34PS	500.6657	-0.4	65.5	298	29.47	44.0	even	ok
	264	C12H5N59O27PS2	500.6665	1.1	65.6	299	24.84	42.0	even	ok
	265	C24H21N35O38PS2	500.6650	-1.9	65.8	300	24.03	34.0	even	ok
	266	C39H7N41NaO23P2	500.6665	1.1	65.8	301	24.41	59.0	even	ok
	267	C36H38N11O49P2S	500.6666	1.4	65.9	302	22.65	26.0	even	ok
	268	C39H42N3NaO54PS	500.6657	-0.4	65.9	303	34.94	22.0	even	ok
	269	C17H8N51O32S2	500.6664	0.9	65.9	304	25.80	41.0	even	ok
	270	C31H40N7O60S	500.6666	1.4	65.9	305	27.62	17.0	even	ok
	271	C27H22N33NaO36PS2	500.6655	-0.8	66.1	306	31.40	35.0	even	ok
	272	C25H14N45O27P2S2	500.6669	1.9	66.1	307	19.95	44.0	even	ok
	273	C36H34N13NaO48PS	500.6653	-1.3	66.1	308	23.42	28.0	even	ok
	274	C28H18N37NaO32PS2	500.6660	0.1	66.1	309	36.89	40.0	even	ok
	275	C27H26N31O37P2S2	500.6669	1.9	66.2	310	23.76	33.0	even	ok
	276	C24H14N43NaO30PS2	500.6651	-1.7	66.4	311	20.61	41.0	even	ok
	277	C23H29N25O49PS	500.6663	0.7	66.5	312	26.70	24.0	even	ok
	278	C42H18N25NaO36P	500.6655	-0.8	66.5	313	25.62	48.0	even	ok

Compound Spectrum SmartFormula Report

Meas. m/z	#	Ion Formula	m/z	err [ppm]	mSigma	# mSigma	Score	rdb	e¯ Conf	N-Rule
	279	C44H27N17NaO39P2	500.6670	2.0	66.6	314	18.67	42.0	even	ok
	280	C35H42N7O53P2S	500.6662	0.5	66.6	315	27.35	21.0	even	ok
	281	C24H18N41O31P2S2	500.6664	1.0	66.6	316	24.66	39.0	even	ok
	282	C14H10N53NaO29PS2	500.6666	1.3	66.7	317	22.75	38.0	even	ok
	283	C15H18N43O38P2S	500.6659	-0.0	66.9	318	30.21	31.0	even	ok
	284	C13H4N57O30S2	500.6655	-0.9	66.9	319	25.05	42.0	even	ok
	285	C28H32N17O54S	500.6662	0.5	66.9	320	27.50	23.0	even	ok
	286	C16H17N41O41PS	500.6649	-2.0	66.9	321	18.59	31.0	even	ok
	287	C26H6N51NaO22PS2	500.6660	0.1	67.3	322	29.54	51.0	even	ok
	288	C22H21N35NaO40S2	500.6669	2.0	67.3	323	22.18	31.0	even	ok
	289	C40H38N7NaO50PS	500.6662	0.5	67.4	324	32.05	27.0	even	ok
	290	C17H23N37NaO40P2S	500.6660	0.2	67.5	325	28.78	27.0	even	ok
	291	C33H45NNaO62S	500.6667	1.5	67.5	326	24.89	13.0	even	ok
	292	C26H42N9O60P2	500.6657	-0.5	67.6	327	26.83	13.0	even	ok
	293	C26H26N29NaO40PS2	500.6651	-1.7	67.6	328	23.74	30.0	even	ok
	294	C45H33N7O50P	500.6659	-0.1	67.7	329	28.78	35.0	even	ok
	295	C18H22N35NaO43PS	500.6650	-1.8	67.7	330	19.08	27.0	even	ok
	296	C25H34N19NaO51PS	500.6664	0.9	67.7	331	24.48	20.0	even	ok
	297	C37H30N17NaO44PS	500.6657	-0.4	67.8	332	27.14	33.0	even	ok
	298	C27H12N41O33S2	500.6649	-2.1	67.8	333	21.06	44.0	even	ok
	299	C24H28N23O52S	500.6653	-1.3	67.9	334	21.68	24.0	even	ok
	300	C19H18N43O33P2S2	500.6651	-1.7	67.9	335	19.66	35.0	even	ok
	301	C40H10N37O27P2	500.6669	1.9	67.9	336	18.79	57.0	even	ok
	302	C38H43N5NaO51P2S	500.6667	1.6	68.1	337	19.90	22.0	even	ok
	303	C29H31N25NaO39P2S2	500.6670	2.1	68.1	338	21.04	29.0	even	ok
	304	C15H9N51NaO32S2	500.6656	-0.7	68.1	339	24.98	38.0	even	ok
	305	C28H47N3NaO62P2	500.6658	-0.3	68.1	340	27.34	9.0	even	ok
	306	C26H30N27O41P2S2	500.6664	1.0	68.2	341	27.94	28.0	even	ok
	307	C16H17N37NaO47	500.6653	-1.3	68.2	342	21.76	28.0	even	ok
	308	C14H12N43O45	500.6652	-1.5	68.2	343	20.74	32.0	even	ok
	309	C12H10N49NaO38P	500.6658	-0.2	68.2	344	27.88	34.0	even	ok
	310	C24H31N27NaO41P2S2	500.6656	-0.6	68.3	345	30.43	25.0	even	ok
	311	C27H19N39NaO29P2S2	500.6670	2.1	68.3	346	17.46	40.0	even	ok
	312	C19H13N45NaO34S2	500.6665	1.1	68.3	347	22.60	37.0	even	ok
	313	C34H9N41O28PS	500.6661	0.3	68.3	348	26.96	53.0	even	ok
	314	C10H5N55O36P	500.6657	-0.4	68.5	350	26.52	38.0	even	ok
	315	C30H37N11NaO56S	500.6663	0.6	68.6	351	24.86	19.0	even	ok
	316	C26H23N35NaO33P2S2	500.6665	1.2	68.7	352	21.85	35.0	even	ok
	317	C36H21N27O38PS	500.6661	0.3	68.8	353	26.43	42.0	even	ok
	318	C23H22N37O35P2S2	500.6660	0.1	69.0	354	27.56	34.0	even	ok
	319	C43H21N21O40P	500.6659	-0.1	69.1	355	27.24	46.0	even	ok
	320	C26H33N17NaO54S	500.6654	-1.1	69.2	356	21.65	20.0	even	ok
	321	C39H32N11O51S	500.6651	-1.7	69.3	359	22.62	31.0	even	ok
	322	C15H18N39NaO44P	500.6663	0.7	69.4	361	23.69	28.0	even	ok
	323	C21H23N37NaO35P2S2	500.6652	-1.5	69.5	362	19.49	31.0	even	ok
	324	C28H25N29O40PS2	500.6659	-0.1	69.5	363	32.40	33.0	even	ok

Compound Spectrum SmartFormula Report

Meas. m/z	#	Ion Formula	m/z	err [ppm]	mSigma	# mSigma	Score	rdb	e⁻ Conf	N-Rule
	325	C29H21N33O36PS2	500.6663	0.8	69.5	364	27.95	38.0	even	ok
	326	C13H13N45O42P	500.6662	0.5	69.5	366	24.59	32.0	even	ok
	327	C28H8N45O29S2	500.6653	-1.2	69.7	367	25.02	49.0	even	ok
	328	C32H47N3NaO57P2S	500.6649	-1.9	69.7	368	17.09	13.0	even	ok
	329	C15H13N49O33PS2	500.6669	2.0	69.9	369	16.74	36.0	even	ok
	330	C29H17N35NaO35S2	500.6650	-1.9	69.9	370	20.58	40.0	even	ok
	331	C28H35N21NaO43P2S2	500.6665	1.2	69.9	371	25.04	24.0	even	ok
	332	C17H20N33O51	500.6656	-0.6	69.9	372	24.15	26.0	even	ok
	333	C9H7N61NaO24P2S2	500.6667	1.5	69.9	373	19.20	39.0	even	ok
	334	C42H15N31NaO29P2	500.6669	2.0	70.0	374	16.52	53.0	even	ok
	335	C47H38NNaO52P	500.6660	0.1	70.0	375	26.72	31.0	even	ok
	336	C11H11N51NaO35P2	500.6668	1.8	70.0	376	17.56	34.0	even	ok
	337	C18H26N33O44P2S	500.6664	0.9	70.0	377	22.39	25.0	even	ok
	338	C26H13N43O30PS2	500.6659	-0.1	70.1	378	26.37	44.0	even	ok
	339	C19H25N27NaO53	500.6657	-0.4	70.1	379	25.00	22.0	even	ok
	340	C25H17N39O34PS2	500.6654	-1.0	70.2	380	21.57	39.0	even	ok
	341	C10H6N59NaO27PS2	500.6657	-0.5	70.4	381	24.10	39.0	even	ok
	342	C19H25N31O47PS	500.6654	-1.1	70.4	382	20.79	25.0	even	ok
	343	C41H34N11NaO46PS	500.6666	1.4	70.4	383	23.19	32.0	even	ok
	344	C40H6N39NaO26P	500.6655	-0.8	70.4	384	22.16	59.0	even	ok
	345	C30H44N3O64S	500.6662	0.5	70.7	386	28.71	12.0	even	ok
	346	C41H41N3O54PS	500.6665	1.2	70.7	387	24.01	25.0	even	ok
	347	C36H14N35NaO30PS	500.6662	0.5	70.7	388	23.74	49.0	even	ok
	348	C20H31N27NaO46P2S	500.6665	1.1	70.8	389	20.82	21.0	even	ok
	349	C25H27N31NaO37P2S2	500.6661	0.3	70.9	390	24.73	30.0	even	ok
	350	C26H37N15O55PS	500.6667	1.6	70.9	392	18.19	18.0	even	ok
	351	C27H29N25O44PS2	500.6654	-1.0	71.0	393	25.15	28.0	even	ok
	352	C16H21N35O48P	500.6666	1.4	71.0	394	18.79	26.0	even	ok
	353	C18H26N29NaO50P	500.6667	1.6	71.0	395	17.86	22.0	even	ok
	354	C30H17N37O32PS2	500.6668	1.7	71.0	396	21.11	43.0	even	ok
	355	C38H26N21NaO40PS	500.6662	0.5	71.2	397	23.23	38.0	even	ok
	356	C21H30N25NaO49PS	500.6655	-0.9	71.3	398	21.06	21.0	even	ok
	357	C9H4N57O35S	500.6663	0.8	71.3	399	21.79	38.0	even	ok
	358	C45H26N15NaO42P	500.6660	0.1	71.4	400	25.43	42.0	even	ok
	359	C16H12N47O36S2	500.6659	0.0	71.4	401	25.62	36.0	even	ok
	360	C30H30N23NaO42PS2	500.6660	0.1	71.4	402	30.33	29.0	even	ok
	361	C41H37N5NaO53S	500.6652	-1.5	71.4	403	21.94	27.0	even	ok
	362	C25H34N23O45P2S2	500.6660	0.1	71.5	404	30.06	23.0	even	ok
	363	C31H26N27NaO38PS2	500.6664	1.0	71.6	405	24.77	34.0	even	ok
	364	C11H9N51NaO37S	500.6664	1.0	71.6	406	20.59	34.0	even	ok
	365	C27H5N49NaO25S2	500.6650	-1.9	71.7	407	16.02	51.0	even	ok
	366	C21H25N31NaO44S2	500.6665	1.1	71.7	408	23.86	26.0	even	ok
	367	C27H9N47O26PS2	500.6663	0.8	71.8	409	21.43	49.0	even	ok
	368	C30H13N39NaO31S2	500.6654	-1.0	71.8	410	24.17	45.0	even	ok
	369	C20H16N41O38S2	500.6668	1.8	71.9	411	16.43	35.0	even	ok
	370	C20H28N23O57	500.6661	0.3	72.1	412	23.34	20.0	even	ok

Compound Spectrum SmartFormula Report

Meas. m/z	#	Ion Formula	m/z	err [ppm]	mSigma	# mSigma	Score	rdb	e¯ Conf	N-Rule
	371	C28H42N9NaO57PS	500.6668	1.8	72.2	414	16.47	14.0	even	ok
	372	C22H33N17NaO59	500.6662	0.5	72.4	415	22.15	16.0	even	ok
	373	C46H32N5O53	500.6649	-2.1	72.5	416	14.72	35.0	even	ok
	374	C40H28N15O47S	500.6655	-0.8	72.5	417	25.09	36.0	even	ok
	375	C27H36N13O58S	500.6657	-0.4	72.5	418	22.54	18.0	even	ok
	376	C41H9N35O30P	500.6659	-0.1	72.6	419	23.91	57.0	even	ok
	377	C29H34N19NaO46PS2	500.6655	-0.8	72.8	420	24.57	24.0	even	ok
	378	C29H24N27O43S2	500.6649	-2.1	72.9	421	17.49	33.0	even	ok
	379	C23H35N23NaO45P2S2	500.6652	-1.5	73.0	422	20.53	20.0	even	ok
	380	C22H26N33O39P2S2	500.6655	-0.8	73.0	423	20.35	29.0	even	ok
	381	C27H39N17NaO47P2S2	500.6661	0.3	73.1	424	27.26	19.0	even	ok
	382	C11H9N55O31PS2	500.6660	0.2	73.2	425	22.99	37.0	even	ok
	383	C35H8N39O31S	500.6651	-1.7	73.2	426	16.24	53.0	even	ok
	384	C32H22N31NaO34PS2	500.6669	1.9	73.2	427	18.49	39.0	even	ok
	385	C30H20N31O39S2	500.6653	-1.2	73.2	428	21.91	38.0	even	ok
	386	C29H38N17O47P2S2	500.6669	1.9	73.3	429	18.26	22.0	even	ok
	387	C21H34N23O50P2S	500.6668	1.8	73.5	430	15.61	19.0	even	ok
	388	C37H20N25O41S	500.6651	-1.7	73.5	431	16.04	42.0	even	ok
	389	C42H37N7O50PS	500.6670	2.1	73.5	432	16.93	30.0	even	ok
	390	C18H17N41NaO38S2	500.6660	0.2	73.5	433	22.73	32.0	even	ok
	391	C37H17N31O34PS	500.6665	1.2	73.8	434	17.84	47.0	even	ok
	392	C11H14N49O36P2S	500.6650	-1.8	73.8	435	15.23	32.0	even	ok
	393	C12H12N47O41S	500.6668	1.7	73.9	436	15.77	32.0	even	ok
	394	C26H33N21O48PS2	500.6650	-1.9	73.9	437	17.87	23.0	even	ok
	395	C29H41N7NaO60S	500.6658	-0.2	73.9	438	22.23	14.0	even	ok
	396	C44H20N19O43	500.6649	-2.1	73.9	439	13.88	46.0	even	ok
	397	C12H8N53O34S2	500.6650	-1.8	74.0	440	15.32	37.0	even	ok
	398	C13H14N49NaO33PS2	500.6661	0.4	74.1	441	21.32	33.0	even	ok
	399	C13H19N43NaO38P2S	500.6651	-1.6	74.1	442	15.84	28.0	even	ok
	400	C22H33N21O53PS	500.6658	-0.2	74.1	443	22.12	19.0	even	ok
	401	C46H29N11O46P	500.6663	0.8	74.1	444	19.63	40.0	even	ok
	402	C29H14N41NaO28PS2	500.6664	1.0	74.1	445	18.75	45.0	even	ok
	403	C28H5N51O22PS2	500.6668	1.7	74.2	446	15.64	54.0	even	ok
	404	C14H17N41NaO43S	500.6669	1.9	74.3	447	14.70	28.0	even	ok
	405	C23H39N17NaO52P2S	500.6669	2.0	74.4	448	14.33	15.0	even	ok
	406	C23H36N13O63	500.6665	1.2	74.6	449	17.20	14.0	even	ok
	407	C39H29N17O44PS	500.6665	1.2	74.6	450	17.25	36.0	even	ok
	408	C42H33N9NaO49S	500.6656	-0.6	74.7	451	24.07	32.0	even	ok
	409	C43H14N29NaO32P	500.6659	0.0	74.7	452	22.46	53.0	even	ok
	410	C42H40NO57S	500.6655	-0.8	74.7	453	23.07	25.0	even	ok
	411	C31H29N21NaO45S2	500.6650	-1.9	74.8	454	17.09	29.0	even	ok
	412	C31H33N19O46PS2	500.6663	0.8	74.8	455	22.80	27.0	even	ok
	413	C22H28N27O48S2	500.6668	1.8	74.9	456	17.56	24.0	even	ok
	414	C24H43N9NaO60P2	500.6649	-2.1	74.9	457	13.58	10.0	even	ok
	415	C35H5N45O24PS	500.6665	1.2	74.9	458	17.13	58.0	even	ok
	416	C32H29N23O42PS2	500.6668	1.7	75.0	459	18.15	32.0	even	ok

Compound Spectrum SmartFormula Report

Meas. m/z	#	Ion Formula	m/z	err [ppm]	mSigma	# mSigma	Score	rdb	e⁻ Conf	N-Rule
	417	C14H13N47NaO36S2	500.6651	-1.6	75.0	460	15.47	33.0	even	ok
	418	C31H43N11NaO49P2S2	500.6670	2.1	75.1	461	16.18	18.0	even	ok
	419	C25H41N7NaO65	500.6666	1.4	75.1	462	16.10	10.0	even	ok
	420	C31H16N35O35S2	500.6658	-0.3	75.1	463	25.08	43.0	even	ok
	421	C24H38N15NaO55PS	500.6659	-0.0	75.1	464	22.12	15.0	even	ok
	422	C31H9N43NaO27S2	500.6659	-0.1	75.2	465	25.96	50.0	even	ok
	423	C40H3N45NaO19P2	500.6669	2.0	75.2	466	13.59	64.0	even	ok
	424	C32H25N25NaO41S2	500.6654	-1.0	75.3	467	21.16	34.0	even	ok
	425	C28H38N15NaO50PS2	500.6651	-1.7	75.5	469	17.66	19.0	even	ok
	426	C37H13N33NaO33S	500.6652	-1.5	75.5	471	15.58	49.0	even	ok
	427	C39H25N19NaO43S	500.6652	-1.5	75.9	472	15.37	38.0	even	ok
	428	C5H3N67NaO22P2S2	500.6658	-0.3	76.0	473	20.31	40.0	even	ok
	429	C24H38N19O49P2S2	500.6655	-0.8	76.1	474	21.70	18.0	even	ok
	430	C25H37N13NaO58S	500.6649	-2.0	76.1	475	13.11	15.0	even	ok
	431	C30H37N15O50PS2	500.6659	-0.1	76.2	476	25.16	22.0	even	ok
	432	C39H22N25NaO36PS	500.6666	1.4	76.2	477	15.52	43.0	even	ok
	433	C46H25N13NaO45	500.6650	-1.9	76.2	478	13.41	42.0	even	ok
	434	C14H22N39O42P2S	500.6655	-0.9	76.3	479	17.42	26.0	even	ok
	435	C24H33N21NaO50S2	500.6669	2.0	76.3	480	15.76	20.0	even	ok
	436	C28H42N13O51P2S2	500.6664	1.0	76.4	481	20.43	17.0	even	ok
	437	C48H34N5NaO48P	500.6664	1.0	76.5	482	17.17	36.0	even	ok
	438	C16H27N33NaO44P2S	500.6656	-0.7	76.8	483	17.87	22.0	even	ok
	439	C33H38N13NaO48PS2	500.6664	1.0	76.8	484	20.21	23.0	even	ok
	440	C41H24N19O43S	500.6660	0.1	76.9	485	24.30	41.0	even	ok
	441	C19H20N37O42S2	500.6664	0.9	77.0	486	16.99	30.0	even	ok
	442	C44H17N25O36P	500.6663	0.8	77.0	487	17.58	51.0	even	ok
	443	C14H17N45O37PS2	500.6665	1.1	77.0	489	16.13	31.0	even	ok
	444	C34H34N17NaO44PS2	500.6669	1.9	77.0	490	15.90	28.0	even	ok
	445	C25H46N5O64P2	500.6652	-1.4	77.1	491	15.15	8.0	even	ok
	446	C37H10N39NaO26PS	500.6666	1.4	77.2	492	14.92	54.0	even	ok
	447	C20H29N27NaO48S2	500.6660	0.2	77.2	493	23.59	21.0	even	ok
	448	C33H21N29NaO37S2	500.6659	-0.1	77.2	494	23.96	39.0	even	ok
	449	C34H22N31O32P2S2	500.6649	-2.0	77.3	495	15.05	42.0	even	ok
	450	C26H43N13NaO51P2S2	500.6656	-0.6	77.5	496	21.44	14.0	even	ok
	451	C30H10N45NaO24PS2	500.6669	1.9	77.6	497	13.02	50.0	even	ok
	452	C8H3N61NaO29P2	500.6664	0.9	77.8	498	13.13	40.0	even	ok
	453	C43H36N5O53S	500.6660	0.1	77.8	499	23.46	30.0	even	ok
	454	C30H47N7NaO53P2S2	500.6665	1.2	78.0	500	18.32	13.0	even	ok
	455	C32H42N9NaO52PS2	500.6660	0.1	78.0	501	23.50	18.0	even	ok
	456	C8H6N55NaO36P	500.6649	-2.0	78.0	502	12.40	35.0	even	ok
	457	C25H41N11O59PS	500.6663	0.7	78.1	503	17.14	13.0	even	ok
	458	C7H5N61O29PS2	500.6651	-1.6	78.2	504	13.69	38.0	even	ok
	459	C32H32N17O49S2	500.6653	-1.2	78.3	505	18.05	27.0	even	ok
	460	C32H12N39O31S2	500.6662	0.6	78.3	506	20.93	48.0	even	ok
	461	C6H6N63O26P2S2	500.6661	0.4	78.3	507	18.02	38.0	even	ok
	462	C34H15N39NaO24P2S2	500.6650	-1.8	78.4	508	15.15	49.0	even	ok

Compound Spectrum SmartFormula Report

Meas. m/z	#	Ion Formula	m/z	err [ppm]	mSigma	# mSigma	Score	rdb	e¯ Conf	N-Rule
	463	C21H30N29O43P2S2	500.6651	-1.7	78.4	509	13.17	24.0	even	ok
	464	C38H16N29O37S	500.6655	-0.8	78.6	510	16.42	47.0	even	ok
	465	C9H6N57O33P2	500.6667	1.6	78.7	511	10.58	38.0	even	ok
	466	C11H14N45NaO42P	500.6654	-1.1	78.7	512	15.29	29.0	even	ok
	467	C33H28N21O45S2	500.6658	-0.3	78.7	513	21.77	32.0	even	ok
	468	C16H22N39NaO39PS2	500.6666	1.3	78.7	514	14.40	27.0	even	ok
	469	C15H16N43O40S2	500.6655	-0.9	78.8	515	15.96	31.0	even	ok
	470	C29H41N11O54PS2	500.6654	-1.0	78.8	516	18.58	17.0	even	ok
	471	C8H11N57NaO28P2S2	500.6662	0.6	78.8	517	16.92	34.0	even	ok
	472	C9H10N55NaO31PS2	500.6652	-1.4	78.9	518	14.01	34.0	even	ok
	473	C47H28N9O49	500.6653	-1.2	79.0	519	14.51	40.0	even	ok
	474	C9H9N51O40P	500.6653	-1.2	79.0	520	14.41	33.0	even	ok
	475	C43H29N13NaO45S	500.6661	0.3	79.1	521	21.42	37.0	even	ok
	476	C17H30N29O48P2S	500.6659	-0.0	79.1	522	18.95	20.0	even	ok
	477	C18H29N27O51PS	500.6649	-2.0	79.2	523	11.68	20.0	even	ok
	478	C31H36N13O53S2	500.6649	-2.1	79.2	524	13.68	22.0	even	ok
	479	C46H22N19NaO38P	500.6664	0.9	79.2	525	15.43	47.0	even	ok
	480	C27H46N5NaO61PS	500.6664	0.9	79.2	526	15.71	9.0	even	ok
	481	C26H40N9O62S	500.6653	-1.3	79.3	527	14.04	13.0	even	ok
	482	C49H37NO52P	500.6668	1.7	79.3	528	12.78	34.0	even	ok
	483	C7H5N57NaO35S	500.6655	-0.8	79.4	529	15.80	35.0	even	ok
	484	C40H25N21O40PS	500.6670	2.1	79.4	530	11.24	41.0	even	ok
	485	C44H13N27NaO35	500.6650	-1.9	79.4	531	11.79	53.0	even	ok
	486	C36H27N25NaO34P2S2	500.6650	-1.8	79.5	532	14.53	38.0	even	ok
	487	C19H35N23NaO50P2S	500.6660	0.2	79.7	535	17.96	16.0	even	ok
	488	C36H4N43O27S	500.6655	-0.8	79.8	536	15.65	58.0	even	ok
	489	C14H22N35NaO48P	500.6658	-0.2	79.8	537	17.91	23.0	even	ok
	490	C20H34N21NaO53PS	500.6650	-1.8	79.9	538	11.97	16.0	even	ok
	491	C36H34N17O42P2S2	500.6649	-2.0	79.9	539	13.59	31.0	even	ok
	492	C29H4N49O25S2	500.6658	-0.3	79.9	540	17.27	54.0	even	ok
	493	C12H17N41O46P	500.6657	-0.4	80.0	541	17.11	27.0	even	ok
	494	C17H21N37NaO42S2	500.6656	-0.7	80.0	542	15.87	27.0	even	ok
	495	C38H13N35O30PS	500.6670	2.1	80.1	543	10.95	52.0	even	ok
	496	C16H24N29O55	500.6652	-1.4	80.1	544	13.13	21.0	even	ok
	497	C18H29N23NaO57	500.6653	-1.3	80.2	545	13.72	17.0	even	ok
	498	C34H41N9O52PS2	500.6668	1.7	80.2	546	14.69	21.0	even	ok
	499	C34H37N11NaO51S2	500.6654	-1.0	80.3	547	17.44	23.0	even	ok
	500	C21H32N23O52S2	500.6664	0.9	80.3	548	17.84	19.0	even	ok
515.0071	1	C71H27N6NaO33P	515.0079	1.6	5.6	1	72.80	63.0	even	ok
	2	C69H18N14NaO30	515.0065	-1.2	6.3	2	67.69	69.0	even	ok
	3	C70H21N10O34	515.0068	-0.5	6.4	3	95.76	67.0	even	ok
	4	C69H22N12O31P	515.0078	1.4	6.5	4	62.83	67.0	even	ok
	5	C61H131N31NaO12S	515.0065	-1.1	6.6	5	68.40	13.0	even	ok
	6	C67H13N20O28	515.0064	-1.4	7.1	6	63.61	73.0	even	ok
	7	C62H134N27O16S	515.0069	-0.4	7.4	7	95.43	11.0	even	ok
	8	C63H140N23NaO15PS	515.0080	1.7	7.4	8	69.32	7.0	even	ok

Compound Spectrum SmartFormula Report

Meas. m/z	#	Ion Formula	m/z	err [ppm]	mSigma	# mSigma	Score	rdb	e⁻ Conf	N-Rule
	9	C59H126N37O10S	515.0064	-1.3	7.4	9	64.24	17.0	even	ok
	10	C72H152N3NaO27P	515.0077	1.3	7.8	10	64.58	0.0	even	ok
	11	C72H26N4NaO36	515.0069	-0.3	8.1	11	96.77	63.0	even	ok
	12	C61H128N37NaO5PS	515.0080	1.7	8.2	12	57.06	18.0	even	ok
	13	C70H150N3O32	515.0062	-1.7	8.3	13	56.26	-1.0	even	ok
	14	C61H135N29O13PS	515.0079	1.5	8.5	15	59.62	11.0	even	ok
	15	C68H19N16NaO27P	515.0075	0.8	8.6	16	72.03	69.0	even	ok
	16	C71H146N7O28	515.0066	-0.9	8.7	17	70.15	4.0	even	ok
	17	C70H147N9O25P	515.0076	1.1	8.7	18	66.56	4.0	even	ok
	18	C64H139N21NaO18S	515.0070	-0.2	8.8	19	96.81	7.0	even	ok
	19	C73H151NNaO30	515.0067	-0.7	8.9	20	87.60	0.0	even	ok
	20	C59H123N43O3PS	515.0079	1.5	8.9	21	59.28	22.0	even	ok
	21	C60H122N41O6S	515.0069	-0.4	9.4	22	76.61	22.0	even	ok
	22	C60H132N33NaO9PS	515.0075	0.8	9.6	23	69.78	13.0	even	ok
	23	C60H6N34NaO18S	515.0067	-0.7	9.7	24	85.08	76.0	even	ok
	24	C70H143N11NaO24	515.0063	-1.6	9.9	26	57.30	6.0	even	ok
	25	C59H119N45NaO2S	515.0065	-1.1	10.4	27	63.65	24.0	even	ok
	26	C71H30NaO40	515.0065	-1.2	10.4	28	75.41	58.0	even	ok
	27	C62H127N35NaO8S	515.0070	-0.3	10.5	29	78.09	18.0	even	ok
	28	C64H15N26O19P2S	515.0062	-1.7	10.5	30	65.13	73.0	even	ok
	29	C69H25N6O38	515.0064	-1.4	10.7	31	71.64	62.0	even	ok
	30	C66H14N22O25P	515.0074	0.6	10.9	32	72.10	73.0	even	ok
	31	C68H138N17O22	515.0062	-1.7	11.0	33	53.43	10.0	even	ok
	32	C73H29O40	515.0073	0.4	11.1	34	89.97	61.0	even	ok
	33	C63H130N31O12S	515.0073	0.4	11.2	35	88.79	16.0	even	ok
	34	C57H114N51S	515.0064	-1.3	11.2	36	59.81	28.0	even	ok
	35	C62H5N34O18S	515.0075	0.8	11.5	37	80.90	79.0	even	ok
	36	C58H127N39O7PS	515.0074	0.6	11.7	38	70.01	17.0	even	ok
	37	C61H9N30O22S	515.0071	-0.1	11.8	39	95.38	74.0	even	ok
	38	C69H144N13NaO21P	515.0073	0.4	11.9	40	73.90	6.0	even	ok
	39	C65H142N17O22S	515.0073	0.4	12.0	41	87.17	5.0	even	ok
	40	C56H128N43OP2S2	515.0062	-1.7	12.1	42	64.32	17.0	even	ok
	41	C54H118N51S2	515.0075	0.9	12.1	43	78.76	23.0	even	ok
	42	C69H15N20NaO23P	515.0079	1.6	12.1	44	54.01	74.0	even	ok
	43	C70H31N2NaO37P	515.0075	0.8	12.3	45	80.63	58.0	even	ok
	44	C59H7N36NaO15PS	515.0077	1.2	12.4	46	60.27	76.0	even	ok
	45	C67H10N26O21P	515.0078	1.4	12.5	47	56.33	78.0	even	ok
	46	C69H151N5O29P	515.0072	0.2	12.6	48	75.72	-1.0	even	ok
	47	C66H20N20NaO21P2S	515.0063	-1.5	12.8	49	65.61	69.0	even	ok
	48	C63H14N24NaO24S	515.0072	0.1	13.1	50	91.57	70.0	even	ok
	49	C65H135N25NaO14S	515.0074	0.6	13.3	51	81.76	12.0	even	ok
	50	C62H3N40O9P2S	515.0062	-1.7	13.3	52	51.31	84.0	even	ok
	51	C52H119N51NaS2	515.0067	-0.7	13.3	53	80.52	20.0	even	ok
	52	C68H9N24O24	515.0068	-0.5	13.4	54	70.04	78.0	even	ok
	53	C68H26N8O35P	515.0074	0.6	13.5	55	82.16	62.0	even	ok
	54	C71H17N14O30	515.0073	0.4	13.8	57	85.47	72.0	even	ok

Compound Spectrum SmartFormula Report

Meas. m/z	#	Ion Formula	m/z	err [ppm]	mSigma	# mSigma	Score	rdb	e¯ Conf	N-Rule
	55	C64H10N28NaO20S	515.0076	1.0	13.9	58	73.88	75.0	even	ok
	56	C67H139N19O19P	515.0072	0.2	14.0	59	73.81	10.0	even	ok
	57	C58H133N37NaO3P2S2	515.0063	-1.5	14.0	60	65.09	13.0	even	ok
	58	C64H8N34NaO11P2S	515.0063	-1.5	14.1	61	63.84	80.0	even	ok
	59	C63H143N17NaO22S	515.0065	-1.1	14.1	62	71.35	2.0	even	ok
	60	C67H147N11NaO24S	515.0074	0.6	14.2	63	80.24	1.0	even	ok
	61	C70H14N18NaO26	515.0069	-0.3	14.2	64	71.69	74.0	even	ok
	62	C56H123N45NaO2S2	515.0076	1.1	14.3	65	72.09	19.0	even	ok
	63	C61H138N23O20S	515.0064	-1.3	14.3	66	67.81	6.0	even	ok
	64	C68H148N13O19P2S	515.0065	-1.2	14.4	67	69.16	4.0	even	ok
	65	C65H11N26NaO21P	515.0070	-0.1	14.5	68	74.42	75.0	even	ok
	66	C67H6N28NaO20	515.0065	-1.2	14.6	69	57.84	80.0	even	ok
	67	C57H124N43NaO3PS	515.0071	-0.1	14.7	70	75.12	19.0	even	ok
	68	C66H7N30NaO17P	515.0075	0.7	15.2	72	63.60	80.0	even	ok
	69	C53H122N47O4S2	515.0071	0.0	15.3	73	89.85	18.0	even	ok
	70	C68H148N9NaO25P	515.0068	-0.5	15.5	74	67.31	1.0	even	ok
	71	C73H22N8NaO32	515.0074	0.6	15.7	75	79.11	68.0	even	ok
	72	C62H144N19NaO19PS	515.0075	0.8	15.9	76	73.98	2.0	even	ok
	73	C64H146N13O26S	515.0069	-0.4	15.9	77	81.22	0.0	even	ok
	74	C66H138N21O18S	515.0078	1.3	16.1	78	65.11	10.0	even	ok
	75	C55H127N41NaO6S2	515.0072	0.2	16.2	79	84.88	14.0	even	ok
	76	C64H17N20O28S	515.0075	0.8	16.3	80	73.47	68.0	even	ok
	77	C67H23N12NaO31P	515.0070	-0.1	16.3	81	71.85	64.0	even	ok
	78	C67H23N16O25P2S	515.0067	-0.8	16.4	82	73.00	67.0	even	ok
	79	C65H11N30O15P2S	515.0067	-0.8	16.4	83	72.86	78.0	even	ok
	80	C65H152N9NaO25PS	515.0080	1.7	16.5	84	58.19	-4.0	even	ok
	81	C63H147N15O23PS	515.0079	1.5	16.5	85	61.17	0.0	even	ok
	82	C55H3N46O7P2S2	515.0064	-1.3	16.7	86	65.01	80.0	even	ok
	83	C66H151N7NaO28S	515.0070	-0.2	16.7	87	83.12	-4.0	even	ok
	84	C70H153N7NaO21P2S	515.0066	-1.0	16.9	89	68.87	0.0	even	ok
	85	C60H139N25O17PS	515.0074	0.6	16.9	90	75.69	6.0	even	ok
	86	C66H27N12O29P2S	515.0062	-1.7	17.0	91	57.42	62.0	even	ok
	87	C63H6N32O19P	515.0069	-0.3	17.1	92	67.91	79.0	even	ok
	88	C55H119N49OPS	515.0070	-0.2	17.3	93	68.59	23.0	even	ok
	89	C65H13N24O24S	515.0080	1.7	17.4	94	57.28	73.0	even	ok
	90	C70H140N17NaO17P	515.0077	1.2	17.6	95	53.61	11.0	even	ok
	91	C59H136N33O7P2S2	515.0067	-0.8	17.6	96	72.18	11.0	even	ok
	92	C68H150N7O28S	515.0078	1.3	17.8	97	62.74	-1.0	even	ok
	93	C74H25N4O36	515.0077	1.3	17.9	98	63.84	66.0	even	ok
	94	C66H136N23NaO15P	515.0068	-0.5	17.9	99	63.95	12.0	even	ok
	95	C57H126N41O6S2	515.0080	1.7	18.0	100	55.57	17.0	even	ok
	96	C66H143N15O23P	515.0067	-0.7	18.1	101	61.24	5.0	even	ok
	97	C68H135N23O15P	515.0076	1.1	18.1	102	55.50	15.0	even	ok
	98	C66H22N14NaO30S	515.0076	1.0	18.1	103	67.63	64.0	even	ok
	99	C59H10N30NaO22S	515.0063	-1.6	18.3	104	57.44	71.0	even	ok
	100	C57H8N40NaO9P2S2	515.0065	-1.1	18.4	105	65.78	76.0	even	ok

Compound Spectrum SmartFormula Report

Meas. m/z	#	Ion Formula	m/z	err [ppm]	mSigma	# mSigma	Score	rdb	e⁻ Conf	N-Rule
	101	C65H18N18O29P	515.0069	-0.3	18.5	106	66.07	68.0	even	ok
	102	C69H134N21O18	515.0066	-0.9	18.5	107	57.74	15.0	even	ok
	103	C68H32N6NaO31P2S	515.0063	-1.5	18.6	108	58.44	58.0	even	ok
	104	C68H143N15NaO20S	515.0079	1.5	18.6	109	58.91	6.0	even	ok
	105	C72H142N11O24	515.0071	0.0	18.7	111	69.88	9.0	even	ok
	106	C67H16N24NaO17P2S	515.0067	-0.7	18.8	112	72.43	74.0	even	ok
	107	C69H28N10NaO27P2S	515.0068	-0.7	18.8	113	72.44	63.0	even	ok
	108	C71H139N15NaO20	515.0067	-0.7	18.9	114	59.84	11.0	even	ok
	109	C57H5N36O20S	515.0062	-1.8	19.1	115	53.70	75.0	even	ok
	110	C56H130N37O10S2	515.0075	0.9	19.2	116	68.18	12.0	even	ok
	111	C60H13N26O26S	515.0066	-0.9	19.2	117	67.61	69.0	even	ok
	112	C58H4N44NaO5P2S2	515.0070	-0.2	19.2	118	79.22	81.0	even	ok
	113	C62H18N20NaO28S	515.0067	-0.7	19.2	119	70.52	65.0	even	ok
	114	C59H136N29NaO13PS	515.0071	-0.0	19.4	120	68.23	8.0	even	ok
	115	C67H154N3O32S	515.0073	0.5	19.6	121	74.78	-6.0	even	ok
	116	C66H136N27O9P2S	515.0065	-1.2	19.6	122	51.67	15.0	even	ok
	117	C75H150NO30	515.0075	0.9	19.6	123	67.67	3.0	even	ok
	118	C64H12N28NaO18P2	515.0080	1.8	19.6	124	43.85	75.0	even	ok
	119	C61H118N45O2S	515.0073	0.4	19.7	125	62.37	27.0	even	ok
	120	C61H141N27NaO9P2S2	515.0068	-0.6	19.9	126	71.84	7.0	even	ok
	121	C68H141N21NaO11P2S	515.0066	-1.0	19.9	127	64.54	11.0	even	ok
	122	C74H147N5NaO26	515.0072	0.2	20.1	128	65.31	5.0	even	ok
	123	C70H155NNaO30S	515.0079	1.5	20.2	129	56.87	-5.0	even	ok
	124	C68H131N25NaO14	515.0063	-1.6	20.3	130	46.53	17.0	even	ok
	125	C58H140N29O11P2S2	515.0062	-1.6	20.3	131	54.52	6.0	even	ok
	126	C64H131N29O13P	515.0067	-0.7	20.3	132	58.26	16.0	even	ok
	127	C71H156N3O25P2S	515.0069	-0.3	20.3	133	75.36	-2.0	even	ok
	128	C61H19N22NaO25PS	515.0077	1.2	20.4	134	61.29	65.0	even	ok
	129	C58H135N31NaO12S2	515.0076	1.1	20.7	135	63.04	8.0	even	ok
	130	C60H132N37O3P2S2	515.0071	0.1	20.9	136	78.70	16.0	even	ok
	131	C67H149N11NaO22P2	515.0078	1.5	20.9	137	47.32	1.0	even	ok
	132	C66H126N31O12	515.0062	-1.7	20.9	138	43.61	21.0	even	ok
	133	C67H132N27NaO11P	515.0073	0.4	20.9	139	61.59	17.0	even	ok
	134	C59H14N28O23PS	515.0076	1.0	21.0	140	63.39	69.0	even	ok
	135	C62H3N36NaO15P	515.0066	-1.0	21.0	141	53.20	81.0	even	ok
	136	C58H11N32NaO19PS	515.0073	0.3	21.0	142	74.50	71.0	even	ok
	137	C64H15N22NaO25P	515.0066	-1.0	21.1	143	53.14	70.0	even	ok
	138	C63H123N39NaO4S	515.0074	0.6	21.2	144	58.04	23.0	even	ok
	139	C74H23N10O27P2	515.0064	-1.3	21.2	145	58.98	71.0	even	ok
	140	C63H21N16O32S	515.0071	-0.0	21.2	146	78.82	63.0	even	ok
	141	C57H131N35O11PS	515.0070	-0.2	21.3	147	63.06	12.0	even	ok
	142	C66H24N14NaO28P2	515.0080	1.8	21.3	148	42.20	64.0	even	ok
	143	C65H140N19NaO19P	515.0064	-1.4	21.5	150	47.83	7.0	even	ok
	144	C60H145N23NaO13P2S2	515.0063	-1.5	21.6	151	55.71	2.0	even	ok
	145	C67H25N10O34S	515.0080	1.7	21.7	152	52.11	62.0	even	ok
	146	C68H19N20O21P2S	515.0071	0.0	21.8	153	78.17	72.0	even	ok

Compound Spectrum SmartFormula Report

Meas. m/z	#	Ion Formula	m/z	err [ppm]	mSigma	# mSigma	Score	rdb	e⁻ Conf	N-Rule
	147	C58H11N36O13P2S2	515.0069	-0.4	21.9	154	71.95	74.0	even	ok
	148	C69H35N2O35P2S	515.0067	-0.8	22.0	155	64.89	56.0	even	ok
	149	C65H26N10NaO34S	515.0072	0.1	22.0	156	75.95	59.0	even	ok
	150	C69H144N17O15P2S	515.0069	-0.3	22.2	157	72.33	9.0	even	ok
	151	C51H123N47NaO4S2	515.0063	-1.5	22.3	158	53.62	15.0	even	ok
	152	C65H127N33O9P	515.0072	0.2	22.3	159	62.14	21.0	even	ok
	153	C62H7N34O16P2	515.0079	1.6	22.4	160	43.52	79.0	even	ok
	154	C68H155NO33P	515.0068	-0.7	22.4	161	55.92	-6.0	even	ok
	155	C65H137N25NaO12P2	515.0078	1.4	22.5	162	45.80	12.0	even	ok
	156	C66H131N29NaO10S	515.0079	1.5	22.5	163	54.32	17.0	even	ok
	157	C64H126N35O8S	515.0078	1.3	22.5	164	47.52	21.0	even	ok
	158	C70H31N6O31P2S	515.0071	0.0	22.6	165	76.68	61.0	even	ok
	159	C67H3N34NaO13P	515.0079	1.6	22.8	166	43.34	85.0	even	ok
	160	C59H7N40O9P2S2	515.0073	0.5	22.9	167	69.36	79.0	even	ok
	161	C76H28N4NaO29P2	515.0065	-1.1	22.9	168	59.49	67.0	even	ok
	162	C49H118N53O2S2	515.0062	-1.7	23.0	169	50.24	19.0	even	ok
	163	C52H126N43O8S2	515.0066	-0.9	23.2	170	62.82	13.0	even	ok
	164	C54H131N37NaO10S2	515.0067	-0.7	23.3	171	65.55	9.0	even	ok
	165	C67H30N4O39P	515.0069	-0.3	23.3	172	71.54	57.0	even	ok
	166	C56H128N39NaO7PS	515.0066	-0.9	23.3	173	51.40	14.0	even	ok
	167	C62H137N31NaO5P2S2	515.0072	0.3	23.5	174	71.50	12.0	even	ok
	168	C65H144N17O20P2	515.0077	1.3	23.5	175	46.83	5.0	even	ok
	169	C62H144N23O13P2S2	515.0071	0.1	23.7	176	73.90	5.0	even	ok
	170	C62H10N28O23P	515.0065	-1.2	23.7	177	48.00	74.0	even	ok
	171	C64H19N20O26P2	515.0079	1.6	23.7	178	42.15	68.0	even	ok
	172	C60H16N30NaO15P2S2	515.0070	-0.2	23.9	179	71.58	70.0	even	ok
	173	C72H13N18O26	515.0077	1.2	23.9	180	56.14	77.0	even	ok
	174	C69H5N28O20	515.0073	0.4	24.1	181	57.52	83.0	even	ok
	175	C65H155N3NaO32S	515.0065	-1.1	24.1	182	57.96	-9.0	even	ok
	176	C63H150N9O30S	515.0064	-1.3	24.1	184	55.27	-5.0	even	ok
	177	C59H138N27O16S2	515.0080	1.8	24.1	185	48.56	6.0	even	ok
	178	C63H135N25O17P	515.0063	-1.5	24.3	186	42.87	11.0	even	ok
	179	C55H5N40O16S2	515.0077	1.3	24.3	187	55.39	75.0	even	ok
	180	C70H24N14NaO23P2S	515.0072	0.2	24.3	188	71.09	68.0	even	ok
	181	C67H152N5NaO29P	515.0064	-1.4	24.4	189	44.99	-4.0	even	ok
	182	C56H6N38O17PS	515.0072	0.1	24.4	191	59.87	75.0	even	ok
	183	C63H128N33NaO9P	515.0064	-1.4	24.4	192	44.77	18.0	even	ok
	184	C53H132N39NaO7PS2	515.0077	1.3	24.5	193	55.15	9.0	even	ok
	185	C71H149N11NaO17P2S	515.0070	-0.2	24.5	194	71.37	5.0	even	ok
	186	C77H31O33P2	515.0069	-0.4	24.7	195	67.38	65.0	even	ok
	187	C66H29N6O38S	515.0075	0.8	24.8	196	61.09	57.0	even	ok
	188	C50H124N49NaOPS2	515.0073	0.4	24.8	197	67.60	15.0	even	ok
	189	C61H148N19O17P2S2	515.0067	-0.8	24.8	198	61.88	0.0	even	ok
	190	C51H127N45O5PS2	515.0076	1.1	25.0	199	57.13	13.0	even	ok
	191	C63H132N31O10P2	515.0077	1.3	25.1	201	45.30	16.0	even	ok
	192	C72H36NaO33P2S	515.0072	0.2	25.1	202	69.73	57.0	even	ok

Compound Spectrum SmartFormula Report

Meas. m/z	#	Ion Formula	m/z	err [ppm]	mSigma	# mSigma	Score	rdb	e¯ Conf	N-Rule
	193	C55H134N33O14S2	515.0071	0.0	25.1	203	72.65	7.0	even	ok
	194	C71H10N22NaO22	515.0074	0.6	25.3	204	53.70	79.0	even	ok
	195	C64H156N5NaO29PS	515.0075	0.8	25.4	205	60.20	-9.0	even	ok
	196	C61H12N34NaO11P2S2	515.0074	0.6	25.5	206	62.81	75.0	even	ok
	197	C68H36NaO38P2	515.0080	1.8	25.6	207	45.98	53.0	even	ok
	198	C55H3N42NaO13PS	515.0068	-0.5	25.6	208	53.38	77.0	even	ok
	199	C57H15N32O17P2S2	515.0064	-1.3	25.6	209	53.64	69.0	even	ok
	200	C74H18N12NaO28	515.0078	1.4	25.6	210	51.45	73.0	even	ok
	201	C66H7N34O11P2S	515.0071	0.0	25.6	211	71.75	83.0	even	ok
	202	C54H123N45O5PS	515.0065	-1.1	25.7	212	46.60	18.0	even	ok
	203	C57H10N34NaO18S2	515.0078	1.4	25.7	213	51.12	71.0	even	ok
	204	C61H148N15NaO23PS	515.0071	-0.0	25.8	214	71.22	-3.0	even	ok
	205	C57H139N27NaO16S2	515.0072	0.2	25.8	215	68.89	3.0	even	ok
	206	C64H124N37NaO5P	515.0068	-0.5	25.9	216	53.64	23.0	even	ok
	207	C62H151N11O27PS	515.0074	0.7	25.9	217	62.08	-5.0	even	ok
	208	C64H149N17NaO15P2S2	515.0072	0.3	26.0	218	67.35	1.0	even	ok
	209	C68H34NaO40S	515.0076	1.0	26.1	219	56.67	53.0	even	ok
	210	C65H4N38NaO7P2S	515.0067	-0.7	26.1	220	51.19	85.0	even	ok
	211	C61H131N35O6PS2	515.0061	-1.8	26.2	221	45.15	16.0	even	ok
	212	C56H132N37O8P2S	515.0080	1.7	26.2	222	39.16	12.0	even	ok
	213	C61H4N38NaO12P2	515.0076	0.9	26.3	223	47.78	81.0	even	ok
	214	C65H159NO33PS	515.0079	1.5	26.4	224	49.17	-11.0	even	ok
	215	C63H153N13NaO19P2S2	515.0068	-0.6	26.5	225	62.09	-4.0	even	ok
	216	C63H16N24NaO22P2	515.0076	1.0	26.6	226	47.35	70.0	even	ok
	217	C65H147N11O27P	515.0063	-1.5	26.7	227	40.64	0.0	even	ok
	218	C66H27N8NaO35P	515.0066	-1.0	26.7	228	46.98	59.0	even	ok
	219	C59H20N26NaO19P2S2	515.0065	-1.1	26.8	229	54.67	65.0	even	ok
	220	C59H143N21O21PS	515.0070	-0.2	26.9	230	66.80	1.0	even	ok
	221	C61H123N39O7P	515.0063	-1.5	27.0	231	40.18	22.0	even	ok
	222	C75H148N7O21P2	515.0062	-1.7	27.1	232	38.88	8.0	even	ok
	223	C64H141N21NaO16P2	515.0074	0.6	27.1	233	51.22	7.0	even	ok
	224	C63H140N27O9P2S2	515.0076	1.0	27.1	234	56.07	10.0	even	ok
	225	C67H156N3O30P2	515.0077	1.3	27.2	235	42.97	-6.0	even	ok
	226	C72H152N7O21P2S	515.0074	0.5	27.3	236	61.84	3.0	even	ok
	227	C61H19N26O19P2S2	515.0073	0.5	27.6	237	62.23	68.0	even	ok
	228	C71H27N10O27P2S	515.0076	0.9	27.7	238	56.16	66.0	even	ok
	229	C62H119N43O3P	515.0067	-0.7	27.7	239	49.25	27.0	even	ok
	230	C68H12N28NaO13P2S	515.0072	0.2	27.8	240	65.68	79.0	even	ok
	231	C60H6N38O12PS2	515.0063	-1.5	27.9	241	48.16	79.0	even	ok
	232	C61H22N16NaO32S	515.0063	-1.6	28.0	242	46.33	60.0	even	ok
	233	C63H143N21O16PS2	515.0061	-1.8	28.1	243	43.18	5.0	even	ok
	234	C60H3N44O5P2S2	515.0078	1.3	28.3	244	49.54	84.0	even	ok
	235	C58H142N23O20S2	515.0075	0.9	28.3	245	55.40	1.0	even	ok
	236	C53H120N49NaOPS	515.0062	-1.8	28.4	247	36.25	20.0	even	ok
	237	C59H17N22O30S	515.0062	-1.8	28.5	248	43.58	64.0	even	ok
	238	C77H153NNaO23P2	515.0063	-1.5	28.5	249	39.48	4.0	even	ok

Compound Spectrum SmartFormula Report

Meas. m/z	#	Ion Formula	m/z	err [ppm]	mSigma	# mSigma	Score	rdb	e⁻ Conf	N-Rule
	239	C53H6N40NaO16S2	515.0069	-0.3	28.6	250	63.17	72.0	even	ok
	240	C55H129N41NaO4P2S	515.0076	1.0	28.6	251	44.50	14.0	even	ok
	241	C64H22N14O33P	515.0065	-1.2	28.6	252	42.88	63.0	even	ok
	242	C68H128N31NaO7P	515.0077	1.2	28.7	253	41.88	22.0	even	ok
	243	C63H136N29NaO8PS2	515.0062	-1.7	28.8	254	44.67	12.0	even	ok
	244	C63H125N39NaO2P2	515.0078	1.4	29.0	255	39.49	23.0	even	ok
	245	C70H130N25O14	515.0071	-0.0	29.0	256	55.44	20.0	even	ok
	246	C66H123N37O5P	515.0076	1.1	29.2	257	43.40	26.0	even	ok
	247	C61H11N30O20P2	515.0075	0.8	29.2	258	46.53	74.0	even	ok
	248	C62H25N12O36S	515.0066	-0.9	29.2	259	53.98	58.0	even	ok
	249	C62H129N35NaO6P2	515.0074	0.6	29.2	260	48.74	18.0	even	ok
	250	C62H15N30O15P2S2	515.0078	1.3	29.3	261	48.35	73.0	even	ok
	251	C64H30N6NaO38S	515.0067	-0.7	29.3	262	56.23	54.0	even	ok
	252	C52H7N42NaO13PS2	515.0079	1.6	29.4	263	44.35	72.0	even	ok
	253	C60H147N17NaO22S2	515.0076	1.1	29.5	264	51.54	-3.0	even	ok
	254	C66H153N7NaO26P2	515.0074	0.6	29.6	265	48.19	-4.0	even	ok
	255	C66H129N35NaOP2S	515.0066	-1.0	29.6	266	43.06	22.0	even	ok
	256	C67H122N35O8	515.0066	-0.9	29.7	267	44.82	26.0	even	ok
	257	C71H23N12NaO26PS	515.0062	-1.7	29.7	269	42.93	68.0	even	ok
	258	C66H31N6O36P2	515.0079	1.7	29.7	270	36.55	57.0	even	ok
	259	C65H145N21NaO11P2S2	515.0077	1.1	29.7	271	50.30	6.0	even	ok
	260	C63H24N20NaO21P2S2	515.0074	0.7	29.8	272	56.64	64.0	even	ok
	261	C62H136N27O14P2	515.0073	0.4	29.8	273	50.00	11.0	even	ok
	262	C69H127N29NaO10	515.0067	-0.7	29.8	274	46.64	22.0	even	ok
	263	C72H135N19NaO16	515.0072	0.2	29.8	275	52.41	16.0	even	ok
	264	C74H157NNaO23P2S	515.0075	0.7	29.9	276	55.82	-1.0	even	ok
	265	C60H23N22O23P2S2	515.0069	-0.4	29.9	277	59.89	63.0	even	ok
	266	C60H152N15O21P2S2	515.0062	-1.6	29.9	278	43.89	-5.0	even	ok
	267	C65H152N13O19P2S2	515.0076	1.0	29.9	279	52.37	-1.0	even	ok
	268	C76H146N5O26	515.0080	1.7	29.9	280	42.63	8.0	even	ok
	269	C69H137N25NaO7P2S	515.0070	-0.2	30.0	281	62.72	16.0	even	ok
	270	C64H156N9O23P2S2	515.0071	0.1	30.0	282	63.65	-6.0	even	ok
	271	C73H138N15O20	515.0075	0.9	30.1	283	44.53	14.0	even	ok
	272	C60H23N18NaO29PS	515.0073	0.3	30.1	284	60.43	60.0	even	ok
	273	C69H15N24O17P2S	515.0075	0.9	30.2	285	52.91	77.0	even	ok
	274	C58H140N25NaO17PS	515.0066	-0.9	30.3	286	43.82	3.0	even	ok
	275	C73H32N4NaO29P2S	515.0076	1.1	30.3	287	50.39	62.0	even	ok
	276	C54H4N44NaO10P2S	515.0078	1.4	30.3	288	38.71	77.0	even	ok
	277	C63H31N8NaO35PS	515.0077	1.2	30.3	289	48.76	54.0	even	ok
	278	C62H11N32NaO14PS2	515.0064	-1.3	30.5	290	47.45	75.0	even	ok
	279	C54H9N36O20S2	515.0073	0.4	30.5	291	59.00	70.0	even	ok
	280	C63H19N18NaO29P	515.0061	-1.8	30.6	292	33.85	65.0	even	ok
	281	C61H26N14O33PS	515.0076	1.0	30.6	293	50.67	58.0	even	ok
	282	C65H148N15NaO18PS2	515.0062	-1.7	30.6	294	42.80	1.0	even	ok
	283	C63H133N35NaOP2S2	515.0077	1.1	30.7	295	49.25	17.0	even	ok
	284	C67H132N31O5P2S	515.0069	-0.4	30.8	296	49.24	20.0	even	ok

Compound Spectrum SmartFormula Report

Meas. m/z	#	Ion Formula	m/z	err [ppm]	mSigma	# mSigma	Score	rdb	e⁻ Conf	N-Rule
	285	C62H157N9NaO23P2S2	515.0063	-1.5	30.8	297	45.07	-9.0	even	ok
	286	C58H18N24O27PS	515.0072	0.2	30.9	298	61.59	64.0	even	ok
	287	C62H8N38NaO7P2S2	515.0079	1.5	31.0	299	44.27	80.0	even	ok
	288	C53H124N47O2P2S	515.0075	0.8	31.0	300	43.87	18.0	even	ok
	289	C57H15N28NaO23PS	515.0068	-0.5	31.1	301	56.30	66.0	even	ok
	290	C61H120N45P2	515.0077	1.3	31.2	302	39.27	27.0	even	ok
	291	C56H14N30NaO22S2	515.0074	0.6	31.2	303	55.70	66.0	even	ok
	292	C65H33N2O42S	515.0071	-0.0	31.2	304	62.60	52.0	even	ok
	293	C65H28N10NaO32P2	515.0076	1.0	31.4	305	42.14	59.0	even	ok
	294	C62H28N16NaO25P2S2	515.0070	-0.2	31.4	306	59.98	59.0	even	ok
	295	C66H119N39NaO4	515.0063	-1.6	31.5	307	35.81	28.0	even	ok
	296	C75H143N9NaO22	515.0076	1.0	31.5	308	41.05	10.0	even	ok
	297	C62H18N24O22PS2	515.0063	-1.5	31.6	309	44.13	68.0	even	ok
	298	C65H120N41NaOP	515.0073	0.4	31.7	310	48.03	28.0	even	ok
	299	C70H140N21O11P2S	515.0074	0.5	31.8	311	55.63	14.0	even	ok
	300	C64H20N24NaO17P2S2	515.0079	1.5	31.9	312	43.22	69.0	even	ok
	301	C60H124N41O4P2	515.0073	0.4	31.9	313	47.56	22.0	even	ok
	302	C66H161N3NaO25P2S2	515.0072	0.3	31.9	314	58.37	-10.0	even	ok
	303	C64H148N13O24P2	515.0073	0.4	32.0	315	47.33	0.0	even	ok
	304	C56H135N31O15PS	515.0065	-1.1	32.0	316	40.13	7.0	even	ok
	305	C64H114N45O2	515.0062	-1.7	32.0	317	33.61	32.0	even	ok
	306	C53H135N33NaO14S2	515.0063	-1.5	32.2	318	42.63	4.0	even	ok
	307	C67H157N7NaO21P2S2	515.0077	1.1	32.3	319	47.10	-5.0	even	ok
	308	C55H10N34O21PS	515.0067	-0.7	32.4	320	52.30	70.0	even	ok
	309	C64H139N25O12PS2	515.0066	-1.0	32.4	321	49.12	10.0	even	ok
	310	C60H8N34NaO16P2	515.0071	0.1	32.4	322	50.14	76.0	even	ok
	311	C72H11N24O17P2	515.0064	-1.3	32.5	323	37.70	82.0	even	ok
	312	C61H150N13O26S2	515.0080	1.8	32.5	324	39.75	-5.0	even	ok
	313	C51H130N39O12S2	515.0062	-1.7	32.5	325	40.13	8.0	even	ok
	314	C71H20N18NaO19P2S	515.0076	1.1	32.6	326	47.66	73.0	even	ok
	315	C64H119N43NaS	515.0079	1.5	32.6	327	35.69	28.0	even	ok
	316	C73H151N5O24PS	515.0064	-1.4	32.7	328	43.48	3.0	even	ok
	317	C69H11N26NaO16PS	515.0062	-1.7	32.9	329	39.62	79.0	even	ok
	318	C72H26N8O30PS	515.0066	-1.0	33.1	330	47.53	66.0	even	ok
	319	C77H24N8NaO25P2	515.0070	-0.2	33.3	331	56.93	72.0	even	ok
	320	C58H144N23O18P2S	515.0080	1.7	33.3	332	32.86	1.0	even	ok
	321	C54H138N29O18S2	515.0067	-0.8	33.3	333	49.48	2.0	even	ok
	322	C66H34O43P	515.0065	-1.1	33.4	334	45.88	52.0	even	ok
	323	C65H155N7O26PS2	515.0061	-1.8	33.4	335	38.03	-6.0	even	ok
	324	C56H143N23NaO20S2	515.0067	-0.7	33.4	336	51.55	-2.0	even	ok
	325	C66H148N17O15P2S2	515.0080	1.8	33.4	337	38.03	4.0	even	ok
	326	C63H23N16O30P2	515.0075	0.8	33.5	338	41.76	63.0	even	ok
	327	C61H133N31NaO10P2	515.0069	-0.3	33.5	339	46.64	13.0	even	ok
	328	C62H127N39O2PS2	515.0066	-1.0	33.5	340	47.62	21.0	even	ok
	329	C55H132N35NaO11PS	515.0062	-1.8	33.6	341	32.00	9.0	even	ok
	330	C64H27N16O25P2S2	515.0078	1.3	33.6	342	43.39	62.0	even	ok

Compound Spectrum SmartFormula Report

Meas. m/z	#	Ion Formula	m/z	err [ppm]	mSigma	# mSigma	Score	rdb	e¯ Conf	N-Rule
	331	C63H160N5O27P2S2	515.0067	-0.8	33.7	343	50.09	-11.0	even	ok
	332	C57H17N26O26S2	515.0077	1.3	33.7	344	44.12	64.0	even	ok
	333	C74H16N18NaO19P2	515.0065	-1.1	33.8	346	38.21	78.0	even	ok
	334	C64H136N31O5P2S2	515.0080	1.8	33.8	347	37.76	15.0	even	ok
	335	C74H35O33P2S	515.0080	1.8	33.8	348	38.33	60.0	even	ok
	336	C64H23N18NaO24PS2	515.0064	-1.3	33.9	349	43.64	64.0	even	ok
	337	C72H145N15NaO13P2S	515.0074	0.7	34.0	350	50.51	10.0	even	ok
	338	C52H136N35NaO11PS2	515.0073	0.4	34.1	351	53.92	4.0	even	ok
	339	C65H31N4NaO39P	515.0061	-1.8	34.3	352	37.14	54.0	even	ok
	340	C63H14N28O18PS2	515.0068	-0.6	34.3	353	51.21	73.0	even	ok
	341	C55H144N25NaO17PS2	515.0077	1.3	34.4	354	43.27	-2.0	even	ok
	342	C66H151N11O22PS2	515.0066	-1.0	34.5	355	46.63	-1.0	even	ok
	343	C57H141N27NaO14P2S	515.0076	1.0	34.6	356	38.24	3.0	even	ok
	344	C75H19N14O23P2	515.0069	-0.4	34.7	357	44.07	76.0	even	ok
	345	C53H139N31O15PS2	515.0077	1.1	34.7	358	45.00	2.0	even	ok
	346	C59H22N20NaO28S2	515.0078	1.4	34.8	359	40.90	60.0	even	ok
	347	C63H145N17NaO20P2	515.0069	-0.3	34.8	360	45.20	2.0	even	ok
	348	C63H31N12O29P2S2	515.0073	0.5	34.8	361	52.01	57.0	even	ok
	349	C50H131N41O9PS2	515.0072	0.2	34.8	362	55.04	8.0	even	ok
	350	C78H27N4O29P2	515.0073	0.5	34.9	363	52.20	70.0	even	ok
	351	C49H128N45NaO5PS2	515.0068	-0.5	34.9	364	51.92	10.0	even	ok
	352	C63H160NNaO33PS	515.0071	-0.0	35.0	365	56.88	-14.0	even	ok
	353	C66H144N19NaO14PS2	515.0067	-0.8	35.1	366	47.96	6.0	even	ok
	354	C58H3N40O14P2	515.0070	-0.1	35.1	367	46.65	80.0	even	ok
	355	C57H146N19O24S2	515.0071	0.0	35.2	368	56.72	-4.0	even	ok
	356	C59H27N18O27P2S2	515.0064	-1.3	35.3	369	42.40	58.0	even	ok
	357	C72H23N14O23P2S	515.0080	1.8	35.4	370	36.82	71.0	even	ok
	358	C70H14N22O20PS	515.0066	-1.0	35.5	372	44.60	77.0	even	ok
	359	C67H160NNaO28PS2	515.0062	-1.7	35.6	373	37.88	-10.0	even	ok
	360	C62H20N20NaO26P2	515.0071	0.1	35.7	374	46.02	65.0	even	ok
	361	C74H31N2NaO32PS	515.0066	-0.9	35.7	375	46.42	62.0	even	ok
	362	C59H151N13NaO26S2	515.0072	0.2	35.8	376	53.86	-8.0	even	ok
	363	C61H155N7O31PS	515.0070	-0.2	35.8	377	53.69	-10.0	even	ok
	364	C67H3N38O7P2S	515.0075	0.9	35.8	378	45.99	88.0	even	ok
	365	C60H152N11NaO27PS	515.0066	-0.9	35.8	379	45.74	-8.0	even	ok
	366	C66H32N10NaO27P2S2	515.0079	1.5	35.9	380	38.94	58.0	even	ok
	367	C47H123N51O3PS2	515.0068	-0.7	36.0	381	48.35	14.0	even	ok
	368	C59H121N45NaP2	515.0069	-0.3	36.0	382	43.63	24.0	even	ok
	369	C64H132N33NaO4PS2	515.0067	-0.8	36.1	383	46.59	17.0	even	ok
	370	C68H160N3O25P2S2	515.0080	1.8	36.2	384	35.34	-7.0	even	ok
	371	C61H32N12NaO29P2S2	515.0065	-1.1	36.2	385	43.37	54.0	even	ok
	372	C59H128N37O8P2	515.0068	-0.5	36.3	386	41.72	17.0	even	ok
	373	C73H148N11O17P2S	515.0078	1.4	36.3	387	39.94	8.0	even	ok
	374	C63H7N36NaO10PS2	515.0069	-0.4	36.4	388	50.41	80.0	even	ok
	375	C54H7N38NaO17PS	515.0064	-1.4	36.4	389	32.97	72.0	even	ok
	376	C55H136N33O12P2S	515.0075	0.8	36.5	390	38.03	7.0	even	ok

Compound Spectrum SmartFormula Report

Meas. m/z	#	Ion Formula	m/z	err [ppm]	mSigma	# mSigma	Score	rdb	e¯ Conf	N-Rule
	377	C65H36N6NaO31P2S2	515.0074	0.7	36.6	391	47.61	53.0	even	ok
	378	C71H139N19O14PS	515.0064	-1.4	36.8	392	39.12	14.0	even	ok
	379	C72H6N26NaO18	515.0078	1.4	36.8	393	32.57	84.0	even	ok
	380	C65H19N22NaO20PS2	515.0069	-0.4	36.9	394	49.82	69.0	even	ok
	381	C58H147N17O25PS	515.0065	-1.1	37.0	395	42.42	-4.0	even	ok
	382	C68H156N5NaO24PS2	515.0067	-0.8	37.0	396	45.61	-5.0	even	ok
	383	C65H35N2O40P2	515.0075	0.8	37.1	397	45.61	52.0	even	ok
	384	C52H10N36NaO20S2	515.0065	-1.2	37.2	398	41.38	67.0	even	ok
	385	C73H136N21O11P2	515.0062	-1.7	37.3	399	30.08	19.0	even	ok
	386	C61H140N23O18P2	515.0068	-0.5	37.4	400	40.61	6.0	even	ok
	387	C50H5N42O18S2	515.0064	-1.3	37.4	401	39.23	71.0	even	ok
	388	C79H152NO23P2	515.0071	0.1	37.7	403	52.56	7.0	even	ok
	389	C65H26N14O28PS2	515.0068	-0.6	37.8	404	46.80	62.0	even	ok
	390	C64H30N10O32PS2	515.0063	-1.5	37.8	405	37.65	57.0	even	ok
	391	C69H8N32NaO9P2S	515.0076	1.1	37.9	406	41.69	84.0	even	ok
	392	C56H16N30NaO20P2S	515.0078	1.4	37.9	407	31.85	66.0	even	ok
	393	C72H19N16NaO22PS	515.0066	-0.9	38.0	408	43.70	73.0	even	ok
	394	C63H34N2NaO42S	515.0063	-1.6	38.1	409	36.06	49.0	even	ok
	395	C60H154N9O30S2	515.0076	0.9	38.1	410	43.23	-10.0	even	ok
	396	C60H15N26O24P2	515.0070	-0.1	38.1	411	43.22	69.0	even	ok
	397	C75H141N15NaO13P2	515.0063	-1.5	38.3	413	30.76	15.0	even	ok
	398	C61H29N8O40S	515.0062	-1.8	38.3	414	34.03	53.0	even	ok
	399	C58H127N35NaO13	515.0078	1.4	38.4	416	31.65	14.0	even	ok
	400	C54H133N37NaO8P2S	515.0072	0.1	38.4	417	42.31	9.0	even	ok
	401	C53H13N32O24S2	515.0068	-0.5	38.6	418	47.15	65.0	even	ok
	402	C76H144N11O17P2	515.0067	-0.8	38.6	420	36.38	13.0	even	ok
	403	C60H156N9O28P2S	515.0080	1.7	38.7	421	34.25	-10.0	even	ok
	404	C56H115N49NaO3	515.0078	1.4	38.7	422	31.48	25.0	even	ok
	405	C75H153N5NaO19P2S	515.0079	1.6	38.7	423	35.68	4.0	even	ok
	406	C67H147N15O18PS2	515.0070	-0.1	38.7	424	50.85	4.0	even	ok
	407	C55H18N26NaO26S2	515.0069	-0.3	38.7	425	48.84	61.0	even	ok
	408	C51H11N38NaO17PS2	515.0075	0.8	38.8	426	43.69	67.0	even	ok
	409	C62H159N3NaO32S2	515.0076	1.1	39.0	427	40.34	-14.0	even	ok
	410	C62H35N8O33P2S2	515.0069	-0.4	39.0	428	47.50	52.0	even	ok
	411	C48H3N48NaO11PS2	515.0070	-0.1	39.0	429	50.47	73.0	even	ok
	412	C73H144N13NaO16PS	515.0065	-1.2	39.1	430	38.60	10.0	even	ok
	413	C59H153N13NaO24P2S	515.0076	1.0	39.1	431	40.69	-8.0	even	ok
	414	C65H135N29O8PS2	515.0070	-0.1	39.3	432	50.02	15.0	even	ok
	415	C49H6N44O15PS2	515.0074	0.6	39.3	433	44.92	71.0	even	ok
	416	C65H157N3NaO30P2	515.0069	-0.3	39.5	434	40.00	-9.0	even	ok
	417	C54H11N36O18P2S	515.0077	1.2	39.5	435	31.96	70.0	even	ok
	418	C54H19N28NaO23PS2	515.0079	1.6	39.6	436	34.15	61.0	even	ok
	419	C79H26N2O32P	515.0063	-1.5	39.6	437	35.63	70.0	even	ok
	420	C52H14N34O21PS2	515.0078	1.5	39.7	438	35.75	65.0	even	ok
	421	C62H35N4NaO39PS	515.0073	0.3	39.7	439	47.05	49.0	even	ok
	422	C62H164NO31P2S2	515.0063	-1.6	39.8	440	34.05	-16.0	even	ok

Compound Spectrum SmartFormula Report

Meas. m/z	#	Ion Formula	m/z	err [ppm]	mSigma	# mSigma	Score	rdb	e⁻ Conf	N-Rule
	423	C66H35N4NaO34PS2	515.0064	-1.3	39.8	441	37.41	53.0	even	ok
	424	C70H11N28O13P2S	515.0080	1.8	39.9	442	32.76	82.0	even	ok
	425	C64H10N32O14PS2	515.0072	0.3	39.9	443	47.59	78.0	even	ok
	426	C59H27N14NaO33PS	515.0068	-0.5	40.0	444	44.75	55.0	even	ok
	427	C68H118N39O4	515.0071	-0.0	40.0	445	41.68	31.0	even	ok
	428	C58H125N41NaO4P2	515.0065	-1.2	40.1	446	31.87	19.0	even	ok
	429	C66H39N2O35P2S2	515.0078	1.3	40.1	447	36.42	51.0	even	ok
	430	C78H149N5NaO19P2	515.0068	-0.6	40.2	448	36.42	9.0	even	ok
	431	C64H40N2NaO35P2S2	515.0070	-0.2	40.2	449	47.78	48.0	even	ok
	432	C71H126N29O10	515.0075	0.9	40.2	450	34.28	25.0	even	ok
	433	C67H31N8NaO30PS2	515.0069	-0.4	40.2	451	45.66	58.0	even	ok
	434	C60H30N10O37PS	515.0072	0.2	40.3	452	48.18	53.0	even	ok
	435	C49H115N55NaOS	515.0080	1.8	40.5	453	26.53	21.0	even	ok
	436	C60H137N27NaO14P2	515.0065	-1.2	40.5	454	31.58	8.0	even	ok
	437	C63H38O43PS	515.0076	1.0	40.6	455	39.05	47.0	even	ok
	438	C70H123N33NaO6	515.0072	0.2	40.6	456	39.71	27.0	even	ok
	439	C52H128N43O6P2S	515.0071	-0.0	40.6	458	40.85	13.0	even	ok
	440	C56H21N22O30S2	515.0073	0.4	40.6	459	45.30	59.0	even	ok
	441	C66H22N18O24PS2	515.0072	0.3	40.8	460	46.42	67.0	even	ok
	442	C59H12N30NaO20P2	515.0067	-0.8	40.8	461	34.41	71.0	even	ok
	443	C53H8N40NaO14P2S	515.0074	0.5	40.9	462	36.44	72.0	even	ok
	444	C73H22N12O26PS	515.0070	-0.2	40.9	463	47.23	71.0	even	ok
	445	C57H22N20O31PS	515.0067	-0.7	40.9	464	41.83	59.0	even	ok
	446	C69H159NO28PS2	515.0070	-0.1	40.9	465	47.97	-7.0	even	ok
	447	C56H122N41O11	515.0077	1.2	41.1	466	30.85	18.0	even	ok
	448	C58H26N16NaO32S2	515.0074	0.6	41.2	467	42.82	55.0	even	ok
	449	C56H19N24NaO27PS	515.0064	-1.4	41.2	468	34.87	61.0	even	ok
	450	C67H115N43Na	515.0067	-0.7	41.2	469	34.65	33.0	even	ok
	451	C57H144N21NaO21PS	515.0062	-1.8	41.2	470	26.15	-2.0	even	ok
	452	C73H131N23NaO12	515.0076	1.0	41.3	471	31.81	21.0	even	ok
	453	C69H152N9NaO20PS2	515.0071	0.1	41.4	472	47.49	0.0	even	ok
	454	C60H139N21NaO23	515.0078	1.4	41.4	473	29.09	3.0	even	ok
	455	C54H110N55O	515.0077	1.2	41.5	474	30.60	29.0	even	ok
	456	C74H147N9O20PS	515.0068	-0.5	41.6	475	42.64	8.0	even	ok
	457	C63H152N9O28P2	515.0069	-0.5	41.7	476	36.19	-5.0	even	ok
	458	C74H134N19O16	515.0080	1.7	41.7	477	26.15	19.0	even	ok
	459	C48H129N47NaO2P2S2	515.0078	1.5	41.8	478	28.18	10.0	even	ok
	460	C47H4N50NaO8P2S2	515.0080	1.8	41.9	479	30.33	73.0	even	ok
	461	C67H140N23NaO10PS2	515.0071	0.1	41.9	480	46.89	11.0	even	ok
	462	C64H32N6NaO36P2	515.0071	0.1	41.9	481	38.87	54.0	even	ok
	463	C71H136N25O7P2S	515.0078	1.4	42.1	482	34.22	19.0	even	ok
	464	C55H147N19NaO24S2	515.0063	-1.5	42.2	483	32.73	-7.0	even	ok
	465	C68H128N35OP2S	515.0074	0.5	42.4	484	35.06	25.0	even	ok
	466	C53H142N25O22S2	515.0062	-1.7	42.5	485	30.91	-3.0	even	ok
	467	C54H14N30O25PS	515.0063	-1.6	42.5	486	32.05	65.0	even	ok
	468	C66H15N26NaO16PS2	515.0073	0.4	42.6	487	42.42	74.0	even	ok

Compound Spectrum SmartFormula Report

Meas. m/z	#	Ion Formula	m/z	err [ppm]	mSigma	# mSigma	Score	rdb	e⁻ Conf	N-Rule
	469	C70H7N30NaO12PS	515.0066	-0.9	42.8	488	38.23	84.0	even	ok
	470	C51H3N46O12P2S	515.0073	0.3	42.8	489	35.95	76.0	even	ok
	471	C58H28N16NaO30P2S	515.0078	1.4	42.9	490	33.27	55.0	even	ok
	472	C56H120N47O2P2	515.0064	-1.3	42.9	491	28.15	23.0	even	ok
	473	C51H125N47NaO2P2S	515.0067	-0.7	43.0	492	32.82	15.0	even	ok
	474	C59H14N24NaO29	515.0080	1.7	43.0	493	25.08	66.0	even	ok
	475	C69H127N33O4PS	515.0064	-1.4	43.1	494	32.83	25.0	even	ok
	476	C58H132N33O12P2	515.0064	-1.3	43.1	495	27.98	12.0	even	ok
	477	C46H124N53P2S2	515.0077	1.3	43.3	496	28.35	14.0	even	ok
	478	C57H7N36O18P2	515.0066	-1.0	43.3	497	30.70	75.0	even	ok
	479	C68H27N12NaO26PS2	515.0073	0.5	43.4	498	41.40	63.0	even	ok
	480	C75H27N6NaO28PS	515.0071	0.0	43.5	499	45.45	67.0	even	ok
	481	C59H29N12O36S2	515.0077	1.3	43.5	500	33.83	53.0	even	ok
	482	C56H150N15O28S2	515.0067	-0.8	43.5	501	37.71	-9.0	even	ok
	483	C67H38O38PS2	515.0068	-0.6	43.5	502	40.02	51.0	even	ok
	484	C58H155N9NaO30S2	515.0068	-0.7	43.6	503	39.24	-13.0	even	ok
	485	C54H148N21NaO21PS2	515.0073	0.4	43.8	504	41.38	-7.0	even	ok
	486	C58H134N27O21	515.0077	1.2	43.8	505	28.51	7.0	even	ok
	487	C62H27N12O34P2	515.0070	-0.1	43.9	506	36.88	58.0	even	ok
	488	C72H4N32NaO9P2	515.0065	-1.1	43.9	507	29.02	89.0	even	ok
	489	C51H140N31NaO15PS2	515.0068	-0.5	44.0	508	40.61	-1.0	even	ok
	490	C62H149N13NaO24P2	515.0065	-1.2	44.0	509	28.70	-3.0	even	ok
	491	C56H23N22O28P2S	515.0077	1.2	44.0	510	33.77	59.0	even	ok
	492	C76H152N3NaO22PS	515.0069	-0.4	44.1	511	41.43	4.0	even	ok
	493	C73H7N28O13P2	515.0069	-0.4	44.1	512	34.05	87.0	even	ok
	494	C68H34N4O34PS2	515.0072	0.3	44.1	513	42.18	56.0	even	ok
	495	C73H141N19NaO9P2S	515.0079	1.6	44.1	514	30.74	15.0	even	ok
	496	C64H3N40NaO6PS2	515.0073	0.4	44.3	515	40.46	85.0	even	ok
	497	C57H148N19O22P2S	515.0075	0.8	44.3	516	30.65	-4.0	even	ok
	498	C70H133N29NaO3P2S	515.0074	0.7	44.3	517	31.82	21.0	even	ok
	499	C52H143N27O19PS2	515.0072	0.2	44.4	518	42.42	-3.0	even	ok
	500	C61H34N6NaO38S2	515.0078	1.5	44.4	519	31.44	49.0	even	ok
529.6821	1	C48H124N55NaPS2	529.6818	-0.6	7.4	1	73.34	16.0	even	ok
	2	C68H38O44P	529.6819	-0.4	7.7	2	91.74	52.0	even	ok
	3	C67H35N4NaO40P	529.6815	-1.1	7.8	3	77.54	54.0	even	ok
	4	C59H148N21NaO22PS	529.6816	-1.0	7.9	4	78.55	-2.0	even	ok
	5	C67H39N2O41P2	529.6829	1.4	7.9	5	70.49	52.0	even	ok
	6	C57H14N34O22PS	529.6821	-0.0	8.0	6	100.00	70.0	even	ok
	7	C56H11N38NaO18PS	529.6818	-0.7	8.0	7	85.79	72.0	even	ok
	8	C49H127N51O4PS2	529.6822	0.0	8.4	8	98.65	14.0	even	ok
	9	C60H151N17O26PS	529.6819	-0.4	8.5	9	91.58	-4.0	even	ok
	10	C65H30N10O38P	529.6815	-1.3	8.6	10	72.84	58.0	even	ok
	11	C54H6N44O16PS	529.6817	-0.9	8.7	11	67.64	76.0	even	ok
	12	C57H143N27O20PS	529.6815	-1.2	8.8	12	73.80	2.0	even	ok
	13	C59H19N28NaO24PS	529.6822	0.2	9.2	13	94.92	66.0	even	ok
	14	C59H152N19O23P2S	529.6829	1.5	9.3	14	67.57	-4.0	even	ok

Compound Spectrum SmartFormula Report

Meas. m/z	#	Ion Formula	m/z	err [ppm]	mSigma	# mSigma	Score	rdb	e¯ Conf	N-Rule
	15	C62H156N11NaO28PS	529.6820	-0.2	9.4	15	93.69	-8.0	even	ok
	16	C51H132N45NaO6PS2	529.6822	0.2	9.5	16	93.12	10.0	even	ok
	17	C66H36N6NaO37P2	529.6825	0.8	9.9	17	81.21	54.0	even	ok
	18	C55H12N40NaO15P2S	529.6828	1.2	10.0	18	60.67	72.0	even	ok
	19	C58H139N31O16PS	529.6819	-0.4	10.3	19	73.68	7.0	even	ok
	20	C58H23N24NaO28PS	529.6818	-0.7	10.6	20	81.92	61.0	even	ok
	21	C57H140N33O13P2S	529.6829	1.5	10.7	21	55.01	7.0	even	ok
	22	C60H144N25NaO18PS	529.6820	-0.2	10.7	22	91.37	3.0	even	ok
	23	C58H149N23NaO19P2S	529.6826	0.8	10.8	23	65.59	-2.0	even	ok
	24	C56H18N30O26PS	529.6817	-0.9	10.9	24	78.04	65.0	even	ok
	25	C68H160NNaO34P	529.6814	-1.5	11.3	26	54.79	-9.0	even	ok
	26	C55H15N34NaO22PS	529.6813	-1.5	11.3	27	64.67	67.0	even	ok
	27	C60H22N24O28PS	529.6826	0.8	11.5	28	77.70	64.0	even	ok
	28	C61H147N21O22PS	529.6824	0.5	11.6	29	84.69	1.0	even	ok
	29	C53H3N48NaO12PS	529.6813	-1.5	11.7	30	53.45	78.0	even	ok
	30	C50H123N55PS2	529.6826	0.9	11.7	31	76.33	19.0	even	ok
	31	C57H136N35NaO12PS	529.6816	-1.1	12.0	33	60.57	9.0	even	ok
	32	C53H7N46O13P2S	529.6827	1.0	12.0	34	61.09	76.0	even	ok
	33	C63H159N7O32PS	529.6824	0.5	12.1	35	83.77	-10.0	even	ok
	34	C52H135N41O10PS2	529.6826	0.9	12.2	36	75.50	8.0	even	ok
	35	C64H31N12O35P2	529.6825	0.6	12.2	37	67.55	58.0	even	ok
	36	C59H26N20O32PS	529.6821	-0.0	12.3	38	92.53	59.0	even	ok
	37	C57H24N26NaO25P2S	529.6828	1.2	12.4	39	69.39	61.0	even	ok
	38	C56H144N29O17P2S	529.6825	0.7	12.9	40	65.80	2.0	even	ok
	39	C50H7N48NaO12PS2	529.6824	0.6	13.0	41	80.10	73.0	even	ok
	40	C55H131N41O10PS	529.6815	-1.2	13.0	42	56.67	13.0	even	ok
	41	C67H32N10NaO33P2	529.6830	1.6	13.2	43	50.68	59.0	even	ok
	42	C61H31N14NaO34PS	529.6822	0.2	13.2	44	87.78	55.0	even	ok
	43	C63H152N15NaO24PS	529.6825	0.6	13.3	45	78.58	-3.0	even	ok
	44	C66H26N14O34P	529.6819	-0.4	13.4	46	82.21	63.0	even	ok
	45	C62H27N18NaO30PS	529.6827	1.0	13.5	47	71.41	60.0	even	ok
	46	C67H161N3NaO31P2	529.6824	0.4	13.5	48	68.84	-9.0	even	ok
	47	C55H19N32O23P2S	529.6827	1.0	13.6	49	71.03	65.0	even	ok
	48	C56H137N37NaO9P2S	529.6826	0.8	13.8	50	61.96	9.0	even	ok
	49	C68H31N8NaO36P	529.6820	-0.3	13.9	51	84.83	59.0	even	ok
	50	C65H164NNaO34PS	529.6825	0.6	13.9	52	77.54	-14.0	even	ok
	51	C65H27N16O31P2	529.6829	1.4	13.9	54	52.56	63.0	even	ok
	52	C51H3N52NaO8PS2	529.6829	1.4	14.1	55	63.14	78.0	even	ok
	53	C50H136N41NaO10PS2	529.6818	-0.6	14.1	56	77.73	5.0	even	ok
	54	C54H140N35NaO12PS2	529.6827	1.1	14.1	57	69.52	4.0	even	ok
	55	C52H128N49NaO2PS2	529.6827	1.1	14.1	58	69.63	15.0	even	ok
	56	C69H34N4O40P	529.6823	0.4	14.2	59	81.65	57.0	even	ok
	57	C47H128N51NaO4PS2	529.6814	-1.5	14.3	60	62.15	11.0	even	ok
	58	C48H131N47O8PS2	529.6817	-0.8	14.3	61	74.13	9.0	even	ok
	59	C61H160N7NaO32PS	529.6816	-1.0	14.7	62	69.13	-13.0	even	ok
	60	C59H155N13O30PS	529.6815	-1.2	15.1	63	65.59	-9.0	even	ok

Compound Spectrum SmartFormula Report

Meas. m/z	#	Ion Formula	m/z	err [ppm]	mSigma	# mSigma	Score	rdb	e⁻ Conf	N-Rule
	61	C58H10N38O18PS	529.6826	0.8	15.1	64	72.55	75.0	even	ok
	62	C65H156N9O29P2	529.6823	0.2	15.6	66	68.77	-5.0	even	ok
	63	C64H158N5O35S	529.6814	-1.4	15.6	67	61.55	-10.0	even	ok
	64	C57H7N42NaO14PS	529.6822	0.2	15.6	68	69.98	77.0	even	ok
	65	C52H4N50NaO9P2S	529.6823	0.3	15.6	69	67.04	78.0	even	ok
	66	C62H146N19O25S	529.6814	-1.4	15.7	70	61.38	1.0	even	ok
	67	C64H155N11O28PS	529.6828	1.3	15.7	71	63.10	-5.0	even	ok
	68	C54H132N43O7P2S	529.6825	0.7	15.7	72	62.28	13.0	even	ok
	69	C51H6N46NaO15S2	529.6814	-1.3	15.7	73	63.32	73.0	even	ok
	70	C51H139N37O14PS2	529.6822	0.1	15.7	74	85.67	3.0	even	ok
	71	C61H21N22O31S	529.6816	-1.0	15.8	75	67.59	64.0	even	ok
	72	C65H40N2NaO41P2	529.6821	-0.1	15.8	76	85.30	49.0	even	ok
	73	C49H137N43NaO7P2S2	529.6828	1.3	15.8	77	63.86	5.0	even	ok
	74	C63H28N16NaO31P2	529.6821	-0.1	15.8	78	70.92	60.0	even	ok
	75	C65H23N18NaO30P	529.6815	-1.1	15.8	79	55.32	65.0	even	ok
	76	C55H141N33NaO13P2S	529.6821	-0.0	15.9	80	71.77	4.0	even	ok
	77	C53H134N39O13S2	529.6816	-1.0	15.9	81	68.39	8.0	even	ok
	78	C51H10N44O16PS2	529.6828	1.3	16.0	82	63.68	71.0	even	ok
	79	C54H16N36NaO19P2S	529.6823	0.4	16.0	83	66.41	67.0	even	ok
	80	C62H34N10O38PS	529.6826	0.8	16.1	84	70.83	53.0	even	ok
	81	C62H163N3O36PS	529.6819	-0.4	16.3	85	79.00	-15.0	even	ok
	82	C53H144N31NaO16PS2	529.6823	0.2	16.5	86	81.21	-1.0	even	ok
	83	C51H122N53O3S2	529.6816	-1.0	16.5	87	67.51	19.0	even	ok
	84	C60H15N32NaO20PS	529.6827	1.0	16.6	88	67.33	71.0	even	ok
	85	C60H161N9NaO29P2S	529.6826	0.8	16.6	89	70.08	-13.0	even	ok
	86	C63H18N24O28P	529.6815	-1.3	16.6	90	51.94	69.0	even	ok
	87	C47H132N49O5P2S2	529.6827	1.1	16.8	91	65.63	9.0	even	ok
	88	C64H24N20NaO27P2	529.6825	0.8	16.9	92	59.12	65.0	even	ok
	89	C61H164N5O33P2S	529.6829	1.5	17.0	93	58.04	-15.0	even	ok
	90	C58H156N15O27P2S	529.6825	0.7	17.6	95	71.71	-9.0	even	ok
	91	C70H159NO34P	529.6822	0.0	17.7	96	82.41	-6.0	even	ok
	92	C53H15N38NaO18PS2	529.6829	1.4	17.7	97	58.55	67.0	even	ok
	93	C64H151N13NaO27S	529.6815	-1.2	17.7	98	61.72	-3.0	even	ok
	94	C64H39N4NaO40PS	529.6827	1.0	17.8	99	65.47	49.0	even	ok
	95	C66H160N5NaO30PS	529.6829	1.5	17.9	100	57.40	-9.0	even	ok
	96	C70H33N2O43	529.6814	-1.5	18.0	101	57.56	57.0	even	ok
	97	C46H3N54NaO10PS2	529.6815	-1.1	18.1	102	63.54	74.0	even	ok
	98	C63H26N16NaO33S	529.6817	-0.9	18.1	103	67.43	60.0	even	ok
	99	C55H139N33NaO15S2	529.6817	-0.8	18.2	104	68.27	4.0	even	ok
	100	C52H11N42O17P2S	529.6822	0.2	18.2	105	66.03	71.0	even	ok
	101	C61H23N22O29P2	529.6820	-0.2	18.3	106	64.82	64.0	even	ok
	102	C53H136N39O11P2S	529.6820	-0.2	18.4	107	65.64	8.0	even	ok
	103	C57H153N19NaO23P2S	529.6821	-0.0	18.4	108	81.80	-7.0	even	ok
	104	C62H19N26O25P2	529.6824	0.6	18.5	109	59.71	69.0	even	ok
	105	C53H127N47NaO5S2	529.6817	-0.8	18.6	110	67.62	15.0	even	ok
	106	C68H157N7NaO27P2	529.6828	1.3	18.9	112	50.08	-4.0	even	ok

Compound Spectrum SmartFormula Report

Meas. m/z	#	Ion Formula	m/z	err [ppm]	mSigma	# mSigma	Score	rdb	e¯ Conf	N-Rule
	107	C57H27N20NaO32PS	529.6813	-1.5	18.9	113	55.74	56.0	even	ok
	108	C47H6N50O14PS2	529.6819	-0.4	18.9	114	73.76	72.0	even	ok
	109	C64H160N5O33P2	529.6818	-0.6	18.9	115	58.96	-10.0	even	ok
	110	C59H9N36O21S	529.6816	-1.1	18.9	116	63.24	75.0	even	ok
	111	C46H129N53NaOP2S2	529.6824	0.4	18.9	117	61.69	11.0	even	ok
	112	C49H11N44NaO16PS2	529.6820	-0.3	18.9	118	76.67	68.0	even	ok
	113	C63H35N8O39P2	529.6820	-0.2	19.0	119	64.05	53.0	even	ok
	114	C63H33N8O41S	529.6816	-1.0	19.0	120	63.26	53.0	even	ok
	115	C52H9N42O19S2	529.6818	-0.6	19.1	121	70.11	71.0	even	ok
	116	C54H147N27O20PS2	529.6826	0.9	19.1	122	65.52	-3.0	even	ok
	117	C64H153N13NaO25P2	529.6819	-0.4	19.4	123	60.66	-3.0	even	ok
	118	C53H129N47NaO3P2S	529.6821	-0.0	19.5	124	66.49	15.0	even	ok
	119	C67H151N11O28P	529.6817	-0.8	19.6	125	55.37	0.0	even	ok
	120	C60H35N10NaO38PS	529.6818	-0.7	19.6	126	68.60	50.0	even	ok
	121	C58H30N16O36PS	529.6817	-0.9	19.6	127	65.67	54.0	even	ok
	122	C69H156N5NaO30P	529.6818	-0.6	19.6	128	57.74	-4.0	even	ok
	123	C66H152N13O25P2	529.6827	1.1	19.6	129	51.64	0.0	even	ok
	124	C60H22N20NaO34	529.6830	1.5	19.6	130	45.45	61.0	even	ok
	125	C61H140N29NaO14PS	529.6825	0.6	19.6	131	69.17	8.0	even	ok
	126	C59H135N35O12PS	529.6824	0.5	20.0	132	59.61	12.0	even	ok
	127	C48H12N46NaO13P2S2	529.6830	1.6	20.1	133	52.76	68.0	even	ok
	128	C45H4N56NaO7P2S2	529.6825	0.8	20.3	134	65.87	74.0	even	ok
	129	C55H128N47O3P2S	529.6829	1.5	20.3	135	45.40	18.0	even	ok
	130	C62H143N25O18PS	529.6828	1.3	20.4	136	57.25	6.0	even	ok
	131	C65H154N9O31S	529.6818	-0.6	20.4	137	69.15	-5.0	even	ok
	132	C56H127N45O6PS	529.6819	-0.4	20.5	138	59.98	18.0	even	ok
	133	C56H152N21NaO22PS2	529.6827	1.1	20.5	139	60.80	-7.0	even	ok
	134	C50H123N51NaO6S	529.6830	1.6	20.6	140	43.84	16.0	even	ok
	135	C46H7N52O11P2S2	529.6829	1.4	20.6	141	54.81	72.0	even	ok
	136	C58H132N39NaO8PS	529.6820	-0.2	20.6	142	62.22	14.0	even	ok
	137	C61H14N30NaO23S	529.6817	-0.9	20.8	143	63.57	71.0	even	ok
	138	C50H14N40O20PS2	529.6824	0.4	20.8	144	71.02	66.0	even	ok
	139	C59H36N12NaO35P2S	529.6828	1.2	20.9	145	58.28	50.0	even	ok
	140	C51H8N46NaO13P2S	529.6819	-0.5	21.0	146	57.93	73.0	even	ok
	141	C61H147N17NaO28	529.6828	1.2	21.0	147	48.82	-2.0	even	ok
	142	C65H38N2NaO43S	529.6817	-0.9	21.1	148	63.33	49.0	even	ok
	143	C54H130N43O9S2	529.6821	-0.1	21.2	149	74.82	13.0	even	ok
	144	C54H14N36NaO21S2	529.6819	-0.4	21.2	150	69.85	67.0	even	ok
	145	C64H29N12O37S	529.6820	-0.2	21.3	151	73.69	58.0	even	ok
	146	C52H135N37NaO16S	529.6830	1.6	21.4	152	43.05	5.0	even	ok
	147	C61H38N6O42PS	529.6821	-0.0	21.4	153	76.84	48.0	even	ok
	148	C55H146N25O23S2	529.6816	-1.0	21.4	154	61.06	-3.0	even	ok
	149	C57H31N18O33P2S	529.6827	1.0	21.5	155	60.27	54.0	even	ok
	150	C58H10N34NaO24	529.6829	1.5	21.5	156	43.72	72.0	even	ok
	151	C56H28N22NaO29P2S	529.6823	0.4	21.5	157	70.87	56.0	even	ok
	152	C52H19N34NaO22PS2	529.6824	0.6	21.6	158	67.03	62.0	even	ok

Compound Spectrum SmartFormula Report

Meas. m/z	#	Ion Formula	m/z	err [ppm]	mSigma	# mSigma	Score	rdb	e¯ Conf	N-Rule
	153	C66H148N15NaO24P	529.6814	-1.5	21.6	159	44.33	2.0	even	ok
	154	C62H32N12NaO35P2	529.6816	-0.9	21.7	160	51.52	55.0	even	ok
	155	C55H148N25O21P2S	529.6820	-0.2	21.7	161	61.25	-3.0	even	ok
	156	C56H142N29O19S2	529.6821	-0.1	21.7	162	74.10	2.0	even	ok
	157	C60H134N33O15S	529.6814	-1.4	21.7	163	53.95	12.0	even	ok
	158	C51H124N53OP2S	529.6820	-0.2	21.7	164	60.97	19.0	even	ok
	159	C51H13N38O23S2	529.6814	-1.5	21.7	165	53.23	66.0	even	ok
	160	C62H148N19O23P2	529.6818	-0.6	21.8	166	55.34	1.0	even	ok
	161	C52H133N43NaO7P2S	529.6817	-0.9	21.9	167	51.98	10.0	even	ok
	162	C53H5N46O15S2	529.6822	0.2	21.9	168	72.55	76.0	even	ok
	163	C64H148N19NaO20PS	529.6829	1.5	22.1	169	52.59	2.0	even	ok
	164	C63H43NaO44PS	529.6822	0.2	22.1	170	72.97	44.0	even	ok
	165	C61H16N30NaO21P2	529.6821	-0.1	22.1	171	62.04	71.0	even	ok
	166	C63H159N3NaO38	529.6828	1.2	22.1	172	47.52	-13.0	even	ok
	167	C58H17N26O32	529.6829	1.4	22.2	173	45.21	65.0	even	ok
	168	C60H20N26NaO25P2	529.6816	-0.9	22.2	174	50.85	66.0	even	ok
	169	C62H34N6NaO44	529.6830	1.5	22.2	175	51.47	50.0	even	ok
	170	C63H157N9NaO29P2	529.6815	-1.3	22.4	176	46.09	-8.0	even	ok
	171	C55H124N49NaO2PS	529.6816	-1.1	22.4	177	48.79	20.0	even	ok
	172	C65H149N17NaO21P2	529.6823	0.4	22.6	178	57.13	2.0	even	ok
	173	C54H23N28O27P2S	529.6822	0.2	22.8	179	71.74	60.0	even	ok
	174	C67H159N3NaO33S	529.6819	-0.4	22.8	180	68.42	-9.0	even	ok
	175	C49H140N37NaO14PS2	529.6814	-1.5	22.8	181	52.04	0.0	even	ok
	176	C62H17N26O27S	529.6820	-0.2	22.9	182	71.10	69.0	even	ok
	177	C53H18N32NaO25S2	529.6814	-1.3	23.0	183	54.31	62.0	even	ok
	178	C53H119N55PS	529.6815	-1.2	23.1	184	45.83	24.0	even	ok
	179	C62H139N27NaO17S	529.6815	-1.2	23.2	185	54.82	8.0	even	ok
	180	C48H118N57O4S	529.6829	1.4	23.2	186	43.53	20.0	even	ok
	181	C57H151N19NaO25S2	529.6817	-0.8	23.2	187	61.33	-7.0	even	ok
	182	C69H27N12NaO32P	529.6824	0.6	23.4	188	64.70	64.0	even	ok
	183	C49H3N52O11P2S	529.6818	-0.7	23.4	189	52.60	77.0	even	ok
	184	C56H135N37NaO11S2	529.6821	0.0	23.5	190	72.92	9.0	even	ok
	185	C59H142N23O26	529.6827	1.0	23.6	191	48.26	2.0	even	ok
	186	C52H148N27NaO20PS2	529.6818	-0.6	23.6	192	63.69	-6.0	even	ok
	187	C50H143N33O18PS2	529.6817	-0.8	23.7	193	60.98	-2.0	even	ok
	188	C50H130N43O14S	529.6829	1.4	23.7	194	42.97	9.0	even	ok
	189	C54H145N29NaO17P2S	529.6817	-0.9	23.7	195	49.94	-1.0	even	ok
	190	C70H30N8O36P	529.6828	1.2	23.8	196	54.03	62.0	even	ok
	191	C66H34N6NaO39S	529.6821	-0.0	23.8	197	72.51	54.0	even	ok
	192	C60H29N12O42	529.6829	1.4	24.0	198	52.03	54.0	even	ok
	193	C63H144N23O19P2	529.6823	0.2	24.0	199	57.56	6.0	even	ok
	194	C56H5N40O22	529.6829	1.4	24.0	200	43.46	76.0	even	ok
	195	C65H20N24NaO23P2	529.6830	1.6	24.1	201	40.46	70.0	even	ok
	196	C58H147N23NaO21S2	529.6822	0.0	24.1	202	71.94	-2.0	even	ok
	197	C59H11N36O19P2	529.6820	-0.3	24.1	203	57.08	75.0	even	ok
	198	C60H27N18O33P2	529.6816	-1.1	24.1	204	46.60	59.0	even	ok

Compound Spectrum SmartFormula Report

Meas. m/z	#	Ion Formula	m/z	err [ppm]	mSigma	# mSigma	Score	rdb	e⁻ Conf	N-Rule
	199	C53H22N30O26PS2	529.6828	1.3	24.3	205	53.25	60.0	even	ok
	200	C67H22N18O30P	529.6823	0.4	24.3	206	54.98	68.0	even	ok
	201	C55H10N40NaO17S2	529.6823	0.4	24.4	207	66.02	72.0	even	ok
	202	C50H128N49O5P2S	529.6816	-1.0	24.5	208	46.88	14.0	even	ok
	203	C61H154N9O36	529.6827	1.0	24.5	209	47.24	-9.0	even	ok
	204	C63H142N23O21S	529.6818	-0.6	24.6	210	63.02	6.0	even	ok
	205	C63H15N30O21P2	529.6829	1.4	24.6	211	42.03	74.0	even	ok
	206	C58H15N32O23P2	529.6816	-1.1	24.8	212	45.86	70.0	even	ok
	207	C55H17N32O25S2	529.6823	0.2	24.9	213	67.84	65.0	even	ok
	208	C51H149N29NaO17P2S2	529.6828	1.3	24.9	214	52.42	-6.0	even	ok
	209	C64H14N28O24P	529.6819	-0.4	25.0	215	53.59	74.0	even	ok
	210	C61H152N15O27P2	529.6814	-1.5	25.0	216	41.39	-4.0	even	ok
	211	C66H19N22NaO26P	529.6820	-0.3	25.1	217	55.62	70.0	even	ok
	212	C64H22N20NaO29S	529.6821	-0.0	25.1	218	70.25	65.0	even	ok
	213	C48H141N39NaO11P2S2	529.6824	0.4	25.2	219	64.33	0.0	even	ok
	214	C72H35N2NaO38P	529.6829	1.4	25.3	220	49.79	58.0	even	ok
	215	C68H21N16O33	529.6813	-1.5	25.3	221	48.92	68.0	even	ok
	216	C54H147N23NaO26S	529.6830	1.6	25.3	222	47.17	-6.0	even	ok
	217	C53H151N23O24PS2	529.6822	0.1	25.4	223	69.48	-8.0	even	ok
	218	C49H144N35O15P2S2	529.6827	1.1	25.4	224	54.30	-2.0	even	ok
	219	C55H27N24NaO28PS2	529.6829	1.4	25.5	225	49.29	56.0	even	ok
	220	C57H14N30NaO28	529.6825	0.7	25.5	226	49.88	67.0	even	ok
	221	C59H135N31NaO18	529.6828	1.2	25.7	227	44.03	9.0	even	ok
	222	C61H145N23NaO19P2	529.6815	-1.3	25.8	228	42.60	3.0	even	ok
	223	C53H20N32NaO23P2S	529.6819	-0.5	25.8	229	52.09	62.0	even	ok
	224	C55H156N17NaO26PS2	529.6823	0.2	26.0	230	66.01	-12.0	even	ok
	225	C52H140N35O15P2S	529.6816	-1.0	26.0	231	45.33	3.0	even	ok
	226	C59H165N5NaO33P2S	529.6821	-0.0	26.1	232	69.15	-18.0	even	ok
	227	C54H21N28O29S2	529.6818	-0.6	26.2	233	60.06	60.0	even	ok
	228	C60H168NO37P2S	529.6825	0.7	26.3	234	59.18	-20.0	even	ok
	229	C46H136N45O9P2S2	529.6823	0.2	26.3	235	65.32	4.0	even	ok
	230	C58H3N46NaO10PS	529.6827	1.0	26.3	236	45.41	82.0	even	ok
	231	C62H39N4O43P2	529.6816	-1.1	26.4	237	53.23	48.0	even	ok
	232	C65H147N17NaO23S	529.6819	-0.4	26.5	238	62.81	2.0	even	ok
	233	C56H11N42O12P2S2	529.6814	-1.4	26.6	239	48.65	75.0	even	ok
	234	C57H138N33O15S2	529.6825	0.7	26.6	240	58.35	7.0	even	ok
	235	C58H23N28O22P2S2	529.6814	-1.4	26.6	241	48.73	64.0	even	ok
	236	C49H127N47NaO10S	529.6825	0.8	26.6	242	47.97	11.0	even	ok
	237	C70H26N10NaO35	529.6814	-1.3	26.6	243	49.86	64.0	even	ok
	238	C58H154N15O29S2	529.6821	-0.1	26.7	245	66.22	-9.0	even	ok
	239	C63H164NO37P2	529.6814	-1.4	26.8	246	39.73	-15.0	even	ok
	240	C63H11N32NaO20P	529.6815	-1.1	26.8	247	43.45	76.0	even	ok
	241	C52H142N29O24S	529.6829	1.4	26.9	248	47.86	-2.0	even	ok
	242	C47H115N61NaS	529.6825	0.7	27.1	249	47.54	22.0	even	ok
	243	C57H22N26NaO27S2	529.6823	0.4	27.1	250	61.89	61.0	even	ok
	244	C57H160N11O31P2S	529.6820	-0.2	27.2	251	64.93	-14.0	even	ok

Compound Spectrum SmartFormula Report

Meas. m/z	#	Ion Formula	m/z	err [ppm]	mSigma	# mSigma	Score	rdb	e⁻ Conf	N-Rule
	245	C62H12N34NaO17P2	529.6825	0.8	27.2	253	47.18	76.0	even	ok
	246	C67H37N2O43S	529.6825	0.6	27.3	254	58.15	52.0	even	ok
	247	C60H151N13NaO32	529.6823	0.3	27.3	255	52.06	-7.0	even	ok
	248	C61H153N19NaO18P2S2	529.6813	-1.6	27.4	256	45.34	-3.0	even	ok
	249	C61H6N38O18P	529.6814	-1.3	27.4	257	40.88	80.0	even	ok
	250	C58H139N27NaO22	529.6823	0.3	27.4	258	52.01	4.0	even	ok
	251	C54H123N51NaOS2	529.6821	0.0	27.4	259	66.76	20.0	even	ok
	252	C62H141N27NaO15P2	529.6819	-0.4	27.5	260	50.52	8.0	even	ok
	253	C48H15N40NaO20PS2	529.6816	-1.1	27.5	261	51.58	63.0	even	ok
	254	C71H29N6O39	529.6818	-0.6	27.6	263	57.85	62.0	even	ok
	255	C51H15N38O21P2S	529.6818	-0.7	27.7	264	47.77	66.0	even	ok
	256	C59H150N19O25S2	529.6825	0.7	27.7	265	56.75	-4.0	even	ok
	257	C56H13N36O21S2	529.6827	1.1	27.8	266	51.71	70.0	even	ok
	258	C65H25N16O33S	529.6825	0.6	27.8	267	57.49	63.0	even	ok
	259	C56H26N22NaO31S2	529.6819	-0.4	27.8	268	60.23	56.0	even	ok
	260	C56H157N15NaO27P2S	529.6817	-0.8	27.9	269	54.57	-12.0	even	ok
	261	C46H10N46O18PS2	529.6815	-1.3	27.9	270	48.75	67.0	even	ok
	262	C57H130N37O16	529.6827	1.0	28.0	271	43.68	13.0	even	ok
	263	C58H8N40NaO15P2	529.6816	-0.9	28.0	272	44.42	77.0	even	ok
	264	C59H26N16NaO38	529.6825	0.7	28.1	274	46.96	56.0	even	ok
	265	C55H9N36O26	529.6824	0.5	28.2	275	48.93	71.0	even	ok
	266	C59H140N29O17P2	529.6814	-1.5	28.2	276	38.31	7.0	even	ok
	267	C59H39N6NaO42PS	529.6813	-1.5	28.3	277	45.15	45.0	even	ok
	268	C60H7N40O15P2	529.6824	0.6	28.4	278	47.93	80.0	even	ok
	269	C56H159N13O30PS2	529.6826	0.9	28.4	279	53.17	-14.0	even	ok
	270	C66H150N13O27S	529.6823	0.3	28.6	280	61.47	0.0	even	ok
	271	C60H5N40O17S	529.6820	-0.2	28.6	281	62.23	80.0	even	ok
	272	C49H18N36O24PS2	529.6819	-0.4	28.7	283	59.18	61.0	even	ok
	273	C60H159N9NaO31S2	529.6822	0.0	28.8	284	64.42	-13.0	even	ok
	274	C51H23N30NaO26PS2	529.6820	-0.3	28.9	285	61.40	57.0	even	ok
	275	C58H16N36NaO14P2S2	529.6815	-1.2	29.0	286	48.20	71.0	even	ok
	276	C59H143N27NaO17S2	529.6826	0.9	29.1	287	52.68	3.0	even	ok
	277	C60H28N22NaO24P2S2	529.6815	-1.2	29.1	288	48.21	60.0	even	ok
	278	C47H122N53O8S	529.6824	0.6	29.1	289	47.15	15.0	even	ok
	279	C45H133N49NaO5P2S2	529.6819	-0.4	29.3	290	48.60	6.0	even	ok
	280	C63H165N5NaO28P2S2	529.6813	-1.6	29.3	291	43.35	-14.0	even	ok
	281	C60H136N33O13P2	529.6818	-0.6	29.4	292	46.40	12.0	even	ok
	282	C59H141N33NaO8P2S2	529.6813	-1.6	29.4	293	43.17	8.0	even	ok
	283	C54H152N21O25P2S	529.6816	-1.0	29.4	294	50.33	-8.0	even	ok
	284	C73H34NaO41	529.6819	-0.5	29.4	295	57.83	58.0	even	ok
	285	C58H164N7NaO32PS2	529.6827	1.1	29.4	296	49.57	-18.0	even	ok
	286	C60H42N2O46PS	529.6817	-0.8	29.5	297	52.56	43.0	even	ok
	287	C47H16N42NaO17P2S2	529.6825	0.8	29.5	298	53.35	63.0	even	ok
	288	C55H126N47O5S2	529.6825	0.7	29.5	299	54.54	18.0	even	ok
	289	C68H147N15O24P	529.6822	0.0	29.6	300	52.73	5.0	even	ok
	290	C50H12N42NaO17P2S	529.6814	-1.3	29.7	301	38.29	68.0	even	ok

Compound Spectrum SmartFormula Report

Meas. m/z	#	Ion Formula	m/z	err [ppm]	mSigma	# mSigma	Score	rdb	e⁻ Conf	N-Rule
	291	C57H158N11O33S2	529.6816	-1.0	29.7	302	50.52	-14.0	even	ok
	292	C50H24N32NaO23P2S2	529.6830	1.6	29.8	303	42.17	57.0	even	ok
	293	C58H146N19O30	529.6822	0.2	29.8	304	50.99	-3.0	even	ok
	294	C71H155N5O30P	529.6826	0.9	29.9	305	42.92	-1.0	even	ok
	295	C66H145N21NaO17P2	529.6828	1.2	30.0	306	38.94	7.0	even	ok
	296	C56H134N33O20	529.6822	0.2	30.1	307	50.77	8.0	even	ok
	297	C48H19N38O21P2S2	529.6829	1.5	30.1	308	44.03	61.0	even	ok
	298	C70H152N9NaO26P	529.6822	0.2	30.1	309	50.05	1.0	even	ok
	299	C61H155N13NaO27S2	529.6826	0.9	30.1	310	51.24	-8.0	even	ok
	300	C56H3N46O13P2	529.6815	-1.1	30.2	311	40.23	81.0	even	ok
	301	C67H30N10NaO35S	529.6826	0.8	30.2	312	51.97	59.0	even	ok
	302	C45H11N48O15P2S2	529.6825	0.6	30.3	313	54.66	67.0	even	ok
	303	C58H18N30NaO23S2	529.6828	1.2	30.3	314	46.45	66.0	even	ok
	304	C57H21N22O36	529.6824	0.5	30.3	315	46.38	60.0	even	ok
	305	C58H40N8NaO39P2S	529.6823	0.4	30.3	316	57.72	45.0	even	ok
	306	C62H10N34NaO19S	529.6821	-0.0	30.4	317	61.98	76.0	even	ok
	307	C44H8N52NaO11P2S2	529.6821	-0.1	30.4	318	61.60	69.0	even	ok
	308	C60H35N14O32P2S2	529.6814	-1.4	30.5	319	44.48	53.0	even	ok
	309	C57H123N49O2PS	529.6824	0.5	30.6	320	46.84	23.0	even	ok
	310	C64H140N27O15P2	529.6827	1.1	30.6	321	40.22	11.0	even	ok
	311	C52H26N26O30PS2	529.6824	0.4	30.7	322	56.41	55.0	even	ok
	312	C67H144N19NaO20P	529.6818	-0.6	30.8	323	44.68	7.0	even	ok
	313	C62H156N15O22P2S2	529.6817	-0.9	30.8	324	50.10	-5.0	even	ok
	314	C50H14N36NaO26S	529.6827	1.1	30.8	325	47.36	63.0	even	ok
	315	C68H155N7NaO29S	529.6824	0.5	30.8	326	55.97	-4.0	even	ok
	316	C58H25N22O31S2	529.6827	1.1	30.8	327	48.02	59.0	even	ok
	317	C65H139N25O18P	529.6817	-0.8	30.8	328	42.72	11.0	even	ok
	318	C51H139N33NaO20S	529.6825	0.8	30.8	329	43.28	0.0	even	ok
	319	C72H154N3O33	529.6816	-1.0	30.9	330	48.86	-1.0	even	ok
	320	C59H43N4O43P2S	529.6827	1.0	30.9	331	48.32	43.0	even	ok
	321	C43H128N55O3P2S2	529.6818	-0.6	31.0	332	44.74	10.0	even	ok
	322	C56H6N44NaO13S2	529.6828	1.2	31.0	333	45.73	77.0	even	ok
	323	C60H131N39O8PS	529.6828	1.3	31.0	334	37.41	17.0	even	ok
	324	C59H163N5NaO35S2	529.6817	-0.8	31.1	335	51.04	-18.0	even	ok
	325	C56H35N14O37P2S	529.6822	0.2	31.1	336	58.88	49.0	even	ok
	326	C53H25N24O33S2	529.6814	-1.5	31.2	337	42.82	55.0	even	ok
	327	C59H128N43NaO4PS	529.6825	0.6	31.2	338	44.25	19.0	even	ok
	328	C57H29N18O35S2	529.6823	0.2	31.2	339	58.20	54.0	even	ok
	329	C55H32N18NaO33P2S	529.6819	-0.5	31.3	340	54.88	51.0	even	ok
	330	C54H31N20NaO32PS2	529.6825	0.6	31.4	341	53.34	51.0	even	ok
	331	C42H3N58O9P2S2	529.6820	-0.2	31.4	342	57.84	73.0	even	ok
	332	C59H4N44NaO11P2	529.6821	-0.1	31.6	343	49.60	82.0	even	ok
	333	C61H38N2NaO48	529.6825	0.7	31.7	344	51.52	45.0	even	ok
	334	C57H131N41NaO7S2	529.6826	0.9	31.7	345	49.47	14.0	even	ok
	335	C54H6N40NaO22	529.6821	-0.2	31.8	346	48.69	73.0	even	ok
	336	C61H130N37O11S	529.6818	-0.6	31.9	347	52.87	17.0	even	ok

Compound Spectrum SmartFormula Report

Meas. m/z	#	Ion Formula	m/z	err [ppm]	mSigma	# mSigma	Score	rdb	e⁻ Conf	N-Rule
	337	C48H7N48O15P2S	529.6813	-1.5	31.9	348	34.52	72.0	even	ok
	338	C60H144N29O12P2S2	529.6816	-0.9	32.0	349	48.51	6.0	even	ok
	339	C56H127N41NaO12	529.6823	0.3	32.1	350	46.56	15.0	even	ok
	340	C63H13N30O23S	529.6825	0.6	32.1	351	51.92	74.0	even	ok
	341	C59H19N32O18P2S2	529.6818	-0.6	32.1	352	52.94	69.0	even	ok
	342	C55H30N18NaO35S2	529.6815	-1.3	32.2	353	43.87	51.0	even	ok
	343	C62H136N33NaO10PS	529.6829	1.5	32.3	354	34.57	13.0	even	ok
	344	C60H146N23O21S2	529.6830	1.5	32.4	355	40.57	1.0	even	ok
	345	C46H119N57NaO4S	529.6821	-0.1	32.4	356	48.57	17.0	even	ok
	346	C61H162N5O35S2	529.6825	0.7	32.4	357	50.54	-15.0	even	ok
	347	C53H27N24O31P2S	529.6818	-0.7	32.5	358	51.07	55.0	even	ok
	348	C51H152N23NaO24PS2	529.6814	-1.5	32.5	359	41.46	-11.0	even	ok
	349	C56H4N50NaO4P2S2	529.6815	-1.2	32.6	360	44.06	82.0	even	ok
	350	C61H31N18O28P2S2	529.6818	-0.5	32.7	361	52.24	58.0	even	ok
	351	C62H40N8NaO34P2S2	529.6815	-1.2	32.7	362	44.15	49.0	even	ok
	352	C64H136N29NaO14P	529.6813	-1.5	32.9	363	33.96	13.0	even	ok
	353	C49H134N39O18S	529.6824	0.6	32.9	364	42.89	4.0	even	ok
	354	C56H18N26NaO32	529.6821	-0.1	33.0	365	47.30	62.0	even	ok
	355	C57H143N23NaO26	529.6819	-0.5	33.0	366	43.47	-1.0	even	ok
	356	C64H168NO32P2S2	529.6817	-0.9	33.1	367	47.41	-16.0	even	ok
	357	C59H34N12NaO37S2	529.6823	0.4	33.1	368	53.38	50.0	even	ok
	358	C58H122N47O5S	529.6814	-1.4	33.1	369	34.29	23.0	even	ok
	359	C60H30N16NaO33S2	529.6828	1.2	33.1	370	43.24	55.0	even	ok
	360	C59H133N37NaO9P2	529.6814	-1.3	33.2	371	35.56	14.0	even	ok
	361	C59H33N8O46	529.6824	0.5	33.3	372	51.66	49.0	even	ok
	362	C68H33N6O39S	529.6829	1.5	33.3	373	40.32	57.0	even	ok
	363	C63H137N31NaO11P2	529.6823	0.4	33.3	374	44.35	13.0	even	ok
	364	C63H135N31NaO13S	529.6819	-0.4	33.3	375	53.12	13.0	even	ok
	365	C52H24N28NaO27P2S	529.6814	-1.3	33.3	376	42.07	57.0	even	ok
	366	C64H161N9NaO24P2S2	529.6817	-0.7	33.3	377	49.13	-9.0	even	ok
	367	C69H158N3O33S	529.6827	1.1	33.4	378	44.43	-6.0	even	ok
	368	C56H159N9NaO36S	529.6830	1.6	33.4	379	38.78	-17.0	even	ok
	369	C52H155N19O28PS2	529.6817	-0.8	33.7	380	48.08	-13.0	even	ok
	370	C54H160N13NaO30PS2	529.6818	-0.6	33.7	381	50.10	-17.0	even	ok
	371	C55H34N16O36PS2	529.6828	1.3	33.8	382	42.29	49.0	even	ok
	372	C60H158N5O40	529.6822	0.2	33.8	383	46.13	-14.0	even	ok
	373	C62H158N9O31S2	529.6830	1.6	33.8	384	39.01	-10.0	even	ok
	374	C57H123N45NaO8	529.6827	1.2	33.9	385	36.11	20.0	even	ok
	375	C55H131N37NaO16	529.6819	-0.5	34.0	386	42.39	10.0	even	ok
	376	C68H31N12O30P2S	529.6816	-1.0	34.0	387	45.47	62.0	even	ok
	377	C65H18N24NaO25S	529.6826	0.8	34.2	388	47.21	70.0	even	ok
	378	C60H127N41NaO7S	529.6815	-1.3	34.2	389	35.03	19.0	even	ok
	379	C58H134N37O11S2	529.6829	1.5	34.2	390	38.75	12.0	even	ok
	380	C60H166NO39S2	529.6821	-0.1	34.3	391	55.05	-20.0	even	ok
	381	C61H132N37O9P2	529.6823	0.2	34.4	392	44.88	17.0	even	ok
	382	C62H149N23NaO14P2S2	529.6817	-0.7	34.4	394	47.69	2.0	even	ok

Compound Spectrum SmartFormula Report

Meas. m/z	#	Ion Formula	m/z	err [ppm]	mSigma	# mSigma	Score	rdb	e¯ Conf	N-Rule
	383	C54H154N15O34S	529.6829	1.4	34.5	395	39.66	-13.0	even	ok
	384	C54H122N47O10	529.6822	0.1	34.5	396	45.54	19.0	even	ok
	385	C64H138N27O17S	529.6823	0.3	34.5	397	53.17	11.0	even	ok
	386	C61H24N26NaO20P2S2	529.6819	-0.4	34.6	398	51.73	65.0	even	ok
	387	C57H39N10NaO38PS2	529.6829	1.4	34.8	399	39.28	45.0	even	ok
	388	C57H7N46O8P2S2	529.6818	-0.6	34.9	400	49.26	80.0	even	ok
	389	C44H114N63O2S	529.6820	-0.3	35.0	401	43.74	21.0	even	ok
	390	C56H33N14O39S2	529.6818	-0.6	35.0	402	48.48	49.0	even	ok
	391	C53H151N19NaO30S	529.6825	0.8	35.1	403	46.61	-11.0	even	ok
	392	C48H131N43NaO14S	529.6821	-0.1	35.1	404	45.33	6.0	even	ok
	393	C65H10N32O20P	529.6823	0.4	35.2	405	42.30	79.0	even	ok
	394	C63H36N12NaO30P2S2	529.6819	-0.4	35.2	406	51.04	54.0	even	ok
	395	C57H128N43O7P2	529.6814	-1.5	35.2	407	32.13	18.0	even	ok
	396	C48H9N42O24S	529.6826	0.9	35.3	408	36.91	67.0	even	ok
	397	C68H18N22O26P	529.6828	1.2	35.4	410	34.05	73.0	even	ok
	398	C54H13N32O30	529.6820	-0.3	35.4	411	42.74	66.0	even	ok
	399	C63H8N38NaO13P2	529.6830	1.6	35.5	412	30.75	81.0	even	ok
	400	C69H156N9O24P2S	529.6814	-1.3	35.5	413	39.70	-1.0	even	ok
	401	C55H163N9O34PS2	529.6822	0.1	35.5	414	54.18	-19.0	even	ok
	402	C55H138N29O24	529.6818	-0.7	35.6	415	39.01	3.0	even	ok
	403	C69H17N20O29	529.6818	-0.6	35.6	416	47.36	73.0	even	ok
	404	C67H15N26NaO22P	529.6824	0.6	35.8	417	40.01	75.0	even	ok
	405	C55H118N51O6	529.6827	1.0	35.9	418	35.99	24.0	even	ok
	406	C61H3N44O11P2	529.6829	1.4	35.9	419	31.94	85.0	even	ok
	407	C58H169NNaO37P2S	529.6817	-0.8	36.0	420	44.69	-23.0	even	ok
	408	C57H168N3NaO36PS2	529.6823	0.2	36.0	421	51.50	-23.0	even	ok
	409	C59H155N9NaO36	529.6819	-0.5	36.0	422	40.35	-12.0	even	ok
	410	C70H36N6NaO32P2S	529.6817	-0.8	36.3	423	44.78	58.0	even	ok
	411	C58H38N8NaO41S2	529.6819	-0.4	36.3	424	48.84	45.0	even	ok
	412	C66H143N21NaO19S	529.6824	0.4	36.3	425	48.74	7.0	even	ok
	413	C64H7N36NaO16P	529.6820	-0.3	36.4	427	42.11	81.0	even	ok
	414	C66H21N20O29S	529.6829	1.5	36.4	428	37.28	68.0	even	ok
	415	C58H132N43O2P2S2	529.6816	-0.9	36.5	429	43.24	17.0	even	ok
	416	C63H43N4O38P2S2	529.6818	-0.5	36.5	430	47.51	47.0	even	ok
	417	C51H146N25O28S	529.6824	0.6	36.5	431	46.87	-7.0	even	ok
	418	C70H23N16NaO28P	529.6829	1.4	36.5	432	31.53	69.0	even	ok
	419	C53H126N43O14	529.6818	-0.7	36.6	433	37.94	14.0	even	ok
	420	C60H37N8O41S2	529.6827	1.1	36.7	434	41.28	48.0	even	ok
	421	C65H164N5O28P2S2	529.6821	-0.1	36.9	435	52.33	-11.0	even	ok
	422	C47H6N46NaO20S	529.6823	0.3	36.9	436	41.53	69.0	even	ok
	423	C71H22N14NaO31	529.6819	-0.5	37.0	437	47.69	69.0	even	ok
	424	C62H47O42P2S2	529.6814	-1.4	37.1	438	37.70	42.0	even	ok
	425	C56H164N7O35P2S	529.6816	-1.0	37.1	439	41.52	-19.0	even	ok
	426	C59H12N40NaO10P2S2	529.6819	-0.4	37.2	440	48.33	76.0	even	ok
	427	C63H152N19O18P2S2	529.6821	-0.1	37.4	441	51.51	0.0	even	ok
	428	C46H126N49O12S	529.6820	-0.3	37.4	442	41.08	10.0	even	ok

Compound Spectrum SmartFormula Report

Meas. m/z	#	Ion Formula	m/z	err [ppm]	mSigma	# mSigma	Score	rdb	e⁻ Conf	N-Rule
	429	C50H27N26NaO30PS2	529.6816	-1.1	37.6	444	40.20	52.0	even	ok
	430	C71H161N3NaO26P2S	529.6815	-1.2	37.6	445	39.39	-5.0	even	ok
	431	C60H129N41NaO5P2	529.6819	-0.5	37.6	446	39.18	19.0	even	ok
	432	C66H9N30O23	529.6813	-1.5	37.6	447	30.03	79.0	even	ok
	433	C48H22N32O28PS2	529.6815	-1.3	37.8	448	38.14	56.0	even	ok
	434	C58H30N12NaO42	529.6821	-0.1	37.8	449	41.76	51.0	even	ok
	435	C72H25N10O35	529.6822	0.2	37.9	450	49.31	67.0	even	ok
	436	C62H27N22O24P2S2	529.6823	0.3	37.9	451	48.27	63.0	even	ok
	437	C50H19N34O25P2S	529.6813	-1.5	38.0	452	29.54	61.0	even	ok
	438	C67H146N17O23S	529.6827	1.1	38.1	453	39.40	5.0	even	ok
	439	C57H150N15O34	529.6818	-0.7	38.3	454	36.43	-8.0	even	ok
	440	C56H144N25NaO23P	529.6829	1.4	38.4	455	30.41	-1.0	even	ok
	441	C53H10N36NaO26	529.6816	-1.0	38.4	457	33.57	68.0	even	ok
	442	C60H137N37NaO4P2S2	529.6817	-0.7	38.6	458	42.73	13.0	even	ok
	443	C68H14N24NaO25	529.6814	-1.3	38.6	459	30.74	75.0	even	ok
	444	C53H119N51NaO6	529.6819	-0.5	38.6	460	37.51	21.0	even	ok
	445	C52H26N22NaO36S	529.6827	1.1	38.6	461	38.67	52.0	even	ok
	446	C62H42N2NaO43S2	529.6828	1.3	38.7	462	37.33	44.0	even	ok
	447	C64H39N8O34P2S2	529.6823	0.3	38.9	464	46.96	52.0	even	ok
	448	C51H30N22O34PS2	529.6819	-0.4	38.9	465	45.74	50.0	even	ok
	449	C71H39N2O36P2S	529.6821	-0.1	39.0	467	48.71	56.0	even	ok
	450	C56H122N51OS2	529.6829	1.5	39.1	468	34.22	23.0	even	ok
	451	C54H135N33NaO20	529.6814	-1.4	39.1	469	29.93	5.0	even	ok
	452	C58H124N47O3P2	529.6818	-0.6	39.1	470	36.14	23.0	even	ok
	453	C53H35N16NaO36PS2	529.6820	-0.2	39.1	471	47.39	46.0	even	ok
	454	C54H132N39NaO13P	529.6829	1.4	39.1	472	29.89	10.0	even	ok
	455	C49H28N28NaO27P2S2	529.6826	0.8	39.1	473	41.62	52.0	even	ok
	456	C46H20N38NaO21P2S2	529.6821	-0.1	39.3	474	49.10	58.0	even	ok
	457	C66H19N26O20P2S	529.6816	-1.0	39.4	475	39.38	73.0	even	ok
	458	C59H41N4O45S2	529.6823	0.2	39.4	476	47.01	43.0	even	ok
	459	C74H30N4NaO37	529.6823	0.4	39.6	477	45.29	63.0	even	ok
	460	C47H23N34O25P2S2	529.6825	0.6	39.6	478	42.85	56.0	even	ok
	461	C54H115N55NaO2	529.6823	0.3	39.7	480	38.17	26.0	even	ok
	462	C50H21N28O34S	529.6826	1.0	39.8	481	39.20	56.0	even	ok
	463	C60H15N36O14P2S2	529.6823	0.3	39.9	482	45.90	74.0	even	ok
	464	C56H25N18O40	529.6820	-0.3	39.9	483	38.01	55.0	even	ok
	465	C65H157N13NaO20P2S2	529.6822	0.1	39.9	484	47.71	-4.0	even	ok
	466	C50H31N24O31P2S2	529.6829	1.5	40.1	485	33.97	50.0	even	ok
	467	C45H123N53NaO8S	529.6816	-0.9	40.1	486	32.57	12.0	even	ok
	468	C44H15N44O19P2S2	529.6820	-0.2	40.1	487	46.16	62.0	even	ok
	469	C57H44N4NaO43P2S	529.6819	-0.5	40.1	488	43.66	40.0	even	ok
	470	C69H151N11NaO25S	529.6828	1.3	40.2	489	35.52	1.0	even	ok
	471	C43H12N48NaO15P2S2	529.6817	-0.9	40.3	490	39.16	64.0	even	ok
	472	C69H143N19O20P	529.6826	0.9	40.5	491	32.73	10.0	even	ok
	473	C58H47O47P2S	529.6822	0.2	40.5	492	46.07	38.0	even	ok
	474	C49H18N32NaO30S	529.6823	0.3	40.5	493	45.17	58.0	even	ok

Compound Spectrum SmartFormula Report

Meas. m/z	#	Ion Formula	m/z	err [ppm]	mSigma	# mSigma	Score	rdb	e¯ Conf	N-Rule
	475	C64H32N16NaO26P2S2	529.6824	0.5	40.5	494	43.24	59.0	even	ok
	476	C52H123N47NaO10	529.6814	-1.4	40.6	495	28.66	16.0	even	ok
	477	C63H6N38NaO15S	529.6826	0.8	40.6	496	39.87	81.0	even	ok
	478	C53H14N34O27P	529.6830	1.6	40.7	497	27.09	66.0	even	ok
	479	C61H140N33O8P2S2	529.6821	-0.1	40.8	498	46.91	11.0	even	ok
	480	C66H135N29O14P	529.6821	0.0	40.8	499	39.45	16.0	even	ok
	481	C56H147N19NaO30	529.6814	-1.4	40.9	500	28.51	-6.0	even	ok
	482	C54H38N12O40PS2	529.6824	0.4	40.9	501	43.18	44.0	even	ok
	483	C51H5N42O24	529.6815	-1.2	40.9	502	29.92	72.0	even	ok
	484	C54H139N31O21P	529.6828	1.2	41.0	503	29.72	3.0	even	ok
	485	C55H39N10O41P2S	529.6818	-0.7	41.0	504	40.88	44.0	even	ok
	486	C51H114N57O4	529.6818	-0.7	41.1	505	33.62	25.0	even	ok
	487	C58H156N11NaO33P	529.6829	1.4	41.1	506	28.21	-12.0	even	ok
	488	C75H33O41	529.6827	1.1	41.1	507	36.88	61.0	even	ok
	489	C50H143N29NaO24S	529.6821	-0.1	41.1	508	38.74	-5.0	even	ok
	490	C55H37N10O43S2	529.6814	-1.5	41.1	509	33.08	44.0	even	ok
	491	C68H140N23NaO16P	529.6822	0.2	41.2	510	37.61	12.0	even	ok
	492	C54H36N14NaO37P2S	529.6814	-1.3	41.2	511	34.21	46.0	even	ok
	493	C71H148N13NaO22P	529.6827	1.1	41.3	512	30.57	6.0	even	ok
	494	C41H7N54O13P2S2	529.6816	-1.1	41.3	513	36.39	68.0	even	ok
	495	C73H150N7O29	529.6821	-0.2	41.3	514	45.40	4.0	even	ok
	496	C68H24N20NaO22P2S	529.6817	-0.8	41.4	515	38.99	69.0	even	ok
	497	C66H44N2NaO36P2S2	529.6824	0.5	41.5	516	42.07	48.0	even	ok
	498	C56H43N6NaO42PS2	529.6825	0.6	41.5	517	40.85	40.0	even	ok
	499	C64H133N35NaO7P2	529.6828	1.2	41.6	518	28.90	18.0	even	ok
	500	C52H130N39O18	529.6813	-1.5	41.7	519	26.50	9.0	even	ok
544.3575	1	C61H147N27NaO18S2	544.3580	0.9	14.8	1	83.95	3.0	even	ok
	2	C61H23N32O19P2S2	544.3572	-0.5	14.9	2	94.33	69.0	even	ok
	3	C64H160N15O23P2S2	544.3571	-0.8	15.1	3	86.24	-5.0	even	ok
	4	C63H157N19NaO19P2S2	544.3567	-1.5	15.1	4	72.06	-3.0	even	ok
	5	C60H20N36NaO15P2S2	544.3569	-1.1	15.2	5	79.38	71.0	even	ok
	6	C59H142N33O16S2	544.3579	0.8	15.3	6	86.89	7.0	even	ok
	7	C66H165N9NaO25P2S2	544.3572	-0.6	15.5	7	89.40	-9.0	even	ok
	8	C63H28N26NaO21P2S2	544.3573	-0.3	15.5	8	97.16	65.0	even	ok
	9	C60H22N30NaO24S2	544.3582	1.3	15.5	9	75.19	66.0	even	ok
	10	C58H17N36O22S2	544.3581	1.1	15.7	10	78.53	70.0	even	ok
	11	C58H10N44NaO14S2	544.3582	1.3	15.7	11	74.97	77.0	even	ok
	12	C69H34N10NaO36S	544.3580	0.9	15.8	12	83.55	59.0	even	ok
	13	C58H15N42O13P2S2	544.3568	-1.3	15.8	13	74.82	75.0	even	ok
	14	C56H5N50O12S2	544.3581	1.1	16.0	14	78.12	81.0	even	ok
	15	C67H29N16O34S	544.3579	0.7	16.2	15	86.58	63.0	even	ok
	16	C62H148N29O13P2S2	544.3571	-0.8	16.3	16	84.01	6.0	even	ok
	17	C57H14N40NaO18S2	544.3577	0.5	16.3	17	91.63	72.0	even	ok
	18	C69H41N2O44S	544.3579	0.7	16.4	18	86.18	52.0	even	ok
	19	C62H32N22NaO25P2S2	544.3569	-1.1	16.4	19	77.68	60.0	even	ok
	20	C64H153N23NaO15P2S2	544.3571	-0.6	16.6	20	87.30	2.0	even	ok

Compound Spectrum SmartFormula Report

Meas. m/z	#	Ion Formula	m/z	err [ppm]	mSigma	# mSigma	Score	rdb	e¯ Conf	N-Rule
	21	C58H139N37NaO12S2	544.3576	0.1	16.8	21	98.58	9.0	even	ok
	22	C64H31N22O25P2S2	544.3577	0.4	16.8	22	92.74	63.0	even	ok
	23	C60H27N28O23P2S2	544.3568	-1.3	16.8	23	73.44	64.0	even	ok
	24	C67H168N5O29P2S2	544.3575	0.0	17.0	24	100.00	-11.0	even	ok
	25	C68H38N6NaO40S	544.3575	0.1	17.0	25	99.17	54.0	even	ok
	26	C65H156N19O19P2S2	544.3575	0.0	17.1	26	99.99	0.0	even	ok
	27	C63H35N18O29P2S2	544.3573	-0.4	17.1	27	90.42	58.0	even	ok
	28	C69H163N3NaO34S	544.3573	-0.3	17.3	28	93.35	-9.0	even	ok
	29	C71H162N3O34S	544.3581	1.2	17.3	29	74.80	-6.0	even	ok
	30	C55H9N46O16S2	544.3577	0.3	17.5	30	93.12	76.0	even	ok
	31	C63H159N13NaO28S2	544.3580	0.9	17.7	32	79.10	-8.0	even	ok
	32	C65H40N12NaO31P2S2	544.3573	-0.3	17.7	33	93.05	54.0	even	ok
	33	C66H26N20NaO30S	544.3575	0.0	17.9	34	97.63	65.0	even	ok
	34	C61H154N19O26S2	544.3579	0.8	18.0	35	82.15	-4.0	even	ok
	35	C56H134N43O10S2	544.3575	-0.1	18.2	36	96.63	13.0	even	ok
	36	C70H159N7NaO30S	544.3578	0.5	18.2	37	86.97	-4.0	even	ok
	37	C66H33N12O38S	544.3574	-0.1	18.2	38	95.37	58.0	even	ok
	38	C66H36N16NaO27P2S2	544.3578	0.5	18.3	39	86.37	59.0	even	ok
	39	C67H161N13NaO21P2S2	544.3576	0.2	18.3	40	93.88	-4.0	even	ok
	40	C59H135N41NaO8S2	544.3580	0.9	18.3	41	78.30	14.0	even	ok
	41	C60H151N23NaO22S2	544.3576	0.1	18.3	42	95.29	-2.0	even	ok
	42	C67H158N9O32S	544.3572	-0.5	18.7	43	87.01	-5.0	even	ok
	43	C70H35N12O31P2S	544.3570	-0.9	18.8	44	78.67	62.0	even	ok
	44	C68H154N13O28S	544.3577	0.3	18.8	45	89.49	0.0	even	ok
	45	C57H130N47O6S2	544.3579	0.8	18.8	46	80.99	18.0	even	ok
	46	C72H40N6NaO33P2S	544.3571	-0.7	19.0	47	81.74	58.0	even	ok
	47	C59H11N46O9P2S2	544.3572	-0.5	19.0	48	86.60	80.0	even	ok
	48	C65H169N5NaO29P2S2	544.3567	-1.5	19.1	49	66.54	-14.0	even	ok
	49	C64H21N26O28S	544.3574	-0.1	19.1	50	93.48	69.0	even	ok
	50	C73H43N2O37P2S	544.3575	-0.0	19.2	51	94.90	56.0	even	ok
	51	C61H16N40NaO11P2S2	544.3573	-0.3	19.4	52	89.52	76.0	even	ok
	52	C54H6N50NaO12S2	544.3573	-0.4	19.4	53	87.94	78.0	even	ok
	53	C58H146N29O20S2	544.3575	-0.1	19.5	54	94.06	2.0	even	ok
	54	C62H19N36O15P2S2	544.3577	0.4	19.6	55	87.55	74.0	even	ok
	55	C66H43N8O35P2S2	544.3577	0.4	19.7	56	87.21	52.0	even	ok
	56	C66H172NO33P2S2	544.3571	-0.8	19.9	57	78.19	-16.0	even	ok
	57	C65H30N20O28PS2	544.3567	-1.5	20.0	58	65.06	63.0	even	ok
	58	C68H164N9O25P2S2	544.3580	0.8	20.1	59	77.16	-6.0	even	ok
	59	C58H8N50NaO5P2S2	544.3569	-1.1	20.1	60	71.61	82.0	even	ok
	60	C65H30N16NaO34S	544.3571	-0.8	20.1	61	78.40	60.0	even	ok
	61	C56H3N56O3P2S2	544.3568	-1.3	20.6	62	67.51	86.0	even	ok
	62	C55H131N47NaO6S2	544.3571	-0.7	20.7	63	78.53	15.0	even	ok
	63	C67H39N12O31P2S2	544.3581	1.2	20.7	64	69.22	57.0	even	ok
	64	C59H26N26NaO28S2	544.3577	0.5	20.7	65	83.45	61.0	even	ok
	65	C64H24N30NaO17P2S2	544.3578	0.5	20.8	66	81.91	70.0	even	ok
	66	C56H127N51NaO2S2	544.3575	0.1	20.8	67	90.59	20.0	even	ok

Compound Spectrum SmartFormula Report

Meas. m/z	#	Ion Formula	m/z	err [ppm]	mSigma	# mSigma	Score	rdb	e¯ Conf	N-Rule
	67	C67H151N17NaO24S	544.3573	-0.3	20.9	68	86.36	2.0	even	ok
	68	C62H34N16NaO34S2	544.3582	1.3	21.0	69	66.95	55.0	even	ok
	69	C68H48N2NaO37P2S2	544.3578	0.5	21.0	70	81.36	48.0	even	ok
	70	C60H29N22O32S2	544.3581	1.1	21.0	71	70.11	59.0	even	ok
	71	C57H143N33NaO16S2	544.3571	-0.7	21.1	72	77.98	4.0	even	ok
	72	C66H155N13NaO28S	544.3569	-1.1	21.2	73	69.78	-3.0	even	ok
	73	C67H22N24NaO26S	544.3580	0.9	21.2	75	74.70	70.0	even	ok
	74	C57H21N32O26S2	544.3577	0.3	21.6	76	85.30	65.0	even	ok
	75	C65H17N30O24S	544.3579	0.7	21.6	77	77.38	74.0	even	ok
	76	C63H18N30NaO24S	544.3571	-0.8	21.7	78	75.58	71.0	even	ok
	77	C70H169N3NaO27P2S2	544.3580	1.0	21.9	79	70.84	-10.0	even	ok
	78	C63H25N22O32S	544.3570	-0.9	21.9	80	72.11	64.0	even	ok
	79	C67H35N14NaO30PS2	544.3568	-1.3	22.0	81	65.53	59.0	even	ok
	80	C54H122N57S2	544.3575	-0.1	22.1	82	88.72	24.0	even	ok
	81	C67H42N6O38PS2	544.3567	-1.5	22.2	83	62.26	52.0	even	ok
	82	C65H146N23O22S	544.3572	-0.5	22.2	84	80.62	6.0	even	ok
	83	C66H159N11O29PS	544.3582	1.4	22.2	85	63.93	-5.0	even	ok
	84	C65H27N26O21P2S2	544.3581	1.2	22.3	86	66.97	68.0	even	ok
	85	C71H160N9O25P2S	544.3568	-1.2	22.3	87	66.25	-1.0	even	ok
	86	C73H165N3NaO27P2S	544.3569	-1.1	22.3	88	69.38	-5.0	even	ok
	87	C56H18N36NaO22S2	544.3573	-0.4	22.4	89	82.57	67.0	even	ok
	88	C63H144N33O9P2S2	544.3575	0.0	22.4	90	89.31	11.0	even	ok
	89	C53H126N53O4S2	544.3570	-0.9	22.6	91	72.09	19.0	even	ok
	90	C64H44N8NaO35P2S2	544.3569	-1.1	22.6	92	68.18	49.0	even	ok
	91	C69H44N6NaO33P2S2	544.3582	1.4	22.7	93	63.16	53.0	even	ok
	92	C60H136N43O3P2S2	544.3570	-0.8	22.7	94	73.26	17.0	even	ok
	93	C62H141N37NaO5P2S2	544.3571	-0.6	22.8	95	76.34	13.0	even	ok
	94	C55H138N39O14S2	544.3570	-0.9	22.8	96	71.80	8.0	even	ok
	95	C62H39N14O33P2S2	544.3568	-1.3	22.8	97	64.69	53.0	even	ok
	96	C64H150N19O26S	544.3568	-1.3	23.1	98	63.79	1.0	even	ok
	97	C65H149N27NaO11P2S2	544.3576	0.2	23.3	99	84.33	7.0	even	ok
	98	C67H42N2NaO44S	544.3571	-0.8	23.3	100	73.19	49.0	even	ok
	99	C75H38NaO42	544.3573	-0.4	23.5	101	80.43	58.0	even	ok
	100	C61H13N36O22S	544.3570	-0.9	23.5	102	69.49	75.0	even	ok
	101	C64H14N34NaO20S	544.3575	0.0	23.6	103	86.34	76.0	even	ok
	102	C66H162N5O36S	544.3568	-1.3	23.7	104	63.08	-10.0	even	ok
	103	C65H47N4O39P2S2	544.3573	-0.4	23.8	105	78.36	47.0	even	ok
	104	C54H13N42O20S2	544.3572	-0.5	23.9	106	76.69	71.0	even	ok
	105	C72H37N2O44	544.3568	-1.4	23.9	107	61.62	57.0	even	ok
	106	C69H47NaO40PS2	544.3568	-1.3	24.0	108	62.80	48.0	even	ok
	107	C69H150N17O24S	544.3581	1.2	24.0	109	64.85	5.0	even	ok
	108	C64H31N18NaO31PS	544.3581	1.1	24.0	110	66.70	60.0	even	ok
	109	C66H152N23O15P2S2	544.3579	0.8	24.1	111	70.77	5.0	even	ok
	110	C67H32N20NaO23P2S2	544.3582	1.4	24.1	112	61.30	64.0	even	ok
	111	C69H163N7O28PS2	544.3570	-1.0	24.2	113	67.71	-6.0	even	ok
	112	C71H155N11NaO26S	544.3582	1.3	24.2	114	61.53	1.0	even	ok

Compound Spectrum SmartFormula Report

Meas. m/z	#	Ion Formula	m/z	err [ppm]	mSigma	# mSigma	Score	rdb	e⁻ Conf	N-Rule
	113	C62H163N9NaO32S2	544.3576	0.1	24.4	115	83.31	-13.0	even	ok
	114	C54H132N49NaO3PS2	544.3581	1.1	24.5	116	65.12	15.0	even	ok
	115	C65H23N28NaO20PS2	544.3568	-1.3	24.5	117	61.85	70.0	even	ok
	116	C73H33N6O40	544.3572	-0.5	24.6	118	75.26	62.0	even	ok
	117	C62H9N40O18S	544.3574	-0.1	24.7	119	82.65	80.0	even	ok
	118	C68H38N10O34PS2	544.3571	-0.6	24.8	120	73.13	57.0	even	ok
	119	C65H37N8O42S	544.3570	-0.9	24.8	121	67.71	53.0	even	ok
	120	C63H166N5O36S2	544.3579	0.8	24.8	122	70.61	-15.0	even	ok
	121	C64H147N25O19PS	544.3582	1.4	25.0	123	60.20	6.0	even	ok
	122	C64H143N27NaO18S	544.3569	-1.1	25.0	124	63.96	8.0	even	ok
	123	C56H144N35NaO13PS2	544.3581	1.1	25.0	125	64.20	4.0	even	ok
	124	C65H156N15NaO25PS	544.3579	0.7	25.1	126	71.48	-3.0	even	ok
	125	C62H19N32NaO21PS	544.3581	1.1	25.2	127	65.10	71.0	even	ok
	126	C60H158N15O30S2	544.3575	-0.1	25.3	128	82.98	-9.0	even	ok
	127	C68H147N21NaO20S	544.3578	0.5	25.3	129	74.51	7.0	even	ok
	128	C68H157N17NaO17P2S2	544.3580	1.0	25.6	131	65.34	1.0	even	ok
	129	C53H10N46NaO16S2	544.3569	-1.2	25.6	132	62.24	73.0	even	ok
	130	C59H155N19NaO26S2	544.3571	-0.7	25.8	133	70.22	-7.0	even	ok
	131	C66H142N27O18S	544.3577	0.3	25.8	134	76.72	11.0	even	ok
	132	C71H31N16O27P2S	544.3575	-0.1	25.8	135	81.90	67.0	even	ok
	133	C67H168NNaO35PS	544.3579	0.7	25.9	136	70.01	-14.0	even	ok
	134	C62H26N24O29PS	544.3580	0.9	26.1	137	66.58	64.0	even	ok
	135	C68H23N26O21P2S	544.3570	-0.9	26.2	138	66.65	73.0	even	ok
	136	C70H28N20NaO23P2S	544.3571	-0.7	26.3	139	69.43	69.0	even	ok
	137	C71H168NNaO30PS2	544.3570	-0.8	26.3	140	67.40	-10.0	even	ok
	138	C60H7N50O5P2S2	544.3577	0.4	26.5	141	75.34	85.0	even	ok
	139	C66H26N24O24PS2	544.3571	-0.6	26.5	142	70.27	68.0	even	ok
	140	C52H127N55OPS2	544.3580	0.9	26.6	143	64.89	19.0	even	ok
	141	C73H36N10NaO29P2S	544.3576	0.1	26.7	144	79.15	63.0	even	ok
	142	C62H138N33O16S	544.3568	-1.3	26.8	145	58.59	12.0	even	ok
	143	C59H4N54NaOP2S2	544.3573	-0.3	26.9	146	75.61	87.0	even	ok
	144	C72H34N8O37P	544.3582	1.3	27.0	147	58.44	62.0	even	ok
	145	C66H43N4NaO41PS	544.3581	1.1	27.0	148	62.22	49.0	even	ok
	146	C54H139N41O11PS2	544.3580	0.9	27.0	149	64.16	8.0	even	ok
	147	C70H43N4NaO36PS2	544.3572	-0.5	27.1	150	72.37	53.0	even	ok
	148	C74H39N6O33P2S	544.3579	0.8	27.2	151	66.88	61.0	even	ok
	149	C57H150N25O24S2	544.3570	-0.9	27.2	152	65.09	-3.0	even	ok
	150	C63H151N21O23PS	544.3578	0.5	27.2	153	70.94	1.0	even	ok
	151	C60H14N38O19PS	544.3580	0.9	27.3	154	64.91	75.0	even	ok
	152	C62H12N44NaO7P2S2	544.3578	0.5	27.3	155	70.90	81.0	even	ok
	153	C72H30N10NaO36	544.3568	-1.2	27.4	156	59.58	64.0	even	ok
	154	C61H6N44NaO14S	544.3571	-0.8	27.4	157	66.36	82.0	even	ok
	155	C51H5N52O14S2	544.3568	-1.4	27.5	158	56.74	77.0	even	ok
	156	C77H37O42	544.3581	1.1	27.6	159	60.65	61.0	even	ok
	157	C71H38N4O41P	544.3578	0.5	27.7	160	71.09	57.0	even	ok
	158	C53H14N44O17PS2	544.3582	1.3	27.8	161	57.18	71.0	even	ok

Compound Spectrum SmartFormula Report

Meas. m/z	#	Ion Formula	m/z	err [ppm]	mSigma	# mSigma	Score	rdb	e⁻ Conf	N-Rule
	159	C63H15N40O11P2S2	544.3581	1.2	27.9	162	59.08	79.0	even	ok
	160	C67H151N21O18PS2	544.3570	-1.0	27.9	163	62.03	5.0	even	ok
	161	C65H163N7O33PS	544.3578	0.5	27.9	164	69.70	-10.0	even	ok
	162	C65H139N31NaO14S	544.3573	-0.3	28.0	165	73.34	13.0	even	ok
	163	C63H144N29NaO15PS	544.3579	0.7	28.2	166	66.58	8.0	even	ok
	164	C61H38N12NaO38S2	544.3578	0.5	28.2	167	70.27	50.0	even	ok
	165	C68H31N18NaO26PS2	544.3572	-0.5	28.6	168	69.74	64.0	even	ok
	166	C76H44NaO35P2S	544.3580	0.9	28.6	169	61.88	57.0	even	ok
	167	C64H38N10O39PS	544.3580	0.9	28.8	170	62.44	53.0	even	ok
	168	C59H33N18O36S2	544.3577	0.3	28.8	171	72.20	54.0	even	ok
	169	C58H30N22NaO32S2	544.3573	-0.4	28.8	172	71.31	56.0	even	ok
	170	C74H158N3O34	544.3570	-0.9	28.9	173	62.25	-1.0	even	ok
	171	C76H34N4NaO38	544.3577	0.5	28.9	174	69.46	63.0	even	ok
	172	C61H23N28NaO25PS	544.3576	0.2	29.0	176	73.05	66.0	even	ok
	173	C70H25N16O34	544.3568	-1.4	29.0	177	54.69	68.0	even	ok
	174	C62H41N8O42S2	544.3581	1.1	29.0	178	58.30	48.0	even	ok
	175	C64H46N2NaO44S2	544.3582	1.3	29.0	179	55.57	44.0	even	ok
	176	C63H134N37O12S	544.3572	-0.5	29.1	180	68.67	17.0	even	ok
	177	C65H20N34NaO13P2S2	544.3582	1.4	29.3	181	54.41	75.0	even	ok
	178	C74H29N10O36	544.3576	0.3	29.3	182	71.61	67.0	even	ok
	179	C58H156N21NaO23PS2	544.3581	1.1	29.3	183	57.93	-7.0	even	ok
	180	C65H10N38NaO16S	544.3580	0.9	29.4	184	62.06	81.0	even	ok
	181	C53H136N45NaO7PS2	544.3577	0.3	29.6	185	70.92	10.0	even	ok
	182	C63H5N44O14S	544.3579	0.7	29.7	186	64.28	85.0	even	ok
	183	C69H156N15NaO20PS2	544.3570	-0.8	29.7	187	62.05	1.0	even	ok
	184	C72H156N13O21P2S	544.3573	-0.4	29.8	188	68.75	4.0	even	ok
	185	C52H11N48NaO13PS2	544.3578	0.6	29.9	189	64.71	73.0	even	ok
	186	C56H25N28O30S2	544.3572	-0.5	29.9	190	66.68	60.0	even	ok
	187	C60H7N46NaO11PS	544.3581	1.0	30.0	191	58.17	82.0	even	ok
	188	C71H31N12NaO33P	544.3578	0.6	30.1	192	64.45	64.0	even	ok
	189	C61H139N35O13PS	544.3578	0.5	30.2	193	66.15	12.0	even	ok
	190	C71H46O40PS2	544.3576	0.2	30.3	194	71.46	51.0	even	ok
	191	C74H161N7NaO23P2S	544.3574	-0.2	30.4	195	70.58	0.0	even	ok
	192	C75H164N3O27P2S	544.3577	0.4	30.4	196	67.58	-2.0	even	ok
	193	C62H148N25NaO19PS	544.3574	-0.1	30.4	197	72.34	3.0	even	ok
	194	C64H160N11NaO29PS	544.3574	-0.1	30.4	198	72.40	-8.0	even	ok
	195	C63H11N42NaO10PS2	544.3568	-1.3	30.4	199	53.65	81.0	even	ok
	196	C72H163NO35P	544.3576	0.1	30.5	200	72.17	-6.0	even	ok
	197	C59H11N42NaO15PS	544.3576	0.2	30.6	201	70.30	77.0	even	ok
	198	C71H153N17NaO17P2S	544.3569	-1.1	30.7	202	57.06	6.0	even	ok
	199	C69H148N23O15P2S	544.3568	-1.2	30.7	203	54.39	10.0	even	ok
	200	C55H22N32NaO26S2	544.3569	-1.2	30.8	204	55.15	62.0	even	ok
	201	C63H35N14NaO35PS	544.3576	0.2	30.8	205	69.74	55.0	even	ok
	202	C75H38N4O36PS	544.3569	-1.1	30.8	206	56.89	61.0	even	ok
	203	C56H151N27O21PS2	544.3580	1.0	31.0	207	58.26	-3.0	even	ok
	204	C70H35N8NaO37P	544.3574	-0.2	31.0	208	70.33	59.0	even	ok

Compound Spectrum SmartFormula Report

Meas. m/z	#	Ion Formula	m/z	err [ppm]	mSigma	# mSigma	Score	rdb	e⁻ Conf	N-Rule
	205	C63H137N41NaOP2S2	544.3576	0.2	31.1	209	70.39	18.0	even	ok
	206	C73H159N5O31P	544.3580	0.9	31.1	210	58.37	-1.0	even	ok
	207	C64H51O43P2S2	544.3568	-1.3	31.1	211	53.46	42.0	even	ok
	208	C64H140N37O5P2S2	544.3579	0.8	31.1	212	60.03	16.0	even	ok
	209	C69H34N14O30PS2	544.3576	0.2	31.2	213	70.06	62.0	even	ok
	210	C59H18N34O23PS	544.3575	0.1	31.2	214	71.84	70.0	even	ok
	211	C73H26N14NaO32	544.3573	-0.4	31.4	215	66.62	69.0	even	ok
	212	C64H14N38O14PS2	544.3571	-0.6	31.6	216	62.07	79.0	even	ok
	213	C70H159N11O24PS2	544.3574	-0.2	31.8	218	69.02	-1.0	even	ok
	214	C51H131N51O5PS2	544.3576	0.1	31.9	219	69.80	14.0	even	ok
	215	C70H42O45P	544.3573	-0.3	31.9	220	66.22	52.0	even	ok
	216	C50H6N54O11PS2	544.3578	0.5	32.0	221	64.13	77.0	even	ok
	217	C69H26N18O31P	544.3578	0.5	32.0	222	64.15	68.0	even	ok
	218	C66H145N31NaO7P2S2	544.3580	1.0	32.3	223	55.76	12.0	even	ok
	219	C53H17N38O24S2	544.3568	-1.3	32.3	224	50.62	66.0	even	ok
	220	C71H21N20O30	544.3572	-0.5	32.4	225	62.49	73.0	even	ok
	221	C62H155N17O27PS	544.3573	-0.3	32.7	226	65.92	-4.0	even	ok
	222	C67H138N31O14S	544.3581	1.2	32.7	227	52.86	16.0	even	ok
	223	C72H27N16NaO29P	544.3583	1.5	32.7	228	48.54	69.0	even	ok
	224	C60H143N31O17PS	544.3573	-0.3	32.7	229	65.72	7.0	even	ok
	225	C55H148N31NaO17PS2	544.3577	0.3	32.7	230	65.59	-1.0	even	ok
	226	C69H143N25NaO16S	544.3582	1.3	32.8	231	50.27	12.0	even	ok
	227	C57H6N48O13PS	544.3575	0.1	32.8	232	69.10	81.0	even	ok
	228	C61H30N20O33PS	544.3575	0.1	32.9	233	68.87	59.0	even	ok
	229	C61H167N5NaO36S2	544.3571	-0.7	33.1	234	59.03	-18.0	even	ok
	230	C62H170NO40S2	544.3575	-0.0	33.2	235	68.71	-20.0	even	ok
	231	C68H30N14O35P	544.3573	-0.3	33.3	236	63.92	63.0	even	ok
	232	C55H26N30O27PS2	544.3582	1.3	33.3	237	49.90	60.0	even	ok
	233	C66H19N32NaO16PS2	544.3572	-0.5	33.3	238	61.95	75.0	even	ok
	234	C71H39N8NaO32PS2	544.3577	0.4	33.5	239	63.51	58.0	even	ok
	235	C76H163NO30PS	544.3567	-1.4	33.6	240	48.06	-2.0	even	ok
	236	C77H159NNaO32	544.3575	0.1	33.7	241	67.07	0.0	even	ok
	237	C71H160N5NaO31P	544.3572	-0.5	34.0	242	60.13	-4.0	even	ok
	238	C70H22N22O27P	544.3582	1.3	34.0	243	49.30	73.0	even	ok
	239	C62H135N39O9PS	544.3582	1.3	34.0	244	40.40	17.0	even	ok
	240	C72H164N5NaO26PS2	544.3575	-0.0	34.0	245	67.84	-5.0	even	ok
	241	C59H162N11O34S2	544.3570	-0.9	34.1	246	55.06	-14.0	even	ok
	242	C75H154N7O30	544.3575	-0.1	34.1	247	66.60	4.0	even	ok
	243	C66H135N35NaO10S	544.3578	0.5	34.2	248	60.25	18.0	even	ok
	244	C69H19N30O17P2S	544.3575	-0.1	34.3	249	66.50	78.0	even	ok
	245	C58H15N38NaO19PS	544.3572	-0.6	34.4	250	58.72	72.0	even	ok
	246	C65H139N35O8PS2	544.3569	-1.0	34.4	251	52.74	16.0	even	ok
	247	C49H3N58NaO7PS2	544.3574	-0.2	34.5	252	64.51	79.0	even	ok
	248	C54H23N34NaO23PS2	544.3579	0.7	34.6	253	57.50	62.0	even	ok
	249	C79H40NaO35P2	544.3569	-1.1	34.6	254	50.94	62.0	even	ok
	250	C77H35N6O33P2	544.3568	-1.3	34.6	255	48.57	66.0	even	ok

Compound Spectrum SmartFormula Report

Meas. m/z	#	Ion Formula	m/z	err [ppm]	mSigma	# mSigma	Score	rdb	e¯ Conf	N-Rule
	251	C64H130N41O8S	544.3577	0.3	34.6	256	62.06	22.0	even	ok
	252	C53H143N37O15PS2	544.3576	0.1	34.7	257	64.90	3.0	even	ok
	253	C50H128N55NaOPS2	544.3572	-0.5	34.8	258	58.90	16.0	even	ok
	254	C72H27N20O23P2S	544.3579	0.8	34.8	259	55.47	72.0	even	ok
	255	C62H131N41NaO8S	544.3569	-1.1	34.9	260	41.89	19.0	even	ok
	256	C71H24N24NaO19P2S	544.3576	0.1	34.9	262	64.71	74.0	even	ok
	257	C70H18N24NaO26	544.3568	-1.2	35.0	263	49.27	75.0	even	ok
	258	C69H39N4NaO41P	544.3570	-1.0	35.0	264	51.99	54.0	even	ok
	259	C67H22N28O20PS2	544.3576	0.2	35.1	265	63.47	73.0	even	ok
	260	C66H11N40O11P2S	544.3570	-0.9	35.2	266	53.43	84.0	even	ok
	261	C68H16N34NaO13P2S	544.3571	-0.7	35.2	267	55.76	80.0	even	ok
	262	C60H27N24NaO29PS	544.3572	-0.6	35.3	268	57.34	61.0	even	ok
	263	C61H152N21NaO23PS	544.3570	-0.9	35.5	269	52.11	-2.0	even	ok
	264	C65H47NaO45PS	544.3576	0.2	35.6	270	61.76	44.0	even	ok
	265	C61H3N54OP2S2	544.3581	1.2	35.6	271	48.81	90.0	even	ok
	266	C64H167N3O37PS	544.3573	-0.3	35.6	272	61.19	-15.0	even	ok
	267	C67H144N29NaO10PS2	544.3570	-0.8	35.9	273	53.07	12.0	even	ok
	268	C74H32N14NaO25P2S	544.3580	0.9	35.9	274	51.60	68.0	even	ok
	269	C60H168N7NaO33PS2	544.3581	1.1	36.0	275	48.87	-18.0	even	ok
	270	C52H18N40O21PS2	544.3578	0.5	36.3	276	57.33	66.0	even	ok
	271	C74H151N11NaO26	544.3571	-0.7	36.3	277	53.90	6.0	even	ok
	272	C60H126N47O6S	544.3568	-1.3	36.4	278	38.32	23.0	even	ok
	273	C72H42N4O36PS2	544.3580	1.0	36.5	279	49.93	56.0	even	ok
	274	C75H25N14O32	544.3581	1.1	36.6	280	48.53	72.0	even	ok
	275	C73H167NO30PS2	544.3579	0.7	36.6	281	54.53	-7.0	even	ok
	276	C70H164NNaO35P	544.3568	-1.4	36.7	282	45.18	-9.0	even	ok
	277	C63H8N48NaO3P2S2	544.3582	1.3	36.7	283	45.20	86.0	even	ok
	278	C77H30N8NaO34	544.3582	1.3	36.7	284	46.15	68.0	even	ok
	279	C56H10N44O17PS	544.3571	-0.8	36.7	285	52.90	76.0	even	ok
	280	C60H136N39NaO9PS	544.3574	-0.1	36.8	286	51.17	14.0	even	ok
	281	C52H140N41NaO11PS2	544.3572	-0.5	36.9	287	55.82	5.0	even	ok
	282	C60H42N8NaO42S2	544.3573	-0.3	36.9	288	58.23	45.0	even	ok
	283	C69H27N22NaO22PS2	544.3577	0.3	37.2	289	57.78	69.0	even	ok
	284	C68H147N25O14PS2	544.3574	-0.2	37.2	290	60.01	10.0	even	ok
	285	C63H42N6O43PS	544.3575	0.1	37.3	291	61.35	48.0	even	ok
	286	C58H163N13O31PS2	544.3580	1.0	37.3	292	49.46	-14.0	even	ok
	287	C61H45N4O46S2	544.3577	0.3	37.4	293	58.07	43.0	even	ok
	288	C67H34N10O39P	544.3569	-1.2	37.4	294	46.66	58.0	even	ok
	289	C61H132N43NaO5PS	544.3579	0.7	37.5	295	44.01	19.0	even	ok
	290	C72H156N9NaO27P	544.3577	0.3	37.5	296	48.51	1.0	even	ok
	291	C58H22N30O27PS	544.3571	-0.8	37.6	297	51.82	65.0	even	ok
	292	C58H37N14O40S2	544.3572	-0.5	37.7	298	54.73	49.0	even	ok
	293	C63H164N7NaO33PS	544.3570	-0.9	37.8	299	49.16	-13.0	even	ok
	294	C59H147N27O21PS	544.3569	-1.1	37.9	300	46.80	2.0	even	ok
	295	C57H34N18NaO36S2	544.3569	-1.2	38.0	301	45.94	51.0	even	ok
	296	C73H26N18O26PS	544.3569	-1.1	38.0	302	47.27	72.0	even	ok

Compound Spectrum SmartFormula Report

Meas. m/z	#	Ion Formula	m/z	err [ppm]	mSigma	# mSigma	Score	rdb	e⁻ Conf	N-Rule
	297	C51H15N44NaO17PS2	544.3574	-0.2	38.1	303	58.82	68.0	even	ok
	298	C74H22N18NaO28	544.3577	0.4	38.1	304	55.16	74.0	even	ok
	299	C57H160N17NaO27PS2	544.3577	0.3	38.3	305	56.63	-12.0	even	ok
	300	C72H17N24O26	544.3576	0.3	38.4	306	56.86	78.0	even	ok
	301	C68H23N22NaO27P	544.3574	-0.2	38.8	307	47.93	70.0	even	ok
	302	C73H152N17O17P2S	544.3577	0.4	38.9	309	54.42	9.0	even	ok
	303	C70H144N27O11P2S	544.3573	-0.4	38.9	310	54.39	15.0	even	ok
	304	C62H39N10NaO39PS	544.3572	-0.6	39.0	311	52.08	50.0	even	ok
	305	C50H135N47O9PS2	544.3571	-0.7	39.0	312	50.53	9.0	even	ok
	306	C70H152N19NaO16PS2	544.3575	-0.0	39.1	313	59.28	6.0	even	ok
	307	C57H16N40NaO16P2S	544.3582	1.2	39.1	314	43.66	72.0	even	ok
	308	C55H29N24O34S2	544.3568	-1.3	39.1	315	42.42	55.0	even	ok
	309	C58H131N45O7PS	544.3573	-0.3	39.2	316	46.18	18.0	even	ok
	310	C72H149N21NaO13P2S	544.3574	-0.2	39.3	317	56.01	11.0	even	ok
	311	C59H127N49O3PS	544.3578	0.5	39.4	318	43.60	23.0	even	ok
	312	C75H31N12NaO28PS	544.3570	-0.9	39.4	319	47.63	68.0	even	ok
	313	C70H151N15O25P	544.3576	0.1	39.4	320	47.84	5.0	even	ok
	314	C59H140N35NaO13PS	544.3570	-0.9	39.5	321	39.01	9.0	even	ok
	315	C70H30N18O26PS2	544.3580	1.0	39.5	322	46.09	67.0	even	ok
	316	C69H155N11O29P	544.3571	-0.7	39.6	323	41.43	0.0	even	ok
	317	C75H157N11NaO19P2S	544.3578	0.6	39.7	324	51.01	5.0	even	ok
	318	C56H3N52NaO9PS	544.3572	-0.6	39.7	325	42.37	83.0	even	ok
	319	C68H13N30O24	544.3568	-1.4	39.8	326	34.50	79.0	even	ok
	320	C68H40N6NaO38P2	544.3579	0.8	39.8	327	47.80	54.0	even	ok
	321	C55H155N23O25PS2	544.3576	0.1	39.9	328	56.37	-8.0	even	ok
	322	C61H159N13O31PS	544.3569	-1.1	39.9	329	44.34	-9.0	even	ok
	323	C69H141N31NaO7P2S	544.3569	-1.1	40.0	330	44.73	17.0	even	ok
	324	C76H160N7O23P2S	544.3582	1.2	40.0	331	42.65	3.0	even	ok
	325	C49H10N50O15PS2	544.3573	-0.3	40.0	332	53.62	72.0	even	ok
	326	C67H136N37O5P2S	544.3568	-1.2	40.1	333	42.59	21.0	even	ok
	327	C59H28N26NaO26P2S	544.3582	1.2	40.1	334	42.45	61.0	even	ok
	328	C63H127N45NaO4S	544.3573	-0.3	40.2	335	44.69	24.0	even	ok
	329	C64H7N46NaO6PS2	544.3572	-0.5	40.2	336	51.56	86.0	even	ok
	330	C67H27N18NaO31P	544.3569	-1.0	40.2	337	37.66	65.0	even	ok
	331	C57H19N34NaO23PS	544.3567	-1.4	40.4	338	40.25	67.0	even	ok
	332	C60H153N23NaO20P2S	544.3580	0.9	40.4	339	46.37	-2.0	even	ok
	333	C76H34N8O32PS	544.3574	-0.2	40.5	340	54.20	66.0	even	ok
	334	C69H19N26NaO23P	544.3578	0.6	40.5	341	41.07	75.0	even	ok
	335	C57H38N16O37PS2	544.3582	1.3	40.6	342	41.08	49.0	even	ok
	336	C72H146N17O24	544.3570	-0.9	40.8	343	38.13	10.0	even	ok
	337	C60H34N16O37PS	544.3571	-0.7	41.0	344	47.31	54.0	even	ok
	338	C71H155N15O20PS2	544.3578	0.6	41.0	345	48.51	4.0	even	ok
	339	C66H18N28O25P	544.3573	-0.4	41.0	346	43.32	74.0	even	ok
	340	C73H152N13NaO23P	544.3581	1.1	41.1	347	35.83	6.0	even	ok
	341	C61H122N51O2S	544.3572	-0.5	41.2	348	41.81	28.0	even	ok
	342	C56H35N20NaO33PS2	544.3579	0.7	41.2	349	48.06	51.0	even	ok

Compound Spectrum SmartFormula Report

Meas. m/z	#	Ion Formula	m/z	err [ppm]	mSigma	# mSigma	Score	rdb	e⁻ Conf	N-Rule
	343	C78H165NNaO25P2S	544.3583	1.4	41.3	350	39.20	-1.0	even	ok
	344	C65H10N42O10PS2	544.3576	0.2	41.4	351	53.78	84.0	even	ok
	345	C54H152N27NaO21PS2	544.3572	-0.5	41.5	352	49.42	-6.0	even	ok
	346	C78H155N5NaO28	544.3580	0.9	41.5	353	44.66	5.0	even	ok
	347	C76H150N11O26	544.3579	0.7	41.5	354	46.67	9.0	even	ok
	348	C51H141N43NaO8P2S2	544.3582	1.3	41.6	355	40.08	5.0	even	ok
	349	C49H132N51NaO5PS2	544.3568	-1.4	41.6	356	39.54	11.0	even	ok
	350	C74H151N15O20PS	544.3567	-1.4	41.7	357	38.74	9.0	even	ok
	351	C70H161N7NaO28P2	544.3582	1.3	41.7	358	33.41	-4.0	even	ok
	352	C73H41N2O39S2	544.3570	-0.8	41.8	359	45.41	56.0	even	ok
	353	C72H35N12NaO28PS2	544.3581	1.2	41.9	360	41.28	63.0	even	ok
	354	C57H135N41O11PS	544.3569	-1.1	42.1	361	34.68	13.0	even	ok
	355	C65H126N45O4S	544.3581	1.2	42.1	362	41.17	27.0	even	ok
	356	C67H131N39NaO6S	544.3582	1.3	42.2	363	39.22	23.0	even	ok
	357	C48H7N54NaO11PS2	544.3570	-1.0	42.2	364	42.87	74.0	even	ok
	358	C66H35N12O36P2	544.3579	0.7	42.2	365	46.71	58.0	even	ok
	359	C78H39N2NaO34PS	544.3575	-0.1	42.3	366	53.63	62.0	even	ok
	360	C67H14N32O21P	544.3577	0.5	42.3	367	40.78	79.0	even	ok
	361	C57H23N32O24P2S	544.3581	1.1	42.4	368	41.77	65.0	even	ok
	362	C71H147N19O21P	544.3580	0.9	42.5	369	36.08	10.0	even	ok
	363	C62H165N9NaO30P2S	544.3580	0.9	42.5	370	43.66	-13.0	even	ok
	364	C54H30N26O31PS2	544.3578	0.5	42.6	371	48.20	55.0	even	ok
	365	C58H148N29O18P2S	544.3579	0.7	42.8	372	45.35	2.0	even	ok
	366	C65H22N24O29P	544.3569	-1.2	42.8	373	33.48	69.0	even	ok
	367	C76H156N9NaO22PS	544.3568	-1.2	42.8	374	39.34	5.0	even	ok
	368	C81H39O35P2	544.3577	0.4	42.9	375	49.40	65.0	even	ok
	369	C78H31N10O29P2	544.3572	-0.5	43.0	376	47.93	71.0	even	ok
	370	C57H128N49NaO3PS	544.3570	-1.0	43.1	377	35.24	20.0	even	ok
	371	C67H15N36NaO12PS2	544.3577	0.3	43.1	378	49.20	80.0	even	ok
	372	C59H31N20NaO33PS	544.3567	-1.4	43.1	379	37.32	56.0	even	ok
	373	C73H160N9NaO22PS2	544.3579	0.8	43.2	380	43.74	0.0	even	ok
	374	C69H148N19NaO21P	544.3572	-0.5	43.2	381	39.04	7.0	even	ok
	375	C52H147N33O19PS2	544.3571	-0.7	43.3	382	44.98	-2.0	even	ok
	376	C80H36N4NaO31P2	544.3573	-0.3	43.4	383	49.37	67.0	even	ok
	377	C61H40N12NaO36P2S	544.3582	1.3	43.5	384	38.56	50.0	even	ok
	378	C68H152N15NaO25P	544.3568	-1.4	43.5	385	31.19	2.0	even	ok
	379	C77H159N5O26PS	544.3572	-0.6	43.6	386	45.75	3.0	even	ok
	380	C70H15N34O13P2S	544.3579	0.8	43.7	387	43.72	83.0	even	ok
	381	C65H132N43NaPS2	544.3570	-0.8	43.7	388	42.83	23.0	even	ok
	382	C67H7N44O7P2S	544.3575	-0.1	43.7	389	51.55	89.0	even	ok
	383	C53H27N30NaO27PS2	544.3574	-0.2	43.7	390	50.37	57.0	even	ok
	384	C49H136N49O6P2S2	544.3581	1.1	43.8	391	39.50	9.0	even	ok
	385	C55H7N48NaO13PS	544.3567	-1.4	43.8	392	30.40	78.0	even	ok
	386	C77H28N14NaO25P2	544.3569	-1.1	44.1	393	39.29	73.0	even	ok
	387	C69H12N38NaO9P2S	544.3576	0.1	44.1	394	50.45	85.0	even	ok
	388	C75H23N20O23P2	544.3568	-1.3	44.1	395	37.41	77.0	even	ok

Compound Spectrum SmartFormula Report

Meas. m/z	#	Ion Formula	m/z	err [ppm]	mSigma	# mSigma	Score	rdb	e¯ Conf	N-Rule
	389	C68H156N13O26P2	544.3581	1.1	44.2	396	32.62	0.0	even	ok
	390	C71H14N28NaO22	544.3573	-0.4	44.3	397	39.27	80.0	even	ok
	391	C66H135N39O4PS2	544.3574	-0.2	44.4	399	49.12	21.0	even	ok
	392	C72H20N28NaO15P2S	544.3580	0.9	44.5	400	40.84	79.0	even	ok
	393	C55H4N54NaO6P2S	544.3582	1.2	44.6	401	31.29	83.0	even	ok
	394	C58H141N37NaO10P2S	544.3580	0.9	44.6	402	34.36	9.0	even	ok
	395	C60H160N15O28P2S	544.3579	0.7	44.7	403	42.87	-9.0	even	ok
	396	C71H29N16O29S2	544.3570	-0.8	44.7	404	41.74	67.0	even	ok
	397	C66H4N48NaO3P2S	544.3571	-0.7	44.8	405	42.86	91.0	even	ok
	398	C68H18N32O16PS2	544.3580	1.0	44.8	406	39.83	78.0	even	ok
	399	C65H15N32NaO21P	544.3569	-1.0	44.9	407	32.93	76.0	even	ok
	400	C66H28N20NaO28P2	544.3579	0.8	45.1	408	34.35	65.0	even	ok
	401	C69H9N34O20	544.3572	-0.5	45.2	409	36.79	84.0	even	ok
	402	C51H144N37NaO15PS2	544.3568	-1.3	45.2	410	35.73	0.0	even	ok
	403	C70H15N30NaO19P	544.3583	1.5	45.2	411	28.87	80.0	even	ok
	404	C56H20N36NaO20P2S	544.3577	0.4	45.2	412	45.44	67.0	even	ok
	405	C69H165N3NaO32P2	544.3578	0.5	45.3	413	37.34	-9.0	even	ok
	406	C67H143N25O19P	544.3571	-0.7	45.3	414	35.17	11.0	even	ok
	407	C51H22N36O25PS2	544.3573	-0.3	45.4	415	46.17	61.0	even	ok
	408	C55H11N46O14P2S	544.3581	1.1	45.5	416	31.93	76.0	even	ok
	409	C59H172N3NaO37PS2	544.3577	0.3	45.5	417	46.27	-23.0	even	ok
	410	C59H35N18O34P2S	544.3581	1.1	45.5	418	38.12	54.0	even	ok
	411	C55H123N55OPS	544.3569	-1.1	45.6	419	31.34	24.0	even	ok
	412	C53H153N29NaO18P2S2	544.3582	1.3	45.8	420	35.52	-6.0	even	ok
	413	C70H144N23NaO17P	544.3576	0.3	45.8	421	38.56	12.0	even	ok
	414	C66H11N36NaO17P	544.3574	-0.2	45.9	422	39.15	81.0	even	ok
	415	C72H154N13O23S2	544.3568	-1.2	45.9	423	36.60	4.0	even	ok
	416	C68H140N33NaO6PS2	544.3575	-0.0	46.0	424	48.76	17.0	even	ok
	417	C67H44N2NaO42P2	544.3575	0.0	46.0	425	48.71	49.0	even	ok
	418	C73H13N28O22	544.3581	1.1	46.2	426	37.20	83.0	even	ok
	419	C59H46N4NaO46S2	544.3569	-1.2	46.2	427	36.50	40.0	even	ok
	420	C75H18N22NaO24	544.3582	1.3	46.3	428	35.43	79.0	even	ok
	421	C62H46N2O47PS	544.3571	-0.7	46.4	429	40.62	43.0	even	ok
	422	C71H14N32O16PS	544.3569	-1.1	46.4	430	37.27	83.0	even	ok
	423	C60H49O50S2	544.3572	-0.5	46.4	431	42.98	38.0	even	ok
	424	C68H10N36O17P	544.3582	1.3	46.4	432	29.26	84.0	even	ok
	425	C48H133N53NaO2P2S2	544.3578	0.5	46.5	433	43.26	11.0	even	ok
	426	C57H167N9O35PS2	544.3576	0.1	46.8	434	46.31	-19.0	even	ok
	427	C70H23N26NaO18PS2	544.3581	1.2	46.9	435	35.81	74.0	even	ok
	428	C47H8N56NaO8P2S2	544.3580	0.8	46.9	436	39.06	74.0	even	ok
	429	C50H19N40NaO21PS2	544.3570	-1.0	46.9	437	37.47	63.0	even	ok
	430	C68H149N21NaO18P2	544.3582	1.3	47.0	438	28.73	7.0	even	ok
	431	C73H34N10NaO31S2	544.3571	-0.7	47.1	439	40.62	63.0	even	ok
	432	C57H41N10O44S2	544.3568	-1.3	47.2	440	33.86	44.0	even	ok
	433	C75H147N15NaO22	544.3575	0.1	47.2	441	38.55	11.0	even	ok
	434	C56H136N43O8P2S	544.3579	0.7	47.3	442	33.22	13.0	even	ok

Compound Spectrum SmartFormula Report

Meas. m/z	#	Ion Formula	m/z	err [ppm]	mSigma	# mSigma	Score	rdb	e⁻ Conf	N-Rule
	435	C59H157N19NaO24P2S	544.3575	0.1	47.3	443	46.35	-7.0	even	ok
	436	C69H143N29O10PS2	544.3578	0.6	47.3	444	40.58	15.0	even	ok
	437	C63H10N38O19P	544.3569	-1.2	47.3	445	29.32	80.0	even	ok
	438	C68H139N29O15P	544.3576	0.1	47.5	446	38.14	16.0	even	ok
	439	C73H19N26NaO18PS	544.3570	-0.9	47.5	447	37.72	79.0	even	ok
	440	C73H142N21O20	544.3575	-0.1	47.7	448	38.12	15.0	even	ok
	441	C51H148N35O16P2S2	544.3581	1.1	47.7	449	35.17	-2.0	even	ok
	442	C56H129N51NaP2S	544.3580	0.9	47.7	450	31.42	20.0	even	ok
	443	C56H164N13NaO31PS2	544.3572	-0.5	47.7	451	41.31	-17.0	even	ok
	444	C64H23N26O26P2	544.3579	0.7	47.8	452	33.22	69.0	even	ok
	445	C58H32N22NaO30P2S	544.3577	0.4	47.8	453	42.07	56.0	even	ok
	446	C79H156N7O23P2	544.3570	-0.8	47.8	454	38.10	8.0	even	ok
	447	C64H123N49NaS	544.3578	0.5	47.9	455	34.43	29.0	even	ok
	448	C67H160N9O30P2	544.3577	0.3	47.9	456	35.96	-5.0	even	ok
	449	C64H6N42O15P	544.3573	-0.4	47.9	457	35.49	85.0	even	ok
	450	C61H43N6NaO43PS	544.3567	-1.4	47.9	458	32.53	45.0	even	ok
	451	C68H6N38NaO16	544.3568	-1.2	47.9	459	28.61	86.0	even	ok
	452	C81H161NNaO25P2	544.3571	-0.7	48.1	460	39.50	4.0	even	ok
	453	C74H22N22O22PS	544.3574	-0.2	48.1	461	43.48	77.0	even	ok
	454	C67H153N17NaO22P2	544.3578	0.5	48.2	462	34.35	2.0	even	ok
	455	C71H140N31O7P2S	544.3577	0.4	48.2	463	41.86	20.0	even	ok
	456	C74H159N7NaO25S2	544.3569	-1.0	48.2	464	35.85	0.0	even	ok
	457	C65H39N8O40P2	544.3574	-0.2	48.4	465	44.03	53.0	even	ok
	458	C62H172NO38P2S	544.3579	0.7	48.7	466	38.05	-20.0	even	ok
	459	C74H148N21O13P2S	544.3582	1.2	48.7	467	33.28	14.0	even	ok
	460	C48H14N46O19PS2	544.3569	-1.2	48.8	468	33.85	67.0	even	ok
	461	C73H145N25NaO9P2S	544.3578	0.6	48.8	469	39.37	16.0	even	ok
	462	C70H137N35NaO3P2S	544.3574	-0.2	48.8	470	42.54	22.0	even	ok
	463	C59H50N2O47PS2	544.3582	1.3	48.9	471	32.29	38.0	even	ok
	464	C58H47N6NaO43PS2	544.3579	0.7	49.0	472	38.24	40.0	even	ok
	465	C45H3N62O6P2S2	544.3579	0.7	49.1	473	38.22	78.0	even	ok
	466	C54H8N50NaO10P2S	544.3577	0.4	49.1	474	33.84	78.0	even	ok
	467	C71H148N23NaO12PS2	544.3579	0.8	49.2	476	36.75	11.0	even	ok
	468	C66H140N29NaO15P	544.3568	-1.4	49.2	477	26.28	13.0	even	ok
	469	C64H16N34NaO18P2	544.3579	0.8	49.3	478	30.45	76.0	even	ok
	470	C54H159N19O29PS2	544.3571	-0.7	49.3	479	37.80	-13.0	even	ok
	471	C66H144N27O16P2	544.3581	1.1	49.3	480	28.10	11.0	even	ok
	472	C57H152N25O22P2S	544.3574	-0.1	49.6	481	43.03	-3.0	even	ok
	473	C67H7N40NaO13P	544.3578	0.6	49.6	482	31.66	86.0	even	ok
	474	C69H17N30O19S2	544.3570	-0.8	49.6	483	36.00	78.0	even	ok
	475	C76H27N16NaO24PS	544.3575	-0.1	49.6	484	43.21	73.0	even	ok
	476	C76H153N15NaO15P2S	544.3583	1.4	49.8	485	30.72	10.0	even	ok
	477	C50H145N39NaO12P2S2	544.3578	0.5	49.8	486	39.09	0.0	even	ok
	478	C65H32N16NaO32P2	544.3575	0.0	49.8	487	36.31	60.0	even	ok
	479	C74H37N6O35S2	544.3575	-0.0	49.9	488	43.45	61.0	even	ok
	480	C56H27N28O28P2S	544.3576	0.3	50.0	489	41.02	60.0	even	ok

Compound Spectrum SmartFormula Report

Meas. m/z	#	Ion Formula	m/z	err [ppm]	mSigma	# mSigma	Score	rdb	e¯ Conf	N-Rule
	481	C72H139N25NaO16	544.3571	-0.7	50.0	490	30.44	17.0	even	ok
	482	C56H42N12O41PS2	544.3578	0.5	50.2	491	38.54	44.0	even	ok
	483	C57H145N33NaO14P2S	544.3575	0.1	50.2	492	35.42	4.0	even	ok
	484	C61H47N4O44P2S	544.3581	1.1	50.5	493	32.77	43.0	even	ok
	485	C65H3N50NaO2PS2	544.3577	0.3	50.5	494	39.62	91.0	even	ok
	486	C72H139N29O10PS	544.3567	-1.4	50.6	495	29.76	20.0	even	ok
	487	C53H156N23NaO25PS2	544.3568	-1.3	50.6	496	30.46	-11.0	even	ok
	488	C61H169N5NaO34P2S	544.3575	0.1	50.7	497	41.74	-18.0	even	ok
	489	C65H148N23O20P2	544.3577	0.3	50.7	498	33.07	6.0	even	ok
	490	C75H162N3O29S2	544.3573	-0.4	50.7	499	39.13	-2.0	even	ok
	491	C77H30N12O28PS	544.3578	0.6	50.8	500	37.08	71.0	even	ok
	492	C55H39N16NaO37PS2	544.3574	-0.2	50.8	501	40.93	46.0	even	ok
	493	C70H134N31O14	544.3570	-0.9	50.9	502	28.37	21.0	even	ok
	494	C71H140N27NaO13P	544.3581	1.1	50.9	503	26.93	17.0	even	ok
	495	C67H136N33NaO11P	544.3572	-0.5	51.1	504	30.88	18.0	even	ok
	496	C55H133N47NaO4P2S	544.3575	0.1	51.1	505	34.53	15.0	even	ok
	497	C49H20N42NaO18P2S2	544.3580	0.8	51.3	507	34.18	63.0	even	ok
	498	C65H163N3NaO39	544.3582	1.2	51.3	508	30.74	-13.0	even	ok
	499	C74H144N23NaO12PS	544.3568	-1.3	51.5	509	30.34	16.0	even	ok
	500	C55H165N15NaO28P2S2	544.3582	1.3	51.6	510	29.75	-17.0	even	ok
552.3542	1	C24H48N11P2	552.3564	3.9	34.0	1	33.22	8.0	even	ok
	2	C28H48N7NaOP	552.3550	1.4	35.5	2	79.28	9.0	even	ok
	3	C26H53N5NaO2P2	552.3567	4.4	41.8	3	21.44	4.0	even	ok
	4	C22H49N11NaP2	552.3540	-0.4	46.8	4	75.48	5.0	even	ok
	5	C23H52N7O4P2	552.3551	1.5	52.1	5	48.43	3.0	even	ok
	6	C31H56NOP2S	552.3552	1.8	52.3	6	57.92	6.0	even	ok
	7	C27H47N9NaS	552.3567	4.5	53.1	7	19.31	9.0	even	ok
	8	C28H56N3NaPS2	552.3545	0.6	53.2	8	100.00	3.0	even	ok
	9	C30H55N3PS2	552.3570	4.9	53.3	9	19.73	6.0	even	ok
	10	C29H57NNaOP2S	552.3528	-2.5	53.8	10	43.70	3.0	even	ok
	11	C33H51N3PS	552.3536	-1.2	54.0	11	66.84	11.0	even	ok
	12	C29H55NNaO3S2	552.3516	-4.8	54.2	12	20.24	3.0	even	ok
	13	C31H54NO3S2	552.3540	-0.5	54.8	13	97.38	6.0	even	ok
	14	C19H43N15NaO3	552.3566	4.2	56.0	14	15.42	6.0	even	ok
	15	C25H55N5O2PS2	552.3529	-2.3	56.1	15	54.06	2.0	even	ok
	16	C26H61NNaOP2S2	552.3562	3.6	56.3	16	33.59	-2.0	even	ok
	17	C26H51N5NaO4S	552.3554	2.1	56.5	17	46.03	4.0	even	ok
	18	C24H46N11O2S	552.3551	1.6	56.8	18	53.66	8.0	even	ok
	19	C27H54NO8S	552.3565	4.1	57.1	19	21.29	2.0	even	ok
	20	C17H38N21O	552.3563	3.7	57.4	20	18.45	10.0	even	ok
	21	C27H42N11O2	552.3517	-4.5	57.6	21	17.11	13.0	even	ok
	22	C21H53N7NaO4P2	552.3526	-2.9	59.3	22	24.23	0.0	even	ok
	23	C19H48N13O2P2	552.3524	-3.4	60.6	23	19.03	4.0	even	ok
	24	C24H59NO6PS2	552.3516	-4.8	61.3	24	16.49	-3.0	even	ok
	25	C23H60N3O3P2S2	552.3546	0.6	61.5	25	74.82	-3.0	even	ok
	26	C22H47N11NaO2S	552.3527	-2.7	62.1	26	30.73	5.0	even	ok

Compound Spectrum SmartFormula Report

Meas. m/z	#	Ion Formula	m/z	err [ppm]	mSigma	# mSigma	Score	rdb	e¯ Conf	N-Rule
	27	C25H55NNaO8S	552.3541	-0.3	62.2	27	63.63	-1.0	even	ok
	28	C23H50N7O6S	552.3538	-0.8	62.4	28	55.62	3.0	even	ok
	29	C25H47N9O3P	552.3534	-1.5	62.5	29	45.86	8.0	even	ok
	30	C20H42N17S	552.3524	-3.2	62.5	30	24.90	9.0	even	ok
	31	C22H51N9O3PS	552.3568	4.6	63.9	31	13.09	3.0	even	ok
	32	C30H43N9Na	552.3534	-1.6	64.8	32	41.45	14.0	even	ok
	33	C29H47N5NaO4	552.3520	-4.0	65.0	33	16.76	9.0	even	ok
	34	C29H51N3O5P	552.3561	3.4	65.4	34	21.49	7.0	even	ok
	35	C31H46N5O4	552.3544	0.4	65.5	35	55.75	12.0	even	ok
	36	C30H50NO8	552.3531	-2.1	66.0	36	34.09	7.0	even	ok
	37	C34H52NOP2	552.3519	-4.3	66.7	37	13.85	11.0	even	ok
	38	C32H42N9	552.3558	2.8	67.4	38	25.03	17.0	even	ok
	39	C27H52N3NaO5P	552.3537	-1.0	67.4	39	44.41	4.0	even	ok
	40	C34H47N3NaO2	552.3560	3.3	68.0	40	20.11	13.0	even	ok
	41	C18H47N11NaO7	552.3552	1.8	68.8	41	25.17	1.0	even	ok
	42	C22H54N3O10S	552.3524	-3.2	69.8	42	19.34	-2.0	even	ok
	43	C16H42N17O5	552.3549	1.3	69.9	43	28.06	5.0	even	ok
	44	C15H39N21NaO	552.3539	-0.7	70.1	44	32.98	7.0	even	ok
	45	C20H52N9NaO3PS	552.3544	0.2	70.9	45	47.14	0.0	even	ok
	46	C17H46N17S2	552.3558	2.9	71.3	46	21.16	4.0	even	ok
	47	C21H55N5O7PS	552.3554	2.2	71.3	47	26.80	-2.0	even	ok
	48	C19H51N11NaO2S2	552.3561	3.4	71.4	48	17.29	0.0	even	ok
	49	C18H47N15OPS	552.3541	-0.3	71.5	49	46.06	4.0	even	ok
	50	C24H51N5O7P	552.3521	-3.9	72.2	50	13.26	3.0	even	ok
	51	C25H57NNaO6P2	552.3553	2.0	72.8	51	26.93	-1.0	even	ok
	52	C19H56N9OP2S2	552.3519	-4.2	73.4	52	11.08	-2.0	even	ok
	53	C21H61N3NaO3P2S2	552.3522	-3.7	73.5	53	13.79	-6.0	even	ok
	54	C19H56N5NaO7PS	552.3530	-2.2	79.3	54	19.73	-5.0	even	ok
	55	C16H48N15NaOPS	552.3517	-4.6	79.5	55	7.24	1.0	even	ok
	56	C22H56N3O8P2	552.3537	-0.9	79.6	56	28.42	-2.0	even	ok
	57	C17H51N11O5PS	552.3527	-2.7	79.8	57	16.18	-1.0	even	ok
	58	C20H59NO11PS	552.3541	-0.2	80.0	58	33.17	-7.0	even	ok
	59	C19H50N7O11	552.3563	3.7	82.1	59	9.78	-1.0	even	ok
	60	C21H55NNaO13	552.3566	4.2	85.3	60	6.82	-5.0	even	ok
	61	C18H54N3O15	552.3549	1.3	90.5	61	16.13	-6.0	even	ok
	62	C17H51N7NaO11	552.3539	-0.6	90.7	62	19.12	-4.0	even	ok
559.0332	1	C73H144N27NaO14P	559.0335	0.5	15.1	1	81.24	17.0	even	ok
	2	C70H7N44NaO10P	559.0337	0.9	15.2	3	74.22	91.0	even	ok
	3	C71H139N33O12P	559.0334	0.4	15.3	4	84.43	21.0	even	ok
	4	C70H136N37NaO8P	559.0330	-0.3	16.2	5	85.29	23.0	even	ok
	5	C74H147N23O18P	559.0339	1.2	16.3	7	66.90	15.0	even	ok
	6	C74H143N25NaO17	559.0325	-1.2	16.5	8	65.58	17.0	even	ok
	7	C71H13N34O21	559.0326	-1.1	16.5	9	82.71	84.0	even	ok
	8	C72H135N37O8P	559.0339	1.2	17.1	10	65.91	26.0	even	ok
	9	C69H11N40NaO14P	559.0332	0.1	17.2	11	86.98	86.0	even	ok
	10	C70H14N36O18P	559.0336	0.7	17.3	12	74.19	84.0	even	ok

Compound Spectrum SmartFormula Report

Meas. m/z	#	Ion Formula	m/z	err [ppm]	mSigma	# mSigma	Score	rdb	e⁻ Conf	N-Rule
	11	C71H132N41NaO4P	559.0335	0.5	17.4	13	77.76	28.0	even	ok
	12	C71H6N42NaO13	559.0327	-0.9	17.4	14	70.79	91.0	even	ok
	13	C72H19N30NaO20P	559.0337	0.9	17.5	15	70.70	80.0	even	ok
	14	C75H146N21O21	559.0329	-0.6	17.5	16	91.50	15.0	even	ok
	15	C73H18N28NaO23	559.0327	-0.9	17.6	17	84.77	80.0	even	ok
	16	C68H131N43O6P	559.0330	-0.4	17.6	18	79.60	27.0	even	ok
	17	C69H127N47O2P	559.0334	0.4	17.6	19	80.60	32.0	even	ok
	18	C67H6N46O12P	559.0331	-0.1	18.2	20	85.30	90.0	even	ok
	19	C72H131N39NaO7	559.0325	-1.2	19.1	21	62.04	28.0	even	ok
	20	C77H151N15NaO23	559.0329	-0.4	19.3	22	92.01	11.0	even	ok
	21	C72H9N38O17	559.0330	-0.3	19.4	23	79.69	89.0	even	ok
	22	C75H156N13NaO24P	559.0335	0.6	19.5	24	89.02	6.0	even	ok
	23	C65H131N45NaO5S	559.0327	-0.8	19.7	25	82.25	24.0	even	ok
	24	C66H3N50NaO8P	559.0328	-0.7	19.7	26	70.72	92.0	even	ok
	25	C66H127N49NaOS	559.0332	-0.0	19.7	27	100.00	29.0	even	ok
	26	C76H155N11NaO27	559.0325	-1.2	19.9	28	73.46	6.0	even	ok
	27	C74H14N32NaO19	559.0331	-0.1	19.9	29	98.34	85.0	even	ok
	28	C67H128N47NaO2P	559.0326	-1.1	20.1	30	63.95	29.0	even	ok
	29	C73H134N35O11	559.0329	-0.6	20.1	31	72.14	26.0	even	ok
	30	C74H21N24O27	559.0331	-0.3	20.1	32	94.38	78.0	even	ok
	31	C63H126N51O3S	559.0326	-1.0	20.3	33	64.71	28.0	even	ok
	32	C62H127N53PS	559.0336	0.8	20.4	34	68.58	28.0	even	ok
	33	C64H132N47NaO2PS	559.0337	0.9	20.5	35	65.42	24.0	even	ok
	34	C72H148N23NaO18P	559.0331	-0.3	20.6	36	77.90	12.0	even	ok
	35	C73H151N19O22P	559.0334	0.4	21.0	37	74.84	10.0	even	ok
	36	C76H159N9O28P	559.0339	1.2	21.0	38	72.54	4.0	even	ok
	37	C70H143N29O16P	559.0330	-0.4	21.6	39	73.23	16.0	even	ok
	38	C76H26N18NaO29	559.0331	-0.1	21.8	40	94.50	74.0	even	ok
	39	C75H139N29NaO13	559.0329	-0.4	21.9	41	72.36	22.0	even	ok
	40	C78H154N11O27	559.0333	0.2	22.2	43	91.26	9.0	even	ok
	41	C65H123N53P	559.0325	-1.2	22.2	44	58.21	33.0	even	ok
	42	C66H134N41O9S	559.0331	-0.2	22.2	45	91.31	22.0	even	ok
	43	C77H158N7O31	559.0329	-0.6	22.3	46	82.76	4.0	even	ok
	44	C75H17N28O23	559.0335	0.5	22.6	47	83.31	83.0	even	ok
	45	C69H140N33NaO12P	559.0326	-1.1	22.7	48	60.57	18.0	even	ok
	46	C67H130N45O5S	559.0335	0.6	22.7	49	82.05	27.0	even	ok
	47	C71H23N26NaO24P	559.0332	0.1	22.7	50	92.62	75.0	even	ok
	48	C63H7N50NaO8PS	559.0339	1.3	23.2	53	67.09	87.0	even	ok
	49	C73H25N20O31	559.0326	-1.1	23.3	54	71.64	73.0	even	ok
	50	C72H26N22O28P	559.0336	0.7	23.5	55	77.81	73.0	even	ok
	51	C74H31N16NaO30P	559.0337	0.9	23.7	56	74.18	69.0	even	ok
	52	C68H139N35NaO11S	559.0332	-0.0	23.7	57	91.71	18.0	even	ok
	53	C79H163NNaO33	559.0330	-0.4	23.8	58	83.55	0.0	even	ok
	54	C64H6N48NaO11S	559.0329	-0.5	23.9	59	82.10	87.0	even	ok
	55	C65H4N52NaO5P2	559.0338	1.1	24.1	60	58.48	92.0	even	ok
	56	C68H8N48NaO4P2S	559.0325	-1.2	24.1	61	67.07	91.0	even	ok

Compound Spectrum SmartFormula Report

Meas. m/z	#	Ion Formula	m/z	err [ppm]	mSigma	# mSigma	Score	rdb	e⁻ Conf	N-Rule
	57	C75H30N14NaO33	559.0327	-0.9	24.2	62	73.63	69.0	even	ok
	58	C76H142N25O17	559.0333	0.2	24.2	63	72.84	20.0	even	ok
	59	C80H159N5NaO29	559.0334	0.4	24.4	64	83.57	5.0	even	ok
	60	C67H135N39O10P	559.0325	-1.2	24.5	65	55.49	22.0	even	ok
	61	C78H147N19NaO19	559.0334	0.4	24.5	66	83.52	16.0	even	ok
	62	C69H135N39NaO7S	559.0336	0.8	24.9	67	74.79	23.0	even	ok
	63	C77H22N22NaO25	559.0336	0.7	24.9	68	75.88	79.0	even	ok
	64	C77H29N14O33	559.0335	0.5	25.0	69	78.78	72.0	even	ok
	65	C69H18N32O22P	559.0332	-0.1	25.2	70	73.33	79.0	even	ok
	66	C66H5N48O11S	559.0337	0.9	25.3	71	70.62	90.0	even	ok
	67	C68H15N36NaO18P	559.0328	-0.7	25.4	73	62.46	81.0	even	ok
	68	C70H136N41O2P2S	559.0327	-0.9	26.2	74	69.34	26.0	even	ok
	69	C65H9N44O15S	559.0333	0.1	26.5	75	83.86	85.0	even	ok
	70	C76H33N10O37	559.0331	-0.2	26.7	76	81.48	67.0	even	ok
	71	C66H10N42O16P	559.0327	-0.9	26.8	77	57.85	85.0	even	ok
	72	C80H35N10O30P2	559.0326	-1.0	26.8	78	67.46	71.0	even	ok
	73	C65H138N37O13S	559.0326	-1.0	26.9	79	67.16	17.0	even	ok
	74	C69H142N31O15S	559.0335	0.6	26.9	80	74.38	16.0	even	ok
	75	C68H141N35NaO9P2	559.0336	0.7	27.0	81	59.96	18.0	even	ok
	76	C74H160N9NaO28P	559.0331	-0.2	27.0	82	80.95	1.0	even	ok
	77	C70H20N34NaO14P2S	559.0325	-1.2	27.1	83	62.83	80.0	even	ok
	78	C79H34N8NaO35	559.0336	0.7	27.1	84	71.90	68.0	even	ok
	79	C71H122N49O	559.0328	-0.6	27.2	85	61.38	37.0	even	ok
	80	C79H150N15O23	559.0338	1.0	27.2	86	66.48	14.0	even	ok
	81	C64H139N39O10PS	559.0336	0.8	27.3	87	70.39	17.0	even	ok
	82	C73H5N42O13	559.0335	0.5	27.3	88	62.51	94.0	even	ok
	83	C66H144N33NaO12PS	559.0337	1.0	27.4	89	67.14	13.0	even	ok
	84	C69H11N44O8P2S	559.0329	-0.6	27.4	90	73.90	89.0	even	ok
	85	C72H148N27O12P2S	559.0327	-0.9	27.4	91	67.58	15.0	even	ok
	86	C78H23N24O20P2	559.0326	-1.0	27.4	92	66.40	82.0	even	ok
	87	C68H10N42NaO13S	559.0338	1.1	27.4	93	64.15	86.0	even	ok
	88	C67H143N31NaO15S	559.0327	-0.8	27.5	94	69.17	13.0	even	ok
	89	C72H155N15O26P	559.0330	-0.4	27.6	95	76.69	5.0	even	ok
	90	C81H162NO33	559.0338	1.0	27.8	96	65.49	3.0	even	ok
	91	C83H165NNaO26P2	559.0325	-1.2	27.9	97	62.61	4.0	even	ok
	92	C75H163N5O32P	559.0334	0.4	27.9	98	76.64	-1.0	even	ok
	93	C67H14N38NaO17S	559.0334	0.3	28.0	99	77.96	81.0	even	ok
	94	C78H38N4NaO39	559.0331	-0.1	28.1	100	82.01	63.0	even	ok
	95	C73H127N43NaO3	559.0329	-0.4	28.5	101	62.09	33.0	even	ok
	96	C63H136N43NaO6PS	559.0333	0.2	28.6	102	66.54	19.0	even	ok
	97	C72H141N35NaO4P2S	559.0328	-0.8	28.6	103	68.53	22.0	even	ok
	98	C71H147N25NaO17S	559.0336	0.8	28.8	104	68.08	12.0	even	ok
	99	C66H136N41O7P2	559.0335	0.6	29.1	105	59.47	22.0	even	ok
	100	C60H128N53NaPS	559.0328	-0.7	29.1	106	58.06	25.0	even	ok
	101	C61H131N49O4PS	559.0332	-0.0	29.1	107	67.65	23.0	even	ok
	102	C67H16N38NaO15P2	559.0338	1.1	29.2	108	51.81	81.0	even	ok

Compound Spectrum SmartFormula Report

Meas. m/z	#	Ion Formula	m/z	err [ppm]	mSigma	# mSigma	Score	rdb	e¯ Conf	N-Rule
	103	C82H40N4NaO32P2	559.0327	-0.8	29.3	109	66.61	67.0	even	ok
	104	C75H10N36NaO15	559.0336	0.7	29.6	110	56.77	90.0	even	ok
	105	C74H130N39O7	559.0333	0.2	29.6	111	64.33	31.0	even	ok
	106	C81H155N9NaO25	559.0338	1.2	29.7	112	59.89	10.0	even	ok
	107	C74H153N21NaO14P2S	559.0328	-0.8	29.7	113	66.82	11.0	even	ok
	108	C80H28N18NaO22P2	559.0327	-0.8	29.8	114	65.66	78.0	even	ok
	109	C71H16N38NaO10P2S	559.0330	-0.4	29.8	115	72.65	85.0	even	ok
	110	C63H10N44NaO15S	559.0325	-1.3	29.9	116	57.46	82.0	even	ok
	111	C68H146N27O19S	559.0331	-0.2	30.0	117	76.44	11.0	even	ok
	112	C71H152N19NaO22P	559.0326	-1.1	30.1	118	51.08	7.0	even	ok
	113	C81H153N15NaO16P2	559.0325	-1.2	30.5	119	58.64	15.0	even	ok
	114	C71H23N30O18P2S	559.0329	-0.6	30.6	120	68.58	78.0	even	ok
	115	C69H4N52NaP2S	559.0330	-0.4	30.7	121	71.09	96.0	even	ok
	116	C62H11N46NaO12PS	559.0335	0.5	30.8	122	69.59	82.0	even	ok
	117	C65H11N44O13P2	559.0337	0.9	31.0	123	51.91	85.0	even	ok
	118	C68H17N34O21S	559.0337	1.0	31.1	124	61.35	79.0	even	ok
	119	C70H151N21NaO21S	559.0332	-0.0	31.2	125	77.14	7.0	even	ok
	120	C60H6N52O10PS	559.0334	0.3	31.2	126	71.89	86.0	even	ok
	121	C79H41O43	559.0335	0.6	31.2	127	67.90	61.0	even	ok
	122	C69H147N25O20P	559.0325	-1.2	31.4	128	47.22	11.0	even	ok
	123	C76H135N33NaO9	559.0334	0.4	31.5	129	58.98	27.0	even	ok
	124	C73H35N12NaO34P	559.0332	0.1	31.5	130	75.13	64.0	even	ok
	125	C70H27N22NaO28P	559.0328	-0.7	31.6	131	64.84	70.0	even	ok
	126	C59H3N60P2S2	559.0326	-1.0	31.6	132	60.16	91.0	even	ok
	127	C65H133N45NaO3P2	559.0332	-0.1	31.7	133	62.71	24.0	even	ok
	128	C64H13N40O19S	559.0328	-0.7	31.8	134	65.33	80.0	even	ok
	129	C73H144N31O8P2S	559.0331	-0.1	31.9	135	74.04	20.0	even	ok
	130	C71H30N18O32P	559.0332	-0.1	31.9	136	74.99	68.0	even	ok
	131	C63H14N42O16PS	559.0338	1.1	32.1	137	57.02	80.0	even	ok
	132	C65H19N36NaO18PS	559.0339	1.3	32.3	138	54.05	76.0	even	ok
	133	C75H37N6O41	559.0326	-1.0	32.4	139	57.98	62.0	even	ok
	134	C74H160N13O22P2S	559.0327	-0.9	32.4	140	60.00	4.0	even	ok
	135	C63H136N47P2S2	559.0329	-0.5	32.4	141	66.41	22.0	even	ok
	136	C66H18N34NaO21S	559.0329	-0.5	32.5	142	66.96	76.0	even	ok
	137	C74H38N8O38P	559.0336	0.7	32.7	143	62.45	62.0	even	ok
	138	C81H31N14O26P2	559.0331	-0.2	32.8	144	71.45	76.0	even	ok
	139	C73H28N24NaO20P2S	559.0330	-0.4	32.8	145	67.62	74.0	even	ok
	140	C83H43O36P2	559.0331	-0.2	32.8	146	71.45	65.0	even	ok
	141	C76H43N2NaO40P	559.0337	0.9	32.9	147	59.48	58.0	even	ok
	142	C64H8N48NaO9P2	559.0333	0.3	32.9	148	58.20	87.0	even	ok
	143	C70H22N28NaO23S	559.0338	1.1	32.9	149	56.01	75.0	even	ok
	144	C79H143N23NaO15	559.0338	1.2	33.0	150	55.30	21.0	even	ok
	145	C77H42NaO43	559.0327	-0.9	33.0	151	59.72	58.0	even	ok
	146	C82H156N11O20P2	559.0329	-0.5	33.0	152	65.33	13.0	even	ok
	147	C61H8N54NaO2P2S2	559.0327	-0.8	33.3	153	60.32	87.0	even	ok
	148	C72H32N20NaO24P2S	559.0325	-1.2	33.4	154	53.96	69.0	even	ok

Compound Spectrum SmartFormula Report

Meas. m/z	#	Ion Formula	m/z	err [ppm]	mSigma	# mSigma	Score	rdb	e⁻ Conf	N-Rule
	149	C72H19N34O14P2S	559.0333	0.2	33.4	155	69.80	83.0	even	ok
	150	C75H156N17O18P2S	559.0331	-0.1	33.4	156	71.37	9.0	even	ok
	151	C59H3N56NaO6PS	559.0330	-0.3	33.4	157	56.91	88.0	even	ok
	152	C77H138N29O13	559.0337	1.0	33.6	158	47.56	25.0	even	ok
	153	C70H7N48O4P2S	559.0333	0.2	33.6	159	69.49	94.0	even	ok
	154	C70H153N21NaO19P2	559.0336	0.7	33.7	160	50.84	7.0	even	ok
	155	C60H130N51O3S2	559.0338	1.0	33.9	161	56.31	23.0	even	ok
	156	C71H154N17O25S	559.0335	0.6	34.1	162	62.23	5.0	even	ok
	157	C63H128N51OP2	559.0331	-0.2	34.2	163	56.72	28.0	even	ok
	158	C76H165N7NaO24P2S	559.0328	-0.8	34.4	164	59.59	0.0	even	ok
	159	C75H149N25NaO10P2S	559.0332	0.0	34.4	165	70.81	16.0	even	ok
	160	C65H141N41NaO2P2S2	559.0330	-0.4	34.5	166	65.64	18.0	even	ok
	161	C76H11N38O10P2	559.0326	-1.0	34.9	167	46.00	93.0	even	ok
	162	C67H21N30O25S	559.0333	0.2	35.0	168	68.00	74.0	even	ok
	163	C62H3N54O7P2	559.0333	0.1	35.1	169	57.18	91.0	even	ok
	164	C68H148N27O17P2	559.0335	0.6	35.3	170	50.86	11.0	even	ok
	165	C83H36N8NaO28P2	559.0332	-0.0	35.3	171	69.54	72.0	even	ok
	166	C68H22N28O26P	559.0327	-0.9	35.3	172	46.99	74.0	even	ok
	167	C84H161N5NaO22P2	559.0330	-0.4	35.4	173	63.99	9.0	even	ok
	168	C62H140N39NaO10PS	559.0328	-0.6	35.5	174	59.58	14.0	even	ok
	169	C62H135N45NaO5S2	559.0338	1.2	35.6	175	51.54	19.0	even	ok
	170	C65H148N29NaO16PS	559.0333	0.2	35.6	176	66.94	8.0	even	ok
	171	C56H126N57OS2	559.0329	-0.6	35.7	177	60.08	24.0	even	ok
	172	C73H159N11NaO27S	559.0336	0.8	35.7	178	57.24	1.0	even	ok
	173	C77H161N11NaO20P2S	559.0332	0.0	35.9	180	68.13	5.0	even	ok
	174	C63H143N35O14PS	559.0332	-0.0	35.9	181	68.56	12.0	even	ok
	175	C74H24N28NaO16P2S	559.0334	0.4	36.0	182	62.77	79.0	even	ok
	176	C69H26N24NaO27S	559.0334	0.3	36.1	183	63.48	70.0	even	ok
	177	C72H12N42NaO6P2S	559.0334	0.4	36.2	184	62.54	90.0	even	ok
	178	C79H19N28O16P2	559.0331	-0.2	36.2	185	65.34	87.0	even	ok
	179	C67H150N23O23S	559.0326	-1.0	36.3	186	53.29	6.0	even	ok
	180	C58H131N51NaO3S2	559.0330	-0.4	36.3	187	61.66	20.0	even	ok
	181	C64H145N37NaO6P2S2	559.0325	-1.2	36.3	188	50.89	13.0	even	ok
	182	C73H164N5NaO32P	559.0326	-1.0	36.3	189	52.45	-4.0	even	ok
	183	C73H137N39NaP2S	559.0332	0.0	36.4	190	67.43	27.0	even	ok
	184	C74H31N20O24P2S	559.0333	0.2	36.5	191	64.27	72.0	even	ok
	185	C62H11N50O6P2S2	559.0331	-0.2	36.7	192	64.70	85.0	even	ok
	186	C74H167NO36P	559.0330	-0.4	36.7	193	61.20	-6.0	even	ok
	187	C73H35N16O28P2S	559.0329	-0.6	36.8	194	58.71	67.0	even	ok
	188	C66H151N25O20PS	559.0336	0.8	36.8	195	55.45	6.0	even	ok
	189	C69H155N17NaO25S	559.0327	-0.8	36.8	196	54.96	2.0	even	ok
	190	C63H7N54O2P2S2	559.0335	0.6	36.8	197	57.89	90.0	even	ok
	191	C67H145N31NaO13P2	559.0332	-0.1	36.8	198	55.06	13.0	even	ok
	192	C68H156N19NaO22PS	559.0337	1.0	36.9	199	52.84	2.0	even	ok
	193	C78H16N32NaO12P2	559.0327	-0.8	37.1	200	45.39	89.0	even	ok
	194	C71H159N11O30P	559.0325	-1.2	37.2	201	48.92	0.0	even	ok

Compound Spectrum SmartFormula Report

Meas. m/z	#	Ion Formula	m/z	err [ppm]	mSigma	# mSigma	Score	rdb	e⁻ Conf	N-Rule
	195	C69H28N24NaO25P2	559.0338	1.1	37.5	202	41.97	70.0	even	ok
	196	C76H152N21O14P2S	559.0336	0.7	38.0	204	55.40	14.0	even	ok
	197	C77H168N3O28P2S	559.0331	-0.1	38.1	205	63.31	-2.0	even	ok
	198	C66H144N37O6P2S2	559.0334	0.3	38.2	206	60.70	16.0	even	ok
	199	C85H164NO26P2	559.0333	0.3	38.4	207	60.51	7.0	even	ok
	200	C81H24N22NaO18P2	559.0332	-0.0	38.5	208	63.84	83.0	even	ok
	201	C59H134N47O7S2	559.0333	0.2	38.6	209	60.98	18.0	even	ok
	202	C84H39N4O32P2	559.0335	0.6	38.7	210	55.16	70.0	even	ok
	203	C75H40N10NaO30P2S	559.0330	-0.4	38.7	211	58.17	63.0	even	ok
	204	C64H16N44NaO8P2S2	559.0332	-0.0	38.7	212	63.81	81.0	even	ok
	205	C67H23N30O23P2	559.0337	0.9	38.8	213	42.39	74.0	even	ok
	206	C65H140N37O11P2	559.0331	-0.2	38.9	214	50.17	17.0	even	ok
	207	C76H36N14NaO26P2S	559.0334	0.4	38.9	215	57.95	68.0	even	ok
	208	C72H165N7NaO29P2	559.0336	0.7	39.1	216	52.88	-4.0	even	ok
	209	C70H29N20O31S	559.0337	1.0	39.1	217	49.86	68.0	even	ok
	210	C70H158N13O29S	559.0331	-0.2	39.1	218	60.54	0.0	even	ok
	211	C65H12N48NaO4P2S2	559.0336	0.8	39.2	219	51.98	86.0	even	ok
	212	C74H140N35O4P2S	559.0336	0.7	39.3	220	53.53	25.0	even	ok
	213	C73H15N38O10P2S	559.0338	1.0	39.5	221	48.59	88.0	even	ok
	214	C65H148N33O10P2S2	559.0329	-0.5	39.5	222	55.31	11.0	even	ok
	215	C60H135N45O8PS	559.0327	-0.8	39.5	223	42.65	18.0	even	ok
	216	C61H139N41NaO9S2	559.0334	0.4	39.6	224	57.04	14.0	even	ok
	217	C65H22N30NaO25S	559.0325	-1.3	39.6	225	44.84	71.0	even	ok
	218	C78H164N7O24P2S	559.0336	0.7	39.7	226	52.79	3.0	even	ok
	219	C75H27N24O20P2S	559.0338	1.0	39.7	227	48.19	77.0	even	ok
	220	C79H141N29NaO6P2	559.0325	-1.2	39.7	228	38.48	26.0	even	ok
	221	C66H20N34NaO19P2	559.0333	0.3	39.7	229	48.56	76.0	even	ok
	222	C61H15N46O10P2S2	559.0326	-1.0	39.9	230	48.61	80.0	even	ok
	223	C61H15N42NaO16PS	559.0330	-0.3	39.9	231	57.61	77.0	even	ok
	224	C72H163N7NaO31S	559.0332	-0.0	40.1	232	61.28	-4.0	even	ok
	225	C67H140N41O2P2S2	559.0338	1.1	40.1	233	46.89	21.0	even	ok
	226	C68H149N31NaO8P2S2	559.0334	0.4	40.4	234	54.86	12.0	even	ok
	227	C64H23N32NaO22PS	559.0335	0.5	40.5	235	54.02	71.0	even	ok
	228	C72H34N14NaO33S	559.0338	1.1	40.6	236	45.73	64.0	even	ok
	229	C59H10N48O14PS	559.0329	-0.5	40.6	237	54.38	81.0	even	ok
	230	C78H157N15NaO16P2S	559.0337	0.8	40.6	238	49.31	10.0	even	ok
	231	C62H18N38O20PS	559.0334	0.3	40.7	239	55.95	75.0	even	ok
	232	C60H122N55O4	559.0339	1.3	40.8	240	36.18	29.0	even	ok
	233	C72H39N8NaO38P	559.0328	-0.7	40.8	241	51.03	59.0	even	ok
	234	C75H126N43O3	559.0337	1.0	40.8	242	39.34	36.0	even	ok
	235	C64H137N41NaO7P2	559.0327	-0.9	41.0	244	40.32	19.0	even	ok
	236	C82H27N18O22P2	559.0335	0.6	41.2	245	51.59	81.0	even	ok
	237	C63H20N40NaO12P2S2	559.0327	-0.8	41.2	246	48.96	76.0	even	ok
	238	C67H153N27NaO12P2S2	559.0330	-0.3	41.3	247	54.93	7.0	even	ok
	239	C64H6N52O5PS2	559.0325	-1.2	41.4	248	44.20	90.0	even	ok
	240	C70H34N14O36P	559.0327	-0.9	41.4	249	47.96	63.0	even	ok

Compound Spectrum SmartFormula Report

Meas. m/z	#	Ion Formula	m/z	err [ppm]	mSigma	# mSigma	Score	rdb	e¯ Conf	N-Rule
	241	C73H42N4O42P	559.0332	-0.1	41.5	250	58.36	57.0	even	ok
	242	C66H25N26O29S	559.0328	-0.6	41.5	251	50.71	69.0	even	ok
	243	C64H15N40O17P2	559.0333	0.1	41.5	252	48.09	80.0	even	ok
	244	C57H6N54NaO9S2	559.0331	-0.1	41.5	253	58.06	83.0	even	ok
	245	C74H44N6NaO34P2S	559.0325	-1.2	41.5	254	43.55	58.0	even	ok
	246	C80H144N25O10P2	559.0329	-0.5	41.6	255	43.34	24.0	even	ok
	247	C61H141N41NaO7P2S	559.0338	1.1	41.6	256	36.91	14.0	even	ok
	248	C76H145N29NaO6P2S	559.0337	0.8	41.9	257	47.73	21.0	even	ok
	249	C65H26N28O26PS	559.0338	1.1	41.9	258	43.92	69.0	even	ok
	250	C68H30N20NaO31S	559.0329	-0.5	42.1	259	52.04	65.0	even	ok
	251	C67H31N22NaO28PS	559.0339	1.3	42.1	260	41.61	65.0	even	ok
	252	C75H20N32NaO12P2S	559.0339	1.2	42.1	261	43.08	84.0	even	ok
	253	C71H3N52P2S	559.0338	1.0	42.2	262	45.18	99.0	even	ok
	254	C76H43N6O34P2S	559.0333	0.2	42.3	263	54.94	61.0	even	ok
	255	C80H169NNaO26P2S	559.0337	0.8	42.3	264	47.05	-1.0	even	ok
	256	C65H19N40O12P2S2	559.0335	0.6	42.3	265	49.82	79.0	even	ok
	257	C77H32N18NaO22P2S	559.0339	1.2	42.4	266	42.72	73.0	even	ok
	258	C83H152N15O16P2	559.0333	0.3	42.4	267	54.25	18.0	even	ok
	259	C62H142N37O13S2	559.0338	1.0	42.5	268	44.75	12.0	even	ok
	260	C69H145N35NaO4P2S2	559.0339	1.2	42.7	269	41.61	17.0	even	ok
	261	C77H131N37NaO5	559.0338	1.2	42.7	270	35.57	32.0	even	ok
	262	C77H39N10O30P2S	559.0338	1.0	42.8	271	44.17	66.0	even	ok
	263	C73H166N3O35S	559.0335	0.6	42.8	272	49.20	-6.0	even	ok
	264	C59H136N47O5P2S	559.0337	1.0	42.8	273	37.35	18.0	even	ok
	265	C63H12N44NaO13P2	559.0329	-0.5	43.1	274	41.60	82.0	even	ok
	266	C71H40N10NaO35P2	559.0338	1.1	43.2	275	42.98	59.0	even	ok
	267	C62H132N47O5P2	559.0326	-1.0	43.3	276	36.11	23.0	even	ok
	268	C77H151N19O17PS	559.0326	-1.1	43.4	277	42.45	14.0	even	ok
	269	C59H119N59Na	559.0336	0.6	43.5	278	39.91	31.0	even	ok
	270	C58H133N51NaOP2S	559.0334	0.3	43.5	279	43.11	20.0	even	ok
	271	C84H32N12NaO24P2	559.0336	0.8	43.7	280	46.04	77.0	even	ok
	272	C57H135N47NaO7S2	559.0325	-1.2	43.7	281	40.66	15.0	even	ok
	273	C64H147N31NaO15S2	559.0339	1.2	43.8	282	41.13	8.0	even	ok
	274	C70H160N13O27P2	559.0335	0.6	43.9	283	40.27	0.0	even	ok
	275	C82H149N19NaO12P2	559.0330	-0.4	43.9	284	42.29	20.0	even	ok
	276	C58H9N50O13S2	559.0335	0.6	43.9	285	48.43	81.0	even	ok
	277	C66H11N46NaO7PS2	559.0326	-1.0	44.0	286	43.10	86.0	even	ok
	278	C58H7N52NaO10PS	559.0326	-1.1	44.1	287	34.68	83.0	even	ok
	279	C69H35N16O33P2	559.0337	0.9	44.1	288	43.81	63.0	even	ok
	280	C85H38N2O35P	559.0325	-1.2	44.2	289	40.83	70.0	even	ok
	281	C69H152N27O12P2S2	559.0338	1.1	44.2	290	41.70	10.0	even	ok
	282	C78H48NaO36P2S	559.0334	0.4	44.3	291	49.75	57.0	even	ok
	283	C64H23N36O16P2S2	559.0331	-0.2	44.4	292	52.42	74.0	even	ok
	284	C67H24N34NaO14P2S2	559.0336	0.8	44.4	293	44.91	75.0	even	ok
	285	C69H33N16O35S	559.0333	0.2	44.4	294	52.53	63.0	even	ok
	286	C69H157N17NaO23P2	559.0332	-0.1	44.6	295	44.56	2.0	even	ok

Compound Spectrum SmartFormula Report

Meas. m/z	#	Ion Formula	m/z	err [ppm]	mSigma	# mSigma	Score	rdb	e¯ Conf	N-Rule
	287	C76H4N46NaO2P2	559.0327	-0.8	44.6	296	36.81	100.0	even	ok
	288	C75H47N2O38P2S	559.0329	-0.6	44.7	297	47.30	56.0	even	ok
	289	C73H8N46NaO2P2S	559.0339	1.2	44.7	298	40.16	95.0	even	ok
	290	C56H136N49NaO4PS2	559.0335	0.6	44.7	299	47.38	15.0	even	ok
	291	C79H163N5O27PS	559.0326	-1.1	44.7	300	41.01	3.0	even	ok
	292	C68H156N23O16P2S2	559.0334	0.3	44.7	301	50.53	5.0	even	ok
	293	C85H157N9NaO18P2	559.0334	0.4	44.8	302	48.75	14.0	even	ok
	294	C75H139N33O7PS	559.0326	-1.1	44.8	303	40.69	25.0	even	ok
	295	C54H131N55O2PS2	559.0334	0.4	44.9	304	49.08	19.0	even	ok
	296	C64H152N25NaO20PS	559.0328	-0.6	44.9	305	46.09	3.0	even	ok
	297	C60H14N44NaO15S2	559.0336	0.7	44.9	306	45.12	77.0	even	ok
	298	C66H157N23NaO16P2S2	559.0326	-1.1	44.9	307	40.19	2.0	even	ok
	299	C76H26N22O23PS	559.0328	-0.8	45.0	308	44.57	77.0	even	ok
	300	C74H14N36O13PS	559.0328	-0.8	45.0	309	44.45	88.0	even	ok
	301	C68H139N39O5PS2	559.0328	-0.7	45.2	310	44.99	21.0	even	ok
	302	C61H7N50O11P2	559.0328	-0.7	45.2	311	37.57	86.0	even	ok
	303	C79H44N4NaO32P2S	559.0339	1.2	45.3	313	39.25	62.0	even	ok
	304	C71H38N10NaO37S	559.0334	0.3	45.3	314	49.17	59.0	even	ok
	305	C67H160N15NaO26PS	559.0333	0.2	45.4	315	50.99	-3.0	even	ok
	306	C62H147N31O18PS	559.0327	-0.8	45.5	316	43.36	7.0	even	ok
	307	C58H138N43O11S2	559.0329	-0.6	45.5	317	45.86	13.0	even	ok
	308	C65H155N21O24PS	559.0332	-0.0	45.7	319	52.50	1.0	even	ok
	309	C57H139N45O8PS2	559.0339	1.2	46.0	320	38.39	13.0	even	ok
	310	C60H143N37NaO13S2	559.0330	-0.4	46.0	321	47.11	9.0	even	ok
	311	C66H28N30NaO18P2S2	559.0332	-0.0	46.0	322	51.92	70.0	even	ok
	312	C66H18N38O15PS2	559.0325	-1.2	46.1	323	38.73	79.0	even	ok
	313	C79H156N13NaO19PS	559.0327	-0.9	46.1	324	41.08	10.0	even	ok
	314	C69H162N9O33S	559.0326	-1.0	46.2	325	40.54	-5.0	even	ok
	315	C67H152N23O21P2	559.0331	-0.2	46.2	326	40.90	6.0	even	ok
	316	C77H7N42O6P2	559.0331	-0.2	46.2	327	41.22	98.0	even	ok
	317	C80H15N32O12P2	559.0335	0.6	46.3	328	44.68	92.0	even	ok
	318	C62H134N41O14	559.0339	1.3	46.6	329	30.57	18.0	even	ok
	319	C71H157N21NaO14P2S2	559.0339	1.3	46.6	330	37.13	6.0	even	ok
	320	C71H167N3NaO35S	559.0327	-0.8	46.6	331	41.84	-9.0	even	ok
	321	C83H26N16O25P	559.0325	-1.2	46.7	332	37.95	81.0	even	ok
	322	C70H161N17NaO18P2S2	559.0334	0.5	46.7	333	45.86	1.0	even	ok
	323	C68H163N11O30PS	559.0336	0.8	46.7	334	41.91	-5.0	even	ok
	324	C70H168N5NaO32PS	559.0337	1.0	46.9	335	39.91	-9.0	even	ok
	325	C63H153N27NaO17P2S	559.0338	1.1	47.2	336	37.64	3.0	even	ok
	326	C86H160N5O22P2	559.0338	1.1	47.4	337	38.21	12.0	even	ok
	327	C57H8N54NaO7P2S	559.0336	0.7	47.4	338	35.30	83.0	even	ok
	328	C77H144N27NaO9PS	559.0327	-0.9	47.5	339	39.43	21.0	even	ok
	329	C78H38N8O33PS	559.0328	-0.8	47.5	340	41.49	66.0	even	ok
	330	C66H149N27NaO17P2	559.0327	-0.9	47.5	341	33.53	8.0	even	ok
	331	C78H31N16NaO25PS	559.0329	-0.6	47.7	343	42.95	73.0	even	ok
	332	C76H19N30NaO15PS	559.0329	-0.6	47.7	344	42.83	84.0	even	ok

Compound Spectrum SmartFormula Report

Meas. m/z	#	Ion Formula	m/z	err [ppm]	mSigma	# mSigma	Score	rdb	e¯ Conf	N-Rule
	333	C70H144N33NaO7PS2	559.0329	-0.5	47.8	345	43.39	17.0	even	ok
	334	C67H14N42O11PS2	559.0330	-0.4	47.9	346	45.31	84.0	even	ok
	335	C67H160N19O20P2S2	559.0329	-0.5	47.9	347	43.60	0.0	even	ok
	336	C61H148N33O15P2S	559.0337	1.0	48.1	348	38.42	7.0	even	ok
	337	C72H41N6O41S	559.0337	1.0	48.1	349	38.54	57.0	even	ok
	338	C61H146N33O17S2	559.0333	0.2	48.3	350	46.29	7.0	even	ok
	339	C67H7N50NaO3PS2	559.0331	-0.2	48.3	351	46.42	91.0	even	ok
	340	C79H12N36NaO8P2	559.0332	-0.0	48.4	352	40.13	94.0	even	ok
	341	C68H23N32NaO17PS2	559.0326	-1.0	48.4	353	37.90	75.0	even	ok
	342	C68H32N20NaO29P2	559.0334	0.3	48.5	354	37.76	65.0	even	ok
	343	C61H131N45NaO10	559.0336	0.7	48.5	355	34.41	20.0	even	ok
	344	C82H20N26NaO14P2	559.0336	0.8	48.5	356	40.05	88.0	even	ok
	345	C70H151N25O15PS2	559.0328	-0.7	48.6	357	40.68	10.0	even	ok
	346	C54H5N56O11S2	559.0326	-1.0	48.8	358	36.87	82.0	even	ok
	347	C55H3N60O5P2S	559.0335	0.5	48.8	359	35.33	87.0	even	ok
	348	C52H3N62NaO4PS2	559.0332	0.1	48.9	360	46.72	84.0	even	ok
	349	C78H132N39P2	559.0329	-0.5	48.9	361	34.94	35.0	even	ok
	350	C56H10N50NaO13S2	559.0327	-0.9	49.0	362	38.36	78.0	even	ok
	351	C63H27N32O20P2S2	559.0327	-1.0	49.0	363	37.43	69.0	even	ok
	352	C63H151N27NaO19S2	559.0334	0.4	49.1	364	43.39	3.0	even	ok
	353	C71H169N3NaO33P2	559.0332	-0.1	49.2	365	46.75	-9.0	even	ok
	354	C85H31N10NaO27P	559.0326	-1.0	49.2	366	36.80	77.0	even	ok
	355	C74H46NaO43S	559.0338	1.1	49.3	367	35.46	53.0	even	ok
	356	C69H165N13NaO22P2S2	559.0330	-0.3	49.4	368	43.47	-4.0	even	ok
	357	C64H144N33O15P2	559.0326	-1.0	49.4	369	30.23	12.0	even	ok
	358	C67H31N26O22P2S2	559.0335	0.6	49.5	370	40.34	68.0	even	ok
	359	C60H19N38NaO20PS	559.0326	-1.1	49.7	371	35.39	72.0	even	ok
	360	C63H27N28NaO26PS	559.0330	-0.3	49.7	372	43.56	66.0	even	ok
	361	C67H34N16NaO35S	559.0325	-1.3	49.7	373	33.63	60.0	even	ok
	362	C55H11N52NaO10PS2	559.0337	0.9	49.8	375	37.21	78.0	even	ok
	363	C80H159N9O23PS	559.0330	-0.3	49.8	376	43.27	8.0	even	ok
	364	C53H6N58O8PS2	559.0336	0.7	49.9	377	38.78	82.0	even	ok
	365	C66H27N26O27P2	559.0333	0.1	49.9	378	37.64	69.0	even	ok
	366	C80H43N2NaO35PS	559.0329	-0.6	50.1	379	40.05	62.0	even	ok
	367	C61H22N34O24PS	559.0329	-0.5	50.1	380	41.25	70.0	even	ok
	368	C65H32N26NaO22P2S2	559.0327	-0.8	50.2	381	37.81	65.0	even	ok
	369	C74H7N44NaO5PS	559.0329	-0.6	50.2	382	39.68	95.0	even	ok
	370	C71H164N13O22P2S2	559.0338	1.1	50.3	383	34.76	-1.0	even	ok
	371	C69H164N9O31P2	559.0331	-0.2	50.4	384	43.38	-5.0	even	ok
	372	C80H137N33NaO2P2	559.0330	-0.4	50.4	385	34.82	31.0	even	ok
	373	C58H14N44O18PS	559.0325	-1.3	50.5	386	32.93	76.0	even	ok
	374	C69H19N36NaO13PS2	559.0331	-0.2	50.5	387	43.56	80.0	even	ok
	375	C66H35N18NaO32PS	559.0335	0.5	50.5	388	40.29	60.0	even	ok
	376	C69H135N43OPS2	559.0332	0.1	50.5	389	44.48	26.0	even	ok
	377	C64H30N24O30PS	559.0334	0.3	50.7	390	41.81	64.0	even	ok
	378	C59H126N51O8	559.0335	0.5	50.7	391	33.56	24.0	even	ok

Compound Spectrum SmartFormula Report

Meas. m/z	#	Ion Formula	m/z	err [ppm]	mSigma	# mSigma	Score	rdb	e^{-} Conf	N-Rule
	379	C65H24N30NaO23P2	559.0329	-0.5	50.8	392	33.24	71.0	even	ok
	380	C78H147N23O13PS	559.0330	-0.3	50.8	393	42.00	19.0	even	ok
	381	C57H13N46O17S2	559.0331	-0.2	50.9	394	42.53	76.0	even	ok
	382	C60H6N48NaO16	559.0338	1.0	51.0	395	29.09	83.0	even	ok
	383	C72H46O46P	559.0327	-0.9	51.0	396	36.29	52.0	even	ok
	384	C72H156N19NaO17PS2	559.0329	-0.5	51.1	397	39.33	6.0	even	ok
	385	C77H22N26O19PS	559.0332	0.0	51.3	398	44.03	82.0	even	ok
	386	C69H36N20NaO24P2S2	559.0336	0.8	51.3	399	36.51	64.0	even	ok
	387	C59H18N40NaO19S2	559.0332	-0.1	51.4	400	43.49	72.0	even	ok
	388	C81H14N30O15P	559.0325	-1.2	51.4	401	32.80	92.0	even	ok
	389	C68H37N12O39S	559.0328	-0.6	51.5	402	37.82	58.0	even	ok
	390	C79H34N12O29PS	559.0332	0.0	51.6	403	43.59	71.0	even	ok
	391	C64H154N23O23S2	559.0338	1.0	51.7	404	33.95	1.0	even	ok
	392	C71H147N29O11PS2	559.0332	0.1	51.8	405	42.78	15.0	even	ok
	393	C67H38N14O36PS	559.0338	1.1	52.0	406	32.53	58.0	even	ok
	394	C70H42N6NaO41S	559.0329	-0.5	52.0	407	38.85	54.0	even	ok
	395	C68H10N46O7PS2	559.0334	0.4	52.2	408	38.98	89.0	even	ok
	396	C69H43N8NaO38PS	559.0339	1.3	52.2	409	30.81	54.0	even	ok
	397	C69H26N28O21PS2	559.0330	-0.4	52.3	410	39.66	73.0	even	ok
	398	C59H20N40NaO17P2S	559.0336	0.7	52.3	411	36.46	72.0	even	ok
	399	C86H34N6O31P	559.0330	-0.4	52.3	412	39.44	75.0	even	ok
	400	C73H169N7NaO24P2S2	559.0339	1.3	52.4	413	31.07	-5.0	even	ok
	401	C63H19N36O21P2	559.0328	-0.7	52.5	414	30.22	75.0	even	ok
	402	C78H3N46O2P2	559.0335	0.6	52.5	415	30.86	103.0	even	ok
	403	C68H30N24O25PS2	559.0326	-1.1	52.6	416	31.90	68.0	even	ok
	404	C70H168N9O26P2S2	559.0334	0.3	52.6	417	39.80	-6.0	even	ok
	405	C82H164N3NaO25PS	559.0331	-0.1	52.6	418	41.27	4.0	even	ok
	406	C60H145N37NaO11P2S	559.0334	0.3	52.7	419	32.75	9.0	even	ok
	407	C84H148N19O12P2	559.0338	1.1	52.7	420	32.50	23.0	even	ok
	408	C66H159N17NaO25S2	559.0339	1.2	52.8	421	31.29	-3.0	even	ok
	409	C87H159N3O25P	559.0328	-0.7	52.9	422	35.50	12.0	even	ok
	410	C81H140N29O6P2	559.0333	0.3	53.0	424	33.02	29.0	even	ok
	411	C58H123N55NaO4	559.0331	-0.1	53.0	425	33.89	26.0	even	ok
	412	C66H35N22O26P2S2	559.0331	-0.2	53.1	426	40.40	63.0	even	ok
	413	C66H164N15O24P2S2	559.0325	-1.3	53.2	427	29.86	-5.0	even	ok
	414	C70H44N6NaO39P2	559.0334	0.3	53.2	428	39.09	54.0	even	ok
	415	C75H10N40O9PS	559.0332	0.0	53.3	429	41.41	93.0	even	ok
	416	C71H47N2O43P2	559.0337	0.9	53.3	430	33.15	52.0	even	ok
	417	C71H140N37NaO3PS2	559.0333	0.3	53.3	431	39.23	22.0	even	ok
	418	C57H15N46O15P2S	559.0335	0.5	53.3	432	36.84	76.0	even	ok
	419	C80H152N17NaO15PS	559.0331	-0.1	53.5	433	40.09	15.0	even	ok
	420	C60H21N36O23S2	559.0335	0.6	53.7	434	36.04	70.0	even	ok
	421	C52H132N55NaO2PS2	559.0326	-1.0	53.8	435	31.63	16.0	even	ok
	422	C83H19N24NaO17P	559.0326	-1.0	53.8	436	31.91	88.0	even	ok
	423	C59H147N33NaO17S2	559.0325	-1.2	53.9	437	30.03	4.0	even	ok
	424	C55H140N45NaO8PS2	559.0331	-0.2	53.9	438	38.74	10.0	even	ok

Compound Spectrum SmartFormula Report

Meas. m/z	#	Ion Formula	m/z	err [ppm]	mSigma	# mSigma	Score	rdb	e⁻ Conf	N-Rule
	425	C76H135N37O3PS	559.0330	-0.3	53.9	439	37.95	30.0	even	ok
	426	C58H140N43O9P2S	559.0333	0.2	54.0	440	32.70	13.0	even	ok
	427	C72H163N11O25PS2	559.0328	-0.7	54.0	441	34.48	-1.0	even	ok
	428	C79H27N20NaO21PS	559.0333	0.2	54.1	442	38.83	78.0	even	ok
	429	C68H169N9NaO26P2S2	559.0326	-1.1	54.2	443	30.35	-9.0	even	ok
	430	C80H8N40NaO4P2	559.0336	0.8	54.2	444	28.00	99.0	even	ok
	431	C71H45N2O45S	559.0333	0.2	54.2	445	38.95	52.0	even	ok
	432	C68H39N12O37P2	559.0333	0.1	54.3	446	39.34	58.0	even	ok
	433	C72H173N3NaO28P2S2	559.0335	0.5	54.3	447	36.26	-10.0	even	ok
	434	C53H135N51O6PS2	559.0330	-0.4	54.3	448	36.71	14.0	even	ok
	435	C81H39N6NaO31PS	559.0333	0.2	54.3	449	38.45	67.0	even	ok
	436	C64H146N27O24	559.0339	1.3	54.4	450	24.05	7.0	even	ok
	437	C70H22N32O17PS2	559.0334	0.4	54.4	451	36.21	78.0	even	ok
	438	C73H152N23NaO13PS2	559.0333	0.3	54.5	452	37.77	11.0	even	ok
	439	C62H26N30NaO25S2	559.0336	0.7	54.5	453	33.64	66.0	even	ok
	440	C68H40N16NaO28P2S2	559.0332	0.0	54.5	454	40.09	59.0	even	ok
	441	C50H127N61PS2	559.0325	-1.2	54.5	455	29.47	20.0	even	ok
	442	C70H35N18NaO27PS2	559.0326	-1.0	54.6	456	31.33	64.0	even	ok
	443	C67H36N16NaO33P2	559.0329	-0.5	54.6	457	35.39	60.0	even	ok
	444	C57H137N47NaO5P2S	559.0329	-0.5	54.7	458	29.82	15.0	even	ok
	445	C71H31N22NaO23PS2	559.0331	-0.2	54.7	459	38.24	69.0	even	ok
	446	C66H164N11NaO30PS	559.0328	-0.6	54.7	460	34.23	-8.0	even	ok
	447	C58H148N35NaO14PS2	559.0335	0.6	54.7	461	34.81	4.0	even	ok
	448	C86H153N13NaO14P2	559.0339	1.2	54.8	462	29.06	19.0	even	ok
	449	C83H145N23NaO8P2	559.0334	0.4	54.8	463	29.97	25.0	even	ok
	450	C56H143N41O12PS2	559.0334	0.4	54.9	464	36.13	8.0	even	ok
	451	C70H15N40NaO9PS2	559.0335	0.6	54.9	465	34.24	85.0	even	ok
	452	C88H39NaO33P	559.0331	-0.2	55.1	466	37.66	71.0	even	ok
	453	C73H159N15O21PS2	559.0333	0.1	55.1	467	38.45	4.0	even	ok
	454	C82H136N33O2P2	559.0338	1.1	55.2	468	25.13	34.0	even	ok
	455	C64H159N17O28PS	559.0327	-0.8	55.2	469	32.29	-4.0	even	ok
	456	C56H118N61O2	559.0330	-0.3	55.4	470	30.21	30.0	even	ok
	457	C69H172NNaO36PS	559.0333	0.2	55.5	471	37.36	-14.0	even	ok
	458	C63H143N31NaO20	559.0336	0.7	55.6	472	27.55	9.0	even	ok
	459	C82H10N34O11P	559.0330	-0.4	55.6	473	35.45	97.0	even	ok
	460	C60H150N29O21S2	559.0329	-0.6	55.6	474	33.69	2.0	even	ok
	461	C67H167N7O34PS	559.0332	0.0	55.7	475	38.61	-10.0	even	ok
	462	C68H161N13NaO27P2	559.0327	-0.9	55.7	476	26.04	-3.0	even	ok
	463	C77H15N34NaO11PS	559.0333	0.2	56.0	478	36.57	89.0	even	ok
	464	C62H155N23NaO23S2	559.0330	-0.4	56.1	479	34.62	-2.0	even	ok
	465	C59H151N31O18PS2	559.0339	1.2	56.2	480	28.02	2.0	even	ok
	466	C55H132N53O3P2S	559.0328	-0.6	56.2	481	27.17	19.0	even	ok
	467	C74H168N5NaO27PS2	559.0329	-0.5	56.3	482	33.46	-5.0	even	ok
	468	C85H147N17O15P	559.0328	-0.7	56.3	483	31.79	23.0	even	ok
	469	C84H22N20O21P	559.0330	-0.4	56.3	484	34.67	86.0	even	ok
	470	C78H140N31NaO5PS	559.0331	-0.1	56.5	485	36.31	26.0	even	ok

Compound Spectrum SmartFormula Report

Meas. m/z	#	Ion Formula	m/z	err [ppm]	mSigma	# mSigma	Score	rdb	e¯ Conf	N-Rule
	471	C65H165N13NaO27P2S	559.0338	1.1	56.7	486	27.92	-8.0	even	ok
	472	C81H155N13O19PS	559.0335	0.5	57.0	487	32.98	13.0	even	ok
	473	C84H141N27NaO4P2	559.0339	1.2	57.0	488	22.60	30.0	even	ok
	474	C69H172N5O30P2S2	559.0329	-0.5	57.0	489	32.84	-11.0	even	ok
	475	C72H27N26NaO19PS2	559.0335	0.6	57.1	490	31.87	74.0	even	ok
	476	C72H143N33O7PS2	559.0337	0.9	57.1	491	29.58	20.0	even	ok
	477	C62H157N23NaO21P2S	559.0334	0.3	57.2	492	33.89	-2.0	even	ok
	478	C66H156N19O25P2	559.0326	-1.0	57.3	493	23.63	1.0	even	ok
	479	C63H160N19O25P2S	559.0337	1.0	57.4	494	28.60	-4.0	even	ok
	480	C61H138N37O18	559.0335	0.5	57.4	495	27.04	13.0	even	ok
	481	C84H15N28NaO13P	559.0331	-0.2	57.5	496	34.64	93.0	even	ok
	482	C75H164N9NaO23PS2	559.0333	0.3	57.6	497	34.03	0.0	even	ok
	483	C69H43N12O32P2S2	559.0336	0.6	57.7	498	31.04	57.0	even	ok
	484	C80H30N16O25PS	559.0337	0.8	57.7	499	29.41	76.0	even	ok
	485	C81H7N38NaO7P	559.0326	-1.0	57.8	500	23.34	99.0	even	ok
	486	C66H158N13O34	559.0339	1.3	57.9	501	25.68	-4.0	even	ok
	487	C60H152N29O19P2S	559.0333	0.2	58.2	502	34.15	2.0	even	ok
	488	C63H158N19O27S2	559.0333	0.2	58.2	503	33.78	-4.0	even	ok
	489	C82H42N2O35PS	559.0337	0.8	58.2	504	28.87	65.0	even	ok
	490	C86H143N21O11P	559.0332	0.1	58.3	505	34.84	28.0	even	ok
	491	C87H152N11NaO17P	559.0329	-0.6	58.3	506	31.09	19.0	even	ok
	492	C71H38N14O31PS2	559.0330	-0.3	58.3	507	32.69	62.0	even	ok
	493	C83H6N38O7P	559.0334	0.4	58.4	508	32.03	102.0	even	ok
	494	C74H155N19O17PS2	559.0337	0.9	58.4	509	28.26	9.0	even	ok
	495	C59H149N33NaO15P2S	559.0329	-0.5	58.4	510	31.68	4.0	even	ok
	496	C51H7N58NaO8PS2	559.0328	-0.7	58.5	511	29.82	79.0	even	ok
	497	C69H6N50O3PS2	559.0339	1.2	58.5	512	25.69	94.0	even	ok
	498	C72H34N18O27PS2	559.0334	0.5	58.6	513	31.55	67.0	even	ok
	499	C65H39N18O30P2S2	559.0327	-1.0	58.6	514	27.63	58.0	even	ok
	500	C56H12N50NaO11P2S	559.0331	-0.1	58.6	515	28.43	78.0	even	ok
573.7090	1	C67H152N29NaO17PS	573.7087	-0.6	21.4	1	85.92	8.0	even	ok
	2	C68H155N25O21PS	573.7090	-0.0	21.4	2	100.00	6.0	even	ok
	3	C76H171NO37P	573.7084	-1.2	21.6	3	73.46	-6.0	even	ok
	4	C66H27N32NaO23PS	573.7089	-0.3	21.6	4	93.18	71.0	even	ok
	5	C55H3N66NaOPS2	573.7091	0.1	21.7	5	97.23	89.0	even	ok
	6	C64H22N38O21PS	573.7088	-0.4	21.7	6	89.27	75.0	even	ok
	7	C65H147N35O15PS	573.7086	-0.8	21.7	7	81.68	12.0	even	ok
	8	C75H46N4O43P	573.7086	-0.8	21.7	8	80.56	57.0	even	ok
	9	C76H42N8O39P	573.7090	-0.1	21.8	9	97.93	62.0	even	ok
	10	C70H160N19NaO23PS	573.7091	0.2	21.9	10	95.39	2.0	even	ok
	11	C75H39N12NaO35P	573.7087	-0.7	22.0	11	83.54	64.0	even	ok
	12	C63H19N42NaO17PS	573.7084	-1.1	22.2	12	74.64	77.0	even	ok
	13	C67H30N28O27PS	573.7092	0.3	22.3	13	90.71	69.0	even	ok
	14	C65H18N42O17PS	573.7092	0.3	22.3	14	90.84	80.0	even	ok
	15	C59H136N53NaOPS2	573.7094	0.5	22.3	15	85.96	20.0	even	ok
	16	C73H34N18O33P	573.7086	-0.8	22.3	16	79.45	68.0	even	ok

Compound Spectrum SmartFormula Report

Meas. m/z	#	Ion Formula	m/z	err [ppm]	mSigma	# mSigma	Score	rdb	e⁻ Conf	N-Rule
	17	C66H153N31NaO14P2S	573.7097	1.1	22.3	17	73.54	8.0	even	ok
	18	C78H47N2NaO41P	573.7091	0.1	22.3	18	95.76	58.0	even	ok
	19	C56H6N62O5PS2	573.7095	0.7	22.4	19	81.89	87.0	even	ok
	20	C64H15N46NaO13PS	573.7089	-0.3	22.4	20	91.30	82.0	even	ok
	21	C76H52NaO42P2	573.7097	1.1	22.5	21	74.27	53.0	even	ok
	22	C74H40N14NaO32P2	573.7097	1.1	22.6	22	74.18	64.0	even	ok
	23	C62H10N52O11PS	573.7088	-0.5	22.6	23	87.28	86.0	even	ok
	24	C67H23N36NaO19PS	573.7093	0.5	22.8	24	86.09	76.0	even	ok
	25	C58H140N49NaO5PS2	573.7089	-0.2	22.9	25	91.65	15.0	even	ok
	26	C69H35N22NaO29PS	573.7093	0.5	22.9	26	85.80	65.0	even	ok
	27	C69H164N15NaO27PS	573.7087	-0.6	23.0	27	82.93	-3.0	even	ok
	28	C56H135N55O3PS2	573.7088	-0.4	23.1	28	87.79	19.0	even	ok
	29	C71H163N15O27PS	573.7095	0.8	23.1	29	79.30	0.0	even	ok
	30	C58H11N56NaO7PS2	573.7095	0.9	23.1	30	77.07	83.0	even	ok
	31	C64H148N37O12P2S	573.7096	1.0	23.2	31	75.41	12.0	even	ok
	32	C67H159N21O25PS	573.7086	-0.8	23.3	32	79.04	1.0	even	ok
	33	C75H172N3O34P2	573.7094	0.6	23.3	33	83.62	-6.0	even	ok
	34	C74H47N6O40P2	573.7096	0.9	23.3	34	76.36	57.0	even	ok
	35	C77H167N5O33P	573.7088	-0.4	23.5	35	86.93	-1.0	even	ok
	36	C60H139N49O5PS2	573.7097	1.2	23.5	36	70.48	18.0	even	ok
	37	C72H35N20O30P2	573.7096	0.9	23.5	37	76.10	68.0	even	ok
	38	C59H143N45O9PS2	573.7093	0.4	23.8	38	86.47	13.0	even	ok
	39	C68H26N32O23PS	573.7097	1.1	23.8	39	71.09	74.0	even	ok
	40	C70H167N11O31PS	573.7090	0.0	23.9	40	94.65	-5.0	even	ok
	41	C61H7N56NaO7PS	573.7084	-1.1	23.9	41	71.66	88.0	even	ok
	42	C57H5N60O8S2	573.7085	-1.0	24.0	42	72.81	87.0	even	ok
	43	C66H143N39O11PS	573.7090	-0.0	24.1	43	94.06	17.0	even	ok
	44	C68H165N17NaO24P2S	573.7097	1.1	24.2	44	70.35	-3.0	even	ok
	45	C76H164N9NaO29P	573.7085	-1.0	24.3	45	72.32	1.0	even	ok
	46	C73H168N9NaO29PS	573.7096	0.9	24.3	46	73.79	-4.0	even	ok
	47	C62H20N44NaO14P2S	573.7094	0.7	24.3	47	79.49	77.0	even	ok
	48	C61H148N39NaO11PS2	573.7094	0.6	24.4	48	81.87	9.0	even	ok
	49	C72H172N5NaO33PS	573.7091	0.2	24.4	49	90.06	-9.0	even	ok
	50	C68H148N33NaO13PS	573.7091	0.2	24.5	50	90.22	13.0	even	ok
	51	C73H44N10NaO36P2	573.7092	0.3	24.6	51	87.24	59.0	even	ok
	52	C70H38N18O33PS	573.7097	1.1	24.7	52	69.64	63.0	even	ok
	53	C74H159N15O27P	573.7084	-1.2	24.7	53	68.30	5.0	even	ok
	54	C69H151N29O17PS	573.7095	0.8	24.8	54	76.45	11.0	even	ok
	55	C65H140N43NaO7PS	573.7087	-0.6	24.8	55	79.38	19.0	even	ok
	56	C66H160N23O22P2S	573.7096	1.0	25.0	56	72.39	1.0	even	ok
	57	C75H165N11NaO26P2	573.7095	0.7	25.0	57	77.21	1.0	even	ok
	58	C54H7N62NaO5PS2	573.7087	-0.7	25.1	58	78.02	84.0	even	ok
	59	C63H145N41NaO8P2S	573.7092	0.3	25.1	59	85.21	14.0	even	ok
	60	C74H169N7NaO30P2	573.7090	-0.1	25.2	60	90.86	-4.0	even	ok
	61	C63H135N49O5PS	573.7086	-0.8	25.2	61	75.35	23.0	even	ok
	62	C59H10N54NaO10S2	573.7086	-0.9	25.3	62	73.98	83.0	even	ok

Compound Spectrum SmartFormula Report

Meas. m/z	#	Ion Formula	m/z	err [ppm]	mSigma	# mSigma	Score	rdb	e⁻ Conf	N-Rule
	63	C55H10N58O9PS2	573.7090	-0.1	25.4	63	90.33	82.0	even	ok
	64	C65H31N28NaO27PS	573.7084	-1.1	25.4	64	69.54	66.0	even	ok
	65	C57H15N52NaO11PS2	573.7091	0.1	25.4	65	89.19	78.0	even	ok
	66	C71H32N24NaO26P2	573.7092	0.3	25.6	66	85.40	70.0	even	ok
	67	C68H39N18NaO33PS	573.7089	-0.3	25.7	68	85.04	60.0	even	ok
	68	C60H15N50O12P2S	573.7093	0.5	25.7	69	80.37	81.0	even	ok
	69	C66H34N24O31PS	573.7088	-0.4	25.7	70	81.65	64.0	even	ok
	70	C72H162N13O30S	573.7085	-1.0	25.7	71	71.13	0.0	even	ok
	71	C71H156N23NaO19PS	573.7096	0.9	25.9	72	71.35	7.0	even	ok
	72	C65H157N27NaO18P2S	573.7092	0.3	26.0	73	83.38	3.0	even	ok
	73	C71H39N16O34P2	573.7091	0.1	26.0	74	87.80	63.0	even	ok
	74	C73H160N17O24P2	573.7094	0.6	26.0	75	78.73	5.0	even	ok
	75	C73H175NO37PS	573.7095	0.8	26.2	76	73.78	-11.0	even	ok
	76	C62H151N35O15PS2	573.7097	1.2	26.2	77	66.13	7.0	even	ok
	77	C61H138N47O8S2	573.7087	-0.6	26.3	78	78.01	18.0	even	ok
	78	C74H30N22O29P	573.7090	-0.1	26.3	79	88.25	73.0	even	ok
	79	C76H35N16NaO31P	573.7091	0.1	26.7	80	86.82	69.0	even	ok
	80	C72H164N13O28P2	573.7089	-0.2	26.7	81	84.32	0.0	even	ok
	81	C58H18N48O15PS2	573.7095	0.7	26.8	82	74.02	76.0	even	ok
	82	C77H38N12O35P	573.7095	0.7	26.8	83	74.08	67.0	even	ok
	83	C69H25N30O26S	573.7087	-0.6	26.8	84	75.90	74.0	even	ok
	84	C69H42N14O37PS	573.7092	0.3	27.0	85	81.34	58.0	even	ok
	85	C63H6N56O7PS	573.7092	0.3	27.1	86	81.55	91.0	even	ok
	86	C53H8N64NaO2P2S2	573.7097	1.1	27.1	87	66.98	84.0	even	ok
	87	C73H27N26NaO25P	573.7087	-0.7	27.2	88	74.06	75.0	even	ok
	88	C71H37N16O36S	573.7087	-0.6	27.3	89	75.25	63.0	even	ok
	89	C63H152N33O16P2S	573.7091	0.2	27.3	90	84.01	7.0	even	ok
	90	C74H167N7NaO32S	573.7086	-0.8	27.4	91	71.58	-4.0	even	ok
	91	C60H23N42NaO17PS2	573.7096	0.9	27.5	92	69.68	72.0	even	ok
	92	C72H28N28NaO22P2	573.7096	1.1	27.5	93	66.51	75.0	even	ok
	93	C65H11N50NaO9PS	573.7093	0.5	27.5	94	77.54	87.0	even	ok
	94	C64H141N45NaO4P2S	573.7097	1.1	27.5	95	54.49	19.0	even	ok
	95	C71H22N32O23P	573.7086	-0.8	27.5	97	70.35	79.0	even	ok
	96	C64H32N30NaO24P2S	573.7094	0.7	27.6	98	73.69	66.0	even	ok
	97	C70H150N27O20S	573.7085	-1.0	27.6	99	68.03	11.0	even	ok
	98	C71H47N8NaO39PS	573.7093	0.5	27.6	100	76.95	54.0	even	ok
	99	C60H13N50O14S2	573.7089	-0.2	27.6	101	82.25	81.0	even	ok
	100	C66H14N46O13PS	573.7097	1.1	27.6	102	65.24	85.0	even	ok
	101	C59H17N46O18S2	573.7085	-1.0	27.8	103	66.73	76.0	even	ok
	102	C79H43N6NaO37P	573.7095	0.9	27.8	104	69.18	63.0	even	ok
	103	C60H8N58NaO4P2S	573.7094	0.7	27.9	105	61.17	88.0	even	ok
	104	C62H3N60NaO3PS	573.7089	-0.3	27.9	106	80.42	93.0	even	ok
	105	C63H143N41NaO10S2	573.7088	-0.4	28.0	107	78.15	14.0	even	ok
	106	C70H36N20NaO30P2	573.7088	-0.5	28.1	108	76.13	65.0	even	ok
	107	C57H144N45NaO9PS2	573.7085	-1.0	28.1	109	66.32	10.0	even	ok
	108	C51H3N70P2S2	573.7096	0.9	28.2	110	68.33	88.0	even	ok

Compound Spectrum SmartFormula Report

Meas. m/z	#	Ion Formula	m/z	err [ppm]	mSigma	# mSigma	Score	rdb	e⁻ Conf	N-Rule
	109	C63H150N33O18S2	573.7087	-0.6	28.4	111	74.26	7.0	even	ok
	110	C62H136N51O2P2S	573.7096	0.9	28.5	112	55.73	23.0	even	ok
	111	C71H30N24NaO28S	573.7088	-0.5	28.5	113	76.04	70.0	even	ok
	112	C60H152N35NaO15PS2	573.7089	-0.2	28.5	114	80.63	4.0	even	ok
	113	C58H147N41O13PS2	573.7088	-0.4	28.6	115	77.41	8.0	even	ok
	114	C62H27N36O22P2S	573.7093	0.5	28.7	116	74.84	70.0	even	ok
	115	C55H139N51O7PS2	573.7084	-1.2	28.8	117	62.32	14.0	even	ok
	116	C61H140N47O6P2S	573.7091	0.2	28.9	118	67.62	18.0	even	ok
	117	C71H176NNaO37PS	573.7087	-0.6	29.0	119	72.47	-14.0	even	ok
	118	C73H42N10NaO38S	573.7088	-0.5	29.0	120	75.32	59.0	even	ok
	119	C61H22N40NaO20S2	573.7086	-0.8	29.0	121	67.90	72.0	even	ok
	120	C69H171N7O35PS	573.7086	-0.8	29.0	122	69.24	-10.0	even	ok
	121	C72H155N21NaO22S	573.7086	-0.8	29.1	123	68.65	7.0	even	ok
	122	C62H149N37NaO12P2S	573.7088	-0.4	29.1	124	75.31	9.0	even	ok
	123	C73H51N2O44P2	573.7091	0.1	29.2	125	81.23	52.0	even	ok
	124	C60H6N58NaO6S2	573.7090	-0.1	29.2	126	82.12	88.0	even	ok
	125	C69H27N30O24P2	573.7091	0.1	29.4	127	67.68	74.0	even	ok
	126	C58H3N64O2P2S	573.7093	0.5	29.4	128	61.54	92.0	even	ok
	127	C62H18N44NaO16S2	573.7090	-0.1	29.4	129	81.96	77.0	even	ok
	128	C73H173N3NaO34P2	573.7086	-0.8	29.6	130	67.31	-9.0	even	ok
	129	C78H37N10O38	573.7085	-1.0	29.6	131	63.76	67.0	even	ok
	130	C72H50N4O43PS	573.7097	1.1	29.7	132	61.79	52.0	even	ok
	131	C78H163N9O29P	573.7093	0.4	29.7	133	75.46	4.0	even	ok
	132	C61H155N31O19PS2	573.7093	0.4	29.9	134	74.90	2.0	even	ok
	133	C68H31N26O28P2	573.7087	-0.7	29.9	135	69.85	69.0	even	ok
	134	C61H24N40NaO18P2S	573.7090	-0.1	29.9	136	80.21	72.0	even	ok
	135	C75H155N19O23P	573.7088	-0.4	29.9	137	74.70	10.0	even	ok
	136	C61H131N55NaS2	573.7088	-0.4	29.9	138	74.44	25.0	even	ok
	137	C75H170N3O36S	573.7089	-0.2	30.0	139	78.83	-6.0	even	ok
	138	C65H155N27NaO20S2	573.7088	-0.4	30.0	140	74.45	3.0	even	ok
	139	C77H160N13NaO25P	573.7089	-0.2	30.1	141	77.43	6.0	even	ok
	140	C70H177N3NaO34P2S	573.7097	1.1	30.1	142	61.14	-14.0	even	ok
	141	C59H12N54NaO8P2S	573.7090	-0.1	30.2	143	66.30	83.0	even	ok
	142	C56H145N47NaO6P2S2	573.7095	0.7	30.3	144	68.05	10.0	even	ok
	143	C63H160N25NaO21PS2	573.7094	0.6	30.4	145	70.94	-2.0	even	ok
	144	C72H48N6NaO40P2	573.7088	-0.5	30.5	146	72.01	54.0	even	ok
	145	C80H168N3NaO31P	573.7094	0.5	30.5	147	71.05	0.0	even	ok
	146	C64H146N37O14S2	573.7092	0.2	30.5	148	77.11	12.0	even	ok
	147	C67H13N44O16S	573.7087	-0.6	30.5	149	69.35	85.0	even	ok
	148	C67H139N43O7PS	573.7095	0.8	30.6	150	66.81	22.0	even	ok
	149	C68H172N9O32P2S	573.7096	1.0	30.7	151	63.18	-10.0	even	ok
	150	C70H23N34O20P2	573.7096	0.9	30.7	152	53.62	79.0	even	ok
	151	C72H157N21NaO20P2	573.7090	-0.1	30.8	153	66.11	7.0	even	ok
	152	C67H169N13NaO28P2S	573.7092	0.3	30.8	154	74.10	-8.0	even	ok
	153	C72H33N20O32S	573.7091	0.2	30.9	155	77.43	68.0	even	ok
	154	C60H144N43O10P2S	573.7087	-0.6	30.9	156	69.17	13.0	even	ok

Compound Spectrum SmartFormula Report

Meas. m/z	#	Ion Formula	m/z	err [ppm]	mSigma	# mSigma	Score	rdb	e⁻ Conf	N-Rule
	155	C66H136N47NaO3PS	573.7091	0.1	30.9	157	77.55	24.0	even	ok
	156	C73H158N17O26S	573.7089	-0.2	31.0	158	76.87	5.0	even	ok
	157	C80H42N4NaO40	573.7086	-0.9	31.1	159	64.41	63.0	even	ok
	158	C54H140N53O4P2S2	573.7094	0.6	31.3	160	69.22	14.0	even	ok
	159	C71H168N9O32P2	573.7085	-1.0	31.4	161	61.57	-5.0	even	ok
	160	C69H144N37NaO9PS	573.7096	0.9	31.4	162	62.63	18.0	even	ok
	161	C64H159N23NaO24S2	573.7084	-1.2	31.5	163	58.19	-2.0	even	ok
	162	C59H19N46O16P2S	573.7089	-0.3	31.5	164	74.23	76.0	even	ok
	163	C56H19N48NaO15PS2	573.7087	-0.7	31.5	165	66.96	73.0	even	ok
	164	C60H137N51NaO2P2S	573.7088	-0.5	31.6	166	59.00	20.0	even	ok
	165	C61H9N54O10S2	573.7094	0.5	31.6	167	69.18	86.0	even	ok
	166	C73H49N2O46S	573.7087	-0.6	31.6	168	67.86	52.0	even	ok
	167	C62H25N36O24S2	573.7089	-0.2	31.6	169	74.74	70.0	even	ok
	168	C62H134N51O4S2	573.7092	0.2	31.8	170	74.88	23.0	even	ok
	169	C71H161N17NaO24P2	573.7086	-0.8	31.8	171	53.02	2.0	even	ok
	170	C74H45N6O42S	573.7091	0.2	31.9	172	75.46	57.0	even	ok
	171	C65H164N19O26P2S	573.7091	0.2	31.9	173	75.06	-4.0	even	ok
	172	C54H14N54O13PS2	573.7086	-0.8	32.0	174	63.40	77.0	even	ok
	173	C69H18N38NaO18S	573.7088	-0.5	32.0	175	69.77	81.0	even	ok
	174	C70H43N12O38P2	573.7087	-0.7	32.1	176	66.36	58.0	even	ok
	175	C67H43N14NaO37PS	573.7084	-1.1	32.1	177	59.36	55.0	even	ok
	176	C67H30N24NaO33	573.7096	1.0	32.1	178	60.47	66.0	even	ok
	177	C57H7N60O6P2S	573.7089	-0.3	32.1	179	60.78	87.0	even	ok
	178	C53H137N57NaP2S2	573.7090	-0.1	32.3	180	76.68	16.0	even	ok
	179	C68H24N34NaO20P2	573.7088	-0.5	32.3	181	57.21	76.0	even	ok
	180	C63H21N40O20S2	573.7094	0.5	32.3	182	67.91	75.0	even	ok
	181	C57H22N44O19PS2	573.7090	-0.1	32.4	183	76.48	71.0	even	ok
	182	C70H152N27O18P2	573.7089	-0.2	32.4	184	61.12	11.0	even	ok
	183	C64H163N21O25PS2	573.7097	1.2	32.5	185	56.80	-4.0	even	ok
	184	C59H27N38NaO21PS2	573.7091	0.1	32.5	186	75.19	67.0	even	ok
	185	C66H151N31NaO16S2	573.7093	0.4	32.6	187	70.46	8.0	even	ok
	186	C81H45O44	573.7089	-0.2	32.7	188	72.65	61.0	even	ok
	187	C64H161N23NaO22P2S	573.7088	-0.4	32.9	189	68.77	-2.0	even	ok
	188	C75H163N11NaO28S	573.7090	-0.0	32.9	190	76.12	1.0	even	ok
	189	C68H46N10O41PS	573.7088	-0.4	32.9	191	68.76	53.0	even	ok
	190	C74H38N14NaO34S	573.7092	0.3	33.0	192	70.73	64.0	even	ok
	191	C70H51N4NaO43PS	573.7089	-0.3	33.0	193	71.49	49.0	even	ok
	192	C66H158N23O24S2	573.7092	0.2	33.0	194	72.44	1.0	even	ok
	193	C68H138N41O10S	573.7085	-1.0	33.1	195	59.31	22.0	even	ok
	194	C64H30N30NaO26S2	573.7090	-0.1	33.2	196	74.64	66.0	even	ok
	195	C69H20N38NaO16P2	573.7092	0.3	33.3	197	59.12	81.0	even	ok
	196	C55H20N50NaO12P2S2	573.7097	1.1	33.4	198	57.35	73.0	even	ok
	197	C58H16N50NaO12P2S	573.7085	-0.9	33.4	199	60.23	78.0	even	ok
	198	C64H131N53OPS	573.7090	-0.0	33.4	200	62.61	28.0	even	ok
	199	C70H21N34O22S	573.7091	0.2	33.6	201	72.49	79.0	even	ok
	200	C58H132N57P2S	573.7087	-0.6	33.6	202	53.77	24.0	even	ok

Compound Spectrum SmartFormula Report

Meas. m/z	#	Ion Formula	m/z	err [ppm]	mSigma	# mSigma	Score	rdb	e¯ Conf	N-Rule
	201	C63H14N48NaO12S2	573.7094	0.7	33.7	203	62.93	82.0	even	ok
	202	C64H139N45NaO6S2	573.7093	0.4	33.7	204	68.56	19.0	even	ok
	203	C75H26N26O25P	573.7095	0.7	33.7	205	62.71	78.0	even	ok
	204	C69H156N23O22P2	573.7085	-1.0	33.9	206	48.09	6.0	even	ok
	205	C76H50NaO44S	573.7092	0.3	33.9	207	68.93	53.0	even	ok
	206	C74H152N23NaO19P	573.7085	-1.0	33.9	208	47.70	12.0	even	ok
	207	C59H143N41NaO15S	573.7096	1.0	34.0	209	56.85	10.0	even	ok
	208	C66H44N16NaO34P2S	573.7094	0.7	34.0	210	62.79	55.0	even	ok
	209	C65H162N19O28S2	573.7087	-0.6	34.0	211	64.79	-4.0	even	ok
	210	C65H25N30O31	573.7095	0.8	34.0	212	60.31	70.0	even	ok
	211	C60H30N34O25PS2	573.7095	0.7	34.1	213	61.80	65.0	even	ok
	212	C65H18N38NaO23	573.7096	1.0	34.1	214	47.98	77.0	even	ok
	213	C74H23N30NaO21P	573.7091	0.1	34.2	215	72.39	80.0	even	ok
	214	C53H15N56O10P2S2	573.7096	0.9	34.2	216	58.81	77.0	even	ok
	215	C73H153N25NaO16P2	573.7095	0.7	34.2	217	51.52	12.0	even	ok
	216	C66H19N40O18P2	573.7087	-0.7	34.2	218	52.15	80.0	even	ok
	217	C65H26N34NaO22S2	573.7095	0.7	34.3	219	61.77	71.0	even	ok
	218	C70H143N35NaO12S	573.7086	-0.8	34.4	220	60.15	18.0	even	ok
	219	C62H156N29O20P2S	573.7087	-0.6	34.4	221	63.49	2.0	even	ok
	220	C72H147N29O17P	573.7084	-1.2	34.4	222	45.00	16.0	even	ok
	221	C77H31N20NaO27P	573.7095	0.9	34.5	223	58.83	74.0	even	ok
	222	C71H54O47PS	573.7092	0.3	34.5	224	67.54	47.0	even	ok
	223	C70H167N7NaO37	573.7094	0.7	34.6	225	62.25	-8.0	even	ok
	224	C57H131N55NaO5S	573.7096	1.0	34.6	226	46.69	21.0	even	ok
	225	C52H12N60NaO6P2S2	573.7092	0.3	34.7	228	68.33	79.0	even	ok
	226	C62H35N28NaO27PS2	573.7096	0.9	34.7	229	58.23	61.0	even	ok
	227	C61H29N32O28S2	573.7085	-1.0	34.7	230	56.42	65.0	even	ok
	228	C67H15N44O14P2	573.7091	0.1	34.7	231	59.26	85.0	even	ok
	229	C64H39N22O32P2S	573.7093	0.5	34.9	232	64.10	59.0	even	ok
	230	C68H163N17NaO26S2	573.7093	0.4	34.9	233	66.28	-3.0	even	ok
	231	C56H4N64NaO2P2S	573.7085	-0.9	35.0	234	48.10	89.0	even	ok
	232	C66H27N36O17P2S2	573.7085	-0.9	35.0	235	56.96	74.0	even	ok
	233	C68H155N21NaO27	573.7094	0.7	35.1	236	51.31	3.0	even	ok
	234	C71H148N31O14P2	573.7094	0.6	35.2	237	52.47	16.0	even	ok
	235	C63H36N26NaO28P2S	573.7090	-0.1	35.3	238	70.11	61.0	even	ok
	236	C76H166N7O32S	573.7094	0.6	35.3	239	61.93	-1.0	even	ok
	237	C67H167N13NaO30S2	573.7088	-0.4	35.4	240	65.14	-8.0	even	ok
	238	C71H146N31O16S	573.7089	-0.2	35.4	241	68.55	16.0	even	ok
	239	C72H26N28NaO24S	573.7092	0.3	35.5	242	66.43	75.0	even	ok
	240	C67H154N27O20S2	573.7096	1.0	35.6	243	55.33	6.0	even	ok
	241	C69H149N31NaO14P2	573.7086	-0.8	35.6	244	48.04	13.0	even	ok
	242	C59H156N31NaO19PS2	573.7085	-1.0	35.6	245	55.15	-1.0	even	ok
	243	C63H34N26NaO30S2	573.7086	-0.8	35.7	246	57.54	61.0	even	ok
	244	C57H138N47O13S	573.7095	0.9	35.8	247	56.79	14.0	even	ok
	245	C75H41N10O38S	573.7096	0.9	35.8	248	55.85	62.0	even	ok
	246	C50H7N66O4P2S2	573.7091	0.1	35.9	249	68.77	83.0	even	ok

Compound Spectrum SmartFormula Report

Meas. m/z	#	Ion Formula	m/z	err [ppm]	mSigma	# mSigma	Score	rdb	e⁻ Conf	N-Rule
	247	C57H151N37O17PS2	573.7084	-1.2	36.1	250	52.02	3.0	even	ok
	248	C65H142N41O10S2	573.7096	1.0	36.1	251	54.61	17.0	even	ok
	249	C69H42N10NaO43	573.7096	1.0	36.2	252	54.41	55.0	even	ok
	250	C76H25N24O28	573.7085	-1.0	36.2	253	53.90	78.0	even	ok
	251	C66H143N35NaO17	573.7094	0.6	36.3	254	49.79	14.0	even	ok
	252	C65H33N26O30S2	573.7094	0.6	36.3	255	61.18	64.0	even	ok
	253	C63H13N44O21	573.7095	0.8	36.4	256	47.40	81.0	even	ok
	254	C68H162N13O35	573.7093	0.5	36.4	258	61.94	-4.0	even	ok
	255	C64H15N50O7P2S2	573.7085	-0.9	36.5	259	54.75	85.0	even	ok
	256	C60H159N27O23PS2	573.7088	-0.4	36.5	260	63.59	-3.0	even	ok
	257	C62H164N21NaO25PS2	573.7089	-0.2	36.5	261	66.13	-7.0	even	ok
	258	C69H157N27NaO13P2S2	573.7084	-1.1	36.5	262	52.10	7.0	even	ok
	259	C61H31N32O26P2S	573.7089	-0.3	36.6	263	65.25	65.0	even	ok
	260	C71H169N13NaO23P2S2	573.7084	-1.1	36.7	264	51.96	-4.0	even	ok
	261	C55H126N61O3S	573.7095	0.9	36.8	265	46.16	25.0	even	ok
	262	C68H39N22O27P2S2	573.7085	-0.9	36.8	266	54.42	63.0	even	ok
	263	C72H18N36O19P	573.7090	-0.1	37.0	267	56.33	84.0	even	ok
	264	C49H4N70NaP2S2	573.7088	-0.5	37.0	268	60.85	85.0	even	ok
	265	C73H151N25NaO18S	573.7090	-0.0	37.1	269	68.18	12.0	even	ok
	266	C63H6N52NaO13	573.7096	1.0	37.3	271	44.32	88.0	even	ok
	267	C68H32N30NaO19P2S2	573.7086	-0.8	37.3	272	56.16	70.0	even	ok
	268	C66H150N27O25	573.7093	0.5	37.3	273	50.50	7.0	even	ok
	269	C78H30N18NaO30	573.7085	-0.9	37.4	274	54.73	74.0	even	ok
	270	C58H157N33NaO16P2S2	573.7095	0.7	37.5	275	56.51	-1.0	even	ok
	271	C78H171NNaO34S	573.7095	0.8	37.6	276	55.97	-5.0	even	ok
	272	C67H144N37O12P2	573.7085	-1.0	37.6	277	43.60	17.0	even	ok
	273	C73H29N24O28S	573.7096	0.9	37.7	278	53.27	73.0	even	ok
	274	C60H28N36NaO22P2S	573.7085	-0.9	37.7	279	53.91	67.0	even	ok
	275	C77H44N10NaO31P2S	573.7084	-1.2	37.8	280	49.61	63.0	even	ok
	276	C76H151N23O19P	573.7093	0.4	37.8	281	61.56	15.0	even	ok
	277	C67H37N16O41	573.7095	0.8	37.8	282	54.55	59.0	even	ok
	278	C69H159N21NaO22S2	573.7097	1.2	37.8	283	49.74	2.0	even	ok
	279	C66H12N48NaO10P2	573.7087	-0.5	37.9	284	49.40	87.0	even	ok
	280	C70H145N35NaO10P2	573.7090	-0.1	37.9	285	55.00	18.0	even	ok
	281	C63H167N17O29PS2	573.7093	0.4	38.0	286	60.73	-9.0	even	ok
	282	C77H46N4NaO40S	573.7097	1.1	38.1	287	50.19	58.0	even	ok
	283	C67H38N20NaO32S2	573.7095	0.7	38.2	288	55.78	60.0	even	ok
	284	C69H166N13O30S2	573.7096	1.0	38.2	289	51.55	-5.0	even	ok
	285	C79H33N14O34	573.7089	-0.2	38.3	290	62.79	72.0	even	ok
	286	C79H159N13O25P	573.7097	1.2	38.3	291	49.14	9.0	even	ok
	287	C56H152N39O14P2S2	573.7094	0.6	38.3	292	57.76	3.0	even	ok
	288	C70H16N42NaO12P2	573.7096	1.0	38.3	293	42.28	86.0	even	ok
	289	C64H37N22O34S2	573.7089	-0.2	38.3	294	62.99	59.0	even	ok
	290	C78H156N17NaO21P	573.7094	0.5	38.3	295	58.19	11.0	even	ok
	291	C67H147N35NaO12S2	573.7097	1.2	38.3	296	49.15	13.0	even	ok
	292	C71H15N40NaO15P	573.7086	-0.7	38.4	297	46.57	86.0	even	ok

Compound Spectrum SmartFormula Report

Meas. m/z	#	Ion Formula	m/z	err [ppm]	mSigma	# mSigma	Score	rdb	e⁻ Conf	N-Rule
	293	C68H170N9O34S2	573.7092	0.2	38.4	298	62.82	-10.0	even	ok
	294	C67H6N52NaO8S	573.7088	-0.5	38.4	299	59.02	92.0	even	ok
	295	C56H11N56O10P2S	573.7084	-1.1	38.4	300	42.03	82.0	even	ok
	296	C65H172N11NaO31PS2	573.7094	0.6	38.5	301	57.54	-13.0	even	ok
	297	C64H138N41O15	573.7093	0.5	38.5	302	48.93	18.0	even	ok
	298	C67H159N17NaO31	573.7090	-0.1	38.5	303	64.11	-2.0	even	ok
	299	C55H149N43NaO10P2S2	573.7090	-0.0	38.6	304	65.21	5.0	even	ok
	300	C66H20N44NaO9P2S2	573.7086	-0.8	38.6	305	54.09	81.0	even	ok
	301	C69H10N46O13P	573.7086	-0.9	38.7	306	44.19	90.0	even	ok
	302	C74H154N21O22S	573.7094	0.6	38.8	307	56.56	10.0	even	ok
	303	C55H10N54NaO15S	573.7094	0.6	38.8	308	56.51	79.0	even	ok
	304	C67H176N5O36P2S	573.7092	0.2	39.0	309	62.38	-15.0	even	ok
	305	C70H44N16NaO29P2S2	573.7086	-0.8	39.0	310	53.71	59.0	even	ok
	306	C61H155N27NaO25S	573.7096	1.1	39.0	311	49.66	-1.0	even	ok
	307	C68H11N48O10P2	573.7096	0.9	39.1	312	43.34	90.0	even	ok
	308	C66H171N9NaO34S2	573.7084	-1.2	39.2	313	47.77	-13.0	even	ok
	309	C68H9N48O12S	573.7091	0.1	39.3	314	62.54	90.0	even	ok
	310	C66H173N9NaO32P2S	573.7088	-0.4	39.3	315	58.24	-13.0	even	ok
	311	C58H23N42O20P2S	573.7084	-1.0	39.3	316	49.31	71.0	even	ok
	312	C68H140N41O8P2	573.7089	-0.2	39.4	317	50.95	22.0	even	ok
	313	C70H160N23O17P2S2	573.7088	-0.5	39.4	319	57.12	5.0	even	ok
	314	C67H145N41NaO3P2S2	573.7084	-1.1	39.5	320	48.06	18.0	even	ok
	315	C66H34N20NaO37	573.7092	0.2	39.5	321	61.13	61.0	even	ok
	316	C62H10N48NaO17	573.7092	0.2	39.7	322	50.89	83.0	even	ok
	317	C66H42N16NaO36S2	573.7090	-0.1	39.7	323	63.10	55.0	even	ok
	318	C64H7N54O8P2	573.7087	-0.7	39.7	324	45.10	91.0	even	ok
	319	C64H22N34NaO27	573.7092	0.2	39.7	325	50.73	72.0	even	ok
	320	C53H144N49O8P2S2	573.7089	-0.2	39.7	326	60.79	9.0	even	ok
	321	C63H130N55S2	573.7096	1.0	39.8	327	49.60	28.0	even	ok
	322	C75H34N18NaO30S	573.7097	1.1	39.8	328	47.98	69.0	even	ok
	323	C81H38N8NaO36	573.7090	-0.1	39.8	329	62.53	68.0	even	ok
	324	C58H31N34NaO25PS2	573.7087	-0.7	39.9	330	53.99	62.0	even	ok
	325	C72H172N9O27P2S2	573.7088	-0.5	40.0	331	56.31	-6.0	even	ok
	326	C70H175N3NaO36S2	573.7093	0.4	40.1	332	57.66	-14.0	even	ok
	327	C56H26N40O23PS2	573.7086	-0.8	40.2	333	51.27	66.0	even	ok
	328	C71H171N7NaO32S2	573.7097	1.2	40.3	334	46.42	-9.0	even	ok
	329	C65H147N31NaO21	573.7090	-0.1	40.3	335	50.79	9.0	even	ok
	330	C69H35N26O23P2S2	573.7090	-0.2	40.5	336	60.35	68.0	even	ok
	331	C78H47N6O35P2S	573.7087	-0.6	40.5	337	54.74	61.0	even	ok
	332	C64H168N15O30P2S	573.7087	-0.6	40.5	338	54.07	-9.0	even	ok
	333	C69H55NaO47PS	573.7084	-1.1	40.5	339	47.68	44.0	even	ok
	334	C59H150N33O23S	573.7096	0.9	40.6	340	49.89	3.0	even	ok
	335	C66H175N7O35PS2	573.7097	1.2	40.6	341	45.79	-15.0	even	ok
	336	C52H141N53NaO4P2S2	573.7086	-0.8	40.6	342	50.58	11.0	even	ok
	337	C53H5N60O13S	573.7093	0.4	40.7	343	55.98	83.0	even	ok
	338	C76H159N15NaO24S	573.7095	0.8	40.8	344	51.29	6.0	even	ok

Compound Spectrum SmartFormula Report

Meas. m/z	#	Ion Formula	m/z	err [ppm]	mSigma	# mSigma	Score	rdb	e¯ Conf	N-Rule
	339	C79H172N3O29P2S	573.7085	-0.9	40.9	345	49.48	-2.0	even	ok
	340	C70H14N42NaO14S	573.7092	0.3	40.9	346	57.59	86.0	even	ok
	341	C66H126N55S	573.7085	-1.0	41.0	347	48.13	33.0	even	ok
	342	C59H34N30O29PS2	573.7090	-0.0	41.0	348	61.04	60.0	even	ok
	343	C64H131N49NaO7	573.7094	0.6	41.1	349	43.79	25.0	even	ok
	344	C61H39N24NaO31PS2	573.7091	0.1	41.1	350	59.79	56.0	even	ok
	345	C67H23N40O13P2S2	573.7089	-0.2	41.3	351	58.89	79.0	even	ok
	346	C56H135N51NaO9S	573.7092	0.3	41.3	352	47.89	16.0	even	ok
	347	C80H158N11O28	573.7087	-0.6	41.3	353	53.05	9.0	even	ok
	348	C82H41N4O40	573.7094	0.5	41.3	354	53.55	66.0	even	ok
	349	C64H29N26O35	573.7091	0.1	41.4	355	60.32	65.0	even	ok
	350	C70H51N8O37P2S2	573.7085	-0.9	41.4	356	48.11	52.0	even	ok
	351	C57H32N36NaO22P2S2	573.7097	1.1	41.5	357	46.14	62.0	even	ok
	352	C67H174N5O38S2	573.7087	-0.6	41.7	358	52.94	-15.0	even	ok
	353	C67H8N52NaO6P2	573.7092	0.3	41.7	359	47.36	92.0	even	ok
	354	C68H148N37O7P2S2	573.7088	-0.5	41.8	360	53.48	16.0	even	ok
	355	C65H135N49NaO2S2	573.7097	1.1	41.8	361	44.80	24.0	even	ok
	356	C72H165N17NaO19P2S2	573.7089	-0.3	41.8	362	55.73	1.0	even	ok
	357	C75H32N24NaO21P2S	573.7084	-1.2	41.8	363	44.36	74.0	even	ok
	358	C68H131N49NaO2S	573.7086	-0.8	41.9	364	49.03	29.0	even	ok
	359	C73H143N33O13P	573.7088	-0.4	42.0	365	45.39	21.0	even	ok
	360	C58H147N37NaO19S	573.7092	0.3	42.0	366	56.33	5.0	even	ok
	361	C62H17N40O25	573.7091	0.0	42.0	367	49.46	76.0	even	ok
	362	C54H24N46NaO16P2S2	573.7092	0.3	42.0	368	55.98	68.0	even	ok
	363	C60H5N54O15	573.7091	0.0	42.1	369	49.48	87.0	even	ok
	364	C75H148N27NaO15P	573.7089	-0.2	42.1	370	47.10	17.0	even	ok
	365	C63H135N45NaO11	573.7090	-0.1	42.1	371	48.32	20.0	even	ok
	366	C55H27N42O20P2S2	573.7096	0.9	42.1	372	47.50	66.0	even	ok
	367	C50H136N59O2P2S2	573.7085	-1.0	42.1	373	46.36	15.0	even	ok
	368	C68H56N2NaO44P2S	573.7094	0.7	42.2	374	50.33	44.0	even	ok
	369	C71H17N38O18S	573.7096	0.9	42.4	375	46.94	84.0	even	ok
	370	C74H177N3NaO29P2S2	573.7089	-0.3	42.4	376	54.95	-10.0	even	ok
	371	C69H134N45O6S	573.7089	-0.2	42.4	377	56.76	27.0	even	ok
	372	C71H47N12O33P2S2	573.7090	-0.2	42.5	378	57.17	57.0	even	ok
	373	C67H137N45NaO4P2	573.7086	-0.9	42.6	379	39.69	24.0	even	ok
	374	C67H45N12O40S2	573.7094	0.6	42.7	380	51.34	53.0	even	ok
	375	C82H163N5NaO30	573.7088	-0.4	42.7	381	53.24	5.0	even	ok
	376	C69H171N3NaO41	573.7090	-0.1	42.7	382	57.19	-13.0	even	ok
	377	C63H142N37O19	573.7089	-0.3	42.7	383	45.68	13.0	even	ok
	378	C65H48N12NaO38P2S	573.7090	-0.1	42.8	384	57.40	50.0	even	ok
	379	C62H42N20O35PS2	573.7095	0.7	42.8	385	48.78	54.0	even	ok
	380	C80H52NaO37P2S	573.7088	-0.4	42.8	386	53.42	57.0	even	ok
	381	C66H51N8O42P2S	573.7093	0.5	42.8	387	51.60	48.0	even	ok
	382	C71H40N20NaO25P2S2	573.7090	0.0	42.9	388	58.50	64.0	even	ok
	383	C65H3N58O4P2	573.7091	0.1	42.9	389	47.56	96.0	even	ok
	384	C75H19N34NaO17P	573.7095	0.9	43.0	390	46.85	85.0	even	ok

Compound Spectrum SmartFormula Report

Meas. m/z	#	Ion Formula	m/z	err [ppm]	mSigma	# mSigma	Score	rdb	e¯ Conf	N-Rule
	385	C52H19N52O14P2S2	573.7091	0.1	43.0	391	56.63	72.0	even	ok
	386	C63H41N18O38S2	573.7085	-1.0	43.1	392	45.00	54.0	even	ok
	387	C62H126N55O5	573.7093	0.5	43.2	393	43.08	29.0	even	ok
	388	C64H47N14NaO37PS2	573.7096	0.9	43.3	394	45.99	50.0	even	ok
	389	C72H56N2NaO39P2S2	573.7086	-0.8	43.4	395	47.62	48.0	even	ok
	390	C51H16N56NaO10P2S2	573.7088	-0.5	43.5	396	51.04	74.0	even	ok
	391	C54H130N57O7S	573.7091	0.1	43.5	397	46.77	20.0	even	ok
	392	C72H140N37NaO9P	573.7085	-1.0	43.6	398	36.74	23.0	even	ok
	393	C71H141N39NaO6P2	573.7095	0.7	43.6	399	40.08	23.0	even	ok
	394	C69H28N34NaO15P2S2	573.7090	-0.0	43.6	400	57.25	75.0	even	ok
	395	C71H139N39NaO8S	573.7090	-0.0	43.7	401	56.72	23.0	even	ok
	396	C56H142N43O17S	573.7091	0.1	43.8	402	55.69	9.0	even	ok
	397	C63H43N18O36P2S	573.7089	-0.3	43.8	403	53.69	54.0	even	ok
	398	C83H166NO34	573.7092	0.2	43.8	404	54.40	3.0	even	ok
	399	C76H35N20O25P2S	573.7087	-0.6	43.9	405	49.69	72.0	even	ok
	400	C69H49N2O51	573.7095	0.8	43.9	406	45.98	48.0	even	ok
	401	C65H46N12NaO40S2	573.7086	-0.8	44.0	407	45.97	50.0	even	ok
	402	C70H153N31NaO9P2S2	573.7088	-0.3	44.0	408	52.30	12.0	even	ok
	403	C57H22N40NaO25S	573.7094	0.6	44.2	409	48.61	68.0	even	ok
	404	C65H154N23O29	573.7089	-0.3	44.2	410	43.84	2.0	even	ok
	405	C73H22N32NaO20S	573.7097	1.1	44.3	411	42.45	80.0	even	ok
	406	C62H40N22NaO32P2S	573.7085	-0.9	44.3	412	45.04	56.0	even	ok
	407	C69H50N6NaO42S2	573.7095	0.7	44.3	413	46.97	49.0	even	ok
	408	C65H132N51O2P2	573.7085	-1.0	44.3	414	36.09	28.0	even	ok
	409	C61H168N17NaO29PS2	573.7085	-1.0	44.4	415	43.43	-12.0	even	ok
	410	C69H136N45O4P2	573.7094	0.5	44.4	416	40.85	27.0	even	ok
	411	C61H130N51O9	573.7089	-0.3	44.5	417	43.40	24.0	even	ok
	412	C67H166N9O39	573.7089	-0.3	44.5	418	52.29	-9.0	even	ok
	413	C74H13N38O18	573.7085	-1.0	44.7	419	42.70	89.0	even	ok
	414	C59H163N23O27PS2	573.7084	-1.2	44.7	420	41.08	-8.0	even	ok
	415	C73H52N6NaO35P2S2	573.7090	0.0	44.8	421	55.33	53.0	even	ok
	416	C49H11N62O8P2S2	573.7087	-0.7	44.8	422	47.13	78.0	even	ok
	417	C72H142N35O12S	573.7094	0.6	44.8	423	47.93	21.0	even	ok
	418	C65H11N54O3P2S2	573.7089	-0.2	44.8	424	53.18	90.0	even	ok
	419	C68H46N6NaO47	573.7092	0.2	45.0	425	52.37	50.0	even	ok
	420	C73H168N13O23P2S2	573.7092	0.3	45.0	426	51.51	-1.0	even	ok
	421	C61H14N44NaO21	573.7087	-0.6	45.0	427	39.85	78.0	even	ok
	422	C62H171N13O33PS2	573.7088	-0.4	45.5	428	49.61	-14.0	even	ok
	423	C76H18N32NaO20	573.7085	-0.9	45.6	429	43.54	85.0	even	ok
	424	C64H176N7NaO35PS2	573.7089	-0.2	45.6	430	51.52	-18.0	even	ok
	425	C60H35N28O30P2S	573.7084	-1.0	45.6	431	41.43	60.0	even	ok
	426	C77H160N17O19P2S	573.7085	-0.9	45.6	432	43.18	9.0	even	ok
	427	C55H17N46O23S	573.7093	0.4	45.8	434	48.41	72.0	even	ok
	428	C77H21N28O24	573.7089	-0.2	45.9	435	50.69	83.0	even	ok
	429	C78H40N14NaO27P2S	573.7088	-0.4	46.0	436	48.64	68.0	even	ok
	430	C60H169N19NaO26P2S2	573.7095	0.7	46.1	437	44.41	-12.0	even	ok

Compound Spectrum SmartFormula Report

Meas. m/z	#	Ion Formula	m/z	err [ppm]	mSigma	# mSigma	Score	rdb	e⁻ Conf	N-Rule
	431	C63H167N13NaO35S	573.7096	1.1	46.1	439	40.66	-12.0	even	ok
	432	C62H139N41NaO15	573.7085	-0.9	46.1	440	35.27	15.0	even	ok
	433	C72H43N16O29P2S2	573.7094	0.6	46.3	441	45.55	62.0	even	ok
	434	C53H127N61NaO3S	573.7087	-0.5	46.3	442	38.96	22.0	even	ok
	435	C73H14N40O15P	573.7094	0.7	46.3	443	37.08	89.0	even	ok
	436	C66H163N13NaO35	573.7085	-0.9	46.4	444	42.13	-7.0	even	ok
	437	C57H161N29NaO20P2S2	573.7090	-0.0	46.5	445	52.26	-6.0	even	ok
	438	C66H49N8O44S2	573.7089	-0.2	46.5	446	50.12	48.0	even	ok
	439	C74H147N29NaO14S	573.7095	0.8	46.5	447	43.67	17.0	even	ok
	440	C66H41N12O45	573.7091	0.1	46.6	448	51.96	54.0	even	ok
	441	C70H31N30O19P2S2	573.7094	0.6	46.6	449	45.13	73.0	even	ok
	442	C58H164N25O24P2S2	573.7094	0.6	46.7	450	45.57	-8.0	even	ok
	443	C71H156N27O13P2S2	573.7092	0.3	46.7	451	49.11	10.0	even	ok
	444	C61H123N59NaO	573.7090	-0.1	46.9	453	41.96	31.0	even	ok
	445	C64H151N27NaO25	573.7085	-0.9	47.0	454	34.44	4.0	even	ok
	446	C67H16N48NaO5P2S2	573.7090	-0.0	47.0	455	51.86	86.0	even	ok
	447	C70H6N50O9P	573.7090	-0.1	47.0	456	42.52	95.0	even	ok
	448	C79H26N22NaO26	573.7090	-0.1	47.2	457	50.71	79.0	even	ok
	449	C68H133N49NaP2	573.7090	-0.1	47.2	458	42.36	29.0	even	ok
	450	C72H11N44NaO11P	573.7091	0.1	47.2	459	42.22	91.0	even	ok
	451	C65H179N3O39PS2	573.7093	0.4	47.2	460	46.85	-20.0	even	ok
	452	C54H14N50NaO19S	573.7089	-0.2	47.3	461	49.44	74.0	even	ok
	453	C61H162N19O33S	573.7096	0.9	47.3	462	41.07	-8.0	even	ok
	454	C49H135N57NaO7S2	573.7094	0.7	47.3	463	43.72	12.0	even	ok
	455	C55H156N35O18P2S2	573.7089	-0.2	47.4	464	48.94	-2.0	even	ok
	456	C75H173N7NaO25P2S2	573.7093	0.4	47.5	465	45.97	-5.0	even	ok
	457	C59H9N50O19	573.7086	-0.7	47.5	466	35.52	82.0	even	ok
	458	C63H26N30NaO31	573.7087	-0.6	47.5	467	37.14	67.0	even	ok
	459	C79H165N11NaO21P2S	573.7086	-0.7	47.6	468	42.56	5.0	even	ok
	460	C68H54N2NaO46S2	573.7090	-0.1	47.7	469	50.34	44.0	even	ok
	461	C54H153N39NaO14P2S2	573.7086	-0.8	47.7	470	41.32	0.0	even	ok
	462	C65H38N16NaO41	573.7087	-0.6	48.0	471	43.90	56.0	even	ok
	463	C80H29N18O30	573.7094	0.5	48.1	472	44.21	77.0	even	ok
	464	C73H20N38NaO11P2S	573.7084	-1.2	48.1	473	36.93	85.0	even	ok
	465	C60H159N23NaO29S	573.7092	0.3	48.1	474	47.09	-6.0	even	ok
	466	C66H180NO40P2S	573.7087	-0.6	48.2	475	43.40	-20.0	even	ok
	467	C60H127N55NaO5	573.7085	-0.9	48.3	477	33.03	26.0	even	ok
	468	C64H158N19O33	573.7084	-1.1	48.3	478	37.95	-3.0	even	ok
	469	C68H4N56NaO2P2	573.7096	1.0	48.3	479	31.84	97.0	even	ok
	470	C74H55N2O39P2S2	573.7094	0.6	48.4	480	42.72	51.0	even	ok
	471	C85H47O37P2	573.7085	-0.9	48.5	481	39.06	65.0	even	ok
	472	C79H43N10O31P2S	573.7092	0.2	48.5	482	47.12	66.0	even	ok
	473	C69H3N54NaO5P	573.7086	-0.7	48.6	483	34.76	97.0	even	ok
	474	C60H134N47O13	573.7084	-1.1	48.6	484	31.25	19.0	even	ok
	475	C51H122N67OS	573.7087	-0.7	48.7	485	34.78	26.0	even	ok
	476	C74H48N10NaO31P2S2	573.7095	0.8	48.8	486	40.46	58.0	even	ok

Compound Spectrum SmartFormula Report

Meas. m/z	#	Ion Formula	m/z	err [ppm]	mSigma	# mSigma	Score	rdb	e⁻ Conf	N-Rule
	477	C47H130N63O5S2	573.7093	0.5	48.8	487	43.62	16.0	even	ok
	478	C52H148N45O12P2S2	573.7085	-1.0	49.0	488	38.06	4.0	even	ok
	479	C73H161N21NaO15P2S2	573.7093	0.4	49.1	489	43.89	6.0	even	ok
	480	C52H9N56O17S	573.7088	-0.3	49.1	490	45.02	78.0	even	ok
	481	C60H43N20NaO35PS2	573.7087	-0.7	49.1	491	41.47	51.0	even	ok
	482	C69H5N52O8S	573.7096	0.9	49.1	492	38.59	95.0	even	ok
	483	C72H36N24NaO21P2S2	573.7095	0.8	49.1	493	40.12	69.0	even	ok
	484	C58H38N26O33PS2	573.7086	-0.8	49.3	494	39.48	55.0	even	ok
	485	C62H146N33O23	573.7084	-1.1	49.4	495	30.62	8.0	even	ok
	486	C68H19N44O9P2S2	573.7094	0.6	49.4	496	41.56	84.0	even	ok
	487	C74H23N34O15P2S	573.7087	-0.6	49.5	497	42.00	83.0	even	ok
	488	C58H154N29O27S	573.7091	0.1	49.6	498	46.80	-2.0	even	ok
	489	C82H34N12NaO32	573.7094	0.7	49.7	499	40.32	73.0	even	ok
	490	C80H168N7O25P2S	573.7090	-0.1	49.7	500	46.69	3.0	even	ok
	491	C61H21N36O29	573.7086	-0.7	49.7	501	33.25	71.0	even	ok
	492	C78H146N25O18	573.7087	-0.6	49.8	502	41.45	20.0	even	ok
	493	C55H139N47NaO13S	573.7087	-0.5	49.8	503	35.11	11.0	even	ok
	494	C63H33N22O39	573.7086	-0.7	49.9	504	39.82	60.0	even	ok
	495	C46H127N67NaOS2	573.7090	-0.1	50.1	505	46.00	18.0	even	ok
	496	C60H15N46NaO18P	573.7097	1.2	50.1	506	29.13	78.0	even	ok
	497	C61H46N16O39PS2	573.7090	-0.0	50.4	507	46.51	49.0	even	ok
	498	C48H10N60NaO13S2	573.7096	1.0	50.4	508	36.32	75.0	even	ok
	499	C63H51N10NaO41PS2	573.7091	0.1	50.5	509	45.39	45.0	even	ok
	500	C59H44N22NaO32P2S2	573.7097	1.1	50.6	510	35.34	51.0	even	ok
588.3833	1	C71H143N39O12P	588.3833	-0.0	8.4	1	93.78	22.0	even	ok
	2	C70H140N43NaO8P	588.3830	-0.6	8.6	2	80.10	24.0	even	ok
	3	C69H15N46NaO14P	588.3832	-0.3	8.7	3	87.11	87.0	even	ok
	4	C67H10N52O12P	588.3831	-0.5	9.3	4	82.73	91.0	even	ok
	5	C73H148N33NaO14P	588.3834	0.1	9.7	5	89.78	18.0	even	ok
	6	C68H135N49O6P	588.3829	-0.8	9.7	6	75.04	28.0	even	ok
	7	C70H18N42O18P	588.3835	0.3	10.3	7	85.18	85.0	even	ok
	8	C68H6N56O8P	588.3835	0.3	10.9	8	84.23	96.0	even	ok
	9	C69H141N45NaO5P2	588.3840	1.1	11.5	9	67.67	24.0	even	ok
	10	C67H3N60NaO4P	588.3832	-0.3	11.5	10	82.60	98.0	even	ok
	11	C72H152N29NaO18P	588.3830	-0.6	12.2	11	74.98	13.0	even	ok
	12	C70H11N50NaO10P	588.3836	0.4	12.3	12	78.82	92.0	even	ok
	13	C70H147N35O16P	588.3829	-0.8	12.9	13	70.80	17.0	even	ok
	14	C62H131N59PS	588.3835	0.3	13.0	14	79.73	29.0	even	ok
	15	C72H23N36NaO20P	588.3836	0.4	13.3	15	92.68	81.0	even	ok
	16	C67H136N51O3P2	588.3839	0.9	13.8	16	67.71	28.0	even	ok
	17	C74H151N29O18P	588.3838	0.7	13.9	17	85.09	16.0	even	ok
	18	C71H14N46O14P	588.3840	1.0	14.4	18	63.99	90.0	even	ok
	19	C73H155N25O22P	588.3833	-0.0	14.6	19	100.00	11.0	even	ok
	20	C71H153N31NaO15P2	588.3840	1.1	15.0	20	63.02	13.0	even	ok
	21	C69H131N53O2P	588.3833	-0.0	15.0	21	82.48	33.0	even	ok
	22	C64H136N53NaO2PS	588.3836	0.5	15.1	22	88.18	25.0	even	ok

Compound Spectrum SmartFormula Report

Meas. m/z	#	Ion Formula	m/z	err [ppm]	mSigma	# mSigma	Score	rdb	e¯ Conf	N-Rule
	23	C75H160N19NaO24P	588.3834	0.1	15.2	23	96.65	7.0	even	ok
	24	C76H156N23NaO20P	588.3839	0.9	15.7	24	78.50	12.0	even	ok
	25	C71H136N47NaO4P	588.3834	0.1	15.7	25	80.04	29.0	even	ok
	26	C73H26N32O24P	588.3840	1.1	15.7	26	74.59	79.0	even	ok
	27	C65H8N58NaO5P2	588.3837	0.6	16.1	27	69.90	93.0	even	ok
	28	C60H3N66NaO2PS	588.3834	0.1	16.2	28	80.18	94.0	even	ok
	29	C72H139N43O8P	588.3838	0.7	16.4	29	67.52	27.0	even	ok
	30	C69H148N37O13P2	588.3839	0.9	16.8	30	63.72	17.0	even	ok
	31	C68H19N42NaO18P	588.3827	-1.1	17.1	31	60.15	82.0	even	ok
	32	C71H27N32NaO24P	588.3832	-0.3	17.3	32	88.59	76.0	even	ok
	33	C69H22N38O22P	588.3831	-0.5	17.6	33	84.53	80.0	even	ok
	34	C76H163N15O28P	588.3838	0.7	17.6	34	78.73	5.0	even	ok
	35	C75H150N27O21	588.3828	-1.0	17.8	35	73.28	16.0	even	ok
	36	C61H6N62O6PS	588.3837	0.7	18.3	36	78.92	92.0	even	ok
	37	C74H144N37NaO10P	588.3839	0.9	18.3	37	62.04	23.0	even	ok
	38	C72H30N28O28P	588.3835	0.3	18.7	38	86.32	74.0	even	ok
	39	C63H3N64O3P2	588.3836	0.5	18.7	39	69.00	97.0	even	ok
	40	C68H145N41NaO9P2	588.3835	0.3	18.9	40	71.76	19.0	even	ok
	41	C63H11N56NaO8PS	588.3838	0.8	19.1	41	74.26	88.0	even	ok
	42	C78H168N9NaO30P	588.3839	0.9	19.1	42	72.96	1.0	even	ok
	43	C74H35N22NaO30P	588.3836	0.5	19.4	43	81.69	70.0	even	ok
	44	C74H25N30O27	588.3830	-0.6	19.5	44	77.62	79.0	even	ok
	45	C62H5N60O9S	588.3827	-1.0	19.6	45	69.57	92.0	even	ok
	46	C72H13N44O17	588.3830	-0.6	19.7	46	77.10	90.0	even	ok
	47	C77H155N21NaO23	588.3829	-0.8	20.1	48	73.06	12.0	even	ok
	48	C77H162N13O31	588.3828	-1.0	20.4	49	69.46	5.0	even	ok
	49	C66H138N47O9S	588.3830	-0.6	20.6	50	76.89	23.0	even	ok
	50	C66H131N55NaOS	588.3831	-0.4	20.8	51	79.67	30.0	even	ok
	51	C63H140N49NaO6PS	588.3832	-0.3	20.8	52	83.34	20.0	even	ok
	52	C60H132N59NaPS	588.3827	-1.0	21.1	53	56.16	26.0	even	ok
	53	C64H10N54NaO11S	588.3828	-0.9	21.1	54	70.48	88.0	even	ok
	54	C67H20N44NaO15P2	588.3837	0.6	21.1	55	62.80	82.0	even	ok
	55	C73H138N41O11	588.3828	-1.0	21.2	56	56.67	27.0	even	ok
	56	C66H140N47O7P2	588.3834	0.1	21.3	58	70.80	23.0	even	ok
	57	C61H135N55O4PS	588.3831	-0.4	21.5	59	65.81	24.0	even	ok
	58	C74H164N15NaO28P	588.3830	-0.6	21.5	60	74.48	2.0	even	ok
	59	C76H30N24NaO29	588.3831	-0.5	21.8	61	77.04	75.0	even	ok
	60	C72H159N21O26P	588.3829	-0.8	21.9	62	70.72	6.0	even	ok
	61	C75H38N18O34P	588.3840	1.1	21.9	63	65.36	68.0	even	ok
	62	C74H18N38NaO19	588.3831	-0.5	22.0	64	76.61	86.0	even	ok
	63	C64H143N45O10PS	588.3835	0.3	22.2	65	78.99	18.0	even	ok
	64	C65H6N58NaO7S	588.3833	-0.1	22.3	66	83.80	93.0	even	ok
	65	C79H167N7NaO33	588.3829	-0.8	22.5	67	69.49	1.0	even	ok
	66	C68H143N41NaO11S	588.3831	-0.4	22.7	68	76.53	19.0	even	ok
	67	C66H148N39NaO12PS	588.3836	0.5	22.7	69	74.93	14.0	even	ok
	68	C75H167N11O32P	588.3833	-0.0	22.8	70	84.39	0.0	even	ok

Compound Spectrum SmartFormula Report

Meas. m/z	#	Ion Formula	m/z	err [ppm]	mSigma	# mSigma	Score	rdb	e⁻ Conf	N-Rule
	69	C65H15N50O13P2	588.3836	0.5	23.1	71	62.69	86.0	even	ok
	70	C77H172N5NaO34P	588.3834	0.1	23.2	72	81.51	-4.0	even	ok
	71	C78H158N17O27	588.3832	-0.2	23.4	73	79.76	10.0	even	ok
	72	C73H165N17NaO25P2	588.3840	1.1	23.4	74	63.17	2.0	even	ok
	73	C75H143N35NaO13	588.3829	-0.8	23.7	75	56.24	23.0	even	ok
	74	C67H134N51O5S	588.3834	0.2	24.0	76	79.27	28.0	even	ok
	75	C65H13N50O15S	588.3832	-0.3	24.3	77	77.05	86.0	even	ok
	76	C76H37N16O37	588.3830	-0.6	24.4	78	69.84	68.0	even	ok
	77	C65H137N51NaO3P2	588.3831	-0.5	24.4	79	60.78	25.0	even	ok
	78	C75H21N34O23	588.3834	0.1	24.9	80	78.78	84.0	even	ok
	79	C59H7N62NaO6PS	588.3829	-0.7	24.9	81	67.97	89.0	even	ok
	80	C71H160N23O23P2	588.3839	0.9	25.3	82	52.92	6.0	even	ok
	81	C64H12N54NaO9P2	588.3833	-0.1	25.3	83	64.70	88.0	even	ok
	82	C76H146N31O17	588.3832	-0.2	25.3	84	76.30	21.0	even	ok
	83	C77H33N20O33	588.3834	0.1	25.4	85	77.81	73.0	even	ok
	84	C62H15N52NaO12PS	588.3834	0.1	25.5	86	78.59	83.0	even	ok
	85	C78H175NO38P	588.3838	0.7	25.5	87	66.18	-6.0	even	ok
	86	C60H10N58O10PS	588.3833	-0.1	25.5	88	78.22	87.0	even	ok
	87	C70H31N28NaO28P	588.3827	-1.1	25.7	89	59.91	71.0	even	ok
	88	C66H9N54O11S	588.3836	0.5	25.8	91	70.01	91.0	even	ok
	89	C70H157N27NaO19P2	588.3835	0.3	25.9	92	61.35	8.0	even	ok
	90	C80H163N11NaO29	588.3833	-0.0	25.9	93	78.20	6.0	even	ok
	91	C80H170N3O37	588.3832	-0.2	26.1	94	75.13	-1.0	even	ok
	92	C73H39N18NaO34P	588.3832	-0.3	26.1	95	73.11	65.0	even	ok
	93	C78H42N10NaO39	588.3831	-0.5	26.3	96	69.74	64.0	even	ok
	94	C71H34N24O32P	588.3831	-0.5	26.3	97	69.96	69.0	even	ok
	95	C67H18N44NaO17S	588.3833	-0.1	26.3	98	76.55	82.0	even	ok
	96	C69H146N37O15S	588.3834	0.2	26.3	99	75.13	17.0	even	ok
	97	C69H139N45NaO7S	588.3835	0.3	26.6	100	71.86	24.0	even	ok
	98	C72H132N51NaP	588.3839	0.9	26.9	102	51.42	34.0	even	ok
	99	C68H150N33O19S	588.3830	-0.6	27.0	103	66.73	12.0	even	ok
	100	C63H18N48O16PS	588.3837	0.7	27.1	104	64.82	81.0	even	ok
	101	C63H132N57OP2	588.3830	-0.6	27.1	105	54.68	29.0	even	ok
	102	C77H26N28NaO25	588.3835	0.3	27.4	106	71.43	80.0	even	ok
	103	C78H151N25NaO19	588.3833	-0.1	27.6	107	75.04	17.0	even	ok
	104	C68H152N33O17P2	588.3834	0.1	27.7	108	61.24	12.0	even	ok
	105	C64H17N46O19S	588.3827	-1.0	27.7	109	58.12	81.0	even	ok
	106	C65H23N42NaO18PS	588.3838	0.8	27.7	110	61.17	77.0	even	ok
	107	C74H42N14O38P	588.3835	0.3	27.7	111	70.53	63.0	even	ok
	108	C62H7N60O7P2	588.3832	-0.3	27.7	112	58.74	92.0	even	ok
	109	C79H38N14NaO35	588.3835	0.3	27.9	113	70.57	69.0	even	ok
	110	C72H6N52NaO9	588.3831	-0.5	28.0	114	55.59	97.0	even	ok
	111	C76H47N8NaO40P	588.3836	0.5	28.2	115	66.91	59.0	even	ok
	112	C68H14N48NaO13S	588.3837	0.7	28.4	117	63.22	87.0	even	ok
	113	C69H32N30NaO25P2	588.3837	0.6	28.5	118	63.60	71.0	even	ok
	114	C70H155N27NaO21S	588.3831	-0.4	28.6	119	66.87	8.0	even	ok

Compound Spectrum SmartFormula Report

Meas. m/z	#	Ion Formula	m/z	err [ppm]	mSigma	# mSigma	Score	rdb	e⁻ Conf	N-Rule
	115	C71H151N31NaO17S	588.3835	0.3	28.7	120	68.30	13.0	even	ok
	116	C66H22N40NaO21S	588.3828	-0.9	28.8	121	59.23	77.0	even	ok
	117	C58H8N64NaO3P2S	588.3839	1.0	28.8	122	47.29	89.0	even	ok
	118	C67H149N37NaO13P2	588.3831	-0.5	29.3	123	54.20	14.0	even	ok
	119	C71H126N55O	588.3828	-1.0	29.4	124	46.93	38.0	even	ok
	120	C81H166N7O33	588.3837	0.6	29.5	125	63.17	4.0	even	ok
	121	C62H144N45NaO10PS	588.3827	-1.0	29.6	126	55.52	15.0	even	ok
	122	C69H15N50O8P2S	588.3828	-1.0	29.7	127	56.36	90.0	even	ok
	123	C79H45N6O43	588.3834	0.1	29.8	128	69.92	62.0	even	ok
	124	C68H21N40O21S	588.3836	0.5	29.9	129	63.44	80.0	even	ok
	125	C65H152N35NaO16PS	588.3832	-0.2	30.1	130	67.47	9.0	even	ok
	126	C56H3N70OP2S	588.3838	0.8	30.2	131	47.91	93.0	even	ok
	127	C70H142N41O11S	588.3839	0.9	30.2	132	56.04	22.0	even	ok
	128	C63H147N41O14PS	588.3831	-0.4	30.3	133	64.55	13.0	even	ok
	129	C73H9N48O13	588.3834	0.1	30.3	134	57.91	95.0	even	ok
	130	C79H154N21O23	588.3837	0.6	30.5	135	61.77	15.0	even	ok
	131	C77H50N4O44P	588.3840	1.1	30.5	136	53.44	57.0	even	ok
	132	C61H4N64NaO3P2	588.3828	-0.9	30.6	137	46.55	94.0	even	ok
	133	C78H29N24O29	588.3839	0.9	30.8	138	56.05	78.0	even	ok
	134	C76H176NNaO38P	588.3830	-0.6	30.8	139	60.08	-9.0	even	ok
	135	C75H14N42NaO15	588.3835	0.3	30.9	140	65.78	91.0	even	ok
	136	C74H171N7O36P	588.3829	-0.8	31.1	141	57.21	-5.0	even	ok
	137	C71H27N36O18P2S	588.3828	-0.9	31.1	142	54.52	79.0	even	ok
	138	C73H131N49NaO3	588.3829	-0.8	31.2	143	47.06	34.0	even	ok
	139	C56H130N63OS2	588.3828	-1.0	31.2	144	54.20	25.0	even	ok
	140	C67H27N36O23P2	588.3836	0.5	31.3	145	51.62	75.0	even	ok
	141	C62H131N55NaO6	588.3839	1.0	31.3	146	44.78	26.0	even	ok
	142	C57H136N59NaPS2	588.3839	0.9	31.4	147	55.06	21.0	even	ok
	143	C68H130N55OS	588.3839	0.9	31.5	148	54.40	33.0	even	ok
	144	C65H144N43O11P2	588.3830	-0.6	31.6	150	49.20	18.0	even	ok
	145	C66H155N31O20PS	588.3836	0.4	31.6	151	63.33	7.0	even	ok
	146	C80H41N10O39	588.3839	0.9	31.7	152	54.73	67.0	even	ok
	147	C67H25N36O25S	588.3832	-0.3	31.8	153	64.74	75.0	even	ok
	148	C68H160N25NaO22PS	588.3836	0.5	32.0	154	60.17	3.0	even	ok
	149	C81H50NaO45	588.3835	0.3	32.0	155	63.74	58.0	even	ok
	150	C60H134N57O3S2	588.3837	0.6	32.0	156	59.39	24.0	even	ok
	151	C78H49N2O47	588.3830	-0.6	32.0	157	58.28	57.0	even	ok
	152	C71H158N23O25S	588.3834	0.2	32.1	158	65.34	6.0	even	ok
	153	C58H135N57NaO3S2	588.3829	-0.8	32.2	159	55.37	21.0	even	ok
	154	C70H26N34NaO23S	588.3837	0.7	32.2	160	57.55	76.0	even	ok
	155	C63H140N53P2S2	588.3828	-0.9	32.2	161	53.93	23.0	even	ok
	156	C83H171NNaO35	588.3838	0.7	32.2	162	56.66	0.0	even	ok
	157	C61H145N47NaO7P2S	588.3837	0.7	32.2	163	57.19	15.0	even	ok
	158	C66H24N40NaO19P2	588.3833	-0.1	32.3	164	54.76	77.0	even	ok
	159	C71H20N44NaO10P2S	588.3829	-0.8	32.4	166	55.19	86.0	even	ok
	160	C75H177N3NaO35P2	588.3840	1.1	32.5	167	50.92	-9.0	even	ok

Compound Spectrum SmartFormula Report

Meas. m/z	#	Ion Formula	m/z	err [ppm]	mSigma	# mSigma	Score	rdb	e⁻ Conf	N-Rule
	161	C72H154N27O21S	588.3839	0.9	32.5	168	52.81	11.0	even	ok
	162	C74H134N45O7	588.3832	-0.2	32.7	169	53.09	32.0	even	ok
	163	C58H6N64NaO5S2	588.3835	0.3	33.1	170	62.08	89.0	even	ok
	164	C81H159N15NaO25	588.3838	0.7	33.1	171	55.48	11.0	even	ok
	165	C73H172N9O33P2	588.3839	0.9	33.1	172	52.44	-5.0	even	ok
	166	C72H169N13NaO29P2	588.3835	0.3	33.1	173	61.70	-3.0	even	ok
	167	C69H30N30NaO27S	588.3833	-0.1	33.3	174	64.73	71.0	even	ok
	168	C76H17N38O19	588.3839	0.9	33.5	175	52.54	89.0	even	ok
	169	C80H34N18NaO31	588.3840	1.0	33.5	176	50.05	74.0	even	ok
	170	C73H32N30NaO20P2S	588.3829	-0.8	33.7	177	53.48	75.0	even	ok
	171	C60H126N61O4	588.3838	0.8	33.9	178	43.91	30.0	even	ok
	172	C73H163N17NaO27S	588.3835	0.3	34.1	179	59.74	2.0	even	ok
	173	C62H139N51NaO5S2	588.3838	0.7	34.1	180	54.05	20.0	even	ok
	174	C61H19N48NaO16PS	588.3829	-0.7	34.2	181	54.43	78.0	even	ok
	175	C64H19N46O17P2	588.3832	-0.3	34.2	182	50.20	81.0	even	ok
	176	C84H172N3O30P2	588.3828	-0.9	34.3	183	51.12	3.0	even	ok
	177	C70H164N19O27P2	588.3834	0.1	34.3	184	62.29	1.0	even	ok
	178	C82H46N4NaO41	588.3840	1.0	34.3	185	48.92	63.0	even	ok
	179	C53H3N72NaPS2	588.3836	0.5	34.4	186	57.55	90.0	even	ok
	180	C69H8N58NaP2S	588.3829	-0.8	34.5	187	52.26	97.0	even	ok
	181	C65H145N47NaO2P2S2	588.3829	-0.7	34.6	188	53.02	19.0	even	ok
	182	C59H14N54O14PS	588.3828	-0.8	34.7	189	51.36	82.0	even	ok
	183	C59H138N53O7S2	588.3832	-0.2	35.0	190	60.50	19.0	even	ok
	184	C77H142N35O13	588.3837	0.5	35.0	191	55.35	26.0	even	ok
	185	C72H43N14NaO38P	588.3827	-1.1	35.0	192	47.84	60.0	even	ok
	186	C76H139N39NaO9	588.3833	-0.1	35.0	193	52.05	28.0	even	ok
	187	C64H27N38NaO22PS	588.3834	0.1	35.1	194	62.06	72.0	even	ok
	188	C62H22N44O20PS	588.3833	-0.1	35.1	195	62.03	76.0	even	ok
	189	C73H148N37O8P2S	588.3830	-0.5	35.1	196	55.51	21.0	even	ok
	190	C75H160N23O18P2S	588.3830	-0.5	35.2	197	55.54	10.0	even	ok
	191	C83H47N6O36P2	588.3830	-0.6	35.3	198	54.52	66.0	even	ok
	192	C70H162N19O29S	588.3830	-0.6	35.3	199	54.41	1.0	even	ok
	193	C59H140N53O5P2S	588.3836	0.5	35.4	200	45.85	19.0	even	ok
	194	C73H39N22O28P2S	588.3828	-0.9	35.7	201	48.60	68.0	even	ok
	195	C75H51N4NaO44P	588.3832	-0.3	35.8	202	57.71	54.0	even	ok
	196	C62H15N56O6P2S2	588.3830	-0.6	35.8	203	53.80	86.0	even	ok
	197	C73H46N10O42P	588.3831	-0.5	35.9	204	55.31	58.0	even	ok
	198	C78H22N32NaO21	588.3839	1.0	36.0	205	47.06	85.0	even	ok
	199	C63H16N50NaO13P2	588.3828	-0.9	36.0	206	40.74	83.0	even	ok
	200	C72H23N40O14P2S	588.3832	-0.2	36.0	207	58.98	84.0	even	ok
	201	C54H6N68O4PS2	588.3840	1.1	36.1	208	46.53	88.0	even	ok
	202	C58H137N57NaOP2S	588.3833	-0.1	36.1	209	50.27	21.0	even	ok
	203	C61H143N47NaO9S2	588.3833	-0.0	36.3	210	60.66	15.0	even	ok
	204	C60H20N50NaO13P2S	588.3839	1.0	36.4	211	46.86	78.0	even	ok
	205	C55H5N66O7S2	588.3830	-0.6	36.4	212	52.11	88.0	even	ok
	206	C59H9N60O9S2	588.3839	0.9	36.5	213	48.37	87.0	even	ok

Compound Spectrum SmartFormula Report

Meas. m/z	#	Ion Formula	m/z	err [ppm]	mSigma	# mSigma	Score	rdb	e⁻ Conf	N-Rule
	207	C81H35N20O26P2	588.3830	-0.6	36.6	214	52.71	77.0	even	ok
	208	C72H167N13NaO31S	588.3831	-0.4	36.6	215	54.81	-3.0	even	ok
	209	C70H33N26O31S	588.3836	0.5	36.6	216	53.45	69.0	even	ok
	210	C65H30N34O26PS	588.3837	0.7	36.7	217	50.92	70.0	even	ok
	211	C66H29N32O29S	588.3827	-1.0	36.9	218	46.20	70.0	even	ok
	212	C59H123N65Na	588.3835	0.2	37.0	219	47.60	32.0	even	ok
	213	C82H160N17O20P2	588.3828	-0.9	37.2	220	47.34	14.0	even	ok
	214	C67H35N28NaO28PS	588.3838	0.8	37.2	221	48.13	66.0	even	ok
	215	C79H147N29NaO15	588.3838	0.7	37.2	222	49.96	22.0	even	ok
	216	C71H44N16NaO35P2	588.3837	0.6	37.3	223	50.88	60.0	even	ok
	217	C58H15N56O11P2S	588.3838	0.9	37.3	224	47.89	82.0	even	ok
	218	C69H161N23NaO23P2	588.3831	-0.5	37.3	225	44.39	3.0	even	ok
	219	C57H10N60NaO9S2	588.3831	-0.5	37.3	226	53.03	84.0	even	ok
	220	C76H54O48P	588.3835	0.3	37.4	227	55.21	52.0	even	ok
	221	C70H11N54O4P2S	588.3832	-0.2	37.5	228	56.65	95.0	even	ok
	222	C64H143N41NaO16	588.3839	1.0	37.5	229	38.13	15.0	even	ok
	223	C74H35N26O24P2S	588.3832	-0.2	37.6	230	56.64	73.0	even	ok
	224	C74H166N13O31S	588.3839	0.9	37.7	231	46.07	0.0	even	ok
	225	C68H34N26NaO31S	588.3828	-0.9	37.8	232	47.26	66.0	even	ok
	226	C75H153N31NaO10P2S	588.3831	-0.4	37.8	233	53.84	17.0	even	ok
	227	C65H152N39O10P2S2	588.3828	-0.9	37.8	234	46.69	12.0	even	ok
	228	C77H165N17NaO20P2S	588.3831	-0.4	37.9	235	53.88	6.0	even	ok
	229	C75H44N16NaO30P2S	588.3829	-0.8	38.0	236	47.90	64.0	even	ok
	230	C63H11N60O2P2S2	588.3835	0.2	38.0	237	55.88	91.0	even	ok
	231	C85H52NaO38P2	588.3831	-0.4	38.1	238	52.85	62.0	even	ok
	232	C64H20N50NaO8P2S2	588.3831	-0.4	38.2	239	52.77	82.0	even	ok
	233	C69H39N22O33P2	588.3836	0.5	38.2	240	51.78	64.0	even	ok
	234	C77H172N9O28P2S	588.3830	-0.5	38.2	241	51.31	-1.0	even	ok
	235	C61H11N56O11P2	588.3827	-1.1	38.2	242	36.65	87.0	even	ok
	236	C61H14N54NaO11S2	588.3840	1.0	38.4	243	43.92	83.0	even	ok
	237	C72H38N20NaO33S	588.3837	0.7	38.5	244	48.77	65.0	even	ok
	238	C66H148N43O6P2S2	588.3833	-0.1	38.5	245	55.99	17.0	even	ok
	239	C68H36N26NaO29P2	588.3833	-0.1	38.5	246	55.97	66.0	even	ok
	240	C74H28N34NaO16P2S	588.3833	-0.0	38.8	247	56.93	80.0	even	ok
	241	C67H156N29O21P2	588.3830	-0.6	39.0	248	40.65	7.0	even	ok
	242	C64H156N31NaO20PS	588.3827	-1.0	39.1	249	43.65	4.0	even	ok
	243	C56H140N55NaO4PS2	588.3834	0.1	39.1	250	55.04	16.0	even	ok
	244	C54H135N61O2PS2	588.3833	-0.0	39.2	251	56.39	20.0	even	ok
	245	C83H40N14NaO28P2	588.3831	-0.4	39.2	252	51.15	73.0	even	ok
	246	C62H146N43O13S2	588.3837	0.6	39.5	253	48.83	13.0	even	ok
	247	C62H138N47O14	588.3838	0.8	39.6	254	37.70	19.0	even	ok
	248	C84H165N11NaO22P2	588.3829	-0.7	39.7	255	46.24	10.0	even	ok
	249	C73H170N9O35S	588.3835	0.2	39.8	256	53.40	-5.0	even	ok
	250	C66H31N32O27P2	588.3832	-0.3	39.9	258	51.91	70.0	even	ok
	251	C67H164N21NaO26PS	588.3832	-0.2	39.9	259	52.48	-2.0	even	ok
	252	C67H157N33NaO12P2S2	588.3829	-0.7	39.9	260	46.13	8.0	even	ok

Compound Spectrum SmartFormula Report

Meas. m/z	#	Ion Formula	m/z	err [ppm]	mSigma	# mSigma	Score	rdb	e⁻ Conf	N-Rule
	253	C65H159N27O24PS	588.3831	-0.4	40.0	261	50.29	2.0	even	ok
	254	C58H13N56O13S2	588.3834	0.1	40.1	262	53.71	82.0	even	ok
	255	C72H16N48NaO6P2S	588.3833	-0.0	40.2	263	54.76	91.0	even	ok
	256	C57H12N60NaO7P2S	588.3835	0.2	40.2	264	43.34	84.0	even	ok
	257	C76H40N20NaO26P2S	588.3833	-0.0	40.2	265	54.77	69.0	even	ok
	258	C69H37N22O35S	588.3832	-0.2	40.5	266	51.62	64.0	even	ok
	259	C79H23N34O16P2	588.3830	-0.6	40.7	267	47.07	88.0	even	ok
	260	C65H16N54NaO4P2S2	588.3835	0.4	40.7	268	49.95	87.0	even	ok
	261	C58H142N49O11S2	588.3828	-1.0	40.7	269	42.36	14.0	even	ok
	262	C57H143N51O8PS2	588.3838	0.7	40.7	270	45.06	14.0	even	ok
	263	C73H141N45NaP2S	588.3831	-0.4	40.7	271	49.72	28.0	even	ok
	264	C79H177N3NaO30P2S	588.3831	-0.3	40.7	272	49.93	-5.0	even	ok
	265	C68H153N37NaO8P2S2	588.3834	0.0	41.1	273	53.55	13.0	even	ok
	266	C59H148N45NaO10PS2	588.3839	0.9	41.1	274	42.64	10.0	even	ok
	267	C64H151N37NaO15S2	588.3838	0.7	41.2	275	44.67	9.0	even	ok
	268	C63H157N33NaO17P2S	588.3837	0.7	41.3	276	44.99	4.0	even	ok
	269	C60H18N50NaO15S2	588.3835	0.3	41.4	277	49.84	78.0	even	ok
	270	C75H175N3NaO37S	588.3835	0.3	41.4	278	49.09	-9.0	even	ok
	271	C76H156N27O14P2S	588.3835	0.2	41.4	279	50.37	15.0	even	ok
	272	C68H167N17O30PS	588.3836	0.4	41.4	280	48.85	-4.0	even	ok
	273	C60H147N43NaO13S2	588.3829	-0.8	41.5	281	43.40	10.0	even	ok
	274	C55H7N66O5P2S	588.3834	0.1	41.6	282	43.31	88.0	even	ok
	275	C71H42N16NaO37S	588.3833	-0.1	41.7	283	51.84	60.0	even	ok
	276	C61H135N51NaO10	588.3835	0.2	41.7	284	41.74	21.0	even	ok
	277	C70H172N11NaO32PS	588.3836	0.5	41.8	285	46.44	-8.0	even	ok
	278	C78H168N13O24P2S	588.3835	0.2	41.8	286	49.76	4.0	even	ok
	279	C75H130N49O3	588.3837	0.5	41.8	287	38.43	37.0	even	ok
	280	C76H47N12O34P2S	588.3832	-0.2	41.8	288	50.52	62.0	even	ok
	281	C74H5N52O9	588.3839	0.9	41.9	289	35.04	100.0	even	ok
	282	C65H23N46O12P2S2	588.3835	0.2	42.0	290	50.04	80.0	even	ok
	283	C61H152N39O15P2S	588.3837	0.5	42.1	291	45.84	8.0	even	ok
	284	C64H27N42O16P2S2	588.3830	-0.6	42.3	292	45.22	75.0	even	ok
	285	C67H144N47O2P2S2	588.3837	0.6	42.3	293	44.42	22.0	even	ok
	286	C60H149N43NaO11P2S	588.3833	-0.1	42.3	294	51.09	10.0	even	ok
	287	C75H51N8O38P2S	588.3828	-0.9	42.4	295	40.60	57.0	even	ok
	288	C75H31N30O20P2S	588.3837	0.6	42.5	296	44.80	78.0	even	ok
	289	C84H43N10O32P2	588.3835	0.2	42.6	297	49.36	71.0	even	ok
	290	C54H4N70NaOP2S	588.3830	-0.5	42.6	298	37.85	90.0	even	ok
	291	C85H168N7O26P2	588.3833	-0.1	42.7	299	49.81	8.0	even	ok
	292	C52H7N68NaO4PS2	588.3832	-0.3	43.1	300	47.36	85.0	even	ok
	293	C72H176N5O37P2	588.3834	0.2	43.1	301	49.13	-10.0	even	ok
	294	C81H28N28NaO18P2	588.3831	-0.4	43.1	302	45.86	84.0	even	ok
	295	C71H173N9NaO33P2	588.3831	-0.5	43.3	303	45.27	-8.0	even	ok
	296	C78H136N45P2	588.3828	-0.9	43.4	304	33.14	36.0	even	ok
	297	C73H19N44O10P2S	588.3837	0.6	43.5	306	43.72	89.0	even	ok
	298	C77H135N43NaO5	588.3838	0.7	43.6	307	35.06	33.0	even	ok

Compound Spectrum SmartFormula Report

Meas. m/z	#	Ion Formula	m/z	err [ppm]	mSigma	# mSigma	Score	rdb	e⁻ Conf	N-Rule
	299	C74H144N41O4P2S	588.3835	0.2	43.7	308	47.29	26.0	even	ok
	300	C68H160N29O16P2S2	588.3833	-0.1	43.7	309	48.59	6.0	even	ok
	301	C65H28N36NaO23P2	588.3828	-0.9	43.7	310	33.03	72.0	even	ok
	302	C63H31N34NaO26PS	588.3829	-0.7	43.9	311	41.90	67.0	even	ok
	303	C61H150N39O17S2	588.3832	-0.2	44.0	312	47.36	8.0	even	ok
	304	C59H130N57O8	588.3834	0.1	44.1	313	40.56	25.0	even	ok
	305	C60H10N54NaO16	588.3837	0.6	44.1	314	35.84	84.0	even	ok
	306	C78H161N21NaO16P2S	588.3836	0.4	44.2	315	44.71	11.0	even	ok
	307	C53H10N64O8PS2	588.3835	0.3	44.2	316	45.81	83.0	even	ok
	308	C77H43N16O30P2S	588.3837	0.6	44.2	317	42.64	67.0	even	ok
	309	C78H52N6NaO36P2S	588.3833	-0.0	44.2	318	49.05	58.0	even	ok
	310	C55H15N58NaO10PS2	588.3836	0.5	44.3	319	43.94	79.0	even	ok
	311	C61H26N40O24PS	588.3828	-0.8	44.3	320	39.65	71.0	even	ok
	312	C66H32N36NaO18P2S2	588.3831	-0.4	44.3	321	44.58	71.0	even	ok
	313	C76H10N46NaO11	588.3839	1.0	44.4	322	31.21	96.0	even	ok
	314	C77H56N2NaO40P2S	588.3829	-0.8	44.4	323	40.22	53.0	even	ok
	315	C67H28N40NaO14P2S2	588.3836	0.4	44.4	324	44.90	76.0	even	ok
	316	C61H21N46O19S2	588.3839	0.9	44.5	325	38.82	76.0	even	ok
	317	C72H174N5O39S	588.3830	-0.6	44.5	326	42.41	-10.0	even	ok
	318	C69H168N15O31P2	588.3830	-0.6	44.5	327	41.90	-4.0	even	ok
	319	C80H173N7NaO26P2S	588.3836	0.4	44.5	328	44.19	0.0	even	ok
	320	C66H19N50O8P2S2	588.3839	1.0	44.7	329	37.90	85.0	even	ok
	321	C74H55NaO48P	588.3827	-1.1	44.7	330	36.74	49.0	even	ok
	322	C82H153N25NaO12P2	588.3829	-0.7	44.8	331	40.08	21.0	even	ok
	323	C72H45N12O41S	588.3836	0.5	44.8	332	42.67	58.0	even	ok
	324	C64H34N30O30PS	588.3833	-0.1	45.0	333	47.39	65.0	even	ok
	325	C66H39N24NaO32PS	588.3834	0.1	45.0	334	47.24	61.0	even	ok
	326	C69H156N33O12P2S2	588.3837	0.6	45.0	335	41.07	11.0	even	ok
	327	C69H149N41NaO4P2S2	588.3838	0.8	45.1	336	39.28	18.0	even	ok
	328	C63H155N33NaO19S2	588.3833	-0.0	45.1	337	47.66	4.0	even	ok
	329	C80H141N39NaO2P2	588.3829	-0.8	45.2	338	32.94	32.0	even	ok
	330	C67H164N25O20P2S2	588.3828	-0.9	45.2	339	38.11	1.0	even	ok
	331	C77H11N48O6P2	588.3830	-0.6	45.3	340	34.37	99.0	even	ok
	332	C77H36N24NaO22P2S	588.3838	0.7	45.4	341	39.59	74.0	even	ok
	333	C86H48N4NaO34P2	588.3835	0.3	45.4	342	43.81	67.0	even	ok
	334	C87H173NNaO28P2	588.3834	0.0	45.4	343	47.45	4.0	even	ok
	335	C63H23N42O21P2	588.3827	-1.1	45.5	344	29.94	76.0	even	ok
	336	C66H155N27NaO26	588.3839	1.0	45.8	345	30.25	4.0	even	ok
	337	C62H32N36NaO23P2S	588.3839	1.0	45.8	346	36.04	67.0	even	ok
	338	C82H31N24O22P2	588.3835	0.2	45.8	347	45.01	82.0	even	ok
	339	C56H18N54O14PS2	588.3840	1.1	45.9	348	35.36	77.0	even	ok
	340	C70H165N23NaO18P2S2	588.3834	0.0	45.9	349	46.55	2.0	even	ok
	341	C57H17N52O17S2	588.3830	-0.6	46.0	350	39.98	77.0	even	ok
	342	C80H148N31O10P2	588.3828	-0.9	46.0	351	30.79	25.0	even	ok
	343	C63H26N40NaO21S2	588.3840	1.1	46.0	352	35.41	72.0	even	ok
	344	C75H24N38NaO12P2S	588.3838	0.7	46.2	353	38.66	85.0	even	ok

Compound Spectrum SmartFormula Report

Meas. m/z	#	Ion Formula	m/z	err [ppm]	mSigma	# mSigma	Score	rdb	e^- Conf	N-Rule
	345	C58H5N60O14	588.3836	0.4	46.3	354	35.12	88.0	even	ok
	346	C74H50N6NaO43S	588.3837	0.7	46.3	355	39.12	54.0	even	ok
	347	C76H149N35NaO6P2S	588.3836	0.4	46.3	356	42.05	22.0	even	ok
	348	C59H24N46NaO17P2S	588.3835	0.3	46.4	357	43.58	73.0	even	ok
	349	C60H27N42O21P2S	588.3838	0.9	46.5	358	36.98	71.0	even	ok
	350	C68H41N18O39S	588.3828	-1.0	46.6	359	35.33	59.0	even	ok
	351	C67H42N20O36PS	588.3837	0.7	46.6	360	38.57	59.0	even	ok
	352	C73H56N2NaO45P2	588.3837	0.6	46.7	361	39.04	49.0	even	ok
	353	C58H127N61NaO4	588.3830	-0.5	46.7	362	33.42	27.0	even	ok
	354	C59H22N46NaO19S2	588.3831	-0.5	46.7	363	40.79	73.0	even	ok
	355	C80H19N38O12P2	588.3834	0.2	46.8	364	43.88	93.0	even	ok
	356	C58H144N49O9P2S	588.3832	-0.2	46.9	365	35.94	14.0	even	ok
	357	C71H7N58P2S	588.3837	0.6	46.9	366	39.66	100.0	even	ok
	358	C79H48N10NaO32P2S	588.3838	0.7	46.9	367	37.75	63.0	even	ok
	359	C69H169N19NaO22P2S2	588.3829	-0.7	47.0	368	37.84	-3.0	even	ok
	360	C69H47N14NaO38PS	588.3838	0.8	47.0	369	36.49	55.0	even	ok
	361	C83H156N21O16P2	588.3833	-0.1	47.2	370	43.62	19.0	even	ok
	362	C79H16N42NaO8P2	588.3831	-0.4	47.3	371	33.82	95.0	even	ok
	363	C70H46N12NaO41S	588.3828	-0.8	47.3	372	36.21	55.0	even	ok
	364	C70H48N12NaO39P2	588.3833	-0.1	47.3	373	43.74	55.0	even	ok
	365	C71H51N8O43P2	588.3836	0.5	47.4	374	39.88	53.0	even	ok
	366	C57H19N52O15P2S	588.3834	0.1	47.4	375	43.93	77.0	even	ok
	367	C64H150N33O24	588.3838	0.8	47.4	376	30.13	8.0	even	ok
	368	C57H141N53NaO5P2S	588.3828	-0.8	47.6	377	29.93	16.0	even	ok
	369	C71H161N27NaO14P2S2	588.3838	0.8	47.6	378	36.42	7.0	even	ok
	370	C56H16N56NaO11P2S	588.3830	-0.5	47.8	379	39.06	79.0	even	ok
	371	C79H164N17O20P2S	588.3839	1.0	47.9	380	33.99	9.0	even	ok
	372	C68H143N45O5PS2	588.3827	-1.1	47.9	381	33.40	22.0	even	ok
	373	C67H35N32O22P2S2	588.3835	0.2	48.0	382	42.08	69.0	even	ok
	374	C81H144N35O6P2	588.3833	-0.2	48.0	383	35.48	30.0	even	ok
	375	C55H144N51NaO8PS2	588.3830	-0.6	48.0	384	37.69	11.0	even	ok
	376	C64H158N29O23S2	588.3837	0.6	48.0	385	38.23	2.0	even	ok
	377	C79H55N2O40P2S	588.3837	0.6	48.2	386	37.92	56.0	even	ok
	378	C78H7N52O2P2	588.3834	0.2	48.2	387	35.09	104.0	even	ok
	379	C68H43N18O37P2	588.3832	-0.3	48.4	388	40.77	59.0	even	ok
	380	C53H139N57O6PS2	588.3829	-0.8	48.4	389	35.68	15.0	even	ok
	381	C68H31N36O18P2S2	588.3839	1.0	48.4	390	33.91	74.0	even	ok
	382	C84H36N18NaO24P2	588.3835	0.3	48.4	392	40.06	78.0	even	ok
	383	C81H176N3O30P2S	588.3839	1.0	48.5	393	33.31	-2.0	even	ok
	384	C82H24N32NaO14P2	588.3835	0.3	48.7	394	39.79	89.0	even	ok
	385	C63H147N37NaO20	588.3835	0.2	48.8	395	33.98	10.0	even	ok
	386	C67H40N22NaO33P2	588.3828	-0.9	48.8	396	34.22	61.0	even	ok
	387	C66H168N17NaO30PS	588.3828	-1.0	48.9	397	33.05	-7.0	even	ok
	388	C87H51O38P2	588.3839	1.0	48.9	398	33.48	65.0	even	ok
	389	C65H6N62OPS2	588.3829	-0.7	49.0	399	35.46	96.0	even	ok
	390	C58H152N41NaO14PS2	588.3834	0.1	49.1	400	41.33	5.0	even	ok

Compound Spectrum SmartFormula Report

Meas. m/z	#	Ion Formula	m/z	err [ppm]	mSigma	# mSigma	Score	rdb	e⁻ Conf	N-Rule
	391	C56H147N47O12PS2	588.3833	-0.0	49.1	401	42.50	9.0	even	ok
	392	C81H15N42O8P2	588.3839	0.9	49.1	402	33.42	98.0	even	ok
	393	C56H122N67O2	588.3829	-0.7	49.3	403	29.67	31.0	even	ok
	394	C84H152N25O12P2	588.3837	0.6	49.3	404	36.43	24.0	even	ok
	395	C60H25N42O23S2	588.3834	0.1	49.3	405	41.13	71.0	even	ok
	396	C55H136N59O3P2S	588.3828	-1.0	49.3	406	27.19	20.0	even	ok
	397	C66H163N23NaO25S2	588.3838	0.7	49.4	407	35.12	-2.0	even	ok
	398	C73H12N52NaO2P2S	588.3838	0.7	49.5	408	35.16	96.0	even	ok
	399	C77H152N31O10P2S	588.3839	1.0	49.6	409	32.35	20.0	even	ok
	400	C85H161N15NaO18P2	588.3834	0.0	49.7	410	41.81	15.0	even	ok
	401	C71H49N8O45S	588.3832	-0.2	49.8	411	39.48	53.0	even	ok
	402	C71H168N19O22P2S2	588.3837	0.6	49.8	412	35.58	0.0	even	ok
	403	C69H176N7NaO36PS	588.3832	-0.2	49.9	413	39.40	-13.0	even	ok
	404	C54H145N53NaO5P2S2	588.3840	1.1	49.9	414	31.40	11.0	even	ok
	405	C67H171N13O34PS	588.3831	-0.4	49.9	415	37.80	-9.0	even	ok
	406	C67H18N48O11PS2	588.3829	-0.7	50.0	416	34.43	85.0	even	ok
	407	C70H155N31O15PS2	588.3827	-1.1	50.0	417	31.35	11.0	even	ok
	408	C83H149N29NaO8P2	588.3833	0.0	50.1	418	34.48	26.0	even	ok
	409	C69H40N26NaO24P2S2	588.3836	0.4	50.1	419	37.92	65.0	even	ok
	410	C66H39N28O26P2S2	588.3830	-0.6	50.1	420	36.06	64.0	even	ok
	411	C65H35N28O31P2	588.3827	-1.0	50.2	421	31.36	65.0	even	ok
	412	C80H12N46NaO4P2	588.3835	0.3	50.2	422	31.77	100.0	even	ok
	413	C62H30N36NaO25S2	588.3835	0.3	50.3	423	38.29	67.0	even	ok
	414	C70H172N15O26P2S2	588.3833	-0.1	50.5	424	39.81	-5.0	even	ok
	415	C60H154N35O21S2	588.3828	-1.0	50.5	425	31.92	3.0	even	ok
	416	C52H140N59O3P2S2	588.3839	0.9	50.5	426	32.24	15.0	even	ok
	417	C59H155N37O18PS2	588.3838	0.7	50.7	427	33.66	3.0	even	ok
	418	C70H148N39NaO7PS2	588.3828	-0.9	50.7	428	32.10	18.0	even	ok
	419	C61H142N43O18	588.3834	0.1	50.8	429	33.24	14.0	even	ok
	420	C65H169N19NaO27P2S	588.3837	0.7	50.8	430	34.02	-7.0	even	ok
	421	C73H54N2NaO47S	588.3833	-0.1	50.8	431	39.77	49.0	even	ok
	422	C68H167N13NaO36	588.3839	1.0	50.9	432	31.11	-7.0	even	ok
	423	C61H160N31NaO20PS2	588.3839	0.9	51.0	433	31.87	-1.0	even	ok
	424	C78H35N22NaO25PS	588.3828	-1.0	51.1	434	31.22	74.0	even	ok
	425	C62H159N29NaO23S2	588.3829	-0.8	51.1	435	32.75	-1.0	even	ok
	426	C62H161N29NaO21P2S	588.3833	-0.1	51.3	436	39.30	-1.0	even	ok
	427	C86H157N19NaO14P2	588.3838	0.8	51.3	437	32.78	20.0	even	ok
	428	C70H179N3O40PS	588.3836	0.4	51.4	438	36.38	-15.0	even	ok
	429	C63H164N25O25P2S	588.3837	0.5	51.4	439	34.78	-3.0	even	ok
	430	C85H39N14O28P2	588.3839	0.9	51.5	440	30.99	76.0	even	ok
	431	C82H140N39O2P2	588.3837	0.6	51.7	441	28.21	35.0	even	ok
	432	C68H44N22NaO28P2S2	588.3831	-0.4	51.8	442	35.70	60.0	even	ok
	433	C67H11N56NaO3PS2	588.3830	-0.6	51.8	443	33.92	92.0	even	ok
	434	C83H27N28O18P2	588.3839	0.9	52.0	444	30.53	87.0	even	ok
	435	C76H23N36NaO15PS	588.3828	-1.0	52.1	445	30.19	85.0	even	ok
	436	C66H162N19O34	588.3838	0.8	52.1	446	31.31	-3.0	even	ok

Compound Spectrum SmartFormula Report

Meas. m/z	#	Ion Formula	m/z	err [ppm]	mSigma	# mSigma	Score	rdb	e¯ Conf	N-Rule
	437	C62H22N40NaO26	588.3837	0.6	52.2	447	28.16	73.0	even	ok
	438	C73H173N13NaO24P2S2	588.3838	0.8	52.2	448	31.67	-4.0	even	ok
	439	C80H47N8NaO35PS	588.3828	-1.0	52.2	449	30.17	63.0	even	ok
	440	C60H156N35O19P2S	588.3832	-0.2	52.3	450	36.67	3.0	even	ok
	441	C86H164N11O22P2	588.3837	0.6	52.4	451	33.02	13.0	even	ok
	442	C72H177N9NaO28P2S2	588.3834	0.0	52.4	452	38.20	-9.0	even	ok
	443	C51H11N64NaO8PS2	588.3827	-1.1	52.5	453	29.09	80.0	even	ok
	444	C59H153N39NaO15P2S	588.3829	-0.8	52.6	454	30.94	5.0	even	ok
	445	C60H139N47NaO14	588.3830	-0.5	52.6	455	27.97	16.0	even	ok
	446	C72H160N25NaO17PS2	588.3828	-0.9	52.7	456	30.20	7.0	even	ok
	447	C69H23N42NaO13PS2	588.3830	-0.6	52.8	457	32.97	81.0	even	ok
	448	C65H159N23NaO30	588.3835	0.2	52.9	458	35.90	-1.0	even	ok
	449	C54H19N54NaO14PS2	588.3832	-0.3	53.0	459	35.28	74.0	even	ok
	450	C82H14N40O11P	588.3829	-0.8	53.0	460	31.21	98.0	even	ok
	451	C54H11N62O9P2S	588.3829	-0.7	53.1	461	26.55	83.0	even	ok
	452	C69H30N34O21PS2	588.3829	-0.7	53.1	462	31.30	74.0	even	ok
	453	C63H33N32O29S2	588.3839	0.9	53.2	463	29.78	65.0	even	ok
	454	C71H180NO41P2	588.3830	-0.6	53.2	464	32.29	-15.0	even	ok
	455	C52H14N60O12PS2	588.3831	-0.5	53.2	465	33.60	78.0	even	ok
	456	C75H140N45P2S	588.3839	1.0	53.4	466	28.74	31.0	even	ok
	457	C85H144N31NaO7P	588.3828	-0.9	53.5	467	29.29	31.0	even	ok
	458	C63H162N25O27S2	588.3832	-0.2	53.5	468	35.66	-3.0	even	ok
	459	C69H176N11O30P2S2	588.3828	-0.9	53.5	469	29.67	-10.0	even	ok
	460	C80H163N15O23PS	588.3829	-0.7	53.7	470	31.09	9.0	even	ok
	461	C84H145N33NaO4P2	588.3838	0.8	53.7	471	25.34	31.0	even	ok
	462	C65H43N20NaO36PS	588.3829	-0.7	53.8	472	31.14	56.0	even	ok
	463	C57H148N45O13P2S	588.3828	-1.0	53.8	473	28.40	9.0	even	ok
	464	C70H43N22O28P2S2	588.3839	1.0	53.9	474	28.57	63.0	even	ok
	465	C60H17N46O24	588.3836	0.4	53.9	475	27.77	77.0	even	ok
	466	C82H175NO33PS	588.3829	-0.7	54.0	476	30.83	-2.0	even	ok
	467	C63H38N26O34PS	588.3829	-0.8	54.1	477	29.52	60.0	even	ok
	468	C72H167N17O25PS2	588.3827	-1.1	54.1	478	27.62	0.0	even	ok
	469	C55H22N50O18PS2	588.3835	0.3	54.2	479	33.81	72.0	even	ok
	470	C57H27N44NaO20PS2	588.3836	0.5	54.3	480	32.41	68.0	even	ok
	471	C65H167N19NaO29S2	588.3833	-0.0	54.4	481	35.97	-7.0	even	ok
	472	C69H139N49OPS2	588.3832	-0.3	54.5	482	33.48	27.0	even	ok
	473	C65H38N26NaO31S2	588.3840	1.1	54.5	483	27.27	61.0	even	ok
	474	C71H151N35O11PS2	588.3832	-0.3	54.6	484	33.39	16.0	even	ok
	475	C50H12N66NaO5P2S2	588.3837	0.6	54.7	485	30.54	80.0	even	ok
	476	C58H134N53O12	588.3829	-0.7	54.8	486	24.98	20.0	even	ok
	477	C79H38N18O29PS	588.3831	-0.4	55.0	487	32.60	72.0	even	ok
	478	C66H46N16O40PS	588.3833	-0.1	55.0	488	34.99	54.0	even	ok
	479	C88H169N5NaO24P2	588.3838	0.8	55.0	489	29.11	9.0	even	ok
	480	C71H181N5NaO32P2S2	588.3829	-0.7	55.0	490	29.57	-14.0	even	ok
	481	C68H51N10NaO42PS	588.3834	0.1	55.0	491	34.75	50.0	even	ok
	482	C84H19N34NaO13P	588.3830	-0.6	55.0	492	30.52	94.0	even	ok

Compound Spectrum SmartFormula Report

Meas. m/z	#	Ion Formula	m/z	err [ppm]	mSigma	# mSigma	Score	rdb	e⁻ Conf	N-Rule
	483	C69H47N18O32P2S2	588.3835	0.2	55.2	493	33.57	58.0	even	ok
	484	C74H11N50NaO5PS	588.3828	-1.0	55.2	494	27.30	96.0	even	ok
	485	C87H156N17NaO17P	588.3828	-0.9	55.3	495	27.69	20.0	even	ok
	486	C59H14N50NaO20	588.3832	-0.2	55.4	496	27.88	79.0	even	ok
	487	C53H10N60NaO14S	588.3839	0.9	55.4	497	27.39	80.0	even	ok
	488	C78H151N29O13PS	588.3829	-0.7	55.5	498	29.34	20.0	even	ok
	489	C64H44N22NaO33P2S	588.3839	1.0	55.5	499	26.71	56.0	even	ok
	490	C48H7N72O3P2S2	588.3836	0.5	55.5	500	31.03	84.0	even	ok
	491	C61H36N32NaO27P2S	588.3835	0.3	55.6	501	32.70	62.0	even	ok
	492	C68H14N52O7PS2	588.3834	0.0	55.6	502	34.64	90.0	even	ok
	493	C77H26N32O19PS	588.3831	-0.4	55.7	503	31.83	83.0	even	ok
	494	C71H35N28NaO23PS2	588.3830	-0.6	55.7	504	30.06	70.0	even	ok
	495	C59H29N38O27S2	588.3830	-0.6	55.8	505	29.60	66.0	even	ok
	496	C58H30N40O24PS2	588.3840	1.1	55.9	506	25.95	66.0	even	ok
	497	C84H26N26O21P	588.3829	-0.8	56.0	507	28.42	87.0	even	ok
	498	C62H39N28O31P2S	588.3839	0.9	56.0	508	27.48	60.0	even	ok
	499	C54H135N57NaO8S	588.3837	0.6	56.1	509	24.50	17.0	even	ok
	500	C73H180N5O32P2S2	588.3837	0.6	56.1	510	29.15	-11.0	even	ok
603.0590	1	C82H147N33NaO12	603.0596	1.0	26.2	1	80.63	27.0	even	ok
	2	C80H142N39O10	603.0595	0.8	26.3	2	84.17	31.0	even	ok
	3	C81H20N42NaO9P2	603.0585	-0.9	26.3	3	82.57	95.0	even	ok
	4	C82H23N38O13P2	603.0589	-0.3	26.3	4	97.12	93.0	even	ok
	5	C78H14N46NaO12	603.0594	0.5	26.7	5	89.91	96.0	even	ok
	6	C84H28N32NaO15P2	603.0589	-0.1	26.8	6	100.00	89.0	even	ok
	7	C85H160N21O17P2	603.0587	-0.6	26.9	7	88.20	19.0	even	ok
	8	C83H148N35O7P2	603.0587	-0.6	26.9	8	87.92	30.0	even	ok
	9	C85H153N29NaO9P2	603.0588	-0.5	26.9	9	91.68	26.0	even	ok
	10	C76H9N52O10	603.0593	0.4	27.2	10	92.79	100.0	even	ok
	11	C86H156N25O13P2	603.0591	0.1	27.2	11	98.68	24.0	even	ok
	12	C87H165N15NaO19P2	603.0588	-0.4	27.4	12	90.79	15.0	even	ok
	13	C81H158N21O24	603.0591	0.1	27.6	13	99.27	15.0	even	ok
	14	C83H32N28NaO19P2	603.0585	-0.9	27.8	14	79.95	84.0	even	ok
	15	C83H163N15NaO26	603.0592	0.2	27.8	15	95.03	11.0	even	ok
	16	C80H155N25NaO20	603.0587	-0.5	27.9	16	88.29	17.0	even	ok
	17	C70H134N55O2S	603.0593	0.4	27.9	17	89.78	33.0	even	ok
	18	C88H161N19NaO15P2	603.0592	0.3	28.0	18	93.32	20.0	even	ok
	19	C85H31N28O19P2	603.0593	0.5	28.0	19	89.24	87.0	even	ok
	20	C81H151N29NaO16	603.0592	0.2	28.0	20	94.72	22.0	even	ok
	21	C79H30N28NaO26	603.0589	-0.2	28.0	21	95.59	80.0	even	ok
	22	C68H6N62NaO4S	603.0591	0.2	28.1	22	95.75	98.0	even	ok
	23	C78H21N38O20	603.0593	0.4	28.1	23	90.54	89.0	even	ok
	24	C72H139N49NaO4S	603.0594	0.6	28.2	24	85.53	29.0	even	ok
	25	C83H19N42O9P2	603.0593	0.5	28.3	25	88.72	98.0	even	ok
	26	C79H146N35O14	603.0591	0.1	28.3	26	97.80	26.0	even	ok
	27	C80H33N24O30	603.0593	0.4	28.3	27	89.92	78.0	even	ok
	28	C78H150N31O18	603.0586	-0.7	28.4	28	83.57	21.0	even	ok

Compound Spectrum SmartFormula Report

Meas. m/z	#	Ion Formula	m/z	err [ppm]	mSigma	# mSigma	Score	rdb	e⁻ Conf	N-Rule
	29	C77H25N34O24	603.0588	-0.3	28.4	29	91.13	84.0	even	ok
	30	C80H26N32NaO22	603.0594	0.5	28.5	31	86.18	85.0	even	ok
	31	C69H9N58O8S	603.0595	0.8	28.5	32	81.05	96.0	even	ok
	32	C82H16N46NaO5P2	603.0589	-0.1	28.5	33	95.92	100.0	even	ok
	33	C77H18N42NaO16	603.0589	-0.2	28.5	34	94.46	91.0	even	ok
	34	C84H166N11O30	603.0595	0.8	28.7	35	79.33	9.0	even	ok
	35	C82H38N18NaO32	603.0594	0.6	28.7	36	85.50	74.0	even	ok
	36	C82H154N25O20	603.0595	0.8	28.7	37	79.36	20.0	even	ok
	37	C75H152N37O9P2S	603.0584	-1.0	28.8	38	75.89	21.0	even	ok
	38	C78H143N39NaO10	603.0587	-0.5	28.8	39	86.18	28.0	even	ok
	39	C79H139N43NaO6	603.0592	0.2	28.8	40	77.57	33.0	even	ok
	40	C85H24N36NaO11P2	603.0594	0.6	28.9	41	83.96	94.0	even	ok
	41	C75H13N48O14	603.0588	-0.4	28.9	42	89.93	95.0	even	ok
	42	C71H14N52NaO10S	603.0596	0.9	28.9	43	76.68	92.0	even	ok
	43	C72H15N54O5P2S	603.0586	-0.7	28.9	44	82.83	95.0	even	ok
	44	C87H36N22NaO21P2	603.0594	0.6	29.1	46	83.39	83.0	even	ok
	45	C88H168N11O23P2	603.0591	0.1	29.1	47	94.25	13.0	even	ok
	46	C76H22N38NaO20	603.0585	-0.9	29.1	48	75.93	86.0	even	ok
	47	C84H159N19NaO22	603.0596	1.0	29.1	49	75.07	16.0	even	ok
	48	C84H35N24O23P2	603.0589	-0.3	29.2	50	90.89	82.0	even	ok
	49	C73H11N58OP2S	603.0591	0.1	29.3	51	95.20	100.0	even	ok
	50	C77H144N45OP2S	603.0593	0.5	29.3	52	85.22	31.0	even	ok
	51	C85H44N14NaO29P2	603.0585	-0.9	29.3	53	77.18	73.0	even	ok
	52	C71H143N45NaO8S	603.0589	-0.1	29.4	54	93.83	24.0	even	ok
	53	C89H164N15O19P2	603.0596	0.9	29.4	55	76.62	18.0	even	ok
	54	C82H167N11NaO30	603.0587	-0.5	29.5	56	85.05	6.0	even	ok
	55	C77H157N31NaO11P2S	603.0585	-0.8	29.6	57	77.92	17.0	even	ok
	56	C86H171N5NaO32	603.0596	1.0	29.6	58	74.15	5.0	even	ok
	57	C77H134N49O4	603.0591	0.1	29.7	59	79.02	37.0	even	ok
	58	C69H138N51O6S	603.0588	-0.3	29.7	60	89.41	28.0	even	ok
	59	C72H146N41O12S	603.0593	0.5	29.8	61	85.61	22.0	even	ok
	60	C74H20N48NaO7P2S	603.0587	-0.5	29.8	62	84.55	91.0	even	ok
	61	C83H170N7O34	603.0591	0.1	29.9	64	93.88	4.0	even	ok
	62	C80H162N17O28	603.0586	-0.7	29.9	65	80.65	10.0	even	ok
	63	C75H6N56NaO6	603.0589	-0.2	30.0	66	75.74	102.0	even	ok
	64	C75H145N45NaOP2S	603.0585	-0.8	30.1	67	76.89	28.0	even	ok
	65	C86H40N18NaO25P2	603.0589	-0.1	30.1	68	92.48	78.0	even	ok
	66	C74H151N35NaO14S	603.0594	0.6	30.1	69	81.49	18.0	even	ok
	67	C85H175NNaO36	603.0592	0.2	30.1	70	89.80	0.0	even	ok
	68	C75H16N52NaO3P2S	603.0592	0.2	30.2	71	89.59	96.0	even	ok
	69	C77H151N33O15P	603.0596	1.0	30.2	72	72.81	21.0	even	ok
	70	C68H135N55NaO2S	603.0585	-0.9	30.3	73	74.98	30.0	even	ok
	71	C90H173N5NaO25P2	603.0592	0.3	30.3	74	88.08	9.0	even	ok
	72	C80H135N47NaO2	603.0596	1.0	30.3	75	61.05	38.0	even	ok
	73	C86H149N33NaO5P2	603.0592	0.3	30.3	76	88.34	31.0	even	ok
	74	C74H27N40O15P2S	603.0586	-0.6	30.3	77	80.17	84.0	even	ok

Compound Spectrum SmartFormula Report

Meas. m/z	#	Ion Formula	m/z	err [ppm]	mSigma	# mSigma	Score	rdb	e⁻ Conf	N-Rule
	75	C87H152N29O9P2	603.0596	0.9	30.4	78	74.86	29.0	even	ok
	76	C75H6N60PS	603.0585	-0.8	30.5	79	75.84	105.0	even	ok
	77	C79H149N39NaO3P2S	603.0594	0.7	30.5	80	79.35	27.0	even	ok
	78	C80H11N52O3P2	603.0588	-0.3	30.5	81	73.14	104.0	even	ok
	79	C87H172N7O27P2	603.0587	-0.6	30.7	82	80.66	8.0	even	ok
	80	C78H130N53	603.0595	0.8	30.9	83	62.96	42.0	even	ok
	81	C86H47N10O33P2	603.0589	-0.3	30.9	84	87.36	71.0	even	ok
	82	C87H56NaO39P2	603.0585	-0.9	31.1	85	74.08	62.0	even	ok
	83	C76H32N34NaO17P2S	603.0587	-0.5	31.2	87	81.82	80.0	even	ok
	84	C78H160N27O15P2S	603.0589	-0.2	31.3	88	87.75	15.0	even	ok
	85	C76H148N41O5P2S	603.0589	-0.2	31.3	89	87.48	26.0	even	ok
	86	C75H23N44O11P2S	603.0591	0.1	31.4	90	90.24	89.0	even	ok
	87	C70H18N48NaO14S	603.0591	0.2	31.5	91	88.17	87.0	even	ok
	88	C75H23N40NaO17P	603.0595	0.7	31.5	92	76.33	86.0	even	ok
	89	C76H131N53Na	603.0587	-0.5	31.6	93	67.00	39.0	even	ok
	90	C68H13N54O12S	603.0590	0.0	31.7	94	91.13	91.0	even	ok
	91	C81H42N14NaO36	603.0589	-0.2	31.7	95	87.60	69.0	even	ok
	92	C67H10N58NaO8S	603.0587	-0.6	31.7	96	79.31	93.0	even	ok
	93	C76H138N45O8	603.0586	-0.7	31.8	97	63.95	32.0	even	ok
	94	C77H11N54NaO2PS	603.0586	-0.7	32.0	98	76.49	101.0	even	ok
	95	C79H163N19O25P	603.0596	1.0	32.0	99	69.66	10.0	even	ok
	96	C79H37N20O34	603.0588	-0.3	32.0	100	83.66	73.0	even	ok
	97	C89H177NNaO29P2	603.0588	-0.4	32.0	101	81.42	4.0	even	ok
	98	C90H163N13O22P	603.0586	-0.8	32.0	102	74.28	18.0	even	ok
	99	C78H34N24NaO30	603.0585	-0.9	32.1	103	70.82	75.0	even	ok
	100	C77H164N23O19P2S	603.0584	-1.0	32.1	104	70.11	10.0	even	ok
	101	C84H144N39O3P2	603.0591	0.1	32.3	105	72.84	35.0	even	ok
	102	C87H26N30O18P	603.0588	-0.5	32.3	106	80.43	92.0	even	ok
	103	C71H21N44O18S	603.0595	0.8	32.3	107	73.61	85.0	even	ok
	104	C88H52N4NaO35P2	603.0590	-0.1	32.4	108	87.62	67.0	even	ok
	105	C88H151N27O12P	603.0586	-0.8	32.4	109	73.42	29.0	even	ok
	106	C74H10N52NaO10	603.0585	-0.9	32.4	110	58.28	97.0	even	ok
	107	C79H139N47PS	603.0588	-0.4	32.5	111	81.20	36.0	even	ok
	108	C87H43N14O29P2	603.0593	0.5	32.5	112	79.81	76.0	even	ok
	109	C82H45N10O40	603.0593	0.4	32.5	113	81.05	67.0	even	ok
	110	C65H5N64O6S	603.0586	-0.7	32.5	114	74.43	97.0	even	ok
	111	C76H19N48O7P2S	603.0595	0.8	32.6	115	71.90	94.0	even	ok
	112	C80H165N21NaO17P2S	603.0590	-0.1	32.6	116	88.12	11.0	even	ok
	113	C78H153N35NaO7P2S	603.0590	-0.1	32.7	117	87.90	22.0	even	ok
	114	C73H26N38NaO20S	603.0596	0.9	32.8	118	69.63	81.0	even	ok
	115	C79H169N17NaO21P2S	603.0585	-0.8	32.8	119	72.02	6.0	even	ok
	116	C84H50N4NaO42	603.0594	0.6	32.9	120	77.06	63.0	even	ok
	117	C91H176NO29P2	603.0596	0.9	32.9	121	70.15	7.0	even	ok
	118	C77H28N38NaO13P2S	603.0592	0.2	32.9	122	83.65	85.0	even	ok
	119	C73H18N46O15P	603.0594	0.6	33.0	123	76.86	90.0	even	ok
	120	C80H148N37NaO6PS	603.0584	-1.0	33.0	124	67.98	27.0	even	ok

Compound Spectrum SmartFormula Report

Meas. m/z	#	Ion Formula	m/z	err [ppm]	mSigma	# mSigma	Score	rdb	e¯ Conf	N-Rule
	121	C78H160N23NaO21P	603.0593	0.4	33.1	125	79.89	12.0	even	ok
	122	C77H35N30O21P2S	603.0591	0.1	33.3	126	85.99	78.0	even	ok
	123	C90H156N21NaO14P	603.0586	-0.6	33.5	127	74.55	25.0	even	ok
	124	C92H168N7NaO24P	603.0587	-0.6	33.6	128	74.52	14.0	even	ok
	125	C89H31N24NaO20P	603.0588	-0.3	33.8	129	80.73	88.0	even	ok
	126	C89H48N8NaO31P2	603.0594	0.6	33.9	130	73.84	72.0	even	ok
	127	C78H24N42NaO9P2S	603.0596	1.0	34.0	131	66.27	90.0	even	ok
	128	C81H144N41NaO2PS	603.0589	-0.2	34.1	132	81.18	32.0	even	ok
	129	C89H38N16O28P	603.0588	-0.4	34.2	133	76.76	81.0	even	ok
	130	C73H155N31NaO18S	603.0589	-0.1	34.3	134	83.10	13.0	even	ok
	131	C85H14N44O8P	603.0587	-0.5	34.4	135	76.28	103.0	even	ok
	132	C76H148N37NaO11P	603.0593	0.4	34.4	136	64.52	23.0	even	ok
	133	C77H35N26NaO27P	603.0595	0.7	34.5	137	70.74	75.0	even	ok
	134	C70H147N41NaO12S	603.0585	-0.9	34.5	138	67.54	19.0	even	ok
	135	C75H139N47O5P	603.0596	1.0	34.6	139	54.53	32.0	even	ok
	136	C73H11N54NaO7P	603.0595	0.7	34.6	140	58.96	97.0	even	ok
	137	C78H14N50O6PS	603.0590	-0.1	34.6	141	83.57	99.0	even	ok
	138	C71H150N37O16S	603.0589	-0.3	34.6	142	79.34	17.0	even	ok
	139	C79H156N31O11P2S	603.0593	0.5	34.6	143	74.63	20.0	even	ok
	140	C76H155N29O19P	603.0592	0.2	34.6	144	80.13	16.0	even	ok
	141	C89H55O39P2	603.0593	0.5	34.7	145	75.42	65.0	even	ok
	142	C79H40N24NaO23P2S	603.0592	0.3	34.7	146	79.77	74.0	even	ok
	143	C80H172N13O25P2S	603.0589	-0.2	34.9	147	80.24	4.0	even	ok
	144	C63H134N61S2	603.0595	0.8	34.9	148	67.91	29.0	even	ok
	145	C69H148N47O3P2S2	603.0591	0.2	34.9	149	81.42	22.0	even	ok
	146	C77H18N46O10PS	603.0585	-0.8	35.0	150	67.93	94.0	even	ok
	147	C68H16N58NaOP2S2	603.0594	0.6	35.0	151	71.81	92.0	even	ok
	148	C82H174N3O38	603.0586	-0.7	35.0	152	71.09	-1.0	even	ok
	149	C81H168N17O21P2S	603.0593	0.5	35.1	153	73.63	9.0	even	ok
	150	C65H15N60O3P2S2	603.0589	-0.3	35.1	154	78.54	91.0	even	ok
	151	C91H159N17O18P	603.0590	-0.0	35.2	155	83.33	23.0	even	ok
	152	C74H158N27O22S	603.0593	0.5	35.2	156	74.66	11.0	even	ok
	153	C78H31N34O17P2S	603.0595	0.8	35.2	157	67.09	83.0	even	ok
	154	C65H139N55NaO2S2	603.0596	1.0	35.3	158	64.32	25.0	even	ok
	155	C64H6N66O3PS	603.0596	0.9	35.3	159	65.15	97.0	even	ok
	156	C83H12N50NaOP2	603.0594	0.6	35.3	160	59.70	105.0	even	ok
	157	C76H39N26O25P2S	603.0586	-0.6	35.3	161	70.91	73.0	even	ok
	158	C87H19N38NaO10P	603.0588	-0.3	35.3	162	77.52	99.0	even	ok
	159	C76H163N21NaO24S	603.0594	0.6	35.5	163	71.06	7.0	even	ok
	160	C75H30N32O25P	603.0594	0.6	35.7	164	71.47	79.0	even	ok
	161	C81H151N33O10PS	603.0588	-0.4	36.0	165	74.39	25.0	even	ok
	162	C67H20N54NaO5P2S2	603.0590	-0.1	36.0	166	79.86	87.0	even	ok
	163	C91H43N10NaO30P	603.0588	-0.3	36.1	167	76.24	77.0	even	ok
	164	C78H44N20NaO27P2S	603.0587	-0.5	36.1	168	72.45	69.0	even	ok
	165	C82H177N7NaO27P2S	603.0590	-0.1	36.1	169	80.72	0.0	even	ok
	166	C74H143N43O9P	603.0592	0.2	36.1	170	64.29	27.0	even	ok

Compound Spectrum SmartFormula Report

Meas. m/z	#	Ion Formula	m/z	err [ppm]	mSigma	# mSigma	Score	rdb	e¯ Conf	N-Rule
	167	C90H34N20O24P	603.0592	0.3	36.1	171	76.17	86.0	even	ok
	168	C71H6N60O5P	603.0594	0.6	36.2	172	59.08	101.0	even	ok
	169	C71H153N41NaO5P2S2	603.0592	0.3	36.2	173	75.67	18.0	even	ok
	170	C93H171N3O28P	603.0590	-0.0	36.4	174	80.94	12.0	even	ok
	171	C86H139N41O2P	603.0586	-0.8	36.4	175	66.24	40.0	even	ok
	172	C80H19N44NaO8PS	603.0591	0.1	36.4	176	79.83	95.0	even	ok
	173	C81H161N25NaO13P2S	603.0594	0.7	36.4	177	68.20	16.0	even	ok
	174	C88H22N34O14P	603.0592	0.3	36.5	178	75.47	97.0	even	ok
	175	C79H10N54O2PS	603.0594	0.7	36.7	179	68.05	104.0	even	ok
	176	C79H23N40NaO12PS	603.0586	-0.7	36.7	180	67.91	90.0	even	ok
	177	C74H136N51NaOP	603.0593	0.4	36.7	181	60.88	34.0	even	ok
	178	C93H164N11NaO20P	603.0591	0.1	36.8	182	78.15	19.0	even	ok
	179	C82H147N37O6PS	603.0592	0.3	36.8	183	73.85	30.0	even	ok
	180	C83H173N11NaO23P2S	603.0594	0.7	36.9	184	67.30	5.0	even	ok
	181	C88H144N35NaO4P	603.0586	-0.6	37.0	185	68.10	36.0	even	ok
	182	C69H148N43NaO9PS	603.0595	0.8	37.0	186	65.27	19.0	even	ok
	183	C80H36N28NaO19P2S	603.0596	1.0	37.1	187	60.96	79.0	even	ok
	184	C74H27N36NaO21P	603.0590	-0.0	37.2	188	79.26	81.0	even	ok
	185	C61H6N68NaO2S2	603.0594	0.6	37.4	189	68.72	94.0	even	ok
	186	C80H43N20O27P2S	603.0595	0.8	37.4	190	63.30	72.0	even	ok
	187	C69H22N44NaO18S	603.0587	-0.6	37.5	192	68.51	82.0	even	ok
	188	C89H147N31O8P	603.0590	-0.0	37.5	193	78.26	34.0	even	ok
	189	C80H172N9NaO31P	603.0593	0.4	37.5	194	71.19	1.0	even	ok
	190	C72H15N50NaO11P	603.0590	-0.0	37.7	195	65.00	92.0	even	ok
	191	C72H30N34NaO24S	603.0591	0.2	37.8	196	74.85	76.0	even	ok
	192	C83H156N27NaO12PS	603.0589	-0.2	37.9	197	73.69	21.0	even	ok
	193	C70H25N40O22S	603.0590	0.0	37.9	198	77.48	80.0	even	ok
	194	C90H27N28NaO16P	603.0593	0.4	37.9	199	69.83	93.0	even	ok
	195	C92H39N14NaO26P	603.0593	0.4	38.0	200	69.68	82.0	even	ok
	196	C80H46N10NaO40	603.0585	-0.9	38.0	201	60.84	64.0	even	ok
	197	C68H152N43O7P2S2	603.0587	-0.6	38.0	202	66.91	17.0	even	ok
	198	C67H17N50O16S	603.0586	-0.7	38.1	203	64.50	86.0	even	ok
	199	C67H143N49O7PS	603.0594	0.6	38.2	204	66.01	23.0	even	ok
	200	C83H54NaO46	603.0589	-0.2	38.2	205	74.14	58.0	even	ok
	201	C68H23N50O9P2S2	603.0593	0.5	38.3	206	68.61	85.0	even	ok
	202	C79H47N16O31P2S	603.0591	0.1	38.3	207	75.31	67.0	even	ok
	203	C79H176N9O29P2S	603.0584	-1.0	38.3	208	59.76	-1.0	even	ok
	204	C81H49N6O44	603.0588	-0.3	38.4	209	70.92	62.0	even	ok
	205	C81H15N48NaO4PS	603.0595	0.8	38.5	210	62.04	100.0	even	ok
	206	C91H152N25NaO10P	603.0591	0.1	38.6	211	74.50	30.0	even	ok
	207	C84H152N31NaO8PS	603.0593	0.5	38.7	212	67.33	26.0	even	ok
	208	C70H157N37NaO9P2S2	603.0588	-0.4	38.7	213	68.48	13.0	even	ok
	209	C83H180N3O31P2S	603.0593	0.5	38.8	214	66.68	-2.0	even	ok
	210	C78H167N15O29P	603.0592	0.3	38.8	215	71.68	5.0	even	ok
	211	C73H140N47NaO5P	603.0588	-0.3	38.8	216	58.24	29.0	even	ok
	212	C72H22N42O19P	603.0589	-0.2	38.9	217	72.95	85.0	even	ok

Compound Spectrum SmartFormula Report

Meas. m/z	#	Ion Formula	m/z	err [ppm]	mSigma	# mSigma	Score	rdb	e⁻ Conf	N-Rule
	213	C72H156N37O9P2S2	603.0596	0.9	38.9	218	59.87	16.0	even	ok
	214	C73H33N30O28S	603.0595	0.8	39.0	219	61.85	74.0	even	ok
	215	C81H181N3NaO31P2S	603.0585	-0.8	39.0	220	61.46	-5.0	even	ok
	216	C70H3N64NaOP	603.0590	-0.0	39.0	221	62.73	103.0	even	ok
	217	C94H167N7O24P	603.0595	0.7	39.1	222	62.72	17.0	even	ok
	218	C71H143N49O2PS2	603.0586	-0.8	39.2	223	61.73	27.0	even	ok
	219	C79H30N32O20PS	603.0585	-0.8	39.2	224	60.80	83.0	even	ok
	220	C92H46N6O34P	603.0592	0.3	39.3	225	69.84	75.0	even	ok
	221	C75H38N24NaO30S	603.0596	0.9	39.3	226	58.53	70.0	even	ok
	222	C75H152N33NaO15P	603.0588	-0.3	39.4	227	57.43	18.0	even	ok
	223	C82H160N23NaO16PS	603.0584	-1.0	39.4	228	57.58	16.0	even	ok
	224	C66H140N53NaO3PS	603.0590	0.0	39.4	229	74.48	25.0	even	ok
	225	C70H28N44NaO11P2S2	603.0594	0.6	39.6	230	63.49	81.0	even	ok
	226	C81H52N10NaO33P2S	603.0592	0.3	39.6	231	70.03	63.0	even	ok
	227	C81H22N40O12PS	603.0594	0.7	39.6	232	62.74	93.0	even	ok
	228	C70H10N56O9P	603.0589	-0.2	39.7	233	59.30	96.0	even	ok
	229	C85H7N52NaP	603.0588	-0.3	39.8	234	68.65	110.0	even	ok
	230	C92H155N21O14P	603.0595	0.7	39.9	235	61.44	28.0	even	ok
	231	C91H50N2O38P	603.0588	-0.4	40.0	236	66.04	70.0	even	ok
	232	C77H164N19NaO25P	603.0588	-0.3	40.0	237	67.87	7.0	even	ok
	233	C86H10N48O4P	603.0592	0.3	40.1	238	68.79	108.0	even	ok
	234	C80H26N36O16PS	603.0590	-0.1	40.1	239	72.35	88.0	even	ok
	235	C79H47N12NaO37P	603.0595	0.7	40.3	240	60.55	64.0	even	ok
	236	C66H24N50NaO9P2S2	603.0585	-0.9	40.4	241	58.16	82.0	even	ok
	237	C66H18N52O13PS	603.0596	0.9	40.7	242	56.28	86.0	even	ok
	238	C61H135N61NaS2	603.0587	-0.5	40.7	243	63.67	26.0	even	ok
	239	C84H172N9NaO26PS	603.0584	-1.0	40.8	244	55.54	5.0	even	ok
	240	C81H42N18O30PS	603.0585	-0.8	40.9	245	58.20	72.0	even	ok
	241	C71H135N53O3P	603.0587	-0.5	40.9	246	52.80	33.0	even	ok
	242	C71H160N33O13P2S2	603.0591	0.2	40.9	247	69.25	11.0	even	ok
	243	C88H15N42NaO6P	603.0593	0.4	41.0	248	64.41	104.0	even	ok
	244	C96H172NNaO26P	603.0595	0.9	41.0	249	56.96	13.0	even	ok
	245	C64H135N59OPS	603.0589	-0.1	41.0	250	69.65	29.0	even	ok
	246	C64H143N51NaO6S2	603.0592	0.2	41.1	251	67.56	20.0	even	ok
	247	C62H138N57O4S2	603.0591	0.1	41.2	252	69.92	24.0	even	ok
	248	C70H18N52O8PS2	603.0588	-0.4	41.3	253	63.77	90.0	even	ok
	249	C72H159N27NaO22S	603.0585	-0.9	41.3	254	56.49	8.0	even	ok
	250	C77H42N18O35P	603.0594	0.6	41.3	255	61.42	68.0	even	ok
	251	C73H148N43NaO4PS2	603.0587	-0.6	41.3	256	60.82	23.0	even	ok
	252	C84H159N23O16PS	603.0592	0.3	41.3	257	65.19	19.0	even	ok
	253	C73H147N39O13P	603.0587	-0.5	41.4	258	52.19	22.0	even	ok
	254	C81H35N26NaO22PS	603.0586	-0.7	41.4	259	59.85	79.0	even	ok
	255	C94H51NaO36P	603.0593	0.5	41.4	260	63.32	71.0	even	ok
	256	C94H160N15NaO16P	603.0595	0.9	41.4	261	56.42	24.0	even	ok
	257	C65H15N56NaO9PS	603.0592	0.4	41.5	262	64.94	88.0	even	ok
	258	C75H167N17NaO28S	603.0589	-0.1	41.6	263	68.56	2.0	even	ok

Compound Spectrum SmartFormula Report

Meas. m/z	#	Ion Formula	m/z	err [ppm]	mSigma	# mSigma	Score	rdb	e⁻ Conf	N-Rule
	259	C75H159N25O23P	603.0587	-0.5	41.6	264	62.28	11.0	even	ok
	260	C83H27N34NaO14PS	603.0595	0.8	41.7	265	56.68	89.0	even	ok
	261	C73H162N23O26S	603.0589	-0.3	41.8	266	65.56	6.0	even	ok
	262	C73H155N35O12PS2	603.0586	-0.8	42.0	267	57.19	16.0	even	ok
	263	C73H165N27NaO15P2S2	603.0592	0.3	42.1	268	64.52	7.0	even	ok
	264	C82H31N30NaO18PS	603.0591	0.1	42.1	269	68.40	84.0	even	ok
	265	C70H11N60NaPS2	603.0588	-0.3	42.1	270	64.82	97.0	even	ok
	266	C76H39N22NaO31P	603.0590	-0.0	42.1	271	69.38	70.0	even	ok
	267	C95H285N12NaO10P	603.0594	0.5	42.1	272	60.59	-39.0	even	ok
	268	C67H27N46O13P2S2	603.0589	-0.3	42.1	273	65.15	80.0	even	ok
	269	C65H146N47O10S2	603.0595	0.8	42.3	274	55.64	18.0	even	ok
	270	C82H14N46NaO7S	603.0585	-0.8	42.3	275	55.48	100.0	even	ok
	271	C82H55N6O37P2S	603.0595	0.9	42.3	276	55.21	61.0	even	ok
	272	C78H51N12O35P2S	603.0586	-0.6	42.5	277	58.48	62.0	even	ok
	273	C69H7N60NaO5P	603.0586	-0.8	42.6	278	46.76	98.0	even	ok
	274	C67H151N41NaO12S2	603.0596	1.0	42.6	279	52.70	14.0	even	ok
	275	C76H170N13O32S	603.0593	0.5	42.7	280	60.85	0.0	even	ok
	276	C69H32N40NaO15P2S2	603.0590	-0.1	42.9	281	66.35	76.0	even	ok
	277	C63H10N62O7PS	603.0591	0.2	42.9	282	64.93	92.0	even	ok
	278	C95H166N5O27	603.0585	-0.9	42.9	283	52.93	17.0	even	ok
	279	C83H47N12NaO32PS	603.0586	-0.7	43.0	284	57.36	68.0	even	ok
	280	C78H175N7NaO34S	603.0594	0.6	43.0	285	57.91	-4.0	even	ok
	281	C83H163N19O20PS	603.0588	-0.4	43.0	286	61.57	14.0	even	ok
	282	C89H140N39NaP	603.0591	0.1	43.1	287	65.95	41.0	even	ok
	283	C80H56N6NaO37P2S	603.0587	-0.5	43.2	288	59.84	58.0	even	ok
	284	C93H154N19O17	603.0585	-0.9	43.2	289	52.40	28.0	even	ok
	285	C72H23N46NaO10PS2	603.0589	-0.3	43.4	290	62.56	86.0	even	ok
	286	C82H21N38O15S	603.0584	-1.0	43.5	291	51.35	93.0	even	ok
	287	C86H164N17NaO18PS	603.0593	0.5	43.5	292	58.96	15.0	even	ok
	288	C84H142N39O5S	603.0587	-0.6	43.5	293	57.86	35.0	even	ok
	289	C90H143N35O4P	603.0594	0.7	43.5	294	55.74	39.0	even	ok
	290	C71H160N29NaO19PS	603.0595	0.8	43.5	295	54.43	8.0	even	ok
	291	C74H34N28O29P	603.0589	-0.2	43.6	296	64.14	74.0	even	ok
	292	C92H29N22O23	603.0586	-0.6	43.6	297	56.90	91.0	even	ok
	293	C85H175N5O30PS	603.0588	-0.4	44.0	298	59.93	3.0	even	ok
	294	C75H160N29NaO14PS2	603.0587	-0.6	44.0	299	56.47	12.0	even	ok
	295	C81H5N56OS	603.0589	-0.3	44.2	300	61.65	109.0	even	ok
	296	C71H19N46NaO15P	603.0586	-0.8	44.2	301	44.69	87.0	even	ok
	297	C62H7N66NaO3PS	603.0588	-0.4	44.3	302	59.33	94.0	even	ok
	298	C60H10N64NaO6S2	603.0589	-0.2	44.5	303	62.20	89.0	even	ok
	299	C92H148N29NaO6P	603.0595	0.9	44.5	304	51.78	35.0	even	ok
	300	C82H38N22O26PS	603.0590	-0.1	44.5	305	63.98	77.0	even	ok
	301	C69H155N35O17PS	603.0594	0.6	44.5	306	55.27	12.0	even	ok
	302	C74H151N39O8PS2	603.0590	-0.0	44.5	307	64.74	21.0	even	ok
	303	C74H168N23O19P2S2	603.0596	0.9	44.7	308	50.76	5.0	even	ok
	304	C95H159N13NaO19	603.0585	-0.8	44.9	311	52.27	24.0	even	ok

Compound Spectrum SmartFormula Report

Meas. m/z	#	Ion Formula	m/z	err [ppm]	mSigma	# mSigma	Score	rdb	e¯ Conf	N-Rule
	305	C94H41N8O33	603.0587	-0.6	44.9	312	54.83	80.0	even	ok
	306	C73H31N32NaO25P	603.0586	-0.8	44.9	313	52.61	76.0	even	ok
	307	C85H168N13NaO22PS	603.0589	-0.2	45.0	314	60.59	10.0	even	ok
	308	C74H144N47NaPS2	603.0591	0.1	45.0	315	62.16	28.0	even	ok
	309	C58H5N70O4S2	603.0588	-0.3	45.0	316	58.97	93.0	even	ok
	310	C90H17N36O13	603.0586	-0.6	45.0	317	54.51	102.0	even	ok
	311	C71H34N30NaO28S	603.0587	-0.6	45.1	318	55.49	71.0	even	ok
	312	C70H35N36O19P2S2	603.0593	0.5	45.2	319	56.52	74.0	even	ok
	313	C68H152N39NaO13PS	603.0590	0.0	45.2	320	63.29	14.0	even	ok
	314	C71H14N56O4PS2	603.0592	0.3	45.2	321	59.25	95.0	even	ok
	315	C83H54N4O40PS	603.0585	-0.8	45.2	322	51.60	61.0	even	ok
	316	C80H179NO39P	603.0592	0.3	45.2	323	59.75	-6.0	even	ok
	317	C63H18N54NaO12S2	603.0594	0.6	45.3	324	55.12	83.0	even	ok
	318	C61H13N60O10S2	603.0593	0.4	45.3	325	57.39	87.0	even	ok
	319	C72H30N38O18PS2	603.0588	-0.4	45.3	326	56.92	79.0	even	ok
	320	C83H17N42O11S	603.0589	-0.2	45.4	327	59.65	98.0	even	ok
	321	C81H59N2O41P2S	603.0591	0.1	45.4	328	61.68	56.0	even	ok
	322	C94H34N16NaO25	603.0587	-0.5	45.5	329	56.18	87.0	even	ok
	323	C86H147N33NaO7S	603.0588	-0.4	45.5	330	56.93	31.0	even	ok
	324	C69H29N36O26S	603.0586	-0.7	45.6	331	52.38	75.0	even	ok
	325	C84H26N32NaO17S	603.0585	-0.8	45.7	332	50.43	89.0	even	ok
	326	C74H42N20NaO34S	603.0591	0.2	45.8	333	59.76	65.0	even	ok
	327	C70H164N29O17P2S2	603.0587	-0.6	45.8	334	53.91	6.0	even	ok
	328	C83H34N26O22PS	603.0594	0.7	45.9	335	52.54	82.0	even	ok
	329	C79H176N5NaO35P	603.0588	-0.3	45.9	336	57.57	-4.0	even	ok
	330	C72H37N26O32S	603.0590	0.0	45.9	337	61.93	69.0	even	ok
	331	C86H154N25O15S	603.0587	-0.6	46.0	338	53.95	24.0	even	ok
	332	C83H10N50NaO3S	603.0590	-0.1	46.1	340	60.67	105.0	even	ok
	333	C91H142N33O7	603.0585	-0.9	46.1	341	48.15	39.0	even	ok
	334	C72H40N30NaO21P2S2	603.0594	0.6	46.3	342	52.44	70.0	even	ok
	335	C84H50N8O36PS	603.0590	-0.1	46.3	343	60.78	66.0	even	ok
	336	C69H14N52O13P	603.0585	-0.9	46.4	344	40.15	91.0	even	ok
	337	C72H169N23NaO19P2S2	603.0588	-0.4	46.4	345	55.24	2.0	even	ok
	338	C93H273N26NaP	603.0593	0.5	46.5	346	53.52	-28.0	even	ok
	339	C92H22N30NaO15	603.0587	-0.5	46.5	347	54.41	98.0	even	ok
	340	C66H147N45O11PS	603.0590	-0.1	46.5	348	59.61	18.0	even	ok
	341	C71H26N38O23P	603.0585	-0.9	46.7	349	47.79	80.0	even	ok
	342	C73H26N42O14PS2	603.0592	0.3	46.8	350	56.51	84.0	even	ok
	343	C76H156N33NaO10PS2	603.0591	0.1	46.9	351	58.70	17.0	even	ok
	344	C84H43N16NaO28PS	603.0591	0.1	46.9	352	59.41	73.0	even	ok
	345	C97H287N6O17	603.0587	-0.5	46.9	353	53.09	-41.0	even	ok
	346	C96H46N2NaO35	603.0587	-0.5	47.1	354	53.63	76.0	even	ok
	347	C75H45N16O38S	603.0595	0.8	47.1	355	48.98	63.0	even	ok
	348	C96H162N9O23	603.0589	-0.2	47.2	356	57.33	22.0	even	ok
	349	C77H171N11O33P	603.0587	-0.5	47.3	357	53.07	0.0	even	ok
	350	C74H35N32NaO20PS2	603.0589	-0.3	47.3	358	55.99	75.0	even	ok

Compound Spectrum SmartFormula Report

Meas. m/z	#	Ion Formula	m/z	err [ppm]	mSigma	# mSigma	Score	rdb	e⁻ Conf	N-Rule
	351	C75H167N21O22PS2	603.0586	-0.8	47.3	359	49.29	5.0	even	ok
	352	C68H8N62NaO2P2	603.0596	0.9	47.3	360	39.52	98.0	even	ok
	353	C93H147N27NaO9	603.0585	-0.8	47.3	361	48.57	35.0	even	ok
	354	C76H163N25O18PS2	603.0590	-0.0	47.4	362	59.66	10.0	even	ok
	355	C77H50N10NaO40S	603.0596	0.9	47.5	363	46.37	59.0	even	ok
	356	C85H22N36NaO13S	603.0590	-0.1	47.6	364	58.14	94.0	even	ok
	357	C73H19N50NaO6PS2	603.0593	0.5	47.6	365	53.00	91.0	even	ok
	358	C65H144N49NaO7PS	603.0586	-0.7	47.7	366	49.21	20.0	even	ok
	359	C85H138N43OS	603.0591	0.2	47.8	367	56.68	40.0	even	ok
	360	C68H30N38O23PS	603.0596	0.9	48.0	368	45.59	75.0	even	ok
	361	C85H39N20NaO24PS	603.0595	0.8	48.0	369	47.15	78.0	even	ok
	362	C88H159N19NaO17S	603.0588	-0.4	48.2	370	52.61	20.0	even	ok
	363	C67H27N42NaO19PS	603.0592	0.4	48.2	371	53.32	77.0	even	ok
	364	C95H37N12O29	603.0591	0.1	48.3	372	56.58	85.0	even	ok
	365	C93H25N26O19	603.0591	0.1	48.5	373	56.29	96.0	even	ok
	366	C87H150N29O11S	603.0591	0.2	48.6	374	55.35	29.0	even	ok
	367	C73H172N19O23P2S2	603.0591	0.2	48.6	375	55.41	0.0	even	ok
	368	C99H292NaO19	603.0588	-0.4	48.6	376	52.59	-45.0	even	ok
	369	C86H55N2NaO38PS	603.0591	0.1	48.6	377	56.37	62.0	even	ok
	370	C68H36N36NaO19P2S2	603.0585	-0.9	48.7	378	45.91	71.0	even	ok
	371	C79H54N4O45P	603.0594	0.6	48.7	379	49.56	57.0	even	ok
	372	C88H5N50O3	603.0586	-0.6	48.7	380	48.81	113.0	even	ok
	373	C94H150N23O13	603.0589	-0.2	48.8	381	54.56	33.0	even	ok
	374	C63H147N47NaO10S2	603.0587	-0.5	48.8	382	50.49	15.0	even	ok
	375	C84H13N46O7S	603.0593	0.5	48.8	383	50.58	103.0	even	ok
	376	C71H145N45NaO6P2	603.0594	0.6	48.8	384	41.27	24.0	even	ok
	377	C86H171N9O26PS	603.0592	0.4	48.9	385	52.32	8.0	even	ok
	378	C75H147N43O4PS2	603.0595	0.7	48.9	386	47.33	26.0	even	ok
	379	C70H20N48NaO12P2	603.0596	0.9	49.0	387	37.55	87.0	even	ok
	380	C78H51N8NaO41P	603.0590	-0.0	49.0	388	56.90	59.0	even	ok
	381	C77H172N15NaO24PS2	603.0587	-0.6	49.0	389	48.81	1.0	even	ok
	382	C72H32N34NaO22P2	603.0596	0.9	49.1	390	44.83	76.0	even	ok
	383	C75H31N36NaO16PS2	603.0593	0.5	49.1	391	50.62	80.0	even	ok
	384	C98H167N3NaO25	603.0590	-0.0	49.1	392	56.23	18.0	even	ok
	385	C61H142N53O8S2	603.0586	-0.7	49.3	393	47.67	19.0	even	ok
	386	C63H139N55O5PS	603.0585	-0.9	49.4	394	44.77	24.0	even	ok
	387	C65H22N48O17PS	603.0591	0.2	49.4	395	53.54	81.0	even	ok
	388	C85H29N28O21S	603.0589	-0.2	49.4	396	52.98	87.0	even	ok
	389	C74H171N13NaO32S	603.0585	-0.9	49.5	397	44.61	-3.0	even	ok
	390	C66H155N37NaO16S2	603.0592	0.2	49.5	398	52.84	9.0	even	ok
	391	C75H177N13NaO25P2S2	603.0592	0.3	49.6	399	51.74	-4.0	even	ok
	392	C84H33N24O25S	603.0584	-1.0	49.6	400	42.96	82.0	even	ok
	393	C64H150N43O14S2	603.0591	0.1	49.6	401	54.75	13.0	even	ok
	394	C78H168N19NaO20PS2	603.0591	0.1	49.6	402	54.05	6.0	even	ok
	395	C73H9N58O3S2	603.0587	-0.6	49.7	403	47.57	100.0	even	ok
	396	C87H143N37NaO3S	603.0592	0.3	49.7	404	51.44	36.0	even	ok

Compound Spectrum SmartFormula Report

Meas. m/z	#	Ion Formula	m/z	err [ppm]	mSigma	# mSigma	Score	rdb	e¯ Conf	N-Rule
	397	C66H3N68P2	603.0595	0.7	49.8	405	38.38	102.0	even	ok
	398	C90H10N44NaO5	603.0587	-0.5	49.8	406	49.25	109.0	even	ok
	399	C95H275N20O7	603.0587	-0.5	49.8	407	48.60	-30.0	even	ok
	400	C77H179N3NaO38S	603.0590	-0.1	50.1	408	53.54	-9.0	even	ok
	401	C76H46N14O39P	603.0589	-0.2	50.2	409	52.82	63.0	even	ok
	402	C75H174N9O36S	603.0589	-0.3	50.2	410	51.27	-5.0	even	ok
	403	C64H19N52NaO13PS	603.0588	-0.4	50.3	411	49.77	83.0	even	ok
	404	C95H30N20NaO21	603.0592	0.3	50.3	412	51.29	92.0	even	ok
	405	C85H46N12O32PS	603.0594	0.7	50.3	413	45.92	71.0	even	ok
	406	C96H155N17NaO15	603.0590	-0.1	50.3	414	54.08	29.0	even	ok
	407	C77H159N29O14PS2	603.0595	0.7	50.4	415	45.19	15.0	even	ok
	408	C97H42N6NaO31	603.0592	0.3	50.4	416	51.04	81.0	even	ok
	409	C69H39N32O23P2S2	603.0589	-0.3	50.5	417	51.11	69.0	even	ok
	410	C75H38N28O24PS2	603.0592	0.3	50.7	418	50.14	73.0	even	ok
	411	C89H155N23NaO13S	603.0592	0.3	50.8	419	49.75	25.0	even	ok
	412	C67H158N33O20S2	603.0595	0.8	50.8	420	43.18	7.0	even	ok
	413	C86H18N40NaO9S	603.0594	0.6	50.9	421	45.47	99.0	even	ok
	414	C91H13N40O9	603.0591	0.1	51.0	422	52.28	107.0	even	ok
	415	C70H27N40O20P2	603.0595	0.7	51.0	423	44.23	80.0	even	ok
	416	C75H43N18NaO35P	603.0586	-0.8	51.0	424	43.95	65.0	even	ok
	417	C86H25N32O17S	603.0593	0.5	51.0	425	47.22	92.0	even	ok
	418	C88H176N3NaO28PS	603.0593	0.5	51.0	426	47.04	4.0	even	ok
	419	C77H152N37NaO6PS2	603.0596	0.9	51.0	427	42.39	22.0	even	ok
	420	C71H44N26NaO25P2S2	603.0590	-0.1	51.1	428	52.12	65.0	even	ok
	421	C69H163N27NaO22S2	603.0596	1.0	51.1	429	40.91	3.0	even	ok
	422	C97H280N14NaO9	603.0588	-0.4	51.1	430	48.66	-34.0	even	ok
	423	C68H15N54O10P2	603.0595	0.7	51.3	431	36.59	91.0	even	ok
	424	C88H166N11O25S	603.0587	-0.6	51.3	432	46.07	13.0	even	ok
	425	C69H140N51O4P2	603.0593	0.4	51.3	433	39.94	28.0	even	ok
	426	C74H42N24O28PS2	603.0588	-0.4	51.4	434	47.55	68.0	even	ok
	427	C77H142N45O3S2	603.0589	-0.2	51.5	435	50.42	31.0	even	ok
	428	C73H172N15NaO29PS	603.0595	0.8	51.5	436	42.86	-3.0	even	ok
	429	C64H145N51NaO4P2S	603.0596	0.9	51.7	437	40.67	20.0	even	ok
	430	C62H14N58O11PS	603.0587	-0.5	51.7	438	45.60	87.0	even	ok
	431	C59H140N59NaOPS2	603.0593	0.4	51.8	439	47.23	21.0	even	ok
	432	C87H34N22NaO23S	603.0590	-0.1	51.8	440	51.24	83.0	even	ok
	433	C73H157N31NaO16P2	603.0594	0.6	51.8	441	37.64	13.0	even	ok
	434	C86H38N18NaO27S	603.0585	-0.8	51.9	442	41.91	78.0	even	ok
	435	C89H162N15O21S	603.0591	0.2	51.9	443	49.84	18.0	even	ok
	436	C75H14N52NaO5S2	603.0587	-0.5	52.0	444	46.24	96.0	even	ok
	437	C87H19N42O4P2S	603.0585	-0.9	52.0	445	40.49	102.0	even	ok
	438	C76H10N56NaOS2	603.0592	0.3	52.1	446	48.42	101.0	even	ok
	439	C76H180N9O29P2S2	603.0596	0.9	52.2	447	40.54	-6.0	even	ok
	440	C97H158N13O19	603.0593	0.5	52.3	448	44.82	27.0	even	ok
	441	C71H167N21O27PS	603.0594	0.6	52.3	449	43.67	1.0	even	ok
	442	C93H18N34NaO11	603.0592	0.3	52.4	450	48.13	103.0	even	ok

Compound Spectrum SmartFormula Report

Meas. m/z	#	Ion Formula	m/z	err [ppm]	mSigma	# mSigma	Score	rdb	e⁻ Conf	N-Rule
	443	C78H175N11O28PS2	603.0590	-0.0	52.4	451	51.32	-1.0	even	ok
	444	C92H138N37O3	603.0589	-0.2	52.4	452	48.76	44.0	even	ok
	445	C88H146N33O7S	603.0596	0.9	52.4	453	40.16	34.0	even	ok
	446	C76H146N41O7S2	603.0585	-0.9	52.5	454	39.91	26.0	even	ok
	447	C75H169N17NaO26P2	603.0594	0.6	52.5	455	44.13	2.0	even	ok
	448	C73H38N24O33P	603.0585	-0.9	52.5	456	40.10	69.0	even	ok
	449	C70H164N25NaO23PS	603.0590	0.0	52.5	457	50.63	3.0	even	ok
	450	C98H283N10O13	603.0592	0.2	52.6	458	48.27	-36.0	even	ok
	451	C96H33N16O25	603.0595	0.9	52.7	459	40.43	90.0	even	ok
	452	C77H43N22NaO26PS2	603.0593	0.5	52.8	460	45.03	69.0	even	ok
	453	C79H164N23NaO16PS2	603.0596	0.9	52.9	461	39.98	11.0	even	ok
	454	C87H51N6NaO34PS	603.0595	0.8	52.9	462	40.58	67.0	even	ok
	455	C59H14N60NaO10S2	603.0585	-0.9	53.0	463	39.29	84.0	even	ok
	456	C89H31N28O14P2S	603.0585	-0.9	53.1	464	39.24	91.0	even	ok
	457	C76H47N18NaO30PS2	603.0589	-0.3	53.1	465	46.93	64.0	even	ok
	458	C62H22N50NaO16S2	603.0589	-0.2	53.1	466	48.02	78.0	even	ok
	459	C79H171N15O24PS2	603.0595	0.7	53.2	467	41.33	4.0	even	ok
	460	C72H47N22O29P2S2	603.0593	0.5	53.3	468	44.07	63.0	even	ok
	461	C88H30N26NaO19S	603.0594	0.7	53.4	469	42.08	88.0	even	ok
	462	C60H17N56O14S2	603.0588	-0.3	53.5	470	45.62	82.0	even	ok
	463	C98H45N2O35	603.0595	0.9	53.5	471	39.32	79.0	even	ok
	464	C94H143N31NaO5	603.0590	-0.1	53.6	472	48.83	40.0	even	ok
	465	C90H171N5NaO27S	603.0588	-0.4	53.6	473	44.60	9.0	even	ok
	466	C68H159N31O21PS	603.0590	-0.1	53.7	474	48.02	7.0	even	ok
	467	C73H46N16NaO38S	603.0587	-0.5	53.8	475	42.68	60.0	even	ok
	468	C79H147N39NaO5S2	603.0590	-0.0	53.8	476	48.64	27.0	even	ok
	469	C71H152N37O14P2	603.0593	0.4	54.0	477	36.57	17.0	even	ok
	470	C89H24N36NaO6P2S	603.0586	-0.8	54.1	478	39.75	98.0	even	ok
	471	C94H21N30O15	603.0595	0.9	54.1	479	38.81	101.0	even	ok
	472	C65H30N40NaO22S2	603.0594	0.6	54.1	480	42.04	72.0	even	ok
	473	C99H163N7NaO21	603.0594	0.7	54.1	481	40.56	23.0	even	ok
	474	C63H25N46O20S2	603.0593	0.4	54.1	482	43.81	76.0	even	ok
	475	C67H12N58NaO6P2	603.0591	0.1	54.1	483	39.14	93.0	even	ok
	476	C77H179N7O32PS2	603.0586	-0.7	54.2	484	39.96	-6.0	even	ok
	477	C90H158N19O17S	603.0596	0.9	54.2	485	37.96	23.0	even	ok
	478	C100H288N4NaO15	603.0592	0.4	54.2	486	44.12	-40.0	even	ok
	479	C71H41N22O36S	603.0586	-0.7	54.2	487	40.37	64.0	even	ok
	480	C74H52N16NaO31P2S2	603.0594	0.6	54.3	488	40.98	59.0	even	ok
	481	C73H164N23O24P2	603.0593	0.4	54.3	489	43.43	6.0	even	ok
	482	C91H167N9NaO23S	603.0592	0.3	54.3	490	44.44	14.0	even	ok
	483	C67H156N35NaO17PS	603.0586	-0.7	54.4	491	40.18	9.0	even	ok
	484	C80H180N5NaO30PS2	603.0591	0.1	54.4	492	46.52	-5.0	even	ok
	485	C68H137N55NaP2	603.0589	-0.2	54.5	493	38.33	30.0	even	ok
	486	C72H176N15O27P2S2	603.0587	-0.6	54.6	494	41.29	-5.0	even	ok
	487	C80H143N43NaOS2	603.0594	0.7	54.7	495	39.78	32.0	even	ok
	488	C76H54N6NaO44S	603.0591	0.2	54.7	496	45.40	54.0	even	ok

Compound Spectrum SmartFormula Report

Meas. m/z	#	Ion Formula	m/z	err [ppm]	mSigma	# mSigma	Score	rdb	e¯ Conf	N-Rule
	489	C78H151N35NaO9S2	603.0586	-0.8	54.7	497	38.86	22.0	even	ok
	490	C74H49N12O42S	603.0591	0.0	54.8	498	47.10	58.0	even	ok
	491	C74H44N20NaO32P2	603.0596	0.9	54.8	499	37.43	65.0	even	ok
	492	C95H146N27O9	603.0593	0.5	54.8	500	41.48	38.0	even	ok
	493	C74H181N9NaO29P2S2	603.0588	-0.4	55.1	501	42.35	-9.0	even	ok
	494	C78H158N27O17S2	603.0585	-0.9	55.4	502	36.48	15.0	even	ok
	495	C91H36N22NaO16P2S	603.0586	-0.8	55.4	503	38.17	87.0	even	ok
	496	C58H15N62NaO7PS2	603.0595	0.7	55.5	504	38.45	84.0	even	ok
	497	C86H45N10O35S	603.0584	-1.0	55.5	505	35.75	71.0	even	ok
	498	C81H176N9NaO26PS2	603.0596	0.9	55.5	506	36.65	0.0	even	ok
	499	C79H184NNaO34PS2	603.0587	-0.6	55.7	507	39.70	-10.0	even	ok
	500	C88H37N18O27S	603.0593	0.5	55.8	508	40.59	81.0	even	ok
603.3937	1	C31H48N33O58	603.3935	-0.4	3.0	1	91.78	26.0	even	ok
	2	C20H24N67O36S	603.3937	-0.1	3.5	2	100.00	44.0	even	ok
	3	C19H21N71NaO32S	603.3933	-0.7	3.6	3	85.58	46.0	even	ok
	4	C33H53N27NaO60	603.3936	-0.3	4.7	5	92.94	22.0	even	ok
	5	C17H16N77O30S	603.3933	-0.8	5.0	6	79.92	50.0	even	ok
	6	C22H29N61NaO38S	603.3938	0.1	5.2	7	96.60	40.0	even	ok
	7	C11H5N95NaO16S2	603.3940	0.5	5.9	8	86.88	58.0	even	ok
	8	C29H46N39NaO51P	603.3941	0.6	6.9	9	81.45	28.0	even	ok
	9	C34H56N23O64	603.3939	0.3	8.1	10	86.75	20.0	even	ok
	10	C23H32N57O42S	603.3942	0.7	8.5	11	78.06	38.0	even	ok
	11	C16H17N79O27PS	603.3943	0.8	8.8	12	74.07	50.0	even	ok
	12	C27H41N45O49P	603.3940	0.5	9.1	13	81.64	32.0	even	ok
	13	C8H4N97O18S2	603.3935	-0.4	9.9	14	81.24	57.0	even	ok
	14	C10H9N91NaO20S2	603.3936	-0.3	9.9	15	84.54	53.0	even	ok
	15	C36H61N17NaO66	603.3940	0.5	10.3	16	80.03	16.0	even	ok
	16	C21H33N57NaO42S	603.3934	-0.7	10.5	17	75.74	35.0	even	ok
	17	C19H28N63O40S	603.3933	-0.8	10.6	18	72.39	39.0	even	ok
	18	C25H37N51NaO44S	603.3942	0.8	10.7	19	71.70	34.0	even	ok
	19	C16H23N77NaO23P2S2	603.3932	-1.0	10.7	20	69.10	46.0	even	ok
	20	C32H44N37O54	603.3939	0.3	10.7	21	82.78	31.0	even	ok
	21	C29H36N47O48	603.3935	-0.4	11.0	22	79.54	37.0	even	ok
	22	C31H41N41NaO50	603.3936	-0.3	11.1	23	82.67	33.0	even	ok
	23	C14H11N91NaO13P2S2	603.3932	-1.0	11.5	24	67.89	57.0	even	ok
	24	C21H20N71O32S	603.3941	0.7	11.8	25	73.52	49.0	even	ok
	25	C18H12N81O26S	603.3937	-0.1	11.9	26	85.91	55.0	even	ok
	26	C34H49N31NaO56	603.3940	0.5	12.0	27	77.53	27.0	even	ok
	27	C20H17N75NaO28S	603.3938	0.1	12.1	28	85.37	51.0	even	ok
	28	C15H14N83NaO23PS	603.3939	0.3	12.2	29	81.61	52.0	even	ok
	29	C26H38N53O39P2S	603.3933	-0.7	12.2	30	71.50	37.0	even	ok
	30	C11H12N87O24S2	603.3939	0.3	12.3	31	80.35	51.0	even	ok
	31	C26H38N49NaO45P	603.3937	-0.1	12.4	32	84.06	34.0	even	ok
	32	C22H36N53O46S	603.3937	-0.1	12.8	33	84.70	33.0	even	ok
	33	C31H58N25NaO61P	603.3941	0.6	13.0	35	72.61	17.0	even	ok
	34	C18H29N65O37PS	603.3943	0.8	13.0	36	68.26	39.0	even	ok

Compound Spectrum SmartFormula Report

Meas. m/z	#	Ion Formula	m/z	err [ppm]	mSigma	# mSigma	Score	rdb	e⁻ Conf	N-Rule
	35	C23H25N65NaO34S	603.3942	0.8	13.1	37	68.61	45.0	even	ok
	36	C15H14N87O17P2S2	603.3935	-0.4	13.2	38	77.68	55.0	even	ok
	37	C35H65N13NaO70	603.3936	-0.3	13.2	39	95.59	11.0	even	ok
	38	C33H60N19O68	603.3935	-0.4	13.3	40	76.31	15.0	even	ok
	39	C13H17N81NaO26S2	603.3940	0.5	13.4	41	75.56	47.0	even	ok
	40	C39H67N13NaO63P2	603.3932	-1.0	13.5	42	65.60	15.0	even	ok
	41	C24H41N47NaO48S	603.3938	0.1	13.7	43	82.45	29.0	even	ok
	42	C17H26N69NaO33PS	603.3939	0.3	13.7	44	79.14	41.0	even	ok
	43	C29H53N31O59P	603.3940	0.5	13.9	45	74.44	21.0	even	ok
	44	C17H9N85NaO22S	603.3933	-0.7	13.9	46	70.73	57.0	even	ok
	45	C28H50N39O49P2S	603.3933	-0.7	14.1	47	69.02	26.0	even	ok
	46	C28H43N47NaO41P2S	603.3934	-0.6	14.3	48	71.71	33.0	even	ok
	47	C27H34N53NaO41P	603.3941	0.6	14.3	49	70.92	39.0	even	ok
	48	C17H26N73O27P2S2	603.3935	-0.4	14.3	50	76.02	44.0	even	ok
	49	C13H9N89O21PS	603.3938	0.1	14.5	51	81.21	56.0	even	ok
	50	C28H50N35NaO55P	603.3937	-0.1	14.5	52	80.82	23.0	even	ok
	51	C15H4N91O20S	603.3933	-0.8	14.7	53	66.62	61.0	even	ok
	52	C24H33N55O43P	603.3936	-0.3	14.7	54	77.32	38.0	even	ok
	53	C16H10N87NaO19PS	603.3943	1.0	14.8	55	63.18	57.0	even	ok
	54	C30H55N33NaO51P2S	603.3934	-0.6	15.0	56	84.95	22.0	even	ok
	55	C40H70N9O67P2	603.3935	-0.4	15.1	57	90.01	13.0	even	ok
	56	C17H19N81NaO19P2S2	603.3936	-0.2	15.2	58	77.68	51.0	even	ok
	57	C15H21N75O31PS	603.3938	0.1	15.4	59	79.61	45.0	even	ok
	58	C25H44N43O52S	603.3942	0.7	15.6	60	81.55	27.0	even	ok
	59	C25H29N59O39P	603.3940	0.5	15.9	61	71.74	43.0	even	ok
	60	C36H68N9O74	603.3939	0.3	15.9	62	89.56	9.0	even	ok
	61	C26H45N41O53P	603.3936	-0.3	16.2	63	75.23	27.0	even	ok
	62	C14H5N93O17PS	603.3942	0.8	16.2	64	64.27	61.0	even	ok
	63	C19H31N67NaO29P2S2	603.3936	-0.2	16.5	65	75.73	40.0	even	ok
	64	C27H49N37NaO54S	603.3942	0.8	16.9	66	75.97	23.0	even	ok
	65	C29H46N43O45P2S	603.3937	0.0	17.0	67	78.87	31.0	even	ok
	66	C42H75N3NaO69P2	603.3936	-0.2	17.1	68	89.83	9.0	even	ok
	67	C38H73N3NaO76	603.3940	0.5	17.1	69	83.83	5.0	even	ok
	68	C14H18N79NaO27PS	603.3934	-0.5	17.3	70	69.33	47.0	even	ok
	69	C18H22N77O23P2S2	603.3940	0.4	17.8	71	70.80	49.0	even	ok
	70	C31H51N37NaO47P2S	603.3938	0.2	17.8	72	89.69	27.0	even	ok
	71	C18H35N63NaO33P2S2	603.3932	-1.0	17.9	73	72.06	35.0	even	ok
	72	C25H42N45NaO49P	603.3932	-0.9	18.0	74	61.62	29.0	even	ok
	73	C12H6N93NaO17PS	603.3934	-0.5	18.0	75	68.16	58.0	even	ok
	74	C23H30N59NaO39P	603.3932	-0.9	18.3	76	61.18	40.0	even	ok
	75	C31H58N29O55P2S	603.3938	0.0	18.4	77	91.87	20.0	even	ok
	76	C20H34N63O33P2S2	603.3940	0.4	18.6	78	83.38	38.0	even	ok
	77	C12H13N85O25PS	603.3934	-0.6	19.3	79	63.69	51.0	even	ok
	78	C24H26N63NaO35P	603.3937	-0.1	19.4	80	72.88	45.0	even	ok
	79	C24H26N67O29P2S	603.3933	-0.7	19.4	81	61.71	48.0	even	ok
	80	C37H55N27NaO53P2	603.3932	-1.0	19.6	82	57.84	26.0	even	ok

Compound Spectrum SmartFormula Report

Meas. m/z	#	Ion Formula	m/z	err [ppm]	mSigma	# mSigma	Score	rdb	e⁻ Conf	N-Rule
	81	C20H27N71NaO25P2S2	603.3941	0.5	20.0	84	64.78	45.0	even	ok
	82	C33H63N23NaO57P2S	603.3938	0.2	20.3	86	84.83	16.0	even	ok
	83	C38H58N23O57P2	603.3935	-0.4	20.4	87	67.05	24.0	even	ok
	84	C32H54N33O51P2S	603.3942	0.8	20.6	89	72.19	25.0	even	ok
	85	C22H39N57NaO35P2S2	603.3941	0.5	20.6	90	76.68	34.0	even	ok
	86	C26H31N61NaO31P2S	603.3934	-0.6	20.6	91	62.90	44.0	even	ok
	87	C19H38N59O37P2S2	603.3935	-0.4	20.9	92	79.70	33.0	even	ok
	88	C30H32N51O44	603.3939	0.3	21.0	93	67.33	42.0	even	ok
	89	C30H62N25O59P2S	603.3933	-0.7	21.2	94	71.75	15.0	even	ok
	90	C10H16N83O28S2	603.3935	-0.4	21.2	95	64.79	46.0	even	ok
	91	C22H21N69O33P	603.3936	-0.3	21.2	96	67.43	49.0	even	ok
	92	C27H34N57O35P2S	603.3937	-0.0	21.4	97	71.98	42.0	even	ok
	93	C12H21N77NaO30S2	603.3936	-0.3	21.5	98	67.07	42.0	even	ok
	94	C32H37N45NaO46	603.3940	0.5	21.5	99	63.99	38.0	even	ok
	95	C15H7N95NaO9P2S2	603.3936	-0.2	21.6	100	67.80	62.0	even	ok
	96	C8H14N89NaO21PS2	603.3941	0.6	21.7	101	60.71	48.0	even	ok
	97	C41H66N13O63P2	603.3940	0.4	21.9	102	77.75	18.0	even	ok
	98	C21H40N49O50S	603.3933	-0.8	21.9	103	57.58	28.0	even	ok
	99	C19H8N85O22S	603.3941	0.7	22.0	104	59.74	60.0	even	ok
	100	C40H63N17NaO59P2	603.3936	-0.2	22.1	105	67.28	20.0	even	ok
	101	C23H45N43NaO52S	603.3934	-0.6	22.2	106	59.87	24.0	even	ok
	102	C21H43N53NaO39P2S2	603.3936	-0.2	22.4	107	80.33	29.0	even	ok
	103	C29H29N55NaO40	603.3936	-0.3	22.4	108	65.51	44.0	even	ok
	104	C16H10N91O13P2S2	603.3940	0.4	22.4	109	64.21	60.0	even	ok
	105	C24H43N47NaO46P2	603.3942	0.8	22.4	110	57.03	29.0	even	ok
	106	C24H48N39O56S	603.3937	-0.1	22.4	111	83.33	22.0	even	ok
	107	C21H13N79NaO24S	603.3942	0.8	22.5	112	56.53	56.0	even	ok
	108	C6H9N95O19PS2	603.3940	0.5	22.5	113	62.25	52.0	even	ok
	109	C22H31N61NaO36P2	603.3942	0.8	22.5	114	56.95	40.0	even	ok
	110	C32H67N19NaO61P2S	603.3934	-0.6	22.6	115	72.64	11.0	even	ok
	111	C19H38N55NaO43PS	603.3939	0.3	22.6	116	65.73	30.0	even	ok
	112	C27H24N61O38	603.3935	-0.4	22.6	117	62.65	48.0	even	ok
	113	C15H29N67NaO36S2	603.3940	0.5	22.6	118	74.71	36.0	even	ok
	114	C5H6N99NaO15PS2	603.3937	-0.1	22.7	119	68.08	54.0	even	ok
	115	C34H59N27NaO53P2S	603.3943	0.9	22.7	120	65.89	21.0	even	ok
	116	C35H72N5O78	603.3935	-0.4	23.0	121	74.94	4.0	even	ok
	117	C29H39N51NaO37P2S	603.3938	0.2	23.0	122	66.83	38.0	even	ok
	118	C26H53N33NaO58S	603.3938	0.1	23.1	123	81.23	18.0	even	ok
	119	C18H5N89NaO18S	603.3938	0.1	23.3	124	67.79	62.0	even	ok
	120	C20H41N51O47PS	603.3943	0.9	23.3	125	54.96	28.0	even	ok
	121	C17H33N61O41PS	603.3938	0.1	23.4	126	67.13	34.0	even	ok
	122	C30H62N21NaO65P	603.3937	-0.1	23.4	128	67.09	12.0	even	ok
	123	C16H30N65NaO37PS	603.3935	-0.5	23.5	129	60.73	36.0	even	ok
	124	C43H71N7NaO65P2	603.3941	0.5	23.7	130	71.67	14.0	even	ok
	125	C34H66N19O61P2S	603.3942	0.8	23.7	131	67.22	14.0	even	ok
	126	C13H24N73O34S2	603.3939	0.3	23.8	132	63.11	40.0	even	ok

Compound Spectrum SmartFormula Report

Meas. m/z	#	Ion Formula	m/z	err [ppm]	mSigma	# mSigma	Score	rdb	e¯ Conf	N-Rule
	127	C33H70N11NaO71P	603.3941	0.6	23.9	133	57.65	6.0	even	ok
	128	C18H15N85NaO15P2S2	603.3941	0.5	24.0	135	59.41	56.0	even	ok
	129	C31H65N17O69P	603.3940	0.5	24.2	136	59.80	10.0	even	ok
	130	C28H57N27O63P	603.3936	-0.3	24.2	137	63.37	16.0	even	ok
	131	C27H54N31NaO59P	603.3932	-0.8	24.4	138	53.71	18.0	even	ok
	132	C22H38N53O44P2	603.3941	0.6	24.4	139	56.91	33.0	even	ok
	133	C30H42N47O41P2S	603.3942	0.7	24.7	140	54.96	36.0	even	ok
	134	C14H25N71O35PS	603.3934	-0.6	24.8	141	56.54	40.0	even	ok
	135	C20H26N67O34P2	603.3941	0.6	24.8	142	56.52	44.0	even	ok
	136	C33H53N31O54PS	603.3932	-0.9	24.8	143	62.86	25.0	even	ok
	137	C21H18N73NaO29P	603.3932	-0.9	24.9	144	52.80	51.0	even	ok
	138	C25H22N67NaO31P	603.3941	0.6	25.3	145	56.20	50.0	even	ok
	139	C22H29N65O32PS2	603.3934	-0.5	25.4	146	68.80	43.0	even	ok
	140	C27H56N29O62S	603.3942	0.7	25.6	147	65.63	16.0	even	ok
	141	C22H46N49O43P2S2	603.3940	0.4	25.6	148	71.32	27.0	even	ok
	142	C36H71N13NaO63P2S	603.3943	0.9	25.7	149	61.39	10.0	even	ok
	143	C33H70N15O65P2S	603.3938	0.0	25.8	150	77.99	9.0	even	ok
	144	C10H11N91NaO18P2S	603.3940	0.4	26.4	151	57.82	53.0	even	ok
	145	C23H17N73O29P	603.3940	0.5	26.4	152	57.14	54.0	even	ok
	146	C29H61N23NaO64S	603.3943	0.8	26.6	153	61.37	12.0	even	ok
	147	C32H47N41NaO43P2S	603.3943	0.9	26.7	154	50.20	32.0	even	ok
	148	C31H41N45O44PS	603.3932	-0.9	26.8	155	59.97	36.0	even	ok
	149	C35H58N25NaO56PS	603.3933	-0.7	27.0	156	62.57	21.0	even	ok
	150	C21H35N57NaO40P2	603.3938	0.1	27.0	157	62.58	35.0	even	ok
	151	C24H51N43NaO45P2S2	603.3941	0.5	27.2	158	65.89	23.0	even	ok
	152	C35H75N9NaO67P2S	603.3938	0.2	27.4	159	72.36	5.0	even	ok
	153	C35H65N17O64PS	603.3932	-0.9	27.4	160	59.38	14.0	even	ok
	154	C24H34N59NaO34PS2	603.3935	-0.4	27.6	161	68.25	39.0	even	ok
	155	C24H41N51O42PS2	603.3934	-0.5	27.6	162	65.47	32.0	even	ok
	156	C20H47N49NaO43P2S2	603.3932	-0.9	27.8	163	57.91	24.0	even	ok
	157	C26H55N33NaO56P2	603.3942	0.8	27.9	164	50.24	18.0	even	ok
	158	C20H19N75NaO26P2	603.3942	0.8	28.1	165	50.15	51.0	even	ok
	159	C19H23N71NaO30P2	603.3938	0.0	28.3	166	60.73	46.0	even	ok
	160	C8H6N97O16P2S	603.3939	0.3	28.6	167	57.18	57.0	even	ok
	161	C33H46N39NaO46PS	603.3933	-0.8	28.7	168	60.02	32.0	even	ok
	162	C36H46N37O47P2	603.3935	-0.4	29.0	169	55.00	35.0	even	ok
	163	C19H30N63O38P2	603.3937	-0.1	29.2	170	58.74	39.0	even	ok
	164	C24H50N39O54P2	603.3941	0.6	29.3	171	50.69	22.0	even	ok
	165	C35H43N41NaO43P2	603.3932	-1.0	29.3	172	46.27	37.0	even	ok
	166	C37H70N11NaO66PS	603.3933	-0.7	29.4	173	59.10	10.0	even	ok
	167	C22H14N77NaO25P	603.3937	-0.1	29.6	174	57.76	56.0	even	ok
	168	C22H14N81O19P2S	603.3933	-0.8	29.7	175	48.79	59.0	even	ok
	169	C26H46N45NaO44PS2	603.3935	-0.4	29.7	176	64.90	28.0	even	ok
	170	C12H23N77NaO28P2S	603.3940	0.4	29.8	177	53.27	42.0	even	ok
	171	C36H61N21O60PS	603.3936	-0.2	29.9	178	68.25	19.0	even	ok
	172	C25H22N71O25P2S	603.3937	-0.0	30.0	179	58.89	53.0	even	ok

Compound Spectrum SmartFormula Report

Meas. m/z	#	Ion Formula	m/z	err [ppm]	mSigma	# mSigma	Score	rdb	e¯ Conf	N-Rule
	173	C45H73NO72P	603.3934	-0.5	30.1	180	61.75	12.0	even	ok
	174	C38H51N31NaO49P2	603.3936	-0.2	30.1	181	55.80	31.0	even	ok
	175	C20H17N79O22PS2	603.3934	-0.5	30.1	182	51.21	54.0	even	ok
	176	C12H28N69O38S2	603.3935	-0.4	30.2	183	63.27	35.0	even	ok
	177	C18H14N81O24P2	603.3941	0.6	30.2	184	49.85	55.0	even	ok
	178	C24H19N75NaO21P2S	603.3934	-0.6	30.3	185	50.22	55.0	even	ok
	179	C25H37N55O38PS2	603.3939	0.2	30.5	186	66.47	37.0	even	ok
	180	C14H33N63NaO40S2	603.3936	-0.3	30.5	187	65.40	31.0	even	ok
	181	C39H54N27O53P2	603.3940	0.4	30.6	188	53.02	29.0	even	ok
	182	C21H50N45O47P2S2	603.3935	-0.4	30.6	189	63.89	22.0	even	ok
	183	C23H47N43NaO50P2	603.3938	0.1	30.6	190	57.34	24.0	even	ok
	184	C17H18N77O28P2	603.3937	-0.1	30.6	191	56.64	50.0	even	ok
	185	C36H78N5O71P2S	603.3942	0.8	30.7	192	57.03	3.0	even	ok
	186	C34H49N35O50PS	603.3936	-0.2	30.8	193	66.71	30.0	even	ok
	187	C23H52N35O60S	603.3933	-0.8	30.9	194	56.23	17.0	even	ok
	188	C32H74N11O69P2S	603.3933	-0.7	31.0	195	57.23	4.0	even	ok
	189	C20H9N83O23P	603.3936	-0.3	31.0	196	53.68	60.0	even	ok
	190	C27H27N65NaO27P2S	603.3938	0.1	31.1	197	55.42	49.0	even	ok
	191	C25H57N29NaO62S	603.3934	-0.6	31.1	198	58.36	13.0	even	ok
	192	C7H3N101NaO12P2S	603.3935	-0.3	31.5	199	52.46	59.0	even	ok
	193	C10H18N83O26P2S	603.3939	0.3	31.5	200	53.14	46.0	even	ok
	194	C28H30N61O31P2S	603.3942	0.7	31.6	201	46.77	47.0	even	ok
	195	C23H25N69O28PS2	603.3939	0.2	31.6	202	64.78	48.0	even	ok
	196	C23H55N39NaO49P2S2	603.3936	-0.2	31.7	203	64.62	18.0	even	ok
	197	C41H59N21NaO55P2	603.3941	0.5	32.0	204	49.08	25.0	even	ok
	198	C18H27N67NaO34P2	603.3933	-0.7	32.1	205	46.84	41.0	even	ok
	199	C34H79N5NaO71P2S	603.3934	-0.6	32.1	206	58.14	0.0	even	ok
	200	C16H3N99NaO5P2S2	603.3941	0.5	32.1	207	49.12	67.0	even	ok
	201	C38H66N15NaO62PS	603.3937	-0.0	32.1	208	67.19	15.0	even	ok
	202	C7H18N85NaO25PS2	603.3937	-0.1	32.2	209	54.50	43.0	even	ok
	203	C22H22N73NaO24PS2	603.3935	-0.4	32.2	210	50.75	50.0	even	ok
	204	C28H20N65O34	603.3939	0.3	32.3	211	51.81	53.0	even	ok
	205	C21H42N49O48P2	603.3937	-0.1	32.3	212	54.44	28.0	even	ok
	206	C30H25N59NaO36	603.3940	0.4	32.5	213	49.55	49.0	even	ok
	207	C33H77N3O79P	603.3940	0.5	32.5	214	58.70	-1.0	even	ok
	208	C10H26N75NaO31PS2	603.3941	0.6	32.6	215	47.00	37.0	even	ok
	209	C15H36N59O44S2	603.3939	0.3	32.6	216	61.34	29.0	even	ok
	210	C27H42N49NaO40PS2	603.3940	0.4	32.7	217	60.54	33.0	even	ok
	211	C36H54N29NaO52PS	603.3937	-0.0	32.8	218	65.91	26.0	even	ok
	212	C38H73N7O70PS	603.3937	-0.2	32.8	219	63.64	8.0	even	ok
	213	C4H10N95NaO19PS2	603.3932	-0.9	32.9	220	43.84	49.0	even	ok
	214	C8H21N81O29PS2	603.3940	0.5	32.9	221	48.60	41.0	even	ok
	215	C5H13N91O23PS2	603.3936	-0.3	32.9	222	51.45	47.0	even	ok
	216	C30H35N55NaO33P2S	603.3943	0.9	33.0	223	43.13	43.0	even	ok
	217	C18H42N51NaO47PS	603.3935	-0.5	33.1	224	48.49	25.0	even	ok
	218	C27H49N41O48PS2	603.3939	0.2	33.1	225	62.21	26.0	even	ok

Compound Spectrum SmartFormula Report

Meas. m/z	#	Ion Formula	m/z	err [ppm]	mSigma	# mSigma	Score	rdb	e¯ Conf	N-Rule
	219	C26H60N25O66S	603.3937	-0.0	33.2	226	64.69	11.0	even	ok
	220	C17H41N53NaO46S2	603.3940	0.5	33.2	227	58.00	25.0	even	ok
	221	C21H50N41NaO53PS	603.3939	0.3	33.4	228	50.77	19.0	even	ok
	222	C9H15N87NaO22P2S	603.3936	-0.3	33.4	229	50.12	48.0	even	ok
	223	C26H53N37O52PS2	603.3934	-0.5	33.4	231	56.91	21.0	even	ok
	224	C37H77N3O74PS	603.3932	-0.9	33.4	232	51.28	3.0	even	ok
	225	C25H30N63NaO30PS2	603.3940	0.4	33.6	233	59.22	44.0	even	ok
	226	C19H45N47O51PS	603.3938	0.1	33.8	234	52.29	23.0	even	ok
	227	C28H65N19NaO68S	603.3938	0.1	33.8	235	62.86	7.0	even	ok
	228	C16H37N57O45PS	603.3934	-0.6	33.8	236	45.61	29.0	even	ok
	229	C29H66N17NaO69P	603.3932	-0.8	33.9	237	42.78	7.0	even	ok
	230	C17H11N85NaO20P2	603.3938	0.0	34.0	238	52.92	57.0	even	ok
	231	C27H17N69NaO30	603.3936	-0.3	34.0	239	49.64	55.0	even	ok
	232	C20H39N53NaO44P2	603.3933	-0.7	34.2	240	44.51	30.0	even	ok
	233	C16H15N81NaO24P2	603.3933	-0.7	34.2	241	44.31	52.0	even	ok
	234	C32H74N7NaO75P	603.3937	-0.1	34.2	242	51.86	1.0	even	ok
	235	C43H61N15O62P	603.3934	-0.5	34.3	243	55.52	23.0	even	ok
	236	C25H12N75O28	603.3935	-0.5	34.3	244	47.34	59.0	even	ok
	237	C16H22N73O32P2	603.3932	-0.8	34.3	245	42.36	45.0	even	ok
	238	C19H6N87NaO19P	603.3932	-0.9	34.4	246	41.87	62.0	even	ok
	239	C30H69N13O73P	603.3936	-0.3	34.6	247	49.40	5.0	even	ok
	240	C22H53N37O57PS	603.3943	0.9	34.7	248	41.74	17.0	even	ok
	241	C24H58N35O53P2S2	603.3940	0.4	34.7	249	57.03	16.0	even	ok
	242	C40H78NNaO72PS	603.3937	0.0	34.9	250	62.58	4.0	even	ok
	243	C35H82NO75P2S	603.3938	0.0	35.1	251	62.06	-2.0	even	ok
	244	C29H54N35NaO50PS2	603.3940	0.4	35.2	252	56.65	22.0	even	ok
	245	C39H69N11O66PS	603.3941	0.6	35.2	253	53.42	13.0	even	ok
	246	C28H58N31NaO54PS2	603.3935	-0.4	35.3	254	56.55	17.0	even	ok
	247	C37H57N25O56PS	603.3941	0.6	35.3	255	53.39	24.0	even	ok
	248	C7H10N93O20P2S	603.3935	-0.5	35.3	256	45.80	52.0	even	ok
	249	C29H29N59O34PS	603.3932	-0.9	35.4	257	40.38	47.0	even	ok
	250	C28H45N45O44PS2	603.3943	1.0	35.7	258	47.45	31.0	even	ok
	251	C45H66N9NaO64P	603.3935	-0.4	36.0	259	55.38	19.0	even	ok
	252	C26H33N59O34PS2	603.3943	0.9	36.0	260	47.17	42.0	even	ok
	253	C18H34N59O42P2	603.3932	-0.8	36.1	261	40.53	34.0	even	ok
	254	C26H63N29NaO55P2S2	603.3941	0.5	36.1	262	52.86	12.0	even	ok
	255	C15H6N91O18P2	603.3937	-0.1	36.1	263	49.25	61.0	even	ok
	256	C29H68N15O72S	603.3942	0.7	36.2	264	50.53	5.0	even	ok
	257	C13H9N85NaO27	603.3942	0.7	36.2	265	41.79	53.0	even	ok
	258	C18H5N93O12PS2	603.3934	-0.5	36.4	266	43.68	65.0	even	ok
	259	C15H21N71NaO37	603.3942	0.7	36.4	267	41.49	42.0	even	ok
	260	C14H10N87O22P2	603.3932	-0.9	36.5	268	39.96	56.0	even	ok
	261	C28H67N19NaO66P2	603.3942	0.8	36.5	269	40.38	7.0	even	ok
	262	C23H10N81NaO21P	603.3941	0.6	36.8	270	42.46	61.0	even	ok
	263	C18H7N89NaO16P2	603.3942	0.8	36.8	271	40.45	62.0	even	ok
	264	C34H42N43NaO42PS	603.3937	-0.0	36.8	272	59.38	37.0	even	ok

Compound Spectrum SmartFormula Report

Meas. m/z	#	Ion Formula	m/z	err [ppm]	mSigma	# mSigma	Score	rdb	e⁻ Conf	N-Rule
	265	C14H35N63NaO38P2S	603.3940	0.4	36.9	273	44.43	31.0	even	ok
	266	C31H34N53NaO36PS	603.3933	-0.8	37.0	274	40.49	43.0	even	ok
	267	C31H73N9NaO74S	603.3943	0.9	37.0	275	47.30	1.0	even	ok
	268	C39H62N19NaO58PS	603.3942	0.7	37.4	276	48.39	20.0	even	ok
	269	C41H74N5NaO68PS	603.3942	0.7	37.4	277	48.29	9.0	even	ok
	270	C26H62N25O64P2	603.3941	0.7	37.5	278	41.14	11.0	even	ok
	271	C46H69N5O68P	603.3939	0.2	37.7	279	55.35	17.0	even	ok
	272	C21H5N87O19P	603.3940	0.5	37.7	280	43.21	65.0	even	ok
	273	C25H59N29NaO60P2	603.3938	0.1	37.8	281	47.66	13.0	even	ok
	274	C20H10N87NaO14PS2	603.3935	-0.4	38.0	282	43.63	61.0	even	ok
	275	C12H30N69O36P2S	603.3939	0.3	38.2	283	44.74	35.0	even	ok
	276	C22H59N35NaO53P2S2	603.3932	-0.9	38.3	284	44.50	13.0	even	ok
	277	C32H37N49O40PS	603.3936	-0.2	38.4	285	45.68	41.0	even	ok
	278	C11H4N91O25	603.3941	0.6	38.5	286	41.09	57.0	even	ok
	279	C13H16N77O35	603.3941	0.6	38.5	287	41.00	46.0	even	ok
	280	C35H45N39O46PS	603.3941	0.6	38.5	288	49.11	35.0	even	ok
	281	C30H57N31O54PS2	603.3943	1.0	38.6	289	43.89	20.0	even	ok
	282	C29H61N27O58PS2	603.3939	0.2	38.7	290	53.65	15.0	even	ok
	283	C11H27N73NaO32P2S	603.3936	-0.3	39.0	291	43.36	37.0	even	ok
	284	C23H54N35O58P2	603.3937	-0.1	39.0	292	45.80	17.0	even	ok
	285	C34H34N51O37P2	603.3935	-0.4	39.3	293	42.22	46.0	even	ok
	286	C24H21N73O24PS2	603.3943	0.9	39.4	294	43.20	53.0	even	ok
	287	C21H13N83O18PS2	603.3939	0.2	39.4	295	44.17	59.0	even	ok
	288	C37H42N41O43P2	603.3940	0.4	39.7	296	41.90	40.0	even	ok
	289	C22H51N39NaO54P2	603.3933	-0.7	39.8	297	38.35	19.0	even	ok
	290	C36H39N45NaO39P2	603.3936	-0.2	40.0	298	43.15	42.0	even	ok
	291	C29H44N43O47S2	603.3933	-0.7	40.0	299	45.56	31.0	even	ok
	292	C33H31N55NaO33P2	603.3932	-1.0	40.2	300	34.95	48.0	even	ok
	293	C23H10N85O15P2S	603.3937	-0.0	40.3	301	45.12	64.0	even	ok
	294	C17H33N57NaO47	603.3942	0.7	40.3	302	37.34	31.0	even	ok
	295	C37H50N33NaO48PS	603.3942	0.7	40.4	303	44.73	31.0	even	ok
	296	C27H32N57O37S2	603.3933	-0.7	40.4	304	45.00	42.0	even	ok
	297	C9H22N79O30P2S	603.3935	-0.5	40.5	305	39.96	41.0	even	ok
	298	C31H66N21NaO60PS2	603.3940	0.4	40.6	306	48.94	11.0	even	ok
	299	C26H18N75O21P2S	603.3942	0.7	40.7	308	36.87	58.0	even	ok
	300	C39H47N35NaO45P2	603.3941	0.5	40.8	309	39.09	36.0	even	ok
	301	C31H78N3NaO79P	603.3932	-0.8	40.9	311	42.76	-4.0	even	ok
	302	C23H62N31O57P2S2	603.3935	-0.3	40.9	312	48.86	11.0	even	ok
	303	C25H15N79NaO17P2S	603.3938	0.1	41.0	313	42.88	60.0	even	ok
	304	C23H62N27NaO63PS	603.3939	0.3	41.0	314	49.70	8.0	even	ok
	305	C14H40N55O48S2	603.3935	-0.4	41.0	315	47.81	24.0	even	ok
	306	C22H7N89NaO11P2S	603.3934	-0.6	41.1	316	37.86	66.0	even	ok
	307	C12H13N81NaO31	603.3937	-0.0	41.1	317	44.02	48.0	even	ok
	308	C21H57N33O61PS	603.3938	0.1	41.2	318	51.48	12.0	even	ok
	309	C4H4N97O23S	603.3943	0.9	41.2	319	34.21	53.0	even	ok
	310	C23H18N77NaO20PS2	603.3940	0.4	41.2	320	40.35	55.0	even	ok

Compound Spectrum SmartFormula Report

Meas. m/z	#	Ion Formula	m/z	err [ppm]	mSigma	# mSigma	Score	rdb	e¯ Conf	N-Rule
	311	C20H46N45O52P2	603.3932	-0.8	41.3	321	35.22	23.0	even	ok
	312	C28H65N23O62PS2	603.3934	-0.5	41.4	322	46.16	10.0	even	ok
	313	C16H45N49NaO50S2	603.3936	-0.3	41.4	323	49.31	20.0	even	ok
	314	C42H73N3NaO71S	603.3932	-0.9	41.7	324	40.89	9.0	even	ok
	315	C25H64N21O70S	603.3933	-0.8	41.7	325	42.39	6.0	even	ok
	316	C28H23N69NaO23P2S	603.3943	0.9	41.8	326	34.27	54.0	even	ok
	317	C40H61N17NaO61S	603.3932	-0.9	41.8	328	40.69	20.0	even	ok
	318	C25H67N25NaO59P2S2	603.3936	-0.2	41.9	329	49.48	7.0	even	ok
	319	C15H28N63O45	603.3941	0.6	42.0	330	37.16	35.0	even	ok
	320	C27H69N15NaO72S	603.3934	-0.6	42.0	331	43.90	2.0	even	ok
	321	C31H49N37NaO49S2	603.3934	-0.5	42.2	333	44.75	27.0	even	ok
	322	C24H65N23O67PS	603.3943	0.9	42.5	334	40.59	6.0	even	ok
	323	C31H56N29O57S2	603.3933	-0.7	42.5	335	42.61	20.0	even	ok
	324	C29H37N51NaO39S2	603.3934	-0.5	42.5	336	44.30	38.0	even	ok
	325	C30H70N17NaO64PS2	603.3935	-0.4	43.0	337	45.99	6.0	even	ok
	326	C10H8N87O29	603.3936	-0.2	43.3	339	39.90	52.0	even	ok
	327	C44H57N19O58P	603.3939	0.2	43.3	340	47.52	28.0	even	ok
	328	C14H3N95NaO14P2	603.3933	-0.7	43.5	341	27.56	63.0	even	ok
	329	C17H48N45O54S2	603.3939	0.3	43.5	342	45.78	18.0	even	ok
	330	C25H20N71O27S2	603.3933	-0.7	43.6	343	41.15	53.0	even	ok
	331	C14H25N67NaO41	603.3937	-0.0	43.8	344	40.89	37.0	even	ok
	332	C20H54N37NaO57PS	603.3935	-0.5	43.8	345	36.37	14.0	even	ok
	333	C27H17N73O24PS	603.3932	-0.9	43.9	346	32.00	58.0	even	ok
	334	C28H13N73NaO26	603.3940	0.4	43.9	347	36.60	60.0	even	ok
	335	C26H8N79O24	603.3939	0.3	43.9	348	38.11	64.0	even	ok
	336	C19H53N39NaO56S2	603.3940	0.5	44.1	349	43.25	14.0	even	ok
	337	C32H69N17O64PS2	603.3943	1.0	44.1	350	37.63	9.0	even	ok
	338	C28H72N11O76S	603.3937	-0.0	44.1	351	48.29	0.0	even	ok
	339	C18H49N43O55PS	603.3934	-0.6	44.2	352	34.44	18.0	even	ok
	340	C41H64N13O65S	603.3935	-0.3	44.5	353	44.42	18.0	even	ok
	341	C38H49N31NaO51S	603.3932	-0.9	44.6	354	37.52	31.0	even	ok
	342	C30H77N5NaO78S	603.3938	0.1	44.6	355	46.75	-4.0	even	ok
	343	C33H61N23NaO59S2	603.3934	-0.5	44.7	356	41.84	16.0	even	ok
	344	C41H49N29O52P	603.3934	-0.5	44.7	357	34.76	34.0	even	ok
	345	C26H70N21O63P2S2	603.3940	0.4	44.7	358	43.35	5.0	even	ok
	346	C46H62N13NaO60P	603.3940	0.4	44.8	359	43.67	24.0	even	ok
	347	C29H22N67NaO26PS	603.3933	-0.8	45.1	360	32.32	54.0	even	ok
	348	C29H73N9O77P	603.3931	-1.0	45.1	361	30.19	0.0	even	ok
	349	C30H40N47O43S2	603.3938	0.0	45.2	362	46.82	36.0	even	ok
	350	C32H52N33O53S2	603.3938	0.0	45.4	363	46.53	25.0	even	ok
	351	C27H25N65NaO29S2	603.3934	-0.6	45.5	364	40.68	49.0	even	ok
	352	C12H20N73O39	603.3936	-0.2	45.7	365	37.30	41.0	even	ok
	353	C25H5N83NaO20	603.3936	-0.3	45.7	366	36.04	66.0	even	ok
	354	C30H25N63O30PS	603.3936	-0.2	45.7	367	37.17	52.0	even	ok
	355	C35H38N47NaO38PS	603.3942	0.7	45.7	368	38.48	42.0	even	ok
	356	C28H75N15NaO65P2S2	603.3941	0.6	45.9	369	40.25	1.0	even	ok

Compound Spectrum SmartFormula Report

Meas. m/z	#	Ion Formula	m/z	err [ppm]	mSigma	# mSigma	Score	rdb	e⁻ Conf	N-Rule
	357	C16H47N49NaO48P2S	603.3940	0.4	46.1	371	34.36	20.0	even	ok
	358	C9H5N91NaO25	603.3933	-0.8	46.1	372	31.39	54.0	even	ok
	359	C43H54N23NaO54P	603.3935	-0.4	46.2	373	34.68	30.0	even	ok
	360	C47H65N9O64P	603.3943	0.9	46.2	374	35.56	22.0	even	ok
	361	C31H73N13O68PS2	603.3939	0.2	46.3	375	43.26	4.0	even	ok
	362	C43H69N7NaO67S	603.3936	-0.2	46.7	376	43.38	14.0	even	ok
	363	C30H79N5NaO76P2	603.3942	0.8	46.7	378	30.35	-4.0	even	ok
	364	C39H52N27O55S	603.3935	-0.3	46.7	379	41.58	29.0	even	ok
	365	C14H42N55O46P2S	603.3939	0.3	47.0	380	34.87	24.0	even	ok
	366	C19H45N43NaO57	603.3942	0.7	47.0	381	30.82	20.0	even	ok
	367	C27H71N15NaO70P2	603.3938	0.1	47.0	382	36.76	2.0	even	ok
	368	C31H80NO82S	603.3942	0.7	47.0	383	37.34	-6.0	even	ok
	369	C13H39N59NaO42P2S	603.3936	-0.3	47.0	384	34.59	26.0	even	ok
	370	C32H30N57NaO32PS	603.3937	-0.0	47.2	385	36.95	48.0	even	ok
	371	C32H45N41NaO45S2	603.3939	0.2	47.4	386	42.29	32.0	even	ok
	372	C28H74N11O74P2	603.3941	0.7	47.4	387	31.14	0.0	even	ok
	373	C33H68N15O67S2	603.3933	-0.7	47.4	388	37.02	9.0	even	ok
	374	C6H16N83O33S	603.3943	1.0	47.4	389	28.56	42.0	even	ok
	375	C28H28N61O33S2	603.3938	0.0	47.5	390	43.75	47.0	even	ok
	376	C34H57N27NaO55S2	603.3939	0.2	47.6	391	41.96	21.0	even	ok
	377	C11H17N77NaO35	603.3933	-0.8	47.8	392	29.94	43.0	even	ok
	378	C25H66N21O68P2	603.3937	-0.1	47.9	393	35.70	6.0	even	ok
	379	C24H63N25NaO64P2	603.3933	-0.7	47.9	394	30.50	8.0	even	ok
	380	C33H78N7NaO70PS2	603.3940	0.4	48.0	395	39.55	0.0	even	ok
	381	C34H64N19O63S2	603.3938	0.1	48.0	396	42.93	14.0	even	ok
	382	C21H6N91NaO10PS2	603.3940	0.3	48.2	397	33.03	66.0	even	ok
	383	C17H40N49O55	603.3941	0.6	48.3	399	30.91	24.0	even	ok
	384	C33H33N53O36PS	603.3941	0.6	48.3	400	31.02	46.0	even	ok
	385	C5H13N87NaO29S	603.3940	0.4	48.4	401	32.71	44.0	even	ok
	386	C41H57N21NaO57S	603.3936	-0.2	48.8	402	40.74	25.0	even	ok
	387	C22H58N31O62P2	603.3932	-0.8	49.0	403	28.23	12.0	even	ok
	388	C24H71N21NaO63P2S2	603.3932	-0.9	49.1	404	32.78	2.0	even	ok
	389	C16H37N53NaO51	603.3937	-0.0	49.3	405	34.84	26.0	even	ok
	390	C22H9N87O14PS2	603.3943	0.9	49.3	406	27.10	64.0	even	ok
	391	C35H73N9NaO69S2	603.3934	-0.5	49.4	407	36.39	5.0	even	ok
	392	C44H72N3O71S	603.3940	0.4	49.5	408	37.40	12.0	even	ok
	393	C30H33N55NaO35S2	603.3939	0.2	49.5	409	39.65	43.0	even	ok
	394	C36H37N45NaO41S	603.3932	-0.9	49.7	410	32.29	42.0	even	ok
	395	C9H12N83O33	603.3932	-0.9	49.7	411	26.98	47.0	even	ok
	396	C3H8N93O27S	603.3939	0.2	50.0	412	32.49	48.0	even	ok
	397	C35H30N55O33P2	603.3940	0.4	50.0	413	31.22	51.0	even	ok
	398	C33H48N37O49S2	603.3942	0.8	50.2	414	33.15	30.0	even	ok
	399	C36H69N13NaO65S2	603.3939	0.2	50.2	415	38.71	10.0	even	ok
	400	C32H22N65O27P2	603.3935	-0.4	50.2	416	30.77	57.0	even	ok
	401	C48H64N7O67	603.3933	-0.7	50.3	417	33.77	22.0	even	ok
	402	C22H66N23NaO67PS	603.3935	-0.5	50.4	418	35.95	3.0	even	ok

Compound Spectrum SmartFormula Report

Meas. m/z	#	Ion Formula	m/z	err [ppm]	mSigma	# mSigma	Score	rdb	e⁻ Conf	N-Rule
	403	C30H77N9O72PS2	603.3934	-0.5	50.5	419	35.39	-1.0	even	ok
	404	C25H13N79NaO19S2	603.3934	-0.6	50.6	420	34.88	60.0	even	ok
	405	C34H27N59NaO29P2	603.3936	-0.2	50.7	421	31.60	53.0	even	ok
	406	C37H35N49NaO35P2	603.3941	0.5	50.7	422	29.30	47.0	even	ok
	407	C35H60N23O59S2	603.3942	0.8	50.8	423	32.49	19.0	even	ok
	408	C14H32N59O49	603.3936	-0.2	50.8	424	32.01	30.0	even	ok
	409	C24H6N89O11P2S	603.3942	0.7	51.0	425	27.41	69.0	even	ok
	410	C42H60N17O61S	603.3940	0.4	51.1	426	35.70	23.0	even	ok
	411	C37H40N41O45S	603.3935	-0.3	51.2	427	36.32	40.0	even	ok
	412	C2H5N97NaO23S	603.3935	-0.4	51.3	428	29.76	50.0	even	ok
	413	C34H81N3O74PS2	603.3943	1.0	51.4	429	30.23	-2.0	even	ok
	414	C31H19N69NaO23P2	603.3932	-1.0	51.4	430	25.15	59.0	even	ok
	415	C25H74N17O67P2S2	603.3935	-0.3	51.6	431	35.77	0.0	even	ok
	416	C23H3N93NaO7P2S	603.3938	0.1	51.6	432	31.42	71.0	even	ok
	417	C26H11N83NaO13P2S	603.3943	0.9	51.7	433	25.62	65.0	even	ok
	418	C25H74N13NaO73PS	603.3939	0.3	51.7	434	36.25	-3.0	even	ok
	419	C23H69N19O71PS	603.3938	0.1	51.7	435	37.69	1.0	even	ok
	420	C31H36N51O39S2	603.3942	0.8	51.9	436	31.51	41.0	even	ok
	421	C32H82N3NaO74PS2	603.3935	-0.4	51.9	437	35.32	-5.0	even	ok
	422	C10H18N79NaO32P	603.3943	0.9	52.0	438	25.26	43.0	even	ok
	423	C26H16N75O23S2	603.3938	0.0	52.1	439	38.19	58.0	even	ok
	424	C50H69NNaO69	603.3934	-0.6	52.1	440	33.34	18.0	even	ok
	425	C13H29N63NaO45	603.3933	-0.8	52.2	441	26.23	32.0	even	ok
	426	C35H53N31NaO51S2	603.3943	0.9	52.4	442	29.60	26.0	even	ok
	427	C27H79N11NaO69P2S2	603.3936	-0.2	52.5	443	36.23	-4.0	even	ok
	428	C45H53N23O54P	603.3943	0.9	52.7	444	29.30	33.0	even	ok
	429	C27H76N7O80S	603.3933	-0.8	52.7	445	30.68	-5.0	even	ok
	430	C36H76N5O73S2	603.3938	0.1	52.9	446	36.93	3.0	even	ok
	431	C39H45N35NaO47S	603.3936	-0.2	53.0	447	35.73	36.0	even	ok
	432	C37H65N17NaO61S2	603.3943	0.9	53.0	448	28.98	15.0	even	ok
	433	C29H81NNaO82S	603.3934	-0.6	53.0	449	31.71	-9.0	even	ok
	434	C44H65N11NaO63S	603.3941	0.6	53.2	450	32.09	19.0	even	ok
	435	C26H77N9O77PS	603.3943	0.9	53.3	451	29.31	-5.0	even	ok
	436	C23H8N85O17S2	603.3933	-0.7	53.5	452	25.38	64.0	even	ok
	437	C25H5N87O14PS	603.3932	-0.9	53.6	453	23.84	69.0	even	ok
	438	C39H37N43O42P	603.3934	-0.5	53.6	454	26.54	45.0	even	ok
	439	C37H72N9O69S2	603.3942	0.8	53.6	455	29.68	8.0	even	ok
	440	C11H24N69O43	603.3932	-0.9	53.8	456	23.79	36.0	even	ok
	441	C28H21N69NaO25S2	603.3939	0.2	53.8	457	34.75	54.0	even	ok
	442	C38H66N19O56P2S2	603.3934	-0.6	53.9	458	30.88	18.0	even	ok
	443	C33H41N45NaO41S2	603.3943	0.9	53.9	459	28.21	37.0	even	ok
	444	C8H13N85O30P	603.3942	0.7	54.0	460	24.83	47.0	even	ok
	445	C35H80NO77S2	603.3933	-0.7	54.2	461	30.12	-2.0	even	ok
	446	C27H10N81NaO16PS	603.3933	-0.8	54.5	462	24.22	65.0	even	ok
	447	C28H13N77O20PS	603.3936	-0.2	54.6	463	28.30	63.0	even	ok
	448	C36H54N33O46P2S2	603.3934	-0.6	54.6	464	30.11	29.0	even	ok

Compound Spectrum SmartFormula Report

Meas. m/z	#	Ion Formula	m/z	err [ppm]	mSigma	# mSigma	Score	rdb	e¯ Conf	N-Rule
	449	C41H42N37NaO44P	603.3935	-0.4	54.8	465	26.64	41.0	even	ok
	450	C40H48N31O51S	603.3940	0.4	54.8	466	31.85	34.0	even	ok
	451	C28H82N7O73P2S2	603.3940	0.4	55.2	467	31.47	-6.0	even	ok
	452	C40H78N5O66P2S2	603.3934	-0.6	55.3	468	29.56	7.0	even	ok
	453	C42H45N33O48P	603.3939	0.2	55.3	469	27.56	39.0	even	ok
	454	C21H57N29NaO67	603.3942	0.7	55.5	471	23.65	9.0	even	ok
	455	C8H6N93NaO22P	603.3943	0.9	55.6	472	18.07	54.0	even	ok
	456	C29H24N65O29S2	603.3942	0.8	55.6	473	28.00	52.0	even	ok
	457	C39H77N3NaO71S2	603.3943	1.0	55.8	474	26.46	4.0	even	ok
	458	C30H18N71NaO22PS	603.3937	-0.0	55.8	475	28.30	59.0	even	ok
	459	C12H30N65NaO42P	603.3943	0.9	56.1	476	22.16	32.0	even	ok
	460	C40H71N13NaO58P2S2	603.3935	-0.5	56.1	477	29.99	14.0	even	ok
	461	C30H87NNaO75P2S2	603.3941	0.6	56.2	478	29.23	-10.0	even	ok
	462	C31H21N67O26PS	603.3941	0.6	56.3	479	24.21	57.0	even	ok
	463	C7H10N89NaO26P	603.3938	0.2	56.4	480	26.93	49.0	even	ok
	464	C46H52N21O57	603.3933	-0.7	56.4	481	27.83	33.0	even	ok
	465	C19H52N35O65	603.3941	0.6	56.5	482	23.89	13.0	even	ok
	466	C42H53N25NaO53S	603.3941	0.6	56.7	483	28.72	30.0	even	ok
	467	C44H50N27NaO50P	603.3940	0.4	56.7	484	25.29	35.0	even	ok
	468	C38H59N27NaO48P2S2	603.3935	-0.5	56.8	485	29.29	25.0	even	ok
	469	C18H49N39NaO61	603.3937	-0.0	56.8	486	27.51	15.0	even	ok
	470	C29H83NNaO80P2	603.3938	0.1	57.3	487	26.63	-9.0	even	ok
	471	C35H28N55O35S	603.3935	-0.3	57.3	488	29.84	51.0	even	ok
	472	C34H42N47O36P2S2	603.3934	-0.6	57.4	489	27.45	40.0	even	ok
	473	C26H75N11NaO74P2	603.3933	-0.7	57.4	490	22.68	-3.0	even	ok
	474	C31H29N59NaO31S2	603.3943	0.9	57.5	491	25.16	48.0	even	ok
	475	C33H26N61NaO28PS	603.3942	0.7	57.7	492	22.14	53.0	even	ok
	476	C10H25N71O40P	603.3942	0.7	57.8	493	21.92	36.0	even	ok
	477	C27H78N7O78P2	603.3937	-0.1	57.9	494	26.09	-5.0	even	ok
	478	C48H57N15NaO59	603.3934	-0.6	57.9	495	27.60	29.0	even	ok
	479	C16H44N45O59	603.3936	-0.2	58.0	497	25.46	19.0	even	ok
	480	C24H70N17O72P2	603.3932	-0.8	58.2	498	21.12	1.0	even	ok
	481	C47H71N7NaO60P2S	603.3932	-0.9	58.6	499	24.82	18.0	even	ok
	482	C15H41N49NaO55	603.3933	-0.7	58.7	500	21.26	21.0	even	ok
	483	C37H33N49NaO37S	603.3936	-0.2	58.9	501	29.48	47.0	even	ok
	484	C41H74N9O62P2S2	603.3938	0.1	59.2	502	29.70	12.0	even	ok
	485	C49H60N11O63	603.3938	0.0	59.3	503	30.22	27.0	even	ok
	486	C36H47N41NaO38P2S2	603.3935	-0.5	59.4	504	26.78	36.0	even	ok
	487	C39H62N23O52P2S2	603.3938	0.1	59.5	505	29.43	23.0	even	ok
	488	C9H22N75NaO36P	603.3938	0.2	59.5	506	24.23	38.0	even	ok
	489	C13H36N55O53	603.3932	-0.9	60.1	507	19.41	25.0	even	ok
	490	C38H36N45O41S	603.3940	0.4	60.2	508	26.71	45.0	even	ok
	491	C33H18N69O23P2	603.3940	0.4	60.8	509	22.08	62.0	even	ok
	492	C48H74N3O64P2S	603.3936	-0.3	60.9	510	26.99	16.0	even	ok
	493	C51H65N5NaO65	603.3939	0.2	61.0	511	27.46	23.0	even	ok
	494	C27H12N79O19S2	603.3942	0.8	61.1	512	23.40	63.0	even	ok

Compound Spectrum SmartFormula Report

Meas. m/z	#	Ion Formula	m/z	err [ppm]	mSigma	# mSigma	Score	rdb	e⁻ Conf	N-Rule
	495	C35H23N63NaO25P2	603.3941	0.5	61.3	513	20.81	58.0	even	ok
	496	C7H17N81O34P	603.3937	0.0	61.3	514	23.66	42.0	even	ok
	497	C43H79N3NaO64P2S2	603.3939	0.3	61.4	515	26.47	8.0	even	ok
	498	C30H10N79O17P2	603.3935	-0.4	61.5	516	21.36	68.0	even	ok
	499	C34H25N59NaO31S	603.3932	-0.9	61.6	517	18.25	53.0	even	ok
	500	C41H67N17NaO54P2S2	603.3939	0.3	61.7	518	26.27	19.0	even	ok
617.7359	1	C63H73O60S2	617.7362	0.5	34.7	1	89.17	29.0	even	ok
	2	C61H74NaO60S2	617.7354	-0.8	34.8	2	83.86	26.0	even	ok
	3	C62H70N4NaO56S2	617.7358	-0.0	35.5	3	100.00	31.0	even	ok
	4	C59H69N6O58S2	617.7353	-0.9	35.7	4	78.24	30.0	even	ok
	5	C60H65N10O54S2	617.7358	-0.2	36.0	5	94.96	35.0	even	ok
	6	C60H75N2NaO57PS2	617.7364	0.9	36.7	6	77.14	26.0	even	ok
	7	C59H62N14NaO50S2	617.7354	-0.8	37.5	7	77.88	37.0	even	ok
	8	C58H70N8O55PS2	617.7363	0.7	37.9	8	78.04	30.0	even	ok
	9	C57H57N20O48S2	617.7353	-0.9	38.5	9	72.63	41.0	even	ok
	10	C63H66N8NaO52S2	617.7363	0.7	39.0	10	76.30	36.0	even	ok
	11	C61H61N14O50S2	617.7362	0.5	39.1	11	79.47	40.0	even	ok
	12	C58H63N16NaO47PS2	617.7364	0.9	39.2	12	72.28	37.0	even	ok
	13	C57H67N12NaO51PS2	617.7359	0.1	40.0	13	86.37	32.0	even	ok
	14	C60H58N18NaO46S2	617.7358	-0.0	40.2	14	88.04	42.0	even	ok
	15	C56H58N22O45PS2	617.7363	0.7	40.4	15	73.10	41.0	even	ok
	16	C58H53N24O44S2	617.7357	-0.2	40.7	16	83.57	46.0	even	ok
	17	C57H74N4O59PS2	617.7359	-0.0	41.1	17	86.63	25.0	even	ok
	18	C55H62N18O49PS2	617.7359	-0.0	41.5	18	85.36	36.0	even	ok
	19	C64H191N5NaO46S2	617.7361	0.4	42.0	19	76.72	-27.0	even	ok
	20	C62H186N11O44S2	617.7360	0.2	42.2	20	79.45	-23.0	even	ok
	21	C57H50N28NaO40S2	617.7354	-0.8	42.4	21	67.98	48.0	even	ok
	22	C67H75O53P2S2	617.7358	-0.1	42.7	22	80.61	33.0	even	ok
	23	C55H55N26NaO41PS2	617.7359	0.1	42.7	23	80.16	43.0	even	ok
	24	C64H67N10O47P2S2	617.7353	-0.8	42.8	24	65.70	39.0	even	ok
	25	C66H72N4NaO49P2S2	617.7354	-0.7	43.0	25	68.32	35.0	even	ok
	26	C56H71N8NaO55PS2	617.7355	-0.6	43.1	26	70.16	27.0	even	ok
	27	C55H45N34O38S2	617.7353	-0.9	43.3	27	63.40	52.0	even	ok
	28	C61H183N15NaO40S2	617.7356	-0.3	43.5	28	74.07	-21.0	even	ok
	29	C56H51N30NaO37PS2	617.7364	0.9	43.8	29	63.70	48.0	even	ok
	30	C54H59N22NaO45PS2	617.7355	-0.6	44.0	30	68.39	38.0	even	ok
	31	C53H50N32O39PS2	617.7358	-0.0	44.2	31	78.97	47.0	even	ok
	32	C54H66N14O53PS2	617.7354	-0.7	44.8	32	63.97	31.0	even	ok
	33	C54H46N36O35PS2	617.7363	0.7	44.9	33	64.44	52.0	even	ok
	34	C65H70N2O57PS	617.7361	0.3	45.1	34	70.98	34.0	even	ok
	35	C61H54N22NaO42S2	617.7363	0.7	45.1	35	64.35	47.0	even	ok
	36	C59H49N28O40S2	617.7362	0.5	45.3	36	66.96	51.0	even	ok
	37	C52H54N28O43PS2	617.7354	-0.7	45.7	37	62.27	42.0	even	ok
	38	C56H78O63PS2	617.7354	-0.7	46.1	38	61.81	20.0	even	ok
	39	C58H46N32NaO36S2	617.7358	-0.0	46.6	39	73.40	53.0	even	ok
	40	C52H47N36NaO35PS2	617.7355	-0.6	46.9	40	62.73	49.0	even	ok

Compound Spectrum SmartFormula Report

Meas. m/z	#	Ion Formula	m/z	err [ppm]	mSigma	# mSigma	Score	rdb	e^- Conf	N-Rule
	41	C67H65N4O56S	617.7355	-0.5	46.9	41	63.57	39.0	even	ok
	42	C56H41N38O34S2	617.7357	-0.2	47.1	42	69.66	57.0	even	ok
	43	C53H43N40NaO31PS2	617.7359	0.1	47.4	43	70.16	54.0	even	ok
	44	C64H67N6NaO53PS	617.7357	-0.2	47.5	44	67.94	36.0	even	ok
	45	C53H67N16O50P2S2	617.7364	0.9	48.5	45	55.03	31.0	even	ok
	46	C50H42N42O33PS2	617.7354	-0.7	48.6	46	57.10	53.0	even	ok
	47	C62H179N19NaO36S2	617.7361	0.4	48.8	47	63.05	-16.0	even	ok
	48	C51H38N46O29PS2	617.7358	-0.0	48.8	48	68.96	58.0	even	ok
	49	C55H38N42NaO30S2	617.7354	-0.8	48.9	49	56.28	59.0	even	ok
	50	C62H62N12O51PS	617.7356	-0.4	49.0	50	62.54	40.0	even	ok
	51	C60H174N25O34S2	617.7360	0.2	49.0	51	65.25	-12.0	even	ok
	52	C51H55N30O40P2S2	617.7364	0.9	49.3	52	53.92	42.0	even	ok
	53	C65H63N14O43P2S2	617.7358	-0.1	49.3	53	66.37	44.0	even	ok
	54	C62H55N24O37P2S2	617.7353	-0.8	49.7	54	53.54	50.0	even	ok
	55	C68H71N4O49P2S2	617.7362	0.6	49.7	55	57.47	38.0	even	ok
	56	C55H79N2O60P2S2	617.7364	0.9	49.8	56	52.95	20.0	even	ok
	57	C67H68N8NaO45P2S2	617.7359	0.0	49.8	57	66.90	40.0	even	ok
	58	C64H60N18NaO39P2S2	617.7354	-0.7	49.9	58	55.75	46.0	even	ok
	59	C65H63N10NaO49PS	617.7362	0.5	49.9	59	59.15	41.0	even	ok
	60	C54H39N44NaO27PS2	617.7364	0.8	49.9	60	53.23	59.0	even	ok
	61	C62H72N4NaO54P2S	617.7363	0.7	50.4	61	55.46	31.0	even	ok
	62	C59H171N29NaO30S2	617.7356	-0.4	50.5	62	60.23	-10.0	even	ok
	63	C68H190NO50S	617.7353	-0.9	50.8	63	51.62	-24.0	even	ok
	64	C63H58N16O47PS	617.7361	0.3	51.0	64	59.61	45.0	even	ok
	65	C57H166N35O28S2	617.7356	-0.5	51.0	65	56.88	-6.0	even	ok
	66	C52H34N50O25PS2	617.7363	0.7	51.0	66	53.88	63.0	even	ok
	67	C65H192N3NaO47PS	617.7355	-0.5	51.0	67	56.13	-27.0	even	ok
	68	C52H64N20NaO46P2S2	617.7360	0.3	51.1	68	59.96	33.0	even	ok
	69	C50H35N50NaO25PS2	617.7355	-0.6	51.5	69	54.40	60.0	even	ok
	70	C67H191N3O47PS	617.7363	0.8	51.6	70	52.03	-24.0	even	ok
	71	C49H43N44O30P2S2	617.7364	0.9	51.9	71	49.86	53.0	even	ok
	72	C54H76N6NaO56P2S2	617.7361	0.3	51.9	72	58.35	22.0	even	ok
	73	C70H66N2NaO54S	617.7361	0.3	52.1	73	57.82	40.0	even	ok
	74	C50H52N34NaO36P2S2	617.7360	0.3	52.1	74	58.16	44.0	even	ok
	75	C68H61N8O52S	617.7360	0.2	52.2	75	59.86	44.0	even	ok
	76	C60H67N10O52P2S	617.7362	0.5	52.3	76	54.66	35.0	even	ok
	77	C59H42N36NaO32S2	617.7363	0.7	52.4	77	51.73	58.0	even	ok
	78	C63H187N9O45PS	617.7354	-0.7	52.5	78	51.45	-23.0	even	ok
	79	C57H37N42O30S2	617.7362	0.5	52.6	79	53.78	62.0	even	ok
	80	C61H76NaO58P2S	617.7358	-0.1	52.6	80	60.73	26.0	even	ok
	81	C66H188N11O37P2S2	617.7356	-0.4	52.9	81	54.66	-19.0	even	ok
	82	C69H196NO43P2S2	617.7360	0.3	53.0	82	56.60	-25.0	even	ok
	83	C50H59N26O44P2S2	617.7360	0.1	53.1	83	58.64	37.0	even	ok
	84	C48H30N56O23PS2	617.7354	-0.8	53.1	84	49.54	64.0	even	ok
	85	C68H193N5NaO39P2S2	617.7357	-0.3	53.3	85	56.23	-23.0	even	ok
	86	C62H55N20NaO43PS	617.7357	-0.2	53.5	86	56.55	47.0	even	ok

Compound Spectrum SmartFormula Report

Meas. m/z	#	Ion Formula	m/z	err [ppm]	mSigma	# mSigma	Score	rdb	e⁻ Conf	N-Rule
	87	C51H31N54NaO21PS2	617.7359	0.1	53.5	87	58.31	65.0	even	ok
	88	C67H58N12NaO48S	617.7356	-0.4	53.7	88	53.73	46.0	even	ok
	89	C52H71N12O54P2S2	617.7360	0.2	53.8	89	57.20	26.0	even	ok
	90	C66H188N7NaO43PS	617.7360	0.2	53.9	90	56.82	-22.0	even	ok
	91	C55H164N41NaO21PS2	617.7362	0.5	54.0	91	51.41	-4.0	even	ok
	92	C56H34N46NaO26S2	617.7358	-0.0	54.0	92	58.40	64.0	even	ok
	93	C48H47N40O34P2S2	617.7360	0.1	54.1	93	56.80	48.0	even	ok
	94	C65H53N18O46S	617.7355	-0.6	54.2	94	50.76	50.0	even	ok
	95	C54H29N52O24S2	617.7357	-0.2	54.5	95	55.41	68.0	even	ok
	96	C60H60N18NaO44P2S	617.7363	0.7	54.6	96	48.77	42.0	even	ok
	97	C59H71N6O56P2S	617.7357	-0.2	54.6	97	54.84	30.0	even	ok
	98	C49H26N60O19PS2	617.7358	-0.0	54.8	98	57.19	69.0	even	ok
	99	C60H50N26O41PS	617.7356	-0.4	54.8	99	52.09	51.0	even	ok
	100	C48H40N48NaO26P2S2	617.7360	0.3	54.9	100	53.34	55.0	even	ok
	101	C64H183N13O41PS	617.7359	0.0	54.9	101	57.00	-18.0	even	ok
	102	C53H159N47O19PS2	617.7361	0.4	55.0	102	51.80	0.0	even	ok
	103	C59H64N14NaO48P2S	617.7358	-0.1	55.3	103	55.76	37.0	even	ok
	104	C49H56N30NaO40P2S2	617.7356	-0.4	55.8	104	49.92	39.0	even	ok
	105	C47H31N58O20P2S2	617.7364	0.9	56.1	105	43.66	64.0	even	ok
	106	C51H68N16NaO50P2S2	617.7356	-0.4	56.2	106	49.37	28.0	even	ok
	107	C53H26N56NaO20S2	617.7354	-0.8	56.3	107	44.50	70.0	even	ok
	108	C58H55N24O42P2S	617.7362	0.5	56.3	108	48.07	46.0	even	ok
	109	C69H186N5O46S	617.7358	-0.1	56.3	109	52.89	-19.0	even	ok
	110	C60H167N33NaO26S2	617.7361	0.4	56.5	110	49.54	-5.0	even	ok
	111	C58H162N39O24S2	617.7360	0.2	56.7	111	51.24	-1.0	even	ok
	112	C46H35N54O24P2S2	617.7359	0.1	56.9	112	52.06	59.0	even	ok
	113	C63H51N28O33P2S2	617.7358	-0.1	56.9	113	52.20	55.0	even	ok
	114	C63H51N24NaO39PS	617.7362	0.5	57.0	114	47.35	52.0	even	ok
	115	C66H59N18O39P2S2	617.7362	0.6	57.0	115	45.80	49.0	even	ok
	116	C47H44N44NaO30P2S2	617.7356	-0.4	57.1	116	47.76	50.0	even	ok
	117	C52H27N58NaO17PS2	617.7364	0.8	57.2	117	42.44	70.0	even	ok
	118	C57H59N20O46P2S	617.7357	-0.2	57.2	118	50.33	41.0	even	ok
	119	C65H56N22NaO35P2S2	617.7359	0.0	57.3	119	52.86	51.0	even	ok
	120	C63H180N17NaO37PS	617.7355	-0.6	57.5	120	45.60	-16.0	even	ok
	121	C60H43N38O27P2S2	617.7353	-0.9	57.5	121	41.74	61.0	even	ok
	122	C48H23N64NaO15PS2	617.7355	-0.6	57.5	122	44.85	71.0	even	ok
	123	C52H156N51NaO15PS2	617.7357	-0.2	57.6	123	50.11	2.0	even	ok
	124	C62H48N32NaO29P2S2	617.7354	-0.7	57.6	124	43.52	57.0	even	ok
	125	C58H68N10NaO52P2S	617.7354	-0.8	57.6	125	42.40	32.0	even	ok
	126	C68H64N12NaO41P2S2	617.7363	0.8	57.7	126	42.81	45.0	even	ok
	127	C47H51N36O38P2S2	617.7355	-0.6	57.9	127	44.73	43.0	even	ok
	128	C68H183N9NaO42S	617.7354	-0.7	57.9	128	42.88	-17.0	even	ok
	129	C61H46N30O37PS	617.7361	0.3	58.0	129	47.74	56.0	even	ok
	130	C50H22N64O15PS2	617.7363	0.7	58.1	130	42.98	74.0	even	ok
	131	C49H63N22O48P2S2	617.7355	-0.6	58.2	131	44.34	32.0	even	ok
	132	C53H80N2NaO60P2S2	617.7356	-0.4	58.3	132	46.22	17.0	even	ok

Compound Spectrum SmartFormula Report

Meas. m/z	#	Ion Formula	m/z	err [ppm]	mSigma	# mSigma	Score	rdb	e⁻ Conf	N-Rule
	133	C57H159N43NaO20S2	617.7356	-0.4	58.3	133	46.87	1.0	even	ok
	134	C67H184N11NaO39PS	617.7364	0.9	58.3	134	40.16	-17.0	even	ok
	135	C66H178N15O40S	617.7353	-0.9	58.5	135	40.31	-13.0	even	ok
	136	C55H154N49O18S2	617.7355	-0.5	58.8	136	44.25	5.0	even	ok
	137	C61H175N23O35PS	617.7354	-0.7	58.8	137	41.82	-12.0	even	ok
	138	C69H57N12O48S	617.7364	0.9	58.9	138	39.41	49.0	even	ok
	139	C46H18N70O13PS2	617.7354	-0.8	59.0	139	40.87	75.0	even	ok
	140	C65H179N17O37PS	617.7363	0.7	59.1	140	40.96	-13.0	even	ok
	141	C45H39N50O28P2S2	617.7355	-0.6	59.2	141	42.74	54.0	even	ok
	142	C46H28N62NaO16P2S2	617.7360	0.3	59.2	142	46.38	66.0	even	ok
	143	C57H52N28NaO38P2S	617.7358	-0.1	59.4	143	48.56	48.0	even	ok
	144	C45H32N58NaO20P2S2	617.7356	-0.4	60.0	144	43.30	61.0	even	ok
	145	C57H74NaO65S	617.7362	0.6	60.0	145	41.36	22.0	even	ok
	146	C68H54N16NaO44S	617.7361	0.3	60.1	146	44.69	51.0	even	ok
	147	C58H48N32NaO34P2S	617.7363	0.6	60.1	147	40.76	53.0	even	ok
	148	C51H75N8O58P2S2	617.7355	-0.6	60.1	148	41.63	21.0	even	ok
	149	C66H49N22O42S	617.7360	0.2	60.2	149	46.23	55.0	even	ok
	150	C55H62N14NaO55S	617.7362	0.6	60.2	150	41.15	33.0	even	ok
	151	C69H63N10NaO44PS2	617.7353	-0.9	60.3	151	37.99	45.0	even	ok
	152	C71H67NaO55P	617.7355	-0.6	60.3	152	40.89	40.0	even	ok
	153	C56H56N24NaO42P2S	617.7354	-0.8	60.4	153	38.59	43.0	even	ok
	154	C60H43N34NaO33PS	617.7357	-0.2	60.5	154	44.92	58.0	even	ok
	155	C57H30N50NaO22S2	617.7363	0.7	60.5	155	39.84	69.0	even	ok
	156	C49H19N68NaO11PS2	617.7359	0.1	60.6	156	46.26	76.0	even	ok
	157	C55H25N56O20S2	617.7362	0.5	60.6	157	41.39	73.0	even	ok
	158	C67H184N15O33P2S2	617.7360	0.3	60.7	158	44.07	-14.0	even	ok
	159	C70H66N6O48PS2	617.7357	-0.3	60.8	159	43.97	43.0	even	ok
	160	C64H176N25O27P2S2	617.7356	-0.4	60.9	160	42.04	-8.0	even	ok
	161	C60H176N25O32P2S	617.7364	0.9	61.1	161	36.47	-12.0	even	ok
	162	C44H23N68O14P2S2	617.7359	0.1	61.1	162	45.28	70.0	even	ok
	163	C66H181N19NaO29P2S2	617.7357	-0.3	61.2	163	43.32	-12.0	even	ok
	164	C69H189N9NaO35P2S2	617.7361	0.4	61.3	164	41.44	-18.0	even	ok
	165	C55H47N34O36P2S	617.7357	-0.2	61.3	165	43.83	52.0	even	ok
	166	C64H176N21NaO33PS	617.7360	0.2	61.3	166	44.48	-11.0	even	ok
	167	C44H45N40O41S2	617.7364	0.8	61.4	167	37.15	44.0	even	ok
	168	C53H152N55NaO11PS2	617.7362	0.5	61.6	168	40.09	7.0	even	ok
	169	C69H62N6O53P	617.7354	-0.8	61.6	169	37.53	44.0	even	ok
	170	C66H71O58P2	617.7355	-0.6	61.6	170	39.40	34.0	even	ok
	171	C45H19N72O10P2S2	617.7364	0.9	61.6	171	36.37	75.0	even	ok
	172	C58H38N40O31PS	617.7356	-0.4	61.7	172	41.40	62.0	even	ok
	173	C56H43N38O32P2S	617.7362	0.5	61.7	173	40.20	57.0	even	ok
	174	C65H46N26NaO38S	617.7356	-0.4	61.8	174	41.12	57.0	even	ok
	175	C42H33N54O31S2	617.7364	0.8	61.8	175	36.76	55.0	even	ok
	176	C47H14N74O9PS2	617.7358	-0.0	61.8	176	45.27	80.0	even	ok
	177	C67H67N4O54P2	617.7359	0.1	61.9	177	44.04	39.0	even	ok
	178	C53H50N28NaO45S	617.7362	0.6	62.0	178	38.81	44.0	even	ok

Compound Spectrum SmartFormula Report

Meas. m/z	#	Ion Formula	m/z	err [ppm]	mSigma	# mSigma	Score	rdb	e⁻ Conf	N-Rule
	179	C72H71NaO50PS2	617.7358	-0.1	62.0	179	43.82	39.0	even	ok
	180	C43H27N64O18P2S2	617.7355	-0.6	62.1	180	38.72	65.0	even	ok
	181	C55H69N6O63S	617.7361	0.4	62.1	181	40.18	26.0	even	ok
	182	C54H22N60NaO16S2	617.7358	-0.1	62.2	182	44.53	75.0	even	ok
	183	C63H41N32O36S	617.7355	-0.6	62.2	183	38.84	61.0	even	ok
	184	C63H65N4O61	617.7364	0.8	62.3	184	36.10	35.0	even	ok
	185	C62H171N27O31PS	617.7359	0.0	62.3	185	44.64	-7.0	even	ok
	186	C73H66O55P	617.7363	0.7	62.3	186	37.21	43.0	even	ok
	187	C53H57N20O53S	617.7361	0.4	62.4	187	39.90	37.0	even	ok
	188	C51H147N61O9PS2	617.7361	0.4	62.5	188	40.41	11.0	even	ok
	189	C52H17N66O14S2	617.7357	-0.2	62.6	189	42.25	79.0	even	ok
	190	C46H57N26O51S2	617.7364	0.8	62.6	190	35.60	33.0	even	ok
	191	C70H182N9O42S	617.7362	0.6	63.1	191	37.39	-14.0	even	ok
	192	C72H187N3NaO44S	617.7363	0.7	63.2	192	35.73	-18.0	even	ok
	193	C68H63N8O50P2	617.7364	0.9	63.7	193	33.87	44.0	even	ok
	194	C40H21N68O21S2	617.7364	0.8	63.7	194	34.51	66.0	even	ok
	195	C71H191N3O42PS2	617.7355	-0.6	64.0	195	36.13	-20.0	even	ok
	196	C59H173N29NaO28P2S	617.7361	0.3	64.0	196	38.78	-10.0	even	ok
	197	C51H45N34O43S	617.7361	0.4	64.1	197	37.59	48.0	even	ok
	198	C43H42N44NaO37S2	617.7360	0.2	64.2	198	39.61	46.0	even	ok
	199	C43H20N72NaO10P2S2	617.7356	-0.4	64.3	199	37.29	72.0	even	ok
	200	C69H179N13NaO38S	617.7359	0.0	64.4	200	41.66	-12.0	even	ok
	201	C51H14N70NaO10S2	617.7354	-0.8	64.4	201	33.73	81.0	even	ok
	202	C72H63N8O45P2S	617.7356	-0.5	64.5	202	36.52	48.0	even	ok
	203	C72H63N4NaO51P	617.7359	0.1	64.6	203	40.29	45.0	even	ok
	204	C54H44N38NaO32P2S	617.7354	-0.8	64.6	204	33.37	54.0	even	ok
	205	C62H69O65	617.7359	0.1	64.6	205	40.60	30.0	even	ok
	206	C67H174N19O36S	617.7358	-0.1	64.6	206	39.88	-8.0	even	ok
	207	C61H39N38NaO29PS	617.7362	0.5	64.8	207	36.30	63.0	even	ok
	208	C41H30N58NaO27S2	617.7360	0.2	64.8	208	38.81	57.0	even	ok
	209	C74H68N2NaO47P2S	617.7357	-0.3	64.8	209	37.63	44.0	even	ok
	210	C55H40N42NaO28P2S	617.7358	-0.1	64.9	210	40.24	59.0	even	ok
	211	C61H168N31NaO27PS	617.7355	-0.6	64.9	211	35.42	-5.0	even	ok
	212	C58H155N47NaO16S2	617.7361	0.4	64.9	212	37.36	6.0	even	ok
	213	C54H66N10NaO59S	617.7358	-0.1	64.9	213	39.71	28.0	even	ok
	214	C64H47N32O29P2S2	617.7362	0.6	64.9	214	34.97	60.0	even	ok
	215	C66H64N8NaO50P2	617.7356	-0.4	65.0	215	36.46	41.0	even	ok
	216	C50H144N65NaO5PS2	617.7357	-0.2	65.0	216	38.78	13.0	even	ok
	217	C45H54N30NaO47S2	617.7360	0.2	65.1	217	38.37	35.0	even	ok
	218	C56H150N53O14S2	617.7360	0.2	65.1	218	38.61	10.0	even	ok
	219	C61H39N42O23P2S2	617.7358	-0.1	65.1	219	39.39	66.0	even	ok
	220	C50H15N72NaO7PS2	617.7364	0.8	65.1	220	32.42	81.0	even	ok
	221	C51H38N42NaO35S	617.7362	0.6	65.2	221	34.76	55.0	even	ok
	222	C48H69N12O61S2	617.7364	0.8	65.3	222	32.39	22.0	even	ok
	223	C64H190NO55	617.7362	0.5	65.3	223	35.60	-28.0	even	ok
	224	C52H54N24NaO49S	617.7358	-0.1	65.4	224	38.93	39.0	even	ok

Compound Spectrum SmartFormula Report

Meas. m/z	#	Ion Formula	m/z	err [ppm]	mSigma	# mSigma	Score	rdb	e⁻ Conf	N-Rule
	225	C63H44N36NaO25P2S2	617.7359	0.0	65.4	225	40.05	62.0	even	ok
	226	C70H58N10O49P	617.7358	-0.0	65.5	226	39.80	49.0	even	ok
	227	C66H52N26NaO31P2S2	617.7363	0.7	65.6	227	32.75	56.0	even	ok
	228	C62H62N8NaO57	617.7360	0.2	65.6	228	37.82	37.0	even	ok
	229	C57H168N35O26P2S	617.7360	0.2	65.7	229	38.09	-6.0	even	ok
	230	C59H34N44O27PS	617.7361	0.3	65.7	230	36.61	67.0	even	ok
	231	C68H192NO48P2	617.7358	-0.2	65.7	231	38.06	-24.0	even	ok
	232	C58H31N52O17P2S2	617.7353	-0.9	65.9	232	31.24	72.0	even	ok
	233	C48H10N78O5PS2	617.7363	0.7	66.0	233	32.84	85.0	even	ok
	234	C60H36N46NaO19P2S2	617.7354	-0.7	66.0	234	32.60	68.0	even	ok
	235	C59H163N37O25PS	617.7354	-0.7	66.1	235	32.50	-1.0	even	ok
	236	C48H139N71O3PS2	617.7356	-0.4	66.2	236	35.74	17.0	even	ok
	237	C66H171N23NaO32S	617.7354	-0.7	66.3	237	32.13	-6.0	even	ok
	238	C41H37N50O35S2	617.7359	0.1	66.4	238	38.14	50.0	even	ok
	239	C46H11N78NaO5PS2	617.7355	-0.6	66.4	239	32.98	82.0	even	ok
	240	C65H172N25NaO29PS	617.7364	0.9	66.5	240	30.31	-6.0	even	ok
	241	C67H60N12NaO46P2	617.7360	0.3	66.6	241	35.91	46.0	even	ok
	242	C61H53N18O51	617.7364	0.8	66.6	242	31.10	46.0	even	ok
	243	C56H36N46NaO24P2S	617.7363	0.6	66.6	243	32.51	64.0	even	ok
	244	C53H35N48O26P2S	617.7357	-0.2	66.6	244	36.33	63.0	even	ok
	245	C55H147N57NaO10S2	617.7356	-0.4	66.7	245	35.00	12.0	even	ok
	246	C64H166N29O30S	617.7353	-0.9	66.8	246	30.20	-2.0	even	ok
	247	C64H59N14O48P2	617.7355	-0.6	66.8	247	32.79	45.0	even	ok
	248	C44H16N76NaO6P2S2	617.7360	0.3	66.8	248	35.69	77.0	even	ok
	249	C39H18N72NaO17S2	617.7360	0.2	66.9	249	36.11	68.0	even	ok
	250	C39H25N64O25S2	617.7359	0.1	67.0	250	37.30	61.0	even	ok
	251	C52H61N16O57S	617.7357	-0.3	67.0	251	35.37	32.0	even	ok
	252	C43H49N36O45S2	617.7359	0.1	67.1	252	37.04	39.0	even	ok
	253	C53H142N63O8S2	617.7355	-0.5	67.1	253	33.04	16.0	even	ok
	254	C63H167N31O27PS	617.7363	0.7	67.2	254	30.92	-2.0	even	ok
	255	C49H33N48O33S	617.7361	0.4	67.3	255	33.64	59.0	even	ok
	256	C67H45N26O38S	617.7364	0.9	67.3	256	29.45	60.0	even	ok
	257	C50H42N38NaO39S	617.7358	-0.1	67.4	257	36.25	50.0	even	ok
	258	C47H66N16NaO57S2	617.7360	0.2	67.4	258	35.30	24.0	even	ok
	259	C50H49N30O47S	617.7357	-0.3	67.6	259	34.61	43.0	even	ok
	260	C60H57N14O55	617.7359	0.1	67.7	260	36.47	41.0	even	ok
	261	C61H66N4NaO61	617.7356	-0.5	67.8	261	32.39	32.0	even	ok
	262	C44H6N84O3PS2	617.7354	-0.8	67.8	262	29.99	86.0	even	ok
	263	C54H73N2O67S	617.7357	-0.3	67.9	263	34.33	21.0	even	ok
	264	C69H188N5O44P2	617.7362	0.5	67.9	264	31.84	-19.0	even	ok
	265	C67H51N24NaO34PS2	617.7353	-0.9	68.0	265	28.95	56.0	even	ok
	266	C69H55N14NaO45P	617.7355	-0.6	68.0	266	31.22	51.0	even	ok
	267	C58H164N39O22P2S	617.7364	0.9	68.0	267	28.59	-1.0	even	ok
	268	C54H31N52O22P2S	617.7362	0.5	68.2	268	32.08	68.0	even	ok
	269	C65H55N18O44P2	617.7359	0.1	68.2	269	35.28	50.0	even	ok
	270	C58H31N48NaO23PS	617.7357	-0.3	68.2	270	34.17	69.0	even	ok

Compound Spectrum SmartFormula Report

Meas. m/z	#	Ion Formula	m/z	err [ppm]	mSigma	# mSigma	Score	rdb	e⁻ Conf	N-Rule
	271	C68H54N20O38PS2	617.7357	-0.3	68.2	271	33.79	54.0	even	ok
	272	C64H183N9NaO47	617.7363	0.6	68.2	272	30.76	-21.0	even	ok
	273	C75H61N2O54	617.7357	-0.2	68.3	273	34.55	48.0	even	ok
	274	C41H15N78O8P2S2	617.7355	-0.6	68.3	274	30.94	76.0	even	ok
	275	C66H42N30NaO34S	617.7361	0.3	68.6	275	33.17	62.0	even	ok
	276	C63H187N5NaO51	617.7358	-0.1	68.6	276	35.10	-26.0	even	ok
	277	C42H11N82O4P2S2	617.7359	0.1	68.6	277	34.76	81.0	even	ok
	278	C64H37N36O32S	617.7360	0.2	68.7	278	34.29	66.0	even	ok
	279	C73H188N5O39P2S	617.7354	-0.8	68.8	279	28.63	-15.0	even	ok
	280	C56H165N39NaO22P2S	617.7356	-0.4	68.8	280	32.16	-4.0	even	ok
	281	C67H189N5NaO44P2	617.7354	-0.8	68.9	281	28.98	-22.0	even	ok
	282	C38H9N82O11S2	617.7364	0.8	69.0	282	28.57	77.0	even	ok
	283	C65H172N29O23P2S2	617.7360	0.3	69.0	283	32.93	-3.0	even	ok
	284	C55H18N64NaO12S2	617.7363	0.7	69.1	284	29.47	80.0	even	ok
	285	C67H50N20O43P	617.7354	-0.8	69.1	285	28.66	55.0	even	ok
	286	C73H188NNaO45P	617.7357	-0.2	69.2	286	33.46	-18.0	even	ok
	287	C53H13N70O10S2	617.7362	0.5	69.2	287	30.60	84.0	even	ok
	288	C40H34N54NaO31S2	617.7356	-0.5	69.3	288	30.74	52.0	even	ok
	289	C45H61N22O55S2	617.7359	0.1	69.3	289	34.18	28.0	even	ok
	290	C56H26N54O21PS	617.7356	-0.4	69.3	290	31.50	73.0	even	ok
	291	C70H59N14NaO40PS2	617.7358	-0.1	69.3	291	33.74	50.0	even	ok
	292	C62H164N39O17P2S2	617.7356	-0.4	69.4	292	31.06	3.0	even	ok
	293	C62H164N35NaO23PS	617.7360	0.2	69.4	293	33.39	0.0	even	ok
	294	C67H177N23NaO25P2S2	617.7361	0.4	69.5	294	31.01	-7.0	even	ok
	295	C48H37N44O37S	617.7357	-0.3	69.5	295	32.19	54.0	even	ok
	296	C64H169N33NaO19P2S2	617.7357	-0.3	69.6	296	32.04	-1.0	even	ok
	297	C49H26N56NaO25S	617.7362	0.6	69.7	297	29.62	66.0	even	ok
	298	C51H140N69NaOPS2	617.7362	0.5	69.8	298	29.99	18.0	even	ok
	299	C42H46N40NaO41S2	617.7356	-0.5	69.8	299	30.24	41.0	even	ok
	300	C52H32N52NaO22P2S	617.7354	-0.8	69.9	300	27.48	65.0	even	ok
	301	C74H58N6NaO50	617.7354	-0.8	69.9	301	27.75	50.0	even	ok
	302	C60H50N22NaO47	617.7360	0.2	69.9	302	32.36	48.0	even	ok
	303	C71H62N10O44PS2	617.7361	0.4	69.9	303	30.51	48.0	even	ok
	304	C51H58N20NaO53S	617.7353	-0.9	69.9	304	27.04	34.0	even	ok
	305	C59H61N10O59	617.7355	-0.6	70.0	305	28.65	36.0	even	ok
	306	C73H59N8NaO47P	617.7364	0.8	70.0	306	27.08	50.0	even	ok
	307	C71H183N7O43P	617.7356	-0.3	70.1	307	31.05	-14.0	even	ok
	308	C62H178N15O45	617.7362	0.5	70.1	308	29.91	-17.0	even	ok
	309	C38H22N68NaO21S2	617.7356	-0.5	70.2	309	29.69	63.0	even	ok
	310	C43H7N86P2S2	617.7364	0.8	70.2	310	26.80	86.0	even	ok
	311	C47H7N82NaOPS2	617.7359	0.1	70.3	311	32.86	87.0	even	ok
	312	C63H34N40NaO28S	617.7356	-0.4	70.3	312	30.22	68.0	even	ok
	313	C60H159N41O21PS	617.7359	0.0	70.4	313	33.52	4.0	even	ok
	314	C53H70N6NaO63S	617.7353	-0.8	70.5	314	26.53	23.0	even	ok
	315	C54H160N45O20P2S	617.7355	-0.5	70.6	315	28.91	0.0	even	ok
	316	C48H30N52NaO29S	617.7358	-0.1	70.6	316	32.10	61.0	even	ok

Compound Spectrum SmartFormula Report

Meas. m/z	#	Ion Formula	m/z	err [ppm]	mSigma	# mSigma	Score	rdb	e¯ Conf	N-Rule
	317	C65H184N11O42P2	617.7353	-0.9	70.6	317	25.95	-18.0	even	ok
	318	C61H182N11O49	617.7357	-0.2	70.7	318	31.28	-22.0	even	ok
	319	C71H54N14O45P	617.7363	0.7	70.7	319	27.63	54.0	even	ok
	320	C49H46N34NaO43S	617.7353	-0.9	70.7	320	26.21	45.0	even	ok
	321	C61H29N46O26S	617.7355	-0.6	70.7	321	28.54	72.0	even	ok
	322	C52H10N74NaO6S2	617.7358	-0.1	70.8	322	32.63	86.0	even	ok
	323	C68H185N9NaO40P2	617.7358	-0.0	70.9	323	32.71	-17.0	even	ok
	324	C66H51N22O40P2	617.7364	0.9	70.9	324	26.09	55.0	even	ok
	325	C49H78N2NaO67S2	617.7360	0.2	70.9	325	30.90	13.0	even	ok
	326	C57H161N43NaO18P2S	617.7361	0.3	70.9	326	30.24	1.0	even	ok
	327	C59H54N18NaO51	617.7356	-0.5	71.0	327	28.78	43.0	even	ok
	328	C37H13N78O15S2	617.7359	0.1	71.0	328	32.24	72.0	even	ok
	329	C50H5N80O4S2	617.7357	-0.2	71.2	329	30.94	90.0	even	ok
	330	C53H28N56NaO18P2S	617.7358	-0.1	71.2	330	31.84	70.0	even	ok
	331	C64H52N22NaO40P2	617.7356	-0.4	71.3	331	28.96	52.0	even	ok
	332	C73H67N4NaO46PS2	617.7362	0.6	71.3	332	27.82	44.0	even	ok
	333	C38H29N60O29S2	617.7355	-0.6	71.5	333	27.14	56.0	even	ok
	334	C44H58N26NaO51S2	617.7356	-0.5	71.6	334	28.29	30.0	even	ok
	335	C47H21N62O23S	617.7361	0.4	71.7	335	28.66	70.0	even	ok
	336	C69H179N17O32PS2	617.7355	-0.6	71.8	336	27.15	-9.0	even	ok
	337	C68H170N23O32S	617.7362	0.6	71.8	337	27.37	-3.0	even	ok
	338	C70H175N17NaO34S	617.7363	0.7	71.8	338	26.17	-7.0	even	ok
	339	C41H8N86NaP2S2	617.7356	-0.4	71.9	339	28.26	83.0	even	ok
	340	C40H41N46O39S2	617.7355	-0.6	71.9	340	26.77	45.0	even	ok
	341	C58H45N28O45	617.7359	0.1	71.9	341	31.19	52.0	even	ok
	342	C59H41N32O41	617.7363	0.8	72.0	342	25.54	57.0	even	ok
	343	C37H6N86NaO7S2	617.7360	0.2	72.4	343	29.55	79.0	even	ok
	344	C36H17N74O19S2	617.7355	-0.7	72.4	344	26.17	67.0	even	ok
	345	C66H180N15O38P2	617.7357	-0.2	72.4	345	29.71	-13.0	even	ok
	346	C55H156N49O16P2S	617.7360	0.2	72.5	346	29.71	5.0	even	ok
	347	C47H73N8O65S2	617.7359	0.1	72.7	347	30.02	17.0	even	ok
	348	C73H59N12O41P2S	617.7360	0.2	72.8	348	28.97	53.0	even	ok
	349	C46H25N58O27S	617.7357	-0.3	72.8	349	28.48	65.0	even	ok
	350	C71H184N11NaO34PS2	617.7356	-0.5	72.8	350	27.26	-13.0	even	ok
	351	C47H34N48NaO33S	617.7353	-0.9	72.8	351	24.17	56.0	even	ok
	352	C59H156N45NaO17PS	617.7355	-0.6	72.9	352	26.38	6.0	even	ok
	353	C70H51N18NaO41P	617.7359	0.1	72.9	353	29.75	56.0	even	ok
	354	C51H23N62O16P2S	617.7357	-0.2	72.9	354	28.76	74.0	even	ok
	355	C62H47N28O38P2	617.7355	-0.6	72.9	355	26.05	56.0	even	ok
	356	C57H49N24O49	617.7355	-0.7	73.1	356	25.44	47.0	even	ok
	357	C67H167N27NaO28S	617.7359	-0.0	73.1	357	30.26	-1.0	even	ok
	358	C70H51N22O35P2S	617.7356	-0.5	73.2	358	26.56	59.0	even	ok
	359	C59H27N52NaO19PS	617.7362	0.5	73.2	359	26.71	74.0	even	ok
	360	C72H187N7O38PS2	617.7359	0.1	73.2	360	29.29	-15.0	even	ok
	361	C75H64N6NaO43P2S	617.7361	0.4	73.3	361	27.29	49.0	even	ok
	362	C65H162N33O26S	617.7358	-0.2	73.4	362	28.90	3.0	even	ok

Compound Spectrum SmartFormula Report

Meas. m/z	#	Ion Formula	m/z	err [ppm]	mSigma	# mSigma	Score	rdb	e⁻ Conf	N-Rule
	363	C62H35N46O19P2S2	617.7362	0.6	73.4	363	25.64	71.0	even	ok
	364	C72H56N16NaO37P2S	617.7356	-0.3	73.4	364	27.40	55.0	even	ok
	365	C61H175N19NaO41	617.7358	-0.1	73.5	365	29.21	-15.0	even	ok
	366	C42H53N32O49S2	617.7355	-0.6	73.6	366	25.12	34.0	even	ok
	367	C56H143N61NaO6S2	617.7361	0.4	73.7	367	27.07	17.0	even	ok
	368	C68H46N24O39P	617.7358	-0.0	73.7	368	29.31	60.0	even	ok
	369	C59H27N56O13P2S2	617.7358	-0.1	73.8	369	28.55	77.0	even	ok
	370	C65H48N26NaO36P2	617.7360	0.3	73.8	370	27.51	57.0	even	ok
	371	C54H138N67O4S2	617.7360	0.2	73.9	371	27.96	21.0	even	ok
	372	C54H24N60NaO14P2S	617.7363	0.6	73.9	372	24.82	75.0	even	ok
	373	C64H40N40NaO21P2S2	617.7363	0.7	74.0	373	24.04	67.0	even	ok
	374	C57H151N51O15PS	617.7354	-0.7	74.0	374	24.21	10.0	even	ok
	375	C60H179N15NaO45	617.7354	-0.8	74.0	375	23.42	-20.0	even	ok
	376	C57H22N58O17PS	617.7361	0.3	74.0	376	26.95	78.0	even	ok
	377	C61H32N50NaO15P2S2	617.7359	0.0	74.1	377	29.15	73.0	even	ok
	378	C62H171N23NaO37	617.7362	0.6	74.1	378	24.67	-10.0	even	ok
	379	C69H181N13NaO36P2	617.7363	0.7	74.2	379	24.17	-12.0	even	ok
	380	C50H59N22NaO50PS	617.7363	0.8	74.4	380	23.54	34.0	even	ok
	381	C36H10N82NaO11S2	617.7355	-0.5	74.4	381	25.27	74.0	even	ok
	382	C74H192NNaO40PS2	617.7360	0.3	74.4	382	26.86	-19.0	even	ok
	383	C46H70N12NaO61S2	617.7356	-0.5	74.7	385	25.20	19.0	even	ok
	384	C56H19N66O7P2S2	617.7353	-0.9	74.7	386	22.47	83.0	even	ok
	385	C74H184N5NaO41P	617.7362	0.5	74.8	387	24.78	-13.0	even	ok
	386	C58H24N60NaO9P2S2	617.7354	-0.7	74.8	388	23.47	79.0	even	ok
	387	C52H71N8NaO60PS	617.7363	0.8	74.9	389	23.01	23.0	even	ok
	388	C64H159N37NaO22S	617.7354	-0.7	75.0	390	23.14	5.0	even	ok
	389	C46H18N66NaO19S	617.7358	-0.2	75.1	391	27.08	72.0	even	ok
	390	C48H47N36NaO40PS	617.7363	0.8	75.1	392	22.92	45.0	even	ok
	391	C63H160N39NaO19PS	617.7364	0.9	75.2	393	21.98	5.0	even	ok
	392	C63H43N32O34P2	617.7359	0.1	75.3	394	27.04	61.0	even	ok
	393	C58H38N36NaO37	617.7360	0.2	75.3	395	26.40	59.0	even	ok
	394	C57H42N32NaO41	617.7355	-0.5	75.3	396	24.37	54.0	even	ok
	395	C52H19N66O12P2S	617.7362	0.5	75.4	397	24.49	79.0	even	ok
	396	C72H179N11O39P	617.7361	0.4	75.4	398	25.17	-9.0	even	ok
	397	C59H170N25O39	617.7357	-0.2	75.4	399	26.03	-11.0	even	ok
	398	C62H154N43O20S	617.7353	-0.9	75.5	400	21.74	9.0	even	ok
	399	C67H176N19O34P2	617.7362	0.5	75.5	401	24.00	-8.0	even	ok
	400	C53H135N71NaS2	617.7356	-0.4	75.5	402	25.12	23.0	even	ok
	401	C77H66O50PS	617.7355	-0.7	75.5	403	23.20	47.0	even	ok
	402	C65H177N19NaO34P2	617.7354	-0.8	75.5	404	22.50	-11.0	even	ok
	403	C76H57N6O50	617.7362	0.5	75.6	405	24.00	53.0	even	ok
	404	C78H62NaO52	617.7363	0.7	75.6	406	22.95	49.0	even	ok
	405	C56H152N53O12P2S	617.7364	0.9	75.7	407	21.48	10.0	even	ok
	406	C59H71N2NaO62P	617.7361	0.4	75.7	408	24.80	27.0	even	ok
	407	C61H155N45O17PS	617.7363	0.7	75.8	409	22.42	9.0	even	ok
	408	C60H166N29O35	617.7362	0.5	76.0	410	23.99	-6.0	even	ok

Compound Spectrum SmartFormula Report

Meas. m/z	#	Ion Formula	m/z	err [ppm]	mSigma	# mSigma	Score	rdb	e¯ Conf	N-Rule
	409	C65H33N40O28S	617.7364	0.9	76.2	411	21.15	71.0	even	ok
	410	C45H22N62NaO23S	617.7353	-0.9	76.2	412	21.21	67.0	even	ok
	411	C67H43N28NaO35P	617.7355	-0.6	76.2	413	22.86	62.0	even	ok
	412	C65H39N38NaO24PS2	617.7353	-0.9	76.2	414	21.17	67.0	even	ok
	413	C66H42N34O28PS2	617.7357	-0.3	76.2	415	24.90	65.0	even	ok
	414	C56H19N62NaO13PS	617.7357	-0.3	76.5	416	24.94	80.0	even	ok
	415	C44H65N18O59S2	617.7355	-0.6	76.5	417	22.44	23.0	even	ok
	416	C48H54N28O48PS	617.7362	0.6	76.6	418	22.56	38.0	even	ok
	417	C34H5N88O9S2	617.7355	-0.7	76.6	419	22.21	78.0	even	ok
	418	C75H54N10NaO46	617.7358	-0.1	76.9	420	25.80	55.0	even	ok
	419	C74H184N9O35P2S	617.7358	-0.1	77.0	421	25.50	-10.0	even	ok
	420	C50H66N14O58PS	617.7362	0.6	77.1	422	22.10	27.0	even	ok
	421	C46H35N50NaO30PS	617.7363	0.7	77.1	423	21.25	56.0	even	ok
	422	C73H49N16O44	617.7357	-0.2	77.1	424	24.64	59.0	even	ok
	423	C63H172N25O32P2	617.7353	-0.9	77.2	425	20.15	-7.0	even	ok
	424	C68H47N28NaO30PS2	617.7358	-0.1	77.2	426	24.91	61.0	even	ok
	425	C56H33N42O35	617.7359	0.1	77.2	427	25.44	63.0	even	ok
	426	C65H38N34O33P	617.7354	-0.8	77.2	428	20.99	66.0	even	ok
	427	C46H42N42O38PS	617.7362	0.6	77.3	429	21.92	49.0	even	ok
	428	C55H37N38O39	617.7355	-0.7	77.4	430	21.53	58.0	even	ok
	429	C64H30N44NaO24S	617.7361	0.3	77.5	431	23.67	73.0	even	ok
	430	C76H189N3NaO37P2S	617.7359	0.1	77.5	432	25.15	-14.0	even	ok
	431	C54H14N68O11PS	617.7356	-0.4	77.5	433	23.00	84.0	even	ok
	432	C57H59N16NaO52P	617.7361	0.4	77.5	434	23.07	38.0	even	ok
	433	C69H50N24O34PS2	617.7361	0.4	77.6	435	22.77	59.0	even	ok
	434	C62H25N50O22S	617.7360	0.2	77.6	436	24.45	77.0	even	ok
	435	C71H176N19O29P2S	617.7354	-0.8	77.6	437	20.39	-4.0	even	ok
	436	C71H176N15NaO35P	617.7357	-0.2	77.7	438	24.13	-7.0	even	ok
	437	C63H160N43O13P2S2	617.7360	0.3	77.7	439	23.64	8.0	even	ok
	438	C73H181N13NaO31P2S	617.7355	-0.7	77.8	440	21.15	-8.0	even	ok
	439	C57H66N8O60P	617.7360	0.2	77.9	441	23.64	31.0	even	ok
	440	C60H152N49NaO13PS	617.7360	0.2	78.0	442	24.07	11.0	even	ok
	441	C53H6N78NaO2S2	617.7363	0.7	78.0	443	20.96	91.0	even	ok
	442	C65H165N37NaO15P2S2	617.7361	0.4	78.2	444	22.29	4.0	even	ok
	443	C60H152N53O7P2S2	617.7356	-0.5	78.3	445	22.05	14.0	even	ok
	444	C62H40N36NaO30P2	617.7356	-0.5	78.3	446	22.00	63.0	even	ok
	445	C66H173N23NaO30P2	617.7358	-0.0	78.4	447	24.45	-6.0	even	ok
	446	C62H157N47NaO9P2S2	617.7357	-0.3	78.5	448	22.77	10.0	even	ok
	447	C69H171N21O33P	617.7356	-0.4	78.5	449	22.40	-3.0	even	ok
	448	C52H78O68PS	617.7362	0.6	78.8	450	20.63	16.0	even	ok
	449	C72H46N20NaO40	617.7354	-0.8	78.8	451	19.68	61.0	even	ok
	450	C64H39N36O30P2	617.7364	0.8	78.8	452	19.26	66.0	even	ok
	451	C71H47N22NaO37P	617.7364	0.8	78.8	453	19.33	61.0	even	ok
	452	C71H55N18NaO36PS2	617.7362	0.6	78.8	454	20.80	55.0	even	ok
	453	C75H62N6NaO45S2	617.7357	-0.3	78.9	455	26.11	49.0	even	ok
	454	C58H147N55O11PS	617.7359	0.0	78.9	456	24.16	15.0	even	ok

Compound Spectrum SmartFormula Report

Meas. m/z	#	Ion Formula	m/z	err [ppm]	mSigma	# mSigma	Score	rdb	e⁻ Conf	N-Rule
	455	C36H27N66NaO22PS2	617.7361	0.4	79.0	457	21.81	58.0	even	ok
	456	C38H39N52NaO32PS2	617.7361	0.4	79.1	458	21.64	47.0	even	ok
	457	C61H22N54NaO18S	617.7356	-0.4	79.3	459	21.36	79.0	even	ok
	458	C44H30N56O28PS	617.7362	0.6	79.3	460	20.29	60.0	even	ok
	459	C69H42N28O35P	617.7363	0.7	79.4	461	19.71	65.0	even	ok
	460	C47H51N32NaO44PS	617.7359	0.0	79.6	463	23.33	40.0	even	ok
	461	C59H17N60O16S	617.7355	-0.6	79.7	464	20.16	83.0	even	ok
	462	C55H54N22O50P	617.7360	0.2	79.8	465	21.95	42.0	even	ok
	463	C49H63N18NaO54PS	617.7359	0.0	79.8	466	23.09	29.0	even	ok
	464	C76H65N2O49S2	617.7360	0.3	80.0	467	25.18	47.0	even	ok
	465	C67H167N31O22PS2	617.7355	-0.6	80.1	468	19.58	2.0	even	ok
	466	C77H182N3O44	617.7360	0.2	80.2	469	21.72	-10.0	even	ok
	467	C40H51N38NaO42PS2	617.7361	0.4	80.4	470	20.47	36.0	even	ok
	468	C45H39N46NaO34PS	617.7359	0.0	80.6	471	22.46	51.0	even	ok
	469	C46H77N4O69S2	617.7355	-0.6	80.6	472	19.12	12.0	even	ok
	470	C55H47N30NaO42P	617.7361	0.4	80.6	473	20.45	49.0	even	ok
	471	C66H158N37O22S	617.7362	0.6	80.8	474	19.26	8.0	even	ok
	472	C68H163N31NaO24S	617.7363	0.7	80.8	475	18.43	4.0	even	ok
	473	C69H172N25NaO24PS2	617.7356	-0.5	81.0	476	19.69	-2.0	even	ok
	474	C56H63N12NaO56P	617.7357	-0.3	81.1	477	20.29	33.0	even	ok
	475	C34H22N72O20PS2	617.7360	0.2	81.2	478	20.72	62.0	even	ok
	476	C70H175N21O28PS2	617.7359	0.1	81.2	479	21.38	-4.0	even	ok
	477	C51H75N4NaO64PS	617.7359	0.0	81.2	480	21.77	18.0	even	ok
	478	C36H34N58O30PS2	617.7360	0.2	81.3	481	20.62	51.0	even	ok
	479	C57H144N59NaO7PS	617.7355	-0.6	81.3	482	18.85	17.0	even	ok
	480	C79H63N2O47P2	617.7353	-0.9	81.5	483	17.17	52.0	even	ok
	481	C71H47N26O31P2S	617.7360	0.2	81.6	484	20.48	64.0	even	ok
	482	C68H39N32NaO31P	617.7359	0.1	81.6	485	21.10	67.0	even	ok
	483	C76H179N7NaO40	617.7356	-0.4	81.6	486	19.67	-8.0	even	ok
	484	C45H46N38O42PS	617.7358	-0.1	81.8	487	20.83	44.0	even	ok
	485	C74H174N13O38	617.7355	-0.5	81.9	488	18.69	-4.0	even	ok
	486	C34H15N80NaO12PS2	617.7361	0.4	81.9	489	19.36	69.0	even	ok
	487	C57H15N66NaO9PS	617.7361	0.5	82.0	490	18.89	85.0	even	ok
	488	C47H58N24O52PS	617.7358	-0.1	82.0	491	20.72	33.0	even	ok
	489	C73H52N20NaO33P2S	617.7361	0.4	82.0	492	19.31	60.0	even	ok
	490	C68H39N36O25P2S	617.7356	-0.5	82.1	493	18.57	70.0	even	ok
	491	C65H155N41NaO18S	617.7359	-0.0	82.2	494	21.09	10.0	even	ok
	492	C47H14N70NaO15S	617.7362	0.6	82.2	495	15.12	77.0	even	ok
	493	C60H23N60O9P2S2	617.7362	0.6	82.3	496	18.07	82.0	even	ok
	494	C72H180N15NaO30PS2	617.7360	0.3	82.3	497	19.64	-8.0	even	ok
	495	C70H44N30NaO27P2S	617.7356	-0.4	82.4	498	19.17	66.0	even	ok
	496	C66H34N38O29P	617.7358	-0.0	82.4	499	20.73	71.0	even	ok
	497	C63H150N47O16S	617.7358	-0.2	82.4	500	20.13	14.0	even	ok
	498	C56H70N4O64P	617.7356	-0.5	82.5	501	18.45	26.0	even	ok
	499	C38H46N44O40PS2	617.7360	0.3	82.5	502	19.55	40.0	even	ok
	500	C54H153N53NaO12P2S	617.7356	-0.4	82.7	503	15.58	7.0	even	ok

Compound Spectrum SmartFormula Report

Meas. m/z	#	Ion Formula	m/z	err [ppm]	mSigma	# mSigma	Score	rdb	e⁻ Conf	N-Rule
632.7447	1	C20H60N41O60S2	632.7443	-0.7	18.6	1	85.22	13.0	even	ok
	2	C22H65N35NaO62S2	632.7444	-0.5	18.7	2	88.87	9.0	even	ok
	3	C32H90N3NaO81PS	632.7452	0.7	19.0	3	84.18	-9.0	even	ok
	4	C32H80N11O78S	632.7445	-0.3	19.1	4	93.23	0.0	even	ok
	5	C30H85N9O79PS	632.7451	0.5	19.4	5	87.13	-5.0	even	ok
	6	C23H68N31O66S2	632.7448	0.0	19.5	6	100.00	7.0	even	ok
	7	C34H85N5NaO80S	632.7446	-0.2	19.5	7	96.32	-4.0	even	ok
	8	C21H56N45O56S2	632.7447	0.0	19.7	8	99.73	18.0	even	ok
	9	C30H78N17NaO71PS	632.7452	0.7	19.9	9	82.69	2.0	even	ok
	10	C35H88NO84S	632.7450	0.4	20.0	10	89.98	-6.0	even	ok
	11	C23H61N39NaO58S2	632.7448	0.2	20.0	11	95.27	14.0	even	ok
	12	C25H73N25NaO68S2	632.7448	0.2	20.1	12	95.01	3.0	even	ok
	13	C29H82N13NaO75PS	632.7447	-0.0	20.1	13	99.13	-3.0	even	ok
	14	C20H53N49NaO52S2	632.7444	-0.5	20.1	14	86.10	20.0	even	ok
	15	C18H48N55O50S2	632.7443	-0.7	20.3	15	82.12	24.0	even	ok
	16	C24H64N35O62S2	632.7452	0.7	20.5	16	80.41	12.0	even	ok
	17	C28H73N23O69PS	632.7451	0.5	20.5	17	85.14	6.0	even	ok
	18	C27H77N19O73PS	632.7446	-0.2	21.0	18	93.58	1.0	even	ok
	19	C26H76N21O72S2	632.7452	0.7	21.7	19	78.26	1.0	even	ok
	20	C29H82N17O69P2S2	632.7443	-0.6	22.0	20	80.89	0.0	even	ok
	21	C27H70N27NaO65PS	632.7447	-0.0	22.3	21	94.33	8.0	even	ok
	22	C26H74N23NaO69PS	632.7443	-0.7	22.5	22	77.31	3.0	even	ok
	23	C16H46N61NaO43PS2	632.7449	0.3	22.6	23	86.36	26.0	even	ok
	24	C31H87N11NaO71P2S2	632.7444	-0.5	23.2	24	82.13	-4.0	even	ok
	25	C25H65N33O63PS	632.7446	-0.2	23.3	25	88.72	12.0	even	ok
	26	C14H41N67O41PS2	632.7448	0.2	23.6	26	87.86	30.0	even	ok
	27	C27H70N31O59P2S2	632.7443	-0.6	24.0	27	77.17	11.0	even	ok
	28	C33H76N15O74S	632.7450	0.4	24.1	28	82.27	5.0	even	ok
	29	C30H68N25O68S	632.7445	-0.3	24.6	29	82.59	11.0	even	ok
	30	C35H81N9NaO76S	632.7451	0.5	24.7	30	77.98	1.0	even	ok
	31	C32H73N19NaO70S	632.7446	-0.2	24.7	31	85.80	7.0	even	ok
	32	C22H52N49O52S2	632.7452	0.7	24.9	32	73.11	23.0	even	ok
	33	C29H75N25NaO61P2S2	632.7444	-0.5	25.0	33	78.81	7.0	even	ok
	34	C32H90N7O75P2S2	632.7448	0.1	25.2	34	86.54	-6.0	even	ok
	35	C19H44N59O46S2	632.7447	0.0	25.3	35	88.15	29.0	even	ok
	36	C21H49N53NaO48S2	632.7448	0.2	25.4	36	84.68	25.0	even	ok
	37	C24H62N37NaO59PS	632.7443	-0.7	25.6	37	71.86	14.0	even	ok
	38	C13H38N71NaO37PS2	632.7445	-0.4	25.9	38	79.06	32.0	even	ok
	39	C30H78N21O65P2S2	632.7448	0.1	26.0	39	85.17	5.0	even	ok
	40	C28H66N31NaO61PS	632.7452	0.7	26.0	40	72.27	13.0	even	ok
	41	C37H85N3O81P	632.7448	0.2	26.2	41	82.75	-1.0	even	ok
	42	C18H41N63NaO42S2	632.7444	-0.5	26.5	42	74.45	31.0	even	ok
	43	C26H61N37O59PS	632.7451	0.5	26.7	43	74.25	17.0	even	ok
	44	C34H95NNaO77P2S2	632.7449	0.2	26.7	44	80.40	-10.0	even	ok
	45	C16H36N69O40S2	632.7443	-0.7	26.9	45	70.75	35.0	even	ok
	46	C32H83N15NaO67P2S2	632.7449	0.2	27.3	46	79.48	1.0	even	ok

Compound Spectrum SmartFormula Report

Meas. m/z	#	Ion Formula	m/z	err [ppm]	mSigma	# mSigma	Score	rdb	e⁻ Conf	N-Rule
	47	C34H87N5NaO78P2	632.7450	0.5	27.8	47	73.24	-4.0	even	ok
	48	C23H63N39NaO56P2S	632.7453	0.8	27.9	48	65.83	14.0	even	ok
	49	C36H82N7NaO77P	632.7445	-0.4	28.4	49	74.56	1.0	even	ok
	50	C33H91NNaO82P2	632.7446	-0.2	28.9	50	77.20	-9.0	even	ok
	51	C33H86N11O71P2S2	632.7452	0.8	28.9	51	65.12	-1.0	even	ok
	52	C25H58N41NaO55PS	632.7447	-0.0	28.9	52	80.91	19.0	even	ok
	53	C32H82N11O76P2	632.7450	0.3	29.3	53	73.60	0.0	even	ok
	54	C14H34N75NaO33PS2	632.7449	0.3	29.4	54	73.92	37.0	even	ok
	55	C34H77N13O75P	632.7444	-0.5	29.5	55	69.77	5.0	even	ok
	56	C11H33N77O35PS2	632.7444	-0.5	29.6	56	57.89	36.0	even	ok
	57	C23H53N47O53PS	632.7446	-0.2	29.9	57	76.04	23.0	even	ok
	58	C38H87N5NaO73P2S	632.7442	-0.8	30.1	58	62.95	0.0	even	ok
	59	C39H90NO77P2S	632.7446	-0.3	30.2	59	73.83	-2.0	even	ok
	60	C25H58N45O49P2S2	632.7443	-0.6	30.3	60	66.54	22.0	even	ok
	61	C31H86N7O80P2	632.7445	-0.4	30.5	61	71.32	-5.0	even	ok
	62	C27H63N39NaO51P2S2	632.7444	-0.5	30.9	62	68.40	18.0	even	ok
	63	C28H66N35O55P2S2	632.7448	0.1	31.0	63	75.69	16.0	even	ok
	64	C31H64N29O64S	632.7450	0.4	31.8	64	68.77	16.0	even	ok
	65	C30H71N29NaO57P2S2	632.7449	0.2	31.9	65	71.10	12.0	even	ok
	66	C33H69N23NaO66S	632.7451	0.5	32.0	66	65.56	12.0	even	ok
	67	C21H58N45O54P2S	632.7452	0.7	32.1	67	51.85	18.0	even	ok
	68	C39H80N5O80	632.7443	-0.7	32.2	68	62.21	4.0	even	ok
	69	C34H85N9O74PS2	632.7442	-0.8	32.3	69	60.57	-1.0	even	ok
	70	C20H40N63O42S2	632.7452	0.7	32.6	70	60.94	34.0	even	ok
	71	C31H74N25O61P2S2	632.7452	0.8	32.6	71	59.47	10.0	even	ok
	72	C30H61N33NaO60S	632.7446	-0.2	32.9	72	70.41	18.0	even	ok
	73	C28H56N39O58S	632.7445	-0.3	33.0	73	67.51	22.0	even	ok
	74	C12H29N81O31PS2	632.7448	0.2	33.1	74	58.48	41.0	even	ok
	75	C19H37N67NaO38S2	632.7448	0.2	33.6	75	69.49	36.0	even	ok
	76	C17H32N73O36S2	632.7447	0.0	33.7	76	72.06	40.0	even	ok
	77	C36H90N3NaO76PS2	632.7443	-0.6	33.9	77	60.67	-5.0	even	ok
	78	C37H78N11NaO73P	632.7449	0.3	34.0	78	65.87	6.0	even	ok
	79	C26H54N45NaO51PS	632.7452	0.7	34.6	79	58.66	24.0	even	ok
	80	C35H73N17O71P	632.7448	0.2	34.7	80	67.31	10.0	even	ok
	81	C31H79N15NaO72P2	632.7446	-0.2	35.0	81	55.12	2.0	even	ok
	82	C20H55N49NaO50P2S	632.7448	0.1	35.1	82	56.15	20.0	even	ok
	83	C27H77N15NaO79	632.7450	0.4	35.3	83	61.95	-2.0	even	ok
	84	C16H29N77NaO32S2	632.7444	-0.5	35.3	84	59.93	42.0	even	ok
	85	C24H49N51O49PS	632.7451	0.5	35.3	85	60.20	28.0	even	ok
	86	C22H50N51NaO49PS	632.7443	-0.7	35.4	86	47.05	25.0	even	ok
	87	C14H24N83O30S2	632.7443	-0.7	35.7	87	56.82	46.0	even	ok
	88	C32H73N23O64PS2	632.7442	-0.8	35.8	88	55.29	10.0	even	ok
	89	C11H26N85NaO27PS2	632.7445	-0.4	35.9	89	51.43	43.0	even	ok
	90	C29H74N21O70P2	632.7445	-0.4	36.8	90	50.51	6.0	even	ok
	91	C18H50N55O48P2S	632.7447	-0.0	36.9	91	55.30	24.0	even	ok
	92	C32H75N19NaO68P2	632.7450	0.5	37.1	92	48.46	7.0	even	ok

Compound Spectrum SmartFormula Report

Meas. m/z	#	Ion Formula	m/z	err [ppm]	mSigma	# mSigma	Score	rdb	e⁻ Conf	N-Rule
	93	C34H78N17NaO66PS2	632.7443	-0.6	37.2	93	55.68	6.0	even	ok
	94	C9H21N91O25PS2	632.7444	-0.5	37.2	94	47.67	47.0	even	ok
	95	C21H51N53NaO46P2S	632.7453	0.8	37.4	95	43.28	25.0	even	ok
	96	C37H78N15O67P2S	632.7446	-0.3	37.7	96	60.83	9.0	even	ok
	97	C36H75N19NaO63P2S	632.7442	-0.8	38.2	97	51.03	11.0	even	ok
	98	C39H83N9NaO69P2S	632.7447	-0.1	38.4	98	62.14	5.0	even	ok
	99	C40H86N5O73P2S	632.7450	0.4	38.4	99	56.90	3.0	even	ok
	100	C26H54N49O45P2S2	632.7448	0.1	38.6	100	62.37	27.0	even	ok
	101	C35H81N13O70PS2	632.7447	-0.1	38.6	101	62.57	4.0	even	ok
	102	C30H70N25O66P2	632.7449	0.3	38.6	102	48.44	11.0	even	ok
	103	C23H46N59O39P2S2	632.7443	-0.6	38.7	103	53.54	33.0	even	ok
	104	C19H46N59O44P2S	632.7452	0.7	38.9	104	43.45	29.0	even	ok
	105	C25H51N53NaO41P2S2	632.7444	-0.5	39.0	105	55.32	29.0	even	ok
	106	C28H59N43NaO47P2S2	632.7449	0.2	39.2	106	58.93	23.0	even	ok
	107	C29H62N39O51P2S2	632.7452	0.8	39.2	107	50.15	21.0	even	ok
	108	C40H76N9O76	632.7447	0.0	40.1	108	60.76	9.0	even	ok
	109	C17H47N59NaO44P2S	632.7444	-0.6	40.1	109	43.51	26.0	even	ok
	110	C42H81N3NaO78	632.7448	0.2	40.2	110	58.37	5.0	even	ok
	111	C37H86N7NaO72PS2	632.7448	0.1	40.2	111	59.65	0.0	even	ok
	112	C14H41N63NaO47S	632.7452	0.8	40.3	112	40.86	27.0	even	ok
	113	C25H65N29NaO69	632.7450	0.4	40.4	113	45.14	9.0	even	ok
	114	C25H72N21O77	632.7449	0.3	40.4	114	46.88	2.0	even	ok
	115	C34H70N21NaO67P	632.7445	-0.4	40.5	115	45.56	12.0	even	ok
	116	C29H52N43O54S	632.7450	0.4	40.9	116	54.18	27.0	even	ok
	117	C31H57N37NaO56S	632.7451	0.5	41.0	117	51.84	23.0	even	ok
	118	C23H46N55NaO45PS	632.7447	-0.0	41.1	118	49.15	30.0	even	ok
	119	C32H65N27O65P	632.7444	-0.5	41.5	119	42.47	16.0	even	ok
	120	C39H73N13NaO72	632.7444	-0.5	41.5	120	50.71	11.0	even	ok
	121	C18H28N77O32S2	632.7452	0.7	41.7	121	47.90	45.0	even	ok
	122	C12H22N89NaO23PS2	632.7449	0.3	41.7	122	44.77	48.0	even	ok
	123	C37H68N19O70	632.7443	-0.7	41.8	123	48.29	15.0	even	ok
	124	C29H67N29NaO62P2	632.7446	-0.2	41.9	124	45.76	13.0	even	ok
	125	C30H61N37O54PS2	632.7442	-0.8	42.0	125	46.69	21.0	even	ok
	126	C38H89N3O76PS2	632.7451	0.6	42.0	126	48.60	-2.0	even	ok
	127	C15H42N65O42P2S	632.7443	-0.7	42.0	127	39.56	30.0	even	ok
	128	C21H41N61O43PS	632.7446	-0.2	42.1	128	46.01	34.0	even	ok
	129	C18H43N63NaO40P2S	632.7448	0.1	42.2	129	46.50	31.0	even	ok
	130	C12H36N69O45S	632.7451	0.6	42.2	130	40.45	31.0	even	ok
	131	C28H49N47NaO50S	632.7446	-0.2	42.3	131	54.68	29.0	even	ok
	132	C23H60N35O67	632.7449	0.3	42.3	132	44.51	13.0	even	ok
	133	C7H19N97NaO18P2S2	632.7450	0.5	42.5	133	41.90	49.0	even	ok
	134	C26H44N53O48S	632.7445	-0.3	42.5	134	52.28	33.0	even	ok
	135	C10H17N95O21PS2	632.7448	0.2	42.7	135	45.33	52.0	even	ok
	136	C24H69N25NaO73	632.7446	-0.3	43.0	136	43.63	4.0	even	ok
	137	C17H25N81NaO28S2	632.7448	0.2	43.1	137	54.00	47.0	even	ok
	138	C32H66N31NaO56PS2	632.7443	-0.6	43.1	138	47.29	17.0	even	ok

Compound Spectrum SmartFormula Report

Meas. m/z	#	Ion Formula	m/z	err [ppm]	mSigma	# mSigma	Score	rdb	e⁻ Conf	N-Rule
	139	C27H62N35O60P2	632.7445	-0.4	43.6	139	41.93	17.0	even	ok
	140	C33H69N27O60PS2	632.7447	-0.1	43.8	140	54.06	15.0	even	ok
	141	C16H38N69O38P2S	632.7447	-0.0	43.8	141	45.67	35.0	even	ok
	142	C5H14N103O16P2S2	632.7449	0.3	44.1	142	41.70	53.0	even	ok
	143	C20H38N65NaO39PS	632.7443	-0.7	45.0	143	36.18	36.0	even	ok
	144	C22H64N31O71	632.7445	-0.4	45.0	144	39.61	8.0	even	ok
	145	C35H74N21NaO62PS2	632.7448	0.1	45.2	145	51.95	11.0	even	ok
	146	C11H33N73NaO41S	632.7448	0.1	45.4	146	43.11	33.0	even	ok
	147	C23H53N43NaO59	632.7450	0.4	45.5	147	39.15	20.0	even	ok
	148	C22H57N39NaO63	632.7446	-0.3	45.5	148	40.59	15.0	even	ok
	149	C9H14N99NaO17PS2	632.7445	-0.4	45.5	149	39.39	54.0	even	ok
	150	C12H29N77NaO37S	632.7452	0.8	45.6	150	35.25	38.0	even	ok
	151	C30H63N33NaO58P2	632.7450	0.5	46.0	151	37.81	18.0	even	ok
	152	C36H77N17O66PS2	632.7451	0.6	46.3	152	43.04	9.0	even	ok
	153	C19H39N67NaO36P2S	632.7453	0.8	46.5	153	33.63	36.0	even	ok
	154	C38H74N19O63P2S	632.7450	0.4	46.6	154	45.23	14.0	even	ok
	155	C35H66N29O57P2S	632.7446	-0.3	46.6	155	47.34	20.0	even	ok
	156	C7H9N105O15PS2	632.7444	-0.5	46.7	156	36.52	58.0	even	ok
	157	C37H71N23NaO59P2S	632.7447	-0.1	47.0	157	48.58	16.0	even	ok
	158	C15H35N73NaO34P2S	632.7444	-0.6	47.2	158	35.53	37.0	even	ok
	159	C15H20N87O26S2	632.7447	0.0	47.3	159	41.41	51.0	even	ok
	160	C40H79N13NaO65P2S	632.7451	0.6	47.3	160	42.47	10.0	even	ok
	161	C21H48N49O57	632.7449	0.3	47.4	161	38.55	24.0	even	ok
	162	C10H24N83O35S	632.7451	0.6	47.4	162	34.84	42.0	even	ok
	163	C28H58N39O56P2	632.7449	0.3	47.4	163	37.81	22.0	even	ok
	164	C24H42N63O35P2S2	632.7448	0.1	47.5	164	48.56	38.0	even	ok
	165	C34H63N33NaO53P2S	632.7442	-0.8	47.5	165	39.22	22.0	even	ok
	166	C4H11N107NaO12P2S2	632.7446	-0.2	47.5	166	38.88	55.0	even	ok
	167	C27H50N53O41P2S2	632.7452	0.8	47.5	167	39.77	32.0	even	ok
	168	C20H52N45O61	632.7445	-0.4	47.5	168	36.74	19.0	even	ok
	169	C35H66N25NaO63P	632.7449	0.3	47.7	169	37.68	17.0	even	ok
	170	C38H82N11NaO68PS2	632.7452	0.8	47.8	170	39.36	5.0	even	ok
	171	C17H34N73O34P2S	632.7452	0.7	47.9	171	33.76	40.0	even	ok
	172	C26H47N57NaO37P2S2	632.7449	0.2	47.9	172	46.09	34.0	even	ok
	173	C21H34N73O29P2S2	632.7443	-0.6	48.1	173	40.91	44.0	even	ok
	174	C23H39N67NaO31P2S2	632.7444	-0.5	48.3	174	42.42	40.0	even	ok
	175	C24H42N59NaO41PS	632.7452	0.7	48.4	175	33.37	35.0	even	ok
	176	C33H61N31O61P	632.7448	0.2	48.4	176	38.35	21.0	even	ok
	177	C41H72N13O72	632.7452	0.7	48.7	177	39.07	14.0	even	ok
	178	C13H30N79O32P2S	632.7443	-0.7	48.9	178	32.30	41.0	even	ok
	179	C22H37N65O39PS	632.7451	0.5	49.1	179	34.10	39.0	even	ok
	180	C2H6N113O10P2S2	632.7445	-0.4	49.2	180	35.48	59.0	even	ok
	181	C14H17N91NaO22S2	632.7444	-0.6	49.2	181	33.75	53.0	even	ok
	182	C12H12N97O20S2	632.7443	-0.7	49.7	182	31.87	57.0	even	ok
	183	C28H49N51O44PS2	632.7442	-0.8	49.8	183	37.06	32.0	even	ok
	184	C40H69N17NaO68	632.7448	0.2	49.9	184	44.10	16.0	even	ok

Compound Spectrum SmartFormula Report

Meas. m/z	#	Ion Formula	m/z	err [ppm]	mSigma	# mSigma	Score	rdb	e¯ Conf	N-Rule
	185	C38H64N23O66	632.7447	0.0	50.0	185	45.76	20.0	even	ok
	186	C42H81N7O72PS	632.7445	-0.4	50.0	186	40.94	8.0	even	ok
	187	C27H55N43NaO52P2	632.7446	-0.2	50.7	187	35.30	24.0	even	ok
	188	C30H54N45NaO46PS2	632.7443	-0.6	50.7	188	37.72	28.0	even	ok
	189	C29H45N51NaO46S	632.7451	0.5	50.7	189	39.08	34.0	even	ok
	190	C27H40N57O44S	632.7450	0.4	50.8	190	40.72	38.0	even	ok
	191	C20H45N53NaO53	632.7445	-0.3	50.8	191	34.60	26.0	even	ok
	192	C9H21N87NaO31S	632.7448	0.1	50.9	192	36.65	44.0	even	ok
	193	C31H57N41O50PS2	632.7447	-0.1	50.9	193	43.76	26.0	even	ok
	194	C32H58N35NaO57P	632.7445	-0.4	51.0	194	33.45	23.0	even	ok
	195	C16H31N77NaO30P2S	632.7448	0.1	51.1	195	35.82	42.0	even	ok
	196	C44H86NNaO74PS	632.7446	-0.3	51.1	196	41.24	4.0	even	ok
	197	C5H7N111NaO8P2S2	632.7450	0.5	51.5	197	32.14	60.0	even	ok
	198	C37H61N27NaO62	632.7444	-0.5	51.6	198	37.73	22.0	even	ok
	199	C39H81N9NaO71S2	632.7442	-0.8	51.6	199	35.10	5.0	even	ok
	200	C21H34N69NaO35PS	632.7447	-0.0	51.7	200	35.97	41.0	even	ok
	201	C30H53N41O55P	632.7444	-0.5	52.0	201	31.16	27.0	even	ok
	202	C33H62N35NaO52PS2	632.7448	0.1	52.0	202	42.38	22.0	even	ok
	203	C25H50N49O50P2	632.7445	-0.4	52.2	203	32.35	28.0	even	ok
	204	C10H10N103NaO13PS2	632.7449	0.3	52.4	204	32.76	59.0	even	ok
	205	C34H65N31O56PS2	632.7451	0.6	52.6	205	35.63	20.0	even	ok
	206	C19H29N75O33PS	632.7446	-0.2	52.6	206	33.65	45.0	even	ok
	207	C14H26N83O28P2S	632.7447	-0.0	52.6	207	35.08	46.0	even	ok
	208	C18H40N59O51	632.7445	-0.4	52.7	208	31.27	30.0	even	ok
	209	C7H16N93O29S	632.7447	-0.1	52.8	209	34.30	48.0	even	ok
	210	C21H41N57NaO49	632.7450	0.4	52.8	210	31.37	31.0	even	ok
	211	C10H17N91NaO27S	632.7452	0.8	53.1	212	28.11	49.0	even	ok
	212	C40H84N5O75S2	632.7446	-0.2	53.2	213	39.19	3.0	even	ok
	213	C8H5N109O11PS2	632.7448	0.2	53.2	214	33.14	63.0	even	ok
	214	C36H70N25NaO58PS2	632.7452	0.8	53.9	215	32.74	16.0	even	ok
	215	C18H50N51NaO54P	632.7451	0.6	54.5	216	28.36	21.0	even	ok
	216	C19H36N63O47	632.7449	0.3	54.6	217	30.87	35.0	even	ok
	217	C8H12N97O25S	632.7451	0.6	54.8	218	27.78	53.0	even	ok
	218	C44H78N9O69P2	632.7443	-0.6	55.3	219	32.71	13.0	even	ok
	219	C18H26N79NaO29PS	632.7443	-0.7	55.4	220	26.24	47.0	even	ok
	220	C46H83N3NaO71P2	632.7444	-0.5	55.6	221	33.82	9.0	even	ok
	221	C36H62N33O53P2S	632.7450	0.4	55.7	222	34.17	25.0	even	ok
	222	C13H23N87NaO24P2S	632.7444	-0.6	55.9	223	27.05	48.0	even	ok
	223	C28H51N47NaO48P2	632.7450	0.5	56.1	225	27.75	29.0	even	ok
	224	C33H54N43O47P2S	632.7446	-0.3	56.2	226	35.13	31.0	even	ok
	225	C38H67N27NaO55P2S	632.7451	0.6	56.2	227	32.21	21.0	even	ok
	226	C6H13N97NaO25S	632.7443	-0.6	56.3	228	26.26	50.0	even	ok
	227	C16H16N91O22S2	632.7452	0.7	56.4	229	25.68	56.0	even	ok
	228	C35H59N37NaO49P2S	632.7446	-0.1	56.5	230	36.18	27.0	even	ok
	229	C16H45N57O52P	632.7450	0.4	56.6	231	27.67	25.0	even	ok
	230	C25H38N67O31P2S2	632.7452	0.8	56.6	232	29.98	43.0	even	ok

Compound Spectrum SmartFormula Report

Meas. m/z	#	Ion Formula	m/z	err [ppm]	mSigma	# mSigma	Score	rdb	e¯ Conf	N-Rule
	231	C17H27N81NaO26P2S	632.7453	0.8	56.6	233	24.59	47.0	even	ok
	232	C35H56N33O60	632.7443	-0.7	56.7	234	25.59	26.0	even	ok
	233	C22H30N77O25P2S2	632.7448	0.1	57.0	235	36.07	49.0	even	ok
	234	C26H37N61NaO40S	632.7446	-0.2	57.2	236	28.91	40.0	even	ok
	235	C32H51N47NaO43P2S	632.7442	-0.8	57.3	237	28.80	33.0	even	ok
	236	C37H69N23NaO61S2	632.7442	-0.8	57.3	238	29.18	16.0	even	ok
	237	C24H35N71NaO27P2S2	632.7449	0.2	57.3	239	34.35	45.0	even	ok
	238	C26H46N53O46P2	632.7449	0.3	57.4	240	27.74	33.0	even	ok
	239	C24H32N67O38S	632.7445	-0.3	57.5	241	27.54	44.0	even	ok
	240	C11H18N93O22P2S	632.7443	-0.7	57.5	242	24.58	52.0	even	ok
	241	C15H22N87O24P2S	632.7452	0.7	57.9	243	24.69	51.0	even	ok
	242	C15H13N95NaO18S2	632.7448	0.2	58.1	244	28.45	58.0	even	ok
	243	C18H33N67NaO43	632.7445	-0.3	58.1	245	27.34	37.0	even	ok
	244	C40H69N21O62PS	632.7445	-0.4	58.3	246	31.45	19.0	even	ok
	245	C4H8N103O23S	632.7442	-0.8	58.3	247	23.55	54.0	even	ok
	246	C13H8N101O16S2	632.7447	0.0	58.3	248	29.31	62.0	even	ok
	247	C7H9N101NaO21S	632.7448	0.1	58.3	249	28.92	55.0	even	ok
	248	C38H72N19O65S2	632.7446	-0.2	58.4	250	33.05	14.0	even	ok
	249	C26H37N65O34PS2	632.7442	-0.8	58.7	251	27.93	43.0	even	ok
	250	C39H60N27O62	632.7452	0.7	58.7	252	28.55	25.0	even	ok
	251	C33H54N39NaO53P	632.7449	0.3	58.8	253	26.60	28.0	even	ok
	252	C42H74N15NaO64PS	632.7446	-0.3	59.1	254	31.81	15.0	even	ok
	253	C29H45N55O40PS2	632.7447	-0.1	59.2	255	33.53	37.0	even	ok
	254	C43H77N11O68PS	632.7449	0.3	59.3	256	31.70	13.0	even	ok
	255	C28H42N59NaO36PS2	632.7443	-0.6	59.3	257	28.54	39.0	even	ok
	256	C31H49N45O51P	632.7448	0.2	59.5	258	27.03	32.0	even	ok
	257	C22H30N73NaO31PS	632.7452	0.7	59.5	259	23.49	46.0	even	ok
	258	C16H38N65NaO44P	632.7451	0.6	59.6	260	24.01	32.0	even	ok
	259	C15H42N61NaO48P	632.7447	-0.1	59.9	261	26.92	27.0	even	ok
	260	C40H77N13NaO67S2	632.7447	-0.1	59.9	262	32.63	10.0	even	ok
	261	C16H28N73O41	632.7445	-0.4	60.0	263	24.69	41.0	even	ok
	262	C31H50N49NaO42PS2	632.7448	0.1	60.0	264	32.70	33.0	even	ok
	263	C5H4N107O19S	632.7447	-0.1	60.1	265	26.97	59.0	even	ok
	264	C20H25N79O29PS	632.7451	0.5	60.2	266	23.97	50.0	even	ok
	265	C32H53N45O46PS2	632.7451	0.6	60.2	267	27.86	31.0	even	ok
	266	C12H5N105NaO12S2	632.7444	-0.6	60.3	268	23.58	64.0	even	ok
	267	C45H82N5NaO70PS	632.7450	0.4	60.4	269	29.37	9.0	even	ok
	268	C25H43N57NaO42P2	632.7446	-0.2	60.6	270	25.62	35.0	even	ok
	269	C14H19N91NaO20P2S	632.7448	0.1	61.0	272	25.97	53.0	even	ok
	270	C34H58N39NaO48PS2	632.7452	0.8	61.3	273	25.71	27.0	even	ok
	271	C41H80N9O71S2	632.7450	0.5	61.3	274	27.98	8.0	even	ok
	272	C14H33N71O42P	632.7450	0.4	61.7	275	23.39	36.0	even	ok
	273	C3H9N105O20PS	632.7452	0.8	61.7	276	21.11	54.0	even	ok
	274	C19H29N71NaO39	632.7450	0.4	61.7	277	23.51	42.0	even	ok
	275	C23H38N63O40P2	632.7445	-0.4	62.0	278	23.47	39.0	even	ok
	276	C13H37N67O46P	632.7446	-0.3	62.0	279	24.12	31.0	even	ok

Compound Spectrum SmartFormula Report

Meas. m/z	#	Ion Formula	m/z	err [ppm]	mSigma	# mSigma	Score	rdb	e⁻ Conf	N-Rule
	277	C8H5N105NaO17S	632.7452	0.8	62.0	280	20.99	60.0	even	ok
	278	C30H46N49NaO47P	632.7445	-0.4	62.0	281	23.31	34.0	even	ok
	279	C12H14N97O18P2S	632.7447	-0.0	62.4	282	25.36	57.0	even	ok
	280	C19H22N83NaO25PS	632.7447	-0.0	62.7	283	25.00	52.0	even	ok
	281	C28H41N55O45P	632.7444	-0.5	62.9	284	21.69	38.0	even	ok
	282	C43H85N3NaO73S2	632.7451	0.6	63.0	285	25.31	4.0	even	ok
	283	C17H24N77O37	632.7449	0.3	63.3	286	23.13	46.0	even	ok
	284	C19H22N87O19P2S2	632.7443	-0.6	63.5	287	20.69	55.0	even	ok
	285	C21H27N81NaO21P2S2	632.7444	-0.5	63.6	288	21.52	51.0	even	ok
	286	C17H17N89O23PS	632.7446	-0.2	63.6	289	23.36	56.0	even	ok
	287	C48H82N3O71P2	632.7452	0.8	64.2	292	23.14	12.0	even	ok
	288	C35H57N37NaO51S2	632.7442	-0.8	64.4	293	22.90	27.0	even	ok
	289	C45H74N13O65P2	632.7448	0.1	64.5	294	27.94	18.0	even	ok
	290	C47H79N7NaO67P2	632.7449	0.2	64.9	295	26.53	14.0	even	ok
	291	C36H60N33O55S2	632.7446	-0.2	65.1	296	26.27	25.0	even	ok
	292	C13H30N75NaO38P	632.7446	-0.1	65.2	297	22.40	38.0	even	ok
	293	C2H6N109NaO16PS	632.7449	0.2	65.2	298	21.92	56.0	even	ok
	294	C42H66N23O59P2	632.7443	-0.6	65.3	299	23.29	24.0	even	ok
	295	C12H34N71NaO42P	632.7442	-0.8	65.4	300	18.22	33.0	even	ok
	296	C34H50N47O43P2S	632.7450	0.4	65.4	301	24.67	36.0	even	ok
	297	C44H71N17NaO61P2	632.7444	-0.5	65.5	302	24.15	20.0	even	ok
	298	C11H11N101NaO14P2S	632.7444	-0.6	65.7	303	19.39	59.0	even	ok
	299	C38H57N31NaO58	632.7448	0.2	65.7	304	21.86	27.0	even	ok
	300	C36H55N41NaO45P2S	632.7451	0.6	65.7	305	23.33	32.0	even	ok
	301	C36H52N37O56	632.7447	0.0	65.8	306	22.61	31.0	even	ok
	302	C23H26N81O21P2S2	632.7452	0.8	66.2	307	21.60	54.0	even	ok
	303	C49H81NO74P	632.7442	-0.8	66.3	308	21.44	12.0	even	ok
	304	C16H14N93NaO19PS	632.7443	-0.8	66.3	309	18.06	58.0	even	ok
	305	C33H47N51NaO39P2S	632.7446	-0.1	66.3	310	25.78	38.0	even	ok
	306	C38H65N27NaO57S2	632.7447	-0.1	66.4	311	26.04	21.0	even	ok
	307	C14H26N79NaO34P	632.7451	0.6	66.5	312	18.92	43.0	even	ok
	308	C27H33N65NaO36S	632.7451	0.5	66.6	313	19.25	45.0	even	ok
	309	C25H28N71O34S	632.7450	0.4	66.7	314	19.99	49.0	even	ok
	310	C26H39N61NaO38P2	632.7450	0.5	66.8	315	19.29	40.0	even	ok
	311	C16H21N81NaO33	632.7445	-0.3	66.8	316	20.24	48.0	even	ok
	312	C9H6N107O12P2S	632.7443	-0.7	67.1	317	17.61	63.0	even	ok
	313	C11H25N81O36P	632.7446	-0.3	67.2	318	20.03	42.0	even	ok
	314	C38H57N35O52PS	632.7445	-0.4	67.2	319	23.00	30.0	even	ok
	315	C39H68N23O61S2	632.7450	0.5	67.3	320	22.69	19.0	even	ok
	316	C15H15N95NaO16P2S	632.7453	0.8	67.4	321	17.04	58.0	even	ok
	317	C14H4N105O12S2	632.7452	0.7	67.5	322	17.52	67.0	even	ok
	318	C35H49N41NaO52	632.7444	-0.6	67.7	323	18.25	33.0	even	ok
	319	C41H65N25O58PS	632.7449	0.3	67.8	324	23.61	24.0	even	ok
	320	C40H62N29NaO54PS	632.7445	-0.3	67.9	325	23.35	26.0	even	ok
	321	C24H34N67O36P2	632.7449	0.3	67.9	326	19.27	44.0	even	ok
	322	C33H44N47O50	632.7443	-0.7	68.0	327	17.28	37.0	even	ok

Compound Spectrum SmartFormula Report

Meas. m/z	#	Ion Formula	m/z	err [ppm]	mSigma	# mSigma	Score	rdb	e⁻ Conf	N-Rule
	323	C24H25N79O24PS2	632.7442	-0.8	68.0	328	20.09	54.0	even	ok
	324	C27H33N69O30PS2	632.7447	-0.1	68.1	329	24.47	48.0	even	ok
	325	C12H21N85O32P	632.7450	0.4	68.5	330	18.40	47.0	even	ok
	326	C14H16N87O31	632.7444	-0.4	68.5	331	18.27	52.0	even	ok
	327	C13H10N101O14P2S	632.7452	0.7	68.5	332	17.09	62.0	even	ok
	328	C24H25N75NaO30S	632.7446	-0.2	68.5	333	19.51	51.0	even	ok
	329	C26H30N73NaO26PS2	632.7443	-0.7	68.6	334	20.60	50.0	even	ok
	330	C43H70N19NaO60PS	632.7450	0.4	68.7	335	21.95	20.0	even	ok
	331	C30H41N59O36PS2	632.7451	0.6	68.7	336	20.70	42.0	even	ok
	332	C22H20N81O28S	632.7445	-0.4	68.8	337	18.54	55.0	even	ok
	333	C41H73N17NaO63S2	632.7451	0.6	68.8	338	20.60	15.0	even	ok
	334	C29H38N63NaO32PS2	632.7448	0.1	68.8	339	24.02	44.0	even	ok
	335	C32H46N53NaO38PS2	632.7452	0.8	69.6	341	19.17	38.0	even	ok
	336	C31H42N53NaO43P	632.7449	0.3	70.1	342	17.87	39.0	even	ok
	337	C10H22N85NaO32P	632.7442	-0.8	70.8	343	14.94	44.0	even	ok
	338	C29H37N59O41P	632.7448	0.2	70.8	344	18.14	43.0	even	ok
	339	C20H18N87NaO21PS	632.7452	0.7	70.8	345	15.74	57.0	even	ok
	340	C23H31N71NaO32P2	632.7446	-0.2	71.0	346	17.60	46.0	even	ok
	341	C17H17N85NaO29	632.7450	0.4	71.4	347	16.61	53.0	even	ok
	342	C18H13N93O19PS	632.7451	0.5	71.5	348	16.03	61.0	even	ok
	343	C12H7N105NaO10P2S	632.7448	0.1	71.6	349	17.83	64.0	even	ok
	344	C45H72N13O67S	632.7444	-0.6	71.8	350	18.59	18.0	even	ok
	345	C11H18N89NaO28P	632.7446	-0.1	72.1	351	17.38	49.0	even	ok
	346	C21H26N77O30P2	632.7445	-0.4	72.3	352	16.11	50.0	even	ok
	347	C31H42N57O37P2S	632.7446	-0.3	72.4	353	16.54	42.0	even	ok
	348	C33H45N51NaO41S2	632.7442	-0.8	72.4	354	17.05	38.0	even	ok
	349	C34H48N47O45S2	632.7446	-0.2	72.7	355	19.80	36.0	even	ok
	350	C15H12N91O27	632.7449	0.3	72.9	357	16.33	57.0	even	ok
	351	C47H77N7NaO69S	632.7444	-0.4	73.0	358	18.55	14.0	even	ok
	352	C20H18N91O15P2S2	632.7448	0.1	73.3	359	16.90	60.0	even	ok
	353	C28H34N63NaO37P	632.7445	-0.4	73.3	360	15.46	45.0	even	ok
	354	C22H23N85NaO17P2S2	632.7449	0.2	73.5	361	16.12	56.0	even	ok
	355	C48H80N3O73S	632.7448	0.1	73.6	362	19.83	12.0	even	ok
	356	C30H39N61NaO33P2S	632.7442	-0.8	73.8	363	13.32	44.0	even	ok
	357	C46H70N17O61P2	632.7452	0.8	73.9	364	16.25	23.0	even	ok
	358	C36H53N41NaO47S2	632.7447	-0.1	73.9	365	19.69	32.0	even	ok
	359	C17H10N97NaO15PS	632.7447	-0.0	74.0	366	16.53	63.0	even	ok
	360	C9H13N95O26P	632.7446	-0.3	74.0	367	15.52	53.0	even	ok
	361	C26H29N69O35P	632.7444	-0.5	74.1	368	14.36	49.0	even	ok
	362	C9H23N87NaO29P2	632.7452	0.7	74.3	369	13.52	44.0	even	ok
	363	C37H56N37O51S2	632.7450	0.5	74.5	370	17.41	30.0	even	ok
	364	C43H62N27O55P2	632.7448	0.1	74.5	371	19.36	29.0	even	ok
	365	C17H10N101O9P2S2	632.7443	-0.6	74.7	372	13.69	66.0	even	ok
	366	C19H15N95NaO11P2S2	632.7444	-0.5	74.7	373	14.28	62.0	even	ok
	367	C12H14N93NaO24P	632.7451	0.6	74.7	374	13.96	54.0	even	ok
	368	C45H67N21NaO57P2	632.7449	0.2	74.7	375	18.44	25.0	even	ok

Compound Spectrum SmartFormula Report

Meas. m/z	#	Ion Formula	m/z	err [ppm]	mSigma	# mSigma	Score	rdb	e¯ Conf	N-Rule
	369	C15H5N103O13PS	632.7446	-0.2	74.8	376	15.42	67.0	even	ok
	370	C37H48N41O52	632.7452	0.7	75.4	377	13.03	36.0	even	ok
	371	C44H79N13NaO60P2S2	632.7443	-0.7	75.6	378	15.41	14.0	even	ok
	372	C39H61N31NaO53S2	632.7451	0.6	75.8	379	15.86	26.0	even	ok
	373	C47H69N15O64P	632.7442	-0.8	75.8	380	15.02	23.0	even	ok
	374	C50H77N5O70P	632.7447	-0.1	75.9	381	18.24	17.0	even	ok
	375	C14H9N95NaO23	632.7445	-0.3	76.3	382	14.12	59.0	even	ok
	376	C7H18N93O27P2	632.7451	0.6	76.3	383	13.04	48.0	even	ok
	377	C49H74N9NaO66P	632.7443	-0.6	76.4	384	15.36	19.0	even	ok
	378	C10H9N99O22P	632.7450	0.4	76.5	385	13.57	58.0	even	ok
	379	C36H45N49O42PS	632.7445	-0.4	76.7	386	16.08	41.0	even	ok
	380	C45H82N9O64P2S2	632.7446	-0.2	76.7	387	17.29	12.0	even	ok
	381	C39H53N39O48PS	632.7449	0.3	76.8	388	16.78	35.0	even	ok
	382	C36H45N45NaO48	632.7448	0.2	77.0	389	14.31	38.0	even	ok
	383	C34H40N51O46	632.7447	0.0	77.2	390	14.76	42.0	even	ok
	384	C38H50N43NaO44PS	632.7445	-0.3	77.2	391	16.38	37.0	even	ok
	385	C25H21N83O20PS2	632.7447	-0.1	77.6	393	17.07	59.0	even	ok
	386	C41H58N33NaO50PS	632.7450	0.4	77.6	394	15.65	31.0	even	ok
	387	C28H29N73O26PS2	632.7451	0.6	77.7	395	14.67	53.0	even	ok
	388	C24H27N75NaO28P2	632.7450	0.5	77.8	396	12.74	51.0	even	ok
	389	C12H4N101O21	632.7444	-0.5	77.9	397	12.73	63.0	even	ok
	390	C25H21N79NaO26S	632.7450	0.5	77.9	398	12.56	56.0	even	ok
	391	C23H16N85O24S	632.7450	0.4	78.0	399	13.01	60.0	even	ok
	392	C27H26N77NaO22PS2	632.7448	0.1	78.1	400	16.85	55.0	even	ok
	393	C47H87N3NaO66P2S2	632.7447	-0.0	78.3	401	16.87	8.0	even	ok
	394	C13H3N109NaO6P2S	632.7452	0.8	78.4	402	11.22	69.0	even	ok
	395	C30H34N67NaO28PS2	632.7452	0.8	78.5	403	13.63	49.0	even	ok
	396	C22H22N81O26P2	632.7449	0.3	78.9	404	12.71	55.0	even	ok
	397	C33H37N55NaO42	632.7444	-0.6	79.1	405	11.77	44.0	even	ok
	398	C31H32N61O40	632.7443	-0.7	79.5	406	11.11	48.0	even	ok
	399	C6H15N97NaO23P2	632.7447	0.0	79.9	407	13.19	50.0	even	ok
	400	C22H13N89NaO20S	632.7446	-0.2	79.9	408	12.54	62.0	even	ok
	401	C43H60N27O57S	632.7444	-0.6	80.2	409	13.37	29.0	even	ok
	402	C20H8N95O18S	632.7445	-0.4	80.3	410	11.89	66.0	even	ok
	403	C31H33N65NaO31S2	632.7442	-0.8	81.1	411	12.10	49.0	even	ok
	404	C32H36N61O35S2	632.7446	-0.2	81.1	412	14.21	47.0	even	ok
	405	C45H65N21NaO59S	632.7444	-0.5	81.2	413	13.39	25.0	even	ok
	406	C46H68N17O63S	632.7448	0.1	81.5	414	14.51	23.0	even	ok
	407	C29H30N67NaO33P	632.7449	0.3	81.6	415	11.44	50.0	even	ok
	408	C15H5N99NaO19	632.7450	0.4	81.7	416	11.11	64.0	even	ok
	409	C21H19N85NaO22P2	632.7446	-0.2	81.9	417	11.48	57.0	even	ok
	410	C34H41N55NaO37S2	632.7447	-0.1	82.1	418	14.17	43.0	even	ok
	411	C27H25N73O31P	632.7448	0.2	82.2	419	11.58	54.0	even	ok
	412	C18H6N101NaO11PS	632.7451	0.7	82.3	420	10.04	68.0	even	ok
	413	C35H44N51O41S2	632.7450	0.5	82.4	421	12.70	41.0	even	ok
	414	C32H38N61O33P2S	632.7450	0.4	82.4	422	10.68	47.0	even	ok

Compound Spectrum SmartFormula Report

Meas. m/z	#	Ion Formula	m/z	err [ppm]	mSigma	# mSigma	Score	rdb	e¯ Conf	N-Rule
	415	C48H73N11NaO65S	632.7449	0.3	82.6	423	13.31	19.0	even	ok
	416	C40H54N37O49P2	632.7443	-0.6	82.7	424	9.95	35.0	even	ok
	417	C34H43N55NaO35P2S	632.7451	0.6	82.7	425	10.12	43.0	even	ok
	418	C42H59N31NaO51P2	632.7444	-0.5	82.8	426	10.34	31.0	even	ok
	419	C42H67N27NaO50P2S2	632.7443	-0.7	82.8	427	11.50	25.0	even	ok
	420	C19H14N91O20P2	632.7445	-0.4	83.1	430	10.49	61.0	even	ok
	421	C49H76N7O69S	632.7453	0.8	83.2	431	11.07	17.0	even	ok
	422	C21H14N95O11P2S2	632.7452	0.8	83.4	432	9.28	65.0	even	ok
	423	C37H49N45NaO43S2	632.7451	0.6	83.6	433	11.61	37.0	even	ok
	424	C29H30N71O27P2S	632.7445	-0.3	83.6	434	10.58	53.0	even	ok
	425	C43H70N23O54P2S2	632.7446	-0.2	83.6	435	13.05	23.0	even	ok
	426	C31H35N65NaO29P2S	632.7446	-0.1	83.7	436	10.95	49.0	even	ok
	427	C18H6N105O5P2S2	632.7448	0.1	84.5	437	10.81	71.0	even	ok
	428	C20H11N99NaO7P2S2	632.7449	0.2	84.6	438	10.34	67.0	even	ok
	429	C26H22N77NaO27P	632.7445	-0.4	84.7	439	9.76	56.0	even	ok
	430	C45H75N17NaO56P2S2	632.7447	-0.0	85.1	440	12.77	19.0	even	ok
	431	C22H13N93O14PS2	632.7442	-0.8	85.2	441	8.53	65.0	even	ok
	432	C48H65N19O60P	632.7447	-0.1	85.4	442	12.39	28.0	even	ok
	433	C24H17N83O25P	632.7444	-0.6	85.5	443	9.05	60.0	even	ok
	434	C45H57N29O54P	632.7442	-0.8	85.6	444	10.08	34.0	even	ok
	435	C24H18N87NaO16PS2	632.7443	-0.7	85.6	445	8.75	61.0	even	ok
	436	C51H73N9O66P	632.7451	0.6	85.7	446	10.59	22.0	even	ok
	437	C9H6N103NaO18P	632.7446	-0.1	85.8	447	8.02	60.0	even	ok
	438	C17H3N109NaOP2S2	632.7444	-0.5	86.0	448	9.01	73.0	even	ok
	439	C47H62N23NaO56P	632.7443	-0.7	86.0	449	10.33	30.0	even	ok
	440	C50H70N13NaO62P	632.7448	0.1	86.0	450	12.17	24.0	even	ok
	441	C46H78N13O60P2S2	632.7451	0.5	86.1	451	10.67	17.0	even	ok
	442	C37H41N53O38PS	632.7449	0.3	86.3	452	11.40	46.0	even	ok
	443	C53H78N3NaO68P	632.7452	0.8	86.4	453	9.81	18.0	even	ok
	444	C7H11N101NaO19P2	632.7452	0.7	86.5	454	6.59	55.0	even	ok
	445	C35H36N55O42	632.7452	0.7	86.8	455	8.20	47.0	even	ok
	446	C36H38N57NaO34PS	632.7445	-0.3	86.9	456	10.99	48.0	even	ok
	447	C39H46N47NaO40PS	632.7450	0.4	86.9	457	10.66	42.0	even	ok
	448	C26H17N87O16PS2	632.7451	0.6	87.2	458	9.94	64.0	even	ok
	449	C48H83N7NaO62P2S2	632.7452	0.7	87.6	459	9.57	13.0	even	ok
	450	C4H10N103O21P2	632.7447	-0.1	87.7	460	7.40	54.0	even	ok
	451	C28H22N81NaO18PS2	632.7452	0.8	87.8	462	9.26	60.0	even	ok
	452	C34H33N59NaO38	632.7448	0.1	88.5	463	8.91	49.0	even	ok
	453	C32H28N65O36	632.7447	-0.0	88.7	464	9.17	53.0	even	ok
	454	C5H6N107O17P2	632.7451	0.6	88.8	465	6.25	59.0	even	ok
	455	C22H15N89NaO18P2	632.7450	0.5	89.0	466	8.00	62.0	even	ok
	456	C54H77NNaO71	632.7442	-0.8	89.0	467	8.66	18.0	even	ok
	457	C41H48N41O47S	632.7443	-0.6	89.1	468	9.18	40.0	even	ok
	458	C23H9N93NaO16S	632.7450	0.5	89.3	469	7.81	67.0	even	ok
	459	C21H4N99O14S	632.7450	0.3	89.5	470	8.07	71.0	even	ok
	460	C43H53N35NaO49S	632.7444	-0.5	90.0	472	9.22	36.0	even	ok

Compound Spectrum SmartFormula Report

Meas. m/z	#	Ion Formula	m/z	err [ppm]	mSigma	# mSigma	Score	rdb	e⁻ Conf	N-Rule
	461	C44H56N31O53S	632.7448	0.1	90.0	473	10.12	34.0	even	ok
	462	C30H24N75O25S2	632.7446	-0.3	90.0	474	9.73	58.0	even	ok
	463	C20H10N95O16P2	632.7449	0.3	90.0	475	7.97	66.0	even	ok
	464	C29H21N79NaO21S2	632.7442	-0.8	90.2	476	8.21	60.0	even	ok
	465	C52H82N3O66P2S	632.7444	-0.5	90.4	477	8.86	16.0	even	ok
	466	C31H25N69NaO32	632.7444	-0.6	90.7	478	7.22	55.0	even	ok
	467	C40H55N41NaO40P2S2	632.7443	-0.7	90.8	479	8.17	36.0	even	ok
	468	C32H29N69NaO27S2	632.7447	-0.1	90.9	480	9.73	54.0	even	ok
	469	C33H32N65O31S2	632.7450	0.5	90.9	481	8.84	52.0	even	ok
	470	C46H61N25NaO55S	632.7449	0.3	91.0	482	9.31	30.0	even	ok
	471	C29H20N75O30	632.7443	-0.7	91.0	483	6.81	59.0	even	ok
	472	C47H64N21O59S	632.7452	0.8	91.3	484	7.82	28.0	even	ok
	473	C41H58N37O44P2S2	632.7446	-0.2	91.3	485	9.37	34.0	even	ok
	474	C44H58N31O51P2	632.7452	0.8	91.7	487	6.46	34.0	even	ok
	475	C3H7N107NaO17P2	632.7443	-0.7	91.8	488	5.31	56.0	even	ok
	476	C35H37N59NaO33S2	632.7451	0.6	91.9	489	8.10	48.0	even	ok
	477	C43H63N31NaO46P2S2	632.7447	-0.0	92.6	490	9.20	30.0	even	ok
	478	C41H50N41O45P2	632.7448	0.1	92.7	491	7.56	40.0	even	ok
	479	C50H85NO69PS2	632.7445	-0.3	92.8	492	8.41	11.0	even	ok
	480	C4H3N111NaO13P2	632.7447	0.0	92.8	493	6.08	61.0	even	ok
	481	C43H55N35NaO47P2	632.7449	0.2	92.9	494	7.21	36.0	even	ok
	482	C19H7N99NaO12P2	632.7446	-0.2	93.0	495	7.11	68.0	even	ok
	483	C27H18N81NaO23P	632.7449	0.3	93.1	496	6.97	61.0	even	ok
	484	C44H66N27O50P2S2	632.7451	0.5	93.3	497	7.78	28.0	even	ok
	485	C30H26N75O23P2S	632.7450	0.4	93.6	498	6.61	58.0	even	ok
	486	C25H13N87O21P	632.7448	0.2	93.7	499	7.04	65.0	even	ok
	487	C32H31N69NaO25P2S	632.7451	0.6	93.8	500	6.27	54.0	even	ok
	488	C38H42N51O39P2	632.7443	-0.6	94.0	501	6.07	46.0	even	ok
	489	C40H47N45NaO41P2	632.7444	-0.5	94.1	502	6.32	42.0	even	ok
	490	C34H33N63O32PS	632.7444	-0.4	94.6	506	6.25	52.0	even	ok
	491	C46H71N21NaO52P2S2	632.7452	0.7	94.7	507	7.00	24.0	even	ok
	492	C27H18N85O17P2S	632.7445	-0.3	94.9	508	6.44	64.0	even	ok
	493	C29H23N79NaO19P2S	632.7446	-0.1	95.0	509	6.68	60.0	even	ok
	494	C49H61N23O56P	632.7451	0.6	95.1	510	6.99	33.0	even	ok
	495	C46H53N33O50P	632.7447	-0.1	95.1	511	8.05	39.0	even	ok
	496	C23H9N97O10PS2	632.7447	-0.1	95.6	512	6.57	70.0	even	ok
	497	C48H58N27NaO52P	632.7448	0.1	95.7	513	7.95	35.0	even	ok
	498	C51H66N17NaO58P	632.7452	0.8	95.8	514	6.49	29.0	even	ok
	499	C25H14N91NaO12PS2	632.7448	0.0	96.1	515	6.50	66.0	even	ok
	500	C24H10N91NaO17P	632.7445	-0.4	96.3	517	5.86	67.0	even	ok
675.4419	1	C55H135N6NaO24S2	675.4428	1.3	13.1	1	68.80	-8.0	even	ok
	2	C56H139N2O26PS2	675.4392	-4.0	13.3	2	18.86	-10.0	even	ok
	3	C54H8N15NaO21P2S2	675.4411	-1.1	13.3	3	73.77	60.0	even	ok
	4	C55H11N11O25P2S2	675.4417	-0.3	13.3	4	95.40	58.0	even	ok
	5	C53H130N12O22S2	675.4427	1.1	13.4	5	73.77	-4.0	even	ok
	6	C53H2N19O22PS2	675.4395	-3.6	13.5	6	24.07	64.0	even	ok

Compound Spectrum SmartFormula Report

Meas. m/z	#	Ion Formula	m/z	err [ppm]	mSigma	# mSigma	Score	rdb	e⁻ Conf	N-Rule
	7	C56H138N2O28S2	675.4433	2.1	13.6	7	49.43	-10.0	even	ok
	8	C52H3N21O19P2S2	675.4410	-1.3	13.7	8	67.83	64.0	even	ok
	9	C57H16N5NaO27P2S2	675.4418	-0.1	13.9	10	100.00	54.0	even	ok
	10	C55H7N13NaO24PS2	675.4396	-3.3	14.0	11	26.80	60.0	even	ok
	11	C55H140N4O23P2S2	675.4407	-1.8	14.1	12	57.13	-10.0	even	ok
	12	C54H136N8NaO21PS2	675.4443	3.5	14.2	13	23.84	-8.0	even	ok
	13	C56H136N8O19P2S2	675.4414	-0.8	14.4	14	81.76	-5.0	even	ok
	14	C52H5N15O28S2	675.4429	1.6	14.4	15	61.60	59.0	even	ok
	15	C54H10N9NaO30S2	675.4431	1.8	14.4	16	56.68	55.0	even	ok
	16	C52H127N16NaO18S2	675.4421	0.3	14.5	17	93.34	-2.0	even	ok
	17	C51H2N19NaO24S2	675.4424	0.8	14.6	18	81.58	61.0	even	ok
	18	C58H141N2NaO21P2S2	675.4415	-0.6	14.7	19	86.83	-9.0	even	ok
	19	C55H6N13NaO26S2	675.4438	2.8	14.7	20	36.02	60.0	even	ok
	20	C56H10N9O28PS2	675.4402	-2.6	15.0	21	39.42	58.0	even	ok
	21	C55H14N5O32PS2	675.4395	-3.5	15.1	22	23.42	53.0	even	ok
	22	C54H137N8NaO19P2S2	675.4402	-2.6	15.2	23	39.34	-8.0	even	ok
	23	C53H11N11NaO27PS2	675.4446	4.0	15.2	25	18.13	55.0	even	ok
	24	C55H133N12NaO15P2S2	675.4408	-1.6	15.2	26	60.45	-3.0	even	ok
	25	C52H131N14O19PS2	675.4441	3.3	15.3	27	26.21	-4.0	even	ok
	26	C56H20NNaO31P2S2	675.4411	-1.1	15.4	28	70.91	49.0	even	ok
	27	C58H19NO31P2S2	675.4423	0.7	15.4	29	83.12	52.0	even	ok
	28	C54H127N16O16PS2	675.4392	-4.0	15.5	30	17.96	1.0	even	ok
	29	C54H126N16O18S2	675.4433	2.1	15.5	31	47.74	1.0	even	ok
	30	C56H132N10NaO18PS2	675.4393	-3.8	15.6	32	20.29	-3.0	even	ok
	31	C54H15N7O29P2S2	675.4410	-1.3	15.6	33	65.50	53.0	even	ok
	32	C55H13N5O34S2	675.4436	2.6	15.7	34	38.91	53.0	even	ok
	33	C50H122N22O16S2	675.4420	0.1	15.7	35	96.71	2.0	even	ok
	34	C57H135N6O22PS2	675.4399	-3.0	15.8	36	31.35	-5.0	even	ok
	35	C53H12N11NaO25P2S2	675.4405	-2.1	15.9	37	47.29	55.0	even	ok
	36	C53H128N18O13P2S2	675.4407	-1.8	15.9	38	54.93	1.0	even	ok
	37	C51H6N17O25PS2	675.4444	3.8	16.0	39	20.23	59.0	even	ok
	38	C56H131N10NaO20S2	675.4435	2.3	16.0	40	43.08	-3.0	even	ok
	39	C56H9N9O30S2	675.4443	3.5	16.2	41	22.93	58.0	even	ok
	40	C53H123N20NaO14S2	675.4428	1.3	16.3	42	64.85	3.0	even	ok
	41	C58H15N3NaO30PS2	675.4403	-2.3	16.3	43	42.31	54.0	even	ok
	42	C52H132N14O17P2S2	675.4400	-2.8	16.5	44	34.62	-4.0	even	ok
	43	C58H9N5O34S	675.4419	0.1	16.5	45	97.00	58.0	even	ok
	44	C51H118N26O12S2	675.4426	1.1	16.7	46	69.35	7.0	even	ok
	45	C57H134N6O24S2	675.4440	3.1	16.7	47	28.78	-5.0	even	ok
	46	C59H140NaO24PS2	675.4400	-2.8	16.8	48	34.11	-9.0	even	ok
	47	C52H124N22NaO11PS2	675.4443	3.5	16.9	49	22.70	3.0	even	ok
	48	C51H7N17O23P2S2	675.4403	-2.3	16.9	50	42.20	59.0	even	ok
	49	C54H139N2NaO28S2	675.4421	0.3	17.0	51	88.66	-13.0	even	ok
	50	C50H3N21NaO21PS2	675.4439	3.0	17.0	52	30.68	61.0	even	ok
	51	C51H128N18NaO15PS2	675.4436	2.6	17.1	53	37.89	-2.0	even	ok
	52	C55H4N19NaO17P2S2	675.4418	-0.1	17.4	54	93.09	65.0	even	ok

Compound Spectrum SmartFormula Report

Meas. m/z	#	Ion Formula	m/z	err [ppm]	mSigma	# mSigma	Score	rdb	e⁻ Conf	N-Rule
	53	C59H15NNaO33PS	675.4436	2.5	17.6	55	38.77	54.0	even	ok
	54	C56H7N15O21P2S2	675.4423	0.6	17.6	56	79.67	63.0	even	ok
	55	C58H14N3NaO32S2	675.4444	3.8	17.8	57	19.70	54.0	even	ok
	56	C52H134N8O26S2	675.4420	0.1	17.8	58	92.43	-9.0	even	ok
	57	C50H119N28O9PS2	675.4441	3.3	17.9	59	24.94	7.0	even	ok
	58	C49H119N26NaO12S2	675.4414	-0.7	17.9	60	78.85	4.0	even	ok
	59	C52H125N22NaO9P2S2	675.4402	-2.6	17.9	61	37.06	3.0	even	ok
	60	C59H139NaO26S2	675.4441	3.3	18.0	62	25.07	-9.0	even	ok
	61	C61H15NO31P2S	675.4406	-1.8	18.2	63	50.82	57.0	even	ok
	62	C50H4N21NaO19P2S2	675.4398	-3.1	18.3	64	27.73	61.0	even	ok
	63	C50H115N30NaO8S2	675.4421	0.3	18.6	65	86.15	9.0	even	ok
	64	C51H129N18NaO13P2S2	675.4395	-3.6	18.7	66	21.72	-2.0	even	ok
	65	C49H123N24O13PS2	675.4435	2.3	18.8	67	40.35	2.0	even	ok
	66	C58H12N9NaO23P2S2	675.4425	0.9	18.8	68	72.73	59.0	even	ok
	67	C51H131N12NaO22S2	675.4415	-0.7	18.8	69	77.54	-7.0	even	ok
	68	C54H143O29PS2	675.4442	3.3	18.8	70	24.25	-15.0	even	ok
	69	C57H6N9NaO30S	675.4414	-0.7	18.9	71	75.46	60.0	even	ok
	70	C57H13NO38S	675.4413	-0.9	19.1	72	70.38	53.0	even	ok
	71	C61H10N3NaO32S	675.4427	1.3	19.1	73	62.73	59.0	even	ok
	72	C59H16NNaO31P2S	675.4394	-3.6	19.2	74	20.65	54.0	even	ok
	73	C57H10N7O31PS	675.4434	2.3	19.2	75	41.26	58.0	even	ok
	74	C60H11N5NaO29PS	675.4442	3.5	19.4	76	22.41	59.0	even	ok
	75	C59H5N9O30S	675.4426	1.0	19.4	77	67.08	63.0	even	ok
	76	C53H140N4NaO25PS2	675.4436	2.6	19.5	78	35.96	-13.0	even	ok
	77	C59H134N2O28S	675.4416	-0.4	19.6	79	83.26	-5.0	even	ok
	78	C56H3N17NaO20PS2	675.4403	-2.4	19.6	80	39.35	65.0	even	ok
	79	C54H144O27P2S2	675.4400	-2.8	19.7	81	32.56	-15.0	even	ok
	80	C53H14N5NaO34S2	675.4424	0.8	20.0	82	72.80	50.0	even	ok
	81	C59H15N5O27P2S2	675.4430	1.6	20.1	83	52.87	57.0	even	ok
	82	C58H6N11O27PS	675.4441	3.3	20.3	84	24.63	63.0	even	ok
	83	C49H126N18O20S2	675.4413	-0.9	20.3	85	70.24	-3.0	even	ok
	84	C60H12N5NaO27P2S	675.4401	-2.6	20.3	86	34.05	59.0	even	ok
	85	C57H132N12O15P2S2	675.4420	0.2	20.4	87	85.59	0.0	even	ok
	86	C51H9N11O32S2	675.4423	0.6	20.5	88	76.90	54.0	even	ok
	87	C53H141N4NaO23P2S2	675.4395	-3.5	20.6	90	20.93	-13.0	even	ok
	88	C57H6N13O24PS2	675.4408	-1.6	20.6	91	53.97	63.0	even	ok
	89	C56H2N17NaO22S2	675.4444	3.7	20.7	92	18.61	65.0	even	ok
	90	C50H120N28O7P2S2	675.4400	-2.8	20.7	93	26.31	7.0	even	ok
	91	C54H124N22O9P2S2	675.4414	-0.8	20.7	94	71.49	6.0	even	ok
	92	C54H17NO38S2	675.4429	1.6	20.8	95	53.87	48.0	even	ok
	93	C51H135N10O23PS2	675.4435	2.4	20.8	96	38.53	-9.0	even	ok
	94	C50H6N15NaO28S2	675.4417	-0.2	20.8	97	84.71	56.0	even	ok
	95	C56H129N16NaO11P2S2	675.4415	-0.6	20.8	98	76.25	2.0	even	ok
	96	C57H11N7O29P2S	675.4393	-3.8	20.9	99	17.62	58.0	even	ok
	97	C47H114N32O10S2	675.4413	-0.9	21.2	100	57.32	8.0	even	ok
	98	C59H137N6NaO17P2S2	675.4422	0.4	21.2	101	79.17	-4.0	even	ok

Compound Spectrum SmartFormula Report

Meas. m/z	#	Ion Formula	m/z	err [ppm]	mSigma	# mSigma	Score	rdb	e¯ Conf	N-Rule
	99	C48H110N36O6S2	675.4420	0.1	21.2	102	71.98	13.0	even	ok
	100	C56H10N5NaO34S	675.4407	-1.7	21.4	103	49.94	55.0	even	ok
	101	C58H2N13NaO26S	675.4421	0.3	21.4	104	82.82	65.0	even	ok
	102	C60H140N2O21P2S2	675.4427	1.2	21.8	105	60.05	-6.0	even	ok
	103	C55H123N20O12PS2	675.4399	-3.0	22.0	107	27.40	6.0	even	ok
	104	C52H15N7NaO31PS2	675.4439	3.0	22.0	108	27.47	50.0	even	ok
	105	C53H18N3O35PS2	675.4444	3.8	22.1	109	17.70	48.0	even	ok
	106	C49H124N24O11P2S2	675.4394	-3.8	22.1	110	14.92	2.0	even	ok
	107	C51H136N10O21P2S2	675.4394	-3.8	22.1	111	17.98	-9.0	even	ok
	108	C49H116N32NaO5PS2	675.4436	2.5	22.1	112	28.50	9.0	even	ok
	109	C59H11N7NaO26PS2	675.4410	-1.4	22.2	113	56.51	59.0	even	ok
	110	C48H123N22NaO16S2	675.4408	-1.7	22.2	114	50.45	-1.0	even	ok
	111	C50H132N14NaO19PS2	675.4429	1.6	22.3	115	52.19	-7.0	even	ok
	112	C55H122N20O14S2	675.4440	3.1	22.5	116	25.56	6.0	even	ok
	113	C52H114N30O8S2	675.4433	2.1	22.5	117	41.32	12.0	even	ok
	114	C53H19N3O33P2S2	675.4403	-2.3	22.5	118	37.53	48.0	even	ok
	115	C57H128N14NaO14PS2	675.4400	-2.8	22.6	119	30.02	2.0	even	ok
	116	C54H120N24NaO8PS2	675.4393	-3.8	22.7	120	17.40	8.0	even	ok
	117	C52H115N30O6PS2	675.4392	-4.0	22.7	121	15.35	12.0	even	ok
	118	C54H119N24NaO10S2	675.4435	2.3	22.8	122	37.47	8.0	even	ok
	119	C56H14N3O35PS	675.4428	1.3	22.8	123	57.31	53.0	even	ok
	120	C52H16N7NaO29P2S2	675.4398	-3.1	22.8	124	25.25	50.0	even	ok
	121	C58H131N10O18PS2	675.4405	-2.0	22.9	125	43.03	0.0	even	ok
	122	C50H10N13O29PS2	675.4438	2.8	23.0	126	29.87	54.0	even	ok
	123	C48H120N28NaO9PS2	675.4429	1.6	23.1	127	42.87	4.0	even	ok
	124	C58H7N11O25P2S	675.4400	-2.8	23.2	128	24.04	63.0	even	ok
	125	C54H5N11O32S	675.4406	-1.9	23.3	129	43.95	59.0	even	ok
	126	C62H135NaO26S	675.4425	0.8	23.3	130	66.54	-4.0	even	ok
	127	C57H127N14NaO16S2	675.4441	3.3	23.3	131	22.42	2.0	even	ok
	128	C49H7N17NaO25PS2	675.4432	2.0	23.8	132	42.29	56.0	even	ok
	129	C56H7N11NaO27PS	675.4429	1.5	23.8	133	43.32	60.0	even	ok
	130	C49H117N32NaO3P2S2	675.4395	-3.6	23.8	134	16.11	9.0	even	ok
	131	C47H111N38O3PS2	675.4435	2.3	23.8	135	30.21	13.0	even	ok
	132	C58H135N4O25PS	675.4431	1.8	23.9	136	37.43	-5.0	even	ok
	133	C53H121N26NaO5P2S2	675.4408	-1.6	24.0	137	41.70	8.0	even	ok
	134	C50H11N13O27P2S2	675.4397	-3.3	24.0	138	22.00	54.0	even	ok
	135	C48H127N20O17PS2	675.4428	1.4	24.0	139	54.38	-3.0	even	ok
	136	C60H14N3O30PS2	675.4415	-0.6	24.0	140	71.16	57.0	even	ok
	137	C46H118N28O14S2	675.4406	-1.9	24.0	141	44.51	3.0	even	ok
	138	C58H131N6NaO24S	675.4411	-1.2	24.1	142	48.48	-3.0	even	ok
	139	C60H136N4NaO20PS2	675.4407	-1.8	24.1	143	45.67	-4.0	even	ok
	140	C62H11N5O27P2S	675.4413	-0.9	24.2	144	64.69	62.0	even	ok
	141	C47H107N40NaO2S2	675.4414	-0.7	24.2	145	57.18	15.0	even	ok
	142	C46H111N36NaO6S2	675.4408	-1.7	24.3	146	39.98	10.0	even	ok
	143	C58H5N11O27S2	675.4393	-3.8	24.3	147	16.81	63.0	even	ok
	144	C51H116N32O3P2S2	675.4407	-1.8	24.6	148	37.81	12.0	even	ok

Compound Spectrum SmartFormula Report

Meas. m/z	#	Ion Formula	m/z	err [ppm]	mSigma	# mSigma	Score	rdb	e¯ Conf	N-Rule
	145	C57H135N2NaO28S	675.4404	-2.2	24.7	149	32.22	-8.0	even	ok
	146	C57H3N15NaO23PS	675.4436	2.5	24.7	150	27.78	65.0	even	ok
	147	C46H115N34O7PS2	675.4428	1.3	25.3	152	44.18	8.0	even	ok
	148	C47H2N23O23PS2	675.4431	1.8	25.3	153	44.63	60.0	even	ok
	149	C55H11N7NaO31PS	675.4422	0.5	25.3	154	70.74	55.0	even	ok
	150	C61H139O24PS2	675.4412	-1.0	25.3	155	59.94	-6.0	even	ok
	151	C63H10N3O30PS	675.4398	-3.1	25.5	156	24.42	62.0	even	ok
	152	C51H138N4O30S2	675.4413	-0.9	25.5	157	62.76	-14.0	even	ok
	153	C57H3N19O17P2S2	675.4430	1.6	25.5	158	47.10	68.0	even	ok
	154	C61H136N2NaO23PS	675.4439	3.0	25.6	159	20.52	-4.0	even	ok
	155	C47H112N38OP2S2	675.4393	-3.8	25.7	160	13.69	13.0	even	ok
	156	C57H139O29PS	675.4425	0.9	25.7	161	52.02	-10.0	even	ok
	157	C60H130N6O24S	675.4423	0.6	25.7	162	56.12	0.0	even	ok
	158	C51H111N34NaO4S2	675.4428	1.3	25.8	163	44.07	14.0	even	ok
	159	C63H9N3O32S	675.4439	3.0	25.9	164	24.54	62.0	even	ok
	160	C56H126N12O22S	675.4410	-1.4	25.9	165	43.14	1.0	even	ok
	161	C45H102N46S2	675.4413	-0.9	25.9	166	51.38	19.0	even	ok
	162	C54H2N17O25PS	675.4428	1.3	25.9	167	44.63	64.0	even	ok
	163	C50H112N36NaOPS2	675.4443	3.5	26.1	168	15.54	14.0	even	ok
	164	C60H10N5NaO29S2	675.4395	-3.6	26.1	169	18.20	59.0	even	ok
	165	C49H133N16NaO16P2S2	675.4444	3.8	26.1	170	16.19	-7.0	even	ok
	166	C49H106N40O2S2	675.4426	1.1	26.3	172	47.00	18.0	even	ok
	167	C62H7N7NaO26PS	675.4393	-3.9	26.3	173	15.38	64.0	even	ok
	168	C50H135N8NaO26S2	675.4408	-1.6	26.3	174	46.10	-12.0	even	ok
	169	C57H4N15NaO21P2S	675.4394	-3.6	26.3	175	14.62	65.0	even	ok
	170	C62H6N7NaO28S	675.4434	2.2	26.4	176	35.65	64.0	even	ok
	171	C44H106N42O4S2	675.4406	-1.9	26.5	177	34.93	14.0	even	ok
	172	C59H131N8O21PS	675.4438	2.8	26.7	178	22.26	0.0	even	ok
	173	C59H8N13NaO19P2S2	675.4431	1.8	26.8	179	42.03	64.0	even	ok
	174	C61H137N2NaO21P2S	675.4398	-3.1	26.8	180	19.77	-4.0	even	ok
	175	C55H15N5O32P2S	675.4443	3.5	26.9	181	18.68	53.0	even	ok
	176	C55H130N8O26S	675.4403	-2.4	26.9	182	27.88	-4.0	even	ok
	177	C57H132N8NaO21PS	675.4426	1.1	27.3	183	46.88	-3.0	even	ok
	178	C55H14NNaO38S	675.4401	-2.7	27.3	184	28.00	50.0	even	ok
	179	C52H144NaO29PS2	675.4430	1.6	27.4	185	46.36	-18.0	even	ok
	180	C47H121N30NaO6P2S2	675.4444	3.8	27.4	186	13.16	4.0	even	ok
	181	C61H135N2NaO23S2	675.4392	-4.0	27.7	187	13.59	-4.0	even	ok
	182	C48H130N14O24S2	675.4406	-1.9	27.7	188	40.96	-8.0	even	ok
	183	C61H8N9NaO23P2S	675.4408	-1.6	27.8	189	37.09	64.0	even	ok
	184	C55H8N13NaO24P2S	675.4444	3.7	27.9	190	13.56	60.0	even	ok
	185	C60H11N9O23P2S2	675.4437	2.6	27.9	191	28.63	62.0	even	ok
	186	C52H18NNaO38S2	675.4417	-0.2	28.0	192	72.04	45.0	even	ok
	187	C59H132N8O19P2S	675.4397	-3.3	28.0	193	17.18	0.0	even	ok
	188	C59H127N10NaO20S	675.4418	-0.2	28.1	194	60.66	2.0	even	ok
	189	C53H2N15NaO28S	675.4401	-2.7	28.2	195	22.73	61.0	even	ok
	190	C61H13NO33S2	675.4400	-2.8	28.3	196	26.42	57.0	even	ok

Compound Spectrum SmartFormula Report

Meas. m/z	#	Ion Formula	m/z	err [ppm]	mSigma	# mSigma	Score	rdb	e⁻ Conf	N-Rule
	191	C47H124N24NaO13PS2	675.4423	0.6	28.4	197	53.66	-1.0	even	ok
	192	C59H3N15O21P2S	675.4406	-1.8	28.4	198	33.64	68.0	even	ok
	193	C55H120N26O5P2S2	675.4420	0.2	28.5	199	71.44	11.0	even	ok
	194	C45H112N38NaO3PS2	675.4423	0.6	28.5	200	53.62	10.0	even	ok
	195	C50H139N6O27PS2	675.4428	1.4	28.7	201	48.67	-14.0	even	ok
	196	C45H115N32NaO10S2	675.4401	-2.6	28.7	202	23.25	5.0	even	ok
	197	C50H13N7O36S2	675.4416	-0.4	28.8	203	66.60	49.0	even	ok
	198	C58H128N16O11P2S2	675.4427	1.2	28.8	204	51.30	5.0	even	ok
	199	C58H2N17O20PS2	675.4415	-0.6	28.9	205	63.40	68.0	even	ok
	200	C57H125N20NaO7P2S2	675.4422	0.4	29.0	206	66.45	7.0	even	ok
	201	C56H136N4NaO25PS	675.4419	0.1	29.0	207	61.26	-8.0	even	ok
	202	C46H3N25O20P2S2	675.4446	4.0	29.0	208	13.24	60.0	even	ok
	203	C49H10N11NaO32S2	675.4411	-1.2	29.1	209	50.90	51.0	even	ok
	204	C53H9N7O36S	675.4399	-2.9	29.2	210	24.15	54.0	even	ok
	205	C57H122N16O18S	675.4416	-0.4	29.2	211	55.67	6.0	even	ok
	206	C47H127N18NaO20S2	675.4401	-2.6	29.2	212	27.69	-6.0	even	ok
	207	C55H127N14O19PS	675.4425	0.8	29.3	213	47.93	1.0	even	ok
	208	C55H123N16NaO18S	675.4404	-2.2	29.3	214	28.79	3.0	even	ok
	209	C62H16N3NaO25P2S2	675.4438	2.8	29.6	215	24.82	58.0	even	ok
	210	C58H128N12NaO17PS	675.4433	2.0	29.6	216	30.07	2.0	even	ok
	211	C45H116N36O4P2S2	675.4443	3.6	29.7	217	14.05	8.0	even	ok
	212	C53H6N13O29PS	675.4421	0.3	29.7	218	56.49	59.0	even	ok
	213	C64H135O24PS	675.4395	-3.5	29.8	219	17.46	-1.0	even	ok
	214	C60H133N10NaO13P2S2	675.4428	1.4	29.8	220	46.37	1.0	even	ok
	215	C49H136N10NaO23PS2	675.4423	0.6	29.9	221	62.05	-12.0	even	ok
	216	C43H103N46NaS2	675.4401	-2.7	29.9	222	22.51	16.0	even	ok
	217	C64H134O26S	675.4437	2.6	30.0	223	27.65	-1.0	even	ok
	218	C53H3N19O22P2S	675.4442	3.5	30.1	224	14.46	64.0	even	ok
	219	C47H5N17O30S2	675.4409	-1.4	30.2	225	45.81	55.0	even	ok
	220	C56H140N2O26P2S	675.4440	3.1	30.2	226	18.16	-10.0	even	ok
	221	C54H127N12NaO22S	675.4398	-3.1	30.3	227	17.36	-2.0	even	ok
	222	C60H7N11NaO22PS2	675.4416	-0.4	30.4	228	65.04	64.0	even	ok
	223	C47H128N22O14P2S2	675.4443	3.6	30.5	229	13.73	-3.0	even	ok
	224	C61H136N6O17P2S2	675.4434	2.2	30.5	230	32.93	-1.0	even	ok
	225	C51H19N3NaO35PS2	675.4432	2.0	30.5	231	36.05	45.0	even	ok
	226	C56H119N24O8PS2	675.4405	-2.0	30.5	232	35.92	11.0	even	ok
	227	C45H119N30O11PS2	675.4421	0.4	30.6	233	54.20	3.0	even	ok
	228	C51H145N2NaO26P2S2	675.4444	3.8	30.7	234	14.46	-18.0	even	ok
	229	C53H110N34O4S2	675.4440	3.1	30.7	235	21.18	17.0	even	ok
	230	C63H131N4NaO22S	675.4431	1.8	30.8	236	38.60	1.0	even	ok
	231	C55H116N28NaO4PS2	675.4400	-2.8	30.9	239	24.66	13.0	even	ok
	232	C43H107N44OPS2	675.4421	0.3	30.9	240	53.97	14.0	even	ok
	233	C45H122N24O18S2	675.4400	-2.8	31.0	241	23.95	-2.0	even	ok
	234	C56H133N10NaO18P2S	675.4441	3.3	31.0	242	16.02	-3.0	even	ok
	235	C43H110N38O8S2	675.4400	-2.9	31.0	243	19.86	9.0	even	ok
	236	C56H123N18O15PS	675.4431	1.8	31.1	244	31.66	6.0	even	ok

Compound Spectrum SmartFormula Report

Meas. m/z	#	Ion Formula	m/z	err [ppm]	mSigma	# mSigma	Score	rdb	e⁻ Conf	N-Rule
	237	C46H2N21NaO26S2	675.4404	-2.2	31.2	245	32.28	57.0	even	ok
	238	C55H115N28NaO6S2	675.4441	3.3	31.3	246	18.68	13.0	even	ok
	239	C63H136N2O21P2S	675.4410	-1.3	31.3	247	39.18	-1.0	even	ok
	240	C54H131N10O23PS	675.4418	-0.1	31.3	248	56.58	-4.0	even	ok
	241	C53H118N22O16S	675.4403	-2.4	31.3	249	24.96	7.0	even	ok
	242	C58H124N18NaO10PS2	675.4407	-1.8	31.4	250	38.36	7.0	even	ok
	243	C54H15N3NaO35PS	675.4416	-0.5	31.4	251	61.09	50.0	even	ok
	244	C47H131N16O21PS2	675.4421	0.4	31.5	252	63.51	-8.0	even	ok
	245	C49H14N9O33PS2	675.4431	1.8	31.5	253	38.33	49.0	even	ok
	246	C54H134N4O30S	675.4396	-3.3	31.7	254	15.06	-9.0	even	ok
	247	C59H127N14O14PS2	675.4412	-1.0	31.8	255	51.27	5.0	even	ok
	248	C63H141NaO19P2S2	675.4435	2.4	31.9	256	28.92	-5.0	even	ok
	249	C54H117N30NaOP2S2	675.4415	-0.6	32.0	257	48.92	13.0	even	ok
	250	C61H10N7O26PS2	675.4422	0.4	32.0	258	61.66	62.0	even	ok
	251	C54H12N9NaO28P2S	675.4437	2.7	32.1	259	20.81	55.0	even	ok
	252	C56H119N20NaO14S	675.4411	-1.2	32.2	260	39.93	8.0	even	ok
	253	C49H140N8O24P2S2	675.4443	3.6	32.2	261	15.71	-14.0	even	ok
	254	C48H11N13NaO29PS2	675.4426	1.0	32.2	262	50.84	51.0	even	ok
	255	C63H7N9O23P2S	675.4420	0.1	32.3	263	66.36	67.0	even	ok
	256	C52H122N18O20S	675.4396	-3.4	32.6	264	14.65	2.0	even	ok
	257	C65H12N3NaO25P2S	675.4421	0.3	32.7	266	61.83	63.0	even	ok
	258	C52H3N17NaO25PS	675.4416	-0.5	32.9	267	48.94	61.0	even	ok
	259	C54H124N18NaO15PS	675.4419	0.1	32.9	268	55.74	3.0	even	ok
	260	C44H113N40NaP2S2	675.4438	2.8	33.1	269	19.72	10.0	even	ok
	261	C61H132N8NaO16PS2	675.4414	-0.8	33.1	270	53.23	1.0	even	ok
	262	C46H125N26NaO10P2S2	675.4438	2.8	33.1	271	19.64	-1.0	even	ok
	263	C60H127N12O17PS	675.4445	3.8	33.1	272	11.07	5.0	even	ok
	264	C53H111N34O2PS2	675.4399	-3.0	33.1	273	17.47	17.0	even	ok
	265	C46H128N20NaO17PS2	675.4416	-0.4	33.2	274	59.67	-6.0	even	ok
	266	C54H128N16O16P2S	675.4440	3.1	33.2	275	16.93	1.0	even	ok
	267	C62H133N6NaO17P2S	675.4405	-2.1	33.2	276	27.19	1.0	even	ok
	268	C44H116N34NaO7PS2	675.4416	-0.4	33.4	277	49.30	5.0	even	ok
	269	C54H19NO36P2S	675.4436	2.5	33.5	278	26.59	48.0	even	ok
	270	C61H126N10O20S	675.4430	1.6	33.5	279	32.72	5.0	even	ok
	271	C55H137N6NaO22P2S	675.4434	2.3	33.6	280	24.61	-8.0	even	ok
	272	C46H6N19O27PS2	675.4424	0.8	33.6	281	52.66	55.0	even	ok
	273	C54H114N26O12S	675.4410	-1.4	33.6	282	35.67	12.0	even	ok
	274	C60H9NO38	675.4396	-3.4	33.6	283	13.73	58.0	even	ok
	275	C48H137N12NaO20P2S2	675.4438	2.8	33.7	284	23.11	-12.0	even	ok
	276	C61H5N5O34	675.4402	-2.4	33.7	286	22.76	63.0	even	ok
	277	C63H15NNaO28PS2	675.4423	0.6	33.8	287	55.18	58.0	even	ok
	278	C60H128N12O15P2S	675.4404	-2.3	33.8	288	24.41	5.0	even	ok
	279	C42H107N42NaO4S2	675.4394	-3.6	34.1	289	11.99	11.0	even	ok
	280	C62H135N4O20PS2	675.4419	-0.0	34.1	290	65.69	-1.0	even	ok
	281	C55H140NaO29PS	675.4413	-0.9	34.1	291	41.37	-13.0	even	ok
	282	C52H6N11NaO32S	675.4394	-3.7	34.2	292	11.50	56.0	even	ok

Compound Spectrum SmartFormula Report

Meas. m/z	#	Ion Formula	m/z	err [ppm]	mSigma	# mSigma	Score	rdb	e⁻ Conf	N-Rule
	283	C50H142O34S2	675.4406	-1.8	34.2	293	35.02	-19.0	even	ok
	284	C64H6N7O26PS	675.4405	-2.1	34.3	294	31.73	67.0	even	ok
	285	C52H107N38NaS2	675.4435	2.3	34.3	295	23.79	19.0	even	ok
	286	C55H120N22NaO11PS	675.4426	1.0	34.4	296	39.41	8.0	even	ok
	287	C61H6N9NaO25S2	675.4401	-2.6	34.5	297	24.99	64.0	even	ok
	288	C52H7N15O26P2S	675.4436	2.5	34.5	298	21.68	59.0	even	ok
	289	C64H10NO33P	675.4431	1.8	34.5	299	29.96	62.0	even	ok
	290	C48H15N11O30P2S2	675.4446	4.0	34.7	300	11.44	49.0	even	ok
	291	C63H3N11NaO22PS	675.4400	-2.9	34.7	301	21.60	69.0	even	ok
	292	C53H128N14NaO19PS	675.4413	-0.9	34.8	302	40.58	-2.0	even	ok
	293	C45H3N23NaO23PS2	675.4419	0.0	34.8	303	64.64	57.0	even	ok
	294	C49H139N4NaO30S2	675.4401	-2.6	34.9	304	24.11	-17.0	even	ok
	295	C52H115N26NaO12S	675.4398	-3.2	35.0	305	15.37	9.0	even	ok
	296	C66H11NNaO28PS	675.4406	-1.9	35.0	306	33.96	63.0	even	ok
	297	C62H11NNaO33P	675.4419	-0.0	35.1	307	53.35	59.0	even	ok
	298	C63H2N11NaO24S	675.4441	3.2	35.1	308	17.62	69.0	even	ok
	299	C52H119N24O13PS	675.4418	-0.2	35.1	309	51.31	7.0	even	ok
	300	C60H126N12O17S2	675.4397	-3.2	35.2	310	17.72	5.0	even	ok
	301	C60H123N14NaO16S	675.4424	0.8	35.2	311	41.72	7.0	even	ok
	302	C44H120N32O8P2S2	675.4436	2.6	35.3	312	20.51	3.0	even	ok
	303	C59H124N16NaO13PS	675.4439	3.0	35.4	313	16.24	7.0	even	ok
	304	C46H132N18O18P2S2	675.4436	2.6	35.4	314	24.46	-8.0	even	ok
	305	C47H12N15NaO26P2S2	675.4441	3.2	35.7	315	17.56	51.0	even	ok
	306	C58H118N20O14S	675.4423	0.6	35.7	316	44.05	11.0	even	ok
	307	C64H6N3NaO32	675.4411	-1.2	35.8	317	35.53	64.0	even	ok
	308	C42H111N40O5PS2	675.4415	-0.6	35.8	318	43.56	9.0	even	ok
	309	C44H119N28NaO14S2	675.4394	-3.6	35.9	319	11.49	0.0	even	ok
	310	C53H132N12O20P2S	675.4433	2.1	35.9	320	25.37	-4.0	even	ok
	311	C60H4N17NaO15P2S2	675.4438	2.8	35.9	321	21.26	69.0	even	ok
	312	C53H115N28O9PS	675.4425	0.8	36.2	322	40.42	12.0	even	ok
	313	C62H4N13NaO19P2S	675.4415	-0.7	36.2	323	42.89	69.0	even	ok
	314	C57H119N22O11PS	675.4438	2.8	36.3	324	17.65	11.0	even	ok
	315	C47H134N10O28S2	675.4400	-2.8	36.3	325	21.01	-13.0	even	ok
	316	C62H9N5O29S2	675.4407	-1.8	36.3	326	33.92	62.0	even	ok
	317	C59H125N16NaO11P2S	675.4398	-3.1	36.4	327	15.54	7.0	even	ok
	318	C52H10N9O33PS	675.4414	-0.7	36.4	328	42.04	54.0	even	ok
	319	C40H102N48O2S2	675.4393	-3.9	36.4	329	9.98	15.0	even	ok
	320	C53H135N6O27PS	675.4411	-1.1	36.4	330	36.25	-9.0	even	ok
	321	C52H13N3O40S	675.4393	-3.9	36.7	332	11.51	49.0	even	ok
	322	C62H131N6NaO19S2	675.4399	-3.0	36.7	333	18.99	1.0	even	ok
	323	C63H11N3O30P2	675.4446	4.0	36.8	334	9.17	62.0	even	ok
	324	C53H125N20NaO12P2S	675.4434	2.3	36.9	335	22.65	3.0	even	ok
	325	C53H111N30NaO8S	675.4404	-2.2	37.0	336	23.66	14.0	even	ok
	326	C61H7N13O19P2S2	675.4443	3.6	37.0	337	13.50	67.0	even	ok
	327	C50H110N32O10S	675.4396	-3.4	37.1	339	12.99	13.0	even	ok
	328	C60H6N7O31P	675.4417	-0.2	37.1	340	47.71	63.0	even	ok

Compound Spectrum SmartFormula Report

Meas. m/z	#	Ion Formula	m/z	err [ppm]	mSigma	# mSigma	Score	rdb	e⁻ Conf	N-Rule
	329	C51H123N20O17PS	675.4411	-1.1	37.2	341	35.46	2.0	even	ok
	330	C45H7N21O24P2S2	675.4439	3.0	37.2	342	18.82	55.0	even	ok
	331	C60H2N9NaO30	675.4397	-3.2	37.3	343	13.92	65.0	even	ok
	332	C54H121N24NaO8P2S	675.4441	3.3	37.3	344	13.68	8.0	even	ok
	333	C57H120N22O9P2S	675.4397	-3.3	37.4	345	13.53	11.0	even	ok
	334	C56H116N30OP2S2	675.4427	1.2	37.4	346	41.43	16.0	even	ok
	335	C49H143N2O31PS2	675.4421	0.4	37.4	347	54.40	-19.0	even	ok
	336	C46H131N14NaO24S2	675.4394	-3.6	37.5	348	13.28	-11.0	even	ok
	337	C51H4N19NaO22P2S	675.4430	1.7	37.7	349	28.22	61.0	even	ok
	338	C63H7N5NaO29P	675.4426	1.0	37.7	350	37.10	64.0	even	ok
	339	C49H17N3O40S2	675.4409	-1.4	37.8	351	37.89	44.0	even	ok
	340	C59H10N3O35P	675.4411	-1.2	38.0	352	33.85	58.0	even	ok
	341	C42H114N34O12S2	675.4393	-3.8	38.0	353	9.61	4.0	even	ok
	342	C48H14N7NaO36S2	675.4404	-2.2	38.0	354	27.15	46.0	even	ok
	343	C57H115N24NaO10S	675.4418	-0.2	38.1	355	47.12	13.0	even	ok
	344	C58H121N24NaO3P2S2	675.4428	1.4	38.1	356	37.64	12.0	even	ok
	345	C63H134N2O23S2	675.4404	-2.2	38.1	357	26.84	-1.0	even	ok
	346	C59H124N20O7P2S2	675.4434	2.2	38.2	358	27.13	10.0	even	ok
	347	C44H123N26O15PS2	675.4415	-0.6	38.2	359	40.93	-2.0	even	ok
	348	C43H117N36NaO4P2S2	675.4431	1.8	38.2	360	26.99	5.0	even	ok
	349	C48H140N6NaO27PS2	675.4416	-0.4	38.4	361	52.25	-17.0	even	ok
	350	C62H130N2O28	675.4400	-2.9	38.5	362	16.28	0.0	even	ok
	351	C65H131N4O20PS	675.4402	-2.5	38.6	363	23.29	4.0	even	ok
	352	C63H12N7NaO21P2S2	675.4445	3.8	38.6	364	11.44	63.0	even	ok
	353	C51H106N36O6S	675.4403	-2.4	38.7	365	20.58	18.0	even	ok
	354	C44H4N25NaO20P2S2	675.4434	2.2	38.7	366	26.53	57.0	even	ok
	355	C61H12N3NaO30P2	675.4434	2.2	38.7	367	22.27	59.0	even	ok
	356	C51H116N28NaO9PS	675.4413	-0.9	38.8	368	36.38	9.0	even	ok
	357	C41H108N44NaOPS2	675.4409	-1.4	38.9	369	30.44	11.0	even	ok
	358	C53H16N5NaO32P2S	675.4431	1.7	38.9	370	27.21	50.0	even	ok
	359	C54H141N2NaO26P2S	675.4428	1.3	38.9	371	32.10	-13.0	even	ok
	360	C55H110N30O8S	675.4416	-0.4	38.9	372	43.31	17.0	even	ok
	361	C65H130N4O22S	675.4443	3.6	39.0	373	12.99	4.0	even	ok
	362	C51H7N13NaO29PS	675.4409	-1.5	39.0	374	29.59	56.0	even	ok
	363	C56H116N26NaO7PS	675.4433	2.0	39.0	375	23.72	13.0	even	ok
	364	C44H126N20O22S2	675.4393	-3.8	39.1	376	11.26	-7.0	even	ok
	365	C46H9N13O34S2	675.4403	-2.4	39.2	377	23.95	50.0	even	ok
	366	C51H120N26O10P2S	675.4433	2.1	39.2	378	23.35	7.0	even	ok
	367	C61H129N14NaO9P2S2	675.4435	2.4	39.3	379	23.96	6.0	even	ok
	368	C61H2N11O27P	675.4424	0.8	39.3	380	38.08	68.0	even	ok
	369	C52H116N30O6P2S	675.4440	3.1	39.3	381	14.49	12.0	even	ok
	370	C52H129N16NaO16P2S	675.4428	1.3	39.4	382	31.77	-2.0	even	ok
	371	C52H132N10NaO23PS	675.4406	-1.9	39.5	383	24.54	-7.0	even	ok
	372	C50H126N16O23S	675.4446	4.0	39.6	384	8.54	-3.0	even	ok
	373	C61H3N15NaO18PS2	675.4423	0.6	39.7	385	47.45	69.0	even	ok
	374	C52H112N32NaO5PS	675.4419	0.0	39.7	386	46.70	14.0	even	ok

Compound Spectrum SmartFormula Report

Meas. m/z	#	Ion Formula	m/z	err [ppm]	mSigma	# mSigma	Score	rdb	e⁻ Conf	N-Rule
	375	C57H115N28O4PS2	675.4412	-1.0	39.9	387	41.40	16.0	even	ok
	376	C46H135N12O25PS2	675.4415	-0.6	39.9	388	47.05	-13.0	even	ok
	377	C62H132N10O13P2S2	675.4441	3.2	39.9	389	15.85	4.0	even	ok
	378	C66H5N3O32	675.4423	0.5	39.9	390	40.22	67.0	even	ok
	379	C45H6N17NaO30S2	675.4397	-3.2	39.9	391	15.78	52.0	even	ok
	380	C62H8N7NaO26P2	675.4440	3.2	40.2	392	13.14	64.0	even	ok
	381	C64H132N6O17P2S	675.4417	-0.3	40.3	393	42.98	4.0	even	ok
	382	C54H111N32O5PS	675.4431	1.8	40.3	394	25.04	17.0	even	ok
	383	C65H6N5O29P	675.4438	2.8	40.3	395	16.40	67.0	even	ok
	384	C66H137NaO19P2S	675.4418	-0.1	40.6	396	45.22	0.0	even	ok
	385	C43H120N30NaO11PS2	675.4409	-1.4	40.6	397	29.15	0.0	even	ok
	386	C48H18N5O37PS2	675.4424	0.8	40.6	398	43.62	44.0	even	ok
	387	C41H112N42O2P2S2	675.4430	1.6	40.6	399	27.51	9.0	even	ok
	388	C45H129N22NaO14P2S2	675.4431	1.8	40.7	400	25.14	-6.0	even	ok
	389	C50H120N24NaO13PS	675.4406	-1.9	40.8	401	23.64	4.0	even	ok
	390	C51H14N5O37PS	675.4407	-1.7	40.8	402	31.29	49.0	even	ok
	391	C48H114N30O13S	675.4446	4.0	40.8	403	8.30	8.0	even	ok
	392	C59H3N11NaO27P	675.4412	-1.0	40.8	404	33.60	65.0	even	ok
	393	C59H120N22NaO6PS2	675.4413	-0.8	40.9	405	43.21	12.0	even	ok
	394	C48H144N4O28P2S2	675.4436	2.6	40.9	406	21.06	-19.0	even	ok
	395	C59H7N9O28P2	675.4432	2.0	40.9	407	22.96	63.0	even	ok
	396	C62H6N11O22PS2	675.4428	1.4	41.0	408	34.73	67.0	even	ok
	397	C49H111N34O7PS	675.4411	-1.2	41.1	409	31.80	13.0	even	ok
	398	C51H11N11O30P2S	675.4429	1.5	41.1	410	27.79	54.0	even	ok
	399	C47H15N9NaO33PS2	675.4419	0.0	41.2	411	54.44	46.0	even	ok
	400	C45H132N16NaO21PS2	675.4409	-1.4	41.3	412	34.39	-11.0	even	ok
	401	C52H136N8O24P2S	675.4426	1.1	41.3	413	32.43	-9.0	even	ok
	402	C60H135O29P	675.4408	-1.6	41.3	414	26.24	-5.0	even	ok
	403	C60H123N18O10PS2	675.4419	-0.0	41.3	415	54.12	10.0	even	ok
	404	C49H2N19O27PS	675.4407	-1.7	41.3	416	25.61	60.0	even	ok
	405	C65H131NaO26	675.4408	-1.7	41.3	417	25.95	1.0	even	ok
	406	C65H7N5O27P2	675.4396	-3.3	41.3	418	11.70	67.0	even	ok
	407	C64H3N13O19P2S	675.4427	1.1	41.3	419	38.18	72.0	even	ok
	408	C64H137N4NaO15P2S2	675.4442	3.4	41.3	420	13.61	0.0	even	ok
	409	C52H138N2O33S	675.4446	4.0	41.5	422	8.07	-14.0	even	ok
	410	C61H131N4O25P	675.4415	-0.7	41.6	423	37.07	0.0	even	ok
	411	C63H129N10NaO13P2S	675.4412	-1.1	41.6	424	32.10	6.0	even	ok
	412	C58H7N7NaO31P	675.4405	-2.0	41.7	425	22.37	60.0	even	ok
	413	C50H107N38O3PS	675.4418	-0.2	41.7	426	42.91	18.0	even	ok
	414	C54H107N34NaO4S	675.4411	-1.2	41.8	428	30.92	19.0	even	ok
	415	C61H124N16O11P2S	675.4410	-1.3	41.8	429	29.66	10.0	even	ok
	416	C50H124N22O14P2S	675.4426	1.1	41.9	430	32.01	2.0	even	ok
	417	C50H127N16O21PS	675.4405	-2.1	41.9	431	21.01	-3.0	even	ok
	418	C67H11N3O25P2S	675.4433	2.1	41.9	432	25.25	66.0	even	ok
	419	C64H136O24P2	675.4443	3.5	42.0	433	10.25	-1.0	even	ok
	420	C66H8N7NaO21P2S	675.4428	1.3	42.0	434	34.75	68.0	even	ok

Compound Spectrum SmartFormula Report

Meas. m/z	#	Ion Formula	m/z	err [ppm]	mSigma	# mSigma	Score	rdb	e¯ Conf	N-Rule
	421	C47H141N8NaO24P2S2	675.4431	1.8	42.1	435	28.99	-17.0	even	ok
	422	C60H3N13O24P2	675.4439	3.0	42.1	436	13.92	68.0	even	ok
	423	C61H127N6NaO24	675.4394	-3.7	42.1	437	9.61	2.0	even	ok
	424	C65H2N7NaO28	675.4417	-0.2	42.2	438	41.21	69.0	even	ok
	425	C50H103N40NaO2S	675.4398	-3.2	42.2	439	12.59	20.0	even	ok
	426	C45H10N15O31PS2	675.4418	-0.2	42.5	440	49.84	50.0	even	ok
	427	C58H11N5O32P2	675.4426	1.0	42.5	441	32.24	58.0	even	ok
	428	C49H24NNaO36P2S2	675.4441	3.2	42.5	442	14.54	40.0	even	ok
	429	C63H126N6O24	675.4406	-1.9	42.6	443	23.00	5.0	even	ok
	430	C62H128N12NaO12PS2	675.4420	0.2	42.6	444	49.70	6.0	even	ok
	431	C57H10N3NaO37	675.4440	3.1	42.7	445	12.81	55.0	even	ok
	432	C62H123N14O14PS	675.4395	-3.5	42.7	446	10.32	10.0	even	ok
	433	C62H122N14O16S	675.4436	2.6	42.8	447	16.53	10.0	even	ok
	434	C41H115N36O9PS2	675.4408	-1.6	42.8	448	25.29	4.0	even	ok
	435	C64H11N5NaO24PS2	675.4430	1.6	42.8	449	30.47	63.0	even	ok
	436	C58H114N26O7S2	675.4397	-3.2	42.8	450	14.36	16.0	even	ok
	437	C59H131N4NaO27	675.4444	3.7	42.8	451	9.35	-3.0	even	ok
	438	C49H123N20NaO19S	675.4440	3.2	42.8	452	12.29	-1.0	even	ok
	439	C65H15NO26P2S2	675.4394	-3.7	42.9	453	11.06	61.0	even	ok
	440	C43H124N28O12P2S2	675.4430	1.6	42.9	454	25.76	-2.0	even	ok
	441	C64H128N8NaO16PS	675.4397	-3.3	42.9	455	11.51	6.0	even	ok
	442	C50H117N30NaO6P2S	675.4428	1.3	43.0	456	28.89	9.0	even	ok
	443	C52H102N40O2S	675.4410	-1.4	43.0	457	27.67	23.0	even	ok
	444	C64H3N9NaO25P	675.4432	2.0	43.0	458	21.91	69.0	even	ok
	445	C43H127N22O19PS2	675.4408	-1.6	43.0	459	30.25	-7.0	even	ok
	446	C51H113N34NaO2P2S	675.4434	2.3	43.0	460	19.23	14.0	even	ok
	447	C62H137NaO24P2	675.4431	1.8	43.0	461	23.76	-4.0	even	ok
	448	C64H132N2NaO23P	675.4423	0.5	43.1	462	36.81	1.0	even	ok
	449	C64H127N8NaO18S	675.4438	2.8	43.1	463	14.78	6.0	even	ok
	450	C48H115N30O11PS	675.4404	-2.1	43.2	464	20.19	8.0	even	ok
	451	C40H111N40NaO7S2	675.4444	3.7	43.3	465	9.18	6.0	even	ok
	452	C53H108N36NaOPS	675.4426	1.0	43.4	466	31.01	19.0	even	ok
	453	C44H7N19NaO27PS2	675.4412	-1.0	43.5	467	37.78	52.0	even	ok
	454	C52H139N2O31PS	675.4405	-2.1	43.6	468	20.12	-14.0	even	ok
	455	C63H131N8O16PS2	675.4426	1.0	43.6	469	37.73	4.0	even	ok
	456	C58H115N26O7PS	675.4445	3.8	43.6	470	8.39	16.0	even	ok
	457	C56H112N32NaPS2	675.4407	-1.8	43.6	471	23.01	18.0	even	ok
	458	C62H2N13NaO21S2	675.4408	-1.6	43.7	472	30.02	69.0	even	ok
	459	C65H2N11O22PS	675.4412	-1.1	43.7	473	36.29	72.0	even	ok
	460	C47H19N7O34P2S2	675.4439	3.0	43.7	474	15.68	44.0	even	ok
	461	C60H121N20NaO7P2S	675.4405	-2.1	43.7	475	20.42	12.0	even	ok
	462	C50H8N15NaO26P2S	675.4424	0.7	43.9	476	34.08	56.0	even	ok
	463	C48H143NaO34S2	675.4394	-3.6	44.0	477	11.15	-22.0	even	ok
	464	C61H119N18NaO12S	675.4431	1.8	44.0	478	22.73	12.0	even	ok
	465	C59H122N12O22	675.4393	-3.9	44.0	479	8.05	6.0	even	ok
	466	C60H119N20NaO9S2	675.4399	-3.0	44.1	480	15.48	12.0	even	ok

Compound Spectrum SmartFormula Report

Meas. m/z	#	Ion Formula	m/z	err [ppm]	mSigma	# mSigma	Score	rdb	e⁻ Conf	N-Rule
	467	C59H114N24O10S	675.4430	1.6	44.1	481	24.68	16.0	even	ok
	468	C56H2N13O29P	675.4404	-2.2	44.1	482	19.09	64.0	even	ok
	469	C51H135N6NaO29S	675.4440	3.2	44.1	483	11.81	-12.0	even	ok
	470	C48H98N46S	675.4396	-3.4	44.1	484	10.67	24.0	even	ok
	471	C58H116N26O5P2S	675.4403	-2.3	44.3	485	18.33	16.0	even	ok
	472	C51H133N12NaO20P2S	675.4421	0.3	44.4	486	38.31	-7.0	even	ok
	473	C47H111N34NaO9S	675.4440	3.2	44.5	487	11.77	10.0	even	ok
	474	C46H16N11NaO30P2S2	675.4434	2.2	44.5	488	22.45	46.0	even	ok
	475	C65H14NO28PS2	675.4435	2.4	44.6	489	20.59	61.0	even	ok
	476	C62H127N8O21P	675.4421	0.3	44.6	490	37.50	5.0	even	ok
	477	C67H7N5NaO24PS	675.4413	-0.9	44.6	491	37.96	68.0	even	ok
	478	C58H4N13NaO24P2	675.4427	1.2	44.7	492	28.20	65.0	even	ok
	479	C50H10N9NaO35S	675.4443	3.6	44.8	493	9.11	51.0	even	ok
	480	C61H122N16O13S2	675.4404	-2.2	44.8	494	22.20	10.0	even	ok
	481	C48H108N38NaO3PS	675.4406	-1.9	44.9	495	20.97	15.0	even	ok
	482	C58H135NaO31	675.4437	2.7	44.9	496	14.96	-8.0	even	ok
	483	C46H102N44O3S	675.4446	4.0	44.9	497	7.43	19.0	even	ok
	484	C68H10NO28PS	675.4418	-0.1	44.9	498	47.92	66.0	even	ok
	485	C42H2N25O25PS2	675.4411	-1.2	45.0	499	33.62	56.0	even	ok
	486	C57H11N3NaO35P	675.4399	-3.0	45.0	500	12.73	55.0	even	ok
	487	C49H108N40P2S	675.4433	2.1	45.1	501	19.86	18.0	even	ok
	488	C59H132N4NaO25P	675.4402	-2.4	45.1	502	16.74	-3.0	even	ok
	489	C55H5N9O35	675.4439	2.9	45.1	503	13.30	59.0	even	ok
	490	C49H124N20NaO17PS	675.4399	-2.9	45.1	504	13.15	-1.0	even	ok
	491	C60H132N6O22P2	675.4429	1.6	45.1	505	24.30	0.0	even	ok
	492	C57H126N10O25	675.4442	3.5	45.1	506	9.85	1.0	even	ok
	493	C65H136N2NaO18PS2	675.4427	1.2	45.2	507	33.48	0.0	even	ok
	494	C46H138N6O32S2	675.4393	-3.8	45.3	508	9.51	-18.0	even	ok
	495	C47H118N26O17S	675.4439	3.0	45.3	509	12.78	3.0	even	ok
	496	C48H112N36O4P2S	675.4426	1.1	45.3	510	29.10	13.0	even	ok
	497	C63H5N9O25S2	675.4414	-0.8	45.3	511	38.16	67.0	even	ok
	498	C42H121N32NaO8P2S2	675.4424	0.8	45.3	512	31.94	0.0	even	ok
	499	C60H128N8NaO21P	675.4409	-1.4	45.4	513	25.29	2.0	even	ok
	500	C40H112N40NaO5PS2	675.4403	-2.4	45.4	514	16.66	6.0	even	ok
697.4550	1	C57H139N6NaO25S2	697.4559	1.2	7.4	1	72.25	-8.0	even	ok
	2	C58H142N2O29S2	697.4564	2.0	7.4	2	52.61	-10.0	even	ok
	3	C57H15N11O26P2S2	697.4548	-0.4	7.6	3	95.44	58.0	even	ok
	4	C55H134N12O23S2	697.4558	1.0	8.2	4	76.62	-4.0	even	ok
	5	C56H14N9NaO31S2	697.4562	1.6	8.3	5	60.25	55.0	even	ok
	6	C59H20N5NaO28P2S2	697.4549	-0.2	8.4	6	100.00	54.0	even	ok
	7	C56H12N15NaO22P2S2	697.4542	-1.2	8.4	7	72.43	60.0	even	ok
	8	C54H9N15O29S2	697.4561	1.4	8.5	8	65.04	59.0	even	ok
	9	C57H144N4O24P2S2	697.4538	-1.8	8.9	9	56.20	-10.0	even	ok
	10	C55H5N19O25S2	697.4567	2.4	9.0	10	42.25	64.0	even	ok
	11	C57H11N13NaO25PS2	697.4527	-3.3	9.1	11	25.98	60.0	even	ok
	12	C54H7N21O20P2S2	697.4541	-1.4	9.4	12	65.90	64.0	even	ok

Compound Spectrum SmartFormula Report

Meas. m/z	#	Ion Formula	m/z	err [ppm]	mSigma	# mSigma	Score	rdb	e⁻ Conf	N-Rule
	13	C54H2N23NaO21S2	697.4562	1.6	9.5	13	59.09	66.0	even	ok
	14	C53H6N19NaO25S2	697.4555	0.7	9.5	14	84.53	61.0	even	ok
	15	C58H24NNaO32P2S2	697.4542	-1.2	9.7	15	70.99	49.0	even	ok
	16	C57H17N5O35S2	697.4567	2.4	9.8	16	41.45	53.0	even	ok
	17	C57H10N13NaO27S2	697.4569	2.6	10.0	17	37.55	60.0	even	ok
	18	C58H14N9O29PS2	697.4533	-2.5	10.0	18	38.52	58.0	even	ok
	19	C60H23NO32P2S2	697.4554	0.6	10.2	19	86.45	52.0	even	ok
	20	C56H19N7O30P2S2	697.4541	-1.4	10.2	20	65.07	53.0	even	ok
	21	C58H140N8O20P2S2	697.4545	-0.8	10.6	21	78.94	-5.0	even	ok
	22	C54H131N16NaO19S2	697.4552	0.3	10.6	22	94.42	-2.0	even	ok
	23	C60H145N2NaO22P2S2	697.4546	-0.6	10.7	23	84.17	-9.0	even	ok
	24	C55H16N11NaO26P2S2	697.4536	-2.1	11.1	25	46.17	55.0	even	ok
	25	C56H141N8NaO20P2S2	697.4533	-2.5	11.3	26	37.71	-8.0	even	ok
	26	C54H135N14O20PS2	697.4573	3.2	11.3	27	27.01	-4.0	even	ok
	27	C60H19N3NaO31PS2	697.4534	-2.3	11.7	28	41.16	54.0	even	ok
	28	C53H4N25NaO16P2S2	697.4536	-2.1	12.1	29	45.19	66.0	even	ok
	29	C59H139N6O23PS2	697.4530	-3.0	12.2	30	29.79	-5.0	even	ok
	30	C56H143N2NaO29S2	697.4552	0.3	12.2	31	91.32	-13.0	even	ok
	31	C57H137N12NaO16P2S2	697.4539	-1.6	12.3	32	57.20	-3.0	even	ok
	32	C56H130N16O19S2	697.4564	2.0	12.4	33	48.12	1.0	even	ok
	33	C52H126N22O17S2	697.4551	0.1	12.5	34	96.62	2.0	even	ok
	34	C58H135N10NaO21S2	697.4566	2.2	12.8	35	43.56	-3.0	even	ok
	35	C53H11N17O24P2S2	697.4534	-2.3	12.8	36	40.69	59.0	even	ok
	36	C59H138N6O25S2	697.4571	2.9	13.1	37	29.45	-5.0	even	ok
	37	C52H7N21NaO22PS2	697.4570	2.8	13.2	38	31.51	61.0	even	ok
	38	C54H136N14O18P2S2	697.4531	-2.7	13.2	39	32.77	-4.0	even	ok
	39	C61H144NaO25PS2	697.4531	-2.8	13.3	40	32.43	-9.0	even	ok
	40	C55H132N18O14P2S2	697.4538	-1.8	13.3	41	51.55	1.0	even	ok
	41	C54H138N8O27S2	697.4551	0.1	13.6	42	94.22	-9.0	even	ok
	42	C55H127N20NaO15S2	697.4559	1.2	13.8	43	64.22	3.0	even	ok
	43	C60H13N5O35S	697.4550	-0.0	14.1	44	94.70	58.0	even	ok
	44	C53H132N18NaO16PS2	697.4567	2.4	14.3	45	38.12	-2.0	even	ok
	45	C53H122N26O13S2	697.4558	1.0	14.5	46	68.22	7.0	even	ok
	46	C61H143NaO27S2	697.4572	3.1	14.6	47	25.63	-9.0	even	ok
	47	C56H147O30PS2	697.4573	3.2	14.6	48	25.25	-15.0	even	ok
	48	C55H3N25O16P2S2	697.4548	-0.4	14.7	49	83.31	69.0	even	ok
	49	C58H11N15O22P2S2	697.4554	0.6	14.8	50	79.31	63.0	even	ok
	50	C57H8N19NaO18P2S2	697.4549	-0.2	14.9	51	88.13	65.0	even	ok
	51	C52H8N21NaO20P2S2	697.4529	-3.1	15.1	52	26.19	61.0	even	ok
	52	C50H2N27O20PS2	697.4569	2.6	15.2	53	33.60	65.0	even	ok
	53	C53H135N12NaO23S2	697.4546	-0.7	15.4	54	74.76	-7.0	even	ok
	54	C61H19NNaO34PS	697.4567	2.3	15.5	55	38.45	54.0	even	ok
	55	C55H18N5NaO35S2	697.4555	0.7	15.6	56	75.01	50.0	even	ok
	56	C51H123N26NaO13S2	697.4546	-0.7	15.6	57	74.19	4.0	even	ok
	57	C55H144N4NaO26PS2	697.4567	2.4	15.8	58	36.84	-13.0	even	ok
	58	C56H148O28P2S2	697.4531	-2.7	15.8	59	31.26	-15.0	even	ok

Compound Spectrum SmartFormula Report

Meas. m/z	#	Ion Formula	m/z	err [ppm]	mSigma	# mSigma	Score	rdb	e⁻ Conf	N-Rule
	59	C60H16N9NaO24P2S2	697.4556	0.8	16.0	60	72.44	59.0	even	ok
	60	C52H123N28O10PS2	697.4573	3.2	16.2	61	24.66	7.0	even	ok
	61	C56H21NO39S2	697.4561	1.4	16.2	62	55.94	48.0	even	ok
	62	C54H129N22NaO10P2S2	697.4533	-2.5	16.2	63	34.09	3.0	even	ok
	63	C56H2N23O19PS2	697.4533	-2.6	16.4	65	33.90	69.0	even	ok
	64	C53H13N11O33S2	697.4554	0.5	16.4	66	78.69	54.0	even	ok
	65	C51H127N24O14PS2	697.4566	2.2	16.4	67	40.18	2.0	even	ok
	66	C59H17NO39S	697.4544	-1.0	16.4	68	66.69	53.0	even	ok
	67	C63H19NO32P2S	697.4538	-1.9	16.7	69	46.78	57.0	even	ok
	68	C52H10N15NaO29S2	697.4548	-0.3	17.1	70	82.41	56.0	even	ok
	69	C52H119N30NaO9S2	697.4552	0.2	17.1	71	83.25	9.0	even	ok
	70	C50H3N27O18P2S2	697.4528	-3.3	17.2	72	22.41	65.0	even	ok
	71	C61H19N5O28P2S2	697.4561	1.5	17.3	73	53.07	57.0	even	ok
	72	C58H7N17NaO21PS2	697.4534	-2.4	17.3	74	36.70	65.0	even	ok
	73	C51H130N18O21S2	697.4544	-0.9	17.4	75	67.05	-3.0	even	ok
	74	C59H10N9NaO31S	697.4545	-0.8	17.4	76	69.99	60.0	even	ok
	75	C59H14N7O32PS	697.4565	2.1	17.5	77	40.56	58.0	even	ok
	76	C53H139N10O24PS2	697.4566	2.2	17.6	78	39.11	-9.0	even	ok
	77	C63H14N3NaO33S	697.4558	1.1	17.7	79	60.92	59.0	even	ok
	78	C49H118N32O11S2	697.4544	-0.9	17.8	80	66.29	8.0	even	ok
	79	C52H124N28O8P2S2	697.4531	-2.7	17.8	81	29.76	7.0	even	ok
	80	C59H10N13O25PS2	697.4539	-1.6	18.1	82	50.83	63.0	even	ok
	81	C62H15N5NaO30PS	697.4573	3.3	18.2	83	21.95	59.0	even	ok
	82	C61H9N9O31S	697.4557	0.9	18.2	84	64.80	63.0	even	ok
	83	C54H19N7NaO32PS2	697.4570	2.8	18.3	85	28.33	50.0	even	ok
	84	C61H138N2O29S	697.4548	-0.4	18.4	86	76.76	-5.0	even	ok
	85	C50H114N36O7S2	697.4551	0.0	18.5	87	85.74	13.0	even	ok
	86	C50H5N21O27S2	697.4547	-0.5	18.7	88	75.00	60.0	even	ok
	87	C59H136N12O16P2S2	697.4551	0.1	18.7	89	83.12	0.0	even	ok
	88	C55H23N3O34P2S2	697.4534	-2.3	18.8	90	36.19	48.0	even	ok
	89	C57H5N15O29S	697.4544	-1.0	19.3	91	62.64	64.0	even	ok
	90	C60H10N11O28PS	697.4572	3.1	19.4	92	23.98	63.0	even	ok
	91	C51H120N32NaO6PS2	697.4567	2.4	19.4	93	34.45	9.0	even	ok
	92	C62H16N5NaO28P2S	697.4532	-2.6	19.4	94	30.83	59.0	even	ok
	93	C58H14N5NaO35S	697.4538	-1.7	19.4	95	46.46	55.0	even	ok
	94	C54H20N7NaO30P2S2	697.4529	-3.1	19.5	96	24.04	50.0	even	ok
	95	C61H141N6NaO18P2S2	697.4553	0.3	19.5	97	77.09	-4.0	even	ok
	96	C58H133N16NaO12P2S2	697.4546	-0.6	19.5	98	70.40	2.0	even	ok
	97	C56H128N22O10P2S2	697.4545	-0.8	19.6	99	65.73	6.0	even	ok
	98	C52H14N13O30PS2	697.4569	2.6	19.6	100	30.56	54.0	even	ok
	99	C52H136N14NaO20PS2	697.4561	1.4	19.7	101	52.09	-7.0	even	ok
	100	C50H124N28NaO10PS2	697.4560	1.4	19.7	102	52.24	4.0	even	ok
	101	C61H15N7NaO27PS2	697.4541	-1.4	19.8	103	53.22	59.0	even	ok
	102	C62H144N2O22P2S2	697.4558	1.1	19.8	104	59.07	-6.0	even	ok
	103	C50H127N22NaO17S2	697.4539	-1.7	19.9	105	47.33	-1.0	even	ok
	104	C49H2N25NaO23S2	697.4542	-1.3	20.4	106	55.36	62.0	even	ok

Compound Spectrum SmartFormula Report

Meas. m/z	#	Ion Formula	m/z	err [ppm]	mSigma	# mSigma	Score	rdb	e⁻ Conf	N-Rule
	105	C60H6N13NaO27S	697.4552	0.2	20.7	107	78.83	65.0	even	ok
	106	C57H127N20O13PS2	697.4530	-3.0	20.8	108	24.91	6.0	even	ok
	107	C60H11N11O26P2S	697.4531	-2.8	20.8	109	26.98	63.0	even	ok
	108	C58H18N3O36PS	697.4559	1.2	20.9	110	56.33	53.0	even	ok
	109	C51H11N17NaO26PS2	697.4563	1.9	20.9	111	42.67	56.0	even	ok
	110	C58H11N11NaO28PS	697.4560	1.4	20.9	112	52.28	60.0	even	ok
	111	C52H15N13O28P2S2	697.4528	-3.3	21.0	113	20.79	54.0	even	ok
	112	C57H126N20O15S2	697.4571	2.9	21.1	114	25.08	6.0	even	ok
	113	C60H139N4O26PS	697.4562	1.7	21.3	115	45.01	-5.0	even	ok
	114	C59H132N14NaO15PS2	697.4531	-2.8	21.3	116	27.39	2.0	even	ok
	115	C60H135N10O19PS2	697.4537	-2.0	21.3	117	39.70	0.0	even	ok
	116	C55H125N26NaO6P2S2	697.4539	-1.6	21.5	118	47.23	8.0	even	ok
	117	C62H18N3O31PS2	697.4546	-0.6	21.6	119	67.45	57.0	even	ok
	118	C59H139N2NaO29S	697.4535	-2.2	21.6	120	36.85	-8.0	even	ok
	119	C60H135N6NaO25S	697.4542	-1.2	21.7	121	55.04	-3.0	even	ok
	120	C54H118N30O9S2	697.4564	2.0	21.7	122	39.88	12.0	even	ok
	121	C56H9N11O33S	697.4537	-1.9	21.7	123	40.56	59.0	even	ok
	122	C56H123N24NaO11S2	697.4566	2.2	21.8	124	36.31	8.0	even	ok
	123	C50H131N20O18PS2	697.4559	1.2	21.8	126	53.83	-3.0	even	ok
	124	C59H131N14NaO17S2	697.4572	3.1	22.0	127	22.07	2.0	even	ok
	125	C48H122N28O15S2	697.4537	-1.9	22.1	128	41.41	3.0	even	ok
	126	C53H120N32O4P2S2	697.4538	-1.8	22.2	129	42.71	12.0	even	ok
	127	C59H7N15NaO24PS	697.4567	2.3	22.4	130	33.37	65.0	even	ok
	128	C53H142N4O31S2	697.4544	-0.9	22.5	131	60.29	-14.0	even	ok
	129	C59H143O30PS	697.4556	0.8	22.5	132	63.14	-10.0	even	ok
	130	C62H140N4NaO21PS2	697.4538	-1.8	22.6	133	42.21	-4.0	even	ok
	131	C49H6N23O24PS2	697.4562	1.7	22.7	134	44.69	60.0	even	ok
	132	C64H139NaO27S	697.4556	0.7	22.8	135	63.24	-4.0	even	ok
	133	C56H6N17O26PS	697.4559	1.2	23.1	136	53.84	64.0	even	ok
	134	C49H115N38O4PS2	697.4566	2.2	23.2	137	29.11	13.0	even	ok
	135	C48H115N36NaO7S2	697.4539	-1.7	23.2	138	36.60	10.0	even	ok
	136	C53H115N34NaO5S2	697.4559	1.2	23.4	139	52.70	14.0	even	ok
	137	C63H140N2NaO24PS	697.4571	2.9	23.4	140	24.71	-4.0	even	ok
	138	C62H134N6O25S	697.4554	0.5	23.4	141	66.63	0.0	even	ok
	139	C63H143O25PS2	697.4543	-1.0	23.6	142	55.90	-6.0	even	ok
	140	C52H139N8NaO27S2	697.4539	-1.7	23.7	143	43.74	-12.0	even	ok
	141	C49H111N40NaO3S2	697.4545	-0.7	23.7	144	51.94	15.0	even	ok
	142	C64H15N5O28P2S	697.4544	-0.9	23.7	145	58.64	62.0	even	ok
	143	C57H134N8O27S	697.4534	-2.4	23.9	146	31.88	-4.0	even	ok
	144	C51H110N40O3S2	697.4558	1.0	23.9	147	56.06	18.0	even	ok
	145	C57H15N7NaO32PS	697.4553	0.4	24.0	148	68.45	55.0	even	ok
	146	C48H119N34O8PS2	697.4559	1.2	24.0	149	42.82	8.0	even	ok
	147	C58H4N23NaO14P2S2	697.4556	0.8	24.2	150	60.96	70.0	even	ok
	148	C59H7N19O18P2S2	697.4561	1.5	24.3	151	45.74	68.0	even	ok
	149	C49H128N24NaO14PS2	697.4554	0.5	24.5	152	66.21	-1.0	even	ok
	150	C54H148NaO30PS2	697.4561	1.5	24.6	153	46.63	-18.0	even	ok

Compound Spectrum SmartFormula Report

Meas. m/z	#	Ion Formula	m/z	err [ppm]	mSigma	# mSigma	Score	rdb	e⁻ Conf	N-Rule
	151	C48H3N27NaO20PS2	697.4557	0.9	24.7	154	57.31	62.0	even	ok
	152	C54H22NNaO39S2	697.4549	-0.3	24.8	155	69.85	45.0	even	ok
	153	C65H14N3O31PS	697.4529	-3.0	24.9	156	21.90	62.0	even	ok
	154	C56H2N19NaO25S	697.4538	-1.7	24.9	157	34.19	66.0	even	ok
	155	C55H6N15NaO29S	697.4532	-2.7	24.9	158	26.13	61.0	even	ok
	156	C47H119N32NaO11S2	697.4532	-2.6	25.0	159	26.99	5.0	even	ok
	157	C65H13N3O33S	697.4571	2.9	25.2	160	23.75	62.0	even	ok
	158	C50H134N14O25S2	697.4538	-1.9	25.4	161	38.59	-8.0	even	ok
	159	C57H18NNaO39S	697.4532	-2.7	25.5	162	25.90	50.0	even	ok
	160	C57H19N5O33P2S	697.4574	3.3	25.5	163	18.42	53.0	even	ok
	161	C61H12N13NaO20P2S2	697.4562	1.7	25.5	164	40.90	64.0	even	ok
	162	C63H12N9NaO24P2S	697.4539	-1.7	25.6	165	41.93	64.0	even	ok
	163	C47H106N46OS2	697.4544	-0.9	25.6	166	46.44	19.0	even	ok
	164	C46H110N42O5S2	697.4537	-1.9	25.6	167	31.78	14.0	even	ok
	165	C58H130N12O23S	697.4541	-1.4	25.6	168	38.83	1.0	even	ok
	166	C52H17N7O37S2	697.4547	-0.5	25.8	169	64.24	49.0	even	ok
	167	C62H5N13O27S	697.4564	1.9	25.9	170	37.40	68.0	even	ok
	168	C64H10N7NaO29S	697.4565	2.1	26.0	171	34.10	64.0	even	ok
	169	C61H7N15O22P2S	697.4538	-1.9	26.2	172	37.94	68.0	even	ok
	170	C52H143N6O28PS2	697.4559	1.2	26.2	173	48.64	-14.0	even	ok
	171	C57H2N21O22PS	697.4565	2.1	26.2	174	28.03	69.0	even	ok
	172	C63H17NO34S2	697.4531	-2.8	26.2	175	24.56	57.0	even	ok
	173	C55H10N13O30PS	697.4552	0.2	26.3	176	69.00	59.0	even	ok
	174	C51H14N11NaO33S2	697.4542	-1.2	26.3	177	48.60	51.0	even	ok
	175	C62H15N9O24P2S2	697.4568	2.5	26.6	178	28.11	62.0	even	ok
	176	C47H123N30O12PS2	697.4552	0.3	26.7	179	66.88	3.0	even	ok
	177	C61H135N8O22PS	697.4569	2.7	26.8	180	21.16	0.0	even	ok
	178	C63H141N2NaO22P2S	697.4529	-3.0	26.8	181	17.47	-4.0	even	ok
	179	C48H112N42NaPS2	697.4560	1.4	26.9	182	37.18	15.0	even	ok
	180	C59H3N21NaO17PS2	697.4541	-1.4	27.1	183	45.10	70.0	even	ok
	181	C59H136N8NaO22PS	697.4557	1.0	27.1	184	44.35	-3.0	even	ok
	182	C50H111N42PS2	697.4572	3.2	27.1	185	16.26	18.0	even	ok
	183	C52H117N36NaP2S2	697.4533	-2.6	27.2	186	22.27	14.0	even	ok
	184	C49H131N18NaO21S2	697.4532	-2.6	27.3	187	25.74	-6.0	even	ok
	185	C61H2N17NaO23S	697.4558	1.1	27.5	188	49.14	70.0	even	ok
	186	C55H13N7O37S	697.4530	-2.9	27.6	189	22.18	54.0	even	ok
	187	C49H9N17O31S2	697.4540	-1.4	27.7	190	43.48	55.0	even	ok
	188	C53H23N3NaO36PS2	697.4563	1.9	27.7	191	36.45	45.0	even	ok
	189	C51H140N10NaO24PS2	697.4554	0.5	27.8	192	61.26	-12.0	even	ok
	190	C60H6N17O21PS2	697.4546	-0.6	27.8	193	58.49	68.0	even	ok
	191	C47H116N38NaO4PS2	697.4554	0.5	27.8	194	51.23	10.0	even	ok
	192	C56H138N4O31S	697.4527	-3.3	28.0	195	17.45	-9.0	even	ok
	193	C61H136N8O20P2S	697.4528	-3.2	28.2	196	15.12	0.0	even	ok
	194	C57H124N26O6P2S2	697.4551	0.1	28.3	197	67.26	11.0	even	ok
	195	C64H20N3NaO26P2S2	697.4569	2.7	28.3	198	24.40	58.0	even	ok
	196	C60H132N16O12P2S2	697.4558	1.1	28.3	199	48.96	5.0	even	ok

Compound Spectrum SmartFormula Report

Meas. m/z	#	Ion Formula	m/z	err [ppm]	mSigma	# mSigma	Score	rdb	e⁻ Conf	N-Rule
	197	C58H140N4NaO26PS	697.4550	-0.0	28.3	200	58.04	-8.0	even	ok
	198	C61H131N10NaO21S	697.4549	-0.2	28.4	201	54.32	2.0	even	ok
	199	C56H16N9NaO29P2S	697.4568	2.6	28.6	202	25.89	55.0	even	ok
	200	C59H129N20NaO8P2S2	697.4553	0.3	28.7	203	62.77	7.0	even	ok
	201	C65H140N2O22P2S	697.4541	-1.3	29.0	204	44.53	-1.0	even	ok
	202	C48H6N21NaO27S2	697.4535	-2.2	29.0	205	30.28	57.0	even	ok
	203	C51H18N9O34PS2	697.4562	1.7	29.0	206	38.54	49.0	even	ok
	204	C55H3N21NaO22PS	697.4553	0.4	29.1	207	50.71	66.0	even	ok
	205	C62H137N10NaO14P2S2	697.4560	1.3	29.2	208	44.38	1.0	even	ok
	206	C47H4N29NaO17P2S2	697.4572	3.0	29.2	209	19.77	62.0	even	ok
	207	C47H126N24O19S2	697.4531	-2.8	29.3	210	22.12	-2.0	even	ok
	208	C57H131N14O20PS	697.4556	0.8	29.3	211	45.12	1.0	even	ok
	209	C62H11N11NaO23PS2	697.4548	-0.4	29.3	212	60.08	64.0	even	ok
	210	C57H127N16NaO19S	697.4535	-2.2	29.4	213	25.57	3.0	even	ok
	211	C56H121N30NaO2P2S2	697.4546	-0.6	29.5	214	56.07	13.0	even	ok
	212	C45H107N46NaOS2	697.4532	-2.6	29.5	215	20.19	16.0	even	ok
	213	C58H144N2O27P2S	697.4571	2.9	29.5	216	17.62	-10.0	even	ok
	214	C59H126N16O19S	697.4547	-0.4	29.6	217	49.66	6.0	even	ok
	215	C49H135N16O22PS2	697.4552	0.3	29.6	218	62.32	-8.0	even	ok
	216	C63H140N6O18P2S2	697.4565	2.1	29.7	219	31.82	-1.0	even	ok
	217	C55H7N19O23P2S	697.4574	3.3	29.8	220	13.95	64.0	even	ok
	218	C45H114N38O9S2	697.4531	-2.8	29.9	222	18.08	9.0	even	ok
	219	C56H131N12NaO23S	697.4529	-3.1	29.9	223	15.50	-2.0	even	ok
	220	C66H138O27S	697.4568	2.5	29.9	224	26.30	-1.0	even	ok
	221	C50H15N13NaO30PS2	697.4557	0.9	30.0	225	50.57	51.0	even	ok
	222	C60H132N12NaO18PS	697.4564	1.9	30.0	226	28.23	2.0	even	ok
	223	C56H19N3NaO36PS	697.4547	-0.6	30.1	227	56.73	50.0	even	ok
	224	C58H123N24O9PS2	697.4536	-2.0	30.2	228	32.21	11.0	even	ok
	225	C60H3N19NaO20PS	697.4573	3.3	30.3	229	13.98	70.0	even	ok
	226	C45H111N44O2PS2	697.4552	0.3	30.3	230	51.28	14.0	even	ok
	227	C57H144NaO30PS	697.4544	-1.0	30.4	231	48.87	-13.0	even	ok
	228	C55H115N34O3PS2	697.4530	-3.0	30.6	232	19.80	17.0	even	ok
	229	C55H114N34O5S2	697.4571	2.9	30.7	233	20.20	17.0	even	ok
	230	C63H14N7O27PS2	697.4553	0.3	30.8	235	59.60	62.0	even	ok
	231	C57H120N28NaO5PS2	697.4531	-2.8	30.8	236	21.88	13.0	even	ok
	232	C56H135N10O24PS	697.4549	-0.2	30.8	237	51.55	-4.0	even	ok
	233	C58H137N10NaO19P2S	697.4572	3.1	31.0	238	15.29	-3.0	even	ok
	234	C65H135N4NaO23S	697.4562	1.7	31.0	239	36.31	1.0	even	ok
	235	C60H128N18NaO11PS2	697.4538	-1.8	31.0	240	34.51	7.0	even	ok
	236	C65H145NaO20P2S2	697.4566	2.3	31.1	241	28.01	-5.0	even	ok
	237	C63H130N10O21S	697.4561	1.5	31.1	242	39.38	5.0	even	ok
	238	C57H119N28NaO7S2	697.4572	3.1	31.2	243	17.88	13.0	even	ok
	239	C61H131N14O15PS2	697.4543	-1.0	31.2	244	46.57	5.0	even	ok
	240	C60H4N19NaO18P2S	697.4532	-2.6	31.3	245	19.46	70.0	even	ok
	241	C48H10N19O28PS2	697.4555	0.7	31.5	246	52.09	55.0	even	ok
	242	C55H122N22O17S	697.4534	-2.4	31.6	247	22.07	7.0	even	ok

Compound Spectrum SmartFormula Report

Meas. m/z	#	Ion Formula	m/z	err [ppm]	mSigma	# mSigma	Score	rdb	e¯ Conf	N-Rule
	243	C58H127N18O16PS	697.4562	1.7	31.6	248	29.60	6.0	even	ok
	244	C48H132N20NaO18PS2	697.4547	-0.5	31.7	249	55.70	-6.0	even	ok
	245	C61H5N15O24S2	697.4531	-2.8	31.7	250	21.45	68.0	even	ok
	246	C54H111N38NaOS2	697.4566	2.2	31.7	251	28.89	19.0	even	ok
	247	C48H129N26NaO11P2S2	697.4569	2.6	31.9	252	19.24	-1.0	even	ok
	248	C52H146O35S2	697.4538	-1.9	31.9	253	33.13	-19.0	even	ok
	249	C50H141N12NaO21P2S2	697.4569	2.6	32.0	254	22.92	-12.0	even	ok
	250	C56H23NO37P2S	697.4567	2.4	32.2	255	26.09	48.0	even	ok
	251	C54H14N9O34PS	697.4545	-0.8	32.2	256	50.32	54.0	even	ok
	252	C54H7N17NaO26PS	697.4547	-0.6	32.3	257	44.68	61.0	even	ok
	253	C54H116N36P2S2	697.4545	-0.8	32.4	258	40.64	17.0	even	ok
	254	C54H126N18O21S	697.4527	-3.3	32.4	259	13.01	2.0	even	ok
	255	C46H120N34NaO8PS2	697.4547	-0.5	32.5	260	45.41	5.0	even	ok
	256	C63H136N8NaO17PS2	697.4545	-0.8	32.5	261	48.46	1.0	even	ok
	257	C65H11N9O24P2S	697.4551	0.1	32.5	262	61.83	67.0	even	ok
	258	C46H117N40NaOP2S2	697.4569	2.6	32.6	263	18.96	10.0	even	ok
	259	C65H19NNaO29PS2	697.4554	0.5	32.7	264	53.38	58.0	even	ok
	260	C58H123N20NaO15S	697.4542	-1.2	32.8	265	35.22	8.0	even	ok
	261	C51H143N4NaO31S2	697.4532	-2.6	32.8	266	22.57	-17.0	even	ok
	262	C67H16N3NaO26P2S	697.4552	0.3	32.9	267	57.79	63.0	even	ok
	263	C47H7N23NaO24PS2	697.4550	-0.1	33.1	268	60.96	57.0	even	ok
	264	C56H128N18NaO16PS	697.4550	-0.0	33.2	269	51.41	3.0	even	ok
	265	C57H141N6NaO23P2S	697.4565	2.1	33.2	270	23.56	-8.0	even	ok
	266	C56H132N16O17P2S	697.4571	2.9	33.4	271	16.07	1.0	even	ok
	267	C64H139N4O21PS2	697.4550	-0.1	33.4	272	60.29	-1.0	even	ok
	268	C63H10N9NaO26S2	697.4533	-2.6	33.5	273	22.74	64.0	even	ok
	269	C54H4N23NaO19P2S	697.4568	2.5	33.6	274	19.13	66.0	even	ok
	270	C64H8N13NaO20P2S	697.4546	-0.7	33.7	275	49.32	69.0	even	ok
	271	C46H127N26O16PS2	697.4546	-0.7	33.8	276	49.56	-2.0	even	ok
	272	C54H11N15O27P2S	697.4567	2.4	33.8	277	20.94	59.0	even	ok
	273	C49H16N15NaO27P2S2	697.4572	3.0	33.9	278	17.57	51.0	even	ok
	274	C64H137N6NaO18P2S	697.4536	-2.1	33.9	279	23.82	1.0	even	ok
	275	C48H136N18O19P2S2	697.4567	2.4	34.0	280	24.11	-8.0	even	ok
	276	C60H3N23O14P2S2	697.4568	2.5	34.3	282	23.36	73.0	even	ok
	277	C56H118N26O13S	697.4541	-1.4	34.3	283	31.33	12.0	even	ok
	278	C63H9N5O35	697.4534	-2.4	34.4	284	19.90	63.0	even	ok
	279	C46H124N32O9P2S2	697.4567	2.4	34.4	285	19.97	3.0	even	ok
	280	C49H138N10O29S2	697.4531	-2.8	34.4	286	19.56	-13.0	even	ok
	281	C66H10N7O27PS	697.4536	-2.1	34.4	287	28.16	67.0	even	ok
	282	C62H132N12O16P2S	697.4535	-2.3	34.6	288	21.31	5.0	even	ok
	283	C55H132N14NaO20PS	697.4544	-1.0	34.6	289	36.51	-2.0	even	ok
	284	C55H20N5NaO33P2S	697.4562	1.6	34.7	290	34.48	50.0	even	ok
	285	C62H130N12O18S2	697.4528	-3.2	34.7	291	15.87	5.0	even	ok
	286	C63H2N17O21PS	697.4529	-3.0	34.8	292	17.14	73.0	even	ok
	287	C52H2N23O24PS	697.4545	-0.8	34.8	293	39.19	65.0	even	ok
	288	C45H2N29O22PS2	697.4549	-0.3	34.9	295	54.87	61.0	even	ok

Compound Spectrum SmartFormula Report

Meas. m/z	#	Ion Formula	m/z	err [ppm]	mSigma	# mSigma	Score	rdb	e⁻ Conf	N-Rule
	289	C44H115N40O6PS2	697.4546	-0.7	35.0	296	39.89	9.0	even	ok
	290	C65H7N11NaO23PS	697.4531	-2.8	35.1	297	18.97	69.0	even	ok
	291	C68H15NNaO29PS	697.4537	-1.9	35.1	298	30.23	63.0	even	ok
	292	C57H124N22NaO12PS	697.4557	0.9	35.2	299	36.41	8.0	even	ok
	293	C64H13N5O30S2	697.4538	-1.8	35.3	300	31.09	62.0	even	ok
	294	C66H14NO34P	697.4562	1.6	35.3	301	27.70	62.0	even	ok
	295	C65H6N11NaO25S	697.4572	3.1	35.4	302	16.70	69.0	even	ok
	296	C51H147N2O32PS2	697.4553	0.3	35.5	303	53.71	-19.0	even	ok
	297	C54H119N26NaO13S	697.4529	-3.1	35.5	304	13.42	9.0	even	ok
	298	C51H21N3O41S2	697.4540	-1.4	35.5	305	36.00	44.0	even	ok
	299	C54H123N24O14PS	697.4549	-0.2	35.5	306	45.74	7.0	even	ok
	300	C47H11N21O25P2S2	697.4570	2.8	35.6	307	18.72	55.0	even	ok
	301	C62H8N17NaO16P2S2	697.4569	2.7	35.6	308	20.42	69.0	even	ok
	302	C64H15NNaO34P	697.4550	-0.1	35.7	309	47.26	59.0	even	ok
	303	C55H136N12O21P2S	697.4564	1.9	35.7	310	24.16	-4.0	even	ok
	304	C55H139N6O28PS	697.4542	-1.2	35.8	311	33.06	-9.0	even	ok
	305	C50H18N7NaO37S2	697.4535	-2.2	35.9	312	25.56	46.0	even	ok
	306	C62H127N14NaO17S	697.4556	0.7	36.0	313	38.46	7.0	even	ok
	307	C47H133N22NaO15P2S2	697.4562	1.7	36.2	314	32.23	-6.0	even	ok
	308	C64H135N6NaO20S2	697.4530	-3.0	36.2	315	17.04	1.0	even	ok
	309	C61H128N16NaO14PS	697.4570	2.9	36.2	316	15.14	7.0	even	ok
	310	C60H122N20O15S	697.4554	0.5	36.5	317	40.47	11.0	even	ok
	311	C50H144N6NaO28PS2	697.4547	-0.5	36.7	318	49.18	-17.0	even	ok
	312	C66H10N3NaO33	697.4542	-1.3	36.7	319	31.00	64.0	even	ok
	313	C55H119N28O10PS	697.4556	0.7	37.0	320	37.20	12.0	even	ok
	314	C62H3N19O18P2S	697.4544	-0.9	37.0	321	35.21	73.0	even	ok
	315	C53H127N20O18PS	697.4542	-1.2	37.2	322	31.75	2.0	even	ok
	316	C59H123N22O12PS	697.4569	2.7	37.2	323	16.39	11.0	even	ok
	317	C48H13N13O35S2	697.4534	-2.4	37.2	324	22.44	50.0	even	ok
	318	C61H129N16NaO12P2S	697.4529	-3.0	37.3	325	13.41	7.0	even	ok
	319	C53H8N19NaO23P2S	697.4562	1.6	37.3	326	26.91	61.0	even	ok
	320	C46H8N25NaO21P2S2	697.4565	2.1	37.3	327	26.09	57.0	even	ok
	321	C55H129N20NaO13P2S	697.4565	2.1	37.3	328	21.24	3.0	even	ok
	322	C65H138N2O24S2	697.4535	-2.2	37.5	329	24.29	-1.0	even	ok
	323	C66H136N6O18P2S	697.4548	-0.3	37.6	330	49.90	4.0	even	ok
	324	C45H121N36NaO5P2S2	697.4562	1.7	37.6	331	25.96	5.0	even	ok
	325	C52H114N32O11S	697.4527	-3.3	37.7	332	11.29	13.0	even	ok
	326	C68H141NaO20P2S	697.4549	-0.1	37.8	333	52.69	0.0	even	ok
	327	C58H120N30O2P2S2	697.4558	1.1	37.8	334	38.64	16.0	even	ok
	328	C61H2N21O17PS2	697.4553	0.3	37.8	335	49.85	73.0	even	ok
	329	C62H10N7O32P	697.4549	-0.3	37.9	336	42.10	63.0	even	ok
	330	C55H115N30NaO9S	697.4535	-2.2	37.9	337	20.55	14.0	even	ok
	331	C64H5N9O31	697.4540	-1.5	38.1	338	27.61	68.0	even	ok
	332	C56H125N24NaO9P2S	697.4572	3.1	38.2	339	12.77	8.0	even	ok
	333	C47H10N17NaO31S2	697.4528	-3.2	38.2	340	14.64	52.0	even	ok
	334	C62H6N9NaO31	697.4528	-3.2	38.2	341	12.03	65.0	even	ok

Compound Spectrum SmartFormula Report

Meas. m/z	#	Ion Formula	m/z	err [ppm]	mSigma	# mSigma	Score	rdb	e⁻ Conf	N-Rule
	335	C53H11N13NaO30PS	697.4540	-1.5	38.3	342	27.00	56.0	even	ok
	336	C61H128N20O8P2S2	697.4565	2.1	38.3	343	25.63	10.0	even	ok
	337	C61H14N3O36P	697.4542	-1.2	38.3	344	30.02	58.0	even	ok
	338	C48H139N12O26PS2	697.4546	-0.7	38.4	345	44.06	-13.0	even	ok
	339	C56H145N2NaO27P2S	697.4559	1.2	38.4	346	30.73	-13.0	even	ok
	340	C59H124N22O10P2S	697.4528	-3.2	38.4	347	11.64	11.0	even	ok
	341	C43H112N44NaO2PS2	697.4540	-1.5	38.4	348	27.54	11.0	even	ok
	342	C60H125N24NaO4P2S2	697.4560	1.3	38.4	349	35.21	12.0	even	ok
	343	C50H22N5O38PS2	697.4555	0.7	38.6	350	43.30	44.0	even	ok
	344	C65H11N5NaO30P	697.4557	0.9	38.8	351	33.92	64.0	even	ok
	345	C59H119N24NaO11S	697.4549	-0.2	39.1	353	41.26	13.0	even	ok
	346	C54H136N10NaO24PS	697.4537	-1.9	39.1	354	22.11	-7.0	even	ok
	347	C67H135N4O21PS	697.4533	-2.5	39.1	355	20.38	4.0	even	ok
	348	C44H3N31O19P2S2	697.4564	1.9	39.2	356	27.11	61.0	even	ok
	349	C50H148N4O29P2S2	697.4567	2.4	39.3	357	20.92	-19.0	even	ok
	350	C63H7N15NaO19PS2	697.4554	0.5	39.3	358	44.95	69.0	even	ok
	351	C63H133N14NaO10P2S2	697.4566	2.3	39.3	359	22.70	6.0	even	ok
	352	C49H19N9NaO34PS2	697.4550	-0.1	39.4	360	51.83	46.0	even	ok
	353	C53H120N28NaO10PS	697.4544	-1.0	39.5	361	32.06	9.0	even	ok
	354	C63H16N3NaO31P2	697.4565	2.1	39.5	362	20.67	59.0	even	ok
	355	C54H133N16NaO17P2S	697.4559	1.2	39.5	363	29.88	-2.0	even	ok
	356	C64H134N2O29	697.4531	-2.8	39.6	364	14.02	0.0	even	ok
	357	C45H124N30NaO12PS2	697.4540	-1.4	39.6	365	26.77	0.0	even	ok
	358	C53H110N36O7S	697.4534	-2.4	39.7	366	17.80	18.0	even	ok
	359	C53H124N26O11P2S	697.4564	1.9	39.7	367	21.79	7.0	even	ok
	360	C53H18N5O38PS	697.4539	-1.7	39.7	368	28.74	49.0	even	ok
	361	C64H136N10O14P2S2	697.4572	3.0	39.8	369	15.16	4.0	even	ok
	362	C51H3N25O21P2S	697.4560	1.4	39.9	370	27.23	65.0	even	ok
	363	C64H126N14O17S	697.4568	2.4	39.9	371	20.39	10.0	even	ok
	364	C47H136N16NaO22PS2	697.4540	-1.4	40.0	372	31.86	-11.0	even	ok
	365	C57H114N30O9S	697.4547	-0.4	40.1	373	37.79	17.0	even	ok
	366	C66H132N8NaO17PS	697.4528	-3.3	40.1	374	13.20	6.0	even	ok
	367	C43H116N42O3P2S2	697.4561	1.5	40.1	375	26.32	9.0	even	ok
	368	C58H120N26NaO8PS	697.4564	1.9	40.2	376	21.78	13.0	even	ok
	369	C59H119N28O5PS2	697.4543	-1.1	40.2	377	36.78	16.0	even	ok
	370	C66H131N8NaO19S	697.4569	2.6	40.2	378	18.30	6.0	even	ok
	371	C54H120N30O7P2S	697.4571	2.9	40.2	379	13.46	12.0	even	ok
	372	C53H15N11O31P2S	697.4560	1.4	40.4	380	26.79	54.0	even	ok
	373	C63H6N11O28P	697.4555	0.7	40.5	381	34.69	68.0	even	ok
	374	C58H116N32NaOPS2	697.4538	-1.8	40.5	382	26.88	18.0	even	ok
	375	C64H10N11O23PS2	697.4560	1.3	40.6	383	33.16	67.0	even	ok
	376	C51H28NNaO37P2S2	697.4572	3.1	40.7	384	14.60	40.0	even	ok
	377	C49H145N8NaO25P2S2	697.4562	1.7	40.7	385	28.48	-17.0	even	ok
	378	C51H6N19O28PS	697.4539	-1.7	40.7	386	23.24	60.0	even	ok
	379	C54H116N32NaO6PS	697.4550	-0.0	40.8	387	41.97	14.0	even	ok
	380	C47H14N15O32PS2	697.4549	-0.3	40.8	388	46.98	50.0	even	ok

Compound Spectrum SmartFormula Report

Meas. m/z	#	Ion Formula	m/z	err [ppm]	mSigma	# mSigma	Score	rdb	e⁻ Conf	N-Rule
	381	C54H140N8O25P2S	697.4557	1.0	40.9	389	30.89	-9.0	even	ok
	382	C41H107N50PS2	697.4539	-1.7	41.0	390	23.66	15.0	even	ok
	383	C52H124N24NaO14PS	697.4537	-1.9	41.0	391	20.91	4.0	even	ok
	384	C68H9N3O33	697.4554	0.5	41.1	392	36.58	67.0	even	ok
	385	C61H124N22NaO7PS2	697.4545	-0.9	41.1	393	38.49	12.0	even	ok
	386	C66H141N4NaO16P2S2	697.4573	3.2	41.2	394	13.04	0.0	even	ok
	387	C64H12N7NaO27P2	697.4572	3.0	41.4	395	12.14	64.0	even	ok
	388	C62H127N18O11PS2	697.4550	-0.1	41.4	396	48.63	10.0	even	ok
	389	C56H115N32O6PS	697.4562	1.7	41.5	397	22.90	17.0	even	ok
	390	C67H10N5O30P	697.4569	2.6	41.5	398	15.07	67.0	even	ok
	391	C63H2N13NaO27	697.4535	-2.2	41.6	399	18.02	70.0	even	ok
	392	C52H131N16O22PS	697.4536	-2.1	41.7	400	18.83	-3.0	even	ok
	393	C61H11N9O29P2	697.4563	1.9	41.8	402	21.22	63.0	even	ok
	394	C61H7N11NaO28P	697.4543	-1.0	41.8	403	29.32	65.0	even	ok
	395	C51H115N34O8PS	697.4542	-1.2	41.8	404	27.90	13.0	even	ok
	396	C45H131N22O20PS2	697.4539	-1.6	41.9	405	27.89	-7.0	even	ok
	397	C45H128N28O13P2S2	697.4561	1.5	41.9	406	24.99	-2.0	even	ok
	398	C43H119N36O10PS2	697.4539	-1.6	41.9	407	23.11	4.0	even	ok
	399	C52H15N9NaO34PS	697.4533	-2.5	42.0	408	19.01	51.0	even	ok
	400	C66H7N13O20P2S	697.4558	1.0	42.0	409	35.28	72.0	even	ok
	401	C49H23N7O35P2S2	697.4570	2.9	42.0	410	15.67	44.0	even	ok
	402	C46H11N19NaO28PS2	697.4543	-1.0	42.0	411	35.28	52.0	even	ok
	403	C52H128N22O15P2S	697.4557	1.0	42.1	412	29.96	2.0	even	ok
	404	C62H139O30P	697.4539	-1.7	42.1	413	22.94	-5.0	even	ok
	405	C50H9N15O34S	697.4573	3.2	42.2	414	12.66	55.0	even	ok
	406	C54H24NNaO37P2S	697.4555	0.6	42.2	415	40.22	45.0	even	ok
	407	C60H11N7NaO32P	697.4536	-2.0	42.3	416	19.60	60.0	even	ok
	408	C69H15N3O26P2S	697.4564	2.0	42.4	417	23.63	66.0	even	ok
	409	C66H15N5NaO25PS2	697.4561	1.5	42.4	418	29.13	63.0	even	ok
	410	C47H140N14O23P2S2	697.4561	1.5	42.5	419	29.44	-13.0	even	ok
	411	C68H12N7NaO22P2S	697.4559	1.2	42.6	420	32.21	68.0	even	ok
	412	C67H135NaO27	697.4539	-1.7	42.7	421	22.32	1.0	even	ok
	413	C67H11N5O28P2	697.4527	-3.3	42.7	422	9.96	67.0	even	ok
	414	C65H133N10NaO14P2S	697.4543	-1.1	42.7	423	27.92	6.0	even	ok
	415	C64H132N12NaO13PS2	697.4551	0.1	42.7	424	46.48	6.0	even	ok
	416	C51H127N20NaO20S	697.4571	3.0	42.7	425	11.76	-1.0	even	ok
	417	C63H135N4O26P	697.4546	-0.7	42.8	426	32.24	0.0	even	ok
	418	C52H111N38O4PS	697.4549	-0.2	42.8	427	37.45	18.0	even	ok
	419	C54H143N2O32PS	697.4536	-2.1	42.8	428	18.30	-14.0	even	ok
	420	C65H4N17NaO16P2S	697.4552	0.3	42.9	429	44.38	74.0	even	ok
	421	C59H14N3NaO38	697.4571	2.9	42.9	430	12.15	55.0	even	ok
	422	C63H128N16O12P2S	697.4541	-1.3	42.9	431	25.71	10.0	even	ok
	423	C60H15N5O33P2	697.4557	0.9	43.0	432	29.91	58.0	even	ok
	424	C48H20N11NaO31P2S2	697.4565	2.1	43.0	433	22.22	46.0	even	ok
	425	C56H111N34NaO5S	697.4542	-1.2	43.1	434	26.69	19.0	even	ok
	426	C60H118N26O8S2	697.4528	-3.2	43.1	435	12.59	16.0	even	ok

Compound Spectrum SmartFormula Report

Meas. m/z	#	Ion Formula	m/z	err [ppm]	mSigma	# mSigma	Score	rdb	e¯ Conf	N-Rule
	427	C62H7N13O25P2	697.4570	2.8	43.3	436	12.81	68.0	even	ok
	428	C52H12N15NaO27P2S	697.4555	0.6	43.4	437	32.48	56.0	even	ok
	429	C52H107N40NaO3S	697.4529	-3.1	43.4	438	10.77	20.0	even	ok
	430	C64H6N13NaO22S2	697.4539	-1.6	43.4	439	27.01	69.0	even	ok
	431	C53H139N6NaO30S	697.4572	3.0	43.4	440	11.48	-12.0	even	ok
	432	C67H6N7NaO29	697.4548	-0.3	43.5	442	35.72	69.0	even	ok
	433	C65H135N8O17PS2	697.4557	0.9	43.5	443	35.57	4.0	even	ok
	434	C50H119N30O12PS	697.4536	-2.1	43.6	444	17.77	8.0	even	ok
	435	C44H6N25O26PS2	697.4542	-1.2	43.7	445	31.24	56.0	even	ok
	436	C52H121N30NaO7P2S	697.4559	1.2	43.7	446	26.65	9.0	even	ok
	437	C44H128N26NaO16PS2	697.4534	-2.4	43.9	447	18.65	-5.0	even	ok
	438	C50H3N23NaO24PS	697.4533	-2.5	43.9	448	14.96	62.0	even	ok
	439	C65H130N6O25	697.4537	-1.9	44.0	449	19.71	5.0	even	ok
	440	C42H122N32O16S2	697.4574	3.3	44.0	450	11.56	-1.0	even	ok
	441	C59H2N17O26P	697.4542	-1.2	44.1	451	25.53	69.0	even	ok
	442	C62H3N15NaO24P	697.4550	-0.1	44.1	452	37.50	70.0	even	ok
	443	C67H18NO29PS2	697.4566	2.3	44.1	453	19.83	61.0	even	ok
	444	C53H117N34NaO3P2S	697.4565	2.1	44.1	454	17.66	14.0	even	ok
	445	C64H141NaO25P2	697.4562	1.6	44.2	455	21.74	-4.0	even	ok
	446	C52H19N7O35P2S	697.4554	0.4	44.2	456	40.50	49.0	even	ok
	447	C53H137N12NaO21P2S	697.4552	0.2	44.2	457	36.07	-7.0	even	ok
	448	C46H137N18NaO19P2S2	697.4555	0.7	44.3	458	37.06	-11.0	even	ok
	449	C62H123N20NaO10S2	697.4530	-3.0	44.3	459	13.61	12.0	even	ok
	450	C67H6N11O23PS	697.4543	-1.1	44.3	460	31.95	72.0	even	ok
	451	C54H106N40O3S	697.4541	-1.4	44.4	461	23.81	23.0	even	ok
	452	C66H7N9NaO26P	697.4563	1.8	44.4	462	19.92	69.0	even	ok
	453	C66H136N2NaO24P	697.4554	0.5	44.5	464	33.19	1.0	even	ok
	454	C46H15N17O29P2S2	697.4564	1.9	44.6	465	23.24	50.0	even	ok
	455	C44H125N32NaO9P2S2	697.4555	0.7	44.6	466	30.64	0.0	even	ok
	456	C66H3N15NaO19PS	697.4537	-1.9	44.7	467	23.17	74.0	even	ok
	457	C58H6N13O30P	697.4535	-2.2	44.8	468	16.65	64.0	even	ok
	458	C55H112N36NaO2PS	697.4557	0.9	44.8	469	28.06	19.0	even	ok
	459	C42H116N40NaO6PS2	697.4534	-2.4	44.9	470	15.04	6.0	even	ok
	460	C63H126N16O14S2	697.4535	-2.2	44.9	471	19.69	10.0	even	ok
	461	C65H9N9O26S2	697.4545	-0.8	45.0	472	34.60	67.0	even	ok
	462	C49H115N34NaO10S	697.4571	3.0	45.0	473	11.06	10.0	even	ok
	463	C40H110N46O6S2	697.4573	3.3	45.0	474	9.40	10.0	even	ok
	464	C62H125N20NaO8P2S	697.4536	-2.1	45.0	475	17.52	12.0	even	ok
	465	C67H140N2NaO19PS2	697.4558	1.1	45.1	476	31.62	0.0	even	ok
	466	C63H123N18NaO13S	697.4562	1.7	45.1	477	20.80	12.0	even	ok
	467	C51H128N20NaO18PS	697.4530	-2.9	45.2	478	11.64	-1.0	even	ok
	468	C69H11N5NaO25PS	697.4544	-0.9	45.2	479	33.51	68.0	even	ok
	469	C61H118N24O11S	697.4561	1.5	45.3	481	22.51	16.0	even	ok
	470	C49H122N26O18S	697.4570	2.8	45.3	482	12.17	3.0	even	ok
	471	C43H3N29NaO22PS2	697.4537	-2.0	45.3	483	21.73	58.0	even	ok
	472	C59H15N3NaO36P	697.4530	-3.0	45.3	484	11.17	55.0	even	ok

Compound Spectrum SmartFormula Report

Meas. m/z	#	Ion Formula	m/z	err [ppm]	mSigma	# mSigma	Score	rdb	e⁻ Conf	N-Rule
	473	C70H14NO29PS	697.4549	-0.2	45.3	485	42.68	66.0	even	ok
	474	C57H9N9O36	697.4570	2.7	45.4	486	12.56	59.0	even	ok
	475	C60H139NaO32	697.4568	2.5	45.5	487	14.00	-8.0	even	ok
	476	C49H22N3NaO41S2	697.4528	-3.2	45.6	488	11.96	41.0	even	ok
	477	C64H2N15O24P	697.4562	1.6	45.6	489	20.96	73.0	even	ok
	478	C60H120N26O6P2S	697.4535	-2.3	45.7	490	15.67	16.0	even	ok
	479	C53H140N6NaO28PS	697.4530	-2.9	45.7	491	11.51	-12.0	even	ok
	480	C60H8N13NaO25P2	697.4558	1.1	45.8	492	25.76	65.0	even	ok
	481	C50H112N38NaO4PS	697.4537	-1.9	45.8	493	18.19	15.0	even	ok
	482	C50H7N21O25P2S	697.4553	0.4	45.8	494	32.26	60.0	even	ok
	483	C51H134N12O28S	697.4570	2.8	45.8	495	11.92	-8.0	even	ok
	484	C51H125N26NaO11P2S	697.4552	0.2	45.9	496	34.47	4.0	even	ok
	485	C45H12N21NaO25P2S2	697.4558	1.1	46.0	497	30.41	52.0	even	ok
	486	C61H136N4NaO26P	697.4534	-2.4	46.1	498	14.46	-3.0	even	ok
	487	C64H131N8O22P	697.4552	0.3	46.1	499	33.69	5.0	even	ok
	488	C59H130N10O26	697.4573	3.3	46.1	500	9.15	1.0	even	ok
	489	C50H116N36O5P2S	697.4557	1.0	46.2	501	26.72	13.0	even	ok
	490	C44H132N24O17P2S2	697.4554	0.5	46.2	502	37.43	-7.0	even	ok
	491	C57H2N17NaO28	697.4571	2.9	46.3	503	11.05	66.0	even	ok
	492	C51H112N40OP2S	697.4564	1.9	46.3	504	18.16	18.0	even	ok
	493	C53H107N42PS	697.4556	0.7	46.4	505	28.74	23.0	even	ok
	494	C52H21NO44S	697.4573	3.2	46.4	506	11.17	44.0	even	ok
	495	C62H136N6O23P2	697.4561	1.4	46.4	507	22.14	0.0	even	ok
	496	C65H131N10NaO16S2	697.4536	-2.0	46.4	508	20.68	6.0	even	ok
	497	C61H140N2O27P2	697.4554	0.5	46.5	509	31.09	-5.0	even	ok
	498	C51H108N42NaPS	697.4544	-1.0	46.7	510	26.07	20.0	even	ok
	499	C59H19NO37P2	697.4550	-0.1	46.7	511	35.14	53.0	even	ok
	500	C62H132N8NaO22P	697.4540	-1.5	46.7	512	21.75	2.0	even	ok
719.4672	1	C76H7N11O19P2	719.4692	2.8	0.8	1	33.13	81.0	even	ok
	2	C76H3N13NaO18P	719.4672	-0.0	2.9	2	100.00	83.0	even	ok
	3	C68H116N30NaPS	719.4672	0.0	3.2	3	99.76	27.0	even	ok
	4	C75H4N15NaO15P2	719.4687	2.0	4.0	4	45.92	83.0	even	ok
	5	C77H135N2O20P	719.4667	-0.6	4.2	5	81.30	13.0	even	ok
	6	C78H134O23	719.4652	-2.7	4.7	6	32.37	13.0	even	ok
	7	C67H110N34OS	719.4656	-2.2	5.0	7	40.96	31.0	even	ok
	8	C79H137N2NaO15P2	719.4690	2.6	5.4	8	34.33	14.0	even	ok
	9	C77H132N8O13P2	719.4689	2.4	5.5	9	37.92	18.0	even	ok
	10	C77H6N9O22P	719.4677	0.7	6.2	10	76.50	81.0	even	ok
	11	C76H132N6NaO16P	719.4662	-1.4	6.4	11	59.25	15.0	even	ok
	12	C69H119N26O4PS	719.4677	0.8	7.2	12	73.38	25.0	even	ok
	13	C78H131N6O16P	719.4674	0.3	7.8	13	84.93	18.0	even	ok
	14	C69H115N28NaO3S	719.4657	-2.1	7.8	14	42.77	27.0	even	ok
	15	C77H128N10NaO12P	719.4669	-0.4	8.0	15	80.84	20.0	even	ok
	16	C75H123N16O10P	719.4667	-0.6	8.5	16	75.16	24.0	even	ok
	17	C77H2N11NaO21	719.4657	-2.1	8.7	17	40.83	83.0	even	ok
	18	C76H129N12NaO9P2	719.4684	1.6	8.9	18	50.59	20.0	even	ok

Compound Spectrum SmartFormula Report

Meas. m/z	#	Ion Formula	m/z	err [ppm]	mSigma	# mSigma	Score	rdb	e¯ Conf	N-Rule
	19	C75H7N9NaO22P	719.4665	-1.0	9.0	19	66.09	78.0	even	ok
	20	C76H136N4O17P2	719.4682	1.5	9.2	20	54.48	13.0	even	ok
	21	C74H127N12O14P	719.4661	-1.6	9.2	21	51.90	19.0	even	ok
	22	C79H11N3NaO24P	719.4678	0.9	9.3	22	80.93	77.0	even	ok
	23	C76H10N5O26P	719.4670	-0.2	9.6	23	84.13	76.0	even	ok
	24	C76H122N14O13	719.4652	-2.7	9.7	24	29.48	24.0	even	ok
	25	C71H124N20NaO6PS	719.4679	1.0	9.8	25	65.29	21.0	even	ok
	26	C80H136NaO18P	719.4676	0.5	9.8	26	76.92	14.0	even	ok
	27	C77H9N3O29	719.4655	-2.3	10.3	27	43.37	76.0	even	ok
	28	C73H2N15O20P	719.4664	-1.2	10.8	28	59.48	82.0	even	ok
	29	C74H124N18O7P2	719.4682	1.4	11.1	29	52.79	24.0	even	ok
	30	C78H127N8NaO15	719.4654	-2.5	11.1	30	31.86	20.0	even	ok
	31	C77H16NNaO25P2	719.4687	2.0	11.2	31	40.26	72.0	even	ok
	32	C74H120N20NaO6P	719.4662	-1.4	11.8	32	53.47	26.0	even	ok
	33	C70H118N24O7S	719.4663	-1.3	11.9	33	65.88	25.0	even	ok
	34	C78H5N7O25	719.4662	-1.4	12.0	34	64.06	81.0	even	ok
	35	C75H11N7O23P2	719.4685	1.9	12.3	35	43.11	76.0	even	ok
	36	C75H133N8NaO13P2	719.4677	0.7	12.4	36	68.19	15.0	even	ok
	37	C79H130N4O19	719.4659	-1.8	12.9	37	44.16	18.0	even	ok
	38	C73H124N16NaO10P	719.4655	-2.3	13.1	38	34.22	21.0	even	ok
	39	C70H115N30PS	719.4684	1.7	13.5	39	45.16	30.0	even	ok
	40	C67H120N26NaO4PS	719.4665	-0.9	13.5	40	62.22	22.0	even	ok
	41	C74H8N11NaO19P2	719.4680	1.1	13.6	41	57.38	78.0	even	ok
	42	C72H127N16O10PS	719.4684	1.7	13.7	42	53.81	19.0	even	ok
	43	C69H122N20O11S	719.4656	-2.2	13.7	43	41.99	20.0	even	ok
	44	C72H115N26O4P	719.4661	-1.6	13.8	44	47.47	30.0	even	ok
	45	C70H128N16NaO10PS	719.4672	0.0	13.9	45	98.39	16.0	even	ok
	46	C68H123N22O8PS	719.4671	-0.2	14.3	46	78.39	20.0	even	ok
	47	C72H123N18NaO9S	719.4664	-1.1	14.5	47	67.61	21.0	even	ok
	48	C80H10NNaO27	719.4663	-1.2	14.6	48	65.73	77.0	even	ok
	49	C65H115N32O2PS	719.4664	-1.1	14.8	49	56.45	26.0	even	ok
	50	C71H127N14NaO13S	719.4657	-2.0	15.0	50	44.99	16.0	even	ok
	51	C73H128N14O11P2	719.4676	0.5	15.2	51	68.86	19.0	even	ok
	52	C73H121N22NaO3P2	719.4677	0.7	15.3	52	64.47	26.0	even	ok
	53	C69H129N18NaO7P2S	719.4687	2.1	15.8	53	35.62	16.0	even	ok
	54	C72H3N17O17P2	719.4679	0.9	15.9	54	58.96	82.0	even	ok
	55	C71H119N22O8P	719.4654	-2.5	16.0	55	29.23	25.0	even	ok
	56	C74H132N10NaO12PS	719.4686	1.9	16.2	56	46.91	15.0	even	ok
	57	C72H120N24NaO2PS	719.4686	1.9	16.3	57	39.15	26.0	even	ok
	58	C71H131N12O14PS	719.4678	0.8	16.3	58	73.81	14.0	even	ok
	59	C78H2N13O18P	719.4684	1.6	16.4	59	43.85	86.0	even	ok
	60	C67H124N24O5P2S	719.4686	1.9	16.7	60	38.34	20.0	even	ok
	61	C77H125N16NaO5P2	719.4690	2.6	17.0	61	27.70	25.0	even	ok
	62	C75H136N2NaO20P	719.4655	-2.3	17.1	62	31.65	10.0	even	ok
	63	C75H120N22O3P2	719.4689	2.4	17.1	63	30.52	29.0	even	ok
	64	C70H115N26NaO6	719.4690	2.5	17.3	64	28.75	27.0	even	ok

Compound Spectrum SmartFormula Report

Meas. m/z	#	Ion Formula	m/z	err [ppm]	mSigma	# mSigma	Score	rdb	e¯ Conf	N-Rule
	65	C66H121N28NaOP2S	719.4680	1.2	17.3	65	51.93	22.0	even	ok
	66	C73H136N6NaO16PS	719.4679	1.0	17.6	66	67.07	10.0	even	ok
	67	C71H2N19O16PS	719.4687	2.1	17.9	67	41.07	82.0	even	ok
	68	C71H112N30NaP	719.4655	-2.3	17.9	68	30.93	32.0	even	ok
	69	C71H116N28OP2	719.4676	0.5	17.9	69	65.35	30.0	even	ok
	70	C72H130N10O17S	719.4663	-1.3	18.3	70	58.15	14.0	even	ok
	71	C73H126N14O13S	719.4669	-0.4	18.4	71	80.98	19.0	even	ok
	72	C76H119N20O6P	719.4674	0.3	18.5	72	69.09	29.0	even	ok
	73	C73H123N20O6PS	719.4691	2.6	18.6	73	30.97	24.0	even	ok
	74	C80H7N7NaO20P	719.4685	1.8	18.8	75	38.26	82.0	even	ok
	75	C72H125N18NaO7P2	719.4670	-0.2	19.0	76	69.75	21.0	even	ok
	76	C75H14NO30P	719.4664	-1.2	19.2	77	60.48	71.0	even	ok
	77	C78H124N14NaO8P	719.4675	0.5	19.3	78	63.81	25.0	even	ok
	78	C73H131N8O18P	719.4654	-2.5	19.3	79	27.39	14.0	even	ok
	79	C69H3N19NaO16PS	719.4675	0.4	19.4	80	77.68	79.0	even	ok
	80	C72H127N12NaO16	719.4690	2.5	19.5	81	27.33	16.0	even	ok
	81	C71H114N28O3S	719.4669	-0.4	19.5	82	65.83	30.0	even	ok
	82	C79H127N10O12P	719.4681	1.2	19.8	83	48.34	23.0	even	ok
	83	C75H116N24NaO2P	719.4669	-0.4	19.8	84	64.03	31.0	even	ok
	84	C68H4N21NaO13P2S	719.4690	2.5	20.0	85	32.02	79.0	even	ok
	85	C73H7N13NaO18PS	719.4689	2.3	20.0	86	35.66	78.0	even	ok
	86	C74H4N17NaO12P2S	719.4654	-2.5	20.1	87	32.38	83.0	even	ok
	87	C73H111N30P	719.4667	-0.6	20.1	88	59.61	35.0	even	ok
	88	C75H135N6O16PS	719.4691	2.6	20.1	89	29.86	13.0	even	ok
	89	C74H135N4NaO19S	719.4664	-1.1	20.2	90	60.34	10.0	even	ok
	90	C68H110N32O4	719.4688	2.3	20.2	91	29.83	31.0	even	ok
	91	C81H10N3O24P	719.4690	2.6	20.3	92	30.94	80.0	even	ok
	92	C70H2N17NaO19S	719.4660	-1.6	20.3	93	48.35	79.0	even	ok
	93	C75H140O21P2	719.4676	0.5	20.4	94	61.74	8.0	even	ok
	94	C74H11N5NaO26P	719.4658	-1.9	20.7	96	35.60	73.0	even	ok
	95	C74H139N2O20PS	719.4684	1.7	20.8	97	46.29	8.0	even	ok
	96	C73H119N22NaO5S	719.4671	-0.2	20.9	98	81.43	26.0	even	ok
	97	C74H110N28O3	719.4652	-2.7	21.1	99	23.39	35.0	even	ok
	98	C75H131N8NaO15S	719.4671	-0.2	21.1	100	81.28	15.0	even	ok
	99	C81H132N4NaO14P	719.4682	1.4	21.6	101	42.97	19.0	even	ok
	100	C77H118N18O9	719.4659	-1.8	21.6	102	36.76	29.0	even	ok
	101	C76H115N22NaO5	719.4654	-2.5	21.6	103	25.62	31.0	even	ok
	102	C74H11N9O20P2S	719.4653	-2.7	21.7	104	28.30	76.0	even	ok
	103	C70H6N15O20PS	719.4680	1.2	21.8	105	56.53	77.0	even	ok
	104	C70H120N24O5P2	719.4669	-0.4	21.9	107	61.61	25.0	even	ok
	105	C70H122N18O14	719.4688	2.3	22.0	108	28.59	20.0	even	ok
	106	C76H132N10O10P2S	719.4656	-2.2	22.3	109	36.58	18.0	even	ok
	107	C74H137N4NaO17P2	719.4670	-0.2	22.3	110	65.05	10.0	even	ok
	108	C72H6N11O24P	719.4657	-2.1	22.3	111	31.38	77.0	even	ok
	109	C65H120N30NaPS2	719.4689	2.4	22.4	112	32.78	22.0	even	ok
	110	C69H132N12NaO14PS	719.4666	-0.9	22.8	113	61.69	11.0	even	ok

Compound Spectrum SmartFormula Report

Meas. m/z	#	Ion Formula	m/z	err [ppm]	mSigma	# mSigma	Score	rdb	e¯ Conf	N-Rule
	111	C81H6N5NaO23	719.4670	-0.3	23.0	114	76.32	82.0	even	ok
	112	C72H11N9NaO22PS	719.4682	1.4	23.0	115	51.02	73.0	even	ok
	113	C79H123N12NaO11	719.4660	-1.6	23.1	116	38.71	25.0	even	ok
	114	C67H127N18O12PS	719.4664	-1.1	23.3	117	56.73	15.0	even	ok
	115	C82H135O18P	719.4688	2.2	23.4	118	29.56	17.0	even	ok
	116	C71H5N13O23S	719.4665	-0.9	23.5	119	60.68	77.0	even	ok
	117	C71H134N6O21S	719.4656	-2.2	23.8	120	34.11	9.0	even	ok
	118	C64H114N34OS2	719.4673	0.1	23.8	121	78.42	26.0	even	ok
	119	C75H138O23S	719.4669	-0.4	23.9	122	72.13	8.0	even	ok
	120	C75H7N13O16P2S	719.4659	-1.8	23.9	123	42.52	81.0	even	ok
	121	C76H16N3NaO22P2S	719.4654	-2.5	24.0	124	29.82	72.0	even	ok
	122	C62H115N34NaOS2	719.4661	-1.6	24.0	125	45.90	23.0	even	ok
	123	C74H15N3O27P2	719.4679	0.9	24.0	126	49.49	71.0	even	ok
	124	C70H135N8O18PS	719.4671	-0.2	24.0	127	76.78	9.0	even	ok
	125	C66H124N26O2P2S2	719.4653	-2.6	24.1	128	27.79	20.0	even	ok
	126	C74H122N18O9S	719.4676	0.6	24.2	129	67.20	24.0	even	ok
	127	C72H140N2NaO20PS	719.4672	0.0	24.2	130	78.90	5.0	even	ok
	128	C74H6N11NaO21S	719.4674	0.2	24.3	131	74.57	78.0	even	ok
	129	C67H107N36Na	719.4683	1.5	24.4	132	38.33	33.0	even	ok
	130	C80H126N8O15	719.4666	-0.8	24.5	133	50.34	23.0	even	ok
	131	C73H139NaO23S	719.4657	-2.0	24.6	134	36.78	5.0	even	ok
	132	C72H132N10O15P2	719.4669	-0.4	24.7	135	57.99	14.0	even	ok
	133	C69H119N22NaO10	719.4683	1.6	25.0	136	37.70	22.0	even	ok
	134	C78H137N4NaO12P2S	719.4658	-2.0	25.0	137	37.72	14.0	even	ok
	135	C73H12N7NaO23P2	719.4673	0.2	25.0	138	61.96	73.0	even	ok
	136	C66H124N22NaO8PS	719.4659	-1.8	25.1	139	33.33	17.0	even	ok
	137	C76H134N4O19S	719.4676	0.6	25.1	140	65.75	13.0	even	ok
	138	C71H141N4NaO17P2S	719.4687	2.1	25.1	141	34.87	5.0	even	ok
	139	C73H10N7NaO25S	719.4667	-0.7	25.1	142	62.65	73.0	even	ok
	140	C69H136N10O15P2S	719.4686	1.9	25.5	143	37.92	9.0	even	ok
	141	C62H119N32O2PS2	719.4681	1.2	25.7	144	50.49	21.0	even	ok
	142	C82H9NO27	719.4675	0.5	25.8	145	66.20	80.0	even	ok
	143	C69H117N28NaOP2	719.4664	-1.2	25.9	146	43.25	27.0	even	ok
	144	C66H119N28NaO3S2	719.4674	0.3	26.0	147	70.27	22.0	even	ok
	145	C73H14N5O26PS	719.4687	2.1	26.1	148	34.17	71.0	even	ok
	146	C68H129N20NaO4P2S2	719.4655	-2.4	26.1	149	29.37	16.0	even	ok
	147	C64H119N28O6PS	719.4657	-2.0	26.4	150	29.52	21.0	even	ok
	148	C64H124N26NaO4PS2	719.4682	1.4	26.6	151	45.68	17.0	even	ok
	149	C77H12N7NaO18P2S	719.4661	-1.6	26.7	152	43.46	77.0	even	ok
	150	C82H131N2NaO17	719.4667	-0.7	26.7	153	51.23	19.0	even	ok
	151	C63H118N30O5S2	719.4666	-0.8	26.9	154	57.49	21.0	even	ok
	152	C76H127N12NaO11S	719.4677	0.8	27.0	156	59.03	20.0	even	ok
	153	C71H7N13O21P2	719.4672	-0.0	27.0	158	62.24	77.0	even	ok
	154	C69H125N24NaP2S2	719.4661	-1.5	27.1	159	44.24	21.0	even	ok
	155	C68H133N14NaO11P2S	719.4680	1.2	27.1	160	41.74	11.0	even	ok
	156	C69H6N13NaO23S	719.4653	-2.6	27.2	161	26.54	74.0	even	ok

Compound Spectrum SmartFormula Report

Meas. m/z	#	Ion Formula	m/z	err [ppm]	mSigma	# mSigma	Score	rdb	e⁻ Conf	N-Rule
	157	C74H120N24P2S	719.4656	-2.2	27.2	162	27.16	29.0	even	ok
	158	C63H116N32NaO2PS	719.4652	-2.8	27.5	163	19.75	23.0	even	ok
	159	C71H129N14NaO11P2	719.4664	-1.2	27.5	164	41.79	16.0	even	ok
	160	C76H125N18NaO2P2S	719.4658	-2.0	27.6	165	35.42	25.0	even	ok
	161	C61H110N38O2S	719.4692	2.8	27.7	166	19.66	27.0	even	ok
	162	C67H114N28O8	719.4682	1.4	27.7	167	38.37	26.0	even	ok
	163	C68H7N15NaO20PS	719.4668	-0.5	27.9	168	63.05	74.0	even	ok
	164	C75H2N15NaO17S	719.4680	1.2	28.1	169	49.44	83.0	even	ok
	165	C66H2N21O18PS	719.4667	-0.7	28.2	170	58.69	78.0	even	ok
	166	C66H128N20O9P2S	719.4679	1.0	28.3	171	43.75	15.0	even	ok
	167	C65H123N24NaO7S2	719.4667	-0.6	28.3	172	59.53	17.0	even	ok
	168	C75H9N7O25S	719.4679	1.0	28.3	173	52.87	76.0	even	ok
	169	C74H13N3O29S	719.4672	0.0	28.7	174	71.24	71.0	even	ok
	170	C79H140O16P2S	719.4663	-1.2	28.7	175	47.60	12.0	even	ok
	171	C72H134N4O24	719.4689	2.3	28.8	176	24.39	9.0	even	ok
	172	C65H125N24NaO5P2S	719.4674	0.2	28.9	177	55.43	17.0	even	ok
	173	C70H4N17NaO17P2	719.4667	-0.8	29.0	178	46.85	79.0	even	ok
	174	C70H9N9O27S	719.4659	-1.8	29.3	179	36.27	72.0	even	ok
	175	C69H10N11O24PS	719.4674	0.3	29.4	180	65.67	72.0	even	ok
	176	C65H127N22O8PS2	719.4688	2.2	29.5	181	30.51	15.0	even	ok
	177	C67H8N17NaO17P2S	719.4683	1.6	29.7	182	39.91	74.0	even	ok
	178	C71H15N5NaO26PS	719.4675	0.4	29.7	183	61.27	68.0	even	ok
	179	C69H132N16O8P2S2	719.4660	-1.7	29.9	184	38.18	14.0	even	ok
	180	C67H122N24O7S2	719.4679	1.0	29.9	185	49.61	20.0	even	ok
	181	C77H115N24O2P	719.4681	1.2	30.1	186	38.31	34.0	even	ok
	182	C72H14N3NaO29S	719.4660	-1.6	30.1	187	38.77	68.0	even	ok
	183	C69H124N20O9P2	719.4662	-1.4	30.2	188	36.30	20.0	even	ok
	184	C74H115N26NaOS	719.4677	0.7	30.2	189	54.79	31.0	even	ok
	185	C84H12NNaO20P2	719.4657	-2.0	30.2	190	32.22	81.0	even	ok
	186	C81H3N11NaO16P	719.4692	2.8	30.3	191	18.53	87.0	even	ok
	187	C82H7N7O18P2	719.4656	-2.2	30.3	192	24.39	85.0	even	ok
	188	C76H3N17O12P2S	719.4666	-0.8	30.3	193	53.12	86.0	even	ok
	189	C70H16N7NaO23P2S	719.4690	2.5	30.4	194	25.16	68.0	even	ok
	190	C71H131N8NaO20	719.4683	1.6	30.4	195	33.09	11.0	even	ok
	191	C77H128N14O6P2S	719.4663	-1.2	30.4	196	45.55	23.0	even	ok
	192	C65H3N23O15P2S	719.4682	1.4	30.4	197	42.57	78.0	even	ok
	193	C68H11N13O21P2S	719.4689	2.3	30.5	199	27.70	72.0	even	ok
	194	C77H130N8O15S	719.4683	1.5	30.5	200	40.71	18.0	even	ok
	195	C78H15N3O22P2S	719.4666	-0.8	30.6	201	52.91	75.0	even	ok
	196	C63H120N30O3P2S	719.4672	0.1	30.7	202	56.34	21.0	even	ok
	197	C77H14NNaO27S	719.4680	1.2	30.9	203	46.18	72.0	even	ok
	198	C67H132N16NaO10PS2	719.4689	2.4	30.9	204	26.79	11.0	even	ok
	199	C67H4N23NaO10P2S2	719.4657	-2.0	30.9	205	31.95	79.0	even	ok
	200	C59H114N36O3S2	719.4653	-2.7	31.1	206	22.53	22.0	even	ok
	201	C66H111N32NaO4	719.4676	0.6	31.1	207	46.63	28.0	even	ok
	202	C70H128N20O4P2S2	719.4667	-0.7	31.1	208	53.47	19.0	even	ok

Compound Spectrum SmartFormula Report

Meas. m/z	#	Ion Formula	m/z	err [ppm]	mSigma	# mSigma	Score	rdb	e¯ Conf	N-Rule
	203	C79H120N18NaO4P	719.4682	1.4	31.2	209	34.42	30.0	even	ok
	204	C61H119N30NaO5S2	719.4654	-2.5	31.3	210	24.86	18.0	even	ok
	205	C66H126N20O11S2	719.4673	0.1	31.6	211	65.05	15.0	even	ok
	206	C80H123N14O8P	719.4687	2.2	31.9	212	24.29	28.0	even	ok
	207	C72H18NO30PS	719.4680	1.2	31.9	213	44.52	66.0	even	ok
	208	C76H5N11O21S	719.4686	1.9	32.0	214	32.87	81.0	even	ok
	209	C71H137N10NaO10P2S2	719.4661	-1.5	32.1	215	39.29	10.0	even	ok
	210	C68H118N28O3S2	719.4686	2.0	32.1	216	31.77	25.0	even	ok
	211	C60H120N32NaO2PS2	719.4669	-0.4	32.2	217	58.11	18.0	even	ok
	212	C69H127N18NaO9S2	719.4681	1.2	32.3	218	43.42	16.0	even	ok
	213	C58H115N38PS2	719.4667	-0.6	32.4	219	54.15	22.0	even	ok
	214	C71H9N7O30	719.4691	2.7	32.6	220	18.02	72.0	even	ok
	215	C69H126N14O18	719.4682	1.4	32.6	221	34.00	15.0	even	ok
	216	C68H136N12O12P2S2	719.4653	-2.6	32.6	222	22.80	9.0	even	ok
	217	C68H136N8NaO18PS	719.4659	-1.8	32.7	223	33.41	6.0	even	ok
	218	C75H118N22O5S	719.4683	1.5	32.9	224	38.50	29.0	even	ok
	219	C82H2N9NaO19	719.4677	0.7	33.0	225	52.30	87.0	even	ok
	220	C79H133N8NaO8P2S	719.4665	-1.0	33.0	226	46.01	19.0	even	ok
	221	C78H8N11NaO14P2S	719.4667	-0.6	33.1	228	53.09	82.0	even	ok
	222	C79H135N2NaO17S	719.4684	1.7	33.3	229	34.94	14.0	even	ok
	223	C73H141NaO21P2	719.4664	-1.2	33.3	230	36.36	5.0	even	ok
	224	C83H132N4O12P2	719.4653	-2.6	33.3	231	18.41	22.0	even	ok
	225	C62H122N26O9S2	719.4659	-1.8	33.3	232	33.83	16.0	even	ok
	226	C69H139N4O22PS	719.4664	-1.1	33.4	233	44.78	4.0	even	ok
	227	C68H131N14NaO13S2	719.4674	0.3	33.4	234	58.70	11.0	even	ok
	228	C68H121N24NaO5P2	719.4657	-2.1	33.4	235	24.12	22.0	even	ok
	229	C77H111N26NaO	719.4660	-1.6	33.4	236	30.24	36.0	even	ok
	230	C70H6N11NaO26	719.4686	2.0	33.5	237	25.58	74.0	even	ok
	231	C66H131N14O16PS	719.4657	-2.0	33.5	238	29.94	10.0	even	ok
	232	C61H123N28O6PS2	719.4674	0.3	33.6	239	58.00	16.0	even	ok
	233	C82H128N8NaO10P	719.4689	2.4	33.6	240	21.09	24.0	even	ok
	234	C78H114N22O5	719.4666	-0.9	33.7	241	40.24	34.0	even	ok
	235	C72H133N14NaO6P2S2	719.4668	-0.6	33.8	242	53.47	15.0	even	ok
	236	C63H128N22NaO8PS2	719.4676	0.5	33.9	243	54.12	12.0	even	ok
	237	C64H106N38O2	719.4675	0.4	33.9	244	46.34	32.0	even	ok
	238	C64H127N20NaO11S2	719.4661	-1.6	34.0	245	36.19	12.0	even	ok
	239	C70H141N6NaO14P2S2	719.4655	-2.4	34.1	246	24.25	5.0	even	ok
	240	C69H3N23O10P2S2	719.4669	-0.3	34.5	247	56.23	82.0	even	ok
	241	C68H7N19O14P2S2	719.4663	-1.3	34.6	248	40.26	77.0	even	ok
	242	C62H129N24NaO5P2S2	719.4691	2.6	34.6	249	21.87	12.0	even	ok
	243	C60H124N30O3P2S2	719.4689	2.4	34.7	250	24.09	16.0	even	ok
	244	C78H10N5NaO23S	719.4687	2.1	34.8	251	27.96	77.0	even	ok
	245	C70H145NaO21P2S	719.4681	1.2	34.8	252	41.30	0.0	even	ok
	246	C68H123N18NaO14	719.4676	0.6	34.9	253	42.35	17.0	even	ok
	247	C70H123N22NaO5S2	719.4687	2.2	34.9	254	27.03	21.0	even	ok
	248	C64H2N25O14PS2	719.4691	2.6	35.0	255	21.69	78.0	even	ok

Compound Spectrum SmartFormula Report

Meas. m/z	#	Ion Formula	m/z	err [ppm]	mSigma	# mSigma	Score	rdb	e⁻ Conf	N-Rule
	249	C80H119N16NaO7	719.4667	-0.7	35.3	256	41.36	30.0	even	ok
	250	C83H5N5O23	719.4682	1.4	35.4	258	37.16	85.0	even	ok
	251	C67H137N10NaO15P2S	719.4674	0.3	35.4	259	56.48	6.0	even	ok
	252	C71H136N6O19P2	719.4662	-1.3	35.4	260	31.88	9.0	even	ok
	253	C77H123N16NaO7S	719.4684	1.7	35.5	261	33.16	25.0	even	ok
	254	C68H140N6O19P2S	719.4679	1.0	35.5	262	43.74	4.0	even	ok
	255	C71H124N24P2S2	719.4673	0.2	35.9	263	57.05	24.0	even	ok
	256	C64H131N18O12PS2	719.4681	1.3	35.9	264	39.17	10.0	even	ok
	257	C72H140N6O14P2S2	719.4667	-0.7	36.0	265	47.32	8.0	even	ok
	258	C66H116N30O3P2	719.4655	-2.3	36.1	266	20.43	26.0	even	ok
	259	C80H136N4O12P2S	719.4670	-0.3	36.2	267	54.72	17.0	even	ok
	260	C66H7N19NaO16PS2	719.4692	2.8	36.3	268	18.89	74.0	even	ok
	261	C70H130N14O13S2	719.4686	2.0	36.3	269	28.49	14.0	even	ok
	262	C68H2N21NaO15S2	719.4684	1.6	36.5	270	33.01	79.0	even	ok
	263	C72H16N3NaO27P2	719.4667	-0.7	36.5	271	38.89	68.0	even	ok
	264	C81H122N12O11	719.4673	0.1	36.5	272	48.19	28.0	even	ok
	265	C65H130N16O15S2	719.4666	-0.8	36.6	273	45.31	10.0	even	ok
	266	C65H128N18NaO12PS	719.4652	-2.8	36.6	274	15.79	12.0	even	ok
	267	C70H12N13NaO16P2S2	719.4664	-1.1	36.6	275	41.19	73.0	even	ok
	268	C79H11N7O18P2S	719.4673	0.1	36.6	276	57.05	80.0	even	ok
	269	C66H136N12NaO14PS2	719.4682	1.4	36.7	277	35.52	6.0	even	ok
	270	C63H122N24O12S	719.4692	2.8	36.8	278	15.59	16.0	even	ok
	271	C69H134N10O17S2	719.4679	1.0	36.9	280	41.43	9.0	even	ok
	272	C65H112N34NaOP	719.4691	2.7	37.0	281	16.18	28.0	even	ok
	273	C71H8N17NaO12P2S2	719.4671	-0.2	37.0	282	55.89	78.0	even	ok
	274	C66H118N24O12	719.4675	0.4	37.3	283	42.38	21.0	even	ok
	275	C69H13N5O31S	719.4652	-2.8	37.3	284	18.63	67.0	even	ok
	276	C62H119N28NaO8S	719.4687	2.0	37.4	285	22.38	18.0	even	ok
	277	C67H11N11NaO24PS	719.4662	-1.4	37.5	286	35.13	69.0	even	ok
	278	C71H144N2O18P2S2	719.4660	-1.7	37.6	287	31.54	3.0	even	ok
	279	C70H133N10NaO15P2	719.4657	-2.1	37.6	288	21.69	11.0	even	ok
	280	C67H135N10NaO17S2	719.4667	-0.6	37.7	289	47.13	6.0	even	ok
	281	C73H136N10O10P2S2	719.4673	0.2	37.8	290	54.12	13.0	even	ok
	282	C64H3N21NaO18PS	719.4655	-2.4	37.8	291	22.67	75.0	even	ok
	283	C67H11N15O18P2S2	719.4656	-2.2	37.9	292	24.40	72.0	even	ok
	284	C65H6N17O22PS	719.4660	-1.6	38.1	293	31.87	73.0	even	ok
	285	C70H11N9O25P2	719.4665	-0.9	38.3	294	34.56	72.0	even	ok
	286	C70H19NNaO30PS	719.4668	-0.5	38.3	295	48.65	63.0	even	ok
	287	C74H145NaO16P2S2	719.4668	-0.5	38.4	296	47.53	4.0	even	ok
	288	C68H14N7O28PS	719.4667	-0.7	38.4	297	45.41	67.0	even	ok
	289	C67H6N17NaO19S2	719.4677	0.7	38.4	298	45.00	74.0	even	ok
	290	C78H126N12O11S	719.4689	2.4	38.5	299	21.49	23.0	even	ok
	291	C83H127N6NaO13	719.4674	0.3	38.6	300	43.00	24.0	even	ok
	292	C73H129N18NaO2P2S2	719.4675	0.4	38.7	301	49.86	20.0	even	ok
	293	C72H135N8NaO15S2	719.4688	2.2	38.7	302	24.33	10.0	even	ok
	294	C79H7N9NaO17PS	719.4652	-2.7	38.8	303	18.38	82.0	even	ok

Compound Spectrum SmartFormula Report

Meas. m/z	#	Ion Formula	m/z	err [ppm]	mSigma	# mSigma	Score	rdb	e⁻ Conf	N-Rule
	295	C71H139N4NaO19S2	719.4681	1.2	38.9	304	36.47	5.0	even	ok
	296	C73H132N12NaO9PS2	719.4653	-2.6	39.0	305	19.04	15.0	even	ok
	297	C60H114N34O6S	719.4685	1.8	39.1	306	23.42	22.0	even	ok
	298	C67H139N8O18PS2	719.4688	2.2	39.2	307	23.71	4.0	even	ok
	299	C70H2N21O13PS2	719.4655	-2.4	39.2	308	21.10	82.0	even	ok
	300	C62H3N25NaO14PS2	719.4679	0.9	39.4	309	40.70	75.0	even	ok
	301	C69H16N9NaO20P2S2	719.4657	-2.0	39.4	310	25.76	68.0	even	ok
	302	C81H16NNaO20P2S	719.4674	0.3	39.4	311	49.89	76.0	even	ok
	303	C63H2N23NaO17S2	719.4664	-1.2	39.6	312	36.87	75.0	even	ok
	304	C69H20N3NaO27P2S	719.4683	1.6	39.8	313	30.71	63.0	even	ok
	305	C65H132N16O13P2S	719.4672	0.1	39.8	314	44.40	10.0	even	ok
	306	C66H12N13NaO21P2S	719.4677	0.7	39.8	315	44.05	69.0	even	ok
	307	C69H8N13NaO21P2	719.4660	-1.7	39.9	316	24.53	74.0	even	ok
	308	C68H128N16O13P2	719.4656	-2.3	40.0	317	18.50	15.0	even	ok
	309	C65H115N28NaO8	719.4670	-0.3	40.0	318	40.86	23.0	even	ok
	310	C78H124N18O2P2S	719.4670	-0.3	40.1	319	49.20	28.0	even	ok
	311	C71H138O28	719.4682	1.4	40.2	320	27.73	4.0	even	ok
	312	C67H15N9O25P2S	719.4682	1.4	40.2	321	32.90	67.0	even	ok
	313	C69H144N2NaO20PS2	719.4689	2.4	40.3	322	20.90	0.0	even	ok
	314	C67H124N20NaO11P	719.4691	2.7	40.3	323	14.74	17.0	even	ok
	315	C69H5N17O19S2	719.4689	2.4	40.4	324	20.98	77.0	even	ok
	316	C70H13N3O34	719.4685	1.8	40.4	325	27.86	67.0	even	ok
	317	C71H15N9O20P2S2	719.4670	-0.3	40.4	326	48.16	71.0	even	ok
	318	C59H111N38NaO2S	719.4680	1.1	40.4	327	30.79	24.0	even	ok
	319	C64H129N20NaO9P2S	719.4667	-0.7	40.5	328	35.78	12.0	even	ok
	320	C75H141N4NaO12P2S2	719.4675	0.4	40.5	330	47.37	9.0	even	ok
	321	C84H130N2O17	719.4679	1.0	40.5	331	31.61	22.0	even	ok
	322	C72H4N21NaO8P2S2	719.4678	0.8	40.6	332	41.37	83.0	even	ok
	323	C68H138N6O21S2	719.4673	0.1	40.6	333	51.28	4.0	even	ok
	324	C64H7N19O19P2S	719.4675	0.5	40.7	334	45.89	73.0	even	ok
	325	C72H11N13O16P2S2	719.4676	0.6	41.0	335	43.57	76.0	even	ok
	326	C80H131N6NaO13S	719.4691	2.6	41.2	336	18.04	19.0	even	ok
	327	C63H6N21O18PS2	719.4684	1.7	41.5	337	28.52	73.0	even	ok
	328	C61H126N22O13S2	719.4653	-2.7	41.5	338	17.22	11.0	even	ok
	329	C70H135N4NaO24	719.4676	0.6	41.5	339	35.31	6.0	even	ok
	330	C72H123N22O3PS2	719.4658	-1.9	41.6	340	25.55	24.0	even	ok
	331	C79H4N15NaO10P2S	719.4674	0.3	41.6	341	47.09	87.0	even	ok
	332	C63H131N16NaO15S2	719.4654	-2.5	41.7	342	18.98	7.0	even	ok
	333	C59H124N28NaO6PS2	719.4662	-1.4	41.7	343	32.19	13.0	even	ok
	334	C72H7N15NaO15PS2	719.4656	-2.2	41.9	344	21.64	78.0	even	ok
	335	C56H116N38NaPS2	719.4655	-2.3	41.9	345	20.97	19.0	even	ok
	336	C68H9N13O23S2	719.4682	1.4	41.9	346	30.85	72.0	even	ok
	337	C81H135N2O15PS	719.4655	-2.4	42.0	347	20.13	17.0	even	ok
	338	C67H3N19O19P2	719.4658	-1.9	42.0	348	21.21	78.0	even	ok
	339	C70H143NaO23S2	719.4674	0.3	42.0	349	46.50	0.0	even	ok
	340	C64H5N19O21S2	719.4669	-0.4	42.1	350	44.76	73.0	even	ok

Compound Spectrum SmartFormula Report

Meas. m/z	#	Ion Formula	m/z	err [ppm]	mSigma	# mSigma	Score	rdb	e⁻ Conf	N-Rule
	341	C62H124N26O7P2S	719.4666	-0.9	42.1	351	31.90	16.0	even	ok
	342	C84H252N3NaO7	719.4671	-0.1	42.1	352	40.82	-39.0	even	ok
	343	C83H3N11O14P2	719.4663	-1.3	42.2	353	27.09	90.0	even	ok
	344	C65H11N15NaO20PS2	719.4685	1.9	42.2	354	25.64	69.0	even	ok
	345	C57H119N34O4PS2	719.4661	-1.5	42.3	355	29.25	17.0	even	ok
	346	C80H10N5O21PS	719.4658	-2.0	42.4	356	24.21	80.0	even	ok
	347	C57H106N44S	719.4678	0.9	42.4	357	31.33	28.0	even	ok
	348	C62H132N18NaO12PS2	719.4669	-0.4	42.5	358	44.26	7.0	even	ok
	349	C74H132N14O6P2S2	719.4680	1.1	42.5	359	34.56	18.0	even	ok
	350	C80H129N12NaO4P2S	719.4671	-0.1	42.6	360	48.74	24.0	even	ok
	351	C73H20N3NaO22P2S2	719.4671	-0.1	42.6	361	48.10	67.0	even	ok
	352	C72H142O23S2	719.4686	2.0	42.6	362	23.89	3.0	even	ok
	353	C63H110N34O6	719.4668	-0.5	42.6	363	35.72	27.0	even	ok
	354	C71H10N11NaO21S2	719.4690	2.6	42.7	364	17.79	73.0	even	ok
	355	C60H127N24O10PS2	719.4668	-0.6	42.7	365	41.34	11.0	even	ok
	356	C70H19N5O24P2S2	719.4663	-1.3	42.7	366	32.45	66.0	even	ok
	357	C81H120N18O2P2	719.4653	-2.6	42.7	367	14.27	33.0	even	ok
	358	C65H119N26O9P	719.4690	2.5	42.8	368	15.24	21.0	even	ok
	359	C76H114N26OS	719.4689	2.4	42.8	369	19.17	34.0	even	ok
	360	C67H140N4NaO22PS	719.4652	-2.8	42.9	370	16.03	1.0	even	ok
	361	C86H11NO20P2	719.4669	-0.4	42.9	371	44.55	84.0	even	ok
	362	C74H135N8O13PS2	719.4658	-1.9	43.0	372	24.63	13.0	even	ok
	363	C65H134N10O22S	719.4692	2.8	43.1	373	15.71	5.0	even	ok
	364	C64H131N14NaO18S	719.4687	2.0	43.1	374	22.94	7.0	even	ok
	365	C66H10N13NaO23S2	719.4670	-0.2	43.1	375	46.20	69.0	even	ok
	366	C68H143O26PS	719.4657	-2.0	43.2	376	23.15	-1.0	even	ok
	367	C80H116N22NaP	719.4689	2.3	43.4	377	16.29	35.0	even	ok
	368	C61H121N30NaO3P2S	719.4660	-1.6	43.4	378	22.90	18.0	even	ok
	369	C68H130N10O22	719.4675	0.4	43.5	379	35.62	10.0	even	ok
	370	C74H16N7NaO18P2S2	719.4678	0.8	43.6	380	37.89	72.0	even	ok
	371	C64H134N12O19S2	719.4659	-1.8	43.6	381	25.72	5.0	even	ok
	372	C70H14N7NaO25S2	719.4684	1.6	43.7	382	26.98	68.0	even	ok
	373	C58H125N30NaO3P2S2	719.4677	0.7	43.9	383	38.39	13.0	even	ok
	374	C61H133N20NaO9P2S2	719.4684	1.7	44.0	384	26.54	7.0	even	ok
	375	C63H135N14O16PS2	719.4674	0.3	44.0	385	43.70	5.0	even	ok
	376	C66H139N6NaO21S2	719.4661	-1.6	44.2	387	27.55	1.0	even	ok
	377	C65H140N8NaO18PS2	719.4676	0.5	44.3	388	40.76	1.0	even	ok
	378	C72H14N7O23PS2	719.4655	-2.4	44.3	389	18.38	71.0	even	ok
	379	C83H125N12NaO4P2	719.4654	-2.4	44.3	390	15.09	29.0	even	ok
	380	C74H128N16NaO5PS2	719.4660	-1.7	44.4	391	25.74	20.0	even	ok
	381	C59H128N26O7P2S2	719.4682	1.5	44.4	392	28.47	11.0	even	ok
	382	C85H8N5NaO16P2	719.4664	-1.1	44.5	393	27.40	86.0	even	ok
	383	C76H144O16P2S2	719.4680	1.1	44.5	394	32.57	7.0	even	ok
	384	C73H7N17O12P2S2	719.4683	1.5	44.5	395	27.74	81.0	even	ok
	385	C63H4N23NaO15P2S	719.4670	-0.3	44.6	396	36.37	75.0	even	ok
	386	C56H120N36OP2S2	719.4676	0.5	44.7	397	40.08	17.0	even	ok

Compound Spectrum SmartFormula Report

Meas. m/z	#	Ion Formula	m/z	err [ppm]	mSigma	# mSigma	Score	rdb	e⁻ Conf	N-Rule
	387	C66H14N11O24PS2	719.4691	2.6	44.7	398	16.55	67.0	even	ok
	388	C80H7N11O14P2S	719.4679	1.0	44.8	399	33.35	85.0	even	ok
	389	C69H10N7NaO30	719.4679	1.0	44.9	400	27.76	69.0	even	ok
	390	C64H141N10NaO15P2S2	719.4691	2.6	45.0	401	16.41	1.0	even	ok
	391	C62H136N16O13P2S2	719.4689	2.4	45.0	402	18.12	5.0	even	ok
	392	C78H119N20NaO3S	719.4691	2.6	45.2	403	16.16	30.0	even	ok
	393	C81H132N8O8P2S	719.4677	0.6	45.3	404	37.92	22.0	even	ok
	394	C59H116N36OP2S	719.4659	-1.8	45.4	405	19.87	22.0	even	ok
	395	C76H137N8NaO8P2S2	719.4681	1.3	45.4	406	29.53	14.0	even	ok
	396	C67H127N14NaO18	719.4670	-0.3	45.4	407	35.21	12.0	even	ok
	397	C84H128N8O8P2	719.4660	-1.7	45.5	408	20.78	27.0	even	ok
	398	C79H123N16O5PS	719.4655	-2.4	45.6	409	18.09	28.0	even	ok
	399	C64H116N30NaO5P	719.4685	1.8	45.7	410	20.09	23.0	even	ok
	400	C67H144N2O23P2S	719.4672	0.1	45.7	411	45.00	-1.0	even	ok
	401	C62H107N38NaO2	719.4663	-1.2	45.7	412	24.97	29.0	even	ok
	402	C76H140N2NaO15PS2	719.4660	-1.7	45.7	413	24.84	9.0	even	ok
	403	C66H141N6NaO19P2S	719.4667	-0.7	45.8	415	37.03	1.0	even	ok
	404	C73H10N11O19PS2	719.4661	-1.5	45.9	416	27.05	76.0	even	ok
	405	C79H110N26O	719.4672	0.1	45.9	417	37.19	39.0	even	ok
	406	C67H13N9O27S2	719.4676	0.5	46.0	418	38.77	67.0	even	ok
	407	C73H3N19NaO11PS2	719.4663	-1.3	46.1	419	29.06	83.0	even	ok
	408	C66H143N4O22PS2	719.4681	1.3	46.2	420	29.38	-1.0	even	ok
	409	C69H136N6NaO21P	719.4691	2.7	46.3	421	12.37	6.0	even	ok
	410	C70H140N2O23P2	719.4656	-2.3	46.4	422	15.46	4.0	even	ok
	411	C67H142N2O25S2	719.4666	-0.8	46.6	423	34.41	-1.0	even	ok
	412	C67H5N13O28	719.4678	0.8	46.6	424	28.36	73.0	even	ok
	413	C74H19NNaO25PS2	719.4656	-2.2	46.7	426	18.93	67.0	even	ok
	414	C62H6N19NaO21S2	719.4657	-2.1	47.2	428	19.86	70.0	even	ok
	415	C75H12N11NaO14P2S2	719.4684	1.7	47.3	429	23.51	77.0	even	ok
	416	C71H17N3O29S2	719.4689	2.4	47.3	430	17.12	66.0	even	ok
	417	C69H18N3NaO29S2	719.4677	0.7	47.3	431	34.91	63.0	even	ok
	418	C63H6N17NaO24S	719.4690	2.4	47.4	432	16.60	70.0	even	ok
	419	C80H3N13NaO13PS	719.4659	-1.8	47.4	433	22.85	87.0	even	ok
	420	C69H23NO28P2S2	719.4656	-2.2	47.4	434	18.75	61.0	even	ok
	421	C66H15N7NaO28PS	719.4655	-2.3	47.5	435	17.35	64.0	even	ok
	422	C82H12N5NaO16P2S	719.4681	1.2	47.5	436	28.56	81.0	even	ok
	423	C86H133N2NaO10P2	719.4661	-1.5	47.5	437	21.30	23.0	even	ok
	424	C81H115N20NaO3	719.4674	0.3	47.6	438	33.42	35.0	even	ok
	425	C65H122N20O16	719.4668	-0.5	47.6	439	30.99	16.0	even	ok
	426	C75H19N3O22P2S2	719.4683	1.5	47.6	440	25.25	70.0	even	ok
	427	C61H7N21NaO18PS2	719.4672	-0.0	47.8	441	42.95	70.0	even	ok
	428	C59H2N27O16PS2	719.4670	-0.2	47.9	442	40.50	74.0	even	ok
	429	C83H137N2NaO10P2S	719.4678	0.8	48.0	443	32.81	18.0	even	ok
	430	C67H18N3O32PS	719.4660	-1.6	48.0	444	24.12	62.0	even	ok
	431	C66H2N17NaO24	719.4673	0.1	48.0	445	34.68	75.0	even	ok
	432	C86H6N3NaO21	719.4690	2.5	48.1	446	15.41	86.0	even	ok

Compound Spectrum SmartFormula Report

Meas. m/z	#	Ion Formula	m/z	err [ppm]	mSigma	# mSigma	Score	rdb	e¯ Conf	N-Rule
	433	C81H128N10NaO7PS	719.4656	-2.2	48.1	447	18.48	24.0	even	ok
	434	C64H10N13O26PS	719.4654	-2.5	48.2	448	15.36	68.0	even	ok
	435	C75H131N12O9PS2	719.4665	-1.0	48.2	449	31.10	18.0	even	ok
	436	C62H126N20O16S	719.4685	1.8	48.2	450	18.00	11.0	even	ok
	437	C62H111N36O3P	719.4683	1.6	48.3	452	20.25	27.0	even	ok
	438	C60H102N44	719.4662	-1.4	48.4	453	21.31	33.0	even	ok
	439	C67H131N12O19P	719.4690	2.5	48.5	454	12.88	10.0	even	ok
	440	C75H15N5NaO21PS2	719.4663	-1.3	48.6	455	27.08	72.0	even	ok
	441	C82H118N16O7	719.4679	1.0	48.7	457	25.04	33.0	even	ok
	442	C61H123N24NaO12S	719.4680	1.1	48.9	458	24.08	13.0	even	ok
	443	C60H8N23NaO15P2S2	719.4687	2.1	49.1	459	19.02	70.0	even	ok
	444	C63H9N15O25S2	719.4662	-1.4	49.1	460	26.00	68.0	even	ok
	445	C77H140N4O12P2S2	719.4687	2.1	49.2	461	19.01	12.0	even	ok
	446	C58H3N29O13P2S2	719.4685	1.9	49.4	462	20.63	74.0	even	ok
	447	C62H10N17O22PS2	719.4677	0.7	49.4	463	32.61	68.0	even	ok
	448	C85H253N5O2P2	719.4657	-2.1	49.4	464	15.36	-36.0	even	ok
	449	C75H128N18O2P2S2	719.4687	2.0	49.4	465	18.95	23.0	even	ok
	450	C69H15N5O29P2	719.4658	-1.9	49.6	466	17.06	67.0	even	ok
	451	C64H15N11NaO24PS2	719.4679	0.9	49.7	467	30.14	64.0	even	ok
	452	C65H14N9NaO27S2	719.4664	-1.2	49.7	468	27.59	64.0	even	ok
	453	C64H119N24NaO12	719.4663	-1.2	49.9	469	22.09	18.0	even	ok
	454	C65H16N9NaO25P2S	719.4670	-0.3	50.0	470	37.23	64.0	even	ok
	455	C74H6N15O15PS2	719.4668	-0.6	50.0	471	33.94	81.0	even	ok
	456	C66H19N5O29P2S	719.4675	0.5	50.2	472	34.69	62.0	even	ok
	457	C87H131NaO15	719.4687	2.1	50.2	473	17.67	23.0	even	ok
	458	C74H3N21O8P2S2	719.4690	2.5	50.4	474	15.09	86.0	even	ok
	459	C59H118N30O10S	719.4678	0.9	50.5	475	24.67	17.0	even	ok
	460	C66H128N16NaO15P	719.4685	1.8	50.5	476	17.32	12.0	even	ok
	461	C81H6N9O17PS	719.4665	-1.0	50.6	477	28.16	85.0	even	ok
	462	C84H123N10NaO9	719.4681	1.2	50.7	478	21.86	29.0	even	ok
	463	C83H15NO20P2S	719.4686	2.0	50.8	479	18.73	79.0	even	ok
	464	C68H12N9NaO25P2	719.4653	-2.6	51.0	480	11.34	69.0	even	ok
	465	C63H11N15O23P2S	719.4669	-0.5	51.0	481	33.98	68.0	even	ok
	466	C82H131N6O11PS	719.4662	-1.4	51.0	482	23.66	22.0	even	ok
	467	C77H136N6NaO11PS2	719.4666	-0.8	51.0	483	30.61	14.0	even	ok
	468	C64H136N12O17P2S	719.4666	-0.9	51.2	484	24.46	5.0	even	ok
	469	C62H8N19NaO19P2S	719.4663	-1.2	51.2	485	25.82	70.0	even	ok
	470	C76H15N7O18P2S2	719.4690	2.5	51.3	487	14.61	75.0	even	ok
	471	C55H119N34NaO6S2	719.4690	2.5	51.4	488	14.19	14.0	even	ok
	472	C58H128N24NaO10PS2	719.4656	-2.3	51.6	489	15.81	8.0	even	ok
	473	C58H115N34NaO6S	719.4673	0.2	51.7	490	30.47	19.0	even	ok
	474	C65H18N7O28PS2	719.4684	1.7	51.7	491	21.03	62.0	even	ok
	475	C75H124N20NaOPS2	719.4666	-0.8	51.8	492	29.85	25.0	even	ok
	476	C61H136N14NaO16PS2	719.4662	-1.3	51.8	493	23.95	2.0	even	ok
	477	C63H133N16NaO13P2S	719.4660	-1.6	51.9	494	17.86	7.0	even	ok
	478	C63H138N8O23S2	719.4653	-2.7	51.9	495	12.74	0.0	even	ok

Compound Spectrum SmartFormula Report

Meas. m/z	#	Ion Formula	m/z	err [ppm]	mSigma	# mSigma	Score	rdb	e⁻ Conf	N-Rule
	479	C66H17N5O31S2	719.4669	-0.4	52.1	496	33.40	62.0	even	ok
	480	C65H143N2NaO25S2	719.4654	-2.5	52.1	497	14.03	-4.0	even	ok
	481	C59H131N20O14PS2	719.4661	-1.5	52.2	498	21.83	6.0	even	ok
	482	C77H133N12NaO4P2S2	719.4688	2.2	52.3	499	15.81	19.0	even	ok
	483	C56H123N30O8PS2	719.4654	-2.5	52.3	500	14.02	12.0	even	ok
	484	C53H114N40O4S2	719.4689	2.3	52.3	501	15.25	18.0	even	ok
	485	C62H114N30O10	719.4662	-1.4	52.3	502	18.96	22.0	even	ok
	486	C67H23NNaO30PS2	719.4685	1.9	52.3	503	18.92	58.0	even	ok
	487	C66H143NaO28S	719.4687	2.0	52.4	504	17.32	-4.0	even	ok
	488	C85H126N6O13	719.4686	1.9	52.5	505	15.05	27.0	even	ok
	489	C76H18NO25PS2	719.4668	-0.6	52.6	506	31.41	70.0	even	ok
	490	C83H243N13O	719.4676	0.6	52.7	507	25.61	-30.0	even	ok
	491	C69H139NaO28	719.4670	-0.3	52.7	508	28.28	1.0	even	ok
	492	C76H11N9NaO17PS2	719.4669	-0.4	52.8	509	33.14	77.0	even	ok
	493	C65H3N19NaO21P	719.4688	2.2	52.8	510	13.36	75.0	even	ok
	494	C64H123N22O13P	719.4683	1.6	52.8	511	17.56	16.0	even	ok
	495	C64H144N4NaO22PS2	719.4669	-0.4	52.9	512	32.61	-4.0	even	ok
	496	C81H125N16NaP2S	719.4678	0.8	52.9	513	28.28	29.0	even	ok
	497	C62H139N10O20PS2	719.4668	-0.6	52.9	514	30.51	0.0	even	ok
	498	C76H8N15NaO10P2S2	719.4691	2.6	53.2	515	12.47	82.0	even	ok
	499	C64H138N6O26S	719.4685	1.9	53.3	516	18.44	0.0	even	ok
	500	C83H11N3NaO19PS	719.4666	-0.8	53.3	517	27.77	81.0	even	ok
741.4805	1	C56H129N30NaO9P2S	741.4821	2.1	8.7	1	41.17	9.0	even	ok
	2	C54H124N36O7P2S	741.4819	1.9	8.9	2	44.95	13.0	even	ok
	3	C65H144N4NaO28P	741.4796	-1.3	9.1	3	59.87	-3.0	even	ok
	4	C66H147O32P	741.4801	-0.5	9.2	4	78.68	-5.0	even	ok
	5	C54H120N38NaO6PS	741.4799	-0.8	9.2	5	71.30	15.0	even	ok
	6	C65H148N2O29P2	741.4816	1.5	9.2	6	54.90	-5.0	even	ok
	7	C55H123N34O10PS	741.4804	-0.1	9.3	7	90.72	13.0	even	ok
	8	C64H19N7NaO34P	741.4799	-0.9	9.5	8	69.44	60.0	even	ok
	9	C62H14N13O32P	741.4797	-1.1	9.7	9	64.33	64.0	even	ok
	10	C52H115N44O4PS	741.4798	-1.0	9.8	10	65.66	19.0	even	ok
	11	C63H139N10O26P	741.4794	-1.5	9.9	11	54.50	1.0	even	ok
	12	C64H16N13NaO27P2	741.4820	2.0	10.2	12	41.30	65.0	even	ok
	13	C62H11N19O25P2	741.4819	1.9	10.4	13	45.07	69.0	even	ok
	14	C57H128N28NaO12PS	741.4806	0.1	10.6	14	88.90	9.0	even	ok
	15	C53H121N40NaO3P2S	741.4814	1.2	10.7	15	59.86	15.0	even	ok
	16	C61H11N17NaO28P	741.4792	-1.8	10.7	16	46.26	66.0	even	ok
	17	C64H23N5O35P2	741.4819	1.9	10.9	17	44.55	58.0	even	ok
	18	C63H20N9NaO31P2	741.4814	1.1	11.0	18	60.91	60.0	even	ok
	19	C64H145N6NaO25P2	741.4811	0.7	11.1	19	70.73	-3.0	even	ok
	20	C65H22N3O38P	741.4804	-0.2	11.1	20	86.21	58.0	even	ok
	21	C63H10N17O28P	741.4804	-0.2	11.3	21	85.75	69.0	even	ok
	22	C62H7N21NaO24P	741.4799	-0.9	11.8	22	66.28	71.0	even	ok
	23	C56H132N24NaO16PS	741.4799	-0.8	12.1	23	67.79	4.0	even	ok
	24	C54H127N30O14PS	741.4798	-1.0	12.2	24	62.91	8.0	even	ok

Compound Spectrum SmartFormula Report

Meas. m/z	#	Ion Formula	m/z	err [ppm]	mSigma	# mSigma	Score	rdb	e⁻ Conf	N-Rule
	25	C51H112N48NaPS	741.4792	-1.7	12.2	25	46.18	21.0	even	ok
	26	C60H2N27O22P	741.4797	-1.1	12.3	26	61.09	75.0	even	ok
	27	C62H136N14NaO22P	741.4789	-2.2	12.4	27	37.04	3.0	even	ok
	28	C65H15N11NaO30P	741.4805	0.0	12.4	28	87.61	65.0	even	ok
	29	C61H15N15O29P2	741.4812	1.0	12.4	29	63.86	64.0	even	ok
	30	C56H3N31NaO18PS	741.4809	0.5	12.4	30	75.70	72.0	even	ok
	31	C59H6N23O26P	741.4790	-2.0	12.4	31	40.93	70.0	even	ok
	32	C53H124N34NaO10PS	741.4792	-1.7	12.5	32	46.11	10.0	even	ok
	33	C51H116N46OP2S	741.4813	1.0	12.5	33	62.42	19.0	even	ok
	34	C56H122N32O13S	741.4789	-2.1	12.5	34	38.09	13.0	even	ok
	35	C61H8N23NaO21P2	741.4814	1.1	12.6	35	59.25	71.0	even	ok
	36	C66H21NO41	741.4789	-2.2	12.8	36	44.19	58.0	even	ok
	37	C62H140N12O23P2	741.4809	0.6	12.9	37	73.03	1.0	even	ok
	38	C58H131N24O16PS	741.4811	0.8	13.1	38	66.58	7.0	even	ok
	39	C64H148NaO32P	741.4789	-2.2	13.3	39	36.49	-8.0	even	ok
	40	C55H133N26NaO13P2S	741.4814	1.2	13.7	40	56.38	4.0	even	ok
	41	C59H140N14NaO22PS	741.4806	0.1	13.9	41	100.00	-2.0	even	ok
	42	C57H135N20O20PS	741.4804	-0.1	13.9	42	83.34	2.0	even	ok
	43	C51H119N40O8PS	741.4791	-1.9	14.0	43	40.99	14.0	even	ok
	44	C66H18N7O34P	741.4811	0.7	14.0	44	66.85	63.0	even	ok
	45	C56H136N22O17P2S	741.4819	1.9	14.1	45	40.60	2.0	even	ok
	46	C59H3N29O19P2	741.4812	0.9	14.2	46	61.94	75.0	even	ok
	47	C58H141N16NaO19P2S	741.4821	2.1	14.2	47	36.94	-2.0	even	ok
	48	C65H141N10NaO21P2	741.4817	1.7	14.2	48	46.03	2.0	even	ok
	49	C57H6N27O22PS	741.4814	1.2	14.4	49	66.76	70.0	even	ok
	50	C64H9N15O31	741.4789	-2.2	14.5	50	35.50	69.0	even	ok
	51	C58H134N18O23S	741.4789	-2.1	14.5	51	44.09	2.0	even	ok
	52	C58H127N26NaO15S	741.4791	-1.9	14.6	52	40.16	9.0	even	ok
	53	C60H12N19NaO25P2	741.4807	0.2	14.6	53	78.75	66.0	even	ok
	54	C48H116N48NaPS2	741.4809	0.5	14.7	54	70.88	16.0	even	ok
	55	C63H136N16O19P2	741.4816	1.5	14.8	55	49.53	6.0	even	ok
	56	C53H128N32O11P2S	741.4813	1.0	14.9	56	59.42	8.0	even	ok
	57	C57H2N29NaO21S	741.4794	-1.5	14.9	57	57.38	72.0	even	ok
	58	C64H135N14O22P	741.4801	-0.6	15.2	58	69.98	6.0	even	ok
	59	C60H136N18NaO18PS	741.4813	1.0	15.2	59	59.38	3.0	even	ok
	60	C66H140N8NaO24P	741.4802	-0.4	15.6	60	73.85	2.0	even	ok
	61	C47H110N52OS2	741.4793	-1.7	15.7	61	44.53	20.0	even	ok
	62	C53H111N48PS	741.4804	-0.1	15.9	62	79.80	24.0	even	ok
	63	C67H143N4O28P	741.4808	0.4	16.0	63	73.66	0.0	even	ok
	64	C61H137N16NaO19P2	741.4804	-0.2	16.1	64	78.15	3.0	even	ok
	65	C68H23NNaO36P	741.4812	0.9	16.1	65	59.79	59.0	even	ok
	66	C59H130N22O19S	741.4796	-1.2	16.2	66	64.32	7.0	even	ok
	67	C60H139N12NaO25S	741.4791	-1.9	16.2	67	46.81	-2.0	even	ok
	68	C49H115N46NaO3S2	741.4794	-1.5	16.2	68	57.48	16.0	even	ok
	69	C52H125N36NaO7P2S	741.4807	0.3	16.2	69	74.66	10.0	even	ok
	70	C66H14N9NaO33	741.4790	-2.0	16.3	70	37.60	65.0	even	ok

Compound Spectrum SmartFormula Report

Meas. m/z	#	Ion Formula	m/z	err [ppm]	mSigma	# mSigma	Score	rdb	e⁻ Conf	N-Rule
	71	C59H11N21NaO24PS	741.4815	1.4	16.4	71	59.38	66.0	even	ok
	72	C55H116N42NaO2PS	741.4806	0.1	16.4	72	79.48	20.0	even	ok
	73	C49H119N44O4PS2	741.4814	1.3	16.5	73	62.44	14.0	even	ok
	74	C63H132N18NaO18P	741.4796	-1.3	16.6	74	51.65	8.0	even	ok
	75	C60H143N10O26PS	741.4811	0.8	16.6	75	74.21	-4.0	even	ok
	76	C53H2N33O20PS	741.4801	-0.6	16.7	76	66.55	71.0	even	ok
	77	C58H7N25O23P2	741.4805	0.0	16.8	77	79.88	70.0	even	ok
	78	C63H23N3NaO38P	741.4792	-1.8	16.8	78	41.22	55.0	even	ok
	79	C56H119N38O6PS	741.4811	0.8	16.8	79	61.93	18.0	even	ok
	80	C55H7N27NaO22PS	741.4802	-0.4	16.9	80	70.71	67.0	even	ok
	81	C59H134N16O26	741.4822	2.3	17.0	81	31.89	2.0	even	ok
	82	C61H139N14O22PS	741.4818	1.7	17.1	82	50.57	1.0	even	ok
	83	C67H17N5O37	741.4796	-1.3	17.2	83	61.40	63.0	even	ok
	84	C63H149N2NaO29P2	741.4804	-0.2	17.2	84	76.63	-8.0	even	ok
	85	C61H127N24O16P	741.4794	-1.5	17.3	85	47.05	12.0	even	ok
	86	C62H133N20NaO15P2	741.4811	0.7	17.4	86	62.56	8.0	even	ok
	87	C56H10N23O26PS	741.4807	0.3	17.6	88	87.08	65.0	even	ok
	88	C61H18N9O36P	741.4791	-2.0	17.7	89	36.97	59.0	even	ok
	89	C57H122N30O16	741.4822	2.3	17.8	90	31.49	13.0	even	ok
	90	C54H8N29NaO19P2S	741.4817	1.6	17.9	91	43.81	67.0	even	ok
	91	C58H5N25O25S	741.4799	-0.8	18.1	92	71.99	70.0	even	ok
	92	C51H124N38NaO6PS2	741.4816	1.5	18.2	93	55.68	10.0	even	ok
	93	C50H120N42O5P2S	741.4806	0.1	18.2	94	76.06	14.0	even	ok
	94	C62H148N4NaO28PS	741.4813	1.0	18.2	95	66.85	-8.0	even	ok
	95	C54H110N46O3S	741.4789	-2.1	18.3	96	33.79	24.0	even	ok
	96	C61H135N16NaO21S	741.4798	-1.0	18.4	97	66.27	3.0	even	ok
	97	C59H132N22O17P2	741.4803	-0.3	18.4	98	70.19	7.0	even	ok
	98	C58H124N32NaO8PS	741.4812	1.0	18.4	99	55.81	14.0	even	ok
	99	C58H15N17NaO28PS	741.4809	0.5	18.5	100	80.22	61.0	even	ok
	100	C52H3N35O17P2S	741.4816	1.4	18.5	101	46.98	71.0	even	ok
	101	C60H128N26O13P2	741.4809	0.6	18.8	103	64.93	12.0	even	ok
	102	C47H107N54NaS	741.4820	2.0	18.9	104	35.20	22.0	even	ok
	103	C57H9N21O29S	741.4792	-1.7	19.0	105	48.65	65.0	even	ok
	104	C62H4N27NaO17P2	741.4820	2.0	19.0	106	34.82	76.0	even	ok
	105	C50H115N48PS2	741.4821	2.2	19.1	107	32.55	19.0	even	ok
	106	C55H11N25O23P2S	741.4822	2.3	19.1	108	30.11	65.0	even	ok
	107	C61H144N8O27P2	741.4803	-0.3	19.1	109	69.29	-4.0	even	ok
	108	C63H144N8NaO24PS	741.4819	1.9	19.3	110	44.17	-3.0	even	ok
	109	C61H142N8O29S	741.4796	-1.2	19.4	111	60.27	-4.0	even	ok
	110	C63H27NO39P2	741.4812	1.0	19.5	112	55.23	53.0	even	ok
	111	C50H118N42O7S2	741.4800	-0.8	19.5	113	71.59	14.0	even	ok
	112	C56H115N40NaO5S	741.4791	-1.9	19.6	114	36.08	20.0	even	ok
	113	C58H131N20NaO22	741.4817	1.6	19.6	115	42.73	4.0	even	ok
	114	C58H2N31O18PS	741.4821	2.1	19.6	116	33.21	75.0	even	ok
	115	C60H14N17O28PS	741.4821	2.1	19.7	117	39.64	64.0	even	ok
	116	C62H24N5NaO35P2	741.4807	0.2	19.8	118	70.65	55.0	even	ok

Compound Spectrum SmartFormula Report

Meas. m/z	#	Ion Formula	m/z	err [ppm]	mSigma	# mSigma	Score	rdb	e⁻ Conf	N-Rule
	117	C57H4N29NaO19P2	741.4800	-0.7	19.9	119	60.80	72.0	even	ok
	118	C58H144N10NaO26PS	741.4799	-0.8	20.0	120	69.46	-7.0	even	ok
	119	C68H142N2O31	741.4793	-1.7	20.0	121	40.67	0.0	even	ok
	120	C60H124N28NaO12P	741.4789	-2.2	20.0	122	31.60	14.0	even	ok
	121	C59H127N28O12PS	741.4818	1.7	20.2	123	39.61	12.0	even	ok
	122	C60H7N25NaO20PS	741.4822	2.3	20.2	124	35.71	71.0	even	ok
	123	C57H6N23NaO28	741.4820	2.0	20.3	125	35.10	67.0	even	ok
	124	C60H10N19NaO27S	741.4800	-0.6	20.3	126	73.57	66.0	even	ok
	125	C59H14N15NaO31S	741.4794	-1.5	20.4	127	51.31	61.0	even	ok
	126	C64H6N21O24P	741.4811	0.7	20.5	128	58.67	74.0	even	ok
	127	C63H3N25NaO20P	741.4805	0.0	20.6	129	74.32	76.0	even	ok
	128	C49H117N46NaOP2S	741.4801	-0.6	20.7	130	61.06	16.0	even	ok
	129	C55H136N20NaO20PS	741.4792	-1.7	20.8	131	39.05	-1.0	even	ok
	130	C45H115N50O2PS2	741.4801	-0.5	20.9	132	62.34	15.0	even	ok
	131	C57H118N36O9S	741.4796	-1.2	20.9	133	48.41	18.0	even	ok
	132	C47H120N44NaO4PS2	741.4802	-0.4	21.0	134	66.21	11.0	even	ok
	133	C48H123N40O8PS2	741.4808	0.4	21.2	135	78.92	9.0	even	ok
	134	C60H19N11O33P2	741.4805	0.0	21.2	136	72.56	59.0	even	ok
	135	C59H18N13O32PS	741.4814	1.2	21.3	137	57.72	59.0	even	ok
	136	C63H147N2NaO31S	741.4798	-1.0	21.4	138	62.27	-8.0	even	ok
	137	C56H119N34NaO12	741.4817	1.6	21.5	139	41.19	15.0	even	ok
	138	C52H127N34O10PS2	741.4821	2.2	21.5	140	36.95	8.0	even	ok
	139	C63H151O32PS	741.4818	1.7	21.5	141	45.89	-10.0	even	ok
	140	C53H131N26O18PS	741.4791	-1.9	21.5	142	35.16	3.0	even	ok
	141	C52H123N36NaO9S2	741.4801	-0.6	21.5	143	73.27	10.0	even	ok
	142	C62H138N12O25S	741.4803	-0.3	21.5	144	80.05	1.0	even	ok
	143	C60H141N12NaO23P2	741.4797	-1.1	21.6	145	50.74	-2.0	even	ok
	144	C56H139N16O24PS	741.4798	-1.0	21.7	146	51.92	-3.0	even	ok
	145	C59H147N6O30PS	741.4804	-0.1	21.8	147	85.05	-9.0	even	ok
	146	C66H11N15NaO26P	741.4812	0.9	21.8	148	53.13	70.0	even	ok
	147	C62H19N11NaO30PS	741.4822	2.3	21.9	149	34.36	60.0	even	ok
	148	C56H126N26O20	741.4815	1.4	22.0	150	44.08	8.0	even	ok
	149	C50H128N34NaO10PS2	741.4809	0.6	22.0	151	72.81	5.0	even	ok
	150	C58H129N26NaO13P2	741.4797	-1.1	22.0	152	50.05	9.0	even	ok
	151	C51H114N46O3S2	741.4806	0.1	22.1	153	82.96	19.0	even	ok
	152	C46H121N46NaOP2S2	741.4817	1.7	22.1	154	38.92	11.0	even	ok
	153	C49H122N38O11S2	741.4793	-1.7	22.2	155	46.74	9.0	even	ok
	154	C58H148N8O27P2S	741.4819	1.9	22.2	156	41.01	-9.0	even	ok
	155	C59H125N30NaO9P2	741.4804	-0.2	22.3	157	68.43	14.0	even	ok
	156	C60H153N2NaO29P2S	741.4821	2.1	22.3	159	37.25	-13.0	even	ok
	157	C61H132N22NaO14PS	741.4819	1.9	22.4	160	34.56	8.0	even	ok
	158	C61H152NaO32PS	741.4806	0.1	22.5	161	83.30	-13.0	even	ok
	159	C60H146N4O33S	741.4790	-2.1	22.6	162	37.39	-9.0	even	ok
	160	C59H16N15NaO29P2	741.4800	-0.7	22.7	163	57.29	61.0	even	ok
	161	C61H23N7NaO34PS	741.4815	1.4	22.8	165	51.66	55.0	even	ok
	162	C59H123N30NaO11S	741.4798	-1.0	22.9	166	49.98	14.0	even	ok

Compound Spectrum SmartFormula Report

Meas. m/z	#	Ion Formula	m/z	err [ppm]	mSigma	# mSigma	Score	rdb	e¯ Conf	N-Rule
	163	C57H145N12NaO23P2S	741.4814	1.2	23.0	167	46.29	-7.0	even	ok
	164	C61H146N2O36	741.4822	2.3	23.0	168	27.91	-9.0	even	ok
	165	C49H119N40NaO10S	741.4820	2.0	23.1	169	32.02	11.0	even	ok
	166	C67H14N11O30P	741.4817	1.6	23.1	170	38.29	68.0	even	ok
	167	C55H129N32NaO6P2S2	741.4788	-2.3	23.2	171	33.45	9.0	even	ok
	168	C64H2N23NaO23	741.4790	-2.0	23.4	172	32.18	76.0	even	ok
	169	C51H127N32NaO13S2	741.4794	-1.5	23.4	173	49.41	5.0	even	ok
	170	C54H137N22NaO17P2S	741.4807	0.3	23.5	174	63.85	-1.0	even	ok
	171	C55H140N18O21P2S	741.4813	1.0	23.5	175	49.31	-3.0	even	ok
	172	C54H11N23NaO26PS	741.4795	-1.3	23.7	176	52.22	62.0	even	ok
	173	C60H17N11O35S	741.4799	-0.8	23.7	177	63.97	59.0	even	ok
	174	C61H13N15O31S	741.4806	0.1	23.8	178	81.22	64.0	even	ok
	175	C54H114N40O10	741.4815	1.4	23.8	179	42.43	19.0	even	ok
	176	C58H136N18O21P2	741.4796	-1.3	23.9	180	44.66	2.0	even	ok
	177	C64H143N6NaO27S	741.4804	-0.1	23.9	181	80.60	-3.0	even	ok
	178	C60H143N6NaO32	741.4817	1.6	24.1	182	38.64	-7.0	even	ok
	179	C57H120N36O7P2	741.4802	-0.4	24.2	183	61.77	18.0	even	ok
	180	C65H5N19O27	741.4796	-1.3	24.2	184	43.80	74.0	even	ok
	181	C53H119N40NaO5S2	741.4808	0.3	24.4	185	74.11	15.0	even	ok
	182	C56H124N32O11P2	741.4796	-1.3	24.5	186	43.90	13.0	even	ok
	183	C63H129N24NaO11P2	741.4817	1.6	24.6	187	37.10	13.0	even	ok
	184	C51H131N30O14PS2	741.4815	1.3	24.7	188	52.20	3.0	even	ok
	185	C57H11N21O27P2	741.4799	-0.9	24.7	189	51.14	65.0	even	ok
	186	C52H132N28O15P2S	741.4806	0.1	24.7	190	65.88	3.0	even	ok
	187	C55H110N44O6	741.4822	2.3	24.8	191	27.10	24.0	even	ok
	188	C47H114N46O8S	741.4819	1.8	24.9	192	33.67	15.0	even	ok
	189	C55H14N19O30PS	741.4801	-0.6	24.9	193	67.04	60.0	even	ok
	190	C65H131N18O18P	741.4808	0.3	24.9	194	60.89	11.0	even	ok
	191	C61H124N30O9P2	741.4816	1.5	25.0	195	39.92	17.0	even	ok
	192	C69H19N5NaO32P	741.4819	1.8	25.0	196	33.57	64.0	even	ok
	193	C61H6N23NaO23S	741.4807	0.3	25.0	197	74.59	71.0	even	ok
	194	C53H126N32O13S2	741.4806	0.2	25.1	198	77.39	8.0	even	ok
	195	C57H19N13NaO32PS	741.4802	-0.4	25.1	199	71.17	56.0	even	ok
	196	C60H126N26O15S	741.4803	-0.3	25.1	200	61.40	12.0	even	ok
	197	C55H123N30NaO16	741.4810	0.7	25.2	201	54.38	10.0	even	ok
	198	C62H131N20NaO17S	741.4804	-0.1	25.2	202	78.11	8.0	even	ok
	199	C65H28NNaO34P2S	741.4788	-2.4	25.5	203	30.83	54.0	even	ok
	200	C62H123N28O12P	741.4801	-0.6	25.5	204	55.91	17.0	even	ok
	201	C62H22N5NaO37S	741.4801	-0.6	25.5	205	65.71	55.0	even	ok
	202	C64H128N22NaO14P	741.4802	-0.4	25.6	206	59.38	13.0	even	ok
	203	C51H129N32NaO11P2S	741.4801	-0.6	25.7	207	54.79	5.0	even	ok
	204	C54H4N35NaO12P2S2	741.4791	-1.9	25.7	208	38.42	72.0	even	ok
	205	C65H144N8O22P2S	741.4790	-2.0	25.8	209	36.04	0.0	even	ok
	206	C67H136N12NaO20P	741.4809	0.5	25.8	210	55.99	7.0	even	ok
	207	C51H3N33NaO20PS	741.4789	-2.2	25.9	211	27.08	68.0	even	ok
	208	C58H138N12O30	741.4815	1.4	25.9	212	40.27	-3.0	even	ok

Compound Spectrum SmartFormula Report

Meas. m/z	#	Ion Formula	m/z	err [ppm]	mSigma	# mSigma	Score	rdb	e¯ Conf	N-Rule
	209	C53H136N24NaO16PS2	741.4816	1.5	25.9	213	46.82	-1.0	even	ok
	210	C56H20N15NaO29P2S	741.4817	1.6	25.9	214	43.96	56.0	even	ok
	211	C67H10N13NaO29	741.4797	-1.1	26.0	215	45.44	70.0	even	ok
	212	C62H26N3O38PS	741.4821	2.1	26.0	216	34.35	53.0	even	ok
	213	C63H18N9NaO33S	741.4807	0.3	26.1	217	72.63	60.0	even	ok
	214	C54H15N21O27P2S	741.4816	1.4	26.2	218	47.47	60.0	even	ok
	215	C52H6N29O24PS	741.4794	-1.5	26.2	219	37.85	66.0	even	ok
	216	C68H139N8O24P	741.4814	1.3	26.2	220	42.22	5.0	even	ok
	217	C52H130N28O17S2	741.4800	-0.7	26.5	221	61.37	3.0	even	ok
	218	C54H120N42P2S2	741.4793	-1.6	26.6	222	43.74	18.0	even	ok
	219	C64H19N11O28P2S	741.4793	-1.6	26.6	223	42.58	63.0	even	ok
	220	C56H132N28O10P2S2	741.4793	-1.6	26.6	224	43.80	7.0	even	ok
	221	C46H111N50NaO4S	741.4813	1.1	26.8	225	44.08	17.0	even	ok
	222	C57H133N22NaO17P2	741.4791	-2.0	26.9	226	30.14	4.0	even	ok
	223	C56H8N25NaO23P2	741.4793	-1.6	27.0	227	36.09	67.0	even	ok
	224	C57H141N18NaO16P2S2	741.4788	-2.3	27.0	228	30.75	-2.0	even	ok
	225	C58H22N9O36PS	741.4807	0.3	27.1	229	70.41	54.0	even	ok
	226	C57H115N42O2PS	741.4818	1.7	27.2	230	33.92	23.0	even	ok
	227	C55H131N26NaO15S2	741.4808	0.3	27.2	231	69.28	4.0	even	ok
	228	C61H120N32NaO8P	741.4796	-1.3	27.3	232	40.76	19.0	even	ok
	229	C49H3N37NaO16PS2	741.4812	0.9	27.3	233	56.01	68.0	even	ok
	230	C65H146N2O31S	741.4810	0.6	27.3	234	63.26	-5.0	even	ok
	231	C57H23N11O33P2S	741.4822	2.3	27.3	235	29.98	54.0	even	ok
	232	C59H18N9NaO38	741.4820	2.0	27.3	236	29.85	56.0	even	ok
	233	C70H18N3NaO35	741.4804	-0.2	27.4	237	72.77	64.0	even	ok
	234	C49H124N38O9P2S	741.4799	-0.8	27.5	238	49.14	9.0	even	ok
	235	C60H121N34NaO5P2	741.4811	0.7	27.6	239	50.13	19.0	even	ok
	236	C53H118N36O14	741.4809	0.5	27.7	240	54.76	14.0	even	ok
	237	C53H111N44NaO6	741.4810	0.7	27.7	241	51.38	21.0	even	ok
	238	C68H13N9O33	741.4802	-0.4	27.8	242	56.60	68.0	even	ok
	239	C57H135N16NaO26	741.4810	0.7	27.8	243	51.07	-1.0	even	ok
	240	C63H134N16O21S	741.4810	0.6	27.8	244	62.70	6.0	even	ok
	241	C54H122N36O9S2	741.4813	1.1	27.8	245	52.92	13.0	even	ok
	242	C70H144N2NaO26P	741.4816	1.4	27.8	246	37.59	1.0	even	ok
	243	C59H115N38O6P	741.4794	-1.5	27.8	247	37.15	23.0	even	ok
	244	C59H21N7O39S	741.4792	-1.7	27.8	248	40.03	54.0	even	ok
	245	C60H27N3NaO38PS	741.4809	0.5	27.8	249	65.03	50.0	even	ok
	246	C53H12N25NaO23P2S	741.4810	0.7	27.9	250	50.43	62.0	even	ok
	247	C66H130N16O21	741.4793	-1.7	27.9	251	33.89	11.0	even	ok
	248	C56H117N40NaO3P2	741.4797	-1.1	27.9	252	43.62	20.0	even	ok
	249	C62H9N19O27S	741.4812	1.0	28.1	253	53.79	69.0	even	ok
	250	C54H135N22NaO19S2	741.4801	-0.6	28.2	254	63.17	-1.0	even	ok
	251	C67H149N2NaO24P2S	741.4791	-1.8	28.2	255	37.35	-4.0	even	ok
	252	C62H7N25O18P2S	741.4793	-1.6	28.2	256	40.88	74.0	even	ok
	253	C55H121N36NaO7P2	741.4790	-2.0	28.3	257	29.03	15.0	even	ok
	254	C60H148N4O31P2	741.4796	-1.2	28.5	258	40.27	-9.0	even	ok

Compound Spectrum SmartFormula Report

Meas. m/z	#	Ion Formula	m/z	err [ppm]	mSigma	# mSigma	Score	rdb	e¯ Conf	N-Rule
	255	C54H107N48NaO2	741.4817	1.6	28.5	259	35.21	26.0	even	ok
	256	C50H2N35NaO19S2	741.4797	-1.1	28.5	260	51.66	68.0	even	ok
	257	C57H148N6NaO30PS	741.4792	-1.7	28.6	261	39.39	-12.0	even	ok
	258	C58H116N40O3P2	741.4809	0.5	28.6	262	52.25	23.0	even	ok
	259	C57H13N15O36	741.4818	1.8	28.7	263	31.59	60.0	even	ok
	260	C51H7N31O21P2S	741.4809	0.5	28.8	264	52.78	66.0	even	ok
	261	C59H120N36NaO4PS	741.4819	1.9	28.8	265	29.92	19.0	even	ok
	262	C56H125N36NaO2P2S2	741.4795	-1.4	28.9	266	44.93	14.0	even	ok
	263	C55H143N12O28PS	741.4791	-1.9	28.9	267	35.74	-8.0	even	ok
	264	C61H26NNaO41S	741.4794	-1.5	28.9	268	42.43	50.0	even	ok
	265	C54H139N20O20PS2	741.4821	2.2	29.0	270	31.01	-3.0	even	ok
	266	C44H106N56O2S	741.4812	0.9	29.0	271	45.08	21.0	even	ok
	267	C63H25NO41S	741.4806	0.1	29.0	272	71.82	53.0	even	ok
	268	C47H127N36O12PS2	741.4801	-0.5	29.0	273	62.28	4.0	even	ok
	269	C58H137N22NaO12P2S2	741.4795	-1.4	29.0	274	44.91	3.0	even	ok
	270	C66H24N5NaO30P2S	741.4794	-1.4	29.0	275	43.70	59.0	even	ok
	271	C50H4N35NaO17P2S	741.4804	-0.2	29.1	276	57.66	68.0	even	ok
	272	C49H132N30NaO14PS2	741.4803	-0.4	29.2	277	66.07	0.0	even	ok
	273	C54H11N27O20P2S2	741.4790	-2.1	29.2	278	32.35	65.0	even	ok
	274	C48H121N42NaO5P2S	741.4794	-1.5	29.3	279	35.18	11.0	even	ok
	275	C54H3N31O21P2	741.4792	-1.8	29.3	280	31.37	71.0	even	ok
	276	C68H135N10NaO23	741.4794	-1.5	29.3	281	35.69	7.0	even	ok
	277	C55H7N31O16P2S2	741.4796	-1.2	29.3	282	48.55	70.0	even	ok
	278	C55H128N28O15P2	741.4789	-2.2	29.4	283	25.87	8.0	even	ok
	279	C64H21N5O37S	741.4813	1.0	29.7	285	51.68	58.0	even	ok
	280	C58H151N2O34PS	741.4798	-1.0	29.8	286	51.90	-14.0	even	ok
	281	C56H10N19NaO32	741.4813	1.1	29.9	287	41.89	62.0	even	ok
	282	C50H6N33O20PS2	741.4817	1.7	29.9	288	38.90	66.0	even	ok
	283	C55H130N22O24	741.4809	0.5	29.9	289	51.80	3.0	even	ok
	284	C48H133N32NaO11P2S2	741.4817	1.7	30.0	290	38.74	0.0	even	ok
	285	C43H116N50NaO2PS2	741.4789	-2.2	30.1	291	25.33	12.0	even	ok
	286	C54H112N46OP2	741.4796	-1.3	30.1	292	38.39	24.0	even	ok
	287	C61H28NNaO39P2	741.4800	-0.7	30.1	293	48.39	50.0	even	ok
	288	C65H139N10NaO23S	741.4811	0.8	30.1	294	55.39	2.0	even	ok
	289	C51H106N50O4	741.4809	0.5	30.2	295	51.70	25.0	even	ok
	290	C56H127N30NaO11S2	741.4814	1.2	30.2	296	46.24	9.0	even	ok
	291	C59H145N8NaO27P2	741.4791	-2.0	30.2	297	27.92	-7.0	even	ok
	292	C46H128N38O9P2S2	741.4816	1.5	30.2	298	41.91	4.0	even	ok
	293	C46H124N40NaO8PS2	741.4796	-1.3	30.3	299	38.28	6.0	even	ok
	294	C57H111N44NaOS	741.4797	-1.0	30.3	300	41.92	25.0	even	ok
	295	C69H138N6O27	741.4799	-0.8	30.3	301	46.44	5.0	even	ok
	296	C58H112N42NaO2P	741.4789	-2.2	30.4	302	24.82	25.0	even	ok
	297	C64H12N19NaO20P2S	741.4794	-1.5	30.5	303	42.10	70.0	even	ok
	298	C52H102N54	741.4815	1.4	30.5	304	36.43	30.0	even	ok
	299	C64H14N13NaO29S	741.4814	1.2	30.6	305	47.06	65.0	even	ok
	300	C58H144N14O20P2S2	741.4794	-1.6	30.6	306	39.93	-4.0	even	ok

Compound Spectrum SmartFormula Report

Meas. m/z	#	Ion Formula	m/z	err [ppm]	mSigma	# mSigma	Score	rdb	e¯ Conf	N-Rule
	301	C44H119N46O6PS2	741.4794	-1.4	30.6	307	35.05	10.0	even	ok
	302	C53H116N42O5P2	741.4789	-2.2	30.9	308	24.86	19.0	even	ok
	303	C56H134N22O19S2	741.4813	1.1	30.9	309	48.97	2.0	even	ok
	304	C54H115N44NaOS2	741.4814	1.2	30.9	310	45.64	20.0	even	ok
	305	C54H6N29NaO21S2	741.4811	0.7	31.0	311	55.29	67.0	even	ok
	306	C57H152N4O31P2S	741.4813	1.0	31.1	312	49.43	-14.0	even	ok
	307	C52H11N27NaO22PS2	741.4819	1.9	31.1	313	34.63	62.0	even	ok
	308	C50H135N26O18PS2	741.4808	0.4	31.1	314	62.65	-2.0	even	ok
	309	C56H16N21NaO22P2S2	741.4791	-1.9	31.2	315	33.86	61.0	even	ok
	310	C52H115N40NaO10	741.4803	-0.2	31.2	316	54.20	16.0	even	ok
	311	C51H131N26NaO20S	741.4820	2.0	31.2	317	26.34	0.0	even	ok
	312	C65H2N25O20P	741.4817	1.6	31.3	318	31.68	79.0	even	ok
	313	C58H114N40O5S	741.4803	-0.3	31.4	319	52.86	23.0	even	ok
	314	C46H116N48O3P2S	741.4792	-1.7	31.4	320	30.71	15.0	even	ok
	315	C59H23N7O37P2	741.4799	-0.9	31.5	321	43.65	54.0	even	ok
	316	C55H138N18O23S2	741.4806	0.2	31.5	322	66.23	-3.0	even	ok
	317	C51H134N24O21S2	741.4793	-1.6	31.6	323	37.64	-2.0	even	ok
	318	C51H5N31O23S2	741.4803	-0.4	31.6	324	62.26	66.0	even	ok
	319	C57H12N25NaO18P2S2	741.4798	-1.0	31.7	325	49.42	66.0	even	ok
	320	C52H140N20NaO20PS2	741.4809	0.6	31.7	326	57.89	-6.0	even	ok
	321	C54H5N25O30	741.4812	0.9	31.7	327	43.07	66.0	even	ok
	322	C57H128N32O6P2S2	741.4800	-0.7	31.9	328	55.34	12.0	even	ok
	323	C71H143NaO29	741.4801	-0.6	32.2	329	47.44	1.0	even	ok
	324	C45H125N42NaO5P2S2	741.4811	0.8	32.3	330	44.35	6.0	even	ok
	325	C63H132N22O12P2S	741.4790	-2.0	32.3	331	25.63	11.0	even	ok
	326	C57H140N14O25P2	741.4789	-2.2	32.3	332	24.15	-3.0	even	ok
	327	C54H127N26NaO20	741.4803	-0.2	32.4	333	52.69	5.0	even	ok
	328	C55H2N33NaO17S2	741.4817	1.6	32.4	334	36.96	72.0	even	ok
	329	C67H27NO34P2S	741.4800	-0.7	32.4	335	53.53	57.0	even	ok
	330	C49H126N32O18S	741.4819	1.8	32.5	336	27.98	4.0	even	ok
	331	C53H139N18NaO23S2	741.4794	-1.5	32.5	337	39.93	-6.0	even	ok
	332	C58H20N11NaO33P2	741.4793	-1.6	32.6	338	31.62	56.0	even	ok
	333	C59H140N18O16P2S2	741.4800	-0.7	32.6	339	54.56	1.0	even	ok
	334	C67H7N19NaO22P	741.4819	1.8	32.7	340	28.05	75.0	even	ok
	335	C62H2N27NaO19S	741.4814	1.2	32.7	341	44.81	76.0	even	ok
	336	C60H149N8NaO22P2S2	741.4795	-1.4	32.8	342	41.07	-8.0	even	ok
	337	C53H15N19NaO30PS	741.4789	-2.2	32.9	343	27.50	57.0	even	ok
	338	C56H3N35O12P2S2	741.4803	-0.3	33.0	344	61.54	75.0	even	ok
	339	C43H120N48O3P2S2	741.4809	0.6	33.0	345	46.55	10.0	even	ok
	340	C66H142N6O27S	741.4816	1.5	33.1	346	38.51	0.0	even	ok
	341	C53H10N25NaO25S2	741.4804	-0.2	33.1	347	63.70	62.0	even	ok
	342	C58H139N16NaO21S2	741.4814	1.3	33.2	348	42.88	-2.0	even	ok
	343	C65H15N15O24P2S	741.4800	-0.7	33.2	349	52.41	68.0	even	ok
	344	C60H119N34NaO7S	741.4804	-0.1	33.2	350	53.55	19.0	even	ok
	345	C56H149N8NaO27P2S	741.4807	0.3	33.2	351	50.49	-12.0	even	ok
	346	C48H123N36NaO14S	741.4813	1.1	33.3	352	37.57	6.0	even	ok

Compound Spectrum SmartFormula Report

Meas. m/z	#	Ion Formula	m/z	err [ppm]	mSigma	# mSigma	Score	rdb	e⁻ Conf	N-Rule
	347	C57H143N12NaO25S2	741.4808	0.4	33.3	353	59.57	-7.0	even	ok
	348	C56H23N9NaO36PS	741.4795	-1.3	33.6	354	41.36	51.0	even	ok
	349	C53H2N29NaO26	741.4806	0.2	33.6	355	52.60	68.0	even	ok
	350	C57H130N26O15S2	741.4820	2.0	33.7	356	30.67	7.0	even	ok
	351	C54H18N15O34PS	741.4794	-1.5	33.7	357	37.97	55.0	even	ok
	352	C50H110N46O8	741.4802	-0.4	33.8	358	47.78	20.0	even	ok
	353	C55H118N40O5S2	741.4820	2.0	33.8	359	30.72	18.0	even	ok
	354	C66H140N12O18P2S	741.4797	-1.1	33.8	360	44.43	5.0	even	ok
	355	C59H153N4NaO26P2S2	741.4788	-2.3	33.9	361	26.11	-13.0	even	ok
	356	C65H17N9O33S	741.4819	1.9	33.9	362	31.49	63.0	even	ok
	357	C54H144N14O25P2S	741.4806	0.1	34.0	364	52.54	-8.0	even	ok
	358	C53H141N18NaO21P2S	741.4801	-0.6	34.0	365	44.86	-6.0	even	ok
	359	C53H143N16O24PS2	741.4815	1.3	34.2	366	41.38	-8.0	even	ok
	360	C50H103N54Na	741.4803	-0.3	34.2	367	50.18	27.0	even	ok
	361	C46H2N39O18PS2	741.4804	-0.1	34.2	369	62.15	67.0	even	ok
	362	C59H147N2NaO36	741.4810	0.7	34.3	370	43.37	-12.0	even	ok
	363	C56H15N17O31P2	741.4792	-1.8	34.4	371	27.74	60.0	even	ok
	364	C59H133N26NaO8P2S2	741.4802	-0.5	34.4	372	55.55	8.0	even	ok
	365	C65H137N16NaO14P2S	741.4791	-1.8	34.5	373	26.53	7.0	even	ok
	366	C48H7N33NaO20PS2	741.4805	0.0	34.5	374	63.74	63.0	even	ok
	367	C49H6N31NaO23S2	741.4790	-2.0	34.6	375	29.89	63.0	even	ok
	368	C55H9N25O25S2	741.4816	1.5	34.6	376	37.88	65.0	even	ok
	369	C57H19N17O26P2S2	741.4796	-1.2	34.7	377	42.57	59.0	even	ok
	370	C52H122N32O18	741.4802	-0.4	34.8	378	46.69	9.0	even	ok
	371	C46H118N42O12S	741.4812	0.9	34.9	379	38.79	10.0	even	ok
	372	C57H26N5O40PS	741.4801	-0.6	35.0	380	52.61	49.0	even	ok
	373	C61H122N30O11S	741.4810	0.6	35.0	381	43.81	17.0	even	ok
	374	C61H145N12NaO18P2S2	741.4802	-0.5	35.1	382	54.72	-3.0	even	ok
	375	C55H24N11NaO33P2S	741.4810	0.7	35.1	383	50.56	51.0	even	ok
	376	C55H148N10NaO26PS2	741.4816	1.5	35.1	385	37.24	-12.0	even	ok
	377	C47H8N35NaO17P2S2	741.4820	2.1	35.2	386	28.28	63.0	even	ok
	378	C45H3N41O15P2S2	741.4819	1.9	35.2	387	30.94	67.0	even	ok
	379	C51H136N24O19P2S	741.4799	-0.8	35.3	388	40.62	-2.0	even	ok
	380	C63H5N23O23S	741.4819	1.9	35.3	389	30.49	74.0	even	ok
	381	C58H15N21O22P2S2	741.4803	-0.3	35.3	390	58.09	64.0	even	ok
	382	C54H142N14O27S2	741.4800	-0.7	35.4	391	49.51	-8.0	even	ok
	383	C71H19N5O30P2	741.4790	-2.1	35.4	392	22.92	67.0	even	ok
	384	C58H8N29NaO14P2S2	741.4804	-0.1	35.5	393	61.23	71.0	even	ok
	385	C68H147NaO29S	741.4818	1.7	35.5	394	33.23	-4.0	even	ok
	386	C67H20N9NaO26P2S	741.4801	-0.5	35.6	395	52.70	64.0	even	ok
	387	C63H119N32O8P	741.4808	0.3	35.6	396	47.08	22.0	even	ok
	388	C53H19N17O31P2S	741.4809	0.5	35.6	397	53.32	55.0	even	ok
	389	C66H127N22O14P	741.4814	1.2	35.8	398	33.49	16.0	even	ok
	390	C61H117N38NaOP2	741.4817	1.6	35.8	399	28.30	24.0	even	ok
	391	C58H32NNaO39P2S	741.4817	1.6	35.9	400	34.25	45.0	even	ok
	392	C57H142N8O34	741.4809	0.5	35.9	401	44.40	-8.0	even	ok

Compound Spectrum SmartFormula Report

Meas. m/z	#	Ion Formula	m/z	err [ppm]	mSigma	# mSigma	Score	rdb	e¯ Conf	N-Rule
	393	C50H133N28NaO15P2S	741.4794	-1.5	36.0	402	29.78	0.0	even	ok
	394	C68H145N6NaO20P2S	741.4798	-0.9	36.0	403	45.16	1.0	even	ok
	395	C56H27N7O37P2S	741.4816	1.4	36.0	404	37.12	49.0	even	ok
	396	C65H124N26NaO10P	741.4809	0.5	36.1	405	43.57	18.0	even	ok
	397	C64H130N20O17S	741.4816	1.5	36.2	406	35.67	11.0	even	ok
	398	C59H135N20NaO17S2	741.4821	2.2	36.2	407	26.19	3.0	even	ok
	399	C57H123N34NaO7S2	741.4821	2.1	36.2	408	26.27	14.0	even	ok
	400	C51H116N42NaO7P	741.4818	1.8	36.3	409	26.27	16.0	even	ok
	401	C54H13N21O29S2	741.4809	0.6	36.3	410	51.51	60.0	even	ok
	402	C45H115N46NaO8S	741.4807	0.2	36.4	411	47.98	12.0	even	ok
	403	C67H22N3NaO35S	741.4821	2.1	36.4	412	26.92	59.0	even	ok
	404	C61H152N4O26P2S2	741.4800	-0.7	36.5	413	49.51	-10.0	even	ok
	405	C49H10N29O24PS2	741.4811	0.8	36.5	414	47.63	61.0	even	ok
	406	C56H147N8NaO29S2	741.4801	-0.5	36.7	415	51.18	-12.0	even	ok
	407	C60H111N42O2P	741.4801	-0.6	36.7	417	42.29	28.0	even	ok
	408	C62H116N36NaO4P	741.4802	-0.4	36.7	418	45.06	24.0	even	ok
	409	C59H24N11NaO28P2S2	741.4798	-1.0	36.8	419	43.50	55.0	even	ok
	410	C57H14N19NaO27S2	741.4817	1.6	36.8	420	32.95	61.0	even	ok
	411	C68H6N17NaO25	741.4804	-0.2	36.8	421	47.86	75.0	even	ok
	412	C59H142N12O25S2	741.4820	2.0	36.8	422	28.18	-4.0	even	ok
	413	C68H132N16NaO16P	741.4816	1.4	36.8	423	30.07	12.0	even	ok
	414	C58H146N8O29S2	741.4813	1.1	36.9	424	41.94	-9.0	even	ok
	415	C50H9N27O27S2	741.4796	-1.3	37.0	425	38.81	61.0	even	ok
	416	C45H128N36NaO12PS2	741.4789	-2.2	37.0	426	25.56	1.0	even	ok
	417	C63H3N29O14P2S	741.4800	-0.7	37.1	427	47.23	79.0	even	ok
	418	C51H15N23NaO26PS2	741.4812	1.0	37.2	428	43.63	57.0	even	ok
	419	C56H23N13O30P2S2	741.4790	-2.1	37.2	429	26.56	54.0	even	ok
	420	C63H127N24NaO13S	741.4811	0.8	37.2	430	38.65	13.0	even	ok
	421	C60H156O30P2S2	741.4794	-1.6	37.3	431	33.81	-15.0	even	ok
	422	C69H135N12O20P	741.4821	2.2	37.3	432	21.18	10.0	even	ok
	423	C56H139N12NaO30	741.4803	-0.2	37.4	433	46.45	-6.0	even	ok
	424	C49H107N50NaO4	741.4797	-1.2	37.4	434	33.32	22.0	even	ok
	425	C53H128N28NaO17P	741.4818	1.8	37.5	435	25.40	5.0	even	ok
	426	C53H143N12NaO30S	741.4820	2.0	37.6	436	26.79	-11.0	even	ok
	427	C60H136N22O12P2S2	741.4807	0.2	37.6	437	55.36	6.0	even	ok
	428	C51H119N36NaO14	741.4797	-1.1	37.6	438	33.21	11.0	even	ok
	429	C48H128N34O13P2S	741.4793	-1.7	37.6	439	26.23	4.0	even	ok
	430	C65H10N17NaO25S	741.4821	2.1	37.6	440	26.13	70.0	even	ok
	431	C59H25NO46	741.4818	1.8	37.7	441	25.12	49.0	even	ok
	432	C60H20N15NaO24P2S2	741.4804	-0.1	37.7	442	57.87	60.0	even	ok
	433	C48H136N26NaO18PS2	741.4796	-1.3	37.8	443	38.09	-5.0	even	ok
	434	C46H131N32O16PS2	741.4794	-1.4	37.9	444	35.02	-1.0	even	ok
	435	C64H118N30O11	741.4793	-1.7	37.9	445	26.26	22.0	even	ok
	436	C56H151N6O30PS2	741.4821	2.2	38.0	446	24.61	-14.0	even	ok
	437	C52H14N21NaO29S2	741.4797	-1.1	38.0	447	40.84	57.0	even	ok
	438	C58H22N5NaO42	741.4813	1.1	38.0	448	34.02	51.0	even	ok

Compound Spectrum SmartFormula Report

Meas. m/z	#	Ion Formula	m/z	err [ppm]	mSigma	# mSigma	Score	rdb	e⁻ Conf	N-Rule
	439	C57H2N33O15PS2	741.4788	-2.3	38.0	449	23.24	75.0	even	ok
	440	C56H18N15NaO31S2	741.4811	0.7	38.0	450	46.05	56.0	even	ok
	441	C69H9N13O29	741.4809	0.5	38.3	451	41.12	73.0	even	ok
	442	C43H110N52O6S	741.4805	0.0	38.3	452	48.28	16.0	even	ok
	443	C66H135N14NaO19S	741.4818	1.7	38.4	453	30.92	7.0	even	ok
	444	C52H3N31NaO23P	741.4821	2.2	38.4	454	20.49	68.0	even	ok
	445	C52H16N21NaO27P2S	741.4804	-0.2	38.5	456	45.44	57.0	even	ok
	446	C68H23N5O30P2S	741.4806	0.2	38.6	457	55.04	62.0	even	ok
	447	C69H148N2O24P2S	741.4804	-0.2	38.6	458	54.15	-1.0	even	ok
	448	C59H152O35P2	741.4789	-2.1	38.7	459	20.56	-14.0	even	ok
	449	C62H148N8O22P2S2	741.4807	0.2	38.7	460	53.67	-5.0	even	ok
	450	C72H17N3O35	741.4816	1.4	38.7	461	34.30	67.0	even	ok
	451	C58H28N7NaO32P2S2	741.4791	-1.9	38.8	462	27.93	50.0	even	ok
	452	C60H151N2NaO31S2	741.4814	1.3	38.8	463	36.86	-13.0	even	ok
	453	C71H140N6NaO22P	741.4823	2.3	38.9	464	18.44	6.0	even	ok
	454	C66H123N24NaO13	741.4794	-1.5	38.9	465	27.86	18.0	even	ok
	455	C49H111N48O5P	741.4817	1.6	38.9	466	26.73	20.0	even	ok
	456	C59H11N25O18P2S2	741.4810	0.6	39.0	467	46.82	69.0	even	ok
	457	C67H126N20O17	741.4799	-0.8	39.1	468	36.99	16.0	even	ok
	458	C61H147N6NaO27S2	741.4821	2.2	39.2	469	24.11	-8.0	even	ok
	459	C49H139N22O22PS2	741.4801	-0.5	39.2	470	48.14	-7.0	even	ok
	460	C65H8N23NaO16P2S	741.4801	-0.6	39.2	471	47.73	75.0	even	ok
	461	C47H137N28NaO15P2S2	741.4811	0.8	39.2	472	44.28	-5.0	even	ok
	462	C56H17N11O40	741.4812	0.9	39.3	473	35.29	55.0	even	ok
	463	C54H134N18O28	741.4802	-0.4	39.3	474	41.49	-2.0	even	ok
	464	C51H144N16NaO24PS2	741.4803	-0.3	39.4	475	51.00	-11.0	even	ok
	465	C50H11N27O25P2S	741.4802	-0.4	39.5	476	41.67	61.0	even	ok
	466	C58H124N36O2P2S2	741.4807	0.2	39.5	477	52.76	17.0	even	ok
	467	C61H120N36O2P2S	741.4790	-2.0	39.5	478	21.15	22.0	even	ok
	468	C52H18N19O30PS2	741.4818	1.7	39.6	479	30.21	55.0	even	ok
	469	C45H132N34O13P2S2	741.4809	0.6	39.7	480	46.79	-1.0	even	ok
	470	C49H8N31NaO21P2S	741.4797	-1.1	39.7	481	31.80	63.0	even	ok
	471	C57H150N4O33S2	741.4806	0.2	39.8	482	53.33	-14.0	even	ok
	472	C59H14N19O25PS2	741.4788	-2.3	39.8	483	22.18	64.0	even	ok
	473	C51H123N34O15P	741.4817	1.6	39.8	484	25.97	9.0	even	ok
	474	C47H102N56O2	741.4795	-1.3	40.0	485	28.74	26.0	even	ok
	475	C49H114N42O12	741.4795	-1.3	40.1	486	28.75	15.0	even	ok
	476	C55H153N4NaO31P2S	741.4801	-0.6	40.1	487	45.99	-17.0	even	ok
	477	C62H141N16NaO14P2S2	741.4808	0.4	40.1	488	48.65	2.0	even	ok
	478	C50H145N18NaO21P2S2	741.4818	1.7	40.1	489	29.74	-11.0	even	ok
	479	C55H14N15NaO36	741.4806	0.2	40.2	490	44.07	57.0	even	ok
	480	C48H140N24O19P2S2	741.4816	1.5	40.2	491	32.27	-7.0	even	ok
	481	C71H14N7NaO31	741.4810	0.7	40.3	492	36.40	69.0	even	ok
	482	C42H107N56NaO2S	741.4800	-0.7	40.3	493	36.74	18.0	even	ok
	483	C60H27N7O32P2S2	741.4803	-0.3	40.4	494	50.85	53.0	even	ok
	484	C69H131N14NaO19	741.4801	-0.6	40.5	495	38.09	12.0	even	ok

Compound Spectrum SmartFormula Report

Meas. m/z	#	Ion Formula	m/z	err [ppm]	mSigma	# mSigma	Score	rdb	e⁻ Conf	N-Rule
	485	C59H7N27NaO17PS2	741.4789	-2.1	40.5	496	23.90	71.0	even	ok
	486	C56H156O35P2S	741.4806	0.1	40.5	497	52.91	-19.0	even	ok
	487	C54H23N13NaO32PS2	741.4819	1.9	40.6	498	26.96	51.0	even	ok
	488	C53H17N17O33S2	741.4803	-0.3	40.7	499	49.11	55.0	even	ok
	489	C40H114N52O6S2	741.4822	2.3	40.9	500	17.87	11.0	even	ok
	490	C44H116N48NaO5PS	741.4822	2.2	40.9	501	18.49	12.0	even	ok
	491	C39H111N56NaO2S2	741.4817	1.6	40.9	502	25.29	13.0	even	ok
	492	C47H3N37O19P2S	741.4795	-1.3	41.1	503	28.38	67.0	even	ok
	493	C43H123N42O10PS2	741.4788	-2.3	41.1	504	17.32	5.0	even	ok
	494	C64H153N2NaO24P2S2	741.4808	0.4	41.2	505	47.16	-9.0	even	ok
	495	C53H131N22NaO24	741.4797	-1.1	41.3	506	30.16	0.0	even	ok
	496	C52H147N12O28PS2	741.4808	0.4	41.3	507	47.87	-13.0	even	ok
	497	C63H125N30NaO4P2S	741.4791	-1.9	41.3	508	22.06	18.0	even	ok
	498	C70H134N10O23	741.4806	0.1	41.4	509	42.87	10.0	even	ok
	499	C61H23N11O28P2S2	741.4810	0.6	41.4	510	43.66	58.0	even	ok
	500	C57H21N11O35S2	741.4816	1.5	41.5	511	31.45	54.0	even	ok
763.4936	1	C65H24N9NaO32P2	763.4945	1.1	16.6	1	60.79	60.0	even	ok
	2	C63H15N17NaO29P	763.4923	-1.8	16.8	2	44.70	66.0	even	ok
	3	C64H18N13O33P	763.4928	-1.1	16.8	3	61.08	64.0	even	ok
	4	C63H19N15O30P2	763.4943	0.9	16.8	4	65.11	64.0	even	ok
	5	C66H27N5O36P2	763.4950	1.8	17.1	6	43.93	58.0	even	ok
	6	C67H152N2O30P2	763.4947	1.4	17.1	7	52.41	-5.0	even	ok
	7	C55H128N34NaO11PS	763.4923	-1.7	17.1	8	45.69	10.0	even	ok
	8	C61H10N23O27P	763.4922	-1.9	17.2	9	40.48	70.0	even	ok
	9	C67H26N3O39P	763.4935	-0.2	17.3	10	100.00	58.0	even	ok
	10	C66H23N7NaO35P	763.4930	-0.9	17.4	11	64.86	60.0	even	ok
	11	C53H123N40O9PS	763.4922	-1.9	17.6	12	41.42	14.0	even	ok
	12	C66H149N6NaO26P2	763.4942	0.7	17.6	13	69.10	-3.0	even	ok
	13	C56H131N30O15PS	763.4929	-1.0	17.6	14	61.60	8.0	even	ok
	14	C60H145N16NaO20P2S	763.4952	2.0	17.7	15	46.07	-2.0	even	ok
	15	C57H137N26NaO14P2S	763.4945	1.1	17.7	16	57.86	4.0	even	ok
	16	C56H128N36O8P2S	763.4950	1.8	17.8	17	42.16	13.0	even	ok
	17	C62H16N19NaO26P2	763.4938	0.2	17.9	18	81.78	66.0	even	ok
	18	C65H143N10O27P	763.4925	-1.4	17.9	19	50.70	1.0	even	ok
	19	C55H132N32O12P2S	763.4944	1.0	17.9	20	62.05	8.0	even	ok
	20	C64H144N12O24P2	763.4940	0.5	18.1	21	73.07	1.0	even	ok
	21	C55H125N40NaO4P2S	763.4945	1.1	18.1	22	57.49	15.0	even	ok
	22	C58H136N24NaO17PS	763.4930	-0.8	18.2	23	65.39	4.0	even	ok
	23	C67H148N4NaO29P	763.4927	-1.3	18.2	24	54.59	-3.0	even	ok
	24	C59H139N20O21PS	763.4936	-0.1	18.3	25	99.96	2.0	even	ok
	25	C58H133N30NaO10P2S	763.4952	2.0	18.3	26	37.99	9.0	even	ok
	26	C55H6N33O21PS	763.4932	-0.6	18.4	27	84.03	71.0	even	ok
	27	C54H129N36NaO8P2S	763.4938	0.3	18.5	28	79.02	10.0	even	ok
	28	C53H120N46O2P2S	763.4944	1.0	18.6	29	61.41	19.0	even	ok
	29	C54H119N44O5PS	763.4929	-1.0	18.6	30	60.14	19.0	even	ok
	30	C58H140N22O18P2S	763.4950	1.8	18.6	31	41.29	2.0	even	ok

Compound Spectrum SmartFormula Report

Meas. m/z	#	Ion Formula	m/z	err [ppm]	mSigma	# mSigma	Score	rdb	e¯ Conf	N-Rule
	31	C65H153N2NaO30P2	763.4935	-0.2	18.6	32	81.12	-8.0	even	ok
	32	C68H151O33P	763.4932	-0.6	18.8	33	71.00	-5.0	even	ok
	33	C65H31NO40P2	763.4943	0.9	18.8	34	74.74	53.0	even	ok
	34	C54H3N37NaO17PS	763.4926	-1.3	18.9	35	52.39	73.0	even	ok
	35	C56H124N38NaO7PS	763.4930	-0.8	19.0	36	64.10	15.0	even	ok
	36	C65H27N3NaO39P	763.4923	-1.8	19.0	37	42.80	55.0	even	ok
	37	C60H11N25O24P2	763.4937	0.0	19.0	38	84.51	70.0	even	ok
	38	C57H11N27NaO23PS	763.4933	-0.4	19.1	39	88.40	67.0	even	ok
	39	C63H22N9O37P	763.4922	-1.9	19.2	40	38.94	59.0	even	ok
	40	C58H7N31NaO19PS	763.4940	0.4	19.2	41	87.94	72.0	even	ok
	41	C64H15N19O26P2	763.4950	1.8	19.3	42	42.05	69.0	even	ok
	42	C53H116N48NaOPS	763.4923	-1.7	19.4	43	43.42	21.0	even	ok
	43	C61H144N14NaO23PS	763.4937	0.1	19.5	44	98.80	-2.0	even	ok
	44	C56H2N37O17PS	763.4938	0.3	19.5	45	77.47	76.0	even	ok
	45	C57H127N34O11PS	763.4935	-0.1	19.6	46	80.80	13.0	even	ok
	46	C52H124N42O6P2S	763.4937	0.1	19.7	47	81.80	14.0	even	ok
	47	C63H148N8O28P2	763.4934	-0.4	19.9	48	74.43	-4.0	even	ok
	48	C66H20N13NaO28P2	763.4951	2.0	19.9	49	37.94	65.0	even	ok
	49	C50H120N48NaOPS2	763.4940	0.5	19.9	50	84.81	16.0	even	ok
	50	C63H141N16NaO20P2	763.4935	-0.2	20.0	51	78.71	3.0	even	ok
	51	C53H4N39NaO14P2S	763.4941	0.6	20.0	52	67.09	73.0	even	ok
	52	C63H12N23NaO22P2	763.4945	1.1	20.1	53	56.63	71.0	even	ok
	53	C54H7N35O18P2S	763.4947	1.3	20.3	54	50.33	71.0	even	ok
	54	C64H28N5NaO36P2	763.4938	0.2	20.3	55	77.47	55.0	even	ok
	55	C56H12N29NaO20P2S	763.4948	1.5	20.4	56	46.31	67.0	even	ok
	56	C61H7N29O20P2	763.4943	0.9	20.4	57	60.47	75.0	even	ok
	57	C59H10N23NaO29	763.4951	1.9	20.6	58	38.90	67.0	even	ok
	58	C49H111N54NaOS	763.4951	1.9	20.6	59	37.80	22.0	even	ok
	59	C60H135N20NaO23	763.4948	1.5	20.7	60	46.53	4.0	even	ok
	60	C62H6N27O23P	763.4928	-1.1	20.8	61	56.00	75.0	even	ok
	61	C60H135N24O17PS	763.4942	0.8	20.8	62	75.80	7.0	even	ok
	62	C59H132N28NaO13PS	763.4937	0.1	20.9	64	80.14	9.0	even	ok
	63	C64H11N21NaO25P	763.4930	-0.9	21.1	65	59.78	71.0	even	ok
	64	C62H23N11O34P2	763.4937	0.0	21.2	66	80.54	59.0	even	ok
	65	C58H14N23O27PS	763.4938	0.3	21.2	67	89.46	65.0	even	ok
	66	C49H114N52O2S2	763.4924	-1.6	21.2	68	51.72	20.0	even	ok
	67	C61H136N22O18P2	763.4934	-0.4	21.3	69	72.01	7.0	even	ok
	68	C59H8N29NaO20P2	763.4931	-0.7	21.3	70	64.23	72.0	even	ok
	69	C65H14N17O29P	763.4935	-0.2	21.4	71	76.31	69.0	even	ok
	70	C58H143N16O25PS	763.4929	-1.0	21.4	72	68.39	-3.0	even	ok
	71	C59H10N27O23PS	763.4945	1.1	21.5	73	64.08	70.0	even	ok
	72	C51H121N46NaO2P2S	763.4932	-0.6	21.5	74	65.40	16.0	even	ok
	73	C62H145N12NaO24P2	763.4928	-1.1	21.7	75	55.04	-2.0	even	ok
	74	C49H124N44NaO5PS2	763.4934	-0.4	21.8	76	85.27	11.0	even	ok
	75	C61H3N31NaO19P	763.4923	-1.8	21.9	77	39.99	77.0	even	ok
	76	C59H149N12NaO24P2S	763.4945	1.2	21.9	78	63.36	-7.0	even	ok

Compound Spectrum SmartFormula Report

Meas. m/z	#	Ion Formula	m/z	err [ppm]	mSigma	# mSigma	Score	rdb	e⁻ Conf	N-Rule
	77	C60H148N10NaO27PS	763.4930	-0.8	21.9	79	72.66	-7.0	even	ok
	78	C68H22N7O35P	763.4942	0.7	21.9	80	75.69	63.0	even	ok
	79	C62H147N10O27PS	763.4942	0.8	22.0	81	73.56	-4.0	even	ok
	80	C57H140N20NaO21PS	763.4923	-1.7	22.0	82	41.30	-1.0	even	ok
	81	C57H5N29O27	763.4949	1.7	22.0	83	41.17	71.0	even	ok
	82	C61H20N15NaO30P2	763.4931	-0.7	22.2	84	63.15	61.0	even	ok
	83	C55H135N26O19PS	763.4922	-1.9	22.2	85	37.63	3.0	even	ok
	84	C58H130N26O21	763.4946	1.3	22.4	86	48.63	8.0	even	ok
	85	C51H123N44O5PS2	763.4946	1.2	22.4	87	61.25	14.0	even	ok
	86	C60H4N33NaO16P2	763.4938	0.2	22.4	88	74.34	77.0	even	ok
	87	C62H143N12NaO26S	763.4922	-1.9	22.4	89	44.57	-2.0	even	ok
	88	C62H140N18NaO19PS	763.4944	0.9	22.5	90	68.03	3.0	even	ok
	89	C60H19N17NaO29PS	763.4940	0.4	22.5	91	81.77	61.0	even	ok
	90	C67H19N11NaO31P	763.4936	-0.0	22.5	92	78.73	65.0	even	ok
	91	C59H6N29NaO22S	763.4925	-1.5	22.6	93	53.10	72.0	even	ok
	92	C51H123N40NaO11S	763.4951	1.9	22.8	94	35.92	11.0	even	ok
	93	C65H140N16O20P2	763.4947	1.4	22.8	95	46.54	6.0	even	ok
	94	C51H119N46NaO4S2	763.4925	-1.5	22.9	96	54.20	16.0	even	ok
	95	C67H145N10NaO22P2	763.4948	1.6	23.1	97	42.59	2.0	even	ok
	96	C57H3N35O18P2	763.4930	-0.9	23.1	98	57.61	76.0	even	ok
	97	C47H119N50O3PS2	763.4932	-0.6	23.2	99	64.61	15.0	even	ok
	98	C62H147N6NaO33	763.4948	1.5	23.2	100	43.83	-7.0	even	ok
	99	C60H152N8O28P2S	763.4951	1.9	23.3	101	44.62	-9.0	even	ok
	100	C49H116N52P2S	763.4930	-0.8	23.3	102	58.75	20.0	even	ok
	101	C61H15N21NaO25PS	763.4946	1.3	23.4	103	56.92	66.0	even	ok
	102	C56H141N22NaO18P2S	763.4938	0.3	23.4	104	70.90	-1.0	even	ok
	103	C59H13N21O30S	763.4923	-1.7	23.5	105	47.94	65.0	even	ok
	104	C70H27NNaO37P	763.4943	0.9	23.5	106	68.06	59.0	even	ok
	105	C60H140N18O22P2	763.4927	-1.2	23.5	107	48.98	2.0	even	ok
	106	C46H120N52P2S2	763.4947	1.4	23.6	108	45.59	15.0	even	ok
	107	C48H125N46NaO2P2S2	763.4948	1.6	23.6	109	41.91	11.0	even	ok
	108	C64H152N4NaO29PS	763.4944	1.0	23.7	110	65.96	-8.0	even	ok
	109	C59H15N21O28P2	763.4930	-0.9	23.8	111	56.93	65.0	even	ok
	110	C50H127N40O9PS2	763.4939	0.3	23.9	112	82.61	9.0	even	ok
	111	C68H18N9NaO34	763.4921	-2.0	23.9	113	41.65	65.0	even	ok
	112	C57H144N18O22P2S	763.4944	1.0	24.0	114	54.32	-3.0	even	ok
	113	C61H151N6O31PS	763.4936	-0.1	24.0	115	88.53	-9.0	even	ok
	114	C54H10N29O25PS	763.4925	-1.5	24.0	116	52.01	66.0	even	ok
	115	C56H121N44NaP2S	763.4952	2.0	24.0	117	33.69	20.0	even	ok
	116	C56H15N23NaO27PS	763.4926	-1.3	24.0	118	56.39	62.0	even	ok
	117	C60H133N26NaO14P2	763.4928	-1.1	24.1	119	52.11	9.0	even	ok
	118	C56H2N33NaO23	763.4944	1.0	24.1	120	53.48	73.0	even	ok
	119	C53H128N38NaO7PS2	763.4947	1.4	24.1	121	54.42	10.0	even	ok
	120	C49H118N46O9S	763.4950	1.8	24.1	122	38.15	15.0	even	ok
	121	C64H137N20NaO16P2	763.4942	0.7	24.2	123	60.08	8.0	even	ok
	122	C54H136N28O16P2S	763.4937	0.1	24.2	124	73.92	3.0	even	ok

Compound Spectrum SmartFormula Report

Meas. m/z	#	Ion Formula	m/z	err [ppm]	mSigma	# mSigma	Score	rdb	e⁻ Conf	N-Rule
	123	C58H123N34NaO13	763.4948	1.5	24.3	125	43.07	15.0	even	ok
	124	C66H139N14O23P	763.4932	-0.6	24.4	126	62.65	6.0	even	ok
	125	C61H134N22O20S	763.4927	-1.2	24.5	127	58.58	7.0	even	ok
	126	C60H131N26NaO16S	763.4922	-1.9	24.5	128	35.35	9.0	even	ok
	127	C60H142N12O31	763.4946	1.3	24.6	129	46.10	-3.0	even	ok
	128	C62H132N26O14P2	763.4940	0.5	24.7	130	63.44	12.0	even	ok
	129	C53H133N32NaO12P2S	763.4932	-0.6	24.8	131	60.98	5.0	even	ok
	130	C63H131N24O17P	763.4925	-1.4	24.8	132	43.47	12.0	even	ok
	131	C63H143N14O23PS	763.4949	1.6	24.9	133	47.58	1.0	even	ok
	132	C65H136N18NaO19P	763.4927	-1.3	24.9	134	47.02	8.0	even	ok
	133	C52H132N34NaO11PS2	763.4940	0.5	25.0	135	75.61	5.0	even	ok
	134	C55H16N25NaO24P2S	763.4941	0.6	25.0	136	71.92	62.0	even	ok
	135	C63H156NaO33PS	763.4937	0.1	25.0	137	87.33	-13.0	even	ok
	136	C57H127N30NaO17	763.4941	0.6	25.1	138	60.48	10.0	even	ok
	137	C61H18N15NaO32S	763.4925	-1.5	25.1	139	50.29	61.0	even	ok
	138	C61H22N13O33PS	763.4945	1.1	25.2	140	58.82	59.0	even	ok
	139	C60H9N25O26S	763.4930	-0.8	25.2	141	67.15	70.0	even	ok
	140	C68H144N8NaO25P	763.4933	-0.4	25.3	142	65.52	2.0	even	ok
	141	C63H146N8O30S	763.4927	-1.2	25.3	143	57.69	-4.0	even	ok
	142	C55H115N48OPS	763.4935	-0.1	25.4	144	70.88	24.0	even	ok
	143	C57H18N19O31PS	763.4932	-0.6	25.6	145	71.95	60.0	even	ok
	144	C69H21N5O38	763.4927	-1.3	25.7	146	55.51	63.0	even	ok
	145	C51H126N38O12S2	763.4924	-1.6	25.7	148	46.93	9.0	even	ok
	146	C48H115N50NaO5S	763.4944	1.1	25.7	149	50.26	17.0	even	ok
	147	C58H12N25NaO24P2	763.4924	-1.6	25.7	150	40.42	67.0	even	ok
	148	C52H122N42O8S2	763.4931	-0.8	25.8	151	67.93	14.0	even	ok
	149	C58H128N32O12P2	763.4927	-1.2	25.9	152	46.31	13.0	even	ok
	150	C69H147N4O29P	763.4939	0.3	25.9	153	65.94	0.0	even	ok
	151	C56H118N40O11	763.4946	1.3	25.9	154	45.01	19.0	even	ok
	152	C56H19N21O28P2S	763.4947	1.3	25.9	155	53.11	60.0	even	ok
	153	C58H24N15NaO30P2S	763.4948	1.5	26.1	156	48.77	56.0	even	ok
	154	C61H22N9NaO39	763.4951	1.9	26.1	157	34.27	56.0	even	ok
	155	C51H128N38O10P2S	763.4930	-0.8	26.2	159	55.17	9.0	even	ok
	156	C59H23N13NaO33PS	763.4933	-0.4	26.2	160	75.73	56.0	even	ok
	157	C62H18N17O29PS	763.4952	2.0	26.2	161	38.26	64.0	even	ok
	158	C59H137N22NaO18P2	763.4922	-1.9	26.2	162	33.27	4.0	even	ok
	159	C59H139N16NaO27	763.4941	0.6	26.2	163	58.80	-1.0	even	ok
	160	C57H120N42NaO3PS	763.4937	0.1	26.3	164	71.10	20.0	even	ok
	161	C63H139N16NaO22S	763.4929	-1.0	26.4	165	60.42	3.0	even	ok
	162	C60H6N31O19PS	763.4952	2.0	26.5	166	38.14	75.0	even	ok
	163	C65H155O33PS	763.4949	1.7	26.7	167	45.45	-10.0	even	ok
	164	C62H152N4O32P2	763.4927	-1.2	26.8	168	45.64	-9.0	even	ok
	165	C59H3N35NaO15PS	763.4946	1.3	26.8	169	44.01	77.0	even	ok
	166	C65H148N8NaO25PS	763.4950	1.8	26.9	170	41.58	-3.0	even	ok
	167	C63H27N7NaO35PS	763.4947	1.3	26.9	171	52.34	55.0	even	ok
	168	C58H123N38O7PS	763.4942	0.8	27.0	172	55.07	18.0	even	ok

Compound Spectrum SmartFormula Report

Meas. m/z	#	Ion Formula	m/z	err [ppm]	mSigma	# mSigma	Score	rdb	e⁻ Conf	N-Rule
	169	C61H129N30NaO10P2	763.4935	-0.2	27.0	173	67.09	14.0	even	ok
	170	C52H3N41NaO13PS2	763.4950	1.8	27.1	174	42.59	73.0	even	ok
	171	C62H3N33O16P2	763.4950	1.8	27.1	175	35.46	80.0	even	ok
	172	C59H17N15O37	763.4949	1.7	27.1	176	36.57	60.0	even	ok
	173	C55H122N36O15	763.4940	0.4	27.2	177	61.49	14.0	even	ok
	174	C53H131N32NaO14S2	763.4925	-1.4	27.2	178	49.30	5.0	even	ok
	175	C65H151N2NaO32S	763.4929	-1.0	27.3	179	59.44	-8.0	even	ok
	176	C52H115N50NaS2	763.4932	-0.6	27.3	180	70.01	21.0	even	ok
	177	C62H14N19NaO28S	763.4932	-0.6	27.3	181	68.58	66.0	even	ok
	178	C64H8N27NaO18P2	763.4951	2.0	27.4	182	32.17	76.0	even	ok
	179	C46H110N56O3S	763.4943	0.9	27.6	183	51.81	21.0	even	ok
	180	C46H123N46O7PS2	763.4925	-1.4	27.7	184	49.14	10.0	even	ok
	181	C48H128N40NaO9PS2	763.4927	-1.2	27.7	185	53.23	6.0	even	ok
	182	C53H135N30O15PS2	763.4946	1.2	27.7	186	54.10	3.0	even	ok
	183	C50H125N42NaO6P2S	763.4925	-1.5	27.7	187	39.72	11.0	even	ok
	184	C56H7N31O22P2	763.4923	-1.7	27.7	188	35.40	71.0	even	ok
	185	C54H127N36NaO10S2	763.4932	-0.6	27.7	189	69.38	10.0	even	ok
	186	C52H8N35NaO18P2S	763.4935	-0.2	27.8	190	64.89	68.0	even	ok
	187	C53H11N31O22P2S	763.4940	0.5	27.9	191	59.91	66.0	even	ok
	188	C58H14N19NaO33	763.4944	1.0	28.0	192	48.76	62.0	even	ok
	189	C57H134N22O25	763.4940	0.4	28.1	193	60.08	3.0	even	ok
	190	C59H124N36O8P2	763.4934	-0.4	28.1	194	61.48	18.0	even	ok
	191	C59H152N6NaO31PS	763.4924	-1.7	28.2	195	43.24	-12.0	even	ok
	192	C57H147N12O29PS	763.4922	-1.9	28.2	196	39.54	-8.0	even	ok
	193	C62H21N11O36S	763.4930	-0.8	28.2	197	62.81	59.0	even	ok
	194	C49H2N43O15PS2	763.4942	0.7	28.2	198	65.26	72.0	even	ok
	195	C61H149N8NaO28P2	763.4922	-1.9	28.3	199	31.80	-7.0	even	ok
	196	C63H32NNaO40P2	763.4931	-0.7	28.4	200	54.94	50.0	even	ok
	197	C60H26N9O37PS	763.4938	0.3	28.4	201	75.70	54.0	even	ok
	198	C60H128N32NaO9PS	763.4944	0.9	28.5	202	49.51	14.0	even	ok
	199	C60H2N33NaO18S	763.4931	-0.6	28.5	203	66.48	77.0	even	ok
	200	C50H3N41O16P2S	763.4933	-0.4	28.8	205	59.50	72.0	even	ok
	201	C63H2N31O19P	763.4935	-0.2	28.9	206	64.14	80.0	even	ok
	202	C64H142N12O26S	763.4934	-0.3	29.1	207	73.48	1.0	even	ok
	203	C57H125N36NaO8P2	763.4922	-1.9	29.2	208	30.91	15.0	even	ok
	204	C55H140N24NaO17PS2	763.4947	1.4	29.2	209	48.21	-1.0	even	ok
	205	C55H115N44NaO7	763.4941	0.6	29.2	210	55.09	21.0	even	ok
	206	C63H136N22NaO15PS	763.4950	1.8	29.2	211	39.46	8.0	even	ok
	207	C49H131N36O13PS2	763.4932	-0.5	29.3	212	67.48	4.0	even	ok
	208	C58H153N8NaO28P2S	763.4938	0.3	29.3	213	74.13	-12.0	even	ok
	209	C51H7N37NaO17PS2	763.4943	0.9	29.3	214	59.28	68.0	even	ok
	210	C62H31N3NaO39PS	763.4940	0.5	29.5	215	69.39	50.0	even	ok
	211	C61H27N7O38P2	763.4930	-0.9	29.5	216	49.94	54.0	even	ok
	212	C48H120N48O4P2S	763.4924	-1.7	29.5	217	34.91	15.0	even	ok
	213	C58H119N40NaO6S	763.4922	-1.9	29.5	218	31.29	20.0	even	ok
	214	C56H9N25O31	763.4943	0.8	29.6	219	50.48	66.0	even	ok

Compound Spectrum SmartFormula Report

Meas. m/z	#	Ion Formula	m/z	err [ppm]	mSigma	# mSigma	Score	rdb	e⁻ Conf	N-Rule
	215	C65H7N25NaO21P	763.4936	-0.0	29.7	220	66.65	76.0	even	ok
	216	C60H155N2O35PS	763.4929	-1.0	29.7	222	56.65	-14.0	even	ok
	217	C70H146N2O32	763.4924	-1.6	29.7	223	35.45	0.0	even	ok
	218	C56H148N14O26P2S	763.4937	0.1	29.7	224	77.84	-8.0	even	ok
	219	C48H132N38O10P2S2	763.4947	1.4	29.7	225	47.23	4.0	even	ok
	220	C51H136N30NaO15PS2	763.4934	-0.4	29.8	226	71.04	0.0	even	ok
	221	C53H135N26NaO21S	763.4951	1.9	29.8	227	30.42	0.0	even	ok
	222	C50H137N32NaO12P2S2	763.4949	1.6	29.8	228	43.33	0.0	even	ok
	223	C53H118N46O4S2	763.4937	0.1	29.9	229	76.84	19.0	even	ok
	224	C61H25N7O40S	763.4924	-1.7	30.0	231	41.35	54.0	even	ok
	225	C66H10N21O25P	763.4942	0.7	30.0	232	52.45	74.0	even	ok
	226	C61H131N28O13PS	763.4949	1.6	30.1	233	35.20	12.0	even	ok
	227	C64H26N5NaO38S	763.4932	-0.6	30.1	234	64.24	55.0	even	ok
	228	C54H134N28O18S2	763.4931	-0.7	30.3	235	61.22	3.0	even	ok
	229	C59H156N4O32P2S	763.4944	1.0	30.3	236	56.08	-14.0	even	ok
	230	C63H17N15O32S	763.4937	0.1	30.3	237	77.32	64.0	even	ok
	231	C60H24N11NaO34P2	763.4925	-1.6	30.3	238	36.36	56.0	even	ok
	232	C56H131N26NaO21	763.4934	-0.3	30.4	239	60.43	5.0	even	ok
	233	C54H119N40NaO11	763.4934	-0.3	30.4	240	60.27	16.0	even	ok
	234	C53H2N39NaO16S2	763.4935	-0.2	30.5	241	74.02	73.0	even	ok
	235	C51H130N32O19S	763.4950	1.8	30.7	242	32.57	4.0	even	ok
	236	C59H122N36O10S	763.4927	-1.2	30.8	243	41.95	18.0	even	ok
	237	C61H5N29O22S	763.4937	0.1	30.8	244	76.55	75.0	even	ok
	238	C55H130N32O14S2	763.4937	0.1	30.9	245	74.75	8.0	even	ok
	239	C58H121N40NaO4P2	763.4928	-1.1	31.1	246	44.02	20.0	even	ok
	240	C53H110N50O5	763.4940	0.4	31.2	247	56.05	25.0	even	ok
	241	C55H6N29NaO27	763.4937	0.1	31.2	248	61.92	68.0	even	ok
	242	C50H127N36NaO15S	763.4945	1.1	31.2	249	43.96	6.0	even	ok
	243	C66H147N6NaO28S	763.4935	-0.1	31.3	250	73.97	-3.0	even	ok
	244	C52H6N35NaO20S2	763.4928	-1.1	31.3	251	52.59	68.0	even	ok
	245	C63H30NNaO42S	763.4925	-1.5	31.3	252	43.53	50.0	even	ok
	246	C68H15N15NaO27P	763.4943	0.9	31.4	253	47.29	70.0	even	ok
	247	C71H23N5NaO33P	763.4950	1.8	31.4	254	38.54	64.0	even	ok
	248	C47H129N42NaO6P2S2	763.4942	0.7	31.4	255	50.27	6.0	even	ok
	249	C44H121N52NaP2S2	763.4935	-0.2	31.6	256	60.42	12.0	even	ok
	250	C62H130N26O16S	763.4934	-0.3	31.6	257	69.00	12.0	even	ok
	251	C45H124N48O4P2S2	763.4940	0.5	31.9	258	53.19	10.0	even	ok
	252	C58H19N17O32P2	763.4923	-1.7	31.9	259	32.10	60.0	even	ok
	253	C61H151N2NaO37	763.4941	0.6	31.9	260	51.14	-12.0	even	ok
	254	C52H139N26O19PS2	763.4939	0.3	32.0	261	68.06	-2.0	even	ok
	255	C56H139N22NaO20S2	763.4932	-0.6	32.0	262	62.69	-1.0	even	ok
	256	C55H123N40NaO6S2	763.4939	0.3	32.0	263	68.65	15.0	even	ok
	257	C56H8N35NaO13P2S2	763.4922	-1.9	32.1	264	35.86	72.0	even	ok
	258	C63H128N30O10P2	763.4947	1.4	32.1	265	37.52	17.0	even	ok
	259	C52H10N33O21PS2	763.4949	1.6	32.1	266	41.05	66.0	even	ok
	260	C65H133N24NaO12P2	763.4948	1.6	32.2	267	34.47	13.0	even	ok

Compound Spectrum SmartFormula Report

Meas. m/z	#	Ion Formula	m/z	err [ppm]	mSigma	# mSigma	Score	rdb	e¯ Conf	N-Rule
	261	C56H111N48NaO3	763.4948	1.5	32.2	268	35.81	26.0	even	ok
	262	C55H145N18NaO22P2S	763.4932	-0.6	32.2	269	51.19	-6.0	even	ok
	263	C61H29NO47	763.4949	1.7	32.4	270	38.50	49.0	even	ok
	264	C47H3N43NaO15PS2	763.4930	-0.9	32.4	271	55.47	69.0	even	ok
	265	C65H22N9NaO34S	763.4938	0.2	32.5	272	69.04	60.0	even	ok
	266	C54H126N32O19	763.4933	-0.4	32.5	273	53.87	9.0	even	ok
	267	C69H18N11O31P	763.4948	1.6	32.6	274	34.03	68.0	even	ok
	268	C61H127N30NaO12S	763.4929	-1.0	32.6	275	43.26	14.0	even	ok
	269	C48H122N42O13S	763.4943	0.9	32.6	276	45.68	10.0	even	ok
	270	C56H116N46O2P2	763.4927	-1.3	32.7	277	39.22	24.0	even	ok
	271	C52H114N46O9	763.4933	-0.4	32.7	278	53.53	20.0	even	ok
	272	C66H6N23NaO24	763.4921	-2.0	32.8	279	28.02	76.0	even	ok
	273	C56H22N15O35PS	763.4925	-1.5	32.8	280	42.32	55.0	even	ok
	274	C54H144N20NaO21PS2	763.4940	0.5	32.9	281	62.44	-6.0	even	ok
	275	C58H27N9NaO37PS	763.4926	-1.3	32.9	282	45.79	51.0	even	ok
	276	C63H10N23NaO24S	763.4938	0.2	32.9	283	68.48	71.0	even	ok
	277	C57H135N26NaO16S2	763.4939	0.3	33.1	284	66.75	4.0	even	ok
	278	C53H138N24O22S2	763.4924	-1.6	33.2	286	39.34	-2.0	even	ok
	279	C53H140N24O20P2S	763.4930	-0.8	33.2	287	46.67	-2.0	even	ok
	280	C46H4N45NaO12P2S2	763.4945	1.1	33.2	288	49.72	69.0	even	ok
	281	C59H146N8O35	763.4940	0.4	33.4	289	52.68	-8.0	even	ok
	282	C54H20N21NaO28P2S	763.4935	-0.2	33.4	290	68.00	57.0	even	ok
	283	C65H29NO42S	763.4937	0.1	33.4	291	71.39	53.0	even	ok
	284	C64H127N28O13P	763.4932	-0.6	33.5	292	50.29	17.0	even	ok
	285	C54H15N27NaO23PS2	763.4950	1.8	33.5	293	36.31	62.0	even	ok
	286	C64H135N20NaO18S	763.4935	-0.1	33.5	294	69.73	8.0	even	ok
	287	C58H136N28O11P2S2	763.4925	-1.6	33.6	295	40.25	7.0	even	ok
	288	C54H106N54O	763.4946	1.3	33.6	296	37.52	30.0	even	ok
	289	C67H9N19O28	763.4927	-1.3	33.7	297	38.01	74.0	even	ok
	290	C52H137N28NaO16P2S	763.4925	-1.5	33.7	298	34.39	0.0	even	ok
	291	C54H5N35O20S2	763.4940	0.5	33.7	299	61.30	71.0	even	ok
	292	C57H28N11NaO34P2S	763.4941	0.7	33.8	300	58.06	51.0	even	ok
	293	C62H125N34NaO6P2	763.4942	0.7	33.8	301	47.80	19.0	even	ok
	294	C70H17N9O34	763.4933	-0.4	33.8	302	63.91	68.0	even	ok
	295	C67H135N18O19P	763.4939	0.3	33.8	303	54.56	11.0	even	ok
	296	C67H148N8O23P2S	763.4921	-2.0	33.8	304	32.25	0.0	even	ok
	297	C47H119N46NaO9S	763.4938	0.2	33.9	305	56.74	12.0	even	ok
	298	C66H23N11O29P2S	763.4924	-1.6	34.0	306	38.71	63.0	even	ok
	299	C66H132N22NaO15P	763.4933	-0.4	34.0	307	52.90	13.0	even	ok
	300	C55H23N17O32P2S	763.4940	0.5	34.0	308	61.63	55.0	even	ok
	301	C48H6N39O19PS2	763.4935	-0.2	34.2	309	67.99	67.0	even	ok
	302	C67H150N2O32S	763.4941	0.6	34.2	310	59.25	-5.0	even	ok
	303	C60H120N40O4P2	763.4940	0.5	34.3	311	50.45	23.0	even	ok
	304	C53H9N31O24S2	763.4934	-0.4	34.4	312	63.27	66.0	even	ok
	305	C55H143N18NaO24S2	763.4925	-1.4	34.4	313	41.46	-6.0	even	ok
	306	C47H7N41O16P2S2	763.4950	1.8	34.5	314	35.13	67.0	even	ok

Compound Spectrum SmartFormula Report

Meas. m/z	#	Ion Formula	m/z	err [ppm]	mSigma	# mSigma	Score	rdb	e⁻ Conf	N-Rule
	307	C63H124N32NaO9P	763.4927	-1.3	34.5	315	37.13	19.0	even	ok
	308	C61H119N38O7P	763.4925	-1.5	34.6	316	34.22	23.0	even	ok
	309	C59H30N5O41PS	763.4932	-0.6	34.6	317	57.92	49.0	even	ok
	310	C58H143N12NaO31	763.4934	-0.3	34.6	318	54.48	-6.0	even	ok
	311	C49H12N35NaO18P2S2	763.4951	2.0	34.7	319	31.89	63.0	even	ok
	312	C56H124N42OP2S2	763.4924	-1.6	34.7	320	38.95	18.0	even	ok
	313	C50H11N33NaO21PS2	763.4937	0.0	34.7	321	70.33	63.0	even	ok
	314	C49H2N39NaO21S	763.4947	1.4	34.7	322	34.19	69.0	even	ok
	315	C52H107N54NaO	763.4934	-0.3	34.7	323	53.97	27.0	even	ok
	316	C69H140N12NaO21P	763.4940	0.5	34.9	324	49.84	7.0	even	ok
	317	C53H120N42NaO8P	763.4949	1.7	34.9	325	30.37	16.0	even	ok
	318	C56H126N36O10S2	763.4944	1.0	35.0	326	49.27	13.0	even	ok
	319	C55H132N28NaO18P	763.4949	1.7	35.0	327	30.19	5.0	even	ok
	320	C58H31N7O38P2S	763.4947	1.4	35.0	328	42.40	49.0	even	ok
	321	C50H132N34O14P2S	763.4924	-1.7	35.1	329	30.47	4.0	even	ok
	322	C60H36NNaO40P2S	763.4948	1.5	35.2	330	38.87	45.0	even	ok
	323	C57H142N18O24S2	763.4937	0.1	35.3	331	66.74	-3.0	even	ok
	324	C53H123N36NaO15	763.4928	-1.1	35.3	332	38.43	11.0	even	ok
	325	C57H11N31O17P2S2	763.4927	-1.2	35.3	333	45.50	70.0	even	ok
	326	C69H14N13NaO30	763.4928	-1.1	35.4	334	39.32	70.0	even	ok
	327	C51H10N31NaO24S2	763.4922	-1.9	35.4	335	31.75	63.0	even	ok
	328	C60H26N5NaO43	763.4944	1.0	35.5	336	40.38	51.0	even	ok
	329	C55H147N16O25PS2	763.4946	1.2	35.5	337	44.46	-8.0	even	ok
	330	C70H143N8O25P	763.4946	1.2	35.5	338	37.40	5.0	even	ok
	331	C58H20N21NaO23P2S2	763.4922	-1.9	35.5	339	32.95	61.0	even	ok
	332	C72H22N3NaO36	763.4935	-0.2	35.7	340	64.74	64.0	even	ok
	333	C64H13N19O28S	763.4944	0.9	35.7	341	49.61	69.0	even	ok
	334	C55H147N12NaO31S	763.4951	2.0	35.7	342	31.37	-11.0	even	ok
	335	C45H114N52O7S	763.4936	0.0	35.7	343	57.41	16.0	even	ok
	336	C60H148N14O21P2S2	763.4925	-1.5	35.7	344	38.17	-4.0	even	ok
	337	C66H25N5O38S	763.4944	0.9	35.8	345	49.31	58.0	even	ok
	338	C65H138N16O22S	763.4941	0.6	35.8	346	57.02	6.0	even	ok
	339	C56H10N29NaO22S2	763.4942	0.7	35.8	347	54.35	67.0	even	ok
	340	C60H141N22NaO13P2S2	763.4926	-1.4	35.8	348	41.23	3.0	even	ok
	341	C69H153N2NaO25P2S	763.4923	-1.8	36.0	349	33.41	-4.0	even	ok
	342	C55H14N25NaO26S2	763.4935	-0.2	36.1	350	64.36	62.0	even	ok
	343	C51H111N50NaO5	763.4928	-1.1	36.2	351	37.48	22.0	even	ok
	344	C68H28N5NaO31P2S	763.4925	-1.4	36.2	352	39.69	59.0	even	ok
	345	C53H142N18O29S	763.4950	1.8	36.3	353	33.85	-7.0	even	ok
	346	C58H138N22O20S2	763.4944	1.0	36.4	355	47.33	2.0	even	ok
	347	C56H138N18O29	763.4933	-0.4	36.4	356	48.91	-2.0	even	ok
	348	C64H11N25O19P2S	763.4924	-1.6	36.4	357	36.21	74.0	even	ok
	349	C58H21N11O41	763.4943	0.8	36.6	358	42.14	55.0	even	ok
	350	C59H117N44NaP2	763.4935	-0.2	36.7	359	52.65	25.0	even	ok
	351	C59H119N42O3PS	763.4949	1.6	36.7	360	29.87	23.0	even	ok
	352	C57H152N10NaO27PS2	763.4947	1.4	36.7	361	39.75	-12.0	even	ok

Compound Spectrum SmartFormula Report

Meas. m/z	#	Ion Formula	m/z	err [ppm]	mSigma	# mSigma	Score	rdb	e¯ Conf	N-Rule
	353	C54H114N50S2	763.4944	1.0	36.8	362	47.15	24.0	even	ok
	354	C48H135N32O17PS2	763.4926	-1.4	36.8	363	39.21	-1.0	even	ok
	355	C58H129N36NaO3P2S2	763.4926	-1.4	36.9	364	39.99	14.0	even	ok
	356	C50H140N26NaO19PS2	763.4927	-1.2	36.9	365	42.39	-5.0	even	ok
	357	C51H14N29O25PS2	763.4942	0.7	37.0	366	52.30	61.0	even	ok
	358	C68H134N16O22	763.4924	-1.6	37.0	367	29.36	11.0	even	ok
	359	C57H4N39NaO9P2S2	763.4929	-1.0	37.0	368	46.80	77.0	even	ok
	360	C72H148N2NaO27P	763.4947	1.4	37.1	369	33.16	1.0	even	ok
	361	C53H127N34O16P	763.4948	1.5	37.2	370	31.01	9.0	even	ok
	362	C59H147N12NaO26S2	763.4939	0.3	37.3	371	59.74	-7.0	even	ok
	363	C51H115N48O6P	763.4948	1.5	37.3	372	31.09	20.0	even	ok
	364	C58H131N30NaO12S2	763.4945	1.2	37.3	373	43.01	9.0	even	ok
	365	C52H15N27O26P2S	763.4933	-0.4	37.3	374	48.19	61.0	even	ok
	366	C51H12N31NaO22P2S	763.4928	-1.1	37.3	375	36.96	63.0	even	ok
	367	C57H18N15NaO37	763.4937	0.1	37.3	376	52.85	57.0	even	ok
	368	C56H146N14O28S2	763.4931	-0.7	37.3	377	51.26	-8.0	even	ok
	369	C46H133N38NaO10P2S2	763.4935	-0.2	37.3	378	62.64	1.0	even	ok
	370	C44H111N56NaO3S	763.4931	-0.7	37.6	379	43.15	18.0	even	ok
	371	C59H16N25NaO19P2S2	763.4929	-1.0	37.6	380	46.21	66.0	even	ok
	372	C51H118N42O13	763.4926	-1.3	37.6	381	33.44	15.0	even	ok
	373	C52H2N37O22P	763.4951	1.9	37.7	382	25.57	72.0	even	ok
	374	C73H147NaO30	763.4932	-0.6	37.7	383	53.81	1.0	even	ok
	375	C49H141N28NaO16P2S2	763.4942	0.7	37.8	384	51.04	-5.0	even	ok
	376	C53H19N23NaO27PS2	763.4943	0.9	37.9	385	47.60	57.0	even	ok
	377	C65H136N22O13P2S	763.4921	-2.0	37.9	386	28.88	11.0	even	ok
	378	C62H153N8NaO23P2S2	763.4926	-1.4	37.9	387	39.13	-8.0	even	ok
	379	C66H18N13NaO30S	763.4945	1.1	37.9	388	43.35	65.0	even	ok
	380	C67H143N10NaO24S	763.4942	0.7	38.0	389	50.34	2.0	even	ok
	381	C57H157N4NaO32P2S	763.4932	-0.6	38.0	390	53.03	-17.0	even	ok
	382	C47H136N34O14P2S2	763.4940	0.5	38.1	391	54.25	-1.0	even	ok
	383	C52H13N27O28S2	763.4927	-1.2	38.1	392	41.07	61.0	even	ok
	384	C57H110N50S	763.4927	-1.2	38.2	393	34.60	29.0	even	ok
	385	C61H124N36NaO5PS	763.4950	1.8	38.2	394	26.25	19.0	even	ok
	386	C55H135N22NaO25	763.4928	-1.1	38.3	395	35.67	0.0	even	ok
	387	C70H139N10NaO24	763.4925	-1.5	38.4	396	30.77	7.0	even	ok
	388	C49H7N37O20P2S	763.4927	-1.3	38.4	397	33.17	67.0	even	ok
	389	C66H16N19NaO21P2S	763.4925	-1.4	38.5	398	37.25	70.0	even	ok
	390	C49H106N56O3	763.4926	-1.3	38.6	399	32.52	26.0	even	ok
	391	C51H143N22O23PS2	763.4932	-0.5	38.6	400	53.31	-7.0	even	ok
	392	C55H152N10O30P2S	763.4930	-0.8	38.6	401	48.73	-13.0	even	ok
	393	C60H143N16NaO22S2	763.4945	1.2	38.7	403	41.32	-2.0	even	ok
	394	C57H6N33NaO18S2	763.4948	1.6	38.7	404	34.99	72.0	even	ok
	395	C58H160O36P2S	763.4937	0.1	38.7	405	61.67	-19.0	even	ok
	396	C41H115N56NaO3S2	763.4948	1.5	38.7	406	29.91	13.0	even	ok
	397	C55H13N21O35	763.4936	-0.1	38.8	407	51.93	61.0	even	ok
	398	C57H144N14NaO28P	763.4949	1.7	38.8	408	27.18	-6.0	even	ok

Compound Spectrum SmartFormula Report

Meas. m/z	#	Ion Formula	m/z	err [ppm]	mSigma	# mSigma	Score	rdb	e⁻ Conf	N-Rule
	399	C58H151N8NaO30S2	763.4932	-0.6	38.8	409	52.66	-12.0	even	ok
	400	C56H119N44NaO2S2	763.4945	1.2	38.9	410	41.28	20.0	even	ok
	401	C59H23N17O27P2S2	763.4927	-1.2	39.0	411	41.45	59.0	even	ok
	402	C61H144N18O17P2S2	763.4931	-0.7	39.1	412	50.26	1.0	even	ok
	403	C53H148N16NaO25PS2	763.4934	-0.4	39.1	413	56.12	-11.0	even	ok
	404	C50H144N24O20P2S2	763.4947	1.4	39.1	414	37.02	-7.0	even	ok
	405	C48H4N41NaO16P2S	763.4921	-2.0	39.1	415	23.41	69.0	even	ok
	406	C60H31N3O42P2	763.4923	-1.7	39.2	416	26.61	49.0	even	ok
	407	C54H18N21NaO30S2	763.4928	-1.1	39.3	417	42.94	57.0	even	ok
	408	C52H149N18NaO22P2S2	763.4949	1.6	39.3	418	33.91	-11.0	even	ok
	409	C57H13N25O26S2	763.4947	1.4	39.3	419	37.29	65.0	even	ok
	410	C69H31NO35P2S	763.4931	-0.7	39.3	420	48.80	57.0	even	ok
	411	C56H17N21O30S2	763.4940	0.5	39.4	421	52.77	60.0	even	ok
	412	C52H139N22NaO25S	763.4945	1.1	39.5	422	35.38	-5.0	even	ok
	413	C71H142N6O28	763.4931	-0.8	39.5	423	39.93	5.0	even	ok
	414	C59H132N32O7P2S2	763.4931	-0.7	39.6	424	49.44	12.0	even	ok
	415	C42H106N62OS	763.4930	-0.9	39.6	425	38.10	22.0	even	ok
	416	C59H115N44NaO2S	763.4929	-1.0	39.6	426	35.94	25.0	even	ok
	417	C39H110N62OS2	763.4947	1.3	39.7	427	31.59	17.0	even	ok
	418	C67H141N16NaO15P2S	763.4922	-1.8	39.8	428	30.06	7.0	even	ok
	419	C66H3N29NaO17P	763.4943	0.9	39.8	429	38.06	81.0	even	ok
	420	C44H115N54O4PS	763.4951	2.0	40.0	430	23.25	16.0	even	ok
	421	C58H7N35O13P2S2	763.4934	-0.3	40.0	431	55.80	75.0	even	ok
	422	C52H124N38NaO12P	763.4943	0.8	40.1	432	38.51	11.0	even	ok
	423	C51H14N25NaO31S	763.4947	1.5	40.1	433	35.42	58.0	even	ok
	424	C54H10N25NaO31	763.4931	-0.8	40.2	434	39.40	63.0	even	ok
	425	C67H6N25O21P	763.4948	1.6	40.2	435	27.93	79.0	even	ok
	426	C53H130N28O23	763.4926	-1.3	40.3	436	31.22	4.0	even	ok
	427	C63H126N30O12S	763.4941	0.6	40.3	437	50.65	17.0	even	ok
	428	C59H134N26O16S2	763.4951	1.9	40.4	438	28.63	7.0	even	ok
	429	C46H7N39NaO19PS2	763.4923	-1.7	40.5	439	30.65	64.0	even	ok
	430	C54H22N19O31PS2	763.4949	1.6	40.5	440	32.83	55.0	even	ok
	431	C50H134N28O23S	763.4943	0.9	40.5	441	37.02	-1.0	even	ok
	432	C44H2N45O17PS2	763.4922	-1.9	40.6	442	27.88	68.0	even	ok
	433	C68H146N6O28S	763.4947	1.4	40.6	443	35.10	0.0	even	ok
	434	C60H150N8O30S2	763.4944	1.0	40.7	444	42.05	-9.0	even	ok
	435	C60H118N40O6S	763.4934	-0.3	40.7	445	45.19	23.0	even	ok
	436	C62H160O31P2S2	763.4925	-1.5	40.7	446	33.53	-15.0	even	ok
	437	C50H115N46NaO9	763.4921	-2.0	40.7	447	22.06	17.0	even	ok
	438	C64H6N27NaO20S	763.4945	1.1	40.7	448	40.30	76.0	even	ok
	439	C55H139N20O26P	763.4948	1.5	40.8	449	28.11	-2.0	even	ok
	440	C50H112N52NaO2P	763.4943	0.8	40.8	450	37.92	22.0	even	ok
	441	C49H9N31O29S	763.4946	1.3	40.9	451	37.62	62.0	even	ok
	442	C67H21N9O34S	763.4950	1.8	40.9	452	29.12	63.0	even	ok
	443	C67H19N15O25P2S	763.4931	-0.7	41.0	453	46.50	68.0	even	ok
	444	C49H131N32NaO19S	763.4938	0.2	41.0	454	46.93	1.0	even	ok

Compound Spectrum SmartFormula Report

Meas. m/z	#	Ion Formula	m/z	err [ppm]	mSigma	# mSigma	Score	rdb	e⁻ Conf	N-Rule
	445	C60H19N21O23P2S2	763.4934	-0.3	41.0	455	54.45	64.0	even	ok
	446	C61H28N11NaO29P2S2	763.4929	-1.0	41.1	456	42.18	55.0	even	ok
	447	C58H22N15NaO32S2	763.4942	0.7	41.2	457	46.93	56.0	even	ok
	448	C54H151N12O29PS2	763.4939	0.3	41.2	458	53.24	-13.0	even	ok
	449	C43H125N48NaO4P2S2	763.4928	-1.0	41.4	459	33.96	7.0	even	ok
	450	C63H156N4O27P2S2	763.4931	-0.7	41.4	460	47.24	-10.0	even	ok
	451	C63H149N12NaO19P2S2	763.4933	-0.5	41.4	461	50.31	-3.0	even	ok
	452	C59H18N19NaO28S2	763.4948	1.6	41.5	462	32.31	61.0	even	ok
	453	C60H32N7NaO33P2S2	763.4922	-1.9	41.5	463	28.14	50.0	even	ok
	454	C57H122N40O6S2	763.4951	1.9	41.5	464	27.87	18.0	even	ok
	455	C44H128N44O8P2S2	763.4934	-0.3	41.5	465	43.86	5.0	even	ok
	456	C59H30NNaO47	763.4937	0.1	41.7	466	56.21	46.0	even	ok
	457	C69H11N19NaO23P	763.4950	1.8	41.7	467	24.61	75.0	even	ok
	458	C56H27N13NaO33PS2	763.4950	1.8	41.7	468	29.11	51.0	even	ok
	459	C47H10N35O23PS2	763.4928	-1.0	41.8	469	40.30	62.0	even	ok
	460	C45H8N41NaO16P2S2	763.4938	0.2	41.9	470	54.49	64.0	even	ok
	461	C61H137N26NaO9P2S2	763.4933	-0.5	41.9	471	49.56	8.0	even	ok
	462	C68H144N12O19P2S	763.4928	-1.1	41.9	472	39.00	5.0	even	ok
	463	C52H5N31O29	763.4929	-0.9	42.0	473	34.92	67.0	even	ok
	464	C56H156N6NaO31PS2	763.4940	0.5	42.0	474	48.91	-17.0	even	ok
	465	C55H150N10O32S2	763.4924	-1.6	42.0	475	31.20	-13.0	even	ok
	466	C49H15N29NaO25PS2	763.4930	-0.9	42.0	476	43.13	58.0	even	ok
	467	C59H154N4O34S2	763.4937	0.1	42.0	477	55.59	-14.0	even	ok
	468	C43H112N58NaPS	763.4946	1.3	42.1	478	30.44	18.0	even	ok
	469	C61H146N12O26S2	763.4951	1.9	42.1	479	27.21	-4.0	even	ok
	470	C54H149N14NaO26P2S	763.4925	-1.5	42.1	480	27.54	-11.0	even	ok
	471	C55H21N17O34S2	763.4934	-0.4	42.2	481	51.47	55.0	even	ok
	472	C65H131N24NaO14S	763.4942	0.7	42.2	482	44.92	13.0	even	ok
	473	C43H3N47O14P2S2	763.4937	0.0	42.3	483	57.06	68.0	even	ok
	474	C47H126N38O17S	763.4936	0.0	42.4	484	47.84	5.0	even	ok
	475	C41H120N54O2P2S2	763.4927	-1.2	42.4	485	30.57	11.0	even	ok
	476	C60H12N29NaO15P2S2	763.4936	-0.1	42.4	486	55.43	71.0	even	ok
	477	C58H34NO45PS	763.4925	-1.5	42.4	487	32.86	44.0	even	ok
	478	C62H123N34NaO8S	763.4935	-0.1	42.5	488	45.64	19.0	even	ok
	479	C50H119N44O10P	763.4941	0.6	42.5	489	38.62	15.0	even	ok
	480	C56H32N7NaO38P2S	763.4935	-0.2	42.6	490	53.35	46.0	even	ok
	481	C53H24N17NaO32P2S	763.4928	-1.1	42.6	491	38.52	52.0	even	ok
	482	C52H127N32NaO19	763.4921	-2.0	42.6	492	21.01	6.0	even	ok
	483	C63H121N38NaO2P2	763.4948	1.6	42.7	493	26.13	24.0	even	ok
	484	C61H116N44P2	763.4947	1.4	42.7	494	28.37	28.0	even	ok
	485	C48H16N31NaO22P2S2	763.4945	1.1	42.7	495	38.42	58.0	even	ok
	486	C62H155N2NaO32S2	763.4946	1.2	42.8	496	36.80	-13.0	even	ok
	487	C54H136N24NaO22P	763.4943	0.8	42.8	497	35.63	0.0	even	ok
	488	C46H11N37O20P2S2	763.4943	0.9	42.8	498	41.25	62.0	even	ok
	489	C54H14N23O32P	763.4951	1.9	42.9	499	22.09	61.0	even	ok
	490	C70H151NaO30S	763.4949	1.6	43.0	500	30.24	-4.0	even	ok

Compound Spectrum SmartFormula Report

Meas. m/z	#	Ion Formula	m/z	err [ppm]	mSigma	# mSigma	Score	rdb	e¯ Conf	N-Rule
	491	C54H27N13O36P2S	763.4933	-0.4	43.0	501	49.56	50.0	even	ok
	492	C57H155N4NaO34S2	763.4925	-1.4	43.0	502	32.96	-17.0	even	ok
	493	C65H9N23O24S	763.4950	1.8	43.1	503	27.48	74.0	even	ok
	494	C71H13N13O30	763.4940	0.5	43.1	504	47.92	73.0	even	ok
	495	C58H150N4O39	763.4933	-0.4	43.2	505	40.76	-13.0	even	ok
	496	C52H144N20O24P2S	763.4924	-1.7	43.2	506	24.56	-7.0	even	ok
	497	C69H24N9NaO27P2S	763.4932	-0.6	43.2	507	46.68	64.0	even	ok
	498	C48H107N58P	763.4941	0.6	43.2	508	37.92	26.0	even	ok
	499	C67H2N27NaO20	763.4928	-1.1	43.3	509	31.65	81.0	even	ok
	500	C69H26N3NaO36S	763.4952	2.0	43.3	510	24.84	59.0	even	ok
785.5072	1	C78H15N17O20P2	785.5072	-0.1	15.0	1	100.00	82.0	even	ok
	2	C80H20N11NaO22P2	785.5073	0.1	15.2	2	98.34	78.0	even	ok
	3	C81H152O24P2	785.5069	-0.4	15.6	3	87.36	8.0	even	ok
	4	C72H133N28NaO4P2S	785.5074	0.2	15.7	4	95.45	22.0	even	ok
	5	C78H139N12NaO19	785.5083	1.4	16.0	5	49.28	16.0	even	ok
	6	C77H14N15NaO25	785.5086	1.7	16.1	6	49.33	79.0	even	ok
	7	C77H12N21NaO16P2	785.5066	-0.8	16.2	7	63.83	84.0	even	ok
	8	C81H23N7O26P2	785.5078	0.8	16.3	8	75.38	76.0	even	ok
	9	C70H128N34O2P2S	785.5072	-0.0	16.3	9	82.69	26.0	even	ok
	10	C75H2N29NaO15	785.5086	1.7	16.4	10	41.00	90.0	even	ok
	11	C82H148N4O20P2	785.5076	0.4	16.5	11	86.25	13.0	even	ok
	12	C75H9N21O23	785.5085	1.6	16.7	12	53.24	83.0	even	ok
	13	C80H149N4NaO20P2	785.5064	-1.1	16.8	13	54.40	10.0	even	ok
	14	C76H134N18O17	785.5082	1.2	16.8	14	52.44	20.0	even	ok
	15	C79H140N14O14P2	785.5069	-0.4	17.0	15	70.60	19.0	even	ok
	16	C81H145N8NaO16P2	785.5070	-0.3	17.0	16	75.07	15.0	even	ok
	17	C73H136N24O8P2S	785.5079	0.8	17.0	17	72.51	20.0	even	ok
	18	C75H7N27O14P2	785.5065	-0.9	17.2	19	58.25	88.0	even	ok
	19	C73H132N26NaO7PS	785.5059	-1.7	17.2	20	48.38	22.0	even	ok
	20	C79H24N7NaO26P2	785.5066	-0.7	17.4	21	74.99	73.0	even	ok
	21	C69H3N37O8P2S	785.5075	0.4	17.5	22	86.14	89.0	even	ok
	22	C83H28NNaO28P2	785.5080	1.0	17.6	23	68.28	72.0	even	ok
	23	C80H27N3O30P2	785.5072	-0.1	17.8	24	94.67	71.0	even	ok
	24	C71H8N31NaO10P2S	785.5077	0.5	17.9	25	80.21	85.0	even	ok
	25	C78H144N10O18P2	785.5062	-1.3	17.9	26	49.11	14.0	even	ok
	26	C72H4N35NaO6P2S	785.5083	1.4	18.1	27	56.10	90.0	even	ok
	27	C77H19N13O24P2	785.5065	-0.9	18.1	28	68.74	77.0	even	ok
	28	C75H141N18NaO10P2S	785.5080	1.0	18.2	29	65.77	16.0	even	ok
	29	C70H2N35O11PS	785.5060	-1.5	18.2	30	52.07	89.0	even	ok
	30	C73H129N32NaP2S	785.5080	1.0	18.3	31	65.81	27.0	even	ok
	31	C78H137N18NaO10P2	785.5064	-1.1	18.4	32	52.39	21.0	even	ok
	32	C74H6N25NaO19	785.5079	0.9	18.6	33	57.61	85.0	even	ok
	33	C75H131N22NaO13	785.5076	0.5	18.8	34	66.22	22.0	even	ok
	34	C67H122N38O5S	785.5085	1.6	18.9	35	49.36	27.0	even	ok
	35	C82H22N5O29P	785.5064	-1.1	19.3	36	61.89	76.0	even	ok
	36	C76H127N26NaO9	785.5083	1.4	19.3	37	46.16	27.0	even	ok

Compound Spectrum SmartFormula Report

Meas. m/z	#	Ion Formula	m/z	err [ppm]	mSigma	# mSigma	Score	rdb	e¯ Conf	N-Rule
	37	C83H147N2O23P	785.5061	-1.5	19.3	38	52.36	13.0	even	ok
	38	C78H146N4O27	785.5082	1.2	19.4	39	59.49	9.0	even	ok
	39	C72H7N29NaO13PS	785.5062	-1.4	19.4	40	55.21	85.0	even	ok
	40	C74H135N22O11PS	785.5064	-1.1	19.5	41	63.19	20.0	even	ok
	41	C74H4N31NaO10P2	785.5060	-1.6	19.5	42	40.95	90.0	even	ok
	42	C76H132N24O8P2	785.5062	-1.3	19.6	43	47.28	25.0	even	ok
	43	C72H11N27O14P2S	785.5082	1.2	19.7	44	58.61	83.0	even	ok
	44	C76H3N31O10P2	785.5072	-0.1	19.8	45	75.24	93.0	even	ok
	45	C74H132N28O4P2S	785.5086	1.7	20.0	46	46.50	25.0	even	ok
	46	C71H137N24NaO8P2S	785.5067	-0.7	20.0	47	72.63	17.0	even	ok
	47	C78H8N25NaO12P2	785.5073	0.1	20.0	48	74.31	89.0	even	ok
	48	C77H143N8NaO23	785.5076	0.5	20.1	49	77.08	11.0	even	ok
	49	C74H122N32O7	785.5082	1.2	20.1	50	49.13	31.0	even	ok
	50	C81H16N15NaO18P2	785.5080	1.0	20.1	51	64.96	83.0	even	ok
	51	C72H123N36OPS	785.5064	-1.1	20.1	52	62.19	31.0	even	ok
	52	C79H11N21O16P2	785.5078	0.8	20.2	53	58.05	87.0	even	ok
	53	C76H16N17NaO20P2	785.5060	-1.6	20.4	55	40.33	79.0	even	ok
	54	C72H140N20O12P2S	785.5072	-0.0	20.5	56	90.96	15.0	even	ok
	55	C73H126N28O11	785.5075	0.3	20.6	57	67.83	26.0	even	ok
	56	C76H144N14O14P2S	785.5086	1.7	20.7	58	45.65	14.0	even	ok
	57	C74H145N14NaO14P2S	785.5074	0.2	20.7	59	85.78	11.0	even	ok
	58	C69H132N30O6P2S	785.5066	-0.9	20.7	60	66.67	21.0	even	ok
	59	C66H119N42NaOS	785.5080	0.9	20.8	61	53.71	29.0	even	ok
	60	C74H16N21NaO16P2S	785.5083	1.4	21.0	62	52.67	79.0	even	ok
	61	C76H140N16NaO13PS	785.5065	-0.9	21.3	64	65.43	16.0	even	ok
	62	C82H19N11O22P2	785.5085	1.6	21.4	65	46.41	81.0	even	ok
	63	C75H144N12NaO17PS	785.5059	-1.7	21.5	66	44.36	11.0	even	ok
	64	C73H3N33NaO9PS	785.5068	-0.5	21.8	67	74.36	90.0	even	ok
	65	C74H128N30NaO3PS	785.5065	-0.9	21.8	68	64.49	27.0	even	ok
	66	C73H10N25O17PS	785.5067	-0.7	22.0	69	69.43	83.0	even	ok
	67	C80H10N19O19P	785.5063	-1.1	22.0	70	58.12	87.0	even	ok
	68	C79H26NNaO35	785.5086	1.7	22.2	71	43.29	68.0	even	ok
	69	C75H148N10O18P2S	785.5079	0.9	22.4	72	64.40	9.0	even	ok
	70	C77H21N7O33	785.5085	1.6	22.5	73	46.90	72.0	even	ok
	71	C76H18N11NaO29	785.5079	0.9	22.5	74	63.38	74.0	even	ok
	72	C73H119N36NaO3	785.5076	0.5	22.7	75	61.00	33.0	even	ok
	73	C68H129N34NaO2P2S	785.5060	-1.5	22.9	76	39.29	23.0	even	ok
	74	C75H138N14O21	785.5075	0.3	23.0	77	64.29	15.0	even	ok
	75	C77H153N4NaO20P2S	785.5080	1.0	23.4	78	58.61	5.0	even	ok
	76	C80H136N18O10P2	785.5076	0.4	23.4	79	62.11	24.0	even	ok
	77	C70H12N27NaO14P2S	785.5070	-0.3	23.5	80	77.00	80.0	even	ok
	78	C72H123N32NaO7	785.5070	-0.3	23.6	81	63.31	28.0	even	ok
	79	C74H13N17O27	785.5078	0.7	23.6	82	66.35	78.0	even	ok
	80	C82H15N13NaO21P	785.5065	-0.9	23.7	83	60.39	83.0	even	ok
	81	C75H15N19NaO19PS	785.5068	-0.5	23.8	84	71.33	79.0	even	ok
	82	C68H7N33O12P2S	785.5068	-0.5	23.9	85	71.58	84.0	even	ok

Compound Spectrum SmartFormula Report

Meas. m/z	#	Ion Formula	m/z	err [ppm]	mSigma	# mSigma	Score	rdb	e¯ Conf	N-Rule
	83	C79H133N22NaO6P2	785.5070	-0.3	24.0	86	64.45	26.0	even	ok
	84	C76H147N8O21PS	785.5064	-1.0	24.0	87	57.41	9.0	even	ok
	85	C77H128N28O4P2	785.5069	-0.4	24.0	88	60.51	30.0	even	ok
	86	C67H4N37NaO8P2S	785.5063	-1.2	24.0	89	54.44	86.0	even	ok
	87	C75H131N26O7PS	785.5071	-0.2	24.1	90	78.73	25.0	even	ok
	88	C71H114N42O	785.5075	0.3	24.3	91	62.65	37.0	even	ok
	89	C77H143N12O17PS	785.5071	-0.2	24.3	92	78.59	14.0	even	ok
	90	C82H141N12NaO12P2	785.5077	0.6	24.3	93	57.02	20.0	even	ok
	91	C66H124N40P2S	785.5059	-1.7	24.5	94	34.75	27.0	even	ok
	92	C71H15N23O18P2S	785.5075	0.4	24.5	95	73.91	78.0	even	ok
	93	C72H14N21O21PS	785.5060	-1.5	24.6	96	45.42	78.0	even	ok
	94	C74H6N29O13PS	785.5074	0.2	24.7	97	78.78	88.0	even	ok
	95	C78H28N3NaO30P2	785.5060	-1.6	24.7	98	44.11	68.0	even	ok
	96	C73H20N17NaO20P2S	785.5077	0.5	24.8	99	68.86	74.0	even	ok
	97	C74H135N18NaO17	785.5070	-0.3	24.9	100	61.70	17.0	even	ok
	98	C85H149N2NaO18P2	785.5084	1.4	24.9	101	47.11	14.0	even	ok
	99	C83H144N8O16P2	785.5082	1.3	25.2	102	42.34	18.0	even	ok
	100	C78H152N2NaO23PS	785.5065	-0.9	25.5	103	59.70	5.0	even	ok
	101	C74H19N15NaO23PS	785.5062	-1.4	25.5	104	48.34	74.0	even	ok
	102	C67H128N36NaOPS2	785.5069	-0.4	25.6	105	70.04	23.0	even	ok
	103	C83H18N9O25P	785.5070	-0.3	25.7	106	74.49	81.0	even	ok
	104	C68H131N28NaO11S	785.5080	1.0	25.8	107	57.43	18.0	even	ok
	105	C76H125N32NaP2	785.5063	-1.1	25.9	108	44.32	32.0	even	ok
	106	C69H134N24O15S	785.5085	1.6	25.9	109	42.13	16.0	even	ok
	107	C70H118N38O5	785.5068	-0.5	26.0	110	56.33	32.0	even	ok
	108	C78H156O24P2S	785.5086	1.7	26.0	111	40.41	3.0	even	ok
	109	C77H136N20NaO9PS	785.5072	-0.0	26.4	112	79.31	21.0	even	ok
	110	C81H135N16O13P	785.5061	-1.5	26.4	113	37.14	24.0	even	ok
	111	C79H148N6NaO19PS	785.5072	-0.0	26.5	114	79.15	10.0	even	ok
	112	C73H10N21NaO23	785.5073	0.0	26.6	115	65.63	80.0	even	ok
	113	C74H23N13O24P2S	785.5082	1.2	26.8	116	49.96	72.0	even	ok
	114	C66H126N34O9S	785.5078	0.8	26.8	117	60.26	22.0	even	ok
	115	C76H11N23NaO15PS	785.5075	0.3	27.0	118	70.32	84.0	even	ok
	116	C72H130N24O15	785.5068	-0.5	27.0	119	55.10	21.0	even	ok
	117	C76H18N15O23PS	785.5074	0.2	27.0	120	74.45	77.0	even	ok
	118	C84H143N6O19P	785.5067	-0.6	27.2	121	62.94	18.0	even	ok
	119	C77H150O31	785.5075	0.3	27.5	122	69.28	4.0	even	ok
	120	C73H149N10NaO18P2S	785.5067	-0.7	27.6	123	61.33	6.0	even	ok
	121	C76H28N7NaO26P2S	785.5083	1.4	27.7	124	45.09	68.0	even	ok
	122	C85H23N3NaO27P	785.5072	-0.1	27.8	125	75.15	77.0	even	ok
	123	C64H127N38O3PS2	785.5061	-1.5	27.9	126	43.32	22.0	even	ok
	124	C83H140N10NaO15P	785.5062	-1.3	27.9	127	38.89	20.0	even	ok
	125	C63H128N40P2S2	785.5076	0.4	28.0	128	66.64	22.0	even	ok
	126	C70H141N20NaO12P2S	785.5060	-1.5	28.1	129	41.97	12.0	even	ok
	127	C71H144N16O16P2S	785.5066	-0.9	28.1	130	56.54	10.0	even	ok
	128	C75H22N11O27PS	785.5067	-0.7	28.2	131	60.51	72.0	even	ok

Compound Spectrum SmartFormula Report

Meas. m/z	#	Ion Formula	m/z	err [ppm]	mSigma	# mSigma	Score	rdb	e¯ Conf	N-Rule
	129	C65H133N34NaO2P2S2	785.5077	0.6	28.3	132	62.12	18.0	even	ok
	130	C71H124N34NaO4P	785.5085	1.6	28.4	133	34.34	28.0	even	ok
	131	C76H147N4NaO27	785.5070	-0.3	28.4	134	68.38	6.0	even	ok
	132	C71H5N27O21	785.5071	-0.1	28.4	135	60.60	84.0	even	ok
	133	C74H152N6O22P2S	785.5072	0.0	28.6	136	75.74	4.0	even	ok
	134	C66H132N32NaO5PS2	785.5062	-1.3	28.7	137	46.18	18.0	even	ok
	135	C68H131N32O5PS2	785.5074	0.2	28.7	138	70.11	21.0	even	ok
	136	C76H157NaO24P2S	785.5074	0.2	28.8	139	71.26	0.0	even	ok
	137	C79H4N29NaO8P2	785.5080	1.0	28.9	140	44.40	94.0	even	ok
	138	C86H148NaO21P	785.5069	-0.5	29.1	141	64.25	14.0	even	ok
	139	C68H136N26O10P2S	785.5059	-1.7	29.2	142	37.50	16.0	even	ok
	140	C78H23N9NaO25PS	785.5075	0.3	29.2	143	66.62	73.0	even	ok
	141	C78H139N16O13PS	785.5077	0.7	29.3	144	59.51	19.0	even	ok
	142	C75H5N27O16S	785.5059	-1.7	29.5	145	36.75	88.0	even	ok
	143	C69H115N42NaO	785.5063	-1.2	29.5	146	39.38	34.0	even	ok
	144	C80H7N25O12P2	785.5085	1.6	29.5	147	32.24	92.0	even	ok
	145	C66H3N39NaO7PS2	785.5072	-0.1	29.5	148	72.36	86.0	even	ok
	146	C67H6N31NaO17S	785.5083	1.3	29.6	149	44.79	81.0	even	ok
	147	C77H27N5NaO29PS	785.5068	-0.5	29.6	150	62.49	68.0	even	ok
	148	C73H136N20NaO14P	785.5085	1.6	29.7	151	33.16	17.0	even	ok
	149	C71H127N28NaO11	785.5063	-1.2	29.7	152	39.30	23.0	even	ok
	150	C65H123N38NaO5S	785.5073	0.1	29.8	153	59.66	24.0	even	ok
	151	C80H151N2O23PS	785.5077	0.7	30.0	154	58.39	8.0	even	ok
	152	C66H136N30O6P2S2	785.5082	1.3	30.1	155	44.89	16.0	even	ok
	153	C77H14N19O19PS	785.5080	1.0	30.3	156	50.07	82.0	even	ok
	154	C69H127N36OPS2	785.5081	1.1	30.5	158	48.63	26.0	even	ok
	155	C70H2N31NaO17	785.5066	-0.8	30.5	159	45.06	86.0	even	ok
	156	C70H136N26NaO7PS2	785.5076	0.4	30.7	160	62.88	17.0	even	ok
	157	C68H141N24NaO8P2S2	785.5084	1.5	30.9	161	40.57	12.0	even	ok
	158	C69H119N40O2P	785.5083	1.4	30.9	162	35.10	32.0	even	ok
	159	C76H25N3O37	785.5078	0.7	31.1	163	55.55	67.0	even	ok
	160	C81H6N23O15P	785.5070	-0.3	31.1	164	65.29	92.0	even	ok
	161	C77H17N13O26S	785.5059	-1.7	31.1	165	35.41	77.0	even	ok
	162	C67H135N28O9PS2	785.5067	-0.6	31.1	166	57.60	16.0	even	ok
	163	C75H22N7NaO33	785.5073	0.0	31.4	168	70.02	69.0	even	ok
	164	C75H2N33O9PS	785.5080	1.0	31.4	169	48.86	93.0	even	ok
	165	C63H118N44O3S	785.5072	-0.1	31.5	170	57.37	28.0	even	ok
	166	C69H123N38NaS2	785.5061	-1.5	31.7	171	39.05	28.0	even	ok
	167	C80H144N10NaO15PS	785.5079	0.8	31.7	172	52.27	15.0	even	ok
	168	C69H16N23NaO18P2S	785.5063	-1.2	31.9	173	45.33	75.0	even	ok
	169	C77H10N21NaO18S	785.5060	-1.6	32.0	174	37.73	84.0	even	ok
	170	C72H24N13NaO24P2S	785.5070	-0.3	32.0	175	63.07	69.0	even	ok
	171	C71H131N26O12P	785.5083	1.4	32.1	176	34.01	21.0	even	ok
	172	C69H122N34O9	785.5062	-1.4	32.2	177	34.13	27.0	even	ok
	173	C76H127N30O3PS	785.5077	0.6	32.3	178	55.45	30.0	even	ok
	174	C70H19N19O22P2S	785.5069	-0.5	32.3	179	58.80	73.0	even	ok

Compound Spectrum SmartFormula Report

Meas. m/z	#	Ion Formula	m/z	err [ppm]	mSigma	# mSigma	Score	rdb	e¯ Conf	N-Rule
	175	C69H130N30O8S2	785.5059	-1.7	32.4	180	35.39	21.0	even	ok
	176	C74H142N10O25	785.5068	-0.5	32.4	181	48.50	10.0	even	ok
	177	C69H140N22NaO11PS2	785.5069	-0.4	32.4	182	59.61	12.0	even	ok
	178	C80H3N27NaO11P	785.5065	-1.0	32.5	183	40.71	94.0	even	ok
	179	C63H2N41O9PS2	785.5064	-1.1	32.6	184	45.60	85.0	even	ok
	180	C62H3N43O6P2S2	785.5079	0.8	32.7	185	51.81	85.0	even	ok
	181	C73H17N13O31	785.5071	-0.1	32.7	186	65.64	73.0	even	ok
	182	C67H6N35O11PS2	785.5077	0.6	32.7	187	55.80	84.0	even	ok
	183	C67H11N29O16P2S	785.5062	-1.3	32.7	188	41.01	79.0	even	ok
	184	C78H124N32P2	785.5075	0.4	32.7	189	49.92	35.0	even	ok
	185	C78H30NO33PS	785.5074	0.2	32.7	190	64.73	66.0	even	ok
	186	C79H26N5O29PS	785.5080	1.0	32.7	191	47.02	71.0	even	ok
	187	C79H19N13NaO21PS	785.5082	1.2	32.8	192	43.58	78.0	even	ok
	188	C71H132N30NaO3PS2	785.5082	1.3	32.9	193	42.35	22.0	even	ok
	189	C83H11N17NaO17P	785.5072	-0.1	32.9	194	66.25	88.0	even	ok
	190	C64H8N37NaO8P2S2	785.5080	1.0	33.0	195	47.84	81.0	even	ok
	191	C68H2N39O7PS2	785.5084	1.5	33.1	196	38.63	89.0	even	ok
	192	C74H26N7O31PS	785.5060	-1.5	33.1	197	37.20	67.0	even	ok
	193	C70H6N29O18P	785.5086	1.8	33.1	198	27.71	84.0	even	ok
	194	C73H27N9O28P2S	785.5075	0.4	33.3	199	59.76	67.0	even	ok
	195	C81H132N22O6P2	785.5082	1.3	33.3	200	34.87	29.0	even	ok
	196	C80H129N26NaO2P2	785.5077	0.6	33.3	201	46.06	31.0	even	ok
	197	C65H7N35NaO11PS2	785.5065	-0.9	33.4	202	48.19	81.0	even	ok
	198	C79H22N7NaO28S	785.5060	-1.6	33.5	203	36.42	73.0	even	ok
	199	C75H32N3NaO30P2S	785.5077	0.6	33.5	204	55.70	63.0	even	ok
	200	C72H14N17NaO27	785.5066	-0.8	33.7	205	49.95	75.0	even	ok
	201	C76H31NNaO33PS	785.5062	-1.4	33.8	206	39.70	63.0	even	ok
	202	C77H7N27NaO11PS	785.5082	1.2	33.8	207	42.59	89.0	even	ok
	203	C73H139N14NaO21	785.5063	-1.2	34.1	208	35.38	12.0	even	ok
	204	C70H143N14NaO21S	785.5080	1.0	34.1	209	46.83	7.0	even	ok
	205	C71H139N22O11PS2	785.5081	1.1	34.2	210	44.18	15.0	even	ok
	206	C79H138N14O16S	785.5062	-1.3	34.5	211	40.90	19.0	even	ok
	207	C78H132N24NaO5PS	785.5079	0.8	34.5	212	48.87	26.0	even	ok
	208	C69H11N29NaO13PS2	785.5078	0.8	34.5	213	49.73	80.0	even	ok
	209	C71H135N24NaO10S2	785.5061	-1.5	34.6	214	36.45	17.0	even	ok
	210	C83H137N16NaO8P2	785.5084	1.4	34.6	215	31.09	25.0	even	ok
	211	C71H146N10O25S	785.5085	1.6	34.7	216	33.95	5.0	even	ok
	212	C81H150O26S	785.5063	-1.2	34.7	217	40.75	8.0	even	ok
	213	C84H14N13O21P	785.5077	0.6	34.7	218	53.28	86.0	even	ok
	214	C68H138N20O19S	785.5078	0.8	34.8	219	49.39	11.0	even	ok
	215	C65H11N33O12P2S2	785.5085	1.7	34.9	220	33.43	79.0	even	ok
	216	C75H148N6NaO24P	785.5085	1.6	34.9	221	28.98	6.0	even	ok
	217	C67H135N24NaO15S	785.5073	0.1	35.0	222	62.75	13.0	even	ok
	218	C70H128N30NaO8P	785.5078	0.7	35.0	223	42.08	23.0	even	ok
	219	C81H147N6O19PS	785.5084	1.5	35.0	224	35.73	13.0	even	ok
	220	C79H123N30O3P	785.5061	-1.5	35.2	225	29.79	35.0	even	ok

Compound Spectrum SmartFormula Report

Meas. m/z	#	Ion Formula	m/z	err [ppm]	mSigma	# mSigma	Score	rdb	e⁻ Conf	N-Rule
	221	C70H126N34O4S2	785.5066	-0.8	35.3	226	47.99	26.0	even	ok
	222	C70H143N18O15PS2	785.5074	0.2	35.4	227	59.29	10.0	even	ok
	223	C78H13N17O22S	785.5065	-0.9	35.4	228	46.71	82.0	even	ok
	224	C70H7N33NaO9PS2	785.5085	1.6	35.4	229	33.39	85.0	even	ok
	225	C69H3N33NaO14P	785.5081	1.1	35.5	230	35.73	86.0	even	ok
	226	C61H129N40NaP2S2	785.5064	-1.1	35.7	231	42.23	19.0	even	ok
	227	C66H10N31O15PS2	785.5070	-0.2	35.9	232	58.27	79.0	even	ok
	228	C64H137N30NaO6P2S2	785.5070	-0.2	35.9	233	58.31	13.0	even	ok
	229	C68H5N33O14S2	785.5062	-1.3	36.0	234	38.42	84.0	even	ok
	230	C65H130N30O13S	785.5072	-0.1	36.2	235	61.23	17.0	even	ok
	231	C71H134N20O19	785.5062	-1.4	36.2	236	30.93	16.0	even	ok
	232	C62H132N36O4P2S2	785.5069	-0.4	36.2	237	54.40	17.0	even	ok
	233	C73H144N16NaO13PS2	785.5082	1.3	36.4	238	38.62	11.0	even	ok
	234	C81H128N24NaO5P	785.5062	-1.3	36.4	239	31.40	31.0	even	ok
	235	C72H18N15O28P	785.5086	1.8	36.4	240	30.43	73.0	even	ok
	236	C72H153N6NaO22P2S	785.5060	-1.5	36.5	241	34.15	1.0	even	ok
	237	C66H139N24O13PS2	785.5061	-1.5	36.8	243	34.80	11.0	even	ok
	238	C86H19N7NaO23P	785.5078	0.8	36.9	244	47.06	82.0	even	ok
	239	C73H143N12O22P	785.5083	1.4	36.9	245	29.94	10.0	even	ok
	240	C73H156N2O26P2S	785.5066	-0.8	36.9	246	45.45	-1.0	even	ok
	241	C72H148N12NaO17PS2	785.5076	0.4	37.0	247	53.45	6.0	even	ok
	242	C64H127N34NaO9S	785.5066	-0.8	37.0	248	47.02	19.0	even	ok
	243	C85H139N10O15P	785.5074	0.2	37.0	249	57.20	23.0	even	ok
	244	C81H143N8NaO18S	785.5064	-1.1	37.0	250	41.32	15.0	even	ok
	245	C82H131N20O9P	785.5067	-0.6	37.1	251	40.91	29.0	even	ok
	246	C68H15N25NaO17PS2	785.5072	-0.1	37.1	252	59.88	75.0	even	ok
	247	C65H140N26O10P2S2	785.5076	0.4	37.1	253	52.95	11.0	even	ok
	248	C79H135N20O9PS	785.5084	1.5	37.2	254	33.91	24.0	even	ok
	249	C80H25N3O32S	785.5065	-0.9	37.2	255	44.69	71.0	even	ok
	250	C70H148N12O20P2S	785.5059	-1.7	37.3	256	30.66	5.0	even	ok
	251	C67H145N20NaO12P2S2	785.5077	0.6	37.3	257	49.36	7.0	even	ok
	252	C77H126N28O6S	785.5062	-1.3	37.3	258	37.83	30.0	even	ok
	253	C68H144N18NaO15PS2	785.5062	-1.3	37.4	259	37.18	7.0	even	ok
	254	C68H123N36O6P	785.5076	0.5	37.6	261	42.04	27.0	even	ok
	255	C83H152NaO21PS	785.5086	1.7	37.7	262	30.60	9.0	even	ok
	256	C75H151NaO31	785.5063	-1.2	37.9	263	38.57	1.0	even	ok
	257	C72H131N28NaO6S2	785.5067	-0.6	37.9	264	48.06	22.0	even	ok
	258	C70H14N25O17PS2	785.5084	1.5	38.0	265	33.87	78.0	even	ok
	259	C80H18N11NaO24S	785.5067	-0.7	38.0	266	46.77	78.0	even	ok
	260	C70H10N27NaO16S2	785.5063	-1.1	38.1	267	39.30	80.0	even	ok
	261	C71H142N16O18S2	785.5059	-1.7	38.1	268	30.60	10.0	even	ok
	262	C72H138N20O14S2	785.5066	-0.8	38.3	269	44.50	15.0	even	ok
	263	C70H9N23O25	785.5064	-1.0	38.4	270	34.24	79.0	even	ok
	264	C69H18N17NaO27S	785.5083	1.3	38.5	271	35.58	70.0	even	ok
	265	C84H136N14NaO11P	785.5069	-0.5	38.8	272	41.74	25.0	even	ok
	266	C66H10N27NaO21S	785.5076	0.5	38.8	273	49.98	76.0	even	ok

Compound Spectrum SmartFormula Report

Meas. m/z	#	Ion Formula	m/z	err [ppm]	mSigma	# mSigma	Score	rdb	e¯ Conf	N-Rule
	267	C87H144N4NaO17P	785.5075	0.4	38.9	274	51.11	19.0	even	ok
	268	C72H140N16NaO18P	785.5078	0.7	39.0	275	37.81	12.0	even	ok
	269	C67H13N23O25S	785.5081	1.1	39.0	276	37.95	74.0	even	ok
	270	C68H148N16O16P2S2	785.5082	1.3	39.1	277	35.50	5.0	even	ok
	271	C78H6N25NaO14S	785.5067	-0.7	39.2	278	45.18	89.0	even	ok
	272	C87H22N3O27P	785.5084	1.4	39.3	279	32.97	80.0	even	ok
	273	C73H146N6O29	785.5062	-1.4	39.4	280	34.13	5.0	even	ok
	274	C81H140N14NaO11PS	785.5086	1.7	39.6	281	29.10	20.0	even	ok
	275	C69H147N14O19PS2	785.5068	-0.6	39.7	282	46.26	5.0	even	ok
	276	C85H13N11O24	785.5062	-1.3	39.7	283	34.55	86.0	even	ok
	277	C79H131N22NaO8S	785.5064	-1.1	39.7	284	38.33	26.0	even	ok
	278	C70H153N10NaO18P2S2	785.5084	1.5	39.8	285	32.15	1.0	even	ok
	279	C64H5N33O19S	785.5075	0.3	39.8	286	51.82	80.0	even	ok
	280	C73H147N10NaO20S2	785.5061	-1.5	40.0	287	31.67	6.0	even	ok
	281	C60H4N43NaO6P2S2	785.5067	-0.7	40.0	288	43.74	82.0	even	ok
	282	C69H18N21O21PS2	785.5077	0.6	40.0	289	45.91	73.0	even	ok
	283	C72H19N19NaO19PS2	785.5085	1.6	40.1	290	29.41	74.0	even	ok
	284	C71H6N31NaO12S2	785.5070	-0.3	40.1	291	51.62	85.0	even	ok
	285	C63H2N37NaO15S	785.5069	-0.4	40.2	292	49.48	82.0	even	ok
	286	C69H6N27NaO21	785.5059	-1.7	40.2	293	23.93	81.0	even	ok
	287	C73H151N8O21PS2	785.5081	1.1	40.3	294	37.47	4.0	even	ok
	288	C82H146N4O22S	785.5069	-0.4	40.4	295	49.11	13.0	even	ok
	289	C74H143N14NaO16S2	785.5067	-0.6	40.6	296	44.69	11.0	even	ok
	290	C63H12N33NaO12P2S2	785.5073	0.1	40.6	297	53.51	76.0	even	ok
	291	C71H152N8NaO21PS2	785.5069	-0.4	40.7	298	48.04	1.0	even	ok
	292	C61H7N39O10P2S2	785.5072	-0.1	40.8	299	54.45	80.0	even	ok
	293	C71H28N9NaO28P2S	785.5063	-1.2	40.8	300	35.96	64.0	even	ok
	294	C88H147O21P	785.5081	1.1	40.9	301	37.21	17.0	even	ok
	295	C67H120N40NaO2P	785.5071	-0.1	40.9	302	43.85	29.0	even	ok
	296	C74H26N3NaO37	785.5066	-0.8	41.1	303	41.22	64.0	even	ok
	297	C71H15N19NaO24P	785.5081	1.1	41.1	304	30.68	75.0	even	ok
	298	C71H122N38S2	785.5073	0.0	41.2	305	54.22	31.0	even	ok
	299	C70H135N22O16P	785.5077	0.5	41.3	306	37.99	16.0	even	ok
	300	C69H23N15O26P2S	785.5062	-1.3	41.4	307	32.66	68.0	even	ok
	301	C72H31N5O32P2S	785.5069	-0.5	41.6	308	46.19	62.0	even	ok
	302	C71H23N15NaO23PS2	785.5078	0.8	41.6	309	41.11	69.0	even	ok
	303	C73H134N24O10S2	785.5073	0.0	41.6	310	53.60	20.0	even	ok
	304	C81H21N7O28S	785.5072	-0.0	41.6	311	53.75	76.0	even	ok
	305	C86H138N8O18	785.5059	-1.7	41.7	312	27.47	23.0	even	ok
	306	C71H13N23O20S2	785.5069	-0.4	41.8	313	46.49	78.0	even	ok
	307	C65H14N27O19PS2	785.5064	-1.1	41.8	314	35.88	74.0	even	ok
	308	C62H122N40O7S	785.5065	-0.9	41.9	315	31.94	23.0	even	ok
	309	C64H15N29O16P2S2	785.5079	0.8	42.0	316	40.31	74.0	even	ok
	310	C87H18N5NaO26	785.5063	-1.1	42.0	317	35.07	82.0	even	ok
	311	C75H156N2NaO23PS2	785.5082	1.3	42.2	318	32.91	0.0	even	ok
	312	C66H20N23NaO18P2S2	785.5080	1.0	42.3	319	37.23	70.0	even	ok

Compound Spectrum SmartFormula Report

Meas. m/z	#	Ion Formula	m/z	err [ppm]	mSigma	# mSigma	Score	rdb	e¯ Conf	N-Rule
	313	C79H9N21O18S	785.5072	-0.0	42.4	320	52.54	87.0	even	ok
	314	C67H19N21NaO21PS2	785.5065	-0.9	42.4	321	37.99	70.0	even	ok
	315	C61H131N34NaO9S2	785.5083	1.4	42.4	322	31.03	14.0	even	ok
	316	C72H21N9O35	785.5065	-1.0	42.4	323	36.92	68.0	even	ok
	317	C70H17N19O24S2	785.5062	-1.3	42.5	324	32.28	73.0	even	ok
	318	C80H134N18O12S	785.5069	-0.4	42.6	325	46.19	24.0	even	ok
	319	C58H123N44NaO3S2	785.5076	0.5	42.7	326	43.95	20.0	even	ok
	320	C59H126N40O7S2	785.5082	1.2	42.9	327	33.14	18.0	even	ok
	321	C69H10N25O22P	785.5079	0.9	43.2	328	31.21	79.0	even	ok
	322	C72H155NaO31S	785.5080	1.0	43.2	329	36.52	-4.0	even	ok
	323	C84H7N21NaO13P	785.5078	0.8	43.2	330	39.66	93.0	even	ok
	324	C61H119N44NaO3S	785.5060	-1.6	43.4	331	22.56	25.0	even	ok
	325	C72H155N4O25PS2	785.5074	0.2	43.4	332	47.51	-1.0	even	ok
	326	C71H18N13NaO31	785.5059	-1.7	43.5	333	26.33	70.0	even	ok
	327	C69H147N10NaO25S	785.5073	0.1	43.5	334	49.67	2.0	even	ok
	328	C74H150N6O24S2	785.5066	-0.8	43.6	335	38.54	4.0	even	ok
	329	C56H118N50OS2	785.5075	0.4	43.6	336	45.65	24.0	even	ok
	330	C65H115N46P	785.5070	-0.3	43.7	337	38.26	33.0	even	ok
	331	C70H150N6O29S	785.5078	0.8	43.8	338	38.64	0.0	even	ok
	332	C88H143N2NaO20	785.5060	-1.5	43.8	339	28.20	19.0	even	ok
	333	C62H3N39NaO12PS	785.5084	1.5	43.8	340	28.12	82.0	even	ok
	334	C69H132N26NaO12P	785.5071	-0.1	43.8	341	40.55	18.0	even	ok
	335	C73H127N32NaO2S2	785.5074	0.2	43.9	342	47.43	27.0	even	ok
	336	C72H9N27O16S2	785.5076	0.4	43.9	343	44.29	83.0	even	ok
	337	C73H18N17NaO22S2	785.5070	-0.3	44.0	344	46.39	74.0	even	ok
	338	C67H23N19O22P2S2	785.5085	1.7	44.2	345	25.91	68.0	even	ok
	339	C79H128N28NaOPS	785.5085	1.7	44.2	346	25.71	31.0	even	ok
	340	C75H139N18NaO12S2	785.5074	0.2	44.2	347	46.92	16.0	even	ok
	341	C72H22N13NaO26S2	785.5064	-1.1	44.3	348	33.17	69.0	even	ok
	342	C83H26NNaO30S	785.5074	0.2	44.4	349	47.70	72.0	even	ok
	343	C67H142N16O23S	785.5072	-0.1	44.4	350	48.90	6.0	even	ok
	344	C75H146N10O20S2	785.5073	0.0	44.4	351	49.33	9.0	even	ok
	345	C63H128N36NaO6PS	785.5081	1.1	44.5	352	27.18	19.0	even	ok
	346	C88H21NO30	785.5069	-0.5	44.6	353	42.63	80.0	even	ok
	347	C73H27N5NaO34P	785.5081	1.1	44.7	354	33.19	64.0	even	ok
	348	C68H22N17O25PS2	785.5070	-0.2	44.7	355	45.82	68.0	even	ok
	349	C66H139N20NaO19S	785.5066	-0.8	44.7	356	38.06	8.0	even	ok
	350	C72H26N11O27PS2	785.5084	1.5	44.8	357	27.96	67.0	even	ok
	351	C63H141N26NaO10P2S2	785.5064	-1.1	44.8	358	32.98	8.0	even	ok
	352	C85H10N17O17P	785.5084	1.4	44.9	359	28.23	91.0	even	ok
	353	C72H2N35NaO8S2	785.5077	0.6	45.0	360	40.33	90.0	even	ok
	354	C81H14N15NaO20S	785.5073	0.1	45.1	361	46.84	83.0	even	ok
	355	C82H2N27O11P	785.5077	0.6	45.1	362	33.52	97.0	even	ok
	356	C82H139N12NaO14S	785.5071	-0.2	45.1	363	45.62	20.0	even	ok
	357	C68H7N29NaO18P	785.5074	0.2	45.2	364	37.84	81.0	even	ok
	358	C66H149N16NaO16P2S2	785.5070	-0.2	45.4	365	45.05	2.0	even	ok

Compound Spectrum SmartFormula Report

Meas. m/z	#	Ion Formula	m/z	err [ppm]	mSigma	# mSigma	Score	rdb	e⁻ Conf	N-Rule
	359	C61H136N32O8P2S2	785.5062	-1.3	45.4	366	29.99	12.0	even	ok
	360	C73H154N2O28S2	785.5059	-1.7	45.5	367	25.00	-1.0	even	ok
	361	C74H152N2NaO28P	785.5078	0.7	45.5	368	31.41	1.0	even	ok
	362	C64H144N22O14P2S2	785.5069	-0.4	45.6	369	42.10	6.0	even	ok
	363	C76H155NaO26S2	785.5067	-0.6	45.6	370	38.87	0.0	even	ok
	364	C76H140N20O7P2S2	785.5060	-1.6	45.7	371	25.63	19.0	even	ok
	365	C70H27N11NaO27PS2	785.5072	-0.1	45.7	372	47.23	64.0	even	ok
	366	C64H134N26O17S	785.5065	-0.9	46.0	374	34.16	12.0	even	ok
	367	C82H124N28NaOP	785.5069	-0.5	46.1	375	33.95	36.0	even	ok
	368	C68H151N10O23PS2	785.5061	-1.5	46.2	376	26.86	0.0	even	ok
	369	C71H22N11O32P	785.5079	0.9	46.2	377	34.17	68.0	even	ok
	370	C67H127N32O10P	785.5070	-0.3	46.3	378	35.55	22.0	even	ok
	371	C61H123N42O4PS	785.5080	1.0	46.4	379	27.72	23.0	even	ok
	372	C84H27N3O25P2S	785.5059	-1.7	46.4	380	24.33	75.0	even	ok
	373	C74H14N21NaO18S2	785.5077	0.6	46.5	381	38.49	79.0	even	ok
	374	C74H31N5NaO29PS2	785.5085	1.6	46.6	382	24.38	63.0	even	ok
	375	C67H152N12O20P2S2	785.5076	0.4	46.7	383	40.46	0.0	even	ok
	376	C70H156N4NaO25PS2	785.5062	-1.3	46.7	384	28.73	-4.0	even	ok
	377	C78H152N6O17P2S2	785.5060	-1.6	46.8	385	24.88	8.0	even	ok
	378	C69H157N6NaO22P2S2	785.5077	0.6	46.8	386	37.71	-4.0	even	ok
	379	C63H131N30NaO13S	785.5060	-1.6	46.8	387	24.58	14.0	even	ok
	380	C77H151N4NaO22S2	785.5074	0.2	46.9	388	43.29	5.0	even	ok
	381	C87H15N11NaO19P	785.5085	1.6	47.0	389	24.37	87.0	even	ok
	382	C60H6N37NaO15S2	785.5086	1.8	47.1	390	22.70	77.0	even	ok
	383	C78H122N32O2S	785.5069	-0.4	47.1	391	40.44	35.0	even	ok
	384	C86H135N14O11P	785.5081	1.1	47.2	392	31.16	28.0	even	ok
	385	C75H15N23O13P2S2	785.5063	-1.2	47.2	393	29.07	82.0	even	ok
	386	C73H3N37O3P2S2	785.5063	-1.2	47.2	394	28.95	93.0	even	ok
	387	C72H147N8O26P	785.5077	0.5	47.4	395	31.76	5.0	even	ok
	388	C74H130N28O6S2	785.5079	0.9	47.5	397	33.28	25.0	even	ok
	389	C86H131N16NaO10	785.5060	-1.5	47.5	398	25.25	30.0	even	ok
	390	C66H2N35O16P	785.5073	0.0	47.5	399	37.50	85.0	even	ok
	391	C86H257N15NaOP	785.5072	0.0	47.6	400	37.79	-33.0	even	ok
	392	C83H127N24O5P	785.5074	0.2	47.8	401	35.31	34.0	even	ok
	393	C68H22N13NaO31S	785.5076	0.5	47.8	402	38.64	65.0	even	ok
	394	C73H25N9O30S2	785.5069	-0.4	47.8	403	39.16	67.0	even	ok
	395	C74H21N13O26S2	785.5076	0.4	47.8	404	39.46	72.0	even	ok
	396	C71H30N3NaO37S	785.5083	1.3	47.9	405	27.16	59.0	even	ok
	397	C85H6N19NaO16	785.5063	-1.1	47.9	406	29.48	93.0	even	ok
	398	C76H142N14O16S2	785.5079	0.9	48.0	407	32.63	14.0	even	ok
	399	C79H2N29NaO10S	785.5073	0.1	48.2	408	42.85	94.0	even	ok
	400	C65H140N22NaO16PS	785.5081	1.2	48.2	409	29.17	8.0	even	ok
	401	C60H120N46NaPS	785.5075	0.3	48.2	410	33.97	25.0	even	ok
	402	C83H142N8O18S	785.5076	0.5	48.3	411	38.30	18.0	even	ok
	403	C69H25N9O35S	785.5081	1.2	48.3	412	29.05	63.0	even	ok
	404	C78H145N14NaO9P2S2	785.5061	-1.4	48.4	413	25.71	15.0	even	ok

Compound Spectrum SmartFormula Report

Meas. m/z	#	Ion Formula	m/z	err [ppm]	mSigma	# mSigma	Score	rdb	e⁻ Conf	N-Rule
	405	C71H30N7O31PS2	785.5077	0.6	48.4	414	36.00	62.0	even	ok
	406	C82H17N11O24S	785.5079	0.8	48.5	415	33.11	81.0	even	ok
	407	C66H17N19O29S	785.5075	0.3	48.5	416	40.21	69.0	even	ok
	408	C65H14N23NaO25S	785.5069	-0.4	48.6	417	39.02	71.0	even	ok
	409	C70H160N2O26P2S2	785.5083	1.3	48.6	418	27.00	-6.0	even	ok
	410	C82H15N17O15P2S	785.5059	-1.7	48.6	419	22.72	86.0	even	ok
	411	C88H140N8NaO13P	785.5082	1.2	48.6	420	27.60	24.0	even	ok
	412	C73H5N31O12S2	785.5082	1.3	48.8	421	27.36	88.0	even	ok
	413	C71H159O29PS2	785.5068	-0.6	48.9	422	35.64	-6.0	even	ok
	414	C90H268NO11P	785.5085	1.6	48.9	423	23.73	-41.0	even	ok
	415	C85H132N18NaO7P	785.5075	0.4	49.1	424	31.95	30.0	even	ok
	416	C66H124N36NaO6P	785.5064	-1.0	49.2	425	25.15	24.0	even	ok
	417	C71H144N12NaO22P	785.5071	-0.1	49.2	426	34.67	7.0	even	ok
	418	C68H133N28NaO9P2	785.5086	1.8	49.2	427	17.70	18.0	even	ok
	419	C62H16N29NaO16P2S2	785.5067	-0.7	49.4	428	33.45	71.0	even	ok
	420	C77H27N9O23P2S2	785.5063	-1.2	49.4	429	27.28	71.0	even	ok
	421	C59H8N39NaO10P2S2	785.5060	-1.6	49.4	430	23.02	77.0	even	ok
	422	C87H260N11O5P	785.5078	0.7	49.4	431	28.18	-35.0	even	ok
	423	C80H127N26NaO4S	785.5071	-0.2	49.4	432	40.07	31.0	even	ok
	424	C90H264N3NaO10	785.5064	-1.0	49.5	433	29.62	-39.0	even	ok
	425	C80H157NaO19P2S2	785.5061	-1.4	49.5	434	24.99	4.0	even	ok
	426	C84H123N28OP	785.5081	1.1	49.6	435	24.20	39.0	even	ok
	427	C63H9N29O23S	785.5068	-0.6	49.6	436	35.41	75.0	even	ok
	428	C63H135N28O14PS	785.5080	1.0	49.7	437	30.11	12.0	even	ok
	429	C73H35NNaO33PS2	785.5079	0.8	49.7	438	32.35	58.0	even	ok
	430	C75H30N3NaO32S2	785.5070	-0.3	49.8	439	39.24	63.0	even	ok
	431	C60H11N35O14P2S2	785.5065	-0.9	49.9	440	30.74	75.0	even	ok
	432	C86H9N15O20	785.5069	-0.5	49.9	441	36.36	91.0	even	ok
	433	C87H134N12O14	785.5066	-0.8	49.9	442	31.59	28.0	even	ok
	434	C77H20N17NaO15P2S2	785.5064	-1.0	49.9	443	28.92	78.0	even	ok
	435	C75H8N31NaO5P2S2	785.5064	-1.1	50.0	444	28.79	89.0	even	ok
	436	C62H6N33NaO19S	785.5063	-1.2	50.2	445	26.43	77.0	even	ok
	437	C76H26N7NaO28S2	785.5077	0.6	50.2	446	34.41	68.0	even	ok
	438	C57H3N45O8P2S2	785.5058	-1.8	50.2	447	20.58	81.0	even	ok
	439	C76H135N22NaO8S2	785.5081	1.1	50.2	448	28.43	21.0	even	ok
	440	C84H126N22O8	785.5059	-1.7	50.3	449	17.75	34.0	even	ok
	441	C65H24N19NaO22P2S2	785.5073	0.1	50.3	450	40.38	65.0	even	ok
	442	C72H29N5O34S2	785.5062	-1.3	50.3	451	25.76	62.0	even	ok
	443	C63H19N25O20P2S2	785.5072	-0.0	50.4	452	41.31	69.0	even	ok
	444	C75H17N17O22S2	785.5082	1.3	50.4	453	25.93	77.0	even	ok
	445	C84H119N30Na	785.5060	-1.5	50.5	454	19.12	41.0	even	ok
	446	C71H35NO36P2S	785.5062	-1.3	50.7	455	24.98	57.0	even	ok
	447	C78H147N8NaO18S2	785.5081	1.1	50.8	456	27.90	10.0	even	ok
	448	C78H154O26S2	785.5079	0.9	50.8	457	29.93	3.0	even	ok
	449	C89H265N5NaO7P	785.5079	0.9	50.9	458	25.13	-39.0	even	ok
	450	C85H147N2NaO20S	785.5077	0.6	51.0	459	33.04	14.0	even	ok

Compound Spectrum SmartFormula Report

Meas. m/z	#	Ion Formula	m/z	err [ppm]	mSigma	# mSigma	Score	rdb	e⁻ Conf	N-Rule
	451	C86H128N22NaO3P	785.5082	1.2	51.0	460	21.48	35.0	even	ok
	452	C86H2N23NaO12	785.5070	-0.3	51.0	461	37.37	98.0	even	ok
	453	C80H5N25O14S	785.5079	0.8	51.1	462	30.66	92.0	even	ok
	454	C84H22N5NaO26S	785.5080	1.0	51.2	463	28.30	77.0	even	ok
	455	C84H20N11NaO17P2S	785.5061	-1.5	51.3	464	22.83	82.0	even	ok
	456	C67H26N13O29PS2	785.5064	-1.1	51.3	465	27.18	63.0	even	ok
	457	C69H139N18O20P	785.5070	-0.3	51.4	466	30.57	11.0	even	ok
	458	C75H10N25NaO14S2	785.5084	1.4	51.5	467	23.18	84.0	even	ok
	459	C89H139N6NaO16	785.5067	-0.7	51.6	469	32.18	24.0	even	ok
	460	C66H27N15O26P2S2	785.5079	0.8	51.7	470	30.24	63.0	even	ok
	461	C64H15N25NaO22PS	785.5084	1.5	51.7	471	22.16	71.0	even	ok
	462	C88H130N16O10	785.5072	0.0	51.8	472	39.99	33.0	even	ok
	463	C60H135N30NaO13S2	785.5077	0.5	51.8	473	33.47	9.0	even	ok
	464	C66H128N34O7P2	785.5085	1.6	51.8	474	17.88	22.0	even	ok
	465	C70H19N15NaO28P	785.5074	0.2	51.8	475	31.02	70.0	even	ok
	466	C64H119N42O4P	785.5063	-1.2	51.8	476	21.52	28.0	even	ok
	467	C69H31N7NaO31PS2	785.5065	-0.9	51.8	477	28.82	59.0	even	ok
	468	C68H32N9NaO28P2S2	785.5080	1.0	51.9	478	27.93	59.0	even	ok
	469	C63H143N20NaO19S2	785.5083	1.4	51.9	479	23.36	3.0	even	ok
	470	C81H130N22O8S	785.5076	0.5	52.0	480	34.24	29.0	even	ok
	471	C79H32N3NaO25P2S2	785.5064	-1.0	52.0	481	27.20	67.0	even	ok
	472	C79H148N10O13P2S2	785.5067	-0.7	52.1	482	30.68	13.0	even	ok
	473	C88H14N9NaO22	785.5070	-0.3	52.2	483	36.09	87.0	even	ok
	474	C88H256N15OP	785.5084	1.5	52.2	484	17.92	-30.0	even	ok
	475	C88H259N9O8	785.5063	-1.2	52.3	485	20.94	-35.0	even	ok
	476	C61H138N26O17S2	785.5082	1.2	52.3	486	25.00	7.0	even	ok
	477	C85H3N25NaO9P	785.5085	1.6	52.4	487	17.30	98.0	even	ok
	478	C57H127N40NaO7S2	785.5070	-0.3	52.4	488	35.38	15.0	even	ok
	479	C85H122N26O4	785.5066	-0.8	52.5	489	24.28	39.0	even	ok
	480	C58H130N36O11S2	785.5075	0.4	52.5	490	34.88	13.0	even	ok
	481	C80H3N31O5P2S	785.5059	-1.7	52.9	491	19.83	97.0	even	ok
	482	C62H10N31O20PS	785.5083	1.3	53.0	492	23.13	75.0	even	ok
	483	C77H136N24O3P2S2	785.5066	-0.7	53.0	493	29.78	24.0	even	ok
	484	C77H22N11NaO24S2	785.5084	1.4	53.1	494	22.00	73.0	even	ok
	485	C68H151N6NaO29S	785.5066	-0.7	53.3	495	29.46	-3.0	even	ok
	486	C88H252N17Na	785.5064	-1.0	53.3	496	21.82	-28.0	even	ok
	487	C69H154N2O33S	785.5072	-0.1	53.4	497	37.43	-5.0	even	ok
	488	C90H135N10NaO12	785.5074	0.2	53.4	498	35.81	29.0	even	ok
	489	C55H122N46O5S2	785.5068	-0.5	53.5	499	32.17	19.0	even	ok
	490	C76H11N27O9P2S2	785.5069	-0.4	53.6	500	33.51	87.0	even	ok
	491	C68H136N22NaO16P	785.5065	-1.0	53.6	501	22.01	13.0	even	ok
	492	C87H5N19O16	785.5075	0.4	53.6	502	33.34	96.0	even	ok
	493	C90H261N9NaO3P	785.5086	1.7	53.7	503	15.67	-34.0	even	ok
	494	C82H10N19NaO16S	785.5080	1.0	53.7	504	26.25	88.0	even	ok
	495	C68H14N21O26P	785.5073	0.1	53.7	505	30.92	74.0	even	ok
	496	C69H35N5O32P2S2	785.5085	1.7	53.7	506	19.35	57.0	even	ok

Compound Spectrum SmartFormula Report

Meas. m/z	#	Ion Formula	m/z	err [ppm]	mSigma	# mSigma	Score	rdb	e¯ Conf	N-Rule
	497	C78H23N13O19P2S2	785.5069	-0.4	53.8	507	33.38	76.0	even	ok
	498	C61H7N35NaO16PS	785.5078	0.7	53.8	508	29.96	77.0	even	ok
	499	C54H119N50NaOS2	785.5063	-1.2	53.8	509	24.20	21.0	even	ok
	500	C77H138N18O12S2	785.5086	1.7	53.9	510	18.51	19.0	even	ok
807.5192	1	C71H27N15O27P2S	807.5193	0.1	14.3	1	100.00	68.0	even	ok
	2	C73H32N9NaO29P2S	807.5194	0.3	14.4	2	94.21	64.0	even	ok
	3	C60H3N49O5P2S2	807.5196	0.5	14.4	3	86.25	86.0	even	ok
	4	C62H8N43NaO7P2S2	807.5198	0.7	14.6	4	80.45	82.0	even	ok
	5	C74H157N6NaO23P2S	807.5191	-0.1	14.9	5	98.71	1.0	even	ok
	6	C75H160N2O27P2S	807.5197	0.6	15.0	6	83.32	-1.0	even	ok
	7	C70H24N19NaO23P2S	807.5188	-0.6	15.1	7	82.96	70.0	even	ok
	8	C71H151N10NaO26S	807.5204	1.5	15.1	8	55.48	2.0	even	ok
	9	C72H152N12O21P2S	807.5190	-0.3	15.1	10	92.55	5.0	even	ok
	10	C61H2N47O8PS2	807.5181	-1.4	15.2	11	59.21	86.0	even	ok
	11	C63H133N40NaOP2S2	807.5195	0.3	15.3	12	90.71	19.0	even	ok
	12	C74H35N5O33P2S	807.5200	0.9	15.4	13	71.47	62.0	even	ok
	13	C65H145N26NaO11P2S2	807.5195	0.3	15.5	14	90.26	8.0	even	ok
	14	C64H136N36O5P2S2	807.5200	1.0	15.5	15	69.76	17.0	even	ok
	15	C63H140N32O9P2S2	807.5193	0.1	15.5	16	95.79	12.0	even	ok
	16	C63H11N39O11P2S2	807.5203	1.3	15.7	17	59.11	80.0	even	ok
	17	C62H137N36NaO5P2S2	807.5188	-0.5	15.9	18	83.46	14.0	even	ok
	18	C69H146N16O24S	807.5203	1.3	16.0	19	59.12	6.0	even	ok
	19	C73H161N2NaO27P2S	807.5185	-0.9	16.1	20	70.42	-4.0	even	ok
	20	C68H19N25O21P2S	807.5186	-0.7	16.2	21	75.81	74.0	even	ok
	21	C63H7N41NaO10PS2	807.5183	-1.2	16.3	22	62.77	82.0	even	ok
	22	C66H141N30NaO7P2S2	807.5201	1.1	16.5	23	63.38	13.0	even	ok
	23	C66H144N24NaO14PS2	807.5180	-1.5	16.5	24	52.93	8.0	even	ok
	24	C71H149N16NaO17P2S	807.5185	-0.9	16.7	25	69.40	7.0	even	ok
	25	C66H148N22O15P2S2	807.5200	1.0	16.7	26	67.85	6.0	even	ok
	26	C64H132N38NaO4PS2	807.5180	-1.5	16.8	27	52.39	19.0	even	ok
	27	C65H16N33NaO13P2S2	807.5204	1.5	16.9	28	53.03	76.0	even	ok
	28	C60H132N42O3P2S2	807.5187	-0.7	17.0	29	76.34	18.0	even	ok
	29	C72H23N19O23P2S	807.5200	0.9	17.0	30	69.37	73.0	even	ok
	30	C69H139N24NaO16S	807.5204	1.5	17.1	31	53.37	13.0	even	ok
	31	C71H20N23NaO19P2S	807.5194	0.3	17.1	32	89.26	75.0	even	ok
	32	C69H15N29O17P2S	807.5193	0.1	17.2	33	94.51	79.0	even	ok
	33	C71H156N8O25P2S	807.5183	-1.1	17.3	34	63.79	0.0	even	ok
	34	C76H159O30PS	807.5182	-1.3	17.4	35	58.41	-1.0	even	ok
	35	C61H12N39NaO11P2S2	807.5191	-0.2	17.6	36	91.61	77.0	even	ok
	36	C68H153N16NaO17P2S2	807.5201	1.2	17.7	37	61.59	2.0	even	ok
	37	C65H6N37NaO16S	807.5200	1.0	17.8	38	65.59	82.0	even	ok
	38	C76H30N3NaO38	807.5197	0.6	17.8	39	77.66	64.0	even	ok
	39	C68H143N20NaO20S	807.5197	0.7	17.8	40	75.82	8.0	even	ok
	40	C75H34N3O36PS	807.5185	-0.9	17.9	41	67.80	62.0	even	ok
	41	C69H144N22O15P2S	807.5183	-1.1	17.9	42	62.73	11.0	even	ok
	42	C74H28N13NaO25P2S	807.5201	1.1	17.9	43	63.10	69.0	even	ok

Compound Spectrum SmartFormula Report

Meas. m/z	#	Ion Formula	m/z	err [ppm]	mSigma	# mSigma	Score	rdb	e¯ Conf	N-Rule
	43	C72H36N5NaO33P2S	807.5188	-0.6	18.0	44	78.27	59.0	even	ok
	44	C62H15N35O15P2S2	807.5196	0.5	18.0	45	79.95	75.0	even	ok
	45	C67H18N23NaO26S	807.5200	1.0	18.1	46	65.03	71.0	even	ok
	46	C65H135N34O8PS2	807.5185	-0.9	18.1	47	69.08	17.0	even	ok
	47	C67H134N30O14S	807.5203	1.3	18.1	48	56.82	17.0	even	ok
	48	C64H20N29NaO17P2S2	807.5198	0.7	18.3	49	74.45	71.0	even	ok
	49	C63H14N33O18PS2	807.5181	-1.3	18.3	50	55.81	75.0	even	ok
	50	C67H16N29NaO17P2S	807.5181	-1.4	18.3	51	54.16	76.0	even	ok
	51	C59H7N45O9P2S2	807.5190	-0.3	18.3	52	84.93	81.0	even	ok
	52	C64H10N37O14PS2	807.5188	-0.5	18.4	53	79.18	80.0	even	ok
	53	C73H39NO37P2S	807.5193	0.1	18.4	54	91.79	57.0	even	ok
	54	C67H147N20O18PS2	807.5185	-0.9	18.7	55	68.35	6.0	even	ok
	55	C63H4N47NaO3P2S2	807.5204	1.5	18.7	56	51.18	87.0	even	ok
	56	C68H12N33NaO13P2S	807.5187	-0.6	18.8	57	76.57	81.0	even	ok
	57	C70H31N11O31P2S	807.5186	-0.7	18.8	58	71.89	63.0	even	ok
	58	C65H19N27NaO20PS2	807.5183	-1.2	19.3	59	59.21	71.0	even	ok
	59	C58H4N49NaO5P2S2	807.5184	-1.0	19.3	60	64.18	83.0	even	ok
	60	C77H26N7NaO34	807.5204	1.4	19.4	61	52.41	69.0	even	ok
	61	C74H25N9O36	807.5196	0.4	19.5	63	79.97	68.0	even	ok
	62	C77H31N5O33P2	807.5183	-1.2	19.5	64	59.14	67.0	even	ok
	63	C65H13N29O24S	807.5199	0.8	19.5	65	67.83	75.0	even	ok
	64	C66H138N26O18S	807.5196	0.5	19.6	66	78.12	12.0	even	ok
	65	C67H140N28NaO10PS2	807.5186	-0.7	19.7	67	71.63	13.0	even	ok
	66	C73H22N17O26PS	807.5185	-0.9	19.7	68	65.08	73.0	even	ok
	67	C69H28N15NaO27P2S	807.5181	-1.4	19.8	69	52.63	65.0	even	ok
	68	C66H7N39O11P2S	807.5186	-0.8	19.8	70	70.02	85.0	even	ok
	69	C73H148N16O17P2S	807.5197	0.6	19.9	71	75.51	10.0	even	ok
	70	C65H23N25O21P2S2	807.5203	1.3	20.0	72	53.81	69.0	even	ok
	71	C66H15N31NaO16PS2	807.5189	-0.3	20.2	73	81.20	76.0	even	ok
	72	C75H21N13O32	807.5202	1.3	20.2	74	55.88	73.0	even	ok
	73	C59H131N40NaO8S2	807.5201	1.1	20.3	75	60.45	15.0	even	ok
	74	C71H158N2O34S	807.5203	1.3	20.3	76	53.93	-5.0	even	ok
	75	C69H152N14NaO20PS2	807.5187	-0.7	20.4	77	70.82	2.0	even	ok
	76	C75H153N10NaO19P2S	807.5198	0.7	20.5	78	69.65	6.0	even	ok
	77	C77H155NaO32	807.5194	0.2	20.5	79	83.71	1.0	even	ok
	78	C66H131N34NaO10S	807.5197	0.7	20.6	80	71.61	19.0	even	ok
	79	C72H145N20NaO13P2S	807.5191	-0.1	20.7	81	87.30	12.0	even	ok
	80	C70H155N6NaO30S	807.5198	0.7	20.9	82	70.88	-3.0	even	ok
	81	C76H156N6O23P2S	807.5203	1.4	21.0	83	51.50	4.0	even	ok
	82	C70H140N26O11P2S	807.5190	-0.3	21.0	84	81.77	16.0	even	ok
	83	C64H149N22NaO15P2S2	807.5188	-0.5	21.0	85	75.15	3.0	even	ok
	84	C67H28N19NaO23P2S2	807.5204	1.5	21.1	86	48.39	65.0	even	ok
	85	C75H27N11NaO28PS	807.5186	-0.8	21.3	87	67.61	69.0	even	ok
	86	C65H152N18O19P2S2	807.5193	0.2	21.6	88	84.16	1.0	even	ok
	87	C64H10N33NaO20S	807.5194	0.2	21.6	89	83.33	77.0	even	ok
	88	C67H157N12NaO21P2S2	807.5195	0.3	21.6	90	79.14	-3.0	even	ok

Compound Spectrum SmartFormula Report

Meas. m/z	#	Ion Formula	m/z	err [ppm]	mSigma	# mSigma	Score	rdb	e¯ Conf	N-Rule
	89	C57H126N46O6S2	807.5199	0.9	21.7	91	63.23	19.0	even	ok
	90	C62H144N28O13P2S2	807.5187	-0.7	21.7	92	69.17	7.0	even	ok
	91	C66H22N23O24PS2	807.5188	-0.5	21.8	93	73.69	69.0	even	ok
	92	C68H156N10NaO24PS2	807.5180	-1.5	22.0	94	47.23	-3.0	even	ok
	93	C68H143N24O14PS2	807.5192	-0.0	22.0	95	86.53	11.0	even	ok
	94	C74H147N14O20PS	807.5182	-1.3	22.1	96	52.66	10.0	even	ok
	95	C64H3N45NaO6PS2	807.5189	-0.3	22.2	97	77.62	87.0	even	ok
	96	C75H150N6O30	807.5193	0.1	22.2	98	85.51	5.0	even	ok
	97	C65H135N30NaO14S	807.5191	-0.2	22.3	99	82.29	14.0	even	ok
	98	C64H126N40O8S	807.5196	0.5	22.3	100	73.85	23.0	even	ok
	99	C68H150N12O28S	807.5196	0.5	22.3	101	73.48	1.0	even	ok
	100	C61H141N32NaO9P2S2	807.5181	-1.3	22.3	102	51.22	9.0	even	ok
	101	C73H22N13NaO32	807.5190	-0.2	22.4	103	80.34	70.0	even	ok
	102	C65H4N43NaO7P2S	807.5181	-1.4	22.5	104	49.35	87.0	even	ok
	103	C74H18N17NaO28	807.5197	0.6	22.7	105	69.99	75.0	even	ok
	104	C69H137N30NaO7P2S	807.5185	-0.9	23.0	106	60.57	18.0	even	ok
	105	C58H6N43NaO14S2	807.5204	1.4	23.0	107	48.23	78.0	even	ok
	106	C67H18N27O20PS2	807.5195	0.3	23.1	108	76.90	74.0	even	ok
	107	C76H30N7O32PS	807.5191	-0.1	23.3	109	82.57	67.0	even	ok
	108	C56H123N50NaO2S2	807.5194	0.2	23.3	110	78.64	21.0	even	ok
	109	C68H160N8O25P2S2	807.5200	1.0	23.3	111	58.78	-5.0	even	ok
	110	C76H152N8NaO22PS	807.5183	-1.1	23.3	112	55.47	6.0	even	ok
	111	C70H155N10O24PS2	807.5192	-0.0	23.4	113	84.20	0.0	even	ok
	112	C68H27N17NaO26PS2	807.5189	-0.3	23.5	114	75.78	65.0	even	ok
	113	C78H156N2O27P2	807.5180	-1.5	23.5	115	45.69	4.0	even	ok
	114	C62H5N39O18S	807.5192	0.0	23.6	116	84.46	81.0	even	ok
	115	C78H151N4NaO28	807.5201	1.1	23.7	117	56.34	6.0	even	ok
	116	C69H30N9NaO36S	807.5200	1.0	23.7	118	57.42	60.0	even	ok
	117	C59H136N38O7P2S2	807.5180	-1.5	23.8	119	45.64	13.0	even	ok
	118	C70H11N33O13P2S	807.5200	0.9	23.9	120	59.99	84.0	even	ok
	119	C67H147N16NaO24S	807.5191	-0.2	23.9	121	79.50	3.0	even	ok
	120	C80H32N3NaO31P2	807.5191	-0.2	23.9	122	79.69	68.0	even	ok
	121	C67H132N36O5P2S	807.5183	-1.1	24.0	123	54.90	22.0	even	ok
	122	C70H165N2NaO27P2S2	807.5202	1.2	24.1	125	53.50	-9.0	even	ok
	123	C73H29N5O40	807.5189	-0.4	24.1	126	72.95	63.0	even	ok
	124	C65H6N41O10PS2	807.5195	0.3	24.1	127	75.30	85.0	even	ok
	125	C78H27N9O29P2	807.5189	-0.3	24.1	128	74.67	72.0	even	ok
	126	C70H148N18NaO16PS2	807.5193	0.1	24.2	129	80.04	7.0	even	ok
	127	C72H13N23O26	807.5196	0.4	24.2	130	72.30	79.0	even	ok
	128	C65H128N42NaPS2	807.5186	-0.7	24.3	131	64.61	24.0	even	ok
	129	C67H127N38NaO6S	807.5204	1.5	24.3	132	45.87	24.0	even	ok
	130	C63H130N36O12S	807.5189	-0.3	24.4	133	73.85	18.0	even	ok
	131	C69H159N6O28PS2	807.5185	-0.9	24.4	134	60.47	-5.0	even	ok
	132	C72H16N27NaO15P2S	807.5201	1.1	24.5	135	54.89	80.0	even	ok
	133	C71H17N19O30	807.5189	-0.4	24.5	136	72.08	74.0	even	ok
	134	C76H146N10O26	807.5199	0.9	24.5	137	59.50	10.0	even	ok

Compound Spectrum SmartFormula Report

Meas. m/z	#	Ion Formula	m/z	err [ppm]	mSigma	# mSigma	Score	rdb	e¯ Conf	N-Rule
	135	C77H155N4O26PS	807.5188	-0.5	24.6	138	70.65	4.0	even	ok
	136	C69H8N37NaO9P2S	807.5194	0.3	24.7	139	75.90	86.0	even	ok
	137	C74H154N2O34	807.5186	-0.8	24.7	140	62.60	0.0	even	ok
	138	C67H25N15O34S	807.5199	0.9	24.7	141	60.34	64.0	even	ok
	139	C63H24N25NaO21P2S2	807.5191	-0.1	24.8	142	78.54	66.0	even	ok
	140	C67H3N43O7P2S	807.5193	0.1	24.9	143	80.24	90.0	even	ok
	141	C65H122N44O4S	807.5203	1.3	25.1	144	48.95	28.0	even	ok
	142	C54H118N56S2	807.5193	0.1	25.2	145	79.92	25.0	even	ok
	143	C60H16N35NaO15P2S2	807.5184	-1.0	25.2	146	56.47	72.0	even	ok
	144	C61H19N31O19P2S2	807.5190	-0.3	25.2	147	73.19	70.0	even	ok
	145	C69H23N21NaO22PS2	807.5196	0.5	25.3	148	68.67	70.0	even	ok
	146	C78H35NNaO34PS	807.5193	0.1	25.4	149	79.43	63.0	even	ok
	147	C74H147N10NaO26	807.5187	-0.6	25.4	150	65.89	7.0	even	ok
	148	C72H160N4NaO26PS2	807.5193	0.1	25.4	151	77.61	-4.0	even	ok
	149	C66H131N38O4PS2	807.5192	-0.0	25.5	152	79.80	22.0	even	ok
	150	C66H22N19NaO30S	807.5194	0.2	25.7	153	75.86	66.0	even	ok
	151	C65H26N19O28PS2	807.5181	-1.3	25.7	154	47.60	64.0	even	ok
	152	C71H164NaO30PS2	807.5187	-0.7	25.8	155	62.93	-9.0	even	ok
	153	C65H142N22O22S	807.5189	-0.3	25.8	156	71.68	7.0	even	ok
	154	C64H27N21O25P2S2	807.5196	0.5	25.8	157	67.29	64.0	even	ok
	155	C71H40NNaO37P2S	807.5181	-1.4	25.9	158	46.06	54.0	even	ok
	156	C66H32N15NaO27P2S2	807.5198	0.7	26.0	159	62.61	60.0	even	ok
	157	C77H24N13NaO25P2	807.5184	-1.0	26.1	160	55.01	74.0	even	ok
	158	C67H11N35NaO12PS2	807.5196	0.5	26.2	161	67.40	81.0	even	ok
	159	C71H10N31O16PS	807.5185	-0.9	26.2	162	56.19	84.0	even	ok
	160	C61H2N43NaO14S	807.5187	-0.7	26.3	163	63.12	83.0	even	ok
	161	C58H11N41O13P2S2	807.5183	-1.2	26.3	164	51.01	76.0	even	ok
	162	C67H31N13NaO30PS2	807.5183	-1.2	26.4	165	50.65	60.0	even	ok
	163	C69H30N13O30PS2	807.5195	0.3	26.6	166	70.78	63.0	even	ok
	164	C72H26N9NaO36	807.5184	-1.1	26.8	167	52.58	65.0	even	ok
	165	C75H19N19O23P2	807.5183	-1.2	27.0	168	49.93	78.0	even	ok
	166	C71H151N14O20PS2	807.5199	0.8	27.1	169	58.47	5.0	even	ok
	167	C61H143N26NaO18S2	807.5201	1.1	27.1	170	51.69	4.0	even	ok
	168	C64H17N25O28S	807.5192	0.0	27.3	171	77.42	70.0	even	ok
	169	C73H15N25NaO18PS	807.5186	-0.8	27.4	172	58.79	80.0	even	ok
	170	C68H136N32NaO6PS2	807.5193	0.1	27.4	173	74.53	18.0	even	ok
	171	C63H123N44NaO4S	807.5191	-0.2	27.5	174	60.66	25.0	even	ok
	172	C68H17N25O23S2	807.5180	-1.5	27.6	175	41.44	74.0	even	ok
	173	C75H14N21NaO24	807.5204	1.4	27.7	176	43.65	80.0	even	ok
	174	C73H151N6NaO30	807.5181	-1.4	27.9	177	43.40	2.0	even	ok
	175	C67H35N11O31P2S2	807.5203	1.3	27.9	178	44.92	58.0	even	ok
	176	C59H138N32O16S2	807.5200	0.9	28.1	179	54.46	8.0	even	ok
	177	C64H139N26NaO18S	807.5184	-1.0	28.1	180	52.26	9.0	even	ok
	178	C71H136N30O7P2S	807.5197	0.6	28.2	181	62.69	21.0	even	ok
	179	C74H18N21O22PS	807.5191	-0.1	28.3	182	73.36	78.0	even	ok
	180	C74H144N20O13P2S	807.5203	1.4	28.3	183	43.71	15.0	even	ok

Compound Spectrum SmartFormula Report

Meas. m/z	#	Ion Formula	m/z	err [ppm]	mSigma	# mSigma	Score	rdb	e⁻ Conf	N-Rule
	181	C73H9N27O22	807.5202	1.2	28.3	184	46.62	84.0	even	ok
	182	C73H141N24NaO9P2S	807.5198	0.7	28.6	185	58.08	17.0	even	ok
	183	C71H35N7NaO32PS2	807.5196	0.5	28.6	186	63.41	59.0	even	ok
	184	C70H26N17O26PS2	807.5201	1.2	28.6	187	48.38	68.0	even	ok
	185	C81H157NaO25P2	807.5188	-0.5	28.6	188	62.80	5.0	even	ok
	186	C72H30N7O37P	807.5204	1.5	28.6	189	42.06	63.0	even	ok
	187	C69H40N5NaO33P2S2	807.5204	1.5	28.7	190	40.52	54.0	even	ok
	188	C58H135N36NaO12S2	807.5194	0.2	28.7	191	69.18	10.0	even	ok
	189	C66H5N39O13S2	807.5180	-1.5	28.8	192	40.14	85.0	even	ok
	190	C70H18N21O27P	807.5204	1.4	28.8	193	42.02	74.0	even	ok
	191	C73H163O30PS2	807.5199	0.8	28.8	194	56.00	-6.0	even	ok
	192	C68H14N31O16PS2	807.5201	1.1	28.8	195	48.24	79.0	even	ok
	193	C68H34N9O34PS2	807.5188	-0.5	29.0	197	62.74	58.0	even	ok
	194	C79H152N6O23P2	807.5187	-0.7	29.0	198	58.25	9.0	even	ok
	195	C63H14N29NaO24S	807.5187	-0.6	29.0	199	59.35	72.0	even	ok
	196	C70H21N15O34	807.5182	-1.2	29.0	200	46.09	69.0	even	ok
	197	C66H161N8NaO25P2S2	807.5188	-0.5	29.2	201	62.48	-8.0	even	ok
	198	C73H155N4O31P	807.5201	1.1	29.3	202	48.91	0.0	even	ok
	199	C76H149N14NaO15P2S	807.5205	1.6	29.3	203	39.20	11.0	even	ok
	200	C69H139N28O10PS2	807.5199	0.8	29.4	204	55.49	16.0	even	ok
	201	C70H133N34NaO3P2S	807.5191	-0.1	29.4	205	71.30	23.0	even	ok
	202	C75H143N14NaO22	807.5194	0.2	29.5	206	56.93	12.0	even	ok
	203	C63H153N18NaO19P2S2	807.5181	-1.3	29.5	207	43.56	-2.0	even	ok
	204	C69H159N2NaO34S	807.5191	-0.2	29.5	208	69.91	-8.0	even	ok
	205	C73H156N8NaO22PS2	807.5200	1.0	29.5	209	51.37	1.0	even	ok
	206	C71H10N27NaO22	807.5190	-0.2	29.6	210	56.53	81.0	even	ok
	207	C64H156N14O23P2S2	807.5187	-0.7	29.6	211	57.79	-4.0	even	ok
	208	C72H142N16O24	807.5186	-0.8	29.7	212	46.28	11.0	even	ok
	209	C61H118N50O2S	807.5189	-0.4	29.8	213	54.12	29.0	even	ok
	210	C62H127N40NaO8S	807.5184	-1.0	29.8	214	41.66	20.0	even	ok
	211	C70H22N19NaO25S2	807.5181	-1.4	30.0	215	42.55	70.0	even	ok
	212	C76H23N15NaO24PS	807.5193	0.1	30.0	216	71.32	74.0	even	ok
	213	C64H119N48NaS	807.5197	0.6	30.0	217	48.25	30.0	even	ok
	214	C72H135N28O10PS	807.5182	-1.3	30.1	218	43.61	21.0	even	ok
	215	C67H164N4O29P2S2	807.5193	0.2	30.2	219	68.83	-10.0	even	ok
	216	C56H130N42O10S2	807.5193	0.1	30.2	220	70.83	14.0	even	ok
	217	C70H14N23NaO26	807.5184	-1.1	30.2	221	40.26	76.0	even	ok
	218	C70H39N3NaO36PS2	807.5190	-0.3	30.2	222	64.83	54.0	even	ok
	219	C64H143N24O19PS	807.5204	1.5	30.3	223	39.30	7.0	even	ok
	220	C70H29N11O33S2	807.5180	-1.5	30.3	224	38.95	63.0	even	ok
	221	C62H134N32O16S	807.5183	-1.2	30.3	225	45.93	13.0	even	ok
	222	C61H148N24O17P2S2	807.5180	-1.5	30.5	226	39.09	2.0	even	ok
	223	C61H9N35O22S	807.5186	-0.8	31.0	227	52.78	76.0	even	ok
	224	C60H3N45NaO11PS	807.5202	1.2	31.0	228	44.73	83.0	even	ok
	225	C74H140N22NaO12PS	807.5183	-1.1	31.0	229	46.23	17.0	even	ok
	226	C67H154N8O32S	807.5189	-0.3	31.0	230	63.49	-4.0	even	ok

Compound Spectrum SmartFormula Report

Meas. m/z	#	Ion Formula	m/z	err [ppm]	mSigma	# mSigma	Score	rdb	e⁻ Conf	N-Rule
	227	C72H31N11NaO28PS2	807.5203	1.3	31.0	231	42.13	64.0	even	ok
	228	C68H10N33NaO15S2	807.5181	-1.4	31.1	232	41.31	81.0	even	ok
	229	C73H138N20O20	807.5193	0.1	31.1	233	58.06	16.0	even	ok
	230	C60H18N29NaO24S2	807.5204	1.4	31.1	234	39.76	67.0	even	ok
	231	C70H19N25NaO18PS2	807.5203	1.3	31.2	235	42.05	75.0	even	ok
	232	C62H131N38O9PS	807.5204	1.5	31.3	236	32.00	18.0	even	ok
	233	C75H143N18O16PS	807.5188	-0.5	31.4	237	60.01	15.0	even	ok
	234	C71H27N11NaO33P	807.5199	0.8	31.5	238	52.72	65.0	even	ok
	235	C55H127N46NaO6S2	807.5187	-0.6	31.6	239	57.00	16.0	even	ok
	236	C71H144N22NaO12PS2	807.5200	1.0	31.6	240	48.92	12.0	even	ok
	237	C72H33NO44	807.5182	-1.2	31.7	241	43.33	58.0	even	ok
	238	C69H5N33O20	807.5189	-0.4	31.7	242	50.52	85.0	even	ok
	239	C73H148N12NaO23P	807.5202	1.3	31.7	243	35.64	7.0	even	ok
	240	C77H26N11O28PS	807.5198	0.7	31.8	244	53.66	72.0	even	ok
	241	C58H13N35O22S2	807.5202	1.3	31.8	245	42.42	71.0	even	ok
	242	C57H10N39NaO18S2	807.5197	0.6	32.0	246	56.02	73.0	even	ok
	243	C82H31N3O31P2	807.5203	1.3	32.0	247	41.10	71.0	even	ok
	244	C79H23N13O25P2	807.5196	0.5	32.0	248	58.42	77.0	even	ok
	245	C72H38N3O36PS2	807.5202	1.2	32.1	249	44.32	57.0	even	ok
	246	C72H150N12O23S2	807.5184	-1.1	32.1	250	46.37	5.0	even	ok
	247	C68H128N40OP2S	807.5190	-0.3	32.2	251	52.29	27.0	even	ok
	248	C66H151N12NaO28S	807.5184	-1.0	32.4	252	47.26	-2.0	even	ok
	249	C81H28N7NaO27P2	807.5198	0.7	32.4	253	54.17	73.0	even	ok
	250	C60H122N46O6S	807.5183	-1.2	32.5	254	36.18	24.0	even	ok
	251	C72H34N5NaO35S2	807.5181	-1.4	32.5	255	40.09	59.0	even	ok
	252	C69H37NO44S	807.5199	0.9	32.6	256	49.86	53.0	even	ok
	253	C68H9N29O24	807.5182	-1.2	32.8	257	34.95	80.0	even	ok
	254	C66H2N45O6PS2	807.5201	1.1	32.8	258	43.94	90.0	even	ok
	255	C68H34N5NaO40S	807.5194	0.2	32.8	259	63.81	55.0	even	ok
	256	C77H148N12NaO18PS	807.5190	-0.3	32.8	260	61.69	11.0	even	ok
	257	C63H140N28NaO15PS	807.5199	0.8	32.8	261	49.82	9.0	even	ok
	258	C55H5N45O16S2	807.5196	0.4	33.1	262	58.06	77.0	even	ok
	259	C60H6N39NaO18S	807.5180	-1.5	33.3	263	37.01	78.0	even	ok
	260	C78H20N17NaO21P2	807.5191	-0.2	33.3	264	63.60	79.0	even	ok
	261	C70H4N41NaO5P2S	807.5201	1.1	33.3	265	44.52	91.0	even	ok
	262	C71H25N15O29S2	807.5187	-0.7	33.4	266	52.13	68.0	even	ok
	263	C53H122N52O4S2	807.5186	-0.8	33.4	267	50.85	20.0	even	ok
	264	C62H15N31NaO21PS	807.5202	1.2	33.5	268	41.90	72.0	even	ok
	265	C62H28N21NaO25P2S2	807.5184	-1.0	33.5	269	46.40	61.0	even	ok
	266	C72H6N31NaO18	807.5197	0.6	33.5	270	45.26	86.0	even	ok
	267	C71H139N20NaO20	807.5181	-1.4	33.7	271	31.28	13.0	even	ok
	268	C65H36N11NaO31P2S2	807.5191	-0.1	33.7	272	63.43	55.0	even	ok
	269	C78H149N10NaO19P2	807.5181	-1.4	33.8	273	32.31	11.0	even	ok
	270	C69H22N17O31P	807.5197	0.6	33.9	274	53.25	69.0	even	ok
	271	C79H31N5NaO30PS	807.5199	0.9	33.9	275	47.41	68.0	even	ok
	272	C69H13N29O19S2	807.5186	-0.7	33.9	276	51.34	79.0	even	ok

Compound Spectrum SmartFormula Report

Meas. m/z	#	Ion Formula	m/z	err [ppm]	mSigma	# mSigma	Score	rdb	e⁻ Conf	N-Rule
	273	C80H22N11O28P	807.5181	-1.4	33.9	277	38.59	77.0	even	ok
	274	C66H29N11O38S	807.5192	0.0	34.0	278	65.58	59.0	even	ok
	275	C78H151N8O22PS	807.5195	0.4	34.0	279	58.20	9.0	even	ok
	276	C54H2N49NaO12S2	807.5190	-0.2	34.0	280	61.16	79.0	even	ok
	277	C63H31N17O29P2S2	807.5190	-0.3	34.0	281	59.32	59.0	even	ok
	278	C64H146N18O26S	807.5183	-1.2	34.2	282	41.79	2.0	even	ok
	279	C71H143N18O21P	807.5201	1.1	34.2	283	36.18	11.0	even	ok
	280	C60H23N27O23P2S2	807.5183	-1.1	34.3	284	42.16	65.0	even	ok
	281	C70H138N26O13S2	807.5184	-1.1	34.3	285	43.67	16.0	even	ok
	282	C74H155N6NaO25S2	807.5185	-0.9	34.6	286	46.86	1.0	even	ok
	283	C72H135N24NaO16	807.5187	-0.6	34.6	287	43.75	18.0	even	ok
	284	C67H38N5O38PS2	807.5181	-1.3	34.7	288	38.33	53.0	even	ok
	285	C82H27N5NaO30P	807.5183	-1.2	34.7	289	40.97	73.0	even	ok
	286	C76H144N16O17P2	807.5180	-1.5	34.8	290	28.95	15.0	even	ok
	287	C65H26N15NaO34S	807.5187	-0.6	34.8	291	51.46	61.0	even	ok
	288	C68H7N39NaO8PS2	807.5203	1.3	34.8	292	38.49	86.0	even	ok
	289	C83H30NO34P	807.5188	-0.5	34.9	294	53.65	71.0	even	ok
	290	C66H155N10O29PS	807.5204	1.5	35.0	295	34.83	-4.0	even	ok
	291	C66H39N7O35P2S2	807.5196	0.5	35.0	296	53.61	53.0	even	ok
	292	C61H128N42NaO5PS	807.5199	0.8	35.0	297	39.36	20.0	even	ok
	293	C67H127N42PS2	807.5198	0.8	35.0	298	48.38	27.0	even	ok
	294	C61H135N34O13PS	807.5198	0.7	35.1	299	50.42	13.0	even	ok
	295	C69H15N25NaO23P	807.5198	0.8	35.1	300	40.19	76.0	even	ok
	296	C68H44NNaO37P2S2	807.5198	0.7	35.2	301	49.86	49.0	even	ok
	297	C52H119N56NaS2	807.5181	-1.4	35.4	302	36.06	22.0	even	ok
	298	C72H152N8NaO27P	807.5196	0.4	35.4	303	45.74	2.0	even	ok
	299	C60H10N37O19PS	807.5201	1.0	35.6	304	42.89	76.0	even	ok
	300	C68H2N37NaO16	807.5183	-1.1	35.6	305	35.05	87.0	even	ok
	301	C76H139N18NaO18	807.5201	1.1	35.6	306	35.25	17.0	even	ok
	302	C68H6N35O17P	807.5204	1.4	35.6	307	29.62	85.0	even	ok
	303	C72H159O35P	807.5194	0.3	35.7	308	57.80	-5.0	even	ok
	304	C71H3N39NaO8PS	807.5186	-0.8	35.7	309	47.64	91.0	even	ok
	305	C63H155N12NaO28S2	807.5201	1.1	35.8	310	41.61	-7.0	even	ok
	306	C80H156N2NaO24PS	807.5197	0.5	35.8	311	52.05	5.0	even	ok
	307	C54H128N48NaO3PS2	807.5202	1.3	35.9	312	38.33	16.0	even	ok
	308	C73H30N9NaO31S2	807.5188	-0.5	36.0	313	52.23	64.0	even	ok
	309	C72H6N35O12PS	807.5191	-0.1	36.0	314	60.46	89.0	even	ok
	310	C73H37NO39S2	807.5187	-0.7	36.2	315	48.70	57.0	even	ok
	311	C80H34NO34PS	807.5205	1.6	36.2	316	32.90	66.0	even	ok
	312	C71H34N3O41P	807.5197	0.6	36.3	317	49.87	58.0	even	ok
	313	C76H15N23O19P2	807.5189	-0.3	36.4	318	46.03	83.0	even	ok
	314	C71H18N23NaO21S2	807.5188	-0.5	36.4	319	51.49	75.0	even	ok
	315	C63H21N21O32S	807.5186	-0.8	36.4	320	46.08	65.0	even	ok
	316	C83H156O25P2	807.5200	1.0	36.4	321	43.14	8.0	even	ok
	317	C74H134N24O16	807.5199	0.9	36.4	322	37.17	21.0	even	ok
	318	C61H150N18O26S2	807.5200	0.9	36.5	323	44.07	-3.0	even	ok

Compound Spectrum SmartFormula Report

Meas. m/z	#	Ion Formula	m/z	err [ppm]	mSigma	# mSigma	Score	rdb	e⁻ Conf	N-Rule
	319	C60H147N22NaO22S2	807.5194	0.3	36.5	324	56.87	-1.0	even	ok
	320	C70H130N30O14	807.5186	-0.8	36.6	325	38.84	22.0	even	ok
	321	C78H25N9O31S	807.5183	-1.1	36.6	326	40.14	72.0	even	ok
	322	C65H152N14NaO25PS	807.5199	0.9	36.7	327	45.00	-2.0	even	ok
	323	C72H143N20NaO15S2	807.5185	-0.9	36.7	328	44.26	12.0	even	ok
	324	C69H132N36NaO2PS2	807.5200	1.0	36.9	329	42.90	23.0	even	ok
	325	C80H148N10O19P2	807.5193	0.1	37.0	330	58.46	14.0	even	ok
	326	C82H153N4NaO21P2	807.5195	0.3	37.2	331	54.80	10.0	even	ok
	327	C74H11N29NaO14PS	807.5193	0.1	37.3	332	59.40	85.0	even	ok
	328	C72H132N34O3P2S	807.5203	1.4	37.4	333	34.88	26.0	even	ok
	329	C58H142N28O20S2	807.5193	0.1	37.6	334	58.59	3.0	even	ok
	330	C59H123N48O3PS	807.5198	0.7	37.7	336	39.35	24.0	even	ok
	331	C67H10N31O21P	807.5197	0.6	37.8	337	40.15	80.0	even	ok
	332	C52H123N54OPS2	807.5201	1.1	37.9	338	39.35	20.0	even	ok
	333	C62H18N25NaO28S	807.5180	-1.5	37.9	339	32.92	67.0	even	ok
	334	C71H136N26NaO13P	807.5202	1.3	37.9	340	30.47	18.0	even	ok
	335	C75H158N2O29S2	807.5190	-0.2	38.0	341	55.19	-1.0	even	ok
	336	C59H7N41NaO15PS	807.5195	0.4	38.0	342	52.32	78.0	even	ok
	337	C70H147N14O25P	807.5194	0.3	38.1	343	45.33	6.0	even	ok
	338	C74H137N28NaO5P2S	807.5205	1.6	38.1	344	31.43	22.0	even	ok
	339	C65H165N4NaO29P2S2	807.5181	-1.3	38.2	345	35.00	-13.0	even	ok
	340	C57H139N32NaO16S2	807.5187	-0.6	38.2	346	48.18	5.0	even	ok
	341	C70H140N22NaO17P	807.5196	0.4	38.3	347	42.54	13.0	even	ok
	342	C75H14N25O18PS	807.5198	0.7	38.3	348	45.48	83.0	even	ok
	343	C79H150N6O25S	807.5180	-1.5	38.5	349	32.27	9.0	even	ok
	344	C71H156N4NaO31P	807.5189	-0.4	38.6	350	51.05	-3.0	even	ok
	345	C63H147N20O23PS	807.5198	0.7	38.6	351	45.81	2.0	even	ok
	346	C75H12N27NaO15P2	807.5184	-1.0	38.6	352	33.39	85.0	even	ok
	347	C70H31N7NaO37P	807.5192	-0.0	38.7	353	57.56	60.0	even	ok
	348	C64H27N17NaO31PS	807.5202	1.2	38.8	354	36.36	61.0	even	ok
	349	C66H168O33P2S2	807.5187	-0.7	38.8	355	45.87	-15.0	even	ok
	350	C80H30N3NaO33S	807.5184	-0.9	38.8	356	40.77	68.0	even	ok
	351	C63H160N10O27P2S2	807.5180	-1.5	38.9	357	31.57	-9.0	even	ok
	352	C83H152N2NaO24P	807.5180	-1.5	39.1	358	30.73	10.0	even	ok
	353	C73H131N28NaO12	807.5194	0.2	39.3	359	44.34	23.0	even	ok
	354	C73H7N33O13P2	807.5183	-1.2	39.5	360	30.25	89.0	even	ok
	355	C68H126N40O3S2	807.5184	-1.1	39.5	361	38.00	27.0	even	ok
	356	C73H146N16O19S2	807.5190	-0.2	39.5	362	52.87	10.0	even	ok
	357	C70H123N42PS	807.5182	-1.3	39.5	363	34.12	32.0	even	ok
	358	C74H33N5O35S2	807.5193	0.1	39.6	364	54.50	62.0	even	ok
	359	C72H21N19O25S2	807.5193	0.1	39.6	365	54.60	73.0	even	ok
	360	C55H134N38O14S2	807.5186	-0.8	39.7	366	43.27	9.0	even	ok
	361	C67H3N39NaO13P	807.5198	0.8	39.7	367	35.71	87.0	even	ok
	362	C69H6N37NaO11S2	807.5188	-0.5	39.8	368	46.87	86.0	even	ok
	363	C73H131N32O6PS	807.5188	-0.5	40.0	369	47.83	26.0	even	ok
	364	C77H19N19NaO20PS	807.5199	0.9	40.0	370	40.42	79.0	even	ok

Compound Spectrum SmartFormula Report

Meas. m/z	#	Ion Formula	m/z	err [ppm]	mSigma	# mSigma	Score	rdb	e¯ Conf	N-Rule
	365	C69H131N32O11P	807.5201	1.1	40.1	371	31.03	22.0	even	ok
	366	C72H128N36NaO2PS	807.5183	-1.1	40.1	372	36.32	28.0	even	ok
	367	C68H19N21NaO27P	807.5192	-0.0	40.3	373	45.84	71.0	even	ok
	368	C62H30N15NaO34S2	807.5204	1.4	40.3	374	31.19	56.0	even	ok
	369	C69H127N34NaO10	807.5181	-1.4	40.4	375	26.08	24.0	even	ok
	370	C59H22N25NaO28S2	807.5197	0.6	40.5	376	44.70	62.0	even	ok
	371	C81H155NaO27S	807.5182	-1.3	40.5	377	33.16	5.0	even	ok
	372	C66H158N4O36S	807.5183	-1.2	40.5	378	35.44	-9.0	even	ok
	373	C62H22N23O29PS	807.5201	1.0	40.5	379	37.46	65.0	even	ok
	374	C71H126N34O10	807.5193	0.1	40.6	380	45.39	27.0	even	ok
	375	C73H2N35NaO14	807.5204	1.4	40.7	381	26.16	91.0	even	ok
	376	C62H144N24NaO19PS	807.5192	0.0	40.8	382	54.79	4.0	even	ok
	377	C60H25N21O32S2	807.5202	1.3	40.8	383	33.41	60.0	even	ok
	378	C68H135N28O15P	807.5194	0.2	40.9	384	42.17	17.0	even	ok
	379	C54H131N42NaO10S2	807.5181	-1.4	40.9	385	31.23	11.0	even	ok
	380	C68H26N13O35P	807.5190	-0.2	41.0	386	51.07	64.0	even	ok
	381	C75H136N26NaO8PS	807.5190	-0.3	41.1	387	49.44	22.0	even	ok
	382	C57H17N31O26S2	807.5196	0.4	41.4	388	46.58	66.0	even	ok
	383	C60H132N38NaO9PS	807.5192	0.0	41.4	389	44.99	15.0	even	ok
	384	C80H19N17O21P2	807.5203	1.3	41.4	390	32.13	82.0	even	ok
	385	C70H131N34NaO5S2	807.5185	-0.9	41.5	391	38.71	23.0	even	ok
	386	C78H22N15O24PS	807.5205	1.6	41.6	392	28.54	77.0	even	ok
	387	C76H139N22O12PS	807.5195	0.4	41.6	393	47.59	20.0	even	ok
	388	C56H14N35NaO22S2	807.5190	-0.2	41.6	394	50.03	68.0	even	ok
	389	C66H7N35NaO17P	807.5192	-0.1	41.8	395	43.93	82.0	even	ok
	390	C79H145N14NaO15P2	807.5188	-0.5	41.9	396	37.02	16.0	even	ok
	391	C69H144N18NaO21P	807.5189	-0.4	41.9	397	38.71	8.0	even	ok
	392	C56H140N34NaO13PS2	807.5202	1.3	41.9	398	32.43	5.0	even	ok
	393	C75H151N10NaO21S2	807.5192	-0.1	42.0	399	52.28	6.0	even	ok
	394	C82H24N11NaO23P2	807.5204	1.5	42.1	400	29.00	78.0	even	ok
	395	C74H26N13NaO27S2	807.5195	0.3	42.2	401	47.85	69.0	even	ok
	396	C61H19N27NaO25PS	807.5195	0.4	42.2	402	46.50	67.0	even	ok
	397	C77H140N20O13P2	807.5187	-0.7	42.3	403	34.25	20.0	even	ok
	398	C70H9N33O15S2	807.5193	0.1	42.4	404	50.66	84.0	even	ok
	399	C67H38NNaO44S	807.5187	-0.6	42.5	405	41.98	50.0	even	ok
	400	C76H13N23O21S	807.5183	-1.1	42.6	406	33.99	83.0	even	ok
	401	C64H40N7NaO35P2S2	807.5184	-1.0	42.7	407	36.35	50.0	even	ok
	402	C67H164NaO35PS	807.5199	0.9	42.8	408	37.96	-13.0	even	ok
	403	C54H9N41O20S2	807.5189	-0.4	42.9	409	45.45	72.0	even	ok
	404	C60H139N30O17PS	807.5191	-0.2	42.9	410	49.34	8.0	even	ok
	405	C66H14N27O25P	807.5190	-0.2	43.0	411	40.10	75.0	even	ok
	406	C78H144N16NaO14PS	807.5196	0.5	43.1	412	42.80	16.0	even	ok
	407	C62H35N13O33P2S2	807.5183	-1.1	43.3	413	33.16	54.0	even	ok
	408	C65H43N3O39P2S2	807.5190	-0.3	43.5	414	45.99	48.0	even	ok
	409	C54H135N40O11PS2	807.5201	1.1	43.6	415	33.51	9.0	even	ok
	410	C78H10N25O18P	807.5181	-1.4	43.6	416	29.61	88.0	even	ok

Compound Spectrum SmartFormula Report

Meas. m/z	#	Ion Formula	m/z	err [ppm]	mSigma	# mSigma	Score	rdb	e⁻ Conf	N-Rule
	411	C71H134N30O9S2	807.5190	-0.2	43.7	417	46.97	21.0	even	ok
	412	C53H6N45NaO16S2	807.5184	-1.1	43.7	418	34.06	74.0	even	ok
	413	C65H33N7O42S	807.5186	-0.8	43.7	419	37.81	54.0	even	ok
	414	C77H39NO32P2S2	807.5180	-1.5	43.7	420	28.29	61.0	even	ok
	415	C70H123N38NaO6	807.5187	-0.6	43.9	421	33.92	29.0	even	ok
	416	C57H2N47O13PS	807.5194	0.2	43.9	422	39.37	82.0	even	ok
	417	C81H18N15O24P	807.5188	-0.5	44.0	423	41.87	82.0	even	ok
	418	C58H127N44O7PS	807.5191	-0.2	44.0	424	39.75	19.0	even	ok
	419	C68H128N36NaO7P	807.5196	0.4	44.2	425	36.20	24.0	even	ok
	420	C80H15N19NaO20P	807.5183	-1.2	44.2	426	31.56	84.0	even	ok
	421	C59H14N33O23PS	807.5194	0.2	44.2	427	46.74	71.0	even	ok
	422	C79H147N12O18PS	807.5202	1.2	44.3	428	31.41	14.0	even	ok
	423	C65H159N6O33PS	807.5198	0.7	44.4	429	38.87	-9.0	even	ok
	424	C64H2N41O15P	807.5190	-0.2	44.5	430	38.37	86.0	even	ok
	425	C78H18N17NaO23S	807.5184	-1.0	44.5	431	34.69	79.0	even	ok
	426	C64H30N11NaO38S	807.5180	-1.5	44.5	432	27.46	56.0	even	ok
	427	C76H137N24NaO9P2	807.5181	-1.4	44.6	433	24.01	22.0	even	ok
	428	C67H139N24O19P	807.5187	-0.6	44.7	434	33.56	12.0	even	ok
	429	C69H151N10O29P	807.5188	-0.6	44.7	435	33.63	1.0	even	ok
	430	C72H14N27NaO17S2	807.5195	0.3	44.8	436	44.52	80.0	even	ok
	431	C67H132N32NaO11P	807.5189	-0.4	45.0	437	35.42	19.0	even	ok
	432	C83H23N9NaO26P	807.5189	-0.4	45.0	438	43.36	78.0	even	ok
	433	C53H132N44NaO7PS2	807.5196	0.4	45.1	439	42.01	11.0	even	ok
	434	C76H154N6O25S2	807.5197	0.6	45.1	440	39.34	4.0	even	ok
	435	C62H159N8NaO32S2	807.5194	0.3	45.3	442	44.47	-12.0	even	ok
	436	C84H26N5O30P	807.5195	0.3	45.4	443	43.68	76.0	even	ok
	437	C75H27N15O22P2S2	807.5180	-1.5	45.4	444	26.90	72.0	even	ok
	438	C74H132N30O7P2	807.5180	-1.5	45.5	445	21.54	26.0	even	ok
	439	C68H118N44O4	807.5186	-0.8	45.5	446	30.22	33.0	even	ok
	440	C77H138N20O15S	807.5180	-1.5	45.6	447	26.37	20.0	even	ok
	441	C75H29N9O31S2	807.5200	1.0	45.6	448	33.54	67.0	even	ok
	442	C63H162N4O36S2	807.5200	0.9	45.7	449	34.05	-14.0	even	ok
	443	C77H11N27O15P2	807.5196	0.5	45.8	450	33.67	88.0	even	ok
	444	C66H39N3NaO41PS	807.5202	1.2	45.9	451	29.69	50.0	even	ok
	445	C64H156N10NaO29PS	807.5192	0.0	45.9	452	47.28	-7.0	even	ok
	446	C73H139N24NaO11S2	807.5192	-0.1	46.0	453	46.62	17.0	even	ok
	447	C79H16N21NaO17P2	807.5197	0.7	46.0	454	31.30	84.0	even	ok
	448	C60H154N14O30S2	807.5193	0.1	46.1	455	46.03	-8.0	even	ok
	449	C81H152N6NaO20PS	807.5203	1.4	46.1	456	27.47	10.0	even	ok
	450	C70H160NaO35P	807.5182	-1.2	46.2	457	29.35	-8.0	even	ok
	451	C59H151N18NaO26S2	807.5188	-0.6	46.3	458	38.54	-6.0	even	ok
	452	C58H11N37NaO19PS	807.5188	-0.5	46.3	459	40.20	73.0	even	ok
	453	C69H124N40NaO3P	807.5202	1.2	46.4	460	24.09	29.0	even	ok
	454	C79H21N13O27S	807.5190	-0.3	46.4	461	42.48	77.0	even	ok
	455	C53H10N43O17PS2	807.5204	1.5	46.4	462	26.09	72.0	even	ok
	456	C73H2N39O8PS	807.5198	0.7	46.5	463	36.24	94.0	even	ok

Compound Spectrum SmartFormula Report

Meas. m/z	#	Ion Formula	m/z	err [ppm]	mSigma	# mSigma	Score	rdb	e¯ Conf	N-Rule
	457	C66H123N42O5P	807.5194	0.2	46.5	464	35.96	28.0	even	ok
	458	C74H127N32NaO8	807.5201	1.1	46.7	465	26.01	28.0	even	ok
	459	C65H11N31NaO21P	807.5185	-0.9	46.7	466	28.02	77.0	even	ok
	460	C83H149N8NaO17P2	807.5201	1.1	46.8	467	29.99	15.0	even	ok
	461	C51H127N50O5PS2	807.5194	0.3	46.9	468	42.34	15.0	even	ok
	462	C69H35N3NaO41P	807.5185	-0.9	47.1	469	33.39	55.0	even	ok
	463	C79H143N14NaO17S	807.5182	-1.3	47.3	470	27.24	16.0	even	ok
	464	C64H34N9O39PS	807.5201	1.0	47.3	471	30.76	54.0	even	ok
	465	C68H145N20NaO18P2	807.5204	1.4	47.4	472	21.29	8.0	even	ok
	466	C76H8N31NaO11P2	807.5191	-0.2	47.4	473	35.92	90.0	even	ok
	467	C72H122N38O6	807.5199	0.9	47.4	474	27.45	32.0	even	ok
	468	C57H146N24O24S2	807.5186	-0.7	47.4	475	34.80	-2.0	even	ok
	469	C57H124N48NaO3PS	807.5186	-0.8	47.5	476	28.06	21.0	even	ok
	470	C52H7N47NaO13PS2	807.5199	0.8	47.5	477	33.96	74.0	even	ok
	471	C70H157N6NaO28P2	807.5204	1.5	47.6	478	21.09	-3.0	even	ok
	472	C74H3N37O9P2	807.5189	-0.3	47.6	479	33.55	94.0	even	ok
	473	C65H127N38O9P	807.5187	-0.6	47.6	480	30.70	23.0	even	ok
	474	C73H17N23O21S2	807.5200	1.0	47.7	481	31.61	78.0	even	ok
	475	C67H23N17NaO31P	807.5185	-0.9	47.7	482	27.27	66.0	even	ok
	476	C62H151N16O27PS	807.5191	-0.1	47.8	483	42.99	-3.0	even	ok
	477	C78H159NaO27S2	807.5198	0.8	47.8	484	33.98	0.0	even	ok
	478	C75H7N33NaO10PS	807.5199	0.9	47.9	485	32.38	90.0	even	ok
	479	C77H32N9NaO24P2S2	807.5182	-1.3	48.0	486	27.07	68.0	even	ok
	480	C56H143N28NaO20S2	807.5181	-1.4	48.0	487	25.49	0.0	even	ok
	481	C68H148N14NaO25P	807.5182	-1.2	48.1	488	23.02	3.0	even	ok
	482	C77H34N3NaO33S2	807.5201	1.1	48.3	489	28.71	63.0	even	ok
	483	C67H119N46OP	807.5201	1.1	48.3	490	24.62	33.0	even	ok
	484	C63H31N13NaO35PS	807.5195	0.4	48.4	491	38.79	56.0	even	ok
	485	C56H6N43O17PS	807.5187	-0.6	48.5	492	35.33	77.0	even	ok
	486	C74H142N20O15S2	807.5197	0.6	48.5	493	35.72	15.0	even	ok
	487	C82H143N12O18P	807.5185	-0.9	48.5	494	31.73	19.0	even	ok
	488	C66H136N28NaO15P	807.5182	-1.2	48.6	495	22.62	14.0	even	ok
	489	C81H26N7NaO29S	807.5191	-0.1	48.6	496	42.24	73.0	even	ok
	490	C50H124N54NaOPS2	807.5189	-0.4	48.8	497	38.18	17.0	even	ok
	491	C80H146N10O21S	807.5187	-0.7	48.8	498	34.63	14.0	even	ok
	492	C76H10N29O14PS	807.5205	1.6	48.9	499	23.20	88.0	even	ok
	493	C85H25N3O33	807.5180	-1.5	49.2	500	23.01	76.0	even	ok
	494	C67H115N48Na	807.5181	-1.4	49.2	501	20.19	35.0	even	ok
	495	C50H2N53O11PS2	807.5197	0.6	49.2	502	34.61	78.0	even	ok
	496	C67H30N9O39P	807.5184	-1.0	49.3	503	29.10	59.0	even	ok
	497	C73H15N29O12P2S2	807.5180	-1.5	49.3	504	23.84	83.0	even	ok
	498	C84H148N6NaO20P	807.5186	-0.7	49.4	505	33.22	15.0	even	ok
	499	C85H151N2O24P	807.5192	-0.1	49.4	506	42.17	13.0	even	ok
	500	C63H6N37O19P	807.5184	-1.1	49.5	507	23.96	81.0	even	ok
829.5333	1	C57H19N37NaO20PS2	829.5336	0.4	15.7	1	89.04	68.0	even	ok
	2	C59H147N30O18PS2	829.5339	0.7	15.7	2	79.40	3.0	even	ok

Compound Spectrum SmartFormula Report

Meas. m/z	#	Ion Formula	m/z	err [ppm]	mSigma	# mSigma	Score	rdb	e¯ Conf	N-Rule
	3	C68H43N3NaO42PS	829.5333	-0.0	16.0	3	99.51	50.0	even	ok
	4	C55H14N43O18PS2	829.5335	0.2	16.1	4	94.04	72.0	even	ok
	5	C58H144N34NaO14PS2	829.5334	0.0	16.1	5	100.00	5.0	even	ok
	6	C69H168NaO36PS	829.5330	-0.4	16.1	6	87.67	-13.0	even	ok
	7	C61H152N24NaO20PS2	829.5340	0.8	16.2	7	73.35	-1.0	even	ok
	8	C58H22N33O24PS2	829.5342	1.0	16.3	8	67.65	66.0	even	ok
	9	C66H38N9O40PS	829.5332	-0.2	16.4	9	93.14	54.0	even	ok
	10	C56H139N40O12PS2	829.5332	-0.2	16.7	10	94.30	9.0	even	ok
	11	C67H163N6O34PS	829.5329	-0.6	16.8	11	81.05	-9.0	even	ok
	12	C67H34N13O36PS	829.5338	0.6	16.9	12	79.30	59.0	even	ok
	13	C60H27N27NaO26PS2	829.5343	1.2	17.0	13	61.72	62.0	even	ok
	14	C54H11N47NaO14PS2	829.5330	-0.4	17.0	14	84.08	74.0	even	ok
	15	C60H146N28O21S2	829.5324	-1.1	17.2	15	62.46	3.0	even	ok
	16	C56H10N47O14PS2	829.5342	1.0	17.2	16	66.54	77.0	even	ok
	17	C60H156N20NaO24PS2	829.5334	0.0	17.2	17	97.47	-6.0	even	ok
	18	C69H39N7NaO38PS	829.5340	0.8	17.4	18	73.31	55.0	even	ok
	19	C65H35N13NaO36PS	829.5326	-0.8	17.4	19	70.92	56.0	even	ok
	20	C66H31N17NaO32PS	829.5333	-0.0	17.6	20	96.19	61.0	even	ok
	21	C58H151N26O22PS2	829.5332	-0.1	17.6	21	92.65	-2.0	even	ok
	22	C58H15N41NaO16PS2	829.5343	1.2	17.7	22	60.92	73.0	even	ok
	23	C55H7N51NaO10PS2	829.5336	0.4	17.8	23	85.59	79.0	even	ok
	24	C59H21N31O27S2	829.5327	-0.8	17.8	24	71.97	66.0	even	ok
	25	C61H159N16O28PS2	829.5339	0.7	18.0	25	75.65	-8.0	even	ok
	26	C64H26N23O30PS	829.5332	-0.2	18.1	26	89.81	65.0	even	ok
	27	C53H2N57O8PS2	829.5335	0.2	18.2	27	90.17	83.0	even	ok
	28	C52H6N53O12PS2	829.5328	-0.6	18.3	28	76.75	78.0	even	ok
	29	C57H148N30NaO18PS2	829.5327	-0.8	18.3	29	71.37	0.0	even	ok
	30	C62H151N22NaO23S2	829.5325	-1.0	18.4	30	65.77	-1.0	even	ok
	31	C55H136N44NaO8PS2	829.5327	-0.8	18.4	31	71.00	11.0	even	ok
	32	C63H164N10NaO30PS2	829.5340	0.8	18.5	32	69.76	-12.0	even	ok
	33	C66H160N10NaO30PS	829.5323	-1.2	18.6	33	59.14	-7.0	even	ok
	34	C68H33N11O39S	829.5323	-1.2	18.6	34	59.04	59.0	even	ok
	35	C63H30N19O34PS	829.5325	-1.0	18.7	35	64.14	60.0	even	ok
	36	C62H158N14O31S2	829.5324	-1.1	18.9	36	60.43	-8.0	even	ok
	37	C57H9N45O17S2	829.5327	-0.8	19.1	37	69.97	77.0	even	ok
	38	C66H164N8O31P2S	829.5344	1.2	19.1	38	57.12	-9.0	even	ok
	39	C61H26N25NaO29S2	829.5328	-0.6	19.2	39	74.93	62.0	even	ok
	40	C68H159N10O30PS	829.5335	0.3	19.5	40	85.85	-4.0	even	ok
	41	C55H143N36O16PS2	829.5325	-1.0	19.5	41	64.65	4.0	even	ok
	42	C70H164N4NaO32PS	829.5337	0.4	19.7	42	80.39	-8.0	even	ok
	43	C71H167O36PS	829.5342	1.1	19.7	43	61.43	-10.0	even	ok
	44	C70H38N5NaO41S	829.5325	-1.0	19.8	44	62.28	55.0	even	ok
	45	C53H131N50O6PS2	829.5325	-1.0	19.8	45	64.12	15.0	even	ok
	46	C53H12N49NaO11P2S2	829.5345	1.4	19.8	46	53.40	74.0	even	ok
	47	C57H135N44O8PS2	829.5339	0.7	19.9	47	73.01	14.0	even	ok
	48	C63H23N27NaO26PS	829.5326	-0.9	20.0	48	67.05	67.0	even	ok

Compound Spectrum SmartFormula Report

Meas. m/z	#	Ion Formula	m/z	err [ppm]	mSigma	# mSigma	Score	rdb	e⁻ Conf	N-Rule
	49	C64H155N16O28PS	829.5322	-1.4	20.0	49	52.89	-3.0	even	ok
	50	C59H31N23NaO30PS2	829.5336	0.4	20.0	50	81.22	57.0	even	ok
	51	C56H23N33NaO24PS2	829.5330	-0.4	20.1	51	79.12	63.0	even	ok
	52	C64H163N8NaO33S2	829.5325	-1.0	20.1	52	63.60	-12.0	even	ok
	53	C59H140N38NaO10PS2	829.5340	0.8	20.1	53	67.72	10.0	even	ok
	54	C66H167N2O38PS	829.5322	-1.4	20.2	54	52.88	-14.0	even	ok
	55	C57H26N29O28PS2	829.5335	0.2	20.2	55	86.11	61.0	even	ok
	56	C59H14N39NaO19S2	829.5328	-0.6	20.3	56	73.11	73.0	even	ok
	57	C64H36N15NaO33P2S	829.5341	1.0	20.3	57	63.69	56.0	even	ok
	58	C63H154N18O27S2	829.5331	-0.3	20.3	58	81.93	-3.0	even	ok
	59	C51H3N57NaO8PS2	829.5323	-1.3	20.4	59	55.25	80.0	even	ok
	60	C67H156N14NaO26PS	829.5330	-0.4	20.7	60	79.45	-2.0	even	ok
	61	C54H18N39O22PS2	829.5328	-0.6	21.0	61	72.63	67.0	even	ok
	62	C56H132N48NaO4PS2	829.5333	0.0	21.1	62	90.24	16.0	even	ok
	63	C54H137N46NaO5P2S2	829.5342	1.0	21.2	63	61.14	11.0	even	ok
	64	C61H18N33O24PS	829.5325	-1.0	21.2	64	60.60	71.0	even	ok
	65	C56H149N32NaO15P2S2	829.5342	1.0	21.3	65	60.78	0.0	even	ok
	66	C60H34N19O34PS2	829.5342	1.0	21.3	66	60.62	55.0	even	ok
	67	C71H41NO45S	829.5330	-0.4	21.4	67	78.47	53.0	even	ok
	68	C58H134N42O11S2	829.5324	-1.1	21.4	68	56.99	14.0	even	ok
	69	C65H151N20O24PS	829.5329	-0.6	21.4	69	73.42	2.0	even	ok
	70	C65H161N12NaO27P2S	829.5338	0.6	21.5	70	71.96	-7.0	even	ok
	71	C51H7N55O9P2S2	829.5343	1.2	21.5	71	55.83	78.0	even	ok
	72	C71H163N2NaO35S	829.5322	-1.4	21.6	72	50.68	-8.0	even	ok
	73	C54H127N54O2PS2	829.5332	-0.2	21.7	73	84.69	20.0	even	ok
	74	C65H42N5O44PS	829.5325	-1.0	21.7	74	60.28	49.0	even	ok
	75	C62H29N21O33S2	829.5334	0.0	21.8	75	88.63	60.0	even	ok
	76	C60H17N35O23S2	829.5333	0.0	21.9	76	88.58	71.0	even	ok
	77	C62H39N13NaO36PS2	829.5343	1.2	22.0	77	55.32	51.0	even	ok
	78	C53H15N43NaO18PS2	829.5323	-1.3	22.0	78	53.51	69.0	even	ok
	79	C62H31N21O31P2S	829.5340	0.8	22.0	79	65.98	60.0	even	ok
	80	C65H159N12NaO29S2	829.5332	-0.2	22.2	80	83.67	-7.0	even	ok
	81	C60H139N36NaO13S2	829.5325	-1.0	22.3	82	60.36	10.0	even	ok
	82	C61H33N17O37S2	829.5327	-0.8	22.3	83	65.63	55.0	even	ok
	83	C62H24N29NaO23P2S	829.5341	0.9	22.3	84	61.17	67.0	even	ok
	84	C55H24N35NaO21P2S2	829.5345	1.4	22.7	85	50.00	63.0	even	ok
	85	C65H166N4O37S2	829.5331	-0.3	22.7	86	77.99	-14.0	even	ok
	86	C65H22N27O26PS	829.5338	0.6	22.9	87	69.92	70.0	even	ok
	87	C64H152N22O21P2S	829.5344	1.2	23.0	88	52.66	2.0	even	ok
	88	C54H144N38O13P2S2	829.5340	0.8	23.0	89	63.10	4.0	even	ok
	89	C52H132N52O3P2S2	829.5340	0.8	23.0	90	63.27	15.0	even	ok
	90	C61H142N32O17S2	829.5331	-0.3	23.1	91	76.90	8.0	even	ok
	91	C67H27N21NaO28PS	829.5340	0.8	23.2	92	64.92	66.0	even	ok
	92	C63H156N18O25P2S	829.5337	0.4	23.3	93	73.79	-3.0	even	ok
	93	C66H48NNaO43P2S	829.5341	1.0	23.5	94	59.25	45.0	even	ok
	94	C63H38N11NaO39S2	829.5328	-0.6	23.5	95	68.48	51.0	even	ok

Compound Spectrum SmartFormula Report

Meas. m/z	#	Ion Formula	m/z	err [ppm]	mSigma	# mSigma	Score	rdb	e⁻ Conf	N-Rule
	95	C59H160N16NaO28PS2	829.5327	-0.8	23.5	96	63.94	-11.0	even	ok
	96	C62H168N6NaO34PS2	829.5334	0.0	23.6	97	84.82	-17.0	even	ok
	97	C64H148N24NaO20PS	829.5323	-1.2	23.6	98	52.86	4.0	even	ok
	98	C64H34N15NaO35S2	829.5335	0.2	23.6	99	80.23	56.0	even	ok
	99	C65H168N4O35P2S	829.5337	0.4	23.7	100	73.06	-14.0	even	ok
	100	C62H22N29NaO25S2	829.5335	0.2	23.7	101	80.35	67.0	even	ok
	101	C60H163N12O32PS2	829.5332	-0.1	23.8	102	81.30	-13.0	even	ok
	102	C56H3N55NaO6PS2	829.5343	1.2	23.8	103	53.60	84.0	even	ok
	103	C60H19N35O21P2S	829.5340	0.8	24.0	104	63.33	71.0	even	ok
	104	C53H19N41O19P2S2	829.5343	1.2	24.1	105	52.59	67.0	even	ok
	105	C50H4N59NaO5P2S2	829.5338	0.5	24.1	106	69.63	80.0	even	ok
	106	C71H43NO43P2	829.5337	0.4	24.2	107	73.92	53.0	even	ok
	107	C64H19N31NaO22PS	829.5333	-0.0	24.2	108	83.15	72.0	even	ok
	108	C73H38N3O42P	829.5328	-0.6	24.3	109	67.28	58.0	even	ok
	109	C57H155N22O26PS2	829.5325	-1.0	24.4	110	58.31	-7.0	even	ok
	110	C66H21N25O29S	829.5323	-1.2	24.5	111	51.78	70.0	even	ok
	111	C61H28N25NaO27P2S	829.5335	0.1	24.6	112	79.82	62.0	even	ok
	112	C63H147N26NaO19S2	829.5332	-0.2	24.7	113	78.92	4.0	even	ok
	113	C62H14N37O20PS	829.5332	-0.2	24.7	114	77.60	76.0	even	ok
	114	C64H43N7O41P2S	829.5340	0.8	24.9	115	61.78	49.0	even	ok
	115	C66H162N8O33S2	829.5337	0.5	24.9	116	70.02	-9.0	even	ok
	116	C62H143N30O18PS	829.5322	-1.4	24.9	117	47.35	8.0	even	ok
	117	C63H171N2O38PS2	829.5339	0.7	24.9	118	64.89	-19.0	even	ok
	118	C53H141N42NaO9P2S2	829.5335	0.2	25.2	119	77.18	6.0	even	ok
	119	C68H26N19NaO31S	829.5325	-1.0	25.3	120	54.97	66.0	even	ok
	120	C64H170O41S2	829.5324	-1.1	25.3	121	52.63	-19.0	even	ok
	121	C72H39N5O39P2	829.5343	1.2	25.5	122	51.11	58.0	even	ok
	122	C52H16N45NaO15P2S2	829.5338	0.5	25.6	123	67.02	69.0	even	ok
	123	C63H149N26NaO17P2S	829.5338	0.6	25.8	124	65.54	4.0	even	ok
	124	C64H165N8NaO31P2S	829.5332	-0.2	25.8	125	75.98	-12.0	even	ok
	125	C69H29N15O35S	829.5330	-0.4	25.9	126	70.77	64.0	even	ok
	126	C57H2N53NaO9S2	829.5328	-0.6	26.0	127	64.17	84.0	even	ok
	127	C63H25N25O29S2	829.5340	0.8	26.1	128	59.24	65.0	even	ok
	128	C58H161N18NaO25P2S2	829.5342	1.0	26.1	129	54.40	-11.0	even	ok
	129	C64H41N7O43S2	829.5334	0.0	26.3	130	79.87	49.0	even	ok
	130	C62H153N22NaO21P2S	829.5332	-0.2	26.3	131	75.04	-1.0	even	ok
	131	C63H40N11NaO37P2S	829.5335	0.1	26.4	132	76.35	51.0	even	ok
	132	C64H150N22O23S2	829.5337	0.5	26.5	133	67.64	2.0	even	ok
	133	C72H35N7NaO38P	829.5323	-1.3	26.5	134	48.17	60.0	even	ok
	134	C69H155N14O26PS	829.5342	1.1	26.5	135	52.91	1.0	even	ok
	135	C58H5N49O13S2	829.5333	0.0	26.6	136	79.76	82.0	even	ok
	136	C59H23N31O25P2S	829.5333	-0.0	26.6	137	79.24	66.0	even	ok
	137	C67H40N11NaO32P2S2	829.5322	-1.4	26.6	138	45.77	55.0	even	ok
	138	C65H37N11O39S2	829.5340	0.8	26.7	139	58.22	54.0	even	ok
	139	C61H11N41NaO16PS	829.5326	-0.9	26.8	140	57.37	78.0	even	ok
	140	C68H167N2NaO35S2	829.5339	0.7	27.0	141	62.33	-13.0	even	ok

Compound Spectrum SmartFormula Report

Meas. m/z	#	Ion Formula	m/z	err [ppm]	mSigma	# mSigma	Score	rdb	e⁻ Conf	N-Rule
	141	C59H16N39NaO17P2S	829.5335	0.1	27.1	142	75.50	73.0	even	ok
	142	C58H35N19NaO34PS2	829.5330	-0.4	27.1	143	67.70	52.0	even	ok
	143	C66H147N24O20PS	829.5335	0.2	27.2	144	72.50	7.0	even	ok
	144	C51H136N48O7P2S2	829.5334	0.0	27.2	145	78.06	10.0	even	ok
	145	C68H152N18NaO22PS	829.5337	0.4	27.2	146	68.09	3.0	even	ok
	146	C71H160N8NaO28PS	829.5344	1.2	27.2	147	48.14	-3.0	even	ok
	147	C70H40N5NaO39P2	829.5331	-0.3	27.3	148	71.82	55.0	even	ok
	148	C71H34N9NaO37S	829.5332	-0.2	27.3	149	72.82	60.0	even	ok
	149	C56H156N24O23P2S2	829.5340	0.9	27.4	150	56.88	-7.0	even	ok
	150	C61H144N32O15P2S	829.5337	0.4	27.4	151	67.32	8.0	even	ok
	151	C50H11N51O13P2S2	829.5337	0.4	27.4	152	68.55	73.0	even	ok
	152	C56H30N25O32PS2	829.5328	-0.6	27.7	153	62.53	56.0	even	ok
	153	C55H27N29NaO28PS2	829.5323	-1.2	27.8	154	47.01	58.0	even	ok
	154	C62H160N14O29P2S	829.5330	-0.4	27.8	155	68.19	-8.0	even	ok
	155	C57H128N52NaPS2	829.5340	0.8	27.9	156	57.08	21.0	even	ok
	156	C59H6N47O14PS	829.5325	-1.0	27.9	157	51.97	82.0	even	ok
	157	C61H43N9NaO40PS2	829.5337	0.4	27.9	158	67.92	46.0	even	ok
	158	C59H38N15O38PS2	829.5335	0.2	27.9	159	72.21	50.0	even	ok
	159	C66H46NNaO45S2	829.5335	0.2	27.9	160	72.53	45.0	even	ok
	160	C60H10N43NaO15S2	829.5335	0.2	28.1	161	72.80	78.0	even	ok
	161	C61H35N17O35P2S	829.5333	-0.0	28.2	162	76.48	55.0	even	ok
	162	C71H36N9NaO35P2	829.5338	0.5	28.2	163	63.26	60.0	even	ok
	163	C65H30N19NaO31S2	829.5342	1.0	28.2	164	52.32	61.0	even	ok
	164	C60H148N28O19P2S	829.5330	-0.4	28.4	165	67.18	3.0	even	ok
	165	C60H12N43NaO13P2S	829.5341	0.9	28.4	166	53.32	78.0	even	ok
	166	C66H155N16NaO25S2	829.5339	0.6	28.5	167	60.41	-2.0	even	ok
	167	C55H153N28NaO19P2S2	829.5335	0.2	28.6	168	71.10	-5.0	even	ok
	168	C65H144N28NaO16PS	829.5330	-0.4	28.7	169	66.07	9.0	even	ok
	169	C72H37N5O41S	829.5337	0.4	28.8	170	65.51	58.0	even	ok
	170	C74H163O36P	829.5325	-1.0	28.9	171	52.19	-5.0	even	ok
	171	C67H42N5NaO41S2	829.5342	1.0	28.9	172	51.38	50.0	even	ok
	172	C69H151N16NaO25S	829.5322	-1.4	28.9	173	42.79	3.0	even	ok
	173	C70H154N12O29S	829.5327	-0.7	28.9	174	57.35	1.0	even	ok
	174	C67H34N9NaO42	829.5344	1.3	29.0	175	44.94	56.0	even	ok
	175	C69H168N4O30P2S2	829.5325	-1.1	29.0	176	49.75	-10.0	even	ok
	176	C56H122N56OS2	829.5324	-1.2	29.1	177	47.64	25.0	even	ok
	177	C57H11N45O15P2S	829.5333	-0.0	29.1	178	74.62	77.0	even	ok
	178	C59H147N26NaO24S	829.5344	1.3	29.1	179	43.62	0.0	even	ok
	179	C65H28N25NaO22P2S2	829.5322	-1.4	29.2	180	43.00	66.0	even	ok
	180	C63H139N34O14PS	829.5329	-0.6	29.3	181	61.08	13.0	even	ok
	181	C57H36N21NaO31P2S2	829.5345	1.4	29.3	182	42.77	52.0	even	ok
	182	C68H35N11O37P2	829.5330	-0.4	29.3	183	64.31	59.0	even	ok
	183	C62H46N5O44PS2	829.5342	1.0	29.5	184	50.19	44.0	even	ok
	184	C61H13N39O19S2	829.5340	0.8	29.5	185	54.76	76.0	even	ok
	185	C58H20N35NaO21P2S	829.5328	-0.7	29.6	186	58.17	68.0	even	ok
	186	C68H43N7O36P2S2	829.5327	-0.7	29.6	187	57.08	53.0	even	ok

Compound Spectrum SmartFormula Report

Meas. m/z	#	Ion Formula	m/z	err [ppm]	mSigma	# mSigma	Score	rdb	e¯ Conf	N-Rule
	187	C58H127N50NaO3S2	829.5325	-1.0	29.7	188	50.74	21.0	even	ok
	188	C49H8N55NaO9P2S2	829.5331	-0.3	29.7	189	67.92	75.0	even	ok
	189	C59H130N46O7S2	829.5331	-0.3	29.7	190	65.85	19.0	even	ok
	190	C69H31N15O33P2	829.5336	0.4	29.8	191	65.06	64.0	even	ok
	191	C73H164N2O33P2	829.5340	0.8	29.8	192	53.97	-5.0	even	ok
	192	C50H133N52NaO3P2S2	829.5328	-0.6	29.9	193	59.09	12.0	even	ok
	193	C63H45N3O47S2	829.5327	-0.8	29.9	194	55.14	44.0	even	ok
	194	C72H159N6NaO31S	829.5329	-0.6	30.0	195	59.76	-3.0	even	ok
	195	C69H44NNaO43P2	829.5324	-1.1	30.1	196	48.31	50.0	even	ok
	196	C53H148N34O17P2S2	829.5334	0.0	30.3	197	72.36	-1.0	even	ok
	197	C55H31N27O29P2S2	829.5343	1.2	30.3	198	45.32	56.0	even	ok
	198	C62H140N36O11P2S	829.5344	1.2	30.3	199	44.53	13.0	even	ok
	199	C60H32N21NaO31P2S	829.5328	-0.7	30.4	200	57.20	57.0	even	ok
	200	C71H165N2NaO33P2	829.5328	-0.6	30.4	201	58.27	-8.0	even	ok
	201	C61H157N18NaO25P2S	829.5325	-1.0	30.5	202	49.09	-6.0	even	ok
	202	C61H135N40NaO9S2	829.5332	-0.2	30.9	203	68.03	15.0	even	ok
	203	C74H34N7O38P	829.5335	0.2	30.9	204	67.68	63.0	even	ok
	204	C61H159N12NaO34S	829.5345	1.3	30.9	205	41.62	-11.0	even	ok
	205	C67H158N12O29S2	829.5344	1.3	30.9	206	42.79	-4.0	even	ok
	206	C54H28N31NaO25P2S2	829.5338	0.6	30.9	207	58.94	58.0	even	ok
	207	C73H162N2O35S	829.5334	0.1	30.9	208	70.43	-5.0	even	ok
	208	C64H172O39P2S	829.5330	-0.4	31.1	209	63.17	-19.0	even	ok
	209	C76H39NNaO40P	829.5336	0.4	31.2	210	63.21	59.0	even	ok
	210	C65H29N15O40	829.5343	1.1	31.2	211	46.12	60.0	even	ok
	211	C57H142N32O22S	829.5343	1.2	31.3	212	44.83	4.0	even	ok
	212	C68H159N6NaO36	829.5341	0.9	31.3	213	49.93	-7.0	even	ok
	213	C66H31N21O26P2S2	829.5327	-0.7	31.3	214	54.58	64.0	even	ok
	214	C67H41NO50	829.5343	1.1	31.4	215	45.78	49.0	even	ok
	215	C63H18N33NaO21S2	829.5342	1.0	31.4	216	48.60	72.0	even	ok
	216	C61H172N2NaO38PS2	829.5327	-0.8	31.5	217	53.13	-22.0	even	ok
	217	C63H10N41O16PS	829.5338	0.6	31.5	218	57.31	81.0	even	ok
	218	C62H136N38NaO10PS	829.5323	-1.2	31.6	219	43.67	15.0	even	ok
	219	C65H15N35NaO18PS	829.5340	0.8	31.6	220	53.37	77.0	even	ok
	220	C59H145N32NaO15P2S	829.5325	-1.0	31.6	221	47.62	5.0	even	ok
	221	C47H3N61O7P2S2	829.5330	-0.4	31.7	222	60.65	79.0	even	ok
	222	C62H138N36O13S2	829.5337	0.5	31.8	223	59.72	13.0	even	ok
	223	C56H15N41O19P2S	829.5326	-0.8	31.8	224	51.34	72.0	even	ok
	224	C70H48NNaO38P2S2	829.5329	-0.5	31.9	225	57.69	49.0	even	ok
	225	C59H167N8O36PS2	829.5326	-0.9	32.0	226	48.73	-18.0	even	ok
	226	C58H7N49O11P2S	829.5340	0.8	32.1	227	43.67	82.0	even	ok
	227	C48H128N58OP2S2	829.5327	-0.8	32.1	228	52.23	16.0	even	ok
	228	C52H145N38NaO13P2S2	829.5328	-0.6	32.1	229	56.09	1.0	even	ok
	229	C52H23N37O23P2S2	829.5337	0.4	32.3	230	60.72	62.0	even	ok
	230	C65H22N23NaO32	829.5344	1.3	32.3	231	41.52	67.0	even	ok
	231	C67H39N7O41P2	829.5323	-1.2	32.4	232	42.27	54.0	even	ok
	232	C73H31N11NaO34P	829.5330	-0.5	32.4	233	59.06	65.0	even	ok

Compound Spectrum SmartFormula Report

Meas. m/z	#	Ion Formula	m/z	err [ppm]	mSigma	# mSigma	Score	rdb	e¯ Conf	N-Rule
	233	C58H27N27O29P2S	829.5326	-0.8	32.5	234	50.67	61.0	even	ok
	234	C72H161N6NaO29P2	829.5335	0.2	32.5	235	64.87	-3.0	even	ok
	235	C67H156N18O20P2S2	829.5324	-1.1	32.6	236	45.42	1.0	even	ok
	236	C67H32N15NaO33P2	829.5324	-1.1	32.7	237	45.23	61.0	even	ok
	237	C72H32N13NaO31P2	829.5345	1.3	32.7	238	39.71	65.0	even	ok
	238	C59H152N24O23P2S	829.5324	-1.2	32.7	239	43.01	-2.0	even	ok
	239	C59H154N18O32S	829.5343	1.2	32.9	240	42.97	-7.0	even	ok
	240	C64H9N39O19S	829.5323	-1.2	32.9	241	42.26	81.0	even	ok
	241	C71H26N17O32P	829.5328	-0.6	32.9	242	54.61	69.0	even	ok
	242	C60H141N36NaO11P2S	829.5332	-0.2	32.9	243	53.14	10.0	even	ok
	243	C63H169N4NaO35P2S	829.5325	-1.0	32.9	244	46.36	-17.0	even	ok
	244	C62H7N45NaO12PS	829.5333	-0.1	33.0	245	67.20	83.0	even	ok
	245	C63H47N3O45P2S	829.5333	-0.0	33.2	246	67.76	44.0	even	ok
	246	C67H17N29O25S	829.5330	-0.4	33.3	247	58.99	75.0	even	ok
	247	C57H135N40NaO14S	829.5344	1.3	33.3	248	32.84	11.0	even	ok
	248	C55H14N39NaO24S	829.5341	0.9	33.4	249	48.57	69.0	even	ok
	249	C64H143N30NaO15S2	829.5339	0.6	33.4	250	53.66	9.0	even	ok
	250	C66H14N33NaO21S	829.5325	-1.0	33.4	251	45.07	77.0	even	ok
	251	C60H2N51O10PS	829.5332	-0.2	33.5	252	62.70	87.0	even	ok
	252	C66H154N12O34	829.5340	0.8	33.5	253	50.81	-3.0	even	ok
	253	C68H36N15NaO28P2S2	829.5329	-0.6	33.5	254	55.32	60.0	even	ok
	254	C60H173N4NaO35P2S2	829.5342	1.0	33.6	255	45.20	-22.0	even	ok
	255	C70H27N19O29P2	829.5343	1.2	33.6	256	42.09	69.0	even	ok
	256	C51H20N41NaO19P2S2	829.5331	-0.3	33.7	257	61.62	64.0	even	ok
	257	C66H38N5NaO46	829.5337	0.5	34.0	258	56.23	51.0	even	ok
	258	C50H140N44O11P2S2	829.5327	-0.8	34.1	259	49.82	5.0	even	ok
	259	C56H139N36NaO18S	829.5338	0.5	34.3	260	54.71	6.0	even	ok
	260	C69H22N23NaO27S	829.5331	-0.2	34.4	261	61.08	71.0	even	ok
	261	C64H26N19NaO36	829.5337	0.5	34.5	263	55.77	62.0	even	ok
	262	C47H127N56NaO6S2	829.5341	0.9	34.5	264	46.05	13.0	even	ok
	263	C58H168N10O33P2S2	829.5340	0.9	34.5	265	47.58	-18.0	even	ok
	264	C62H44N7NaO41P2S	829.5328	-0.7	34.5	266	51.69	46.0	even	ok
	265	C69H161N12NaO22P2S2	829.5326	-0.9	34.6	267	46.57	-3.0	even	ok
	266	C63H16N39NaO12P2S2	829.5322	-1.4	34.9	268	37.17	77.0	even	ok
	267	C56H8N49NaO11P2S	829.5328	-0.7	34.9	269	42.37	79.0	even	ok
	268	C61H164N10O33P2S	829.5324	-1.2	34.9	270	40.83	-13.0	even	ok
	269	C67H163N2NaO40	829.5334	0.1	34.9	271	62.48	-12.0	even	ok
	270	C57H165N14NaO29P2S2	829.5335	0.2	35.0	272	60.57	-16.0	even	ok
	271	C65H146N26O19S2	829.5344	1.3	35.0	273	38.71	7.0	even	ok
	272	C58H136N42O9P2S	829.5330	-0.4	35.0	274	47.38	14.0	even	ok
	273	C70H25N19O31S	829.5337	0.4	35.0	275	56.18	69.0	even	ok
	274	C46H2N59NaO12S2	829.5344	1.3	35.1	276	38.55	76.0	even	ok
	275	C70H23N21NaO28P	829.5323	-1.3	35.1	277	38.84	71.0	even	ok
	276	C60H131N44O8PS	829.5322	-1.4	35.2	278	30.64	19.0	even	ok
	277	C69H160N8O31P2	829.5327	-0.8	35.2	279	40.22	-4.0	even	ok
	278	C58H151N22NaO28S	829.5338	0.5	35.2	280	53.34	-5.0	even	ok

Compound Spectrum SmartFormula Report

Meas. m/z	#	Ion Formula	m/z	err [ppm]	mSigma	# mSigma	Score	rdb	e¯ Conf	N-Rule
	279	C67H143N28O16PS	829.5342	1.1	35.3	281	42.74	12.0	even	ok
	280	C75H43NO38P2S	829.5324	-1.1	35.4	282	41.21	57.0	even	ok
	281	C49H15N47O17P2S2	829.5330	-0.4	35.4	283	55.37	68.0	even	ok
	282	C68H28N19NaO29P2	829.5331	-0.3	35.5	284	48.77	66.0	even	ok
	283	C57H4N53NaO7P2S	829.5334	0.1	35.5	285	51.21	84.0	even	ok
	284	C61H137N40NaO7P2S	829.5338	0.6	35.6	286	43.02	15.0	even	ok
	285	C53H9N45O22S	829.5339	0.7	35.6	287	49.25	73.0	even	ok
	286	C55H130N46O12S	829.5343	1.2	35.7	288	33.49	15.0	even	ok
	287	C75H159N4O32P	829.5332	-0.2	35.7	289	60.47	0.0	even	ok
	288	C69H148N22NaO18PS	829.5344	1.2	35.8	290	39.05	8.0	even	ok
	289	C57H39N15NaO38PS2	829.5323	-1.2	35.8	291	38.64	47.0	even	ok
	290	C60H47N5NaO44PS2	829.5330	-0.4	35.9	292	54.61	41.0	even	ok
	291	C57H26N25NaO34S	829.5341	0.9	35.9	294	45.33	58.0	even	ok
	292	C69H39N11O32P2S2	829.5334	0.1	36.2	295	61.21	58.0	even	ok
	293	C64H19N35O16P2S2	829.5327	-0.7	36.2	296	48.06	75.0	even	ok
	294	C55H160N20O27P2S2	829.5334	0.0	36.2	297	62.07	-12.0	even	ok
	295	C58H42N11O42PS2	829.5328	-0.6	36.3	298	50.65	45.0	even	ok
	296	C64H33N11O44	829.5336	0.3	36.3	299	56.54	55.0	even	ok
	297	C60H39N13O39P2S	829.5327	-0.8	36.3	300	46.07	50.0	even	ok
	298	C66H140N32NaO12PS	829.5337	0.4	36.4	301	54.36	14.0	even	ok
	299	C64H135N38O10PS	829.5335	0.2	36.4	302	57.77	18.0	even	ok
	300	C57H140N38O13P2S	829.5324	-1.2	36.5	303	32.43	9.0	even	ok
	301	C72H30N13NaO33S	829.5338	0.6	36.5	304	50.61	65.0	even	ok
	302	C45H122N62O4S2	829.5340	0.8	36.7	305	46.79	17.0	even	ok
	303	C53H2N53NaO14S	829.5341	0.9	36.7	306	37.26	80.0	even	ok
	304	C70H164N8O26P2S2	829.5331	-0.3	36.8	307	56.77	-5.0	even	ok
	305	C70H156N12O27P2	829.5334	0.0	36.9	308	51.13	1.0	even	ok
	306	C61H50NO48PS2	829.5335	0.2	37.0	309	57.37	39.0	even	ok
	307	C59H132N46O5P2S	829.5337	0.4	37.1	310	44.14	19.0	even	ok
	308	C63H17N29O30	829.5343	1.1	37.1	311	33.12	71.0	even	ok
	309	C65H158N8O38	829.5333	-0.0	37.2	312	60.54	-8.0	even	ok
	310	C54H3N55O9P2S	829.5326	-0.8	37.2	313	37.13	83.0	even	ok
	311	C48H12N51NaO13P2S2	829.5325	-1.1	37.3	314	40.33	70.0	even	ok
	312	C54H157N24NaO23P2S2	829.5328	-0.6	37.3	315	49.23	-10.0	even	ok
	313	C68H142N26O19S	829.5327	-0.8	37.3	316	46.23	12.0	even	ok
	314	C56H146N28O26S	829.5336	0.4	37.4	317	53.76	-1.0	even	ok
	315	C61H6N47NaO11S2	829.5342	1.0	37.4	318	41.77	83.0	even	ok
	316	C66H23N25O27P2	829.5330	-0.4	37.4	319	43.53	70.0	even	ok
	317	C61H166N4O42S	829.5343	1.2	37.4	320	38.04	-18.0	even	ok
	318	C74H156N8NaO28P	829.5327	-0.8	37.5	321	45.03	2.0	even	ok
	319	C65H27N21O31P2	829.5323	-1.2	37.6	322	30.68	65.0	even	ok
	320	C59H48N7NaO41P2S2	829.5345	1.4	37.7	323	34.45	41.0	even	ok
	321	C67H139N30NaO15S	829.5322	-1.4	37.8	324	34.02	14.0	even	ok
	322	C73H33N9O37S	829.5344	1.2	37.9	325	36.88	63.0	even	ok
	323	C55H21N31O32S	829.5339	0.7	37.9	326	46.20	62.0	even	ok
	324	C72H151N14O26P	829.5325	-1.0	38.0	327	41.25	6.0	even	ok

Compound Spectrum SmartFormula Report

Meas. m/z	#	Ion Formula	m/z	err [ppm]	mSigma	# mSigma	Score	rdb	e¯ Conf	N-Rule
	325	C66H24N29NaO18P2S2	829.5329	-0.6	38.1	328	48.97	71.0	even	ok
	326	C49H139N42NaO16S2	829.5341	0.9	38.2	329	41.69	2.0	even	ok
	327	C70H147N20NaO21S	829.5329	-0.6	38.2	330	48.43	8.0	even	ok
	328	C57H43N13O39P2S2	829.5343	1.2	38.4	331	36.70	45.0	even	ok
	329	C71H150N16O25S	829.5334	0.1	38.4	332	58.24	6.0	even	ok
	330	C56H40N17NaO35P2S2	829.5338	0.6	38.4	333	48.49	47.0	even	ok
	331	C71H44N5NaO34P2S2	829.5336	0.3	38.5	334	54.17	54.0	even	ok
	332	C52H6N49NaO18S	829.5334	0.1	38.6	335	57.94	75.0	even	ok
	333	C57H133N46NaO5P2S	829.5325	-1.0	38.8	336	32.91	16.0	even	ok
	334	C55H123N54NaO4S	829.5344	1.3	38.8	337	28.52	22.0	even	ok
	335	C65H144N32O10P2S2	829.5324	-1.1	38.9	338	38.46	12.0	even	ok
	336	C52H152N30O21P2S2	829.5327	-0.8	38.9	339	44.01	-6.0	even	ok
	337	C66H147N20NaO26	829.5341	0.9	38.9	340	34.24	4.0	even	ok
	338	C72H169N2NaO28P2S2	829.5333	-0.1	39.0	341	56.74	-9.0	even	ok
	339	C60H163N8NaO38S	829.5338	0.5	39.0	342	48.11	-16.0	even	ok
	340	C63H30N15NaO40	829.5331	-0.3	39.2	343	51.75	57.0	even	ok
	341	C46H7N57O11P2S2	829.5323	-1.2	39.2	344	35.36	74.0	even	ok
	342	C69H24N23NaO25P2	829.5338	0.5	39.2	345	39.95	71.0	even	ok
	343	C60H126N50O3S2	829.5337	0.5	39.3	346	49.21	24.0	even	ok
	344	C75H30N11O34P	829.5342	1.0	39.3	347	39.43	68.0	even	ok
	345	C54H35N23O33P2S2	829.5337	0.4	39.5	348	50.27	51.0	even	ok
	346	C54H127N50NaO8S	829.5338	0.5	39.5	349	39.90	17.0	even	ok
	347	C54H134N42O16S	829.5336	0.4	39.6	351	42.31	10.0	even	ok
	348	C73H155N10NaO27S	829.5335	0.2	39.7	352	53.07	2.0	even	ok
	349	C62H21N25O34	829.5336	0.3	39.7	353	43.05	66.0	even	ok
	350	C75H38N3NaO39S	829.5345	1.4	39.8	354	32.31	59.0	even	ok
	351	C77H35N5NaO36P	829.5343	1.2	39.8	355	36.08	64.0	even	ok
	352	C65H151N16NaO30	829.5334	0.1	39.9	356	45.71	-1.0	even	ok
	353	C67H27N25O22P2S2	829.5334	0.1	40.0	357	55.32	69.0	even	ok
	354	C47H134N48O14S2	829.5340	0.8	40.0	358	42.60	6.0	even	ok
	355	C55H143N32NaO22S	829.5331	-0.3	40.1	359	51.59	1.0	even	ok
	356	C53H32N27NaO29P2S2	829.5331	-0.2	40.1	360	52.12	53.0	even	ok
	357	C48H14N45NaO22S2	829.5344	1.3	40.2	361	33.56	65.0	even	ok
	358	C54H18N35NaO28S	829.5334	0.1	40.2	362	55.26	64.0	even	ok
	359	C74H27N15NaO30P	829.5336	0.4	40.4	363	49.82	70.0	even	ok
	360	C69H153N16NaO23P2	829.5328	-0.6	40.4	364	37.41	3.0	even	ok
	361	C72H22N21O28P	829.5335	0.2	40.4	365	52.91	74.0	even	ok
	362	C67H149N26NaO12P2S2	829.5326	-0.9	40.5	366	39.66	8.0	even	ok
	363	C58H129N50NaOP2S	829.5332	-0.2	40.5	367	43.38	21.0	even	ok
	364	C62H131N44NaO5S2	829.5339	0.6	40.6	368	44.47	20.0	even	ok
	365	C74H158N6O31S	829.5341	0.9	40.6	369	40.17	0.0	even	ok
	366	C67H19N29O23P2	829.5336	0.4	40.6	370	41.04	75.0	even	ok
	367	C58H158N14O36S	829.5336	0.4	40.9	371	48.76	-12.0	even	ok
	368	C73H157N10NaO25P2	829.5342	1.0	40.9	372	31.40	2.0	even	ok
	369	C62H14N33NaO26	829.5337	0.5	41.0	373	39.17	73.0	even	ok
	370	C53H118N60O2S	829.5343	1.2	41.0	374	29.14	26.0	even	ok

Compound Spectrum SmartFormula Report

Meas. m/z	#	Ion Formula	m/z	err [ppm]	mSigma	# mSigma	Score	rdb	e¯ Conf	N-Rule
	371	C65H42NNaO50	829.5331	-0.3	41.0	375	49.35	46.0	even	ok
	372	C55H128N52O3P2S	829.5323	-1.2	41.0	376	28.62	20.0	even	ok
	373	C59H38N11NaO44S	829.5341	0.9	41.1	377	39.33	47.0	even	ok
	374	C64H142N26O24	829.5340	0.8	41.1	378	34.65	8.0	even	ok
	375	C63H3N49NaO8PS	829.5340	0.8	41.1	379	41.68	88.0	even	ok
	376	C65H20N29NaO23P2	829.5324	-1.1	41.1	380	30.05	72.0	even	ok
	377	C63H132N42NaO6PS	829.5330	-0.4	41.3	381	39.76	20.0	even	ok
	378	C76H29N9O37	829.5327	-0.8	41.3	382	40.53	68.0	even	ok
	379	C51H27N33O27P2S2	829.5330	-0.4	41.5	383	47.14	57.0	even	ok
	380	C63H134N40O9S2	829.5344	1.3	41.5	384	32.62	18.0	even	ok
	381	C63H10N37NaO22	829.5344	1.3	41.5	385	27.25	78.0	even	ok
	382	C72H47NO38P2S2	829.5341	0.9	41.5	386	38.65	52.0	even	ok
	383	C61H25N21O38	829.5329	-0.5	41.5	387	45.55	61.0	even	ok
	384	C61H127N48O4PS	829.5329	-0.6	41.8	388	36.73	24.0	even	ok
	385	C46H9N51O20S2	829.5343	1.1	41.9	389	34.70	69.0	even	ok
	386	C71H152N16O23P2	829.5340	0.8	41.9	390	32.90	6.0	even	ok
	387	C52H122N56O6S	829.5336	0.4	42.0	391	39.73	21.0	even	ok
	388	C68H152N22O16P2S2	829.5331	-0.3	42.0	392	49.16	6.0	even	ok
	389	C46H131N52NaO10S2	829.5334	0.1	42.1	393	51.49	8.0	even	ok
	390	C69H32N19NaO24P2S2	829.5335	0.3	42.2	394	49.16	65.0	even	ok
	391	C65H5N43O15S	829.5330	-0.4	42.2	395	46.45	86.0	even	ok
	392	C76H163NaO33S	829.5342	1.0	42.2	396	35.62	-4.0	even	ok
	393	C67H148N22O21P2	829.5327	-0.8	42.3	397	33.14	7.0	even	ok
	394	C73H31N15O28P2S	829.5324	-1.1	42.4	398	34.05	68.0	even	ok
	395	C53H138N38O20S	829.5330	-0.4	42.4	399	45.50	5.0	even	ok
	396	C60H128N50OP2S	829.5344	1.2	42.4	400	27.12	24.0	even	ok
	397	C52H13N41O26S	829.5333	-0.1	42.4	401	51.61	68.0	even	ok
	398	C78H34N3NaO39	829.5328	-0.6	42.4	402	42.18	64.0	even	ok
	399	C63H146N22O28	829.5333	-0.0	42.4	403	43.71	3.0	even	ok
	400	C61H4N53NaO2P2S2	829.5322	-1.4	42.6	404	30.12	88.0	even	ok
	401	C50H24N37NaO23P2S2	829.5325	-1.1	42.6	405	34.93	59.0	even	ok
	402	C44H119N66NaS2	829.5334	0.1	42.6	406	42.34	19.0	even	ok
	403	C64H2N47NaO11S	829.5325	-1.1	42.7	407	35.05	88.0	even	ok
	404	C57H33N17O42S	829.5339	0.7	42.8	408	40.32	51.0	even	ok
	405	C57H155N18NaO32S	829.5331	-0.3	42.9	409	47.79	-10.0	even	ok
	406	C59H177NaO39P2S2	829.5335	0.2	43.0	410	48.67	-27.0	even	ok
	407	C67H10N37NaO17S	829.5331	-0.2	43.0	411	48.32	82.0	even	ok
	408	C64H162N4O42	829.5326	-0.8	43.1	412	37.92	-13.0	even	ok
	409	C68H13N33O21S	829.5337	0.4	43.1	413	45.28	80.0	even	ok
	410	C53H131N46NaO12S	829.5331	-0.3	43.2	414	39.38	12.0	even	ok
	411	C63H37N7O48	829.5329	-0.5	43.2	415	43.62	50.0	even	ok
	412	C62H7N49O6P2S2	829.5327	-0.7	43.3	416	39.56	86.0	even	ok
	413	C63H15N35O21P2	829.5323	-1.3	43.3	417	26.15	76.0	even	ok
	414	C60H9N39O24	829.5336	0.3	43.4	418	39.00	77.0	even	ok
	415	C61H18N29NaO30	829.5331	-0.3	43.5	419	38.24	68.0	even	ok
	416	C45H6N55NaO16S2	829.5337	0.5	43.5	420	43.53	71.0	even	ok

Compound Spectrum SmartFormula Report

Meas. m/z	#	Ion Formula	m/z	err [ppm]	mSigma	# mSigma	Score	rdb	e⁻ Conf	N-Rule
	417	C61H5N43O20	829.5343	1.1	43.5	421	27.86	82.0	even	ok
	418	C64H155N12NaO34	829.5328	-0.7	43.8	422	33.13	-6.0	even	ok
	419	C75H36N9NaO30P2S	829.5325	-1.0	43.9	423	35.19	64.0	even	ok
	420	C66H16N33NaO19P2	829.5331	-0.3	43.9	424	38.64	77.0	even	ok
	421	C70H157N16NaO18P2S2	829.5333	-0.1	44.0	425	49.37	2.0	even	ok
	422	C51H151N28NaO26S2	829.5341	0.9	44.1	426	35.27	-9.0	even	ok
	423	C44H126N58O8S2	829.5333	-0.0	44.1	427	50.05	12.0	even	ok
	424	C76H155N8O28P	829.5339	0.6	44.2	428	39.99	5.0	even	ok
	425	C60H124N52NaPS	829.5323	-1.2	44.2	429	25.92	26.0	even	ok
	426	C48H19N43O21P2S2	829.5323	-1.2	44.3	430	30.81	63.0	even	ok
	427	C70H18N27NaO23S	829.5338	0.6	44.3	431	41.00	76.0	even	ok
	428	C56H30N21NaO38S	829.5334	0.1	44.4	433	49.07	53.0	even	ok
	429	C78H160N2NaO30P	829.5340	0.8	44.5	434	36.93	1.0	even	ok
	430	C70H35N15O28P2S2	829.5341	0.9	44.5	435	35.60	63.0	even	ok
	431	C64H12N43NaO8P2S2	829.5329	-0.6	44.8	436	40.53	82.0	even	ok
	432	C70H149N20NaO19P2	829.5335	0.2	44.9	437	38.88	8.0	even	ok
	433	C59H51NNaO48PS2	829.5323	-1.2	44.9	438	30.24	36.0	even	ok
	434	C55H150N24O30S	829.5330	-0.4	45.0	439	42.35	-6.0	even	ok
	435	C51H10N45NaO22S	829.5327	-0.7	45.0	440	37.56	70.0	even	ok
	436	C65H131N42O6PS	829.5342	1.0	45.0	441	32.89	23.0	even	ok
	437	C71H21N23O27S	829.5343	1.2	45.0	442	30.34	74.0	even	ok
	438	C63H139N30NaO20	829.5334	0.1	45.0	443	39.65	10.0	even	ok
	439	C76H39N5O34P2S	829.5331	-0.3	45.2	444	44.01	62.0	even	ok
	440	C67H136N36NaO8PS	829.5343	1.2	45.3	445	30.14	19.0	even	ok
	441	C65H168NaO41P	829.5343	1.1	45.3	446	31.43	-17.0	even	ok
	442	C75H152N12NaO24P	829.5333	0.0	45.5	448	48.80	7.0	even	ok
	443	C64H11N39O17P2	829.5330	-0.4	45.6	449	34.59	81.0	even	ok
	444	C49H146N34O24S2	829.5340	0.8	45.7	450	36.27	-5.0	even	ok
	445	C71H19N25NaO24P	829.5330	-0.5	45.7	451	34.30	76.0	even	ok
	446	C70H20N27NaO21P2	829.5345	1.3	45.7	452	23.35	76.0	even	ok
	447	C62H34N11NaO44	829.5324	-1.1	45.7	453	30.80	52.0	even	ok
	448	C51H126N52O10S	829.5330	-0.5	45.8	454	34.33	16.0	even	ok
	449	C51H119N60NaO2S	829.5331	-0.3	45.9	455	36.33	23.0	even	ok
	450	C71H160N12O22P2S2	829.5338	0.5	45.9	456	39.51	0.0	even	ok
	451	C77H154N6O31	829.5324	-1.2	46.0	457	30.37	5.0	even	ok
	452	C59H13N35O28	829.5329	-0.5	46.0	458	33.30	72.0	even	ok
	453	C65H15N39O12P2S2	829.5334	0.1	46.1	459	46.69	80.0	even	ok
	454	C69H14N31O22P	829.5328	-0.6	46.1	460	31.69	80.0	even	ok
	455	C68H144N26O17P2	829.5334	0.0	46.2	461	39.62	12.0	even	ok
	456	C54H25N27O36S	829.5333	-0.1	46.3	462	46.27	57.0	even	ok
	457	C62H143N26NaO24	829.5328	-0.7	46.3	463	30.75	5.0	even	ok
	458	C62H150N18O32	829.5326	-0.9	46.4	464	28.64	-2.0	even	ok
	459	C43H123N62NaO4S2	829.5328	-0.7	46.5	465	36.78	14.0	even	ok
	460	C68H15N33O19P2	829.5343	1.2	46.5	466	24.71	80.0	even	ok
	461	C73H26N17NaO29S	829.5345	1.4	46.6	467	26.72	70.0	even	ok
	462	C60H170O46S	829.5337	0.4	46.6	468	41.38	-23.0	even	ok

Compound Spectrum SmartFormula Report

Meas. m/z	#	Ion Formula	m/z	err [ppm]	mSigma	# mSigma	Score	rdb	e¯ Conf	N-Rule
	463	C64H135N34NaO16	829.5341	0.9	46.6	469	27.68	15.0	even	ok
	464	C79H159NaO33	829.5325	-1.0	46.6	470	32.17	1.0	even	ok
	465	C66H130N40O9S	829.5327	-0.8	46.8	471	35.44	23.0	even	ok
	466	C63H132N46P2S2	829.5324	-1.1	46.8	472	30.66	23.0	even	ok
	467	C72H40N9NaO30P2S2	829.5342	1.1	46.9	473	30.86	59.0	even	ok
	468	C48H143N38NaO20S2	829.5334	0.1	47.0	474	44.56	-3.0	even	ok
	469	C58H52N3NaO45P2S2	829.5338	0.6	47.1	475	37.90	36.0	even	ok
	470	C50H26N31NaO32S2	829.5344	1.3	47.1	476	27.47	54.0	even	ok
	471	C69H138N30O15S	829.5334	0.1	47.2	477	45.57	17.0	even	ok
	472	C76H161N6NaO24P2S	829.5322	-1.3	47.2	478	27.15	1.0	even	ok
	473	C54H147N28NaO26S	829.5324	-1.1	47.3	479	30.20	-4.0	even	ok
	474	C59H6N43NaO20	829.5330	-0.3	47.3	480	34.09	79.0	even	ok
	475	C61H134N36O18	829.5333	-0.1	47.4	481	37.79	14.0	even	ok
	476	C68H135N34NaO11S	829.5328	-0.6	47.4	482	37.27	19.0	even	ok
	477	C60H2N47NaO16	829.5337	0.5	47.4	483	32.67	84.0	even	ok
	478	C63H163N6O39P	829.5341	1.0	47.8	484	31.51	-13.0	even	ok
	479	C62H38N9O45P	829.5344	1.3	47.8	485	26.79	50.0	even	ok
	480	C56H47N9O43P2S2	829.5337	0.4	47.9	486	39.49	40.0	even	ok
	481	C67H20N33NaO14P2S2	829.5335	0.2	47.9	487	41.69	76.0	even	ok
	482	C59H167N4NaO42S	829.5331	-0.3	47.9	488	41.40	-21.0	even	ok
	483	C60H29N17O42	829.5323	-1.3	48.0	489	26.61	56.0	even	ok
	484	C55H44N13NaO39P2S2	829.5331	-0.2	48.0	490	41.70	42.0	even	ok
	485	C77H164N2O28P2S	829.5328	-0.7	48.1	491	35.25	-1.0	even	ok
	486	C73H165N6NaO24P2S2	829.5339	0.7	48.1	492	34.62	-4.0	even	ok
	487	C65H137N40NaO2P2S2	829.5326	-0.9	48.1	493	31.77	19.0	even	ok
	488	C71H143N24NaO17S	829.5335	0.2	48.2	494	41.72	13.0	even	ok
	489	C53H22N31NaO32S	829.5327	-0.7	48.2	495	34.22	59.0	even	ok
	490	C49H114N66S	829.5330	-0.5	48.4	496	31.64	27.0	even	ok
	491	C60H22N25NaO34	829.5324	-1.1	48.4	497	23.63	63.0	even	ok
	492	C48H21N37O30S2	829.5343	1.1	48.5	498	28.58	58.0	even	ok
	493	C68H11N35NaO18P	829.5323	-1.3	48.5	499	22.23	82.0	even	ok
	494	C62H130N40O14	829.5340	0.8	48.5	500	28.08	19.0	even	ok
	495	C72H146N20O21S	829.5341	0.9	48.6	501	32.02	11.0	even	ok
	496	C46H138N44O18S2	829.5333	-0.0	48.8	502	43.74	1.0	even	ok
	497	C60H138N32O22	829.5326	-0.9	48.8	503	26.55	9.0	even	ok
	498	C63H156N14NaO31P	829.5343	1.1	49.0	504	23.60	-6.0	even	ok
	499	C66H140N36O6P2S2	829.5331	-0.3	49.1	505	39.96	17.0	even	ok
	500	C53H39N19O37P2S2	829.5330	-0.4	49.1	506	37.92	46.0	even	ok

Cmpd 4, 4.7 min

Compound Spectrum SmartFormula Report

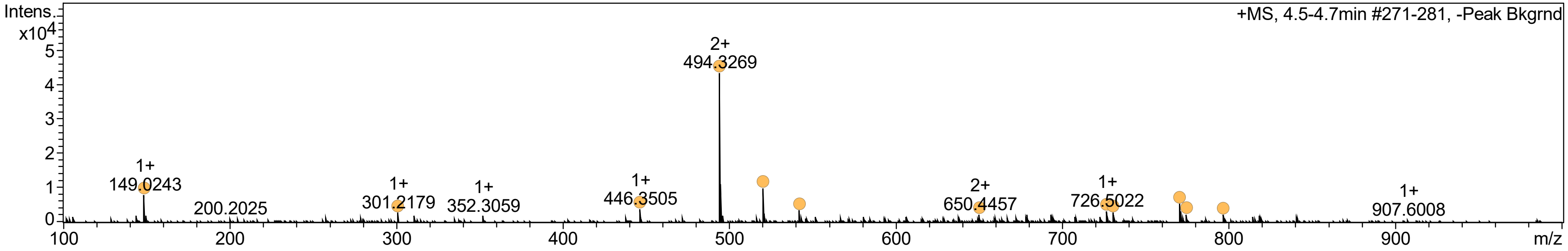

Meas. m/z	#	Ion Formula	m/z	err [ppm]	mSigma	# mSigma	Score	rdb	e⁻ Conf	N-Rule
149.0243	1	C5H7N2NaP	149.0239	-2.7	13.4	1	100.00	4.0	even	ok
301.2179	1	C12H35N2O2P2	301.2168	-3.5	48.1	1	82.77	-2.0	even	ok
	2	C13H29N6S	301.2169	-3.3	56.4	2	88.50	3.0	even	ok
	3	C15H34NaO2S	301.2172	-2.4	57.3	3	100.00	-1.0	even	ok
	4	C15H36NaP2	301.2184	1.8	61.8	4	70.59	-1.0	even	ok
446.3505	1	C26H50NNaOP	446.3522	3.9	27.8	1	40.71	3.0	even	ok
	2	C28H45N3Na	446.3506	0.2	28.6	2	100.00	8.0	even	ok
	3	C25H44N5O2	446.3490	-3.5	28.6	3	45.28	7.0	even	ok
	4	C23H49N3O3P	446.3506	0.2	35.6	4	82.82	2.0	even	ok
	5	C20H51N5NaP2	446.3512	1.6	44.9	5	48.86	-1.0	even	ok
	6	C21H54NO4P2	446.3523	3.9	47.7	6	23.38	-3.0	even	ok
	7	C22H48N5O2S	446.3523	4.1	47.8	7	29.65	2.0	even	ok
	8	C18H44N11S	446.3496	-1.9	49.9	8	51.49	3.0	even	ok
	9	C20H49N5NaO2S	446.3499	-1.3	51.2	9	57.12	-1.0	even	ok
	10	C21H52NO6S	446.3510	1.1	55.0	10	53.02	-3.0	even	ok
	11	C17H50N7O2P2	446.3496	-2.1	58.1	11	28.68	-2.0	even	ok
	12	C19H55NNaO4P2	446.3499	-1.4	58.2	12	33.15	-6.0	even	ok
	13	C17H48N7O4S	446.3483	-4.9	59.1	13	15.62	-2.0	even	ok
	14	C18H54N3NaO3PS	446.3516	2.4	59.5	14	33.50	-6.0	even	ok
	15	C19H53NNaO6S	446.3486	-4.3	60.5	15	18.52	-6.0	even	ok
	16	C25H52NOS2	446.3485	-4.5	61.9	16	20.50	1.0	even	ok
494.3269	1	CH2N3NaO49P2S2	494.3280	2.3	41.5	2	64.41	4.0	even	ok
520.3673	1	C23H54NO11	520.3691	3.6	6.7	1	46.00	-2.0	even	ok
	2	C22H51N5NaO7	520.3681	1.5	9.6	2	85.88	0.0	even	ok
	3	C20H56N7O2P2S	520.3686	2.6	10.6	3	62.03	-2.0	even	ok
	4	C22H61NNaO4P2S	520.3689	3.1	12.5	4	49.76	-6.0	even	ok
	5	C21H55NNaO11	520.3667	-1.0	12.7	5	93.06	-5.0	even	ok
	6	C20H46N11O5	520.3678	1.0	12.7	6	70.01	4.0	even	ok
	7	C21H55N5O5PS	520.3656	-3.2	13.9	7	46.73	-2.0	even	ok
	8	C19H50N7O9	520.3665	-1.6	14.3	8	78.14	-1.0	even	ok
	9	C20H56N3NaO8P	520.3697	4.7	16.5	9	23.94	-5.0	even	ok
	10	C25H57NNaO4P2	520.3655	-3.4	16.7	10	30.87	-1.0	even	ok
	11	C19H43N15NaO	520.3667	-1.0	17.3	11	63.37	6.0	even	ok

Compound Spectrum SmartFormula Report

Meas. m/z	#	Ion Formula	m/z	err [ppm]	mSigma	# mSigma	Score	rdb	e⁻ Conf	N-Rule
	12	C22H51N9OPS	520.3669	-0.6	18.0	12	92.36	3.0	even	ok
	13	C23H52N7O2P2	520.3652	-3.9	18.1	13	24.40	3.0	even	ok
	14	C18H47N11NaO5	520.3654	-3.6	18.9	14	27.09	1.0	even	ok
	15	C23H47N9NaO3	520.3694	4.1	19.6	15	21.84	5.0	even	ok
	16	C24H56N3NaO3PS	520.3672	-0.1	19.9	16	100.00	-1.0	even	ok
	17	C21H42N15O	520.3691	3.6	20.7	17	26.42	9.0	even	ok
	18	C18H51N9O6P	520.3694	4.2	20.9	18	20.61	-1.0	even	ok
	19	C16H42N17O3	520.3651	-4.2	21.3	19	20.77	5.0	even	ok
	20	C27H56NO4P2	520.3679	1.2	22.8	20	71.36	2.0	even	ok
	21	C18H54N3O13	520.3651	-4.1	23.5	21	26.55	-6.0	even	ok
	22	C17H48N13NaO2P	520.3684	2.1	23.8	22	40.60	1.0	even	ok
	23	C15H43N19P	520.3681	1.6	26.2	23	45.01	5.0	even	ok
	24	C26H53N5NaP2	520.3668	-0.8	28.7	24	52.09	4.0	even	ok
	25	C26H55N3O3PS	520.3696	4.5	29.9	25	19.26	2.0	even	ok
	26	C24H46N11S	520.3653	-3.8	30.9	26	25.56	8.0	even	ok
	27	C19H51N11NaS2	520.3663	-1.9	32.1	27	47.01	0.0	even	ok
	28	C26H51N5NaO2S	520.3656	-3.3	32.5	28	30.11	4.0	even	ok
	29	C27H54NO6S	520.3666	-1.2	34.1	29	55.00	2.0	even	ok
	30	C20H54N7O4S2	520.3673	0.1	34.6	30	88.09	-2.0	even	ok
	31	C29H51N3O3P	520.3663	-1.9	36.0	31	42.68	7.0	even	ok
	32	C22H59NNaO6S2	520.3676	0.6	36.4	32	74.54	-6.0	even	ok
	33	C23H60N3OP2S2	520.3648	-4.8	37.0	33	14.18	-3.0	even	ok
	34	C21H50N11S2	520.3687	2.7	38.0	34	32.05	3.0	even	ok
	35	C23H55N5NaO2S2	520.3689	3.2	38.2	35	33.04	-1.0	even	ok
	36	C28H52N5P2	520.3692	3.8	39.4	36	15.34	7.0	even	ok
	37	C28H50N5O2S	520.3680	1.4	43.5	37	41.00	7.0	even	ok
	38	C32H52NNaOP	520.3679	1.2	48.1	38	37.75	8.0	even	ok
	39	C31H51N3NaS	520.3696	4.5	55.3	39	9.70	8.0	even	ok
	40	C34H47N3Na	520.3662	-2.0	62.1	40	18.97	13.0	even	ok
	41	C36H46N3	520.3686	2.6	73.2	41	10.48	16.0	even	ok
542.3285	1	C31H45N5NaS	542.3288	0.4	11.9	1	100.00	12.0	even	ok
	2	C32H48NO4S	542.3299	2.4	13.5	2	55.01	10.0	even	ok
	3	C33H44N5S	542.3312	4.9	16.6	3	18.73	15.0	even	ok
	4	C30H49NNaO4S	542.3275	-2.0	17.4	4	57.95	7.0	even	ok
	5	C28H44N7O2S	542.3272	-2.5	17.6	5	48.59	11.0	even	ok
	6	C29H50N3NaOPS	542.3304	3.5	19.0	6	32.94	7.0	even	ok
	7	C35H44NO4	542.3265	-3.8	19.7	7	28.61	15.0	even	ok
	8	C34H45N3OP	542.3295	1.7	21.7	8	58.11	15.0	even	ok
	9	C32H46N3NaOP	542.3271	-2.7	24.1	9	39.57	12.0	even	ok
	10	C32H50NO2P2	542.3311	4.8	24.8	10	16.60	10.0	even	ok
	11	C36H40N5	542.3278	-1.3	27.3	11	57.28	20.0	even	ok
	12	C27H48N3O6S	542.3258	-5.0	27.5	12	13.91	6.0	even	ok
	13	C26H49N5O3PS	542.3288	0.5	29.8	13	66.89	6.0	even	ok
	14	C25H48N7O2S2	542.3305	3.7	32.0	14	28.33	6.0	even	ok
	15	C30H51NNaO2P2	542.3287	0.3	32.3	15	65.71	7.0	even	ok
	16	C27H53NNaO4S2	542.3308	4.2	32.7	16	22.29	2.0	even	ok

Compound Spectrum SmartFormula Report

Meas. m/z	#	Ion Formula	m/z	err [ppm]	mSigma	# mSigma	Score	rdb	e¯ Conf	N-Rule
	17	C27H45N5NaO5	542.3313	5.1	37.3	17	10.56	8.0	even	ok
	18	C23H49N7NaO2S2	542.3281	-0.8	38.0	18	63.77	3.0	even	ok
	19	C25H40N11O3	542.3310	4.5	39.2	19	12.86	12.0	even	ok
	20	C28H46N7P2	542.3284	-0.2	39.5	20	42.11	11.0	even	ok
	21	C24H52N3O6S2	542.3292	1.2	40.0	21	53.53	1.0	even	ok
	22	C24H50N5NaO3PS	542.3264	-3.9	40.3	22	16.49	3.0	even	ok
	23	C21H44N13S2	542.3279	-1.3	40.4	23	41.78	7.0	even	ok
	24	C25H53NO7PS	542.3275	-2.0	40.6	24	33.87	1.0	even	ok
	25	C22H45N11OPS	542.3261	-4.4	41.2	25	12.78	7.0	even	ok
	26	C27H50N3O4P2	542.3271	-2.7	41.3	26	26.31	6.0	even	ok
	27	C23H51N7NaP2S	542.3294	1.6	43.2	27	35.16	3.0	even	ok
	28	C24H54N3O4P2S	542.3305	3.6	43.3	28	17.62	1.0	even	ok
	29	C26H49NNaO9	542.3300	2.6	45.8	29	23.60	3.0	even	ok
	30	C20H48N9O4S2	542.3265	-3.7	47.2	30	18.31	2.0	even	ok
	31	C22H53N3NaO6S2	542.3268	-3.2	47.2	31	22.47	-2.0	even	ok
	32	C24H44N7O7	542.3297	2.1	47.5	32	26.74	7.0	even	ok
	33	C21H54N5NaO3PS2	542.3298	2.3	48.3	33	30.44	-2.0	even	ok
	34	C22H57NO7PS2	542.3309	4.3	49.8	34	13.46	-4.0	even	ok
	35	C26H47N7NaP2	542.3260	-4.6	50.0	35	6.82	8.0	even	ok
	36	C19H49N11OPS2	542.3295	1.8	52.5	36	25.23	2.0	even	ok
	37	C22H55N3NaO4P2S	542.3281	-0.9	54.1	37	30.97	-2.0	even	ok
	38	C20H50N9O2P2S	542.3278	-1.4	55.0	38	26.09	2.0	even	ok
	39	C23H41N11NaO3	542.3286	0.1	56.6	39	25.75	9.0	even	ok
	40	C23H48N3O11	542.3283	-0.4	56.8	40	31.92	2.0	even	ok
	41	C22H45N7NaO7	542.3273	-2.4	57.9	41	17.57	4.0	even	ok
	42	C20H58NNaO7PS2	542.3285	-0.2	58.1	42	40.23	-7.0	even	ok
	43	C18H53N7O5PS2	542.3282	-0.7	58.2	43	35.44	-3.0	even	ok
	44	C21H36N17O	542.3283	-0.4	58.7	44	22.42	13.0	even	ok
	45	C19H49N7NaO7S	542.3306	3.9	62.3	45	8.58	-1.0	even	ok
	46	C17H50N11NaOPS2	542.3271	-2.7	62.4	46	13.62	-1.0	even	ok
	47	C16H41N17NaOS	542.3293	1.4	63.1	47	20.01	5.0	even	ok
	48	C17H44N13O5S	542.3304	3.3	63.2	48	10.27	3.0	even	ok
	49	C19H54N5O6P2S	542.3265	-3.9	66.0	49	7.53	-3.0	even	ok
	50	C21H49N3NaO11	542.3259	-4.8	67.6	50	4.60	-1.0	even	ok
	51	C20H40N13O5	542.3270	-2.9	68.7	51	7.56	8.0	even	ok
	52	C21H53NO12P	542.3300	2.7	70.3	52	10.23	-3.0	even	ok
	53	C19H37N17NaO	542.3259	-4.8	70.4	53	3.08	10.0	even	ok
	54	C20H50N5NaO8P	542.3289	0.7	71.2	54	17.93	-1.0	even	ok
	55	C21H46N9NaO4P	542.3303	3.2	71.3	55	6.16	4.0	even	ok
	56	C19H41N15O2P	542.3300	2.6	73.3	56	6.92	8.0	even	ok
	57	C18H53N3NaO11S	542.3293	1.4	73.5	57	13.64	-6.0	even	ok
	58	C15H45N13NaO5S	542.3280	-1.1	74.0	58	14.50	0.0	even	ok
	59	C16H48N9O9S	542.3290	0.9	74.3	59	15.22	-2.0	even	ok
	60	C19H54NNaO12P	542.3276	-1.8	81.1	60	9.01	-6.0	even	ok
	61	C17H49N7O10P	542.3273	-2.3	82.4	61	7.23	-2.0	even	ok
	62	C18H45N11O6P	542.3286	0.2	83.9	62	9.19	3.0	even	ok

Compound Spectrum SmartFormula Report

Meas. m/z	#	Ion Formula	m/z	err [ppm]	mSigma	# mSigma	Score	rdb	e⁻ Conf	N-Rule
	63	C17H42N15NaO2P	542.3276	-1.8	85.3	63	5.65	5.0	even	ok
	64	C15H37N21P	542.3273	-2.3	87.1	64	4.42	9.0	even	ok
	65	C16H46N11NaO6P	542.3262	-4.3	96.1	65	1.38	0.0	even	ok
	66	C14H41N17O4P	542.3260	-4.8	97.8	66	1.01	4.0	even	ok
650.4457	1	C15H2N35NaO34P2	650.4433	-3.8	83.0	1	38.63	34.0	even	ok
	2	C13H6N33NaO37P2	650.4482	3.8	87.9	2	30.71	29.0	even	ok
	3	C10H5N35O39P2	650.4474	2.6	94.2	5	43.70	28.0	even	ok
	4	C9H2N39NaO35P2	650.4469	1.8	96.0	6	56.82	30.0	even	ok
726.5022	1	C26H74N13O2P2S2	726.5000	-3.0	11.7	1	33.41	-2.0	even	ok
	2	C28H79N7NaO4P2S2	726.5002	-2.6	11.9	2	41.33	-6.0	even	ok
	3	C24H64N21OS2	726.5039	2.4	12.0	3	47.79	4.0	even	ok
	4	C26H69N15NaO3S2	726.5041	2.7	12.4	4	38.71	0.0	even	ok
	5	C29H82N3O8P2S2	726.5013	-1.2	13.0	5	80.34	-8.0	even	ok
	6	C27H72N11O7S2	726.5052	4.2	13.3	6	15.02	-2.0	even	ok
	7	C25H73N11NaO7S2	726.5028	0.9	13.9	7	87.45	-5.0	even	ok
	8	C22H65N21NaOS2	726.5015	-1.0	13.9	8	85.45	1.0	even	ok
	9	C23H68N17O5S2	726.5025	0.5	13.9	9	100.00	-1.0	even	ok
	10	C29H77N5NaO9S2	726.5055	4.6	14.4	10	11.07	-6.0	even	ok
	11	C26H76N7O11S2	726.5039	2.4	15.4	11	44.46	-7.0	even	ok
	12	C31H77NNaO13S	726.5008	-1.9	15.4	12	56.13	-6.0	even	ok
	13	C28H81NNaO13S2	726.5042	2.8	16.1	13	35.70	-11.0	even	ok
	14	C29H75N11NaP2S2	726.5016	-0.8	16.4	14	86.41	-1.0	even	ok
	15	C30H78N7O4P2S2	726.5026	0.7	16.6	15	89.60	-3.0	even	ok
	16	C32H83NNaO6P2S2	726.5029	1.1	17.8	16	76.01	-7.0	even	ok
	17	C28H76N3O15S	726.4992	-4.1	18.0	17	14.59	-7.0	even	ok
	18	C29H72N7O11S	726.5005	-2.3	18.5	18	35.02	-2.0	even	ok
	19	C33H76NO13S	726.5032	1.4	18.5	19	64.73	-3.0	even	ok
	20	C30H74N9NaO3PS2	726.4986	-4.9	18.6	20	7.94	-1.0	even	ok
	21	C31H77N5O7PS2	726.4997	-3.4	19.5	21	22.13	-3.0	even	ok
	22	C30H78N3NaO10PS	726.5038	2.2	19.6	22	34.97	-6.0	even	ok
	23	C28H73N9O8PS	726.5035	1.8	21.4	23	40.44	-2.0	even	ok
	24	C28H69N11NaO7S	726.4994	-3.7	21.8	24	13.88	0.0	even	ok
	25	C32H73N5NaO9S	726.5021	-0.0	22.7	25	77.03	-1.0	even	ok
	26	C26H64N17O5S	726.4992	-4.1	23.6	26	10.25	4.0	even	ok
	27	C30H68N11O7S	726.5018	-0.4	23.7	27	66.98	3.0	even	ok
	28	C31H74N7NaO6PS	726.5051	4.1	23.7	28	10.65	-1.0	even	ok
	29	C27H77N5O12PS	726.5022	0.0	23.7	29	76.34	-7.0	even	ok
	30	C31H74N11P2S2	726.5040	2.5	24.7	30	33.67	2.0	even	ok
	31	C27H70N13NaO4PS	726.5024	0.4	24.9	31	66.42	0.0	even	ok
	32	C29H69N13O4PS	726.5048	3.7	25.0	32	13.41	3.0	even	ok
	33	C33H79N5NaO2P2S2	726.5043	2.9	25.7	33	26.64	-2.0	even	ok
	34	C28H83NNaO11P2S	726.5054	4.5	25.8	34	7.42	-11.0	even	ok
	35	C26H74N9NaO8PS	726.5011	-1.5	26.4	35	42.72	-5.0	even	ok
	36	C34H82NO6P2S2	726.5053	4.4	26.4	36	10.09	-4.0	even	ok
	37	C29H65N15NaO3S	726.5008	-1.9	26.7	37	34.91	5.0	even	ok
	38	C25H65N19O2PS	726.5021	-0.0	26.9	38	70.85	4.0	even	ok

Compound Spectrum SmartFormula Report

Meas. m/z	#	Ion Formula	m/z	err [ppm]	mSigma	# mSigma	Score	rdb	e⁻ Conf	N-Rule
	39	C34H72N5O9S	726.5045	3.3	27.0	39	20.90	2.0	even	ok
	40	C32H73N9O3PS2	726.5010	-1.6	27.6	40	49.04	2.0	even	ok
	41	C26H78N7O9P2S	726.5051	4.1	27.7	41	9.37	-7.0	even	ok
	42	C27H60N21OS	726.5005	-2.3	28.1	42	28.07	9.0	even	ok
	43	C26H71N15NaOP2S	726.5054	4.5	28.4	43	7.06	0.0	even	ok
	44	C24H69N15O6PS	726.5008	-1.9	28.4	44	34.32	-1.0	even	ok
	45	C28H66N17NaPS	726.5038	2.2	28.5	45	28.83	5.0	even	ok
	46	C34H78N3NaO5PS2	726.5013	-1.2	28.8	46	55.94	-2.0	even	ok
	47	C35H79NNaO6P2S	726.4996	-3.6	29.4	47	12.94	-2.0	even	ok
	48	C33H74N7O4P2S	726.4993	-4.0	30.0	48	9.89	2.0	even	ok
	49	C25H75N11NaO5P2S	726.5041	2.6	30.5	49	21.89	-5.0	even	ok
	50	C23H66N19NaO2PS	726.4997	-3.3	31.5	50	14.46	1.0	even	ok
	51	C33H69N9NaO5S	726.5035	1.8	32.0	51	32.35	4.0	even	ok
	52	C31H64N15O3S	726.5032	1.4	32.4	52	37.83	8.0	even	ok
	53	C29H76NO18	726.5057	4.9	32.7	53	4.76	-7.0	even	ok
	54	C33H78NO11P2	726.5045	3.2	33.3	54	15.10	-3.0	even	ok
	55	C21H61N25PS	726.4995	-3.7	33.7	55	10.72	5.0	even	ok
	56	C30H73N5O12P	726.4988	-4.6	33.8	56	5.53	-2.0	even	ok
	57	C33H74N3NaO10P	726.5004	-2.4	34.4	57	22.54	-1.0	even	ok
	58	C31H79NNaO11P2	726.5021	-0.1	35.5	58	55.27	-6.0	even	ok
	59	C36H72NO13	726.4998	-3.2	35.5	59	13.96	2.0	even	ok
	60	C28H73N5NaO14	726.5046	3.4	36.2	60	12.24	-5.0	even	ok
	61	C31H69N9O8P	726.5001	-2.8	36.3	61	17.41	3.0	even	ok
	62	C35H73N3O10P	726.5028	0.9	36.8	62	41.33	2.0	even	ok
	63	C32H75N5NaO7P2	726.5034	1.7	37.4	63	29.26	-1.0	even	ok
	64	C29H74N7O9P2	726.5018	-0.5	37.8	64	46.01	-2.0	even	ok
	65	C27H64N15O8	726.5057	4.9	37.8	65	4.21	4.0	even	ok
	66	C37H78NO6P2S	726.5020	-0.3	37.9	66	49.80	1.0	even	ok
	67	C35H74N7NaOPS2	726.5026	0.6	38.1	67	55.03	3.0	even	ok
	68	C26H68N11O12	726.5043	3.0	38.6	68	14.50	-1.0	even	ok
	69	C34H74N5O7P2	726.5058	5.0	38.6	69	3.62	2.0	even	ok
	70	C36H77N3O5PS2	726.5037	2.1	38.7	70	29.47	1.0	even	ok
	71	C26H61N19NaO4	726.5046	3.4	38.7	71	11.56	6.0	even	ok
	72	C35H69N5NaO9	726.4987	-4.7	38.9	72	4.66	4.0	even	ok
	73	C28H78N3O13P2	726.5004	-2.4	39.4	73	20.26	-7.0	even	ok
	74	C30H70N11O5P2	726.5031	1.3	39.5	74	32.62	3.0	even	ok
	75	C36H75N5NaO2P2S	726.5009	-1.7	39.7	75	27.13	3.0	even	ok
	76	C34H70N11P2S	726.5006	-2.1	39.9	76	22.49	7.0	even	ok
	77	C34H68N11O2S2	726.4993	-3.9	40.1	77	10.12	7.0	even	ok
	78	C33H64N11O7	726.4985	-5.1	40.2	78	3.32	8.0	even	ok
	79	C27H77NNaO18	726.5033	1.6	40.3	79	28.91	-10.0	even	ok
	80	C30H66N13NaO4P	726.4991	-4.3	40.4	80	6.11	5.0	even	ok
	81	C34H70N7NaO6P	726.5017	-0.6	40.4	81	42.09	4.0	even	ok
	82	C24H56N25O2	726.5043	3.0	40.8	82	13.78	10.0	even	ok
	83	C36H73N5NaO4S2	726.4996	-3.5	41.3	83	12.58	3.0	even	ok
	84	C23H60N21O6	726.5030	1.2	41.6	84	32.86	5.0	even	ok

Compound Spectrum SmartFormula Report

Meas. m/z	#	Ion Formula	m/z	err [ppm]	mSigma	# mSigma	Score	rdb	e⁻ Conf	N-Rule
	85	C28H71N11NaO5P2	726.5007	-2.0	41.8	85	22.80	0.0	even	ok
	86	C32H65N13O4P	726.5015	-1.0	42.0	86	35.14	8.0	even	ok
	87	C37H76NO8S2	726.5007	-2.0	42.2	87	27.77	1.0	even	ok
	88	C25H65N15NaO8	726.5033	1.5	42.3	88	27.55	1.0	even	ok
	89	C34H65N13NaOS	726.5048	3.6	42.4	89	8.89	9.0	even	ok
	90	C28H61N19O2P	726.4988	-4.7	42.5	90	4.34	9.0	even	ok
	91	C33H71N9NaO3P2	726.5047	3.5	42.5	91	9.40	4.0	even	ok
	92	C25H72N7O16	726.5030	1.2	42.5	92	31.87	-6.0	even	ok
	93	C27H75N7NaO9P2	726.4994	-3.8	42.9	93	7.70	-5.0	even	ok
	94	C29H67N15NaOP2	726.5020	-0.1	43.6	94	44.07	5.0	even	ok
	95	C26H66N17O3P2	726.5004	-2.4	44.1	95	17.65	4.0	even	ok
	96	C31H66N15OP2	726.5045	3.2	44.2	96	11.36	8.0	even	ok
	97	C38H74NNaO8P	726.5044	3.1	44.3	97	11.57	3.0	even	ok
	98	C37H68N5O9	726.5012	-1.4	44.8	98	27.49	7.0	even	ok
	99	C22H57N25NaO2	726.5019	-0.3	45.0	99	40.29	7.0	even	ok
	100	C36H69N7O6P	726.5041	2.7	45.3	100	14.04	7.0	even	ok
	101	C25H70N13O7P2	726.4991	-4.2	45.3	101	5.52	-1.0	even	ok
	102	C22H64N17O10	726.5017	-0.7	45.3	102	35.26	0.0	even	ok
	103	C24H69N11NaO12	726.5019	-0.3	45.6	103	39.79	-4.0	even	ok
	104	C39H73N3O5PS	726.5003	-2.5	45.6	104	19.34	6.0	even	ok
	105	C31H62N17NaP	726.5004	-2.4	45.8	105	16.36	10.0	even	ok
	106	C36H65N9NaO5	726.5001	-2.8	47.8	106	12.30	9.0	even	ok
	107	C21H61N21NaO6	726.5006	-2.2	48.2	107	17.50	2.0	even	ok
	108	C37H73N7OPS2	726.5050	3.9	48.3	108	7.67	6.0	even	ok
	109	C35H66N11NaO2P	726.5031	1.3	48.5	109	25.74	9.0	even	ok
	110	C34H60N15O3	726.4998	-3.2	48.7	110	9.54	13.0	even	ok
	111	C38H74N5O2P2S	726.5033	1.6	49.0	111	22.35	6.0	even	ok
	112	C39H78NNaO3PS2	726.5053	4.3	49.5	112	5.63	2.0	even	ok
	113	C33H61N17P	726.5028	0.9	49.7	113	28.82	13.0	even	ok
	114	C19H56N27O4	726.5003	-2.5	50.3	114	13.47	6.0	even	ok
	115	C37H69N9NaS2	726.5010	-1.6	51.1	115	25.34	8.0	even	ok
	116	C38H72N5O4S2	726.5020	-0.2	51.6	116	42.99	6.0	even	ok
	117	C38H70N7NaOPS	726.4992	-4.0	52.1	117	5.18	8.0	even	ok
	118	C40H74NO6P2	726.4986	-4.9	52.3	118	2.65	6.0	even	ok
	119	C39H70N5NaO4P	726.5058	5.0	54.2	119	2.38	8.0	even	ok
	120	C20H52N31	726.5016	-0.7	54.4	120	19.98	11.0	even	ok
	121	C40H69N3NaO7	726.5028	0.9	54.6	121	25.07	8.0	even	ok
	122	C37H65N11O2P	726.5055	4.6	54.9	122	3.15	12.0	even	ok
	123	C38H64N9O5	726.5025	0.5	55.0	123	28.26	12.0	even	ok
	124	C42H74NNaO3PS	726.5019	-0.3	56.4	124	35.45	7.0	even	ok
	125	C37H61N13NaO	726.5014	-1.0	57.6	125	21.45	14.0	even	ok
	126	C41H68N5O4S	726.4987	-4.8	58.0	126	2.96	11.0	even	ok
	127	C41H78NOP2S2	726.4995	-3.7	58.9	127	6.37	5.0	even	ok
	128	C41H75N3NaP2S	726.5049	3.8	60.7	128	4.53	7.0	even	ok
	129	C39H68N9S2	726.5034	1.7	61.6	129	17.92	11.0	even	ok
	130	C18H53N31Na	726.4992	-4.0	61.6	130	2.86	8.0	even	ok

Compound Spectrum SmartFormula Report

Meas. m/z	#	Ion Formula	m/z	err [ppm]	mSigma	# mSigma	Score	rdb	e⁻ Conf	N-Rule
	131	C40H69N7OPS	726.5016	-0.7	62.4	131	20.39	11.0	even	ok
	132	C41H73N3NaO2S2	726.5036	2.0	62.8	132	14.36	7.0	even	ok
	133	C41H70N5O2P2	726.4999	-3.1	62.9	133	6.65	11.0	even	ok
	134	C42H68N3O7	726.5052	4.2	63.4	134	3.21	11.0	even	ok
	135	C41H65N7NaO3	726.5041	2.7	65.4	135	7.53	13.0	even	ok
	136	C39H60N13O	726.5038	2.3	65.7	136	9.15	17.0	even	ok
	137	C42H64N9S	726.5000	-3.0	68.4	137	7.18	16.0	even	ok
	138	C44H73NO3PS	726.5043	3.0	68.6	138	7.09	10.0	even	ok
	139	C44H69N3NaO2S	726.5003	-2.6	69.1	139	8.69	12.0	even	ok
	140	C44H71N3NaP2	726.5015	-0.8	73.6	140	12.87	12.0	even	ok
	141	C45H70NNaO3P	726.4986	-5.0	74.9	141	1.16	12.0	even	ok
	142	C46H68N3O2S	726.5027	0.7	81.8	142	12.15	15.0	even	ok
	143	C46H70N3P2	726.5039	2.5	83.2	143	4.27	15.0	even	ok
	144	C47H69NO3P	726.5010	-1.6	84.9	144	5.92	15.0	even	ok
	145	C49H69NNaS	726.5043	2.9	93.3	145	2.66	16.0	even	ok
	146	C49H64N3O2	726.4993	-3.9	98.6	146	0.90	20.0	even	ok
730.5333	1	C39H72N9S2	730.5347	1.9	49.4	1	56.01	9.0	even	ok
	2	C37H73N9NaS2	730.5323	-1.4	49.6	2	68.25	6.0	even	ok
	3	C41H82NOP2S2	730.5308	-3.5	50.0	3	22.84	3.0	even	ok
	4	C37H77N7OPS2	730.5363	4.1	50.9	4	14.13	4.0	even	ok
	5	C41H77N3NaO2S2	730.5349	2.3	51.3	5	43.80	5.0	even	ok
	6	C39H82NNaO3PS2	730.5366	4.5	52.4	6	10.19	0.0	even	ok
	7	C38H76N5O4S2	730.5333	0.0	53.1	7	100.00	4.0	even	ok
	8	C35H78N7NaOPS2	730.5339	0.8	54.1	8	74.25	1.0	even	ok
	9	C34H72N11O2S2	730.5306	-3.6	54.1	9	18.10	5.0	even	ok
	10	C36H77N5NaO4S2	730.5309	-3.2	55.4	10	22.18	1.0	even	ok
	11	C36H81N3O5PS2	730.5350	2.3	57.1	11	35.46	-1.0	even	ok
	12	C31H68N15O3S	730.5345	1.6	58.2	12	37.93	6.0	even	ok
	13	C37H80NO8S2	730.5320	-1.8	58.6	13	43.62	-1.0	even	ok
	14	C31H78N11P2S2	730.5353	2.7	60.8	14	25.08	0.0	even	ok
	15	C32H77N9O3PS2	730.5323	-1.4	61.0	15	48.20	0.0	even	ok
	16	C39H64N13O	730.5351	2.5	61.1	16	22.31	15.0	even	ok
	17	C37H65N13NaO	730.5327	-0.8	61.1	17	48.40	12.0	even	ok
	18	C33H83N5NaO2P2S2	730.5356	3.1	61.5	18	19.68	-4.0	even	ok
	19	C41H74N5O2P2	730.5312	-2.8	61.8	19	18.38	9.0	even	ok
	20	C34H82N3NaO5PS2	730.5326	-1.0	61.8	20	54.63	-4.0	even	ok
	21	C44H75N3NaP2	730.5328	-0.6	61.9	21	49.86	10.0	even	ok
	22	C37H69N11O2P	730.5368	4.8	62.5	22	4.80	10.0	even	ok
	23	C35H81NNaO8S2	730.5296	-5.1	62.5	23	4.72	-4.0	even	ok
	24	C41H69N7NaO3	730.5354	2.9	63.2	24	16.79	11.0	even	ok
	25	C43H69N7OP	730.5296	-5.1	63.6	25	3.60	14.0	even	ok
	26	C34H86NO6P2S2	730.5366	4.6	64.0	26	6.67	-6.0	even	ok
	27	C33H65N17P	730.5341	1.1	64.2	27	38.35	11.0	even	ok
	28	C38H68N9O5	730.5338	0.7	64.9	28	43.71	10.0	even	ok
	29	C35H70N11NaO2P	730.5344	1.5	65.5	29	31.32	7.0	even	ok
	30	C34H64N15O3	730.5311	-3.0	65.7	30	14.58	11.0	even	ok

Compound Spectrum SmartFormula Report

Meas. m/z	#	Ion Formula	m/z	err [ppm]	mSigma	# mSigma	Score	rdb	e⁻ Conf	N-Rule
	31	C45H74NNaO3P	730.5299	-4.7	65.9	31	4.51	10.0	even	ok
	32	C40H78NO6P2	730.5299	-4.7	66.2	32	4.63	4.0	even	ok
	33	C46H74N3P2	730.5352	2.7	66.4	33	16.97	13.0	even	ok
	34	C40H73N3NaO7	730.5341	1.1	66.8	34	35.54	6.0	even	ok
	35	C28H73N15OPS2	730.5296	-5.0	67.0	35	4.14	1.0	even	ok
	36	C36H69N9NaO5	730.5314	-2.6	67.2	36	17.17	7.0	even	ok
	37	C42H72N3O7	730.5365	4.4	67.2	37	5.59	9.0	even	ok
	38	C30H78N9NaO3PS2	730.5299	-4.7	67.4	38	5.53	-3.0	even	ok
	39	C29H79N11NaP2S2	730.5329	-0.6	67.8	39	51.39	-3.0	even	ok
	40	C29H69N15NaO3S	730.5321	-1.7	68.0	40	26.47	3.0	even	ok
	41	C27H64N21OS	730.5318	-2.1	68.3	41	21.90	7.0	even	ok
	42	C36H73N7O6P	730.5354	3.0	69.0	42	13.22	5.0	even	ok
	43	C31H66N17NaP	730.5317	-2.2	69.6	43	19.51	8.0	even	ok
	44	C31H81N5O7PS2	730.5310	-3.2	69.7	44	13.90	-5.0	even	ok
	45	C28H70N17NaPS	730.5351	2.4	69.9	45	17.07	3.0	even	ok
	46	C30H82N7O4P2S2	730.5339	0.9	69.9	46	42.24	-5.0	even	ok
	47	C32H87NNaO6P2S2	730.5342	1.3	70.4	47	35.78	-9.0	even	ok
	48	C38H78NNaO8P	730.5357	3.3	70.5	48	9.97	1.0	even	ok
	49	C37H72N5O9	730.5325	-1.1	70.8	49	29.78	5.0	even	ok
	50	C47H73NO3P	730.5323	-1.4	70.9	50	26.59	13.0	even	ok
	51	C29H73N13O4PS	730.5361	3.9	71.2	51	6.75	1.0	even	ok
	52	C31H70N15OP2	730.5358	3.4	72.1	52	9.15	6.0	even	ok
	53	C32H69N13O4P	730.5328	-0.7	72.7	53	32.40	6.0	even	ok
	54	C33H75N9NaO3P2	730.5360	3.8	73.0	54	6.91	2.0	even	ok
	55	C33H68N11O7	730.5298	-4.8	73.6	55	3.10	6.0	even	ok
	56	C34H74N7NaO6P	730.5330	-0.3	73.8	56	35.45	2.0	even	ok
	57	C35H73N5NaO9	730.5300	-4.4	74.8	57	3.98	2.0	even	ok
	58	C24H68N21OS2	730.5352	2.6	74.8	58	16.51	2.0	even	ok
	59	C26H73N15NaO3S2	730.5354	2.9	75.0	59	13.23	-2.0	even	ok
	60	C27H76N11O7S2	730.5365	4.4	77.0	60	4.66	-4.0	even	ok
	61	C35H77N3O10P	730.5341	1.1	77.0	61	23.68	0.0	even	ok
	62	C42H78NNaO3PS	730.5332	-0.1	77.0	62	42.19	5.0	even	ok
	63	C26H78N13O2P2S2	730.5313	-2.8	77.3	63	13.28	-4.0	even	ok
	64	C29H81N5NaO9S2	730.5368	4.8	77.3	64	3.43	-8.0	even	ok
	65	C39H77N3O5PS	730.5316	-2.3	77.4	65	17.07	4.0	even	ok
	66	C28H83N7NaO4P2S2	730.5315	-2.4	77.5	66	16.23	-8.0	even	ok
	67	C42H68N9S	730.5313	-2.7	78.1	67	13.17	14.0	even	ok
	68	C36H76NO13	730.5311	-3.0	78.2	68	9.18	0.0	even	ok
	69	C41H72N5O4S	730.5300	-4.6	78.4	69	3.90	9.0	even	ok
	70	C28H65N19O2P	730.5301	-4.4	78.6	70	3.51	7.0	even	ok
	71	C49H68N3O2	730.5306	-3.7	79.0	71	5.75	18.0	even	ok
	72	C30H85NO11PS2	730.5296	-5.0	79.0	72	2.66	-10.0	even	ok
	73	C30H70N13NaO4P	730.5304	-4.0	79.3	73	4.50	3.0	even	ok
	74	C29H71N15NaOP2	730.5333	0.1	79.3	74	30.93	3.0	even	ok
	75	C29H86N3O8P2S2	730.5326	-0.9	79.4	75	28.84	-10.0	even	ok
	76	C28H73N11NaO7S	730.5307	-3.5	79.5	76	6.32	-2.0	even	ok

Compound Spectrum SmartFormula Report

Meas. m/z	#	Ion Formula	m/z	err [ppm]	mSigma	# mSigma	Score	rdb	e⁻ Conf	N-Rule
	77	C26H68N17O5S	730.5305	-3.9	79.9	77	4.83	2.0	even	ok
	78	C44H77NO3PS	730.5356	3.2	80.1	78	9.30	8.0	even	ok
	79	C44H73N3NaO2S	730.5316	-2.4	80.3	79	14.81	10.0	even	ok
	80	C27H74N13NaO4PS	730.5337	0.6	81.5	80	23.94	-2.0	even	ok
	81	C25H69N19O2PS	730.5334	0.2	82.0	81	26.61	2.0	even	ok
	82	C30H74N11O5P2	730.5344	1.5	82.0	82	16.29	1.0	even	ok
	83	C31H73N9O8P	730.5314	-2.6	82.1	83	9.88	1.0	even	ok
	84	C41H79N3NaP2S	730.5362	4.0	82.3	84	4.02	5.0	even	ok
	85	C38H74N7NaOPS	730.5305	-3.8	82.4	85	4.70	6.0	even	ok
	86	C28H77N9O8PS	730.5348	2.1	82.7	86	12.46	-4.0	even	ok
	87	C32H79N5NaO7P2	730.5347	1.9	82.7	87	13.31	-3.0	even	ok
	88	C34H74N11P2S	730.5319	-1.9	82.7	88	13.49	5.0	even	ok
	89	C33H78N3NaO10P	730.5317	-2.2	83.0	89	11.64	-3.0	even	ok
	90	C34H69N13NaOS	730.5361	3.8	83.1	90	4.37	7.0	even	ok
	91	C34H76N5O9S	730.5358	3.5	83.2	91	6.93	0.0	even	ok
	92	C38H78N5O2P2S	730.5346	1.8	83.4	92	13.76	4.0	even	ok
	93	C26H75N15NaOP2S	730.5367	4.7	83.7	93	2.29	-2.0	even	ok
	94	C40H73N7OPS	730.5329	-0.5	83.8	94	22.72	9.0	even	ok
	95	C36H79N5NaO2P2S	730.5322	-1.5	84.4	95	14.93	1.0	even	ok
	96	C46H72N3O2S	730.5340	0.9	85.3	96	22.64	13.0	even	ok
	97	C33H82NO11P2	730.5358	3.4	85.6	97	5.29	-5.0	even	ok
	98	C24H60N25O2	730.5356	3.2	86.7	98	5.62	8.0	even	ok
	99	C52H69NNa	730.5322	-1.5	86.8	99	13.76	19.0	even	ok
	100	C26H65N19NaO4	730.5359	3.6	87.2	100	4.33	4.0	even	ok
	101	C37H82NO6P2S	730.5333	-0.0	88.3	101	21.49	-1.0	even	ok
	102	C33H73N9NaO5S	730.5348	2.0	89.2	102	9.69	2.0	even	ok
	103	C33H78N7O4P2S	730.5306	-3.7	89.5	103	3.60	0.0	even	ok
	104	C26H70N17O3P2	730.5317	-2.1	89.5	104	8.96	2.0	even	ok
	105	C33H80NO13S	730.5345	1.6	89.6	105	14.11	-5.0	even	ok
	106	C27H68N15O8	730.5370	5.1	89.7	106	1.33	2.0	even	ok
	107	C28H75N11NaO5P2	730.5320	-1.8	89.9	107	10.56	-2.0	even	ok
	108	C49H73NNaS	730.5356	3.2	90.4	108	6.18	14.0	even	ok
	109	C35H83NNaO6P2S	730.5309	-3.3	90.9	109	4.32	-4.0	even	ok
	110	C30H77N5O12P	730.5301	-4.4	92.2	110	1.99	-4.0	even	ok
	111	C29H78N7O9P2	730.5331	-0.3	92.4	111	16.58	-4.0	even	ok
	112	C31H83NNaO11P2	730.5334	0.1	93.0	112	17.22	-8.0	even	ok
	113	C26H78N9NaO8PS	730.5324	-1.2	93.1	113	11.45	-7.0	even	ok
	114	C31H81NNaO13S	730.5321	-1.7	93.8	114	11.60	-8.0	even	ok
	115	C31H78N7NaO6PS	730.5364	4.3	95.2	115	1.89	-3.0	even	ok
	116	C30H72N11O7S	730.5331	-0.2	95.2	116	15.00	1.0	even	ok
	117	C22H61N25NaO2	730.5332	-0.1	95.5	117	15.36	5.0	even	ok
	118	C32H77N5NaO9S	730.5334	0.2	96.5	118	14.25	-3.0	even	ok
	119	C54H68N	730.5346	1.8	96.7	119	7.53	22.0	even	ok
	120	C23H64N21O6	730.5343	1.4	97.7	120	8.76	3.0	even	ok
	121	C25H69N15NaO8	730.5346	1.8	98.0	121	7.30	-1.0	even	ok
770.5290	1	C56H68NO	770.5295	0.7	28.7	1	100.00	24.0	even	ok

Compound Spectrum SmartFormula Report

Meas. m/z	#	Ion Formula	m/z	err [ppm]	mSigma	# mSigma	Score	rdb	e⁻ Conf	N-Rule
	2	C54H69NNaO	770.5271	-2.4	30.6	2	42.75	21.0	even	ok
	3	C51H68N3O3	770.5255	-4.5	35.5	3	8.78	20.0	even	ok
	4	C50H69N5P	770.5285	-0.6	40.6	4	75.63	20.0	even	ok
	5	C49H73NO4P	770.5272	-2.4	46.5	5	28.63	15.0	even	ok
	6	C48H70N5NaP	770.5261	-3.8	49.7	6	10.64	17.0	even	ok
	7	C48H74N3OP2	770.5302	1.5	51.6	7	38.10	15.0	even	ok
	8	C45H68N7O4	770.5327	4.8	57.0	8	3.56	16.0	even	ok
	9	C44H65N11Na	770.5317	3.5	59.9	9	9.53	18.0	even	ok
	10	C46H75N3NaOP2	770.5278	-1.6	61.9	10	25.92	12.0	even	ok
	11	C44H72N3O8	770.5314	3.1	65.4	11	9.94	11.0	even	ok
	12	C43H69N7NaO4	770.5303	1.7	67.8	12	19.97	13.0	even	ok
	13	C38H69N13PS	770.5252	-5.0	67.9	13	2.18	12.0	even	ok
	14	C41H64N13O2	770.5300	1.4	70.1	14	21.76	17.0	even	ok
	15	C43H74N5O3P2	770.5261	-3.7	72.3	15	5.08	11.0	even	ok
	16	C42H73N3NaO8	770.5290	-0.0	76.6	16	28.03	8.0	even	ok
	17	C36H69N13NaO2S	770.5310	2.6	78.1	17	8.38	9.0	even	ok
	18	C40H68N9O6	770.5287	-0.4	78.8	18	22.89	12.0	even	ok
	19	C34H64N19S	770.5307	2.2	79.8	19	9.60	13.0	even	ok
	20	C38H79N5NaO3P2S	770.5271	-2.5	80.8	20	8.19	3.0	even	ok
	21	C39H65N13NaO2	770.5276	-1.8	81.0	21	11.72	14.0	even	ok
	22	C41H74N5NaO5P	770.5320	3.9	81.4	22	3.18	8.0	even	ok
	23	C36H74N11OP2S	770.5268	-2.8	82.6	23	6.15	7.0	even	ok
	24	C37H60N19	770.5274	-2.1	83.2	24	8.89	18.0	even	ok
	25	C39H69N11O3P	770.5317	3.5	83.5	25	3.78	12.0	even	ok
	26	C39H72N5O10	770.5274	-2.1	88.0	26	7.32	7.0	even	ok
	27	C53H72NOS	770.5329	5.1	89.4	27	1.04	19.0	even	ok
	28	C38H69N9NaO6	770.5263	-3.5	89.9	28	2.87	9.0	even	ok
	29	C33H68N15O4S	770.5294	0.5	90.2	29	13.51	8.0	even	ok
	30	C51H73NNaOS	770.5305	2.0	90.5	30	8.91	16.0	even	ok
	31	C40H78NNaO9P	770.5306	2.1	90.6	31	6.50	3.0	even	ok
	32	C45H76N3O3S2	770.5323	4.2	91.3	32	1.96	10.0	even	ok
	33	C32H65N19NaS	770.5283	-0.9	91.5	33	11.13	10.0	even	ok
	34	C36H64N15O4	770.5260	-3.9	92.0	34	2.02	13.0	even	ok
	35	C38H73N7O7P	770.5304	1.8	92.6	35	7.16	7.0	even	ok
	36	C48H72N3O3S	770.5289	-0.1	92.6	36	17.19	15.0	even	ok
	37	C34H74N11NaO3PS	770.5327	4.8	92.7	37	0.96	4.0	even	ok
	38	C35H78N7O5P2S	770.5255	-4.6	93.0	38	1.12	2.0	even	ok
	39	C47H73N5PS	770.5319	3.7	94.3	39	2.49	15.0	even	ok
	40	C32H69N17OPS	770.5324	4.4	94.4	40	1.20	8.0	even	ok
	41	C37H70N11NaO3P	770.5293	0.4	94.6	41	11.62	9.0	even	ok
	42	C43H77N3NaO3S2	770.5299	1.1	95.2	42	10.70	7.0	even	ok
	43	C41H72N9OS2	770.5296	0.7	95.9	43	12.00	11.0	even	ok
	44	C46H73N3NaO3S	770.5265	-3.3	96.2	44	3.18	12.0	even	ok
	45	C35H65N17OP	770.5290	0.0	96.7	45	11.89	13.0	even	ok
	46	C42H78N5NaPS2	770.5328	5.0	96.9	46	0.81	7.0	even	ok
	47	C43H82NO2P2S2	770.5257	-4.3	97.0	47	1.42	5.0	even	ok

Compound Spectrum SmartFormula Report

Meas. m/z	#	Ion Formula	m/z	err [ppm]	mSigma	# mSigma	Score	rdb	e⁻ Conf	N-Rule
	48	C46H77NO4PS	770.5305	2.0	97.1	48	6.44	10.0	even	ok
	49	C44H68N9OS	770.5262	-3.6	97.2	49	2.37	16.0	even	ok
	50	C38H76NO14	770.5260	-3.9	97.6	50	1.58	2.0	even	ok
	51	C45H74N5NaPS	770.5295	0.6	98.3	51	11.28	12.0	even	ok
	52	C36H71N13NaP2	770.5323	4.3	99.3	52	1.07	9.0	even	ok
774.5595	1	C29H85NNaO19	774.5608	1.7	65.2	1	54.61	-12.0	even	ok
	2	C27H80N7O17	774.5605	1.4	65.2	2	64.63	-8.0	even	ok
	3	C26H77N11NaO13	774.5595	-0.0	67.2	3	100.00	-6.0	even	ok
	4	C24H72N17O11	774.5592	-0.4	67.5	4	88.04	-2.0	even	ok
	5	C23H69N21NaO7	774.5581	-1.8	69.7	5	45.46	0.0	even	ok
	6	C30H86N3O14P2	774.5580	-2.0	73.5	6	35.77	-9.0	even	ok
	7	C31H84NO19	774.5632	4.8	74.9	7	4.65	-9.0	even	ok
	8	C29H83N7NaO10P2	774.5569	-3.3	75.4	8	14.57	-7.0	even	ok
	9	C27H78N13O8P2	774.5566	-3.7	75.6	9	11.22	-3.0	even	ok
	10	C28H76N11O13	774.5619	3.1	76.4	10	16.52	-3.0	even	ok
	11	C30H81N5NaO15	774.5621	3.4	76.4	11	12.92	-7.0	even	ok
	12	C26H75N17NaO4P2	774.5555	-5.1	77.7	12	3.34	-1.0	even	ok
	13	C27H73N15NaO9	774.5608	1.7	78.2	13	33.67	-1.0	even	ok
	14	C25H68N21O7	774.5605	1.3	78.4	14	39.65	3.0	even	ok
	15	C23H66N27NaP	774.5624	3.8	79.8	15	8.57	5.0	even	ok
	16	C24H65N25NaO3	774.5594	-0.0	80.5	16	59.87	5.0	even	ok
	17	C22H60N31O	774.5592	-0.4	81.0	17	51.93	9.0	even	ok
	18	C31H82N7O10P2	774.5593	-0.2	84.7	18	47.19	-4.0	even	ok
	19	C33H87NNaO12P2	774.5596	0.1	84.8	19	48.44	-8.0	even	ok
	20	C32H81N5O13P	774.5563	-4.1	86.2	20	5.39	-4.0	even	ok
	21	C30H79N11NaO6P2	774.5582	-1.6	86.5	21	25.15	-2.0	even	ok
	22	C28H74N17O4P2	774.5579	-2.0	86.5	22	20.95	2.0	even	ok
	23	C29H72N15O9	774.5632	4.8	87.6	23	2.79	2.0	even	ok
	24	C27H71N21NaP2	774.5569	-3.3	88.6	24	8.40	4.0	even	ok
	25	C29H85N5O13PS	774.5597	0.3	88.9	25	48.80	-9.0	even	ok
	26	C28H69N19NaO5	774.5621	3.4	89.3	26	7.64	4.0	even	ok
	27	C26H64N25O3	774.5618	3.1	89.4	27	9.72	8.0	even	ok
	28	C35H84NO14S	774.5607	1.6	89.8	28	27.39	-5.0	even	ok
	29	C23H69N25OPS	774.5570	-3.2	91.8	29	7.93	3.0	even	ok
	30	C30H84N3O16S	774.5567	-3.6	92.9	30	7.28	-9.0	even	ok
	31	C25H74N19NaO3PS	774.5573	-2.9	93.7	31	9.23	-1.0	even	ok
	32	C35H86NO12P2	774.5620	3.2	94.7	32	6.85	-5.0	even	ok
	33	C26H77N15O7PS	774.5583	-1.5	95.4	33	17.98	-3.0	even	ok
	34	C32H78N11O6P2	774.5606	1.5	96.0	34	17.33	1.0	even	ok
	35	C34H83N5NaO8P2	774.5609	1.9	96.1	35	14.44	-3.0	even	ok
	36	C28H82N9NaO9PS	774.5586	-1.1	97.3	36	19.31	-7.0	even	ok
	37	C33H77N9O9P	774.5576	-2.4	97.6	37	10.32	1.0	even	ok
	38	C37H87NNaO7P2S	774.5571	-3.1	97.6	38	8.25	-4.0	even	ok
	39	C31H75N15NaO2P2	774.5596	0.1	97.6	39	27.54	3.0	even	ok
	40	C29H70N21P2	774.5593	-0.2	97.6	40	26.48	7.0	even	ok
	41	C35H82N3NaO11P	774.5579	-2.0	97.9	41	12.40	-3.0	even	ok

Compound Spectrum SmartFormula Report

Meas. m/z	#	Ion Formula	m/z	err [ppm]	mSigma	# mSigma	Score	rdb	e¯ Conf	N-Rule
	42	C32H86N3NaO11PS	774.5613	2.4	98.5	42	12.43	-8.0	even	ok
	43	C30H69N19O3P	774.5563	-4.1	99.0	43	3.01	7.0	even	ok
	44	C32H74N13NaO5P	774.5566	-3.7	99.1	44	3.94	3.0	even	ok
	45	C36H80N5O10S	774.5620	3.3	100.1	45	6.32	0.0	even	ok
796.5373	1	C65H45N3NaO38PS2	796.5373	0.0	48.5	1	100.00	46.0	even	ok
	2	C63H40N9O36PS2	796.5372	-0.1	49.0	2	94.44	50.0	even	ok
	3	C67H44N3O38PS2	796.5385	1.5	49.1	3	53.54	49.0	even	ok
	4	C68H43NO41S2	796.5370	-0.3	49.6	4	87.02	49.0	even	ok
	5	C66H41N7NaO34PS2	796.5380	0.9	49.7	5	71.01	51.0	even	ok
	6	C66H170NaO32PS2	796.5370	-0.3	49.8	6	86.54	-17.0	even	ok
	7	C67H40N5NaO37S2	796.5365	-1.0	49.8	7	66.58	51.0	even	ok
	8	C65H35N11O35S2	796.5364	-1.2	49.8	8	61.64	55.0	even	ok
	9	C64H36N13O32PS2	796.5379	0.7	50.0	9	75.48	55.0	even	ok
	10	C62H37N13NaO32PS2	796.5367	-0.8	50.3	10	71.04	52.0	even	ok
	11	C62H44N5O40PS2	796.5365	-1.0	50.3	11	66.19	45.0	even	ok
	12	C64H165N6O30PS2	796.5369	-0.5	50.5	12	79.55	-13.0	even	ok
	13	C68H169O32PS2	796.5383	1.2	50.9	13	60.04	-14.0	even	ok
	14	C63H169N2O34PS2	796.5362	-1.4	50.9	14	55.24	-18.0	even	ok
	15	C65H171N2NaO29P2S2	796.5385	1.5	51.0	15	50.32	-17.0	even	ok
	16	C63H33N17NaO28PS2	796.5373	0.0	51.1	16	92.54	57.0	even	ok
	17	C60H32N19O30PS2	796.5365	-1.0	51.2	17	64.26	56.0	even	ok
	18	C63H50NNaO39P2S2	796.5382	1.1	51.3	18	62.08	41.0	even	ok
	19	C61H28N23O26PS2	796.5372	-0.2	51.7	19	86.86	61.0	even	ok
	20	C67H166N4NaO28PS2	796.5377	0.5	51.7	20	76.78	-12.0	even	ok
	21	C68H165N2NaO31S2	796.5362	-1.4	51.8	21	53.20	-12.0	even	ok
	22	C66H160N8O29S2	796.5361	-1.6	51.9	22	48.73	-8.0	even	ok
	23	C63H166N8O27P2S2	796.5384	1.4	52.0	23	53.17	-13.0	even	ok
	24	C63H162N10NaO26PS2	796.5364	-1.2	52.0	24	57.76	-11.0	even	ok
	25	C61H38N15NaO29P2S2	796.5382	1.1	52.0	25	60.88	52.0	even	ok
	26	C65H161N10O26PS2	796.5376	0.3	52.1	26	80.80	-8.0	even	ok
	27	C61H45N7O37P2S2	796.5380	0.9	52.4	27	64.70	45.0	even	ok
	28	C61H157N16O24PS2	796.5362	-1.4	52.9	28	51.72	-7.0	even	ok
	29	C65H32N17O28PS2	796.5385	1.5	53.0	29	47.57	60.0	even	ok
	30	C62H170N4O31P2S2	796.5377	0.5	53.0	30	73.08	-18.0	even	ok
	31	C59H33N21O27P2S2	796.5380	0.9	53.2	31	63.29	56.0	even	ok
	32	C60H25N27NaO22PS2	796.5367	-0.8	53.3	32	64.56	63.0	even	ok
	33	C64H158N14NaO22PS2	796.5370	-0.3	53.5	33	76.98	-6.0	even	ok
	34	C66H31N15O31S2	796.5370	-0.3	53.6	34	76.71	60.0	even	ok
	35	C62H163N12NaO23P2S2	796.5379	0.7	53.8	35	67.01	-11.0	even	ok
	36	C69H39N5O37S2	796.5377	0.5	53.8	36	72.15	54.0	even	ok
	37	C68H36N9NaO33S2	796.5372	-0.2	53.8	37	80.84	56.0	even	ok
	38	C64H29N21NaO24PS2	796.5380	0.9	53.9	38	62.36	62.0	even	ok
	39	C60H42N11NaO33P2S2	796.5375	0.2	53.9	39	79.25	47.0	even	ok
	40	C65H28N19NaO27S2	796.5365	-1.0	54.1	40	57.99	62.0	even	ok
	41	C62H153N20O20PS2	796.5369	-0.5	54.2	41	70.64	-2.0	even	ok
	42	C58H20N33O20PS2	796.5365	-1.0	54.2	42	58.29	67.0	even	ok

Compound Spectrum SmartFormula Report

Meas. m/z	#	Ion Formula	m/z	err [ppm]	mSigma	# mSigma	Score	rdb	e⁻ Conf	N-Rule
	43	C63H23N25O25S2	796.5364	-1.2	54.2	43	53.57	66.0	even	ok
	44	C62H24N27O22PS2	796.5379	0.7	54.3	44	66.15	66.0	even	ok
	45	C63H159N16NaO19P2S2	796.5385	1.5	54.6	45	45.16	-6.0	even	ok
	46	C61H167N8NaO27P2S2	796.5372	-0.1	54.9	46	78.84	-16.0	even	ok
	47	C59H26N29NaO19P2S2	796.5382	1.1	54.9	47	55.81	63.0	even	ok
	48	C60H158N18O21P2S2	796.5377	0.5	54.9	48	68.99	-7.0	even	ok
	49	C58H30N25NaO23P2S2	796.5375	0.2	55.1	49	76.54	58.0	even	ok
	50	C58H37N17O31P2S2	796.5374	0.0	55.2	50	80.57	51.0	even	ok
	51	C61H154N22O17P2S2	796.5384	1.4	55.5	51	47.64	-2.0	even	ok
	52	C68H162N8NaO24PS2	796.5384	1.3	55.7	52	47.80	-7.0	even	ok
	53	C61H21N31NaO18PS2	796.5373	0.0	55.7	53	80.19	68.0	even	ok
	54	C66H157N14O22PS2	796.5382	1.2	55.8	54	51.58	-3.0	even	ok
	55	C61H150N24NaO16PS2	796.5364	-1.2	56.0	55	50.71	0.0	even	ok
	56	C61H174O35P2S2	796.5371	-0.3	56.0	56	71.76	-23.0	even	ok
	57	C57H21N35O17P2S2	796.5380	0.9	56.0	57	57.91	67.0	even	ok
	58	C70H164N2O31S2	796.5374	0.1	56.1	58	76.14	-9.0	even	ok
	59	C60H49N3O41P2S2	796.5374	0.1	56.1	59	78.24	40.0	even	ok
	60	C67H156N12O25S2	796.5368	-0.7	56.2	60	61.46	-3.0	even	ok
	61	C59H162N14O25P2S2	796.5371	-0.3	56.3	61	71.03	-12.0	even	ok
	62	C68H45NO39P2S	796.5377	0.5	56.3	62	67.57	49.0	even	ok
	63	C59H16N37O16PS2	796.5372	-0.2	56.3	63	74.84	72.0	even	ok
	64	C70H40N3O38PS	796.5369	-0.6	56.3	64	64.50	54.0	even	ok
	65	C69H161N6NaO27S2	796.5369	-0.5	56.4	65	65.41	-7.0	even	ok
	66	C56H25N31O21P2S2	796.5373	0.0	56.4	66	77.64	62.0	even	ok
	67	C58H161N12NaO30S2	796.5385	1.5	56.8	67	43.39	-15.0	even	ok
	68	C65H154N18NaO18PS2	796.5377	0.5	56.9	68	65.23	-1.0	even	ok
	69	C59H145N30O14PS2	796.5362	-1.4	56.9	69	45.34	4.0	even	ok
	70	C66H153N16NaO21S2	796.5362	-1.4	57.1	70	44.83	-1.0	even	ok
	71	C59H155N22NaO17P2S2	796.5372	-0.2	57.1	71	73.22	-5.0	even	ok
	72	C57H34N21NaO27P2S2	796.5368	-0.6	57.2	72	61.62	53.0	even	ok
	73	C64H148N22O19S2	796.5361	-1.6	57.2	73	40.99	3.0	even	ok
	74	C63H149N24O16PS2	796.5376	0.3	57.4	74	68.53	3.0	even	ok
	75	C69H41N5O35P2S	796.5384	1.3	57.5	75	45.87	54.0	even	ok
	76	C60H151N26NaO13P2S2	796.5379	0.7	57.6	76	59.37	0.0	even	ok
	77	C59H46N7NaO37P2S2	796.5368	-0.6	57.6	77	60.89	42.0	even	ok
	78	C60H171N4NaO31P2S2	796.5365	-1.0	57.8	78	52.23	-21.0	even	ok
	79	C56H149N26NaO20S2	796.5385	1.5	57.9	79	42.10	-4.0	even	ok
	80	C72H45NO34P2S2	796.5364	-1.1	57.9	80	49.22	53.0	even	ok
	81	C58H13N41NaO12PS2	796.5367	-0.8	58.1	81	55.03	74.0	even	ok
	82	C69H37N7NaO34PS	796.5363	-1.2	58.1	82	45.98	56.0	even	ok
	83	C56H18N39NaO13P2S2	796.5375	0.2	58.2	83	69.24	69.0	even	ok
	84	C56H156N18O28S2	796.5383	1.3	58.3	84	44.88	-11.0	even	ok
	85	C64H43NO46S	796.5383	1.2	58.4	85	45.98	45.0	even	ok
	86	C58H159N18NaO21P2S2	796.5365	-1.0	58.4	86	51.03	-10.0	even	ok
	87	C67H42N5NaO35P2S	796.5371	-0.2	58.5	87	68.57	51.0	even	ok
	88	C57H150N28O15P2S2	796.5371	-0.3	58.5	88	65.83	-1.0	even	ok

Compound Spectrum SmartFormula Report

Meas. m/z	#	Ion Formula	m/z	err [ppm]	mSigma	# mSigma	Score	rdb	e¯ Conf	N-Rule
	89	C63H20N31O18PS2	796.5385	1.5	58.7	89	39.72	71.0	even	ok
	90	C55H29N27O25P2S2	796.5367	-0.8	58.7	90	54.72	57.0	even	ok
	91	C55H22N35NaO17P2S2	796.5368	-0.6	58.7	91	58.40	64.0	even	ok
	92	C64H36N9NaO38S	796.5384	1.4	58.7	92	42.07	52.0	even	ok
	93	C58H146N32O11P2S2	796.5377	0.5	58.8	93	61.03	4.0	even	ok
	94	C62H146N28NaO12PS2	796.5370	-0.4	58.9	94	64.33	5.0	even	ok
	95	C57H41N13O35P2S2	796.5367	-0.8	59.0	95	54.24	46.0	even	ok
	96	C67H27N19O27S2	796.5377	0.5	59.1	96	60.87	65.0	even	ok
	97	C56H8N47O10PS2	796.5365	-1.0	59.1	97	49.62	78.0	even	ok
	98	C67H32N13O32PS	796.5362	-1.4	59.1	98	41.07	60.0	even	ok
	99	C66H46NNaO39P2S	796.5365	-1.1	59.1	99	48.61	46.0	even	ok
	100	C58H168N4O38S2	796.5384	1.3	59.2	100	43.45	-22.0	even	ok
	101	C64H19N29O21S2	796.5370	-0.4	59.3	101	63.57	71.0	even	ok
	102	C58H166N10O29P2S2	796.5364	-1.2	59.3	102	46.05	-17.0	even	ok
	103	C54H144N32O18S2	796.5383	1.3	59.4	103	43.44	0.0	even	ok
	104	C66H24N23NaO23S2	796.5372	-0.2	59.4	104	67.13	67.0	even	ok
	105	C69H32N13NaO29S2	796.5379	0.7	59.5	105	56.02	61.0	even	ok
	106	C57H14N43NaO9P2S2	796.5382	1.0	59.5	106	48.01	74.0	even	ok
	107	C71H165O32PS	796.5366	-0.9	59.6	107	50.08	-9.0	even	ok
	108	C54H13N45O11P2S2	796.5373	0.0	59.6	108	70.11	73.0	even	ok
	109	C70H35N9O33S2	796.5384	1.3	59.6	109	42.01	59.0	even	ok
	110	C68H38N9NaO31P2S	796.5378	0.6	59.6	110	56.75	56.0	even	ok
	111	C60H141N34O10PS2	796.5369	-0.5	59.6	111	58.95	9.0	even	ok
	112	C73H41NNaO36PS	796.5377	0.4	59.7	112	60.90	55.0	even	ok
	113	C54H28N25NaO30S2	796.5381	1.0	59.7	113	49.00	54.0	even	ok
	114	C65H37N11O33P2S	796.5370	-0.4	59.8	114	61.60	55.0	even	ok
	115	C62H17N35NaO14PS2	796.5380	0.9	59.8	115	51.51	73.0	even	ok
	116	C61H147N30NaO9P2S2	796.5385	1.5	59.9	116	38.08	5.0	even	ok
	117	C56H154N24O19P2S2	796.5364	-1.2	60.0	117	44.88	-6.0	even	ok
	118	C71H36N7O34PS	796.5375	0.3	60.1	118	63.94	59.0	even	ok
	119	C52H16N39NaO20S2	796.5381	1.0	60.1	119	48.48	65.0	even	ok
	120	C63H16N33NaO17S2	796.5365	-1.0	60.1	120	47.53	73.0	even	ok
	121	C60H12N41O12PS2	796.5379	0.7	60.2	121	54.55	77.0	even	ok
	122	C62H31N15O36S	796.5383	1.2	60.2	122	43.36	56.0	even	ok
	123	C53H17N41O15P2S2	796.5367	-0.8	60.2	123	51.75	68.0	even	ok
	124	C61H11N39O15S2	796.5364	-1.2	60.3	124	43.82	77.0	even	ok
	125	C72H40N3NaO35S2	796.5385	1.5	60.4	125	37.62	55.0	even	ok
	126	C55H153N22NaO24S2	796.5378	0.6	60.4	126	55.42	-9.0	even	ok
	127	C63H40N5NaO42S	796.5378	0.6	60.6	127	56.30	47.0	even	ok
	128	C64H41N7O37P2S	796.5363	-1.2	60.7	128	42.70	50.0	even	ok
	129	C55H9N49O7P2S2	796.5380	0.9	60.7	129	49.76	78.0	even	ok
	130	C54H137N40NaO10S2	796.5385	1.5	60.8	130	38.30	7.0	even	ok
	131	C66H33N15O29P2S	796.5377	0.5	60.8	131	58.30	60.0	even	ok
	132	C59H142N36O7P2S2	796.5384	1.4	60.8	132	40.12	9.0	even	ok
	133	C70H166N2O29P2S	796.5381	0.9	60.8	133	48.23	-9.0	even	ok
	134	C56H38N17NaO31P2S2	796.5361	-1.5	60.9	134	37.93	48.0	even	ok

Compound Spectrum SmartFormula Report

Meas. m/z	#	Ion Formula	m/z	err [ppm]	mSigma	# mSigma	Score	rdb	e⁻ Conf	N-Rule
	135	C54H26N31NaO21P2S2	796.5361	-1.5	60.9	135	37.77	59.0	even	ok
	136	C57H165N8NaO34S2	796.5378	0.6	60.9	136	54.37	-20.0	even	ok
	137	C56H147N32NaO11P2S2	796.5365	-1.0	60.9	137	46.75	1.0	even	ok
	138	C65H161N6NaO32S	796.5381	1.0	61.1	138	45.82	-11.0	even	ok
	139	C57H143N36NaO7P2S2	796.5372	-0.2	61.1	139	63.82	6.0	even	ok
	140	C56H40N11NaO40S2	796.5381	1.0	61.2	140	46.45	43.0	even	ok
	141	C52H23N31O28S2	796.5380	0.8	61.3	141	49.97	58.0	even	ok
	142	C68H167N2NaO29P2S	796.5369	-0.6	61.3	142	54.69	-12.0	even	ok
	143	C59H138N38NaO6PS2	796.5364	-1.2	61.6	143	41.90	11.0	even	ok
	144	C70H33N11NaO30PS	796.5370	-0.4	61.7	144	57.42	61.0	even	ok
	145	C50H11N45O18S2	796.5380	0.8	61.8	145	49.30	69.0	even	ok
	146	C53H141N36NaO14S2	796.5378	0.6	61.8	146	52.99	2.0	even	ok
	147	C59H9N45NaO8PS2	796.5373	0.0	61.8	147	65.46	79.0	even	ok
	148	C66H150N22NaO14PS2	796.5384	1.3	62.0	148	38.78	4.0	even	ok
	149	C53H148N28O22S2	796.5377	0.4	62.1	149	55.95	-5.0	even	ok
	150	C53H10N49NaO7P2S2	796.5368	-0.6	62.1	150	51.94	75.0	even	ok
	151	C68H152N16O21S2	796.5374	0.1	62.1	151	62.26	2.0	even	ok
	152	C64H145N28O12PS2	796.5382	1.2	62.2	152	41.77	8.0	even	ok
	153	C64H165N2NaO36S	796.5375	0.2	62.3	153	60.65	-16.0	even	ok
	154	C61H35N11O40S	796.5376	0.4	62.3	154	56.77	51.0	even	ok
	155	C52H132N46O8S2	796.5383	1.3	62.3	155	39.46	11.0	even	ok
	156	C68H28N17O28PS	796.5369	-0.6	62.3	156	52.58	65.0	even	ok
	157	C64H34N15NaO29P2S	796.5365	-1.1	62.3	157	43.42	57.0	even	ok
	158	C62H24N23NaO28S	796.5384	1.4	62.3	158	37.32	63.0	even	ok
	159	C71H160N6O27S2	796.5381	1.0	62.4	159	44.97	-4.0	even	ok
	160	C50H4N53NaO10S2	796.5381	1.0	62.4	160	44.99	76.0	even	ok
	161	C55H160N14O32S2	796.5377	0.5	62.5	161	55.06	-16.0	even	ok
	162	C57H4N51O6PS2	796.5372	-0.2	62.5	162	60.75	83.0	even	ok
	163	C69H34N13NaO27P2S	796.5385	1.5	62.5	163	35.84	61.0	even	ok
	164	C54H142N38O9P2S2	796.5364	-1.2	62.5	164	41.04	5.0	even	ok
	165	C55H138N42O5P2S2	796.5371	-0.3	62.5	165	57.30	10.0	even	ok
	166	C70H157N10NaO23S2	796.5376	0.3	62.5	166	57.88	-2.0	even	ok
	167	C57H133N44O4PS2	796.5362	-1.4	62.6	167	37.41	15.0	even	ok
	168	C63H156N12O30S	796.5380	0.9	62.6	168	46.77	-7.0	even	ok
	169	C54H35N17O38S2	796.5380	0.8	62.7	169	47.52	47.0	even	ok
	170	C65H144N26O15S2	796.5367	-0.7	62.7	170	49.39	8.0	even	ok
	171	C58H50N3NaO41P2S2	796.5362	-1.5	62.7	171	35.74	37.0	even	ok
	172	C66H162N8O27P2S	796.5367	-0.8	62.7	172	48.68	-8.0	even	ok
	173	C61H28N19NaO32S	796.5378	0.6	62.8	173	52.47	58.0	even	ok
	174	C67H149N20NaO17S2	796.5369	-0.5	62.8	174	52.68	4.0	even	ok
	175	C52H14N45NaO11P2S2	796.5361	-1.5	62.8	175	35.29	70.0	even	ok
	176	C65H166N4O31P2S	796.5361	-1.6	62.9	176	33.34	-13.0	even	ok
	177	C58H139N40NaO3P2S2	796.5379	0.7	63.0	177	49.50	11.0	even	ok
	178	C73H165NaO29S2	796.5382	1.2	63.0	178	40.71	-8.0	even	ok
	179	C69H163N6NaO25P2S	796.5375	0.3	63.0	179	57.66	-7.0	even	ok
	180	C54H6N53NaO3P2S2	796.5375	0.2	63.1	180	58.88	80.0	even	ok

Compound Spectrum SmartFormula Report

Meas. m/z	#	Ion Formula	m/z	err [ppm]	mSigma	# mSigma	Score	rdb	e¯ Conf	N-Rule
	181	C65H30N19NaO25P2S	796.5371	-0.2	63.2	181	58.31	62.0	even	ok
	182	C67H29N19O25P2S	796.5383	1.3	63.4	182	37.73	65.0	even	ok
	183	C51H20N35NaO24S2	796.5374	0.1	63.4	183	59.33	60.0	even	ok
	184	C51H136N42O12S2	796.5377	0.4	63.5	184	53.37	6.0	even	ok
	185	C63H142N32NaO8PS2	796.5377	0.5	63.5	185	52.29	10.0	even	ok
	186	C51H5N55O5P2S2	796.5367	-0.8	63.6	186	45.95	79.0	even	ok
	187	C64H141N30NaO11S2	796.5362	-1.4	63.7	187	35.65	10.0	even	ok
	188	C72H161N4O28PS	796.5372	-0.1	63.8	188	59.35	-4.0	even	ok
	189	C60H19N29O26S	796.5383	1.2	63.8	189	38.40	67.0	even	ok
	190	C62H29N21O27P2S	796.5363	-1.2	63.9	190	38.07	61.0	even	ok
	191	C62H136N36O9S2	796.5361	-1.6	63.9	191	32.54	14.0	even	ok
	192	C62H160N8O34S	796.5373	0.0	63.9	192	60.59	-12.0	even	ok
	193	C61H137N38O6PS2	796.5376	0.3	64.0	193	54.85	14.0	even	ok
	194	C70H33N15O24P2S2	796.5364	-1.1	64.0	194	39.94	64.0	even	ok
	195	C62H44NNaO46S	796.5371	-0.3	64.1	195	55.35	42.0	even	ok
	196	C56H134N46OP2S2	796.5377	0.5	64.2	196	50.83	15.0	even	ok
	197	C67H158N12O23P2S	796.5374	0.1	64.2	197	58.71	-3.0	even	ok
	198	C49H8N49NaO14S2	796.5374	0.1	64.2	198	57.81	71.0	even	ok
	199	C67H25N21NaO24PS	796.5363	-1.3	64.3	199	37.11	67.0	even	ok
	200	C59H23N25O30S	796.5376	0.4	64.4	201	52.79	62.0	even	ok
	201	C53H32N21NaO34S2	796.5374	0.1	64.4	202	57.19	49.0	even	ok
	202	C52H145N32NaO18S2	796.5371	-0.2	64.4	203	55.61	-3.0	even	ok
	203	C54H157N18NaO28S2	796.5371	-0.2	64.5	205	55.67	-14.0	even	ok
	204	C63H25N25O23P2S	796.5370	-0.4	64.5	206	52.32	66.0	even	ok
	205	C72H38N9NaO26P2S2	796.5366	-0.9	64.6	207	42.11	60.0	even	ok
	206	C60H32N15NaO36S	796.5371	-0.3	64.7	208	53.90	53.0	even	ok
	207	C73H41N5O30P2S2	796.5371	-0.3	64.8	209	54.09	58.0	even	ok
	208	C51H129N50NaO4S2	796.5378	0.6	65.0	210	47.59	13.0	even	ok
	209	C54H135N46NaOP2S2	796.5365	-1.0	65.1	211	40.27	12.0	even	ok
	210	C49H15N41O22S2	796.5373	-0.0	65.2	212	57.72	64.0	even	ok
	211	C74H37N5NaO32PS	796.5383	1.3	65.2	213	35.70	60.0	even	ok
	212	C65H20N27O22PS	796.5362	-1.4	65.2	214	33.12	71.0	even	ok
	213	C62H153N16NaO26S	796.5375	0.2	65.3	215	54.69	-5.0	even	ok
	214	C72H32N11O30PS	796.5382	1.1	65.3	216	38.42	64.0	even	ok
	215	C52H125N54NaS2	796.5385	1.4	65.3	217	32.78	18.0	even	ok
	216	C65H159N12NaO23P2S	796.5362	-1.4	65.3	218	33.15	-6.0	even	ok
	217	C63H149N20NaO22S	796.5381	1.0	65.5	219	39.37	0.0	even	ok
	218	C71H158N8NaO24PS	796.5367	-0.8	65.5	220	43.70	-2.0	even	ok
	219	C66H26N23NaO21P2S	796.5378	0.6	65.6	221	46.23	67.0	even	ok
	220	C61H8N45O8PS2	796.5385	1.5	65.6	222	31.34	82.0	even	ok
	221	C65H15N33O17S2	796.5377	0.5	65.7	223	48.49	76.0	even	ok
	222	C60H134N42NaO2PS2	796.5370	-0.4	65.7	224	50.78	16.0	even	ok
	223	C60H39N7O44S	796.5370	-0.5	65.8	225	48.93	46.0	even	ok
	224	C56H47N3O48S2	796.5380	0.8	65.8	226	42.48	36.0	even	ok
	225	C73H31N9O33S	796.5367	-0.8	65.9	227	43.11	64.0	even	ok
	226	C68H23N23O23S2	796.5384	1.3	65.9	228	33.92	70.0	even	ok

Compound Spectrum SmartFormula Report

Meas. m/z	#	Ion Formula	m/z	err [ppm]	mSigma	# mSigma	Score	rdb	e¯ Conf	N-Rule
	227	C75H36N3NaO35S	796.5368	-0.6	66.0	229	46.02	60.0	even	ok
	228	C47H3N55O12S2	796.5373	-0.0	66.0	230	55.86	75.0	even	ok
	229	C51H27N27O32S2	796.5373	-0.0	66.0	231	56.05	53.0	even	ok
	230	C67H20N27NaO19S2	796.5378	0.7	66.1	232	44.73	72.0	even	ok
	231	C50H133N46NaO8S2	796.5371	-0.2	66.2	233	52.20	8.0	even	ok
	232	C52H152N24O26S2	796.5370	-0.4	66.2	234	49.30	-10.0	even	ok
	233	C50H140N38O16S2	796.5370	-0.4	66.2	235	49.10	1.0	even	ok
	234	C69H153N14O22PS	796.5366	-0.9	66.2	236	39.64	2.0	even	ok
	235	C62H7N43O11S2	796.5370	-0.4	66.2	237	49.79	82.0	even	ok
	236	C50H2N59NaOP2S2	796.5361	-1.5	66.4	238	31.00	81.0	even	ok
	237	C64H12N37NaO13S2	796.5372	-0.2	66.4	239	52.69	78.0	even	ok
	238	C70H159N10NaO21P2S	796.5382	1.1	66.4	240	36.94	-2.0	even	ok
	239	C58H129N48PS2	796.5369	-0.5	66.4	241	46.48	20.0	even	ok
	240	C58H27N21O34S	796.5370	-0.5	66.5	242	47.53	57.0	even	ok
	241	C70H28N17NaO25S2	796.5385	1.5	66.6	243	30.46	66.0	even	ok
	242	C59H16N33NaO22S	796.5378	0.5	66.6	244	45.98	69.0	even	ok
	243	C61H157N12NaO30S	796.5368	-0.7	66.6	245	44.15	-10.0	even	ok
	244	C49H124N56O2S2	796.5377	0.4	66.6	246	47.85	17.0	even	ok
	245	C71H29N15NaO26PS	796.5377	0.4	66.7	247	47.77	66.0	even	ok
	246	C64H21N29O19P2S	796.5377	0.4	66.7	248	47.44	71.0	even	ok
	247	C66H155N16NaO19P2S	796.5369	-0.6	66.7	249	45.10	-1.0	even	ok
	248	C61H164N4O38S	796.5367	-0.8	66.8	250	41.03	-17.0	even	ok
	249	C63H154N18O21P2S	796.5360	-1.6	66.9	251	28.79	-2.0	even	ok
	250	C60H148N22O24S	796.5373	0.0	67.0	252	54.54	-1.0	even	ok
	251	C72H28N13NaO29S	796.5362	-1.4	67.0	253	30.90	66.0	even	ok
	252	C60H5N49NaO4PS2	796.5380	0.8	67.0	254	40.24	84.0	even	ok
	253	C61H144N26O20S	796.5380	0.9	67.0	255	40.14	4.0	even	ok
	254	C69H24N21O24PS	796.5375	0.3	67.1	256	50.07	70.0	even	ok
	255	C55H44N7NaO44S2	796.5374	0.2	67.1	257	51.82	38.0	even	ok
	256	C58H20N29NaO26S	796.5371	-0.3	67.2	258	49.35	64.0	even	ok
	257	C62H22N29NaO19P2S	796.5365	-1.1	67.2	259	36.51	68.0	even	ok
	258	C68H154N16O19P2S	796.5381	0.9	67.3	260	38.52	2.0	even	ok
	259	C71H158N12O18P2S2	796.5361	-1.5	67.3	261	30.01	1.0	even	ok
	260	C61H4N47NaO7S2	796.5365	-1.0	67.3	262	36.85	84.0	even	ok
	261	C60H12N37NaO18S	796.5384	1.4	67.5	264	31.19	74.0	even	ok
	262	C48H12N45NaO18S2	796.5368	-0.7	67.6	265	41.71	66.0	even	ok
	263	C61H45N3NaO43PS	796.5386	1.6	67.6	266	28.07	42.0	even	ok
	264	C71H36N3NaO40	796.5381	1.0	67.6	267	37.42	56.0	even	ok
	265	C74H166N2O24P2S2	796.5368	-0.6	67.8	268	42.55	-5.0	even	ok
	266	C73H163N6NaO20P2S2	796.5363	-1.3	67.8	269	31.98	-3.0	even	ok
	267	C48H128N52O6S2	796.5370	-0.4	68.0	270	45.98	12.0	even	ok
	268	C50H24N31NaO28S2	796.5368	-0.7	68.1	271	41.03	55.0	even	ok
	269	C64H150N22O17P2S	796.5367	-0.8	68.1	272	40.11	3.0	even	ok
	270	C59H36N11NaO40S	796.5364	-1.1	68.2	273	34.30	48.0	even	ok
	271	C57H11N39O20S	796.5376	0.4	68.2	274	46.19	73.0	even	ok
	272	C59H33N17NaO33PS	796.5386	1.6	68.2	275	27.52	53.0	even	ok

Compound Spectrum SmartFormula Report

Meas. m/z	#	Ion Formula	m/z	err [ppm]	mSigma	# mSigma	Score	rdb	e⁻ Conf	N-Rule
	273	C59H152N18O28S	796.5367	-0.8	68.4	276	38.57	-6.0	even	ok
	274	C51H149N28NaO22S2	796.5365	-1.1	68.4	277	34.86	-8.0	even	ok
	275	C68H35N5O42	796.5373	-0.0	68.5	278	51.09	55.0	even	ok
	276	C62H170NaO37PS	796.5383	1.2	68.6	279	32.21	-21.0	even	ok
	277	C53H39N13O42S2	796.5373	-0.0	68.6	280	51.20	42.0	even	ok
	278	C60H17N35O17P2S	796.5363	-1.2	68.7	281	31.98	72.0	even	ok
	279	C49H137N42NaO12S2	796.5365	-1.1	68.8	283	34.28	3.0	even	ok
	280	C68H21N25NaO20PS	796.5370	-0.4	68.9	284	44.36	72.0	even	ok
	281	C56H15N35O24S	796.5369	-0.5	68.9	285	43.42	68.0	even	ok
	282	C58H7N43O16S	796.5383	1.2	69.0	286	32.06	78.0	even	ok
	283	C69H31N9O38	796.5380	0.8	69.1	287	38.12	60.0	even	ok
	284	C75H162N2NaO26PS	796.5380	0.9	69.1	288	36.29	-3.0	even	ok
	285	C57H24N25NaO30S	796.5364	-1.1	69.2	289	32.91	59.0	even	ok
	286	C63H18N33NaO15P2S	796.5371	-0.2	69.2	290	46.82	73.0	even	ok
	287	C69H148N20O17S2	796.5381	1.0	69.3	291	35.26	7.0	even	ok
	288	C73H157N8O24PS	796.5379	0.7	69.3	292	38.67	1.0	even	ok
	289	C67H39NO46	796.5366	-0.9	69.3	293	36.39	50.0	even	ok
	290	C60H161N8NaO34S	796.5361	-1.5	69.3	294	27.76	-15.0	even	ok
	291	C66H140N30O11S2	796.5374	0.1	69.4	295	48.19	13.0	even	ok
	292	C46H7N51O16S2	796.5366	-0.9	69.4	296	36.34	70.0	even	ok
	293	C59H40N9O41PS	796.5385	1.4	69.4	297	28.61	46.0	even	ok
	294	C64H138N36NaO4PS2	796.5384	1.3	69.5	298	29.80	15.0	even	ok
	295	C66H16N31O18PS	796.5368	-0.6	69.5	299	40.57	76.0	even	ok
	296	C67H22N27NaO17P2S	796.5385	1.5	69.6	300	27.93	72.0	even	ok
	297	C67H151N20NaO15P2S	796.5375	0.3	69.6	301	45.63	4.0	even	ok
	298	C62H133N42O2PS2	796.5382	1.2	69.7	302	32.05	19.0	even	ok
	299	C68H145N24NaO13S2	796.5376	0.3	69.7	303	44.90	9.0	even	ok
	300	C74H156N6O27S	796.5364	-1.1	69.8	304	32.08	1.0	even	ok
	301	C76H161NaO29S	796.5365	-1.0	69.8	305	34.59	-3.0	even	ok
	302	C51H153N26O23PS2	796.5385	1.5	69.8	306	27.39	-10.0	even	ok
	303	C48H19N37O26S2	796.5366	-0.9	69.8	307	35.87	59.0	even	ok
	304	C59H145N26NaO20S	796.5368	-0.7	69.8	308	39.15	1.0	even	ok
	305	C60H141N30NaO16S	796.5375	0.2	69.8	309	46.46	6.0	even	ok
	306	C71H153N14NaO19S2	796.5382	1.1	69.8	310	32.00	3.0	even	ok
	307	C49H141N40O13PS2	796.5385	1.5	69.8	311	27.44	1.0	even	ok
	308	C57H31N17O38S	796.5363	-1.3	70.0	312	29.63	52.0	even	ok
	309	C60H158N14NaO27PS	796.5383	1.2	70.1	313	30.56	-10.0	even	ok
	310	C57H28N23O31PS	796.5384	1.4	70.1	314	27.97	57.0	even	ok
	311	C63H132N40O5S2	796.5367	-0.7	70.2	315	37.61	19.0	even	ok
	312	C65H137N34NaO7S2	796.5369	-0.5	70.2	316	40.19	15.0	even	ok
	313	C52H36N17NaO38S2	796.5368	-0.7	70.2	317	37.98	44.0	even	ok
	314	C49H144N34O20S2	796.5363	-1.2	70.2	318	30.16	-4.0	even	ok
	315	C65H17N33O15P2S	796.5383	1.3	70.4	319	29.37	76.0	even	ok
	316	C60H165N6O35PS	796.5382	1.1	70.4	320	32.49	-17.0	even	ok
	317	C57H21N31NaO23PS	796.5386	1.6	70.5	321	25.38	64.0	even	ok
	318	C59H43N3O48S	796.5363	-1.3	70.5	322	29.14	41.0	even	ok

Compound Spectrum SmartFormula Report

Meas. m/z	#	Ion Formula	m/z	err [ppm]	mSigma	# mSigma	Score	rdb	e¯ Conf	N-Rule
	319	C61H13N39O13P2S	796.5370	-0.4	70.5	323	41.96	77.0	even	ok
	320	C72H37N5O35P2	796.5367	-0.8	70.6	324	35.76	59.0	even	ok
	321	C47H132N48O10S2	796.5363	-1.2	70.7	325	29.58	7.0	even	ok
	322	C65H146N26O13P2S	796.5374	0.1	70.8	326	46.41	8.0	even	ok
	323	C47H125N56NaO2S2	796.5365	-1.1	70.8	327	31.73	14.0	even	ok
	324	C63H147N26NaO13P2S	796.5362	-1.4	70.8	328	27.05	5.0	even	ok
	325	C72H154N12NaO20PS	796.5374	0.1	70.9	329	46.48	3.0	even	ok
	326	C55H19N31O28S	796.5363	-1.3	71.1	330	28.35	63.0	even	ok
	327	C56H8N43NaO16S	796.5371	-0.3	71.1	331	42.55	75.0	even	ok
	328	C68H21N29O14P2S2	796.5364	-1.1	71.2	332	30.69	75.0	even	ok
	329	C58H149N22NaO24S	796.5361	-1.5	71.2	333	25.79	-4.0	even	ok
	330	C47H13N47NaO15PS2	796.5382	1.2	71.3	334	29.99	66.0	even	ok
	331	C70H149N18O18PS	796.5372	-0.1	71.3	335	45.02	7.0	even	ok
	332	C72H161NaO34	796.5378	0.6	71.3	336	37.74	-7.0	even	ok
	333	C67H32N9NaO38	796.5367	-0.7	71.4	337	36.10	57.0	even	ok
	334	C62H129N44NaOS2	796.5362	-1.4	71.4	338	26.89	21.0	even	ok
	335	C47H129N54O3PS2	796.5385	1.5	71.5	339	25.88	12.0	even	ok
	336	C71H29N19O20P2S2	796.5371	-0.3	71.6	340	42.11	69.0	even	ok
	337	C65H13N35NaO14PS	796.5363	-1.3	71.6	341	28.38	78.0	even	ok
	338	C57H140N32O18S	796.5367	-0.8	71.6	342	34.14	5.0	even	ok
	339	C69H160N2O36	796.5370	-0.4	71.7	343	40.05	-8.0	even	ok
	340	C70H26N23NaO16P2S2	796.5366	-0.9	71.7	344	32.43	71.0	even	ok
	341	C49H25N33NaO25PS2	796.5383	1.2	71.8	345	29.34	55.0	even	ok
	342	C50H31N23O36S2	796.5366	-0.9	71.9	346	33.32	48.0	even	ok
	343	C55H12N39NaO20S	796.5364	-1.1	71.9	347	29.73	70.0	even	ok
	344	C58H37N13NaO37PS	796.5379	0.8	71.9	348	34.93	48.0	even	ok
	345	C68H28N13NaO34	796.5374	0.1	71.9	349	43.82	62.0	even	ok
	346	C58H153N20O25PS	796.5382	1.1	72.0	350	30.75	-6.0	even	ok
	347	C45H4N55NaO12S2	796.5361	-1.6	72.0	351	24.39	72.0	even	ok
	348	C47H16N41NaO22S2	796.5361	-1.5	72.1	352	24.38	61.0	even	ok
	349	C50H150N30NaO19PS2	796.5380	0.8	72.2	353	33.73	-8.0	even	ok
	350	C66H36N5NaO42	796.5361	-1.6	72.2	354	24.13	52.0	even	ok
	351	C73H34N13NaO22P2S2	796.5372	-0.1	72.4	356	43.35	65.0	even	ok
	352	C55H16N37O21PS	796.5384	1.4	72.4	357	25.74	68.0	even	ok
	353	C63H8N41O12PS	796.5362	-1.4	72.5	358	25.29	82.0	even	ok
	354	C48H138N44NaO9PS2	796.5380	0.8	72.5	359	33.37	3.0	even	ok
	355	C75H38N3NaO33P2	796.5375	0.2	72.6	360	41.67	60.0	even	ok
	356	C74H37N9O26P2S2	796.5378	0.6	72.7	361	36.41	63.0	even	ok
	357	C77H35N3O35S	796.5380	0.9	72.7	362	31.80	63.0	even	ok
	358	C64H14N37NaO11P2S	796.5378	0.6	72.7	363	35.66	78.0	even	ok
	359	C66H40N3O43P	796.5381	1.0	72.8	364	30.55	50.0	even	ok
	360	C70H156N6O32	796.5377	0.4	72.8	365	38.10	-3.0	even	ok
	361	C74H27N13O29S	796.5374	0.1	72.9	366	43.12	69.0	even	ok
	362	C56H25N27NaO27PS	796.5379	0.7	72.9	367	33.73	59.0	even	ok
	363	C72H25N19NaO22PS	796.5383	1.3	73.0	368	26.88	71.0	even	ok
	364	C59H162N10NaO31PS	796.5376	0.4	73.1	369	38.29	-15.0	even	ok

Compound Spectrum SmartFormula Report

Meas. m/z	#	Ion Formula	m/z	err [ppm]	mSigma	# mSigma	Score	rdb	e⁻ Conf	N-Rule
	365	C70H20N25O20PS	796.5382	1.1	73.1	370	28.87	75.0	even	ok
	366	C65H27N15O36	796.5366	-0.9	73.2	371	31.45	61.0	even	ok
	367	C76H32N7NaO31S	796.5375	0.2	73.2	372	40.21	65.0	even	ok
	368	C45H8N53O13PS2	796.5381	1.0	73.2	373	30.11	70.0	even	ok
	369	C69H146N22NaO14PS	796.5367	-0.8	73.2	374	32.84	9.0	even	ok
	370	C66H11N37O13S2	796.5384	1.3	73.2	375	25.95	81.0	even	ok
	371	C71H34N9NaO31P2	796.5361	-1.5	73.3	376	23.95	61.0	even	ok
	372	C63H3N47O7S2	796.5377	0.5	73.4	377	36.63	87.0	even	ok
	373	C66H23N19O32	796.5373	-0.1	73.5	378	42.18	66.0	even	ok
	374	C47H20N39O23PS2	796.5381	1.0	73.6	379	29.56	59.0	even	ok
	375	C73H33N9O31P2	796.5373	0.0	73.7	380	42.36	64.0	even	ok
	376	C65H8N41NaO9S2	796.5378	0.7	73.7	381	33.87	83.0	even	ok
	377	C71H19N23O23S	796.5367	-0.8	73.7	382	32.17	75.0	even	ok
	378	C76H42N3NaO28P2S2	796.5379	0.7	73.7	383	32.68	59.0	even	ok
	379	C49H28N27NaO32S2	796.5361	-1.5	73.7	384	22.96	50.0	even	ok
	380	C73H24N17NaO25S	796.5368	-0.6	73.8	385	34.40	71.0	even	ok
	381	C56H32N19O35PS	796.5378	0.6	73.8	386	34.77	52.0	even	ok
	382	C68H16N31NaO15S2	796.5385	1.5	73.8	387	23.36	77.0	even	ok
	383	C51H37N19NaO35PS2	796.5383	1.2	73.8	388	27.05	44.0	even	ok
	384	C69H24N17NaO30	796.5381	1.0	73.9	389	29.68	67.0	even	ok
	385	C67H141N28O12PS	796.5366	-1.0	73.9	390	29.76	13.0	even	ok
	386	C68H147N24NaO11P2S	796.5382	1.1	73.9	391	28.00	9.0	even	ok
	387	C54H48N3NaO48S2	796.5368	-0.7	73.9	392	33.15	33.0	even	ok
	388	C73H157N4NaO30	796.5385	1.4	74.1	393	23.73	-2.0	even	ok
	389	C58H44N5O45PS	796.5378	0.6	74.3	395	34.02	41.0	even	ok
	390	C46H133N50O7PS2	796.5378	0.6	74.5	396	33.19	7.0	even	ok
	391	C66H33N11NaO35P	796.5382	1.2	74.6	397	26.53	57.0	even	ok
	392	C69H17N29NaO16PS	796.5377	0.4	74.7	398	35.56	77.0	even	ok
	393	C59H169N2O39PS	796.5375	0.2	74.7	399	38.15	-22.0	even	ok
	394	C78H36NO36P	796.5372	-0.2	74.7	400	38.74	63.0	even	ok
	395	C66H142N30O9P2S	796.5381	0.9	74.8	401	29.17	13.0	even	ok
	396	C54H20N33O25PS	796.5378	0.6	74.8	402	33.49	63.0	even	ok
	397	C57H150N24NaO21PS	796.5376	0.4	74.9	403	35.81	-4.0	even	ok
	398	C70H16N27NaO19S	796.5362	-1.5	75.0	404	22.83	77.0	even	ok
	399	C69H146N26O8P2S2	796.5361	-1.5	75.0	405	22.43	12.0	even	ok
	400	C57H157N16O29PS	796.5375	0.2	75.0	406	37.76	-11.0	even	ok
	401	C67H12N35O14PS	796.5375	0.2	75.1	407	37.23	81.0	even	ok
	402	C72H154N16O14P2S2	796.5368	-0.6	75.1	408	32.20	6.0	even	ok
	403	C52H43N9O46S2	796.5366	-0.9	75.4	409	29.20	37.0	even	ok
	404	C67H165O37P	796.5378	0.6	75.4	410	32.06	-13.0	even	ok
	405	C71H151N20NaO10P2S2	796.5363	-1.3	75.4	411	23.95	8.0	even	ok
	406	C49H32N25O33PS2	796.5381	1.0	75.5	412	27.35	48.0	even	ok
	407	C75H36N7O29PS2	796.5363	-1.3	75.6	413	23.88	63.0	even	ok
	408	C65H37N7NaO39P	796.5376	0.3	75.8	414	35.30	52.0	even	ok
	409	C74H159N10NaO16P2S2	796.5369	-0.5	75.8	415	33.48	2.0	even	ok
	410	C44H5N57NaO9PS2	796.5376	0.3	75.8	416	35.22	72.0	even	ok

Compound Spectrum SmartFormula Report

Meas. m/z	#	Ion Formula	m/z	err [ppm]	mSigma	# mSigma	Score	rdb	e¯ Conf	N-Rule
	411	C75H162N6O20P2S2	796.5375	0.2	75.9	417	36.65	0.0	even	ok
	412	C46H17N43NaO19PS2	796.5376	0.3	75.9	418	35.01	61.0	even	ok
	413	C74H153N12O20PS	796.5386	1.6	76.0	419	20.67	6.0	even	ok
	414	C55H29N23NaO31PS	796.5372	-0.1	76.4	420	37.18	54.0	even	ok
	415	C78H160O29S	796.5378	0.5	76.5	421	31.66	0.0	even	ok
	416	C57H41N9NaO41PS	796.5372	-0.1	76.6	422	37.00	43.0	even	ok
	417	C77H33N5NaO32P	796.5366	-0.8	76.6	423	28.08	65.0	even	ok
	418	C77H41NNaO31PS2	796.5364	-1.1	76.8	424	24.62	59.0	even	ok
	419	C77H167NaO22P2S2	796.5376	0.4	76.8	425	33.22	-4.0	even	ok
	420	C51H40N13NaO42S2	796.5361	-1.5	76.9	426	20.39	39.0	even	ok
	421	C66H9N39NaO10PS	796.5370	-0.4	76.9	427	32.55	83.0	even	ok
	422	C75H152N10O23S	796.5371	-0.3	77.0	428	33.95	6.0	even	ok
	423	C76H163NaO27P2	796.5372	-0.2	77.0	429	35.49	-3.0	even	ok
	424	C67H136N34O7S2	796.5381	1.0	77.1	430	26.24	18.0	even	ok
	425	C77H157N4NaO25S	796.5372	-0.1	77.2	431	35.73	2.0	even	ok
	426	C76H34N7NaO29P2	796.5381	1.0	77.2	432	25.23	65.0	even	ok
	427	C58H166N6NaO35PS	796.5370	-0.5	77.2	433	31.83	-20.0	even	ok
	428	C75H28N11O30P	796.5365	-1.0	77.3	434	25.42	69.0	even	ok
	429	C73H150N16NaO16PS	796.5380	0.9	77.3	435	26.60	8.0	even	ok
	430	C53H49N5NaO45PS2	796.5383	1.2	77.3	436	23.52	33.0	even	ok
	431	C64H128N44OS2	796.5374	0.1	77.5	437	35.44	24.0	even	ok
	432	C65H10N41NaO7P2S	796.5385	1.5	77.5	438	20.66	83.0	even	ok
	433	C48H29N29NaO29PS2	796.5376	0.3	77.5	439	32.82	50.0	even	ok
	434	C69H141N28NaO9S2	796.5382	1.1	77.6	440	23.87	14.0	even	ok
	435	C71H145N22O14PS	796.5379	0.7	77.6	441	28.29	12.0	even	ok
	436	C64H4N45O8PS	796.5368	-0.6	77.6	442	29.73	87.0	even	ok
	437	C66H133N38NaO3S2	796.5375	0.3	77.7	443	33.08	20.0	even	ok
	438	C44H12N49O17PS2	796.5374	0.2	77.8	445	34.49	65.0	even	ok
	439	C56H154N20NaO25PS	796.5370	-0.5	77.9	446	30.99	-9.0	even	ok
	440	C61H137N34NaO12S	796.5381	1.0	78.0	447	20.56	11.0	even	ok
	441	C74H149N14NaO19S	796.5365	-1.0	78.0	448	25.17	8.0	even	ok
	442	C72H144N20O17S	796.5364	-1.1	78.0	449	23.30	12.0	even	ok
	443	C74H29N13O27P2	796.5380	0.9	78.1	450	26.26	69.0	even	ok
	444	C58H136N36O14S	796.5373	0.0	78.2	451	29.74	10.0	even	ok
	445	C64H41N3NaO43P	796.5369	-0.5	78.3	452	29.85	47.0	even	ok
	446	C53H24N29O29PS	796.5371	-0.3	78.4	453	32.40	58.0	even	ok
	447	C55H36N15O39PS	796.5371	-0.3	78.5	454	32.34	47.0	even	ok
	448	C57H4N47NaO12S	796.5377	0.5	78.5	455	24.38	80.0	even	ok
	449	C77H33N9O26P2S	796.5361	-1.5	78.6	456	18.88	68.0	even	ok
	450	C51H44N11O43PS2	796.5381	1.0	78.9	457	23.88	37.0	even	ok
	451	C79H38N3NaO28P2S	796.5362	-1.4	79.1	458	20.18	64.0	even	ok
	452	C70H142N26NaO10PS	796.5374	0.1	79.2	459	33.70	14.0	even	ok
	453	C56H161N12O33PS	796.5368	-0.6	79.2	460	27.56	-16.0	even	ok
	454	C66H9N43O4P2S2	796.5364	-1.1	79.2	461	22.40	86.0	even	ok
	455	C69H17N33O10P2S2	796.5371	-0.3	79.3	462	31.12	80.0	even	ok
	456	C46H24N35O27PS2	796.5374	0.2	79.3	463	32.45	54.0	even	ok

Compound Spectrum SmartFormula Report

Meas. m/z	#	Ion Formula	m/z	err [ppm]	mSigma	# mSigma	Score	rdb	e⁻ Conf	N-Rule
	457	C45H120N62S2	796.5363	-1.3	79.6	464	17.41	18.0	even	ok
	458	C59H132N40O10S	796.5380	0.8	79.6	465	20.73	15.0	even	ok
	459	C68H14N37NaO6P2S2	796.5366	-1.0	79.7	466	23.73	82.0	even	ok
	460	C68H137N32O8PS	796.5372	-0.1	79.7	467	32.46	18.0	even	ok
	461	C78H162O27P2	796.5384	1.3	79.7	468	19.94	0.0	even	ok
	462	C54H3N49O14S	796.5369	-0.5	79.7	469	23.81	79.0	even	ok
	463	C54H149N26O23PS	796.5368	-0.6	79.9	470	26.76	-5.0	even	ok
	464	C71H22N27NaO12P2S2	796.5372	-0.1	80.0	471	32.11	76.0	even	ok
	465	C72H25N23O16P2S2	796.5378	0.6	80.0	472	27.37	74.0	even	ok
	466	C58H146N28NaO17PS	796.5383	1.2	80.0	473	17.37	1.0	even	ok
	467	C57H48NO49PS	796.5371	-0.3	80.1	474	30.46	36.0	even	ok
	468	C60H10N43NaO9P2S	796.5365	-1.1	80.2	475	18.44	79.0	even	ok
	469	C64H143N30NaO9P2S	796.5368	-0.6	80.2	476	22.36	10.0	even	ok
	470	C62H36N9O41P	796.5368	-0.7	80.3	477	25.73	51.0	even	ok
	471	C43H9N53NaO13PS2	796.5369	-0.5	80.4	478	27.44	67.0	even	ok
	472	C50H41N15NaO39PS2	796.5376	0.3	80.5	479	29.04	39.0	even	ok
	473	C53H7N45O18S	796.5363	-1.3	80.7	480	16.19	74.0	even	ok
	474	C62H9N43O9P2S	796.5377	0.4	80.7	481	23.17	82.0	even	ok
	475	C75H23N17O25S	796.5380	0.9	80.8	482	23.20	74.0	even	ok
	476	C74H30N17NaO18P2S2	796.5379	0.7	80.9	484	24.62	70.0	even	ok
	477	C54H33N19NaO35PS	796.5366	-0.9	81.0	485	22.65	49.0	even	ok
	478	C72H15N27O19S	796.5374	0.1	81.2	486	31.09	80.0	even	ok
	479	C52H21N33NaO25PS	796.5366	-0.9	81.2	487	22.41	60.0	even	ok
	480	C77H28N11NaO27S	796.5382	1.1	81.2	488	21.12	70.0	even	ok
	481	C79H32N5O32P	796.5379	0.7	81.2	489	24.97	68.0	even	ok
	482	C57H167N8NaO32P2S	796.5385	1.4	81.2	490	18.03	-20.0	even	ok
	483	C78H158N2NaO26P	796.5364	-1.2	81.3	491	19.94	2.0	even	ok
	484	C55H9N45NaO13PS	796.5386	1.6	81.3	492	13.93	75.0	even	ok
	485	C75H33N13O22P2S2	796.5384	1.4	81.3	493	18.13	68.0	even	ok
	486	C74H20N21NaO21S	796.5375	0.2	81.4	494	29.05	76.0	even	ok
	487	C70H13N33NaO12PS	796.5383	1.3	81.5	495	19.25	82.0	even	ok
	488	C45H21N39NaO23PS2	796.5369	-0.5	81.5	496	26.25	56.0	even	ok
	489	C57H133N40NaO10S	796.5368	-0.7	81.5	497	20.63	12.0	even	ok
	490	C73H162N2O29P2	796.5364	-1.2	81.6	498	16.54	-4.0	even	ok
	491	C62H138N36O7P2S	796.5367	-0.8	81.7	499	19.69	14.0	even	ok
	492	C56H137N36NaO14S	796.5361	-1.5	81.7	500	14.21	7.0	even	ok
	493	C68H8N39O10PS	796.5382	1.1	81.7	501	20.64	86.0	even	ok
	494	C68H157N6NaO32	796.5365	-1.1	81.7	502	17.24	-6.0	even	ok
	495	C68H161N4O33P	796.5385	1.5	81.8	503	14.43	-8.0	even	ok
	496	C80H31N3O35	796.5364	-1.2	81.8	504	19.51	68.0	even	ok
	497	C58H5N49O7P2S	796.5363	-1.2	81.8	505	15.97	83.0	even	ok
	498	C66H4N45NaO5S2	796.5385	1.5	81.8	506	17.02	88.0	even	ok
	499	C55H158N16NaO29PS	796.5363	-1.3	81.9	507	18.64	-14.0	even	ok
	500	C76H153N8O24P	796.5362	-1.4	82.0	508	17.88	6.0	even	ok

Compound Spectrum SmartFormula Report

Cmpd 5, 4.9 min

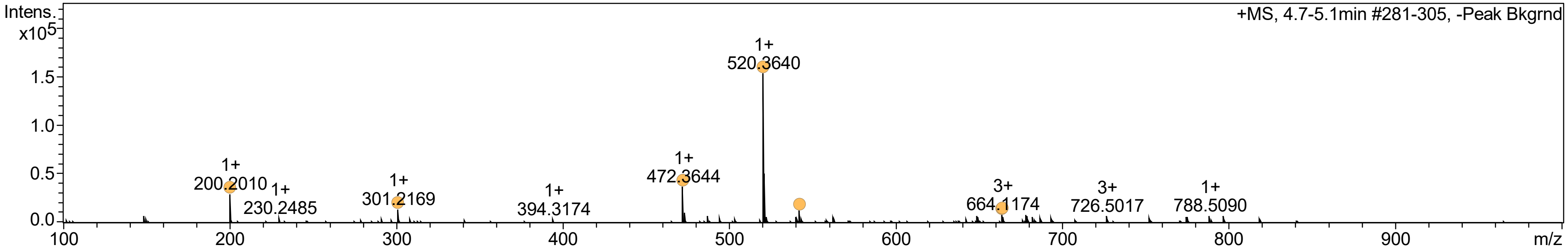

Meas. m/z	#	Ion Formula	m/z	err [ppm]	mSigma	# mSigma	Score	rdb	e⁻ Conf	N-Rule
200.2010	1	C12H26NO	200.2009	-0.6	20.0	1	100.00	1.0	even	ok
301.2169	1	C13H29N6S	301.2169	-0.1	14.4	1	100.00	3.0	even	ok
	2	C15H34NaO2S	301.2172	0.9	16.0	2	87.77	-1.0	even	ok
	3	C15H36NaP2	301.2184	5.1	18.5	3	30.94	-1.0	even	ok
	4	C20H29O2	301.2162	-2.4	18.9	4	49.86	7.0	even	ok
	5	C12H33N2O4S	301.2156	-4.5	24.0	5	40.85	-2.0	even	ok
	6	C12H35N2O2P2	301.2168	-0.3	30.0	6	51.79	-2.0	even	ok
472.3644	1	C23H52N3NaO3P	472.3638	-1.1	6.4	1	98.23	0.0	even	ok
	2	C25H51N3O3P	472.3663	3.9	7.1	2	43.24	3.0	even	ok
	3	C21H47N9OP	472.3636	-1.7	7.3	3	83.74	4.0	even	ok
	4	C25H47N5NaO2	472.3622	-4.6	10.4	4	31.64	5.0	even	ok
	5	C26H50NO6	472.3633	-2.4	10.6	5	88.44	3.0	even	ok
	6	C20H46N11S	472.3653	1.9	20.5	6	82.58	4.0	even	ok
	7	C20H51N5O5P	472.3622	-4.6	20.5	7	26.58	-1.0	even	ok
	8	C22H51N5NaO2S	472.3656	2.5	22.5	8	67.18	0.0	even	ok
	9	C21H57NNaO4P2	472.3655	2.4	22.7	9	52.19	-5.0	even	ok
	10	C18H47N11NaS	472.3629	-3.2	22.8	10	54.15	1.0	even	ok
	11	C27H46N5O2	472.3646	0.4	23.4	11	81.39	8.0	even	ok
	12	C19H52N7O2P2	472.3652	1.8	23.7	12	59.80	-1.0	even	ok
	13	C19H50N7O4S	472.3640	-0.9	26.4	13	91.35	-1.0	even	ok
	14	C23H54NO6S	472.3666	4.8	26.5	14	28.84	-2.0	even	ok
	15	C21H55NNaO6S	472.3642	-0.3	27.9	15	100.00	-5.0	even	ok
	16	C15H43N15NaO	472.3667	4.9	33.1	16	17.21	2.0	even	ok
	17	C18H54N3O8S	472.3626	-3.8	35.6	17	33.02	-6.0	even	ok
	18	C30H47N3Na	472.3662	3.9	37.6	18	22.65	9.0	even	ok
	19	C21H55N5PS2	472.3631	-2.7	42.9	19	37.61	-2.0	even	ok
	20	C27H54NOS2	472.3641	-0.5	49.4	20	67.14	2.0	even	ok
520.3640	1	C27H42N11	520.3619	-4.1	8.2	1	24.51	13.0	even	ok
	2	C29H51N3O3P	520.3663	4.2	8.2	2	30.74	7.0	even	ok
	3	C29H47N5NaO2	520.3622	-3.6	9.3	3	39.60	9.0	even	ok
	4	C27H52N3NaO3P	520.3638	-0.4	10.8	4	100.00	4.0	even	ok
	5	C25H47N9OP	520.3636	-0.9	12.1	5	64.52	8.0	even	ok

Compound Spectrum SmartFormula Report

Meas. m/z	#	Ion Formula	m/z	err [ppm]	mSigma	# mSigma	Score	rdb	e⁻ Conf	N-Rule
	6	C30H50NO6	520.3633	-1.5	12.4	6	73.32	7.0	even	ok
	7	C31H46N5O2	520.3646	1.1	19.2	7	71.85	12.0	even	ok
	8	C24H51N5O5P	520.3622	-3.5	22.2	8	31.37	3.0	even	ok
	9	C24H46N11S	520.3653	2.4	26.1	9	42.19	8.0	even	ok
	10	C25H57NNaO4P2	520.3655	2.8	27.3	10	26.84	-1.0	even	ok
	11	C23H52N7O2P2	520.3652	2.3	27.9	11	31.59	3.0	even	ok
	12	C26H51N5NaO2S	520.3656	2.9	28.2	12	33.63	4.0	even	ok
	13	C22H47N11NaS	520.3629	-2.2	28.7	13	41.55	5.0	even	ok
	14	C34H47N3Na	520.3662	4.2	30.9	14	19.56	13.0	even	ok
	15	C27H54NO6S	520.3666	5.0	32.2	15	13.33	2.0	even	ok
	16	C23H50N7O4S	520.3640	-0.2	32.3	16	65.22	3.0	even	ok
	17	C25H55NNaO6S	520.3642	0.3	33.8	17	60.64	-1.0	even	ok
	18	C20H52N9NaOPS	520.3645	0.9	37.9	18	47.52	0.0	even	ok
	19	C21H51N7NaO4S	520.3615	-4.8	38.0	19	12.35	0.0	even	ok
	20	C21H53N7NaO2P2	520.3628	-2.4	40.5	20	22.13	0.0	even	ok
	21	C21H55N5O5PS	520.3656	3.0	40.7	21	23.76	-2.0	even	ok
	22	C22H54N3O8S	520.3626	-2.8	41.2	22	25.42	-2.0	even	ok
	23	C19H48N13P2	520.3625	-2.9	41.4	23	18.04	4.0	even	ok
	24	C22H56N3O6P2	520.3639	-0.3	41.4	24	37.47	-2.0	even	ok
	25	C25H55N5PS2	520.3631	-1.8	43.5	25	40.02	2.0	even	ok
	26	C19H50N7O9	520.3665	4.6	44.6	26	11.19	-1.0	even	ok
	27	C24H59NO4PS2	520.3618	-4.4	47.1	27	14.36	-3.0	even	ok
	28	C29H55NNaOS2	520.3617	-4.5	48.4	28	13.43	3.0	even	ok
	29	C17H51N11O3PS	520.3629	-2.2	48.5	29	24.88	-1.0	even	ok
	30	C19H56N5NaO5PS	520.3632	-1.6	48.9	30	28.86	-5.0	even	ok
	31	C23H60N3OP2S2	520.3648	1.4	50.1	31	29.98	-3.0	even	ok
	32	C18H47N11NaO5	520.3654	2.6	50.4	32	15.53	1.0	even	ok
	33	C16H42N17O3	520.3651	2.0	51.2	33	17.96	5.0	even	ok
	34	C20H59NO9PS	520.3643	0.4	51.5	34	36.31	-7.0	even	ok
	35	C19H51N11NaS2	520.3663	4.2	51.5	35	10.72	0.0	even	ok
	36	C21H61N3NaOP2S2	520.3623	-3.3	53.5	36	14.74	-6.0	even	ok
	37	C31H54NOS2	520.3641	0.2	53.7	37	44.80	6.0	even	ok
	38	C20H57N3NaO6P2	520.3615	-4.9	53.9	38	5.50	-5.0	even	ok
	39	C18H54N3O13	520.3651	2.0	56.4	39	20.21	-6.0	even	ok
	40	C17H51N7NaO9	520.3640	-0.0	63.9	40	19.79	-4.0	even	ok
542.3465	1	C32H48NO6	542.3476	2.0	12.0	1	62.98	10.0	even	ok
	2	C30H49N5PS	542.3441	-4.5	12.8	2	22.73	10.0	even	ok
	3	C31H45N5NaO2	542.3465	0.0	15.0	3	100.00	12.0	even	ok
	4	C28H54N3OP2S	542.3457	-1.5	15.9	4	67.25	5.0	even	ok
	5	C29H40N11	542.3463	-0.5	16.8	5	85.72	16.0	even	ok
	6	C30H49NNaO6	542.3452	-2.5	16.8	6	48.56	7.0	even	ok
	7	C33H44N5O2	542.3490	4.4	18.2	7	21.47	15.0	even	ok
	8	C28H44N7O4	542.3449	-3.0	18.7	8	38.80	11.0	even	ok
	9	C29H50N3NaO3P	542.3482	3.0	20.9	9	36.16	7.0	even	ok
	10	C24H45N11NaS	542.3472	1.3	21.8	10	63.47	8.0	even	ok
	11	C25H48N7O4S	542.3483	3.2	23.3	11	31.94	6.0	even	ok

Compound Spectrum SmartFormula Report

Meas. m/z	#	Ion Formula	m/z	err [ppm]	mSigma	# mSigma	Score	rdb	e¯ Conf	N-Rule
	12	C27H53NNaO6S	542.3486	3.7	23.5	12	25.85	2.0	even	ok
	13	C27H41N11Na	542.3439	-5.0	24.6	13	10.97	13.0	even	ok
	14	C27H45N9OP	542.3479	2.5	26.2	14	29.00	11.0	even	ok
	15	C27H53N5PS2	542.3475	1.7	28.2	15	60.97	5.0	even	ok
	16	C25H54N5NaPS2	542.3450	-2.8	30.0	16	40.69	2.0	even	ok
	17	C26H49N5O5P	542.3466	0.1	30.1	17	70.57	6.0	even	ok
	18	C28H52N3O3S2	542.3445	-3.8	31.1	18	25.98	5.0	even	ok
	19	C25H58N3OP2S2	542.3491	4.7	31.8	19	17.22	0.0	even	ok
	20	C31H53NNaOS2	542.3461	-0.9	32.7	20	68.50	6.0	even	ok
	21	C23H49N7NaO4S	542.3459	-1.2	32.8	21	49.84	3.0	even	ok
	22	C26H57NO4PS2	542.3461	-0.8	33.2	22	68.74	0.0	even	ok
	23	C21H44N13O2S	542.3456	-1.7	33.3	23	42.46	7.0	even	ok
	24	C24H52N3O8S	542.3470	0.8	34.2	24	53.97	1.0	even	ok
	25	C36H48NOS	542.3451	-2.6	34.9	25	30.06	14.0	even	ok
	26	C22H50N9NaOPS	542.3489	4.3	35.0	26	15.33	3.0	even	ok
	27	C25H46N9NaOP	542.3455	-1.9	36.7	27	27.52	8.0	even	ok
	28	C33H52NOS2	542.3485	3.6	38.7	28	23.91	9.0	even	ok
	29	C25H53NO9P	542.3452	-2.4	39.4	29	29.04	1.0	even	ok
	30	C24H50N5NaO5P	542.3442	-4.4	40.5	30	12.93	3.0	even	ok
	31	C23H59N3NaOP2S2	542.3467	0.3	41.6	31	49.86	-3.0	even	ok
	32	C24H54N3O6P2	542.3482	3.1	43.1	32	20.25	1.0	even	ok
	33	C20H53N7NaO4S2	542.3493	5.0	44.2	33	10.73	-2.0	even	ok
	34	C22H53N3NaO8S	542.3446	-3.7	44.2	34	15.78	-2.0	even	ok
	35	C20H48N9O6S	542.3443	-4.2	44.6	35	12.50	2.0	even	ok
	36	C21H54N5NaO5PS	542.3475	1.8	46.4	36	28.55	-2.0	even	ok
	37	C19H49N11O3PS	542.3473	1.3	47.0	37	32.65	2.0	even	ok
	38	C22H57NO9PS	542.3486	3.8	47.4	38	13.52	-4.0	even	ok
	39	C22H45N11O3P	542.3439	-4.9	48.5	39	6.04	7.0	even	ok
	40	C18H48N13O2S2	542.3490	4.5	48.8	40	9.57	2.0	even	ok
	41	C23H51N7NaO2P2	542.3472	1.1	51.2	41	22.71	3.0	even	ok
	42	C20H58N5O3P2S2	542.3451	-2.7	51.6	42	18.35	-4.0	even	ok
	43	C21H46N13P2	542.3469	0.6	53.2	43	24.39	7.0	even	ok
	44	C19H57N3NaO8S2	542.3479	2.5	53.9	44	22.52	-7.0	even	ok
	45	C17H50N11NaO3PS	542.3449	-3.1	57.8	45	12.98	-1.0	even	ok
	46	C20H58NNaO9PS	542.3462	-0.6	57.8	46	28.06	-7.0	even	ok
	47	C18H53N7O7PS	542.3459	-1.1	58.4	47	24.10	-3.0	even	ok
	48	C15H45N17OPS	542.3446	-3.6	58.6	48	10.32	3.0	even	ok
	49	C19H49N7NaO9	542.3484	3.4	61.2	49	10.25	-1.0	even	ok
	50	C22H55N3NaO6P2	542.3458	-1.3	62.1	50	15.17	-2.0	even	ok
	51	C20H50N9O4P2	542.3456	-1.8	63.8	51	12.27	2.0	even	ok
	52	C19H47N13NaP2	542.3445	-3.8	65.2	52	5.71	4.0	even	ok
	53	C18H53N3NaO13	542.3471	0.9	71.7	53	15.85	-6.0	even	ok
	54	C17H44N13O7	542.3481	2.9	72.2	54	6.32	3.0	even	ok
	55	C16H48N9O11	542.3468	0.4	72.8	55	17.24	-2.0	even	ok
	56	C16H41N17NaO3	542.3470	0.9	73.2	56	11.27	5.0	even	ok
	57	C14H36N23O	542.3468	0.4	74.9	57	11.99	9.0	even	ok

Compound Spectrum SmartFormula Report

Meas. m/z	#	Ion Formula	m/z	err [ppm]	mSigma	# mSigma	Score	rdb	e⁻ Conf	N-Rule
	58	C19H54N5O8P2	542.3442	-4.3	75.2	58	3.17	-3.0	even	ok
	59	C15H45N13NaO7	542.3457	-1.5	84.6	59	6.01	0.0	even	ok
663.7827	1	C93H192N11O31S	663.7830	0.4	15.3	1	86.53	5.0	even	ok
	2	C89H59N24O31S	663.7827	0.0	15.4	2	96.18	74.0	even	ok
	3	C92H189N15NaO27S	663.7826	-0.1	15.5	3	93.14	7.0	even	ok
	4	C78H35N58O9S2	663.7830	0.4	15.5	4	87.52	92.0	even	ok
	5	C91H64N18NaO33S	663.7828	0.2	15.5	5	92.40	70.0	even	ok
	6	C82H168N45O9S2	663.7832	0.7	15.6	6	77.69	23.0	even	ok
	7	C80H40N52NaO11S2	663.7831	0.5	15.6	7	83.75	88.0	even	ok
	8	C81H165N49NaO5S2	663.7829	0.2	15.7	8	90.92	25.0	even	ok
	9	C95H197N5NaO33S	663.7831	0.5	16.0	9	81.91	1.0	even	ok
	10	C90H55N28O27S	663.7832	0.7	16.0	10	78.16	79.0	even	ok
	11	C90H184N21O25S	663.7826	-0.3	16.1	11	88.39	11.0	even	ok
	12	C88H56N28NaO27S	663.7824	-0.5	16.1	12	82.23	76.0	even	ok
	13	C77H32N62NaO5S2	663.7826	-0.2	16.2	13	90.43	94.0	even	ok
	14	C79H160N55O3S2	663.7828	0.1	16.4	14	93.33	29.0	even	ok
	15	C80H169N45NaO9S2	663.7824	-0.5	16.7	15	82.50	20.0	even	ok
	16	C81H172N41O13S2	663.7828	0.1	16.8	16	92.38	18.0	even	ok
	17	C92H196N7O35S	663.7826	-0.3	16.9	17	87.10	0.0	even	ok
	18	C89H52N32NaO23S	663.7828	0.2	16.9	18	89.94	81.0	even	ok
	19	C92H67N14O37S	663.7832	0.7	17.0	19	76.56	68.0	even	ok
	20	C95H73N10O34P2S	663.7823	-0.6	17.0	20	78.97	67.0	even	ok
	21	C83H177N35NaO15S2	663.7829	0.2	17.2	21	88.20	14.0	even	ok
	22	C86H51N34O25S	663.7823	-0.7	17.2	22	76.98	80.0	even	ok
	23	C94H201NNaO37S	663.7826	-0.1	17.3	23	89.93	-4.0	even	ok
	24	C75H27N68O3S2	663.7825	-0.3	17.3	24	85.00	98.0	even	ok
	25	C84H49N44O12P2S2	663.7826	-0.3	17.3	25	86.54	85.0	even	ok
	26	C85H174N41O6P2S2	663.7824	-0.6	17.3	26	79.43	22.0	even	ok
	27	C78H28N66NaOS2	663.7831	0.5	17.3	27	81.00	99.0	even	ok
	28	C98H71N4O43	663.7825	-0.3	17.4	28	84.90	67.0	even	ok
	29	C78H164N51O7S2	663.7823	-0.6	17.5	29	77.73	24.0	even	ok
	30	C98H203NNaO30P2S	663.7822	-0.7	17.6	30	74.41	0.0	even	ok
	31	C87H47N38O21S	663.7827	0.0	17.8	31	91.83	85.0	even	ok
	32	C87H186N27O16P2S2	663.7824	-0.5	18.0	32	78.48	11.0	even	ok
	33	C87H179N35NaO8P2S2	663.7825	-0.4	18.1	33	81.59	18.0	even	ok
	34	C93H185N19NaO23S	663.7831	0.5	18.1	34	78.53	12.0	even	ok
	35	C97H78N4NaO36P2S	663.7824	-0.5	18.4	35	80.05	63.0	even	ok
	36	C91H180N25O21S	663.7830	0.4	18.4	36	81.33	16.0	even	ok
	37	C79H44N48NaO15S2	663.7826	-0.2	18.5	37	86.32	83.0	even	ok
	38	C77H39N54O13S2	663.7825	-0.3	18.7	38	82.77	87.0	even	ok
	39	C86H54N38NaO14P2S2	663.7827	-0.1	18.7	39	87.43	81.0	even	ok
	40	C93H61N24O24P2S	663.7823	-0.6	18.8	40	75.94	78.0	even	ok
	41	C90H68N14NaO37S	663.7824	-0.5	18.8	41	77.93	65.0	even	ok
	42	C90H194N13NaO28PS	663.7832	0.7	18.9	42	73.47	2.0	even	ok
	43	C88H63N20O35S	663.7823	-0.7	18.9	43	74.44	69.0	even	ok
	44	C84H180N31O19S2	663.7832	0.7	19.2	44	72.00	12.0	even	ok

Compound Spectrum SmartFormula Report

Meas. m/z	#	Ion Formula	m/z	err [ppm]	mSigma	# mSigma	Score	rdb	e⁻ Conf	N-Rule
	45	C95H66N18NaO26P2S	663.7824	-0.5	19.2	45	78.51	74.0	even	ok
	46	C82H37N58O2P2S2	663.7826	-0.3	19.4	46	82.80	96.0	even	ok
	47	C89H191N21NaO18P2S2	663.7825	-0.4	19.6	47	79.18	7.0	even	ok
	48	C88H182N31O12P2S2	663.7828	0.1	19.6	48	85.67	16.0	even	ok
	49	C84H42N52NaO4P2S2	663.7826	-0.1	19.8	49	85.29	92.0	even	ok
	50	C80H47N44O19S2	663.7830	0.4	20.0	50	79.56	81.0	even	ok
	51	C96H69N14O30P2S	663.7828	0.1	20.1	51	85.90	72.0	even	ok
	52	C93H76N4NaO43S	663.7828	0.2	20.1	52	97.86	59.0	even	ok
	53	C98H81O40P2S	663.7828	0.1	20.1	53	100.00	61.0	even	ok
	54	C96H76N2O44P	663.7831	0.5	20.3	54	75.64	62.0	even	ok
	55	C86H44N42NaO17S	663.7824	-0.5	20.3	55	75.29	87.0	even	ok
	56	C101H72N2NaO41	663.7831	0.5	20.3	56	76.05	68.0	even	ok
	57	C91H71N10O41S	663.7827	0.0	20.3	57	86.81	63.0	even	ok
	58	C87H57N34O18P2S2	663.7830	0.4	20.3	58	90.59	79.0	even	ok
	59	C88H189N19O26PS	663.7831	0.6	20.4	59	74.40	6.0	even	ok
	60	C90H177N29NaO17S	663.7826	-0.1	20.4	60	83.92	18.0	even	ok
	61	C86H61N30O22P2S2	663.7826	-0.3	20.5	61	94.62	74.0	even	ok
	62	C85H45N48O8P2S2	663.7830	0.4	20.7	62	77.24	90.0	even	ok
	63	C99H67N8O39	663.7830	0.4	20.8	63	78.39	72.0	even	ok
	64	C82H52N38NaO21S2	663.7831	0.5	20.9	64	74.84	77.0	even	ok
	65	C88H182N27NaO18PS	663.7832	0.7	21.2	65	70.15	13.0	even	ok
	66	C99H196NO37	663.7823	-0.6	21.2	66	71.76	4.0	even	ok
	67	C90H187N25NaO14P2S2	663.7829	0.3	21.2	67	79.55	12.0	even	ok
	68	C97H194N11O24P2S	663.7826	-0.2	21.3	68	80.56	9.0	even	ok
	69	C88H43N42O17S	663.7832	0.7	21.3	69	70.06	90.0	even	ok
	70	C98H74N8NaO32P2S	663.7829	0.2	21.5	70	80.24	68.0	even	ok
	71	C90H194N17O22P2S2	663.7828	0.1	21.6	71	95.61	5.0	even	ok
	72	C88H172N35O15S	663.7825	-0.3	21.6	72	78.57	22.0	even	ok
	73	C96H191N15NaO20P2S	663.7822	-0.8	21.6	73	68.12	11.0	even	ok
	74	C80H176N37O17S2	663.7823	-0.6	21.7	74	71.26	13.0	even	ok
	75	C75H37N60NaO6PS2	663.7832	0.6	21.8	75	70.26	89.0	even	ok
	76	C82H181N31NaO19S2	663.7824	-0.5	21.8	76	74.17	9.0	even	ok
	77	C84H39N48O15S	663.7823	-0.7	21.9	77	69.61	91.0	even	ok
	78	C86H170N45O2P2S2	663.7828	0.1	22.0	78	81.57	27.0	even	ok
	79	C87H50N42NaO10P2S2	663.7831	0.6	22.0	79	71.92	86.0	even	ok
	80	C86H61N26NaO28PS	663.7829	0.3	22.1	80	77.12	71.0	even	ok
	81	C99H199N5NaO26P2S	663.7827	-0.1	22.1	81	82.34	5.0	even	ok
	82	C88H66N24NaO24P2S2	663.7827	-0.1	22.2	82	94.74	70.0	even	ok
	83	C89H62N28NaO20P2S2	663.7831	0.6	22.2	83	83.38	75.0	even	ok
	84	C76H162N57NaPS2	663.7830	0.4	22.7	84	75.10	26.0	even	ok
	85	C88H175N39NaO4P2S2	663.7829	0.3	22.7	85	77.14	23.0	even	ok
	86	C94H79O47S	663.7832	0.7	22.8	86	78.76	57.0	even	ok
	87	C87H186N23NaO22PS	663.7827	0.0	22.9	87	82.12	8.0	even	ok
	88	C96H69N10NaO36P	663.7832	0.6	22.9	88	68.52	69.0	even	ok
	89	C98H64N12NaO35	663.7826	-0.2	23.1	89	78.15	74.0	even	ok
	90	C86H177N33O16PS	663.7831	0.6	23.1	90	70.14	17.0	even	ok

Compound Spectrum SmartFormula Report

Meas. m/z	#	Ion Formula	m/z	err [ppm]	mSigma	# mSigma	Score	rdb	e⁻ Conf	N-Rule
	91	C89H198N13O26P2S2	663.7824	-0.5	23.2	91	81.96	0.0	even	ok
	92	C100H202NO30P2S	663.7830	0.5	23.3	92	71.84	3.0	even	ok
	93	C85H189N21NaO25S2	663.7829	0.2	23.3	93	90.14	3.0	even	ok
	94	C84H49N40NaO18PS	663.7829	0.3	23.3	94	75.20	82.0	even	ok
	95	C95H73N6NaO40P	663.7827	-0.0	23.3	95	81.12	64.0	even	ok
	96	C73H32N66O4PS2	663.7831	0.5	23.4	96	70.76	93.0	even	ok
	97	C90H201N5O36PS	663.7831	0.6	23.5	97	69.33	-5.0	even	ok
	98	C87H40N46NaO13S	663.7828	0.1	23.5	98	78.15	92.0	even	ok
	99	C92H199N11NaO24P2S2	663.7829	0.3	23.6	99	88.05	1.0	even	ok
	100	C83H184N27O23S2	663.7828	0.1	23.7	100	79.57	7.0	even	ok
	101	C78H174N43NaO10PS2	663.7830	0.4	23.7	101	73.39	15.0	even	ok
	102	C84H56N32O26PS	663.7828	0.2	23.7	102	77.40	75.0	even	ok
	103	C94H57N28O20P2S	663.7828	0.1	24.1	103	78.79	83.0	even	ok
	104	C89H198N9NaO32PS	663.7827	0.0	24.1	104	79.75	-3.0	even	ok
	105	C96H59N18O33	663.7825	-0.3	24.3	105	72.99	78.0	even	ok
	106	C96H62N22NaO22P2S	663.7829	0.2	24.6	106	74.94	79.0	even	ok
	107	C85H35N52O11S	663.7827	0.0	24.8	107	78.95	96.0	even	ok
	108	C91H173N33NaO13S	663.7831	0.5	24.8	108	67.97	23.0	even	ok
	109	C93H54N32NaO16P2S	663.7824	-0.5	24.8	109	69.32	85.0	even	ok
	110	C90H75N6O45S	663.7823	-0.6	24.8	110	76.42	58.0	even	ok
	111	C85H181N29O20PS	663.7827	-0.1	24.9	111	76.33	12.0	even	ok
	112	C91H203N7NaO28P2S2	663.7825	-0.4	24.9	112	82.23	-4.0	even	ok
	113	C94H64N16O34P	663.7831	0.5	24.9	113	68.35	73.0	even	ok
	114	C76H169N49O8PS2	663.7829	0.2	25.0	114	74.12	19.0	even	ok
	115	C100H192N5O33	663.7828	0.1	25.1	115	77.27	9.0	even	ok
	116	C91H49N38O14P2S	663.7823	-0.6	25.1	116	65.99	89.0	even	ok
	117	C97H65N18O26P2S	663.7832	0.7	25.2	117	62.94	77.0	even	ok
	118	C85H38N56NaP2S2	663.7831	0.5	25.3	118	66.96	97.0	even	ok
	119	C92H80NaO47S	663.7824	-0.5	25.4	119	78.82	54.0	even	ok
	120	C82H44N46O16PS	663.7828	0.2	25.4	120	74.50	86.0	even	ok
	121	C87H193N15O30PS	663.7827	-0.1	25.4	121	75.51	1.0	even	ok
	122	C93H68N12O38P	663.7826	-0.2	25.5	122	74.33	68.0	even	ok
	123	C89H168N39O11S	663.7830	0.4	25.6	123	69.51	27.0	even	ok
	124	C89H69N20O28P2S2	663.7830	0.4	25.7	124	80.26	68.0	even	ok
	125	C79H51N40O23S2	663.7825	-0.3	25.9	125	70.64	76.0	even	ok
	126	C72H29N70NaPS2	663.7827	-0.0	25.9	126	76.37	95.0	even	ok
	127	C77H49N46NaO16PS2	663.7832	0.7	26.0	127	63.80	78.0	even	ok
	128	C100H76N2O39PS	663.7822	-0.7	26.1	128	71.96	66.0	even	ok
	129	C86H192N17O29S2	663.7832	0.7	26.1	129	71.96	1.0	even	ok
	130	C83H53N36NaO22PS	663.7825	-0.4	26.2	130	69.15	77.0	even	ok
	131	C81H56N34NaO25S2	663.7826	-0.2	26.5	131	72.52	72.0	even	ok
	132	C89H52N36O17PS2	663.7825	-0.4	26.5	132	79.02	84.0	even	ok
	133	C94H77N2NaO44P	663.7823	-0.7	26.5	133	62.06	59.0	even	ok
	134	C88H73N12NaO38PS	663.7829	0.3	26.5	134	69.64	60.0	even	ok
	135	C92H189N19O21PS2	663.7823	-0.7	26.6	135	72.28	10.0	even	ok
	136	C96H198N3NaO34P	663.7825	-0.3	26.7	136	69.22	1.0	even	ok

Compound Spectrum SmartFormula Report

Meas. m/z	#	Ion Formula	m/z	err [ppm]	mSigma	# mSigma	Score	rdb	e¯ Conf	N-Rule
	137	C75H44N52O14PS2	663.7831	0.5	26.8	137	65.41	82.0	even	ok
	138	C85H174N37NaO12PS	663.7827	0.0	26.8	138	75.23	19.0	even	ok
	139	C75H166N53NaO4PS2	663.7825	-0.3	26.9	139	68.94	21.0	even	ok
	140	C98H190N15O20P2S	663.7830	0.5	27.1	140	65.91	14.0	even	ok
	141	C90H177N33O11PS2	663.7823	-0.7	27.2	141	60.94	21.0	even	ok
	142	C92H182N27NaO13PS2	663.7824	-0.6	27.2	142	74.23	17.0	even	ok
	143	C86H68N18O36PS	663.7828	0.2	27.3	143	71.15	64.0	even	ok
	144	C86H190N19NaO26PS	663.7823	-0.7	27.3	144	61.81	3.0	even	ok
	145	C82H59N30O29S2	663.7830	0.4	27.3	145	78.63	70.0	even	ok
	146	C95H182N25O14P2S	663.7826	-0.2	27.4	146	70.17	20.0	even	ok
	147	C97H187N19NaO16P2S	663.7827	-0.1	27.4	147	72.88	16.0	even	ok
	148	C97H194N7NaO30P	663.7830	0.4	27.4	148	67.46	6.0	even	ok
	149	C74H41N56NaO10PS2	663.7827	-0.0	27.6	149	73.60	84.0	even	ok
	150	C86H170N41NaO8PS	663.7832	0.7	27.7	150	60.63	24.0	even	ok
	151	C91H74N14NaO30P2S2	663.7831	0.6	27.8	151	73.32	64.0	even	ok
	152	C99H60N16NaO31	663.7831	0.5	27.8	152	64.32	79.0	even	ok
	153	C99H189N9NaO29	663.7824	-0.5	27.8	153	64.38	11.0	even	ok
	154	C100H195N9NaO22P2S	663.7831	0.6	27.9	154	62.06	10.0	even	ok
	155	C84H32N56NaO7S	663.7824	-0.5	27.9	155	63.31	98.0	even	ok
	156	C100H69N10NaO31PS	663.7823	-0.6	27.9	156	71.98	73.0	even	ok
	157	C85H65N22NaO32PS	663.7825	-0.4	28.1	157	66.27	66.0	even	ok
	158	C84H178N33NaO16PS	663.7823	-0.7	28.2	158	60.40	14.0	even	ok
	159	C81H48N42O20PS	663.7824	-0.5	28.3	159	63.14	81.0	even	ok
	160	C88H165N43NaO7S	663.7826	-0.1	28.3	160	70.06	29.0	even	ok
	161	C88H73N16O32P2S2	663.7826	-0.2	28.3	161	79.36	63.0	even	ok
	162	C91H57N30NaO19PS2	663.7826	-0.3	28.5	162	78.49	80.0	even	ok
	163	C84H64N24NaO31S2	663.7831	0.5	28.5	163	73.40	66.0	even	ok
	164	C82H188N23O27S2	663.7823	-0.6	28.5	164	71.20	2.0	even	ok
	165	C92H206N3O32P2S2	663.7828	0.1	28.5	165	81.48	-6.0	even	ok
	166	C97H55N22O29	663.7830	0.3	28.7	166	65.60	83.0	even	ok
	167	C94H194N13NaO23PS2	663.7824	-0.6	28.7	167	71.77	6.0	even	ok
	168	C93H61N20NaO30P	663.7827	-0.0	28.7	168	71.49	75.0	even	ok
	169	C87H40N50O7PS2	663.7825	-0.4	28.8	169	64.03	95.0	even	ok
	170	C73H161N59O2PS2	663.7824	-0.5	28.8	170	63.26	25.0	even	ok
	171	C82H37N54NaO8PS	663.7829	0.3	28.9	171	66.16	93.0	even	ok
	172	C72H36N62O8PS2	663.7826	-0.2	29.0	172	68.44	88.0	even	ok
	173	C94H193N9O32P	663.7824	-0.5	29.0	173	62.91	5.0	even	ok
	174	C83H169N43O10PS	663.7826	-0.1	29.1	174	69.17	23.0	even	ok
	175	C81H41N50NaO12PS	663.7825	-0.4	29.1	175	64.49	88.0	even	ok
	176	C84H193N17NaO29S2	663.7824	-0.5	29.1	176	73.20	-2.0	even	ok
	177	C92H65N16NaO34P	663.7823	-0.7	29.2	177	58.19	70.0	even	ok
	178	C86H31N56O7S	663.7832	0.7	29.2	178	58.56	101.0	even	ok
	179	C97H184N15O27	663.7823	-0.6	29.3	179	59.60	15.0	even	ok
	180	C95H189N13O28P	663.7829	0.2	29.5	180	66.81	10.0	even	ok
	181	C83H60N28O30PS	663.7824	-0.5	29.5	181	61.41	70.0	even	ok
	182	C91H64N22O27PS2	663.7825	-0.4	29.5	182	73.63	73.0	even	ok

Compound Spectrum SmartFormula Report

Meas. m/z	#	Ion Formula	m/z	err [ppm]	mSigma	# mSigma	Score	rdb	e⁻ Conf	N-Rule
	183	C82H27N62O5S	663.7823	-0.7	29.6	183	58.21	102.0	even	ok
	184	C86H160N49O5S	663.7825	-0.3	29.7	184	65.14	33.0	even	ok
	185	C84H165N47O6PS	663.7831	0.5	29.7	185	60.29	28.0	even	ok
	186	C93H185N23O17PS2	663.7827	-0.0	29.8	186	81.45	15.0	even	ok
	187	C80H186N29NaO20PS2	663.7830	0.4	29.9	187	63.43	4.0	even	ok
	188	C88H165N47OPS2	663.7823	-0.7	29.9	188	56.98	32.0	even	ok
	189	C101H72N6O35PS	663.7827	-0.1	30.1	189	79.85	71.0	even	ok
	190	C105H74N2NaO34P2	663.7826	-0.1	30.1	190	67.41	72.0	even	ok
	191	C90H78N10NaO34P2S2	663.7827	-0.1	30.1	191	79.01	59.0	even	ok
	192	C95H53N32O16P2S	663.7832	0.7	30.2	192	56.09	88.0	even	ok
	193	C101H188N9O29	663.7832	0.7	30.2	193	56.27	14.0	even	ok
	194	C103H69N8O32P2	663.7826	-0.3	30.2	194	64.65	76.0	even	ok
	195	C94H57N24NaO26P	663.7832	0.6	30.4	195	57.70	80.0	even	ok
	196	C89H45N44NaO9PS2	663.7825	-0.3	30.4	196	64.21	91.0	even	ok
	197	C78H181N35O18PS2	663.7829	0.2	30.4	197	65.18	8.0	even	ok
	198	C90H48N40O13PS2	663.7829	0.3	30.5	198	75.06	89.0	even	ok
	199	C77H178N39NaO14PS2	663.7825	-0.3	30.6	199	63.18	10.0	even	ok
	200	C94H50N36NaO12P2S	663.7829	0.2	30.8	200	64.89	90.0	even	ok
	201	C92H45N42O10P2S	663.7828	0.1	30.9	201	67.29	94.0	even	ok
	202	C71H33N66NaO4PS2	663.7823	-0.7	31.0	202	55.71	90.0	even	ok
	203	C91H56N26O28P	663.7826	-0.2	31.0	203	65.12	79.0	even	ok
	204	C89H205NO40PS	663.7827	-0.1	31.0	204	66.26	-10.0	even	ok
	205	C90H170N41NaO3PS2	663.7824	-0.6	31.1	205	57.89	28.0	even	ok
	206	C94H201N5O31PS2	663.7823	-0.7	31.1	206	64.96	-1.0	even	ok
	207	C80H32N60O6PS	663.7828	0.2	31.1	207	65.18	97.0	even	ok
	208	C88H202N5NaO36PS	663.7823	-0.6	31.2	208	56.35	-8.0	even	ok
	209	C96H52N26NaO25	663.7826	-0.2	31.4	209	64.11	85.0	even	ok
	210	C79H36N56O10PS	663.7824	-0.5	31.5	210	58.36	92.0	even	ok
	211	C92H60N26O23PS2	663.7829	0.3	31.6	211	72.93	78.0	even	ok
	212	C85H196N13O33S2	663.7828	0.1	31.7	212	76.72	-4.0	even	ok
	213	C95H190N17NaO19PS2	663.7828	0.1	31.8	213	75.79	11.0	even	ok
	214	C75H173N45O12PS2	663.7824	-0.5	31.8	214	58.90	14.0	even	ok
	215	C93H69N16NaO29PS2	663.7826	-0.3	31.8	215	72.48	69.0	even	ok
	216	C85H28N60NaO3S	663.7828	0.1	31.9	216	64.22	103.0	even	ok
	217	C103H77NaO37PS	663.7828	0.1	31.9	217	76.51	67.0	even	ok
	218	C102H197N3O29PS	663.7825	-0.4	31.9	218	60.23	8.0	even	ok
	219	C95H197N9O27PS2	663.7827	-0.0	32.2	219	76.83	4.0	even	ok
	220	C92H52N30O24P	663.7831	0.5	32.4	220	57.32	84.0	even	ok
	221	C92H53N34NaO15PS2	663.7830	0.4	32.4	221	68.86	85.0	even	ok
	222	C91H42N46NaO6P2S	663.7824	-0.5	32.4	222	57.69	96.0	even	ok
	223	C100H185N13NaO25	663.7829	0.2	32.5	223	62.38	16.0	even	ok
	224	C91H173N37O7PS2	663.7827	-0.0	32.5	224	65.09	26.0	even	ok
	225	C94H47N32O23	663.7825	-0.3	32.8	225	59.50	89.0	even	ok
	226	C87H201N7NaO35S2	663.7829	0.2	32.8	226	71.74	-8.0	even	ok
	227	C94H203NNaO35P2	663.7831	0.5	32.9	227	56.39	-4.0	even	ok
	228	C98H57N24NaO21PS	663.7823	-0.6	32.9	228	54.51	84.0	even	ok

Compound Spectrum SmartFormula Report

Meas. m/z	#	Ion Formula	m/z	err [ppm]	mSigma	# mSigma	Score	rdb	e⁻ Conf	N-Rule
	229	C91H73N10O39P2	663.7832	0.7	33.0	229	53.65	63.0	even	ok
	230	C82H166N47NaO6PS	663.7823	-0.7	33.0	230	53.59	25.0	even	ok
	231	C89H161N47NaO3S	663.7831	0.5	33.0	231	55.85	34.0	even	ok
	232	C89H37N52O4P2S	663.7823	-0.6	33.1	232	54.35	100.0	even	ok
	233	C83H23N66OS	663.7827	0.0	33.3	233	64.47	107.0	even	ok
	234	C83H186N27O21P2S	663.7832	0.7	33.4	234	52.30	7.0	even	ok
	235	C96H178N29O10P2S	663.7830	0.5	33.4	235	56.59	25.0	even	ok
	236	C94H186N17NaO24P	663.7825	-0.3	33.5	236	58.67	12.0	even	ok
	237	C98H183N23NaO12P2S	663.7831	0.6	33.6	237	54.02	21.0	even	ok
	238	C98H180N19O23	663.7828	0.1	33.6	238	63.05	20.0	even	ok
	239	C83H162N51NaO2PS	663.7827	0.0	33.7	239	63.72	30.0	even	ok
	240	C76H53N42NaO20PS2	663.7827	-0.0	33.9	240	63.19	73.0	even	ok
	241	C81H63N26O33S2	663.7825	-0.3	33.9	241	68.06	65.0	even	ok
	242	C94H65N20NaO25PS2	663.7830	0.4	33.9	242	66.21	74.0	even	ok
	243	C91H81N6O38P2S2	663.7830	0.4	33.9	243	65.64	57.0	even	ok
	244	C99H60N20O25PS	663.7827	-0.1	34.0	244	61.98	82.0	even	ok
	245	C94H181N27O13PS2	663.7832	0.6	34.1	245	61.26	20.0	even	ok
	246	C87H156N53OS	663.7830	0.4	34.1	246	56.68	38.0	even	ok
	247	C93H178N31NaO9PS2	663.7828	0.1	34.2	247	61.24	22.0	even	ok
	248	C104H194N5O26P2	663.7824	-0.6	34.4	248	53.45	13.0	even	ok
	249	C79H61N32NaO26PS2	663.7832	0.7	34.4	249	51.88	67.0	even	ok
	250	C79H68N24O34PS2	663.7831	0.5	34.5	250	62.95	60.0	even	ok
	251	C77H56N38O24PS2	663.7831	0.5	34.5	251	54.02	71.0	even	ok
	252	C87H77N8NaO42PS	663.7825	-0.4	34.5	252	56.62	55.0	even	ok
	253	C97H202N3NaO29PS2	663.7828	0.1	34.5	253	70.63	0.0	even	ok
	254	C74H48N48O18PS2	663.7826	-0.2	34.6	254	59.71	77.0	even	ok
	255	C95H175N33NaO6P2S	663.7827	-0.1	34.6	255	60.96	27.0	even	ok
	256	C88H36N54O3PS2	663.7829	0.3	34.6	256	58.15	100.0	even	ok
	257	C83H68N20NaO35S2	663.7826	-0.2	34.7	257	69.27	61.0	even	ok
	258	C81H73N18NaO36PS2	663.7832	0.7	34.9	258	59.72	56.0	even	ok
	259	C102H68N10O31PS	663.7831	0.6	34.9	259	60.94	76.0	even	ok
	260	C96H193N13O23PS2	663.7832	0.6	34.9	260	59.93	9.0	even	ok
	261	C93H170N39O4P2S	663.7826	-0.2	35.0	261	58.04	31.0	even	ok
	262	C73H45N52NaO14PS2	663.7823	-0.7	35.0	262	50.39	79.0	even	ok
	263	C90H53N30NaO24P	663.7823	-0.7	35.1	263	50.13	81.0	even	ok
	264	C81H174N41O11P2S	663.7832	0.7	35.1	264	50.18	18.0	even	ok
	265	C88H80N4O46PS	663.7829	0.2	35.2	265	58.50	53.0	even	ok
	266	C79H29N64NaO2PS	663.7825	-0.4	35.2	266	55.35	99.0	even	ok
	267	C85H72N14O40PS	663.7824	-0.5	35.2	267	53.32	59.0	even	ok
	268	C92H198N7O33P2	663.7830	0.4	35.3	268	55.24	0.0	even	ok
	269	C101H65N14NaO27PS	663.7828	0.1	35.4	269	60.10	78.0	even	ok
	270	C100H185N17O19PS	663.7825	-0.4	35.5	270	54.94	19.0	even	ok
	271	C81H202N11NaO34PS2	663.7825	-0.3	35.5	271	65.42	-12.0	even	ok
	272	C81H58N34NaO23P2S	663.7830	0.5	35.5	272	53.40	72.0	even	ok
	273	C95H182N21NaO20P	663.7830	0.3	35.5	273	55.28	17.0	even	ok
	274	C93H76N8O37PS2	663.7825	-0.4	35.6	274	63.41	62.0	even	ok

Compound Spectrum SmartFormula Report

Meas. m/z	#	Ion Formula	m/z	err [ppm]	mSigma	# mSigma	Score	rdb	e⁻ Conf	N-Rule
	275	C94H72N12O33PS2	663.7829	0.3	35.7	275	65.73	67.0	even	ok
	276	C92H181N23O22P	663.7824	-0.5	35.7	276	53.12	16.0	even	ok
	277	C79H190N25NaO24PS2	663.7825	-0.3	35.7	277	55.60	-1.0	even	ok
	278	C85H198N13O31P2S	663.7832	0.7	35.8	278	49.14	-4.0	even	ok
	279	C80H193N21O28PS2	663.7829	0.2	35.8	279	56.75	-3.0	even	ok
	280	C81H157N57PS	663.7826	-0.1	35.9	280	58.20	34.0	even	ok
	281	C82H205N7O38PS2	663.7829	0.2	35.9	281	65.90	-14.0	even	ok
	282	C93H86NaO40P2S2	663.7831	0.6	36.0	282	59.70	53.0	even	ok
	283	C92H82NaO45P2	663.7828	0.1	36.0	283	58.02	54.0	even	ok
	284	C90H41N48NaO5PS2	663.7830	0.4	36.0	284	53.82	96.0	even	ok
	285	C89H61N24O29P2	663.7832	0.7	36.1	285	49.65	74.0	even	ok
	286	C82H198N15NaO30PS2	663.7830	0.4	36.1	286	54.15	-7.0	even	ok
	287	C77H185N31O22PS2	663.7824	-0.4	36.1	287	52.86	3.0	even	ok
	288	C79H46N48NaO13P2S	663.7830	0.5	36.1	288	52.68	83.0	even	ok
	289	C84H210NNaO40PS2	663.7830	0.4	36.2	289	62.90	-18.0	even	ok
	290	C97H48N30NaO21	663.7831	0.5	36.2	290	52.19	90.0	even	ok
	291	C104H65N12O28P2	663.7830	0.4	36.3	291	53.32	81.0	even	ok
	292	C91H49N34NaO20P	663.7827	-0.0	36.3	292	59.08	86.0	even	ok
	293	C106H70N6NaO30P2	663.7831	0.5	36.3	293	51.09	77.0	even	ok
	294	C84H200N9O37S2	663.7823	-0.6	36.4	294	58.57	-9.0	even	ok
	295	C90H70N14NaO35P2	663.7828	0.1	36.4	295	57.53	65.0	even	ok
	296	C82H183N31NaO17P2S	663.7828	0.2	36.4	296	56.75	9.0	even	ok
	297	C78H65N28NaO30PS2	663.7827	-0.0	36.5	297	59.13	62.0	even	ok
	298	C102H190N11NaO21PS	663.7826	-0.2	36.5	298	55.63	15.0	even	ok
	299	C97H177N23NaO19	663.7824	-0.5	36.6	299	51.70	22.0	even	ok
	300	C104H73N4NaO33PS	663.7832	0.7	36.6	300	55.78	72.0	even	ok
	301	C105H198NNaO27PS	663.7830	0.4	36.6	301	60.90	9.0	even	ok
	302	C76H60N34O28PS2	663.7826	-0.2	36.7	302	56.57	66.0	even	ok
	303	C93H41N46O6P2S	663.7832	0.7	36.9	303	47.34	99.0	even	ok
	304	C87H84O50PS	663.7824	-0.5	37.0	304	51.07	48.0	even	ok
	305	C73H171N51NaO5P2S2	663.7831	0.5	37.0	305	50.68	16.0	even	ok
	306	C75H57N38NaO24PS2	663.7823	-0.7	37.1	306	47.73	68.0	even	ok
	307	C95H43N36O19	663.7830	0.3	37.3	307	52.85	94.0	even	ok
	308	C92H191N15NaO25P2	663.7831	0.5	37.4	308	50.22	7.0	even	ok
	309	C80H77N14NaO40PS2	663.7827	-0.0	37.5	309	67.31	51.0	even	ok
	310	C84H195N17NaO27P2S	663.7829	0.2	37.5	310	55.03	-2.0	even	ok
	311	C77H24N70PS	663.7824	-0.5	37.5	311	49.88	103.0	even	ok
	312	C93H177N27O18P	663.7829	0.2	37.5	312	54.57	21.0	even	ok
	313	C84H71N16O39S2	663.7830	0.4	37.6	313	60.70	59.0	even	ok
	314	C103H62N16NaO24P2	663.7826	-0.1	37.6	314	55.60	83.0	even	ok
	315	C79H53N40O21P2S	663.7829	0.3	37.6	315	52.67	76.0	even	ok
	316	C86H205N3NaO39S2	663.7824	-0.5	37.6	316	59.12	-13.0	even	ok
	317	C78H72N20O38PS2	663.7826	-0.2	37.6	317	64.50	55.0	even	ok
	318	C108H72O37P	663.7825	-0.4	37.6	318	51.18	75.0	even	ok
	319	C103H193N7O25PS	663.7829	0.3	37.8	319	52.76	13.0	even	ok
	320	C83H75N12O43S2	663.7825	-0.3	37.8	320	61.46	54.0	even	ok

Compound Spectrum SmartFormula Report

Meas. m/z	#	Ion Formula	m/z	err [ppm]	mSigma	# mSigma	Score	rdb	e¯ Conf	N-Rule
	321	C95H81N2NaO39PS2	663.7826	-0.3	37.9	321	62.06	58.0	even	ok
	322	C95H172N29O17	663.7823	-0.6	38.0	322	47.63	26.0	even	ok
	323	C96H77N6NaO35PS2	663.7830	0.4	38.1	323	59.23	63.0	even	ok
	324	C90H77N6O43P2	663.7827	-0.0	38.1	324	56.75	58.0	even	ok
	325	C101H57N22O22P2	663.7826	-0.3	38.1	325	52.79	87.0	even	ok
	326	C90H85N2O42P2S2	663.7826	-0.2	38.1	326	62.06	52.0	even	ok
	327	C81H80N10O44PS2	663.7831	0.5	38.2	327	57.02	49.0	even	ok
	328	C96H45N38NaO11PS	663.7823	-0.6	38.2	328	47.40	95.0	even	ok
	329	C92H38N50NaO2P2S	663.7829	0.2	38.2	329	53.66	101.0	even	ok
	330	C97H48N34O15PS	663.7827	-0.1	38.3	330	55.27	93.0	even	ok
	331	C79H197N17O32PS2	663.7824	-0.4	38.4	331	49.87	-8.0	even	ok
	332	C92H169N41O3PS2	663.7832	0.6	38.4	332	46.94	31.0	even	ok
	333	C89H44N40O18P	663.7826	-0.2	38.5	333	53.65	90.0	even	ok
	334	C83H85N4NaO46PS2	663.7832	0.7	38.5	334	54.13	45.0	even	ok
	335	C77H41N54O11P2S	663.7829	0.3	38.6	335	51.45	87.0	even	ok
	336	C99H176N23O19	663.7832	0.7	38.6	336	45.43	25.0	even	ok
	337	C90H33N56P2S	663.7828	0.1	38.7	337	55.09	105.0	even	ok
	338	C83H70N20NaO33P2S	663.7830	0.5	38.7	338	48.99	61.0	even	ok
	339	C80H178N37O15P2S	663.7828	0.0	38.8	339	55.41	13.0	even	ok
	340	C88H65N20O33P2	663.7827	-0.0	38.8	340	55.55	69.0	even	ok
	341	C92H45N38NaO16P	663.7832	0.6	38.8	341	46.47	91.0	even	ok
	342	C86H76N10NaO41S2	663.7831	0.5	38.9	342	56.29	55.0	even	ok
	343	C71H166N57O3P2S2	663.7830	0.4	38.9	343	50.22	20.0	even	ok
	344	C80H171N45NaO7P2S	663.7828	0.2	38.9	344	53.15	20.0	even	ok
	345	C92H43N42O12S2	663.7824	-0.6	39.0	345	55.05	94.0	even	ok
	346	C75H183N37NaO15P2S2	663.7831	0.5	39.1	346	47.80	5.0	even	ok
	347	C91H202N3O37P2	663.7825	-0.3	39.1	347	50.94	-5.0	even	ok
	348	C91H195N11NaO29P2	663.7826	-0.2	39.1	348	52.92	2.0	even	ok
	349	C85H80N6NaO45S2	663.7826	-0.2	39.1	349	61.74	50.0	even	ok
	350	C99H53N28NaO17PS	663.7828	0.1	39.2	350	54.45	89.0	even	ok
	351	C94H55N28O22S2	663.7824	-0.6	39.3	351	54.71	83.0	even	ok
	352	C86H207N3NaO37P2S	663.7829	0.2	39.4	352	52.26	-13.0	even	ok
	353	C82H190N23O25P2S	663.7828	0.0	39.4	353	54.35	2.0	even	ok
	354	C85H82N6NaO43P2S	663.7830	0.5	39.6	354	47.76	50.0	even	ok
	355	C70H41N60O9P2S2	663.7832	0.7	39.8	355	44.84	83.0	even	ok
	356	C79H162N55OP2S	663.7832	0.7	39.9	356	44.26	29.0	even	ok
	357	C97H185N19NaO18S2	663.7823	-0.7	39.9	357	51.29	16.0	even	ok
	358	C90H186N21O23P2	663.7830	0.4	39.9	358	48.93	11.0	even	ok
	359	C107H195N3NaO24P2	663.7829	0.3	40.0	359	50.45	14.0	even	ok
	360	C77H34N62NaO3P2S	663.7830	0.5	40.0	360	47.55	94.0	even	ok
	361	C81H65N26O31P2S	663.7830	0.3	40.1	361	49.24	65.0	even	ok
	362	C100H56N24O21PS	663.7831	0.6	40.1	362	45.53	87.0	even	ok
	363	C88H58N28NaO25P2	663.7828	0.1	40.1	363	52.15	76.0	even	ok
	364	C81H209N3O42PS2	663.7824	-0.4	40.2	364	55.47	-19.0	even	ok
	365	C105H190N9O22P2	663.7828	0.1	40.2	365	52.10	18.0	even	ok
	366	C94H40N40NaO15	663.7826	-0.2	40.2	366	50.89	96.0	even	ok

Compound Spectrum SmartFormula Report

Meas. m/z	#	Ion Formula	m/z	err [ppm]	mSigma	# mSigma	Score	rdb	e⁻ Conf	N-Rule
	367	C77H69N24NaO34PS2	663.7823	-0.7	40.4	367	43.83	57.0	even	ok
	368	C73H178N43O13P2S2	663.7830	0.4	40.5	368	48.02	9.0	even	ok
	369	C72H53N46O19P2S2	663.7832	0.7	40.5	369	43.96	72.0	even	ok
	370	C95H173N33NaO8S2	663.7823	-0.7	40.5	370	50.35	27.0	even	ok
	371	C78H50N44NaO17P2S	663.7826	-0.2	40.5	371	50.26	78.0	even	ok
	372	C96H171N37NaO2P2S	663.7831	0.6	40.6	372	44.94	32.0	even	ok
	373	C90H40N44O14P	663.7831	0.5	40.8	373	46.02	95.0	even	ok
	374	C84H202N9O35P2S	663.7828	0.0	40.8	374	52.25	-9.0	even	ok
	375	C94H48N36NaO14S2	663.7824	-0.4	40.8	375	54.62	90.0	even	ok
	376	C94H166N43P2S	663.7830	0.4	40.8	376	46.59	36.0	even	ok
	377	C83H77N12O41P2S	663.7830	0.3	40.9	377	48.05	54.0	even	ok
	378	C98H173N31O9PS	663.7825	-0.4	40.9	378	47.44	30.0	even	ok
	379	C89H74N10NaO39P2	663.7824	-0.5	41.0	379	45.06	60.0	even	ok
	380	C77H195N23NaO25P2S2	663.7831	0.5	41.1	380	45.22	-6.0	even	ok
	381	C98H173N27NaO15	663.7829	0.2	41.2	381	49.70	27.0	even	ok
	382	C102H61N18NaO23PS	663.7832	0.7	41.4	382	42.02	83.0	even	ok
	383	C86H83N2O49S2	663.7830	0.4	41.4	383	54.61	48.0	even	ok
	384	C96H60N22NaO24S2	663.7824	-0.4	41.4	384	53.75	79.0	even	ok
	385	C100H178N25NaO11PS	663.7826	-0.2	41.5	385	48.63	26.0	even	ok
	386	C99H197N5NaO28S2	663.7823	-0.7	41.5	386	49.21	5.0	even	ok
	387	C78H166N51O5P2S	663.7828	0.0	41.5	387	51.51	24.0	even	ok
	388	C92H174N31NaO14P	663.7825	-0.3	41.5	388	47.34	23.0	even	ok
	389	C69H38N64NaO5P2S2	663.7828	0.1	41.6	389	49.96	85.0	even	ok
	390	C89H190N17O27P2	663.7825	-0.3	41.7	390	47.44	6.0	even	ok
	391	C92H35N46O13	663.7825	-0.3	41.7	391	47.00	100.0	even	ok
	392	C87H49N38O19P2	663.7832	0.7	41.8	392	42.64	85.0	even	ok
	393	C101H181N21O15PS	663.7829	0.3	41.8	393	47.36	24.0	even	ok
	394	C96H67N14O32S2	663.7824	-0.6	41.8	394	51.05	72.0	even	ok
	395	C81H187N27NaO21P2S	663.7824	-0.5	41.9	395	44.57	4.0	even	ok
	396	C104H187N13NaO18P2	663.7825	-0.4	41.9	396	45.50	20.0	even	ok
	397	C79H175N41NaO11P2S	663.7824	-0.5	42.2	397	44.11	15.0	even	ok
	398	C80H62N30NaO27P2S	663.7826	-0.2	42.2	398	48.08	67.0	even	ok
	399	C71H50N50NaO15P2S2	663.7828	0.1	42.2	399	49.07	74.0	even	ok
	400	C76H38N58NaO7P2S	663.7826	-0.2	42.3	400	47.78	89.0	even	ok
	401	C75H190N29O23P2S2	663.7830	0.4	42.4	401	45.49	-2.0	even	ok
	402	C96H168N33O13	663.7828	0.0	42.4	402	49.93	31.0	even	ok
	403	C102H306N14NaO15	663.7831	0.6	42.5	403	42.82	-42.0	even	ok
	404	C72H175N47NaO9P2S2	663.7826	-0.2	42.5	404	48.25	11.0	even	ok
	405	C87H62N24NaO29P2	663.7824	-0.6	42.5	405	43.04	71.0	even	ok
	406	C75H29N68OP2S	663.7829	0.3	42.6	406	46.15	98.0	even	ok
	407	C88H41N44NaO14P	663.7823	-0.7	42.6	407	40.87	92.0	even	ok
	408	C102H182N19O16P2	663.7824	-0.6	42.6	408	42.80	24.0	even	ok
	409	C82H74N16NaO37P2S	663.7826	-0.2	42.6	409	47.55	56.0	even	ok
	410	C96H176N29O12S2	663.7826	-0.2	42.6	410	55.67	25.0	even	ok
	411	C79H81N10NaO44PS2	663.7823	-0.7	42.6	411	48.08	46.0	even	ok
	412	C86H53N34O23P2	663.7827	-0.0	42.7	412	49.91	80.0	even	ok

Compound Spectrum SmartFormula Report

Meas. m/z	#	Ion Formula	m/z	err [ppm]	mSigma	# mSigma	Score	rdb	e¯ Conf	N-Rule
	413	C103H186N15NaO17PS	663.7830	0.4	42.8	413	44.21	20.0	even	ok
	414	C98H188N15O22S2	663.7826	-0.2	42.8	414	55.51	14.0	even	ok
	415	C82H89NaO50PS2	663.7827	-0.0	42.8	415	58.23	40.0	even	ok
	416	C76H45N50O15P2S	663.7825	-0.4	42.8	416	45.27	82.0	even	ok
	417	C83H199N13NaO31P2S	663.7824	-0.5	42.9	417	43.41	-7.0	even	ok
	418	C80H84N6O48PS2	663.7826	-0.1	43.0	418	55.82	44.0	even	ok
	419	C87H69N16O37P2	663.7823	-0.7	43.3	419	40.45	64.0	even	ok
	420	C95H51N32O18S2	663.7828	0.1	43.4	420	55.75	88.0	even	ok
	421	C106H60N14O27P	663.7824	-0.4	43.5	421	43.54	86.0	even	ok
	422	C67H33N70O3P2S2	663.7827	-0.0	43.5	422	48.82	89.0	even	ok
	423	C104H63N12O30S	663.7826	-0.2	43.5	423	53.61	81.0	even	ok
	424	C104H58N20NaO20P2	663.7831	0.5	43.5	424	42.00	88.0	even	ok
	425	C90H169N37O12P	663.7824	-0.5	43.7	425	42.74	27.0	even	ok
	426	C74H65N32O29P2S2	663.7832	0.7	43.7	426	40.13	61.0	even	ok
	427	C105H188N9O24S	663.7824	-0.5	43.8	427	48.83	18.0	even	ok
	428	C90H31N56O2S2	663.7823	-0.6	43.8	428	41.18	105.0	even	ok
	429	C102H53N26O18P2	663.7830	0.4	43.8	429	43.42	92.0	even	ok
	430	C69H45N56O13P2S2	663.7827	-0.0	43.8	430	48.41	78.0	even	ok
	431	C108H65N8NaO29P	663.7825	-0.3	43.9	431	44.80	82.0	even	ok
	432	C78H57N36O25P2S	663.7825	-0.3	43.9	432	43.96	71.0	even	ok
	433	C74H187N33NaO19P2S2	663.7826	-0.2	44.0	433	46.39	0.0	even	ok
	434	C90H179N29NaO15P2	663.7831	0.5	44.0	434	41.96	18.0	even	ok
	435	C108H320NaO18P2	663.7827	-0.0	44.0	435	47.84	-49.0	even	ok
	436	C109H68N4O33P	663.7829	0.3	44.0	436	45.12	80.0	even	ok
	437	C93H39N46O8S2	663.7828	0.1	44.0	437	54.85	99.0	even	ok
	438	C79H182N33O19P2S	663.7823	-0.6	44.0	438	40.16	8.0	even	ok
	439	C89H183N25NaO19P2	663.7826	-0.2	44.1	439	45.98	13.0	even	ok
	440	C95H36N48O5PS	663.7827	-0.1	44.1	440	46.98	104.0	even	ok
	441	C102H306N18O9PS	663.7827	0.0	44.1	441	48.07	-39.0	even	ok
	442	C93H170N35NaO10P	663.7830	0.3	44.2	442	43.72	28.0	even	ok
	443	C89H81N2O47P2	663.7823	-0.7	44.2	443	39.42	53.0	even	ok
	444	C98H72N8NaO34S2	663.7825	-0.4	44.3	444	49.73	68.0	even	ok
	445	C70H170N53O7P2S2	663.7825	-0.3	44.3	445	44.13	15.0	even	ok
	446	C101H303N22NaO5PS	663.7824	-0.5	44.3	446	41.24	-37.0	even	ok
	447	C80H69N22O35P2S	663.7825	-0.3	44.3	447	43.56	60.0	even	ok
	448	C106H315N6O16P2	663.7826	-0.2	44.4	448	45.44	-45.0	even	ok
	449	C98H181N23NaO14S2	663.7827	-0.0	44.5	449	54.89	21.0	even	ok
	450	C97H41N42NaO7PS	663.7828	0.0	44.5	450	46.95	100.0	even	ok
	451	C89H37N48NaO10P	663.7827	-0.0	44.6	451	46.89	97.0	even	ok
	452	C98H44N38O11PS	663.7831	0.6	44.6	452	40.13	98.0	even	ok
	453	C81H194N19O29P2S	663.7823	-0.6	44.6	453	39.52	-3.0	even	ok
	454	C77H170N47O9P2S	663.7823	-0.6	44.7	454	39.38	19.0	even	ok
	455	C94H33N52NaOPS	663.7823	-0.6	44.7	455	39.50	106.0	even	ok
	456	C97H63N18O28S2	663.7828	0.1	44.7	456	53.59	77.0	even	ok
	457	C104H311N12NaO11PS	663.7828	0.1	44.8	457	45.36	-43.0	even	ok
	458	C74H33N64O5P2S	663.7825	-0.4	44.8	458	42.70	93.0	even	ok

Compound Spectrum SmartFormula Report

Meas. m/z	#	Ion Formula	m/z	err [ppm]	mSigma	# mSigma	Score	rdb	e⁻ Conf	N-Rule
	459	C95H36N44NaO11	663.7830	0.5	44.9	459	41.19	101.0	even	ok
	460	C73H62N36NaO25P2S2	663.7828	0.1	44.9	460	45.34	63.0	even	ok
	461	C100H193N9NaO24S2	663.7827	-0.0	45.0	461	54.21	10.0	even	ok
	462	C100H200NO32S2	663.7826	-0.2	45.0	462	52.19	3.0	even	ok
	463	C85H57N30O27P2	663.7823	-0.7	45.1	463	38.32	75.0	even	ok
	464	C106H68N6NaO32S	663.7827	-0.1	45.2	464	53.06	77.0	even	ok
	465	C86H68N14NaO42	663.7832	0.7	45.2	465	37.53	61.0	even	ok
	466	C107H193N3NaO26S	663.7825	-0.4	45.3	466	48.80	14.0	even	ok
	467	C84H56N28NaO32	663.7832	0.7	45.3	467	37.55	72.0	even	ok
	468	C92H36N50NaO4S2	663.7824	-0.4	45.3	468	41.12	101.0	even	ok
	469	C105H314N8O15PS	663.7832	0.7	45.4	469	38.12	-45.0	even	ok
	470	C95H165N37NaO9	663.7824	-0.5	45.4	470	40.42	33.0	even	ok
	471	C79H207N9NaO35P2S2	663.7831	0.5	45.4	471	39.92	-17.0	even	ok
	472	C97H56N26NaO20S2	663.7829	0.2	45.5	472	50.55	84.0	even	ok
	473	C76H77N18O39P2S2	663.7832	0.7	45.5	473	44.37	50.0	even	ok
	474	C77H163N55NaOP2S	663.7824	-0.5	45.5	474	40.07	26.0	even	ok
	475	C100H49N32NaO13PS	663.7832	0.7	45.5	475	37.47	94.0	even	ok
	476	C84H86N2NaO47P2S	663.7826	-0.2	45.5	476	43.83	45.0	even	ok
	477	C88H80NaO52	663.7832	0.7	45.6	477	37.09	50.0	even	ok
	478	C101H50N30NaO14P2	663.7826	-0.1	45.6	478	44.45	94.0	even	ok
	479	C72H182N39O17P2S2	663.7825	-0.3	45.6	479	42.49	4.0	even	ok
	480	C95H44N40NaO10S2	663.7829	0.2	45.7	480	50.23	95.0	even	ok
	481	C96H169N37NaO4S2	663.7827	-0.1	45.8	481	52.75	32.0	even	ok
	482	C87H193N11NaO36	663.7830	0.5	46.0	482	40.22	-2.0	even	ok
	483	C86H46N42NaO15P2	663.7828	0.1	46.0	483	44.19	87.0	even	ok
	484	C93H31N50O9	663.7830	0.3	46.1	484	41.48	105.0	even	ok
	485	C91H165N41O8P	663.7829	0.2	46.1	485	43.03	32.0	even	ok
	486	C68H42N60NaO9P2S2	663.7824	-0.5	46.1	486	38.93	80.0	even	ok
	487	C83H206N5O39P2S	663.7823	-0.6	46.2	487	37.85	-14.0	even	ok
	488	C99H45N36O12P2	663.7825	-0.3	46.3	488	41.88	98.0	even	ok
	489	C105H312N10NaO12P2	663.7823	-0.7	46.4	489	36.58	-43.0	even	ok
	490	C88H174N35O13P2	663.7830	0.4	46.4	490	40.74	22.0	even	ok
	491	C71H57N42O23P2S2	663.7827	-0.0	46.5	491	44.95	67.0	even	ok
	492	C110H193NO27P	663.7827	-0.0	46.5	492	44.39	17.0	even	ok
	493	C77H202N15O33P2S2	663.7830	0.4	46.6	493	40.24	-13.0	even	ok
	494	C98H79O42S2	663.7824	-0.6	46.6	494	44.63	61.0	even	ok
	495	C87H178N31O17P2	663.7825	-0.3	46.7	495	41.02	17.0	even	ok
	496	C87H32N54O8P	663.7826	-0.2	46.7	496	42.46	101.0	even	ok
	497	C107H185N11O21P	663.7823	-0.7	46.7	497	36.23	23.0	even	ok
	498	C85H50N38NaO19P2	663.7824	-0.6	46.8	498	37.99	82.0	even	ok
	499	C93H160N43O7	663.7823	-0.6	46.9	499	37.07	37.0	even	ok
	500	C109H190N5NaO23P	663.7823	-0.6	46.9	500	37.66	19.0	even	ok
664.1174	1	C43H67N39O52P	664.1173	-0.1	18.1	1	98.54	32.0	even	ok
	2	C45H72N33NaO54P	664.1174	0.0	18.7	2	100.00	28.0	even	ok
	3	C34H48N67NaO32PS	664.1176	0.4	18.9	3	90.81	46.0	even	ok
	4	C42H64N43NaO48P	664.1169	-0.7	19.4	4	68.43	34.0	even	ok

Compound Spectrum SmartFormula Report

Meas. m/z	#	Ion Formula	m/z	err [ppm]	mSigma	# mSigma	Score	rdb	e⁻ Conf	N-Rule
	5	C31H40N77NaO26PS	664.1171	-0.3	19.5	5	75.41	52.0	even	ok
	6	C32H43N73O30PS	664.1175	0.2	19.7	6	77.42	50.0	even	ok
	7	C29H35N83O24PS	664.1171	-0.5	19.7	7	72.09	56.0	even	ok
	8	C20H16N111NaO4PS2	664.1174	0.0	19.7	8	81.76	70.0	even	ok
	9	C31H47N69O34PS	664.1171	-0.5	19.8	9	86.51	45.0	even	ok
	10	C18H11N117O2PS2	664.1173	-0.1	19.8	10	79.07	74.0	even	ok
	11	C33H52N63NaO36PS	664.1171	-0.3	19.8	11	90.00	41.0	even	ok
	12	C21H19N107O8PS2	664.1177	0.5	19.9	12	69.87	68.0	even	ok
	13	C46H75N29O58P	664.1177	0.6	20.0	13	83.48	26.0	even	ok
	14	C44H76N29NaO58P	664.1169	-0.6	20.1	14	80.91	23.0	even	ok
	15	C23H24N101NaO10PS2	664.1178	0.7	20.6	15	65.92	64.0	even	ok
	16	C23H31N93O18PS2	664.1177	0.6	20.8	16	82.08	57.0	even	ok
	17	C19H20N107NaO8PS2	664.1169	-0.7	20.8	17	66.21	65.0	even	ok
	18	C39H60N51O43P2	664.1178	0.7	20.9	18	65.08	38.0	even	ok
	19	C20H23N103O12PS2	664.1173	-0.1	21.0	19	77.17	63.0	even	ok
	20	C34H55N59O40PS	664.1175	0.2	21.0	20	90.07	39.0	even	ok
	21	C22H28N97NaO14PS2	664.1174	0.0	21.1	21	79.19	59.0	even	ok
	22	C48H80N23NaO60P	664.1178	0.7	21.1	22	77.97	22.0	even	ok
	23	C45H79N25O62P	664.1173	-0.1	21.3	23	92.25	21.0	even	ok
	24	C41H72N37O53P2	664.1178	0.7	21.3	24	77.33	27.0	even	ok
	25	C44H63N43O48P	664.1177	0.6	21.4	25	81.08	37.0	even	ok
	26	C25H36N87NaO20PS2	664.1178	0.7	21.6	26	77.31	53.0	even	ok
	27	C36H60N53NaO42PS	664.1176	0.4	21.7	27	85.23	35.0	even	ok
	28	C47H84N19NaO64P	664.1174	0.0	21.9	28	93.11	17.0	even	ok
	29	C46H68N37NaO50P	664.1178	0.7	22.3	29	76.07	33.0	even	ok
	30	C27H33N89NaO17P2S	664.1177	0.5	22.6	30	66.79	58.0	even	ok
	31	C36H50N61O39S	664.1170	-0.6	22.7	31	77.50	44.0	even	ok
	32	C41H55N53O42P	664.1173	-0.1	22.7	32	74.37	43.0	even	ok
	33	C38H57N55NaO39P2	664.1175	0.2	22.7	33	73.37	40.0	even	ok
	34	C43H60N47NaO44P	664.1174	0.0	23.0	34	76.05	39.0	even	ok
	35	C25H26N95O17S2	664.1172	-0.3	23.0	35	84.92	62.0	even	ok
	36	C29H45N75NaO27P2S	664.1177	0.5	23.1	36	65.90	47.0	even	ok
	37	C30H31N87O20PS	664.1175	0.2	23.2	37	71.73	61.0	even	ok
	38	C32H36N81NaO22PS	664.1176	0.4	23.6	38	68.38	57.0	even	ok
	39	C40H69N41NaO49P2	664.1175	0.2	23.6	39	71.78	29.0	even	ok
	40	C40H52N57NaO38P	664.1169	-0.7	23.7	40	62.20	45.0	even	ok
	41	C16H16N115O3P2S2	664.1178	0.7	23.8	41	61.18	69.0	even	ok
	42	C48H87N15O68P	664.1177	0.6	23.9	42	76.40	15.0	even	ok
	43	C25H28N95O15P2S	664.1176	0.4	24.0	43	67.51	62.0	even	ok
	44	C36H52N61O37P2	664.1174	0.0	24.2	44	73.83	44.0	even	ok
	45	C29H28N91NaO16PS	664.1171	-0.3	24.2	45	67.99	63.0	even	ok
	46	C38H55N55NaO41S	664.1170	-0.5	24.2	46	78.15	40.0	even	ok
	47	C21H12N115NaPS2	664.1178	0.7	24.2	47	60.93	75.0	even	ok
	48	C27H40N81O25P2S	664.1176	0.4	24.3	48	66.96	51.0	even	ok
	49	C34H38N75O29S	664.1170	-0.6	24.5	49	74.35	55.0	even	ok
	50	C27H23N97O14PS	664.1170	-0.5	24.5	50	64.75	67.0	even	ok

Compound Spectrum SmartFormula Report

Meas. m/z	#	Ion Formula	m/z	err [ppm]	mSigma	# mSigma	Score	rdb	e¯ Conf	N-Rule
	51	C27H31N89NaO19S2	664.1173	-0.1	24.6	51	85.27	58.0	even	ok
	52	C21H32N93NaO18PS2	664.1169	-0.7	24.6	52	73.23	54.0	even	ok
	53	C38H64N47O47P2	664.1174	0.0	24.8	53	72.63	33.0	even	ok
	54	C37H48N65O33P2	664.1178	0.7	25.0	54	59.58	49.0	even	ok
	55	C50H92N9NaO70P	664.1178	0.7	25.1	55	71.32	11.0	even	ok
	56	C22H35N89O22PS2	664.1173	-0.1	25.6	56	83.71	52.0	even	ok
	57	C27H38N81O27S2	664.1172	-0.3	25.8	57	79.93	51.0	even	ok
	58	C36H43N69NaO31S	664.1170	-0.5	25.8	58	75.32	51.0	even	ok
	59	C38H62N47O49S	664.1170	-0.6	25.8	59	72.40	33.0	even	ok
	60	C26H37N85NaO21P2S	664.1172	-0.2	25.8	60	68.50	53.0	even	ok
	61	C24H40N83NaO24PS2	664.1174	0.0	25.9	61	85.36	48.0	even	ok
	62	C33H59N55O44PS	664.1171	-0.5	26.1	62	75.19	34.0	even	ok
	63	C37H61N51NaO43P2	664.1170	-0.5	26.3	63	61.47	35.0	even	ok
	64	C50H82N17O67	664.1172	-0.3	26.3	64	79.00	20.0	even	ok
	65	C35H64N49NaO46PS	664.1172	-0.3	26.3	65	77.96	30.0	even	ok
	66	C25H19N103NaO9S2	664.1173	-0.1	26.4	66	81.65	69.0	even	ok
	67	C24H25N99NaO11P2S	664.1172	-0.2	26.5	67	67.26	64.0	even	ok
	68	C39H58N51O45S	664.1174	0.1	26.6	68	82.87	38.0	even	ok
	69	C31H57N61NaO37P2S	664.1177	0.5	26.7	69	72.88	36.0	even	ok
	70	C35H49N65NaO33P2	664.1170	-0.5	26.7	70	60.84	46.0	even	ok
	71	C48H70N31O57	664.1172	-0.3	26.7	71	78.16	31.0	even	ok
	72	C46H88N15NaO68P	664.1169	-0.6	26.9	72	69.71	12.0	even	ok
	73	C28H34N85O23S2	664.1176	0.4	27.0	73	74.82	56.0	even	ok
	74	C23H14N109O7S2	664.1172	-0.3	27.2	74	64.16	73.0	even	ok
	75	C40H67N41NaO51S	664.1171	-0.5	27.2	75	72.98	29.0	even	ok
	76	C29H43N75NaO29S2	664.1173	-0.1	27.3	76	80.22	47.0	even	ok
	77	C37H46N65O35S	664.1174	0.1	27.3	77	81.67	49.0	even	ok
	78	C42H81N27NaO59P2	664.1175	0.2	27.3	78	79.05	18.0	even	ok
	79	C24H32N91O19P2S	664.1172	-0.3	27.4	79	63.43	57.0	even	ok
	80	C36H45N69NaO29P2	664.1175	0.2	27.5	80	65.94	51.0	even	ok
	81	C25H43N79O28PS2	664.1177	0.6	27.6	81	70.33	46.0	even	ok
	82	C25H21N103NaO7P2S	664.1177	0.5	27.7	82	59.56	69.0	even	ok
	83	C43H84N23O63P2	664.1178	0.7	27.7	83	66.74	16.0	even	ok
	84	C35H56N57O41P2	664.1169	-0.6	27.9	84	56.69	39.0	even	ok
	85	C26H22N99O13S2	664.1176	0.4	27.9	85	73.26	67.0	even	ok
	86	C40H76N33O57P2	664.1174	0.0	28.0	86	80.82	22.0	even	ok
	87	C36H67N45O50PS	664.1175	0.2	28.0	87	76.75	28.0	even	ok
	88	C52H87N11NaO69	664.1173	-0.1	28.1	88	78.76	16.0	even	ok
	89	C22H20N105O9P2S	664.1172	-0.3	28.3	89	62.05	68.0	even	ok
	90	C27H48N73NaO30PS2	664.1178	0.7	28.3	90	66.22	42.0	even	ok
	91	C50H75N25NaO59	664.1173	-0.1	28.4	91	78.17	27.0	even	ok
	92	C41H63N45NaO47S	664.1175	0.2	28.4	92	76.39	34.0	even	ok
	93	C33H44N71O31P2	664.1169	-0.6	28.5	93	55.88	50.0	even	ok
	94	C47H91N11O72P	664.1173	-0.1	28.5	94	78.41	10.0	even	ok
	95	C38H72N39NaO52PS	664.1176	0.4	28.7	95	72.58	24.0	even	ok
	96	C30H39N79NaO25S2	664.1177	0.5	28.8	96	68.69	52.0	even	ok

Compound Spectrum SmartFormula Report

Meas. m/z	#	Ion Formula	m/z	err [ppm]	mSigma	# mSigma	Score	rdb	e⁻ Conf	N-Rule
	97	C34H40N75O27P2	664.1174	0.0	28.8	97	66.31	55.0	even	ok
	98	C39H51N59NaO37S	664.1175	0.2	29.0	98	75.54	45.0	even	ok
	99	C23H16N109O5P2S	664.1176	0.4	29.0	99	60.17	73.0	even	ok
	100	C18H28N101O13P2S2	664.1178	0.7	29.0	100	54.09	58.0	even	ok
	101	C49H96N5NaO74P	664.1174	0.0	29.1	101	78.86	6.0	even	ok
	102	C17H25N105NaO9P2S2	664.1175	0.2	29.4	102	62.82	60.0	even	ok
	103	C28H27N93NaO15S2	664.1177	0.5	29.6	103	67.50	63.0	even	ok
	104	C29H52N67O35P2S	664.1176	0.4	29.7	104	58.85	40.0	even	ok
	105	C42H51N57O38P	664.1177	0.5	29.9	105	55.71	48.0	even	ok
	106	C52H94N3O77	664.1172	-0.3	30.1	106	72.34	9.0	even	ok
	107	C41H70N37O55S	664.1174	0.1	30.1	107	76.19	27.0	even	ok
	108	C28H49N71NaO31P2S	664.1173	-0.2	30.1	108	61.91	42.0	even	ok
	109	C30H46N71O33S2	664.1176	0.4	30.1	109	69.26	45.0	even	ok
	110	C39H43N67O32P	664.1173	-0.1	30.5	110	61.93	54.0	even	ok
	111	C44H56N51NaO40P	664.1178	0.7	30.5	111	52.52	44.0	even	ok
	112	C51H78N21O63	664.1176	0.4	30.5	112	68.70	25.0	even	ok
	113	C41H48N61NaO34P	664.1174	0.0	30.6	113	63.80	50.0	even	ok
	114	C15H20N111O7P2S2	664.1174	0.0	30.6	114	63.39	64.0	even	ok
	115	C30H38N75O34	664.1178	0.6	30.7	115	53.03	51.0	even	ok
	116	C39H73N37NaO53P2	664.1170	-0.5	30.9	116	55.23	24.0	even	ok
	117	C53H90N7O73	664.1176	0.4	30.9	117	67.89	14.0	even	ok
	118	C40H54N55O41S	664.1178	0.7	31.1	118	61.14	43.0	even	ok
	119	C46H58N45O47	664.1172	-0.3	31.1	119	70.23	42.0	even	ok
	120	C28H19N101O10PS	664.1175	0.2	31.2	120	59.59	72.0	even	ok
	121	C42H66N41O51S	664.1179	0.7	31.2	121	60.80	32.0	even	ok
	122	C50H99NO78P	664.1177	0.6	31.3	122	64.09	4.0	even	ok
	123	C30H24N95NaO12PS	664.1176	0.3	31.3	123	57.08	68.0	even	ok
	124	C26H44N77O29P2S	664.1172	-0.3	31.3	124	57.76	46.0	even	ok
	125	C14H17N115NaO3P2S2	664.1170	-0.5	31.8	125	53.85	66.0	even	ok
	126	C43H75N31NaO57S	664.1175	0.2	31.8	126	70.27	23.0	even	ok
	127	C33H37N79NaO23P2	664.1170	-0.5	31.8	127	53.68	57.0	even	ok
	128	C32H51N65NaO35S2	664.1177	0.5	31.9	128	63.61	41.0	even	ok
	129	C22H13N113NaOP2S	664.1172	-0.2	32.0	129	58.92	75.0	even	ok
	130	C38H40N71NaO28P	664.1169	-0.7	32.0	130	50.96	56.0	even	ok
	131	C33H45N75NaO22P2S2	664.1169	-0.7	32.1	131	59.52	51.0	even	ok
	132	C37H68N43O51P2	664.1169	-0.6	32.1	132	51.33	28.0	even	ok
	133	C29H50N67O37S2	664.1172	-0.3	32.1	133	68.74	40.0	even	ok
	134	C40H74N33O59S	664.1170	-0.6	32.4	134	61.89	22.0	even	ok
	135	C53H83N15NaO65	664.1177	0.5	32.4	135	62.84	21.0	even	ok
	136	C48H63N39NaO49	664.1173	-0.1	32.4	136	70.66	38.0	even	ok
	137	C27H16N105NaO6PS	664.1171	-0.3	32.7	137	55.42	74.0	even	ok
	138	C32H50N61O44	664.1178	0.6	32.8	138	50.31	40.0	even	ok
	139	C23H44N79NaO28PS2	664.1169	-0.6	32.8	139	60.32	43.0	even	ok
	140	C35H57N61NaO32P2S2	664.1169	-0.7	32.8	140	58.54	40.0	even	ok
	141	C55H95NNaO75	664.1177	0.6	32.9	141	61.98	10.0	even	ok
	142	C32H26N89O19S	664.1169	-0.6	33.0	142	50.42	66.0	even	ok

Compound Spectrum SmartFormula Report

Meas. m/z	#	Ion Formula	m/z	err [ppm]	mSigma	# mSigma	Score	rdb	e⁻ Conf	N-Rule
	143	C18H11N113NaO8S	664.1176	0.4	33.0	143	53.24	71.0	even	ok
	144	C35H36N79O23P2	664.1178	0.7	33.0	144	49.20	60.0	even	ok
	145	C28H26N89O24	664.1178	0.6	33.1	145	50.10	62.0	even	ok
	146	C25H11N111O4PS	664.1170	-0.5	33.1	146	52.66	78.0	even	ok
	147	C37H39N73NaO27S	664.1175	0.2	33.2	147	68.14	56.0	even	ok
	148	C12H12N121OP2S2	664.1169	-0.6	33.3	148	49.59	70.0	even	ok
	149	C29H35N79NaO30	664.1174	0.1	33.4	149	57.91	53.0	even	ok
	150	C31H55N61NaO39S2	664.1173	-0.1	33.4	150	69.12	36.0	even	ok
	151	C31H32N85O21P2	664.1169	-0.7	33.5	151	49.31	61.0	even	ok
	152	C42H79N27NaO61S	664.1171	-0.5	33.6	152	62.51	18.0	even	ok
	153	C49H66N35O53	664.1176	0.4	33.7	153	63.47	36.0	even	ok
	154	C20H23N99NaO18S	664.1177	0.4	33.9	154	51.97	60.0	even	ok
	155	C34H31N83NaO21S	664.1170	-0.5	34.0	155	51.27	62.0	even	ok
	156	C45H72N37O48P2S	664.1170	-0.5	34.1	156	60.25	31.0	even	ok
	157	C24H47N75O32PS2	664.1173	-0.1	34.2	157	68.10	41.0	even	ok
	158	C20H40N87O23P2S2	664.1178	0.7	34.3	158	56.83	47.0	even	ok
	159	C26H52N69NaO34PS2	664.1174	0.0	34.5	159	69.11	37.0	even	ok
	160	C38H42N69O31S	664.1178	0.7	34.5	160	56.20	54.0	even	ok
	161	C31H47N65NaO40	664.1174	0.1	34.6	161	56.18	42.0	even	ok
	162	C34H48N71O26P2S2	664.1172	-0.2	34.6	162	65.51	49.0	even	ok
	163	C33H69N47NaO47P2S	664.1177	0.5	34.7	163	59.81	25.0	even	ok
	164	C30H61N57NaO41P2S	664.1173	-0.2	34.7	164	66.39	31.0	even	ok
	165	C35H34N79O25S	664.1174	0.1	34.7	165	56.81	60.0	even	ok
	166	C35H71N41O54PS	664.1171	-0.4	34.8	166	60.95	23.0	even	ok
	167	C31H33N89NaO12P2S2	664.1169	-0.8	34.8	167	55.37	62.0	even	ok
	168	C44H78N27O61S	664.1179	0.7	34.9	168	55.32	21.0	even	ok
	169	C31H64N53O45P2S	664.1176	0.4	35.0	169	61.74	29.0	even	ok
	170	C37H76N35NaO56PS	664.1172	-0.3	35.1	170	63.06	19.0	even	ok
	171	C16H6N119O6S	664.1176	0.3	35.1	171	52.61	75.0	even	ok
	172	C44H93N13NaO69P2	664.1175	0.2	35.4	172	64.67	7.0	even	ok
	173	C51H71N29NaO55	664.1177	0.5	35.4	173	58.34	32.0	even	ok
	174	C41H85N23NaO63P2	664.1170	-0.5	35.4	174	59.13	13.0	even	ok
	175	C27H30N85O28	664.1173	-0.0	35.5	175	55.95	57.0	even	ok
	176	C47H84N23O58P2S	664.1170	-0.5	35.6	176	58.09	20.0	even	ok
	177	C24H10N113O3S2	664.1176	0.4	35.6	177	50.54	78.0	even	ok
	178	C48H100NNaO78P	664.1169	-0.6	35.7	178	56.19	1.0	even	ok
	179	C34H33N83NaO19P2	664.1175	0.2	35.7	179	53.88	62.0	even	ok
	180	C18H18N105O16S	664.1176	0.3	35.8	180	51.61	64.0	even	ok
	181	C42H88N19O67P2	664.1174	0.0	35.8	181	66.49	11.0	even	ok
	182	C36H60N57O36P2S2	664.1172	-0.2	35.9	182	63.54	38.0	even	ok
	183	C43H60N51O38P2S	664.1170	-0.5	35.9	183	57.33	42.0	even	ok
	184	C47H77N31NaO50P2S	664.1171	-0.4	36.1	184	59.57	27.0	even	ok
	185	C39H80N29O61P2	664.1169	-0.6	36.2	185	55.55	17.0	even	ok
	186	C45H96N9O73P2	664.1178	0.7	36.3	186	53.82	5.0	even	ok
	187	C27H23N93NaO20	664.1174	0.1	36.4	187	53.80	64.0	even	ok
	188	C27H55N65O38PS2	664.1177	0.6	36.4	188	56.42	35.0	even	ok

Compound Spectrum SmartFormula Report

Meas. m/z	#	Ion Formula	m/z	err [ppm]	mSigma	# mSigma	Score	rdb	e⁻ Conf	N-Rule
	189	C32H58N57O43S2	664.1176	0.4	36.4	189	59.07	34.0	even	ok
	190	C29H42N71O38	664.1173	-0.0	36.4	190	54.71	46.0	even	ok
	191	C43H82N23O65S	664.1174	0.1	36.6	191	64.60	16.0	even	ok
	192	C32H36N85O16P2S2	664.1172	-0.2	36.7	192	62.01	60.0	even	ok
	193	C36H53N65NaO28P2S2	664.1173	-0.1	36.7	193	64.52	45.0	even	ok
	194	C37H69N47NaO42P2S2	664.1169	-0.7	36.8	194	52.84	29.0	even	ok
	195	C32H28N89O17P2	664.1174	0.0	36.9	195	54.23	66.0	even	ok
	196	C38H79N31O60PS	664.1175	0.2	36.9	196	61.33	17.0	even	ok
	197	C19H37N91NaO19P2S2	664.1175	0.2	37.0	197	51.78	49.0	even	ok
	198	C29H60N59NaO40PS2	664.1178	0.7	37.0	198	53.11	31.0	even	ok
	199	C26H15N107NaO5S2	664.1177	0.5	37.1	199	46.65	74.0	even	ok
	200	C56H89N11NaO62P2	664.1169	-0.7	37.1	200	52.33	20.0	even	ok
	201	C40H84N25NaO62PS	664.1176	0.4	37.5	201	57.99	13.0	even	ok
	202	C49H89N17NaO60P2S	664.1171	-0.4	37.6	202	57.38	16.0	even	ok
	203	C45H65N45NaO40P2S	664.1171	-0.4	37.8	203	56.88	38.0	even	ok
	204	C17H32N97O17P2S2	664.1174	0.0	37.8	204	52.63	53.0	even	ok
	205	C38H65N51NaO38P2S2	664.1173	-0.1	37.9	205	62.52	34.0	even	ok
	206	C34H63N51NaO45S2	664.1177	0.6	37.9	206	54.36	30.0	even	ok
	207	C45H87N17NaO67S	664.1175	0.2	38.1	207	59.71	12.0	even	ok
	208	C17H15N109NaO12S	664.1172	-0.2	38.1	208	49.54	66.0	even	ok
	209	C16H29N101NaO13P2S2	664.1170	-0.5	38.1	209	45.83	55.0	even	ok
	210	C15H3N123NaO2S	664.1172	-0.2	38.2	210	49.37	77.0	even	ok
	211	C25H18N99O18	664.1173	-0.0	38.4	211	51.75	68.0	even	ok
	212	C34H62N47O54	664.1178	0.7	38.5	212	43.24	29.0	even	ok
	213	C34H41N79NaO18P2S2	664.1173	-0.1	38.6	213	61.29	56.0	even	ok
	214	C26H27N89NaO24	664.1170	-0.6	38.6	214	44.27	59.0	even	ok
	215	C22H35N85NaO28S	664.1177	0.4	38.6	215	45.89	49.0	even	ok
	216	C28H56N63O39P2S	664.1172	-0.3	38.6	216	47.90	35.0	even	ok
	217	C28H39N75NaO34	664.1170	-0.6	38.7	217	44.16	48.0	even	ok
	218	C48H80N27O54P2S	664.1174	0.1	39.0	218	59.65	25.0	even	ok
	219	C26H14N103O14	664.1178	0.6	39.0	219	42.93	73.0	even	ok
	220	C40H39N71O28P	664.1177	0.5	39.1	220	44.02	59.0	even	ok
	221	C14H24N107O11P2S2	664.1169	-0.6	39.3	221	42.52	59.0	even	ok
	222	C9H6N125O4S2	664.1178	0.6	39.3	222	42.45	71.0	even	ok
	223	C57H92N7O66P2	664.1172	-0.2	39.4	223	57.80	18.0	even	ok
	224	C33H59N51NaO50	664.1174	0.1	39.4	224	49.38	31.0	even	ok
	225	C42H44N65NaO30P	664.1178	0.7	39.5	225	41.72	55.0	even	ok
	226	C37H56N61O32P2S2	664.1177	0.5	39.5	226	53.47	43.0	even	ok
	227	C47H54N49O43	664.1176	0.4	39.8	227	54.25	47.0	even	ok
	228	C31H25N93NaO13P2	664.1170	-0.5	39.9	228	43.37	68.0	even	ok
	229	C49H96N9O68P2S	664.1170	-0.5	40.0	229	51.66	9.0	even	ok
	230	C38H72N43O46P2S2	664.1172	-0.2	40.1	230	56.84	27.0	even	ok
	231	C46H68N41O44P2S	664.1174	0.1	40.1	231	58.00	36.0	even	ok
	232	C20H30N91O26S	664.1176	0.3	40.1	232	45.90	53.0	even	ok
	233	C15H10N115O10S	664.1171	-0.4	40.1	233	45.04	70.0	even	ok
	234	C39H36N75NaO24P	664.1174	0.0	40.3	234	49.67	61.0	even	ok

Compound Spectrum SmartFormula Report

Meas. m/z	#	Ion Formula	m/z	err [ppm]	mSigma	# mSigma	Score	rdb	e^- Conf	N-Rule
	235	C37H31N81O22P	664.1173	-0.1	40.3	235	47.91	65.0	even	ok
	236	C31H62N53O47S2	664.1172	-0.3	40.4	236	55.38	29.0	even	ok
	237	C41H48N65O28P2S	664.1170	-0.6	40.7	237	50.38	53.0	even	ok
	238	C26H34N81O32	664.1169	-0.7	40.7	238	39.99	52.0	even	ok
	239	C24H22N95O22	664.1169	-0.7	40.8	239	39.92	63.0	even	ok
	240	C8H3N129NaS2	664.1174	0.1	40.8	240	47.71	73.0	even	ok
	241	C35H44N75O22P2S2	664.1177	0.5	40.8	241	51.71	54.0	even	ok
	242	C42H86N19O69S	664.1170	-0.6	40.8	242	49.65	11.0	even	ok
	243	C50H92N13O64P2S	664.1174	0.1	40.8	243	56.63	14.0	even	ok
	244	C31H54N57O48	664.1173	-0.0	40.9	244	48.57	35.0	even	ok
	245	C28H12N109NaO2PS	664.1176	0.3	41.1	245	44.28	79.0	even	ok
	246	C49H59N43NaO45	664.1177	0.5	41.1	246	50.15	43.0	even	ok
	247	C26H7N115PS	664.1175	0.2	41.1	247	46.06	83.0	even	ok
	248	C39H68N47O42P2S2	664.1177	0.5	41.1	248	51.04	32.0	even	ok
	249	C50H85N21NaO56P2S	664.1175	0.3	41.2	249	54.05	21.0	even	ok
	250	C46H90N13O71S	664.1179	0.8	41.2	250	46.70	10.0	even	ok
	251	C29H20N99O11P2	664.1169	-0.7	41.4	251	39.88	72.0	even	ok
	252	C59H97NNaO68P2	664.1173	-0.1	41.5	252	56.77	14.0	even	ok
	253	C54H77N25NaO52P2	664.1169	-0.7	41.5	253	46.42	31.0	even	ok
	254	C19H27N95NaO22S	664.1172	-0.2	41.5	254	45.16	55.0	even	ok
	255	C30H24N99O6P2S2	664.1172	-0.2	41.5	255	54.26	71.0	even	ok
	256	C33H67N47NaO49S2	664.1173	-0.1	41.6	256	55.78	25.0	even	ok
	257	C44H46N59O37	664.1172	-0.3	41.7	257	44.29	53.0	even	ok
	258	C39H61N55NaO34P2S2	664.1178	0.6	41.7	258	48.26	39.0	even	ok
	259	C24H15N103NaO14	664.1170	-0.6	41.9	259	40.33	70.0	even	ok
	260	C30H14N103O9S	664.1169	-0.6	41.9	260	39.72	77.0	even	ok
	261	C44H91N13NaO71S	664.1171	-0.5	41.9	261	50.22	7.0	even	ok
	262	C51H101N3NaO70P2S	664.1171	-0.4	41.9	262	51.10	5.0	even	ok
	263	C40H77N37NaO48P2S2	664.1173	-0.1	42.0	263	56.00	23.0	even	ok
	264	C36H28N85NaO18P	664.1169	-0.7	42.1	264	38.96	67.0	even	ok
	265	C48H73N35NaO46P2S	664.1175	0.3	42.1	265	52.69	32.0	even	ok
	266	C25H56N65NaO38PS2	664.1169	-0.6	42.2	266	47.07	32.0	even	ok
	267	C43H53N59NaO30P2S	664.1171	-0.4	42.3	267	50.24	49.0	even	ok
	268	C30H51N61NaO44	664.1170	-0.6	42.3	268	40.06	37.0	even	ok
	269	C25H11N107NaO10	664.1174	0.1	42.5	269	45.59	75.0	even	ok
	270	C21H49N77NaO29P2S2	664.1175	0.2	42.5	270	53.36	38.0	even	ok
	271	C33H22N93O15S	664.1174	0.1	42.6	271	45.98	71.0	even	ok
	272	C32H19N97NaO11S	664.1170	-0.5	42.6	272	40.63	73.0	even	ok
	273	C18H41N87NaO23P2S2	664.1170	-0.5	42.7	273	48.57	44.0	even	ok
	274	C36H74N33O64	664.1178	0.7	42.7	274	46.16	18.0	even	ok
	275	C46H51N53NaO39	664.1173	-0.1	42.8	275	44.67	49.0	even	ok
	276	C37H49N69NaO24P2S2	664.1178	0.6	42.8	276	46.80	50.0	even	ok
	277	C35H71N37NaO60	664.1174	0.1	42.8	277	53.81	20.0	even	ok
	278	C33H24N93O13P2	664.1178	0.7	42.9	278	37.86	71.0	even	ok
	279	C52H97N7NaO66P2S	664.1175	0.3	43.0	279	51.29	10.0	even	ok
	280	C19H44N83O27P2S2	664.1174	0.0	43.0	280	54.69	42.0	even	ok

Compound Spectrum SmartFormula Report

Meas. m/z	#	Ion Formula	m/z	err [ppm]	mSigma	# mSigma	Score	rdb	e^- Conf	N-Rule
	281	C55H80N21O56P2	664.1172	-0.2	43.1	281	52.12	29.0	even	ok
	282	C32H29N93NaO8P2S2	664.1173	-0.1	43.2	282	53.91	67.0	even	ok
	283	C39H81N33NaO52P2S2	664.1169	-0.7	43.3	283	44.38	18.0	even	ok
	284	C17H22N101O20S	664.1171	-0.4	43.3	284	41.31	59.0	even	ok
	285	C41H73N41NaO44P2S2	664.1178	0.6	43.3	285	46.05	28.0	even	ok
	286	C32H73N43NaO51P2S	664.1173	-0.2	43.3	286	52.65	20.0	even	ok
	287	C22H52N73O33P2S2	664.1178	0.7	43.4	287	44.45	36.0	even	ok
	288	C35H27N87NaO17S	664.1175	0.2	43.7	288	42.88	67.0	even	ok
	289	C26H59N61O42PS2	664.1173	-0.1	43.8	289	52.63	30.0	even	ok
	290	C30H68N49O49P2S	664.1172	-0.3	43.8	290	49.90	24.0	even	ok
	291	C44H56N55O34P2S	664.1174	0.1	43.9	291	52.22	47.0	even	ok
	292	C35H81N33NaO57P2S	664.1177	0.5	43.9	292	46.48	14.0	even	ok
	293	C28H46N67O42	664.1169	-0.7	44.1	293	36.51	41.0	even	ok
	294	C22H10N109O12	664.1169	-0.7	44.1	294	36.31	74.0	even	ok
	295	C28H64N55NaO44PS2	664.1174	0.0	44.1	295	53.17	26.0	even	ok
	296	C43H97N9NaO73P2	664.1170	-0.5	44.1	296	46.78	2.0	even	ok
	297	C33H76N39O55P2S	664.1176	0.4	44.1	297	48.17	18.0	even	ok
	298	C36H30N83O21S	664.1178	0.7	44.3	298	35.92	65.0	even	ok
	299	C37H83N27O64PS	664.1171	-0.4	44.4	299	46.97	12.0	even	ok
	300	C23H6N113O8	664.1173	-0.0	44.4	300	43.74	79.0	even	ok
	301	C34H70N43O53S2	664.1176	0.4	44.4	301	47.28	23.0	even	ok
	302	C41H92N15O71P2	664.1169	-0.6	44.6	302	44.17	6.0	even	ok
	303	C39H88N21NaO66PS	664.1172	-0.3	44.7	303	48.51	8.0	even	ok
	304	C33H32N89O12P2S2	664.1177	0.5	44.7	304	46.36	65.0	even	ok
	305	C45H94N9O75S	664.1174	0.1	44.7	305	51.48	5.0	even	ok
	306	C44H100N5O77P2	664.1174	0.1	44.9	306	51.75	0.0	even	ok
	307	C57H85N15NaO58P2	664.1173	-0.1	44.9	307	51.41	25.0	even	ok
	308	C41H80N33O52P2S2	664.1177	0.5	45.4	308	45.20	21.0	even	ok
	309	C32H21N97NaO9P2	664.1175	0.2	45.6	309	41.11	73.0	even	ok
	310	C46H61N49NaO36P2S	664.1175	0.3	45.7	310	47.63	43.0	even	ok
	311	C36H75N37NaO55S2	664.1177	0.6	45.8	311	43.60	19.0	even	ok
	312	C24H47N71NaO38S	664.1177	0.5	45.9	312	37.30	38.0	even	ok
	313	C29H67N51O48PS2	664.1177	0.6	46.1	313	43.07	24.0	even	ok
	314	C47H99N3NaO77S	664.1175	0.2	46.1	314	47.67	1.0	even	ok
	315	C11H18N111O14S2	664.1178	0.6	46.2	315	34.87	60.0	even	ok
	316	C40H84N29O56P2S2	664.1172	-0.2	46.5	316	47.44	16.0	even	ok
	317	C35H37N83NaO14P2S2	664.1178	0.6	46.5	317	42.13	61.0	even	ok
	318	C30H16N103O7P2	664.1174	0.0	46.6	318	41.39	77.0	even	ok
	319	C31H72N45NaO50PS2	664.1178	0.7	46.6	319	40.52	20.0	even	ok
	320	C40H91N17O70PS	664.1175	0.2	46.7	320	46.68	6.0	even	ok
	321	C58H88N11O62P2	664.1177	0.5	46.9	321	43.41	23.0	even	ok
	322	C10H15N115NaO10S2	664.1174	0.1	46.9	322	39.99	62.0	even	ok
	323	C22H42N77O36S	664.1176	0.3	47.1	323	37.57	42.0	even	ok
	324	C42H96N11NaO72PS	664.1176	0.4	47.2	324	44.10	2.0	even	ok
	325	C13H8N121NaO3PS	664.1178	0.6	47.2	325	34.45	72.0	even	ok
	326	C43H85N27NaO54P2S2	664.1178	0.6	47.4	327	40.83	17.0	even	ok

Compound Spectrum SmartFormula Report

Meas. m/z	#	Ion Formula	m/z	err [ppm]	mSigma	# mSigma	Score	rdb	e¯ Conf	N-Rule
	327	C16H36N93O21P2S2	664.1169	-0.6	47.5	328	33.74	48.0	even	ok
	328	C21H39N81NaO32S	664.1172	-0.2	47.7	329	37.81	44.0	even	ok
	329	C24H32N87NaO25P	664.1175	0.3	47.8	330	37.34	54.0	even	ok
	330	C52H65N39NaO42P2	664.1169	-0.8	47.9	331	38.47	42.0	even	ok
	331	C33H66N43O58	664.1173	-0.0	48.0	332	39.72	24.0	even	ok
	332	C22H3N117NaO4	664.1170	-0.6	48.0	333	33.72	81.0	even	ok
	333	C42H89N23NaO58P2S2	664.1173	-0.1	48.2	334	46.83	12.0	even	ok
	334	C8H10N121O8S2	664.1173	-0.0	48.3	335	39.22	66.0	even	ok
	335	C22H20N101NaO15P	664.1175	0.3	48.4	336	36.79	65.0	even	ok
	336	C32H63N47NaO54	664.1170	-0.6	48.6	337	33.42	26.0	even	ok
	337	C52H87N15O63PS	664.1169	-0.7	48.7	338	38.34	19.0	even	ok
	338	C60H93N5NaO64P2	664.1178	0.6	48.9	339	39.19	19.0	even	ok
	339	C53H68N35O46P2	664.1172	-0.2	48.9	340	43.87	40.0	even	ok
	340	C19H34N87O30S	664.1171	-0.4	49.2	341	34.83	48.0	even	ok
	341	C41H63N49O41PS2	664.1171	-0.4	49.2	342	41.76	37.0	even	ok
	342	C50H75N29O53PS	664.1169	-0.7	49.3	343	37.62	30.0	even	ok
	343	C15H20N107NaO13PS	664.1178	0.6	49.4	344	32.22	61.0	even	ok
	344	C11H3N127OPS	664.1177	0.5	49.4	345	33.66	76.0	even	ok
	345	C7H7N125NaO4S2	664.1170	-0.6	49.4	346	32.52	68.0	even	ok
	346	C38H27N85O18P	664.1177	0.5	49.5	347	32.72	70.0	even	ok
	347	C29H13N107NaO3P2	664.1170	-0.5	49.6	348	32.75	79.0	even	ok
	348	C40H32N79NaO20P	664.1178	0.7	49.7	349	31.13	66.0	even	ok
	349	C33H74N39O57S2	664.1172	-0.3	49.7	350	42.37	18.0	even	ok
	350	C42H44N69O24P2S	664.1174	0.1	49.8	351	43.91	58.0	even	ok
	351	C39H51N63O31PS2	664.1171	-0.4	49.9	352	40.78	48.0	even	ok
	352	C22H27N93O23P	664.1174	0.1	50.0	353	36.35	58.0	even	ok
	353	C30H58N53O52	664.1169	-0.7	50.0	354	30.66	30.0	even	ok
	354	C26H44N73NaO35P	664.1175	0.3	50.2	355	34.75	43.0	even	ok
	355	C44H98N5O79S	664.1170	-0.6	50.2	356	37.85	0.0	even	ok
	356	C26H59N57NaO48S	664.1177	0.5	50.2	357	39.32	27.0	even	ok
	357	C54H99NO73PS	664.1169	-0.7	50.5	358	36.37	8.0	even	ok
	358	C55H73N29NaO48P2	664.1173	-0.1	50.5	359	43.47	36.0	even	ok
	359	C20H15N107O13P	664.1174	0.1	50.7	360	35.69	69.0	even	ok
	360	C35H79N33NaO59S2	664.1173	-0.1	50.7	361	42.72	14.0	even	ok
	361	C43H75N35O51PS2	664.1171	-0.4	50.8	362	39.82	26.0	even	ok
	362	C27H8N113OP2	664.1169	-0.7	50.9	363	30.14	83.0	even	ok
	363	C37H24N89NaO14P	664.1174	0.0	50.9	364	36.47	72.0	even	ok
	364	C54H92N9NaO65PS	664.1170	-0.6	51.0	365	37.35	15.0	even	ok
	365	C24H54N63O46S	664.1176	0.3	51.0	366	40.01	31.0	even	ok
	366	C37H83N23NaO70	664.1174	0.1	51.0	367	42.18	9.0	even	ok
	367	C35H19N95O12P	664.1173	-0.1	51.1	369	34.99	76.0	even	ok
	368	C42H34N73O27	664.1172	-0.3	51.1	370	33.53	64.0	even	ok
	369	C41H93N19NaO62P2S2	664.1169	-0.7	51.2	371	35.12	7.0	even	ok
	370	C38H86N19O74	664.1178	0.7	51.4	372	35.68	7.0	even	ok
	371	C13H15N113O11PS	664.1177	0.5	51.4	373	31.64	65.0	even	ok
	372	C44H49N63NaO26P2S	664.1175	0.3	51.4	374	40.25	54.0	even	ok

Compound Spectrum SmartFormula Report

Meas. m/z	#	Ion Formula	m/z	err [ppm]	mSigma	# mSigma	Score	rdb	e⁻ Conf	N-Rule
	373	C52H80N23NaO55PS	664.1170	-0.6	51.5	375	36.71	26.0	even	ok
	374	C43H68N43NaO43PS2	664.1172	-0.2	51.5	376	40.52	33.0	even	ok
	375	C43H92N19O62P2S2	664.1177	0.5	51.6	377	37.47	10.0	even	ok
	376	C20H53N73NaO33P2S2	664.1170	-0.5	51.6	378	37.36	33.0	even	ok
	377	C20H8N115NaO5P	664.1175	0.2	51.7	379	33.27	76.0	even	ok
	378	C35H78N29O68	664.1173	-0.0	51.8	380	42.44	13.0	even	ok
	379	C34H75N33NaO64	664.1170	-0.6	51.9	381	36.35	15.0	even	ok
	380	C56H76N25O52P2	664.1177	0.5	51.9	382	37.36	34.0	even	ok
	381	C39H36N79O18P2S	664.1170	-0.6	51.9	383	30.18	64.0	even	ok
	382	C44H39N67NaO29	664.1173	-0.1	51.9	384	34.00	60.0	even	ok
	383	C45H42N63O33	664.1176	0.4	52.0	385	31.71	58.0	even	ok
	384	C23H61N63NaO39P2S2	664.1175	0.2	52.0	386	40.25	27.0	even	ok
	385	C31H10N107O5S	664.1174	0.0	52.1	387	34.79	82.0	even	ok
	386	C24H39N79O33P	664.1174	0.1	52.1	388	34.01	47.0	even	ok
	387	C48H63N43O43PS	664.1169	-0.7	52.1	389	34.40	41.0	even	ok
	388	C18H48N79O31P2S2	664.1169	-0.6	52.1	390	35.19	37.0	even	ok
	389	C27H68N51NaO48PS2	664.1169	-0.6	52.1	391	35.09	21.0	even	ok
	390	C41H56N57NaO33PS2	664.1172	-0.2	52.2	392	39.64	44.0	even	ok
	391	C33H25N97NaO4P2S2	664.1178	0.6	52.3	393	35.47	72.0	even	ok
	392	C21H56N69O37P2S2	664.1174	0.0	52.3	394	41.42	31.0	even	ok
	393	C30H7N111NaOS	664.1170	-0.5	52.5	395	30.21	84.0	even	ok
	394	C34H85N29NaO61P2S	664.1173	-0.1	52.8	396	39.73	9.0	even	ok
	395	C33H15N101NaO7S	664.1175	0.2	52.9	397	32.61	78.0	even	ok
	396	C21H24N97NaO19P	664.1171	-0.4	52.9	398	30.51	60.0	even	ok
	397	C37H39N77O21PS2	664.1171	-0.4	52.9	399	37.12	59.0	even	ok
	398	C34H18N97O11S	664.1178	0.7	52.9	400	27.77	76.0	even	ok
	399	C34H16N99NaO8P	664.1169	-0.7	53.0	401	28.16	78.0	even	ok
	400	C45H80N29NaO53PS2	664.1172	-0.2	53.1	402	38.62	22.0	even	ok
	401	C24H64N59O43P2S2	664.1178	0.7	53.1	403	33.16	25.0	even	ok
	402	C32H80N35O59P2S	664.1172	-0.3	53.1	404	37.82	13.0	even	ok
	403	C47H47N57NaO35	664.1177	0.5	53.1	405	29.34	54.0	even	ok
	404	C41H41N73NaO20P2S	664.1171	-0.4	53.3	406	30.08	60.0	even	ok
	405	C45H97N13NaO64P2S2	664.1178	0.6	53.4	407	33.90	6.0	even	ok
	406	C36H82N29O63S2	664.1176	0.4	53.5	408	35.91	12.0	even	ok
	407	C31H12N107O3P2	664.1178	0.7	53.6	409	27.50	82.0	even	ok
	408	C58H81N19NaO54P2	664.1178	0.6	53.6	410	33.87	30.0	even	ok
	409	C12H12N117NaO7PS	664.1173	-0.1	53.7	411	32.77	67.0	even	ok
	410	C37H93N19NaO67P2S	664.1177	0.5	53.8	412	34.48	3.0	even	ok
	411	C35H88N25O65P2S	664.1176	0.4	53.8	413	35.85	7.0	even	ok
	412	C28H71N47O52PS2	664.1173	-0.1	53.8	414	38.89	19.0	even	ok
	413	C43H104NO81P2	664.1169	-0.6	53.9	415	33.39	-5.0	even	ok
	414	C19H12N111NaO9P	664.1171	-0.4	54.0	416	29.39	71.0	even	ok
	415	C17H32N93NaO23PS	664.1178	0.6	54.2	417	27.75	50.0	even	ok
	416	C55H95N5O69PS	664.1173	-0.0	54.2	418	39.38	13.0	even	ok
	417	C30H76N41NaO54PS2	664.1174	0.0	54.2	419	39.12	15.0	even	ok
	418	C50H68N37NaO45PS	664.1170	-0.6	54.2	420	33.67	37.0	even	ok

Compound Spectrum SmartFormula Report

Meas. m/z	#	Ion Formula	m/z	err [ppm]	mSigma	# mSigma	Score	rdb	e¯ Conf	N-Rule
	419	C53H83N19O59PS	664.1173	-0.0	54.3	421	39.16	24.0	even	ok
	420	C42H96N15O66P2S2	664.1172	-0.2	54.4	422	37.35	5.0	even	ok
	421	C23H36N83NaO29P	664.1171	-0.4	54.4	423	29.15	49.0	even	ok
	422	C39H95N13O74PS	664.1171	-0.4	54.5	424	34.62	1.0	even	ok
	423	C45H87N21O61PS2	664.1171	-0.3	54.6	425	35.45	15.0	even	ok
	424	C38H87N23NaO65S2	664.1177	0.6	54.7	426	33.16	8.0	even	ok
	425	C44H71N39O47PS2	664.1176	0.3	54.7	427	35.64	31.0	even	ok
	426	C41H100N7NaO76PS	664.1172	-0.3	54.8	428	35.70	-3.0	even	ok
	427	C13H30N97O24S2	664.1178	0.7	54.9	429	26.71	49.0	even	ok
	428	C12H27N101NaO20S2	664.1174	0.1	54.9	430	31.17	51.0	even	ok
	429	C42H59N53O37PS2	664.1176	0.3	55.0	431	35.37	42.0	even	ok
	430	C39H44N71NaO23PS2	664.1172	-0.2	55.0	432	36.19	55.0	even	ok
	431	C28H56N59NaO45P	664.1175	0.3	55.0	433	29.82	32.0	even	ok
	432	C19H19N103O17P	664.1170	-0.6	55.2	434	27.23	64.0	even	ok
	433	C31H20N103O2P2S2	664.1177	0.5	55.4	435	27.93	76.0	even	ok
	434	C10H7N123O5PS	664.1172	-0.2	55.8	436	29.50	71.0	even	ok
	435	C23H51N67NaO42S	664.1172	-0.2	55.8	437	29.46	33.0	even	ok
	436	C15H27N99O21PS	664.1177	0.5	55.8	438	27.42	54.0	even	ok
	437	C44H101N9NaO68P2S2	664.1173	-0.0	55.9	439	36.93	1.0	even	ok
	438	C10H22N107O18S2	664.1173	-0.0	56.0	440	30.85	55.0	even	ok
	439	C31H79N37O58PS2	664.1177	0.6	56.2	441	31.45	13.0	even	ok
	440	C17H7N117O7P	664.1170	-0.6	56.4	442	26.14	75.0	even	ok
	441	C21H31N89O27P	664.1170	-0.6	56.5	443	26.14	53.0	even	ok
	442	C46H83N25O57PS2	664.1176	0.3	56.5	444	33.55	20.0	even	ok
	443	C9H19N111NaO14S2	664.1170	-0.6	56.5	445	26.04	57.0	even	ok
	444	C51H71N33O49PS	664.1173	-0.0	56.5	446	36.38	35.0	even	ok
	445	C55H88N13NaO61PS	664.1174	0.1	56.6	447	35.42	20.0	even	ok
	446	C26H51N65O43P	664.1174	0.1	56.7	448	29.36	36.0	even	ok
	447	C47H92N15NaO63PS2	664.1172	-0.2	56.7	449	34.41	11.0	even	ok
	448	C33H84N31NaO60PS2	664.1178	0.7	56.7	450	29.57	9.0	even	ok
	449	C42H103N3O80PS	664.1175	0.2	56.8	451	33.99	-5.0	even	ok
	450	C21H46N73O40S	664.1171	-0.4	56.9	452	27.31	37.0	even	ok
	451	C46H51N57O33PS	664.1169	-0.7	56.9	453	29.44	52.0	even	ok
	452	C46H76N33NaO49PS2	664.1177	0.5	57.0	454	31.72	27.0	even	ok
	453	C44H64N47NaO39PS2	664.1177	0.5	57.3	455	31.52	38.0	even	ok
	454	C40H47N67O27PS2	664.1176	0.3	57.3	456	32.81	53.0	even	ok
	455	C14H24N103NaO17PS	664.1173	-0.1	57.5	457	29.02	56.0	even	ok
	456	C53H61N43NaO38P2	664.1173	-0.1	57.6	458	34.62	47.0	even	ok
	457	C32H70N39O62	664.1169	-0.7	57.8	459	23.97	19.0	even	ok
	458	C35H27N91O11PS2	664.1171	-0.4	57.8	460	31.65	70.0	even	ok
	459	C7H14N117O12S2	664.1169	-0.7	57.8	461	23.88	61.0	even	ok
	460	C25H48N69NaO39P	664.1171	-0.4	58.3	462	25.71	38.0	even	ok
	461	C54H64N39O42P2	664.1177	0.5	58.5	463	30.25	45.0	even	ok
	462	C14H39N87NaO30S2	664.1174	0.1	58.7	464	33.03	40.0	even	ok
	463	C53H76N27NaO51PS	664.1174	0.1	58.7	465	33.07	31.0	even	ok
	464	C48H56N51NaO35PS	664.1170	-0.6	58.8	466	28.92	48.0	even	ok

Compound Spectrum SmartFormula Report

Meas. m/z	#	Ion Formula	m/z	err [ppm]	mSigma	# mSigma	Score	rdb	e⁻ Conf	N-Rule
	465	C48H88N19NaO59PS2	664.1177	0.5	58.8	467	29.85	16.0	even	ok
	466	C15H42N83O34S2	664.1178	0.7	59.0	468	27.91	38.0	even	ok
	467	C45H104N5O72P2S2	664.1177	0.5	59.1	469	29.31	-1.0	even	ok
	468	C28H71N43NaO58S	664.1177	0.5	59.3	470	29.37	16.0	even	ok
	469	C12H19N109O15PS	664.1172	-0.2	59.3	471	26.26	60.0	even	ok
	470	C12H34N93O28S2	664.1173	-0.0	59.5	472	33.11	44.0	even	ok
	471	C42H52N61NaO29PS2	664.1177	0.4	59.5	473	29.30	49.0	even	ok
	472	C25H63N53NaO52S	664.1172	-0.2	59.5	474	31.35	22.0	even	ok
	473	C56H91N9O65PS	664.1178	0.7	59.6	475	27.43	18.0	even	ok
	474	C35H86N25O67S2	664.1172	-0.2	59.6	476	30.95	7.0	even	ok
	475	C37H32N85NaO13PS2	664.1172	-0.2	59.7	477	30.97	66.0	even	ok
	476	C18H3N121O3P	664.1174	0.1	59.8	478	21.31	80.0	even	ok
	477	C60H83N13O61P	664.1171	-0.4	59.8	479	29.70	28.0	even	ok
	478	C26H66N49O56S	664.1176	0.3	59.9	480	30.01	20.0	even	ok
	479	C56H69N33NaO44P2	664.1178	0.6	60.0	481	27.53	41.0	even	ok
	480	C47H99N7O71PS2	664.1171	-0.3	60.1	482	29.62	4.0	even	ok
	481	C39H95N9NaO80	664.1174	0.1	60.1	483	31.43	-2.0	even	ok
	482	C43H105N5NaO72P2S2	664.1169	-0.7	60.1	484	26.38	-4.0	even	ok
	483	C23H43N75O37P	664.1170	-0.5	60.1	485	23.18	42.0	even	ok
	484	C48H95N11O67PS2	664.1176	0.3	60.3	486	29.58	9.0	even	ok
	485	C23H58N59O50S	664.1171	-0.4	60.3	487	29.34	26.0	even	ok
	486	C36H87N19NaO74	664.1170	-0.5	60.3	488	27.65	4.0	even	ok
	487	C36H15N99O8P	664.1177	0.5	60.5	489	23.03	81.0	even	ok
	488	C37H91N19NaO69S2	664.1173	-0.1	60.5	490	31.21	3.0	even	ok
	489	C38H20N93NaO10P	664.1178	0.7	60.6	491	21.98	77.0	even	ok
	490	C49H59N47O39PS	664.1173	-0.0	60.7	492	31.67	46.0	even	ok
	491	C37H90N15O78	664.1174	-0.0	60.7	493	31.85	2.0	even	ok
	492	C40H98N5O84	664.1178	0.7	60.7	494	26.26	-4.0	even	ok
	493	C37H24N93O8P2S	664.1170	-0.6	60.9	495	22.51	75.0	even	ok
	494	C19H44N79NaO33PS	664.1178	0.6	60.9	496	22.20	39.0	even	ok
	495	C34H82N25O72	664.1169	-0.7	61.1	497	25.79	8.0	even	ok
	496	C22H65N59NaO43P2S2	664.1170	-0.5	61.2	498	27.40	22.0	even	ok
	497	C11H16N113NaO11PS	664.1169	-0.8	61.3	499	20.95	62.0	even	ok
	498	C54H79N23O55PS	664.1178	0.7	61.3	500	25.92	29.0	even	ok
	499	C40H22N87O17	664.1172	-0.3	61.4	501	23.94	75.0	even	ok
	500	C51H56N49O36P2	664.1172	-0.2	61.5	502	24.32	51.0	even	ok

Cmpd 6, 8.1 min

Compound Spectrum SmartFormula Report

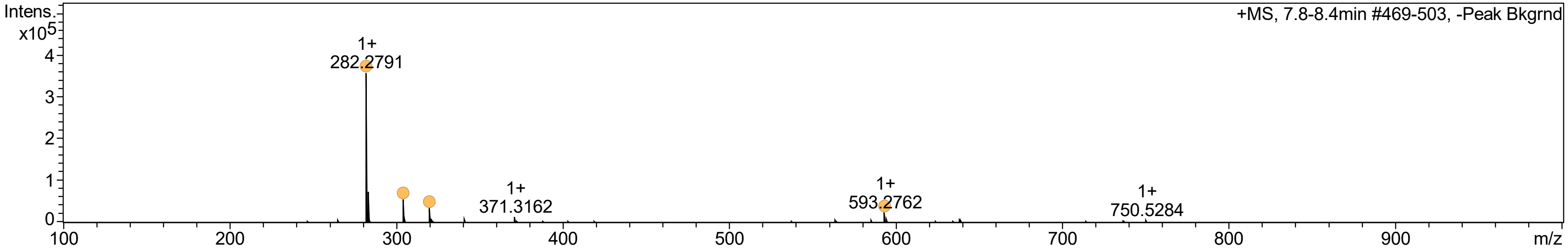

Meas. m/z	#	Ion Formula	m/z	err [ppm]	mSigma	# mSigma	Score	rdb	e^- Conf	N-Rule
282.2791	1	C18H36NO	282.2791	0.2	2.1	1	100.00	2.0	even	ok
304.2612	1	C18H35NNaO	304.2611	-0.4	8.6	1	100.00	2.0	even	ok
	2	C14H35N5P	304.2625	4.2	8.7	2	55.10	1.0	even	ok
	3	C12H36N5NaP	304.2601	-3.8	21.3	3	46.17	-2.0	even	ok
	4	C13H39NO4P	304.2611	-0.2	22.1	4	77.47	-4.0	even	ok
320.2354	1	C15H34N3O2S	320.2366	3.9	14.5	1	100.00	1.0	even	ok
	2	C13H35N3NaO2S	320.2342	-3.6	25.1	2	84.22	-2.0	even	ok
	3	C11H30N9S	320.2339	-4.5	26.4	3	68.62	2.0	even	ok
	4	C21H31NNa	320.2349	-1.6	32.7	4	74.83	7.0	even	ok
	5	C16H35NO3P	320.2349	-1.5	36.5	5	69.05	1.0	even	ok
	6	C13H37N3NaP2	320.2355	0.4	49.9	6	55.35	-2.0	even	ok
593.2762	1	C34H34N8NaO	593.2748	-2.4	7.3	1	58.58	22.0	even	ok
	2	C34H38N6O2P	593.2788	4.4	9.1	2	22.41	20.0	even	ok
	3	C36H43NaO4P	593.2791	4.9	10.0	3	17.05	16.0	even	ok
	4	C35H37N4O5	593.2758	-0.6	10.3	4	100.00	20.0	even	ok
	5	C38H43O2P2	593.2733	-4.9	10.6	5	16.65	19.0	even	ok
	6	C36H33N8O	593.2772	1.7	12.4	6	69.94	25.0	even	ok
	7	C38H38N2NaO3	593.2775	2.1	14.4	7	57.03	21.0	even	ok
	8	C33H38N4NaO5	593.2734	-4.7	16.3	8	17.33	17.0	even	ok
	9	C32H39N6NaO2P	593.2764	0.4	18.0	9	91.03	17.0	even	ok
	10	C34H41O9	593.2745	-2.9	18.5	10	39.35	15.0	even	ok
	11	C30H34N12P	593.2762	-0.1	18.7	11	96.55	21.0	even	ok
	12	C33H42N2O6P	593.2775	2.2	19.3	12	50.42	15.0	even	ok
	13	C31H38N8NaOS	593.2781	3.3	28.2	13	26.43	17.0	even	ok
	14	C31H43N6P2S	593.2740	-3.8	28.4	14	20.88	15.0	even	ok
	15	C35H47O2P2S	593.2767	0.8	28.6	15	65.03	14.0	even	ok
	16	C31H43N2NaO6P	593.2751	-1.9	29.5	16	44.81	12.0	even	ok
	17	C28H35N12NaP	593.2737	-4.1	29.8	17	16.84	18.0	even	ok
	18	C33H48NaO2P2S	593.2742	-3.3	29.8	18	25.12	11.0	even	ok
	19	C29H38N8O4P	593.2748	-2.3	30.1	19	37.14	16.0	even	ok
	20	C32H41N4O5S	593.2792	5.1	32.0	20	9.60	15.0	even	ok
	21	C30H44N4NaO3P2	593.2781	3.2	32.0	21	25.25	12.0	even	ok
	22	C31H47O7P2	593.2792	5.0	32.7	22	10.04	10.0	even	ok

Compound Spectrum SmartFormula Report

Meas. m/z	#	Ion Formula	m/z	err [ppm]	mSigma	# mSigma	Score	rdb	e¯ Conf	N-Rule
	23	C37H42N2OPS	593.2750	-2.0	32.9	23	38.88	19.0	even	ok
	24	C28H39N10OP2	593.2778	2.7	32.9	24	30.13	16.0	even	ok
	25	C28H37N10O3S	593.2765	0.6	34.9	25	58.81	16.0	even	ok
	26	C30H42N4NaO5S	593.2768	1.0	35.9	26	50.11	12.0	even	ok
	27	C31H45O9S	593.2779	2.8	39.3	27	24.20	10.0	even	ok
	28	C28H42N4O8P	593.2735	-4.6	41.7	28	9.74	11.0	even	ok
	29	C24H33N16OS	593.2738	-4.0	41.9	29	13.36	17.0	even	ok
	30	C26H38N10NaO3S	593.2741	-3.5	42.3	30	16.56	13.0	even	ok
	31	C39H37N4S	593.2733	-4.8	42.5	31	8.40	24.0	even	ok
	32	C25H39N12NaPS	593.2771	1.5	43.0	32	35.20	13.0	even	ok
	33	C29H48NaO7P2	593.2767	0.9	43.5	33	42.07	7.0	even	ok
	34	C27H43N6O5P2	593.2765	0.4	44.3	34	46.87	11.0	even	ok
	35	C32H47N2NaOPS2	593.2760	-0.4	44.5	35	58.85	11.0	even	ok
	36	C27H41N6O7S	593.2752	-1.7	44.9	36	31.58	11.0	even	ok
	37	C26H42N8O4PS	593.2782	3.3	45.3	37	16.39	11.0	even	ok
	38	C28H47N6P2S2	593.2773	1.9	45.5	38	36.08	10.0	even	ok
	39	C29H46NaO9S	593.2755	-1.2	45.6	39	36.09	7.0	even	ok
	40	C28H47N2NaO6PS	593.2785	3.8	45.7	40	12.99	7.0	even	ok
	41	C34H46N2OPS2	593.2784	3.6	46.2	41	17.30	14.0	even	ok
	42	C34H42N4NaS2	593.2743	-3.2	46.2	42	21.28	16.0	even	ok
	43	C30H52NaO2P2S2	593.2776	2.4	46.9	43	29.11	6.0	even	ok
	44	C29H46N4O3PS2	593.2743	-3.1	47.3	44	21.13	10.0	even	ok
	45	C26H48N6NaP2S2	593.2749	-2.1	49.7	45	29.28	7.0	even	ok
	46	C35H45O4S2	593.2754	-1.4	49.8	46	37.77	14.0	even	ok
	47	C26H40N10NaOP2	593.2754	-1.4	50.4	47	22.51	13.0	even	ok
	48	C36H41N4S2	593.2767	0.9	50.9	48	42.94	19.0	even	ok
	49	C23H34N14NaO4	593.2780	3.0	52.4	49	15.52	14.0	even	ok
	50	C27H51N2O4P2S2	593.2760	-0.3	52.7	50	46.69	5.0	even	ok
	51	C24H37N10O8	593.2790	4.8	52.8	51	6.37	12.0	even	ok
	52	C22H38N14O2PS	593.2755	-1.2	54.4	52	27.96	12.0	even	ok
	53	C24H43N8NaO4PS	593.2758	-0.7	54.4	53	32.09	8.0	even	ok
	54	C21H37N16OS2	593.2772	1.7	55.2	54	28.77	12.0	even	ok
	55	C25H44N6NaO5P2	593.2741	-3.6	55.4	55	10.61	8.0	even	ok
	56	C26H45N2O11S	593.2739	-4.0	55.7	56	8.91	6.0	even	ok
	57	C23H44N10NaOP2S	593.2788	4.3	55.8	57	7.36	8.0	even	ok
	58	C26H47N2O9P2	593.2751	-1.8	55.8	58	21.78	6.0	even	ok
	59	C23H42N10NaO3S2	593.2775	2.2	56.0	59	23.72	8.0	even	ok
	60	C25H46N4O8PS	593.2768	1.1	56.4	60	27.00	6.0	even	ok
	61	C23H47N8O2P2S2	593.2733	-4.9	57.5	61	6.47	6.0	even	ok
	62	C25H52N2NaO4P2S2	593.2736	-4.4	58.2	62	8.16	2.0	even	ok
	63	C24H45N6O7S2	593.2786	4.0	58.8	63	9.92	6.0	even	ok
	64	C26H50NaO9S2	593.2788	4.5	59.7	64	7.55	2.0	even	ok
	65	C21H29N20O2	593.2777	2.5	61.2	65	10.58	18.0	even	ok
	66	C25H46NaO14	593.2780	3.0	63.8	66	10.59	3.0	even	ok
	67	C22H38N10NaO8	593.2766	0.7	63.8	67	23.38	9.0	even	ok
	68	C23H41N6O12	593.2777	2.5	64.4	68	12.55	7.0	even	ok

Compound Spectrum SmartFormula Report

Meas. m/z	#	Ion Formula	m/z	err [ppm]	mSigma	# mSigma	Score	rdb	e⁻ Conf	N-Rule
	69	C20H41N12O5S2	593.2759	-0.5	64.4	69	30.00	7.0	even	ok
	70	C23H39N12O3P2	593.2738	-4.1	64.7	70	4.63	12.0	even	ok
	71	C20H33N16O6	593.2763	0.2	64.7	71	25.70	13.0	even	ok
	72	C22H46N6NaO7S2	593.2762	-0.1	65.0	72	33.10	3.0	even	ok
	73	C21H47N8NaO4PS2	593.2792	5.0	65.1	73	4.73	3.0	even	ok
	74	C23H47N4NaO8PS	593.2744	-3.0	65.9	74	9.87	3.0	even	ok
	75	C21H42N10O6PS	593.2742	-3.4	66.0	75	8.00	7.0	even	ok
	76	C22H48N6NaO5P2S	593.2774	2.1	67.5	76	13.31	3.0	even	ok
	77	C23H49N2O11S2	593.2772	1.7	67.6	77	18.67	1.0	even	ok
	78	C20H43N12O3P2S	593.2772	1.6	67.7	78	15.57	7.0	even	ok
	79	C24H50O12PS	593.2755	-1.2	67.8	79	17.75	1.0	even	ok
	80	C23H51N2O9P2S	593.2785	3.9	69.1	80	5.83	1.0	even	ok
	81	C19H38N16NaOS2	593.2748	-2.3	69.2	81	11.26	9.0	even	ok
	82	C18H42N12NaO5S2	593.2735	-4.6	71.8	82	4.53	4.0	even	ok
	83	C19H42N14O2PS2	593.2789	4.5	72.2	83	3.78	7.0	even	ok
	84	C19H45N8O9S2	593.2745	-2.8	74.1	84	9.79	2.0	even	ok
	85	C19H30N20NaO2	593.2753	-1.6	74.1	85	9.30	15.0	even	ok
	86	C18H46N10O6PS2	593.2775	2.2	74.5	86	11.99	2.0	even	ok
	87	C21H50N2NaO11S2	593.2748	-2.3	74.6	87	11.59	-2.0	even	ok
	88	C20H51N4NaO8PS2	593.2778	2.7	74.9	88	9.83	-2.0	even	ok
	89	C16H34N20NaO2S	593.2787	4.1	75.1	89	4.11	10.0	even	ok
	90	C17H25N26	593.2750	-2.0	75.3	90	7.56	19.0	even	ok
	91	C21H42N6NaO12	593.2753	-1.5	75.3	91	11.90	4.0	even	ok
	92	C14H29N26S	593.2784	3.7	75.7	92	5.04	14.0	even	ok
	93	C22H45N2O16	593.2764	0.3	76.0	93	16.78	2.0	even	ok
	94	C19H37N12O10	593.2750	-2.0	76.1	94	9.82	8.0	even	ok
	95	C21H54O12PS2	593.2789	4.5	77.2	95	3.86	-4.0	even	ok
	96	C20H43N8NaO9P	593.2783	3.5	78.1	96	4.94	4.0	even	ok
	97	C18H44N12NaO3P2S	593.2747	-2.5	78.1	97	7.69	4.0	even	ok
	98	C16H39N18OP2S	593.2745	-2.9	78.5	98	6.25	8.0	even	ok
	99	C18H38N14O7P	593.2780	3.0	78.9	99	5.88	8.0	even	ok
	100	C21H52N2NaO9P2S	593.2761	-0.2	79.2	100	15.09	-2.0	even	ok
	101	C19H47N8O7P2S	593.2758	-0.7	79.4	101	13.23	2.0	even	ok
	102	C16H37N18O3S2	593.2732	-5.1	79.9	102	2.03	8.0	even	ok
	103	C17H43N14NaO2PS2	593.2765	0.4	80.9	103	13.17	4.0	even	ok
	104	C16H47N10NaO6PS2	593.2751	-1.8	82.7	104	10.10	-1.0	even	ok
	105	C18H49N4O13S2	593.2732	-5.1	84.1	105	2.16	-3.0	even	ok
	106	C17H50N6O10PS2	593.2762	-0.0	84.7	106	15.68	-3.0	even	ok
	107	C19H55NaO12PS2	593.2765	0.5	85.0	107	13.86	-7.0	even	ok
	108	C15H38N16NaO6S	593.2773	1.9	86.8	108	6.65	5.0	even	ok
	109	C20H46N2NaO16	593.2740	-3.8	86.9	109	2.98	-1.0	even	ok
	110	C18H34N16NaO6	593.2739	-3.8	87.2	110	2.19	10.0	even	ok
	111	C13H33N22O4S	593.2770	1.4	87.2	111	7.66	9.0	even	ok
	112	C18H41N8O14	593.2737	-4.3	87.6	112	2.29	3.0	even	ok
	113	C18H46N6NaO12S	593.2787	4.1	87.9	113	2.40	-1.0	even	ok
	114	C16H41N12O10S	593.2784	3.7	88.2	114	2.99	3.0	even	ok

Compound Spectrum SmartFormula Report

Meas. m/z	#	Ion Formula	m/z	err [ppm]	mSigma	# mSigma	Score	rdb	e⁻ Conf	N-Rule
	115	C16H29N22O4	593.2737	-4.3	88.3	115	1.65	14.0	even	ok
	116	C19H47N4NaO13P	593.2769	1.2	89.5	116	7.28	-1.0	even	ok
	117	C17H48N8NaO7P2S	593.2734	-4.7	89.9	117	1.64	-1.0	even	ok
	118	C20H50O17P	593.2780	3.0	90.1	118	3.64	-3.0	even	ok
	119	C17H42N10O11P	593.2767	0.8	90.3	119	8.09	3.0	even	ok
	120	C17H35N18NaO3P	593.2769	1.2	90.6	120	5.24	10.0	even	ok
	121	C18H51N4O11P2S	593.2745	-2.9	91.2	121	3.68	-3.0	even	ok
	122	C15H30N24OP	593.2767	0.8	91.7	122	5.72	14.0	even	ok
	123	C12H30N26NaS	593.2760	-0.4	93.5	123	7.77	11.0	even	ok
	124	C15H38N20PS2	593.2762	-0.0	98.3	124	6.83	8.0	even	ok
	125	C14H42N12NaO10S	593.2760	-0.4	98.6	125	6.15	0.0	even	ok
	126	C17H50N2NaO16S	593.2773	1.9	99.8	126	3.69	-6.0	even	ok
	127	C15H45N8O14S	593.2770	1.4	100.0	127	4.27	-2.0	even	ok

Cmpd 7, 13.8 min

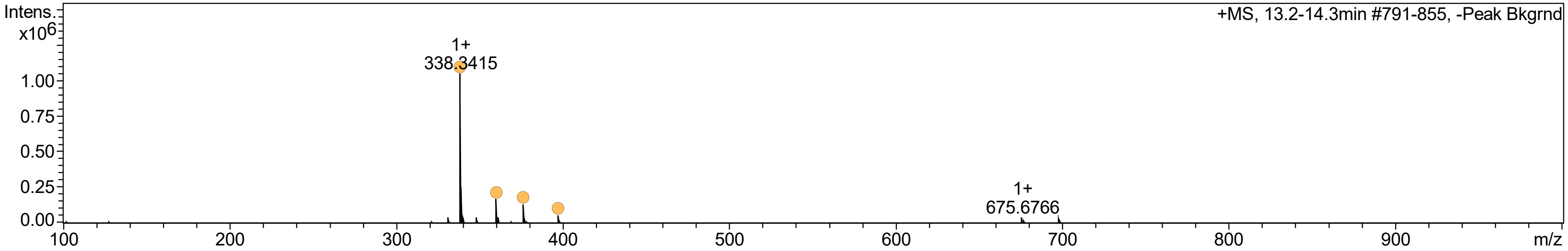

Meas. m/z	#	Ion Formula	m/z	err [ppm]	mSigma	# mSigma	Score	rdb	e⁻ Conf	N-Rule
338.3415	1	C22H44NO	338.3417	0.7	1.9	1	100.00	2.0	even	ok
	2	C16H45N5P	338.3407	-2.3	28.3	2	45.57	-2.0	even	ok
	3	C17H49NNaOS	338.3427	3.6	28.4	3	47.30	-6.0	even	ok
360.3239	1	C19H42N3O3	360.3221	-5.0	3.0	1	46.06	1.0	even	ok
	2	C18H43N5P	360.3251	3.3	6.6	2	64.41	1.0	even	ok
	3	C22H43NNaO	360.3237	-0.5	11.3	3	100.00	2.0	even	ok
	4	C16H44N5NaP	360.3227	-3.4	19.0	4	50.27	-2.0	even	ok
	5	C17H47NO4P	360.3237	-0.4	19.2	5	86.65	-4.0	even	ok
	6	C16H46N3O3S	360.3254	4.4	25.7	6	45.67	-4.0	even	ok
376.2979	1	C19H42N3O2S	376.2992	3.6	2.9	1	100.00	1.0	even	ok
	2	C17H43N3NaO2S	376.2968	-2.8	13.4	2	99.22	-2.0	even	ok
	3	C15H38N9S	376.2965	-3.5	14.5	3	81.85	2.0	even	ok
	4	C20H43NO3P	376.2975	-1.0	26.0	4	81.20	1.0	even	ok
	5	C25H39NNa	376.2975	-1.1	33.6	5	66.42	7.0	even	ok
	6	C17H45N3NaP2	376.2981	0.6	37.8	6	64.67	-2.0	even	ok
397.4157	1	C25H53N2O	397.4152	-1.0	1.1	1	100.00	1.0	even	ok

Compound Spectrum SmartFormula Report

Meas. m/z	#	Ion Formula	m/z	err [ppm]	mSigma	# mSigma	Score	rdb	e⁻ Conf	N-Rule
	2	C23H58O2P	397.4169	3.1	17.5	2	47.47	-4.0	even	ok
	3	C20H58N2NaOS	397.4162	1.4	29.2	3	71.21	-7.0	even	ok
	4	C21H59NaO2P	397.4145	-2.9	30.2	4	37.17	-7.0	even	ok
	5	C19H54N6P	397.4142	-3.6	31.5	5	30.26	-3.0	even	ok

Cmpd 8, 17.2 min

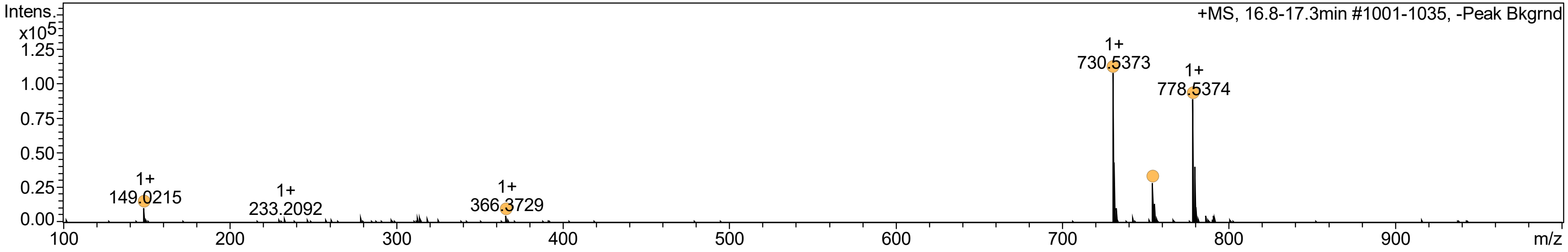

Meas. m/z	#	Ion Formula	m/z	err [ppm]	mSigma	# mSigma	Score	rdb	e⁻ Conf	N-Rule
149.0215	1	C6H6NaO3	149.0209	-4.0	31.4	1	100.00	4.0	even	ok
366.3729	1	C24H48NO	366.3730	0.3	40.6	1	100.00	2.0	even	ok
	2	C19H53NNaOS	366.3740	3.0	68.0	2	34.22	-6.0	even	ok
	3	C18H49N5P	366.3720	-2.5	70.5	3	25.98	-2.0	even	ok
730.5373	1	C35H77N3O10P	730.5341	-4.4	2.5	1	12.87	0.0	even	ok
	2	C32H68N13O6	730.5410	5.0	5.5	2	7.31	6.0	even	ok
	3	C34H78N5O7P2	730.5371	-0.3	7.0	3	100.00	0.0	even	ok
	4	C37H79N3NaO5P2	730.5387	1.9	8.5	4	51.46	1.0	even	ok
	5	C35H74N9O3P2	730.5384	1.5	9.1	5	60.52	5.0	even	ok
	6	C31H65N17NaO2	730.5399	3.6	9.4	6	19.91	8.0	even	ok
	7	C36H73N7O6P	730.5354	-2.6	9.8	7	36.40	5.0	even	ok
	8	C33H75N9NaO3P2	730.5360	-1.8	10.1	8	53.89	2.0	even	ok
	9	C38H78NNaO8P	730.5357	-2.2	10.4	9	43.88	1.0	even	ok
	10	C29H60N23	730.5397	3.2	11.8	10	24.21	12.0	even	ok
	11	C35H70N11NaO2P	730.5344	-4.0	11.8	11	14.22	7.0	even	ok
	12	C30H69N17PS	730.5375	0.2	12.1	12	93.42	6.0	even	ok
	13	C31H70N15OP2	730.5358	-2.1	12.5	13	42.92	6.0	even	ok
	14	C33H65N17P	730.5341	-4.4	12.5	14	10.64	11.0	even	ok
	15	C33H77N3NaO12	730.5399	3.6	12.9	15	18.45	-3.0	even	ok
	16	C31H72N9O10	730.5397	3.2	14.1	16	22.92	1.0	even	ok
	17	C32H74N11NaO2PS	730.5377	0.6	14.4	17	78.83	2.0	even	ok
	18	C28H70N17NaPS	730.5351	-3.1	14.8	18	24.59	3.0	even	ok
	19	C30H69N13NaO6	730.5386	1.8	15.4	19	48.64	3.0	even	ok
	20	C31H68N15O3S	730.5345	-3.9	16.2	20	14.35	6.0	even	ok
	21	C33H82NO11P2	730.5358	-2.1	17.0	21	39.51	-5.0	even	ok

Compound Spectrum SmartFormula Report

Meas. m/z	#	Ion Formula	m/z	err [ppm]	mSigma	# mSigma	Score	rdb	e⁻ Conf	N-Rule
	22	C28H64N19O4	730.5383	1.4	17.3	22	55.26	7.0	even	ok
	23	C28H74N15OP2S	730.5391	2.5	17.6	23	32.65	1.0	even	ok
	24	C32H79N5NaO7P2	730.5347	-3.6	17.9	24	16.85	-3.0	even	ok
	25	C29H73N13O4PS	730.5361	-1.6	17.9	25	49.13	1.0	even	ok
	26	C30H79N9NaO3P2S	730.5394	2.9	18.2	26	26.09	-3.0	even	ok
	27	C33H77N7O6PS	730.5388	2.1	18.5	27	39.64	0.0	even	ok
	28	C33H73N9NaO5S	730.5348	-3.5	18.6	28	17.58	2.0	even	ok
	29	C31H78N7NaO6PS	730.5364	-1.2	19.2	29	56.27	-3.0	even	ok
	30	C30H74N11O5P2	730.5344	-4.0	19.7	30	12.56	1.0	even	ok
	31	C27H61N23Na	730.5372	-0.1	19.7	31	83.18	9.0	even	ok
	32	C38H75N7NaOP2	730.5401	3.8	19.9	32	14.52	6.0	even	ok
	33	C34H76N5O9S	730.5358	-2.0	20.2	33	48.22	0.0	even	ok
	34	C35H82NNaO8PS	730.5391	2.4	20.8	34	31.09	-4.0	even	ok
	35	C34H73N11O2PS	730.5402	3.9	21.1	35	12.88	5.0	even	ok
	36	C31H82N5O7P2S	730.5405	4.3	21.4	36	9.42	-5.0	even	ok
	37	C37H69N11O2P	730.5368	-0.7	21.5	37	64.90	10.0	even	ok
	38	C40H77NO8P	730.5381	1.1	22.0	38	55.47	4.0	even	ok
	39	C39H74N5NaO4P	730.5371	-0.3	22.2	39	72.85	6.0	even	ok
	40	C33H80NO13S	730.5345	-3.9	22.5	40	15.85	-5.0	even	ok
	41	C32H81N3O10PS	730.5375	0.2	22.8	41	74.50	-5.0	even	ok
	42	C34H69N13NaOS	730.5361	-1.7	23.0	42	43.09	7.0	even	ok
	43	C36H78N5NaO4PS	730.5404	4.3	23.5	43	9.33	1.0	even	ok
	44	C24H65N23NaS	730.5406	4.5	23.9	44	7.65	4.0	even	ok
	45	C38H68N9O5	730.5338	-4.8	24.2	45	6.06	10.0	even	ok
	46	C40H73N3NaO7	730.5341	-4.4	25.2	46	7.94	6.0	even	ok
	47	C30H76N5O14	730.5383	1.4	25.4	47	46.09	-4.0	even	ok
	48	C37H77N3NaO7S	730.5374	0.2	25.4	48	89.16	1.0	even	ok
	49	C27H74N13NaO4PS	730.5337	-4.9	25.6	49	5.46	-2.0	even	ok
	50	C29H73N9NaO10	730.5373	-0.1	25.6	50	73.26	-2.0	even	ok
	51	C35H72N9O5S	730.5372	-0.2	26.0	51	69.78	5.0	even	ok
	52	C26H75N15NaOP2S	730.5367	-0.8	26.9	52	55.59	-2.0	even	ok
	53	C27H68N15O8	730.5370	-0.5	27.0	53	62.84	2.0	even	ok
	54	C28H77N9O8PS	730.5348	-3.4	27.9	54	14.76	-4.0	even	ok
	55	C26H65N19NaO4	730.5359	-1.9	28.2	55	34.06	4.0	even	ok
	56	C30H82N3NaO10PS	730.5351	-3.1	28.4	56	18.41	-8.0	even	ok
	57	C27H78N11O5P2S	730.5378	0.6	28.6	57	56.77	-4.0	even	ok
	58	C29H83N5NaO7P2S	730.5381	1.0	28.8	58	49.12	-8.0	even	ok
	59	C28H74N11NaO7P	730.5403	4.0	29.4	59	9.73	-2.0	even	ok
	60	C24H60N25O2	730.5356	-2.3	30.0	60	27.03	8.0	even	ok
	61	C27H73N17PS2	730.5408	4.8	30.6	61	6.47	1.0	even	ok
	62	C26H69N17O5P	730.5400	3.6	31.0	62	12.13	2.0	even	ok
	63	C30H86NO11P2S	730.5391	2.5	31.3	63	23.76	-10.0	even	ok
	64	C38H78N5O2P2S	730.5346	-3.7	31.3	64	11.40	4.0	even	ok
	65	C31H78N11P2S2	730.5353	-2.8	32.1	65	24.81	0.0	even	ok
	66	C25H66N21NaOP	730.5389	2.2	32.4	66	27.14	4.0	even	ok
	67	C25H74N17NaPS2	730.5384	1.5	32.8	67	45.33	-2.0	even	ok

Compound Spectrum SmartFormula Report

Meas. m/z	#	Ion Formula	m/z	err [ppm]	mSigma	# mSigma	Score	rdb	e⁻ Conf	N-Rule
	68	C28H72N15O3S2	730.5378	0.7	33.4	68	61.12	1.0	even	ok
	69	C36H68N13OS	730.5385	1.6	33.6	69	34.13	10.0	even	ok
	70	C41H73N5O4P	730.5395	2.9	33.6	70	17.36	9.0	even	ok
	71	C40H70N9NaP	730.5384	1.5	34.0	71	35.90	11.0	even	ok
	72	C30H82N7O4P2S2	730.5339	-4.6	34.2	72	6.99	-5.0	even	ok
	73	C39H76N3O7S	730.5398	3.5	34.2	73	15.56	4.0	even	ok
	74	C33H83N5NaO2P2S2	730.5356	-2.4	34.2	74	28.93	-4.0	even	ok
	75	C26H73N15NaO3S2	730.5354	-2.6	34.4	75	26.44	-2.0	even	ok
	76	C31H73N13NaOS2	730.5395	2.9	34.4	76	21.30	2.0	even	ok
	77	C30H77N9NaO5S2	730.5381	1.1	35.4	77	50.42	-3.0	even	ok
	78	C23H69N19NaO4S	730.5393	2.7	35.6	78	19.06	-1.0	even	ok
	79	C38H73N7NaO3S	730.5388	2.0	35.7	79	27.06	6.0	even	ok
	80	C39H64N13O	730.5351	-3.0	35.9	80	15.98	15.0	even	ok
	81	C26H77N13O4PS2	730.5395	3.0	36.0	81	19.83	-4.0	even	ok
	82	C32H87NNaO6P2S2	730.5342	-4.2	36.1	82	8.87	-9.0	even	ok
	83	C24H68N21OS2	730.5352	-2.9	36.4	83	20.24	2.0	even	ok
	84	C28H77N5NaO14	730.5359	-1.9	36.7	84	27.72	-7.0	even	ok
	85	C29H80NO18	730.5370	-0.4	36.9	85	49.44	-9.0	even	ok
	86	C41H69N7NaO3	730.5354	-2.6	36.9	86	19.34	11.0	even	ok
	87	C42H72N3O7	730.5365	-1.1	37.0	87	38.21	9.0	even	ok
	88	C24H72N15O8S	730.5404	4.2	37.1	88	7.25	-3.0	even	ok
	89	C26H77N9NaO10S	730.5406	4.5	37.2	89	5.47	-7.0	even	ok
	90	C28H82N7NaO6PS2	730.5398	3.4	37.4	90	15.17	-8.0	even	ok
	91	C27H76N11O7S2	730.5365	-1.1	37.8	91	47.57	-4.0	even	ok
	92	C26H72N11O12	730.5356	-2.3	37.9	92	22.29	-3.0	even	ok
	93	C34H86NO6P2S2	730.5366	-0.9	37.9	93	50.52	-6.0	even	ok
	94	C32H76N9O5S2	730.5405	4.4	38.0	94	7.38	0.0	even	ok
	95	C25H69N15NaO8	730.5346	-3.7	38.4	95	9.31	-1.0	even	ok
	96	C31H80N5O9S2	730.5392	2.6	39.1	96	23.16	-5.0	even	ok
	97	C35H82N5O2P2S2	730.5380	0.9	39.1	97	49.60	-1.0	even	ok
	98	C29H81N5NaO9S2	730.5368	-0.7	39.4	98	52.63	-8.0	even	ok
	99	C23H64N21O6	730.5343	-4.1	39.8	99	6.87	3.0	even	ok
	100	C28H81N3O15P	730.5400	3.7	39.8	100	9.54	-9.0	even	ok
	101	C27H78N7NaO11P	730.5389	2.2	40.0	101	22.07	-7.0	even	ok
	102	C35H78N7NaOPS2	730.5339	-4.7	40.1	102	5.76	1.0	even	ok
	103	C34H81N3NaO7S2	730.5408	4.8	40.2	103	5.19	-4.0	even	ok
	104	C29H85N3O10PS2	730.5409	4.8	40.8	104	4.91	-10.0	even	ok
	105	C25H73N13O9P	730.5386	1.8	41.3	105	25.59	-3.0	even	ok
	106	C24H70N17NaO5P	730.5376	0.3	42.2	106	44.21	-1.0	even	ok
	107	C41H79N3NaP2S	730.5362	-1.5	42.5	107	28.30	5.0	even	ok
	108	C30H84NO13S2	730.5379	0.7	42.9	108	47.18	-10.0	even	ok
	109	C36H81N3O5PS2	730.5350	-3.2	43.4	109	14.37	-1.0	even	ok
	110	C22H65N23O3P	730.5373	-0.0	43.7	110	46.46	3.0	even	ok
	111	C42H69N9P	730.5408	4.8	45.4	111	3.62	14.0	even	ok
	112	C38H83N3NaP2S2	730.5396	3.1	45.4	112	14.33	0.0	even	ok
	113	C37H77N7OPS2	730.5363	-1.4	47.7	113	32.31	4.0	even	ok

Compound Spectrum SmartFormula Report

Meas. m/z	#	Ion Formula	m/z	err [ppm]	mSigma	# mSigma	Score	rdb	e¯ Conf	N-Rule
	114	C27H81NNaO18	730.5346	-3.7	48.1	114	7.14	-12.0	even	ok
	115	C43H78N3P2S	730.5386	1.8	48.6	115	26.16	8.0	even	ok
	116	C43H68N7O3	730.5378	0.7	48.6	116	32.66	14.0	even	ok
	117	C39H82NNaO3PS2	730.5366	-1.0	49.7	117	35.42	0.0	even	ok
	118	C45H73NNaO5	730.5381	1.1	49.8	118	27.32	10.0	even	ok
	119	C44H77NO3PS	730.5356	-2.3	52.7	119	17.92	8.0	even	ok
	120	C46H74N3P2	730.5352	-2.8	57.3	120	9.35	13.0	even	ok
	121	C39H72N9S2	730.5347	-3.6	58.4	121	6.90	9.0	even	ok
	122	C41H81NO3PS2	730.5390	2.3	58.4	122	14.89	3.0	even	ok
	123	C41H77N3NaO2S2	730.5349	-3.2	60.4	123	8.23	5.0	even	ok
	124	C46H69N5NaO	730.5394	2.9	61.5	124	7.78	15.0	even	ok
	125	C47H72NO5	730.5405	4.4	61.8	125	2.95	13.0	even	ok
	126	C46H72N3O2S	730.5340	-4.6	66.1	126	2.72	13.0	even	ok
	127	C43H76N3O2S2	730.5373	0.0	70.1	127	24.43	8.0	even	ok
	128	C49H73NNaS	730.5356	-2.4	77.4	128	7.27	14.0	even	ok
	129	C46H77NNaS2	730.5390	2.3	80.2	129	6.86	9.0	even	ok
	130	C51H72NS	730.5380	0.9	88.6	130	8.60	17.0	even	ok
754.5370	1	C42H70N9NaP	754.5384	1.9	8.1	1	52.00	13.0	even	ok
	2	C39H69N11O2P	754.5368	-0.3	10.2	2	97.20	12.0	even	ok
	3	C41H64N13O	754.5351	-2.4	10.7	3	37.17	17.0	even	ok
	4	C41H74N5NaO4P	754.5371	0.1	11.0	4	100.00	8.0	even	ok
	5	C40H68N9O5	754.5338	-4.2	11.0	5	11.56	12.0	even	ok
	6	C40H75N7NaOP2	754.5401	4.1	11.3	6	12.73	8.0	even	ok
	7	C43H73N5O4P	754.5395	3.3	11.8	7	21.66	11.0	even	ok
	8	C42H73N3NaO7	754.5341	-3.8	12.8	8	14.71	8.0	even	ok
	9	C43H69N7NaO3	754.5354	-2.1	13.2	9	43.14	13.0	even	ok
	10	C42H77NO8P	754.5381	1.5	14.4	10	54.61	6.0	even	ok
	11	C44H72N3O7	754.5365	-0.7	16.7	11	74.43	11.0	even	ok
	12	C44H69N9P	754.5408	5.1	18.9	12	4.90	16.0	even	ok
	13	C37H70N11NaO2P	754.5344	-3.4	20.2	13	16.72	9.0	even	ok
	14	C35H65N17P	754.5341	-3.8	21.0	14	12.73	13.0	even	ok
	15	C40H78NNaO8P	754.5357	-1.7	21.4	15	44.71	3.0	even	ok
	16	C38H73N7O6P	754.5354	-2.0	21.4	16	37.33	7.0	even	ok
	17	C39H79N3NaO5P2	754.5387	2.3	23.0	17	31.11	3.0	even	ok
	18	C37H74N9O3P2	754.5384	1.9	23.5	18	37.22	7.0	even	ok
	19	C45H68N7O3	754.5378	1.1	23.8	19	53.61	16.0	even	ok
	20	C47H73NNaO5	754.5381	1.5	26.1	20	43.43	12.0	even	ok
	21	C40H73N7NaO3S	754.5388	2.4	28.5	21	32.71	8.0	even	ok
	22	C43H79N3NaP2S	754.5362	-1.0	28.6	22	62.59	7.0	even	ok
	23	C38H68N13OS	754.5385	2.0	29.4	23	31.07	12.0	even	ok
	24	C36H69N13NaOS	754.5361	-1.2	29.6	24	45.93	9.0	even	ok
	25	C40H78N5O2P2S	754.5346	-3.1	30.0	25	16.17	6.0	even	ok
	26	C37H72N9O5S	754.5372	0.3	30.0	26	79.61	7.0	even	ok
	27	C48H74N3P2	754.5352	-2.3	31.1	27	26.05	15.0	even	ok
	28	C39H82NO6P2S	754.5333	-4.9	31.4	28	5.27	1.0	even	ok
	29	C36H73N11O2PS	754.5402	4.2	31.4	29	7.44	7.0	even	ok

Compound Spectrum SmartFormula Report

Meas. m/z	#	Ion Formula	m/z	err [ppm]	mSigma	# mSigma	Score	rdb	e⁻ Conf	N-Rule
	30	C39H77N3NaO7S	754.5374	0.6	31.5	30	67.52	3.0	even	ok
	31	C33H65N17NaO2	754.5399	3.9	31.7	31	9.15	10.0	even	ok
	32	C41H76N3O7S	754.5398	3.8	32.1	32	12.29	6.0	even	ok
	33	C31H60N23	754.5397	3.6	32.8	33	11.58	14.0	even	ok
	34	C44H78NNaO3PS	754.5332	-5.0	32.9	34	4.87	7.0	even	ok
	35	C38H78N5NaO4PS	754.5404	4.6	32.9	35	5.36	3.0	even	ok
	36	C37H77N3O10P	754.5341	-3.8	33.0	36	9.71	2.0	even	ok
	37	C35H75N9NaO3P2	754.5360	-1.2	34.8	37	38.95	4.0	even	ok
	38	C36H78N5O7P2	754.5371	0.2	35.3	38	57.31	2.0	even	ok
	39	C45H78N3P2S	754.5386	2.2	35.4	39	30.82	10.0	even	ok
	40	C33H70N15OP2	754.5358	-1.6	35.8	40	32.22	8.0	even	ok
	41	C48H69N5NaO	754.5394	3.3	35.8	41	13.03	17.0	even	ok
	42	C32H69N17PS	754.5375	0.7	36.3	42	47.22	8.0	even	ok
	43	C36H76N5O9S	754.5358	-1.5	36.4	43	41.39	2.0	even	ok
	44	C33H68N15O3S	754.5345	-3.3	36.4	44	12.41	8.0	even	ok
	45	C37H82NNaO8PS	754.5391	2.8	36.6	45	21.01	-2.0	even	ok
	46	C34H74N11NaO2PS	754.5377	1.0	36.9	46	40.31	4.0	even	ok
	47	C35H73N9NaO5S	754.5348	-2.9	37.4	47	15.27	4.0	even	ok
	48	C49H72NO5	754.5405	4.7	37.7	48	4.40	15.0	even	ok
	49	C35H77N7O6PS	754.5388	2.4	39.9	49	19.04	2.0	even	ok
	50	C46H77NO3PS	754.5356	-1.8	40.0	50	33.32	10.0	even	ok
	51	C34H77N5NaO9S	754.5334	-4.7	42.1	51	4.74	-1.0	even	ok
	52	C35H77N3NaO12	754.5399	3.9	43.4	52	6.65	-1.0	even	ok
	53	C32H69N13NaO6	754.5386	2.2	43.4	53	20.17	5.0	even	ok
	54	C33H72N9O10	754.5397	3.6	44.0	54	8.49	3.0	even	ok
	55	C30H64N19O4	754.5383	1.8	44.3	55	23.64	9.0	even	ok
	56	C29H61N23Na	754.5372	0.4	44.3	56	41.92	11.0	even	ok
	57	C35H80NO13S	754.5345	-3.3	44.6	57	12.51	-3.0	even	ok
	58	C34H81N3O10PS	754.5375	0.7	44.6	58	46.76	-3.0	even	ok
	59	C30H70N17NaPS	754.5351	-2.5	44.9	59	15.72	5.0	even	ok
	60	C37H78N7NaOPS2	754.5339	-4.1	45.6	60	7.11	3.0	even	ok
	61	C40H83N3NaP2S2	754.5396	3.5	46.1	61	10.72	2.0	even	ok
	62	C33H73N13NaOS2	754.5395	3.3	46.4	62	11.80	4.0	even	ok
	63	C33H78N11P2S2	754.5353	-2.2	46.4	63	22.06	2.0	even	ok
	64	C34H79N5NaO7P2	754.5347	-3.0	46.4	64	11.27	-1.0	even	ok
	65	C32H72N11O7S	754.5331	-5.1	46.4	65	2.45	3.0	even	ok
	66	C37H82N5O2P2S2	754.5380	1.3	46.4	66	34.28	1.0	even	ok
	67	C31H73N13O4PS	754.5361	-1.1	47.0	67	29.38	3.0	even	ok
	68	C35H82NO11P2	754.5358	-1.6	47.0	68	23.73	-3.0	even	ok
	69	C33H78N7NaO6PS	754.5364	-0.7	47.3	69	33.62	-1.0	even	ok
	70	C31H71N15NaOP2	754.5333	-4.8	47.3	70	3.01	5.0	even	ok
	71	C32H74N11O5P2	754.5344	-3.4	47.3	71	8.66	3.0	even	ok
	72	C39H77N7OPS2	754.5363	-0.9	47.6	72	39.66	6.0	even	ok
	73	C35H83N5NaO2P2S2	754.5356	-1.9	47.8	73	25.59	-2.0	even	ok
	74	C30H74N15OP2S	754.5391	2.8	48.0	74	11.95	3.0	even	ok
	75	C32H79N9NaO3P2S	754.5394	3.2	48.1	75	9.48	-1.0	even	ok

Compound Spectrum SmartFormula Report

Meas. m/z	#	Ion Formula	m/z	err [ppm]	mSigma	# mSigma	Score	rdb	e⁻ Conf	N-Rule
	76	C38H81N3O5PS2	754.5350	-2.6	49.3	76	16.19	1.0	even	ok
	77	C41H82NNaO3PS2	754.5366	-0.5	49.8	77	42.58	2.0	even	ok
	78	C34H76N9O5S2	754.5405	4.7	49.8	78	3.76	2.0	even	ok
	79	C33H82N5O7P2S	754.5405	4.6	50.2	79	3.18	-3.0	even	ok
	80	C48H72N3O2S	754.5340	-4.0	50.7	80	6.52	15.0	even	ok
	81	C36H86NO6P2S2	754.5366	-0.5	51.1	81	41.56	-4.0	even	ok
	82	C36H81N3NaO7S2	754.5408	5.1	51.3	82	2.62	-2.0	even	ok
	83	C40H76N5O4S2	754.5333	-4.8	51.4	83	3.24	6.0	even	ok
	84	C30H72N15O3S2	754.5378	1.2	51.5	84	31.46	3.0	even	ok
	85	C32H77N9NaO5S2	754.5381	1.5	52.6	85	25.87	-1.0	even	ok
	86	C41H72N9S2	754.5347	-3.1	52.8	86	11.31	11.0	even	ok
	87	C32H82N7O4P2S2	754.5339	-4.0	53.4	87	5.83	-3.0	even	ok
	88	C43H81NO3PS2	754.5390	2.7	54.4	88	13.51	5.0	even	ok
	89	C34H87NNaO6P2S2	754.5342	-3.6	54.4	89	7.34	-7.0	even	ok
	90	C26H65N23NaS	754.5406	4.8	54.9	90	2.33	6.0	even	ok
	91	C31H73N9NaO10	754.5373	0.4	55.1	91	30.13	0.0	even	ok
	92	C43H77N3NaO2S2	754.5349	-2.7	55.1	92	13.13	7.0	even	ok
	93	C32H76N5O14	754.5383	1.8	55.7	93	16.60	-2.0	even	ok
	94	C28H65N19NaO4	754.5359	-1.4	55.7	94	19.80	6.0	even	ok
	95	C33H80N5O9S2	754.5392	2.9	55.8	95	11.06	-3.0	even	ok
	96	C29H68N15O8	754.5370	0.0	55.9	96	32.99	4.0	even	ok
	97	C26H68N21OS2	754.5352	-2.4	55.9	97	15.10	4.0	even	ok
	98	C29H74N13NaO4PS	754.5337	-4.3	56.3	98	3.41	0.0	even	ok
	99	C27H69N19O2PS	754.5334	-4.7	56.4	99	2.53	4.0	even	ok
	100	C27H74N17NaPS2	754.5384	1.9	56.5	100	18.87	0.0	even	ok
	101	C28H73N15NaO3S2	754.5354	-2.0	56.6	101	17.93	0.0	even	ok
	102	C26H60N25O2	754.5356	-1.8	56.8	102	16.12	10.0	even	ok
	103	C28H75N15NaOP2S	754.5367	-0.3	57.9	103	27.80	0.0	even	ok
	104	C30H74N11NaO7P	754.5403	4.3	57.9	104	3.14	0.0	even	ok
	105	C33H83NNaO11P2	754.5334	-4.8	58.1	105	2.17	-6.0	even	ok
	106	C30H77N9O8PS	754.5348	-2.9	58.1	106	8.49	-2.0	even	ok
	107	C32H82N3NaO10PS	754.5351	-2.5	58.2	107	10.48	-6.0	even	ok
	108	C28H69N17O5P	754.5400	4.0	58.9	108	4.01	4.0	even	ok
	109	C28H77N13O4PS2	754.5395	3.4	59.0	109	7.70	-2.0	even	ok
	110	C27H66N21NaOP	754.5389	2.6	59.0	110	10.00	6.0	even	ok
	111	C29H76N11O7S2	754.5365	-0.6	59.3	111	30.19	-2.0	even	ok
	112	C31H83N5NaO7P2S	754.5381	1.4	59.4	112	17.27	-6.0	even	ok
	113	C29H78N11O5P2S	754.5378	1.1	59.4	113	20.17	-2.0	even	ok
	114	C30H82N7NaO6PS2	754.5398	3.7	59.7	114	5.84	-6.0	even	ok
	115	C31H81N5NaO9S2	754.5368	-0.2	60.2	115	33.26	-6.0	even	ok
	116	C51H73NNaS	754.5356	-1.8	60.4	116	17.42	16.0	even	ok
	117	C32H86NO11P2S	754.5391	2.9	61.3	117	7.75	-8.0	even	ok
	118	C45H76N3O2S2	754.5373	0.5	62.0	118	28.72	10.0	even	ok
	119	C32H84NO13S2	754.5379	1.2	63.1	119	21.38	-8.0	even	ok
	120	C25H72N17O5S2	754.5338	-4.2	65.0	120	3.51	-1.0	even	ok
	121	C27H77N11NaO7S2	754.5341	-3.8	65.5	121	4.52	-5.0	even	ok

Compound Spectrum SmartFormula Report

Meas. m/z	#	Ion Formula	m/z	err [ppm]	mSigma	# mSigma	Score	rdb	e¯ Conf	N-Rule
	122	C26H78N13NaO4PS2	754.5371	0.2	65.8	122	28.10	-5.0	even	ok
	123	C25H69N19NaO4S	754.5393	3.1	66.5	123	5.71	1.0	even	ok
	124	C30H77N5NaO14	754.5359	-1.4	66.7	124	13.70	-5.0	even	ok
	125	C23H64N25O2S	754.5390	2.7	66.9	125	7.04	5.0	even	ok
	126	C27H69N15NaO8	754.5346	-3.2	67.2	126	5.17	1.0	even	ok
	127	C31H80NO18	754.5370	0.0	67.4	127	22.15	-7.0	even	ok
	128	C28H72N11O12	754.5356	-1.8	67.5	128	11.24	-1.0	even	ok
	129	C28H77N9NaO10S	754.5406	4.8	67.9	129	1.48	-5.0	even	ok
	130	C28H80N7O11S2	754.5352	-2.4	68.0	130	10.05	-7.0	even	ok
	131	C26H72N15O8S	754.5404	4.5	68.0	131	1.99	-1.0	even	ok
	132	C27H81N9O8PS2	754.5382	1.6	68.0	132	15.02	-7.0	even	ok
	133	C25H64N21O6	754.5343	-3.6	68.2	133	3.91	5.0	even	ok
	134	C24H61N25NaO2	754.5332	-5.0	68.3	134	1.32	7.0	even	ok
	135	C29H86N3NaO10PS2	754.5384	1.9	68.6	135	12.34	-11.0	even	ok
	136	C30H85NNaO13S2	754.5355	-2.0	68.6	136	11.92	-11.0	even	ok
	137	C48H77NNaS2	754.5390	2.6	69.4	137	8.27	11.0	even	ok
	138	C29H81N5O12PS	754.5335	-4.7	69.5	138	1.63	-7.0	even	ok
	139	C29H78N7NaO11P	754.5389	2.6	69.5	139	6.86	-5.0	even	ok
	140	C27H79N11NaO5P2S	754.5354	-2.1	69.6	140	8.76	-5.0	even	ok
	141	C25H74N17O3P2S	754.5351	-2.5	69.9	141	7.09	-1.0	even	ok
	142	C30H81N3O15P	754.5400	4.0	69.9	142	2.69	-7.0	even	ok
	143	C26H70N17NaO5P	754.5376	0.8	70.3	143	15.38	1.0	even	ok
	144	C27H73N13O9P	754.5386	2.2	70.4	144	8.12	-1.0	even	ok
	145	C30H87NNaO11P2S	754.5367	-0.3	70.8	145	17.73	-11.0	even	ok
	146	C28H82N7O9P2S	754.5364	-0.7	71.0	146	15.49	-7.0	even	ok
	147	C53H72NS	754.5380	1.4	71.1	147	14.81	19.0	even	ok
	148	C24H65N23O3P	754.5373	0.4	71.4	148	16.85	5.0	even	ok
	149	C28H84NO18S	754.5404	4.5	72.6	149	2.08	-12.0	even	ok
	150	C25H71N19NaO2P2	754.5406	4.7	73.6	150	1.30	1.0	even	ok
	151	C56H68N	754.5346	-3.1	76.6	151	3.79	24.0	even	ok
	152	C29H81NNaO18	754.5346	-3.2	78.4	152	3.41	-10.0	even	ok
	153	C26H73N11NaO12	754.5332	-5.0	78.7	153	0.89	-4.0	even	ok
	154	C27H76N7O16	754.5343	-3.5	79.1	154	2.60	-6.0	even	ok
	155	C27H81N5NaO14S	754.5393	3.1	79.4	155	3.47	-10.0	even	ok
	156	C28H82N3NaO15P	754.5376	0.8	81.0	156	10.07	-10.0	even	ok
	157	C25H74N13NaO9P	754.5362	-1.0	81.7	157	9.12	-4.0	even	ok
	158	C26H77N9O13P	754.5373	0.4	81.9	158	11.10	-6.0	even	ok
	159	C23H69N19O7P	754.5359	-1.4	82.7	159	7.50	0.0	even	ok
	160	C22H66N23NaO3P	754.5349	-2.8	82.8	160	3.63	2.0	even	ok
	161	C23H66N25P2	754.5403	4.4	86.3	161	0.78	5.0	even	ok
778.5374	1	C34H69N17PS	778.5375	0.2	6.4	1	98.86	10.0	even	ok
	2	C36H74N11NaO2PS	778.5377	0.5	8.3	2	84.44	6.0	even	ok
	3	C39H77N3O10P	778.5341	-4.2	8.9	3	10.42	4.0	even	ok
	4	C35H68N15O3S	778.5345	-3.7	9.7	4	14.75	10.0	even	ok
	5	C42H78NNaO8P	778.5357	-2.1	9.8	5	40.68	5.0	even	ok
	6	C38H73N7NaO8	778.5413	5.1	10.0	6	4.78	6.0	even	ok

Compound Spectrum SmartFormula Report

Meas. m/z	#	Ion Formula	m/z	err [ppm]	mSigma	# mSigma	Score	rdb	e¯ Conf	N-Rule
	7	C40H73N7O6P	778.5354	-2.4	10.8	7	32.67	9.0	even	ok
	8	C37H73N9NaO5S	778.5348	-3.3	11.0	8	23.27	6.0	even	ok
	9	C41H79N3NaO5P2	778.5387	1.8	11.1	9	47.05	5.0	even	ok
	10	C37H77N7O6PS	778.5388	1.9	11.2	10	55.03	4.0	even	ok
	11	C36H68N13O6	778.5410	4.7	12.5	11	6.27	10.0	even	ok
	12	C39H74N9O3P2	778.5384	1.4	13.0	12	53.88	9.0	even	ok
	13	C39H82NNaO8PS	778.5391	2.2	13.2	13	43.73	0.0	even	ok
	14	C38H78N5O7P2	778.5371	-0.3	13.8	14	81.32	4.0	even	ok
	15	C32H70N17NaPS	778.5351	-2.9	14.4	15	22.60	7.0	even	ok
	16	C39H70N11NaO2P	778.5344	-3.8	14.4	16	12.26	11.0	even	ok
	17	C38H76N5O9S	778.5358	-2.0	14.7	17	49.56	4.0	even	ok
	18	C38H73N11O2PS	778.5402	3.6	14.9	18	14.22	9.0	even	ok
	19	C36H77N5NaO9S	778.5334	-5.0	15.1	19	5.44	1.0	even	ok
	20	C40H78N5NaO4PS	778.5404	4.0	15.6	20	13.42	5.0	even	ok
	21	C33H73N13O4PS	778.5361	-1.6	16.0	21	46.95	5.0	even	ok
	22	C37H65N17P	778.5341	-4.2	16.1	22	8.97	15.0	even	ok
	23	C35H78N7NaO6PS	778.5364	-1.2	16.3	23	54.89	1.0	even	ok
	24	C35H65N17NaO2	778.5399	3.3	16.4	24	16.85	12.0	even	ok
	25	C38H69N13NaOS	778.5361	-1.6	17.2	25	44.82	11.0	even	ok
	26	C36H81N3O10PS	778.5375	0.2	17.2	26	100.00	-1.0	even	ok
	27	C37H75N9NaO3P2	778.5360	-1.7	17.3	27	42.97	6.0	even	ok
	28	C34H79N9NaO3P2S	778.5394	2.6	17.3	28	25.68	1.0	even	ok
	29	C43H78N3O5P2	778.5411	4.8	17.5	29	4.97	8.0	even	ok
	30	C37H77N3NaO12	778.5399	3.3	17.6	30	16.27	1.0	even	ok
	31	C32H74N15OP2S	778.5391	2.3	17.6	31	31.41	5.0	even	ok
	32	C39H72N9O5S	778.5372	-0.2	17.9	32	96.23	9.0	even	ok
	33	C37H80NO13S	778.5345	-3.7	18.3	33	15.73	-1.0	even	ok
	34	C33H60N23	778.5397	3.0	18.9	34	20.31	16.0	even	ok
	35	C35H82N5O7P2S	778.5405	4.0	19.2	35	9.61	-1.0	even	ok
	36	C44H77NO8P	778.5381	1.0	19.3	36	56.17	8.0	even	ok
	37	C35H72N9O10	778.5397	3.0	19.5	37	19.85	5.0	even	ok
	38	C35H70N15OP2	778.5358	-2.1	19.7	38	33.95	10.0	even	ok
	39	C42H75N7NaOP2	778.5401	3.5	19.8	39	14.10	10.0	even	ok
	40	C41H77N3NaO7S	778.5374	0.1	20.1	40	95.53	5.0	even	ok
	41	C43H74N5NaO4P	778.5371	-0.4	20.6	41	69.56	10.0	even	ok
	42	C41H69N11O2P	778.5368	-0.7	20.7	42	60.90	14.0	even	ok
	43	C34H69N13NaO6	778.5386	1.6	21.8	43	40.81	7.0	even	ok
	44	C37H82NO11P2	778.5358	-2.0	22.0	44	32.53	-1.0	even	ok
	45	C42H68N9O5	778.5338	-4.6	22.1	45	5.72	14.0	even	ok
	46	C44H73N3NaO7	778.5341	-4.2	22.6	46	7.61	10.0	even	ok
	47	C42H78N5O2P2S	778.5346	-3.5	23.4	47	15.60	8.0	even	ok
	48	C36H79N5NaO7P2	778.5347	-3.4	24.0	48	13.44	1.0	even	ok
	49	C32H64N19O4	778.5383	1.2	24.0	49	45.81	11.0	even	ok
	50	C34H82N3NaO10PS	778.5351	-2.9	24.4	50	23.02	-4.0	even	ok
	51	C28H65N23NaS	778.5406	4.2	25.7	51	7.15	8.0	even	ok
	52	C40H68N13OS	778.5385	1.5	25.8	52	49.38	14.0	even	ok

Compound Spectrum SmartFormula Report

Meas. m/z	#	Ion Formula	m/z	err [ppm]	mSigma	# mSigma	Score	rdb	e¯ Conf	N-Rule
	53	C34H74N11O5P2	778.5344	-3.8	26.1	53	9.88	5.0	even	ok
	54	C31H74N13NaO4PS	778.5337	-4.7	26.1	54	4.86	2.0	even	ok
	55	C31H73N17PS2	778.5408	4.5	26.1	55	7.02	5.0	even	ok
	56	C29H69N19O2PS	778.5334	-5.0	26.8	56	3.49	6.0	even	ok
	57	C31H61N23Na	778.5372	-0.1	26.9	57	65.54	13.0	even	ok
	58	C35H78N11P2S2	778.5353	-2.7	26.9	58	25.70	4.0	even	ok
	59	C34H86NO11P2S	778.5391	2.3	27.2	59	31.54	-6.0	even	ok
	60	C32H77N9O8PS	778.5348	-3.3	27.4	60	13.59	0.0	even	ok
	61	C42H73N7NaO3S	778.5388	1.8	27.5	61	39.86	10.0	even	ok
	62	C33H78N11NaO2PS2	778.5411	4.8	27.8	62	4.96	1.0	even	ok
	63	C32H72N15O3S2	778.5378	0.6	28.6	63	65.69	5.0	even	ok
	64	C30H75N15NaOP2S	778.5367	-0.8	28.7	64	49.10	2.0	even	ok
	65	C31H65N21OP	778.5413	5.1	28.8	65	3.11	11.0	even	ok
	66	C33H83N5NaO7P2S	778.5381	0.9	28.9	66	46.89	-4.0	even	ok
	67	C37H83N5NaO2P2S2	778.5356	-2.3	29.0	67	30.12	0.0	even	ok
	68	C35H73N13NaOS2	778.5395	2.7	29.1	68	23.44	6.0	even	ok
	69	C31H78N11O5P2S	778.5378	0.6	29.4	69	53.26	0.0	even	ok
	70	C28H68N21OS2	778.5352	-2.8	29.5	70	22.01	6.0	even	ok
	71	C43H76N3O7S	778.5398	3.2	29.6	71	16.90	8.0	even	ok
	72	C34H76N5O14	778.5383	1.3	29.6	72	39.95	0.0	even	ok
	73	C29H74N17NaPS2	778.5384	1.4	29.6	73	46.96	2.0	even	ok
	74	C34H82N7O4P2S2	778.5339	-4.4	29.6	74	7.09	-1.0	even	ok
	75	C34H77N9NaO5S2	778.5381	1.0	30.5	75	54.55	1.0	even	ok
	76	C30H73N15NaO3S2	778.5354	-2.4	30.6	76	26.56	2.0	even	ok
	77	C33H73N9NaO10	778.5373	-0.1	30.8	77	59.88	2.0	even	ok
	78	C45H73N5O4P	778.5395	2.7	31.2	78	17.81	13.0	even	ok
	79	C36H87NNaO6P2S2	778.5342	-4.0	31.3	79	9.06	-5.0	even	ok
	80	C44H70N9NaP	778.5384	1.3	32.2	80	35.98	15.0	even	ok
	81	C30H77N13O4PS2	778.5395	2.8	32.6	81	20.94	0.0	even	ok
	82	C31H68N15O8	778.5370	-0.5	32.6	82	50.66	6.0	even	ok
	83	C38H86NO6P2S2	778.5366	-0.9	32.8	83	53.20	-2.0	even	ok
	84	C36H76N9O5S2	778.5405	4.1	32.8	84	8.22	4.0	even	ok
	85	C33H77N7O11P	778.5413	5.1	33.6	85	2.73	0.0	even	ok
	86	C32H82N7NaO6PS2	778.5398	3.1	33.7	86	16.17	-4.0	even	ok
	87	C31H76N11O7S2	778.5365	-1.1	33.9	87	48.59	0.0	even	ok
	88	C43H64N13O	778.5351	-2.9	33.9	88	15.37	19.0	even	ok
	89	C46H72N3O7	778.5365	-1.1	34.0	89	38.00	13.0	even	ok
	90	C39H82N5O2P2S2	778.5380	0.8	34.0	90	54.23	3.0	even	ok
	91	C45H79N3NaP2S	778.5362	-1.5	34.1	91	40.74	9.0	even	ok
	92	C35H80N5O9S2	778.5392	2.4	34.2	92	25.38	-1.0	even	ok
	93	C45H69N7NaO3	778.5354	-2.5	34.5	93	18.82	15.0	even	ok
	94	C30H65N19NaO4	778.5359	-1.8	34.5	94	26.73	8.0	even	ok
	95	C31H81N5O12PS	778.5335	-5.0	34.9	95	3.62	-5.0	even	ok
	96	C38H81N3NaO7S2	778.5408	4.5	35.0	96	5.81	0.0	even	ok
	97	C39H78N7NaOPS2	778.5339	-4.4	35.1	97	5.95	5.0	even	ok
	98	C32H74N11NaO7P	778.5403	3.7	35.1	98	8.18	2.0	even	ok

Compound Spectrum SmartFormula Report

Meas. m/z	#	Ion Formula	m/z	err [ppm]	mSigma	# mSigma	Score	rdb	e⁻ Conf	N-Rule
	99	C33H81N5NaO9S2	778.5368	-0.7	35.2	99	54.20	-4.0	even	ok
	100	C28H60N25O2	778.5356	-2.2	36.6	100	20.96	12.0	even	ok
	101	C33H85N3O10PS2	778.5409	4.5	36.9	101	5.32	-6.0	even	ok
	102	C30H69N17O5P	778.5400	3.4	37.0	102	10.07	6.0	even	ok
	103	C27H69N19NaO4S	778.5393	2.5	37.4	103	17.53	3.0	even	ok
	104	C30H77N9NaO10S	778.5406	4.2	37.6	104	5.27	-3.0	even	ok
	105	C27H72N17O5S2	778.5338	-4.5	37.8	105	5.08	1.0	even	ok
	106	C26H73N19O2PS2	778.5368	-0.7	37.8	106	51.24	1.0	even	ok
	107	C28H72N15O8S	778.5404	3.9	38.1	107	6.88	1.0	even	ok
	108	C44H72N7O3S	778.5412	4.9	38.1	108	3.54	13.0	even	ok
	109	C25H64N25O2S	778.5390	2.1	38.2	109	20.96	7.0	even	ok
	110	C28H78N13NaO4PS2	778.5371	-0.3	38.3	110	57.57	-3.0	even	ok
	111	C40H81N3O5PS2	778.5350	-3.0	38.4	111	15.03	3.0	even	ok
	112	C29H77N11NaO7S2	778.5341	-4.2	38.5	112	6.68	-3.0	even	ok
	113	C34H84NO13S2	778.5379	0.7	38.6	113	50.73	-6.0	even	ok
	114	C27H79N15NaOP2S2	778.5401	3.5	38.7	114	10.84	-3.0	even	ok
	115	C29H66N21NaOP	778.5389	2.0	39.1	115	21.95	8.0	even	ok
	116	C29H79N11NaO5P2S	778.5354	-2.5	40.4	116	15.71	-3.0	even	ok
	117	C33H80NO18	778.5370	-0.5	40.5	117	41.38	-5.0	even	ok
	118	C32H87NNaO11P2S	778.5367	-0.8	40.6	118	36.39	-9.0	even	ok
	119	C42H83N3NaP2S2	778.5396	2.9	40.7	119	15.81	4.0	even	ok
	120	C29H81N9O8PS2	778.5382	1.0	40.8	120	40.87	-5.0	even	ok
	121	C28H82N11O5P2S2	778.5412	4.9	40.8	121	3.41	-5.0	even	ok
	122	C30H82N7O9P2S	778.5364	-1.2	41.1	122	30.97	-5.0	even	ok
	123	C32H77N5NaO14	778.5359	-1.8	41.1	123	22.55	-3.0	even	ok
	124	C27H74N17O3P2S	778.5351	-2.9	41.2	124	12.32	1.0	even	ok
	125	C30H80N7O11S2	778.5352	-2.8	41.3	125	16.35	-5.0	even	ok
	126	C31H86N3NaO10PS2	778.5384	1.4	41.6	126	34.20	-9.0	even	ok
	127	C32H85NNaO13S2	778.5355	-2.4	42.2	127	19.74	-9.0	even	ok
	128	C30H72N11O12	778.5356	-2.2	42.7	128	17.88	1.0	even	ok
	129	C46H69N9P	778.5408	4.4	43.1	129	3.77	18.0	even	ok
	130	C41H77N7OPS2	778.5363	-1.3	43.1	130	33.94	8.0	even	ok
	131	C48H74N3NaO2P	778.5411	4.8	43.9	131	2.71	14.0	even	ok
	132	C29H69N15NaO8	778.5346	-3.6	43.9	132	7.26	3.0	even	ok
	133	C29H81N5NaO14S	778.5393	2.5	44.1	133	18.07	-8.0	even	ok
	134	C32H81N3O15P	778.5400	3.4	44.2	134	8.20	-5.0	even	ok
	135	C47H78N3P2S	778.5386	1.6	45.1	135	27.88	12.0	even	ok
	136	C31H78N7NaO11P	778.5389	2.0	45.1	136	18.44	-3.0	even	ok
	137	C30H84NO18S	778.5404	3.9	45.2	137	6.97	-10.0	even	ok
	138	C43H82NNaO3PS2	778.5366	-1.0	45.4	138	37.09	4.0	even	ok
	139	C27H64N21O6	778.5343	-3.9	45.6	139	5.29	7.0	even	ok
	140	C47H68N7O3	778.5378	0.6	45.9	140	33.78	18.0	even	ok
	141	C29H73N13O9P	778.5386	1.6	46.7	141	21.08	1.0	even	ok
	142	C49H73NNaO5	778.5381	1.0	46.9	142	28.50	14.0	even	ok
	143	C28H70N17NaO5P	778.5376	0.3	48.1	143	35.49	3.0	even	ok
	144	C26H73N15NaO8S	778.5379	0.8	49.0	144	28.90	-2.0	even	ok

Compound Spectrum SmartFormula Report

Meas. m/z	#	Ion Formula	m/z	err [ppm]	mSigma	# mSigma	Score	rdb	e⁻ Conf	N-Rule
	145	C48H77NO3PS	778.5356	-2.2	49.2	145	18.29	12.0	even	ok
	146	C23H65N25NaO2S	778.5366	-1.0	49.6	146	26.22	4.0	even	ok
	147	C27H76N11O12S	778.5390	2.1	49.8	147	14.96	-4.0	even	ok
	148	C24H68N21O6S	778.5377	0.4	49.8	148	32.21	2.0	even	ok
	149	C29H84N3O15S2	778.5338	-4.5	49.9	149	3.63	-10.0	even	ok
	150	C26H65N23O3P	778.5373	-0.1	49.9	150	35.81	7.0	even	ok
	151	C21H60N31S	778.5363	-1.3	50.7	151	21.75	8.0	even	ok
	152	C25H74N17NaO5PS	778.5409	4.6	51.8	152	2.53	-2.0	even	ok
	153	C31H81NNaO18	778.5346	-3.6	52.0	153	5.75	-8.0	even	ok
	154	C28H83N7NaO9P2S	778.5340	-4.2	52.1	154	3.38	-8.0	even	ok
	155	C27H71N19NaO2P2	778.5406	4.1	52.4	155	3.71	3.0	even	ok
	156	C23H69N23O3PS	778.5407	4.2	52.8	156	3.30	2.0	even	ok
	157	C29H86N3O13P2S	778.5351	-2.9	52.8	157	8.83	-10.0	even	ok
	158	C26H78N13O7P2S	778.5338	-4.6	52.8	158	2.45	-4.0	even	ok
	159	C29H76N7O16	778.5343	-3.9	53.3	159	4.23	-4.0	even	ok
	160	C25H66N25P2	778.5403	3.8	54.3	160	4.60	7.0	even	ok
	161	C43H72N9S2	778.5347	-3.5	54.4	161	7.14	13.0	even	ok
	162	C45H81NO3PS2	778.5390	2.1	54.6	162	16.25	7.0	even	ok
	163	C50H74N3P2	778.5352	-2.7	54.8	163	9.27	17.0	even	ok
	164	C30H82N3NaO15P	778.5376	0.3	55.6	164	27.97	-8.0	even	ok
	165	C45H77N3NaO2S2	778.5349	-3.1	56.6	165	8.49	9.0	even	ok
	166	C28H77N9O13P	778.5373	-0.1	57.0	166	28.73	-4.0	even	ok
	167	C27H74N13NaO9P	778.5362	-1.4	58.0	167	16.36	-2.0	even	ok
	168	C50H69N5NaO	778.5394	2.7	58.9	168	8.22	19.0	even	ok
	169	C51H72NO5	778.5405	4.0	59.0	169	3.17	17.0	even	ok
	170	C29H83N5NaO12P2	778.5406	4.1	59.3	170	2.93	-8.0	even	ok
	171	C25H69N19O7P	778.5359	-1.8	59.6	171	13.04	2.0	even	ok
	172	C27H78N11O10P2	778.5403	3.8	60.8	172	3.67	-4.0	even	ok
	173	C24H66N23NaO3P	778.5349	-3.2	60.9	173	5.59	4.0	even	ok
	174	C26H75N15NaO6P2	778.5392	2.4	62.0	174	8.70	-2.0	even	ok
	175	C22H61N29OP	778.5346	-3.5	62.7	175	4.11	8.0	even	ok
	176	C50H72N3O2S	778.5340	-4.3	63.0	176	2.74	17.0	even	ok
	177	C24H70N21O4P2	778.5389	2.0	63.6	177	10.01	2.0	even	ok
	178	C47H76N3O2S2	778.5373	-0.0	66.8	178	26.18	12.0	even	ok
	179	C27H81N5O17P	778.5360	-1.8	67.7	179	9.87	-9.0	even	ok
	180	C26H78N9NaO13P	778.5349	-3.2	68.3	180	4.34	-7.0	even	ok
	181	C23H67N25NaP2	778.5379	0.7	74.0	181	9.56	4.0	even	ok
	182	C53H73NNaS	778.5356	-2.3	74.7	182	7.41	18.0	even	ok
	183	C50H77NNaS2	778.5390	2.1	77.3	183	7.40	13.0	even	ok
	184	C55H72NS	778.5380	0.8	86.1	184	9.15	21.0	even	ok
	185	C58H68N	778.5346	-3.5	99.2	185	0.95	26.0	even	ok

Cmpd 9, 18.1 min

Compound Spectrum SmartFormula Report

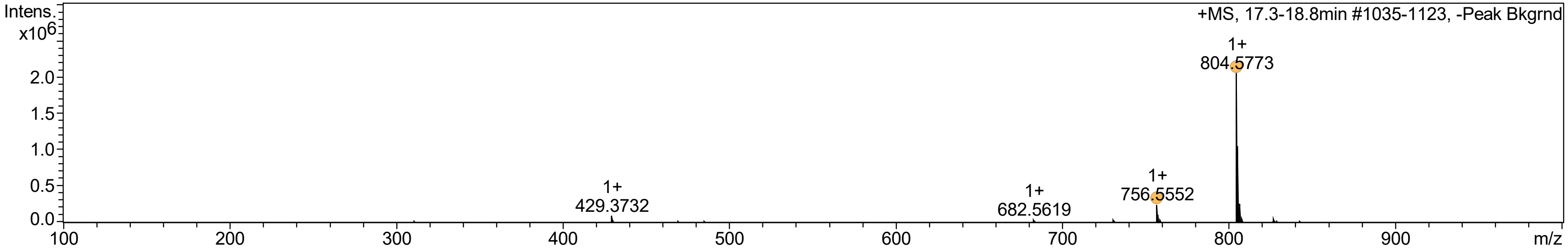

Meas. m/z	#	Ion Formula	m/z	err [ppm]	mSigma	# mSigma	Score	rdb	e⁻ Conf	N-Rule
756.5552	1	C41H80N3O5P2	756.5568	2.1	5.3	1	54.66	5.0	even	ok
	2	C42H79NO8P	756.5538	-1.8	5.3	2	62.07	5.0	even	ok
	3	C40H80NNaO8P	756.5514	-5.0	7.3	3	7.07	2.0	even	ok
	4	C41H76N5NaO4P	756.5527	-3.3	7.4	4	26.85	7.0	even	ok
	5	C39H71N11O2P	756.5524	-3.6	8.6	5	20.50	11.0	even	ok
	6	C40H77N7NaOP2	756.5557	0.7	9.3	6	94.76	7.0	even	ok
	7	C37H71N11NaO4	756.5583	4.1	10.1	7	14.40	8.0	even	ok
	8	C39H81N3NaO5P2	756.5544	-1.1	12.1	8	77.57	2.0	even	ok
	9	C35H66N17O2	756.5580	3.7	12.5	9	17.98	12.0	even	ok
	10	C36H71N13NaOS	756.5517	-4.5	13.3	10	9.51	8.0	even	ok
	11	C36H75N11O2PS	756.5558	0.8	14.0	11	82.45	6.0	even	ok
	12	C37H76N9O3P2	756.5541	-1.4	14.3	12	63.44	6.0	even	ok
	13	C42H76N7OP2	756.5581	3.9	15.2	13	15.26	10.0	even	ok
	14	C44H81NNaO3P2	756.5584	4.2	15.4	14	11.55	6.0	even	ok
	15	C37H74N9O5S	756.5528	-3.1	15.9	15	31.09	6.0	even	ok
	16	C38H80N5NaO4PS	756.5561	1.2	16.0	16	68.30	2.0	even	ok
	17	C39H76N9NaPS	756.5574	3.0	17.1	17	26.93	7.0	even	ok
	18	C43H75N5O4P	756.5551	-0.1	17.1	18	100.00	10.0	even	ok
	19	C39H79N3NaO7S	756.5531	-2.8	18.0	19	37.34	2.0	even	ok
	20	C42H72N9NaP	756.5541	-1.5	18.3	20	57.27	12.0	even	ok
	21	C37H78N3O12	756.5580	3.7	18.3	21	15.86	1.0	even	ok
	22	C32H71N17PS	756.5531	-2.7	18.4	22	30.25	7.0	even	ok
	23	C39H83NO8PS	756.5572	2.6	18.4	23	40.48	0.0	even	ok
	24	C34H76N11NaO2PS	756.5534	-2.4	18.7	24	37.01	3.0	even	ok
	25	C40H79N5O4PS	756.5585	4.4	18.8	25	12.12	5.0	even	ok
	26	C36H75N7NaO8	756.5569	2.3	19.1	26	37.52	3.0	even	ok
	27	C38H70N13OS	756.5542	-1.4	19.6	27	59.03	11.0	even	ok
	28	C40H75N7NaO3S	756.5544	-1.0	20.1	28	85.22	7.0	even	ok
	29	C44H74N3O7	756.5521	-4.0	20.2	29	12.20	10.0	even	ok
	30	C37H84NNaO8PS	756.5547	-0.6	20.4	30	99.16	-3.0	even	ok
	31	C36H78N5O9S	756.5515	-4.9	20.4	31	7.66	1.0	even	ok
	32	C34H70N13O6	756.5567	1.9	20.8	32	43.78	7.0	even	ok
	33	C35H79N7O6PS	756.5545	-0.9	21.5	33	67.44	1.0	even	ok

Compound Spectrum SmartFormula Report

Meas. m/z	#	Ion Formula	m/z	err [ppm]	mSigma	# mSigma	Score	rdb	e¯ Conf	N-Rule
	34	C34H80N9O3P2S	756.5575	3.0	21.6	34	23.77	1.0	even	ok
	35	C36H85N3NaO5P2S	756.5577	3.4	22.1	35	18.57	-3.0	even	ok
	36	C33H67N17NaO2	756.5556	0.5	22.7	36	76.46	9.0	even	ok
	37	C41H78N3O7S	756.5555	0.4	23.3	37	98.10	5.0	even	ok
	38	C36H80N5O7P2	756.5527	-3.2	23.5	38	20.05	1.0	even	ok
	39	C43H81N3NaP2S	756.5519	-4.4	24.7	39	10.59	6.0	even	ok
	40	C31H62N23	756.5553	0.2	24.8	40	82.49	13.0	even	ok
	41	C35H77N9NaO3P2	756.5517	-4.6	25.1	41	6.92	3.0	even	ok
	42	C33H72N15OP2	756.5514	-5.0	27.1	42	4.85	7.0	even	ok
	43	C30H71N17NaO2S	756.5590	5.0	28.2	43	4.74	4.0	even	ok
	44	C34H83N3O10PS	756.5531	-2.7	28.6	44	30.34	-4.0	even	ok
	45	C28H66N23S	756.5587	4.6	28.6	45	6.40	8.0	even	ok
	46	C44H71N9P	756.5565	1.7	29.0	46	41.23	15.0	even	ok
	47	C42H74N7O3S	756.5568	2.2	29.1	47	40.06	10.0	even	ok
	48	C33H80N7NaO6PS	756.5521	-4.1	29.7	48	9.21	-2.0	even	ok
	49	C31H75N13O4PS	756.5518	-4.5	29.7	49	6.91	2.0	even	ok
	50	C46H76N3NaO2P	756.5567	2.1	29.8	50	33.70	11.0	even	ok
	51	C35H79N3NaO12	756.5556	0.5	30.0	51	64.30	-2.0	even	ok
	52	C44H79NNaO5S	756.5571	2.6	31.2	52	31.08	6.0	even	ok
	53	C32H81N9NaO3P2S	756.5551	-0.2	31.2	53	70.80	-2.0	even	ok
	54	C33H74N9O10	756.5553	0.2	31.3	54	70.40	2.0	even	ok
	55	C30H76N15OP2S	756.5548	-0.5	31.6	55	61.93	2.0	even	ok
	56	C33H75N13NaOS2	756.5551	-0.1	31.8	56	89.50	3.0	even	ok
	57	C45H70N7O3	756.5535	-2.3	32.0	57	28.63	15.0	even	ok
	58	C32H71N13NaO6	756.5542	-1.2	32.3	58	46.53	4.0	even	ok
	59	C33H84N5O7P2S	756.5561	1.2	32.8	59	45.84	-4.0	even	ok
	60	C47H75NNaO5	756.5537	-1.9	33.1	60	33.62	11.0	even	ok
	61	C35H74N13OS2	756.5575	3.1	33.6	61	21.23	6.0	even	ok
	62	C34H80N5NaO9P	756.5586	4.5	33.7	62	6.22	-2.0	even	ok
	63	C37H84N5O2P2S2	756.5536	-2.1	33.7	63	38.10	0.0	even	ok
	64	C30H66N19O4	756.5540	-1.6	34.0	64	37.84	8.0	even	ok
	65	C35H84NO11P2	756.5514	-5.0	34.2	65	4.12	-4.0	even	ok
	66	C45H80N3P2S	756.5543	-1.2	34.9	66	55.19	9.0	even	ok
	67	C32H75N11O7P	756.5583	4.1	35.2	67	7.96	2.0	even	ok
	68	C34H78N9O5S2	756.5562	1.3	35.5	68	51.52	1.0	even	ok
	69	C31H80N11NaO2PS2	756.5568	2.1	35.6	69	35.66	-2.0	even	ok
	70	C30H74N15O3S2	756.5535	-2.2	35.6	70	33.39	2.0	even	ok
	71	C29H63N23Na	756.5529	-3.0	35.7	71	16.93	10.0	even	ok
	72	C37H79N7NaO3S2	756.5578	3.5	35.8	72	15.78	2.0	even	ok
	73	C36H88NO6P2S2	756.5523	-3.8	36.4	73	12.02	-5.0	even	ok
	74	C31H72N15NaO3P	756.5572	2.7	36.5	74	19.79	4.0	even	ok
	75	C40H85N3NaP2S2	756.5552	0.1	36.6	75	79.45	1.0	even	ok
	76	C32H79N9NaO5S2	756.5538	-1.9	37.0	76	38.94	-2.0	even	ok
	77	C32H83N3NaO12S	756.5590	5.0	37.0	77	4.70	-7.0	even	ok
	78	C36H83N3NaO7S2	756.5565	1.7	37.3	78	41.52	-3.0	even	ok
	79	C29H67N21OP	756.5570	2.4	38.3	79	23.24	8.0	even	ok

Compound Spectrum SmartFormula Report

Meas. m/z	#	Ion Formula	m/z	err [ppm]	mSigma	# mSigma	Score	rdb	e¯ Conf	N-Rule
	80	C39H79N7OPS2	756.5520	-4.3	38.7	80	8.30	5.0	even	ok
	81	C32H83N7O6PS2	756.5578	3.5	38.7	81	14.14	-4.0	even	ok
	82	C29H75N17PS2	756.5565	1.7	38.8	82	39.31	2.0	even	ok
	83	C45H75N5NaOS	756.5585	4.3	39.1	83	7.74	11.0	even	ok
	84	C38H82N3O7S2	756.5589	4.9	39.5	84	4.90	0.0	even	ok
	85	C26H67N23NaS	756.5563	1.4	39.5	85	35.24	5.0	even	ok
	86	C29H75N13NaO6S	756.5576	3.2	39.9	86	13.28	-1.0	even	ok
	87	C34H88NNaO8PS2	756.5581	3.9	40.0	87	10.54	-8.0	even	ok
	88	C27H70N19O4S	756.5573	2.9	40.3	88	16.52	3.0	even	ok
	89	C33H82N5O9S2	756.5548	-0.4	40.3	89	63.87	-4.0	even	ok
	90	C41H84NNaO3PS2	756.5522	-3.9	41.0	90	10.26	1.0	even	ok
	91	C30H78N9O10S	756.5587	4.6	41.3	91	4.56	-3.0	even	ok
	92	C32H78N5O14	756.5540	-1.6	42.4	92	30.42	-3.0	even	ok
	93	C28H77N15NaOP2S	756.5524	-3.7	42.5	93	8.82	-1.0	even	ok
	94	C28H79N13O4PS2	756.5552	-0.0	42.8	94	67.82	-3.0	even	ok
	95	C31H85N5NaO7P2S	756.5537	-1.9	42.9	95	25.41	-7.0	even	ok
	96	C31H75N9NaO10	756.5529	-3.0	42.9	96	14.04	-1.0	even	ok
	97	C29H78N11O7S2	756.5522	-4.0	43.2	97	8.90	-3.0	even	ok
	98	C29H80N11O5P2S	756.5534	-2.3	43.3	98	20.71	-3.0	even	ok
	99	C29H85N9NaO3P2S2	756.5584	4.3	43.4	99	7.08	-7.0	even	ok
	100	C30H84N7NaO6PS2	756.5554	0.3	43.6	100	60.46	-7.0	even	ok
	101	C42H84N3P2S2	756.5576	3.3	43.7	101	14.65	4.0	even	ok
	102	C27H76N17NaPS2	756.5541	-1.4	43.7	102	39.02	-1.0	even	ok
	103	C31H83N5NaO9S2	756.5524	-3.6	44.2	103	11.29	-7.0	even	ok
	104	C32H88NO11P2S	756.5548	-0.5	44.2	104	44.42	-9.0	even	ok
	105	C29H70N15O8	756.5526	-3.4	44.3	105	10.63	3.0	even	ok
	106	C33H84NNaO13P	756.5572	2.7	44.6	106	15.70	-7.0	even	ok
	107	C48H71N5NaO	756.5551	-0.1	45.0	107	49.62	16.0	even	ok
	108	C49H74NO5	756.5562	1.3	45.2	108	32.15	14.0	even	ok
	109	C28H67N19NaO4	756.5516	-4.8	45.4	109	3.59	5.0	even	ok
	110	C31H79N7O11P	756.5570	2.4	45.9	110	18.63	-3.0	even	ok
	111	C27H80N15OP2S2	756.5581	3.9	46.1	111	8.66	-3.0	even	ok
	112	C31H87N3O10PS2	756.5565	1.7	46.3	112	31.60	-9.0	even	ok
	113	C30H76N11NaO7P	756.5559	1.0	46.7	113	35.28	-1.0	even	ok
	114	C32H86NO13S2	756.5535	-2.2	47.1	114	24.45	-9.0	even	ok
	115	C29H82N5O14S	756.5573	2.9	47.4	115	16.74	-8.0	even	ok
	116	C28H71N17O5P	756.5556	0.6	48.3	116	38.69	3.0	even	ok
	117	C43H83NO3PS2	756.5547	-0.7	48.3	117	46.30	4.0	even	ok
	118	C27H68N21NaOP	756.5546	-0.8	49.5	118	34.01	5.0	even	ok
	119	C29H77N13NaO4P2	756.5589	4.9	50.6	119	2.77	-1.0	even	ok
	120	C25H71N19NaO4S	756.5549	-0.3	51.2	120	38.63	0.0	even	ok
	121	C28H79N9NaO10S	756.5563	1.5	51.5	121	24.68	-6.0	even	ok
	122	C23H66N25O2S	756.5547	-0.7	52.0	122	33.16	4.0	even	ok
	123	C26H74N15O8S	756.5560	1.1	52.0	123	28.48	-2.0	even	ok
	124	C27H72N19O2P2	756.5586	4.5	52.3	124	3.57	3.0	even	ok
	125	C31H82NO18	756.5526	-3.4	53.7	125	8.09	-8.0	even	ok

Compound Spectrum SmartFormula Report

Meas. m/z	#	Ion Formula	m/z	err [ppm]	mSigma	# mSigma	Score	rdb	e⁻ Conf	N-Rule
	126	C30H79N5NaO14	756.5516	-4.8	53.9	126	2.81	-6.0	even	ok
	127	C24H72N21NaOPS	756.5579	3.6	53.9	127	6.70	0.0	even	ok
	128	C25H75N17O5PS	756.5590	5.0	54.4	128	2.19	-2.0	even	ok
	129	C30H89NNaO11P2S	756.5524	-3.7	54.6	129	6.21	-12.0	even	ok
	130	C28H84N7O9P2S	756.5521	-4.1	55.0	130	4.68	-8.0	even	ok
	131	C30H83N3O15P	756.5556	0.6	57.0	131	29.29	-8.0	even	ok
	132	C50H70N5O	756.5575	3.0	57.0	132	8.85	19.0	even	ok
	133	C29H80N7NaO11P	756.5546	-0.8	57.4	133	26.65	-6.0	even	ok
	134	C27H75N13O9P	756.5543	-1.2	58.8	134	21.97	-2.0	even	ok
	135	C45H78N3O2S2	756.5530	-2.9	58.8	135	11.53	9.0	even	ok
	136	C26H72N17NaO5P	756.5532	-2.6	59.6	136	10.65	0.0	even	ok
	137	C29H84N5O12P2	756.5586	4.5	60.4	137	2.71	-8.0	even	ok
	138	C28H81N9NaO8P2	756.5576	3.1	61.1	138	7.32	-6.0	even	ok
	139	C24H67N23O3P	756.5529	-3.0	61.2	139	8.11	4.0	even	ok
	140	C26H76N15O6P2	756.5573	2.8	62.5	140	8.73	-2.0	even	ok
	141	C25H73N19NaO2P2	756.5562	1.4	63.5	141	17.37	0.0	even	ok
	142	C48H79NNaS2	756.5546	-0.7	68.2	142	23.44	10.0	even	ok
	143	C28H84N3NaO15P	756.5532	-2.6	68.4	143	7.90	-11.0	even	ok
	144	C23H68N25P2	756.5559	1.0	74.6	144	10.13	4.0	even	ok
	145	C53H74NS	756.5536	-2.0	74.9	145	10.42	18.0	even	ok
	146	C50H78NS2	756.5570	2.4	78.7	146	7.24	13.0	even	ok
804.5773	1	C41H71N15P	804.5749	-2.9	4.9	1	30.40	15.0	even	ok
	2	C43H76N9NaO2P	804.5752	-2.6	5.1	2	37.96	11.0	even	ok
	3	C43H80N7O3P2	804.5792	2.5	7.7	3	39.29	9.0	even	ok
	4	C46H81N5NaOP2	804.5809	4.5	8.2	4	8.72	10.0	even	ok
	5	C45H85NNaO5P2	804.5795	2.8	8.3	5	31.20	5.0	even	ok
	6	C44H79N5O6P	804.5762	-1.3	8.5	6	73.46	9.0	even	ok
	7	C45H75N9O2P	804.5776	0.4	10.9	7	100.00	14.0	even	ok
	8	C45H78N3O9	804.5733	-5.0	11.7	8	5.11	9.0	even	ok
	9	C47H80N3NaO4P	804.5779	0.7	13.2	9	83.76	10.0	even	ok
	10	C45H71N11NaO	804.5735	-4.6	13.6	10	6.74	16.0	even	ok
	11	C39H71N15NaO2	804.5807	4.3	15.3	11	8.67	12.0	even	ok
	12	C42H80N5NaO6P	804.5738	-4.3	15.9	12	9.09	6.0	even	ok
	13	C46H74N7O5	804.5746	-3.3	15.9	13	18.97	14.0	even	ok
	14	C40H75N11O4P	804.5736	-4.6	16.4	14	6.64	10.0	even	ok
	15	C37H66N21	804.5805	4.0	16.4	15	11.33	16.0	even	ok
	16	C43H83NO10P	804.5749	-2.9	17.6	16	24.09	4.0	even	ok
	17	C48H79NNaO7	804.5749	-3.0	18.3	17	23.03	10.0	even	ok
	18	C41H81N7NaO3P2	804.5768	-0.5	18.3	18	82.17	6.0	even	ok
	19	C42H84N3O7P2	804.5779	0.8	19.1	19	73.04	4.0	even	ok
	20	C39H76N13OP2	804.5766	-0.9	19.4	20	69.95	10.0	even	ok
	21	C48H76N7NaP	804.5792	2.4	23.0	21	29.68	15.0	even	ok
	22	C40H80N9NaO2PS	804.5786	1.6	24.1	22	56.67	6.0	even	ok
	23	C42H79N9O2PS	804.5810	4.6	24.7	23	6.96	9.0	even	ok
	24	C49H79N3O4P	804.5803	3.7	24.8	24	11.40	13.0	even	ok
	25	C39H74N13O3S	804.5753	-2.5	24.8	25	34.61	10.0	even	ok

Compound Spectrum SmartFormula Report

Meas. m/z	#	Ion Formula	m/z	err [ppm]	mSigma	# mSigma	Score	rdb	e⁻ Conf	N-Rule
	26	C42H75N11NaOS	804.5769	-0.5	24.9	26	91.80	11.0	even	ok
	27	C38H75N15PS	804.5783	1.3	25.2	27	52.28	10.0	even	ok
	28	C47H70N11O	804.5759	-1.7	25.7	28	42.63	19.0	even	ok
	29	C41H79N7NaO5S	804.5756	-2.1	26.5	29	40.79	6.0	even	ok
	30	C44H84N3NaO4PS	804.5812	4.9	26.9	30	4.80	5.0	even	ok
	31	C38H75N11NaO6	804.5794	2.7	27.0	31	23.28	7.0	even	ok
	32	C41H83NNaO12	804.5807	4.3	27.3	32	6.59	1.0	even	ok
	33	C41H83N5O6PS	804.5796	2.9	27.6	33	24.02	4.0	even	ok
	34	C49H75N5NaO3	804.5762	-1.3	27.7	34	48.24	15.0	even	ok
	35	C39H78N7O10	804.5805	4.0	27.7	35	8.75	5.0	even	ok
	36	C36H70N17O4	804.5791	2.3	28.0	36	28.15	11.0	even	ok
	37	C35H67N21Na	804.5781	1.0	28.3	37	54.92	13.0	even	ok
	38	C43H78N7O5S	804.5780	0.9	28.6	38	71.28	9.0	even	ok
	39	C50H78NO7	804.5773	0.0	29.7	39	76.08	13.0	even	ok
	40	C46H84N3O2P2S	804.5754	-2.3	30.0	40	33.48	8.0	even	ok
	41	C37H79N11O4PS	804.5769	-0.4	30.0	41	82.66	5.0	even	ok
	42	C40H85N3NaO7P2	804.5755	-2.2	30.1	42	28.63	1.0	even	ok
	43	C42H82N3O9S	804.5766	-0.8	30.2	43	71.14	4.0	even	ok
	44	C36H76N15NaPS	804.5759	-1.7	30.6	44	36.33	7.0	even	ok
	45	C38H78N9O7S	804.5739	-4.1	30.8	45	9.05	5.0	even	ok
	46	C45H83NNaO7S	804.5782	1.2	30.9	46	58.00	5.0	even	ok
	47	C39H84N5NaO6PS	804.5772	-0.1	30.9	47	91.25	1.0	even	ok
	48	C44H74N11OS	804.5793	2.5	31.0	48	28.51	14.0	even	ok
	49	C38H80N9O5P2	804.5752	-2.5	31.0	49	22.72	5.0	even	ok
	50	C37H77N13NaOP2	804.5741	-3.9	31.2	50	8.82	7.0	even	ok
	51	C40H83N3NaO9S	804.5742	-3.8	31.9	51	11.66	1.0	even	ok
	52	C39H88N3O7P2S	804.5813	5.0	33.1	52	3.96	-1.0	even	ok
	53	C46H79N5NaO3S	804.5796	2.9	33.4	53	21.49	10.0	even	ok
	54	C36H80N13OP2S	804.5799	3.3	33.4	54	12.80	5.0	even	ok
	55	C40H87NO10PS	804.5783	1.3	33.8	55	52.74	-1.0	even	ok
	56	C38H85N7NaO3P2S	804.5802	3.7	34.0	56	9.73	1.0	even	ok
	57	C52H81NNaP2	804.5736	-4.5	35.6	57	4.66	14.0	even	ok
	58	C47H82NO7S	804.5807	4.2	36.8	58	7.21	8.0	even	ok
	59	C49H85NNaP2S	804.5770	-0.3	37.2	59	71.42	9.0	even	ok
	60	C37H79N7NaO10	804.5781	1.0	38.8	60	41.84	2.0	even	ok
	61	C36H83N7O8PS	804.5756	-2.1	39.0	61	30.60	0.0	even	ok
	62	C38H88NNaO10PS	804.5759	-1.7	39.4	62	36.42	-4.0	even	ok
	63	C38H82N3O14	804.5791	2.3	39.5	63	20.87	0.0	even	ok
	64	C34H71N17NaO4	804.5767	-0.7	39.7	64	46.50	8.0	even	ok
	65	C35H74N13O8	804.5778	0.6	39.7	65	47.10	6.0	even	ok
	66	C48H79N5OPS	804.5737	-4.4	39.7	66	5.83	13.0	even	ok
	67	C37H89N3NaO7P2S	804.5789	2.0	39.8	67	31.20	-4.0	even	ok
	68	C51H74N5O3	804.5786	1.7	40.0	68	29.26	18.0	even	ok
	69	C32H66N23O2	804.5764	-1.0	40.9	69	38.92	12.0	even	ok
	70	C33H75N17O2PS	804.5742	-3.7	41.0	70	7.54	6.0	even	ok
	71	C35H80N11NaO4PS	804.5745	-3.4	41.2	71	9.77	2.0	even	ok

Compound Spectrum SmartFormula Report

Meas. m/z	#	Ion Formula	m/z	err [ppm]	mSigma	# mSigma	Score	rdb	e⁻ Conf	N-Rule
	72	C36H80N9NaO7P	804.5811	4.7	41.8	72	3.25	2.0	even	ok
	73	C39H84N9P2S2	804.5761	-1.5	42.0	73	38.66	4.0	even	ok
	74	C34H81N13NaOP2S	804.5775	0.3	42.3	74	49.56	2.0	even	ok
	75	C37H85N9NaP2S2	804.5737	-4.5	42.5	75	5.02	1.0	even	ok
	76	C37H84N5O9P2	804.5739	-4.2	42.7	76	4.94	0.0	even	ok
	77	C34H75N15O5P	804.5808	4.4	42.9	77	4.29	6.0	even	ok
	78	C33H72N19NaOP	804.5797	3.0	43.2	78	11.92	8.0	even	ok
	79	C39H79N11NaOS2	804.5803	3.7	43.5	79	8.84	6.0	even	ok
	80	C41H89N3NaO2P2S2	804.5764	-1.1	44.0	80	42.99	0.0	even	ok
	81	C35H84N9O5P2S	804.5786	1.6	44.2	81	26.55	0.0	even	ok
	82	C36H78N13O3S2	804.5787	1.7	44.8	82	31.27	5.0	even	ok
	83	C32H74N19OS2	804.5760	-1.6	45.9	83	32.15	6.0	even	ok
	84	C33H80N15NaPS2	804.5792	2.5	45.9	84	20.11	2.0	even	ok
	85	C38H88N5O4P2S2	804.5747	-3.1	46.0	85	12.95	-1.0	even	ok
	86	C38H83N7NaO5S2	804.5789	2.1	46.5	86	24.68	1.0	even	ok
	87	C43H88N3O2P2S2	804.5788	1.9	46.7	87	27.40	3.0	even	ok
	88	C34H84N7NaO8PS	804.5732	-5.1	46.8	88	2.53	-3.0	even	ok
	89	C51H84NP2S	804.5794	2.7	46.9	89	16.90	12.0	even	ok
	90	C43H84N5NaOPS2	804.5747	-3.2	46.9	90	12.19	5.0	even	ok
	91	C32H78N13O8S	804.5812	4.8	47.0	91	3.11	1.0	even	ok
	92	C34H79N13NaO3S2	804.5762	-1.3	47.1	92	36.70	2.0	even	ok
	93	C40H82N7O5S2	804.5813	5.1	47.3	93	2.48	4.0	even	ok
	94	C42H88NNaO5PS2	804.5734	-4.8	47.3	94	3.10	0.0	even	ok
	95	C54H80NP2	804.5761	-1.5	48.2	95	25.32	17.0	even	ok
	96	C35H87N3O12PS	804.5743	-3.7	48.6	96	7.65	-5.0	even	ok
	97	C34H83N11O4PS2	804.5803	3.8	48.9	97	7.28	0.0	even	ok
	98	C34H88N5O9P2S	804.5773	-0.0	49.6	98	55.47	-5.0	even	ok
	99	C39H86N3O9S2	804.5800	3.4	50.1	99	9.38	-1.0	even	ok
	100	C36H88N5NaO6PS2	804.5806	4.1	50.1	100	5.30	-4.0	even	ok
	101	C35H82N9O7S2	804.5773	0.1	50.3	101	53.33	0.0	even	ok
	102	C36H83N3NaO14	804.5767	-0.7	50.5	102	34.13	-3.0	even	ok
	103	C46H89NNaP2S2	804.5804	3.9	50.6	103	6.38	4.0	even	ok
	104	C44H87NO5PS2	804.5758	-1.8	50.7	104	24.71	3.0	even	ok
	105	C31H75N17NaO4S	804.5801	3.5	50.7	105	6.80	3.0	even	ok
	106	C30H75N19NaOS2	804.5736	-4.6	50.9	106	3.44	3.0	even	ok
	107	C29H70N23O2S	804.5798	3.2	50.9	107	8.69	7.0	even	ok
	108	C33H75N13NaO8	804.5754	-2.3	51.2	108	14.68	3.0	even	ok
	109	C34H78N9O12	804.5764	-1.0	51.4	109	28.83	1.0	even	ok
	110	C37H87N3NaO9S2	804.5776	0.4	51.7	110	45.35	-4.0	even	ok
	111	C37H92NO8P2S2	804.5734	-4.8	52.0	111	2.80	-6.0	even	ok
	112	C31H70N19O6	804.5751	-2.7	52.3	112	11.45	7.0	even	ok
	113	C30H67N23NaO2	804.5740	-4.0	52.6	113	4.30	9.0	even	ok
	114	C45H83N5OPS2	804.5771	-0.2	52.7	114	47.66	8.0	even	ok
	115	C54H75N3NaO	804.5802	3.7	53.0	115	5.50	19.0	even	ok
	116	C30H79N17O2PS2	804.5776	0.4	53.2	116	42.69	1.0	even	ok
	117	C35H84N5NaO11P	804.5797	3.0	53.4	117	8.71	-3.0	even	ok

Compound Spectrum SmartFormula Report

Meas. m/z	#	Ion Formula	m/z	err [ppm]	mSigma	# mSigma	Score	rdb	e¯ Conf	N-Rule
	118	C31H78N15O5S2	804.5746	-3.3	53.6	118	9.25	1.0	even	ok
	119	C33H85N9NaO5P2S	804.5762	-1.3	53.7	119	23.10	-3.0	even	ok
	120	C36H87NO15P	804.5808	4.4	53.8	120	3.06	-5.0	even	ok
	121	C31H85N13NaOP2S2	804.5809	4.5	53.8	121	3.41	-3.0	even	ok
	122	C31H80N15O3P2S	804.5759	-1.7	53.9	122	19.33	1.0	even	ok
	123	C32H84N11NaO4PS2	804.5779	0.8	54.0	123	36.47	-3.0	even	ok
	124	C28H62N29	804.5737	-4.4	54.0	124	3.07	13.0	even	ok
	125	C33H79N11O9P	804.5794	2.7	54.4	125	10.63	1.0	even	ok
	126	C32H76N15NaO5P	804.5784	1.4	54.5	126	22.20	3.0	even	ok
	127	C33H83N9NaO7S2	804.5749	-2.9	54.5	127	11.44	-3.0	even	ok
	128	C30H71N21O3P	804.5781	1.0	55.7	128	24.97	7.0	even	ok
	129	C33H87N7O8PS2	804.5790	2.1	56.7	129	17.55	-5.0	even	ok
	130	C33H87N3NaO14S	804.5801	3.5	57.1	130	6.87	-8.0	even	ok
	131	C31H82N9O12S	804.5798	3.2	57.1	131	8.84	-4.0	even	ok
	132	C46H82N3O4S2	804.5741	-3.9	57.3	132	5.10	8.0	even	ok
	133	C34H86N5O11S2	804.5760	-1.6	57.4	133	22.65	-5.0	even	ok
	134	C35H92NNaO10PS2	804.5792	2.5	57.6	134	13.89	-9.0	even	ok
	135	C31H77N17NaO2P2	804.5814	5.1	58.0	135	1.38	3.0	even	ok
	136	C29H72N23P2	804.5811	4.7	59.4	136	1.84	7.0	even	ok
	137	C33H92NO13P2S	804.5759	-1.7	59.8	137	20.09	-10.0	even	ok
	138	C26H75N23PS2	804.5749	-2.9	59.8	138	9.82	2.0	even	ok
	139	C28H80N17NaO2PS2	804.5752	-2.5	60.1	139	12.15	-2.0	even	ok
	140	C27H71N23NaO2S	804.5774	0.2	61.4	140	28.73	4.0	even	ok
	141	C47H78N7S2	804.5755	-2.2	61.7	141	13.87	13.0	even	ok
	142	C25H66N29S	804.5771	-0.2	62.0	142	28.17	8.0	even	ok
	143	C30H82N11O9S2	804.5733	-4.9	62.2	143	1.74	-4.0	even	ok
	144	C30H79N13NaO8S	804.5787	1.8	62.3	144	13.50	-2.0	even	ok
	145	C29H83N13O6PS2	804.5763	-1.2	62.3	145	23.01	-4.0	even	ok
	146	C28H74N19O6S	804.5785	1.5	62.6	146	15.97	2.0	even	ok
	147	C32H79N9NaO12	804.5740	-4.0	62.7	147	3.11	-2.0	even	ok
	148	C28H84N15O3P2S2	804.5793	2.5	62.7	148	11.43	-4.0	even	ok
	149	C31H88N7NaO8PS2	804.5766	-0.9	62.8	149	26.31	-8.0	even	ok
	150	C32H87N5NaO11S2	804.5736	-4.6	62.9	150	2.35	-8.0	even	ok
	151	C33H82N5O16	804.5751	-2.7	63.0	151	8.10	-4.0	even	ok
	152	C30H89N9NaO5P2S2	804.5796	2.8	63.1	152	9.05	-8.0	even	ok
	153	C30H74N15O10	804.5738	-4.4	63.7	153	2.24	2.0	even	ok
	154	C49H83NNaO2S2	804.5757	-1.9	64.1	154	15.49	9.0	even	ok
	155	C29H81N15NaO3P2S	804.5735	-4.7	64.5	155	1.63	-2.0	even	ok
	156	C54H78NO2S	804.5748	-3.1	64.5	156	7.31	17.0	even	ok
	157	C27H76N21OP2S	804.5732	-5.0	65.0	157	1.16	2.0	even	ok
	158	C34H88NNaO15P	804.5784	1.4	65.0	158	15.47	-8.0	even	ok
	159	C32H91N3O12PS2	804.5776	0.5	65.3	159	28.39	-10.0	even	ok
	160	C32H89N5NaO9P2S	804.5748	-3.0	65.3	160	6.03	-8.0	even	ok
	161	C31H92N5O9P2S2	804.5806	4.2	65.3	161	3.09	-10.0	even	ok
	162	C33H90NO15S2	804.5746	-3.3	65.5	162	6.26	-10.0	even	ok
	163	C30H84N11O7P2S	804.5746	-3.4	65.6	163	4.67	-4.0	even	ok

Compound Spectrum SmartFormula Report

Meas. m/z	#	Ion Formula	m/z	err [ppm]	mSigma	# mSigma	Score	rdb	e¯ Conf	N-Rule
	164	C31H80N11NaO9P	804.5770	-0.3	65.9	164	23.63	-2.0	even	ok
	165	C32H83N7O13P	804.5781	1.0	65.9	165	17.55	-4.0	even	ok
	166	C29H83N9NaO12S	804.5774	0.2	66.1	166	30.32	-7.0	even	ok
	167	C29H75N17O7P	804.5768	-0.6	67.0	167	19.95	2.0	even	ok
	168	C28H72N21NaO3P	804.5757	-2.0	67.3	168	10.62	4.0	even	ok
	169	C30H86N5O16S	804.5785	1.5	67.4	169	16.70	-9.0	even	ok
	170	C26H67N27OP	804.5754	-2.3	68.6	170	8.30	8.0	even	ok
	171	C31H93NNaO13P2S	804.5735	-4.7	68.8	171	1.77	-13.0	even	ok
	172	C31H84N9O10P2	804.5811	4.8	69.0	172	1.29	-4.0	even	ok
	173	C30H81N13NaO6P2	804.5800	3.4	69.2	173	3.87	-2.0	even	ok
	174	C28H76N19O4P2	804.5797	3.1	70.4	174	4.74	2.0	even	ok
	175	C27H73N23NaP2	804.5787	1.8	70.9	175	10.39	4.0	even	ok
	176	C23H67N29NaS	804.5747	-3.2	72.9	176	4.07	5.0	even	ok
	177	C26H75N19NaO6S	804.5761	-1.5	73.1	177	10.92	-1.0	even	ok
	178	C51H82NO2S2	804.5781	1.1	73.2	178	16.34	12.0	even	ok
	179	C24H70N25O4S	804.5758	-1.8	73.7	179	8.96	3.0	even	ok
	180	C27H78N15O10S	804.5771	-0.2	74.2	180	18.08	-3.0	even	ok
	181	C32H86NO20	804.5738	-4.3	74.7	181	1.52	-9.0	even	ok
	182	C28H84N11NaO9PS	804.5804	3.9	75.9	182	2.08	-7.0	even	ok
	183	C30H84N7NaO13P	804.5757	-2.0	77.3	183	7.33	-7.0	even	ok
	184	C31H87N3O17P	804.5768	-0.6	77.5	184	13.48	-9.0	even	ok
	185	C28H79N13O11P	804.5754	-2.3	78.3	185	5.77	-3.0	even	ok
	186	C27H76N17NaO7P	804.5743	-3.6	78.5	186	2.34	-1.0	even	ok
	187	C24H67N27NaO3	804.5812	4.9	79.0	187	0.74	5.0	even	ok
	188	C25H71N23O5P	804.5741	-4.0	79.7	188	1.69	3.0	even	ok
	189	C22H62N33O	804.5810	4.6	80.3	189	0.97	9.0	even	ok
	190	C29H85N9NaO10P2	804.5787	1.8	80.5	190	7.11	-7.0	even	ok
	191	C30H88N5O14P2	804.5798	3.1	80.5	191	3.18	-9.0	even	ok
	192	C27H80N15O8P2	804.5784	1.4	81.6	192	8.09	-3.0	even	ok
	193	C26H77N19NaO4P2	804.5773	0.1	81.9	193	13.65	-1.0	even	ok
	194	C24H72N25O2P2	804.5771	-0.3	83.2	194	12.24	3.0	even	ok

Cmpd 10, 19.7 min

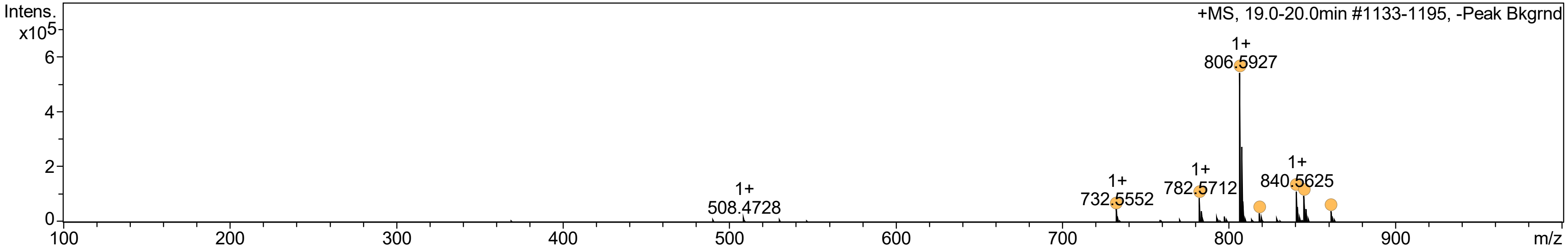

Compound Spectrum SmartFormula Report

Meas. m/z	#	Ion Formula	m/z	err [ppm]	mSigma	# mSigma	Score	rdb	e¯ Conf	N-Rule
732.5552	1	C40H79NO8P	732.5538	-1.9	1.5	1	61.24	3.0	even	ok
	2	C39H80N3O5P2	732.5568	2.2	5.3	2	49.02	3.0	even	ok
	3	C39H76N5NaO4P	732.5527	-3.3	5.4	3	25.90	5.0	even	ok
	4	C37H71N11O2P	732.5524	-3.7	7.5	4	19.48	9.0	even	ok
	5	C38H77N7NaOP2	732.5557	0.7	9.3	5	85.49	5.0	even	ok
	6	C42H81NNaO3P2	732.5584	4.4	11.4	6	11.11	4.0	even	ok
	7	C40H76N7OP2	732.5581	4.0	11.6	7	14.60	8.0	even	ok
	8	C41H75N5O4P	732.5551	-0.1	13.0	8	100.00	8.0	even	ok
	9	C35H71N11NaO4	732.5583	4.2	13.1	9	12.13	6.0	even	ok
	10	C34H71N13NaOS	732.5517	-4.7	14.0	10	8.77	6.0	even	ok
	11	C40H72N9NaP	732.5541	-1.5	14.5	11	57.15	10.0	even	ok
	12	C37H76N9NaPS	732.5574	3.1	15.1	12	25.11	5.0	even	ok
	13	C33H66N17O2	732.5580	3.9	15.3	13	15.21	10.0	even	ok
	14	C37H81N3NaO5P2	732.5544	-1.1	15.4	14	67.05	0.0	even	ok
	15	C34H75N11O2PS	732.5558	0.9	15.7	15	71.89	4.0	even	ok
	16	C42H74N3O7	732.5521	-4.1	15.9	16	12.44	8.0	even	ok
	17	C36H80N5NaO4PS	732.5561	1.3	17.2	17	59.99	0.0	even	ok
	18	C36H70N13OS	732.5542	-1.4	17.3	18	57.29	9.0	even	ok
	19	C35H76N9O3P2	732.5541	-1.5	17.4	19	55.06	4.0	even	ok
	20	C35H74N9O5S	732.5528	-3.2	18.0	20	22.17	4.0	even	ok
	21	C37H79N3NaO7S	732.5531	-2.8	18.2	21	34.56	0.0	even	ok
	22	C38H79N5O4PS	732.5585	4.5	19.0	22	8.60	3.0	even	ok
	23	C37H83NO8PS	732.5572	2.7	19.2	23	35.66	-2.0	even	ok
	24	C38H75N7NaO3S	732.5544	-1.0	19.8	24	63.32	5.0	even	ok
	25	C39H78N3O7S	732.5555	0.5	21.4	25	92.20	3.0	even	ok
	26	C30H71N17PS	732.5531	-2.8	22.2	26	25.86	5.0	even	ok
	27	C32H76N11NaO2PS	732.5534	-2.4	22.3	27	31.74	1.0	even	ok
	28	C35H78N3O12	732.5580	3.9	22.4	28	12.97	-1.0	even	ok
	29	C34H78N5O9S	732.5515	-5.0	22.7	29	6.82	-1.0	even	ok
	30	C34H75N7NaO8	732.5569	2.4	22.9	30	30.96	1.0	even	ok
	31	C41H81N3NaP2S	732.5519	-4.5	23.9	31	8.06	4.0	even	ok
	32	C32H70N13O6	732.5567	2.0	24.5	32	36.24	5.0	even	ok
	33	C33H79N7O6PS	732.5545	-0.9	24.8	33	57.85	-1.0	even	ok
	34	C42H71N9P	732.5565	1.8	24.8	34	40.86	13.0	even	ok
	35	C32H80N9O3P2S	732.5575	3.1	25.4	35	19.54	-1.0	even	ok
	36	C35H84NNaO8PS	732.5547	-0.6	25.5	36	65.28	-5.0	even	ok
	37	C44H76N3NaO2P	732.5567	2.2	25.6	37	33.46	9.0	even	ok
	38	C34H85N3NaO5P2S	732.5577	3.5	25.6	38	15.31	-5.0	even	ok
	39	C40H74N7O3S	732.5568	2.3	25.9	39	38.69	8.0	even	ok
	40	C31H67N17NaO2	732.5556	0.6	26.0	40	63.97	7.0	even	ok
	41	C34H80N5O7P2	732.5527	-3.3	27.2	41	17.12	-1.0	even	ok
	42	C43H70N7O3	732.5535	-2.3	27.6	42	29.48	13.0	even	ok
	43	C29H62N23	732.5553	0.2	28.0	43	69.21	11.0	even	ok
	44	C42H79NNaO5S	732.5571	2.7	28.1	44	30.00	4.0	even	ok
	45	C33H77N9NaO3P2	732.5517	-4.7	28.5	45	5.97	1.0	even	ok
	46	C45H75NNaO5	732.5537	-1.9	28.8	46	34.66	9.0	even	ok

Compound Spectrum SmartFormula Report

Meas. m/z	#	Ion Formula	m/z	err [ppm]	mSigma	# mSigma	Score	rdb	e⁻ Conf	N-Rule
	47	C43H80N3P2S	732.5543	-1.2	31.3	47	55.84	7.0	even	ok
	48	C26H66N23S	732.5587	4.8	32.7	48	5.15	6.0	even	ok
	49	C31H75N13NaOS2	732.5551	-0.1	32.9	49	80.22	1.0	even	ok
	50	C33H74N13OS2	732.5575	3.2	33.2	50	19.16	4.0	even	ok
	51	C31H80N7NaO6PS	732.5521	-4.2	33.6	51	7.80	-4.0	even	ok
	52	C35H84N5O2P2S2	732.5536	-2.1	33.7	52	35.29	-2.0	even	ok
	53	C29H75N13O4PS	732.5518	-4.6	33.7	53	5.84	0.0	even	ok
	54	C33H79N3NaO12	732.5556	0.6	33.9	54	52.56	-4.0	even	ok
	55	C38H85N3NaP2S2	732.5552	0.1	35.2	55	74.52	-1.0	even	ok
	56	C31H74N9O10	732.5553	0.2	35.2	56	57.64	0.0	even	ok
	57	C30H81N9NaO3P2S	732.5551	-0.1	35.3	57	58.81	-4.0	even	ok
	58	C35H79N7NaO3S2	732.5578	3.6	35.3	58	14.27	0.0	even	ok
	59	C43H75N5NaOS	732.5585	4.5	35.4	59	7.60	9.0	even	ok
	60	C32H83N3O10PS	732.5531	-2.8	35.5	60	18.95	-6.0	even	ok
	61	C28H76N15OP2S	732.5548	-0.5	35.7	61	51.43	0.0	even	ok
	62	C30H71N13NaO6	732.5542	-1.2	36.0	62	39.14	2.0	even	ok
	63	C32H78N9O5S2	732.5562	1.4	36.4	63	45.20	-1.0	even	ok
	64	C31H84N5O7P2S	732.5561	1.3	36.7	64	37.23	-6.0	even	ok
	65	C37H79N7OPS2	732.5520	-4.4	37.0	65	8.09	3.0	even	ok
	66	C27H75N17PS2	732.5565	1.8	37.1	66	36.94	0.0	even	ok
	67	C32H80N5NaO9P	732.5586	4.7	37.5	67	5.01	-4.0	even	ok
	68	C28H66N19O4	732.5540	-1.6	37.6	68	31.90	6.0	even	ok
	69	C34H88NO6P2S2	732.5523	-3.9	37.7	69	10.84	-7.0	even	ok
	70	C28H74N15O3S2	732.5535	-2.3	37.9	70	29.15	0.0	even	ok
	71	C29H80N11NaO2PS2	732.5568	2.2	38.0	71	29.98	-4.0	even	ok
	72	C34H83N3NaO7S2	732.5565	1.8	38.1	72	36.53	-5.0	even	ok
	73	C30H75N11O7P	732.5583	4.3	38.9	73	6.43	0.0	even	ok
	74	C27H63N23Na	732.5529	-3.1	39.0	74	14.41	8.0	even	ok
	75	C36H82N3O7S2	732.5589	5.1	39.0	75	4.40	-2.0	even	ok
	76	C30H79N9NaO5S2	732.5538	-1.9	39.1	76	34.08	-4.0	even	ok
	77	C39H84NNaO3PS2	732.5522	-4.0	39.3	77	10.02	-1.0	even	ok
	78	C29H72N15NaO3P	732.5572	2.8	40.0	78	16.14	2.0	even	ok
	79	C46H71N5NaO	732.5551	-0.1	40.5	79	51.75	14.0	even	ok
	80	C47H74NO5	732.5562	1.4	40.8	80	32.82	12.0	even	ok
	81	C30H83N7O6PS2	732.5578	3.7	41.0	81	11.85	-6.0	even	ok
	82	C40H84N3P2S2	732.5576	3.4	41.2	82	14.02	2.0	even	ok
	83	C27H67N21OP	732.5570	2.5	41.7	83	18.99	6.0	even	ok
	84	C32H88NNaO8PS2	732.5581	4.0	42.1	84	8.85	-10.0	even	ok
	85	C31H82N5O9S2	732.5548	-0.4	42.3	85	55.75	-6.0	even	ok
	86	C24H67N23NaS	732.5563	1.5	43.6	86	28.27	3.0	even	ok
	87	C27H75N13NaO6S	732.5576	3.4	44.0	87	10.59	-3.0	even	ok
	88	C25H70N19O4S	732.5573	3.0	44.4	88	13.17	1.0	even	ok
	89	C28H78N9O10S	732.5587	4.8	45.3	89	3.62	-5.0	even	ok
	90	C41H83NO3PS2	732.5547	-0.7	45.7	90	46.04	2.0	even	ok
	91	C27H78N11O7S2	732.5522	-4.1	46.1	91	7.65	-5.0	even	ok
	92	C30H78N5O14	732.5540	-1.6	46.3	92	25.14	-5.0	even	ok

Compound Spectrum SmartFormula Report

Meas. m/z	#	Ion Formula	m/z	err [ppm]	mSigma	# mSigma	Score	rdb	e¯ Conf	N-Rule
	93	C28H84N7NaO6PS2	732.5554	0.4	46.6	93	50.01	-9.0	even	ok
	94	C26H77N15NaOP2S	732.5524	-3.8	46.6	94	7.31	-3.0	even	ok
	95	C29H75N9NaO10	732.5529	-3.1	46.7	95	11.70	-3.0	even	ok
	96	C29H83N5NaO9S2	732.5524	-3.7	46.9	96	9.71	-9.0	even	ok
	97	C29H85N5NaO7P2S	732.5537	-2.0	47.0	97	20.90	-9.0	even	ok
	98	C27H80N11O5P2S	732.5534	-2.4	47.4	98	17.04	-5.0	even	ok
	99	C27H70N15O8	732.5526	-3.4	48.0	99	8.88	1.0	even	ok
	100	C30H88NO11P2S	732.5548	-0.5	48.3	100	36.34	-11.0	even	ok
	101	C31H84NNaO13P	732.5572	2.9	48.5	101	12.54	-9.0	even	ok
	102	C26H67N19NaO4	732.5516	-4.9	48.9	102	3.03	3.0	even	ok
	103	C29H87N3O10PS2	732.5565	1.8	49.2	103	26.05	-11.0	even	ok
	104	C29H79N7O11P	732.5570	2.5	49.7	104	14.92	-5.0	even	ok
	105	C30H86NO13S2	732.5535	-2.2	49.7	105	20.95	-11.0	even	ok
	106	C28H76N11NaO7P	732.5559	1.0	50.3	106	28.48	-3.0	even	ok
	107	C26H71N17O5P	732.5556	0.6	51.8	107	31.30	1.0	even	ok
	108	C48H70N5O	732.5575	3.2	52.5	108	9.15	17.0	even	ok
	109	C25H68N21NaOP	732.5546	-0.8	52.9	109	28.27	3.0	even	ok
	110	C27H77N13NaO4P2	732.5589	5.1	54.2	110	2.20	-3.0	even	ok
	111	C43H78N3O2S2	732.5530	-2.9	55.5	111	11.95	7.0	even	ok
	112	C25H72N19O2P2	732.5586	4.7	55.7	112	2.85	1.0	even	ok
	113	C29H82NO18	732.5526	-3.4	57.6	113	6.63	-10.0	even	ok
	114	C28H79N5NaO14	732.5516	-4.9	57.7	114	2.32	-8.0	even	ok
	115	C28H83N3O15P	732.5556	0.6	60.8	115	23.23	-10.0	even	ok
	116	C27H80N7NaO11P	732.5546	-0.8	61.1	116	21.71	-8.0	even	ok
	117	C25H75N13O9P	732.5543	-1.2	62.4	117	17.94	-4.0	even	ok
	118	C24H72N17NaO5P	732.5532	-2.7	63.1	118	8.77	-2.0	even	ok
	119	C46H79NNaS2	732.5546	-0.7	64.5	119	24.77	8.0	even	ok
	120	C51H74NS	732.5536	-2.1	70.6	120	11.39	16.0	even	ok
	121	C48H78NS2	732.5570	2.5	74.6	121	7.62	11.0	even	ok
782.5712	1	C44H81NO8P	782.5694	-2.2	1.9	1	46.32	6.0	even	ok
	2	C43H78N5NaO4P	782.5684	-3.6	5.0	2	18.37	8.0	even	ok
	3	C43H82N3O5P2	782.5724	1.6	5.0	3	60.50	6.0	even	ok
	4	C41H73N11O2P	782.5681	-3.9	7.1	4	13.55	12.0	even	ok
	5	C42H79N7NaOP2	782.5714	0.2	8.9	5	98.61	8.0	even	ok
	6	C40H76N7O8	782.5750	4.9	11.3	6	5.71	7.0	even	ok
	7	C44H78N7OP2	782.5738	3.3	11.3	7	19.83	11.0	even	ok
	8	C46H83NNaO3P2	782.5740	3.7	11.4	8	15.35	7.0	even	ok
	9	C39H73N11NaO4	782.5739	3.5	13.0	9	16.61	9.0	even	ok
	10	C45H77N5O4P	782.5708	-0.5	13.1	10	82.77	11.0	even	ok
	11	C44H74N9NaP	782.5697	-1.9	14.3	11	44.49	13.0	even	ok
	12	C38H73N13NaOS	782.5674	-4.8	14.6	12	5.67	9.0	even	ok
	13	C41H78N9NaPS	782.5731	2.4	14.8	13	40.53	8.0	even	ok
	14	C37H68N17O2	782.5736	3.2	15.2	14	20.38	13.0	even	ok
	15	C41H83N3NaO5P2	782.5700	-1.5	15.5	15	53.05	3.0	even	ok
	16	C39H83N7NaOP2S	782.5747	4.5	15.9	16	7.00	3.0	even	ok
	17	C38H77N11O2PS	782.5715	0.4	16.1	17	82.14	7.0	even	ok

Compound Spectrum SmartFormula Report

Meas. m/z	#	Ion Formula	m/z	err [ppm]	mSigma	# mSigma	Score	rdb	e¯ Conf	N-Rule
	18	C40H82N5NaO4PS	782.5717	0.7	16.2	18	89.74	3.0	even	ok
	19	C46H76N3O7	782.5678	-4.3	16.4	19	8.30	11.0	even	ok
	20	C40H72N13OS	782.5698	-1.7	16.9	20	56.39	12.0	even	ok
	21	C39H76N9O5S	782.5685	-3.5	17.0	21	20.06	7.0	even	ok
	22	C39H78N9O3P2	782.5697	-1.8	17.5	22	42.73	7.0	even	ok
	23	C42H81N5O4PS	782.5741	3.8	18.4	23	15.15	6.0	even	ok
	24	C41H81N3NaO7S	782.5687	-3.1	18.9	24	24.65	3.0	even	ok
	25	C42H77N7NaO3S	782.5701	-1.4	19.2	25	63.77	8.0	even	ok
	26	C41H85NO8PS	782.5728	2.1	19.8	26	44.37	1.0	even	ok
	27	C34H73N17PS	782.5688	-3.1	22.2	27	18.77	8.0	even	ok
	28	C36H78N11NaO2PS	782.5690	-2.7	22.3	28	23.51	4.0	even	ok
	29	C37H81N7O6PS	782.5701	-1.3	22.4	29	60.76	2.0	even	ok
	30	C39H80N3O12	782.5737	3.2	22.5	30	17.34	2.0	even	ok
	31	C43H80N3O7S	782.5711	-0.0	22.7	31	100.00	6.0	even	ok
	32	C45H83N3NaP2S	782.5675	-4.7	22.9	32	6.80	7.0	even	ok
	33	C38H77N7NaO8	782.5726	1.8	23.1	33	38.26	4.0	even	ok
	34	C38H87N3NaO5P2S	782.5734	2.8	23.1	34	26.63	-2.0	even	ok
	35	C39H86NNaO8PS	782.5704	-1.0	23.1	35	69.85	-2.0	even	ok
	36	C36H72N13O6	782.5723	1.5	24.7	36	43.87	8.0	even	ok
	37	C46H73N9P	782.5721	1.2	25.0	37	48.87	16.0	even	ok
	38	C36H82N9O3P2S	782.5731	2.5	25.3	38	25.19	2.0	even	ok
	39	C48H78N3NaO2P	782.5724	1.6	25.9	39	40.62	12.0	even	ok
	40	C35H69N17NaO2	782.5712	0.1	26.3	40	72.21	10.0	even	ok
	41	C44H76N7O3S	782.5725	1.7	27.4	41	46.09	11.0	even	ok
	42	C38H82N5O7P2	782.5684	-3.5	27.5	42	12.01	2.0	even	ok
	43	C47H72N7O3	782.5691	-2.6	28.1	43	21.69	16.0	even	ok
	44	C33H64N23	782.5710	-0.3	28.3	44	64.82	14.0	even	ok
	45	C37H79N9NaO3P2	782.5673	-4.9	28.9	45	3.85	4.0	even	ok
	46	C49H77NNaO5	782.5694	-2.3	29.4	46	25.90	12.0	even	ok
	47	C46H81NNaO5S	782.5728	2.0	29.6	47	36.35	7.0	even	ok
	48	C36H85N3O10PS	782.5688	-3.1	31.8	48	18.90	-3.0	even	ok
	49	C32H73N17NaO2S	782.5746	4.4	32.2	49	5.50	5.0	even	ok
	50	C47H82N3P2S	782.5699	-1.6	32.7	50	42.33	10.0	even	ok
	51	C35H82N7NaO6PS	782.5677	-4.4	33.5	51	5.24	-1.0	even	ok
	52	C33H77N13O4PS	782.5674	-4.8	33.7	52	3.84	3.0	even	ok
	53	C37H81N3NaO12	782.5712	0.1	34.2	53	59.35	-1.0	even	ok
	54	C39H86N5O2P2S2	782.5693	-2.4	34.5	54	25.98	1.0	even	ok
	55	C34H83N9NaO3P2S	782.5707	-0.6	35.2	55	48.66	-1.0	even	ok
	56	C35H76N9O10	782.5710	-0.3	35.5	56	54.43	3.0	even	ok
	57	C32H78N15OP2S	782.5704	-1.0	35.7	57	41.78	3.0	even	ok
	58	C30H68N23S	782.5743	4.0	35.9	58	6.68	9.0	even	ok
	59	C37H86N5NaO4PS2	782.5751	5.0	36.2	59	3.56	-2.0	even	ok
	60	C39H81N7NaO3S2	782.5735	2.9	36.4	60	18.34	3.0	even	ok
	61	C34H73N13NaO6	782.5699	-1.6	36.4	61	30.39	5.0	even	ok
	62	C42H87N3NaP2S2	782.5709	-0.4	36.6	62	63.90	2.0	even	ok
	63	C35H86N5O7P2S	782.5718	0.8	36.6	63	43.87	-3.0	even	ok

Compound Spectrum SmartFormula Report

Meas. m/z	#	Ion Formula	m/z	err [ppm]	mSigma	# mSigma	Score	rdb	e⁻ Conf	N-Rule
	64	C36H80N9O5S2	782.5718	0.9	36.9	64	52.65	2.0	even	ok
	65	C47H77N5NaOS	782.5741	3.8	37.0	65	10.11	12.0	even	ok
	66	C36H82N5NaO9P	782.5742	3.9	37.9	66	6.95	-1.0	even	ok
	67	C50H77N3O2P	782.5748	4.6	37.9	67	3.89	15.0	even	ok
	68	C38H90NO6P2S2	782.5679	-4.1	38.0	68	7.32	-4.0	even	ok
	69	C32H68N19O4	782.5696	-2.0	38.1	69	24.26	9.0	even	ok
	70	C41H81N7OPS2	782.5676	-4.5	38.5	70	5.17	6.0	even	ok
	71	C38H85N3NaO7S2	782.5721	1.2	38.7	71	43.25	-2.0	even	ok
	72	C35H77N13NaOS2	782.5708	-0.5	39.1	72	56.63	4.0	even	ok
	73	C34H77N11O7P	782.5740	3.6	39.4	73	8.71	3.0	even	ok
	74	C31H65N23Na	782.5685	-3.3	39.6	74	10.13	11.0	even	ok
	75	C40H84N3O7S2	782.5745	4.3	40.1	75	6.13	1.0	even	ok
	76	C35H81N11O2PS2	782.5748	4.7	40.1	76	4.41	2.0	even	ok
	77	C37H76N13OS2	782.5732	2.6	40.3	77	20.55	7.0	even	ok
	78	C34H85N3NaO12S	782.5746	4.4	40.4	78	5.49	-6.0	even	ok
	79	C32H80N9O10S	782.5743	4.1	40.5	79	7.31	-2.0	even	ok
	80	C33H74N15NaO3P	782.5729	2.2	40.6	80	20.14	5.0	even	ok
	81	C43H86NNaO3PS2	782.5679	-4.2	40.9	81	6.53	2.0	even	ok
	82	C34H85N7O6PS2	782.5735	3.0	41.0	82	15.72	-3.0	even	ok
	83	C50H73N5NaO	782.5707	-0.6	41.2	83	42.06	17.0	even	ok
	84	C31H77N17PS2	782.5721	1.2	41.6	84	39.41	3.0	even	ok
	85	C51H76NO5	782.5718	0.8	41.7	85	37.66	15.0	even	ok
	86	C36H90NNaO8PS2	782.5738	3.3	42.1	86	11.98	-7.0	even	ok
	87	C35H84N5O9S2	782.5705	-0.9	42.3	87	45.44	-3.0	even	ok
	88	C31H69N21OP	782.5726	1.8	42.3	88	23.18	9.0	even	ok
	89	C33H82N11NaO2PS2	782.5724	1.6	42.6	89	32.42	-1.0	even	ok
	90	C32H76N15O3S2	782.5691	-2.6	42.7	90	19.05	3.0	even	ok
	91	C44H86N3P2S2	782.5733	2.7	43.1	91	17.38	5.0	even	ok
	92	C34H90NO11P2S	782.5704	-0.9	43.2	92	42.89	-8.0	even	ok
	93	C31H77N13NaO6S	782.5733	2.7	43.9	93	13.82	0.0	even	ok
	94	C34H81N9NaO5S2	782.5694	-2.2	44.0	94	22.60	-1.0	even	ok
	95	C29H72N19O4S	782.5730	2.3	44.4	95	16.82	4.0	even	ok
	96	C28H69N23NaS	782.5719	1.0	45.9	96	31.44	6.0	even	ok
	97	C33H85N5NaO9S2	782.5681	-3.9	46.6	97	6.75	-6.0	even	ok
	98	C34H80N5O14	782.5696	-2.0	46.7	98	19.17	-2.0	even	ok
	99	C30H79N15NaOP2S	782.5680	-4.0	46.8	99	5.00	0.0	even	ok
	100	C29H78N17NaPS2	782.5697	-1.8	46.9	100	25.58	0.0	even	ok
	101	C33H87N5NaO7P2S	782.5694	-2.3	46.9	101	15.86	-6.0	even	ok
	102	C33H77N9NaO10	782.5686	-3.3	47.2	102	8.24	0.0	even	ok
	103	C31H82N11O5P2S	782.5691	-2.7	47.4	103	12.66	-2.0	even	ok
	104	C45H85NO3PS2	782.5703	-1.1	47.7	104	35.02	5.0	even	ok
	105	C31H72N15O8	782.5683	-3.7	48.6	105	6.11	4.0	even	ok
	106	C32H90N5O7P2S2	782.5751	5.1	48.7	106	2.40	-8.0	even	ok
	107	C33H89N3O10PS2	782.5722	1.3	48.9	107	31.68	-8.0	even	ok
	108	C35H86NNaO13P	782.5729	2.2	49.0	108	15.70	-6.0	even	ok
	109	C29H82N15OP2S2	782.5738	3.4	49.3	109	9.51	-2.0	even	ok

Compound Spectrum SmartFormula Report

Meas. m/z	#	Ion Formula	m/z	err [ppm]	mSigma	# mSigma	Score	rdb	e¯ Conf	N-Rule
	110	C30H81N13O4PS2	782.5708	-0.5	49.3	110	42.94	-2.0	even	ok
	111	C34H88NO13S2	782.5692	-2.6	49.5	111	15.76	-8.0	even	ok
	112	C30H69N19NaO4	782.5672	-5.1	49.6	112	1.91	6.0	even	ok
	113	C31H80N11O7S2	782.5678	-4.3	49.8	113	4.62	-2.0	even	ok
	114	C31H87N9NaO3P2S2	782.5741	3.7	49.8	114	7.23	-6.0	even	ok
	115	C32H86N7NaO6PS2	782.5711	-0.1	50.1	115	47.41	-6.0	even	ok
	116	C33H81N7O11P	782.5726	1.9	50.3	116	18.25	-2.0	even	ok
	117	C31H84N5O14S	782.5730	2.3	50.9	117	17.21	-7.0	even	ok
	118	C32H78N11NaO7P	782.5716	0.5	51.0	118	32.34	0.0	even	ok
	119	C30H73N17O5P	782.5713	0.1	52.5	119	34.84	4.0	even	ok
	120	C52H72N5O	782.5731	2.5	53.5	120	11.45	20.0	even	ok
	121	C29H70N21NaOP	782.5702	-1.2	53.7	121	22.05	6.0	even	ok
	122	C31H79N13NaO4P2	782.5745	4.3	55.0	122	3.08	0.0	even	ok
	123	C31H90N3NaO10PS2	782.5697	-1.8	55.4	123	19.81	-11.0	even	ok
	124	C26H77N19O2PS2	782.5681	-3.9	55.5	124	5.26	-1.0	even	ok
	125	C30H81N9NaO10S	782.5719	1.0	55.6	125	23.19	-5.0	even	ok
	126	C28H82N13NaO4PS2	782.5684	-3.5	55.9	126	6.79	-5.0	even	ok
	127	C28H76N15O8S	782.5717	0.6	56.1	127	26.23	-1.0	even	ok
	128	C29H74N19O2P2	782.5743	4.0	56.6	128	3.89	4.0	even	ok
	129	C27H73N19NaO4S	782.5706	-0.7	57.1	129	24.18	1.0	even	ok
	130	C25H68N25O2S	782.5703	-1.1	57.7	130	20.49	5.0	even	ok
	131	C47H80N3O2S2	782.5686	-3.2	57.9	131	7.95	10.0	even	ok
	132	C29H82N11NaO7PS	782.5749	4.8	57.9	132	1.85	-5.0	even	ok
	133	C29H85N9O8PS2	782.5695	-2.2	58.0	133	15.05	-7.0	even	ok
	134	C33H84NO18	782.5683	-3.7	58.1	134	4.58	-7.0	even	ok
	135	C32H81N5NaO14	782.5672	-5.0	58.3	135	1.47	-5.0	even	ok
	136	C32H91NNaO11P2S	782.5680	-4.0	58.6	136	3.48	-11.0	even	ok
	137	C27H77N17O5PS	782.5746	4.4	58.6	137	2.46	-1.0	even	ok
	138	C30H86N7O9P2S	782.5677	-4.4	59.1	138	2.56	-7.0	even	ok
	139	C26H74N21NaOPS	782.5736	3.1	59.6	139	6.62	1.0	even	ok
	140	C29H85N5NaO14S	782.5706	-0.7	60.2	140	27.39	-10.0	even	ok
	141	C30H88NO18S	782.5717	0.6	61.3	141	27.48	-12.0	even	ok
	142	C32H85N3O15P	782.5713	0.1	61.4	142	25.97	-7.0	even	ok
	143	C31H82N7NaO11P	782.5702	-1.2	61.8	143	16.99	-5.0	even	ok
	144	C29H77N13O9P	782.5699	-1.6	63.2	144	13.74	-1.0	even	ok
	145	C28H74N17NaO5P	782.5689	-2.9	64.0	145	6.21	1.0	even	ok
	146	C31H86N5O12P2	782.5743	4.0	64.9	146	2.90	-7.0	even	ok
	147	C30H83N9NaO8P2	782.5732	2.6	65.5	147	7.27	-5.0	even	ok
	148	C26H69N23O3P	782.5686	-3.3	65.6	148	4.63	5.0	even	ok
	149	C28H78N15O6P2	782.5729	2.2	67.0	149	8.49	-1.0	even	ok
	150	C26H77N15NaO8S	782.5692	-2.5	67.1	150	7.51	-4.0	even	ok
	151	C50H81NNaS2	782.5703	-1.2	67.1	151	18.12	11.0	even	ok
	152	C27H80N11O12S	782.5703	-1.1	67.8	152	14.53	-6.0	even	ok
	153	C27H75N19NaO2P2	782.5719	0.9	68.0	153	15.76	1.0	even	ok
	154	C23H69N25NaO2S	782.5679	-4.2	68.5	154	2.16	2.0	even	ok
	155	C24H72N21O6S	782.5690	-2.8	69.1	155	5.61	0.0	even	ok

Compound Spectrum SmartFormula Report

Meas. m/z	#	Ion Formula	m/z	err [ppm]	mSigma	# mSigma	Score	rdb	e⁻ Conf	N-Rule
	156	C25H70N25P2	782.5716	0.5	69.6	156	17.03	5.0	even	ok
	157	C30H86N3NaO15P	782.5689	-2.9	72.8	157	4.54	-10.0	even	ok
	158	C55H76NS	782.5693	-2.4	72.9	158	7.87	19.0	even	ok
	159	C28H81N9O13P	782.5686	-3.3	74.1	159	3.41	-6.0	even	ok
	160	C27H78N13NaO9P	782.5675	-4.6	74.6	160	1.16	-4.0	even	ok
	161	C24H69N23NaO5	782.5744	4.2	75.6	161	1.68	2.0	even	ok
	162	C30H90NO16P2	782.5729	2.3	75.9	162	6.02	-12.0	even	ok
	163	C25H73N19O7P	782.5672	-5.0	76.0	163	0.80	0.0	even	ok
	164	C29H87N5NaO12P2	782.5719	0.9	76.3	164	11.43	-10.0	even	ok
	165	C22H64N29O3	782.5741	3.8	77.1	165	2.09	6.0	even	ok
	166	C52H80NS2	782.5727	1.9	77.6	166	8.47	14.0	even	ok
806.5927	1	C44H81N5O6P	806.5919	-1.0	1.1	1	81.52	8.0	even	ok
	2	C45H87NNaO5P2	806.5952	3.1	3.6	2	23.59	4.0	even	ok
	3	C45H80N3O9	806.5889	-4.7	4.4	3	6.61	8.0	even	ok
	4	C43H78N9NaO2P	806.5908	-2.3	4.7	4	39.22	10.0	even	ok
	5	C43H82N7O3P2	806.5949	2.8	6.0	5	28.63	8.0	even	ok
	6	C41H73N15P	806.5906	-2.6	7.2	6	30.36	14.0	even	ok
	7	C46H83N5NaOP2	806.5965	4.8	9.5	7	5.54	9.0	even	ok
	8	C45H77N9O2P	806.5932	0.7	11.2	8	75.75	13.0	even	ok
	9	C47H82N3NaO4P	806.5935	1.0	12.2	9	64.31	9.0	even	ok
	10	C43H85NO10P	806.5906	-2.6	12.6	10	27.70	3.0	even	ok
	11	C42H82N5NaO6P	806.5895	-3.9	12.9	11	10.59	5.0	even	ok
	12	C45H73N11NaO	806.5892	-4.3	14.5	12	7.39	15.0	even	ok
	13	C46H76N7O5	806.5902	-3.0	14.5	13	20.60	13.0	even	ok
	14	C40H77N11O4P	806.5892	-4.3	14.7	14	7.66	9.0	even	ok
	15	C39H73N15NaO2	806.5964	4.6	14.7	15	5.74	11.0	even	ok
	16	C42H86N3O7P2	806.5936	1.1	16.0	16	58.42	3.0	even	ok
	17	C48H81NNaO7	806.5905	-2.7	16.2	17	25.09	9.0	even	ok
	18	C37H68N21	806.5961	4.3	16.8	18	7.46	15.0	even	ok
	19	C40H82N9NaO2PS	806.5942	1.9	16.9	19	48.35	5.0	even	ok
	20	C38H77N15PS	806.5939	1.6	17.0	20	46.19	9.0	even	ok
	21	C41H83N7NaO3P2	806.5925	-0.2	17.3	21	80.09	5.0	even	ok
	22	C39H76N13O3S	806.5909	-2.2	17.5	22	41.42	9.0	even	ok
	23	C44H87N3NaO2P2S	806.5886	-5.0	17.8	23	4.72	4.0	even	ok
	24	C42H81N9O2PS	806.5966	4.9	18.1	24	5.21	8.0	even	ok
	25	C42H77N11NaOS	806.5925	-0.2	18.6	25	100.00	10.0	even	ok
	26	C41H81N7NaO5S	806.5912	-1.8	19.0	26	48.59	5.0	even	ok
	27	C39H78N13OP2	806.5922	-0.6	19.3	27	67.70	9.0	even	ok
	28	C41H85N5O6PS	806.5953	3.2	20.1	28	19.72	3.0	even	ok
	29	C43H80N7O5S	806.5936	1.2	22.0	29	62.15	8.0	even	ok
	30	C42H84N3O9S	806.5923	-0.5	22.7	30	81.41	3.0	even	ok
	31	C41H85NNaO12	806.5964	4.6	23.4	31	4.72	0.0	even	ok
	32	C37H81N11O4PS	806.5926	-0.1	23.7	32	90.88	4.0	even	ok
	33	C38H80N9O7S	806.5896	-3.8	24.0	33	11.61	4.0	even	ok
	34	C36H78N15NaPS	806.5915	-1.4	24.0	34	42.20	6.0	even	ok
	35	C48H78N7NaP	806.5949	2.7	24.0	35	20.53	14.0	even	ok

Compound Spectrum SmartFormula Report

Meas. m/z	#	Ion Formula	m/z	err [ppm]	mSigma	# mSigma	Score	rdb	e⁻ Conf	N-Rule
	36	C39H86N5NaO6PS	806.5929	0.2	24.1	36	85.95	0.0	even	ok
	37	C45H85NNaO7S	806.5939	1.5	24.3	37	50.08	4.0	even	ok
	38	C49H81N3O4P	806.5959	4.0	24.3	38	7.71	12.0	even	ok
	39	C39H80N7O10	806.5961	4.3	24.5	39	6.26	4.0	even	ok
	40	C46H86N3O2P2S	806.5910	-2.0	24.7	40	38.50	7.0	even	ok
	41	C40H85N3NaO9S	806.5899	-3.5	24.8	41	14.90	0.0	even	ok
	42	C38H77N11NaO6	806.5950	3.0	25.1	42	17.07	6.0	even	ok
	43	C47H72N11O	806.5916	-1.3	26.3	43	41.66	18.0	even	ok
	44	C44H76N11OS	806.5950	2.8	26.4	44	22.41	13.0	even	ok
	45	C36H72N17O4	806.5948	2.6	26.7	45	20.63	10.0	even	ok
	46	C40H89NO10PS	806.5939	1.6	26.8	46	46.24	-2.0	even	ok
	47	C36H82N13OP2S	806.5956	3.6	27.0	47	10.20	4.0	even	ok
	48	C38H87N7NaO3P2S	806.5959	3.9	27.2	48	7.73	0.0	even	ok
	49	C49H77N5NaO3	806.5919	-1.0	27.6	49	47.25	14.0	even	ok
	50	C40H87N3NaO7P2	806.5911	-1.9	28.0	50	30.47	0.0	even	ok
	51	C35H69N21Na	806.5937	1.3	28.2	51	41.08	12.0	even	ok
	52	C50H80NO7	806.5929	0.3	28.5	52	60.38	12.0	even	ok
	53	C46H81N5NaO3S	806.5952	3.2	28.5	53	16.82	9.0	even	ok
	54	C38H82N9O5P2	806.5909	-2.2	29.5	54	24.17	4.0	even	ok
	55	C37H79N13NaOP2	806.5898	-3.6	30.8	55	9.63	6.0	even	ok
	56	C47H84NO7S	806.5963	4.5	31.6	56	5.41	7.0	even	ok
	57	C36H85N7O8PS	806.5912	-1.8	33.3	57	35.68	-1.0	even	ok
	58	C38H90NNaO10PS	806.5915	-1.4	33.4	58	42.39	-5.0	even	ok
	59	C49H87NNaP2S	806.5927	-0.0	33.6	59	74.26	8.0	even	ok
	60	C37H91N3NaO7P2S	806.5945	2.3	34.3	60	25.92	-5.0	even	ok
	61	C39H86N9P2S2	806.5917	-1.2	34.8	61	46.16	3.0	even	ok
	62	C37H87N9NaP2S2	806.5893	-4.1	35.2	62	6.77	0.0	even	ok
	63	C35H82N11NaO4PS	806.5902	-3.1	35.3	63	12.12	1.0	even	ok
	64	C33H77N17O2PS	806.5899	-3.4	35.5	64	9.39	5.0	even	ok
	65	C38H84N3O14	806.5948	2.6	36.1	65	16.23	-1.0	even	ok
	66	C48H81N5OPS	806.5894	-4.1	36.1	66	7.09	12.0	even	ok
	67	C37H81N7NaO10	806.5937	1.3	36.2	67	33.41	1.0	even	ok
	68	C52H83NNaP2	806.5893	-4.2	36.3	68	5.08	13.0	even	ok
	69	C39H81N11NaOS2	806.5959	4.0	36.5	69	7.19	5.0	even	ok
	70	C41H91N3NaO2P2S2	806.5920	-0.8	36.8	70	50.86	-1.0	even	ok
	71	C34H83N13NaOP2S	806.5932	0.6	37.2	71	43.49	1.0	even	ok
	72	C36H80N13O3S2	806.5943	2.0	37.5	72	27.82	4.0	even	ok
	73	C35H76N13O8	806.5934	0.9	37.6	73	37.62	5.0	even	ok
	74	C40H85N7O3PS2	806.5887	-4.9	38.1	74	3.28	3.0	even	ok
	75	C35H86N9O5P2S	806.5942	1.9	38.4	75	22.67	-1.0	even	ok
	76	C34H73N17NaO4	806.5924	-0.4	38.5	76	45.99	7.0	even	ok
	77	C38H90N5O4P2S2	806.5904	-2.8	38.7	77	16.66	-2.0	even	ok
	78	C32H76N19OS2	806.5916	-1.3	38.9	78	38.70	5.0	even	ok
	79	C33H82N15NaPS2	806.5949	2.7	39.0	79	17.22	1.0	even	ok
	80	C38H85N7NaO5S2	806.5946	2.4	39.2	80	21.75	0.0	even	ok
	81	C36H82N9NaO7P	806.5967	5.0	40.0	81	2.19	1.0	even	ok

Compound Spectrum SmartFormula Report

Meas. m/z	#	Ion Formula	m/z	err [ppm]	mSigma	# mSigma	Score	rdb	e¯ Conf	N-Rule
	82	C51H76N5O3	806.5943	2.0	40.0	82	21.30	17.0	even	ok
	83	C34H81N13NaO3S2	806.5919	-1.0	40.0	83	43.85	1.0	even	ok
	84	C43H90N3O2P2S2	806.5944	2.2	40.1	84	23.85	2.0	even	ok
	85	C32H68N23O2	806.5921	-0.7	40.1	85	38.52	11.0	even	ok
	86	C42H90NNaO5PS2	806.5890	-4.5	40.2	86	4.26	-1.0	even	ok
	87	C37H86N5O9P2	806.5895	-3.9	40.5	87	5.76	-1.0	even	ok
	88	C43H86N5NaOPS2	806.5904	-2.9	40.6	88	15.34	4.0	even	ok
	89	C34H77N15O5P	806.5964	4.7	41.5	89	2.91	5.0	even	ok
	90	C34H85N11O4PS2	806.5960	4.1	42.0	90	5.97	-1.0	even	ok
	91	C34H86N7NaO8PS	806.5888	-4.7	42.0	91	3.30	-4.0	even	ok
	92	C33H74N19NaOP	806.5954	3.3	42.6	92	8.36	7.0	even	ok
	93	C39H88N3O9S2	806.5956	3.7	42.8	93	7.90	-2.0	even	ok
	94	C36H90N5NaO6PS2	806.5962	4.4	43.1	94	4.30	-5.0	even	ok
	95	C35H84N9O7S2	806.5930	0.4	43.2	95	50.70	-1.0	even	ok
	96	C35H89N3O12PS	806.5899	-3.4	43.3	96	9.60	-6.0	even	ok
	97	C51H86NP2S	806.5951	3.0	44.1	97	12.85	11.0	even	ok
	98	C44H89NO5PS2	806.5914	-1.5	44.3	98	29.79	2.0	even	ok
	99	C37H89N3NaO9S2	806.5932	0.7	44.5	99	42.83	-5.0	even	ok
	100	C30H77N19NaOS2	806.5892	-4.3	44.5	100	4.63	2.0	even	ok
	101	C34H90N5O9P2S	806.5929	0.3	44.8	101	49.83	-6.0	even	ok
	102	C37H94NO8P2S2	806.5891	-4.5	44.9	102	3.89	-7.0	even	ok
	103	C46H91NNaP2S2	806.5960	4.2	45.0	103	5.03	3.0	even	ok
	104	C31H77N17NaO4S	806.5957	3.8	45.8	104	5.33	2.0	even	ok
	105	C29H72N23O2S	806.5955	3.5	46.4	105	6.85	6.0	even	ok
	106	C30H81N17O2PS2	806.5933	0.7	47.0	106	39.32	0.0	even	ok
	107	C31H80N15O5S2	806.5903	-3.0	47.0	107	11.93	0.0	even	ok
	108	C32H81N13O6PS	806.5886	-5.1	47.2	108	1.64	0.0	even	ok
	109	C45H85N5OPS2	806.5928	0.1	47.2	109	49.07	7.0	even	ok
	110	C32H86N11NaO4PS2	806.5935	1.1	47.5	110	33.40	-4.0	even	ok
	111	C36H85N3NaO14	806.5924	-0.4	47.7	111	35.64	-4.0	even	ok
	112	C31H87N13NaOP2S2	806.5965	4.8	47.7	112	2.67	-4.0	even	ok
	113	C33H85N9NaO7S2	806.5906	-2.6	47.8	113	14.64	-4.0	even	ok
	114	C45H81N7NaS2	806.5887	-4.9	48.6	114	2.34	9.0	even	ok
	115	C54H82NP2	806.5917	-1.2	48.7	115	24.58	16.0	even	ok
	116	C34H80N9O12	806.5921	-0.7	48.8	116	30.15	0.0	even	ok
	117	C33H87N9NaO5P2S	806.5918	-1.0	48.9	117	26.28	-4.0	even	ok
	118	C33H77N13NaO8	806.5910	-2.0	49.3	118	15.79	2.0	even	ok
	119	C31H82N15O3P2S	806.5916	-1.4	49.4	119	22.05	0.0	even	ok
	120	C33H89N7O8PS2	806.5946	2.4	50.1	120	15.47	-6.0	even	ok
	121	C34H88N5O11S2	806.5916	-1.3	50.6	121	27.83	-6.0	even	ok
	122	C31H72N19O6	806.5907	-2.4	50.8	122	12.36	6.0	even	ok
	123	C35H94NNaO10PS2	806.5949	2.8	50.8	123	12.14	-10.0	even	ok
	124	C36H89NO15P	806.5964	4.7	50.8	124	2.19	-6.0	even	ok
	125	C35H86N5NaO11P	806.5954	3.3	51.1	125	6.45	-4.0	even	ok
	126	C30H69N23NaO2	806.5897	-3.7	51.8	126	4.80	8.0	even	ok
	127	C46H84N3O4S2	806.5898	-3.6	51.9	127	6.56	7.0	even	ok

Compound Spectrum SmartFormula Report

Meas. m/z	#	Ion Formula	m/z	err [ppm]	mSigma	# mSigma	Score	rdb	e¯ Conf	N-Rule
	128	C33H89N3NaO14S	806.5957	3.8	52.2	128	5.43	-9.0	even	ok
	129	C33H81N11O9P	806.5951	3.0	52.4	129	7.91	0.0	even	ok
	130	C31H84N9O12S	806.5955	3.5	52.6	130	7.03	-5.0	even	ok
	131	C32H78N15NaO5P	806.5940	1.7	53.2	131	17.04	2.0	even	ok
	132	C54H77N3NaO	806.5959	4.0	53.3	132	3.66	18.0	even	ok
	133	C28H64N29	806.5894	-4.1	53.4	133	3.45	12.0	even	ok
	134	C28H82N17NaO2PS2	806.5909	-2.2	54.5	134	15.00	-3.0	even	ok
	135	C30H73N21O3P	806.5937	1.3	54.7	135	19.23	6.0	even	ok
	136	C33H94NO13P2S	806.5916	-1.4	55.2	136	23.17	-11.0	even	ok
	137	C30H84N11O9S2	806.5889	-4.6	56.0	137	2.43	-5.0	even	ok
	138	C29H85N13O6PS2	806.5919	-0.9	56.4	138	27.36	-5.0	even	ok
	139	C32H89N5NaO11S2	806.5892	-4.3	56.5	139	3.24	-9.0	even	ok
	140	C31H90N7NaO8PS2	806.5922	-0.6	56.8	140	31.10	-9.0	even	ok
	141	C47H80N7S2	806.5911	-1.9	57.2	141	16.32	12.0	even	ok
	142	C26H77N23PS2	806.5906	-2.6	57.3	142	11.10	1.0	even	ok
	143	C27H73N23NaO2S	806.5931	0.5	57.5	143	25.18	3.0	even	ok
	144	C30H81N13NaO8S	806.5944	2.1	57.6	144	11.45	-3.0	even	ok
	145	C28H76N19O6S	806.5941	1.8	58.1	145	13.60	1.0	even	ok
	146	C25H68N29S	806.5928	0.1	58.3	146	27.69	7.0	even	ok
	147	C29H74N23P2	806.5967	5.0	58.8	147	1.20	6.0	even	ok
	148	C33H92NO15S2	806.5903	-2.9	59.0	148	8.25	-11.0	even	ok
	149	C32H93N3O12PS2	806.5933	0.8	59.0	149	26.77	-11.0	even	ok
	150	C49H85NNaO2S2	806.5914	-1.6	59.6	150	18.08	8.0	even	ok
	151	C33H84N5O16	806.5908	-2.4	60.2	151	9.19	-5.0	even	ok
	152	C32H81N9NaO12	806.5897	-3.7	60.5	152	3.66	-3.0	even	ok
	153	C29H83N15NaO3P2S	806.5891	-4.4	60.5	153	2.10	-3.0	even	ok
	154	C32H91N5NaO9P2S	806.5905	-2.7	60.6	154	7.40	-9.0	even	ok
	155	C30H86N11O7P2S	806.5902	-3.0	61.2	155	5.76	-5.0	even	ok
	156	C27H78N21OP2S	806.5889	-4.7	61.3	156	1.49	1.0	even	ok
	157	C30H76N15O10	806.5894	-4.0	61.7	157	2.65	1.0	even	ok
	158	C29H85N9NaO12S	806.5931	0.5	62.0	158	26.86	-8.0	even	ok
	159	C54H80NO2S	806.5904	-2.8	62.2	159	8.29	16.0	even	ok
	160	C34H90NNaO15P	806.5940	1.7	62.4	160	12.48	-9.0	even	ok
	161	C30H88N5O16S	806.5941	1.8	62.9	161	14.34	-10.0	even	ok
	162	C32H85N7O13P	806.5937	1.3	63.6	162	14.21	-5.0	even	ok
	163	C31H82N11NaO9P	806.5927	0.0	64.0	163	23.63	-3.0	even	ok
	164	C29H77N17O7P	806.5924	-0.3	65.4	164	20.19	1.0	even	ok
	165	C28H74N21NaO3P	806.5913	-1.7	66.3	165	11.06	3.0	even	ok
	166	C31H86N9O10P2	806.5967	5.0	67.1	166	0.89	-5.0	even	ok
	167	C30H83N13NaO6P2	806.5957	3.7	67.7	167	2.77	-3.0	even	ok
	168	C26H69N27OP	806.5911	-2.0	67.8	168	8.69	7.0	even	ok
	169	C26H77N19NaO6S	806.5917	-1.2	69.2	169	12.49	-2.0	even	ok
	170	C28H78N19O4P2	806.5954	3.4	69.2	170	3.41	1.0	even	ok
	171	C51H84NO2S2	806.5938	1.4	69.2	171	14.07	11.0	even	ok
	172	C23H69N29NaS	806.5904	-2.9	69.6	172	4.84	4.0	even	ok
	173	C27H80N15O10S	806.5928	0.1	69.9	173	18.38	-4.0	even	ok

Compound Spectrum SmartFormula Report

Meas. m/z	#	Ion Formula	m/z	err [ppm]	mSigma	# mSigma	Score	rdb	e¯ Conf	N-Rule
	174	C24H72N25O4S	806.5914	-1.5	69.9	174	10.29	2.0	even	ok
	175	C27H75N23NaP2	806.5943	2.1	70.2	175	7.75	3.0	even	ok
	176	C32H88NO20	806.5894	-4.0	71.7	176	1.88	-10.0	even	ok
	177	C31H89N3O17P	806.5924	-0.3	74.8	177	14.29	-10.0	even	ok
	178	C30H86N7NaO13P	806.5913	-1.6	75.1	178	8.01	-8.0	even	ok
	179	C28H81N13O11P	806.5911	-2.0	76.4	179	6.34	-4.0	even	ok
	180	C27H78N17NaO7P	806.5900	-3.3	76.9	180	2.66	-2.0	even	ok
	181	C30H90N5O14P2	806.5954	3.4	78.2	181	2.40	-10.0	even	ok
	182	C25H73N23O5P	806.5897	-3.7	78.3	182	1.94	2.0	even	ok
	183	C29H87N9NaO10P2	806.5943	2.1	78.6	183	5.57	-8.0	even	ok
	184	C24H70N27NaOP	806.5886	-5.0	79.2	184	0.59	4.0	even	ok
	185	C22H64N33O	806.5966	4.9	79.4	185	0.65	8.0	even	ok
818.5931	1	C45H81N5O6P	818.5919	-1.5	4.3	1	63.77	9.0	even	ok
	2	C44H78N9NaO2P	818.5908	-2.8	5.9	2	29.08	11.0	even	ok
	3	C46H87NNaO5P2	818.5952	2.5	6.2	3	33.69	5.0	even	ok
	4	C47H83N5NaOP2	818.5965	4.2	7.8	4	9.61	10.0	even	ok
	5	C44H82N7O3P2	818.5949	2.2	7.9	5	40.27	9.0	even	ok
	6	C42H73N15P	818.5906	-3.1	8.2	6	22.05	15.0	even	ok
	7	C46H77N9O2P	818.5932	0.2	9.5	7	100.00	14.0	even	ok
	8	C48H82N3NaO4P	818.5935	0.5	10.6	8	86.54	10.0	even	ok
	9	C46H73N11NaO	818.5892	-4.8	12.6	9	5.00	16.0	even	ok
	10	C47H76N7O5	818.5902	-3.5	12.9	10	15.28	14.0	even	ok
	11	C49H81NNaO7	818.5905	-3.1	14.7	11	19.04	10.0	even	ok
	12	C44H85NO10P	818.5906	-3.1	15.2	12	19.46	4.0	even	ok
	13	C43H82N5NaO6P	818.5895	-4.4	15.3	13	6.78	6.0	even	ok
	14	C40H73N15NaO2	818.5964	4.0	16.7	14	9.17	12.0	even	ok
	15	C41H77N11O4P	818.5892	-4.7	16.9	15	4.79	10.0	even	ok
	16	C39H77N15PS	818.5939	1.0	17.4	16	77.09	10.0	even	ok
	17	C43H86N3O7P2	818.5936	0.6	18.5	17	72.72	4.0	even	ok
	18	C41H82N9NaO2PS	818.5942	1.4	18.6	18	64.24	6.0	even	ok
	19	C43H81N9O2PS	818.5966	4.3	18.7	19	8.73	9.0	even	ok
	20	C38H68N21	818.5961	3.7	18.7	20	11.65	16.0	even	ok
	21	C43H77N11NaOS	818.5925	-0.7	19.0	21	86.09	11.0	even	ok
	22	C40H76N13O3S	818.5909	-2.6	19.0	22	30.64	10.0	even	ok
	23	C42H83N7NaO3P2	818.5925	-0.7	19.4	23	66.08	6.0	even	ok
	24	C42H81N7NaO5S	818.5912	-2.3	20.6	24	36.69	6.0	even	ok
	25	C45H86N3NaO4PS	818.5969	4.6	20.9	25	6.11	5.0	even	ok
	26	C40H78N13OP2	818.5922	-1.1	21.3	26	54.81	10.0	even	ok
	27	C42H85N5O6PS	818.5953	2.7	21.9	27	28.57	4.0	even	ok
	28	C49H78N7NaP	818.5949	2.1	22.1	28	31.12	15.0	even	ok
	29	C50H81N3O4P	818.5959	3.5	22.6	29	12.79	13.0	even	ok
	30	C44H80N7O5S	818.5936	0.6	22.7	30	80.53	9.0	even	ok
	31	C43H84N3O9S	818.5923	-1.0	24.2	31	66.92	4.0	even	ok
	32	C48H72N11O	818.5916	-1.8	24.4	32	35.01	19.0	even	ok
	33	C47H86N3O2P2S	818.5910	-2.5	24.7	33	29.68	8.0	even	ok
	34	C46H85NNaO7S	818.5939	1.0	24.9	34	66.34	5.0	even	ok

Compound Spectrum SmartFormula Report

Meas. m/z	#	Ion Formula	m/z	err [ppm]	mSigma	# mSigma	Score	rdb	e¯ Conf	N-Rule
	35	C38H81N11O4PS	818.5926	-0.6	25.8	35	75.29	5.0	even	ok
	36	C50H77N5NaO3	818.5919	-1.5	25.8	36	40.55	15.0	even	ok
	37	C42H85NNaO12	818.5964	4.0	25.9	37	7.44	1.0	even	ok
	38	C39H80N9O7S	818.5896	-4.3	25.9	38	7.53	5.0	even	ok
	39	C45H76N11OS	818.5950	2.3	26.0	39	33.13	14.0	even	ok
	40	C40H86N5NaO6PS	818.5929	-0.3	26.4	40	84.60	1.0	even	ok
	41	C37H78N15NaPS	818.5915	-1.9	26.5	41	31.95	7.0	even	ok
	42	C39H87N7NaO3P2S	818.5959	3.4	26.7	42	15.51	1.0	even	ok
	43	C41H85N3NaO9S	818.5899	-3.9	26.8	43	9.88	1.0	even	ok
	44	C51H80NO7	818.5929	-0.2	26.9	44	68.75	13.0	even	ok
	45	C40H80N7O10	818.5961	3.7	26.9	45	9.61	5.0	even	ok
	46	C39H77N11NaO6	818.5950	2.4	27.4	46	23.90	7.0	even	ok
	47	C47H81N5NaO3S	818.5952	2.6	28.4	47	25.34	10.0	even	ok
	48	C40H90N3O7P2S	818.5969	4.7	28.8	48	4.90	-1.0	even	ok
	49	C41H89NO10PS	818.5939	1.0	28.9	49	59.24	-1.0	even	ok
	50	C37H72N17O4	818.5948	2.0	28.9	50	28.22	11.0	even	ok
	51	C37H82N13OP2S	818.5956	3.0	29.5	51	14.88	5.0	even	ok
	52	C36H69N21Na	818.5937	0.7	30.3	52	51.72	13.0	even	ok
	53	C41H87N3NaO7P2	818.5911	-2.4	30.3	53	22.38	1.0	even	ok
	54	C48H84NO7S	818.5963	3.9	31.6	54	8.95	8.0	even	ok
	55	C39H82N9O5P2	818.5909	-2.7	31.8	55	17.34	5.0	even	ok
	56	C38H79N13NaOP2	818.5898	-4.0	33.0	56	6.31	7.0	even	ok
	57	C50H87NNaP2S	818.5927	-0.5	33.0	57	65.84	9.0	even	ok
	58	C51H77N7P	818.5973	5.1	34.3	58	2.30	18.0	even	ok
	59	C53H83NNaP2	818.5893	-4.6	34.5	59	3.51	14.0	even	ok
	60	C37H85N7O8PS	818.5912	-2.3	35.5	60	26.45	0.0	even	ok
	61	C49H81N5OPS	818.5894	-4.5	35.6	61	4.79	13.0	even	ok
	62	C39H90NNaO10PS	818.5915	-1.9	35.7	62	32.07	-4.0	even	ok
	63	C40H86N9P2S2	818.5917	-1.7	35.9	63	36.58	4.0	even	ok
	64	C38H91N3NaO7P2S	818.5945	1.7	36.6	64	34.48	-4.0	even	ok
	65	C33H73N21NaS	818.5971	4.9	36.6	65	2.70	8.0	even	ok
	66	C36H86N9O5P2S	818.5942	1.4	36.7	66	40.93	0.0	even	ok
	67	C38H87N9NaP2S2	818.5893	-4.6	36.8	67	4.30	1.0	even	ok
	68	C40H81N11NaOS2	818.5959	3.4	37.4	68	11.20	6.0	even	ok
	69	C36H82N11NaO4PS	818.5902	-3.6	37.8	69	8.12	2.0	even	ok
	70	C42H91N3NaO2P2S2	818.5920	-1.3	37.8	70	41.24	0.0	even	ok
	71	C34H77N17O2PS	818.5899	-3.9	38.0	71	6.13	6.0	even	ok
	72	C52H76N5O3	818.5943	1.4	38.3	72	30.87	18.0	even	ok
	73	C39H84N3O14	818.5948	2.1	38.5	73	21.95	0.0	even	ok
	74	C38H81N7NaO10	818.5937	0.8	38.7	74	41.50	2.0	even	ok
	75	C37H80N13O3S2	818.5943	1.5	38.9	75	37.18	5.0	even	ok
	76	C35H83N13NaOP2S	818.5932	0.1	39.7	76	51.74	2.0	even	ok
	77	C36H76N13O8	818.5934	0.4	40.0	77	45.76	6.0	even	ok
	78	C39H90N5O4P2S2	818.5904	-3.3	40.2	78	11.66	-1.0	even	ok
	79	C39H85N7NaO5S2	818.5946	1.8	40.5	79	29.77	1.0	even	ok
	80	C44H90N3O2P2S2	818.5944	1.6	40.7	80	32.89	3.0	even	ok

Compound Spectrum SmartFormula Report

Meas. m/z	#	Ion Formula	m/z	err [ppm]	mSigma	# mSigma	Score	rdb	e⁻ Conf	N-Rule
	81	C35H73N17NaO4	818.5924	-0.9	40.8	81	36.98	8.0	even	ok
	82	C44H86N5NaOPS2	818.5904	-3.3	41.1	82	11.01	5.0	even	ok
	83	C41H84N7O5S2	818.5970	4.8	41.1	83	3.30	4.0	even	ok
	84	C43H90NNaO5PS2	818.5890	-5.0	41.1	84	2.67	0.0	even	ok
	85	C35H81N13NaO3S2	818.5919	-1.5	41.8	85	34.50	2.0	even	ok
	86	C37H82N9NaO7P	818.5967	4.4	42.3	86	3.53	2.0	even	ok
	87	C33H68N23O2	818.5921	-1.2	42.4	87	30.33	12.0	even	ok
	88	C38H86N5O9P2	818.5895	-4.4	42.9	88	3.63	0.0	even	ok
	89	C43H89NNaO7S2	818.5973	5.1	43.2	89	2.23	0.0	even	ok
	90	C52H86NP2S	818.5951	2.4	43.3	90	19.42	12.0	even	ok
	91	C35H85N11O4PS2	818.5960	3.5	43.8	91	9.08	0.0	even	ok
	92	C35H77N15O5P	818.5964	4.1	43.8	92	4.56	6.0	even	ok
	93	C40H88N3O9S2	818.5956	3.1	44.1	93	11.86	-1.0	even	ok
	94	C35H85N7NaO10S	818.5971	4.9	44.2	94	2.71	-3.0	even	ok
	95	C33H80N13O8S	818.5968	4.5	44.4	95	3.72	1.0	even	ok
	96	C45H89NO5PS2	818.5914	-2.0	44.8	96	23.37	3.0	even	ok
	97	C37H90N5NaO6PS2	818.5962	3.8	44.8	97	6.72	-4.0	even	ok
	98	C36H84N9O7S2	818.5930	-0.2	44.9	98	54.59	0.0	even	ok
	99	C34H74N19NaOP	818.5954	2.8	44.9	99	11.91	8.0	even	ok
	100	C47H91NNaP2S2	818.5960	3.6	45.1	100	8.08	4.0	even	ok
	101	C36H89N3O12PS	818.5899	-3.9	45.6	101	6.27	-5.0	even	ok
	102	C38H89N3NaO9S2	818.5932	0.2	46.1	102	52.25	-4.0	even	ok
	103	C34H82N15NaPS2	818.5949	2.2	46.5	103	20.32	2.0	even	ok
	104	C33H76N19OS2	818.5916	-1.8	46.6	104	25.19	6.0	even	ok
	105	C38H94NO8P2S2	818.5891	-4.9	46.6	105	2.39	-6.0	even	ok
	106	C55H82NP2	818.5917	-1.7	46.9	106	21.06	17.0	even	ok
	107	C35H90N5O9P2S	818.5929	-0.2	47.1	107	49.78	-5.0	even	ok
	108	C46H85N5OPS2	818.5928	-0.4	47.3	108	46.63	8.0	even	ok
	109	C32H77N17NaO4S	818.5957	3.2	48.3	109	7.77	3.0	even	ok
	110	C30H72N23O2S	818.5955	2.9	48.9	110	9.73	7.0	even	ok
	111	C32H80N15O5S2	818.5903	-3.4	49.0	111	8.12	1.0	even	ok
	112	C33H86N11NaO4PS2	818.5935	0.6	49.5	112	41.16	-3.0	even	ok
	113	C34H85N9NaO7S2	818.5906	-3.1	49.7	113	10.22	-3.0	even	ok
	114	C37H85N3NaO14	818.5924	-0.9	50.1	114	28.33	-3.0	even	ok
	115	C31H77N19NaOS2	818.5892	-4.7	51.2	115	2.48	3.0	even	ok
	116	C35H80N9O12	818.5921	-1.2	51.3	116	23.46	1.0	even	ok
	117	C34H87N9NaO5P2S	818.5918	-1.5	51.4	117	20.01	-3.0	even	ok
	118	C55H77N3NaO	818.5959	3.4	51.6	118	6.14	19.0	even	ok
	119	C34H77N13NaO8	818.5910	-2.5	51.7	119	11.29	3.0	even	ok
	120	C32H82N15O3P2S	818.5916	-1.9	51.9	120	16.41	1.0	even	ok
	121	C47H84N3O4S2	818.5898	-4.1	52.0	121	4.51	8.0	even	ok
	122	C34H89N7O8PS2	818.5946	1.9	52.0	122	20.75	-5.0	even	ok
	123	C35H88N5O11S2	818.5916	-1.8	52.4	123	21.24	-5.0	even	ok
	124	C36H94NNaO10PS2	818.5949	2.2	52.7	124	16.70	-9.0	even	ok
	125	C32H72N19O6	818.5907	-2.9	53.2	125	8.62	7.0	even	ok
	126	C37H89NO15P	818.5964	4.1	53.3	126	3.39	-5.0	even	ok

Compound Spectrum SmartFormula Report

Meas. m/z	#	Ion Formula	m/z	err [ppm]	mSigma	# mSigma	Score	rdb	e⁻ Conf	N-Rule
	127	C31H81N17O2PS2	818.5933	0.2	53.4	127	41.35	1.0	even	ok
	128	C36H86N5NaO11P	818.5954	2.8	53.5	128	9.08	-3.0	even	ok
	129	C32H87N13NaOP2S2	818.5965	4.2	53.9	129	3.72	-3.0	even	ok
	130	C31H69N23NaO2	818.5897	-4.2	54.1	130	3.05	9.0	even	ok
	131	C34H89N3NaO14S	818.5957	3.2	54.5	131	7.94	-8.0	even	ok
	132	C32H84N9O12S	818.5955	2.9	54.8	132	10.03	-4.0	even	ok
	133	C34H81N11O9P	818.5951	2.4	54.8	133	10.86	1.0	even	ok
	134	C33H78N15NaO5P	818.5940	1.1	55.5	134	21.44	3.0	even	ok
	135	C29H64N29	818.5894	-4.5	55.7	135	2.14	13.0	even	ok
	136	C33H91N5NaO9P2S	818.5905	-3.2	56.5	136	7.79	-8.0	even	ok
	137	C48H80N7S2	818.5911	-2.4	57.0	137	12.75	13.0	even	ok
	138	C31H73N21O3P	818.5937	0.8	57.1	138	23.66	7.0	even	ok
	139	C34H94NO13P2S	818.5916	-1.9	57.5	139	17.29	-10.0	even	ok
	140	C31H84N11O9S2	818.5889	-5.1	58.0	140	1.45	-4.0	even	ok
	141	C30H85N13O6PS2	818.5919	-1.4	58.5	141	21.15	-4.0	even	ok
	142	C33H89N5NaO11S2	818.5892	-4.7	58.5	142	1.99	-8.0	even	ok
	143	C32H90N7NaO8PS2	818.5922	-1.1	58.8	143	24.58	-8.0	even	ok
	144	C50H85NNaO2S2	818.5914	-2.1	59.4	144	14.44	9.0	even	ok
	145	C31H91N9NaO5P2S2	818.5952	2.6	59.4	145	10.67	-8.0	even	ok
	146	C32H79N17NaO2P2	818.5970	4.8	59.5	146	1.47	3.0	even	ok
	147	C27H77N23PS2	818.5906	-3.1	59.7	147	7.57	2.0	even	ok
	148	C28H73N23NaO2S	818.5931	-0.1	60.0	148	28.18	4.0	even	ok
	149	C29H82N17NaO2PS2	818.5909	-2.7	60.0	149	9.48	-2.0	even	ok
	150	C31H81N13NaO8S	818.5944	1.6	60.1	150	14.73	-2.0	even	ok
	151	C29H76N19O6S	818.5941	1.3	60.6	151	17.09	2.0	even	ok
	152	C34H92NO15S2	818.5903	-3.4	60.9	152	5.58	-10.0	even	ok
	153	C33H93N3O12PS2	818.5933	0.2	61.0	153	32.08	-10.0	even	ok
	154	C30H74N23P2	818.5967	4.4	61.1	154	1.91	7.0	even	ok
	155	C32H94N5O9P2S2	818.5963	3.9	61.4	155	3.84	-10.0	even	ok
	156	C55H80NO2S	818.5904	-3.3	61.5	156	6.22	17.0	even	ok
	157	C26H68N29S	818.5928	-0.4	62.3	157	23.07	8.0	even	ok
	158	C29H86N15O3P2S2	818.5949	2.2	62.6	158	11.84	-4.0	even	ok
	159	C34H84N5O16	818.5908	-2.9	62.7	159	6.34	-4.0	even	ok
	160	C33H81N9NaO12	818.5897	-4.2	62.9	160	2.30	-2.0	even	ok
	161	C30H83N15NaO3P2S	818.5891	-4.8	63.0	161	1.25	-2.0	even	ok
	162	C28H77N21O3PS	818.5971	4.9	63.2	162	1.15	2.0	even	ok
	163	C31H86N11O7P2S	818.5902	-3.5	63.7	163	3.79	-4.0	even	ok
	164	C31H76N15O10	818.5894	-4.5	64.2	164	1.62	2.0	even	ok
	165	C30H85N9NaO12S	818.5931	-0.0	64.3	165	30.51	-7.0	even	ok
	166	C35H90NNaO15P	818.5940	1.1	64.9	166	15.53	-8.0	even	ok
	167	C31H88N5O16S	818.5941	1.3	65.2	167	18.10	-9.0	even	ok
	168	C33H85N7O13P	818.5937	0.8	66.1	168	17.29	-4.0	even	ok
	169	C32H82N11NaO9P	818.5927	-0.5	66.5	169	19.13	-2.0	even	ok
	170	C32H95NNaO13P2S	818.5892	-4.8	67.0	170	1.37	-13.0	even	ok
	171	C30H89N7O13PS	818.5971	4.9	67.2	171	1.23	-9.0	even	ok
	172	C30H77N17O7P	818.5924	-0.8	67.9	172	15.87	2.0	even	ok

Compound Spectrum SmartFormula Report

Meas. m/z	#	Ion Formula	m/z	err [ppm]	mSigma	# mSigma	Score	rdb	e¯ Conf	N-Rule
	173	C34H91N3NaO12P2	818.5970	4.8	68.3	173	1.07	-8.0	even	ok
	174	C29H74N21NaO3P	818.5913	-2.2	68.6	174	8.00	4.0	even	ok
	175	C52H84NO2S2	818.5938	0.9	68.9	175	18.98	12.0	even	ok
	176	C32H86N9O10P2	818.5967	4.5	69.6	176	1.40	-4.0	even	ok
	177	C31H83N13NaO6P2	818.5957	3.1	70.2	177	3.95	-2.0	even	ok
	178	C27H69N27OP	818.5911	-2.5	70.2	178	6.14	8.0	even	ok
	179	C29H78N19O4P2	818.5954	2.8	71.6	179	4.75	2.0	even	ok
	180	C27H77N19NaO6S	818.5917	-1.7	71.7	180	9.25	-1.0	even	ok
	181	C28H80N15O10S	818.5928	-0.4	72.4	181	16.12	-3.0	even	ok
	182	C25H72N25O4S	818.5914	-2.0	72.5	182	7.45	3.0	even	ok
	183	C28H75N23NaP2	818.5943	1.5	72.6	183	9.85	4.0	even	ok
	184	C24H69N29NaS	818.5904	-3.3	73.1	184	3.08	5.0	even	ok
	185	C33H88NO20	818.5894	-4.5	74.2	185	1.14	-9.0	even	ok
	186	C29H86N11NaO9PS	818.5961	3.6	74.2	186	2.39	-7.0	even	ok
	187	C26H78N21NaO3PS	818.5947	2.0	74.5	187	7.16	-1.0	even	ok
	188	C27H81N17O7PS	818.5958	3.3	74.9	188	3.02	-3.0	even	ok
	189	C32H89N3O17P	818.5924	-0.8	77.4	189	11.12	-9.0	even	ok
	190	C31H86N7NaO13P	818.5913	-2.1	77.6	190	5.73	-7.0	even	ok
	191	C29H81N13O11P	818.5911	-2.5	78.8	191	4.43	-3.0	even	ok
	192	C28H78N17NaO7P	818.5900	-3.8	79.4	192	1.70	-1.0	even	ok
	193	C25H69N27NaO3	818.5969	4.6	80.3	193	0.78	5.0	even	ok
	194	C31H90N5O14P2	818.5954	2.8	80.7	194	3.30	-9.0	even	ok
	195	C26H73N23O5P	818.5897	-4.1	80.8	195	1.20	3.0	even	ok
	196	C30H87N9NaO10P2	818.5943	1.5	81.1	196	7.00	-7.0	even	ok
	197	C23H64N33O	818.5966	4.3	81.9	197	1.00	9.0	even	ok
	198	C28H82N15O8P2	818.5941	1.2	82.4	198	7.81	-3.0	even	ok
	199	C27H79N19NaO4P2	818.5930	-0.1	83.1	199	11.49	-1.0	even	ok
	200	C25H74N25O2P2	818.5927	-0.5	84.6	200	9.56	3.0	even	ok
	201	C30H90N3NaO17P	818.5900	-3.8	88.8	201	1.16	-12.0	even	ok
	202	C27H81N13NaO13	818.5969	4.6	89.3	202	0.53	-6.0	even	ok
840.5625	1	C43H79N5O9P	840.5610	-1.8	6.8	1	49.62	8.0	even	ok
	2	C44H85NNaO8P2	840.5643	2.1	8.8	2	40.98	4.0	even	ok
	3	C46H84NO8P2	840.5667	4.9	8.9	3	4.05	7.0	even	ok
	4	C42H76N9NaO5P	840.5599	-3.1	9.7	4	20.56	10.0	even	ok
	5	C42H80N7O6P2	840.5640	1.7	10.9	5	47.72	8.0	even	ok
	6	C45H81N5NaO4P2	840.5656	3.7	10.9	6	12.67	9.0	even	ok
	7	C44H75N9O5P	840.5623	-0.2	11.6	7	94.23	13.0	even	ok
	8	C46H80N3NaO7P	840.5626	0.1	11.7	8	97.69	9.0	even	ok
	9	C43H76N11O2P2	840.5653	3.3	11.9	9	16.24	13.0	even	ok
	10	C40H71N15O3P	840.5596	-3.4	12.0	10	15.26	14.0	even	ok
	11	C40H76N13NaOPS	840.5646	2.5	12.1	11	36.45	10.0	even	ok
	12	C37H71N17NaO2S	840.5590	-4.2	13.2	12	9.27	11.0	even	ok
	13	C35H66N23S	840.5587	-4.6	13.4	13	5.41	15.0	even	ok
	14	C45H74N7O8	840.5593	-3.8	13.4	14	11.03	13.0	even	ok
	15	C39H70N17O2S	840.5614	-1.4	13.8	15	68.75	14.0	even	ok
	16	C43H72N13NaOP	840.5613	-1.5	14.0	16	51.55	15.0	even	ok

Compound Spectrum SmartFormula Report

Meas. m/z	#	Ion Formula	m/z	err [ppm]	mSigma	# mSigma	Score	rdb	e¯ Conf	N-Rule
	17	C41H77N11NaO2P2	840.5629	0.5	14.1	17	81.48	10.0	even	ok
	18	C44H71N11NaO4	840.5583	-5.0	14.4	18	3.26	15.0	even	ok
	19	C47H79NNaO10	840.5596	-3.4	14.4	19	14.21	9.0	even	ok
	20	C37H75N15O3PS	840.5630	0.6	15.2	20	95.13	9.0	even	ok
	21	C42H83NO13P	840.5597	-3.4	15.4	21	14.41	3.0	even	ok
	22	C41H79N9O5PS	840.5657	3.8	15.7	22	13.05	8.0	even	ok
	23	C41H75N11NaO4S	840.5616	-1.0	16.0	23	77.28	10.0	even	ok
	24	C39H80N9NaO5PS	840.5633	0.9	16.1	24	81.08	5.0	even	ok
	25	C38H74N13O6S	840.5600	-3.0	16.2	25	24.73	9.0	even	ok
	26	C41H80N5NaO9P	840.5586	-4.7	16.5	26	4.57	5.0	even	ok
	27	C39H72N17P2	840.5626	0.1	16.6	27	87.82	14.0	even	ok
	28	C39H74N11O9	840.5665	4.8	16.7	28	4.01	9.0	even	ok
	29	C36H76N17P2S	840.5660	4.2	17.0	29	7.40	9.0	even	ok
	30	C38H81N11NaO2P2S	840.5663	4.5	17.2	30	5.40	5.0	even	ok
	31	C40H79N7NaO8S	840.5603	-2.6	17.7	31	30.32	5.0	even	ok
	32	C43H84N3NaO7PS	840.5660	4.1	17.8	32	9.35	4.0	even	ok
	33	C39H75N11O7P	840.5583	-5.0	18.4	33	3.14	9.0	even	ok
	34	C38H71N15NaO5	840.5655	3.5	18.7	34	12.18	11.0	even	ok
	35	C44H81N7NaOP2S	840.5591	-4.1	19.0	35	9.37	9.0	even	ok
	36	C40H83N5O9PS	840.5644	2.2	19.1	36	38.84	3.0	even	ok
	37	C41H84N3O10P2	840.5626	0.2	19.3	37	82.56	3.0	even	ok
	38	C42H78N7O8S	840.5627	0.2	19.5	38	100.00	8.0	even	ok
	39	C42H71N15NaS	840.5630	0.6	20.7	39	86.20	15.0	even	ok
	40	C36H66N21O3	840.5652	3.2	20.9	40	15.10	15.0	even	ok
	41	C40H81N7NaO6P2	840.5616	-1.1	21.0	41	53.76	5.0	even	ok
	42	C41H82N3O12S	840.5614	-1.4	21.1	42	59.48	3.0	even	ok
	43	C44H83NNaO10S	840.5630	0.6	21.7	43	83.94	4.0	even	ok
	44	C45H84N3O5P2S	840.5601	-2.8	21.9	44	24.29	7.0	even	ok
	45	C45H71N13OP	840.5637	1.4	22.5	45	45.89	18.0	even	ok
	46	C48H79N3O7P	840.5650	3.0	22.6	46	17.12	12.0	even	ok
	47	C47H76N7NaO3P	840.5639	1.7	23.0	47	38.11	14.0	even	ok
	48	C38H76N13O4P2	840.5613	-1.4	23.0	48	43.63	9.0	even	ok
	49	C43H74N11O4S	840.5640	1.8	23.4	49	44.17	13.0	even	ok
	50	C37H78N9O10S	840.5587	-4.6	23.8	50	5.50	4.0	even	ok
	51	C36H79N11O7PS	840.5617	-1.0	24.2	51	66.17	4.0	even	ok
	52	C38H84N5NaO9PS	840.5620	-0.7	24.4	52	75.95	0.0	even	ok
	53	C39H83N3NaO12S	840.5590	-4.2	24.5	53	7.40	0.0	even	ok
	54	C46H70N11O4	840.5607	-2.2	24.9	54	27.57	18.0	even	ok
	55	C35H76N15NaO3PS	840.5606	-2.3	25.0	55	26.20	6.0	even	ok
	56	C37H85N7NaO6P2S	840.5649	2.9	25.2	56	21.37	0.0	even	ok
	57	C33H71N21OPS	840.5603	-2.6	25.3	57	20.95	10.0	even	ok
	58	C37H73N17NaP2	840.5602	-2.7	25.5	58	19.24	11.0	even	ok
	59	C45H79N5NaO6S	840.5643	2.2	25.6	59	34.55	9.0	even	ok
	60	C45H67N15Na	840.5596	-3.5	25.7	60	11.06	20.0	even	ok
	61	C40H83NNaO15	840.5655	3.5	25.8	61	10.30	0.0	even	ok
	62	C48H75N5NaO6	840.5610	-1.9	25.9	62	32.82	14.0	even	ok

Compound Spectrum SmartFormula Report

Meas. m/z	#	Ion Formula	m/z	err [ppm]	mSigma	# mSigma	Score	rdb	e⁻ Conf	N-Rule
	63	C49H78NO10	840.5620	-0.6	26.2	63	60.25	12.0	even	ok
	64	C39H87NO13PS	840.5630	0.6	26.8	64	73.65	-2.0	even	ok
	65	C38H88N3O10P2S	840.5660	4.2	27.0	65	7.33	-2.0	even	ok
	66	C34H77N17NaP2S	840.5636	1.3	27.1	66	43.00	6.0	even	ok
	67	C38H78N7O13	840.5652	3.2	27.2	67	12.97	4.0	even	ok
	68	C35H80N13O4P2S	840.5647	2.6	28.1	68	20.11	4.0	even	ok
	69	C37H75N11NaO9	840.5641	1.9	28.1	69	29.67	6.0	even	ok
	70	C46H80N7OP2S	840.5615	-1.2	28.6	70	53.41	12.0	even	ok
	71	C46H82NO10S	840.5654	3.4	28.6	71	13.22	7.0	even	ok
	72	C35H70N17O7	840.5639	1.6	29.9	72	34.24	10.0	even	ok
	73	C48H85NNaO3P2S	840.5618	-0.9	30.7	73	59.27	8.0	even	ok
	74	C44H70N15S	840.5654	3.4	30.8	74	12.66	18.0	even	ok
	75	C39H85N3NaO10P2	840.5602	-2.7	30.9	75	17.08	0.0	even	ok
	76	C32H70N23S2	840.5620	-0.6	31.1	76	67.85	10.0	even	ok
	77	C34H67N21NaO3	840.5628	0.3	31.6	77	58.29	12.0	even	ok
	78	C36H74N17O2S2	840.5647	2.6	32.0	78	21.75	9.0	even	ok
	79	C34H75N17NaO2S2	840.5623	-0.2	32.5	79	74.39	6.0	even	ok
	80	C37H80N9O8P2	840.5600	-3.0	32.6	80	12.93	4.0	even	ok
	81	C38H84N9O3P2S2	840.5608	-2.0	32.6	81	31.98	3.0	even	ok
	82	C34H80N11NaO7PS	840.5593	-3.9	32.8	82	8.37	1.0	even	ok
	83	C46H75N9NaO2S	840.5657	3.7	32.8	83	9.18	14.0	even	ok
	84	C47H79N5O4PS	840.5585	-4.8	33.2	84	3.49	12.0	even	ok
	85	C41H85N7NaOP2S2	840.5624	-0.1	33.5	85	76.26	4.0	even	ok
	86	C32H62N27O	840.5625	0.0	33.7	86	62.31	16.0	even	ok
	87	C39H75N15NaS2	840.5664	4.6	33.8	87	4.28	10.0	even	ok
	88	C36H85N9NaO3P2S2	840.5584	-4.9	34.0	88	3.16	0.0	even	ok
	89	C38H79N11NaO4S2	840.5650	3.0	34.0	89	16.37	5.0	even	ok
	90	C34H79N15O3PS2	840.5664	4.6	34.0	90	4.08	4.0	even	ok
	91	C35H83N7O11PS	840.5603	-2.6	34.1	91	21.36	-1.0	even	ok
	92	C37H88NNaO13PS	840.5606	-2.3	34.1	92	26.49	-5.0	even	ok
	93	C36H77N13NaO4P2	840.5589	-4.3	34.1	93	4.32	6.0	even	ok
	94	C49H75N7O3P	840.5664	4.6	34.4	94	3.37	17.0	even	ok
	95	C40H89N3NaO5P2S2	840.5611	-1.7	34.5	95	36.80	-1.0	even	ok
	96	C51H81NNaO3P2	840.5584	-4.9	34.6	96	2.38	13.0	even	ok
	97	C47H78N5O6S	840.5667	5.0	35.2	97	2.62	12.0	even	ok
	98	C40H79N11O2PS2	840.5592	-4.0	35.3	98	7.11	8.0	even	ok
	99	C36H89N3NaO10P2S	840.5636	1.3	35.4	99	43.55	-5.0	even	ok
	100	C36H84N9NaO5PS2	840.5667	4.9	35.4	100	2.83	0.0	even	ok
	101	C51H80NNaO5P	840.5666	4.9	35.5	101	2.35	13.0	even	ok
	102	C30H71N23NaS2	840.5596	-3.4	35.6	102	11.19	7.0	even	ok
	103	C34H84N9O8P2S	840.5633	1.0	35.6	103	50.61	-1.0	even	ok
	104	C31H71N21NaO3S	840.5662	4.3	35.7	104	4.01	7.0	even	ok
	105	C35H78N13O6S2	840.5634	1.0	35.9	105	48.60	4.0	even	ok
	106	C34H72N19O2P2	840.5586	-4.6	36.1	106	2.99	10.0	even	ok
	107	C33H68N23NaP	840.5658	3.9	36.3	107	5.94	12.0	even	ok
	108	C29H66N27OS	840.5659	4.0	36.4	108	5.33	11.0	even	ok

Compound Spectrum SmartFormula Report

Meas. m/z	#	Ion Formula	m/z	err [ppm]	mSigma	# mSigma	Score	rdb	e¯ Conf	N-Rule
	109	C47H66N15	840.5620	-0.6	36.8	109	46.20	23.0	even	ok
	110	C32H75N17O5PS	840.5590	-4.2	36.9	110	4.48	5.0	even	ok
	111	C42H88N3O5P2S2	840.5635	1.2	37.2	111	43.94	2.0	even	ok
	112	C37H88N5O7P2S2	840.5595	-3.6	37.3	112	9.28	-2.0	even	ok
	113	C37H83N7NaO8S2	840.5637	1.4	37.4	113	39.71	0.0	even	ok
	114	C42H84N5NaO4PS2	840.5595	-3.6	37.6	114	8.89	4.0	even	ok
	115	C49H71N9NaO2	840.5623	-0.3	37.7	115	51.20	19.0	even	ok
	116	C39H82N7O8S2	840.5661	4.2	37.7	116	5.23	3.0	even	ok
	117	C50H74N5O6	840.5634	1.0	37.8	117	37.60	17.0	even	ok
	118	C30H75N21OPS2	840.5637	1.4	38.0	118	38.56	5.0	even	ok
	119	C31H74N19O4S2	840.5607	-2.1	38.3	119	25.34	5.0	even	ok
	120	C37H82N3O17	840.5639	1.6	38.4	120	27.39	-1.0	even	ok
	121	C32H80N15NaO3PS2	840.5640	1.7	38.6	121	31.72	1.0	even	ok
	122	C33H81N13NaO4P2S	840.5623	-0.3	38.7	122	49.15	1.0	even	ok
	123	C36H79N7NaO13	840.5628	0.3	38.8	123	48.17	1.0	even	ok
	124	C33H79N13NaO6S2	840.5610	-1.8	39.3	124	30.02	1.0	even	ok
	125	C43H84N7OP2S2	840.5649	2.8	39.3	125	16.32	7.0	even	ok
	126	C31H76N19O2P2S	840.5620	-0.6	39.5	126	42.34	5.0	even	ok
	127	C41H87NNaO10S2	840.5664	4.6	39.8	127	3.62	-1.0	even	ok
	128	C34H74N13O11	840.5625	0.0	40.3	128	52.09	5.0	even	ok
	129	C43H80N9NaPS2	840.5608	-2.0	40.5	129	25.35	9.0	even	ok
	130	C38H86N3O12S2	840.5647	2.7	41.0	130	17.03	-2.0	even	ok
	131	C50H84NO3P2S	840.5642	2.0	41.2	131	26.12	11.0	even	ok
	132	C43H87NO8PS2	840.5605	-2.4	41.2	132	20.37	2.0	even	ok
	133	C33H83N11O7PS2	840.5650	3.0	41.4	133	13.03	-1.0	even	ok
	134	C33H71N17NaO7	840.5615	-1.3	41.4	134	30.34	7.0	even	ok
	135	C45H89NNaO3P2S2	840.5651	3.1	41.6	135	12.02	3.0	even	ok
	136	C48H75N9PS	840.5598	-3.2	41.7	136	11.26	17.0	even	ok
	137	C34H82N9O10S2	840.5621	-0.5	42.2	137	50.82	-1.0	even	ok
	138	C35H88N5NaO9PS2	840.5653	3.3	42.3	138	9.87	-5.0	even	ok
	139	C35H80N9NaO10P	840.5658	3.9	42.7	139	4.93	1.0	even	ok
	140	C33H83N7NaO13S	840.5662	4.4	43.0	140	4.06	-4.0	even	ok
	141	C36H84N5O12P2	840.5586	-4.6	43.1	141	2.50	-1.0	even	ok
	142	C31H66N23O5	840.5612	-1.6	43.1	142	24.38	11.0	even	ok
	143	C31H78N13O11S	840.5659	4.0	43.4	143	5.45	0.0	even	ok
	144	C36H87N3NaO12S2	840.5623	-0.2	43.4	144	55.79	-5.0	even	ok
	145	C50H80N3NaO2PS	840.5601	-2.9	43.7	145	13.62	13.0	even	ok
	146	C44H83N5O4PS2	840.5619	-0.8	43.9	146	44.06	7.0	even	ok
	147	C33H75N15O8P	840.5655	3.6	44.3	147	6.23	5.0	even	ok
	148	C34H87N3O15PS	840.5590	-4.2	44.3	148	4.62	-6.0	even	ok
	149	C29H75N19NaO4S2	840.5583	-5.0	44.6	149	2.05	2.0	even	ok
	150	C30H63N27NaO	840.5601	-2.9	44.8	150	10.59	13.0	even	ok
	151	C28H76N21NaOPS2	840.5613	-1.5	45.1	151	30.95	2.0	even	ok
	152	C36H91NO13PS2	840.5664	4.6	45.3	152	2.97	-7.0	even	ok
	153	C32H72N19NaO4P	840.5644	2.3	45.6	153	15.01	7.0	even	ok
	154	C33H88N5O12P2S	840.5620	-0.6	46.1	154	44.18	-6.0	even	ok

Compound Spectrum SmartFormula Report

Meas. m/z	#	Ion Formula	m/z	err [ppm]	mSigma	# mSigma	Score	rdb	e^- Conf	N-Rule
	155	C53H80NO3P2	840.5608	-2.0	46.6	155	17.06	16.0	even	ok
	156	C30H78N15O8S2	840.5594	-3.7	46.9	156	6.30	0.0	even	ok
	157	C29H79N17O5PS2	840.5624	-0.2	47.2	157	50.56	0.0	even	ok
	158	C30H75N17NaO7S	840.5648	2.8	47.4	158	10.56	2.0	even	ok
	159	C30H67N25O2P	840.5642	2.0	47.5	159	17.41	11.0	even	ok
	160	C32H83N9NaO10S2	840.5597	-3.4	47.5	160	8.11	-4.0	even	ok
	161	C31H84N11NaO7PS2	840.5626	0.2	47.6	161	50.41	-4.0	even	ok
	162	C27H67N27NaOS	840.5635	1.2	47.7	162	26.61	8.0	even	ok
	163	C28H80N19O2P2S2	840.5654	3.4	47.8	163	8.21	0.0	even	ok
	164	C30H85N13NaO4P2S2	840.5656	3.7	48.0	164	6.23	-4.0	even	ok
	165	C28H70N23O5S	840.5645	2.4	48.1	165	12.92	6.0	even	ok
	166	C45H82N3O7S2	840.5589	-4.3	48.5	166	3.54	7.0	even	ok
	167	C45H79N9PS2	840.5632	0.8	48.9	167	37.41	12.0	even	ok
	168	C51H70N9O2	840.5647	2.6	49.7	168	10.95	22.0	even	ok
	169	C32H87N7O11PS2	840.5637	1.4	49.9	169	27.19	-6.0	even	ok
	170	C35H83N3NaO17	840.5615	-1.2	49.9	170	23.80	-4.0	even	ok
	171	C31H88N9O8P2S2	840.5667	5.0	50.1	171	1.78	-6.0	even	ok
	172	C33H86N5O14S2	840.5607	-2.1	50.2	172	18.18	-6.0	even	ok
	173	C32H85N9NaO8P2S	840.5609	-1.9	50.4	173	16.65	-4.0	even	ok
	174	C34H92NNaO13PS2	840.5640	1.8	50.6	174	22.34	-10.0	even	ok
	175	C29H77N19NaO2P2S	840.5596	-3.5	50.7	175	5.49	2.0	even	ok
	176	C53H75N3NaO4	840.5650	2.9	51.0	176	8.35	18.0	even	ok
	177	C30H80N15O6P2S	840.5606	-2.2	51.1	177	13.36	0.0	even	ok
	178	C33H78N9O15	840.5612	-1.6	51.2	178	19.32	0.0	even	ok
	179	C47H84N3NaO2PS2	840.5635	1.1	51.2	179	29.95	8.0	even	ok
	180	C27H72N25P2S	840.5593	-3.8	51.7	180	4.04	6.0	even	ok
	181	C32H75N13NaO11	840.5601	-2.8	51.9	181	8.62	2.0	even	ok
	182	C29H79N13NaO11S	840.5635	1.2	53.0	182	28.18	-3.0	even	ok
	183	C35H87NO18P	840.5655	3.6	53.1	183	4.74	-6.0	even	ok
	184	C30H70N19O9	840.5598	-3.2	53.4	184	6.44	6.0	even	ok
	185	C32H87N3NaO17S	840.5648	2.8	53.4	185	10.88	-9.0	even	ok
	186	C34H84N5NaO14P	840.5645	2.3	53.5	186	11.72	-4.0	even	ok
	187	C46H78N7O3S2	840.5602	-2.7	53.7	187	10.95	12.0	even	ok
	188	C30H82N9O15S	840.5646	2.4	53.8	188	13.45	-5.0	even	ok
	189	C29H67N23NaO5	840.5588	-4.5	54.6	189	2.10	8.0	even	ok
	190	C52H79N3O2PS	840.5625	-0.0	54.7	190	42.62	16.0	even	ok
	191	C32H79N11O12P	840.5642	2.0	54.9	191	13.72	0.0	even	ok
	192	C25H75N23O3PS2	840.5597	-3.4	55.1	192	6.58	1.0	even	ok
	193	C27H80N17NaO5PS2	840.5600	-3.0	55.1	193	8.47	-3.0	even	ok
	194	C31H89N5NaO12P2S	840.5596	-3.5	55.7	194	5.93	-9.0	even	ok
	195	C31H76N15NaO8P	840.5631	0.7	55.8	195	25.19	2.0	even	ok
	196	C48H83NNaO5S2	840.5605	-2.4	56.1	196	12.68	8.0	even	ok
	197	C27H62N29O3	840.5585	-4.8	56.3	197	1.44	12.0	even	ok
	198	C32H92NO16P2S	840.5607	-2.2	56.5	198	14.20	-11.0	even	ok
	199	C31H87N5NaO14S2	840.5583	-5.0	56.7	199	1.43	-9.0	even	ok
	200	C28H83N13O9PS2	840.5610	-1.8	56.9	200	18.15	-5.0	even	ok

Compound Spectrum SmartFormula Report

Meas. m/z	#	Ion Formula	m/z	err [ppm]	mSigma	# mSigma	Score	rdb	e⁻ Conf	N-Rule
	201	C30H88N7NaO11PS2	840.5613	-1.4	57.1	201	21.51	-9.0	even	ok
	202	C52H75N5NaOS	840.5585	-4.8	57.4	202	1.65	18.0	even	ok
	203	C29H71N21O6P	840.5628	0.4	57.4	203	27.29	6.0	even	ok
	204	C28H68N25NaO2P	840.5618	-0.9	58.7	204	21.16	8.0	even	ok
	205	C32H90NO18S2	840.5594	-3.7	59.0	205	4.35	-11.0	even	ok
	206	C53H78NO5S	840.5595	-3.6	59.2	206	4.95	16.0	even	ok
	207	C31H91N3O15PS2	840.5624	-0.2	59.2	207	34.71	-11.0	even	ok
	208	C26H71N23NaO5S	840.5621	-0.4	59.3	208	25.02	3.0	even	ok
	209	C27H74N19O9S	840.5632	0.8	59.8	209	20.99	1.0	even	ok
	210	C30H77N17NaO5P2	840.5661	4.3	59.9	210	2.10	2.0	even	ok
	211	C24H66N29O3S	840.5619	-0.8	60.3	211	21.18	7.0	even	ok
	212	C26H63N31P	840.5615	-1.2	60.5	212	17.08	12.0	even	ok
	213	C49H83N3O2PS2	840.5659	4.0	60.7	213	3.22	11.0	even	ok
	214	C28H72N23O3P2	840.5658	3.9	61.5	214	2.67	6.0	even	ok
	215	C28H80N15NaO8PS	840.5665	4.7	61.7	215	1.28	-3.0	even	ok
	216	C55H75N3O2P	840.5591	-4.0	62.3	216	2.43	21.0	even	ok
	217	C25H72N25NaO2PS	840.5651	3.1	62.3	217	5.03	3.0	even	ok
	218	C28H81N15NaO6P2S	840.5582	-5.1	62.3	218	0.86	-3.0	even	ok
	219	C32H82N5O19	840.5599	-3.2	62.4	219	4.83	-5.0	even	ok
	220	C26H75N21O6PS	840.5662	4.4	62.5	220	1.71	1.0	even	ok
	221	C31H79N9NaO15	840.5588	-4.4	62.8	221	1.62	-3.0	even	ok
	222	C29H84N11O10P2S	840.5593	-3.8	62.8	222	2.84	-5.0	even	ok
	223	C49H79N5NaOS2	840.5618	-0.8	62.8	223	23.74	13.0	even	ok
	224	C54H71N7Na	840.5663	4.5	63.0	224	1.48	23.0	even	ok
	225	C23H67N31PS	840.5649	2.8	63.4	225	6.17	7.0	even	ok
	226	C28H83N9NaO15S	840.5622	-0.4	63.4	226	27.31	-8.0	even	ok
	227	C26H78N15O13S	840.5619	-0.8	64.0	227	23.39	-4.0	even	ok
	228	C29H74N15O13	840.5585	-4.8	64.1	228	1.12	1.0	even	ok
	229	C29H86N5O19S	840.5632	0.8	64.3	229	22.37	-10.0	even	ok
	230	C33H88NNaO18P	840.5631	0.7	64.6	230	18.62	-9.0	even	ok
	231	C50H82NO5S2	840.5629	0.5	65.6	231	25.06	11.0	even	ok
	232	C31H83N7O16P	840.5628	0.4	65.9	232	20.34	-5.0	even	ok
	233	C55H80NNaPS	840.5641	1.9	66.2	233	12.11	17.0	even	ok
	234	C30H80N11NaO12P	840.5618	-0.9	66.4	234	16.29	-3.0	even	ok
	235	C28H75N17O10P	840.5615	-1.2	67.9	235	13.26	1.0	even	ok
	236	C32H89N3NaO15P2	840.5661	4.3	68.1	236	1.55	-9.0	even	ok
	237	C54H74N5OS	840.5609	-2.0	68.7	237	10.75	21.0	even	ok
	238	C27H72N21NaO6P	840.5604	-2.5	68.8	238	6.23	3.0	even	ok
	239	C30H84N9O13P2	840.5658	3.9	69.5	239	1.99	-5.0	even	ok
	240	C29H81N13NaO9P2	840.5648	2.7	70.2	240	5.20	-3.0	even	ok
	241	C25H67N27O4P	840.5601	-2.8	70.4	241	4.68	7.0	even	ok
	242	C25H75N19NaO9S	840.5608	-2.0	70.9	242	7.64	-2.0	even	ok
	243	C24H64N31NaP	840.5591	-4.1	71.7	243	1.62	9.0	even	ok
	244	C27H76N19O7P2	840.5645	2.3	71.7	244	6.11	1.0	even	ok
	245	C26H73N23NaO3P2	840.5634	1.1	72.8	245	11.79	3.0	even	ok
	246	C51H78N5OS2	840.5642	2.0	73.3	246	8.70	16.0	even	ok

Compound Spectrum SmartFormula Report

Meas. m/z	#	Ion Formula	m/z	err [ppm]	mSigma	# mSigma	Score	rdb	e⁻ Conf	N-Rule
	247	C31H86NO23	840.5585	-4.8	73.7	247	0.80	-10.0	even	ok
	248	C24H68N29OP2	840.5631	0.7	74.5	248	12.82	7.0	even	ok
	249	C58H76NNaP	840.5608	-2.1	75.7	249	6.16	22.0	even	ok
	250	C30H87N3O20P	840.5615	-1.2	77.0	250	9.45	-10.0	even	ok
	251	C29H84N7NaO16P	840.5604	-2.5	77.3	251	4.54	-8.0	even	ok
	252	C57H79NPS	840.5665	4.8	77.7	252	0.83	20.0	even	ok
	253	C27H79N13O14P	840.5602	-2.8	78.6	253	3.44	-4.0	even	ok
	254	C26H76N17NaO10P	840.5591	-4.1	79.3	254	1.22	-2.0	even	ok
	255	C23H67N27NaO6	840.5660	4.1	80.4	255	1.11	4.0	even	ok
	256	C24H71N23O8P	840.5588	-4.4	80.8	256	0.85	2.0	even	ok
	257	C21H62N33O4	840.5657	3.8	81.9	257	1.40	8.0	even	ok
	258	C20H59N37Na	840.5646	2.5	83.1	258	3.48	10.0	even	ok
	259	C60H75NP	840.5632	0.8	88.4	259	7.16	25.0	even	ok
845.5175	1	C42H62N16NaO2	845.5134	-4.8	2.8	1	4.32	20.0	even	ok
	2	C42H66N14O3P	845.5174	-0.0	5.6	2	100.00	18.0	even	ok
	3	C44H71N8NaO5P	845.5177	0.3	5.8	3	91.55	14.0	even	ok
	4	C43H65N12O6	845.5145	-3.6	5.9	4	13.11	18.0	even	ok
	5	C43H72N10NaO2P2	845.5207	3.8	7.5	5	10.41	14.0	even	ok
	6	C45H70N6NaO8	845.5147	-3.3	7.9	6	16.48	14.0	even	ok
	7	C45H67N12NaOP	845.5191	1.9	8.4	7	41.58	19.0	even	ok
	8	C44H75N6O6P2	845.5218	5.1	8.5	8	3.04	12.0	even	ok
	9	C41H67N16P2	845.5204	3.5	9.0	9	13.38	18.0	even	ok
	10	C45H74N4O9P	845.5188	1.5	9.1	10	48.93	12.0	even	ok
	11	C46H70N8O5P	845.5201	3.1	10.4	11	17.28	17.0	even	ok
	12	C44H61N16O2	845.5158	-2.0	11.0	12	36.64	23.0	even	ok
	13	C46H73N2O12	845.5158	-2.0	12.1	13	36.13	12.0	even	ok
	14	C48H75N2NaO7P	845.5204	3.5	12.9	14	12.75	13.0	even	ok
	15	C46H66N10NaO4	845.5161	-1.7	13.0	15	42.55	19.0	even	ok
	16	C47H69N6O8	845.5171	-0.4	15.6	16	72.72	17.0	even	ok
	17	C44H74N2NaO12	845.5134	-4.8	15.6	17	3.45	9.0	even	ok
	18	C43H75N4NaO9P	845.5164	-1.3	16.8	18	47.99	9.0	even	ok
	19	C40H67N14NaO3P	845.5150	-2.9	17.1	19	18.11	15.0	even	ok
	20	C41H70N10O7P	845.5161	-1.6	17.3	20	40.00	13.0	even	ok
	21	C49H74NaO10	845.5174	-0.1	18.1	21	78.01	13.0	even	ok
	22	C44H78O13P	845.5175	-0.0	18.5	22	78.46	7.0	even	ok
	23	C38H62N20OP	845.5148	-3.2	18.5	23	13.74	19.0	even	ok
	24	C42H76N6NaO6P2	845.5194	2.2	19.2	24	26.83	9.0	even	ok
	25	C43H79N2O10P2	845.5204	3.5	20.0	25	10.65	7.0	even	ok
	26	C37H61N22S	845.5165	-1.2	20.2	26	58.85	19.0	even	ok
	27	C40H71N12O4P2	845.5191	1.9	20.3	27	32.02	13.0	even	ok
	28	C47H66N12OP	845.5215	4.7	20.6	28	3.52	22.0	even	ok
	29	C49H72N6NaOP2	845.5135	-4.7	20.8	29	3.52	18.0	even	ok
	30	C39H68N16NaP2	845.5180	0.6	20.9	30	59.09	15.0	even	ok
	31	C39H66N16NaO2S	845.5168	-0.9	21.6	31	66.41	15.0	even	ok
	32	C50H75N2O5P2	845.5146	-3.4	22.4	32	10.63	16.0	even	ok
	33	C49H71N6NaO3P	845.5217	5.0	22.4	33	2.41	18.0	even	ok

Compound Spectrum SmartFormula Report

Meas. m/z	#	Ion Formula	m/z	err [ppm]	mSigma	# mSigma	Score	rdb	e⁻ Conf	N-Rule
	34	C41H65N16O2S	845.5192	2.0	22.8	34	36.28	18.0	even	ok
	35	C43H75N8O3P2S	845.5153	-2.6	22.8	35	24.01	12.0	even	ok
	36	C39H70N14O3PS	845.5208	3.9	23.1	36	8.58	13.0	even	ok
	37	C47H62N14Na	845.5174	-0.1	23.8	37	68.63	24.0	even	ok
	38	C41H75N8NaO5PS	845.5211	4.3	24.5	38	6.16	9.0	even	ok
	39	C45H80N2NaO5P2S	845.5155	-2.3	25.0	39	28.48	8.0	even	ok
	40	C43H70N10NaO4S	845.5194	2.3	25.0	40	28.17	14.0	even	ok
	41	C48H65N10O4	845.5185	1.2	25.1	41	42.50	22.0	even	ok
	42	C40H69N12O6S	845.5178	0.4	25.1	42	74.09	13.0	even	ok
	43	C42H79N4O7P2S	845.5139	-4.2	26.4	43	6.24	7.0	even	ok
	44	C46H76N6NaOP2S	845.5169	-0.7	26.5	44	63.29	13.0	even	ok
	45	C42H74N6NaO8S	845.5181	0.7	26.9	45	62.31	9.0	even	ok
	46	C50H70N4NaO6	845.5188	1.5	27.2	46	34.33	18.0	even	ok
	47	C36H65N18O4S	845.5151	-2.8	27.6	47	19.54	14.0	even	ok
	48	C44H66N14NaS	845.5208	3.9	27.6	48	8.04	19.0	even	ok
	49	C35H62N22NaS	845.5141	-4.0	28.1	49	5.63	16.0	even	ok
	50	C37H71N14NaO3PS	845.5184	1.1	28.1	50	51.44	10.0	even	ok
	51	C38H70N12NaO6S	845.5154	-2.4	28.4	51	24.00	10.0	even	ok
	52	C42H79NaO13P	845.5150	-2.9	28.4	52	14.20	4.0	even	ok
	53	C39H71N10NaO7P	845.5137	-4.5	28.7	53	3.70	10.0	even	ok
	54	C45H70N10O2PS	845.5136	-4.6	28.7	54	4.14	17.0	even	ok
	55	C44H73N6O8S	845.5205	3.6	28.7	55	10.26	12.0	even	ok
	56	C37H65N16O7	845.5217	4.9	28.9	56	2.30	14.0	even	ok
	57	C40H74N6O11P	845.5148	-3.2	29.1	57	10.94	8.0	even	ok
	58	C36H62N20NaO3	845.5206	3.7	29.2	58	7.46	16.0	even	ok
	59	C51H73O10	845.5198	2.8	29.3	59	15.03	16.0	even	ok
	60	C37H66N16O5P	845.5134	-4.8	29.9	60	2.59	14.0	even	ok
	61	C47H79N2O5P2S	845.5179	0.5	30.0	61	62.58	11.0	even	ok
	62	C43H77N2O12S	845.5192	2.0	30.5	62	30.09	7.0	even	ok
	63	C38H74N10O7PS	845.5195	2.4	30.6	63	23.99	8.0	even	ok
	64	C34H57N26O	845.5203	3.4	30.6	64	9.43	20.0	even	ok
	65	C35H66N20OPS	845.5181	0.8	30.9	65	44.62	14.0	even	ok
	66	C46H78NaO10S	845.5208	3.9	31.0	66	7.33	8.0	even	ok
	67	C41H80N2NaO10P2	845.5180	0.7	31.0	67	46.57	4.0	even	ok
	68	C47H75N4NaO4PS	845.5139	-4.3	31.1	68	5.36	13.0	even	ok
	69	C39H73N8O10S	845.5165	-1.2	31.3	69	45.82	8.0	even	ok
	70	C40H79N4NaO9PS	845.5198	2.7	31.4	70	18.85	4.0	even	ok
	71	C36H72N16NaP2S	845.5214	4.6	31.9	71	2.92	10.0	even	ok
	72	C39H75N8O8P2	845.5178	0.3	32.0	72	51.85	8.0	even	ok
	73	C38H72N12NaO4P2	845.5167	-0.9	32.1	73	40.17	10.0	even	ok
	74	C41H78N2NaO12S	845.5168	-0.8	32.4	74	51.88	4.0	even	ok
	75	C51H71N6OP2	845.5159	-1.9	33.3	75	24.34	21.0	even	ok
	76	C36H67N18O2P2	845.5164	-1.3	33.5	76	33.19	14.0	even	ok
	77	C41H82O13PS	845.5208	4.0	34.3	77	6.49	2.0	even	ok
	78	C48H78O8PS	845.5150	-3.0	34.5	78	14.05	11.0	even	ok
	79	C48H75N6OP2S	845.5193	2.1	34.8	79	25.01	16.0	even	ok

Compound Spectrum SmartFormula Report

Meas. m/z	#	Ion Formula	m/z	err [ppm]	mSigma	# mSigma	Score	rdb	e¯ Conf	N-Rule
	80	C53H76NaO3P2	845.5162	-1.5	35.1	80	27.89	17.0	even	ok
	81	C49H61N14	845.5198	2.8	36.3	81	12.74	27.0	even	ok
	82	C35H69N14O8S	845.5138	-4.4	36.7	82	4.24	9.0	even	ok
	83	C48H71N8NaPS	845.5152	-2.7	36.7	83	16.68	18.0	even	ok
	84	C37H74N8NaO10S	845.5141	-4.0	37.1	84	5.69	5.0	even	ok
	85	C50H80NaO3P2S	845.5196	2.5	37.1	85	19.08	12.0	even	ok
	86	C34H70N16O5PS	845.5168	-0.8	37.4	86	46.23	9.0	even	ok
	87	C36H75N10NaO7PS	845.5171	-0.5	37.6	87	52.83	5.0	even	ok
	88	C51H66N8NaO2	845.5201	3.1	38.0	88	9.56	23.0	even	ok
	89	C38H77N4O14S	845.5152	-2.8	39.5	89	14.59	3.0	even	ok
	90	C52H69N4O6	845.5212	4.4	39.5	90	3.15	21.0	even	ok
	91	C49H74N4O4PS	845.5163	-1.4	39.6	91	32.91	16.0	even	ok
	92	C37H78N6O11PS	845.5181	0.8	39.6	92	44.34	3.0	even	ok
	93	C38H70N16PS2	845.5143	-3.8	39.7	93	6.58	13.0	even	ok
	94	C38H74N6NaO13	845.5206	3.7	39.7	94	5.63	5.0	even	ok
	95	C34H65N22S2	845.5198	2.8	40.0	95	13.99	14.0	even	ok
	96	C39H83NaO13PS	845.5184	1.1	40.0	96	37.71	-1.0	even	ok
	97	C33H67N20NaOPS	845.5157	-2.1	40.1	97	17.88	11.0	even	ok
	98	C36H79N8O8P2S	845.5211	4.3	40.2	98	4.00	3.0	even	ok
	99	C39H77N2O17	845.5217	5.0	40.4	99	1.69	3.0	even	ok
	100	C38H84N2NaO10P2S	845.5214	4.6	40.4	100	2.89	-1.0	even	ok
	101	C35H66N16NaO7	845.5193	2.1	40.6	101	17.41	11.0	even	ok
	102	C36H69N12O11	845.5203	3.4	40.6	102	7.20	9.0	even	ok
	103	C39H78N2O15P	845.5134	-4.8	40.8	103	1.98	3.0	even	ok
	104	C36H75N14OP2S2	845.5159	-1.8	41.4	104	25.08	8.0	even	ok
	105	C36H70N16NaO2S2	845.5201	3.1	41.6	105	10.52	10.0	even	ok
	106	C40H75N10NaO2PS2	845.5146	-3.4	41.7	106	8.19	9.0	even	ok
	107	C33H61N22O5	845.5190	1.8	41.9	107	20.40	15.0	even	ok
	108	C40H79N8O3P2S2	845.5186	1.4	42.3	108	31.42	7.0	even	ok
	109	C32H58N26NaO	845.5179	0.5	42.5	109	36.64	17.0	even	ok
	110	C38H80N8NaO3P2S2	845.5162	-1.5	42.9	110	28.84	4.0	even	ok
	111	C43H80N6NaOP2S2	845.5202	3.3	43.1	111	9.06	8.0	even	ok
	112	C35H76N12NaO4P2S	845.5201	3.1	43.1	112	8.54	5.0	even	ok
	113	C33H71N18O2P2S	845.5198	2.7	43.2	113	10.79	9.0	even	ok
	114	C32H66N22NaS2	845.5174	-0.0	43.3	114	52.98	11.0	even	ok
	115	C37H76N8NaO8P2	845.5154	-2.5	43.6	115	12.27	5.0	even	ok
	116	C38H79N4O12P2	845.5164	-1.3	43.7	116	25.43	3.0	even	ok
	117	C42H84N2NaO5P2S2	845.5189	1.7	44.2	117	24.98	3.0	even	ok
	118	C50H73N2O7S	845.5133	-4.9	44.4	118	1.90	16.0	even	ok
	119	C42H74N10O2PS2	845.5170	-0.6	44.8	119	41.26	12.0	even	ok
	120	C35H71N14O6P2	845.5151	-2.8	44.8	120	9.43	9.0	even	ok
	121	C39H79N6NaO6PS2	845.5132	-5.0	45.1	121	1.72	4.0	even	ok
	122	C37H73N12O6S2	845.5212	4.4	45.1	122	3.24	8.0	even	ok
	123	C34H76N14NaOP2S2	845.5135	-4.7	45.2	123	2.47	5.0	even	ok
	124	C32H70N20OPS2	845.5215	4.8	45.4	124	2.26	9.0	even	ok
	125	C34H68N18NaO2P2	845.5140	-4.1	45.4	125	3.34	11.0	even	ok

Compound Spectrum SmartFormula Report

Meas. m/z	#	Ion Formula	m/z	err [ppm]	mSigma	# mSigma	Score	rdb	e⁻ Conf	N-Rule
	126	C41H78N6O6PS2	845.5156	-2.2	45.4	126	18.05	7.0	even	ok
	127	C39H83N4O7P2S2	845.5173	-0.2	46.3	127	45.63	2.0	even	ok
	128	C33H69N18O4S2	845.5185	1.2	46.3	128	29.99	9.0	even	ok
	129	C34H75N14NaO3PS2	845.5218	5.1	46.3	129	1.56	5.0	even	ok
	130	C50H70N8PS	845.5176	0.2	46.8	130	45.94	21.0	even	ok
	131	C39H78N6NaO8S2	845.5215	4.7	46.8	131	2.24	4.0	even	ok
	132	C44H83N2O5P2S2	845.5213	4.5	46.8	132	2.69	6.0	even	ok
	133	C32H63N24P2	845.5137	-4.4	46.9	133	2.35	15.0	even	ok
	134	C44H79N4NaO4PS2	845.5173	-0.3	47.1	134	43.89	8.0	even	ok
	135	C43H83NaO8PS2	845.5159	-1.9	47.5	135	20.70	3.0	even	ok
	136	C35H79N6NaO11PS	845.5157	-2.1	47.5	136	18.23	0.0	even	ok
	137	C35H74N12NaO6S2	845.5188	1.5	47.5	137	24.48	5.0	even	ok
	138	C33H74N12O9PS	845.5155	-2.4	47.5	138	14.80	4.0	even	ok
	139	C55H75O3P2	845.5186	1.3	47.7	139	21.94	20.0	even	ok
	140	C35H79N10O5P2S2	845.5146	-3.4	48.2	140	7.03	3.0	even	ok
	141	C37H81O18S	845.5138	-4.3	48.6	141	3.07	-2.0	even	ok
	142	C40H82N2O10PS2	845.5143	-3.8	48.6	142	5.15	2.0	even	ok
	143	C34H80N8NaO8P2S	845.5187	1.5	48.8	143	24.50	0.0	even	ok
	144	C43H73N8O5S2	845.5140	-4.1	49.0	144	3.67	12.0	even	ok
	145	C52H75N2NaO2PS	845.5179	0.5	49.0	145	37.96	17.0	even	ok
	146	C53H66N8P	845.5143	-3.8	49.1	146	3.88	26.0	even	ok
	147	C36H82N2O15PS	845.5168	-0.8	49.2	147	33.22	-2.0	even	ok
	148	C37H84N4NaO7P2S2	845.5149	-3.1	49.2	148	8.79	-1.0	even	ok
	149	C45H75N8NaPS2	845.5186	1.3	49.2	149	26.19	13.0	even	ok
	150	C35H83N4O12P2S	845.5198	2.7	50.3	150	10.88	-2.0	even	ok
	151	C29H62N26NaOS	845.5213	4.5	50.6	151	2.00	12.0	even	ok
	152	C36H77N8O10S2	845.5199	2.8	50.7	152	10.17	3.0	even	ok
	153	C29H65N24O2S2	845.5158	-2.0	50.7	153	17.61	10.0	even	ok
	154	C45H82O8PS2	845.5183	1.0	50.8	154	29.05	6.0	even	ok
	155	C55H71N2NaO2P	845.5145	-3.5	50.9	155	4.84	22.0	even	ok
	156	C45H78N2NaO7S2	845.5143	-3.8	51.3	156	4.58	8.0	even	ok
	157	C30H71N20NaOPS2	845.5191	1.9	51.3	157	17.84	6.0	even	ok
	158	C31H70N18NaO4S2	845.5161	-1.6	51.4	158	20.79	6.0	even	ok
	159	C44H69N12OS2	845.5153	-2.6	51.5	159	11.84	17.0	even	ok
	160	C37H78N2NaO17	845.5193	2.1	51.5	160	12.57	0.0	even	ok
	161	C51H69N6O3S	845.5146	-3.4	51.6	161	6.52	21.0	even	ok
	162	C32H71N16NaO5PS	845.5144	-3.7	51.6	162	4.09	6.0	even	ok
	163	C30H66N22O3PS	845.5141	-4.0	52.0	163	3.05	10.0	even	ok
	164	C38H82N2NaO12S2	845.5201	3.1	52.0	164	7.66	-1.0	even	ok
	165	C34H70N12NaO11	845.5179	0.5	52.1	165	27.37	6.0	even	ok
	166	C35H73N8O15	845.5190	1.8	52.3	166	14.87	4.0	even	ok
	167	C38H87O11P2S2	845.5160	-1.8	52.4	167	18.29	-3.0	even	ok
	168	C46H78N4O4PS2	845.5197	2.6	52.8	168	11.19	11.0	even	ok
	169	C32H65N18O9	845.5176	0.2	53.3	169	29.96	10.0	even	ok
	170	C31H62N22NaO5	845.5166	-1.1	53.6	170	20.54	12.0	even	ok
	171	C31H72N18NaO2P2S	845.5174	-0.1	53.6	171	30.27	6.0	even	ok

Compound Spectrum SmartFormula Report

Meas. m/z	#	Ion Formula	m/z	err [ppm]	mSigma	# mSigma	Score	rdb	e¯ Conf	N-Rule
	172	C31H74N16O5PS2	845.5202	3.2	53.7	172	7.09	4.0	even	ok
	173	C53H74NaO5S	845.5149	-3.0	53.8	173	7.83	17.0	even	ok
	174	C46H74N6NaO3S2	845.5156	-2.2	53.9	174	13.60	13.0	even	ok
	175	C32H73N14O8S2	845.5172	-0.4	54.1	175	34.18	4.0	even	ok
	176	C29H67N24P2S	845.5171	-0.5	54.2	176	26.32	10.0	even	ok
	177	C33H79N10NaO7PS2	845.5204	3.5	54.4	177	5.35	0.0	even	ok
	178	C32H75N14O6P2S	845.5184	1.1	54.8	178	19.18	4.0	even	ok
	179	C34H78N8NaO10S2	845.5175	-0.0	54.9	179	37.45	0.0	even	ok
	180	C29H57N28O3	845.5163	-1.4	55.0	180	16.72	16.0	even	ok
	181	C36H80N4NaO12P2	845.5140	-4.1	55.2	181	2.50	0.0	even	ok
	182	C37H83O16P2	845.5151	-2.8	55.4	182	6.90	-2.0	even	ok
	183	C33H71N14NaO8P	845.5209	4.1	55.5	183	2.58	6.0	even	ok
	184	C34H83N6O9P2S2	845.5133	-5.0	56.2	184	1.28	-2.0	even	ok
	185	C34H75N10O10P2	845.5137	-4.4	56.2	185	1.78	4.0	even	ok
	186	C31H74N12NaO11S	845.5213	4.5	56.5	186	2.05	1.0	even	ok
	187	C31H66N20O6P	845.5206	3.7	56.7	187	3.30	10.0	even	ok
	188	C29H69N18O9S	845.5210	4.2	56.8	188	2.77	5.0	even	ok
	189	C47H74N8PS2	845.5210	4.2	57.0	189	2.80	16.0	even	ok
	190	C36H88NaO11P2S2	845.5135	-4.7	57.0	190	1.74	-6.0	even	ok
	191	C34H82N6O11PS2	845.5215	4.8	57.1	191	1.55	-2.0	even	ok
	192	C30H63N24NaO2P	845.5196	2.5	57.3	192	8.37	12.0	even	ok
	193	C47H77N2O7S2	845.5167	-1.0	57.4	193	23.93	11.0	even	ok
	194	C34H83N2NaO15PS	845.5144	-3.6	57.7	194	4.26	-5.0	even	ok
	195	C27H66N24NaO2S2	845.5134	-4.8	57.8	195	1.46	7.0	even	ok
	196	C35H81N4O14S2	845.5185	1.2	57.8	196	20.85	-2.0	even	ok
	197	C32H78N8O13PS	845.5141	-4.0	57.8	197	3.19	-1.0	even	ok
	198	C36H87NaO13PS2	845.5218	5.1	58.0	198	1.07	-6.0	even	ok
	199	C28H58N30P	845.5193	2.1	58.8	199	9.83	16.0	even	ok
	200	C33H84N4NaO12P2S	845.5174	-0.1	59.2	200	31.75	-5.0	even	ok
	201	C49H79N2NaO2PS2	845.5213	4.5	59.4	201	1.89	12.0	even	ok
	202	C31H79N10O10P2S	845.5171	-0.4	59.4	202	27.83	-1.0	even	ok
	203	C54H74N2O2PS	845.5203	3.3	59.6	203	5.10	20.0	even	ok
	204	C28H69N20O6S2	845.5145	-3.5	59.9	204	4.32	5.0	even	ok
	205	C30H74N14NaO8S2	845.5148	-3.2	60.3	205	5.54	1.0	even	ok
	206	C27H70N22O3PS2	845.5175	-0.0	60.4	206	31.60	5.0	even	ok
	207	C34H87O16P2S	845.5185	1.2	60.5	207	19.77	-7.0	even	ok
	208	C29H75N16NaO5PS2	845.5178	0.3	60.7	208	27.91	1.0	even	ok
	209	C58H69O5	845.5140	-4.2	61.1	209	2.39	25.0	even	ok
	210	C26H71N24P2S2	845.5205	3.5	61.2	210	4.17	5.0	even	ok
	211	C28H76N18NaO2P2S2	845.5207	3.9	61.4	211	3.14	1.0	even	ok
	212	C48H73N6O3S2	845.5180	0.6	61.8	212	23.92	16.0	even	ok
	213	C54H70N4NaOS	845.5163	-1.5	62.1	213	16.10	22.0	even	ok
	214	C28H66N22NaO5S	845.5199	2.9	62.3	214	5.18	7.0	even	ok
	215	C31H77N10O12S2	845.5158	-1.9	62.7	215	11.99	-1.0	even	ok
	216	C30H78N12O9PS2	845.5188	1.6	62.8	216	14.60	-1.0	even	ok
	217	C26H61N28O3S	845.5197	2.6	62.9	217	6.40	11.0	even	ok

Compound Spectrum SmartFormula Report

Meas. m/z	#	Ion Formula	m/z	err [ppm]	mSigma	# mSigma	Score	rdb	e⁻ Conf	N-Rule
	218	C32H83N6NaO11PS2	845.5191	1.9	63.3	218	11.93	-5.0	even	ok
	219	C33H82N4NaO14S2	845.5161	-1.6	63.3	219	14.13	-5.0	even	ok
	220	C57H70N2O2P	845.5169	-0.6	63.7	220	17.79	25.0	even	ok
	221	C33H74N8NaO15	845.5166	-1.1	63.7	221	14.75	1.0	even	ok
	222	C34H77N4O19	845.5177	0.2	64.0	222	20.86	-1.0	even	ok
	223	C50H78NaO5S2	845.5183	1.0	64.2	223	19.06	12.0	even	ok
	224	C55H73O5S	845.5173	-0.2	64.4	224	25.76	20.0	even	ok
	225	C31H69N14O13	845.5163	-1.4	64.7	225	12.10	5.0	even	ok
	226	C30H66N18NaO9	845.5152	-2.7	64.9	226	5.67	7.0	even	ok
	227	C27H68N24NaP2S	845.5147	-3.3	65.2	227	3.49	7.0	even	ok
	228	C30H76N14NaO6P2S	845.5160	-1.7	65.4	228	10.03	1.0	even	ok
	229	C33H86N2O15PS2	845.5202	3.2	65.7	229	4.69	-7.0	even	ok
	230	C28H71N20O4P2S	845.5158	-2.0	65.9	230	8.13	5.0	even	ok
	231	C34H85O18S2	845.5172	-0.4	65.9	231	23.00	-7.0	even	ok
	232	C35H83NaO18P	845.5209	4.1	66.0	232	1.78	-5.0	even	ok
	233	C51H79N2P2S2	845.5154	-2.4	66.0	233	7.99	15.0	even	ok
	234	C28H61N24O7	845.5150	-3.0	66.1	234	4.29	11.0	even	ok
	235	C27H58N28NaO3	845.5139	-4.3	66.6	235	1.47	13.0	even	ok
	236	C30H78N8NaO15S	845.5200	2.9	66.9	236	5.46	-4.0	even	ok
	237	C32H75N10NaO12P	845.5196	2.5	66.9	237	5.97	1.0	even	ok
	238	C33H78N6O16P	845.5206	3.7	66.9	238	2.30	-1.0	even	ok
	239	C28H73N14O13S	845.5197	2.6	67.2	239	6.81	0.0	even	ok
	240	C30H70N16O10P	845.5193	2.1	68.0	240	7.07	5.0	even	ok
	241	C25H53N34O	845.5136	-4.6	68.0	241	1.02	17.0	even	ok
	242	C31H81N4O19S	845.5210	4.2	68.1	242	1.85	-6.0	even	ok
	243	C29H67N20NaO6P	845.5182	0.9	68.4	243	13.59	7.0	even	ok
	244	C25H71N22NaO3PS2	845.5151	-2.9	68.5	244	5.42	2.0	even	ok
	245	C23H66N28OPS2	845.5148	-3.2	68.6	245	4.24	6.0	even	ok
	246	C32H88NaO16P2S	845.5161	-1.7	69.6	246	10.84	-10.0	even	ok
	247	C27H62N26O4P	845.5179	0.5	69.7	247	14.88	11.0	even	ok
	248	C29H78N10NaO12S2	845.5134	-4.8	69.8	248	0.97	-4.0	even	ok
	249	C24H72N24NaP2S2	845.5181	0.7	69.8	249	17.50	2.0	even	ok
	250	C30H83N6O14P2S	845.5158	-2.0	69.9	250	8.86	-6.0	even	ok
	251	C51H74N4NaOS2	845.5196	2.5	70.2	251	6.35	17.0	even	ok
	252	C26H74N18O7PS2	845.5161	-1.6	70.3	252	11.19	0.0	even	ok
	253	C26H59N30NaP	845.5169	-0.7	70.4	253	13.54	13.0	even	ok
	254	C28H79N12NaO9PS2	845.5164	-1.3	70.4	254	13.19	-4.0	even	ok
	255	C25H75N20O4P2S2	845.5191	2.0	71.3	255	8.78	0.0	even	ok
	256	C27H80N14NaO6P2S2	845.5194	2.3	71.4	256	7.17	-4.0	even	ok
	257	C30H81N6O16S2	845.5145	-3.5	71.9	257	2.84	-6.0	even	ok
	258	C28H68N22NaO3P2	845.5212	4.4	72.0	258	1.03	7.0	even	ok
	259	C32H86NaO18S2	845.5148	-3.2	72.4	259	3.63	-10.0	even	ok
	260	C29H82N8O13PS2	845.5175	0.0	72.4	260	20.53	-6.0	even	ok
	261	C31H87N2NaO15PS2	845.5178	0.3	72.7	261	18.02	-10.0	even	ok
	262	C28H83N10O10P2S2	845.5205	3.5	73.1	262	2.68	-6.0	even	ok
	263	C56H69N4OS	845.5187	1.4	73.3	263	11.07	25.0	even	ok

Compound Spectrum SmartFormula Report

Meas. m/z	#	Ion Formula	m/z	err [ppm]	mSigma	# mSigma	Score	rdb	e¯ Conf	N-Rule
	264	C52H77O5S2	845.5207	3.8	73.3	264	2.16	15.0	even	ok
	265	C30H88N4NaO12P2S2	845.5208	3.9	73.3	265	2.01	-10.0	even	ok
	266	C26H63N28OP2	845.5209	4.1	73.4	266	1.33	11.0	even	ok
	267	C24H62N28NaO3S	845.5173	-0.3	73.9	267	14.17	8.0	even	ok
	268	C27H70N18NaO9S	845.5186	1.3	74.1	268	8.87	2.0	even	ok
	269	C25H65N24O7S	845.5183	1.0	74.6	269	10.18	6.0	even	ok
	270	C22H57N34OS	845.5170	-0.6	74.6	270	12.11	12.0	even	ok
	271	C32H78N4NaO19	845.5152	-2.6	75.3	271	3.88	-4.0	even	ok
	272	C33H81O23	845.5163	-1.4	75.7	272	8.12	-6.0	even	ok
	273	C29H70N14NaO13	845.5139	-4.2	76.2	273	1.04	2.0	even	ok
	274	C30H73N10O17	845.5150	-3.0	76.2	274	2.96	0.0	even	ok
	275	C26H71N20NaO6PS	845.5216	4.9	76.4	275	0.56	2.0	even	ok
	276	C23H63N30NaPS	845.5203	3.3	76.6	276	2.33	8.0	even	ok
	277	C26H74N14NaO13S	845.5173	-0.3	76.7	277	15.94	-3.0	even	ok
	278	C26H72N20NaO4P2S	845.5134	-4.9	76.9	278	0.54	2.0	even	ok
	279	C29H80N10NaO10P2S	845.5147	-3.3	77.1	279	2.26	-4.0	even	ok
	280	C24H66N26O4PS	845.5213	4.5	77.1	280	0.76	6.0	even	ok
	281	C60H71NaP	845.5186	1.3	77.2	281	8.08	26.0	even	ok
	282	C29H82N4NaO19S	845.5186	1.3	77.3	282	9.71	-9.0	even	ok
	283	C27H65N20O11	845.5136	-4.6	77.3	283	0.72	6.0	even	ok
	284	C27H75N16O8P2S	845.5144	-3.6	77.6	284	1.69	0.0	even	ok
	285	C27H77N10O17S	845.5183	1.0	77.7	285	11.23	-5.0	even	ok
	286	C31H79N6NaO16P	845.5182	0.9	78.3	286	9.23	-4.0	even	ok
	287	C30H85O23S	845.5197	2.6	78.5	287	4.39	-11.0	even	ok
	288	C32H82N2O20P	845.5193	2.2	78.5	288	4.70	-6.0	even	ok
	289	C28H83N6NaO16PS	845.5216	4.9	79.2	289	0.62	-9.0	even	ok
	290	C29H74N12O14P	845.5180	0.6	79.4	290	10.18	0.0	even	ok
	291	C28H84N6NaO14P2S	845.5134	-4.9	79.4	291	0.62	-9.0	even	ok
	292	C28H71N16NaO10P	845.5169	-0.7	79.5	292	9.55	2.0	even	ok
	293	C26H78N12O14PS	845.5213	4.5	79.6	293	0.85	-5.0	even	ok
	294	C59H65N4O	845.5153	-2.6	79.9	294	3.34	30.0	even	ok
	295	C29H87N2O18P2S	845.5144	-3.6	80.4	295	1.92	-11.0	even	ok
	296	C26H66N22O8P	845.5166	-1.0	80.8	296	7.85	6.0	even	ok
	297	C25H63N26NaO4P	845.5155	-2.3	81.2	297	3.86	8.0	even	ok
	298	C30H80N8NaO13P2	845.5212	4.4	81.5	298	0.70	-4.0	even	ok
	299	C23H58N32O2P	845.5153	-2.6	82.6	299	2.93	12.0	even	ok
	300	C28H75N14O11P2	845.5209	4.1	82.7	300	0.91	0.0	even	ok
	301	C27H72N18NaO7P2	845.5199	2.8	83.0	301	2.50	2.0	even	ok
	302	C25H67N24O5P2	845.5196	2.5	84.3	302	2.97	6.0	even	ok
	303	C24H64N28NaOP2	845.5185	1.2	84.9	303	5.99	8.0	even	ok
	304	C23H66N24NaO7S	845.5159	-1.8	85.6	304	4.22	3.0	even	ok
	305	C20H58N34NaOS	845.5146	-3.4	85.8	305	1.40	9.0	even	ok
	306	C21H61N30O5S	845.5156	-2.2	86.3	306	3.36	7.0	even	ok
	307	C24H69N20O11S	845.5170	-0.6	86.3	307	7.57	1.0	even	ok
	308	C31H82NaO23	845.5139	-4.2	86.9	308	0.68	-9.0	even	ok
	309	C29H77N6O21	845.5136	-4.6	87.8	309	0.48	-5.0	even	ok

Compound Spectrum SmartFormula Report

Meas. m/z	#	Ion Formula	m/z	err [ppm]	mSigma	# mSigma	Score	rdb	e⁻ Conf	N-Rule
	310	C25H75N16NaO10PS	845.5203	3.3	88.1	310	1.43	-3.0	even	ok
	311	C30H83N2NaO20P	845.5169	-0.7	89.8	311	6.23	-9.0	even	ok
	312	C62H70P	845.5210	4.1	90.3	312	0.65	29.0	even	ok
	313	C28H78N8O18P	845.5166	-1.0	90.8	313	5.16	-5.0	even	ok
	314	C27H75N12NaO14P	845.5156	-2.3	90.8	314	2.59	-3.0	even	ok
	315	C25H70N18O12P	845.5153	-2.6	91.9	315	1.98	1.0	even	ok
	316	C24H67N22NaO8P	845.5142	-3.9	92.2	316	0.73	3.0	even	ok
	317	C21H58N32NaO4	845.5211	4.3	92.9	317	0.50	9.0	even	ok
	318	C29H84N4NaO17P2	845.5199	2.8	92.9	318	1.61	-9.0	even	ok
	319	C30H87O21P2	845.5210	4.1	93.0	319	0.58	-11.0	even	ok
	320	C22H62N28O6P	845.5139	-4.2	93.5	320	0.52	7.0	even	ok
	321	C27H79N10O15P2	845.5196	2.5	93.9	321	1.93	-5.0	even	ok
	322	C26H76N14NaO11P2	845.5185	1.3	94.1	322	3.98	-3.0	even	ok
	323	C19H53N38O2	845.5208	3.9	94.2	323	0.63	13.0	even	ok
	324	C24H71N20O9P2	845.5183	0.9	95.3	324	4.40	1.0	even	ok
	325	C23H68N24NaO5P2	845.5172	-0.3	95.7	325	5.49	3.0	even	ok
	326	C21H63N30O3P2	845.5169	-0.7	97.0	326	4.53	7.0	even	ok
861.4917	1	C39H71N14P2S2	861.4897	-2.3	4.8	1	39.46	13.0	even	ok
	2	C39H66N16NaOS2	861.4939	2.6	5.7	2	30.81	15.0	even	ok
	3	C41H76N8NaO2P2S2	861.4900	-1.9	6.0	3	47.36	9.0	even	ok
	4	C44H74N2NaO11S	861.4906	-1.3	7.7	4	65.16	9.0	even	ok
	5	C44H78O12PS	861.4946	3.4	8.8	5	15.75	7.0	even	ok
	6	C40H69N12O5S2	861.4950	3.8	9.1	6	10.82	13.0	even	ok
	7	C42H79N4O6P2S2	861.4911	-0.7	9.4	7	84.15	7.0	even	ok
	8	C42H69N8O9S	861.4903	-1.6	9.9	8	52.62	13.0	even	ok
	9	C46H73N2O11S	861.4930	1.5	10.0	9	56.53	12.0	even	ok
	10	C45H74N4O8PS	861.4959	5.0	10.1	10	3.52	12.0	even	ok
	11	C42H74N6NaO7S2	861.4953	4.2	11.3	11	7.71	9.0	even	ok
	12	C45H75N4O6P2S	861.4877	-4.6	11.5	12	5.00	12.0	even	ok
	13	C36H65N18O3S2	861.4923	0.7	11.5	13	79.77	14.0	even	ok
	14	C38H70N12NaO5S2	861.4926	1.0	11.5	14	68.79	10.0	even	ok
	15	C43H75N4NaO8PS	861.4935	2.2	12.0	15	36.59	9.0	even	ok
	16	C45H70N6NaO7S	861.4919	0.3	12.1	16	95.34	14.0	even	ok
	17	C43H75N8O2P2S2	861.4924	0.9	12.3	17	73.95	12.0	even	ok
	18	C43H78N2O9PS2	861.4881	-4.2	12.8	18	7.51	7.0	even	ok
	19	C37H71N14NaO2PS2	861.4956	4.5	12.9	19	5.29	10.0	even	ok
	20	C43H65N12O5S	861.4916	-0.1	13.0	20	100.00	18.0	even	ok
	21	C44H71N8NaO4PS	861.4949	3.7	13.3	21	11.09	14.0	even	ok
	22	C37H72N14NaP2S2	861.4873	-5.0	13.5	22	3.01	10.0	even	ok
	23	C41H66N12NaO5S	861.4892	-2.9	13.5	23	22.05	15.0	even	ok
	24	C35H66N20PS2	861.4953	4.2	13.7	24	7.16	14.0	even	ok
	25	C39H73N8O9S2	861.4936	2.3	13.8	25	32.74	8.0	even	ok
	26	C40H80N4NaO6P2S2	861.4887	-3.5	14.0	26	13.45	4.0	even	ok
	27	C38H75N10O4P2S2	861.4884	-3.8	14.1	27	10.14	8.0	even	ok
	28	C41H70N10O6PS	861.4933	1.8	14.3	28	42.67	13.0	even	ok
	29	C45H80N2NaO4P2S2	861.4927	1.2	14.4	29	60.80	8.0	even	ok

Compound Spectrum SmartFormula Report

Meas. m/z	#	Ion Formula	m/z	err [ppm]	mSigma	# mSigma	Score	rdb	e¯ Conf	N-Rule
	30	C43H71N10NaOPS2	861.4884	-3.8	14.5	30	9.69	14.0	even	ok
	31	C41H78N2NaO11S2	861.4939	2.6	14.8	31	25.79	4.0	even	ok
	32	C42H66N14O2PS	861.4946	3.4	14.9	32	14.16	18.0	even	ok
	33	C39H61N18O3S	861.4889	-3.2	15.8	33	16.46	19.0	even	ok
	34	C41H73N4O13S	861.4889	-3.2	15.8	34	16.61	8.0	even	ok
	35	C41H83O10P2S2	861.4897	-2.2	16.0	35	32.31	2.0	even	ok
	36	C42H62N16NaOS	861.4905	-1.3	16.2	36	55.08	20.0	even	ok
	37	C44H74N6O5PS2	861.4894	-2.6	16.9	37	24.78	12.0	even	ok
	38	C40H67N14NaO2PS	861.4922	0.6	17.7	38	74.05	15.0	even	ok
	39	C40H70N8NaO9S	861.4879	-4.4	18.0	39	5.29	10.0	even	ok
	40	C42H79NaO12PS	861.4922	0.6	18.2	40	72.99	4.0	even	ok
	41	C48H76N2NaO4P2S	861.4893	-2.7	18.9	41	21.87	13.0	even	ok
	42	C46H79NaO7PS2	861.4897	-2.3	19.1	42	29.47	8.0	even	ok
	43	C46H71N8O2P2S	861.4890	-3.1	19.2	43	17.09	17.0	even	ok
	44	C38H62N20PS	861.4919	0.3	20.0	44	80.28	19.0	even	ok
	45	C40H74N6O10PS	861.4919	0.3	20.1	45	79.74	8.0	even	ok
	46	C38H65N14O7S	861.4876	-4.7	20.1	46	3.65	14.0	even	ok
	47	C47H69N6O7S	861.4943	3.0	20.1	47	16.82	17.0	even	ok
	48	C49H74NaO9S	861.4946	3.4	20.7	48	12.86	13.0	even	ok
	49	C34H66N18NaO3S2	861.4899	-2.1	21.2	49	32.32	11.0	even	ok
	50	C37H74N8NaO9S2	861.4912	-0.5	21.4	50	71.47	5.0	even	ok
	51	C46H66N10NaO3S	861.4932	1.8	21.4	51	37.70	19.0	even	ok
	52	C44H61N16OS	861.4929	1.5	21.5	52	45.06	23.0	even	ok
	53	C35H69N14O7S2	861.4910	-0.8	21.7	53	61.77	9.0	even	ok
	54	C32H61N24OS2	861.4896	-2.4	22.1	54	25.69	15.0	even	ok
	55	C39H71N10NaO6PS	861.4909	-0.9	22.5	55	57.59	10.0	even	ok
	56	C41H80N2NaO9P2S	861.4952	4.1	22.5	56	6.57	4.0	even	ok
	57	C46H76N6NaP2S2	861.4940	2.7	22.8	57	19.93	13.0	even	ok
	58	C38H77N4O13S2	861.4923	0.7	22.9	58	62.70	3.0	even	ok
	59	C36H75N10NaO6PS2	861.4942	3.0	23.4	59	16.62	5.0	even	ok
	60	C39H83NaO12PS2	861.4956	4.5	24.0	60	4.14	-1.0	even	ok
	61	C39H84NaO10P2S2	861.4873	-5.0	24.0	61	2.45	-1.0	even	ok
	62	C34H70N16O4PS2	861.4939	2.6	24.1	62	20.72	9.0	even	ok
	63	C33H67N20NaPS2	861.4929	1.4	24.2	63	44.30	11.0	even	ok
	64	C37H78N6O10PS2	861.4953	4.2	24.2	64	5.67	3.0	even	ok
	65	C39H75N8O7P2S	861.4949	3.8	24.5	65	8.43	8.0	even	ok
	66	C47H79N2O4P2S2	861.4951	4.0	24.6	66	6.95	11.0	even	ok
	67	C37H66N16O4PS	861.4906	-1.3	24.7	67	46.91	14.0	even	ok
	68	C40H77O17S	861.4876	-4.7	24.9	68	3.32	3.0	even	ok
	69	C45H70N10OPS2	861.4908	-1.1	25.2	69	51.38	17.0	even	ok
	70	C38H72N12NaO3P2S	861.4938	2.5	27.0	70	21.03	10.0	even	ok
	71	C47H75N4NaO3PS2	861.4910	-0.7	27.1	71	57.01	13.0	even	ok
	72	C50H75N2O4P2S	861.4917	0.1	27.9	72	72.72	16.0	even	ok
	73	C47H74O12P	861.4912	-0.5	28.2	73	48.98	12.0	even	ok
	74	C39H78N2O14PS	861.4906	-1.3	28.7	74	42.92	3.0	even	ok
	75	C49H72N6NaP2S	861.4907	-1.2	28.9	75	44.53	18.0	even	ok

Compound Spectrum SmartFormula Report

Meas. m/z	#	Ion Formula	m/z	err [ppm]	mSigma	# mSigma	Score	rdb	e¯ Conf	N-Rule
	76	C48H78O7PS2	861.4921	0.5	29.2	76	59.67	11.0	even	ok
	77	C46H69N8O4S2	861.4878	-4.5	29.7	77	3.65	17.0	even	ok
	78	C36H63N20NaPS	861.4895	-2.5	30.0	78	15.83	16.0	even	ok
	79	C38H75N6NaO10PS	861.4895	-2.5	30.3	79	19.84	5.0	even	ok
	80	C48H66N10OPS	861.4874	-5.0	30.4	80	2.26	22.0	even	ok
	81	C51H74O7PS	861.4887	-3.4	30.4	81	9.98	16.0	even	ok
	82	C49H69N2O11	861.4896	-2.4	30.5	82	16.63	17.0	even	ok
	83	C48H65N10O3S	861.4956	4.6	30.7	83	3.32	22.0	even	ok
	84	C50H71N4NaO3PS	861.4877	-4.6	30.9	84	3.15	18.0	even	ok
	85	C50H70N4NaO5S	861.4959	4.9	31.3	85	2.33	18.0	even	ok
	86	C33H70N14NaO7S2	861.4886	-3.6	31.7	86	8.07	6.0	even	ok
	87	C48H74N2NaO6S2	861.4881	-4.2	31.7	87	4.77	13.0	even	ok
	88	C36H78N4NaO13S2	861.4899	-2.1	31.7	88	25.49	0.0	even	ok
	89	C36H67N18OP2S	861.4936	2.2	32.0	89	18.51	14.0	even	ok
	90	C36H70N12O8PS	861.4892	-2.8	32.1	90	15.07	9.0	even	ok
	91	C46H75N2O9P2	861.4942	3.0	32.2	91	10.78	12.0	even	ok
	92	C34H73N10O11S2	861.4896	-2.4	32.2	92	20.46	4.0	even	ok
	93	C48H70N4O8P	861.4926	1.1	32.4	93	34.75	17.0	even	ok
	94	C46H71N4NaO8P	861.4902	-1.7	32.4	94	24.16	14.0	even	ok
	95	C31H65N20O5S2	861.4883	-3.9	32.5	95	5.93	10.0	even	ok
	96	C45H75NaO12P	861.4888	-3.3	32.6	96	8.29	9.0	even	ok
	97	C38H79N4O11P2S	861.4936	2.2	32.7	97	22.53	3.0	even	ok
	98	C37H81O17S2	861.4910	-0.8	32.9	98	47.73	-2.0	even	ok
	99	C49H76NaO7P2	861.4958	4.8	33.1	99	1.93	13.0	even	ok
	100	C35H79N6NaO10PS2	861.4929	1.4	33.9	100	34.87	0.0	even	ok
	101	C48H66N6NaO7	861.4885	-3.7	34.1	101	5.88	19.0	even	ok
	102	C35H67N16NaO4PS	861.4882	-4.1	34.1	102	5.12	11.0	even	ok
	103	C37H76N8NaO7P2S	861.4925	1.0	34.4	103	42.77	5.0	even	ok
	104	C32H71N16NaO4PS2	861.4915	-0.1	34.4	104	60.11	6.0	even	ok
	105	C33H74N12O8PS2	861.4926	1.1	34.6	105	40.33	4.0	even	ok
	106	C36H82N2O14PS2	861.4940	2.7	34.6	106	15.94	-2.0	even	ok
	107	C44H66N10O6P	861.4899	-2.1	34.8	107	18.78	18.0	even	ok
	108	C47H71N6O5P2	861.4956	4.5	35.0	108	2.57	17.0	even	ok
	109	C43H70N6O10P	861.4886	-3.6	35.1	109	5.93	13.0	even	ok
	110	C30H66N22O2PS2	861.4913	-0.5	35.5	110	51.68	10.0	even	ok
	111	C46H61N12O5	861.4882	-4.0	35.6	111	4.23	23.0	even	ok
	112	C47H67N8NaO4P	861.4915	-0.2	36.3	112	45.22	19.0	even	ok
	113	C35H71N14O5P2S	861.4922	0.6	36.3	113	47.06	9.0	even	ok
	114	C45H72N6NaO5P2	861.4932	1.7	36.4	114	21.98	14.0	even	ok
	115	C34H80N8NaO7P2S2	861.4959	4.9	36.4	115	2.13	0.0	even	ok
	116	C42H66N10NaO8	861.4957	4.7	36.7	116	2.03	15.0	even	ok
	117	C42H73N2O16	861.4955	4.4	36.7	117	2.79	8.0	even	ok
	118	C32H75N14O5P2S2	861.4956	4.6	37.3	118	2.90	4.0	even	ok
	119	C44H76N2NaO9P2	861.4918	0.2	37.3	119	44.15	9.0	even	ok
	120	C31H72N18NaOP2S2	861.4945	3.3	37.5	120	8.91	6.0	even	ok
	121	C45H62N14O2P	861.4912	-0.5	38.1	121	37.99	23.0	even	ok

Compound Spectrum SmartFormula Report

Meas. m/z	#	Ion Formula	m/z	err [ppm]	mSigma	# mSigma	Score	rdb	e¯ Conf	N-Rule
	122	C49H74N4O3PS2	861.4934	2.1	38.2	122	21.52	16.0	even	ok
	123	C52H70NaO9	861.4912	-0.5	38.3	123	37.29	18.0	even	ok
	124	C51H71N6P2S	861.4931	1.6	38.6	124	27.63	21.0	even	ok
	125	C51H71N2NaO6P	861.4942	2.9	38.6	125	9.41	18.0	even	ok
	126	C43H67N12O3P2	861.4929	1.4	38.8	126	24.63	18.0	even	ok
	127	C43H63N14NaO2P	861.4888	-3.3	39.0	127	6.95	20.0	even	ok
	128	C46H68N10NaOP2	861.4945	3.3	39.0	128	7.07	19.0	even	ok
	129	C50H65N6O7	861.4909	-0.9	39.0	129	31.81	22.0	even	ok
	130	C42H67N10NaO6P	861.4875	-4.9	39.1	130	1.63	15.0	even	ok
	131	C47H65N12S2	861.4891	-3.0	39.1	131	11.18	22.0	even	ok
	132	C40H61N16O6	861.4955	4.4	39.2	132	2.64	19.0	even	ok
	133	C37H79N2NaO14PS	861.4882	-4.1	39.2	133	4.53	0.0	even	ok
	134	C53H76NaO2P2S	861.4933	1.9	39.3	134	22.46	17.0	even	ok
	135	C49H66N8O4P	861.4939	2.6	39.7	135	11.57	22.0	even	ok
	136	C33H62N22O2PS	861.4879	-4.4	39.8	136	2.59	15.0	even	ok
	137	C42H71N8O7P2	861.4915	-0.1	39.9	137	41.67	13.0	even	ok
	138	C41H70N6NaO12	861.4944	3.2	40.1	138	7.59	10.0	even	ok
	139	C32H58N26NaS	861.4951	3.9	40.3	139	3.89	17.0	even	ok
	140	C34H70N12NaO10S	861.4951	4.0	40.7	140	4.75	6.0	even	ok
	141	C35H74N8O12PS	861.4879	-4.4	40.8	141	3.18	4.0	even	ok
	142	C49H70N6NaO2S2	861.4894	-2.7	40.9	142	13.50	18.0	even	ok
	143	C52H70N4O3PS	861.4901	-1.9	41.1	143	22.51	21.0	even	ok
	144	C41H58N20P	861.4885	-3.6	41.3	144	4.97	24.0	even	ok
	145	C37H83O15P2S	861.4922	0.7	41.9	145	40.29	-2.0	even	ok
	146	C54H69O9	861.4936	2.2	41.9	146	17.26	21.0	even	ok
	147	C49H62N10NaO3	861.4899	-2.1	42.0	147	15.06	24.0	even	ok
	148	C35H82NaO17S2	861.4886	-3.6	42.2	148	6.18	-5.0	even	ok
	149	C32H65N18O8S	861.4948	3.6	42.4	149	6.04	10.0	even	ok
	150	C34H68N18NaOP2S	861.4912	-0.6	42.4	150	32.67	11.0	even	ok
	151	C39H65N12O10	861.4941	2.8	42.6	151	9.05	14.0	even	ok
	152	C50H73N2O6S2	861.4905	-1.4	42.7	152	27.57	16.0	even	ok
	153	C33H77N6O15S2	861.4883	-3.9	42.7	153	4.58	-1.0	even	ok
	154	C48H63N12NaP	861.4928	1.4	43.0	154	22.41	24.0	even	ok
	155	C47H57N16O	861.4896	-2.4	43.0	155	11.84	28.0	even	ok
	156	C36H80N4NaO11P2S	861.4912	-0.6	43.1	156	40.33	0.0	even	ok
	157	C39H58N20NaO2	861.4944	3.1	43.2	157	7.02	21.0	even	ok
	158	C41H68N12NaO3P2	861.4905	-1.4	43.8	158	21.62	15.0	even	ok
	159	C41H75N4O11P2	861.4902	-1.7	44.0	159	18.14	8.0	even	ok
	160	C34H83N2NaO14PS2	861.4916	-0.1	44.4	160	46.13	-5.0	even	ok
	161	C34H75N10O9P2S	861.4909	-0.9	44.7	161	33.39	4.0	even	ok
	162	C31H75N12NaO8PS2	861.4902	-1.7	44.8	162	22.15	1.0	even	ok
	163	C32H78N8O12PS2	861.4913	-0.5	45.0	163	39.97	-1.0	even	ok
	164	C29H70N18O6PS2	861.4899	-2.0	45.8	164	17.79	5.0	even	ok
	165	C37H53N26	861.4941	2.8	45.8	165	8.34	25.0	even	ok
	166	C28H67N22NaO2PS2	861.4889	-3.3	46.0	166	7.36	7.0	even	ok
	167	C40H74N2NaO16	861.4931	1.6	46.2	167	17.96	5.0	even	ok

Compound Spectrum SmartFormula Report

Meas. m/z	#	Ion Formula	m/z	err [ppm]	mSigma	# mSigma	Score	rdb	e⁻ Conf	N-Rule
	168	C38H62N16NaO6	861.4930	1.6	46.2	168	18.08	16.0	even	ok
	169	C53H71N2O4P2	861.4884	-3.8	46.4	169	3.56	21.0	even	ok
	170	C39H63N18OP2	861.4902	-1.7	46.4	170	16.83	19.0	even	ok
	171	C33H84N4NaO11P2S2	861.4945	3.3	46.8	171	6.80	-5.0	even	ok
	172	C26H62N28PS2	861.4886	-3.6	47.2	172	5.43	11.0	even	ok
	173	C34H87O15P2S2	861.4956	4.6	47.3	173	2.17	-7.0	even	ok
	174	C40H72N8NaO7P2	861.4891	-2.9	47.5	174	7.26	10.0	even	ok
	175	C31H79N10O9P2S2	861.4943	3.0	47.7	175	8.55	-1.0	even	ok
	176	C30H76N14NaO5P2S2	861.4932	1.8	47.7	176	19.56	1.0	even	ok
	177	C52H67N6NaO2P	861.4955	4.5	48.2	177	1.86	23.0	even	ok
	178	C38H69N8O14	861.4928	1.3	48.6	178	19.89	9.0	even	ok
	179	C53H66N4NaO5	861.4925	1.0	48.7	179	22.65	23.0	even	ok
	180	C36H57N22O4	861.4928	1.3	48.8	180	19.91	20.0	even	ok
	181	C50H62N12P	861.4953	4.2	48.8	181	2.50	27.0	even	ok
	182	C28H71N20O3P2S2	861.4929	1.4	48.8	182	22.66	5.0	even	ok
	183	C31H62N22NaO4S	861.4937	2.4	48.9	183	10.29	12.0	even	ok
	184	C34H77N4O18S	861.4948	3.6	48.9	184	4.95	-1.0	even	ok
	185	C52H68N6NaP2	861.4873	-5.1	48.9	185	0.96	23.0	even	ok
	186	C51H61N10O3	861.4923	0.7	49.0	186	25.95	27.0	even	ok
	187	C52H75N2NaOPS2	861.4951	3.9	49.8	187	3.70	17.0	even	ok
	188	C33H74N8NaO14S	861.4937	2.4	49.8	188	12.40	1.0	even	ok
	189	C55H75O2P2S	861.4958	4.7	49.9	189	1.70	20.0	even	ok
	190	C38H67N14O5P2	861.4889	-3.3	50.0	190	5.23	14.0	even	ok
	191	C40H79O15P2	861.4889	-3.3	50.5	191	5.21	3.0	even	ok
	192	C29H57N28O2S	861.4935	2.1	50.9	192	11.91	16.0	even	ok
	193	C39H75N4NaO13P	861.4960	5.1	51.2	193	0.91	5.0	even	ok
	194	C37H63N18NaO3P	861.4960	5.1	51.2	194	0.92	16.0	even	ok
	195	C31H69N14O12S	861.4935	2.1	51.3	195	14.59	5.0	even	ok
	196	C33H72N14NaO5P2S	861.4898	-2.1	51.4	196	11.18	6.0	even	ok
	197	C37H66N12NaO10	861.4917	0.0	51.6	197	30.76	11.0	even	ok
	198	C35H84NaO15P2S	861.4898	-2.1	52.3	198	13.68	-5.0	even	ok
	199	C51H69N6O2S2	861.4918	0.1	52.3	199	36.27	21.0	even	ok
	200	C35H54N26Na	861.4917	0.0	52.5	200	30.09	22.0	even	ok
	201	C30H66N18NaO8S	861.4924	0.8	52.6	201	27.14	7.0	even	ok
	202	C55H71N2NaOPS	861.4917	0.0	52.9	202	37.14	22.0	even	ok
	203	C31H67N20O3P2S	861.4895	-2.5	53.3	203	8.50	10.0	even	ok
	204	C39H76N4NaO11P2	861.4878	-4.5	53.4	204	1.56	5.0	even	ok
	205	C37H70N10O11P	861.4958	4.8	53.5	205	1.20	9.0	even	ok
	206	C37H64N18NaOP2	861.4878	-4.5	53.6	206	1.54	16.0	even	ok
	207	C35H58N24OP	861.4958	4.7	53.7	207	1.20	20.0	even	ok
	208	C33H79N6O13P2S	861.4896	-2.5	53.8	208	10.57	-1.0	even	ok
	209	C35H61N18O8	861.4914	-0.3	54.0	209	26.08	15.0	even	ok
	210	C53H74NaO4S2	861.4921	0.5	54.2	210	30.24	17.0	even	ok
	211	C54H65N6O2S	861.4884	-3.8	54.9	211	3.65	26.0	even	ok
	212	C32H76N10NaO9P2S	861.4885	-3.7	55.0	212	3.91	1.0	even	ok
	213	C30H79N8NaO12PS2	861.4889	-3.3	55.2	213	5.60	-4.0	even	ok

Compound Spectrum SmartFormula Report

Meas. m/z	#	Ion Formula	m/z	err [ppm]	mSigma	# mSigma	Score	rdb	e¯ Conf	N-Rule
	214	C27H70N18NaO8S2	861.4958	4.7	55.3	214	1.42	2.0	even	ok
	215	C31H82N4O16PS2	861.4899	-2.0	55.5	215	13.28	-6.0	even	ok
	216	C37H71N10O9P2	861.4875	-4.8	55.7	216	1.04	9.0	even	ok
	217	C56H70NaO4S	861.4887	-3.4	56.1	217	4.65	22.0	even	ok
	218	C28H74N14O10PS2	861.4886	-3.6	56.1	218	4.17	0.0	even	ok
	219	C27H71N18NaO6PS2	861.4875	-4.8	56.1	219	1.28	2.0	even	ok
	220	C37H73N4O18	861.4914	-0.3	56.2	220	24.40	4.0	even	ok
	221	C25H65N24O6S2	861.4955	4.4	56.3	221	1.90	6.0	even	ok
	222	C36H67N14NaO7P	861.4947	3.5	56.6	222	3.47	11.0	even	ok
	223	C24H62N28NaO2S2	861.4944	3.2	56.6	223	5.66	8.0	even	ok
	224	C56H72NaO2P2	861.4900	-2.0	56.8	224	10.48	22.0	even	ok
	225	C54H67N6P2	861.4897	-2.3	56.9	225	8.51	26.0	even	ok
	226	C34H58N22NaO4	861.4904	-1.5	57.2	226	13.32	17.0	even	ok
	227	C32H88NaO15P2S2	861.4932	1.8	57.3	227	14.40	-10.0	even	ok
	228	C22H57N34S2	861.4941	2.9	57.8	228	6.97	12.0	even	ok
	229	C29H80N10NaO9P2S2	861.4919	0.2	58.0	229	29.42	-4.0	even	ok
	230	C55H65N4O5	861.4949	3.8	58.0	230	2.58	26.0	even	ok
	231	C30H83N6O13P2S2	861.4929	1.5	58.1	231	16.79	-6.0	even	ok
	232	C33H81O22S	861.4935	2.1	58.7	232	11.43	-6.0	even	ok
	233	C36H70N8NaO14	861.4904	-1.5	58.7	233	12.77	6.0	even	ok
	234	C38H79NaO17P	861.4947	3.5	58.8	234	3.19	0.0	even	ok
	235	C27H75N16O7P2S2	861.4916	-0.1	59.0	235	29.62	0.0	even	ok
	236	C34H62N20O5P	861.4944	3.2	59.0	236	4.18	15.0	even	ok
	237	C26H72N20NaO3P2S2	861.4905	-1.3	59.2	237	17.13	2.0	even	ok
	238	C32H78N4NaO18S	861.4924	0.8	59.3	238	21.71	-4.0	even	ok
	239	C54H62N8NaO	861.4939	2.6	59.6	239	6.50	28.0	even	ok
	240	C32H53N28O2	861.4901	-1.8	59.7	240	10.22	21.0	even	ok
	241	C28H61N24O6S	861.4921	0.5	60.3	241	19.41	11.0	even	ok
	242	C24H67N26OP2S2	861.4902	-1.7	60.4	242	13.82	6.0	even	ok
	243	C30H73N10O16S	861.4921	0.5	60.7	243	23.84	0.0	even	ok
	244	C34H65N14O12	861.4901	-1.8	61.0	244	9.86	10.0	even	ok
	245	C36H74N6O15P	861.4944	3.2	61.0	245	3.87	4.0	even	ok
	246	C29H70N14NaO12S	861.4911	-0.7	61.7	246	21.27	2.0	even	ok
	247	C27H58N28NaO2S	861.4910	-0.7	62.1	247	16.69	13.0	even	ok
	248	C33H59N24NaOP	861.4933	1.9	62.2	248	8.87	17.0	even	ok
	249	C31H79N6NaO15PS	861.4954	4.3	62.8	249	1.69	-4.0	even	ok
	250	C29H67N20NaO5PS	861.4954	4.3	62.8	250	1.37	7.0	even	ok
	251	C30H71N16O7P2S	861.4882	-4.0	62.9	251	1.79	5.0	even	ok
	252	C32H83N2O17P2S	861.4882	-4.0	63.2	252	2.24	-6.0	even	ok
	253	C35H71N10NaO11P	861.4934	2.0	63.5	253	8.40	6.0	even	ok
	254	C33H62N18NaO8	861.4890	-3.1	63.7	254	3.87	12.0	even	ok
	255	C57H70N2OPS	861.4941	2.8	63.9	255	5.85	25.0	even	ok
	256	C25H53N34S	861.4908	-1.1	64.0	256	13.45	17.0	even	ok
	257	C54H70N4NaS2	861.4934	2.0	64.2	257	9.88	22.0	even	ok
	258	C29H74N12O13PS	861.4951	4.0	64.3	258	2.19	0.0	even	ok
	259	C27H62N26O3PS	861.4951	4.0	64.7	259	1.74	11.0	even	ok

Compound Spectrum SmartFormula Report

Meas. m/z	#	Ion Formula	m/z	err [ppm]	mSigma	# mSigma	Score	rdb	e¯ Conf	N-Rule
	260	C36H77O22	861.4901	-1.8	65.0	260	8.64	-1.0	even	ok
	261	C29H82N4NaO18S2	861.4958	4.8	65.0	261	1.01	-9.0	even	ok
	262	C26H74N14NaO12S2	861.4944	3.2	65.6	262	4.13	-3.0	even	ok
	263	C29H83N4NaO16PS2	861.4875	-4.8	65.6	263	0.94	-9.0	even	ok
	264	C55H73O4S2	861.4945	3.3	65.7	264	3.91	20.0	even	ok
	265	C27H77N10O16S2	861.4955	4.4	65.8	265	1.36	-5.0	even	ok
	266	C33H66N16O9P	861.4931	1.6	65.8	266	9.36	10.0	even	ok
	267	C31H57N24O6	861.4887	-3.4	66.0	267	2.76	16.0	even	ok
	268	C30H86O20PS2	861.4886	-3.6	66.0	268	3.00	-11.0	even	ok
	269	C24H69N20O10S2	861.4941	2.9	66.5	269	5.12	1.0	even	ok
	270	C58H71O2P2	861.4924	0.8	66.5	270	13.69	25.0	even	ok
	271	C23H66N24NaO6S2	861.4931	1.6	66.6	271	11.36	3.0	even	ok
	272	C57H66N4NaS	861.4900	-1.9	66.9	272	9.66	27.0	even	ok
	273	C35H74N4NaO18	861.4890	-3.1	67.0	273	3.48	1.0	even	ok
	274	C58H69O4S	861.4911	-0.7	67.3	274	17.90	25.0	even	ok
	275	C21H61N30O4S2	861.4928	1.3	67.7	275	12.99	7.0	even	ok
	276	C20H58N34NaS2	861.4917	0.1	68.2	276	21.79	9.0	even	ok
	277	C28H84N6NaO13P2S2	861.4905	-1.3	68.3	277	12.56	-9.0	even	ok
	278	C29H87N2O17P2S2	861.4916	-0.1	68.5	278	21.33	-11.0	even	ok
	279	C32H63N20NaO5P	861.4920	0.4	68.5	279	15.24	12.0	even	ok
	280	C30H54N28NaO2	861.4877	-4.6	68.9	280	0.79	18.0	even	ok
	281	C33H69N10O16	861.4888	-3.4	69.1	281	2.49	5.0	even	ok
	282	C31H82NaO22S	861.4911	-0.7	69.1	282	16.44	-9.0	even	ok
	283	C35H78N2O19P	861.4931	1.6	69.5	283	8.12	-1.0	even	ok
	284	C58H67N2NaOP	861.4883	-3.9	69.7	284	1.58	27.0	even	ok
	285	C29H77N6O20S	861.4908	-1.0	70.3	285	13.56	-5.0	even	ok
	286	C27H65N20O10S	861.4908	-1.0	70.3	286	10.78	6.0	even	ok
	287	C30H58N26O3P	861.4917	0.1	70.8	287	15.78	16.0	even	ok
	288	C28H74N10NaO16S	861.4897	-2.3	71.0	288	6.59	-3.0	even	ok
	289	C28H49N34	861.4874	-5.0	71.2	289	0.52	22.0	even	ok
	290	C32H66N14NaO12	861.4877	-4.6	71.3	290	0.73	7.0	even	ok
	291	C34H75N6NaO15P	861.4920	0.4	71.6	291	13.53	1.0	even	ok
	292	C26H62N24NaO6S	861.4897	-2.3	71.7	292	5.11	8.0	even	ok
	293	C30H83N2NaO19PS	861.4941	2.8	72.4	293	4.45	-9.0	even	ok
	294	C26H69N16O14S	861.4894	-2.6	72.4	294	5.03	1.0	even	ok
	295	C28H71N16NaO9PS	861.4940	2.8	72.7	295	3.54	2.0	even	ok
	296	C31H64N22NaO2P2	861.4950	3.9	73.4	296	1.41	12.0	even	ok
	297	C24H57N30O4S	861.4894	-2.6	73.4	297	3.83	12.0	even	ok
	298	C30H61N20O10	861.4874	-5.0	73.5	298	0.48	11.0	even	ok
	299	C28H78N8O17PS	861.4938	2.4	73.7	299	5.32	-5.0	even	ok
	300	C32H70N12O13P	861.4917	0.1	73.7	300	14.11	5.0	even	ok
	301	C61H65O4	861.4877	-4.6	74.5	301	0.86	30.0	even	ok
	302	C26H66N22O7PS	861.4938	2.4	74.5	302	4.16	6.0	even	ok
	303	C27H75N12NaO13PS	861.4927	1.2	74.5	303	10.63	-3.0	even	ok
	304	C23H54N34NaS	861.4884	-3.8	75.1	304	1.34	14.0	even	ok
	305	C29H59N28P2	861.4947	3.5	75.7	305	1.71	16.0	even	ok

Compound Spectrum SmartFormula Report

Meas. m/z	#	Ion Formula	m/z	err [ppm]	mSigma	# mSigma	Score	rdb	e¯ Conf	N-Rule
	306	C25H63N26NaO3PS	861.4927	1.2	75.9	306	8.09	8.0	even	ok
	307	C56H69N4S2	861.4958	4.8	75.9	307	0.64	25.0	even	ok
	308	C31H67N16NaO9P	861.4907	-1.2	76.0	308	8.20	7.0	even	ok
	309	C34H78NaO22	861.4877	-4.6	76.1	309	0.62	-4.0	even	ok
	310	C33H76N8NaO12P2	861.4950	3.9	76.2	310	1.25	1.0	even	ok
	311	C23H58N32OPS	861.4924	0.9	77.8	311	8.78	12.0	even	ok
	312	C32H73N6O20	861.4874	-4.9	77.9	312	0.41	0.0	even	ok
	313	C29H62N22O7P	861.4904	-1.5	78.2	313	6.37	11.0	even	ok
	314	C59H65N4S	861.4924	0.9	78.2	314	10.63	30.0	even	ok
	315	C31H71N14O10P2	861.4947	3.6	78.3	315	1.52	5.0	even	ok
	316	C28H81N2O24S	861.4894	-2.6	80.1	316	3.76	-10.0	even	ok
	317	C24H64N28NaP2S	861.4957	4.7	80.2	317	0.50	8.0	even	ok
	318	C33H79N2NaO19P	861.4907	-1.1	80.4	318	6.91	-4.0	even	ok
	319	C60H66N2OP	861.4907	-1.1	80.5	319	7.06	30.0	even	ok
	320	C27H78N6NaO20S	861.4884	-3.8	80.6	320	1.38	-8.0	even	ok
	321	C28H59N26NaO3P	861.4893	-2.7	80.7	321	2.65	13.0	even	ok
	322	C30H68N18NaO6P2	861.4937	2.3	80.7	322	3.51	7.0	even	ok
	323	C25H66N20NaO10S	861.4884	-3.8	81.7	323	1.04	3.0	even	ok
	324	C25H73N12O18S	861.4881	-4.1	81.8	324	0.97	-4.0	even	ok
	325	C31H74N8O17P	861.4904	-1.5	82.4	325	5.41	0.0	even	ok
	326	C28H63N24O4P2	861.4934	2.0	82.9	326	3.94	11.0	even	ok
	327	C26H54N32OP	861.4890	-3.0	82.9	327	1.90	17.0	even	ok
	328	C33H83O20P2	861.4947	3.6	83.1	328	1.24	-6.0	even	ok
	329	C23H61N26O8S	861.4881	-4.2	83.3	329	0.72	7.0	even	ok
	330	C30H71N12NaO13P	861.4893	-2.7	84.3	330	2.30	2.0	even	ok
	331	C25H70N18O11PS	861.4924	0.9	84.6	331	6.60	1.0	even	ok
	332	C32H80N4NaO16P2	861.4937	2.3	84.9	332	2.93	-4.0	even	ok
	333	C62H61N4	861.4891	-3.0	85.3	333	2.20	35.0	even	ok
	334	C27H60N28NaP2	861.4923	0.7	85.4	334	6.76	13.0	even	ok
	335	C24H67N22NaO7PS	861.4914	-0.4	85.8	335	7.81	3.0	even	ok
	336	C28H66N18O11P	861.4891	-3.0	86.3	336	1.66	6.0	even	ok
	337	C30H75N10O14P2	861.4934	2.0	86.8	337	3.31	0.0	even	ok
	338	C26H76N14NaO10P2S	861.4957	4.7	87.0	338	0.37	-3.0	even	ok
	339	C22H62N28O5PS	861.4911	-0.7	87.4	339	6.36	7.0	even	ok
	340	C27H63N22NaO7P	861.4880	-4.3	88.5	340	0.52	8.0	even	ok
	341	C24H71N20O8P2S	861.4954	4.3	88.6	341	0.48	1.0	even	ok
	342	C25H57N28O7	861.4960	5.0	88.7	342	0.25	12.0	even	ok
	343	C29H72N14NaO10P2	861.4923	0.8	88.8	343	5.80	2.0	even	ok
	344	C21H59N32NaOPS	861.4900	-1.9	88.9	344	3.17	9.0	even	ok
	345	C23H68N24NaO4P2S	861.4944	3.1	89.9	345	1.35	3.0	even	ok
	346	C25H58N28O5P	861.4877	-4.6	90.6	346	0.34	12.0	even	ok
	347	C27H67N20O8P2	861.4920	0.4	90.8	347	6.08	6.0	even	ok
	348	C24H54N32NaO3	861.4949	3.7	91.0	348	0.77	14.0	even	ok
	349	C21H63N30O2P2S	861.4941	2.8	91.6	349	1.59	7.0	even	ok
	350	C30H78N4O21P	861.4891	-3.0	91.6	350	1.33	-5.0	even	ok
	351	C26H64N24NaO4P2	861.4910	-0.8	93.1	351	4.72	8.0	even	ok

Compound Spectrum SmartFormula Report

Meas. m/z	#	Ion Formula	m/z	err [ppm]	mSigma	# mSigma	Score	rdb	e¯ Conf	N-Rule
	352	C22H49N38O	861.4946	3.4	93.1	352	0.92	18.0	even	ok
	353	C29H75N8NaO17P	861.4880	-4.3	93.2	353	0.43	-3.0	even	ok
	354	C27H69N14O17	861.4960	5.0	93.4	354	0.20	1.0	even	ok
	355	C31H84NaO20P2	861.4923	0.8	94.0	355	4.58	-9.0	even	ok
	356	C27H70N14O15P	861.4877	-4.6	95.1	356	0.28	1.0	even	ok
	357	C24H59N30O2P2	861.4907	-1.1	95.2	357	3.69	12.0	even	ok
	358	C26H66N18NaO13	861.4949	3.7	95.2	358	0.63	3.0	even	ok
	359	C29H79N6O18P2	861.4921	0.4	95.8	359	4.83	-5.0	even	ok
	360	C24H61N24O11	861.4946	3.4	97.1	360	0.76	7.0	even	ok
	361	C28H76N10NaO14P2	861.4910	-0.8	97.6	361	3.86	-3.0	even	ok
	362	C23H58N28NaO7	861.4935	2.2	99.1	362	1.71	9.0	even	ok
	363	C26H71N16O12P2	861.4907	-1.1	99.4	363	3.04	1.0	even	ok
	364	C29H81O27	861.4960	5.0	99.8	364	0.15	-10.0	even	ok

Cmpd 11, 20.2 min

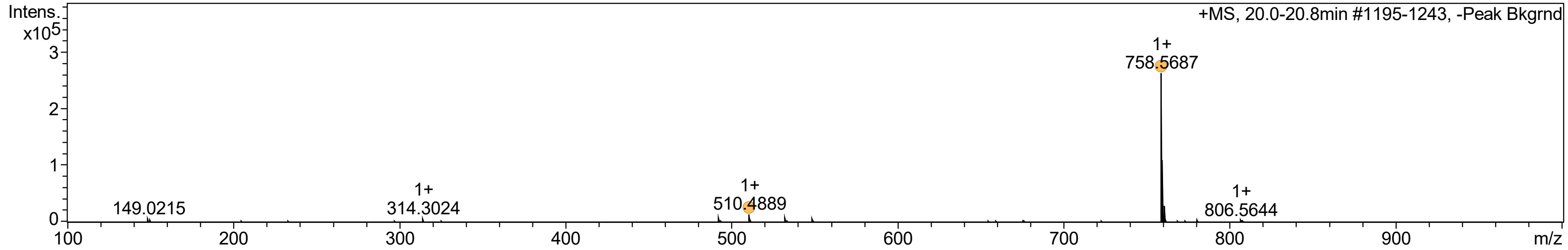

Meas. m/z	#	Ion Formula	m/z	err [ppm]	mSigma	# mSigma	Score	rdb	e¯ Conf	N-Rule
510.4889	1	C26H65N5O2P	510.4870	-3.6	3.1	1	40.52	-2.0	even	ok
	2	C27H71NNaOP2	510.4903	2.8	4.5	2	53.01	-6.0	even	ok
	3	C24H70N3O3P2	510.4887	-0.4	15.8	3	83.28	-7.0	even	ok
	4	C29H66N3NaP	510.4887	-0.4	15.8	4	81.91	-1.0	even	ok
	5	C23H65N7NaOS	510.4864	-5.0	19.9	5	22.63	-5.0	even	ok
	6	C23H69N5O2PS	510.4904	3.0	21.4	6	47.64	-7.0	even	ok
	7	C25H64N7OS	510.4888	-0.3	22.0	7	100.00	-2.0	even	ok
	8	C24H68N3O5S	510.4874	-2.9	23.9	8	46.86	-7.0	even	ok
	9	C27H69NNaO3S	510.4890	0.3	24.3	9	94.09	-6.0	even	ok
	10	C31H65N3P	510.4911	4.3	24.9	10	27.37	2.0	even	ok
	11	C32H64NO3	510.4881	-1.6	28.0	11	62.93	2.0	even	ok
	12	C29H68NO3S	510.4914	5.0	31.2	12	17.11	-3.0	even	ok
	13	C26H73NOPS2	510.4866	-4.5	40.1	13	20.87	-8.0	even	ok
758.5687	1	C37H81N3O10P	758.5654	-4.3	4.5	1	11.10	0.0	even	ok
	2	C36H82N5O7P2	758.5684	-0.3	7.1	2	91.00	0.0	even	ok
	3	C34H72N13O6	758.5723	4.8	8.0	3	6.78	6.0	even	ok

Compound Spectrum SmartFormula Report

Meas. m/z	#	Ion Formula	m/z	err [ppm]	mSigma	# mSigma	Score	rdb	e¯ Conf	N-Rule
	4	C35H81N3NaO12	758.5712	3.4	8.6	4	19.20	-3.0	even	ok
	5	C30H74N17NaPS	758.5664	-3.0	9.5	5	24.43	3.0	even	ok
	6	C33H76N9O10	758.5710	3.0	10.5	6	23.55	1.0	even	ok
	7	C35H79N9NaO3P2	758.5673	-1.7	11.2	7	47.76	2.0	even	ok
	8	C32H73N17PS	758.5688	0.2	11.3	8	89.36	6.0	even	ok
	9	C33H69N17NaO2	758.5712	3.4	11.9	9	18.22	8.0	even	ok
	10	C30H78N15OP2S	758.5704	2.3	12.0	10	34.76	1.0	even	ok
	11	C35H86NO11P2	758.5671	-2.1	12.9	11	38.70	-5.0	even	ok
	12	C32H83N9NaO3P2S	758.5707	2.7	13.0	12	27.73	-3.0	even	ok
	13	C32H73N13NaO6	758.5699	1.6	13.0	13	48.40	3.0	even	ok
	14	C31H77N13O4PS	758.5674	-1.6	13.0	14	49.05	1.0	even	ok
	15	C33H74N15OP2	758.5671	-2.1	13.5	15	38.03	6.0	even	ok
	16	C34H78N11NaO2PS	758.5690	0.5	13.8	16	75.35	2.0	even	ok
	17	C31H64N23	758.5710	3.0	13.9	17	22.23	12.0	even	ok
	18	C39H83N3NaO5P2	758.5700	1.8	14.0	18	44.07	1.0	even	ok
	19	C37H78N9O3P2	758.5697	1.4	14.4	19	51.91	5.0	even	ok
	20	C33H82N7NaO6PS	758.5677	-1.2	14.7	20	55.94	-3.0	even	ok
	21	C34H83N5NaO7P2	758.5660	-3.5	14.9	21	16.03	-3.0	even	ok
	22	C38H77N7O6P	758.5667	-2.5	15.3	22	29.48	5.0	even	ok
	23	C30H68N19O4	758.5696	1.3	15.3	23	54.51	7.0	even	ok
	24	C40H82NNaO8P	758.5670	-2.1	15.7	24	35.72	1.0	even	ok
	25	C33H72N15O3S	758.5658	-3.8	16.1	25	12.84	6.0	even	ok
	26	C33H86N5O7P2S	758.5718	4.1	16.4	26	10.09	-5.0	even	ok
	27	C34H85N3O10PS	758.5688	0.2	16.7	27	100.00	-5.0	even	ok
	28	C32H78N11O5P2	758.5657	-3.9	17.1	28	11.84	1.0	even	ok
	29	C37H74N11NaO2P	758.5657	-3.9	17.2	29	11.41	7.0	even	ok
	30	C35H81N7O6PS	758.5701	1.9	17.4	30	38.53	0.0	even	ok
	31	C35H69N17P	758.5654	-4.3	17.7	31	8.54	11.0	even	ok
	32	C37H86NNaO8PS	758.5704	2.3	17.8	32	39.44	-4.0	even	ok
	33	C35H77N9NaO5S	758.5661	-3.4	18.6	33	15.75	2.0	even	ok
	34	C29H65N23Na	758.5685	-0.1	18.6	34	77.58	9.0	even	ok
	35	C35H84NO13S	758.5658	-3.8	19.5	35	15.11	-5.0	even	ok
	36	C29H78N13NaO4PS	758.5650	-4.8	19.8	36	5.50	-2.0	even	ok
	37	C36H80N5O9S	758.5671	-2.0	19.8	37	43.90	0.0	even	ok
	38	C32H80N5O14	758.5696	1.3	20.7	38	48.47	-4.0	even	ok
	39	C28H79N15NaOP2S	758.5680	-0.8	21.3	39	57.34	-2.0	even	ok
	40	C31H77N9NaO10	758.5686	-0.1	21.6	40	73.11	-2.0	even	ok
	41	C26H69N23NaS	758.5719	4.3	22.1	41	7.68	4.0	even	ok
	42	C30H81N9O8PS	758.5661	-3.4	22.2	42	15.05	-4.0	even	ok
	43	C32H86N3NaO10PS	758.5664	-3.0	22.9	43	18.79	-8.0	even	ok
	44	C29H82N11O5P2S	758.5691	0.6	22.9	44	61.18	-4.0	even	ok
	45	C31H87N5NaO7P2S	758.5694	0.9	23.1	45	53.07	-8.0	even	ok
	46	C36H77N11O2PS	758.5715	3.7	23.3	46	11.80	5.0	even	ok
	47	C29H72N15O8	758.5683	-0.5	23.4	47	62.24	2.0	even	ok
	48	C41H82N3O5P2	758.5724	5.0	24.2	48	4.26	4.0	even	ok
	49	C37H76N9O5S	758.5685	-0.2	25.2	49	80.93	5.0	even	ok

Compound Spectrum SmartFormula Report

Meas. m/z	#	Ion Formula	m/z	err [ppm]	mSigma	# mSigma	Score	rdb	e⁻ Conf	N-Rule
	50	C28H69N19NaO4	758.5672	-1.9	25.2	50	33.00	4.0	even	ok
	51	C40H79N7NaOP2	758.5714	3.6	25.3	51	12.35	6.0	even	ok
	52	C38H82N5NaO4PS	758.5717	4.1	25.6	52	8.58	1.0	even	ok
	53	C32H90NO11P2S	758.5704	2.3	25.6	53	25.95	-10.0	even	ok
	54	C36H73N13NaOS	758.5674	-1.7	25.8	54	36.63	7.0	even	ok
	55	C30H78N11NaO7P	758.5716	3.8	25.8	55	10.18	-2.0	even	ok
	56	C39H73N11O2P	758.5681	-0.8	26.9	56	52.22	10.0	even	ok
	57	C42H81NO8P	758.5694	1.0	27.1	57	46.81	4.0	even	ok
	58	C39H81N3NaO7S	758.5687	0.1	27.3	58	80.45	1.0	even	ok
	59	C26H64N25O2	758.5669	-2.3	27.3	59	26.01	8.0	even	ok
	60	C41H78N5NaO4P	758.5684	-0.4	27.5	60	58.79	6.0	even	ok
	61	C28H73N17O5P	758.5713	3.5	27.7	61	12.59	2.0	even	ok
	62	C40H72N9O5	758.5651	-4.7	29.4	62	4.76	10.0	even	ok
	63	C27H70N21NaOP	758.5702	2.0	29.8	63	27.57	4.0	even	ok
	64	C31H82N11NaO2PS2	758.5724	5.0	29.9	64	4.68	-3.0	even	ok
	65	C25H73N19NaO4S	758.5706	2.5	30.0	65	20.94	-1.0	even	ok
	66	C42H77N3NaO7	758.5654	-4.3	30.3	66	6.27	6.0	even	ok
	67	C30H76N15O3S2	758.5691	0.7	31.2	67	61.06	1.0	even	ok
	68	C33H82N11P2S2	758.5666	-2.7	31.2	68	22.83	0.0	even	ok
	69	C28H81N9NaO10S	758.5719	4.3	31.4	69	6.12	-7.0	even	ok
	70	C26H76N15O8S	758.5717	4.0	31.4	70	8.09	-3.0	even	ok
	71	C32H86N7O4P2S2	758.5652	-4.5	31.6	71	6.65	-5.0	even	ok
	72	C31H84NO18	758.5683	-0.5	31.9	72	51.12	-9.0	even	ok
	73	C28H81N13O4PS2	758.5708	2.8	32.0	73	21.04	-4.0	even	ok
	74	C30H81N5NaO14	758.5672	-1.9	32.2	74	28.14	-7.0	even	ok
	75	C29H77N17PS2	758.5721	4.6	32.6	75	5.96	1.0	even	ok
	76	C23H68N25O2S	758.5703	2.2	32.7	76	23.95	3.0	even	ok
	77	C27H78N17NaPS2	758.5697	1.4	32.7	77	43.04	-2.0	even	ok
	78	C32H81N9NaO5S2	758.5694	1.0	33.3	78	50.34	-3.0	even	ok
	79	C26H72N21OS2	758.5665	-2.9	33.3	79	19.73	2.0	even	ok
	80	C35H87N5NaO2P2S2	758.5669	-2.4	33.4	80	26.62	-4.0	even	ok
	81	C30H86N7NaO6PS2	758.5711	3.2	33.5	81	16.08	-8.0	even	ok
	82	C28H76N11O12	758.5669	-2.3	33.5	82	22.49	-3.0	even	ok
	83	C34H91NNaO6P2S2	758.5655	-4.1	33.6	83	8.43	-9.0	even	ok
	84	C33H77N13NaOS2	758.5708	2.8	34.0	84	20.56	2.0	even	ok
	85	C29H80N11O7S2	758.5678	-1.1	34.2	85	47.48	-4.0	even	ok
	86	C27H73N15NaO8	758.5659	-3.7	34.5	86	9.20	-1.0	even	ok
	87	C40H82N5O2P2S	758.5659	-3.6	34.6	87	9.37	4.0	even	ok
	88	C28H77N15NaO3S2	758.5667	-2.5	34.9	88	23.50	-2.0	even	ok
	89	C40H77N7NaO3S	758.5701	1.9	35.1	89	32.71	6.0	even	ok
	90	C30H85N3O15P	758.5713	3.5	35.2	90	10.36	-9.0	even	ok
	91	C29H82N7NaO11P	758.5702	2.1	35.9	91	23.50	-7.0	even	ok
	92	C31H85N5NaO9S2	758.5681	-0.7	35.9	92	52.50	-8.0	even	ok
	93	C25H68N21O6	758.5656	-4.0	36.2	93	6.75	3.0	even	ok
	94	C27H81N11NaO7S2	758.5654	-4.3	36.8	94	6.89	-7.0	even	ok
	95	C33H84N5O9S2	758.5705	2.4	37.0	95	23.36	-5.0	even	ok

Compound Spectrum SmartFormula Report

Meas. m/z	#	Ion Formula	m/z	err [ppm]	mSigma	# mSigma	Score	rdb	e¯ Conf	N-Rule
	96	C36H90NO6P2S2	758.5679	-1.0	37.0	96	47.03	-6.0	even	ok
	97	C41H80N3O7S	758.5711	3.3	37.0	97	13.88	4.0	even	ok
	98	C31H89N3O10PS2	758.5722	4.6	37.1	98	5.25	-10.0	even	ok
	99	C38H72N13OS	758.5698	1.5	37.2	99	29.49	10.0	even	ok
	100	C27H77N13O9P	758.5699	1.7	37.4	100	27.07	-3.0	even	ok
	101	C34H80N9O5S2	758.5718	4.2	37.5	101	7.21	0.0	even	ok
	102	C26H74N17NaO5P	758.5689	0.3	38.7	102	45.90	-1.0	even	ok
	103	C43H77N5O4P	758.5708	2.8	38.8	103	14.49	9.0	even	ok
	104	C42H74N9NaP	758.5697	1.4	39.3	104	29.60	11.0	even	ok
	105	C32H88NO13S2	758.5692	0.7	39.5	105	48.95	-10.0	even	ok
	106	C36H85N3NaO7S2	758.5721	4.6	39.8	106	5.08	-4.0	even	ok
	107	C37H86N5O2P2S2	758.5693	0.8	39.8	107	46.08	-1.0	even	ok
	108	C28H84N7O11S2	758.5665	-2.9	40.0	108	16.69	-9.0	even	ok
	109	C24H69N23O3P	758.5686	-0.1	40.4	109	46.49	3.0	even	ok
	110	C37H82N7NaOPS2	758.5652	-4.5	41.1	110	4.98	1.0	even	ok
	111	C41H68N13O	758.5664	-2.9	41.2	111	12.45	15.0	even	ok
	112	C30H89NNaO13S2	758.5668	-2.5	41.3	112	19.97	-13.0	even	ok
	113	C43H83N3NaP2S	758.5675	-1.5	41.7	113	32.79	5.0	even	ok
	114	C44H76N3O7	758.5678	-1.2	42.0	114	30.23	9.0	even	ok
	115	C43H73N7NaO3	758.5667	-2.6	42.1	115	15.11	11.0	even	ok
	116	C25H75N19NaO2P2	758.5719	4.2	42.9	116	4.88	-1.0	even	ok
	117	C29H85NNaO18	758.5659	-3.6	43.3	117	7.34	-12.0	even	ok
	118	C38H85N3O5PS2	758.5663	-3.1	44.3	118	12.60	-1.0	even	ok
	119	C27H80N7O16	758.5656	-4.0	44.4	119	5.45	-8.0	even	ok
	120	C42H76N7O3S	758.5725	5.1	45.6	120	2.85	9.0	even	ok
	121	C40H87N3NaP2S2	758.5709	2.9	47.3	121	12.97	0.0	even	ok
	122	C39H81N7OPS2	758.5676	-1.4	49.7	122	27.59	4.0	even	ok
	123	C44H73N9P	758.5721	4.6	50.6	123	2.99	14.0	even	ok
	124	C46H78N3NaO2P	758.5724	4.9	51.6	124	2.14	10.0	even	ok
	125	C41H86NNaO3PS2	758.5679	-1.0	51.7	125	30.30	0.0	even	ok
	126	C45H82N3P2S	758.5699	1.7	52.5	126	22.03	8.0	even	ok
	127	C45H72N7O3	758.5691	0.6	53.8	127	26.29	14.0	even	ok
	128	C47H77NNaO5	758.5694	1.0	54.9	128	22.04	10.0	even	ok
	129	C46H81NO3PS	758.5669	-2.3	56.6	129	14.28	8.0	even	ok
	130	C43H85NO3PS2	758.5703	2.2	61.1	130	12.98	3.0	even	ok
	131	C41H76N9S2	758.5660	-3.5	61.3	131	5.60	9.0	even	ok
	132	C48H78N3P2	758.5665	-2.8	62.6	132	7.04	13.0	even	ok
	133	C43H81N3NaO2S2	758.5662	-3.2	63.2	133	6.70	5.0	even	ok
	134	C48H73N5NaO	758.5707	2.7	66.8	134	6.19	15.0	even	ok
	135	C49H76NO5	758.5718	4.1	67.0	135	2.38	13.0	even	ok
	136	C48H76N3O2S	758.5653	-4.5	70.3	136	2.08	13.0	even	ok
	137	C45H80N3O2S2	758.5686	-0.0	73.4	137	20.23	8.0	even	ok
	138	C51H77NNaS	758.5669	-2.3	81.8	138	5.49	14.0	even	ok
	139	C48H81NNaS2	758.5703	2.1	83.9	139	5.60	9.0	even	ok
	140	C53H76NS	758.5693	0.9	93.1	140	6.66	17.0	even	ok

Compound Spectrum SmartFormula Report

Cmpd 12, 28.4 min

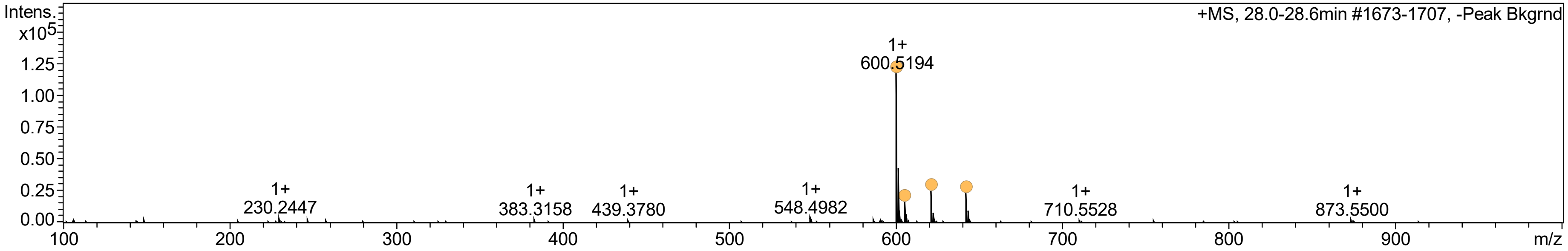

Meas. m/z	#	Ion Formula	m/z	err [ppm]	mSigma	# mSigma	Score	rdb	e⁻ Conf	N-Rule
600.5194	1	C33H71NNaO6	600.5174	-3.5	3.7	1	37.04	-1.0	even	ok
	2	C31H66N7O4	600.5171	-3.9	5.4	2	28.72	3.0	even	ok
	3	C32H72N3NaO3P	600.5204	1.5	6.9	3	75.62	-1.0	even	ok
	4	C30H67N9OP	600.5201	1.1	9.2	4	84.12	3.0	even	ok
	5	C35H70NO6	600.5198	0.6	13.2	5	90.26	2.0	even	ok
	6	C27H67N11NaS	600.5194	-0.1	14.2	6	100.00	0.0	even	ok
	7	C34H67N5NaO2	600.5187	-1.2	14.3	7	72.56	4.0	even	ok
	8	C32H62N11	600.5184	-1.7	14.4	8	62.11	8.0	even	ok
	9	C31H76N3OP2S	600.5179	-2.6	14.5	9	44.49	-3.0	even	ok
	10	C29H66N11S	600.5218	3.9	14.6	10	24.18	3.0	even	ok
	11	C29H71N5O5P	600.5187	-1.2	15.2	11	72.63	-2.0	even	ok
	12	C31H71N5NaO2S	600.5221	4.4	16.9	12	18.10	-1.0	even	ok
	13	C28H68N9NaOP	600.5177	-2.9	17.1	13	36.20	0.0	even	ok
	14	C30H77NNaO4P2	600.5220	4.3	17.6	14	18.91	-6.0	even	ok
	15	C28H70N7O4S	600.5205	1.7	17.9	15	57.81	-2.0	even	ok
	16	C28H72N7O2P2	600.5217	3.8	19.4	16	23.07	-2.0	even	ok
	17	C30H75NNaO6S	600.5207	2.2	19.5	17	47.21	-6.0	even	ok
	18	C24H66N13O2S	600.5178	-2.8	23.3	18	34.06	-1.0	even	ok
	19	C26H71N7NaO4S	600.5180	-2.3	23.8	19	40.65	-5.0	even	ok
	20	C25H72N9NaOPS	600.5210	2.7	24.4	20	34.70	-5.0	even	ok
	21	C36H66N5O2	600.5211	2.8	24.9	21	32.80	7.0	even	ok
	22	C28H75NO9P	600.5174	-3.4	25.5	22	24.72	-7.0	even	ok
	23	C27H74N3O8S	600.5191	-0.5	26.6	23	68.19	-7.0	even	ok
	24	C26H75N5O5PS	600.5221	4.4	26.7	24	14.15	-7.0	even	ok
	25	C27H76N3O6P2	600.5204	1.6	29.1	25	46.66	-7.0	even	ok
	26	C28H76N5NaPS2	600.5172	-3.7	31.8	26	22.73	-6.0	even	ok
	27	C28H80N3OP2S2	600.5213	3.0	32.6	27	30.53	-8.0	even	ok
	28	C30H75N5PS2	600.5196	0.3	34.0	28	76.01	-3.0	even	ok
	29	C26H73N7NaO2P2	600.5193	-0.2	34.2	29	46.43	-5.0	even	ok
	30	C25H75N3NaO8S	600.5167	-4.5	34.8	30	11.06	-10.0	even	ok
	31	C29H79NO4PS2	600.5183	-1.9	35.4	31	43.93	-8.0	even	ok
	32	C24H68N13P2	600.5190	-0.7	36.1	32	39.12	-1.0	even	ok

Compound Spectrum SmartFormula Report

Meas. m/z	#	Ion Formula	m/z	err [ppm]	mSigma	# mSigma	Score	rdb	e¯ Conf	N-Rule
	33	C23H67N11NaO5	600.5219	4.1	37.5	33	13.11	-4.0	even	ok
	34	C31H74N3O3S2	600.5166	-4.7	37.8	34	11.68	-3.0	even	ok
	35	C34H75NNaOS2	600.5182	-2.0	42.4	35	35.63	-2.0	even	ok
	36	C21H62N17O3	600.5216	3.6	44.5	36	10.20	0.0	even	ok
	37	C39H70NOS	600.5173	-3.6	48.5	37	12.13	6.0	even	ok
	38	C36H74NOS2	600.5206	2.0	50.0	38	28.56	1.0	even	ok
605.4752	1	C41H65OS	605.4751	-0.3	13.3	1	100.00	10.0	even	ok
	2	C39H66NaOS	605.4727	-4.2	14.7	2	20.73	7.0	even	ok
	3	C35H66N4PS	605.4740	-2.0	26.7	3	44.17	6.0	even	ok
	4	C37H65O6	605.4776	3.9	31.7	4	17.19	6.0	even	ok
	5	C36H70NaOS2	605.4760	1.3	32.9	5	59.38	2.0	even	ok
	6	C36H62N4NaO2	605.4765	2.1	33.5	6	35.67	8.0	even	ok
	7	C34H57N10	605.4762	1.6	35.5	7	40.03	12.0	even	ok
	8	C36H67N2OP2	605.4723	-4.8	37.8	8	8.86	6.0	even	ok
	9	C34H70O4PS	605.4727	-4.2	38.2	9	12.38	1.0	even	ok
	10	C33H69N2O3S2	605.4744	-1.3	39.4	10	49.83	1.0	even	ok
	11	C32H70N4PS2	605.4774	3.6	39.5	11	20.05	1.0	even	ok
	12	C33H71N2OP2S	605.4757	0.8	40.8	12	45.87	1.0	even	ok
	13	C35H66NaO6	605.4752	-0.1	42.9	13	51.57	3.0	even	ok
	14	C33H61N6O4	605.4749	-0.6	44.6	14	43.53	7.0	even	ok
	15	C32H58N10Na	605.4738	-2.3	46.2	15	23.10	9.0	even	ok
	16	C34H67N2NaO3P	605.4782	4.8	47.0	16	6.74	3.0	even	ok
	17	C30H71N4NaPS2	605.4750	-0.4	47.0	17	53.39	-2.0	even	ok
	18	C31H74O4PS2	605.4761	1.4	48.7	18	37.59	-4.0	even	ok
	19	C32H62N8OP	605.4779	4.4	48.8	19	8.28	7.0	even	ok
	20	C29H62N10NaS	605.4772	3.2	49.0	20	14.49	4.0	even	ok
	21	C30H65N6O4S	605.4783	5.0	49.3	21	5.70	2.0	even	ok
	22	C31H72N2NaOP2S	605.4733	-3.2	52.0	22	13.36	-2.0	even	ok
	23	C32H65N2O8	605.4735	-2.8	54.6	23	14.89	2.0	even	ok
	24	C31H62N6NaO4	605.4725	-4.5	55.7	24	6.07	4.0	even	ok
	25	C29H75NaO4PS2	605.4737	-2.6	56.9	25	18.74	-7.0	even	ok
	26	C27H70N6O2PS2	605.4734	-3.0	56.9	26	15.35	-3.0	even	ok
	27	C29H57N12O2	605.4722	-5.0	57.3	27	4.41	8.0	even	ok
	28	C28H76N2NaOP2S2	605.4767	2.4	58.2	28	19.65	-7.0	even	ok
	29	C31H66N4O5P	605.4765	2.2	58.5	29	16.80	2.0	even	ok
	30	C30H63N8NaOP	605.4755	0.4	59.7	30	28.40	4.0	even	ok
	31	C28H66N6NaO4S	605.4758	1.0	60.5	31	23.18	-1.0	even	ok
	32	C29H69N2O8S	605.4769	2.8	60.9	32	12.01	-3.0	even	ok
	33	C26H61N12O2S	605.4756	0.6	61.4	33	25.69	3.0	even	ok
	34	C25H75N4O3P2S2	605.4750	-0.3	68.7	34	26.46	-8.0	even	ok
	35	C30H70O9P	605.4752	-0.0	68.7	35	22.62	-3.0	even	ok
	36	C29H67N4NaO5P	605.4741	-1.8	69.6	36	12.92	-1.0	even	ok
	37	C27H62N10O3P	605.4738	-2.3	71.2	37	10.27	3.0	even	ok
	38	C27H70N2NaO8S	605.4745	-1.2	72.0	38	14.60	-6.0	even	ok
	39	C29H71N2O6P2	605.4782	4.9	72.5	39	2.76	-3.0	even	ok
	40	C24H62N12NaO2S	605.4732	-3.4	72.6	40	5.99	0.0	even	ok

Compound Spectrum SmartFormula Report

Meas. m/z	#	Ion Formula	m/z	err [ppm]	mSigma	# mSigma	Score	rdb	e⁻ Conf	N-Rule
	41	C25H65N8O6S	605.4742	-1.6	72.8	41	12.15	-2.0	even	ok
	42	C28H68N6NaO2P2	605.4771	3.1	73.5	42	6.58	-1.0	even	ok
	43	C22H57N18S	605.4729	-3.9	73.6	43	4.60	4.0	even	ok
	44	C26H71N4NaO5PS	605.4775	3.8	74.8	44	4.65	-6.0	even	ok
	45	C24H66N10O3PS	605.4772	3.3	75.7	45	5.59	-2.0	even	ok
	46	C28H71NaO9P	605.4728	-4.0	79.9	46	3.32	-6.0	even	ok
	47	C26H66N6O7P	605.4725	-4.5	81.3	47	2.45	-2.0	even	ok
	48	C27H72N2NaO6P2	605.4758	0.9	83.6	48	9.98	-6.0	even	ok
	49	C24H69N4O10S	605.4729	-3.9	84.3	49	3.02	-7.0	even	ok
	50	C26H63N12P2	605.4768	2.7	84.5	50	3.88	3.0	even	ok
	51	C25H67N8O4P2	605.4755	0.5	85.1	51	10.70	-2.0	even	ok
	52	C25H75NaO9PS	605.4762	1.5	86.3	52	7.30	-11.0	even	ok
	53	C22H61N12O7	605.4781	4.7	92.6	53	1.34	-1.0	even	ok
	54	C24H71N4O8P2	605.4742	-1.8	95.3	54	4.56	-7.0	even	ok
	55	C24H64N12NaP2	605.4744	-1.3	97.3	55	3.63	0.0	even	ok
621.4486	1	C24H62N12NaOS2	621.4503	2.7	8.0	1	45.94	0.0	even	ok
	2	C24H67N10P2S2	621.4461	-4.0	8.1	2	24.92	-2.0	even	ok
	3	C26H72N4NaO2P2S2	621.4464	-3.5	9.2	3	30.86	-6.0	even	ok
	4	C27H65N4O9S	621.4467	-3.1	9.4	4	30.16	-2.0	even	ok
	5	C25H65N8O5S2	621.4514	4.5	11.3	5	18.16	-2.0	even	ok
	6	C30H66N2NaO7S	621.4483	-0.5	11.4	6	76.25	-1.0	even	ok
	7	C28H71NaO8PS	621.4499	2.1	11.6	7	44.03	-6.0	even	ok
	8	C28H71N4O2P2S2	621.4488	0.3	11.6	8	100.00	-3.0	even	ok
	9	C27H75O6P2S2	621.4475	-1.8	12.4	9	61.21	-8.0	even	ok
	10	C28H61N8O5S	621.4480	-1.0	12.4	10	65.45	3.0	even	ok
	11	C29H67N4NaO4PS	621.4513	4.3	12.7	11	15.52	-1.0	even	ok
	12	C26H62N8NaO5S	621.4456	-4.8	12.8	12	11.29	0.0	even	ok
	13	C27H70N2NaO7S2	621.4517	4.9	13.1	13	13.45	-6.0	even	ok
	14	C26H66N6O6PS	621.4497	1.7	13.8	14	49.97	-2.0	even	ok
	15	C27H62N10O2PS	621.4510	3.8	14.4	15	19.15	3.0	even	ok
	16	C21H61N14O3S2	621.4487	0.1	14.7	16	98.91	-1.0	even	ok
	17	C23H66N8NaO5S2	621.4490	0.6	15.1	17	86.87	-5.0	even	ok
	18	C29H70N2O5PS2	621.4458	-4.5	15.6	18	16.45	-3.0	even	ok
	19	C27H58N12NaOS	621.4469	-2.7	16.0	19	32.27	5.0	even	ok
	20	C25H63N10NaO2PS	621.4486	-0.0	17.1	20	77.59	0.0	even	ok
	21	C24H69N4O9S2	621.4500	2.3	17.7	21	45.63	-7.0	even	ok
	22	C23H58N16PS	621.4483	-0.5	19.4	22	65.66	4.0	even	ok
	23	C31H67N4O2P2S	621.4454	-5.1	19.5	23	8.41	2.0	even	ok
	24	C31H72N2NaP2S2	621.4504	2.9	20.0	24	33.27	-2.0	even	ok
	25	C32H65N2O7S	621.4507	3.4	20.2	25	21.76	2.0	even	ok
	26	C25H70N2O10PS	621.4483	-0.5	21.1	26	63.68	-7.0	even	ok
	27	C31H62N6NaO3S	621.4496	1.6	21.9	27	43.10	4.0	even	ok
	28	C29H57N12OS	621.4494	1.2	22.2	28	49.92	8.0	even	ok
	29	C30H66N6OPS2	621.4472	-2.3	22.2	29	41.01	2.0	even	ok
	30	C24H67N6NaO6PS	621.4473	-2.2	22.8	30	34.49	-5.0	even	ok
	31	C32H71NaO3PS2	621.4474	-1.9	24.0	31	47.05	-2.0	even	ok

Compound Spectrum SmartFormula Report

Meas. m/z	#	Ion Formula	m/z	err [ppm]	mSigma	# mSigma	Score	rdb	e⁻ Conf	N-Rule
	32	C22H62N12O4PS	621.4470	-2.6	24.7	32	27.42	-1.0	even	ok
	33	C24H71N4O7P2S	621.4513	4.4	25.2	33	11.54	-7.0	even	ok
	34	C23H68N8NaO3P2S	621.4503	2.6	27.1	34	25.87	-5.0	even	ok
	35	C21H59N16NaPS	621.4459	-4.3	27.2	35	11.07	1.0	even	ok
	36	C21H63N14OP2S	621.4500	2.2	29.1	36	29.70	-1.0	even	ok
	37	C34H68N2NaP2S	621.4471	-2.5	30.3	37	25.46	3.0	even	ok
	38	C31H67NaO8P	621.4466	-3.3	30.4	38	17.84	-1.0	even	ok
	39	C33H66O8P	621.4490	0.6	31.4	39	48.31	2.0	even	ok
	40	C29H62N6O6P	621.4463	-3.7	32.5	40	13.54	3.0	even	ok
	41	C34H70O3PS2	621.4499	2.0	33.5	41	35.95	1.0	even	ok
	42	C30H68N2NaO5P2	621.4496	1.5	33.9	42	33.66	-1.0	even	ok
	43	C32H61N8S2	621.4455	-5.0	34.8	43	7.84	7.0	even	ok
	44	C32H63N4NaO4P	621.4479	-1.1	35.2	44	37.23	4.0	even	ok
	45	C26H66N2NaO12	621.4508	3.5	36.0	45	13.85	-5.0	even	ok
	46	C34H66N2NaO2S2	621.4458	-4.5	36.0	46	9.93	3.0	even	ok
	47	C27H67N4O7P2	621.4480	-1.1	36.1	47	37.10	-2.0	even	ok
	48	C28H63N8O3P2	621.4493	1.1	36.1	48	36.94	3.0	even	ok
	49	C30H58N10O2P	621.4476	-1.6	37.0	49	30.58	8.0	even	ok
	50	C31H64N6NaOP2	621.4509	3.7	37.7	50	12.14	4.0	even	ok
	51	C24H61N8O10	621.4505	3.1	38.3	51	16.18	-1.0	even	ok
	52	C35H61N2O7	621.4473	-2.1	38.5	52	24.45	7.0	even	ok
	53	C34H62N4O4P	621.4503	2.7	39.3	53	18.16	7.0	even	ok
	54	C24H54N16NaO2	621.4508	3.5	39.5	54	12.72	6.0	even	ok
	55	C26H71O11P2	621.4466	-3.2	39.5	55	14.50	-7.0	even	ok
	56	C26H64N8NaO3P2	621.4469	-2.8	39.8	56	17.53	0.0	even	ok
	57	C36H67N2P2S	621.4495	1.4	40.4	57	29.86	6.0	even	ok
	58	C23H58N12NaO6	621.4494	1.3	41.5	58	29.32	1.0	even	ok
	59	C34H58N6NaO3	621.4463	-3.8	41.7	59	10.31	9.0	even	ok
	60	C25H68N4NaO7P2	621.4455	-4.9	42.6	60	5.24	-5.0	even	ok
	61	C33H59N8NaP	621.4493	1.0	42.7	61	31.47	9.0	even	ok
	62	C37H66O3PS	621.4465	-3.4	42.7	62	11.97	6.0	even	ok
	63	C32H53N12O	621.4460	-4.2	42.8	63	7.86	13.0	even	ok
	64	C23H65N4O14	621.4492	0.9	43.8	64	31.61	-6.0	even	ok
	65	C21H53N18O4	621.4492	0.9	43.8	65	31.75	5.0	even	ok
	66	C22H49N22	621.4505	3.0	44.7	66	10.23	10.0	even	ok
	67	C24H59N14OP2	621.4466	-3.2	45.2	67	9.20	4.0	even	ok
	68	C22H62N8NaO10	621.4481	-0.8	46.4	68	30.22	-4.0	even	ok
	69	C36H65N2O2S2	621.4482	-0.7	46.4	69	39.32	6.0	even	ok
	70	C38H62NaO5	621.4489	0.5	48.1	70	31.16	8.0	even	ok
	71	C20H57N14O8	621.4478	-1.3	48.5	71	24.63	0.0	even	ok
	72	C36H57N6O3	621.4487	0.1	48.6	72	34.60	12.0	even	ok
	73	C35H58N8P	621.4517	4.9	48.6	73	4.52	12.0	even	ok
	74	C21H63N10NaO7P	621.4511	4.0	50.8	74	7.07	-4.0	even	ok
	75	C20H50N22Na	621.4481	-0.8	51.2	75	19.49	7.0	even	ok
	76	C19H54N18NaO4	621.4468	-3.0	56.4	76	7.35	2.0	even	ok
	77	C39H63N2P2	621.4461	-4.0	56.4	77	5.76	11.0	even	ok

Compound Spectrum SmartFormula Report

Meas. m/z	#	Ion Formula	m/z	err [ppm]	mSigma	# mSigma	Score	rdb	e¯ Conf	N-Rule
	78	C40H61O5	621.4514	4.4	56.8	78	4.67	11.0	even	ok
	79	C39H66NaS2	621.4498	1.9	57.4	79	18.33	7.0	even	ok
	80	C17H49N24O2	621.4465	-3.4	58.8	80	5.48	6.0	even	ok
	81	C39H58N4NaO	621.4503	2.7	58.9	81	10.30	13.0	even	ok
	82	C42H62NaS	621.4464	-3.5	69.8	82	4.82	12.0	even	ok
	83	C44H61S	621.4488	0.4	81.4	83	10.03	15.0	even	ok
	84	C47H57	621.4455	-5.0	91.9	84	0.82	20.0	even	ok
642.5659	1	C36H77N5PS	642.5632	-4.3	4.6	1	25.30	2.0	even	ok
	2	C34H77N5NaO2S	642.5690	4.8	11.8	2	15.73	-1.0	even	ok
	3	C32H72N11S	642.5687	4.4	12.5	3	20.32	3.0	even	ok
	4	C38H76NO6	642.5667	1.2	15.1	4	83.94	2.0	even	ok
	5	C34H82N3OP2S	642.5648	-1.7	15.4	5	71.12	-3.0	even	ok
	6	C39H72N5O2	642.5681	3.3	18.5	6	32.46	7.0	even	ok
	7	C37H73N5NaO2	642.5656	-0.4	19.1	7	100.00	4.0	even	ok
	8	C35H68N11	642.5654	-0.9	21.4	8	83.43	8.0	even	ok
	9	C36H77NNaO6	642.5643	-2.5	22.4	9	43.95	-1.0	even	ok
	10	C33H81N5PS2	642.5666	1.0	22.6	10	97.51	-3.0	even	ok
	11	C33H81NNaO6S	642.5677	2.7	23.2	11	38.79	-6.0	even	ok
	12	C30H73N11NaS	642.5663	0.6	23.7	12	84.83	0.0	even	ok
	13	C31H76N7O4S	642.5674	2.3	23.8	13	46.35	-2.0	even	ok
	14	C34H72N7O4	642.5640	-2.9	24.7	14	34.19	3.0	even	ok
	15	C34H80N3O3S2	642.5636	-3.7	25.6	15	28.95	-3.0	even	ok
	16	C31H82N5NaPS2	642.5641	-2.8	26.2	16	45.02	-6.0	even	ok
	17	C37H81NNaOS2	642.5652	-1.2	26.8	17	84.04	-2.0	even	ok
	18	C35H78N3NaO3P	642.5673	2.2	27.1	18	45.89	-1.0	even	ok
	19	C33H69N11Na	642.5630	-4.6	27.9	19	12.81	5.0	even	ok
	20	C31H86N3OP2S2	642.5682	3.6	28.4	20	28.74	-8.0	even	ok
	21	C42H76NOS	642.5642	-2.7	29.2	21	35.11	6.0	even	ok
	22	C32H85NO4PS2	642.5652	-1.1	29.3	22	80.59	-8.0	even	ok
	23	C33H73N9OP	642.5670	1.7	29.5	23	51.58	3.0	even	ok
	24	C33H76N3O8	642.5627	-5.0	32.0	24	8.88	-2.0	even	ok
	25	C39H80NOS2	642.5676	2.6	33.2	25	40.91	1.0	even	ok
	26	C30H86NNaO4PS2	642.5628	-4.8	35.0	26	11.58	-11.0	even	ok
	27	C29H77N7NaO4S	642.5650	-1.4	35.0	27	49.75	-5.0	even	ok
	28	C30H80N3O8S	642.5661	0.2	35.4	28	71.98	-7.0	even	ok
	29	C29H87N3NaOP2S2	642.5658	-0.2	35.5	29	90.80	-11.0	even	ok
	30	C27H72N13O2S	642.5647	-1.9	36.0	30	41.17	-1.0	even	ok
	31	C32H77N5O5P	642.5657	-0.4	36.5	31	67.23	-2.0	even	ok
	32	C28H78N9NaOPS	642.5680	3.2	38.2	32	21.41	-5.0	even	ok
	33	C29H81N5O5PS	642.5691	4.9	38.2	33	8.30	-7.0	even	ok
	34	C31H74N9NaOP	642.5646	-2.0	38.9	34	35.86	0.0	even	ok
	35	C33H83NNaO4P2	642.5690	4.7	39.0	35	8.97	-6.0	even	ok
	36	C31H78N7O2P2	642.5687	4.3	41.1	36	11.05	-2.0	even	ok
	37	C31H81NO9P	642.5643	-2.4	45.4	37	25.03	-7.0	even	ok
	38	C28H81N3NaO8S	642.5637	-3.5	46.5	38	14.54	-10.0	even	ok
	39	C30H78N5NaO5P	642.5633	-4.1	47.1	39	10.31	-5.0	even	ok

Compound Spectrum SmartFormula Report

Meas. m/z	#	Ion Formula	m/z	err [ppm]	mSigma	# mSigma	Score	rdb	e⁻ Conf	N-Rule
	40	C26H76N9O6S	642.5634	-4.0	47.4	40	11.18	-6.0	even	ok
	41	C28H73N11O3P	642.5630	-4.5	49.0	41	7.53	-1.0	even	ok
	42	C30H82N3O6P2	642.5673	2.2	49.7	42	24.48	-7.0	even	ok
	43	C29H79N7NaO2P2	642.5663	0.5	51.4	43	41.71	-5.0	even	ok
	44	C26H73N11NaO5	642.5688	4.5	58.0	44	5.68	-4.0	even	ok
	45	C24H68N17O3	642.5686	4.1	59.8	45	6.93	0.0	even	ok
	46	C28H83N3NaO6P2	642.5649	-1.5	60.5	46	22.44	-10.0	even	ok
	47	C27H74N13P2	642.5660	0.1	61.0	47	25.86	-1.0	even	ok
	48	C26H78N9O4P2	642.5647	-2.0	62.2	48	17.91	-6.0	even	ok
	49	C25H75N13NaP2	642.5636	-3.6	73.1	49	4.17	-4.0	even	ok

Cmpd 13, 30.5 min

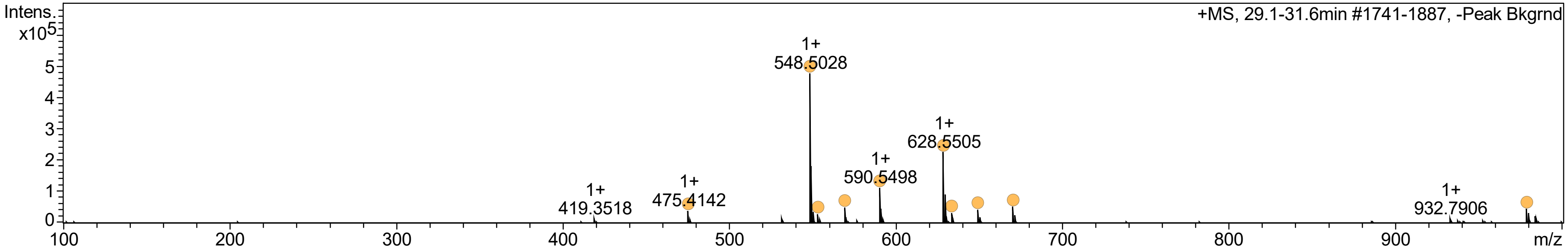

Meas. m/z	#	Ion Formula	m/z	err [ppm]	mSigma	# mSigma	Score	rdb	e⁻ Conf	N-Rule
475.4142	1	C29H56NaO3	475.4122	-4.3	5.7	1	37.57	2.0	even	ok
	2	C27H51N6O	475.4119	-4.9	7.4	2	21.87	6.0	even	ok
	3	C31H55O3	475.4146	0.8	8.2	3	100.00	5.0	even	ok
	4	C28H57N2NaP	475.4152	2.0	10.2	4	53.74	2.0	even	ok
	5	C25H56N4O2P	475.4135	-1.4	23.0	5	48.11	1.0	even	ok
	6	C24H55N6OS	475.4153	2.2	23.1	6	51.99	1.0	even	ok
	7	C26H60NaO3S	475.4155	2.8	24.3	7	42.66	-3.0	even	ok
	8	C22H56N6NaOS	475.4129	-2.9	30.1	8	36.62	-2.0	even	ok
	9	C24H60O6P	475.4122	-4.2	30.9	9	22.80	-4.0	even	ok
	10	C23H59N2O5S	475.4139	-0.6	32.8	10	60.99	-4.0	even	ok
	11	C25H64OPS2	475.4131	-2.4	38.2	11	42.66	-5.0	even	ok
	12	C23H61N2O3P2	475.4152	2.1	39.2	12	27.16	-4.0	even	ok
	13	C17H47N16	475.4164	4.6	50.2	13	8.61	3.0	even	ok
	14	C21H62N2NaO3P2	475.4128	-3.0	52.0	14	14.38	-7.0	even	ok
	15	C19H57N8OP2	475.4125	-3.6	53.4	15	11.41	-3.0	even	ok
548.5028	1	C33H67NNaO3	548.5013	-2.8	4.7	1	64.48	1.0	even	ok
	2	C31H62N7O	548.5010	-3.3	6.5	2	51.47	5.0	even	ok
	3	C35H66NO3	548.5037	1.6	6.8	3	90.99	4.0	even	ok
	4	C32H68N3NaP	548.5043	2.7	9.0	4	61.45	1.0	even	ok

Compound Spectrum SmartFormula Report

Meas. m/z	#	Ion Formula	m/z	err [ppm]	mSigma	# mSigma	Score	rdb	e¯ Conf	N-Rule
	5	C29H67N5O2P	548.5027	-0.3	20.5	5	100.00	0.0	even	ok
	6	C28H66N7OS	548.5044	2.9	22.5	6	43.77	0.0	even	ok
	7	C30H71NNaO3S	548.5047	3.4	23.7	7	34.94	-4.0	even	ok
	8	C26H67N7NaOS	548.5020	-1.5	31.0	8	56.40	-3.0	even	ok
	9	C28H71NO6P	548.5014	-2.7	31.5	9	37.80	-5.0	even	ok
	10	C27H70N3O5S	548.5031	0.4	32.3	10	72.93	-5.0	even	ok
	11	C27H72N3O3P2	548.5043	2.8	34.8	11	34.03	-5.0	even	ok
	12	C27H68N5NaO2P	548.5003	-4.6	36.6	12	10.97	-3.0	even	ok
	13	C29H75NOPS2	548.5022	-1.1	39.5	13	63.44	-6.0	even	ok
	14	C31H70N3S2	548.5006	-4.1	40.1	14	21.15	-1.0	even	ok
	15	C25H71N3NaO5S	548.5007	-4.0	41.0	15	17.85	-8.0	even	ok
	16	C23H66N9O3S	548.5004	-4.5	41.8	16	13.87	-4.0	even	ok
	17	C24H72N5NaO2PS	548.5037	1.5	43.1	17	41.18	-8.0	even	ok
	18	C25H75NO6PS	548.5047	3.4	44.1	18	20.22	-10.0	even	ok
	19	C21H58N17	548.5056	5.0	51.3	19	6.13	2.0	even	ok
	20	C25H73N3NaO3P2	548.5019	-1.6	53.1	20	22.04	-8.0	even	ok
	21	C23H68N9OP2	548.5017	-2.1	54.5	21	17.93	-4.0	even	ok
553.4586	1	C31H58N6NaO	553.4564	-3.9	5.4	1	34.20	6.0	even	ok
	2	C32H61N2O5	553.4575	-1.9	6.4	2	70.04	4.0	even	ok
	3	C31H62N4O2P	553.4605	3.5	7.5	3	39.47	4.0	even	ok
	4	C33H57N6O	553.4588	0.5	9.9	4	100.00	9.0	even	ok
	5	C35H62NaO3	553.4591	1.0	11.2	5	85.67	5.0	even	ok
	6	C29H63N4NaO2P	553.4581	-0.9	17.9	6	76.75	1.0	even	ok
	7	C30H66O6P	553.4592	1.0	18.1	7	73.42	-1.0	even	ok
	8	C27H58N10P	553.4578	-1.4	21.8	8	45.93	5.0	even	ok
	9	C28H62N6NaOS	553.4598	2.2	23.6	9	45.53	1.0	even	ok
	10	C29H65N2O5S	553.4609	4.1	27.3	10	19.48	-1.0	even	ok
	11	C34H66OPS	553.4567	-3.5	27.6	11	25.77	3.0	even	ok
	12	C28H67NaO6P	553.4567	-3.3	28.9	12	26.80	-4.0	even	ok
	13	C26H62N6O4P	553.4565	-3.8	29.9	13	21.19	0.0	even	ok
	14	C25H61N8O3S	553.4582	-0.7	31.4	14	59.11	0.0	even	ok
	15	C24H62N10PS	553.4612	4.7	31.7	15	13.49	0.0	even	ok
	16	C27H68N2NaO3P2	553.4597	2.1	32.0	16	38.81	-4.0	even	ok
	17	C27H66N2NaO5S	553.4585	-0.2	32.2	17	65.54	-4.0	even	ok
	18	C25H63N8OP2	553.4595	1.6	38.2	18	29.12	0.0	even	ok
	19	C29H71NaOPS2	553.4576	-1.7	39.7	19	44.53	-5.0	even	ok
	20	C23H62N8NaO3S	553.4558	-5.1	39.7	20	9.13	-3.0	even	ok
	21	C22H63N10NaPS	553.4588	0.3	40.9	21	50.49	-3.0	even	ok
	22	C31H66N2NaS2	553.4560	-4.7	41.0	22	13.00	0.0	even	ok
	23	C31H70OPS2	553.4600	2.6	41.1	23	31.97	-2.0	even	ok
	24	C25H71N4P2S2	553.4590	0.7	41.5	24	56.19	-6.0	even	ok
	25	C24H65N4O7S	553.4568	-3.1	42.0	25	20.51	-5.0	even	ok
	26	C23H66N6O4PS	553.4598	2.3	42.8	26	27.39	-5.0	even	ok
	27	C25H71NaO6PS	553.4601	2.8	43.0	27	22.72	-9.0	even	ok
	28	C26H70N2O3PS2	553.4560	-4.7	43.1	28	12.68	-6.0	even	ok
	29	C24H67N4O5P2	553.4581	-0.8	44.1	29	40.94	-5.0	even	ok

Compound Spectrum SmartFormula Report

Meas. m/z	#	Ion Formula	m/z	err [ppm]	mSigma	# mSigma	Score	rdb	e¯ Conf	N-Rule
	30	C33H65N2S2	553.4584	-0.4	45.7	30	54.65	3.0	even	ok
	31	C21H54N16Na	553.4610	4.3	47.8	31	7.87	3.0	even	ok
	32	C23H64N8NaOP2	553.4571	-2.8	51.1	32	13.58	-3.0	even	ok
	33	C23H66N2NaO10	553.4610	4.3	51.5	33	9.30	-8.0	even	ok
	34	C21H61N8O8	553.4607	3.8	52.4	34	11.37	-4.0	even	ok
	35	C20H58N12NaO4	553.4596	1.9	60.3	35	13.60	-2.0	even	ok
	36	C18H53N18O2	553.4593	1.4	61.6	36	15.18	2.0	even	ok
569.4326	1	C32H61N2O4S	569.4347	3.6	4.8	1	40.39	4.0	even	ok
	2	C31H58N6NaS	569.4336	1.8	8.1	2	78.17	6.0	even	ok
	3	C30H62N2NaO4S	569.4323	-0.6	13.5	3	100.00	1.0	even	ok
	4	C32H67NaPS2	569.4314	-2.1	15.2	4	77.46	0.0	even	ok
	5	C28H57N8O2S	569.4320	-1.1	15.3	5	84.52	5.0	even	ok
	6	C29H66N2O2PS2	569.4298	-4.9	17.2	6	21.68	-1.0	even	ok
	7	C29H63N4NaOPS	569.4352	4.7	17.7	7	19.36	1.0	even	ok
	8	C34H66PS2	569.4338	2.2	21.1	8	66.25	3.0	even	ok
	9	C37H62PS	569.4304	-3.8	22.9	9	26.88	8.0	even	ok
	10	C25H61N8O2S2	569.4353	4.9	23.2	10	19.61	0.0	even	ok
	11	C27H61N4O6S	569.4306	-3.4	25.2	11	29.70	0.0	even	ok
	12	C27H71O3P2S2	569.4314	-2.0	25.7	12	63.05	-6.0	even	ok
	13	C35H57N2O4	569.4313	-2.3	25.8	13	45.87	9.0	even	ok
	14	C33H62O5P	569.4329	0.6	27.0	14	73.79	4.0	even	ok
	15	C28H67NaO5PS	569.4339	2.3	27.5	15	43.27	-4.0	even	ok
	16	C34H58N4OP	569.4343	3.0	28.7	16	32.79	9.0	even	ok
	17	C26H62N6O3PS	569.4336	1.8	28.9	17	49.50	0.0	even	ok
	18	C34H54N6Na	569.4302	-4.1	29.8	18	19.15	11.0	even	ok
	19	C32H59N4NaOP	569.4319	-1.2	31.0	19	56.66	6.0	even	ok
	20	C23H62N8NaO2S2	569.4329	0.6	31.5	20	82.93	-3.0	even	ok
	21	C38H58NaO2	569.4329	0.6	31.6	21	67.23	10.0	even	ok
	22	C36H53N6	569.4326	0.1	32.8	22	73.57	14.0	even	ok
	23	C24H65N4O6S2	569.4340	2.5	33.2	23	44.09	-5.0	even	ok
	24	C31H63NaO5P	569.4305	-3.6	33.3	24	22.66	1.0	even	ok
	25	C21H57N14S2	569.4327	0.1	35.2	25	68.14	1.0	even	ok
	26	C29H58N6O3P	569.4303	-4.1	35.6	26	17.07	5.0	even	ok
	27	C30H64N2NaO2P2	569.4335	1.7	37.8	27	41.84	1.0	even	ok
	28	C40H57O2	569.4353	4.8	38.4	28	11.14	13.0	even	ok
	29	C25H66N2O7PS	569.4323	-0.5	39.4	29	55.61	-5.0	even	ok
	30	C24H63N6NaO3PS	569.4312	-2.4	40.0	30	30.75	-3.0	even	ok
	31	C22H58N12OPS	569.4309	-2.9	41.5	31	24.53	1.0	even	ok
	32	C22H66N4NaO6S2	569.4316	-1.7	41.9	32	45.86	-8.0	even	ok
	33	C24H67N4O4P2S	569.4353	4.7	42.9	33	10.12	-5.0	even	ok
	34	C28H59N8P2	569.4332	1.2	43.0	34	31.56	5.0	even	ok
	35	C23H69O10S2	569.4327	0.2	43.5	35	67.85	-10.0	even	ok
	36	C23H64N8NaP2S	569.4342	2.9	43.8	36	23.14	-3.0	even	ok
	37	C27H63N4O4P2	569.4319	-1.2	45.5	37	39.05	0.0	even	ok
	38	C26H62N2NaO9	569.4348	3.8	48.8	38	13.31	-3.0	even	ok
	39	C23H67N2NaO7PS	569.4299	-4.7	50.6	39	8.08	-8.0	even	ok

Compound Spectrum SmartFormula Report

Meas. m/z	#	Ion Formula	m/z	err [ppm]	mSigma	# mSigma	Score	rdb	e⁻ Conf	N-Rule
	40	C24H57N8O7	569.4345	3.3	50.8	40	15.56	1.0	even	ok
	41	C26H60N8NaP2	569.4308	-3.1	52.8	41	12.27	2.0	even	ok
	42	C26H67O8P2	569.4306	-3.5	52.9	42	13.36	-5.0	even	ok
	43	C23H54N12NaO3	569.4334	1.5	58.8	43	17.88	3.0	even	ok
	44	C23H61N4O11	569.4331	1.0	59.1	44	27.04	-4.0	even	ok
	45	C22H58N8NaO7	569.4321	-0.9	60.8	45	26.14	-2.0	even	ok
	46	C21H49N18O	569.4331	1.0	61.0	46	19.14	7.0	even	ok
	47	C22H65O15	569.4318	-1.4	68.4	47	17.45	-9.0	even	ok
	48	C21H62N4NaO11	569.4307	-3.2	69.6	48	8.52	-7.0	even	ok
	49	C20H53N14O5	569.4318	-1.4	70.3	49	12.14	2.0	even	ok
	50	C19H57N10O9	569.4304	-3.7	71.2	50	6.48	-3.0	even	ok
	51	C19H50N18NaO	569.4307	-3.3	72.3	51	5.73	4.0	even	ok
	52	C21H59N10NaO4P	569.4351	4.4	72.9	52	3.41	-2.0	even	ok
	53	C19H54N16O2P	569.4348	3.9	75.0	53	3.98	2.0	even	ok
590.5498	1	C38H72NO3	590.5507	1.5	3.6	1	100.00	4.0	even	ok
	2	C36H73NNaO3	590.5483	-2.6	13.5	2	57.72	1.0	even	ok
	3	C34H68N7O	590.5480	-3.0	15.4	3	45.77	5.0	even	ok
	4	C35H74N3NaP	590.5513	2.5	18.0	4	53.56	1.0	even	ok
	5	C31H72N7OS	590.5514	2.7	25.1	5	42.87	0.0	even	ok
	6	C33H77NNaO3S	590.5516	3.2	25.5	6	34.81	-4.0	even	ok
	7	C32H73N5O2P	590.5496	-0.2	29.3	7	85.76	0.0	even	ok
	8	C29H73N7NaOS	590.5490	-1.4	34.5	8	54.22	-3.0	even	ok
	9	C34H76N3S2	590.5475	-3.8	34.6	9	25.76	-1.0	even	ok
	10	C30H76N3O5S	590.5500	0.4	36.3	10	68.63	-5.0	even	ok
	11	C32H81NOPS2	590.5492	-1.0	36.4	11	72.32	-6.0	even	ok
	12	C31H77NO6P	590.5483	-2.5	39.9	12	31.92	-5.0	even	ok
	13	C30H82NNaOPS2	590.5468	-5.1	40.5	13	11.28	-9.0	even	ok
	14	C30H74N5NaO2P	590.5472	-4.3	40.7	14	13.82	-3.0	even	ok
	15	C28H69N11P	590.5470	-4.8	42.2	15	10.28	1.0	even	ok
	16	C30H78N3O3P2	590.5513	2.6	43.6	16	27.86	-5.0	even	ok
	17	C28H77N3NaO5S	590.5476	-3.7	46.2	17	16.20	-8.0	even	ok
	18	C26H72N9O3S	590.5473	-4.1	46.5	18	12.75	-4.0	even	ok
	19	C26H77N7NaOS2	590.5523	4.3	46.7	19	14.38	-8.0	even	ok
	20	C27H78N5NaO2PS	590.5506	1.4	48.2	20	36.85	-8.0	even	ok
	21	C25H73N11PS	590.5503	0.9	48.8	21	41.88	-4.0	even	ok
	22	C28H81NO6PS	590.5517	3.2	49.4	22	17.95	-10.0	even	ok
	23	C28H79N3NaO3P2	590.5489	-1.5	54.9	23	29.15	-8.0	even	ok
	24	C24H64N17	590.5525	4.6	61.5	24	4.56	2.0	even	ok
	25	C26H76N3O10	590.5525	4.7	62.4	25	5.83	-9.0	even	ok
	26	C25H73N7NaO6	590.5515	2.8	62.7	26	13.65	-7.0	even	ok
	27	C23H68N13O4	590.5512	2.4	64.0	27	15.74	-3.0	even	ok
	28	C26H74N9OP2	590.5486	-2.0	64.6	28	13.40	-4.0	even	ok
	29	C22H65N17Na	590.5501	0.6	74.4	29	14.49	-1.0	even	ok
628.5505	1	C37H74NO6	628.5511	0.9	3.0	1	100.00	2.0	even	ok
	2	C36H71N5NaO2	628.5500	-0.8	5.4	2	98.98	4.0	even	ok
	3	C34H66N11	628.5497	-1.2	7.0	3	83.53	8.0	even	ok

Compound Spectrum SmartFormula Report

Meas. m/z	#	Ion Formula	m/z	err [ppm]	mSigma	# mSigma	Score	rdb	e⁻ Conf	N-Rule
	4	C35H75NNaO6	628.5487	-2.9	8.8	4	41.53	-1.0	even	ok
	5	C33H70N7O4	628.5484	-3.4	10.5	5	32.54	3.0	even	ok
	6	C32H67N11Na	628.5473	-5.1	12.8	6	11.96	5.0	even	ok
	7	C34H76N3NaO3P	628.5517	1.8	13.0	7	60.61	-1.0	even	ok
	8	C38H70N5O2	628.5524	3.0	14.7	8	35.42	7.0	even	ok
	9	C32H71N9OP	628.5514	1.4	15.0	9	68.43	3.0	even	ok
	10	C31H70N11S	628.5531	4.1	15.7	10	19.89	3.0	even	ok
	11	C33H80N3OP2S	628.5492	-2.1	17.5	11	50.16	-3.0	even	ok
	12	C33H75N5NaO2S	628.5534	4.6	17.5	12	14.86	-1.0	even	ok
	13	C35H75N5PS	628.5475	-4.7	17.7	13	13.45	2.0	even	ok
	14	C29H71N11NaS	628.5507	0.3	21.4	14	83.71	0.0	even	ok
	15	C30H74N7O4S	628.5518	2.0	24.1	15	45.15	-2.0	even	ok
	16	C31H75N5O5P	628.5500	-0.7	24.8	16	68.35	-2.0	even	ok
	17	C32H79NNaO6S	628.5520	2.4	25.1	17	36.94	-6.0	even	ok
	18	C30H72N9NaOP	628.5490	-2.4	25.9	18	36.15	0.0	even	ok
	19	C32H81NNaO4P2	628.5533	4.5	27.1	19	12.77	-6.0	even	ok
	20	C30H76N7O2P2	628.5530	4.0	28.7	20	15.81	-2.0	even	ok
	21	C26H70N13O2S	628.5491	-2.3	32.4	21	33.06	-1.0	even	ok
	22	C28H75N7NaO4S	628.5493	-1.8	32.4	22	39.33	-5.0	even	ok
	23	C27H76N9NaOPS	628.5523	2.9	33.8	23	24.15	-5.0	even	ok
	24	C32H79N5PS2	628.5509	0.6	34.1	24	70.25	-3.0	even	ok
	25	C30H80N5NaPS2	628.5485	-3.2	34.4	25	26.29	-6.0	even	ok
	26	C29H78N3O8S	628.5504	-0.1	34.6	26	63.71	-7.0	even	ok
	27	C28H79N5O5PS	628.5534	4.6	35.5	27	9.43	-7.0	even	ok
	28	C30H79NO9P	628.5487	-2.9	35.7	28	23.59	-7.0	even	ok
	29	C30H84N3OP2S2	628.5526	3.3	35.9	29	24.26	-8.0	even	ok
	30	C29H76N5NaO5P	628.5476	-4.6	36.1	30	9.59	-5.0	even	ok
	31	C33H78N3O3S2	628.5479	-4.1	37.4	31	15.01	-3.0	even	ok
	32	C27H71N11O3P	628.5473	-5.0	37.6	32	7.03	-1.0	even	ok
	33	C31H83NO4PS2	628.5496	-1.5	37.9	33	48.48	-8.0	even	ok
	34	C29H80N3O6P2	628.5517	1.9	39.1	34	32.39	-7.0	even	ok
	35	C36H79NNaOS2	628.5495	-1.5	39.8	35	45.12	-2.0	even	ok
	36	C28H77N7NaO2P2	628.5506	0.2	39.9	36	54.65	-5.0	even	ok
	37	C41H74NOS	628.5486	-3.1	40.8	37	18.63	6.0	even	ok
	38	C27H79N3NaO8S	628.5480	-4.0	43.9	38	10.93	-10.0	even	ok
	39	C25H74N9O6S	628.5477	-4.4	44.0	39	8.49	-6.0	even	ok
	40	C38H78NOS2	628.5519	2.3	46.1	40	28.43	1.0	even	ok
	41	C26H72N13P2	628.5503	-0.3	47.5	41	32.47	-1.0	even	ok
	42	C25H71N11NaO5	628.5532	4.3	47.6	42	8.26	-4.0	even	ok
	43	C23H66N17O3	628.5529	3.8	49.0	43	10.11	0.0	even	ok
	44	C27H81N3NaO6P2	628.5493	-1.9	50.4	44	22.95	-10.0	even	ok
633.5067	1	C37H70NaO6	633.5065	-0.4	4.0	1	100.00	3.0	even	ok
	2	C35H65N6O4	633.5062	-0.8	6.3	2	84.40	7.0	even	ok
	3	C38H71N2OP2	633.5036	-4.9	8.0	3	12.57	6.0	even	ok
	4	C39H69O6	633.5089	3.4	8.6	4	28.86	6.0	even	ok
	5	C36H71N2NaO3P	633.5095	4.3	8.7	5	17.47	3.0	even	ok

Compound Spectrum SmartFormula Report

Meas. m/z	#	Ion Formula	m/z	err [ppm]	mSigma	# mSigma	Score	rdb	e¯ Conf	N-Rule
	6	C34H62N10Na	633.5051	-2.5	9.8	6	42.45	9.0	even	ok
	7	C38H66N4NaO2	633.5078	1.7	9.8	7	58.84	8.0	even	ok
	8	C36H61N10	633.5075	1.3	10.3	8	68.24	12.0	even	ok
	9	C34H66N8OP	633.5092	3.9	11.1	9	21.43	7.0	even	ok
	10	C35H75N2OP2S	633.5070	0.4	15.1	10	80.71	1.0	even	ok
	11	C34H69N2O8	633.5048	-3.0	15.7	11	31.29	2.0	even	ok
	12	C33H66N6NaO4	633.5038	-4.6	16.8	12	12.40	4.0	even	ok
	13	C31H66N10NaS	633.5085	2.8	16.9	13	32.82	4.0	even	ok
	14	C36H74O4PS	633.5040	-4.3	18.4	14	14.71	1.0	even	ok
	15	C31H61N12O2	633.5035	-5.1	18.7	15	9.05	8.0	even	ok
	16	C33H76N2NaOP2S	633.5046	-3.4	19.4	16	23.71	-2.0	even	ok
	17	C33H70N4O5P	633.5078	1.8	19.6	17	47.44	2.0	even	ok
	18	C32H69N6O4S	633.5096	4.5	20.1	18	12.70	2.0	even	ok
	19	C37H70N4PS	633.5053	-2.2	20.3	19	39.85	6.0	even	ok
	20	C32H67N8NaOP	633.5068	0.1	21.2	20	78.03	4.0	even	ok
	21	C34H74NaO6S	633.5098	4.9	21.4	21	9.43	-2.0	even	ok
	22	C28H65N12O2S	633.5069	0.2	27.1	22	65.50	3.0	even	ok
	23	C30H70N6NaO4S	633.5071	0.7	27.3	23	57.53	-1.0	even	ok
	24	C31H73N2O8S	633.5082	2.4	29.8	24	29.64	-3.0	even	ok
	25	C32H74O9P	633.5065	-0.3	30.3	25	59.10	-3.0	even	ok
	26	C31H71N4NaO5P	633.5054	-2.0	31.0	26	33.11	-1.0	even	ok
	27	C29H66N10O3P	633.5051	-2.5	32.5	27	26.55	3.0	even	ok
	28	C32H75N4NaPS2	633.5063	-0.7	32.7	28	63.63	-2.0	even	ok
	29	C31H75N2O6P2	633.5095	4.4	33.9	29	9.77	-3.0	even	ok
	30	C34H74N4PS2	633.5087	3.1	34.1	30	23.48	1.0	even	ok
	31	C41H70NaOS	633.5040	-4.3	34.6	31	9.76	7.0	even	ok
	32	C30H72N6NaO2P2	633.5084	2.7	34.9	32	22.65	-1.0	even	ok
	33	C33H78O4PS2	633.5074	1.0	36.3	33	51.61	-4.0	even	ok
	34	C30H80N2NaOP2S2	633.5080	2.0	36.6	34	36.95	-7.0	even	ok
	35	C28H67N12P2	633.5081	2.3	36.6	35	26.19	3.0	even	ok
	36	C29H74N6O2PS2	633.5047	-3.2	36.7	36	21.28	-3.0	even	ok
	37	C26H66N12NaO2S	633.5045	-3.6	37.0	37	14.16	0.0	even	ok
	38	C24H61N18S	633.5042	-4.0	37.5	38	11.07	4.0	even	ok
	39	C35H73N2O3S2	633.5057	-1.6	37.8	39	41.10	1.0	even	ok
	40	C31H79NaO4PS2	633.5050	-2.8	38.0	40	25.28	-7.0	even	ok
	41	C29H74N2NaO8S	633.5058	-1.4	38.5	41	34.09	-6.0	even	ok
	42	C27H69N8O6S	633.5055	-1.9	38.5	42	28.98	-2.0	even	ok
	43	C28H75N4NaO5PS	633.5088	3.3	40.0	43	14.93	-6.0	even	ok
	44	C26H70N10O3PS	633.5085	2.9	40.3	44	18.28	-2.0	even	ok
	45	C29H78O9PS	633.5099	5.0	41.6	45	5.51	-8.0	even	ok
	46	C30H75NaO9P	633.5041	-4.1	41.7	46	9.12	-6.0	even	ok
	47	C38H74NaOS2	633.5073	1.0	41.8	47	45.31	2.0	even	ok
	48	C28H70N6O7P	633.5038	-4.6	43.0	48	6.80	-2.0	even	ok
	49	C27H79N4O3P2S2	633.5063	-0.6	43.8	49	48.01	-8.0	even	ok
	50	C29H76N2NaO6P2	633.5071	0.6	45.2	50	37.04	-6.0	even	ok
	51	C43H69OS	633.5064	-0.6	45.9	51	36.71	10.0	even	ok

Compound Spectrum SmartFormula Report

Meas. m/z	#	Ion Formula	m/z	err [ppm]	mSigma	# mSigma	Score	rdb	e⁻ Conf	N-Rule
	52	C27H71N8O4P2	633.5068	0.1	46.7	52	40.14	-2.0	even	ok
	53	C40H73OS2	633.5097	4.8	49.2	53	6.31	5.0	even	ok
	54	C26H73N4O10S	633.5042	-4.0	50.1	54	7.78	-7.0	even	ok
	55	C24H71N10NaO3PS	633.5061	-0.9	50.9	55	28.01	-5.0	even	ok
	56	C26H78N2NaO8S2	633.5092	3.9	51.2	56	9.86	-11.0	even	ok
	57	C22H66N16OPS	633.5058	-1.4	51.5	57	23.75	-1.0	even	ok
	58	C27H79NaO9PS	633.5075	1.2	51.7	58	25.34	-11.0	even	ok
	59	C25H74N6O7PS	633.5072	0.7	52.0	59	28.86	-7.0	even	ok
	60	C26H70N6NaO9	633.5096	4.6	53.2	60	4.85	-5.0	even	ok
	61	C24H65N12O7	633.5094	4.2	54.5	61	6.04	-1.0	even	ok
	62	C26H68N12NaP2	633.5057	-1.5	54.7	62	15.24	0.0	even	ok
	63	C23H62N16NaO3	633.5083	2.5	55.1	63	13.70	1.0	even	ok
	64	C26H75N4O8P2	633.5055	-2.0	57.3	64	15.93	-7.0	even	ok
	65	C25H72N8NaO4P2	633.5044	-3.7	57.9	65	7.24	-5.0	even	ok
	66	C25H74N2NaO13	633.5083	2.5	64.2	66	10.03	-10.0	even	ok
	67	C21H57N22O	633.5080	2.1	65.0	67	8.83	5.0	even	ok
	68	C23H69N8O11	633.5080	2.1	65.3	68	11.58	-6.0	even	ok
	69	C22H66N12NaO7	633.5070	0.4	65.7	69	20.20	-4.0	even	ok
	70	C23H67N14O2P2	633.5041	-4.1	68.2	70	2.99	-1.0	even	ok
649.4800	1	C35H65N6O3S	649.4833	5.1	6.1	1	11.42	7.0	even	ok
	2	C36H72N2NaP2S	649.4784	-2.6	9.1	2	44.41	3.0	even	ok
	3	C34H69N2O7S	649.4820	3.0	10.7	3	34.68	2.0	even	ok
	4	C33H66N6NaO3S	649.4809	1.4	12.3	4	67.59	4.0	even	ok
	5	C38H71N2P2S	649.4808	1.1	12.6	5	73.32	6.0	even	ok
	6	C39H70O3PS	649.4778	-3.5	14.2	6	25.90	6.0	even	ok
	7	C31H61N12OS	649.4807	1.0	14.4	7	75.24	8.0	even	ok
	8	C33H76N2NaP2S2	649.4817	2.6	15.0	8	48.35	-2.0	even	ok
	9	C32H70N6OPS2	649.4785	-2.4	15.3	9	52.85	2.0	even	ok
	10	C33H71N4O2P2S	649.4767	-5.1	16.5	10	9.57	2.0	even	ok
	11	C34H75NaO3PS2	649.4787	-2.0	16.7	11	61.51	-2.0	even	ok
	12	C34H65N8S2	649.4768	-5.0	17.2	12	12.59	7.0	even	ok
	13	C36H74O3PS2	649.4812	1.7	18.6	13	65.60	1.0	even	ok
	14	C36H70N2NaO2S2	649.4771	-4.5	19.3	14	15.86	3.0	even	ok
	15	C32H70N2NaO7S	649.4796	-0.7	22.0	15	70.05	-1.0	even	ok
	16	C31H74N2O5PS2	649.4771	-4.5	22.8	16	15.23	-3.0	even	ok
	17	C30H75N4O2P2S2	649.4801	0.1	23.0	17	100.00	-3.0	even	ok
	18	C30H65N8O5S	649.4793	-1.1	23.5	18	59.00	3.0	even	ok
	19	C37H65N2O7	649.4786	-2.2	24.1	19	38.98	7.0	even	ok
	20	C29H62N12NaOS	649.4782	-2.8	24.8	20	29.55	5.0	even	ok
	21	C31H71N4NaO4PS	649.4826	3.9	25.8	21	15.70	-1.0	even	ok
	22	C40H66NaO5	649.4802	0.3	26.0	22	70.82	8.0	even	ok
	23	C38H69N2O2S2	649.4795	-0.8	27.1	23	74.46	6.0	even	ok
	24	C29H66N10O2PS	649.4823	3.5	27.5	24	19.13	3.0	even	ok
	25	C38H61N6O3	649.4800	-0.1	27.7	25	72.36	12.0	even	ok
	26	C36H66N4O4P	649.4816	2.4	28.2	26	31.48	7.0	even	ok
	27	C36H62N6NaO3	649.4776	-3.8	28.2	27	15.89	9.0	even	ok

Compound Spectrum SmartFormula Report

Meas. m/z	#	Ion Formula	m/z	err [ppm]	mSigma	# mSigma	Score	rdb	e⁻ Conf	N-Rule
	28	C39H67N2NaO2P	649.4832	4.9	28.5	28	7.98	8.0	even	ok
	29	C26H66N12NaOS2	649.4816	2.4	28.8	29	38.80	0.0	even	ok
	30	C27H69N8O5S2	649.4827	4.1	30.4	30	16.12	-2.0	even	ok
	31	C35H70O8P	649.4803	0.4	30.4	31	62.79	2.0	even	ok
	32	C37H62N8P	649.4830	4.5	30.4	32	10.02	12.0	even	ok
	33	C34H57N12O	649.4773	-4.2	30.5	33	11.73	13.0	even	ok
	34	C29H74N2NaO7S2	649.4830	4.5	30.5	34	12.39	-6.0	even	ok
	35	C42H65O5	649.4827	4.0	30.8	35	13.15	11.0	even	ok
	36	C28H76N4NaO2P2S2	649.4777	-3.6	31.5	36	20.89	-6.0	even	ok
	37	C26H71N10P2S2	649.4774	-4.0	31.7	37	16.34	-2.0	even	ok
	38	C35H63N8NaP	649.4806	0.8	32.2	38	53.07	9.0	even	ok
	39	C41H67N2P2	649.4774	-4.1	32.9	39	12.31	11.0	even	ok
	40	C29H79O6P2S2	649.4788	-1.9	33.0	40	43.46	-8.0	even	ok
	41	C41H62N4NaO	649.4816	2.4	33.9	41	28.03	13.0	even	ok
	42	C34H67N4NaO4P	649.4792	-1.3	33.9	42	43.60	4.0	even	ok
	43	C29H69N4O9S	649.4780	-3.2	34.1	43	19.30	-2.0	even	ok
	44	C28H66N8NaO5S	649.4769	-4.8	34.8	44	7.39	0.0	even	ok
	45	C34H71N2O5P2	649.4833	5.0	35.1	45	6.52	2.0	even	ok
	46	C32H62N10O2P	649.4789	-1.7	36.3	46	35.00	8.0	even	ok
	47	C41H70NaS2	649.4811	1.7	36.3	47	44.18	7.0	even	ok
	48	C30H75NaO8PS	649.4812	1.9	36.4	48	32.61	-6.0	even	ok
	49	C28H70N6O6PS	649.4810	1.4	37.7	49	37.04	-2.0	even	ok
	50	C33H68N6NaOP2	649.4822	3.3	38.5	50	15.72	4.0	even	ok
	51	C27H67N10NaO2PS	649.4799	-0.2	38.7	51	53.42	0.0	even	ok
	52	C33H71NaO8P	649.4779	-3.3	39.0	52	15.69	-1.0	even	ok
	53	C25H70N8NaO5S2	649.4803	0.4	39.2	53	62.72	-5.0	even	ok
	54	C23H65N14O3S2	649.4800	-0.1	39.5	54	68.09	-1.0	even	ok
	55	C25H62N16PS	649.4796	-0.6	40.3	55	45.13	4.0	even	ok
	56	C26H73N4O9S2	649.4813	2.0	40.6	56	34.20	-7.0	even	ok
	57	C24H71N10NaO2PS2	649.4833	5.0	41.0	57	6.99	-5.0	even	ok
	58	C31H66N6O6P	649.4776	-3.8	41.2	58	11.73	3.0	even	ok
	59	C44H66NaS	649.4777	-3.5	41.4	59	13.21	12.0	even	ok
	60	C22H66N16PS2	649.4830	4.6	41.4	60	9.09	-1.0	even	ok
	61	C32H72N2NaO5P2	649.4809	1.3	43.7	61	33.25	-1.0	even	ok
	62	C30H67N8O3P2	649.4806	0.9	45.9	62	36.00	3.0	even	ok
	63	C46H65S	649.4801	0.2	47.8	63	51.97	15.0	even	ok
	64	C27H74N2O10PS	649.4796	-0.6	48.6	64	35.75	-7.0	even	ok
	65	C26H71N6NaO6PS	649.4786	-2.3	49.0	65	19.38	-5.0	even	ok
	66	C24H66N12O4PS	649.4783	-2.7	50.4	66	15.34	-1.0	even	ok
	67	C27H61N12O6	649.4832	4.8	50.9	67	4.71	4.0	even	ok
	68	C23H63N16NaPS	649.4772	-4.3	51.4	68	6.20	1.0	even	ok
	69	C26H75N4O7P2S	649.4826	4.0	52.0	69	7.49	-7.0	even	ok
	70	C25H72N8NaO3P2S	649.4816	2.3	52.7	70	16.76	-5.0	even	ok
	71	C29H71N4O7P2	649.4793	-1.2	52.9	71	25.91	-2.0	even	ok
	72	C26H58N16NaO2	649.4821	3.2	53.3	72	11.19	6.0	even	ok
	73	C23H67N14OP2S	649.4813	1.9	54.2	73	19.13	-1.0	even	ok

Compound Spectrum SmartFormula Report

Meas. m/z	#	Ion Formula	m/z	err [ppm]	mSigma	# mSigma	Score	rdb	e¯ Conf	N-Rule
	74	C28H68N8NaO3P2	649.4782	-2.9	55.2	74	12.26	0.0	even	ok
	75	C28H70N2NaO12	649.4821	3.2	57.1	75	9.85	-5.0	even	ok
	76	C26H63N14OP2	649.4779	-3.3	57.3	76	9.26	4.0	even	ok
	77	C26H65N8O10	649.4818	2.7	58.9	77	11.42	-1.0	even	ok
	78	C25H75N2NaO10PS	649.4772	-4.3	59.9	78	4.76	-10.0	even	ok
	79	C24H53N22	649.4818	2.7	60.7	79	8.13	10.0	even	ok
	80	C25H62N12NaO6	649.4807	1.1	60.8	80	20.69	1.0	even	ok
	81	C28H75O11P2	649.4779	-3.3	61.2	81	8.19	-7.0	even	ok
	82	C23H57N18O4	649.4805	0.7	62.8	82	22.21	5.0	even	ok
	83	C27H72N4NaO7P2	649.4768	-4.9	63.0	83	2.95	-5.0	even	ok
	84	C49H61	649.4768	-5.0	63.3	84	2.74	20.0	even	ok
	85	C25H69N4O14	649.4805	0.7	67.9	85	18.43	-6.0	even	ok
	86	C24H66N8NaO10	649.4794	-1.0	69.4	86	16.02	-4.0	even	ok
	87	C22H61N14O8	649.4791	-1.4	71.2	87	12.94	0.0	even	ok
	88	C22H54N22Na	649.4794	-1.0	72.0	88	10.84	7.0	even	ok
	89	C21H58N18NaO4	649.4781	-3.0	72.9	89	6.03	2.0	even	ok
	90	C23H67N10NaO7P	649.4824	3.6	73.5	90	4.29	-4.0	even	ok
	91	C21H62N16O5P	649.4821	3.2	75.3	91	5.02	0.0	even	ok
	92	C19H53N24O2	649.4778	-3.5	83.7	92	2.36	6.0	even	ok
	93	C20H59N20NaOP	649.4811	1.6	86.5	93	4.92	2.0	even	ok
670.5975	1	C41H76N5O2	670.5994	2.7	5.9	1	46.80	7.0	even	ok
	2	C40H80NO6	670.5980	0.8	8.6	2	100.00	2.0	even	ok
	3	C39H77N5NaO2	670.5969	-0.8	10.7	3	93.67	4.0	even	ok
	4	C37H72N11	670.5967	-1.3	13.0	4	77.56	8.0	even	ok
	5	C38H81N5PS	670.5945	-4.5	15.2	5	14.35	2.0	even	ok
	6	C38H81NNaO6	670.5956	-2.8	20.3	6	33.94	-1.0	even	ok
	7	C34H76N11S	670.6000	3.8	20.5	7	20.34	3.0	even	ok
	8	C36H81N5NaO2S	670.6003	4.2	21.1	8	15.61	-1.0	even	ok
	9	C36H76N7O4	670.5953	-3.2	22.0	9	26.40	3.0	even	ok
	10	C36H86N3OP2S	670.5961	-2.0	23.3	10	45.75	-3.0	even	ok
	11	C35H73N11Na	670.5943	-4.8	23.6	11	9.63	5.0	even	ok
	12	C37H82N3NaO3P	670.5986	1.6	24.4	12	52.98	-1.0	even	ok
	13	C35H77N9OP	670.5983	1.2	26.3	13	59.45	3.0	even	ok
	14	C44H80NOS	670.5955	-3.0	28.5	14	32.78	6.0	even	ok
	15	C32H77N11NaS	670.5976	0.2	29.8	15	75.83	0.0	even	ok
	16	C33H80N7O4S	670.5987	1.8	31.5	16	42.23	-2.0	even	ok
	17	C35H85NNaO6S	670.5990	2.2	31.6	17	35.26	-6.0	even	ok
	18	C35H85N5PS2	670.5979	0.5	33.9	18	77.95	-3.0	even	ok
	19	C39H85NNaOS2	670.5965	-1.5	36.2	19	51.47	-2.0	even	ok
	20	C36H84N3O3S2	670.5949	-3.9	36.3	20	15.70	-3.0	even	ok
	21	C34H81N5O5P	670.5970	-0.8	36.5	21	53.50	-2.0	even	ok
	22	C33H86N5NaPS2	670.5954	-3.1	36.8	22	25.29	-6.0	even	ok
	23	C33H78N9NaOP	670.5959	-2.4	37.6	23	27.85	0.0	even	ok
	24	C33H90N3OP2S2	670.5995	3.0	38.8	24	25.19	-8.0	even	ok
	25	C35H87NNaO4P2	670.6003	4.1	38.8	25	10.76	-6.0	even	ok
	26	C34H89NO4PS2	670.5965	-1.5	39.9	26	47.64	-8.0	even	ok

Compound Spectrum SmartFormula Report

Meas. m/z	#	Ion Formula	m/z	err [ppm]	mSigma	# mSigma	Score	rdb	e⁻ Conf	N-Rule
	27	C33H82N7O2P2	670.6000	3.7	40.5	27	13.20	-2.0	even	ok
	28	C41H84NOS2	670.5989	2.1	41.0	28	36.71	1.0	even	ok
	29	C31H81N7NaO4S	670.5963	-1.8	41.5	29	32.07	-5.0	even	ok
	30	C29H76N13O2S	670.5960	-2.2	41.9	30	26.52	-1.0	even	ok
	31	C32H84N3O8S	670.5974	-0.2	42.9	31	53.36	-7.0	even	ok
	32	C30H82N9NaOPS	670.5993	2.7	43.6	32	20.75	-5.0	even	ok
	33	C31H85N5O5PS	670.6004	4.2	44.7	33	8.33	-7.0	even	ok
	34	C31H91N3NaOP2S2	670.5971	-0.6	44.8	34	56.19	-11.0	even	ok
	35	C32H90NNaO4PS2	670.5941	-5.1	44.9	35	5.96	-11.0	even	ok
	36	C33H85NO9P	670.5956	-2.8	47.3	36	17.54	-7.0	even	ok
	37	C32H82N5NaO5P	670.5946	-4.4	48.0	37	6.98	-5.0	even	ok
	38	C30H77N11O3P	670.5943	-4.8	49.5	38	5.06	-1.0	even	ok
	39	C32H86N3O6P2	670.5986	1.7	50.9	39	25.58	-7.0	even	ok
	40	C31H83N7NaO2P2	670.5976	0.1	51.8	40	42.39	-5.0	even	ok
	41	C30H85N3NaO8S	670.5950	-3.8	53.1	41	8.43	-10.0	even	ok
	42	C29H78N13P2	670.5973	-0.3	53.5	42	37.66	-1.0	even	ok
	43	C28H80N9O6S	670.5947	-4.2	53.6	43	6.45	-6.0	even	ok
	44	C29H86N5NaO5PS	670.5979	0.7	55.3	44	32.11	-10.0	even	ok
	45	C27H81N11O3PS	670.5977	0.2	56.0	45	35.66	-6.0	even	ok
	46	C30H89NO9PS	670.5990	2.2	56.4	46	16.96	-12.0	even	ok
	47	C28H77N11NaO5	670.6001	3.9	59.6	47	6.40	-4.0	even	ok
	48	C26H72N17O3	670.5999	3.5	61.0	48	7.75	0.0	even	ok
	49	C30H87N3NaO6P2	670.5962	-1.9	62.4	49	16.09	-10.0	even	ok
	50	C28H82N9O4P2	670.5960	-2.3	63.8	50	12.77	-6.0	even	ok
	51	C28H84N3O13	670.5999	3.5	69.9	51	5.60	-11.0	even	ok
	52	C27H81N7NaO9	670.5988	1.9	70.3	52	12.02	-9.0	even	ok
	53	C27H79N13NaP2	670.5949	-3.9	74.3	53	2.82	-4.0	even	ok
978.8325	1	C55H105N13P	978.8348	2.4	34.9	1	24.58	11.0	even	ok
	2	C56H104N11O3	978.8318	-0.7	34.9	2	92.52	11.0	even	ok
	3	C58H114N3O4P2	978.8279	-4.7	35.2	3	2.77	5.0	even	ok
	4	C53H106N13NaP	978.8324	-0.1	35.8	4	94.80	8.0	even	ok
	5	C52H100N17O	978.8291	-3.4	36.2	5	9.08	12.0	even	ok
	6	C56H114N3NaO6P	978.8337	1.3	36.9	6	62.55	2.0	even	ok
	7	C58H109N5NaO5	978.8321	-0.4	37.1	7	100.00	7.0	even	ok
	8	C57H110N7NaO2P	978.8351	2.7	37.2	8	18.22	7.0	even	ok
	9	C58H113N3O6P	978.8362	3.8	37.3	9	7.80	5.0	even	ok
	10	C61H115NNaO2P2	978.8295	-3.0	37.5	10	16.34	6.0	even	ok
	11	C55H108N7O7	978.8305	-2.0	37.6	11	36.86	6.0	even	ok
	12	C53H110N11OP2	978.8365	4.1	37.7	12	4.35	6.0	even	ok
	13	C54H105N11NaO3	978.8294	-3.1	37.9	13	11.52	8.0	even	ok
	14	C59H105N9NaO	978.8334	1.0	38.2	14	72.92	12.0	even	ok
	15	C59H110N7P2	978.8292	-3.3	39.0	15	9.53	10.0	even	ok
	16	C55H115N5NaO3P2	978.8367	4.3	39.0	16	2.99	2.0	even	ok
	17	C57H113NNaO9	978.8308	-1.8	39.4	17	43.19	2.0	even	ok
	18	C54H109N9O4P	978.8335	1.0	39.4	18	55.30	6.0	even	ok
	19	C53H109N7NaO7	978.8281	-4.5	40.1	19	2.99	3.0	even	ok

Compound Spectrum SmartFormula Report

Meas. m/z	#	Ion Formula	m/z	err [ppm]	mSigma	# mSigma	Score	rdb	e⁻ Conf	N-Rule
	20	C59H112NO9	978.8332	0.7	41.1	20	77.88	5.0	even	ok
	21	C53H113N5O8P	978.8321	-0.4	42.1	21	88.93	1.0	even	ok
	22	C60H108N5O5	978.8345	2.1	42.1	22	32.30	10.0	even	ok
	23	C50H105N15O2P	978.8308	-1.7	43.0	23	31.81	7.0	even	ok
	24	C54H112N3O11	978.8291	-3.4	43.2	24	9.49	1.0	even	ok
	25	C51H104N13O5	978.8278	-4.8	43.5	25	1.51	7.0	even	ok
	26	C51H111N11NaOP2	978.8341	1.6	43.7	26	34.09	3.0	even	ok
	27	C52H110N9NaO4P	978.8311	-1.4	43.8	27	37.58	3.0	even	ok
	28	C63H114NO2P2	978.8319	-0.6	44.6	28	75.28	9.0	even	ok
	29	C61H104N9O	978.8358	3.4	46.0	29	8.62	15.0	even	ok
	30	C54H115N7NaP2S	978.8302	-2.3	46.1	30	23.63	2.0	even	ok
	31	C63H110N3NaOP	978.8279	-4.7	46.1	31	1.96	11.0	even	ok
	32	C52H114N7O5P2	978.8351	2.7	46.3	32	13.67	1.0	even	ok
	33	C50H110N13NaPS	978.8358	3.4	47.1	33	8.95	3.0	even	ok
	34	C54H119NNaO7P2	978.8354	3.0	47.1	34	10.31	-3.0	even	ok
	35	C49H104N17OS	978.8325	0.0	47.6	35	87.50	7.0	even	ok
	36	C63H109N3NaO3	978.8361	3.7	48.6	36	5.90	11.0	even	ok
	37	C51H114N9O2P2S	978.8286	-4.0	48.9	37	4.41	1.0	even	ok
	38	C51H109N11NaO3S	978.8328	0.3	49.1	38	74.13	3.0	even	ok
	39	C53H108N11O3S	978.8352	2.8	49.3	39	14.76	6.0	even	ok
	40	C52H117NO12P	978.8308	-1.7	50.2	40	32.41	-4.0	even	ok
	41	C51H113N9O4PS	978.8368	4.5	50.4	41	2.37	1.0	even	ok
	42	C47H105N17NaOS	978.8301	-2.4	50.7	42	18.66	4.0	even	ok
	43	C56H109N9NaOS	978.8368	4.4	50.8	43	2.46	7.0	even	ok
	44	C46H101N21P	978.8281	-4.5	51.0	44	1.80	8.0	even	ok
	45	C48H106N15NaO2P	978.8284	-4.2	51.0	45	2.55	4.0	even	ok
	46	C46H100N21O2	978.8363	3.9	51.2	46	3.39	8.0	even	ok
	47	C48H105N15NaO4	978.8366	4.2	51.4	47	2.42	4.0	even	ok
	48	C52H112N7O7S	978.8338	1.4	52.7	48	37.29	1.0	even	ok
	49	C47H109N15O2PS	978.8342	1.7	52.7	49	30.38	2.0	even	ok
	50	C48H115N11NaOP2S	978.8374	5.0	52.9	50	1.01	-2.0	even	ok
	51	C49H109N11O6P	978.8294	-3.1	53.1	51	7.68	2.0	even	ok
	52	C51H114N5NaO8P	978.8297	-2.8	53.5	52	9.88	-2.0	even	ok
	53	C48H108N13O5S	978.8312	-1.3	53.7	53	37.25	2.0	even	ok
	54	C49H114N9NaO4PS	978.8344	2.0	53.7	54	24.05	-2.0	even	ok
	55	C56H114N7P2S	978.8326	0.1	53.7	55	69.01	5.0	even	ok
	56	C48H110N13O3P2	978.8324	-0.0	54.0	56	56.75	2.0	even	ok
	57	C50H115N7NaO5P2	978.8327	0.2	54.1	57	52.22	-2.0	even	ok
	58	C50H113N7NaO7S	978.8314	-1.1	54.9	58	42.46	-2.0	even	ok
	59	C55H118N3O4P2S	978.8313	-1.2	55.1	59	38.33	0.0	even	ok
	60	C53H119N3NaO4P2S	978.8289	-3.7	55.1	60	4.98	-3.0	even	ok
	61	C65H109N3OP	978.8303	-2.2	55.7	61	18.59	14.0	even	ok
	62	C58H119NNaO2P2S	978.8329	0.4	56.0	62	56.60	1.0	even	ok
	63	C51H118N3O9P2	978.8338	1.3	56.3	63	27.67	-4.0	even	ok
	64	C50H117N5O8PS	978.8355	3.1	56.6	64	8.70	-4.0	even	ok
	65	C55H113N5NaO5S	978.8355	3.0	56.7	65	9.00	2.0	even	ok

Compound Spectrum SmartFormula Report

Meas. m/z	#	Ion Formula	m/z	err [ppm]	mSigma	# mSigma	Score	rdb	e⁻ Conf	N-Rule
	66	C53H118N3NaO6PS	978.8371	4.7	56.8	66	1.35	-3.0	even	ok
	67	C50H118NNaO12P	978.8284	-4.2	57.4	67	2.64	-7.0	even	ok
	68	C48H112N7O12	978.8363	4.0	57.5	68	3.42	-3.0	even	ok
	69	C50H117NNaO14	978.8366	4.2	57.7	69	2.44	-7.0	even	ok
	70	C57H113N5O3PS	978.8296	-2.9	57.8	70	9.83	5.0	even	ok
	71	C44H104N19O3S	978.8285	-4.1	57.9	71	2.88	3.0	even	ok
	72	C43H105N21PS	978.8315	-1.0	58.1	72	38.82	3.0	even	ok
	73	C56H117NO7PS	978.8283	-4.3	58.4	73	2.26	0.0	even	ok
	74	C45H110N15NaO2PS	978.8317	-0.7	58.5	74	44.57	-1.0	even	ok
	75	C46H109N13NaO5S	978.8288	-3.8	58.6	75	3.90	-1.0	even	ok
	76	C54H117NNaO9S	978.8341	1.7	59.4	76	24.90	-3.0	even	ok
	77	C56H116NO9S	978.8365	4.1	60.6	77	2.49	0.0	even	ok
	78	C44H101N21NaO2	978.8339	1.5	60.8	78	21.60	5.0	even	ok
	79	C46H113N11O6PS	978.8328	0.3	60.9	79	50.00	-3.0	even	ok
	80	C45H114N13O3P2S	978.8358	3.4	61.0	80	5.54	-3.0	even	ok
	81	C60H118NO2P2S	978.8353	2.9	61.1	81	9.04	4.0	even	ok
	82	C42H96N27	978.8337	1.2	61.2	82	25.46	9.0	even	ok
	83	C47H112N9O9S	978.8298	-2.7	61.2	83	10.51	-3.0	even	ok
	84	C47H119N7NaO5P2S	978.8361	3.7	61.4	84	4.04	-7.0	even	ok
	85	C48H118N5NaO8PS	978.8331	0.6	61.5	85	42.76	-7.0	even	ok
	86	C60H114N3NaOPS	978.8312	-1.3	62.0	86	29.82	6.0	even	ok
	87	C47H109N11NaO8	978.8353	2.9	62.3	87	7.10	-1.0	even	ok
	88	C45H104N17O6	978.8350	2.6	62.3	88	9.11	3.0	even	ok
	89	C51H116N3O11S	978.8325	0.0	62.5	89	53.91	-4.0	even	ok
	90	C46H111N13NaO3P2	978.8300	-2.5	63.8	90	9.18	-1.0	even	ok
	91	C48H122N3O9P2S	978.8372	4.8	63.8	91	1.01	-9.0	even	ok
	92	C48H113N7O10P	978.8281	-4.5	63.9	92	1.20	-3.0	even	ok
	93	C44H106N19OP2	978.8297	-2.8	64.2	93	7.08	3.0	even	ok
	94	C59H108N7O2S	978.8280	-4.6	64.2	94	1.24	10.0	even	ok
	95	C49H119N3NaO9P2	978.8314	-1.1	65.2	95	23.11	-7.0	even	ok
	96	C47H114N9O7P2	978.8311	-1.4	65.2	96	19.39	-3.0	even	ok
	97	C41H106N21NaPS	978.8291	-3.5	65.7	97	4.26	0.0	even	ok
	98	C39H100N27S	978.8370	4.6	65.9	98	1.12	4.0	even	ok
	99	C49H117N3NaO11S	978.8301	-2.4	65.9	99	11.36	-7.0	even	ok
	100	C41H105N21NaO2S	978.8373	4.9	66.0	100	0.77	0.0	even	ok
	101	C51H119N7NaP2S2	978.8336	1.1	66.1	101	27.97	-3.0	even	ok
	102	C61H113NNaO4S	978.8283	-4.3	66.8	102	1.62	6.0	even	ok
	103	C50H113N11OPS2	978.8303	-2.2	67.2	103	12.89	1.0	even	ok
	104	C48H114N11NaOPS2	978.8279	-4.7	67.3	104	1.02	-2.0	even	ok
	105	C47H116N3O16	978.8350	2.6	67.3	105	9.45	-8.0	even	ok
	106	C46H108N17OS2	978.8359	3.5	67.4	106	4.13	2.0	even	ok
	107	C42H109N17O4PS	978.8301	-2.4	67.5	107	10.96	-2.0	even	ok
	108	C53H118N7P2S2	978.8360	3.6	67.6	108	3.61	0.0	even	ok
	109	C44H114N11NaO6PS	978.8304	-2.1	67.7	109	13.61	-6.0	even	ok
	110	C49H121NO12PS	978.8342	1.7	67.9	110	18.03	-9.0	even	ok
	111	C41H110N19OP2S	978.8331	0.7	68.4	111	33.10	-2.0	even	ok

Compound Spectrum SmartFormula Report

Meas. m/z	#	Ion Formula	m/z	err [ppm]	mSigma	# mSigma	Score	rdb	e¯ Conf	N-Rule
	112	C43H115N13NaO3P2S	978.8334	0.9	68.5	112	28.45	-6.0	even	ok
	113	C67H104N5	978.8286	-3.9	68.6	113	2.37	19.0	even	ok
	114	C48H118N9O2P2S2	978.8320	-0.5	68.6	114	35.00	-4.0	even	ok
	115	C48H113N11NaO3S2	978.8361	3.7	69.0	115	2.88	-2.0	even	ok
	116	C52H118N5NaO3PS2	978.8306	-1.9	69.1	116	14.87	-3.0	even	ok
	117	C52H108N13S2	978.8287	-3.9	69.1	117	2.41	6.0	even	ok
	118	C62H113N3OPS	978.8336	1.2	69.5	118	23.72	9.0	even	ok
	119	C46H116N5O13S	978.8285	-4.1	69.6	119	1.94	-8.0	even	ok
	120	C44H109N17NaOS2	978.8335	1.0	69.7	120	26.28	-1.0	even	ok
	121	C52H122N3O4P2S2	978.8347	2.2	69.8	121	11.66	-5.0	even	ok
	122	C45H117N7O10PS	978.8315	-1.0	69.8	122	25.87	-8.0	even	ok
	123	C55H123NNaO2P2S2	978.8363	3.9	69.8	123	2.44	-4.0	even	ok
	124	C50H123N3NaO4P2S2	978.8322	-0.2	70.2	124	37.59	-8.0	even	ok
	125	C44H114N15P2S2	978.8293	-3.3	70.2	125	4.55	-3.0	even	ok
	126	C44H118N9O7P2S	978.8345	2.0	70.3	126	13.18	-8.0	even	ok
	127	C46H123N3NaO9P2S	978.8347	2.3	70.6	127	10.47	-12.0	even	ok
	128	C49H117N7O5PS2	978.8290	-3.6	70.8	128	3.22	-4.0	even	ok
	129	C62H109N5NaS	978.8296	-3.0	70.9	129	5.98	11.0	even	ok
	130	C46H119N9NaO2P2S2	978.8296	-3.0	71.3	130	5.73	-7.0	even	ok
	131	C54H117N5O3PS2	978.8330	0.5	71.4	131	31.47	0.0	even	ok
	132	C54H113N7NaO2S2	978.8289	-3.6	71.4	132	3.03	2.0	even	ok
	133	C40H97N27Na	978.8312	-1.3	72.0	133	16.66	6.0	even	ok
	134	C53H117N3NaO6S2	978.8276	-5.0	72.4	134	0.56	-3.0	even	ok
	135	C43H105N17NaO6	978.8326	0.1	72.4	135	29.04	0.0	even	ok
	136	C51H122NNaO7PS2	978.8293	-3.3	72.5	136	4.05	-8.0	even	ok
	137	C49H116N7O7S2	978.8372	4.8	72.6	137	0.68	-4.0	even	ok
	138	C45H112N13O5S2	978.8345	2.1	72.9	138	11.41	-3.0	even	ok
	139	C41H100N23O4	978.8323	-0.2	72.9	139	27.97	4.0	even	ok
	140	C53H121NO7PS2	978.8317	-0.8	72.9	140	25.48	-5.0	even	ok
	141	C47H122NNaO12PS	978.8318	-0.7	73.4	141	26.34	-12.0	even	ok
	142	C46H113N7NaO12	978.8339	1.5	73.5	142	13.57	-6.0	even	ok
	143	C44H108N13O10	978.8337	1.2	73.7	143	16.06	-2.0	even	ok
	144	C57H118N3NaOPS2	978.8346	2.2	74.2	144	10.17	1.0	even	ok
	145	C47H117N7NaO7S2	978.8348	2.4	74.2	145	8.66	-7.0	even	ok
	146	C47H122N5O6P2S2	978.8306	-1.9	74.4	146	12.51	-9.0	even	ok
	147	C37H101N27NaS	978.8346	2.2	74.4	147	10.04	1.0	even	ok
	148	C42H106N19NaO3P	978.8356	3.2	74.6	148	3.37	0.0	even	ok
	149	C63H112NO4S	978.8307	-1.9	74.7	149	12.65	9.0	even	ok
	150	C40H109N21PS2	978.8348	2.4	75.1	150	8.19	-2.0	even	ok
	151	C40H101N25OP	978.8353	2.9	75.2	151	4.31	4.0	even	ok
	152	C45H114N9NaO9P	978.8369	4.6	75.3	152	0.71	-6.0	even	ok
	153	C55H116N3O6S2	978.8300	-2.5	75.4	153	7.35	0.0	even	ok
	154	C45H115N9NaO7P2	978.8287	-3.9	75.4	154	1.58	-6.0	even	ok
	155	C43H109N15O7P	978.8367	4.3	75.6	155	0.99	-2.0	even	ok
	156	C41H108N19O3S2	978.8318	-0.6	75.7	156	25.27	-2.0	even	ok
	157	C40H110N17NaO4PS	978.8277	-4.9	75.7	157	0.58	-5.0	even	ok

Compound Spectrum SmartFormula Report

Meas. m/z	#	Ion Formula	m/z	err [ppm]	mSigma	# mSigma	Score	rdb	e⁻ Conf	N-Rule
	158	C40H109N17NaO6S	978.8360	3.6	75.8	158	2.71	-5.0	even	ok
	159	C38H104N23O4S	978.8357	3.3	75.9	159	3.63	-1.0	even	ok
	160	C42H114N15NaO2PS2	978.8351	2.7	75.9	160	6.24	-6.0	even	ok
	161	C43H110N15O5P2	978.8284	-4.2	75.9	161	1.12	-2.0	even	ok
	162	C48H121N3O9PS2	978.8276	-4.9	76.0	162	0.51	-9.0	even	ok
	163	C45H117N3NaO16	978.8326	0.1	76.0	163	31.45	-11.0	even	ok
	164	C56H112N7O2S2	978.8313	-1.2	76.2	164	18.75	5.0	even	ok
	165	C43H113N13NaO5S2	978.8321	-0.4	76.6	165	27.84	-6.0	even	ok
	166	C46H118N5O11P2	978.8298	-2.8	76.7	166	4.49	-8.0	even	ok
	167	C53H2NaO12P2S2	978.8355	3.1	77.0	167	4.12	54.0	even	ok
	168	C39H111N19NaOP2S	978.8307	-1.8	77.1	168	12.00	-5.0	even	ok
	169	C41H113N13O8PS	978.8288	-3.8	77.3	169	2.06	-7.0	even	ok
	170	C43H118N7NaO10PS	978.8291	-3.5	77.4	170	2.78	-11.0	even	ok
	171	C48H120N3O11S2	978.8359	3.5	77.5	171	2.78	-9.0	even	ok
	172	C41H112N13O10S	978.8370	4.7	77.5	172	0.71	-7.0	even	ok
	173	C43H118N11O4P2S2	978.8279	-4.6	77.6	173	0.73	-8.0	even	ok
	174	C43H117N7NaO12S	978.8373	4.9	77.7	174	0.49	-11.0	even	ok
	175	C42H119N9NaO7P2S	978.8321	-0.4	78.4	175	25.17	-11.0	even	ok
	176	C40H114N15O5P2S	978.8318	-0.7	78.5	176	21.89	-7.0	even	ok
	177	C45H123N5NaO6P2S2	978.8282	-4.3	78.5	177	1.00	-12.0	even	ok
	178	C43H117N11O6PS2	978.8362	3.8	78.6	178	1.91	-8.0	even	ok
	179	C58H117NNaO4S2	978.8316	-0.9	78.7	179	19.92	1.0	even	ok
	180	C45H121NO17P	978.8367	4.3	78.9	180	1.08	-13.0	even	ok
	181	C44H121N3O14PS	978.8301	-2.4	79.2	181	7.07	-13.0	even	ok
	182	C50HN2O14P2S2	978.8339	1.4	79.4	182	13.87	53.0	even	ok
	183	C44H116N9O9S2	978.8332	0.7	79.5	183	20.78	-8.0	even	ok
	184	C45H122N5NaO8PS2	978.8365	4.1	79.6	184	1.33	-12.0	even	ok
	185	C43H122N5O11P2S	978.8331	0.7	80.1	185	21.02	-13.0	even	ok
	186	C64H108N5S	978.8320	-0.5	80.2	186	22.68	14.0	even	ok
	187	C59H117N3OPS2	978.8370	4.6	80.4	187	0.65	4.0	even	ok
	188	C46H121N3NaO11S2	978.8335	1.0	80.6	188	17.07	-12.0	even	ok
	189	C37H104N25OS2	978.8292	-3.4	80.6	189	2.68	-1.0	even	ok
	190	C39H109N19NaO3S2	978.8294	-3.1	81.2	190	3.46	-5.0	even	ok
	191	C38H110N21NaPS2	978.8324	-0.0	81.3	191	26.36	-5.0	even	ok
	192	C46H126NO10P2S2	978.8293	-3.3	81.4	192	2.97	-14.0	even	ok
	193	C59H113N5NaS2	978.8330	0.5	81.4	193	21.61	6.0	even	ok
	194	C48H2N2NaO14P2S2	978.8315	-1.0	81.8	195	16.31	50.0	even	ok
	195	C46HN2O19P2S	978.8364	4.0	81.9	196	1.31	49.0	even	ok
	196	C49HNaO17PS2	978.8285	-4.1	83.4	198	1.14	50.0	even	ok
	197	C39H113N17O4PS2	978.8335	1.0	83.6	199	14.89	-7.0	even	ok
	198	C40H112N15O7S2	978.8305	-2.0	83.7	200	7.88	-7.0	even	ok
	199	C38H114N19OP2S2	978.8365	4.1	83.7	201	1.09	-7.0	even	ok
	200	C39H101N23NaO4	978.8299	-2.6	83.8	202	3.85	1.0	even	ok
	201	C42H109N13NaO10	978.8313	-1.2	84.1	203	10.34	-5.0	even	ok
	202	C41H118N11NaO6PS2	978.8338	1.3	84.2	204	12.28	-11.0	even	ok
	203	C40H119N13NaO3P2S2	978.8368	4.4	84.2	205	0.76	-11.0	even	ok

Compound Spectrum SmartFormula Report

Meas. m/z	#	Ion Formula	m/z	err [ppm]	mSigma	# mSigma	Score	rdb	e⁻ Conf	N-Rule
	204	C42H117N9NaO9S2	978.8308	-1.7	84.4	206	9.37	-11.0	even	ok
	205	C37H96N29O2	978.8296	-2.9	84.5	207	2.90	5.0	even	ok
	206	C40H104N19O8	978.8310	-1.5	84.6	208	8.47	-1.0	even	ok
	207	C36H105N23NaO4S	978.8333	0.8	84.7	209	16.02	-4.0	even	ok
	208	C34H100N29O2S	978.8330	0.5	85.0	211	18.26	0.0	even	ok
	209	C43H112N9O14	978.8323	-0.2	85.2	212	17.10	-7.0	even	ok
	210	C60H116NO4S2	978.8340	1.6	85.3	213	9.95	4.0	even	ok
	211	C39H113N13NaO10S	978.8346	2.2	85.9	214	6.26	-10.0	even	ok
	212	C37H108N19O8S	978.8343	1.9	86.0	215	7.71	-6.0	even	ok
	213	C44H2N2NaO19P2S	978.8340	1.5	86.3	216	9.81	46.0	even	ok
	214	C41H110N15NaO7P	978.8342	1.8	86.4	217	6.53	-5.0	even	ok
	215	C38H102N25NaOP	978.8329	0.4	86.4	218	14.43	1.0	even	ok
	216	C42H121N7O10PS2	978.8348	2.4	86.7	219	5.06	-13.0	even	ok
	217	C42H116N5O18	978.8310	-1.5	86.7	220	9.77	-12.0	even	ok
	218	C44H118N5NaO13P	978.8356	3.2	86.9	221	2.03	-11.0	even	ok
	219	C39H105N21O5P	978.8340	1.5	87.1	222	7.70	-1.0	even	ok
	220	C43H120N5O13S2	978.8319	-0.6	87.1	223	15.90	-13.0	even	ok
	221	C42H122N3NaO14PS	978.8277	-4.8	87.3	224	0.37	-16.0	even	ok
	222	C42H113N11O11P	978.8353	2.9	87.3	225	2.60	-7.0	even	ok
	223	C44H126NNaO12PS2	978.8351	2.7	87.4	226	3.85	-17.0	even	ok
	224	C40H116N9O14S	978.8357	3.3	87.4	227	2.23	-12.0	even	ok
	225	C42H121N3NaO16S	978.8360	3.6	87.5	228	1.66	-16.0	even	ok
	226	C45HN4O16P2S2	978.8299	-2.7	87.6	229	3.96	49.0	even	ok
	227	C45H122NO15P2	978.8284	-4.1	88.2	230	0.68	-13.0	even	ok
	228	C45HNaO22PS	978.8310	-1.5	88.4	231	9.14	46.0	even	ok
	229	C41H123N5NaO11P2S	978.8307	-1.8	88.5	232	7.52	-16.0	even	ok
	230	C40H111N17NaO4P2	978.8372	4.9	88.9	233	0.27	-5.0	even	ok
	231	C61H112N5S2	978.8354	2.9	89.5	234	2.83	9.0	even	ok
	232	C36H108N21O5S2	978.8278	-4.8	89.7	235	0.37	-6.0	even	ok
	233	C38H106N23O2P2	978.8370	4.6	89.7	236	0.38	-1.0	even	ok
	234	C42H126NO15P2S	978.8318	-0.7	90.0	237	13.56	-18.0	even	ok
	235	C38H113N15NaO7S2	978.8281	-4.5	90.1	238	0.53	-10.0	even	ok
	236	C39H116N11O11S2	978.8292	-3.4	92.4	239	1.64	-12.0	even	ok
	237	C41H121N5NaO13S2	978.8295	-3.1	92.9	240	2.12	-16.0	even	ok
	238	C43HN4NaO18PS2	978.8357	3.3	93.7	241	1.67	46.0	even	ok
	239	C43HO25P2	978.8277	-4.9	94.9	244	0.24	45.0	even	ok
	240	C42H124NO17S2	978.8305	-2.0	95.4	245	4.77	-18.0	even	ok
	241	C44H5O20P2S2	978.8285	-4.0	95.4	246	0.71	44.0	even	ok
	242	C38H105N19NaO8	978.8286	-4.0	95.6	247	0.59	-4.0	even	ok
	243	C41H113N9NaO14	978.8299	-2.6	95.8	248	2.29	-10.0	even	ok
	244	C41HN4O21P2S	978.8324	-0.1	96.0	249	13.59	45.0	even	ok
	245	C36H100N25O6	978.8283	-4.3	96.3	250	0.41	0.0	even	ok
	246	C44H4O22PS2	978.8368	4.4	96.3	251	0.44	44.0	even	ok
	247	C39H108N15O12	978.8296	-2.9	96.4	252	1.74	-6.0	even	ok
	248	C41H120NO22	978.8297	-2.9	97.1	253	2.12	-17.0	even	ok
	249	C40H114N11NaO11P	978.8329	0.4	98.2	255	8.49	-10.0	even	ok

Compound Spectrum SmartFormula Report

Meas. m/z	#	Ion Formula	m/z	err [ppm]	mSigma	# mSigma	Score	rdb	e¯ Conf	N-Rule
	250	C37H106N21NaO5P	978.8316	-0.9	98.3	256	6.60	-4.0	even	ok
	251	C43H122NNaO17P	978.8343	1.8	98.5	257	3.77	-16.0	even	ok
	252	C38H109N17O9P	978.8326	0.2	98.8	258	9.32	-6.0	even	ok
	253	C41H117N7O15P	978.8340	1.5	99.0	259	4.46	-12.0	even	ok
	254	C35H101N27O3P	978.8313	-1.2	99.1	260	5.39	0.0	even	ok

Cmpd 14, 33.2 min

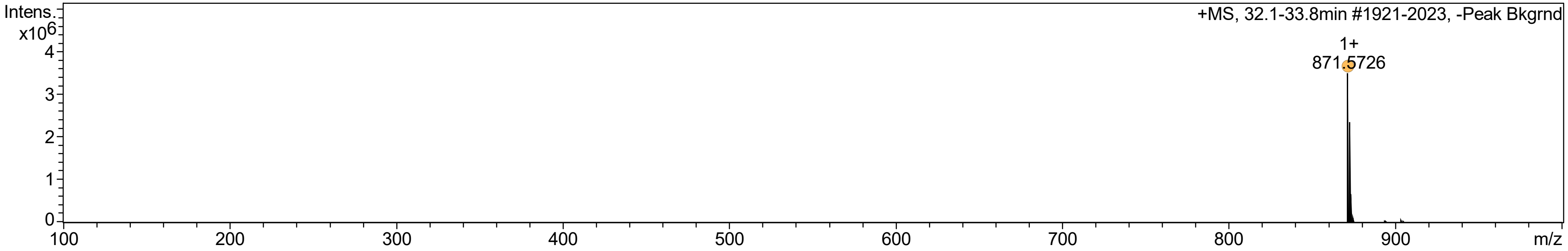

Meas. m/z	#	Ion Formula	m/z	err [ppm]	mSigma	# mSigma	Score	rdb	e¯ Conf	N-Rule
871.5726	1	C56H71N8O	871.5745	2.3	16.1	1	42.17	26.0	even	ok
	2	C60H76N2OP	871.5690	-4.1	16.7	2	9.57	25.0	even	ok
	3	C58H76N2NaO3	871.5748	2.6	17.4	3	32.94	22.0	even	ok
	4	C58H81O2P2	871.5706	-2.2	18.3	4	42.18	20.0	even	ok
	5	C54H72N8NaO	871.5721	-0.5	24.2	5	91.84	23.0	even	ok
	6	C55H75N4O5	871.5732	0.7	25.9	6	79.34	21.0	even	ok
	7	C56H81NaO4P	871.5765	4.5	27.1	7	5.19	17.0	even	ok
	8	C56H82NaO2P2	871.5682	-5.0	27.2	8	3.14	17.0	even	ok
	9	C54H76N6O2P	871.5762	4.2	27.3	9	7.09	21.0	even	ok
	10	C54H79O9	871.5719	-0.8	33.2	10	81.10	16.0	even	ok
	11	C55H81N2NaOPS	871.5699	-3.0	33.7	11	21.04	17.0	even	ok
	12	C57H76N4NaS	871.5683	-4.9	33.9	12	3.61	22.0	even	ok
	13	C57H80N2OPS	871.5723	-0.2	34.0	13	100.00	20.0	even	ok
	14	C55H85O2P2S	871.5740	1.7	35.2	14	49.49	15.0	even	ok
	15	C53H76N4NaO5	871.5708	-2.0	35.9	15	31.43	18.0	even	ok
	16	C51H71N10O3	871.5705	-2.3	36.5	16	25.11	22.0	even	ok
	17	C51H76N8NaOS	871.5755	3.4	37.5	17	13.97	18.0	even	ok
	18	C58H79O4S	871.5694	-3.7	37.8	18	10.87	20.0	even	ok
	19	C52H77N6NaO2P	871.5738	1.4	38.5	19	41.72	18.0	even	ok
	20	C51H81N6P2S	871.5713	-1.4	38.9	20	51.31	16.0	even	ok
	21	C59H75N4S	871.5707	-2.1	39.1	21	33.65	25.0	even	ok
	22	C53H80N2O6P	871.5749	2.6	39.1	22	19.07	16.0	even	ok
	23	C52H80N4O3PS	871.5683	-4.8	39.2	23	3.28	16.0	even	ok
	24	C50H72N12P	871.5735	1.1	39.5	24	47.90	22.0	even	ok

Compound Spectrum SmartFormula Report

Meas. m/z	#	Ion Formula	m/z	err [ppm]	mSigma	# mSigma	Score	rdb	e¯ Conf	N-Rule
	25	C53H86NaO2P2S	871.5716	-1.1	39.6	25	59.47	12.0	even	ok
	26	C52H79N4O5S	871.5766	4.6	40.3	26	4.12	16.0	even	ok
	27	C48H75N10O3S	871.5739	1.5	45.7	27	40.00	17.0	even	ok
	28	C50H80N4NaO5S	871.5742	1.8	46.1	28	32.91	13.0	even	ok
	29	C49H82N6NaP2S	871.5689	-4.2	46.4	29	5.33	13.0	even	ok
	30	C47H76N12PS	871.5769	5.0	46.5	30	2.37	17.0	even	ok
	31	C52H80NaO9	871.5695	-3.6	47.7	31	7.23	13.0	even	ok
	32	C50H85N2O4P2S	871.5700	-3.0	48.1	32	14.59	11.0	even	ok
	33	C51H83O9S	871.5752	3.1	48.4	33	13.19	11.0	even	ok
	34	C50H75N6O7	871.5692	-3.9	48.5	34	5.32	17.0	even	ok
	35	C51H81N2NaO6P	871.5724	-0.1	50.5	35	52.48	13.0	even	ok
	36	C49H76N8O4P	871.5722	-0.4	51.5	36	45.06	17.0	even	ok
	37	C48H73N12NaP	871.5711	-1.7	51.5	37	24.83	19.0	even	ok
	38	C54H80N4NaS2	871.5717	-1.0	53.3	38	41.14	17.0	even	ok
	39	C52H85N2NaOPS2	871.5733	0.9	53.6	39	43.84	12.0	even	ok
	40	C50H82N4NaO3P2	871.5754	3.3	53.6	40	7.43	13.0	even	ok
	41	C54H84N2OPS2	871.5757	3.6	53.8	41	7.02	15.0	even	ok
	42	C46H76N10NaO3S	871.5715	-1.2	53.8	42	36.54	14.0	even	ok
	43	C51H85O7P2	871.5765	4.5	53.8	43	2.38	11.0	even	ok
	44	C44H71N16OS	871.5712	-1.6	53.9	44	30.67	18.0	even	ok
	45	C51H79N6O2S2	871.5700	-2.9	54.3	45	12.75	16.0	even	ok
	46	C48H77N10OP2	871.5752	3.0	54.8	46	9.25	17.0	even	ok
	47	C45H77N12NaPS	871.5745	2.2	55.3	47	19.93	14.0	even	ok
	48	C47H79N6O7S	871.5725	-0.0	55.4	48	58.64	12.0	even	ok
	49	C49H84NaO9S	871.5728	0.3	55.5	49	52.22	8.0	even	ok
	50	C53H84NaO4S2	871.5703	-2.6	56.1	50	15.23	12.0	even	ok
	51	C48H85N2NaO6PS	871.5758	3.7	56.5	51	5.83	8.0	even	ok
	52	C46H80N8O4PS	871.5755	3.4	56.6	52	7.68	12.0	even	ok
	53	C56H79N4S2	871.5741	1.7	56.9	53	25.15	20.0	even	ok
	54	C48H85N6P2S2	871.5747	2.4	57.1	54	15.90	11.0	even	ok
	55	C55H83O4S2	871.5727	0.2	57.2	55	51.51	15.0	even	ok
	56	C50H90NaO2P2S2	871.5750	2.8	57.8	56	12.36	7.0	even	ok
	57	C49H84N4O3PS2	871.5717	-1.0	57.8	57	36.28	11.0	even	ok
	58	C50H83N2O6S2	871.5687	-4.4	59.4	58	2.81	11.0	even	ok
	59	C45H80N10OPS2	871.5690	-4.1	62.0	59	3.62	12.0	even	ok
	60	C47H85N4NaO3PS2	871.5693	-3.7	62.4	60	4.79	8.0	even	ok
	61	C46H86N6NaP2S2	871.5723	-0.3	62.4	61	41.44	8.0	even	ok
	62	C42H72N16NaOS	871.5688	-4.3	63.2	62	2.71	15.0	even	ok
	63	C47H77N8NaO4P	871.5698	-3.2	63.4	63	5.84	14.0	even	ok
	64	C48H80N4O8P	871.5708	-2.0	63.4	64	13.95	12.0	even	ok
	65	C44H68N18Na	871.5767	4.7	63.8	65	1.43	20.0	even	ok
	66	C45H80N6NaO7S	871.5701	-2.8	64.0	66	10.01	9.0	even	ok
	67	C43H75N12O5S	871.5699	-3.1	64.3	67	7.77	13.0	even	ok
	68	C45H72N14O2P	871.5695	-3.5	64.6	68	4.29	18.0	even	ok
	69	C47H89N2O4P2S2	871.5734	0.9	64.6	69	29.71	6.0	even	ok
	70	C48H88O7PS2	871.5704	-2.5	65.1	70	11.55	6.0	even	ok

Compound Spectrum SmartFormula Report

Meas. m/z	#	Ion Formula	m/z	err [ppm]	mSigma	# mSigma	Score	rdb	e⁻ Conf	N-Rule
	71	C46H83N2O11S	871.5712	-1.5	65.4	71	21.02	7.0	even	ok
	72	C49H86NaO7P2	871.5741	1.8	65.6	72	14.63	8.0	even	ok
	73	C44H81N8NaO4PS	871.5731	0.7	65.7	73	31.97	9.0	even	ok
	74	C42H76N14O2PS	871.5729	0.3	66.1	74	35.93	13.0	even	ok
	75	C47H81N6O5P2	871.5738	1.5	66.7	75	16.85	12.0	even	ok
	76	C45H84N4O8PS	871.5742	1.9	66.8	76	16.32	7.0	even	ok
	77	C46H78N10NaOP2	871.5728	0.2	66.9	77	29.23	14.0	even	ok
	78	C43H82N10NaOP2S	871.5761	4.1	67.7	78	2.86	9.0	even	ok
	79	C41H75N16OS2	871.5746	2.3	68.6	79	11.68	13.0	even	ok
	80	C43H80N10NaO3S2	871.5748	2.6	69.0	80	9.22	9.0	even	ok
	81	C43H85N8O2P2S2	871.5707	-2.2	71.0	81	11.78	7.0	even	ok
	82	C44H83N6O7S2	871.5759	3.9	71.0	82	3.16	7.0	even	ok
	83	C45H90N2NaO4P2S2	871.5709	-1.8	71.1	83	14.33	3.0	even	ok
	84	C46H88NaO9S2	871.5762	4.2	71.6	84	2.29	3.0	even	ok
	85	C44H84N2NaO11S	871.5688	-4.3	74.3	85	1.83	4.0	even	ok
	86	C46H80N4NaO10	871.5767	4.7	74.4	86	0.96	9.0	even	ok
	87	C42H79N8O9S	871.5685	-4.6	74.7	87	1.30	8.0	even	ok
	88	C46H81N4NaO8P	871.5684	-4.7	75.2	88	0.90	9.0	even	ok
	89	C47H84O12P	871.5695	-3.5	75.3	89	2.91	7.0	even	ok
	90	C44H75N10O8	871.5764	4.4	75.4	90	1.27	13.0	even	ok
	91	C43H72N14NaO4	871.5753	3.2	75.6	91	3.82	15.0	even	ok
	92	C40H77N14NaO2PS	871.5704	-2.4	76.0	92	8.25	10.0	even	ok
	93	C43H85N4NaO8PS	871.5718	-0.9	76.2	93	19.70	4.0	even	ok
	94	C39H76N16NaOS2	871.5722	-0.4	76.3	94	23.57	10.0	even	ok
	95	C44H76N10O6P	871.5681	-5.1	76.4	95	0.61	13.0	even	ok
	96	C38H72N20PS	871.5702	-2.7	76.6	96	6.40	14.0	even	ok
	97	C41H80N10O6PS	871.5715	-1.2	76.7	97	16.57	8.0	even	ok
	98	C41H67N20O2	871.5750	2.9	76.9	98	4.66	19.0	even	ok
	99	C44H88O12PS	871.5729	0.4	77.2	99	23.59	2.0	even	ok
	100	C40H79N12O5S2	871.5732	0.8	78.1	100	19.07	8.0	even	ok
	101	C42H86N6NaO5P2S	871.5748	2.6	78.3	101	6.81	4.0	even	ok
	102	C42H84N6NaO7S2	871.5735	1.1	78.3	102	16.26	4.0	even	ok
	103	C46H85N2O9P2	871.5725	-0.1	78.5	103	19.82	7.0	even	ok
	104	C45H82N6NaO5P2	871.5714	-1.3	78.7	104	11.55	9.0	even	ok
	105	C41H86N8NaO2P2S2	871.5683	-4.9	78.8	105	0.80	4.0	even	ok
	106	C40H81N12O3P2S	871.5745	2.2	78.9	106	8.28	8.0	even	ok
	107	C39H80N14O2PS2	871.5762	4.2	79.0	107	1.67	8.0	even	ok
	108	C41H85N8NaO4PS2	871.5765	4.5	79.1	108	1.21	4.0	even	ok
	109	C43H89N2O9P2S	871.5759	3.8	79.1	109	2.47	2.0	even	ok
	110	C43H77N12O3P2	871.5711	-1.6	79.9	110	9.22	13.0	even	ok
	111	C43H87N2O11S2	871.5746	2.3	80.1	111	7.45	2.0	even	ok
	112	C42H89N4O6P2S2	871.5693	-3.7	80.5	112	2.51	2.0	even	ok
	113	C36H72N20NaO2S	871.5760	4.0	86.2	113	1.57	11.0	even	ok
	114	C38H80N12NaO5S2	871.5708	-2.0	86.2	114	7.20	5.0	even	ok
	115	C45H84NaO14	871.5753	3.2	86.2	115	2.46	4.0	even	ok
	116	C36H75N18O3S2	871.5705	-2.3	86.3	116	5.85	9.0	even	ok

Compound Spectrum SmartFormula Report

Meas. m/z	#	Ion Formula	m/z	err [ppm]	mSigma	# mSigma	Score	rdb	e¯ Conf	N-Rule
	117	C39H81N10NaO6PS	871.5691	-4.0	86.5	117	1.56	5.0	even	ok
	118	C42H89NaO12PS	871.5705	-2.4	86.7	118	5.36	-1.0	even	ok
	119	C37H76N16O4PS	871.5688	-4.3	87.2	119	1.11	9.0	even	ok
	120	C40H84N6O10PS	871.5702	-2.7	87.2	120	4.18	3.0	even	ok
	121	C43H79N6O12	871.5750	2.9	87.3	121	3.01	8.0	even	ok
	122	C42H76N10NaO8	871.5740	1.6	87.3	122	6.74	10.0	even	ok
	123	C37H81N14NaO2PS2	871.5738	1.5	87.5	123	9.26	5.0	even	ok
	124	C35H76N20PS2	871.5735	1.1	87.7	124	10.85	9.0	even	ok
	125	C41H88N2NaO11S2	871.5722	-0.4	87.7	125	14.80	-1.0	even	ok
	126	C39H83N8O9S2	871.5719	-0.8	87.8	126	12.86	3.0	even	ok
	127	C40H71N16O6	871.5737	1.3	88.5	127	7.61	14.0	even	ok
	128	C41H90N2NaO9P2S	871.5734	1.0	88.8	128	10.86	-1.0	even	ok
	129	C39H68N20NaO2	871.5726	0.1	88.9	129	12.80	16.0	even	ok
	130	C40H89N4NaO8PS2	871.5752	3.0	88.9	130	3.16	-1.0	even	ok
	131	C38H82N12NaO3P2S	871.5721	-0.5	88.9	131	13.55	5.0	even	ok
	132	C38H84N10O6PS2	871.5749	2.7	89.0	132	4.02	3.0	even	ok
	133	C39H85N8O7P2S	871.5732	0.7	89.4	133	12.25	3.0	even	ok
	134	C37H63N26	871.5724	-0.2	90.3	134	11.45	20.0	even	ok
	135	C44H86N2NaO9P2	871.5701	-2.8	90.4	135	2.67	4.0	even	ok
	136	C41H92O12PS2	871.5762	4.2	90.4	136	1.02	-3.0	even	ok
	137	C41H77N12NaO5P	871.5770	5.1	90.8	137	0.33	10.0	even	ok
	138	C42H81N8O7P2	871.5698	-3.2	91.6	138	1.98	8.0	even	ok
	139	C41H78N12NaO3P2	871.5687	-4.4	91.9	139	0.65	10.0	even	ok
	140	C39H72N18O3P	871.5767	4.7	92.1	140	0.44	14.0	even	ok
	141	C39H73N18OP2	871.5684	-4.7	93.3	141	0.44	14.0	even	ok
	142	C34H76N18NaO3S2	871.5681	-5.1	95.2	142	0.34	6.0	even	ok
	143	C37H84N8NaO9S2	871.5695	-3.5	96.3	143	1.49	0.0	even	ok
	144	C35H79N14O7S2	871.5692	-3.8	96.4	144	1.12	4.0	even	ok
	145	C38H84N6NaO12S	871.5760	4.0	96.6	145	0.98	0.0	even	ok
	146	C35H76N16NaO6S	871.5747	2.4	96.7	146	3.38	6.0	even	ok
	147	C33H77N20NaPS2	871.5711	-1.6	96.9	147	5.52	6.0	even	ok
	148	C34H67N26S	871.5757	3.6	97.2	148	1.03	15.0	even	ok
	149	C36H79N12O10S	871.5757	3.6	97.2	149	1.27	4.0	even	ok
	150	C33H71N22O4S	871.5744	2.1	97.5	150	4.04	10.0	even	ok
	151	C39H88N2O14PS	871.5688	-4.3	97.7	151	0.70	-2.0	even	ok
	152	C36H85N10NaO6PS2	871.5725	-0.1	97.7	152	10.77	0.0	even	ok
	153	C38H87N4O13S2	871.5706	-2.3	97.8	153	3.53	-2.0	even	ok
	154	C34H80N16O4PS2	871.5722	-0.4	98.0	154	9.43	4.0	even	ok
	155	C39H93NaO12PS2	871.5738	1.5	98.8	155	5.54	-6.0	even	ok
	156	C42H83N2O16	871.5737	1.3	99.0	156	4.71	3.0	even	ok
	157	C41H80N6NaO12	871.5726	0.1	99.0	157	8.07	5.0	even	ok
	158	C37H88N6O10PS2	871.5735	1.1	99.1	158	6.45	-2.0	even	ok
	159	C35H86N12NaO3P2S2	871.5755	3.3	99.4	159	1.49	0.0	even	ok
	160	C37H86N8NaO7P2S	871.5708	-2.1	99.5	160	3.79	0.0	even	ok
	161	C33H81N18OP2S2	871.5752	3.0	99.7	161	1.89	4.0	even	ok
	162	C38H89N4O11P2S	871.5718	-0.8	100.0	162	7.17	-2.0	even	ok

Compound Spectrum SmartFormula Report

Cmpd 15, 34.4 min

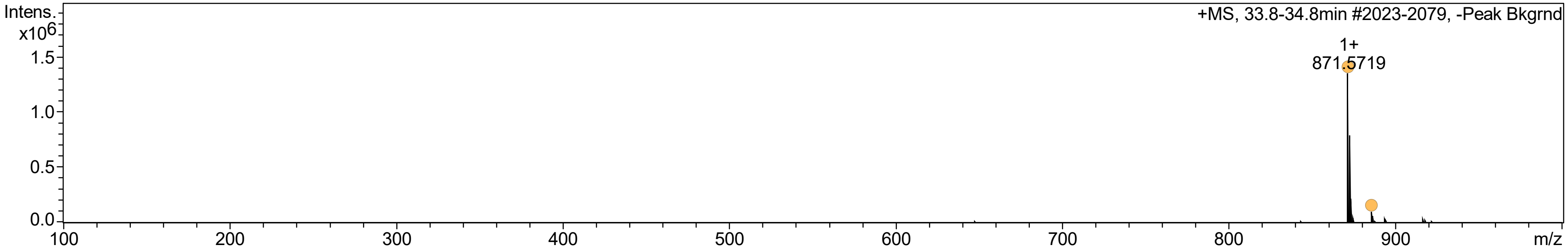

Meas. m/z	#	Ion Formula	m/z	err [ppm]	mSigma	# mSigma	Score	rdb	e¯ Conf	N-Rule
871.5719	1	C47H67N16O	871.5678	-4.6	4.3	1	4.43	23.0	even	ok
	2	C49H72N10NaO3	871.5681	-4.3	5.5	2	6.01	19.0	even	ok
	3	C48H73N12NaP	871.5711	-0.9	5.7	3	69.54	19.0	even	ok
	4	C49H76N8O4P	871.5722	0.3	7.5	4	84.23	17.0	even	ok
	5	C50H72N12P	871.5735	1.9	7.9	5	39.20	22.0	even	ok
	6	C50H82N4NaO3P2	871.5754	4.1	8.5	6	6.95	13.0	even	ok
	7	C51H81N2NaO6P	871.5724	0.7	8.7	7	72.27	13.0	even	ok
	8	C48H77N10OP2	871.5752	3.8	9.0	8	9.27	17.0	even	ok
	9	C50H75N6O7	871.5692	-3.1	9.5	9	16.59	17.0	even	ok
	10	C52H77N6NaO2P	871.5738	2.2	10.2	10	30.69	18.0	even	ok
	11	C52H80NaO9	871.5695	-2.8	11.7	11	20.32	13.0	even	ok
	12	C51H71N10O3	871.5705	-1.5	13.1	12	43.21	22.0	even	ok
	13	C53H80N2O6P	871.5749	3.4	13.4	13	11.57	16.0	even	ok
	14	C53H76N4NaO5	871.5708	-1.2	15.6	14	48.76	18.0	even	ok
	15	C54H79O9	871.5719	-0.0	16.9	15	100.00	16.0	even	ok
	16	C49H79N2O11	871.5678	-4.6	17.3	16	3.51	12.0	even	ok
	17	C47H77N8NaO4P	871.5698	-2.4	17.6	17	23.16	14.0	even	ok
	18	C48H80N4O8P	871.5708	-1.2	18.5	18	46.94	12.0	even	ok
	19	C45H72N14O2P	871.5695	-2.7	18.7	19	18.06	18.0	even	ok
	20	C49H86NaO7P2	871.5741	2.6	20.1	20	19.68	8.0	even	ok
	21	C54H77N6P2	871.5679	-4.5	20.5	21	3.73	21.0	even	ok
	22	C47H81N6O5P2	871.5738	2.2	20.9	22	24.09	12.0	even	ok
	23	C46H78N10NaOP2	871.5728	1.0	21.0	23	48.19	14.0	even	ok
	24	C54H76N6O2P	871.5762	5.0	22.4	24	2.17	21.0	even	ok
	25	C56H82NaO2P2	871.5682	-4.2	22.6	25	4.90	17.0	even	ok
	26	C45H77N12NaPS	871.5745	3.0	22.7	26	16.98	14.0	even	ok
	27	C49H82N6NaP2S	871.5689	-3.4	22.8	27	12.38	13.0	even	ok
	28	C44H71N16OS	871.5712	-0.8	23.2	28	64.72	18.0	even	ok
	29	C46H76N10NaO3S	871.5715	-0.4	24.8	29	71.45	14.0	even	ok
	30	C54H72N8NaO	871.5721	0.3	25.4	30	59.39	23.0	even	ok
	31	C48H86N2NaO4P2S	871.5676	-4.9	26.0	31	2.63	8.0	even	ok
	32	C46H80N8O4PS	871.5755	4.2	26.1	32	5.41	12.0	even	ok

Compound Spectrum SmartFormula Report

Meas. m/z	#	Ion Formula	m/z	err [ppm]	mSigma	# mSigma	Score	rdb	e¯ Conf	N-Rule
	33	C50H85N2O4P2S	871.5700	-2.2	26.6	33	28.06	11.0	even	ok
	34	C48H75N10O3S	871.5739	2.3	26.6	34	25.21	17.0	even	ok
	35	C42H72N16NaOS	871.5688	-3.5	27.2	35	9.97	15.0	even	ok
	36	C55H75N4O5	871.5732	1.5	27.5	36	31.92	21.0	even	ok
	37	C48H85N2NaO6PS	871.5758	4.5	27.7	37	3.78	8.0	even	ok
	38	C51H81N6P2S	871.5713	-0.6	28.0	38	61.47	16.0	even	ok
	39	C47H79N6O7S	871.5725	0.8	28.5	39	56.67	12.0	even	ok
	40	C50H80N4NaO5S	871.5742	2.6	28.9	40	19.11	13.0	even	ok
	41	C46H81N4NaO8P	871.5684	-3.9	29.5	41	5.17	9.0	even	ok
	42	C42H76N14O2PS	871.5729	1.1	29.6	42	46.79	13.0	even	ok
	43	C43H72N14NaO4	871.5753	4.0	29.6	43	5.05	15.0	even	ok
	44	C43H75N12O5S	871.5699	-2.3	29.9	44	23.76	13.0	even	ok
	45	C47H84O12P	871.5695	-2.7	30.2	45	14.03	7.0	even	ok
	46	C44H81N8NaO4PS	871.5731	1.5	30.2	46	39.02	9.0	even	ok
	47	C43H82N10NaOP2S	871.5761	4.9	30.3	47	2.45	9.0	even	ok
	48	C49H84NaO9S	871.5728	1.1	30.3	48	46.64	8.0	even	ok
	49	C53H86NaO2P2S	871.5716	-0.3	30.5	49	65.99	12.0	even	ok
	50	C44H76N10O6P	871.5681	-4.3	30.5	50	3.72	13.0	even	ok
	51	C45H80N6NaO7S	871.5701	-2.0	30.8	51	28.49	9.0	even	ok
	52	C41H67N20O2	871.5750	3.6	31.0	52	6.54	19.0	even	ok
	53	C51H76N8NaOS	871.5755	4.2	31.6	53	4.93	18.0	even	ok
	54	C45H82N6NaO5P2	871.5714	-0.5	32.7	54	45.95	9.0	even	ok
	55	C51H83O9S	871.5752	3.9	32.8	55	6.40	11.0	even	ok
	56	C52H80N4O3PS	871.5683	-4.1	32.8	56	5.38	16.0	even	ok
	57	C46H85N2O9P2	871.5725	0.7	32.8	57	42.12	7.0	even	ok
	58	C45H84N4O8PS	871.5742	2.7	32.9	58	16.84	7.0	even	ok
	59	C46H83N2O11S	871.5712	-0.7	33.9	59	50.42	7.0	even	ok
	60	C43H77N12O3P2	871.5711	-0.8	34.0	60	38.72	13.0	even	ok
	61	C58H81O2P2	871.5706	-1.4	35.3	61	28.14	20.0	even	ok
	62	C41H76N12NaO5S	871.5675	-5.1	36.3	62	1.76	10.0	even	ok
	63	C40H77N14NaO2PS	871.5704	-1.6	37.2	63	29.76	10.0	even	ok
	64	C38H72N20PS	871.5702	-1.9	37.5	64	24.49	14.0	even	ok
	65	C56H71N8O	871.5745	3.1	38.3	65	8.76	26.0	even	ok
	66	C42H79N8O9S	871.5685	-3.8	38.4	66	5.72	8.0	even	ok
	67	C55H85O2P2S	871.5740	2.5	38.8	67	16.91	15.0	even	ok
	68	C41H80N10O6PS	871.5715	-0.4	38.9	68	51.16	8.0	even	ok
	69	C44H84N2NaO11S	871.5688	-3.5	39.0	69	7.49	4.0	even	ok
	70	C43H85N4NaO8PS	871.5718	-0.1	39.2	70	57.34	4.0	even	ok
	71	C42H86N6NaO5P2S	871.5748	3.4	40.0	71	8.29	4.0	even	ok
	72	C40H81N12O3P2S	871.5745	3.0	40.0	72	10.73	8.0	even	ok
	73	C58H76N2NaO3	871.5748	3.4	40.3	73	6.38	22.0	even	ok
	74	C55H81N2NaOPS	871.5699	-2.2	40.7	74	19.10	17.0	even	ok
	75	C45H84NaO14	871.5753	4.0	40.8	75	3.75	4.0	even	ok
	76	C44H88O12PS	871.5729	1.1	41.4	76	34.22	2.0	even	ok
	77	C42H76N10NaO8	871.5740	2.4	41.4	77	12.84	10.0	even	ok
	78	C43H79N6O12	871.5750	3.7	41.6	78	4.90	8.0	even	ok

Compound Spectrum SmartFormula Report

Meas. m/z	#	Ion Formula	m/z	err [ppm]	mSigma	# mSigma	Score	rdb	e¯ Conf	N-Rule
	79	C43H89N2O9P2S	871.5759	4.6	41.8	79	2.52	2.0	even	ok
	80	C40H71N16O6	871.5737	2.1	42.6	80	15.35	14.0	even	ok
	81	C46H86N6NaP2S2	871.5723	0.5	42.6	81	44.65	8.0	even	ok
	82	C39H68N20NaO2	871.5726	0.9	43.1	82	29.64	16.0	even	ok
	83	C41H75N16OS2	871.5746	3.1	43.5	83	9.19	13.0	even	ok
	84	C45H80N10OPS2	871.5690	-3.3	43.9	84	7.97	12.0	even	ok
	85	C44H86N2NaO9P2	871.5701	-2.0	44.5	85	15.14	4.0	even	ok
	86	C37H63N26	871.5724	0.6	44.6	86	32.70	20.0	even	ok
	87	C43H85N8O2P2S2	871.5707	-1.4	44.7	87	27.78	7.0	even	ok
	88	C43H80N10NaO3S2	871.5748	3.4	45.2	88	6.74	9.0	even	ok
	89	C48H85N6P2S2	871.5747	3.2	45.5	89	7.77	11.0	even	ok
	90	C42H81N8O7P2	871.5698	-2.4	45.6	90	11.88	8.0	even	ok
	91	C47H85N4NaO3PS2	871.5693	-2.9	46.0	91	9.68	8.0	even	ok
	92	C39H76N16NaOS2	871.5722	0.3	46.0	92	42.78	10.0	even	ok
	93	C41H78N12NaO3P2	871.5687	-3.6	46.0	93	4.56	10.0	even	ok
	94	C45H90N2NaO4P2S2	871.5709	-1.1	46.3	94	31.22	3.0	even	ok
	95	C47H89N2O4P2S2	871.5734	1.7	46.4	95	21.93	6.0	even	ok
	96	C36H72N20NaO2S	871.5760	4.8	46.5	96	1.84	11.0	even	ok
	97	C39H81N14P2S2	871.5680	-4.5	46.8	97	2.49	8.0	even	ok
	98	C44H84N6O5PS2	871.5677	-4.8	47.0	98	1.72	7.0	even	ok
	99	C39H81N10NaO6PS	871.5691	-3.2	47.4	99	7.87	5.0	even	ok
	100	C39H73N18OP2	871.5684	-3.9	47.5	100	3.28	14.0	even	ok
	101	C37H76N16O4PS	871.5688	-3.5	47.8	101	5.98	9.0	even	ok
	102	C41H86N8NaO2P2S2	871.5683	-4.1	47.8	102	3.31	4.0	even	ok
	103	C50H90NaO2P2S2	871.5750	3.6	47.8	103	5.55	7.0	even	ok
	104	C39H80N14O2PS2	871.5762	5.0	47.9	104	1.34	8.0	even	ok
	105	C46H89NaO7PS2	871.5680	-4.5	48.7	105	2.28	3.0	even	ok
	106	C44H83N6O7S2	871.5759	4.7	48.8	106	1.91	7.0	even	ok
	107	C40H84N6O10PS	871.5702	-1.9	48.9	107	17.87	3.0	even	ok
	108	C42H89NaO12PS	871.5705	-1.6	48.9	108	21.54	-1.0	even	ok
	109	C38H82N12NaO3P2S	871.5721	0.3	49.1	109	40.13	5.0	even	ok
	110	C40H79N12O5S2	871.5732	1.6	49.1	110	21.85	8.0	even	ok
	111	C49H84N4O3PS2	871.5717	-0.2	49.7	111	40.74	11.0	even	ok
	112	C48H88O7PS2	871.5704	-1.7	49.8	112	19.65	6.0	even	ok
	113	C41H90N2NaO9P2S	871.5734	1.8	50.1	113	18.42	-1.0	even	ok
	114	C49H80N6NaO2S2	871.5676	-4.8	50.2	114	1.49	13.0	even	ok
	115	C39H85N8O7P2S	871.5732	1.5	50.2	115	22.00	3.0	even	ok
	116	C42H84N6NaO7S2	871.5735	1.9	50.4	116	17.43	4.0	even	ok
	117	C46H88NaO9S2	871.5762	5.0	50.6	117	1.28	3.0	even	ok
	118	C42H89N4O6P2S2	871.5693	-2.9	50.8	118	8.64	2.0	even	ok
	119	C57H80N2OPS	871.5723	0.6	50.9	119	33.92	20.0	even	ok
	120	C60H76N2OP	871.5690	-3.3	51.5	120	4.93	25.0	even	ok
	121	C36H73N20NaPS	871.5678	-4.7	52.2	121	1.30	11.0	even	ok
	122	C34H67N26S	871.5757	4.4	52.4	122	1.72	15.0	even	ok
	123	C35H76N20PS2	871.5735	1.9	52.7	123	15.95	9.0	even	ok
	124	C36H75N18O3S2	871.5705	-1.5	52.8	124	20.22	9.0	even	ok

Compound Spectrum SmartFormula Report

Meas. m/z	#	Ion Formula	m/z	err [ppm]	mSigma	# mSigma	Score	rdb	e¯ Conf	N-Rule
	125	C41H80N6NaO12	871.5726	0.9	53.2	125	21.79	5.0	even	ok
	126	C57H76N4NaS	871.5683	-4.1	53.2	126	2.90	22.0	even	ok
	127	C37H81N14NaO2PS2	871.5738	2.2	53.3	127	12.79	5.0	even	ok
	128	C42H83N2O16	871.5737	2.1	53.4	128	11.03	3.0	even	ok
	129	C38H80N12NaO5S2	871.5708	-1.2	53.6	129	23.36	5.0	even	ok
	130	C43H87N2O11S2	871.5746	3.1	53.7	130	6.69	2.0	even	ok
	131	C50H83N2O6S2	871.5687	-3.6	54.0	131	4.37	11.0	even	ok
	132	C39H75N12O10	871.5724	0.6	54.3	132	24.25	9.0	even	ok
	133	C52H85N2NaOPS2	871.5733	1.7	54.5	133	17.53	12.0	even	ok
	134	C38H72N16NaO6	871.5713	-0.6	54.5	134	23.32	11.0	even	ok
	135	C36H77N18OP2S	871.5718	-0.0	55.4	135	28.67	9.0	even	ok
	136	C38H84N10O6PS2	871.5749	3.5	55.8	136	4.69	3.0	even	ok
	137	C36H67N22O4	871.5710	-1.0	55.9	137	19.31	15.0	even	ok
	138	C58H79O4S	871.5694	-2.9	56.0	138	7.55	20.0	even	ok
	139	C39H83N8O9S2	871.5719	0.0	56.3	139	34.87	3.0	even	ok
	140	C40H81N8NaO9P	871.5756	4.3	56.5	140	1.67	5.0	even	ok
	141	C35H64N26Na	871.5699	-2.2	56.6	141	9.50	17.0	even	ok
	142	C40H89N4NaO8PS2	871.5752	3.8	56.6	142	3.45	-1.0	even	ok
	143	C51H79N6O2S2	871.5700	-2.1	56.8	143	12.66	16.0	even	ok
	144	C35H76N16NaO6S	871.5747	3.2	57.0	144	5.57	6.0	even	ok
	145	C41H85N4O11P2	871.5685	-3.9	57.3	145	2.44	3.0	even	ok
	146	C41H88N2NaO11S2	871.5722	0.4	57.3	146	29.98	-1.0	even	ok
	147	C33H71N22O4S	871.5744	2.9	57.4	147	7.05	10.0	even	ok
	148	C38H84N6NaO12S	871.5760	4.8	57.7	148	1.28	0.0	even	ok
	149	C38H76N14O7P	871.5754	4.0	57.7	149	2.18	9.0	even	ok
	150	C38H85N6NaO10PS	871.5678	-4.7	57.8	150	1.38	0.0	even	ok
	151	C36H79N12O10S	871.5757	4.4	57.9	151	1.77	4.0	even	ok
	152	C36H80N12O8PS	871.5675	-5.0	58.1	152	0.97	4.0	even	ok
	153	C37H73N18NaO3P	871.5743	2.8	58.2	153	5.99	11.0	even	ok
	154	C41H93O10P2S2	871.5680	-4.4	58.4	154	1.75	-3.0	even	ok
	155	C39H88N2O14PS	871.5688	-3.5	59.0	155	4.23	-2.0	even	ok
	156	C32H71N24OS2	871.5679	-4.6	59.1	156	1.47	10.0	even	ok
	157	C53H84NaO4S2	871.5703	-1.8	59.3	157	14.17	12.0	even	ok
	158	C41H92O12PS2	871.5762	5.0	59.4	158	0.92	-3.0	even	ok
	159	C34H76N18NaO3S2	871.5681	-4.3	59.4	159	2.01	6.0	even	ok
	160	C37H86N8NaO7P2S	871.5708	-1.3	59.6	160	18.50	0.0	even	ok
	161	C35H68N24OP	871.5740	2.5	59.7	161	7.17	15.0	even	ok
	162	C33H77N20NaPS2	871.5711	-0.8	60.0	162	22.39	6.0	even	ok
	163	C35H81N14O5P2S	871.5705	-1.6	60.2	163	15.28	4.0	even	ok
	164	C38H89N4O11P2S	871.5718	-0.0	60.6	164	30.30	-2.0	even	ok
	165	C35H79N14O7S2	871.5692	-3.0	61.6	165	5.48	4.0	even	ok
	166	C34H80N16O4PS2	871.5722	0.4	62.0	166	25.33	4.0	even	ok
	167	C37H84N8NaO9S2	871.5695	-2.7	62.1	167	6.86	0.0	even	ok
	168	C36H85N10NaO6PS2	871.5725	0.7	62.3	168	21.89	0.0	even	ok
	169	C54H84N2OPS2	871.5757	4.4	62.5	169	1.54	15.0	even	ok
	170	C33H81N18OP2S2	871.5752	3.8	62.7	170	2.74	4.0	even	ok

Compound Spectrum SmartFormula Report

Meas. m/z	#	Ion Formula	m/z	err [ppm]	mSigma	# mSigma	Score	rdb	e¯ Conf	N-Rule
	171	C35H86N12NaO3P2S2	871.5755	4.1	62.9	171	2.02	0.0	even	ok
	172	C32H68N26NaS	871.5733	1.7	63.4	172	10.40	12.0	even	ok
	173	C54H80N4NaS2	871.5717	-0.2	64.1	173	25.03	17.0	even	ok
	174	C59H75N4S	871.5707	-1.3	64.3	174	15.15	25.0	even	ok
	175	C37H88N6O10PS2	871.5735	1.9	64.6	175	10.67	-2.0	even	ok
	176	C38H87N4O13S2	871.5706	-1.5	64.6	176	13.75	-2.0	even	ok
	177	C40H84N2NaO16	871.5713	-0.6	64.9	177	16.52	0.0	even	ok
	178	C39H93NaO12PS2	871.5738	2.3	65.1	178	8.54	-6.0	even	ok
	179	C38H79N8O14	871.5710	-1.0	65.9	179	13.79	4.0	even	ok
	180	C37H76N12NaO10	871.5700	-2.2	66.0	180	6.93	6.0	even	ok
	181	C34H78N18NaOP2S	871.5694	-2.8	66.4	181	4.43	6.0	even	ok
	182	C55H83O4S2	871.5727	1.0	67.2	182	16.19	15.0	even	ok
	183	C35H71N18O8	871.5697	-2.5	67.3	183	5.34	10.0	even	ok
	184	C34H80N12NaO10S	871.5733	1.7	67.4	184	11.21	1.0	even	ok
	185	C34H68N22NaO4	871.5686	-3.7	67.8	185	1.99	12.0	even	ok
	186	C32H75N18O8S	871.5730	1.4	67.9	186	13.10	5.0	even	ok
	187	C37H88N2NaO16S	871.5747	3.2	68.0	187	3.76	-5.0	even	ok
	188	C39H85N4NaO13P	871.5743	2.8	68.1	188	4.21	0.0	even	ok
	189	C40H88O17P	871.5754	4.0	68.1	189	1.50	-2.0	even	ok
	190	C35H83N8O14S	871.5744	2.9	68.3	190	4.79	-1.0	even	ok
	191	C32H63N28O2	871.5683	-4.1	69.2	191	1.40	16.0	even	ok
	192	C37H80N10O11P	871.5740	2.5	69.2	192	5.09	4.0	even	ok
	193	C36H77N14NaO7P	871.5729	1.2	69.6	193	10.49	6.0	even	ok
	194	C30H76N22O2PS2	871.5695	-2.7	69.9	194	5.32	5.0	even	ok
	195	C32H81N16NaO4PS2	871.5698	-2.4	69.9	195	6.65	1.0	even	ok
	196	C36H90N4NaO11P2S	871.5694	-2.8	70.1	196	4.89	-5.0	even	ok
	197	C34H85N10O9P2S	871.5691	-3.1	70.6	197	3.75	-1.0	even	ok
	198	C34H72N20O5P	871.5727	0.9	70.9	198	11.67	10.0	even	ok
	199	C34H83N10O11S2	871.5679	-4.6	70.9	199	0.98	-1.0	even	ok
	200	C37H93O15P2S	871.5705	-1.6	71.0	200	10.45	-7.0	even	ok
	201	C31H82N18NaOP2S2	871.5728	1.1	71.1	201	13.60	1.0	even	ok
	202	C36H88N4NaO13S2	871.5682	-4.3	71.3	202	1.33	-5.0	even	ok
	203	C33H69N24NaOP	871.5716	-0.3	71.6	203	14.85	12.0	even	ok
	204	C33H84N12O8PS2	871.5709	-1.1	71.6	204	12.77	-1.0	even	ok
	205	C35H89N6NaO10PS2	871.5711	-0.8	71.8	205	14.76	-5.0	even	ok
	206	C32H85N14O5P2S2	871.5739	2.3	72.6	206	6.36	-1.0	even	ok
	207	C34H90N8NaO7P2S2	871.5741	2.6	72.7	207	5.09	-5.0	even	ok
	208	C35H78N16NaO4P2	871.5759	4.7	73.2	208	0.64	6.0	even	ok
	209	C37H91O17S2	871.5692	-3.0	73.5	209	3.60	-7.0	even	ok
	210	C56H79N4S2	871.5741	2.5	73.6	210	5.18	20.0	even	ok
	211	C36H92N2O14PS2	871.5722	0.4	73.8	211	16.37	-7.0	even	ok
	212	C35H93N4O11P2S2	871.5752	3.8	74.5	212	1.76	-7.0	even	ok
	213	C33H73N22O2P2	871.5757	4.4	74.7	213	0.85	10.0	even	ok
	214	C31H72N22NaO4S	871.5720	0.1	75.1	214	13.77	7.0	even	ok
	215	C29H67N28O2S	871.5717	-0.2	75.9	215	13.14	11.0	even	ok
	216	C36H80N8NaO14	871.5686	-3.7	77.5	216	1.38	1.0	even	ok

Compound Spectrum SmartFormula Report

Meas. m/z	#	Ion Formula	m/z	err [ppm]	mSigma	# mSigma	Score	rdb	e¯ Conf	N-Rule
	217	C37H83N4O18	871.5697	-2.5	77.6	217	3.65	-1.0	even	ok
	218	C30H76N18NaO8S	871.5706	-1.4	77.8	218	8.85	2.0	even	ok
	219	C33H84N8NaO14S	871.5720	0.1	77.8	219	15.42	-4.0	even	ok
	220	C30H73N24NaOPS	871.5750	3.6	77.9	220	1.56	7.0	even	ok
	221	C33H82N14NaO5P2S	871.5681	-4.3	78.2	221	0.75	1.0	even	ok
	222	C31H79N14O12S	871.5717	-0.2	78.4	222	14.98	0.0	even	ok
	223	C31H76N20O5PS	871.5760	4.8	78.4	223	0.47	5.0	even	ok
	224	C28H76N22NaO4S2	871.5754	4.0	78.6	224	1.27	2.0	even	ok
	225	C34H75N14O12	871.5683	-4.0	78.7	225	0.98	5.0	even	ok
	226	C34H87N4O18S	871.5731	1.4	78.8	226	8.61	-6.0	even	ok
	227	C31H77N20O3P2S	871.5678	-4.7	79.0	227	0.52	5.0	even	ok
	228	C38H89NaO17P	871.5730	1.3	79.7	228	7.05	-5.0	even	ok
	229	C31H85N12NaO8PS2	871.5685	-3.9	79.9	229	1.33	-4.0	even	ok
	230	C32H85N10NaO11PS	871.5750	3.6	80.0	230	1.77	-4.0	even	ok
	231	C29H80N18O6PS2	871.5682	-4.2	80.0	231	0.98	0.0	even	ok
	232	C35H94NaO15P2S	871.5681	-4.3	80.6	232	0.86	-10.0	even	ok
	233	C30H80N16O9PS	871.5747	3.3	80.7	233	2.26	0.0	even	ok
	234	C33H88N6O15PS	871.5761	4.8	80.7	234	0.52	-6.0	even	ok
	235	C36H84N6O15P	871.5727	0.9	80.8	235	7.89	-1.0	even	ok
	236	C35H81N10NaO11P	871.5716	-0.3	80.9	236	10.34	1.0	even	ok
	237	C33H89N6O13P2S	871.5678	-4.7	81.1	237	0.61	-6.0	even	ok
	238	C30H86N14NaO5P2S2	871.5714	-0.5	81.3	238	11.83	-4.0	even	ok
	239	C26H71N28O2S2	871.5751	3.7	81.3	239	1.53	6.0	even	ok
	240	C28H81N20O3P2S2	871.5712	-0.8	81.5	240	10.23	0.0	even	ok
	241	C32H88N8O12PS2	871.5695	-2.7	81.6	241	3.40	-6.0	even	ok
	242	C34H93N2NaO14PS2	871.5698	-2.4	81.6	242	4.24	-10.0	even	ok
	243	C33H76N16O9P	871.5713	-0.6	82.2	243	8.63	5.0	even	ok
	244	C32H73N20NaO5P	871.5703	-1.8	82.7	244	4.54	7.0	even	ok
	245	C33H94N4NaO11P2S2	871.5728	1.1	82.7	245	8.55	-10.0	even	ok
	246	C31H89N10O9P2S2	871.5725	0.8	82.7	246	9.91	-6.0	even	ok
	247	C37H90N2NaO14P2	871.5759	4.7	82.9	247	0.43	-5.0	even	ok
	248	C30H68N26O3P	871.5700	-2.2	84.1	248	3.51	11.0	even	ok
	249	C35H85N8O12P2	871.5757	4.4	84.1	249	0.57	-1.0	even	ok
	250	C34H97O15P2S2	871.5739	2.3	84.4	250	3.95	-12.0	even	ok
	251	C34H82N12NaO8P2	871.5746	3.1	84.5	251	1.69	1.0	even	ok
	252	C32H77N18O6P2	871.5743	2.8	85.8	252	2.05	5.0	even	ok
	253	C31H74N22NaO2P2	871.5733	1.6	86.4	253	4.44	7.0	even	ok
	254	C27H68N28NaO2S	871.5693	-2.9	87.2	254	1.76	8.0	even	ok
	255	C28H71N24O6S	871.5704	-1.7	87.6	255	3.94	6.0	even	ok
	256	C29H69N28P2	871.5730	1.3	87.9	256	4.95	11.0	even	ok
	257	C29H80N14NaO12S	871.5693	-2.9	88.2	257	2.13	-3.0	even	ok
	258	C32H88N4NaO18S	871.5707	-1.4	88.3	258	5.75	-9.0	even	ok
	259	C30H83N10O16S	871.5704	-1.7	88.8	259	4.70	-5.0	even	ok
	260	C27H75N20O10S	871.5690	-3.3	88.9	260	1.60	1.0	even	ok
	261	C27H80N18NaO8S2	871.5740	2.5	88.9	261	2.88	-3.0	even	ok
	262	C25H75N24O6S2	871.5737	2.1	89.2	262	3.54	1.0	even	ok

Compound Spectrum SmartFormula Report

Meas. m/z	#	Ion Formula	m/z	err [ppm]	mSigma	# mSigma	Score	rdb	e¯ Conf	N-Rule
	263	C36H87O22	871.5684	-4.0	89.2	263	0.64	-6.0	even	ok
	264	C33H91O22S	871.5717	-0.2	89.2	264	9.56	-11.0	even	ok
	265	C25H63N34S	871.5690	-3.3	89.3	265	1.25	12.0	even	ok
	266	C29H77N20NaO5PS	871.5736	2.0	89.6	266	2.99	2.0	even	ok
	267	C24H72N28NaO2S2	871.5727	0.9	90.0	267	6.71	3.0	even	ok
	268	C30H88N8NaO14S2	871.5754	4.0	90.2	268	0.77	-9.0	even	ok
	269	C28H83N14O12S2	871.5751	3.7	90.3	269	1.03	-5.0	even	ok
	270	C27H72N26O3PS	871.5734	1.7	90.5	270	3.49	6.0	even	ok
	271	C31H89N6NaO15PS	871.5736	2.0	90.5	271	3.57	-9.0	even	ok
	272	C29H84N12O13PS	871.5734	1.7	91.1	272	4.21	-5.0	even	ok
	273	C32H92N2O19PS	871.5747	3.3	91.2	273	1.43	-11.0	even	ok
	274	C31H92N4O16PS2	871.5682	-4.2	91.6	274	0.60	-11.0	even	ok
	275	C35H88N2O19P	871.5713	-0.6	92.3	275	5.62	-6.0	even	ok
	276	C34H85N6NaO15P	871.5703	-1.8	92.3	276	3.01	-4.0	even	ok
	277	C32H80N12O13P	871.5700	-2.1	93.5	277	2.34	0.0	even	ok
	278	C31H77N16NaO9P	871.5689	-3.4	93.8	278	0.94	2.0	even	ok
	279	C28H68N26NaO5	871.5758	4.5	94.6	279	0.30	8.0	even	ok
	280	C29H72N22O7P	871.5686	-3.7	95.2	280	0.67	6.0	even	ok
	281	C34H89N4O16P2	871.5743	2.8	95.5	281	1.32	-6.0	even	ok
	282	C33H86N8NaO12P2	871.5733	1.6	95.7	282	2.92	-4.0	even	ok
	283	C28H69N26NaO3P	871.5676	-4.9	95.8	283	0.19	8.0	even	ok
	284	C26H63N32O3	871.5755	4.2	96.0	284	0.39	12.0	even	ok
	285	C31H81N14O10P2	871.5730	1.3	97.0	285	3.28	0.0	even	ok
	286	C30H78N18NaO6P2	871.5719	0.1	97.4	286	5.46	2.0	even	ok
	287	C28H84N10NaO16S	871.5680	-4.5	98.6	287	0.34	-8.0	even	ok
	288	C31H92NaO22S	871.5693	-2.9	98.8	288	1.33	-14.0	even	ok
	289	C28H73N24O4P2	871.5716	-0.3	98.8	289	4.77	6.0	even	ok
	290	C26H72N24NaO6S	871.5680	-4.5	98.9	290	0.27	3.0	even	ok
	291	C29H87N6O20S	871.5690	-3.2	99.3	291	1.01	-10.0	even	ok
	292	C27H70N28NaP2	871.5706	-1.5	99.6	292	2.62	8.0	even	ok
	293	C24H67N30O4S	871.5677	-4.8	99.8	293	0.18	7.0	even	ok
885.5869	1	C49H75N12NaP	885.5867	-0.2	16.1	1	100.00	19.0	even	ok
	2	C49H79N10OP2	885.5908	4.4	16.1	2	5.84	17.0	even	ok
	3	C48H69N16O	885.5835	-3.9	18.0	3	8.63	23.0	even	ok
	4	C51H84N4NaO3P2	885.5911	4.7	18.2	4	4.01	13.0	even	ok
	5	C46H74N14O2P	885.5851	-2.1	18.5	5	37.59	18.0	even	ok
	6	C47H80N10NaOP2	885.5884	1.6	18.9	6	47.98	14.0	even	ok
	7	C48H79N8NaO4P	885.5854	-1.7	19.6	7	44.64	14.0	even	ok
	8	C50H78N8O4P	885.5878	1.0	19.9	8	66.67	17.0	even	ok
	9	C50H74N10NaO3	885.5838	-3.6	20.4	9	10.96	19.0	even	ok
	10	C48H83N6O5P2	885.5895	2.8	21.6	10	20.08	12.0	even	ok
	11	C52H83N2NaO6P	885.5881	1.3	22.3	11	53.94	13.0	even	ok
	12	C50H88NaO7P2	885.5897	3.2	22.7	12	15.24	8.0	even	ok
	13	C49H82N4O8P	885.5865	-0.5	23.0	13	76.37	12.0	even	ok
	14	C51H74N12P	885.5892	2.5	23.8	14	24.93	22.0	even	ok
	15	C51H77N6O7	885.5848	-2.4	24.3	15	26.24	17.0	even	ok

Compound Spectrum SmartFormula Report

Meas. m/z	#	Ion Formula	m/z	err [ppm]	mSigma	# mSigma	Score	rdb	e¯ Conf	N-Rule
	16	C50H81N2O11	885.5835	-3.9	25.5	16	7.41	12.0	even	ok
	17	C44H75N14NaO2P	885.5827	-4.8	26.2	17	3.03	15.0	even	ok
	18	C53H79N6NaO2P	885.5894	2.8	26.3	18	18.65	18.0	even	ok
	19	C44H74N14NaO4	885.5910	4.5	26.3	19	3.87	15.0	even	ok
	20	C42H69N20O2	885.5907	4.2	26.3	20	5.35	19.0	even	ok
	21	C42H70N20P	885.5824	-5.1	26.4	21	2.11	19.0	even	ok
	22	C53H82NaO9	885.5851	-2.1	26.9	22	30.61	13.0	even	ok
	23	C45H78N10O6P	885.5838	-3.6	28.0	23	9.59	13.0	even	ok
	24	C47H83N4NaO8P	885.5841	-3.2	28.3	24	12.48	9.0	even	ok
	25	C52H73N10O3	885.5862	-0.9	29.0	25	56.71	22.0	even	ok
	26	C46H84N6NaO5P2	885.5871	0.1	29.1	26	77.72	9.0	even	ok
	27	C44H79N12O3P2	885.5868	-0.2	29.2	27	75.91	13.0	even	ok
	28	C54H82N2O6P	885.5905	4.0	29.7	28	6.07	16.0	even	ok
	29	C48H86O12P	885.5851	-2.0	30.9	29	28.53	7.0	even	ok
	30	C47H87N2O9P2	885.5881	1.3	31.1	30	43.00	7.0	even	ok
	31	C55H81O9	885.5875	0.6	31.5	31	74.71	16.0	even	ok
	32	C54H78N4NaO5	885.5864	-0.6	31.6	32	61.38	18.0	even	ok
	33	C46H79N12NaPS	885.5901	3.6	32.9	33	10.47	14.0	even	ok
	34	C43H74N16NaOS	885.5844	-2.8	34.0	34	18.95	15.0	even	ok
	35	C45H73N16OS	885.5868	-0.1	34.2	35	86.12	18.0	even	ok
	36	C47H83N8O2P2S	885.5829	-4.5	34.7	36	4.01	12.0	even	ok
	37	C55H79N6P2	885.5836	-3.8	34.9	37	6.61	21.0	even	ok
	38	C43H78N14O2PS	885.5885	1.8	35.4	38	38.02	13.0	even	ok
	39	C50H84N6NaP2S	885.5846	-2.7	36.2	39	19.82	13.0	even	ok
	40	C47H78N10NaO3S	885.5871	0.2	36.3	40	78.93	14.0	even	ok
	41	C49H88N2NaO4P2S	885.5832	-4.2	36.6	41	5.27	8.0	even	ok
	42	C47H82N8O4PS	885.5912	4.8	36.7	42	2.87	12.0	even	ok
	43	C40H70N20NaO2	885.5883	1.5	36.7	43	33.98	16.0	even	ok
	44	C45H83N8NaO4PS	885.5888	2.1	36.9	44	30.09	9.0	even	ok
	45	C57H84NaO2P2	885.5839	-3.5	37.3	45	8.24	17.0	even	ok
	46	C43H78N10NaO8	885.5896	3.0	37.4	46	11.89	10.0	even	ok
	47	C38H65N26	885.5880	1.2	37.4	47	39.50	20.0	even	ok
	48	C44H77N12O5S	885.5855	-1.6	37.5	48	38.91	13.0	even	ok
	49	C41H73N16O6	885.5894	2.7	37.7	49	15.07	14.0	even	ok
	50	C39H74N20PS	885.5858	-1.3	38.8	50	45.40	14.0	even	ok
	51	C46H87NaO12P	885.5827	-4.8	38.9	51	2.23	4.0	even	ok
	52	C44H82N6O10P	885.5825	-5.1	38.9	52	1.57	8.0	even	ok
	53	C44H81N6O12	885.5907	4.2	39.1	53	3.84	8.0	even	ok
	54	C46H82N6NaO7S	885.5858	-1.3	39.1	54	44.34	9.0	even	ok
	55	C46H86NaO14	885.5910	4.5	39.2	55	2.76	4.0	even	ok
	56	C40H73N18O3S	885.5828	-4.7	39.3	56	3.08	14.0	even	ok
	57	C41H79N14NaO2PS	885.5861	-1.0	39.6	57	52.18	10.0	even	ok
	58	C42H80N12NaO3P2	885.5844	-2.9	39.7	58	12.33	10.0	even	ok
	59	C55H74N8NaO	885.5878	0.9	39.7	59	42.05	23.0	even	ok
	60	C51H87N2O4P2S	885.5856	-1.5	40.0	60	39.26	11.0	even	ok
	61	C48H81N6O7S	885.5882	1.4	40.1	61	40.91	12.0	even	ok

Compound Spectrum SmartFormula Report

Meas. m/z	#	Ion Formula	m/z	err [ppm]	mSigma	# mSigma	Score	rdb	e¯ Conf	N-Rule
	62	C46H86N4O8PS	885.5898	3.3	40.3	62	11.21	7.0	even	ok
	63	C42H78N12NaO5S	885.5831	-4.3	40.3	63	4.17	10.0	even	ok
	64	C45H88N2NaO9P2	885.5857	-1.4	40.3	64	33.02	4.0	even	ok
	65	C49H77N10O3S	885.5895	2.9	40.4	65	14.92	17.0	even	ok
	66	C40H75N18OP2	885.5841	-3.2	40.4	66	9.37	14.0	even	ok
	67	C43H83N8O7P2	885.5854	-1.7	40.6	67	27.38	8.0	even	ok
	68	C41H83N12O3P2S	885.5902	3.6	41.8	68	7.97	8.0	even	ok
	69	C50H86NaO9S	885.5885	1.7	42.2	69	32.21	8.0	even	ok
	70	C42H82N10O6PS	885.5872	0.2	42.3	70	66.00	8.0	even	ok
	71	C56H77N4O5	885.5888	2.1	42.5	71	19.67	21.0	even	ok
	72	C52H83N6P2S	885.5870	0.0	42.5	72	71.21	16.0	even	ok
	73	C43H88N6NaO5P2S	885.5904	3.9	42.6	73	5.83	4.0	even	ok
	74	C47H85N2O11S	885.5869	-0.1	42.7	74	68.83	7.0	even	ok
	75	C51H82N4NaO5S	885.5898	3.2	42.8	75	10.83	13.0	even	ok
	76	C44H87N4NaO8PS	885.5874	0.6	43.3	76	56.46	4.0	even	ok
	77	C43H81N8O9S	885.5842	-3.1	43.3	77	11.59	8.0	even	ok
	78	C52H86O7PS	885.5826	-4.9	44.2	78	2.13	11.0	even	ok
	79	C45H86N2NaO11S	885.5845	-2.8	44.6	79	14.36	4.0	even	ok
	80	C54H88NaO2P2S	885.5872	0.3	45.0	80	58.97	12.0	even	ok
	81	C52H78N8NaOS	885.5912	4.7	46.2	81	2.30	18.0	even	ok
	82	C45H90O12PS	885.5885	1.8	46.3	82	27.94	2.0	even	ok
	83	C52H85O9S	885.5909	4.4	46.6	83	3.15	11.0	even	ok
	84	C53H82N4O3PS	885.5840	-3.4	47.3	84	8.58	16.0	even	ok
	85	C38H78N16O4PS	885.5845	-2.8	47.8	85	13.35	9.0	even	ok
	86	C40H83N10NaO6PS	885.5848	-2.5	48.2	86	16.63	5.0	even	ok
	87	C39H74N16NaO6	885.5869	-0.0	48.4	87	48.33	11.0	even	ok
	88	C37H79N18OP2S	885.5875	0.6	48.4	88	47.99	9.0	even	ok
	89	C39H84N12NaO3P2S	885.5877	0.9	48.7	89	41.35	5.0	even	ok
	90	C36H66N26Na	885.5856	-1.5	48.8	90	23.84	17.0	even	ok
	91	C42H82N6NaO12	885.5883	1.5	48.9	91	23.94	5.0	even	ok
	92	C37H69N22O4	885.5867	-0.3	49.1	92	41.99	15.0	even	ok
	93	C40H77N12O10	885.5880	1.2	49.3	93	28.03	9.0	even	ok
	94	C59H83O2P2	885.5863	-0.8	49.3	94	34.79	20.0	even	ok
	95	C43H85N2O16	885.5894	2.7	50.3	95	10.42	3.0	even	ok
	96	C41H86N6O10PS	885.5858	-1.3	50.6	96	32.54	3.0	even	ok
	97	C40H87N8O7P2S	885.5888	2.1	50.7	97	19.73	3.0	even	ok
	98	C42H85N4O13S	885.5828	-4.6	50.9	98	2.23	3.0	even	ok
	99	C41H83N8NaO9P	885.5913	4.9	51.0	99	1.35	5.0	even	ok
	100	C37H75N20NaPS	885.5834	-4.0	51.1	100	3.43	11.0	even	ok
	101	C38H75N18NaO3P	885.5899	3.4	51.1	101	6.03	11.0	even	ok
	102	C42H92N2NaO9P2S	885.5891	2.4	51.2	102	15.71	-1.0	even	ok
	103	C43H91NaO12PS	885.5861	-1.0	51.3	103	37.36	-1.0	even	ok
	104	C41H84N8NaO7P2	885.5830	-4.4	51.4	104	2.24	5.0	even	ok
	105	C39H78N14O7P	885.5910	4.6	51.6	105	1.87	9.0	even	ok
	106	C57H73N8O	885.5902	3.7	52.0	106	4.58	26.0	even	ok
	107	C36H70N24OP	885.5897	3.1	52.1	107	7.61	15.0	even	ok

Compound Spectrum SmartFormula Report

Meas. m/z	#	Ion Formula	m/z	err [ppm]	mSigma	# mSigma	Score	rdb	e⁻ Conf	N-Rule
	108	C39H79N14O5P2	885.5828	-4.7	52.2	108	1.56	9.0	even	ok
	109	C42H87N4O11P2	885.5841	-3.2	52.3	109	6.69	3.0	even	ok
	110	C56H87O2P2S	885.5897	3.1	53.3	110	9.17	15.0	even	ok
	111	C59H78N2NaO3	885.5905	4.0	54.3	111	3.17	22.0	even	ok
	112	C44H87N8O2P2S2	885.5863	-0.7	54.4	112	37.81	7.0	even	ok
	113	C47H88N6NaP2S2	885.5879	1.1	54.4	113	31.24	8.0	even	ok
	114	C35H69N26S	885.5914	5.0	54.8	114	1.07	15.0	even	ok
	115	C42H88N8NaO2P2S2	885.5839	-3.4	55.1	115	6.33	4.0	even	ok
	116	C56H83N2NaOPS	885.5856	-1.5	55.1	116	24.46	17.0	even	ok
	117	C44H82N10NaO3S2	885.5905	4.0	55.7	117	3.66	9.0	even	ok
	118	C34H73N22O4S	885.5900	3.5	55.8	118	5.89	10.0	even	ok
	119	C36H78N16NaO6S	885.5903	3.8	55.9	119	4.42	6.0	even	ok
	120	C46H82N10OPS2	885.5847	-2.6	56.0	120	12.05	12.0	even	ok
	121	C46H92N2NaO4P2S2	885.5866	-0.4	56.3	121	40.68	3.0	even	ok
	122	C41H81N12O5S2	885.5889	2.2	57.3	122	15.25	8.0	even	ok
	123	C37H82N12O8PS	885.5831	-4.3	57.4	123	2.60	4.0	even	ok
	124	C45H86N6O5PS2	885.5833	-4.1	57.4	124	3.19	7.0	even	ok
	125	C39H87N6NaO10PS	885.5834	-4.0	57.6	125	3.52	0.0	even	ok
	126	C37H81N12O10S	885.5914	5.0	57.8	126	1.20	4.0	even	ok
	127	C48H91N2O4P2S2	885.5890	2.3	58.3	127	13.50	6.0	even	ok
	128	C48H87N4NaO3PS2	885.5849	-2.3	58.3	128	13.97	8.0	even	ok
	129	C36H83N14O5P2S	885.5861	-0.9	58.4	129	30.13	4.0	even	ok
	130	C38H88N8NaO7P2S	885.5864	-0.6	58.4	130	34.73	0.0	even	ok
	131	C43H91N4O6P2S2	885.5850	-2.2	58.5	131	14.25	2.0	even	ok
	132	C49H87N6P2S2	885.5903	3.8	58.7	132	3.93	11.0	even	ok
	133	C40H83N14P2S2	885.5836	-3.7	58.9	133	4.21	8.0	even	ok
	134	C43H86N6NaO7S2	885.5892	2.5	59.0	134	11.61	4.0	even	ok
	135	C40H78N16NaOS2	885.5878	1.0	59.4	135	28.39	10.0	even	ok
	136	C47H91NaO7PS2	885.5836	-3.8	59.4	136	4.03	3.0	even	ok
	137	C40H90N2O14PS	885.5845	-2.8	59.7	137	9.25	-2.0	even	ok
	138	C42H77N16OS2	885.5902	3.7	59.7	138	4.30	13.0	even	ok
	139	C57H82NaO4S	885.5826	-4.9	60.1	139	1.24	17.0	even	ok
	140	C38H78N12NaO10	885.5856	-1.5	60.1	140	16.72	6.0	even	ok
	141	C39H91N4O11P2S	885.5875	0.6	60.2	141	32.76	-2.0	even	ok
	142	C41H86N2NaO16	885.5870	0.0	60.5	142	32.81	0.0	even	ok
	143	C35H70N22NaO4	885.5843	-3.0	60.5	143	5.87	12.0	even	ok
	144	C36H73N18O8	885.5853	-1.8	60.9	144	13.55	10.0	even	ok
	145	C39H81N8O14	885.5867	-0.3	61.0	145	28.85	4.0	even	ok
	146	C51H92NaO2P2S2	885.5906	4.1	61.1	146	2.68	7.0	even	ok
	147	C41H92N4NaO6P2S2	885.5826	-4.9	61.2	147	1.15	-1.0	even	ok
	148	C39H86N10O6PS2	885.5905	4.1	61.3	148	2.92	3.0	even	ok
	149	C48H77N12S2	885.5830	-4.4	61.4	149	1.95	17.0	even	ok
	150	C33H65N28O2	885.5840	-3.4	61.5	150	4.37	16.0	even	ok
	151	C36H78N20PS2	885.5892	2.5	61.7	151	10.37	9.0	even	ok
	152	C49H90O7PS2	885.5860	-1.1	62.1	152	24.84	6.0	even	ok
	153	C41H91N4NaO8PS2	885.5908	4.4	62.5	153	2.04	-1.0	even	ok

Compound Spectrum SmartFormula Report

Meas. m/z	#	Ion Formula	m/z	err [ppm]	mSigma	# mSigma	Score	rdb	e¯ Conf	N-Rule
	154	C44H89N2O11S2	885.5902	3.7	62.6	154	3.86	2.0	even	ok
	155	C40H87N4NaO13P	885.5899	3.4	62.7	155	4.08	0.0	even	ok
	156	C40H85N8O9S2	885.5875	0.7	62.8	156	29.13	3.0	even	ok
	157	C37H79N14NaO7P	885.5886	1.9	62.9	157	12.41	6.0	even	ok
	158	C38H83N14NaO2PS2	885.5895	2.8	62.9	158	7.87	5.0	even	ok
	159	C50H86N4O3PS2	885.5873	0.5	63.1	159	31.66	11.0	even	ok
	160	C37H77N18O3S2	885.5862	-0.8	63.2	160	26.52	9.0	even	ok
	161	C33H70N26NaS	885.5890	2.3	63.2	161	9.37	12.0	even	ok
	162	C38H82N10O11P	885.5897	3.1	63.4	162	5.18	4.0	even	ok
	163	C35H80N18NaOP2S	885.5851	-2.1	63.5	163	10.25	6.0	even	ok
	164	C34H71N24NaOP	885.5873	0.3	63.7	164	25.95	12.0	even	ok
	165	C41H90O17P	885.5910	4.6	63.7	165	1.24	-2.0	even	ok
	166	C35H74N20O5P	885.5883	1.5	63.8	166	14.49	10.0	even	ok
	167	C50H82N6NaO2S2	885.5833	-4.1	63.8	167	2.46	13.0	even	ok
	168	C41H91O15P2	885.5828	-4.7	64.0	168	1.07	-2.0	even	ok
	169	C42H90N2NaO11S2	885.5878	1.0	64.1	169	23.99	-1.0	even	ok
	170	C42H95O10P2S2	885.5836	-3.7	64.3	170	3.53	-3.0	even	ok
	171	C39H82N12NaO5S2	885.5865	-0.5	64.5	171	29.10	5.0	even	ok
	172	C61H78N2OP	885.5846	-2.6	64.9	172	6.95	25.0	even	ok
	173	C58H82N2OPS	885.5880	1.2	65.0	173	21.08	20.0	even	ok
	174	C35H82N12NaO10S	885.5890	2.3	65.9	174	10.57	1.0	even	ok
	175	C33H77N18O8S	885.5887	2.0	65.9	175	12.98	5.0	even	ok
	176	C33H73N24OS2	885.5835	-3.9	66.0	176	2.91	10.0	even	ok
	177	C34H79N20NaPS2	885.5868	-0.2	66.3	177	31.38	6.0	even	ok
	178	C38H86N8NaO9S2	885.5851	-2.0	66.5	178	12.16	0.0	even	ok
	179	C34H75N22O2P2	885.5913	4.9	66.9	179	0.77	10.0	even	ok
	180	C35H78N18NaO3S2	885.5838	-3.6	67.0	180	3.75	6.0	even	ok
	181	C58H78N4NaS	885.5839	-3.4	67.2	181	4.31	22.0	even	ok
	182	C36H85N8O14S	885.5900	3.5	67.6	182	3.89	-1.0	even	ok
	183	C51H85N2O6S2	885.5844	-2.9	67.6	183	6.25	11.0	even	ok
	184	C38H90N2NaO16S	885.5903	3.8	67.7	184	2.91	-5.0	even	ok
	185	C34H83N18OP2S2	885.5908	4.4	68.4	185	1.60	4.0	even	ok
	186	C37H92N4NaO11P2S	885.5851	-2.1	68.5	186	10.82	-5.0	even	ok
	187	C53H87N2NaOPS2	885.5890	2.3	68.5	187	9.71	12.0	even	ok
	188	C35H87N10O9P2S	885.5848	-2.4	68.6	188	8.71	-1.0	even	ok
	189	C38H90N6O10PS2	885.5892	2.5	68.6	189	8.05	-2.0	even	ok
	190	C35H82N16O4PS2	885.5879	1.0	69.0	190	19.85	4.0	even	ok
	191	C36H88N12NaO3P2S2	885.5911	4.7	69.2	191	1.12	0.0	even	ok
	192	C39H89N4O13S2	885.5862	-0.8	69.5	192	21.24	-2.0	even	ok
	193	C40H95NaO12PS2	885.5895	2.9	69.6	193	6.14	-6.0	even	ok
	194	C36H81N14O7S2	885.5849	-2.4	69.9	194	8.73	4.0	even	ok
	195	C37H87N10NaO6PS2	885.5881	1.3	69.9	195	16.31	0.0	even	ok
	196	C59H81O4S	885.5850	-2.2	70.1	196	9.70	20.0	even	ok
	197	C38H95O15P2S	885.5861	-0.9	70.1	197	20.07	-7.0	even	ok
	198	C52H81N6O2S2	885.5857	-1.4	70.9	198	15.07	16.0	even	ok
	199	C37H82N8NaO14	885.5843	-3.0	71.9	199	3.94	1.0	even	ok

Compound Spectrum SmartFormula Report

Meas. m/z	#	Ion Formula	m/z	err [ppm]	mSigma	# mSigma	Score	rdb	e⁻ Conf	N-Rule
	200	C32H74N22NaO4S	885.5876	0.8	72.1	200	15.92	7.0	even	ok
	201	C34H74N18NaO8	885.5829	-4.5	72.2	201	0.95	7.0	even	ok
	202	C63H73N4	885.5830	-4.5	72.6	202	1.25	30.0	even	ok
	203	C35H77N14O12	885.5840	-3.3	72.6	203	2.96	5.0	even	ok
	204	C38H85N4O18	885.5853	-1.8	72.7	204	8.92	-1.0	even	ok
	205	C32H69N24O6	885.5826	-4.9	73.2	205	0.65	11.0	even	ok
	206	C54H86NaO4S2	885.5860	-1.1	73.4	206	16.17	12.0	even	ok
	207	C35H85N10O11S2	885.5835	-3.9	73.8	207	2.20	-1.0	even	ok
	208	C31H78N22O2PS2	885.5852	-2.0	74.1	208	9.37	5.0	even	ok
	209	C36H91N6NaO10PS2	885.5868	-0.2	74.3	209	23.41	-5.0	even	ok
	210	C30H69N28O2S	885.5874	0.5	74.4	210	16.71	11.0	even	ok
	211	C39H91NaO17P	885.5886	1.9	74.5	211	8.05	-5.0	even	ok
	212	C37H90N4NaO13S2	885.5838	-3.6	74.5	212	2.86	-5.0	even	ok
	213	C36H83N10NaO11P	885.5873	0.4	74.6	213	17.27	1.0	even	ok
	214	C33H83N16NaO4PS2	885.5854	-1.7	74.6	214	11.12	1.0	even	ok
	215	C32H84N18NaOP2S2	885.5884	1.7	74.7	215	11.23	1.0	even	ok
	216	C34H82N14NaO7S2	885.5825	-5.1	74.9	216	0.60	1.0	even	ok
	217	C31H78N18NaO8S	885.5863	-0.7	74.9	217	18.10	2.0	even	ok
	218	C34H84N14NaO5P2S	885.5837	-3.6	75.1	218	2.08	1.0	even	ok
	219	C37H86N6O15P	885.5883	1.6	75.2	219	9.46	-1.0	even	ok
	220	C33H75N20NaO5P	885.5859	-1.2	75.3	220	11.61	7.0	even	ok
	221	C32H79N20O3P2S	885.5834	-4.0	75.4	221	1.53	5.0	even	ok
	222	C34H78N16O9P	885.5870	0.0	75.5	222	18.82	5.0	even	ok
	223	C31H75N24NaOPS	885.5906	4.1	75.8	223	1.25	7.0	even	ok
	224	C34H86N8NaO14S	885.5876	0.8	76.0	224	16.99	-4.0	even	ok
	225	C32H81N14O12S	885.5874	0.5	76.2	225	19.39	0.0	even	ok
	226	C31H70N26O3P	885.5856	-1.5	76.3	226	9.41	11.0	even	ok
	227	C36H95N4O11P2S2	885.5909	4.4	76.7	227	1.16	-7.0	even	ok
	228	C55H86N2OPS2	885.5914	5.0	76.7	228	0.61	15.0	even	ok
	229	C37H94N2O14PS2	885.5879	1.0	76.8	229	14.67	-7.0	even	ok
	230	C33H87N14O5P2S2	885.5895	2.9	77.0	230	4.52	-1.0	even	ok
	231	C34H86N12O8PS2	885.5865	-0.5	77.1	231	18.47	-1.0	even	ok
	232	C38H93O17S2	885.5849	-2.3	77.2	232	6.64	-7.0	even	ok
	233	C33H87N10NaO11PS	885.5906	4.2	77.5	233	1.44	-4.0	even	ok
	234	C35H84N12NaO8P2	885.5903	3.7	77.5	234	1.74	1.0	even	ok
	235	C35H89N4O18S	885.5887	2.0	77.6	235	8.31	-6.0	even	ok
	236	C35H92N8NaO7P2S2	885.5898	3.2	77.6	236	3.43	-5.0	even	ok
	237	C31H82N16O9PS	885.5904	3.8	77.8	237	1.93	0.0	even	ok
	238	C36H87N8O12P2	885.5913	4.9	77.9	238	0.50	-1.0	even	ok
	239	C60H77N4S	885.5863	-0.7	78.0	239	16.47	25.0	even	ok
	240	C55H82N4NaS2	885.5873	0.4	78.3	240	18.25	17.0	even	ok
	241	C33H79N18O6P2	885.5900	3.4	78.5	241	2.21	5.0	even	ok
	242	C32H76N22NaO2P2	885.5889	2.2	78.5	242	5.55	7.0	even	ok
	243	C36H96NaO15P2S	885.5837	-3.6	78.7	243	2.27	-10.0	even	ok
	244	C34H91N6O13P2S	885.5835	-3.9	78.8	244	1.69	-6.0	even	ok
	245	C30H71N28P2	885.5886	1.9	79.7	245	6.49	11.0	even	ok

Compound Spectrum SmartFormula Report

Meas. m/z	#	Ion Formula	m/z	err [ppm]	mSigma	# mSigma	Score	rdb	e¯ Conf	N-Rule
	246	C27H74N28PS2	885.5825	-5.0	80.8	246	0.49	6.0	even	ok
	247	C29H79N22NaO2PS2	885.5828	-4.7	81.1	247	0.69	2.0	even	ok
	248	C27H73N28O2S2	885.5907	4.3	81.2	248	1.12	6.0	even	ok
	249	C29H78N22NaO4S2	885.5910	4.6	81.5	249	0.80	2.0	even	ok
	250	C56H85O4S2	885.5884	1.6	81.7	250	8.79	15.0	even	ok
	251	C33H90N8O12PS2	885.5852	-2.0	82.8	251	6.65	-6.0	even	ok
	252	C30H82N18O6PS2	885.5838	-3.5	83.1	252	2.07	0.0	even	ok
	253	C36H94NaO17S2	885.5825	-5.1	83.2	253	0.44	-10.0	even	ok
	254	C35H95N2NaO14PS2	885.5855	-1.7	83.3	254	7.92	-10.0	even	ok
	255	C32H87N12NaO8PS2	885.5841	-3.2	83.6	255	2.67	-4.0	even	ok
	256	C34H96N4NaO11P2S2	885.5884	1.7	83.6	256	7.79	-10.0	even	ok
	257	C29H83N20O3P2S2	885.5868	-0.1	83.6	257	16.25	0.0	even	ok
	258	C36H86N4NaO18	885.5829	-4.5	83.7	258	0.61	-4.0	even	ok
	259	C31H88N14NaO5P2S2	885.5871	0.2	83.9	259	15.92	-4.0	even	ok
	260	C34H81N10O16	885.5827	-4.9	84.4	260	0.42	0.0	even	ok
	261	C28H70N28NaO2S	885.5849	-2.3	84.4	261	4.20	8.0	even	ok
	262	C37H89O22	885.5840	-3.3	84.4	262	1.86	-6.0	even	ok
	263	C26H65N34S	885.5847	-2.6	84.8	263	3.31	12.0	even	ok
	264	C30H82N14NaO12S	885.5850	-2.3	85.3	264	5.10	-3.0	even	ok
	265	C28H77N20O10S	885.5847	-2.6	85.6	265	4.03	1.0	even	ok
	266	C30H79N20NaO5PS	885.5893	2.6	85.7	266	3.06	2.0	even	ok
	267	C29H73N24O6S	885.5860	-1.1	85.8	267	8.02	6.0	even	ok
	268	C35H99O15P2S2	885.5895	2.9	85.8	268	3.13	-12.0	even	ok
	269	C32H91N10O9P2S2	885.5882	1.4	86.1	269	8.40	-6.0	even	ok
	270	C35H87N6NaO15P	885.5859	-1.2	86.3	270	7.45	-4.0	even	ok
	271	C33H90N4NaO18S	885.5863	-0.7	86.3	271	11.43	-9.0	even	ok
	272	C31H85N10O16S	885.5860	-1.0	86.5	272	9.78	-5.0	even	ok
	273	C32H79N16NaO9P	885.5846	-2.7	86.9	273	2.81	2.0	even	ok
	274	C36H90N2O19P	885.5870	0.0	86.9	274	11.74	-6.0	even	ok
	275	C33H82N12O13P	885.5856	-1.5	87.2	275	6.07	0.0	even	ok
	276	C28H74N26O3PS	885.5890	2.3	87.6	276	3.53	6.0	even	ok
	277	C34H93O22S	885.5874	0.5	87.8	277	11.98	-11.0	even	ok
	278	C57H81N4S2	885.5897	3.1	87.8	278	2.39	20.0	even	ok
	279	C30H74N22O7P	885.5843	-3.0	87.9	279	2.11	6.0	even	ok
	280	C32H91N6NaO15PS	885.5893	2.6	87.9	280	3.45	-9.0	even	ok
	281	C29H71N26NaO3P	885.5832	-4.2	87.9	281	0.72	8.0	even	ok
	282	C27H65N32O3	885.5912	4.8	88.1	282	0.38	12.0	even	ok
	283	C30H86N12O13PS	885.5890	2.3	88.2	283	4.26	-5.0	even	ok
	284	C23H69N34S2	885.5880	1.2	88.9	284	8.03	7.0	even	ok
	285	C25H74N28NaO2S2	885.5883	1.5	89.0	285	6.74	3.0	even	ok
	286	C27H66N32OP	885.5829	-4.5	89.1	286	0.49	12.0	even	ok
	287	C33H94N2O19PS	885.5904	3.9	89.2	287	1.19	-11.0	even	ok
	288	C34H88N8NaO12P2	885.5889	2.2	89.2	288	3.53	-4.0	even	ok
	289	C35H91N4O16P2	885.5900	3.4	89.6	289	1.38	-6.0	even	ok
	290	C31H80N18NaO6P2	885.5876	0.7	90.0	290	7.91	2.0	even	ok
	291	C32H83N14O10P2	885.5886	1.9	90.1	291	4.15	0.0	even	ok

Compound Spectrum SmartFormula Report

Meas. m/z	#	Ion Formula	m/z	err [ppm]	mSigma	# mSigma	Score	rdb	e⁻ Conf	N-Rule
	292	C31H91N8NaO12PS2	885.5828	-4.7	90.4	292	0.47	-9.0	even	ok
	293	C31H90N8NaO14S2	885.5910	4.6	90.8	293	0.53	-9.0	even	ok
	294	C26H77N24O6S2	885.5894	2.8	90.8	294	2.81	1.0	even	ok
	295	C28H82N18NaO8S2	885.5897	3.1	91.0	295	2.18	-3.0	even	ok
	296	C29H75N24O4P2	885.5873	0.4	91.1	296	8.61	6.0	even	ok
	297	C28H72N28NaP2	885.5862	-0.8	91.3	297	7.05	8.0	even	ok
	298	C32H94N4O16PS2	885.5838	-3.5	92.3	298	1.41	-11.0	even	ok
	299	C29H86N14O10PS2	885.5825	-5.0	92.6	299	0.30	-5.0	even	ok
	300	C29H85N14O12S2	885.5907	4.3	93.0	300	0.67	-5.0	even	ok
	301	C29H86N10NaO16S	885.5836	-3.8	95.7	301	0.97	-8.0	even	ok
	302	C27H74N24NaO6S	885.5836	-3.8	96.0	302	0.76	3.0	even	ok
	303	C27H81N16O14S	885.5833	-4.1	96.1	303	0.70	-4.0	even	ok
	304	C25H69N30O4S	885.5833	-4.1	96.4	304	0.55	7.0	even	ok
	305	C32H94NaO22S	885.5850	-2.2	96.7	305	3.11	-14.0	even	ok
	306	C30H89N6O20S	885.5847	-2.6	96.9	306	2.47	-10.0	even	ok
	307	C29H83N16NaO9PS	885.5879	1.1	97.4	307	4.62	-3.0	even	ok
	308	C34H91N2NaO19P	885.5846	-2.7	98.0	308	1.72	-9.0	even	ok
	309	C26H75N26NaO3PS	885.5866	-0.4	98.1	309	6.24	3.0	even	ok
	310	C31H83N12NaO13P	885.5832	-4.2	98.5	310	0.45	-3.0	even	ok
	311	C28H74N22NaO9	885.5901	3.6	98.5	311	0.79	3.0	even	ok
	312	C24H70N32OPS	885.5863	-0.7	98.6	312	5.32	7.0	even	ok
	313	C29H77N18O13	885.5912	4.8	98.7	313	0.24	1.0	even	ok
	314	C32H86N8O17P	885.5843	-3.0	98.8	314	1.30	-5.0	even	ok
	315	C27H78N22O7PS	885.5877	0.8	99.2	315	4.95	1.0	even	ok
	316	C29H78N18O11P	885.5830	-4.5	99.4	316	0.31	1.0	even	ok
	317	C26H69N28O7	885.5899	3.3	99.6	317	0.99	7.0	even	ok
	318	C28H84N18NaO6P2S	885.5909	4.5	99.6	318	0.31	-3.0	even	ok
	319	C25H66N32NaO3	885.5888	2.1	99.7	319	2.41	9.0	even	ok

Cmpd 16, 35.1 min

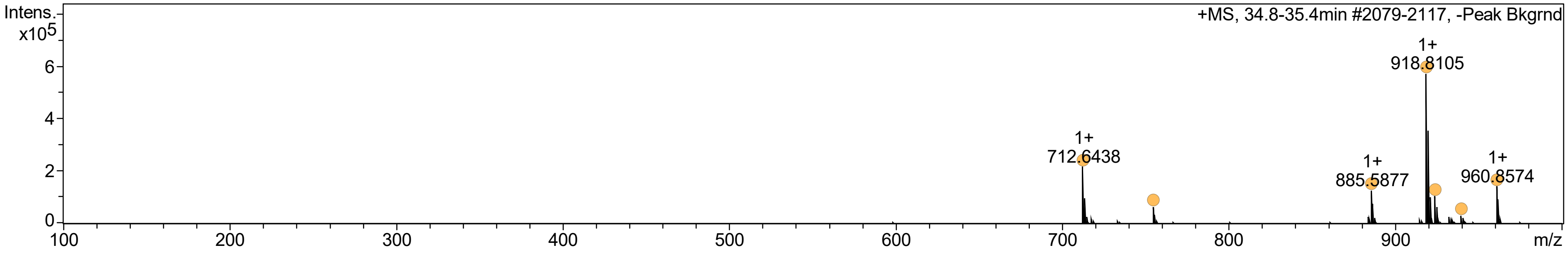

Meas. m/z	#	Ion Formula	m/z	err [ppm]	mSigma	# mSigma	Score	rdb	e⁻ Conf	N-Rule
712.6438	1	C41H87NNaO6	712.6426	-1.8	3.4	1	65.04	-1.0	even	ok

Compound Spectrum SmartFormula Report

Meas. m/z	#	Ion Formula	m/z	err [ppm]	mSigma	# mSigma	Score	rdb	e⁻ Conf	N-Rule
	2	C39H82N7O4	712.6423	-2.2	5.0	2	52.76	3.0	even	ok
	3	C40H88N3NaO3P	712.6456	2.4	6.5	3	46.77	-1.0	even	ok
	4	C38H83N9OP	712.6453	2.0	8.9	4	54.09	3.0	even	ok
	5	C38H79N11Na	712.6412	-3.7	9.2	5	21.23	5.0	even	ok
	6	C38H86N3O8	712.6409	-4.1	10.9	6	16.03	-2.0	even	ok
	7	C35H83N11NaS	712.6446	1.0	13.7	7	73.96	0.0	even	ok
	8	C43H86NO6	712.6450	1.6	13.9	8	59.57	2.0	even	ok
	9	C40H78N11	712.6436	-0.3	14.4	9	92.97	8.0	even	ok
	10	C42H83N5NaO2	712.6439	0.1	14.5	10	100.00	4.0	even	ok
	11	C39H92N3OP2S	712.6431	-1.1	15.1	11	71.04	-3.0	even	ok
	12	C37H87N5O5P	712.6439	0.1	15.3	12	97.12	-2.0	even	ok
	13	C37H82N11S	712.6470	4.4	15.5	13	11.64	3.0	even	ok
	14	C37H93N3NaOP2S	712.6407	-4.4	15.6	14	11.31	-6.0	even	ok
	15	C38H91NNaO6S	712.6459	2.9	17.3	15	35.85	-6.0	even	ok
	16	C36H84N9NaOP	712.6429	-1.4	17.5	16	59.99	0.0	even	ok
	17	C36H86N7O4S	712.6457	2.5	17.5	17	35.28	-2.0	even	ok
	18	C38H93NNaO4P2	712.6472	4.7	17.8	18	8.90	-6.0	even	ok
	19	C39H87N5NaO2S	712.6473	4.8	18.0	19	8.29	-1.0	even	ok
	20	C36H88N7O2P2	712.6469	4.3	19.8	20	11.33	-2.0	even	ok
	21	C32H82N13O2S	712.6430	-1.2	22.4	21	57.13	-1.0	even	ok
	22	C34H87N7NaO4S	712.6432	-0.8	22.8	22	65.53	-5.0	even	ok
	23	C35H90N3O8S	712.6443	0.7	22.9	23	87.17	-7.0	even	ok
	24	C33H88N9NaOPS	712.6462	3.4	23.5	24	19.59	-5.0	even	ok
	25	C41H87N5PS	712.6414	-3.4	24.3	25	18.85	2.0	even	ok
	26	C36H91NO9P	712.6426	-1.8	25.7	26	42.78	-7.0	even	ok
	27	C44H82N5O2	712.6463	3.4	25.7	27	17.65	7.0	even	ok
	28	C34H91N5O5PS	712.6473	4.9	25.7	28	6.74	-7.0	even	ok
	29	C35H88N5NaO5P	712.6415	-3.3	26.7	29	19.35	-5.0	even	ok
	30	C33H83N11O3P	712.6412	-3.6	28.4	30	14.61	-1.0	even	ok
	31	C35H92N3O6P2	712.6456	2.4	29.6	31	28.18	-7.0	even	ok
	32	C34H89N7NaO2P2	712.6445	0.9	30.9	32	52.27	-5.0	even	ok
	33	C30H83N13NaO2S	712.6406	-4.6	32.1	33	6.92	-4.0	even	ok
	34	C36H92N5NaPS2	712.6424	-2.0	32.3	34	40.20	-6.0	even	ok
	35	C28H78N19S	712.6403	-5.0	32.5	35	5.10	0.0	even	ok
	36	C32H84N13P2	712.6442	0.6	32.8	36	57.17	-1.0	even	ok
	37	C36H96N3OP2S2	712.6465	3.7	32.9	37	16.23	-8.0	even	ok
	38	C31H86N9O6S	712.6416	-3.1	33.6	38	17.75	-6.0	even	ok
	39	C33H91N3NaO8S	712.6419	-2.7	33.6	39	22.04	-10.0	even	ok
	40	C34H97N3NaOP2S2	712.6441	0.3	34.0	40	75.52	-11.0	even	ok
	41	C33H91N7O2PS2	712.6408	-4.3	34.4	41	10.18	-7.0	even	ok
	42	C32H92N5NaO5PS	712.6449	1.5	35.0	42	38.35	-10.0	even	ok
	43	C30H87N11O3PS	712.6446	1.1	35.3	43	44.48	-6.0	even	ok
	44	C38H91N5PS2	712.6448	1.3	35.6	44	49.83	-3.0	even	ok
	45	C35H96NNaO4PS2	712.6411	-3.9	35.9	45	12.77	-11.0	even	ok
	46	C37H95NO4PS2	712.6435	-0.5	36.0	46	66.19	-8.0	even	ok
	47	C33H95NO9PS	712.6460	3.0	36.7	47	17.70	-12.0	even	ok

Compound Spectrum SmartFormula Report

Meas. m/z	#	Ion Formula	m/z	err [ppm]	mSigma	# mSigma	Score	rdb	e¯ Conf	N-Rule
	48	C29H86N13O2S2	712.6463	3.5	38.1	48	15.72	-6.0	even	ok
	49	C31H83N11NaO5	712.6471	4.5	38.3	49	6.18	-4.0	even	ok
	50	C31H91N7NaO4S2	712.6466	3.9	39.1	50	11.91	-10.0	even	ok
	51	C39H90N3O3S2	712.6418	-2.9	39.8	51	21.78	-3.0	even	ok
	52	C29H78N17O3	712.6468	4.2	40.0	52	7.80	0.0	even	ok
	53	C33H93N3NaO6P2	712.6432	-0.9	41.1	53	40.27	-10.0	even	ok
	54	C31H88N9O4P2	712.6429	-1.3	42.6	54	33.17	-6.0	even	ok
	55	C30H85N13NaP2	712.6418	-2.8	43.9	55	15.77	-4.0	even	ok
	56	C30H90N5O10S	712.6403	-5.0	45.1	56	3.67	-11.0	even	ok
	57	C42H91NNaOS2	712.6434	-0.6	45.1	57	50.61	-2.0	even	ok
	58	C47H86NOS	712.6425	-1.9	46.4	58	28.60	6.0	even	ok
	59	C31H90N3O13	712.6468	4.2	48.5	59	6.05	-11.0	even	ok
	60	C30H87N7NaO9	712.6457	2.7	48.9	60	14.82	-9.0	even	ok
	61	C28H82N13O7	712.6455	2.3	50.4	61	17.34	-5.0	even	ok
	62	C27H79N17NaO3	712.6444	0.8	51.3	62	31.56	-3.0	even	ok
	63	C25H74N23O	712.6441	0.4	52.9	63	34.19	1.0	even	ok
	64	C44H90NOS2	712.6458	2.8	53.5	64	15.07	1.0	even	ok
754.6911	1	C46H92NO6	754.6919	1.1	1.7	1	75.38	2.0	even	ok
	2	C45H89N5NaO2	754.6908	-0.3	4.0	2	100.00	4.0	even	ok
	3	C43H84N11	754.6906	-0.6	6.5	3	84.35	8.0	even	ok
	4	C47H88N5O2	754.6933	2.9	11.1	4	26.02	7.0	even	ok
	5	C44H93NNaO6	754.6895	-2.0	12.9	5	40.78	-1.0	even	ok
	6	C42H88N7O4	754.6892	-2.4	14.5	6	32.49	3.0	even	ok
	7	C41H85N11Na	754.6882	-3.8	16.3	7	12.83	5.0	even	ok
	8	C44H93N5PS	754.6884	-3.5	16.8	8	19.47	2.0	even	ok
	9	C43H94N3NaO3P	754.6925	1.9	17.1	9	39.96	-1.0	even	ok
	10	C42H93N5NaO2S	754.6942	4.2	17.7	10	11.93	-1.0	even	ok
	11	C40H88N11S	754.6939	3.8	17.9	11	12.49	3.0	even	ok
	12	C42H98N3OP2S	754.6900	-1.3	18.1	12	63.71	-3.0	even	ok
	13	C41H89N9OP	754.6922	1.5	19.0	13	45.76	3.0	even	ok
	14	C50H89N3Na	754.6949	5.1	24.1	14	4.13	8.0	even	ok
	15	C39H92N7O4S	754.6926	2.1	24.3	15	39.96	-2.0	even	ok
	16	C38H89N11NaS	754.6915	0.6	24.6	16	58.71	0.0	even	ok
	17	C41H97NNaO6S	754.6929	2.4	25.0	17	32.37	-6.0	even	ok
	18	C41H92N3O8	754.6879	-4.2	25.7	18	8.07	-2.0	even	ok
	19	C40H99N3NaOP2S	754.6876	-4.5	27.4	19	5.90	-6.0	even	ok
	20	C40H93N5O5P	754.6909	-0.2	29.5	20	60.22	-2.0	even	ok
	21	C39H90N9NaOP	754.6898	-1.6	30.7	21	33.61	0.0	even	ok
	22	C41H99NNaO4P2	754.6942	4.1	32.0	22	7.36	-6.0	even	ok
	23	C39H94N7O2P2	754.6939	3.7	33.6	23	9.23	-2.0	even	ok
	24	C38H96N3O8S	754.6913	0.3	33.8	24	66.40	-7.0	even	ok
	25	C37H97N5O5PS	754.6943	4.2	34.9	25	7.71	-7.0	even	ok
	26	C41H97N5PS2	754.6918	0.9	35.7	26	50.13	-3.0	even	ok
	27	C37H93N7NaO4S	754.6902	-1.1	35.8	27	36.87	-5.0	even	ok
	28	C35H88N13O2S	754.6899	-1.5	36.0	28	31.29	-1.0	even	ok
	29	C39H98N5NaPS2	754.6893	-2.3	36.1	29	26.94	-6.0	even	ok

Compound Spectrum SmartFormula Report

Meas. m/z	#	Ion Formula	m/z	err [ppm]	mSigma	# mSigma	Score	rdb	e¯ Conf	N-Rule
	30	C50H92NOS	754.6894	-2.2	37.0	30	27.54	6.0	even	ok
	31	C36H94N9NaOPS	754.6932	2.8	37.5	31	15.06	-5.0	even	ok
	32	C39H102N3OP2S2	754.6934	3.1	37.6	32	15.74	-8.0	even	ok
	33	C42H96N3O3S2	754.6888	-3.0	39.1	33	15.90	-3.0	even	ok
	34	C40H101NO4PS2	754.6904	-0.8	39.5	34	46.79	-8.0	even	ok
	35	C39H97NO9P	754.6895	-2.0	40.6	35	21.85	-7.0	even	ok
	36	C38H94N5NaO5P	754.6885	-3.4	41.3	36	9.41	-5.0	even	ok
	37	C36H97N7O2PS2	754.6877	-4.4	41.5	37	5.70	-7.0	even	ok
	38	C45H97NNaOS2	754.6904	-0.9	41.6	38	43.37	-2.0	even	ok
	39	C37H103N3NaOP2S2	754.6910	-0.1	41.8	39	57.45	-11.0	even	ok
	40	C36H97N3NaO8S	754.6889	-2.9	42.4	40	15.71	-10.0	even	ok
	41	C38H102NNaO4PS2	754.6880	-4.0	42.6	41	7.35	-11.0	even	ok
	42	C36H89N11O3P	754.6882	-3.8	42.9	42	6.98	-1.0	even	ok
	43	C38H98N3O6P2	754.6925	2.0	44.4	43	19.96	-7.0	even	ok
	44	C36H101NO9PS	754.6929	2.5	45.2	44	18.66	-12.0	even	ok
	45	C37H95N7NaO2P2	754.6915	0.6	45.4	45	35.39	-5.0	even	ok
	46	C33H89N13NaO2S	754.6875	-4.7	46.5	46	3.13	-4.0	even	ok
	47	C35H90N13P2	754.6912	0.2	47.1	47	38.13	-1.0	even	ok
	48	C31H84N19S	754.6872	-5.1	47.2	48	2.25	0.0	even	ok
	49	C32H92N13O2S2	754.6933	3.0	47.5	49	13.11	-6.0	even	ok
	50	C34H92N9O6S	754.6886	-3.3	47.6	50	8.58	-6.0	even	ok
	51	C34H97N7NaO4S2	754.6936	3.3	48.1	51	10.19	-10.0	even	ok
	52	C47H96NOS2	754.6928	2.3	48.2	52	18.81	1.0	even	ok
	53	C35H98N5NaO5PS	754.6918	1.1	49.2	53	26.07	-10.0	even	ok
	54	C33H93N11O3PS	754.6916	0.7	49.8	54	29.58	-6.0	even	ok
	55	C34H102N5O3P2S2	754.6894	-2.2	49.9	55	18.70	-12.0	even	ok
	56	C35H100N3O8S2	754.6946	4.7	50.7	56	3.31	-12.0	even	ok
	57	C34H99N7NaO2P2S	754.6948	5.0	51.6	57	2.05	-10.0	even	ok
	58	C32H94N13P2S	754.6946	4.6	52.3	58	2.73	-6.0	even	ok
	59	C33H96N5O10S	754.6872	-5.1	53.1	59	2.38	-11.0	even	ok
	60	C34H89N11NaO5	754.6940	4.0	53.3	60	4.53	-4.0	even	ok
	61	C32H84N17O3	754.6938	3.6	54.8	61	5.61	0.0	even	ok
	62	C36H99N3NaO6P2	754.6901	-1.2	56.1	62	19.65	-10.0	even	ok
	63	C34H94N9O4P2	754.6899	-1.6	57.6	63	15.90	-6.0	even	ok
	64	C33H91N13NaP2	754.6888	-3.0	58.6	64	7.19	-4.0	even	ok
	65	C34H102NNaO9PS	754.6905	-0.7	61.0	65	20.38	-15.0	even	ok
	66	C34H96N3O13	754.6938	3.6	63.8	66	4.11	-11.0	even	ok
	67	C33H93N7NaO9	754.6927	2.2	64.2	67	9.51	-9.0	even	ok
	68	C31H88N13O7	754.6924	1.8	65.6	68	10.91	-5.0	even	ok
	69	C30H85N17NaO3	754.6914	0.4	66.4	69	18.91	-3.0	even	ok
	70	C28H80N23O	754.6911	0.0	67.9	70	20.13	1.0	even	ok
	71	C33H98N5O8P2	754.6885	-3.4	68.4	71	4.07	-11.0	even	ok
	72	C32H95N9NaO4P2	754.6874	-4.8	69.2	72	1.38	-9.0	even	ok
	73	C29H86N19NaP	754.6943	4.4	70.3	73	1.86	-3.0	even	ok
885.5877	1	C54H82N2O6P	885.5905	3.1	4.0	1	14.32	16.0	even	ok
	2	C55H81O9	885.5875	-0.2	4.2	2	100.00	16.0	even	ok

Compound Spectrum SmartFormula Report

Meas. m/z	#	Ion Formula	m/z	err [ppm]	mSigma	# mSigma	Score	rdb	e¯ Conf	N-Rule
	3	C54H78N4NaO5	885.5864	-1.4	5.9	3	44.33	18.0	even	ok
	4	C52H73N10O3	885.5862	-1.8	7.1	4	36.20	22.0	even	ok
	5	C53H79N6NaO2P	885.5894	1.9	8.2	5	31.75	18.0	even	ok
	6	C53H82NaO9	885.5851	-2.9	8.3	6	15.55	13.0	even	ok
	7	C51H77N6O7	885.5848	-3.3	10.4	7	11.57	17.0	even	ok
	8	C51H74N12P	885.5892	1.6	10.6	8	36.74	22.0	even	ok
	9	C50H84N6NaP2S	885.5846	-3.6	11.5	9	10.96	13.0	even	ok
	10	C52H83N2NaO6P	885.5881	0.4	13.2	10	62.77	13.0	even	ok
	11	C50H74N10NaO3	885.5838	-4.5	13.3	11	3.53	19.0	even	ok
	12	C52H83N6P2S	885.5870	-0.8	13.7	12	64.85	16.0	even	ok
	13	C49H77N10O3S	885.5895	2.1	13.8	13	33.21	17.0	even	ok
	14	C55H78N6O2P	885.5918	4.7	13.9	14	2.86	21.0	even	ok
	15	C55H79N6P2	885.5836	-4.7	14.0	15	2.87	21.0	even	ok
	16	C57H84NaO2P2	885.5839	-4.3	14.1	16	3.98	17.0	even	ok
	17	C57H83NaO4P	885.5921	5.0	14.5	17	2.00	17.0	even	ok
	18	C51H87N2O4P2S	885.5856	-2.4	14.8	18	26.58	11.0	even	ok
	19	C50H78N8O4P	885.5878	0.1	15.6	19	67.69	17.0	even	ok
	20	C51H82N4NaO5S	885.5898	2.4	15.8	20	25.86	13.0	even	ok
	21	C48H69N16O	885.5835	-4.8	15.9	21	2.39	23.0	even	ok
	22	C52H87O7P2	885.5922	5.0	16.1	22	1.85	11.0	even	ok
	23	C54H88NaO2P2S	885.5872	-0.5	16.1	23	71.03	12.0	even	ok
	24	C56H77N4O5	885.5888	1.3	16.7	24	39.42	21.0	even	ok
	25	C55H74N8NaO	885.5878	0.1	17.2	25	66.59	23.0	even	ok
	26	C52H78N8NaOS	885.5912	3.9	17.3	26	7.32	18.0	even	ok
	27	C45H73N16OS	885.5868	-1.0	17.3	27	56.58	18.0	even	ok
	28	C47H78N10NaO3S	885.5871	-0.7	17.5	28	65.13	14.0	even	ok
	29	C53H82N4O3PS	885.5840	-4.2	18.4	29	5.11	16.0	even	ok
	30	C51H84N4NaO3P2	885.5911	3.8	18.5	30	6.12	13.0	even	ok
	31	C46H79N12NaPS	885.5901	2.7	18.5	31	19.07	14.0	even	ok
	32	C49H75N12NaP	885.5867	-1.1	18.6	32	41.62	19.0	even	ok
	33	C52H85O9S	885.5909	3.6	19.6	33	9.21	11.0	even	ok
	34	C49H88N2NaO4P2S	885.5832	-5.1	19.9	34	1.99	8.0	even	ok
	35	C48H81N6O7S	885.5882	0.5	20.0	35	65.27	12.0	even	ok
	36	C47H82N8O4PS	885.5912	3.9	20.1	36	6.64	12.0	even	ok
	37	C49H87N2NaO6PS	885.5915	4.2	20.4	37	4.84	8.0	even	ok
	38	C53H81N4O5S	885.5922	5.1	20.8	38	1.93	16.0	even	ok
	39	C50H86NaO9S	885.5885	0.9	20.9	39	55.60	8.0	even	ok
	40	C49H79N10OP2	885.5908	3.5	20.9	40	7.72	17.0	even	ok
	41	C50H81N2O11	885.5835	-4.8	21.3	41	2.16	12.0	even	ok
	42	C56H87O2P2S	885.5897	2.2	25.0	42	24.01	15.0	even	ok
	43	C49H82N4O8P	885.5865	-1.4	25.7	43	30.41	12.0	even	ok
	44	C59H83O2P2	885.5863	-1.6	25.7	44	26.83	20.0	even	ok
	45	C43H74N16NaOS	885.5844	-3.7	26.6	45	7.07	15.0	even	ok
	46	C56H83N2NaOPS	885.5856	-2.4	27.1	46	19.80	17.0	even	ok
	47	C46H82N6NaO7S	885.5858	-2.2	27.3	47	22.94	9.0	even	ok
	48	C44H77N12O5S	885.5855	-2.5	27.5	48	18.39	13.0	even	ok

Compound Spectrum SmartFormula Report

Meas. m/z	#	Ion Formula	m/z	err [ppm]	mSigma	# mSigma	Score	rdb	e¯ Conf	N-Rule
	49	C48H79N8NaO4P	885.5854	-2.6	27.5	49	13.54	14.0	even	ok
	50	C50H88NaO7P2	885.5897	2.3	28.4	50	16.48	8.0	even	ok
	51	C57H73N8O	885.5902	2.8	28.9	51	11.42	26.0	even	ok
	52	C45H83N8NaO4PS	885.5888	1.2	28.9	52	39.04	9.0	even	ok
	53	C47H85N2O11S	885.5869	-1.0	29.0	53	43.88	7.0	even	ok
	54	C43H78N14O2PS	885.5885	0.9	29.5	54	45.02	13.0	even	ok
	55	C46H74N14O2P	885.5851	-2.9	29.7	55	10.12	18.0	even	ok
	56	C46H86N4O8PS	885.5898	2.4	30.1	56	18.30	7.0	even	ok
	57	C59H78N2NaO3	885.5905	3.1	30.2	57	8.64	22.0	even	ok
	58	C48H83N6O5P2	885.5895	2.0	30.4	58	19.32	12.0	even	ok
	59	C44H84N10NaOP2S	885.5918	4.6	31.3	59	2.64	9.0	even	ok
	60	C49H87N6P2S2	885.5903	3.0	31.9	60	11.61	11.0	even	ok
	61	C47H88N6NaP2S2	885.5879	0.2	32.1	61	55.62	8.0	even	ok
	62	C47H80N10NaOP2	885.5884	0.8	32.5	62	35.28	14.0	even	ok
	63	C46H82N10OPS2	885.5847	-3.4	32.8	63	7.57	12.0	even	ok
	64	C48H87N4NaO3PS2	885.5849	-3.1	33.7	64	9.66	8.0	even	ok
	65	C51H92NaO2P2S2	885.5906	3.3	34.1	65	8.49	7.0	even	ok
	66	C48H91N2O4P2S2	885.5890	1.5	34.7	66	29.63	6.0	even	ok
	67	C42H77N16OS2	885.5902	2.8	35.3	67	11.82	13.0	even	ok
	68	C50H86N4O3PS2	885.5873	-0.4	35.7	68	47.33	11.0	even	ok
	69	C50H82N6NaO2S2	885.5833	-5.0	36.0	69	1.48	13.0	even	ok
	70	C44H82N10NaO3S2	885.5905	3.1	36.4	70	8.94	9.0	even	ok
	71	C48H86O12P	885.5851	-2.9	36.8	71	8.53	7.0	even	ok
	72	C44H87N8O2P2S2	885.5863	-1.6	37.1	72	25.84	7.0	even	ok
	73	C49H90O7PS2	885.5860	-1.9	37.4	73	20.89	6.0	even	ok
	74	C46H92N2NaO4P2S2	885.5866	-1.3	37.4	74	30.50	3.0	even	ok
	75	C45H86N6O5PS2	885.5833	-5.0	37.5	75	1.49	7.0	even	ok
	76	C45H86N2NaO11S	885.5845	-3.7	37.5	76	5.43	4.0	even	ok
	77	C43H81N8O9S	885.5842	-4.0	37.9	77	4.01	8.0	even	ok
	78	C47H83N4NaO8P	885.5841	-4.1	38.0	78	2.86	9.0	even	ok
	79	C58H82N2OPS	885.5880	0.3	38.0	79	46.32	20.0	even	ok
	80	C45H77N10O8	885.5920	4.9	38.2	80	1.28	13.0	even	ok
	81	C47H91NaO7PS2	885.5836	-4.6	38.7	81	2.04	3.0	even	ok
	82	C45H85N6O7S2	885.5916	4.3	38.8	82	2.77	7.0	even	ok
	83	C44H87N4NaO8PS	885.5874	-0.3	39.5	83	44.73	4.0	even	ok
	84	C41H79N14NaO2PS	885.5861	-1.8	39.8	84	20.80	10.0	even	ok
	85	C44H74N14NaO4	885.5910	3.7	39.8	85	4.14	15.0	even	ok
	86	C51H85N2O6S2	885.5844	-3.8	39.9	86	4.62	11.0	even	ok
	87	C45H78N10O6P	885.5838	-4.4	39.9	87	1.98	13.0	even	ok
	88	C53H87N2NaOPS2	885.5890	1.4	39.9	88	26.40	12.0	even	ok
	89	C42H82N10O6PS	885.5872	-0.6	40.1	89	38.60	8.0	even	ok
	90	C47H90NaO9S2	885.5918	4.7	40.1	90	1.92	3.0	even	ok
	91	C45H90O12PS	885.5885	0.9	40.4	91	33.80	2.0	even	ok
	92	C58H78N4NaS	885.5839	-4.3	40.6	92	2.87	22.0	even	ok
	93	C39H74N20PS	885.5858	-2.1	40.7	93	16.59	14.0	even	ok
	94	C40H78N16NaOS2	885.5878	0.1	40.8	94	46.46	10.0	even	ok

Compound Spectrum SmartFormula Report

Meas. m/z	#	Ion Formula	m/z	err [ppm]	mSigma	# mSigma	Score	rdb	e⁻ Conf	N-Rule
	95	C47H87N2O9P2	885.5881	0.5	41.1	95	32.10	7.0	even	ok
	96	C61H78N2OP	885.5846	-3.5	41.7	96	4.63	25.0	even	ok
	97	C42H69N20O2	885.5907	3.4	41.8	97	5.17	19.0	even	ok
	98	C43H88N6NaO5P2S	885.5904	3.1	41.9	98	8.17	4.0	even	ok
	99	C52H81N6O2S2	885.5857	-2.3	42.2	99	14.51	16.0	even	ok
	100	C44H91N2O9P2S	885.5915	4.3	42.4	100	2.70	2.0	even	ok
	101	C46H84N6NaO5P2	885.5871	-0.7	42.5	101	27.45	9.0	even	ok
	102	C41H83N12O3P2S	885.5902	2.8	42.7	102	10.21	8.0	even	ok
	103	C40H83N14P2S2	885.5836	-4.6	42.8	103	1.88	8.0	even	ok
	104	C59H81O4S	885.5850	-3.1	43.0	104	8.00	20.0	even	ok
	105	C42H88N8NaO2P2S2	885.5839	-4.3	43.2	105	2.58	4.0	even	ok
	106	C41H81N12O5S2	885.5889	1.3	43.2	106	25.34	8.0	even	ok
	107	C43H86N6NaO7S2	885.5892	1.6	43.3	107	21.22	4.0	even	ok
	108	C40H82N14O2PS2	885.5919	4.7	43.3	108	1.70	8.0	even	ok
	109	C42H87N8NaO4PS2	885.5922	5.0	43.8	109	1.18	4.0	even	ok
	110	C44H79N12O3P2	885.5868	-1.1	44.5	110	22.36	13.0	even	ok
	111	C54H86NaO4S2	885.5860	-2.0	44.8	111	16.61	12.0	even	ok
	112	C43H91N4O6P2S2	885.5850	-3.1	44.8	112	7.39	2.0	even	ok
	113	C44H89N2O11S2	885.5902	2.8	45.9	113	8.74	2.0	even	ok
	114	C46H86NaO14	885.5910	3.7	47.7	114	3.27	4.0	even	ok
	115	C55H86N2OPS2	885.5914	4.1	48.1	115	2.66	15.0	even	ok
	116	C44H81N6O12	885.5907	3.4	49.2	116	4.12	8.0	even	ok
	117	C55H82N4NaS2	885.5873	-0.5	49.8	117	31.33	17.0	even	ok
	118	C37H77N18O3S2	885.5862	-1.7	50.0	118	16.57	9.0	even	ok
	119	C43H91NaO12PS	885.5861	-1.8	50.0	119	15.57	-1.0	even	ok
	120	C39H82N12NaO5S2	885.5865	-1.4	50.2	120	19.73	5.0	even	ok
	121	C40H83N10NaO6PS	885.5848	-3.3	50.4	121	5.10	5.0	even	ok
	122	C43H78N10NaO8	885.5896	2.2	50.4	122	9.92	10.0	even	ok
	123	C37H74N20NaO2S	885.5917	4.4	50.5	123	1.79	11.0	even	ok
	124	C41H86N6O10PS	885.5858	-2.1	50.7	124	12.50	3.0	even	ok
	125	C38H83N14NaO2PS2	885.5895	2.0	51.1	125	13.65	5.0	even	ok
	126	C36H78N20PS2	885.5892	1.7	51.2	126	16.51	9.0	even	ok
	127	C38H78N16O4PS	885.5845	-3.7	51.3	127	3.76	9.0	even	ok
	128	C42H90N2NaO11S2	885.5878	0.1	52.0	128	33.31	-1.0	even	ok
	129	C40H85N8O9S2	885.5875	-0.2	52.2	129	32.30	3.0	even	ok
	130	C60H77N4S	885.5863	-1.5	52.2	130	17.08	25.0	even	ok
	131	C41H73N16O6	885.5894	1.8	52.2	131	11.46	14.0	even	ok
	132	C42H92N2NaO9P2S	885.5891	1.6	52.5	132	16.81	-1.0	even	ok
	133	C39H86N10O6PS2	885.5905	3.2	52.8	133	5.39	3.0	even	ok
	134	C41H91N4NaO8PS2	885.5908	3.5	53.0	134	4.09	-1.0	even	ok
	135	C56H85O4S2	885.5884	0.7	53.2	135	24.86	15.0	even	ok
	136	C39H84N12NaO3P2S	885.5877	0.0	53.2	136	33.09	5.0	even	ok
	137	C45H88N2NaO9P2	885.5857	-2.2	53.2	137	8.57	4.0	even	ok
	138	C40H87N8O7P2S	885.5888	1.2	53.3	138	19.47	3.0	even	ok
	139	C40H70N20NaO2	885.5883	0.6	53.8	139	20.47	16.0	even	ok
	140	C42H95O10P2S2	885.5836	-4.6	54.1	140	1.36	-3.0	even	ok

Compound Spectrum SmartFormula Report

Meas. m/z	#	Ion Formula	m/z	err [ppm]	mSigma	# mSigma	Score	rdb	e¯ Conf	N-Rule
	141	C37H79N18OP2S	885.5875	-0.3	54.3	141	29.26	9.0	even	ok
	142	C42H94O12PS2	885.5919	4.7	54.5	142	1.20	-3.0	even	ok
	143	C43H83N8O7P2	885.5854	-2.6	55.0	143	6.50	8.0	even	ok
	144	C38H65N26	885.5880	0.3	55.8	144	21.90	20.0	even	ok
	145	C42H80N12NaO3P2	885.5844	-3.8	56.5	145	2.30	10.0	even	ok
	146	C37H75N20NaPS	885.5834	-4.9	57.4	146	0.73	11.0	even	ok
	147	C35H69N26S	885.5914	4.1	57.6	147	1.57	15.0	even	ok
	148	C40H75N18OP2	885.5841	-4.1	58.4	148	1.60	14.0	even	ok
	149	C35H78N18NaO3S2	885.5838	-4.4	58.6	149	1.40	6.0	even	ok
	150	C33H73N24OS2	885.5835	-4.7	58.8	150	0.99	10.0	even	ok
	151	C57H81N4S2	885.5897	2.3	59.7	151	8.61	20.0	even	ok
	152	C38H86N8NaO9S2	885.5851	-2.9	59.9	152	5.32	0.0	even	ok
	153	C36H81N14O7S2	885.5849	-3.2	60.0	153	4.12	4.0	even	ok
	154	C34H79N20NaPS2	885.5868	-1.1	60.2	154	17.07	6.0	even	ok
	155	C39H86N6NaO12S	885.5917	4.5	60.3	155	1.29	0.0	even	ok
	156	C43H85N2O16	885.5894	1.9	60.5	156	8.68	3.0	even	ok
	157	C39H87N6NaO10PS	885.5834	-4.8	60.9	157	0.83	0.0	even	ok
	158	C36H78N16NaO6S	885.5903	2.9	61.0	158	5.04	6.0	even	ok
	159	C37H81N12O10S	885.5914	4.1	61.1	159	1.73	4.0	even	ok
	160	C37H87N10NaO6PS2	885.5881	0.5	61.2	160	21.57	0.0	even	ok
	161	C39H89N4O13S2	885.5862	-1.7	61.2	161	11.61	-2.0	even	ok
	162	C40H90N2O14PS	885.5845	-3.6	61.3	162	2.75	-2.0	even	ok
	163	C42H82N6NaO12	885.5883	0.7	61.4	163	15.87	5.0	even	ok
	164	C35H82N16O4PS2	885.5879	0.2	61.4	164	24.27	4.0	even	ok
	165	C34H73N22O4S	885.5900	2.6	62.0	165	6.19	10.0	even	ok
	166	C40H95NaO12PS2	885.5895	2.0	62.3	166	9.40	-6.0	even	ok
	167	C38H90N6O10PS2	885.5892	1.7	62.7	167	11.25	-2.0	even	ok
	168	C36H88N12NaO3P2S2	885.5911	3.8	62.8	168	2.17	0.0	even	ok
	169	C40H77N12O10	885.5880	0.3	63.0	169	17.12	9.0	even	ok
	170	C34H83N18OP2S2	885.5908	3.5	63.1	170	2.86	4.0	even	ok
	171	C38H88N8NaO7P2S	885.5864	-1.5	63.7	171	12.18	0.0	even	ok
	172	C39H91N4O11P2S	885.5875	-0.3	63.8	172	21.35	-2.0	even	ok
	173	C37H91N8O7P2S2	885.5922	5.1	64.0	173	0.59	-2.0	even	ok
	174	C39H74N16NaO6	885.5869	-0.9	64.2	174	13.01	11.0	even	ok
	175	C36H83N14O5P2S	885.5861	-1.8	64.7	175	9.80	4.0	even	ok
	176	C41H83N8NaO9P	885.5913	4.0	65.5	176	1.32	5.0	even	ok
	177	C42H87N4O11P2	885.5841	-4.1	65.9	177	1.25	3.0	even	ok
	178	C37H69N22O4	885.5867	-1.2	66.0	178	10.45	15.0	even	ok
	179	C39H78N14O7P	885.5910	3.7	67.2	179	1.67	9.0	even	ok
	180	C36H66N26Na	885.5856	-2.4	67.6	180	4.76	17.0	even	ok
	181	C38H75N18NaO3P	885.5899	2.5	68.6	181	4.24	11.0	even	ok
	182	C33H70N26NaS	885.5890	1.4	69.5	182	8.17	12.0	even	ok
	183	C37H90N4NaO13S2	885.5838	-4.4	70.0	183	0.95	-5.0	even	ok
	184	C35H85N10O11S2	885.5835	-4.7	70.1	184	0.68	-1.0	even	ok
	185	C36H70N24OP	885.5897	2.2	70.5	185	4.92	15.0	even	ok
	186	C33H83N16NaO4PS2	885.5854	-2.6	70.7	186	4.71	1.0	even	ok

Compound Spectrum SmartFormula Report

Meas. m/z	#	Ion Formula	m/z	err [ppm]	mSigma	# mSigma	Score	rdb	e⁻ Conf	N-Rule
	187	C38H90N2NaO16S	885.5903	2.9	70.8	187	3.51	-5.0	even	ok
	188	C31H78N22O2PS2	885.5852	-2.9	71.1	188	3.66	5.0	even	ok
	189	C38H93O17S2	885.5849	-3.2	71.1	189	2.79	-7.0	even	ok
	190	C35H82N12NaO10S	885.5890	1.4	71.4	190	9.45	1.0	even	ok
	191	C36H91N6NaO10PS2	885.5868	-1.0	71.4	191	11.51	-5.0	even	ok
	192	C36H85N8O14S	885.5900	2.6	71.6	192	4.33	-1.0	even	ok
	193	C34H86N12O8PS2	885.5865	-1.4	71.7	193	9.69	-1.0	even	ok
	194	C33H77N18O8S	885.5887	1.1	72.4	194	10.76	5.0	even	ok
	195	C37H94N2O14PS2	885.5879	0.2	72.4	195	16.22	-7.0	even	ok
	196	C35H80N18NaOP2S	885.5851	-3.0	72.5	196	2.54	6.0	even	ok
	197	C41H86N2NaO16	885.5870	-0.9	72.5	197	9.66	0.0	even	ok
	198	C32H84N18NaOP2S2	885.5884	0.8	72.7	198	12.25	1.0	even	ok
	199	C35H92N8NaO7P2S2	885.5898	2.3	73.1	199	5.05	-5.0	even	ok
	200	C33H87N14O5P2S2	885.5895	2.0	73.5	200	6.13	-1.0	even	ok
	201	C39H81N8O14	885.5867	-1.2	74.0	201	7.82	4.0	even	ok
	202	C37H92N4NaO11P2S	885.5851	-3.0	74.1	202	3.01	-5.0	even	ok
	203	C36H95N4O11P2S2	885.5909	3.5	74.3	203	1.88	-7.0	even	ok
	204	C34H83N14NaO7PS	885.5920	4.8	74.3	204	0.54	1.0	even	ok
	205	C38H95O15P2S	885.5861	-1.8	74.3	205	6.91	-7.0	even	ok
	206	C38H78N12NaO10	885.5856	-2.4	75.0	206	3.65	6.0	even	ok
	207	C35H87N10O9P2S	885.5848	-3.3	75.1	207	2.24	-1.0	even	ok
	208	C41H90O17P	885.5910	3.7	75.6	208	1.21	-2.0	even	ok
	209	C40H87N4NaO13P	885.5899	2.5	76.4	209	3.13	0.0	even	ok
	210	C36H73N18O8	885.5853	-2.7	76.6	210	2.72	10.0	even	ok
	211	C35H70N22NaO4	885.5843	-3.9	77.9	211	0.94	12.0	even	ok
	212	C38H82N10O11P	885.5897	2.2	78.0	212	3.65	4.0	even	ok
	213	C37H79N14NaO7P	885.5886	1.0	79.1	213	6.99	6.0	even	ok
	214	C33H65N28O2	885.5840	-4.2	79.7	214	0.64	16.0	even	ok
	215	C29H78N22NaO4S2	885.5910	3.7	80.5	215	1.26	2.0	even	ok
	216	C35H74N20O5P	885.5883	0.7	80.8	216	7.55	10.0	even	ok
	217	C32H74N22NaO4S	885.5876	-0.1	81.0	217	9.49	7.0	even	ok
	218	C32H87N12NaO8PS2	885.5841	-4.1	81.1	218	0.87	-4.0	even	ok
	219	C30H81N18O8S2	885.5921	4.9	81.3	219	0.36	0.0	even	ok
	220	C30H82N18O6PS2	885.5838	-4.4	81.6	220	0.62	0.0	even	ok
	221	C35H95N2NaO14PS2	885.5855	-2.6	81.8	221	3.08	-10.0	even	ok
	222	C34H86N8NaO14S	885.5876	-0.1	81.8	222	11.52	-4.0	even	ok
	223	C35H89N4O18S	885.5887	1.1	82.1	223	7.28	-6.0	even	ok
	224	C33H90N8O12PS2	885.5852	-2.9	82.1	224	2.40	-6.0	even	ok
	225	C34H71N24NaOP	885.5873	-0.5	82.2	225	7.62	12.0	even	ok
	226	C30H69N28O2S	885.5874	-0.4	82.2	226	7.99	11.0	even	ok
	227	C32H81N14O12S	885.5874	-0.4	82.7	227	9.83	0.0	even	ok
	228	C31H78N18NaO8S	885.5863	-1.6	82.8	228	5.45	2.0	even	ok
	229	C31H88N14NaO5P2S2	885.5871	-0.7	83.2	229	8.50	-4.0	even	ok
	230	C36H80N16NaO4P2	885.5916	4.4	83.4	230	0.47	6.0	even	ok
	231	C34H96N4NaO11P2S2	885.5884	0.8	83.6	231	7.91	-10.0	even	ok
	232	C29H83N20O3P2S2	885.5868	-1.0	83.8	232	7.16	0.0	even	ok

Compound Spectrum SmartFormula Report

Meas. m/z	#	Ion Formula	m/z	err [ppm]	mSigma	# mSigma	Score	rdb	e⁻ Conf	N-Rule
	233	C36H95NaO17PS	885.5920	4.8	83.9	233	0.36	-10.0	even	ok
	234	C32H91N10O9P2S2	885.5882	0.5	84.0	234	8.93	-6.0	even	ok
	235	C34H84N14NaO5P2S	885.5837	-4.5	84.0	235	0.40	1.0	even	ok
	236	C27H73N28O2S2	885.5907	3.4	84.1	236	1.44	6.0	even	ok
	237	C32H78N20O5PS	885.5917	4.5	84.2	237	0.40	5.0	even	ok
	238	C31H75N24NaOPS	885.5906	3.3	84.5	238	1.24	7.0	even	ok
	239	C36H96NaO15P2S	885.5837	-4.5	84.5	239	0.49	-10.0	even	ok
	240	C35H99O15P2S2	885.5895	2.0	84.6	240	3.90	-12.0	even	ok
	241	C33H87N10NaO11PS	885.5906	3.3	84.6	241	1.53	-4.0	even	ok
	242	C34H90N6O15PS	885.5917	4.5	84.7	242	0.49	-6.0	even	ok
	243	C34H75N22O2P2	885.5913	4.1	85.1	243	0.60	10.0	even	ok
	244	C38H85N4O18	885.5853	-2.7	85.1	244	1.94	-1.0	even	ok
	245	C32H79N20O3P2S	885.5834	-4.8	85.2	245	0.27	5.0	even	ok
	246	C34H91N6O13P2S	885.5835	-4.8	85.4	246	0.34	-6.0	even	ok
	247	C31H82N16O9PS	885.5904	3.0	85.6	247	1.90	0.0	even	ok
	248	C37H82N8NaO14	885.5843	-3.9	85.9	248	0.68	1.0	even	ok
	249	C35H77N14O12	885.5840	-4.2	87.4	249	0.47	5.0	even	ok
	250	C39H91NaO17P	885.5886	1.0	87.5	250	4.91	-5.0	even	ok
	251	C37H86N6O15P	885.5883	0.7	89.0	251	5.33	-1.0	even	ok
	252	C36H83N10NaO11P	885.5873	-0.5	89.8	252	5.54	1.0	even	ok
	253	C28H82N18NaO8S2	885.5897	2.2	90.9	253	2.66	-3.0	even	ok
	254	C31H90N8NaO14S2	885.5910	3.7	91.3	254	0.79	-9.0	even	ok
	255	C38H92N2NaO14P2	885.5916	4.4	91.3	255	0.33	-5.0	even	ok
	256	C34H78N16O9P	885.5870	-0.8	91.4	256	4.49	5.0	even	ok
	257	C26H77N24O6S2	885.5894	1.9	91.5	257	3.17	1.0	even	ok
	258	C29H85N14O12S2	885.5907	3.4	91.7	258	1.02	-5.0	even	ok
	259	C33H90N4NaO18S	885.5863	-1.6	92.2	259	3.65	-9.0	even	ok
	260	C32H93N4O18S2	885.5921	4.9	92.3	260	0.22	-11.0	even	ok
	261	C32H94N4O16PS2	885.5838	-4.4	92.5	261	0.39	-11.0	even	ok
	262	C34H93O22S	885.5874	-0.4	92.5	262	6.48	-11.0	even	ok
	263	C33H75N20NaO5P	885.5859	-2.0	92.6	263	2.20	7.0	even	ok
	264	C36H87N8O12P2	885.5913	4.1	92.9	264	0.42	-1.0	even	ok
	265	C30H82N14NaO12S	885.5850	-3.1	93.0	265	1.23	-3.0	even	ok
	266	C31H85N10O16S	885.5860	-1.9	93.1	266	2.91	-5.0	even	ok
	267	C25H74N28NaO2S2	885.5883	0.7	93.3	267	5.53	3.0	even	ok
	268	C29H73N24O6S	885.5860	-1.9	93.7	268	2.25	6.0	even	ok
	269	C23H69N34S2	885.5880	0.4	93.8	269	6.17	7.0	even	ok
	270	C35H84N12NaO8P2	885.5903	2.9	93.9	270	1.16	1.0	even	ok
	271	C28H70N28NaO2S	885.5849	-3.1	94.0	271	0.93	8.0	even	ok
	272	C28H77N20O10S	885.5847	-3.4	94.0	272	0.90	1.0	even	ok
	273	C31H70N26O3P	885.5856	-2.4	94.3	273	1.65	11.0	even	ok
	274	C32H91N6NaO15PS	885.5893	1.8	95.0	274	2.89	-9.0	even	ok
	275	C33H94N2O19PS	885.5904	3.0	95.1	275	1.24	-11.0	even	ok
	276	C26H65N34S	885.5847	-3.4	95.3	276	0.67	12.0	even	ok
	277	C33H79N18O6P2	885.5900	2.5	95.5	277	1.36	5.0	even	ok
	278	C30H79N20NaO5PS	885.5893	1.8	95.9	278	2.23	2.0	even	ok

Compound Spectrum SmartFormula Report

Meas. m/z	#	Ion Formula	m/z	err [ppm]	mSigma	# mSigma	Score	rdb	e⁻ Conf	N-Rule
	279	C30H86N12O13PS	885.5890	1.5	95.9	279	3.32	-5.0	even	ok
	280	C37H89O22	885.5840	-4.2	96.3	280	0.32	-6.0	even	ok
	281	C32H76N22NaO2P2	885.5889	1.3	96.8	281	2.73	7.0	even	ok
	282	C28H74N26O3PS	885.5890	1.5	97.1	282	2.53	6.0	even	ok
	283	C30H71N28P2	885.5886	1.0	98.5	283	2.96	11.0	even	ok
	284	C29H87N14O10P2S	885.5920	4.8	98.8	284	0.18	-5.0	even	ok
	285	C36H90N2O19P	885.5870	-0.8	100.0	285	3.05	-6.0	even	ok
918.8105	1	C52H101N11NaO	918.8083	-2.4	4.1	1	33.50	8.0	even	ok
	2	C52H105N9O2P	918.8123	2.0	5.4	2	45.83	6.0	even	ok
	3	C54H110N3NaO4P	918.8126	2.3	6.5	3	36.40	2.0	even	ok
	4	C53H104N7O5	918.8093	-1.3	7.7	4	66.85	6.0	even	ok
	5	C55H109NNaO7	918.8096	-1.0	9.1	5	95.76	2.0	even	ok
	6	C55H106N7NaP	918.8140	3.7	11.3	6	9.75	7.0	even	ok
	7	C56H110N3O2P2	918.8068	-4.1	11.5	7	6.85	5.0	even	ok
	8	C51H105N7NaO5	918.8069	-3.9	13.0	8	7.96	3.0	even	ok
	9	C49H100N13O3	918.8067	-4.2	13.7	9	5.73	7.0	even	ok
	10	C56H109N3O4P	918.8150	4.9	13.8	10	2.64	5.0	even	ok
	11	C54H100N11O	918.8107	0.2	14.1	11	100.00	11.0	even	ok
	12	C52H108N3O9	918.8080	-2.7	14.7	12	21.95	1.0	even	ok
	13	C50H106N9NaO2P	918.8099	-0.6	15.9	13	78.90	3.0	even	ok
	14	C51H109N5O6P	918.8110	0.5	16.2	14	83.16	1.0	even	ok
	15	C56H105N5NaO3	918.8110	0.5	16.4	15	84.29	7.0	even	ok
	16	C57H108NO7	918.8120	1.6	17.3	16	56.54	5.0	even	ok
	17	C48H101N15P	918.8097	-1.0	17.3	17	66.23	7.0	even	ok
	18	C52H115NNaO5P2	918.8143	4.1	18.2	18	6.02	-3.0	even	ok
	19	C50H110N7O3P2	918.8140	3.8	19.2	19	8.01	1.0	even	ok
	20	C49H110N9P2S	918.8075	-3.3	22.3	20	14.18	1.0	even	ok
	21	C49H105N11NaOS	918.8116	1.2	22.8	21	63.96	3.0	even	ok
	22	C51H115N3NaO2P2S	918.8077	-3.0	23.8	22	17.89	-3.0	even	ok
	23	C59H111NNaP2	918.8084	-2.3	24.2	23	24.74	6.0	even	ok
	24	C53H114N3O2P2S	918.8102	-0.4	25.3	24	89.53	0.0	even	ok
	25	C51H104N11OS	918.8141	3.8	25.6	25	8.11	6.0	even	ok
	26	C53H110N5NaOPS	918.8061	-4.8	25.7	26	2.78	2.0	even	ok
	27	C58H104N5O3	918.8134	3.1	26.0	27	16.07	10.0	even	ok
	28	C50H108N7O5S	918.8127	2.4	26.5	28	27.97	1.0	even	ok
	29	C45H105N15PS	918.8130	2.7	27.1	29	21.39	2.0	even	ok
	30	C46H104N13O3S	918.8100	-0.5	27.3	30	80.74	2.0	even	ok
	31	C47H110N9NaO2PS	918.8133	3.0	27.7	31	16.43	-2.0	even	ok
	32	C49H110N5NaO6P	918.8086	-2.1	27.8	32	26.52	-2.0	even	ok
	33	C53H109N5NaO3S	918.8143	4.1	28.0	33	5.62	2.0	even	ok
	34	C50H113NO10P	918.8097	-0.9	28.1	34	52.50	-4.0	even	ok
	35	C48H109N7NaO5S	918.8103	-0.2	28.3	35	89.59	-2.0	even	ok
	36	C52H113NNaO7S	918.8130	2.7	28.4	36	21.19	-3.0	even	ok
	37	C47H105N11O4P	918.8083	-2.4	29.0	37	20.73	2.0	even	ok
	38	C48H114N5O4P2S	918.8061	-4.8	29.4	38	2.66	-4.0	even	ok
	39	C54H113NO5PS	918.8072	-3.7	29.5	39	8.80	0.0	even	ok

Compound Spectrum SmartFormula Report

Meas. m/z	#	Ion Formula	m/z	err [ppm]	mSigma	# mSigma	Score	rdb	e⁻ Conf	N-Rule
	40	C46H102N15NaP	918.8072	-3.6	29.7	40	7.66	4.0	even	ok
	41	C44H96N21	918.8152	5.1	29.9	41	1.45	8.0	even	ok
	42	C48H113N5O6PS	918.8144	4.2	30.3	42	5.10	-4.0	even	ok
	43	C56H115NNaP2S	918.8118	1.4	30.4	43	49.89	1.0	even	ok
	44	C49H114N3O7P2	918.8127	2.3	31.1	44	21.08	-4.0	even	ok
	45	C48H111N7NaO3P2	918.8116	1.2	31.4	45	43.41	-2.0	even	ok
	46	C49H112N3O9S	918.8114	0.9	31.4	46	60.85	-4.0	even	ok
	47	C55H109N5OPS	918.8085	-2.2	32.8	47	27.26	5.0	even	ok
	48	C46H106N13OP2	918.8113	0.8	32.9	48	48.83	2.0	even	ok
	49	C42H100N19OS	918.8073	-3.5	33.7	49	9.59	3.0	even	ok
	50	C44H105N13NaO3S	918.8076	-3.2	33.9	50	12.57	-1.0	even	ok
	51	C43H106N15NaPS	918.8106	0.1	34.9	51	80.13	-1.0	even	ok
	52	C45H108N9O7S	918.8087	-2.0	35.9	52	29.20	-3.0	even	ok
	53	C47H113N3NaO9S	918.8090	-1.7	36.4	53	35.06	-7.0	even	ok
	54	C44H109N11O4PS	918.8117	1.3	36.5	54	44.87	-3.0	even	ok
	55	C46H114N5NaO6PS	918.8120	1.6	36.7	55	37.48	-7.0	even	ok
	56	C61H110NP2	918.8108	0.3	37.3	56	55.74	9.0	even	ok
	57	C45H115N7NaO3P2S	918.8150	4.8	37.6	57	2.07	-7.0	even	ok
	58	C43H110N13OP2S	918.8147	4.5	37.8	58	2.93	-3.0	even	ok
	59	C61H105N3NaO	918.8150	4.9	37.9	59	1.98	11.0	even	ok
	60	C47H117NO10PS	918.8130	2.7	38.8	60	15.77	-9.0	even	ok
	61	C58H114NP2S	918.8142	4.0	39.5	61	5.02	4.0	even	ok
	62	C48H114NNaO10P	918.8073	-3.6	39.7	62	5.99	-7.0	even	ok
	63	C45H105N11NaO6	918.8142	3.9	40.0	63	4.06	-1.0	even	ok
	64	C46H109N7O8P	918.8070	-3.9	40.8	64	4.32	-3.0	even	ok
	65	C45H106N11NaO4P	918.8059	-5.0	41.2	65	1.18	-1.0	even	ok
	66	C43H100N17O4	918.8139	3.6	41.4	66	5.28	3.0	even	ok
	67	C46H114N9P2S2	918.8108	0.3	42.1	67	60.20	-4.0	even	ok
	68	C42H97N21Na	918.8128	2.5	42.2	68	13.93	5.0	even	ok
	69	C46H109N11NaOS2	918.8150	4.9	42.7	69	1.66	-2.0	even	ok
	70	C47H115N3NaO7P2	918.8102	-0.3	43.1	70	47.43	-7.0	even	ok
	71	C48H119N3NaO2P2S2	918.8111	0.6	43.7	71	50.35	-8.0	even	ok
	72	C43H109N9NaO7S	918.8063	-4.6	43.8	72	2.23	-6.0	even	ok
	73	C41H104N15O5S	918.8060	-4.9	43.9	73	1.56	-2.0	even	ok
	74	C50H114N5NaOPS2	918.8095	-1.2	44.2	74	38.45	-3.0	even	ok
	75	C57H104N7S	918.8068	-4.0	44.3	75	4.23	10.0	even	ok
	76	C50H118N3O2P2S2	918.8135	3.3	44.4	76	8.62	-5.0	even	ok
	77	C45H110N9O5P2	918.8100	-0.6	44.4	77	40.05	-3.0	even	ok
	78	C47H113N7O3PS2	918.8078	-2.9	44.5	78	11.54	-4.0	even	ok
	79	C44H115N9NaP2S2	918.8084	-2.3	44.9	79	18.68	-7.0	even	ok
	80	C44H107N13NaOP2	918.8089	-1.8	45.2	80	20.90	-1.0	even	ok
	81	C42H110N11NaO4PS	918.8093	-1.4	45.2	81	33.44	-6.0	even	ok
	82	C44H112N5O11S	918.8074	-3.4	45.4	82	7.06	-8.0	even	ok
	83	C40H105N17O2PS	918.8090	-1.7	45.6	83	27.65	-2.0	even	ok
	84	C49H108N9O2S2	918.8062	-4.7	45.8	84	1.87	1.0	even	ok
	85	C43H108N13O3S2	918.8134	3.1	46.3	85	9.14	-3.0	even	ok

Compound Spectrum SmartFormula Report

Meas. m/z	#	Ion Formula	m/z	err [ppm]	mSigma	# mSigma	Score	rdb	e⁻ Conf	N-Rule
	86	C49H118NNaO5PS2	918.8081	-2.6	46.3	86	13.97	-8.0	even	ok
	87	C45H118NNaO10PS	918.8106	0.1	46.5	87	58.05	-12.0	even	ok
	88	C43H113N7O8PS	918.8103	-0.2	46.5	88	56.12	-8.0	even	ok
	89	C53H119NNaP2S2	918.8151	5.0	46.7	89	1.27	-4.0	even	ok
	90	C59H109NNaO2S	918.8071	-3.7	46.7	90	5.34	6.0	even	ok
	91	C41H111N13NaOP2S	918.8123	1.9	47.2	91	22.72	-6.0	even	ok
	92	C45H113N7NaO5S2	918.8137	3.4	47.6	92	6.70	-7.0	even	ok
	93	C44H119N3NaO7P2S	918.8136	3.4	47.8	93	7.10	-12.0	even	ok
	94	C45H118N5O4P2S2	918.8095	-1.1	47.9	94	35.22	-9.0	even	ok
	95	C51H117NO5PS2	918.8105	0.0	48.1	95	57.57	-5.0	even	ok
	96	C42H114N9O5P2S	918.8133	3.1	48.1	96	9.20	-8.0	even	ok
	97	C51H113N3NaO4S2	918.8065	-4.4	48.1	97	2.46	-3.0	even	ok
	98	C52H113N5OPS2	918.8119	1.5	48.7	98	28.45	0.0	even	ok
	99	C52H109N7NaS2	918.8078	-3.0	49.4	99	9.66	2.0	even	ok
	100	C46H117N3O7PS2	918.8065	-4.4	49.4	100	2.47	-9.0	even	ok
	101	C39H104N19OS2	918.8107	0.2	49.9	101	50.49	-2.0	even	ok
	102	C40H110N15NaPS2	918.8140	3.8	50.4	102	4.49	-6.0	even	ok
	103	C41H109N13NaO3S2	918.8110	0.5	50.6	103	43.46	-6.0	even	ok
	104	C46H116N3O9S2	918.8148	4.6	50.9	104	1.83	-9.0	even	ok
	105	C45H112N3O14	918.8139	3.7	51.8	105	3.85	-8.0	even	ok
	106	C44H109N7NaO10	918.8128	2.5	51.8	106	10.42	-6.0	even	ok
	107	C41H114N11O2P2S2	918.8068	-4.0	52.0	107	3.24	-8.0	even	ok
	108	C64H104NO2	918.8062	-4.8	52.6	108	1.46	14.0	even	ok
	109	C43H119N5NaO4P2S2	918.8071	-3.7	52.7	109	4.31	-12.0	even	ok
	110	C41H113N11O4PS2	918.8151	4.9	52.8	110	1.17	-8.0	even	ok
	111	C42H104N13O8	918.8125	2.2	53.0	111	12.50	-2.0	even	ok
	112	C53H112N3O4S2	918.8089	-1.8	53.2	112	20.18	0.0	even	ok
	113	C42H112N9O7S2	918.8121	1.7	53.4	113	21.65	-8.0	even	ok
	114	C41H101N17NaO4	918.8115	1.0	53.6	114	24.88	0.0	even	ok
	115	C44H117N3NaO9S2	918.8123	2.0	54.3	115	17.27	-12.0	even	ok
	116	C38H105N17NaO4S	918.8148	4.7	55.0	116	1.45	-5.0	even	ok
	117	C39H96N23O2	918.8112	0.7	55.1	117	27.52	4.0	even	ok
	118	C43H116NO15S	918.8060	-4.9	55.3	118	1.12	-13.0	even	ok
	119	C44H122NO8P2S2	918.8082	-2.6	55.3	119	10.92	-14.0	even	ok
	120	C38H106N17NaO2PS	918.8066	-4.3	55.4	120	2.27	-5.0	even	ok
	121	C36H100N23O2S	918.8146	4.4	55.6	121	2.00	-1.0	even	ok
	122	C41H114N7NaO8PS	918.8079	-2.8	55.7	122	8.95	-11.0	even	ok
	123	C36H101N23PS	918.8063	-4.6	56.1	123	1.59	-1.0	even	ok
	124	C44H114N5O9P2	918.8086	-2.1	56.1	124	12.34	-8.0	even	ok
	125	C39H109N13O6PS	918.8077	-3.1	56.1	125	6.84	-7.0	even	ok
	126	C37H105N19NaOS2	918.8083	-2.4	56.6	126	11.87	-5.0	even	ok
	127	C43H111N9NaO5P2	918.8076	-3.2	56.6	127	4.90	-6.0	even	ok
	128	C54H108N7S2	918.8102	-0.3	56.8	128	38.52	5.0	even	ok
	129	C42H117N3O12PS	918.8090	-1.6	56.8	129	19.72	-13.0	even	ok
	130	C61H108NO2S	918.8095	-1.1	57.5	130	26.54	9.0	even	ok
	131	C40H102N19NaOP	918.8145	4.3	57.6	131	1.69	0.0	even	ok

Compound Spectrum SmartFormula Report

Meas. m/z	#	Ion Formula	m/z	err [ppm]	mSigma	# mSigma	Score	rdb	e¯ Conf	N-Rule
	132	C40H115N9NaO5P2S	918.8109	0.4	57.7	132	35.76	-11.0	even	ok
	133	C41H106N15O3P2	918.8073	-3.5	58.1	133	3.53	-2.0	even	ok
	134	C38H110N15O3P2S	918.8107	0.1	58.3	134	39.68	-7.0	even	ok
	135	C41H118N5O9P2S	918.8120	1.6	58.5	135	19.10	-13.0	even	ok
	136	C38H108N15O5S2	918.8094	-1.2	58.7	136	23.39	-7.0	even	ok
	137	C37H109N17O2PS2	918.8124	2.0	59.2	137	14.47	-7.0	even	ok
	138	C40H113N9NaO7S2	918.8097	-0.9	59.2	138	27.01	-11.0	even	ok
	139	C56H113NNaO2S2	918.8105	-0.0	59.3	139	39.87	1.0	even	ok
	140	C39H114N11NaO4PS2	918.8127	2.3	59.5	140	11.59	-11.0	even	ok
	141	C41H116N5O11S2	918.8107	0.2	61.6	141	34.44	-13.0	even	ok
	142	C40H117N7O8PS2	918.8137	3.5	61.6	142	4.13	-13.0	even	ok
	143	C42H122NNaO10PS2	918.8140	3.8	62.1	143	3.03	-17.0	even	ok
	144	C43H113N3NaO14	918.8115	1.0	63.5	144	17.77	-11.0	even	ok
	145	C41H108N9O12	918.8112	0.7	64.7	145	19.81	-7.0	even	ok
	146	C40H105N13NaO8	918.8101	-0.4	65.1	146	22.34	-5.0	even	ok
	147	C37H109N13NaO8S	918.8135	3.2	65.5	147	4.47	-10.0	even	ok
	148	C34H101N23NaO2S	918.8122	1.8	65.7	148	13.51	-4.0	even	ok
	149	C40H117N3NaO14S	918.8148	4.7	65.9	149	0.99	-16.0	even	ok
	150	C35H104N19O6S	918.8132	2.9	66.1	150	5.68	-6.0	even	ok
	151	C40H118N3NaO12PS	918.8066	-4.3	66.1	151	1.59	-16.0	even	ok
	152	C38H112N9O12S	918.8146	4.4	66.3	152	1.37	-12.0	even	ok
	153	C38H100N19O6	918.8098	-0.7	66.4	153	18.55	-1.0	even	ok
	154	C38H113N9O10PS	918.8063	-4.6	66.6	154	1.12	-12.0	even	ok
	155	C42H114N5NaO11P	918.8145	4.3	67.0	155	1.21	-11.0	even	ok
	156	C37H97N23NaO2	918.8088	-1.9	67.2	156	9.40	1.0	even	ok
	157	C43H118NO13P2	918.8073	-3.5	67.7	157	2.55	-13.0	even	ok
	158	C42H115N5NaO9P2	918.8062	-4.7	68.1	158	0.75	-11.0	even	ok
	159	C58H112NO2S2	918.8129	2.6	68.2	159	6.96	4.0	even	ok
	160	C40H109N11O9P	918.8142	4.0	68.3	160	1.59	-7.0	even	ok
	161	C39H117N5NaO11S2	918.8083	-2.4	68.3	161	7.97	-16.0	even	ok
	162	C35H92N29	918.8085	-2.2	68.8	162	7.21	5.0	even	ok
	163	C39H106N15NaO5P	918.8131	2.8	68.8	163	4.51	-5.0	even	ok
	164	C40H110N11O7P2	918.8059	-5.0	69.4	164	0.50	-7.0	even	ok
	165	C42HO18P2S2	918.8074	-3.4	70.0	165	3.28	44.0	even	ok
	166	C37H101N21O3P	918.8128	2.5	70.3	166	5.42	-1.0	even	ok
	167	C40H120NO15S2	918.8094	-1.2	70.5	167	15.53	-18.0	even	ok
	168	C32H96N29S	918.8119	1.5	74.3	168	9.44	0.0	even	ok
	169	C40H112N5O16	918.8099	-0.7	76.3	169	12.86	-12.0	even	ok
	170	C39H109N9NaO12	918.8088	-1.9	76.6	170	6.65	-10.0	even	ok
	171	C40HNaO20PS2	918.8132	3.0	77.4	171	3.63	41.0	even	ok
	172	C37H104N15O10	918.8085	-2.2	77.9	172	5.14	-6.0	even	ok
	173	C36H101N19NaO6	918.8074	-3.4	78.4	173	1.97	-4.0	even	ok
	174	C41H118NNaO15P	918.8131	2.8	78.6	174	3.08	-16.0	even	ok
	175	C39H113N7O13P	918.8128	2.5	79.8	175	3.72	-12.0	even	ok
	176	C34H96N25O4	918.8072	-3.7	79.9	176	1.40	0.0	even	ok
	177	C38H110N11NaO9P	918.8118	1.4	80.2	177	7.92	-10.0	even	ok

Compound Spectrum SmartFormula Report

Meas. m/z	#	Ion Formula	m/z	err [ppm]	mSigma	# mSigma	Score	rdb	e¯ Conf	N-Rule
	178	C33H93N29Na	918.8061	-4.8	80.8	178	0.39	2.0	even	ok
	179	C36H105N17O7P	918.8115	1.1	81.6	179	8.83	-6.0	even	ok
	180	C35H102N21NaO3P	918.8104	-0.1	82.3	180	13.19	-4.0	even	ok
	181	C33H97N27OP	918.8102	-0.4	83.8	181	10.95	0.0	even	ok
	182	C37H111N13NaO6P2	918.8148	4.6	83.9	182	0.43	-10.0	even	ok
	183	C38HO23P2S	918.8099	-0.7	84.9	183	11.63	40.0	even	ok
	184	C36HN4O19P2S2	918.8146	4.5	87.6	185	0.56	40.0	even	ok
	185	C39H116NO20	918.8085	-2.2	88.0	186	3.41	-17.0	even	ok
	186	C38H113N5NaO16	918.8075	-3.3	88.1	187	1.33	-15.0	even	ok
	187	C36H108N11O14	918.8072	-3.6	89.3	188	0.95	-11.0	even	ok
	188	C35H105N15NaO10	918.8061	-4.8	89.7	189	0.27	-9.0	even	ok
	189	C36H2NaO23P2S	918.8075	-3.3	94.5	191	1.32	37.0	even	ok
	190	C35HN2NaO22PS2	918.8092	-1.4	94.9	192	5.09	37.0	even	ok
	191	C34H2N4NaO19P2S2	918.8122	1.8	96.1	193	3.75	37.0	even	ok
	192	C35H5O23P2S2	918.8133	3.0	97.6	194	1.47	35.0	even	ok
923.7662	1	C50H96N14P	923.7675	1.4	10.3	1	54.05	11.0	even	ok
	2	C52H101N8NaO2P	923.7677	1.7	10.6	2	44.87	7.0	even	ok
	3	C51H95N12O3	923.7645	-1.9	10.7	3	39.08	11.0	even	ok
	4	C54H101N8P2	923.7619	-4.7	10.9	4	3.18	10.0	even	ok
	5	C53H100N6NaO5	923.7647	-1.6	12.2	5	45.93	7.0	even	ok
	6	C54H100N8O2P	923.7701	4.3	13.1	6	4.72	10.0	even	ok
	7	C56H106N2NaO2P2	923.7622	-4.4	13.2	7	4.28	6.0	even	ok
	8	C53H104N4O6P	923.7688	2.8	13.4	8	18.54	5.0	even	ok
	9	C54H103N2O9	923.7658	-0.4	14.3	9	100.00	5.0	even	ok
	10	C54H96N10NaO	923.7661	-0.1	15.0	10	88.88	12.0	even	ok
	11	C56H105N2NaO4P	923.7704	4.6	15.5	11	3.21	6.0	even	ok
	12	C49H96N12NaO3	923.7621	-4.5	17.7	12	3.39	8.0	even	ok
	13	C55H99N6O5	923.7671	1.0	18.0	13	55.56	10.0	even	ok
	14	C47H91N18O	923.7618	-4.8	18.4	14	2.36	12.0	even	ok
	15	C57H104NaO7	923.7674	1.3	18.5	15	58.40	6.0	even	ok
	16	C50H99N8O7	923.7631	-3.3	19.1	16	10.67	6.0	even	ok
	17	C52H104N2NaO9	923.7634	-3.0	19.4	17	13.86	2.0	even	ok
	18	C52H108O10P	923.7675	1.4	19.8	18	55.59	0.0	even	ok
	19	C51H105N4NaO6P	923.7664	0.2	20.4	19	76.78	2.0	even	ok
	20	C48H97N14NaP	923.7650	-1.2	20.4	20	47.07	8.0	even	ok
	21	C49H100N10O4P	923.7661	-0.1	20.8	21	79.94	6.0	even	ok
	22	C50H106N6NaO3P2	923.7694	3.5	22.7	22	8.79	2.0	even	ok
	23	C51H109N2O7P2	923.7705	4.6	23.4	23	2.59	0.0	even	ok
	24	C48H101N12OP2	923.7691	3.1	23.6	24	11.31	6.0	even	ok
	25	C58H105N2O2P2	923.7646	-1.7	23.9	25	32.33	9.0	even	ok
	26	C51H105N8P2S	923.7653	-1.0	26.1	26	58.90	5.0	even	ok
	27	C59H104O5P	923.7616	-5.0	26.1	27	1.95	9.0	even	ok
	28	C49H106N8NaP2S	923.7629	-3.6	26.1	28	8.74	2.0	even	ok
	29	C47H100N14PS	923.7708	5.0	26.2	29	1.89	6.0	even	ok
	30	C56H95N10O	923.7685	2.5	26.5	30	18.36	15.0	even	ok
	31	C59H103O7	923.7698	3.9	28.2	31	6.03	9.0	even	ok

Compound Spectrum SmartFormula Report

Meas. m/z	#	Ion Formula	m/z	err [ppm]	mSigma	# mSigma	Score	rdb	e⁻ Conf	N-Rule
[truncated: 570,218 more chars]
